# Selective Inhibition of Lipid Oxidation by a Quercetagetin-Rich Extract from *Tagetes erecta*: A Chemometric Approach Using OPLS-DA and Selectivity Index Analysis

**DOI:** 10.3390/molecules31162891

**Published:** 2026-08-19

**Authors:** Xiuqiong Huang, Mengxuan Fang, Ziyue Wang, Qing Zheng, Zhixing Qing, Jianguo Zeng

**Affiliations:** 1 College of Food and Chemical Engineering, Shaoyang University, Shaoyang 422000, China; huangxqiong@163.com (X.H.); lensray777@gmail.com (M.F.); 19991766841@163.com (Z.W.); qz@hnsyu.edu.cn (Q.Z.); 2 Yuelushan Laboratory, Changsha 410128, China; 3 Hunan Key Laboratory of Traditional Chinese Veterinary Medicine, Hunan Agricultural University, Changsha 410128, China

**Keywords:** quercetagetin, *Tagetes erecta*, soybean oil, lipid oxidation, OPLS-DA, selectivity index, structure–activity relationship, natural antioxidant

## Abstract

This study investigates the antioxidant efficacy and selectivity of a quercetagetin-rich extract (QG, 89% purity) from *Tagetes erecta* in soybean oil under accelerated storage conditions (65 °C, 56 days). Using gas chromatography–mass spectrometry (GC-MS) fatty acid profiling, Headspace Solid-Phase Microextraction Gas Chromatography–Mass Spectrometry (HS-SPME-GC-MS) volatile analysis, and Orthogonal Partial Least Squares Discriminant Analysis (OPLS-DA) chemometrics, QG at 200 ppm was compared against a solvent control and propyl gallate (PG). QG preserved polyunsaturated fatty acids 5.2-fold more effectively than the control (*p* < 0.01), though it was less effective than PG in terms of overall Polyunsaturated Fatty Acid (PUFA) preservation. OPLS-DA-VIP analysis identified hexanal, hexanoic acid, and (*E*)-2-nonenal as preferentially suppressed volatiles (inhibition rates: 74.2%, 100%, and 81.2%, respectively), exceeding the average total volatile reduction (65.7%). A quantitative Selectivity Index (SI) combined with kinetic analysis supported preferential—rather than non-selective—inhibition of specific oxidation endpoints. Structure–activity relationship analysis suggested that the selectivity of the QG-rich extract may be associated with the catechol B-ring and unique 6-hydroxyl substitution of quercetagetin, its major component. The extract’s purity corresponds to a registered feed additive (Ministry of Agriculture and Rural Affairs (MOA) No. 618, 2022), ensuring industrial applicability. These findings establish the quercetagetin-rich extract as a promising selective natural antioxidant for PUFA-rich oils.

## 1. Introduction

The fatty acid composition of edible oils is a critical determinant of both their nutritional value and oxidative stability [1,2]. Soybean oil, one of the most widely consumed vegetable oils globally, is rich in polyunsaturated fatty acids (PUFAs)—particularly linoleic acid (C18:2) and α-linolenic acid (C18:3)—that are highly susceptible to oxidative degradation during processing and storage [1,3]. This oxidation not only generates undesirable flavors and odors but also reduces nutritional quality and produces potentially harmful lipid oxidation products [4,5].

Lipid oxidation proceeds via a complex, free-radical-mediated chain reaction [2,6,7]. The degradation of unsaturated fatty acids generates primary oxidation products, namely hydroperoxides, which further decompose into secondary volatile compounds including aldehydes, ketones, alcohols, and acids [2,8,9]. These secondary volatiles are responsible for rancid off-flavors and serve as the principal markers of quality deterioration [10,11]. Among them, hexanal—a primary degradation product of the 13-hydroperoxide of linoleic acid—is the most widely recognized indicator of lipid oxidation [12,13]. In addition to its sensory impact, hexanal has been linked to respiratory and inflammatory problems in livestock, underscoring the importance of its reduction in feed applications [14]. Lipid oxidation also presents a significant challenge in the production of animal feed: feedstuffs containing vegetable oils are particularly prone to oxidative degradation, resulting in diminished palatability, depletion of fat-soluble vitamins, and the potential generation of toxic aldehydes that adversely affect animal health [14,15].

To address oxidative deterioration, synthetic antioxidants such as propyl gallate (PG), butylated hydroxytoluene (BHT), and tert-butylhydroquinone (TBHQ) are widely used [16,17,18]. However, the concept of “green chemistry” has gained significant traction in the food and feed industries, promoting the use of renewable, non-toxic, and environmentally friendly additives [19,20,21]. Concurrently, both consumer and regulatory pressures for “clean-label” products have intensified, prompting a paradigm shift away from synthetic antioxidants [17]. For example, although PG is effective, concerns regarding its potential hepatotoxicity, endocrine-disrupting effects, and harmful degradation products have led to a clear preference for natural alternatives [22]. In this context, plant-derived flavonoids have emerged as promising candidates for green and clean-label antioxidant strategies, due to their natural origin, biodegradability, and favorable safety profiles [23,24].

Quercetagetin (3,3′,4′,5,6,7-hexahydroxyflavone) is a flavonol that fulfills the structural criteria for potent radical scavenging: a catechol group on the B-ring (3′,4′-dihydroxy), a 3-hydroxy group, a 4-keto moiety, a 2,3-double bond, and additional hydroxyl groups at the 5, 6, and 7 positions on the A-ring [25,26,27]. This polyhydroxylated configuration confers enhanced electron-donating capacity and superior metal-chelating ability relative to flavonoids with fewer hydroxyl groups [28,29]. In a noteworthy development, Announcement No.618 by the Ministry of Agriculture and Rural Affairs of the People’s Republic of China (2022) officially recognized a quercetagetin-rich extract (QG, ≥85% purity) as a feed additive [30], underscoring its acknowledged safety and applicability in animal nutrition [31,32,33,34]. Consequently, assessing the effectiveness of this QG-rich extract in feed-grade soybean oil under accelerated storage conditions offers a practical foundation for its use as a sustainable, clean-label antioxidant in the feed industry.

*Tagetes erecta* L. (marigold) is a rich natural source of quercetagetin, with flower extracts containing significant amounts of this polyhydroxylated flavonol. The industrial utilization of marigold extracts has primarily focused on lutein production, leaving quercetagetin as an underutilized but valuable byproduct. Converting this byproduct into a value-added antioxidant additive represents an opportunity for comprehensive utilization of plant resources, aligning with green chemistry principles.

Despite the increasing commercial interest in quercetagetin, two critical knowledge gaps remain. First, most published studies have used research-grade purified compounds, leaving the efficacy of the registered commercial-grade extract largely uncharacterized [32,34]. Second, although flavonoid antioxidants are conventionally assumed to act as broad-spectrum radical scavengers [35], recent kinetic and chemometric evidence suggests that some flavonoids may preferentially suppress specific branches of the lipid oxidation cascade [36]. Whether quercetagetin exhibits such pathway selectivity has not been directly tested.

This study addresses these gaps through six specific contributions: (1) systematic characterization of the dynamic changes in fatty acid composition during a 56-day accelerated storage period; (2) establishment of a temporal correlation between PUFA degradation and the emergence of specific volatile compounds, thereby elucidating the sequential oxidation cascade; (3) quantitative evaluation of the dose-dependent protective effects of a commercial-grade QG-rich extract (at 100 and 200 ppm) on fatty acid profiles, benchmarked against the synthetic antioxidant PG; (4) chemometric (PCA and OPLS-DA) demonstration that QG exerts a distinct inhibition pattern—rather than a non-selective one—on key volatile markers such as hexanal and hexanoic acid; (5) introduction of a quantitative selectivity index (SI) that compares the inhibition rate of each VIP volatile with the total volatile reduction, combined with a kinetic artifact analysis to support the observation of preferential—rather than non-selective—inhibition of specific oxidation endpoints; and (6) systematic structure–activity relationship (SAR) analysis comparing the differential efficacy of QG vs. PG across multiple oxidation endpoints (fatty acid preservation and volatile suppression patterns). Conventional antioxidant evaluation methods—such as peroxide value (PV), acid value (AV), or simple total volatile counts—provide only aggregate measures of oxidation inhibition and cannot distinguish between uniform (non-selective) and differential (selective) suppression of individual oxidation products. The Selectivity Index (SI) introduced here addresses this gap by quantifying, for each volatile compound, whether its inhibition by the antioxidant exceeds, equals, or falls below the average total volatile reduction, thereby providing a dimension of selectivity that is absent from conventional assays. This approach is fundamentally distinct from our prior work, which employed DPPH-HPLC-Q-TOF-MS/MS screening, PV/AV evaluation, and basic PCA/PLS-DA without VIP analysis, selectivity quantification, or kinetic artifact controls. Additionally, we optimized the headspace solid-phase microextraction (HS-SPME) extraction conditions for volatile analysis and established a chemometric evaluation workflow, providing a methodological reference for similar antioxidant efficacy studies. The use of a commercial-grade extract with 89% purity—identical to that of the registered feed additive—ensures direct industrial applicability, transforming a potential methodological limitation into a translational strength.

## 2. Results

### 2.1. Dynamic Alterations in Fatty Acid Composition During Accelerated Storage

To examine the influence of oxidation on the fatty acid profile of soybean oil, we first characterized the dynamic alterations in fatty acids within the solvent control group (CK, 0.2% ethanol, no antioxidant) over a 56-day accelerated storage period at 65 °C. A total of 13 fatty acids were consistently identified and quantified, comprising 7 saturated fatty acids (SFAs: C14:0, C16:0, C17:0, C18:0, C20:0, C22:0, C24:0) and 6 unsaturated fatty acids (UFAs: C16:1, C17:1, C18:1, C18:2, C18:3, C20:3). The predominant fatty acids were palmitic acid (C16:0), stearic acid (C18:0), oleic acid (C18:1), linoleic acid (C18:2), and α-linolenic acid (C18:3) (Table 1).

From day 0 to day 28, both SFAs and UFAs maintained relative stability, with a slight increase observed by day 28. However, a pronounced decline in all UFAs was noted between day 28 and day 56, indicative of significant oxidative degradation. The concentration of linoleic acid (C18:2), the predominant PUFA, decreased substantially from 2058.74 ± 166.02 μg/g on day 28 to 169.15 ± 120.66 μg/g by day 56. Similarly, oleic acid (C18:1) and α-linolenic acid (C18:3) decreased from 969.05 ± 71.33 μg/g and 284.17 ± 17.10 μg/g on day 28 to 477.60 ± 211.07 μg/g and 69.75 ± 11.02 μg/g on day 56, respectively. The ratio of unsaturated to saturated fatty acids (UFA/SFA) and the proportion of unsaturated fatty acids in the total fatty acid content (UFA/TFA (total fatty acids)) reached their lowest levels by day 56, with UFA/TFA declining from 83.09% on day 0 to 66.30% on day 56. These findings indicate that, by day 56, the soybean oil had undergone significant oxidative rancidity, predominantly due to the degradation of PUFAs—particularly linoleic and α-linolenic acids.

### 2.2. Impact of the Quercetagetin-Rich Extract on SFA, MUFA, and PUFA Dynamics

To assess the protective efficacy of QG, we analyzed the contents of SFAs, monounsaturated fatty acids (MUFAs), and preserved polyunsaturated fatty acids (PUFAs) across treatment groups (CK: solvent control; PG200: propyl gallate at 200 ppm; QG100: quercetagetin-rich extract at 100 ppm; QG200: quercetagetin-rich extract at 200 ppm) over the oxidation period (Table 2 and Supplementary Materials). The antioxidant properties of the extracts were most effective in preserving PUFAs, which are particularly vulnerable to oxidative degradation. By day 56, the PUFA concentration in the CK group had decreased significantly to 239.93 ± 132.76 μg/g. In contrast, the QG200 group maintained substantially higher PUFA levels at 1254.83 ± 88.54 μg/g, approximately 5.2-fold greater than the control (*p* < 0.01, false discovery rate (FDR)-corrected). The positive control PG200 demonstrated the strongest overall protective effect, maintaining PUFA levels of 1917.84 ± 108.51 μg/g. These results confirm that the QG-rich extract effectively mitigates oil oxidation, primarily by preventing PUFA degradation. Notably, QG200 represents the highest concentration that remained completely soluble in soybean oil, providing substantial and statistically significant protection.

### 2.3. Preservation of Key Unsaturated Fatty Acids by QG

**Figure A1:**
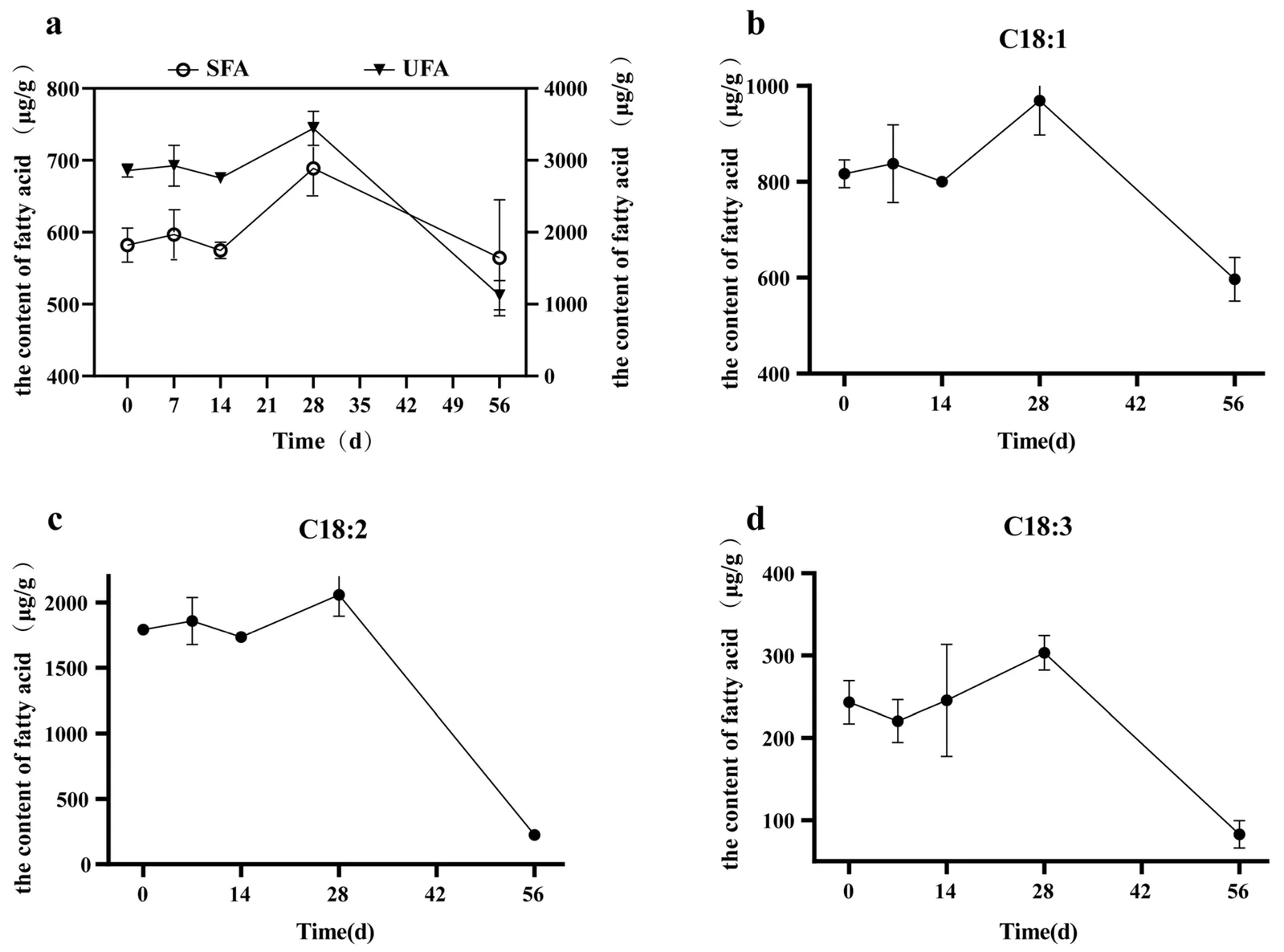
Changes in fatty acids of soybean oil during different accelerated storage periods. (**a**) Changes in total SFAs and UFAs; (**b**) changes in oleic acid (C18:1); (**c**) changes in linoleic acid (C18:2); (**d**) changes in α-linolenic acid (C18:3).

To further delineate the protective effects on the major unsaturated fatty acids C18:1, C18:2, and C18:3, their concentrations were monitored over 56 days (Figure A1). The most significant differences were observed in C18:2 levels. All experimental groups initially exhibited an increase, followed by a marked decline after 28 days. By day 56, the CK group retained only 169.15 ± 120.66 μg/g of C18:2, whereas the QG200 group maintained substantially higher levels (1127.57 ± 106.03 μg/g), indicating a strong preservation effect (*p* < 0.01 vs. CK). The PG group exhibited the highest retention of C18:2 (1730.52 ± 100.32 μg/g on day 56) and effectively maintained C18:3 levels throughout the storage period, with concentrations remaining stable from 171.79 ± 23.82 μg/g on day 0 to 178.92 ± 3.59 μg/g on day 56 (Table A1), confirming PG’s broad-spectrum protective capacity across all major PUFAs.

**Figure 1:**
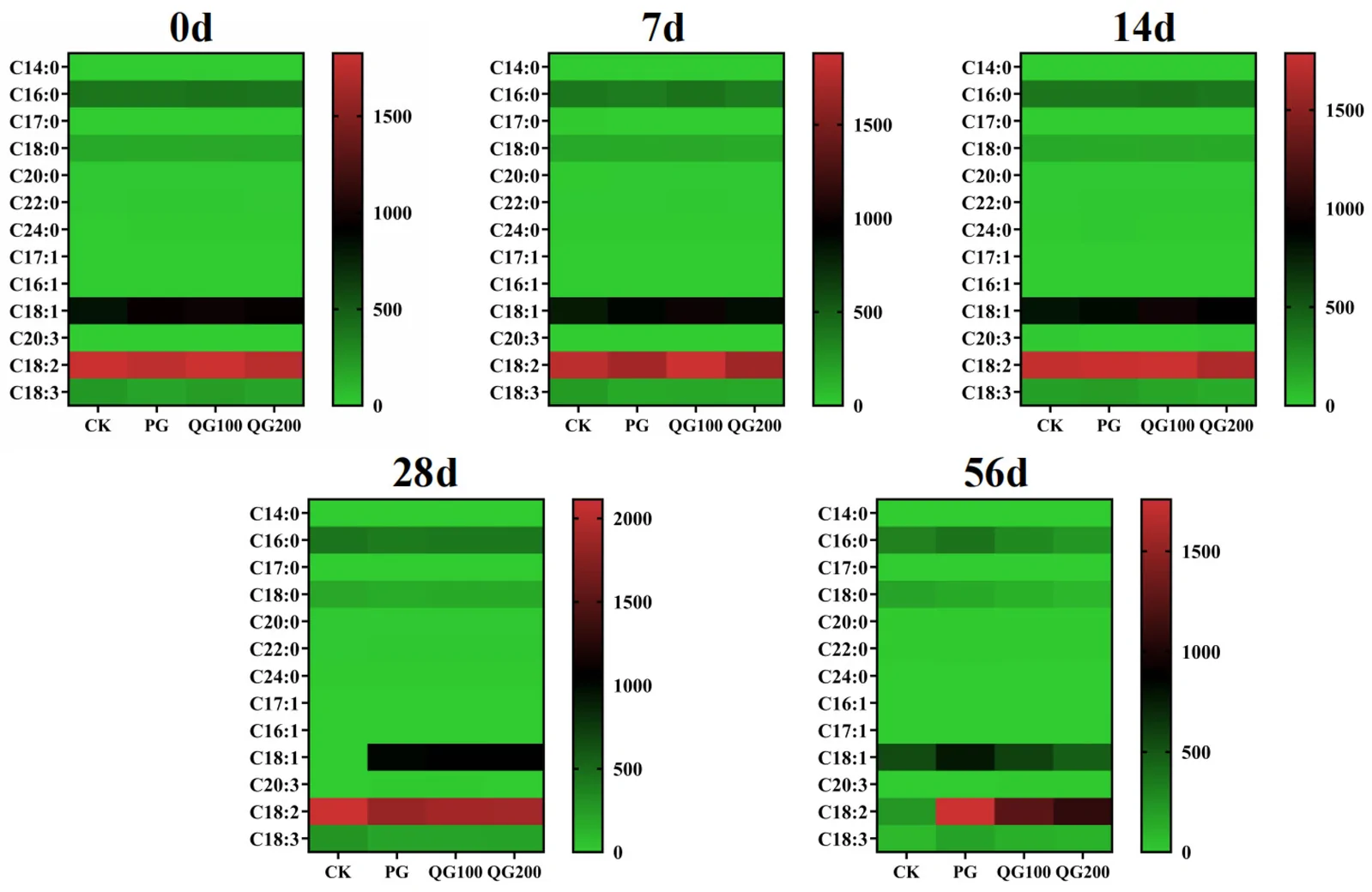
Heatmap of fatty acid changes. Changes in fatty acid composition and content of soybean oil across treatments (CK, PG, QG100, QG200) during accelerated storage (days 0, 7, 14, 28, 56).

For C18:3, the most oxidation-susceptible fatty acid, the CK group exhibited a gradual decline from 239.89 ± 25.57 μg/g on day 0 to 69.75 ± 11.02 μg/g by day 56. In contrast, the QG200 group maintained significantly higher C18:3 levels throughout, retaining 119.75 ± 23.10 μg/g on day 56. Notably, the PG group maintained the most stable C18:3 levels throughout storage (171.79 ± 23.82 μg/g on day 0 to 78.92 ± 3.59 μg/g on day 56), demonstrating superior protection of this highly susceptible fatty acid. These patterns are corroborated by the heatmap of fatty acid composition changes across treatments and time points (Figure 1), which distinctly separates antioxidant-treated samples from CK, especially at advanced oxidation stages. Together, these findings demonstrate that QG, particularly at 200 ppm, effectively preserves the critical unsaturated fatty acids—most notably C18:2 and C18:3—during extended oxidation, surpassing the unsupplemented control. The data indicate a dose- and time-dependent protective effect of QG against oxidative degradation of PUFAs in soybean oil, underscoring its potential as a natural antioxidant for oil stabilization.

### 2.4. Optimization of HS-SPME Extraction Conditions

**Figure 2:**
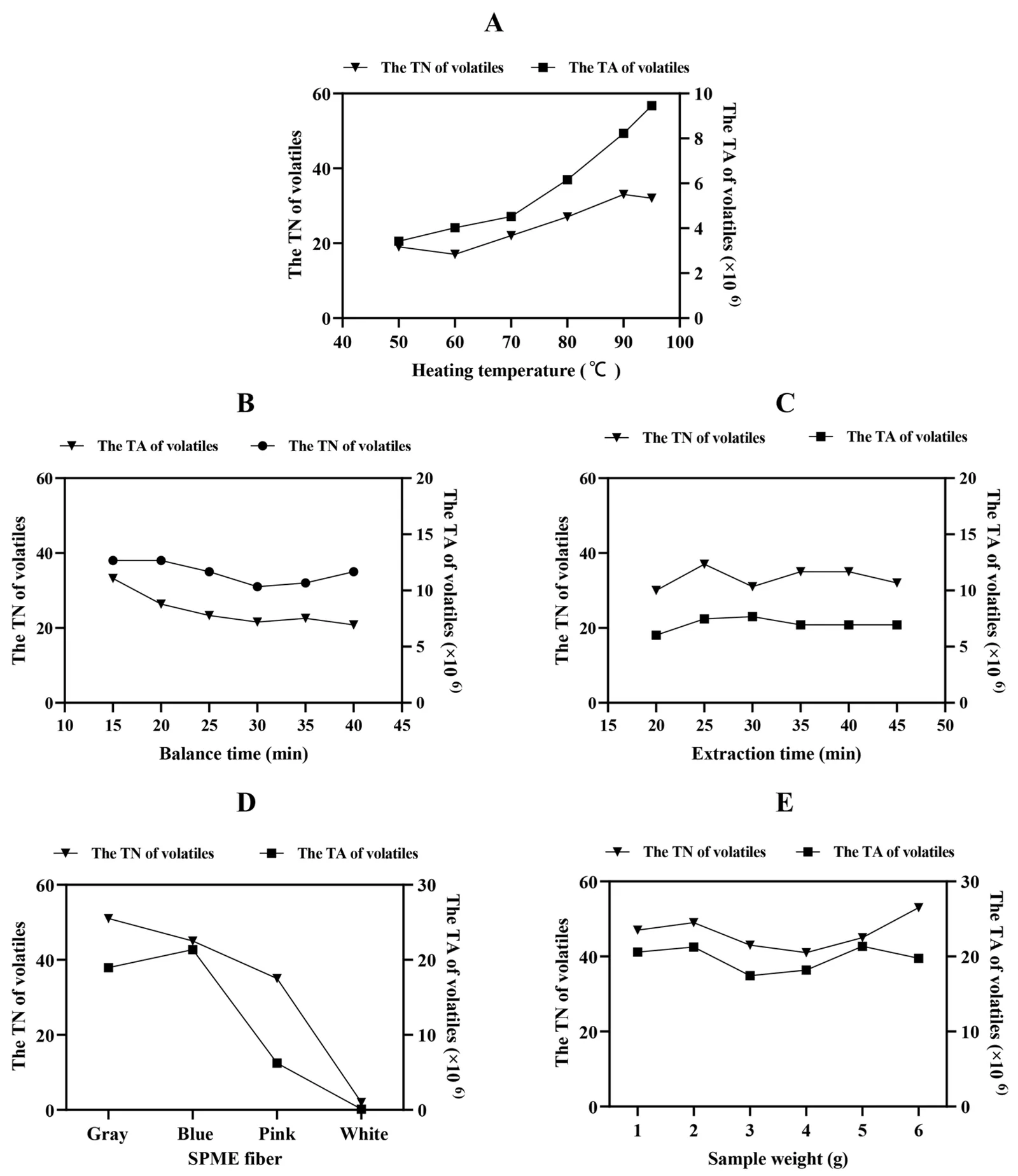
Effects of HS-SPME extraction parameters on the number (TN) and total peak area (TA) of volatile components. (**A**) Heating temperature; (**B**) balance time; (**C**) extraction time; (**D**) SPME fiber; (**E**) sample weight.

The optimal conditions for HS-SPME extraction were as follows: 2.0000 g of soybean oil in a 15 mL headspace vial, equilibrated at 95 °C for 15 min, and extracted for 35 min using a 50/30 μm DVB/CAR/PDMS fiber (Gray). The superior performance of the Gray fiber (50/30 μm DVB/CAR/PDMS) can be attributed to its biphasic adsorbent coating, which combines Carboxen (polar, for small molecules) and divinylbenzene (non-polar, for larger molecules) on a polydimethylsiloxane backbone, thereby capturing the broadest range of volatile compounds (Figure 2).

### 2.5. Evolution of Volatile Compounds During Soybean Oil Oxidation

**Figure 3:**
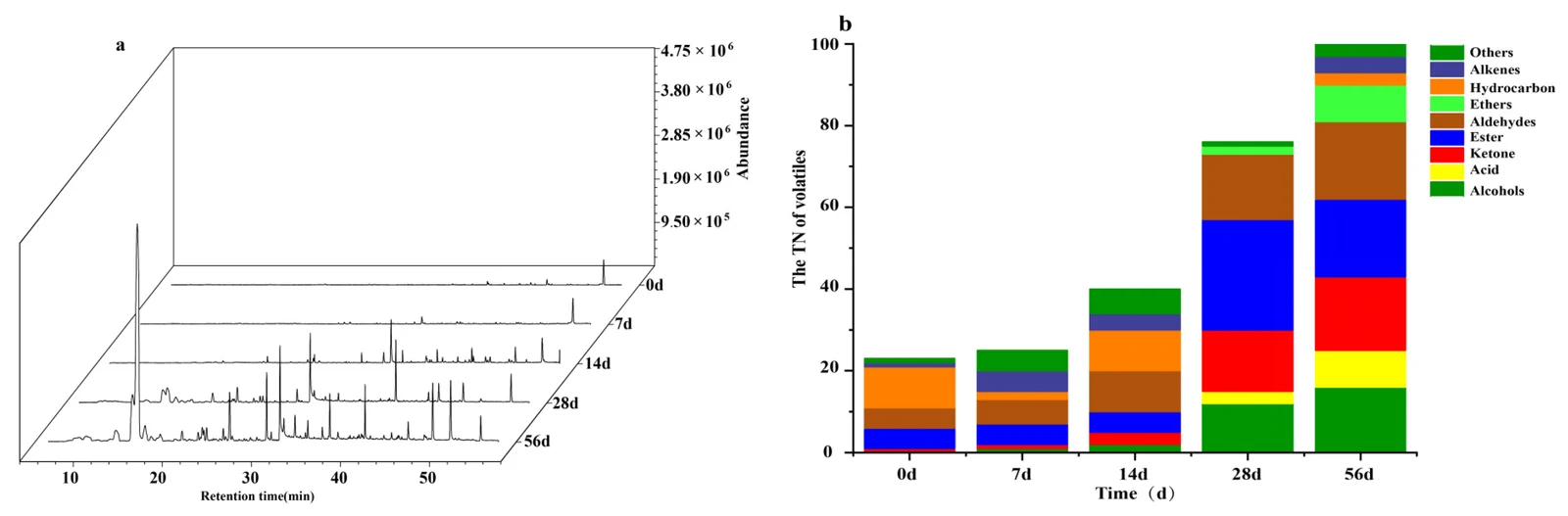
Changes in volatile compound classes during accelerated storage. (**a**) Total ion current chromatograms of volatile compounds at different oxidation stages; (**b**) stacked bar chart showing the classification and relative abundance of volatile substances, illustrating the changing proportion of each class over time.

Throughout the 56-day accelerated oxidation period, a total of 153 volatile compounds were tentatively identified in the control group (Figure 3a; see Table A2 for the full list). These compounds were classified into nine chemical categories: alcohols (25), ethers (9), aldehydes (22), acids (8), ketones (23), alkanes (13), alkenes (7), esters (32), and miscellaneous compounds (14). Figure 3b illustrates the substantial alterations in the volatile profile over time. Secondary oxidation products—comprising aldehydes, acids, esters, alcohols, and ketones—constituted over 90% of the total volatiles and exhibited significant accumulation as oxidation advanced (*p* < 0.05), particularly after day 28. The stacked bar chart vividly demonstrates the dynamic changes in the relative abundance of each volatile class, with aldehydes and acids showing the most pronounced increases at later stages. This accumulation temporally coincided with the marked decline in PUFAs (Section 2.1), thereby confirming the sequential relationship between PUFA degradation and the formation of secondary volatiles.

### 2.6. Dose-Dependent Inhibition of Volatile Compounds by QG

**Figure 4:**
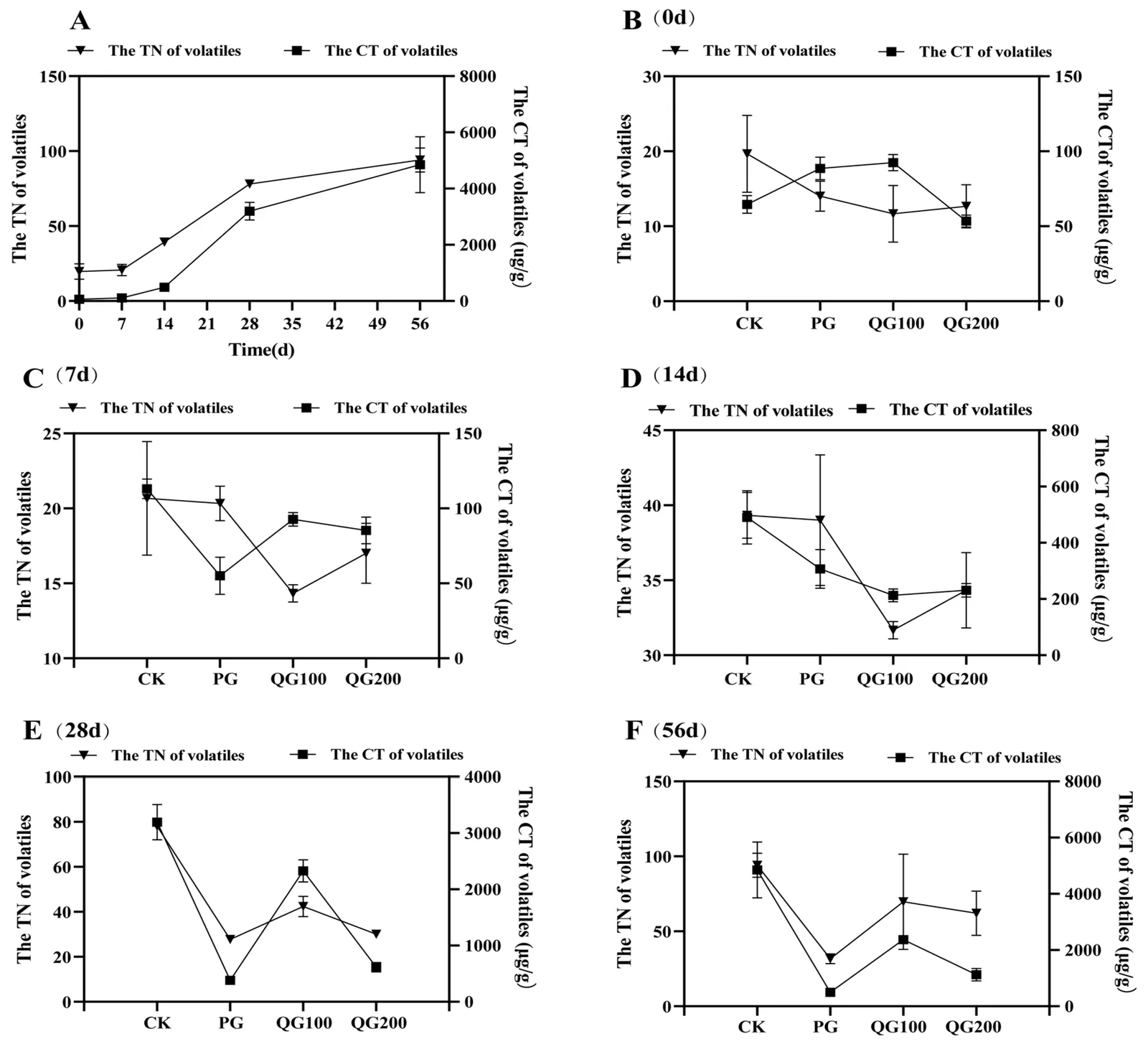
Effects of different oxidation periods and antioxidant treatments on the number of volatile substances (TN) and total content (CT, μg/g) in soybean oil. (**A**) Changes in TN and CT from Day 0 to Day 56; (**B**) Day 0; (**C**) day 7; (**D**) day 14; (**E**) day 28; (**F**) day 56. Bars represent the mean ± standard deviation (SD) (*n* = 3) of TN (left axis) and CT (right axis).

The administration of QG200 resulted in a significant reduction in both the number and total concentration of volatile compounds (Figure 4). Relative to the control, samples treated with 200 ppm QG demonstrated a marked decrease in volatile compounds across all oxidation time points. Although the inhibitory effect of QG was marginally less pronounced than that of PG, it remained highly efficacious.

### 2.7. Impact on Major Classes of Volatile Compounds

**Figure 5:**
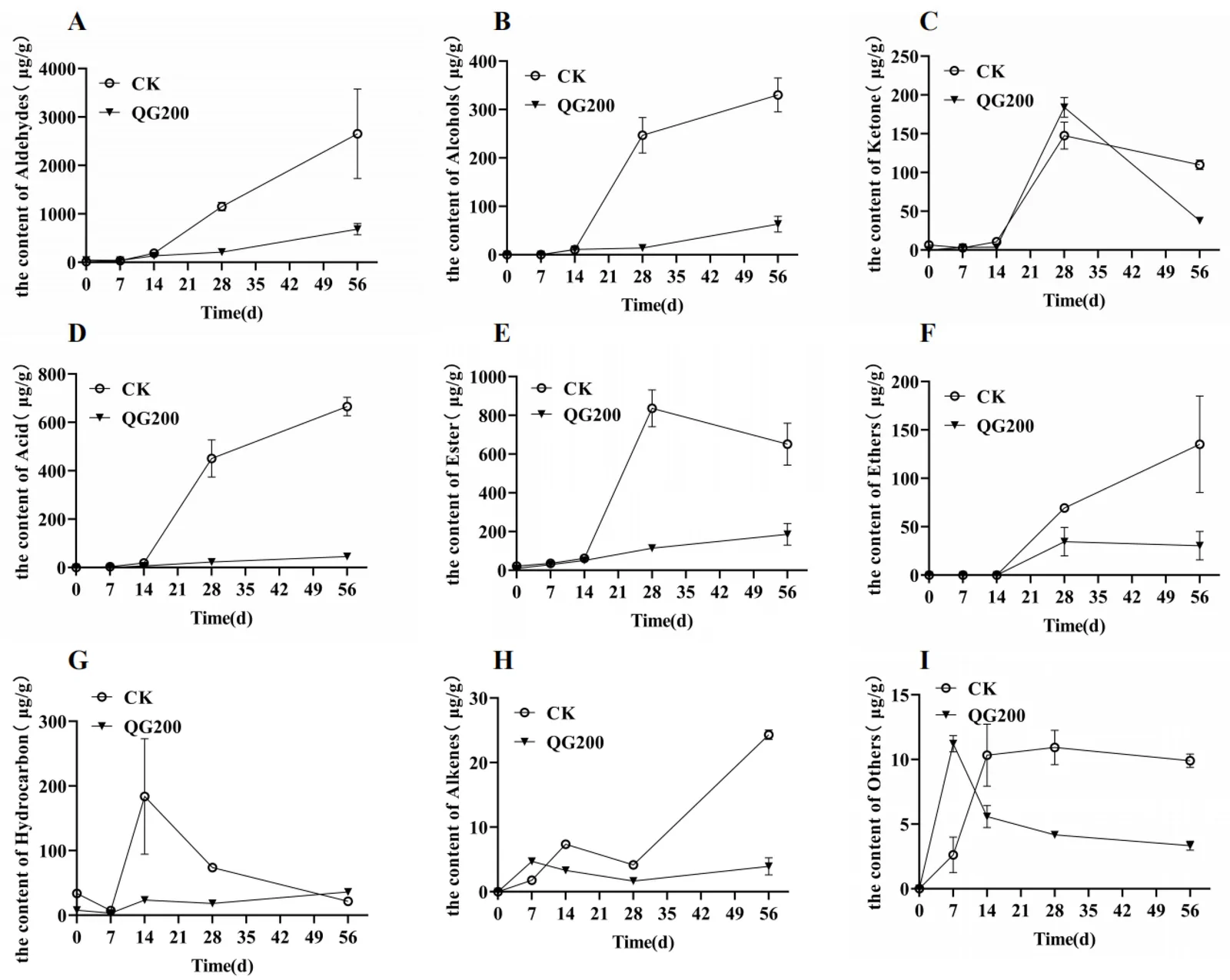
Changes in different classes of volatile substances at various oxidation stages. (**A**) Aldehydes; (**B**) alcohols; (**C**) ketones; (**D**) acids; (**E**) esters; (**F**) ethers; (**G**) hydrocarbon; (**H**) alkenes; (**I**) others.

The application of QG resulted in a significant suppression of the accumulation of all major classes of volatile compounds (Figure 5). After 56 days of oxidation, the concentrations of aldehydes, alcohols, ketones, acids, and esters in the QG200-treated group were reduced by averages of 74.24%, 80.89%, 65.65%, 100%, and 69.27%, respectively, compared to the control group (*p* < 0.01). The most pronounced reduction was observed in acids (100%), followed by alcohols (80.89%) and aldehydes (74.24%). These results indicate that QG is highly effective in suppressing the entire cascade of secondary volatile formation resulting from lipid oxidation. PG200 exerted an even stronger suppressive effect, with reductions of 92.3%, 91.5%, 88.2%, 96.8%, and 89.1% for the same five classes, respectively.

### 2.8. Multivariate Analysis Reveals Selective Inhibition by QG

**Figure 6:**
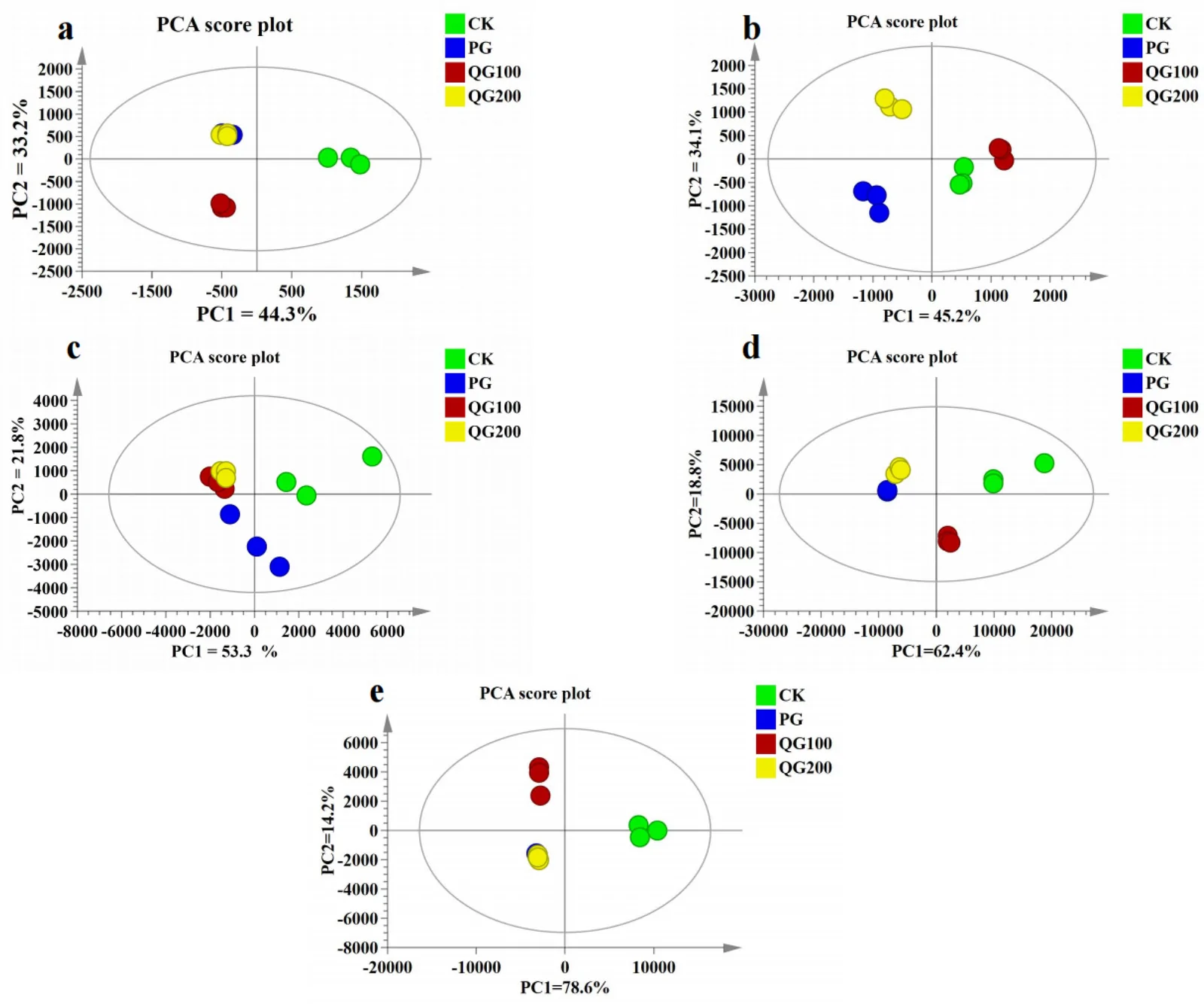
Principal component analysis (PCA) score plots of volatile profiles under different treatments (CK, QG200, PG200) across accelerated storage periods (days 0, 7, 14, 28, and 56). The (**a**–**e**) adjacent to the data points denote storage days of 0, 7, 14, 28, and 56, respectively. Percentages in parentheses indicate the variance explained by each principal component.

To investigate whether QG functions as a non-selective scavenger or as a selective regulator of volatile oxidation products, an orthogonal partial least squares discriminant analysis (OPLS-DA) was performed on the GC-MS volatile compound datasets collected on days 28 and 56, comparing the CK and QG200 groups. The principal component analysis (PCA) score plots (Figure 6) revealed a distinct separation of samples based on both treatment type and storage duration. Importantly, the QG200 group clustered closely with the positive control PG200, particularly at advanced oxidation stages (days 28 and 56), suggesting a similar overall modulation of the volatile profile.

**Figure 7:**
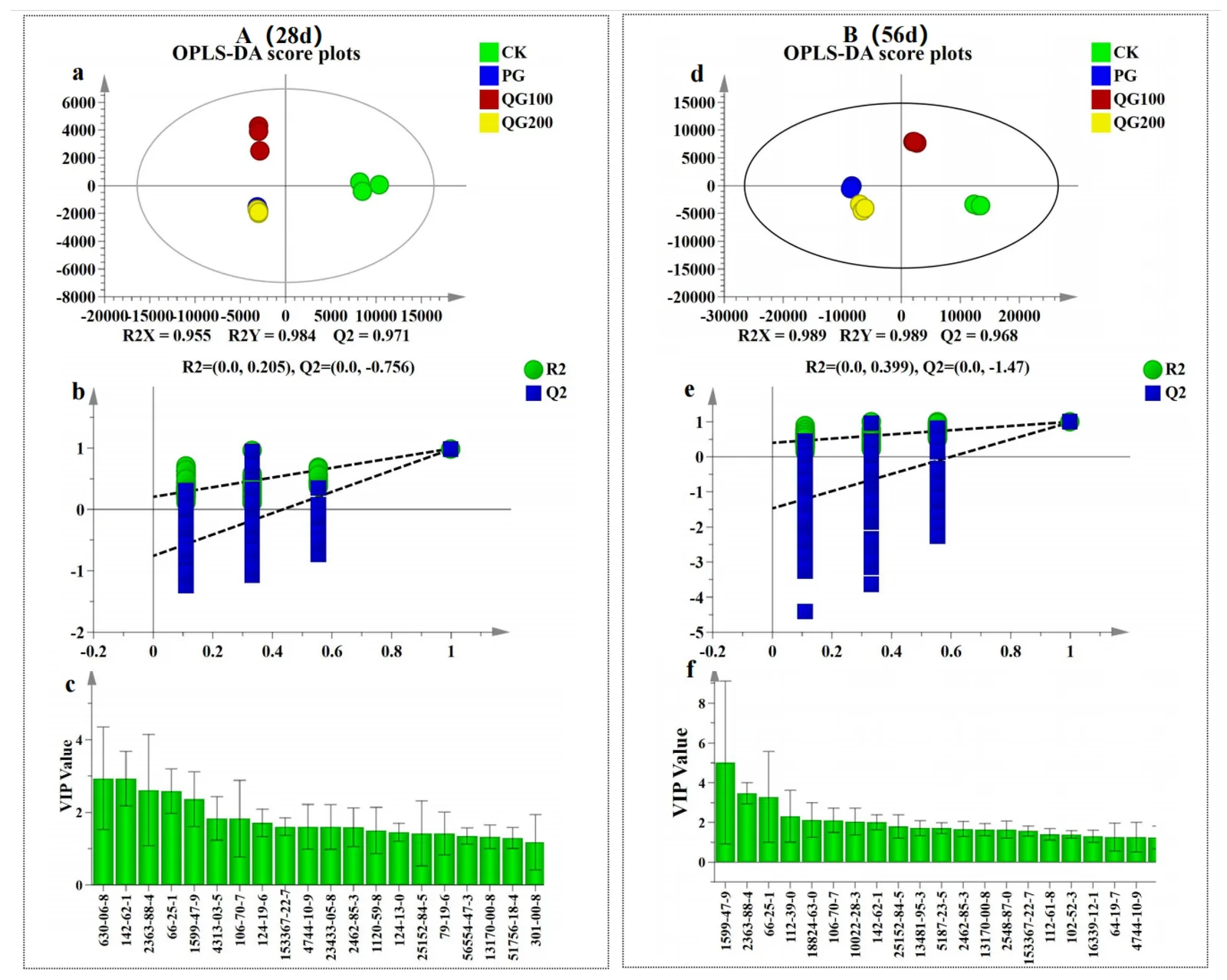
Orthogonal partial least squares discriminant analysis (OPLS-DA) of volatile compounds (CK vs. QG200, day 56). (**a**) OPLS-DA score plot (Day 28); (**b**) permutation test (200 iterations) (Day 28); (**c**) VIP scores (Day 28); (**d**) OPLS-DA score plot (Day 56); (**e**) permutation test (200 iterations) (Day 56); (**f**) VIP scores (Day 56) identifying key volatile compounds responsible for group discrimination.

OPLS-DA was then used to pinpoint the specific volatile compounds primarily responsible for the differentiation between CK and QG200. The OPLS-DA model on day 28 demonstrated excellent fit and predictive capability, with R^2^X = 0.955, R^2^Y = 0.984, and Q^2^ = 0.971 (Figure 7a). Following 200 permutation tests, the Q^2^ regression line intercept was negative (Q^2^-intercept = −0.756), confirming model robustness and the absence of overfitting (Figure 7b). The OPLS-DA model on day 56 demonstrated excellent fit and predictive capability, with R^2^X = 0.989, R^2^Y = 0.989, and Q^2^ = 0.968 (Figure 7d). Both R^2^Y and Q^2^ values exceeded 0.9, indicating a robust model. Following 200 permutation tests, the Q^2^ regression line intercept was negative (Q^2^-intercept = −1.47), confirming model robustness and the absence of overfitting (Figure 7e). The OPLS-DA model was built on 6 observations (3 CK + 3 QG200, day 56) with 153 X-variables (volatile compounds). Cross-validation was performed using the default 7-fold method in SIMCA 14.1. The model utilized 1 predictive component and 2 orthogonal components. VIP stability was assessed by comparing VIP rankings between the 28-day and 56-day models (Figure 7c,f).

### 2.9. Quantitative Selectivity Index and Temporal Dynamics of Key Volatile Compounds

Compounds such as hexanal dimethyl acetal (VIP score increased from 2.36 to 5.01), (*E*,*E*)-2,4-decadienal (from 2.61 to 3.47), and hexanal (from 2.58 to 3.28) demonstrated significant increases in VIP scores over time, highlighting their role as late-stage discriminators. The dramatic VIP rise of hexanal dimethyl acetal (2.36 → 5.01) is consistent with its nature as an acetal derivative of hexanal, formed via aldehyde–methanol interactions that intensify as oxidation progresses and methanol becomes available from methyl ester hydrolysis; its accumulation thus mirrors the escalating production of its aldehyde precursor. Similarly, (*E*,*E*)-2,4-decadienal, a well-established hallmark decomposition product of linoleic acid hydroperoxide fragmentation via the 13-hydroperoxide pathway, further corroborates the driving role of PUFA autoxidation in shaping the late-stage volatile profile. Hexanoic acid (VIP score decreased from 2.93 to 2.01) and nonanal (from 1.71 to 1.24) exhibited declining VIP scores, suggesting greater discriminatory power at earlier stages. The dynamic profiles demonstrate that QG200 selectively suppresses distinct volatile classes at various oxidation stages, with the most significant effects observed during the late stage (day 56) when secondary oxidation products accumulate.

To quantitatively demonstrate this selectivity, we defined a Selectivity Index (SI) for each VIP-identified volatile on day 56 as:

**SI = Inhibition rate of the compound/Average inhibition rate of total volatiles**

The average reduction in total volatile compounds was 65.7%. In contrast, hexanal exhibited an inhibition rate of 74.24% (SI = 1.13), hexanoic acid exhibited an inhibition rate of 100% (SI = 1.52), and (*E*)-2-nonenal exhibited an inhibition rate of 81.2% (SI = 1.24) (Table 3). SI > 1 indicates that the compound is preferentially suppressed relative to the average, and SI < 1 indicates that the compound is suppressed less than the average (i.e., the antioxidant is relatively permissive for that compound). Among the 12 VIP volatiles, ten showed SI > 1 (one showed SI = 1.00 and one showed SI < 1), The SI is presented as an exploratory descriptive metric.

To exclude the possibility that the observed selectivity is merely a kinetic artifact, we calculated the formation rates of selected volatile compounds between days 28 and 56 in the control group, and compared these rates with the inhibition rates observed in QG200. Hexanal, which exhibited high selectivity (SI = 1.13), had a formation rate of 38.5 μg/g/day, whereas a non-selective compound such as 2-heptanone (SI ≈ 1.0) had a rate of 12.3 μg/g/day. The selective inhibition by QG (74.24% for hexanal vs. 65.65% for 2-heptanone) was not directly proportional to these formation rates. If the inhibition were purely kinetic, one would expect higher formation rates to yield lower inhibition (more substrate to scavenge); the opposite is observed for hexanal. This supports the presence of preferential—rather than non-selective—inhibition of specific oxidation endpoints rather than a kinetic artifact.

## 3. Discussion

### 3.1. Overview of Key Findings

This study shows that a commercial-grade quercetagetin-rich extract (QG, 89% purity) effectively preserves PUFAs and inhibits secondary volatile formation in soybean oil under accelerated storage. The QG-rich extract operates through two complementary mechanisms: (i) protecting oxidation-prone PUFAs, particularly C18:2 and C18:3, from degradation and (ii) preferentially suppressing specific volatile rancidity markers, including hexanal. The principal innovation is the chemometric demonstration—via OPLS-DA, a quantitative SI, and a kinetic artifact analysis—that the QG-rich extract does not function as a non-selective radical scavenger but exhibits a distinct inhibition pattern. This preferential regulation is substantiated by multivariate analysis and quantitative comparison of inhibition rates against total volatile reduction, though the detailed molecular mechanism warrants further investigation.

### 3.2. Structure–Activity Relationships of Quercetagetin

The significant antioxidant activity of QG can be rationalized through the well-established structure–activity relationship (SAR) of flavonoids [37]. Previous studies have demonstrated that flavonoids with polyhydroxylated substitutions on both the A and B rings, a 2,3-double bond, a free 3-hydroxyl substitution, and a 4-keto moiety exhibit significant antiperoxidative properties [26,38]. Quercetagetin (3,3′,4′,5,6,7-hexahydroxyflavone) fulfills all of these structural criteria, including the catechol group on the B-ring (3′,4′-dihydroxy), a 3-hydroxy group, a 4-keto moiety, a 2,3-double bond, and additional hydroxyl groups at the 5, 6, and 7 positions on the A-ring. This polyhydroxylated configuration endows quercetagetin with enhanced electron-donating capacity and superior metal-chelating ability relative to flavonoids with fewer hydroxyl groups [29,39].

The contribution of each hydroxyl group to antioxidant activity has been systematically dissected by Sekher, who showed that the antioxidant efficacy of flavonols such as quercetin is significantly influenced by the presence of free hydroxyl groups at the 3, 5, 7, 3′, and 4′ positions, whereas glycosylation generally diminishes this activity [40,41]. The 6-OH group, unique to quercetagetin among common dietary flavonols, provides an additional electron-donating site and may participate in the chelation of pro-oxidant metals (e.g., Fe^2+^, Cu^+^) [42,43,44]. Our results with the QG-rich extract, which retains all these hydroxyl groups, corroborate this SAR and explain its robust protection of PUFAs and selective suppression of volatile oxidation markers in soybean oil. We note that the SAR analysis is based on the major component (quercetagetin, 89%). The minor flavonoids (patuletin, quercetin) also possess multiple hydroxyl groups and may contribute to the overall antioxidant activity. Without testing purified quercetagetin alongside the extract and individual minor components, we cannot definitively attribute the observed selectivity solely to quercetagetin.

In our previous research on marigold (*Tagetes erecta* L.) extracts, we established that quercetagetin is the primary antioxidant component. The biological effects of pure quercetagetin have also been described recently [45], supporting its potential as a b*i*oactive compound beyond antioxidant applications. It displayed the most significant DPPH radical-scavenging activity among four identified flavonoids: quercetagetin-7-*O*-glucoside, quercetagetin, quercetin, and patuletin [44]. That study also demonstrated the efficacy of quercetagetin in inhibiting the production of oxidation products in soybean oil, as evidenced by reductions in both peroxide and acid values. The current study builds upon those findings by offering a comprehensive analysis of the temporal dynamics of fatty acid degradation and volatile compound formation, by introducing a chemometric demonstration of selective inhibition, and by benchmarking QG against the synthetic antioxidant PG.

### 3.3. Comparison with Synthetic Antioxidants: Differential Protection of PUFAs

A notable finding is the distinct efficacy profiles of QG vs. PG in protecting PUFAs and suppressing volatile oxidation products. PG, as a synthetic antioxidant, demonstrated superior overall preservation of total PUFAs—particularly linoleic acid (C18:2, 1730.52 ± 100.32 μg/g on day 56)—and effectively maintained C18:3 levels throughout storage (171.79 ± 23.82 μg/g on day 0 to 178.92 ± 3.59 μg/g on day 56), confirming its broad-spectrum radical-scavenging capacity. In contrast, while QG200 provided significant PUFA protection relative to the control (*p* < 0.01), it was less effective than PG in preserving total PUFA levels, including C18:3 (119.75 ± 23.10 μg/g on day 56 vs. 178.92 ± 3.59 μg/g for PG). However, the SI analysis reveals that QG exhibits a selective volatile suppression pattern (SI > 1 for 10 of 12 VIP compounds) that is qualitatively distinct from PG’s broad-spectrum protection. This suggests that while PG provides superior overall radical scavenging, QG’s mechanism may involve preferential interception of specific hydroperoxide decomposition pathways—a dimension of antioxidant selectivity not captured by conventional PUFA preservation metrics alone. The complementary protection profiles of QG and PG have practical implications: in applications where targeted suppression of specific rancidity-associated volatiles (e.g., hexanal, (*E*)-2-nonenal) is critical, QG may offer advantages despite lower overall PUFA preservation [26,46].

### 3.4. Selective Inhibition of Volatile Oxidation Markers: Mechanistic Insights

The principal innovation of this study lies in the observation that QG distinctly inhibits specific volatile oxidation products, as opposed to exerting a non-selective inhibitory effect. The OPLS-DA analysis identified hexanal (VIP = 3.28, SI = 1.13), hexanoic acid (VIP = 2.01, SI = 1.52), and (*E*)-2-nonenal (VIP = 1.80, SI = 1.24) as the most significantly suppressed compounds, all with SI > 1, confirming preferential suppression relative to the total volatile reduction. This pattern of selectivity implies that QG may specifically interfere with certain decomposition pathways of hydroperoxide isomers, rather than indiscriminately scavenging all radical species. We emphasize that the SI and VIP analyses provide evidence of unequal endpoint changes—i.e., that certain volatile compounds are suppressed more than others. This is consistent with, but does not directly establish, pathway-specific inhibition at the mechanistic level. The mechanistic interpretations presented here should be considered hypotheses warranting further investigation. Direct evidence would require analysis of hydroperoxide isomer profiling.

Hexanal is a primary degradation product resulting from the homolytic β-scission of the 13-hydroperoxide of linoleic acid [47,48]. The preferential suppression of hexanal by QG (SI = 1.13) indicates that QG may either (1) selectively inhibit the formation of the 13-hydroperoxide isomer or (2) intercept the decomposition of this specific hydroperoxide before it cleaves into hexanal. The temporal correlation observed—a marked decline in linoleic acid between days 28 and 56, coinciding precisely with the increase in hexanal—supports the hypothesis that QG acts at the hydroperoxide-decomposition stage. Recent work has shown that quercetin effectively suppresses aldehyde formation in thermally treated soybean oil by trapping early lipid oxidation intermediates, such as 13-oxo-octadecadienoic acid, thereby preventing their decomposition into volatile carbonyl compounds [49]. Considering the structural similarity between quercetin and quercetagetin (which differ solely by the presence of an additional hydroxyl group at the C-6 position), it is reasonable to hypothesize that quercetagetin exhibits a comparable intermediate-trapping mechanism [50]. The additional 6-OH may augment its electron-donating capability and enhance its capacity to form stable adducts with reactive carbonyl species.

The selectivity pattern observed for the other major VIP volatiles further supports the pathway-level selectivity hypothesis. The high SI for hexanoic acid (1.52) suggests that QG intercepts not only the aldehyde-forming β-scission but also the secondary oxidation of aldehydes to their corresponding acids, possibly via the Fenton-type hydroxyl radical generation that is itself modulated by the metal-chelating activity of the 6-OH group [51]. The moderate SI for (*E*)-2-nonenal (1.24)—a downstream oxidation product of oleic acid—indicates that QG’s protective effect extends beyond the linoleic acid cascade to other UFA-derived volatiles.

### 3.5. Comparison with Non-Flavonoid Antioxidants: Mechanistic Implications

The kinetic partitioning effect observed here is conceptually pertinent to recent comparative kinetic studies on flavonoid–α-tocopherol interactions. Olicheva et al. demonstrated that dihydroquercetin, quercetin, rutin, and morin exhibit either additive or mildly subadditive antioxidant interactions with α-tocopherol, with no indication of synergistic enhancement [52]. Notably, their lag-time assay revealed a biphasic radical-scavenging pattern, wherein α-tocopherol predominated during the initial phase, while flavonoids became active in the subsequent phase [53]. This suggests that lipophilic antioxidants may preferentially protect flavonoids from premature oxidation in polar media. In the present study, the biphasic degradation pattern observed in the CK group—where PUFA levels remained relatively stable during the lag phase (0–28 d) followed by a sharp decline from day 28 onward—is consistent with such kinetic partitioning [54].

### 3.6. Translational Relevance: From Natural Product Chemistry to Practical Applications

The targeted suppression of hexanal by QG holds significant implications for animal feed applications. Hexanal not only serves as an indicator of rancidity but is also linked to respiratory and inflammatory problems in livestock [14,34]. Recent research has shown that the supplementation of broiler diets with quercetagetin at 20 ppm under high-stocking-density conditions markedly reduces oxidative stress, evidenced by lower serum levels of corticosterone (CORT) and adrenocorticotropic hormone (ACTH), decreased pro-inflammatory cytokines (IL-1β and IL-6), and improved liver antioxidant enzyme activities (GSH-Px and T-SOD) [34]. These effects are ascribed to QG’s capacity to scavenge excessive reactive oxygen species and mitigate cellular oxidative damage. Notably, the preferential suppression of (*E*)-2-nonenal (SI = 1.24) by the QG-rich extract is of particular toxicological significance. (*E*)-2-Nonenal is a well-documented cytotoxic α,β-unsaturated aldehyde that can cause cellular damage through protein adduction and oxidative stress. It is a major contributor to the “aged” or “cardboard” off-flavor in oxidized oils and has been implicated in age-related diseases. The selective suppression of this compound, beyond its sensory implications, suggests a potential health-protective dimension of the QG-rich extract. Future studies should specifically quantify the most toxicologically significant aldehydes (e.g., 4-hydroxy-2-nonenal, malondialdehyde, acrolein) and assess whether the QG-rich extract exhibits selectivity toward these compounds.

Moreover, our in vivo findings further substantiate the translational potential of QG. In a broiler model subjected to H_2_O_2_-induced oxidative stress, dietary supplementation with QG (100 ppm) significantly alleviated the deterioration of meat quality by reducing reactive oxygen species (ROS) and oxidation products while enhancing antioxidant enzyme activities and upregulating the Nrf2 signaling pathway in breast muscle [33]. These in vivo observations are consistent with our current in vitro results, wherein QG preserved PUFAs and selectively suppressed volatile aldehydes such as hexanal. Collectively, QG mitigates lipid oxidation through integrated redox regulation and targeted suppression of specific oxidative markers, reinforcing its potential as an effective feed additive to combat oxidative rancidity and enhance the quality of animal products.

The commercial significance of our findings is highlighted by the fact that the QG-rich extract used in this study, with a purity of 89%, is identical to a product already registered as a feed additive in China (MOA Announcement No. 618, 2022) [13]. The specific technical requirements for this feed additive, including the minimum content of quercetagetin (≥80.0% on dry basis) and the maximum limits for moisture (≤5.0%), crude ash (≤1.0%), total arsenic (≤3.0 ppm), and lead (≤3.0 ppm), as well as the standardized analytical methods for identification and quantification, are comprehensively specified in the product standard NYSL-1003-2022. Consequently, our study assesses the practical efficacy of a product with industrial relevance, rather than a research-grade pure compound, thereby ensuring direct applicability to the feed industry.

From an application perspective, the solubility limit of QG in soybean oil (complete dissolution up to 200 ppm) defines a practical upper bound for its use as a direct additive. The 200 ppm concentration evaluated in this study provided substantial protection—approximately 5.2-fold greater PUFA preservation and >74% reduction in aldehydes compared to the control—within the fully soluble range, making it a practical reference point for industrial formulation. Compared to PG, QG offers regulatory advantages as it has already been approved for use in feed in China, while PG faces increasing consumer and regulatory pressure due to safety concerns. The clean-label compatibility and natural origin of QG further align with current market trends favoring plant-derived additives. These factors collectively position QG as a viable, sustainable alternative to synthetic antioxidants in feed applications.

### 3.7. Methodological Considerations and Study Limitations

Several methodological considerations merit discussion. First, the Schaal oven test at 65 °C offers a rapid and controlled environment for evaluating antioxidant efficacy [55], but it may not accurately replicate the complex kinetics of long-term storage at ambient temperatures. Although the Q10 rule-of-thumb suggests that 56 days at 65 °C approximately equates to 12–15 months at 25 °C [56], this remains an estimation. Future validation under real-time storage conditions (e.g., 25 °C and 40 °C over 12 months) is essential to ascertain practical applicability. Second, while our volatile analysis demonstrated significant reductions in key odor-active compounds (hexanal, 1-octen-3-ol), we did not conduct gas chromatography–olfactometry (GC-O) or quantitative sensory evaluations with consumer panels [57]. GC-O is recommended for future work. Third, the solubility constraints of QG in soybean oil (complete dissolution at ≤200 ppm, turbidity at 300 ppm, precipitation at ≥400 ppm) limit the maximum feasible concentration; future research should investigate solubility enhancers, emulsifiers, or encapsulation technologies. Fourth, the impurities (~10.8% other flavonoids such as patuletin and quercetin) reflect the commercial reality of the registered feed additive and augment translational relevance; subsequent studies should examine the intrinsic activity of ≥98% pure quercetagetin alongside the extract to deconvolute individual contributions. Fifth, while OPLS-DA, SI, and kinetic artifact analyses provide strong indirect evidence of preferential regulation, direct chemical evidence—EPR trapping of radical intermediates and density functional theory (DFT) calculation of bond dissociation enthalpy (BDE)/ionization potential (IP) at each hydroxyl site [58,59]—is warranted in future studies.

Additional limitations relevant to the methodology and practical application include: (1) HS-SPME extraction at 95 °C may induce thermal decomposition of hydroperoxide intermediates, potentially generating artifactual volatiles that do not reflect the true volatile profile at ambient storage temperatures. While this temperature was optimized for maximum extraction efficiency, future studies should consider lower extraction temperatures or on-fiber derivatization to minimize hydroperoxide decomposition artifacts. (2) The Selectivity Index (SI) has not been formally validated: it has not been compared with existing selectivity metrics, no sensitivity analysis has been performed to assess how SI values are affected by changes in the volatile dataset, the SI is inherently dependent on the choice of the denominator (average total volatile reduction), and the SI does not account for the absolute concentration of each compound—a compound with a high SI but very low absolute concentration may be less practically significant than one with a moderate SI but high absolute concentration. (3) ANCOVA was not conducted to assess whether confounding variables (e.g., compound volatility, molecular weight, boiling point) influenced the PCA/OPLS-DA results. (4) The QG-rich extract has limited solubility in soybean oil (complete dissolution only up to 200 ppm) and very limited solubility in water, which may restrict its use in water-based food systems. Solubility enhancement strategies (emulsification, encapsulation, liposomal delivery) should be investigated. (5) Quercetagetin is sensitive to light and oxygen, which may affect its long-term stability during storage; the stability of the extract under various packaging and storage conditions should be evaluated. (6) Specific toxicological studies for food applications are limited in most countries; while the QG-rich extract is registered as a feed additive in China (MOA No. 618, 2022), international regulatory assessments for food preservative use are lacking. (7) The QG-rich extract is currently approved only as a feed additive in China; food preservative approval in other jurisdictions would require additional safety and efficacy dossiers. (8) The accelerated storage model has additional limitations: the Arrhenius relationship may not hold uniformly across all oxidation pathways, as different reactions have different activation energies; temperature affects the diffusion coefficients of antioxidants and oxidation intermediates in the oil matrix; open Erlenmeyer flasks allow oxygen access but also permit volatile loss; and the absence of light, moisture, and microbial activity means the model captures only thermal autoxidation.

Our prior work using DPPH-HPLC-Q-TOF-MS/MS identified quercetagetin as the predominant antioxidant in marigold flowers and confirmed its superior radical-scavenging activity compared to ascorbic acid and quercetin [60], which underpins the choice of this extract for the present study. The current study adopts the substrate-loss + volatile-formation approach as the primary outcome, providing a more direct, mechanistically interpretable, and industrially relevant measure of antioxidant performance than any single chemical assay.

### 3.8. Future Directions

Building on these findings, several future research directions are warranted. (i) Electron paramagnetic resonance (EPR) spectroscopy could directly detect free radical intermediates and clarify the stages at which the QG-rich extract intervenes in the oxidation cascade. (ii) In vitro hydroperoxide-decomposition assays (with and without QG) could verify whether the observed preferential suppression is due to inhibition of hydroperoxide formation or interception of hydroperoxide decomposition. (iii) Kinetic studies comparing QG reaction rates with various peroxyl radical species could offer quantitative insights into its selectivity profile. (iv) DFT calculations of BDE, IP, and proton affinity of quercetagetin at each hydroxyl site could provide a molecular-level rationale for the observed SAR [61]. (v) Comparative study with ≥98% pure quercetagetin at equimolar concentrations, alongside the 89% extract and individual minor flavonoid components (patuletin, quercetin), would deconvolute the contributions of each component. (vi) Other PUFA-rich oil matrices should be tested to assess generalizability [62,63]. (vii) Long-term feeding trials in livestock are needed to establish dose–response relationships between dietary QG, serum hexanal levels, and animal health outcomes. (viii) The SI should be formally validated through sensitivity analysis and comparison with alternative normalization approaches. (ix) The most toxicologically significant aldehydes (e.g., 4-hydroxy-2-nonenal, malondialdehyde, acrolein) should be quantified.

## 4. Materials and Methods

### 4.1. Materials and Chemicals

**Figure A2:**
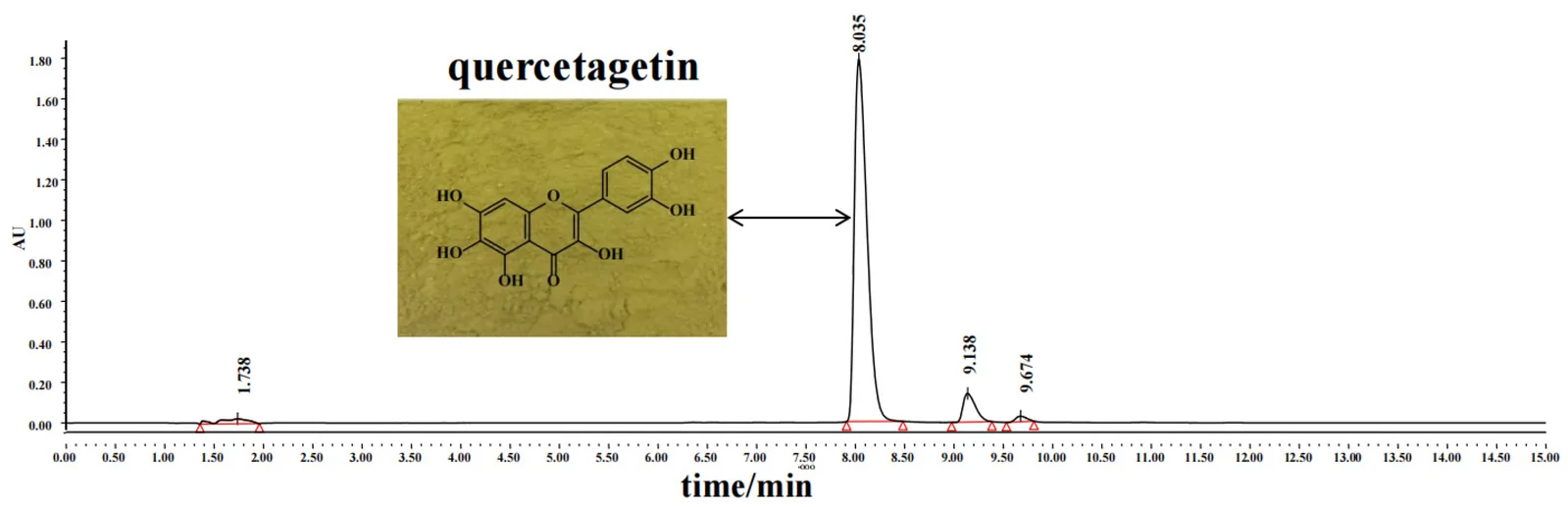
HPLC chromatogram of quercetagetin extracted from *Tagetes erecta* L.

A commercial-grade quercetagetin-rich extract (QG), with an approximate purity of 89% (CAS: 90-18-6; batch No.: 3-0270-200717), was supplied by Hebei Chenguang Biotechnology Co., Ltd. (Handan, China). The stability of this QG-rich extract was verified through HPLC analysis conducted before and after a storage period of 56 days, which indicated no significant degradation (inter-day peak area variation < 5%, *n* = 3). This specific purity level is not a limitation but a critical aspect of the study, as it corresponds to the commercially registered feed additive in China (MOA, 2022) [64]. Consequently, our findings provide a direct evaluation of the efficacy of the industrially relevant product, as opposed to a research-grade pure compound. The primary impurities, constituting approximately 10.8%, were identified as residual flavonoids, including patuletin and quercetin, through LC-MS analysis (Figure A2). In this manuscript, “QG” refers to the commercial-grade extract rich in quercetagetin. The aim of our study is to assess the overall efficacy of this multicomponent extract as a natural antioxidant for practical applications, without attributing the effects solely to the isolated activity of quercetagetin. The solubility of the QG-rich extract in soybean oil was evaluated by visually inspecting solutions at concentrations of 100 and 200 ppm under constant stirring at 25 °C for 2 h, followed by centrifugation at 4000 rpm for 10 min to detect any precipitate formation.

Complete dissolution was observed up to 200 ppm, while slight turbidity was noted at 300 ppm, and precipitation occurred at concentrations of 400 ppm and above (including 400 and 800 ppm tested in preliminary experiments), which would compromise the accuracy of quantitative analysis. The concentrations of 100 and 200 ppm were selected based on three considerations: (1) solubility experiments demonstrating complete dissolution only up to 200 ppm; (2) alignment with the typical industrial application range for synthetic antioxidants (100–200 ppm in food and feed, per regulatory guidelines such as GB 2760-2024) [65]; and (3) consistency with our prior dose–response study, where 200 ppm was identified as the minimum effective concentration among 100, 200, 400, 800, and 1600 ppm.

### 4.2. Preparation of Oil Samples and Accelerated Storage Conditions

Soybean oil was categorized into four distinct groups for the analysis of fatty acids and volatile compounds: (1) a solvent control group (CK) comprising 0.2% ethanol without any antioxidants; (2) a group containing a quercetagetin-rich extract at a concentration of 100 ppm (referred to as QG100), with 0.2% ethanol; (3) a group containing a quercetagetin-rich extract at a concentration of 200 ppm (referred to as QG200), with 0.2% ethanol; and (4) a group with propyl gallate (PG) at 200 ppm (referred to as PG200), also with 0.2% ethanol. All groups contained an identical ethanol concentration (0.2% *v*/*v*) to eliminate any potential solvent effects on oxidation kinetics. Preliminary experiments verified that 0.2% ethanol alone did not exhibit significant pro-oxidant or antioxidant activity under the conditions tested. The rationale for dose selection is detailed in Section 4.1. All samples were placed in open Erlenmeyer flasks and subjected to accelerated oxidation by storing them in a constant-temperature oven at 65.0 ± 0.5 °C, following the Schaal oven test protocol. According to the Q10 rule of thumb, which posits that a 10 °C increase in temperature approximately doubles the reaction rate, storage for 56 days at 65 °C is roughly equivalent to 12–15 months of storage at ambient temperature (25 °C), reflecting a typical commercial shelf-life duration. It is important to note that this is an approximation, and validation under real-time storage conditions is essential. The Schaal oven test at 65 ± 0.5 °C was employed as the accelerated storage condition. This protocol is widely used in lipid oxidation studies and provides a controlled environment for evaluating antioxidant efficacy over a compressed timeframe. However, several limitations of this model should be acknowledged: (1) the Arrhenius relationship may not hold uniformly across all oxidation pathways, as different reactions (e.g., hydroperoxide formation vs. decomposition vs. secondary volatile formation) have different activation energies; (2) temperature affects the diffusion coefficients of antioxidants and oxidation intermediates in the oil matrix; at 65 °C, diffusion rates are higher than at ambient temperature, potentially altering the spatial distribution of antioxidants and their interaction with lipid radicals; (3) open Erlenmeyer flasks were used, allowing oxygen access but also permitting volatile loss; while this is consistent with the Schaal protocol, it may affect the mass balance of volatile compounds; (4) the absence of light, moisture, and microbial activity—factors present under real storage conditions—means that the model captures only thermal autoxidation. Validation under real-time storage conditions (25 °C and 40 °C, 12 months) is recommended. The samples were stirred and their positions rotated daily. Aliquots were collected for analysis at intervals of 0, 7, 14, 28, and 56 days. For each treatment group, three independent oil samples (*n* = 3) were prepared in separate Erlenmeyer flasks and stored under identical conditions. At each sampling time point (0, 7, 14, 28, and 56 days), one aliquot was removed from each independent flask. Each aliquot was then subjected to independent fatty acid methylation and GC-MS analysis, as described in Section 4.3.

### 4.3. Determination of Fatty Acid Composition

#### 4.3.1. Preparation of Fatty Acid Methyl Esters (FAMEs)

The preparation of fatty acid methyl esters (FAMEs) was conducted following the methodology outlined by Tang [66], with specific modifications. An acid–base combined method was employed for the methylation of fatty acids. A 20 mg sample of oil was accurately weighed and placed into a 15 mL screw-thread centrifuge tube. To this, 20 μL of triundecanoin, at a concentration of 100 mg/mL, was added as an internal standard. Subsequently, 1 mL of a 1 mol/L potassium hydroxide (KOH) methanol solution was introduced, and the mixture was subjected to ultrasonication at 40 °C for 30 min. After standing for 15 min, the solution was evaporated to dryness using a water bath maintained at 70 °C. Following evaporation, 2 mL of a 5% sulfuric acid (H_2_SO_4_) methanol solution was added, and the mixture was ultrasonicated at 70 °C for 60 min before being rapidly cooled to room temperature. Precisely 2 mL of *n*-hexane was then added, and the mixture was shaken for 2 min, followed by a 10-min standing period. Approximately 1.5 mL of the upper *n*-hexane extract was transferred to a 10 mL centrifuge tube, followed by the addition of approximately 0.5 to 1 g of anhydrous sodium sulfate. The resulting mixture was agitated for 1 min and then allowed to stand undisturbed for 5 min. Subsequently, the upper layer was carefully transferred to a sample vial for subsequent analysis.

#### 4.3.2. Gas Chromatography–Mass Spectrometry (GC-MS) Analysis of Fatty Acids

The GC-MS analysis was conducted utilizing a Shimadzu GC-MS-QP2010 system (Shimadzu, Kyoto, Japan), which was equipped with an HP-88 capillary column (dimensions: 100.0 m × 0.32 mm × 0.25 μm; Agilent, Santa Clara, CA, USA). Helium, with a purity of 99.999%, served as the carrier gas at a flow rate of 1.04 mL/min. The injector temperature was maintained at 250 °C, operating with a split ratio of 20:1. The temperature program for the column was initiated at 120 °C and held for 1 min, followed by an increase to 175 °C at a rate of 10 °C/min, where it was held for 10 min. Subsequently, the temperature was elevated to 210 °C at a rate of 5 °C/min and held for 5 min, and finally increased to 230 °C at a rate of 5 °C/min, where it was held for 15 min. The ion source temperature was set at 200 °C, and the interface temperature was maintained at 220 °C. Mass spectra were acquired in full-scan mode across a range of 50–700 *m*/*z*. An injection volume of 1 μL was utilized. Identification of fatty acids was achieved by comparing the obtained mass spectra with the NIST 17 mass spectral library and by matching retention times with those of authentic standards.

#### 4.3.3. Quantification of Fatty Acids

The absolute quantification of fatty acids was conducted utilizing the internal standard method. The calculation was based on the following formula:

$$m\;=\;\frac{A}{A_{C11}}\times \;\frac{\rho_{C11}\times V_{C11}}{M}\times 100\%$$

where:

/m/ represents the total content of all fatty acid methyl esters (FAMEs) in the sample, expressed in milligrams (mg).

/A/ denotes the total peak area of all fatty acids.

/AC11/ is the peak area of the internal standard (triglyceride of undecanoic acid) added to the sample.

/ρC11/ is the concentration of triglycerides of undecanoic acid in milligrams per milliliter (mg/mL).

/VC11/ is the volume of triglycerides of undecanoic acid added to the sample in milliliters (mL).

/M/ is the mass of the sample in milligrams (mg).

Response factors for individual fatty acid methyl esters were determined using authentic standards. The internal standard, triundecanoin (C11:0), was introduced at a known concentration of 100 mg/mL, with 20 μL added per sample. The limit of detection (LOD) for each fatty acid, defined by a signal-to-noise ratio of 3:1, was established through the analysis of serially diluted standards and was found to range from 0.5 to 2.0 μg/g, contingent upon the specific fatty acid.

### 4.4. Analysis of Volatile Compounds via HS-SPME-GC-MS

#### 4.4.1. Optimization of HS-SPME Extraction Conditions

In accordance with the methodology delineated by Taviano et al. [67], the extraction conditions for volatile compounds in soybean oil were optimized utilizing a manual headspace sampling system. Five key factors influencing extraction efficiency were investigated through single-factor experiments: extraction temperature, equilibration time, extraction time, SPME fiber coating, and sample mass. The evaluation indices employed were the total number (TN) and total peak area (TA) of the identified volatile compounds. Optimization experiments were conducted in triplicate, and the optimal conditions were identified based on the maximum TN and TA values. Statistical differences among the conditions were assessed using one-way ANOVA with a significance level of *p* < 0.05. The optimal conditions for HS-SPME extraction were established as follows: 2.0000 g of soybean oil in a 15 mL headspace vial, equilibrated at 95 °C for 15 min, followed by extraction for 35 min using a 50/30 μm DVB/CAR/PDMS fiber (Cat. No. Gray, 57328-U, Merck KGaA, Darmstadt, Germany). The DVB/CAR/PDMS tri-phase coating combines polar (CAR) and non-polar (PDMS) adsorption capabilities with mesoporous DVB, enabling broad-spectrum extraction of volatile compounds spanning a wide polarity range—making it particularly suitable for complex volatile mixtures in oxidized oils. Desorption was carried out for 5 min in the GC injection port. The combined equilibration (15 min) and extraction (35 min) at 95 °C may promote thermal decomposition of hydroperoxides. However, since identical conditions were applied to all treatment groups, any such artifacts would be systematic rather than treatment-specific, preserving the validity of relative comparisons.

#### 4.4.2. Gas Chromatography–Mass Spectrometry (GC-MS) Conditions for Volatile Compounds

The separation of volatile compounds was achieved using an HP-5MS capillary column (dimensions: 30 m × 0.25 mm, film thickness: 0.25 μm; Agilent Technologies, Santa Clara, CA, USA). The temperature of the gas chromatography (GC) oven was programmed as follows: an initial hold at 40 °C for 3 min, followed by a ramp to 120 °C at a rate of 5 °C per minute and subsequently to 240 °C at a rate of 10 °C per minute, with a final hold for 5 min. The injector was maintained at a temperature of 250 °C, and injections were performed in splitless mode. High-purity helium (99.99%) was utilized as the carrier gas, with a constant flow rate of 1.0 mL per minute. Mass spectrometric detection was conducted using electron ionization (EI) at an energy of 70 eV. The ion source and interface were maintained at temperatures of 230 °C and 280 °C, respectively. Data acquisition was performed in full-scan mode, covering a mass-to-charge ratio (*m*/*z*) range of 45–500.

#### 4.4.3. Identification and Quantification of Volatile Compounds

The identification of volatile compounds was accomplished by comparing their mass spectra with those in the NIST.17 mass spectral library, with a similarity threshold of ≥80%. Semi-quantification was conducted using an internal standard method, employing α-asarone as the reference compound. Each sample was analyzed in triplicate, and the results were expressed in terms of micrograms per gram of oil (µg/g). A quality control (QC) sample was prepared by pooling aliquots from all samples and was injected at intervals of every ten injections to assess instrument stability. The relative standard deviation (RSD) of the internal standard peak areas remained below 10% throughout all analytical runs. Volatile compounds were identified by comparing their mass spectra with the NIST 17 mass spectral library (similarity threshold ≥80%). Retention indices were not calculated, and authentic standards were not used for compound confirmation. This represents a limitation of the study, as some identifications—particularly for structurally similar isomers—may be tentative. Future studies should incorporate linear retention index (LRI) verification and authentic standard confirmation.

### 4.5. Statistical and Chemometric Analyses

All analyses were conducted in triplicate, with data presented as mean ± standard deviation (SD). Statistical evaluations were carried out utilizing IBM SPSS Statistics 19.0 (IBM Corp., Chicago, IL, USA) and GraphPad Prism 8.0. A one-way analysis of variance (ANOVA), followed by Duncan’s multiple range test, was employed to ascertain significant differences among groups, with a threshold of *p* < 0.05. A factorial design (treatment × time) was applied to analyze fatty acid data (Table 1, Table 2 and Table A1) and volatile compound class data (Figure 5), while one-way ANOVA was used for HS-SPME optimization (Figure 2) and cross-sectional comparisons at individual time points (Figure 4 and Figure 6). To address the issue of multiple comparisons, particularly concerning the 13 fatty acids across 5 time points and 3 treatment groups, as well as the 9 volatile classes across the same time points and treatment groups, false discovery rate (FDR) correction was applied using the Benjamini–Hochberg method. Adjusted *p*-values, corrected for the Benjamini–Hochberg FDR, are presented in all pertinent tables, with significance determined at *p* < 0.05 for all analyses. Additionally, principal component analysis (PCA), orthogonal partial least squares discriminant analysis (OPLS-DA), and hierarchical clustering analysis were conducted using SIMCA 14.1. The X matrix for PCA and OPLS-DA comprised the semi-quantified concentrations (μg/g) of individual volatile compounds identified by HS-SPME-GC-MS.

No molecular descriptors or physicochemical properties were included as additional variables. In the evaluation of OPLS-DA models, the quality of the models was determined using R^2^Y, which measures goodness of fit, and Q^2^, which assesses predictive ability. A Q^2^ value exceeding 0.5 was deemed acceptable, while a value surpassing 0.9 indicated excellent predictive capability. To ensure model robustness, permutation tests comprising 200 iterations were conducted, with negative Q^2^-intercept values serving as confirmation of the absence of overfitting. Hierarchical clustering analysis was also performed on the volatile compound dataset. The resulting dendrogram confirmed the group separation observed in PCA and OPLS-DA, with CK and QG200 samples clustering separately at advanced oxidation stages.

## 5. Conclusions

This study demonstrates that a commercial-grade QG-rich extract (89% purity) effectively protects against oxidative deterioration in soybean oil under accelerated storage conditions. The QG-rich extract operates through two complementary mechanisms: (i) safeguarding PUFAs—particularly C18:2 and C18:3—from degradation, consequently reducing the substrate available for the formation of secondary volatiles, and (ii) observing a distinct inhibition pattern in which QG preferentially suppresses specific rancidity markers—notably hexanoic acid (SI = 1.52), hexanal dimethyl acetal (SI = 1.35), octanoic acid (SI = 1.30), and (*E*)-2-nonenal (SI = 1.24)—rather than functioning as a non-selective radical scavenger. The selectivity, quantified via SI and supported by kinetic artifact analysis, represents a key empirical observation that warrants further mechanistic investigation.

Within the solubility limit of QG in soybean oil (complete dissolution up to 200 ppm), this concentration affords substantial protection—approximately 5.2-fold greater PUFA preservation and >74% reduction in aldehydes compared to the control—recommending 200 ppm as a practical reference point for industrial application. The optimized HS-SPME-GC-MS workflow and chemometric evaluation method established in this study provide a useful analytical framework for future antioxidant studies. The moderate purity (89%) of the extract should be viewed not as a limitation but as a translational advantage, as it aligns with the purity level of the commercially registered feed additive in China (MOA Announcement No. 618, 2022), ensuring direct applicability for feed applications. By incorporating principles of green chemistry and maintaining clean-label compatibility, this QG-rich extract offers a sustainable, regulatory-approved alternative to synthetic antioxidants.

From a broader perspective, quercetagetin has potential commercial applications for food preservation. However, the emphasis is often placed on the compound itself rather than on the extract from its source, *Tagetes erecta*, which may contain additional antioxidant substances that could enhance its effectiveness as a food preservative and potentially as a nutraceutical. Future research should investigate the comparative effects of pure quercetagetin versus the QG-rich extract, as the biological effects of pure quercetagetin have been described recently [45], and the contribution of non-QG components in the extract warrants systematic evaluation. Future research should validate these findings under real-time storage conditions, investigate the efficacy of QG in other PUFA-rich oil matrices, and probe the molecular basis of the observed inhibition selectivity using EPR spectroscopy and DFT calculations.

## Figures and Tables

**Table 1:** Changes in fatty acid composition and content of soybean oil in the control group (CK) at different oxidation stages.

Fatty Acid	Fatty Acid Content at Different Oxidation Stages (μg/g)				
	0 d	7 d	14 d	28 d	56 d
C14:0	2.58 ± 0.13	2.99 ± 0.83	2.45 ± 0.26	2.75 ± 0.41	1.31 ± 1.13
C16:0	382.02 ± 12.66	394.07 ± 42.38	375.04 ± 9.84	451.78 ± 42.69	274.53 ± 126.36
C17:0	3.86 ± 0.72	4.83 ± 1.61	3.85 ± 1.11	4.48 ± 0.07	2.10 ± 1.84
C18:0	160.21 ± 8.47	163.09 ± 17.66	156.04 ± 0.98	186.00 ± 16.39	262.24 ± 186.23
C20:0	12.95 ± 1.52	11.77 ± 1.34	12.92 ± 1.38	16.92 ± 2.32	11.35 ± 1.72
C22:0	15.95 ± 1.42	15.75 ± 1.74	9.46 ± 8.20	18.58 ± 1.15	12.16 ± 3.12
C24:0	4.66 ± 2.09	4.95 ± 2.16	3.57 ± 3.17	8.34 ± 0.89	ND
C17:1	1.82 ± 0.15	1.57 ± 1.37	1.15 ± 0.99	0.76 ± 1.31	ND
C16:1	3.68 ± 0.37	1.74 ± 1.59	3.02 ± 0.34	3.19 ± 0.67	0.63 ± 1.09
C18:1	816.72 ± 29.12	837.67 ± 81.14	800.45 ± 10.84	969.05 ± 71.33	477.60 ± 211.07
C20:3	ND	ND	10.29 ± 9.35	6.40 ± 11.09	1.03 ± 1.79
C18:2	1793.50 ± 38.91	1858.42 ± 177.42	1734.17 ± 45.19	2058.74 ± 166.02	169.15 ± 120.66
C18:3	239.89 ± 25.57	213.18 ± 26.46	189.85 ± 6.45	284.17 ± 17.10	69.75 ± 11.02
SFA	582.23 ± 23.50	597.45 ± 64.29	563.33 ± 21.26	688.86 ± 63.55	563.70 ± 77.14
MUFA	822.23 ± 28.99	840.97 ± 81.31	804.61 ± 9.62	972.99 ± 70.20	478.23 ± 211.81
PUFA	2033.40 ± 59.68	2071.59 ± 199.64	1934.31 ± 52.36	2349.31 ± 185.69	239.93 ± 132.76
UFA	2855.63 ± 88.64	2912.57 ± 280.95	2738.92 ± 57.77	3322.31 ± 255.19	718.16 ± 344.24
UFA/SFA	4.91 ± 0.05	4.88 ± 0.05	4.86 ± 0.08	4.83 ± 0.12	1.33 ± 0.70
UFA/TFA	83.07 ± 0.13%	82.99 ± 0.16%	82.94 ± 0.24%	82.84 ± 0.35%	53.61 ± 16.96%

Note: Values are expressed as mean ± standard deviation (*n* = 3). ND: not detected. SFA: saturated fatty acid; MUFA: monounsaturated fatty acid; PUFA: polyunsaturated fatty acid. UFA = MUFA + PUFA; TFA = SFA + UFA.

**Table 2:** Variation in saturated, monounsaturated and polyunsaturated fatty acid contents of soybean oil under different treatments.

Fatty Acid Type	Group	Fatty Acid Content (μg/g)				
		0 d	7 d	14 d	28 d	56 d
SFA	CK	582.23 ± 23.50	597.45 ± 64.29	563.33 ± 21.26	688.86 ± 63.55	563.70 ± 77.14
	PG	585.87 ± 31.38	561.37 ± 44.02	555.24 ± 60.91	609.39 ± 128.56	545.62 ± 35.09
	QG100	628.57 ± 51.63	614.24 ± 27.55	595.46 ± 21.88	660.20 ± 19.18	395.21 ± 82.52
	QG200	572.73 ± 28.90	549.70 ± 16.99	554.25 ± 34.19	662.17 ± 13.51	344.96 ± 40.02
MUFA	CK	822.23 ± 28.99	840.97 ± 81.31	804.61 ± 9.62	972.99 ± 70.20	478.23 ± 211.81
	PG	950.86 ± 48.51	914.90 ± 64.44	879.74 ± 74.87	1126.82 ± 88.59	773.34 ± 51.95
	QG100	985.78 ± 48.61	988.33 ± 47.42	963.94 ± 23.30	1053.92 ± 36.72	605.60 ± 42.51
	QG200	930.86 ± 35.71	878.89 ± 25.30	894.08 ± 45.35	1058.71 ± 11.61	499.63 ± 58.39
PUFA	CK	2033.40 ± 59.68	2071.59 ± 199.64	1934.31 ± 52.36	2349.31 ± 185.69	239.93 ± 132.76
	PG	1949.57 ± 98.95	1874.02 ± 103.52	1929.33 ± 112.50	2122.11 ± 155.63	1917.84 ± 108.51
	QG100	2086.31 ± 171.05	2022.06 ± 79.20	1964.29 ± 33.21	2111.24 ± 80.54	1396.24 ± 183.99
	QG200	1914.50 ± 66.05	1825.27 ± 45.24	1823.70 ± 84.78	2113.82 ± 16.46	1254.83 ± 88.54

Note: Values are expressed as mean ± standard deviation (*n* = 3). SFA: saturated fatty acid; MUFA: monounsaturated fatty acid; PUFA: polyunsaturated fatty acid. CK: control; PG: propyl gallate; QG100: quercetagetin 100 ppm; QG200: quercetagetin 200 ppm.

**Table 3:** Key volatile compounds with VIP scores ≥ 1.0 on day 56 (CK vs. QG200), with inhibition rates and selectivity indices.

Volatile Compound	VIP Score (56 d)	Trend in QG200 vs. Control	Inhibition Rate (%) in QG200	Selectivity Index (SI)
Hexanal dimethyl acetal	5.01	**↓↓↓**	88.5	1.35
(*E*,*E*)-2,4-Decadienal	3.47	**↓**	68.2	1.04
Hexanal	3.28	**↓**	74.24	1.13
Methyl hexanoate	2.11	**↓**	70.1	1.07
Hexanoic acid	2.01	**↓**	100.00	1.52
(*E*)-2-Nonenal	1.80	**↓**	81.2	1.24
Methyl 10-octadecenoate	1.72	**↓**	65.9	1.00
5-Ethyl-1,3-dioxane-5-methanol	1.71	**↓**	69.3	1.05
Methyl linoleate	1.67	**↓**	64.8	0.99
(*E*)-2-Octenal	1.64	**↓**	76.5	1.16
1-Octen-3-ol	1.18	**↓**	72.1	1.10
Octanoic acid	1.09	**↓**	85.4	1.30

Note: SI = (Inhibition rate of compound)/(Average inhibition rate of total volatiles = 65.7%). SI > 1 indicates preferential suppression of the compound by QG200. Arrows indicate the degree to which each compound is reduced in QG200 relative to the control, compared with the average reduction of all volatiles: ↓↓↓, markedly reduced (reduction substantially exceeds the average); ↓, reduced to a degree comparable to or slightly above the average.

## Data Availability

The data presented in this study are available on request from the corresponding authors.

## Supplementary Materials

The following supporting information can be downloaded at: https://www.mdpi.com/article/10.3390/molecules31162891/s1.

## Appendix A

This appendix provides supplementary data on volatile compounds, fatty acid composition, and compound purity related to the oxidation of soybean oil and the fermentation of *Tagetes erecta* L. (marigold). The following tables and figures support the findings presented in the main text.

**Table A1:** Changes in fatty acid composition and content of soybean oil in different treatments during different oxidation periods.

Time (d)	Fatty Acid	Fatty Acid Content at Different Oxidation Stages (μg/g)			
		CK	PG	QG100	QG200
0 d					
	C14:0	2.58 ± 0.13	3.30 ± 0.71	2.84 ± 0.75	2.66 ± 0.65
	C16:0	382.02 ± 12.66	384.87 ± 21.68	410.66 ± 37.15	379.06 ± 17.64
	C17:0	3.86 ± 0.72	2.87 ± 0.31	3.52 ± 0.40	3.36 ± 1.67
	C18:0	160.21 ± 8.47	153.33 ± 7.10	166.87 ± 12.82	152.89 ± 11.77
	C20:0	12.95 ± 1.52	15.65 ± 0.48	16.93 ± 1.99	15.02 ± 1.03
	C22:0	15.95 ± 1.42	19.80 ± 0.60	20.96 ± 0.84	12.62 ± 10.97
	C24:0	4.66 ± 2.09	6.04 ± 1.45	6.79 ± 1.17	7.12 ± 1.13
	C17:1	1.82 ± 0.15	0.51 ± 0.89	1.75 ± 1.52	ND
	C16:1	3.68 ± 0.37	1.93 ± 0.81	3.28 ± 0.37	2.55 ± 0.36
	C18:1	816.72 ± 29.12	948.42 ± 48.57	980.75 ± 48.14	928.31 ± 36.05
	C20:3	ND	ND	5.26 ± 9.11	ND
	C18:2	1793.5 ± 38.91	1777.78 ± 78.28	1881.82 ± 167.17	1729.22 ± 40.42
	C18:3	213.18 ± 26.46	171.79 ± 23.82	199.23 ± 16.79	185.28 ± 27.90
7 d					
	C14:0	2.99 ± 0.83	2.61 ± 0.72	3.08 ± 0.15	2.13 ± 0.21
	C16:0	394.07 ± 42.38	367.12 ± 24.03	402.31 ± 18.92	359.54 ± 12.36
	C17:0	4.83 ± 1.61	3.68 ± 0.40	3.56 ± 0.47	3.71 ± 0.12
	C18:0	163.09 ± 17.66	150.02 ± 15.23	164.72 ± 7.81	146.11 ± 6.73
	C20:0	11.77 ± 1.34	14.42 ± 1.66	15.58 ± 0.76	14.53 ± 0.56
	C22:0	15.75 ± 1.74	17.36 ± 1.95	20.19 ± 1.12	18.36 ± 0.95
	C24:0	4.95 ± 2.16	6.16 ± 1.49	4.81 ± 4.20	5.32 ± 0.45
	C17:1	1.57 ± 1.37	1.52 ± 1.37	1.50 ± 1.30	ND
	C16:1	1.74 ± 1.59	2.74 ± 0.09	3.13 ± 0.38	3.02 ± 0.56
	C18:1	837.67 ± 81.14	910.64 ± 64.04	983.70 ± 47.49	875.86 ± 25.58
	C20:3	ND	4.94 ± 8.55	5.84 ± 10.11	1.53 ± 2.64
	C18:2	1858.42 ± 177.42	1706.60 ± 95.50	1849.83 ± 64.70	1660.72 ± 37.42
	C18:3	213.18 ± 26.46	162.48 ± 14.79	166.39 ± 9.62	163.03 ± 11.69
14 d					
	C14:0	2.45 ± 0.26	2.69 ± 0.76	2.62 ± 0.86	2.59 ± 0.21
	C16:0	375.04 ± 9.84	366.46 ± 33.03	393.66 ± 13.48	361.58 ± 20.25
	C17:0	3.85 ± 1.11	3.54 ± 0.63	3.39 ± 0.19	3.64 ± 1.55
	C18:0	156.04 ± 0.98	144.30 ± 16.65	159.76 ± 2.42	146.41 ± 9.34
	C20:0	12.92 ± 1.38	8.87 ± 8.44	15.85 ± 1.78	15.36 ± 1.84
	C22:0	9.46 ± 8.20	19.14 ± 5.85	13.77 ± 10.36	18.11 ± 1.45
	C24:0	3.57 ± 3.17	10.24 ± 12.86	6.40 ± 1.15	6.58 ± 1.22
	C17:1	1.15 ± 0.99	0.57 ± 0.99	0.58 ± 1.00	0.87 ± 1.51
	C16:1	3.02 ± 0.34	3.14 ± 1.10	2.99 ± 0.34	2.91 ± 1.07
	C18:1	800.45 ± 10.84	876.03 ± 76.35	960.38 ± 23.45	890.29 ± 43.29
	C20:3	10.29 ± 9.35	ND	1.61 ± 2.79	10.99 ± 9.52
	C18:2	1734.17 ± 45.19	1728.89 ± 108.85	1794.17 ± 33.05	1662.16 ± 73.02
	C18:3	189.85 ± 6.45	200.44 ± 16.85	168.51 ± 2.40	150.54 ± 21.54
28 d					
	C14:0	2.75 ± 0.41	2.84 ± 0.28	3.49 ± 0.76	3.19 ± 0.88
	C16:0	451.78 ± 42.69	433.96 ± 27.61	428.27 ± 12.35	429.89 ± 7.35
	C17:0	4.48 ± 0.07	3.04 ± 0.08	3.53 ± 0.71	4.59 ± 1.04
	C18:0	186.00 ± 16.39	120.27 ± 104.68	176.77 ± 5.33	175.94 ± 3.88
	C20:0	16.92 ± 2.32	17.48 ± 2.86	17.83 ± 0.31	17.42 ± 0.56
	C22:0	18.58 ± 1.15	23.39 ± 2.60	21.50 ± 1.22	22.59 ± 0.65
	C24:0	8.34 ± 0.89	8.41 ± 1.11	8.80 ± 0.38	8.54 ± 0.56
	C17:1	0.76 ± 1.31	55.83 ± 94.23	1.46 ± 1.68	1.22 ± 1.06
	C16:1	3.19 ± 0.67	3.00 ± 0.57	3.26 ± 0.30	3.47 ± 1.02
	C18:1	969.05 ± 71.33	1067.98 ± 75.34	1049.20 ± 36.05	1054.02 ± 13.37
	C20:3	6.40 ± 11.09	9.58 ± 7.66	9.23 ± 8.87	5.21 ± 9.03
	C18:2	2058.74 ± 166.02	1901.57 ± 142.79	1889.76 ± 72.95	1895.55 ± 17.49
	C18:3	284.17 ± 17.10	210.97 ± 18.14	212.26 ± 17.27	213.06 ± 5.70
56 d					
	C14:0	1.31 ± 1.13	0.99 ± 1.71	2 ± 0.29	1.7 ± 0.05
	C16:0	274.53 ± 126.36	375.53 ± 20.71	294.06 ± 21.55	237.79 ± 26.82
	C17:0	2.1 ± 1.84	ND	2.8 ± 0.6	2.81 ± 0.84
	C18:0	262.24 ± 186.23	146.23 ± 13.32	112.94 ± 6.23	88.88 ± 10.67
	C20:0	11.35 ± 1.72	10.91 ± 0.53	8.65 ± 1.02	6.34 ± 1.25
	C22:0	12.16 ± 3.12	8.68 ± 5.06	9 ± 0.46	6.75 ± 1.41
	C24:0	0 ± 0	3.28 ± 0.91	1.13 ± 1.95	0.69 ± 1.2
	C16:1	0 ± 0	0.60 ± 1.04	0 ± 0	0.71 ± 1.22
	C17:1	0.63 ± 1.09	2.07 ± 1.81	2.01 ± 0.17	2.03 ± 0.45
	C18:1	477.6 ± 211.07	770.67 ± 49.55	603.59 ± 42.53	496.89 ± 58.47
	C20:3	1.03 ± 1.79	8.40 ± 7.63	3.81 ± 3.3	7.5 ± 0.84
	C18:2	169.15 ± 120.66	1730.52 ± 100.32	1288.82 ± 101.24	1127.57 ± 106.03
	C18:3	69.75 ± 11.02	178.92 ± 3.59	140.44 ± 35.58	119.75 ± 23.1

Note: Values are expressed as mean ± standard deviation (*n* = 3). ND: not detected. CK: control; PG: propyl gallate; QG100: quercetagetin 100 ppm; QG200: quercetagetin 200 ppm.

**Table A2:** Changes in the types and contents of volatile substances in soybean oil at different oxidation stages.

No.	Chemical Name	Volatile Substance Content (μg/g)									
		0 d		7 d		14 d		28 d		56 d	
		CK	QG200	CK	QG200	CK	QG200	CK	QG200	CK	QG200
	Alcohols	ND	ND	ND	ND	10.46 ± 0.46	10.73 ± 2.99	246.92 ± 36.85	13.98 ± 0.94	330.31 ± 35.1	63.11 ± 16.38
1	1-octene-3-ol	ND	ND	ND	ND	1.99 ± 0.09	1.79 ± 0.46	15.07 ± 1.8	ND	27.72 ± 2.23	8.43 ± 2.59
2	2-cyclopentane ethanol	ND	ND	ND	ND	3.77 ± 0.52	4.27 ± 2.53	ND	ND	ND	ND
3	5-ethyl-1,3-dioxane-5-methanol	ND	ND	ND	ND	4.71 ± 0.56	4.67 ± 0.94	26.73 ± 0.19	5.3 ± 0.39	153.06 ± 34.16	28.13 ± 10.27
4	1-octanol	ND	ND	ND	ND	ND	ND	15.31 ± 1.22	ND	ND	ND
5	1,3-octanediol	ND	ND	ND	ND	ND	ND	130.45 ± 35.17	ND	42.95 ± 0.86	ND
6	cyclohexanol, 1-(1,2-propadien-1-yl)	ND	ND	ND	ND	ND	ND	10.42 ± 1.39	ND	5.55 ± 0	ND
7	1-nonen-4-ol	ND	ND	ND	ND	ND	ND	3.1 ± 0.85	ND	2.2 ± 0.18	ND
8	1-pentanol	ND	ND	ND	ND	ND	ND	30.62 ± 0.33	ND	28.97 ± 2.15	ND
9	3,5-octadien-2-ol	ND	ND	ND	ND	ND	ND	ND	8.68 ± 1.36	37.83 ± 1.28	23.57 ± 4.58
10	bicyclo[3.1.1]heptan-2-ol,4,6,6-trimethyl-	ND	ND	ND	ND	ND	ND	15.21 ± 1.28	ND	ND	ND
11	(*S*)-(+)-5-methyl-1-heptanol	ND	ND	ND	ND	ND	ND	ND	ND	7.99 ± 1.9	ND
12	3-decyn-2-ol	ND	ND	ND	ND	ND	ND	ND	ND	8.76 ± 1.69	2.97 ± 0.21
13	2,5-dimethylcyclohexanol	ND	ND	ND	ND	ND	ND	ND	ND	5.7 ± 0.96	ND
14	1-hexyne-3-ol	ND	ND	ND	ND	ND	ND	ND	ND	9.57 ± 0	ND
	Ethers	ND	ND	ND	ND	2.47 ± 0.69	ND	69.39 ± 2.71	34.64 ± 14.77	135.19 ± 49.95	30.47 ± 14.77
15	4-vinylcyclohexene dioxide	ND	ND	ND	ND	2.47 ± 0.69	ND	ND	ND	ND	ND
16	1,1-dimethoxynonane	ND	ND	ND	ND	ND	ND	15.18 ± 5.99	ND	31.73 ± 4.73	ND
17	[(hexyloxy)methyl]oxirane	ND	ND	ND	ND	ND	ND	5.1 ± 2.11	ND	ND	ND
18	propionaldehyde dimethyl acetal	ND	ND	ND	ND	ND	ND	8.61 ± 2.64	30.47 ± 14.77	ND	ND
19	1,1,3,3-tetramethoxypropane	ND	ND	ND	ND	ND	ND	55.67 ± 0	4.17 ± 0.01	91.81 ± 45.13	ND
20	1,1-dimethoxyoctane	ND	ND	ND	ND	ND	ND	ND	ND	144.58 ± 10.6	ND
21	1,1-dimethoxydecane	ND	ND	ND	ND	ND	ND	ND	ND	3.03 ± 0.62	ND
	Aldehydes	16.51 ± 1.98	44.59 ± 3.13	33.41 ± 4.46	40.43 ± 1.41	102.86 ± 20.78	135.37 ± 12.4	1153.13 ± 89.49	211.49 ± 49.16	2655.97 ± 924.75	684.12 ± 118.22
22	trans,trans-2,4-decadienal	2.49 ± 1.18	12.82 ± 2.62	16.05 ± 2.77	22.22 ± 0.09	ND	ND	8.2 ± 3	94.11 ± 30.44	91.87 ± 4.67	259.97 ± 28.61
23	(*E,E*)-2,4-heptadienal	6.7 ± 1.80	20.62 ± 8.59	7.17 ± 1.28	6.16 ± 0.64	18.58 ± 2.21	12.28 ± 1.43	ND	ND	3.3 ± 0.75	34.54 ± 4.28
24	(*E*)-2-heptenal	6.26 ± 1.39	5.14 ± 1.96	2.11 ± 0.08	ND	6.03 ± 1.27	4.49 ± 0.46	19.3 ± 1.41	15.07 ± 5.25	44.31 ± 6.22	35.7 ± 12.28
25	trans-2,4-sebacic aldehyde	1.06 ± 0.23	6.01 ± 0.88	4.35 ± 0.65	5.82 ± 0.91	99.83 ± 18.32	64.17 ± 3.87	2.44 ± 0.79	32.12 ± 9.38	28.85 ± 2.68	70.52 ± 18.28
26	trans,trans-2,4-nonadienal	ND	ND	1.02 ± 0.14	ND	0.4 ± 0.89	2.18 ± 0.47	ND	ND	10.66 ± 2.67	ND
27	(*Z*)-7-hexadecenal	ND	ND	2.71 ± 0.11	ND	ND	ND	ND	ND	ND	ND
28	decadienal,(*E*,*Z*)-2,4-decadienal	ND	ND	ND	6.22 ± 0.25	23.22 ± 3.01	18.18 ± 1.89	ND	ND	ND	ND
29	nonanal	ND	ND	ND	ND	1.89 ± 0.38	3.1 ± 0.96	144.6 ± 25.7	4.17 ± 0	59.82 ± 13.83	2.37 ± 1
30	2-undecenal	ND	ND	ND	ND	1.47 ± 0.29	2.3 ± 0.88	50.65 ± 19.29	ND	42.85 ± 6.13	6.34 ± 3.22
31	trans-2-octenal	ND	ND	ND	ND	2.9 ± 0.40	3.04 ± 0.21	47.41 ± 4.85	ND	153.75 ± 4.12	34.06 ± 9.91
32	trans-2-decene aldehyde	ND	ND	ND	ND	1.41 ± 0.51	2.01 ± 0.67	ND	ND	ND	ND
33	(*E*,*E*)-2,4-nonadienal	ND	ND	ND	ND	3.06 ± 0.37	2.86 ± 0.10	4.09 ± 0.99	5.33 ± 1.42	ND	6.29 ± 1.5
34	*n*-hexaldehyde	ND	ND	ND	ND	9.77 ± 2.02	8.48 ± 1.30	350.82 ± 46.3	26.14 ± 4.87	609.14 ± 146.13	68.56 ± 24.5
36	valeraldehyde	ND	ND	ND	ND	ND	ND	30.91 ± 9.26	ND	25.98 ± 6.68	ND
37	heptaldehyde	ND	ND	ND	ND	ND	ND	24.42 ± 5.31	ND	13.76 ± 5.11	ND
38	decyl aldehyde	ND	ND	ND	ND	ND	ND	48.89 ± 10.44	ND	22.04 ± 5.32	ND
39	hexanal dimethyl acetal	ND	ND	ND	ND	ND	ND	206.07 ± 86.36	ND	1460.05 ± 1086.85	152.29 ± 35.03
40	trans-2-nonenal	ND	ND	ND	ND	ND	ND	10.64 ± 2.83	ND	16.11 ± 5.98	1.86 ± 0.49
41	oxo-2-hexenal, 4-oxo-	ND	ND	ND	ND	ND	ND	7.55 ± 1.2	ND	ND	2.48 ± 0.44
42	2-decenal, (*Z*)-2-decenal	ND	ND	ND	ND	ND	ND	66.68 ± 24.89	ND	47.05 ± 9.68	ND
43	4-heptenal	ND	ND	ND	ND	ND	ND	6.53 ± 2.28	ND	ND	7.36 ± 5.67
44	undecan-4-olide	ND	ND	ND	ND	ND	ND	4.51 ± 1.35	ND	ND	ND
45	octanal	ND	ND	ND	ND	ND	ND	105.46 ± 9.36	ND	ND	ND
46	undecanal dimethyl acetal	ND	ND	ND	ND	ND	ND	ND	ND	12.03 ± 1.63	ND
47	cis-4-decenal	ND	ND	ND	ND	ND	ND	ND	ND	4.71 ± 0.44	ND
48	(2*E*,4E)-2,4-octadienal	ND	ND	ND	ND	ND	ND	ND	ND	1.14 ± 0	ND
49	(*E*,*E*)-2,4-dodecadienal	ND	ND	ND	ND	ND	ND	ND	ND	ND	1.78 ± 0.47
	Acid	10.91 ± 2.59	7.00 ± 0.13	3.35 ± 0.05	ND	ND	ND	445.79 ± 76.57	ND	549.08 ± 16.77	ND
50	linoleic acid	10.91 ± 4.94	7.00 ± 0.13	2.35 ± 0.15	ND	ND	ND	ND	ND	ND	ND
51	dl-3-methylvaleric acid	ND	ND	ND	ND	ND	ND	13.85 ± 2.17	ND	18.26 ± 0	ND
52	butanoic acid, 2,2-dimethyl-, ethenyl ester	ND	ND	ND	ND	ND	ND	87.73 ± 16.41	10.94 ± 0.98	161.22 ± 39.5	7.3 ± 1.68
53	hexanoic acid	ND	ND	ND	ND	ND	ND	427.25 ± 78.67	ND	232.9 ± 78.8	ND
54	hexanoic acid, 9-decen-1-yl ester	ND	ND	ND	ND	ND	ND	30.98 ± 1.06	ND	ND	ND
55	cyclopentanecarboxylic acid, 1-methyl-	ND	ND	ND	ND	ND	ND	4.68 ± 0.26	ND	ND	ND
56	acetic acid	ND	ND	ND	ND	ND	ND	ND	ND	102.15 ± 18.29	ND
57	isovaleric acid	ND	ND	ND	ND	ND	ND	ND	ND	14.66 ± 1.11	ND
58	2-octynoic acid	ND	ND	ND	ND	ND	ND	ND	ND	3.37 ± 1.5	ND
59	trimethylthioacetic s-acid	ND	ND	ND	ND	ND	ND	ND	ND	3.78 ± 0.39	ND
60	trans-2-oceenoic acid	ND	ND	ND	ND	ND	ND	ND	ND	12.81 ± 0.35	ND
	Ketones	ND	ND	2.86 ± 0.11	3.49 ± 0.46	10.82 ± 0.69	3.54 ± 0.86	145.64 ± 17.35	184.01 ± 12.75	109.9 ± 6.07	37.75 ± 2.99
61	1,8(2*H*,5*H*)-naphthalenedione, hexahydro-8a-methyl-, cis	ND	ND	2.86 ± 0.11	3.49 ± 0.46	5.69 ± 0.64	ND	ND	ND	ND	ND
62	5-methyl-3-methylene-5-hexen-2-one	ND	ND	ND	ND	5.13 ± 0.11	3.54 ± 0.86	ND	80.05 ± 9.79	ND	ND
63	pentadecan-6-one	ND	ND	ND	ND	ND	ND	3.5 ± 0.27	ND	ND	ND
64	2-heptanone	ND	ND	ND	ND	ND	ND	48.49 ± 6.03	ND	39.16 ± 3.42	4.78 ± 0.78
65	2-methyl-1,3-cyclohexanedione	ND	ND	ND	ND	ND	ND	8.35 ± 1.24	23.06 ± 4.28	ND	ND
66	3-nonen-2-one	ND	ND	ND	ND	ND	ND	12.01 ± 4.82	ND	23.91 ± 5.04	3.9 ± 2.3
67	2-pentadecanone	ND	ND	ND	ND	ND	ND	12.07 ± 2.4	ND	ND	ND
68	2-heptadecanone	ND	ND	ND	ND	ND	ND	4.72 ± 1.52	ND	ND	ND
69	6-dodecanone	ND	ND	ND	ND	ND	ND	8.41 ± 0.2	ND	6.18 ± 0.37	ND
70	6-tetradecanone	ND	ND	ND	ND	ND	ND	5.76 ± 1.99	ND	ND	ND
71	2-decanone	ND	ND	ND	ND	ND	ND	7.51 ± 0.14	ND	1.59 ± 0.39	ND
72	2-nonanone	ND	ND	ND	ND	ND	ND	7.87 ± 1.79	ND	2.14 ± 0.53	ND
73	6-undecanone	ND	ND	ND	ND	ND	ND	9.28 ± 0.56	ND	6.95 ± 1.4	ND
74	1-cyclopropyl	ND	ND	ND	ND	ND	ND	9.07 ± 2.54	ND	ND	ND
75	2-octanone	ND	ND	ND	ND	ND	ND	8.3 ± 1.98	10.94 ± 1.98	ND	ND
76	heptyldihydro-4-methyl-, (4*R*,5*R*)-rel	ND	ND	ND	ND	ND	ND	4.12 ± 1.43	ND	ND	ND
77	3,5-octadien-2-one	ND	ND	ND	ND	ND	ND	ND	34.64 ± 6.18	ND	13.86 ± 1.25
78	3-octen-2-one	ND	ND	ND	ND	ND	ND	ND	10.94 ± 1.27	ND	ND
79	d-(-)-pantolactone	ND	ND	ND	ND	ND	ND	ND	8.27 ± 0.96	ND	ND
80	6-tridecanone	ND	ND	ND	ND	ND	ND	ND	ND	3.65 ± 0.86	ND
81	2-nonadecanone	ND	ND	ND	ND	ND	ND	ND	ND	6.96 ± 0.64	ND
82	2,2-dimethyl-3-heptanone	ND	ND	ND	ND	ND	ND	ND	ND	8.09 ± 4.89	5.02 ± 1.6
83	3,5-octanedione, 4,6-dimethyl-	ND	ND	ND	ND	ND	ND	ND	ND	7.43 ± 2.14	ND
84	3-octanone	ND	ND	ND	ND	ND	ND	ND	ND	1.69 ± 0	ND
85	3-hexanone, 2,5-dimethyl-4-nitro-	ND	ND	ND	ND	ND	ND	ND	ND	2.17 ± 0	ND
	Esters	43.50 ± 10.61	21.14 ± 5.48	11.04 ± 0.75	29.02 ± 4.74	60.97 ± 9.03	50.33 ± 12.9	833.66 ± 95.35	136.57 ± 13.35	753.10 ± 114.21	231.42 ± 56.77
86	Methyl 10-octadecenoate	7.76 ± 2.62	ND	1.27 ± 0.13	ND	ND	ND	5.02 ± 0	23.06 ± 4.28	102.11 ± 34.15	45.94 ± 11.34
87	Vinyl 2,2-dimethylbutanoate	ND	ND	ND	ND	ND	ND	2.06 ± 0.84	ND	ND	ND
88	methyl palmitate	12.89 ± 1.97	10.37 ± 6.66	4.77 ± 0.18	5.94 ± 1.15	14.78 ± 1.53	11.24 ± 2.88	271.91 ± 41.1	54.26 ± 11.9	201.59 ± 100.2	72.57 ± 44.67
89	methyl stearate	2.37 ± 0.92	ND	1.08 ± 0	0.8 ± 0.09	2 ± 0.31	1.6 ± 0.39	43.46 ± 2.34	8.53 ± 0.21	5.13 ± 1.78	ND
90	methyl octadeca-9,12-dienoate	20.48 ± 7.93	ND	ND	ND	ND	ND	ND	39.77 ± 0.54	24.92 ± 8.29	68.96 ± 8.4
91	elaidic acid ethyl ester	ND	4.55 ± 1.02	ND	3.36 ± 0.85	ND	ND	ND	ND	ND	ND
92	ethyl palmitate	ND	6.22 ± 1.18	1.61 ± 0.57	4.05 ± 0.33	4.64 ± 1.22	3.62 ± 1.28	ND	ND	ND	ND
93	methyl (*E*)-octadec-11-enoate	ND	ND	2.31 ± 0	2.64 ± 0.69	6.65 ± 0.77	ND	ND	ND	ND	ND
94	ethyl linoleate	ND	ND	ND	5.87 ± 1.87	ND	6.19 ± 1.22	ND	ND	ND	ND
95	2,4-decadienoic acid methyl ester	ND	ND	ND	1.49 ± 0.43	ND	ND	ND	ND	ND	ND
96	methyl linolenate	ND	ND	ND	ND	1.88 ± 0.91	ND	ND	ND	ND	4.55 ± 0.64
97	methyl trans-9-octadecenoate	ND	ND	ND	ND	10.15 ± 1.99	8.43 ± 3.57	ND	ND	ND	ND
98	methyl 2-oxohexanoate	ND	ND	ND	ND	ND	1.97 ± 0.59	2.47 ± 0.86	ND	ND	ND
99	methyl heptanoate	ND	ND	ND	ND	ND	ND	11.76 ± 7.21	ND	5.81 ± 0.55	ND
100	caprylic acid methyl ester	ND	ND	ND	ND	ND	ND	16.41 ± 8	ND	8.02 ± 3.88	ND
101	heptyl acetate	ND	ND	ND	ND	ND	ND	6.56 ± 0.35	ND	ND	ND
102	2-octenoic acid, 2-hexenyl ester, (*E*,*E*)- (9ci)	ND	ND	ND	ND	ND	ND	128.85 ± 15	ND	146.71 ± 25.63	16.41 ± 5.01
103	methyl nonanoate	ND	ND	ND	ND	ND	ND	14.85 ± 11.12	ND	ND	ND
104	9-oxo-nonanoic acid methyl ester	ND	ND	ND	ND	ND	ND	8.17 ± 2.95	ND	13.5 ± 7.76	1.32 ± 0.12
105	hexanethioic acid s-heptyl ester	ND	ND	ND	ND	ND	ND	4.1 ± 1.22	ND	1.82 ± 0.04	ND
106	vinyl hexanoate	ND	ND	ND	ND	ND	ND	25.93 ± 4.03	ND	50.23 ± 11.19	8.04 ± 2.22
107	hexadecanoic acid, pentyl ester	ND	ND	ND	ND	ND	ND	5.25 ± 1.57	ND	ND	ND
108	*n*-octyl caproate	ND	ND	ND	ND	ND	ND	10.84 ± 0.8	ND	ND	ND
109	1-butyl nitrite	ND	ND	ND	ND	ND	ND	21.88 ± 0.5	ND	ND	ND
110	13-octadecenoic acid methyl ester	ND	ND	ND	ND	ND	ND	89.89 ± 7.72	ND	ND	ND
111	γ-hexalactone	ND	ND	ND	ND	ND	ND	17.6 ± 3.19	ND	7.57 ± 0.37	ND
112	*n*-heptyl caproate	ND	ND	ND	ND	ND	ND	2.45 ± 0.86	ND	ND	ND
113	ethyl stearate	ND	ND	ND	ND	ND	ND	2.9 ± 0.92	ND	ND	ND
114	heptacosanoic acid methyl ester	ND	ND	ND	ND	ND	ND	1.8 ± 0.69	ND	ND	ND
115	9,15-octadecadienoic acid methyl ester	ND	ND	ND	ND	ND	ND	6.91 ± 1.46	ND	ND	ND
116	gamma-octanoic lactone	ND	ND	ND	ND	ND	ND	5.57 ± 1.85	ND	ND	ND
117	4,8,12-trimethyltridecanoic acid methyl ester	ND	ND	ND	ND	ND	ND	3.16 ± 0.01	ND	ND	ND
118	methyl oleate	ND	ND	ND	ND	ND	ND	ND	ND	19.47 ± 0.23	ND
119	delta-nonalactone	ND	ND	ND	ND	ND	ND	ND	ND	6.49 ± 0.65	ND
120	4-dodecanolide	ND	ND	ND	ND	ND	ND	ND	ND	2.7 ± 0.29	ND
	Alkanes	33.84 ± 3.02	7.91 ± 0.22	7.36 ± 0.12	3.3 ± 0.03	183.95 ± 89.41	23.5 ± 2.24	73.87 ± 4.23	18.41 ± 3.55	21.73 ± 0.23	36.05 ± 1.04
121	*n*-octacosane	16.51 ± 3.63	4.17 ± 0.17	2.19 ± 0.25	1.21 ± 0	33.74 ± 17.85	ND	ND	ND	ND	16.37 ± 1.62
122	2-allylcyclohexane-1,3-dione	6.52 ± 0.31	ND	ND	ND	ND	ND	ND	ND	ND	ND
123	tetratetracontane	5.5 ± 0	ND	ND	ND	15.61 ± 2.03	ND	3.29 ± 0.91	ND	ND	ND
124	heneicosane	2.1 ± 0	ND	1.69 ± 0	0.67 ± 0	10.68 ± 2.51	4.43 ± 2.24	ND	ND	ND	10.24 ± 1.24
125	hexatriacontane	2.88 ± 0	ND	ND	ND	ND	ND	ND	ND	ND	ND
126	tetracosane, 2-methyl-	6.84 ± 0.61	ND	ND	ND	12.89 ± 4.73	ND	ND	ND	ND	ND
127	27-methyloctacosane	ND	3.74 ± 0.25	ND	ND	ND	ND	ND	ND	ND	ND
128	*n*-pentacosane	ND	ND	1.54 ± 0.14	ND	13.07 ± 2.28	7.91 ± 1.66	ND	18.41 ± 3.55	ND	ND
129	*n*-heptacosane	ND	ND	1.94 ± 0	1.44 ± 0.31	ND	ND	ND	ND	ND	ND
130	*n*-tetracontane	ND	ND	ND	ND	65.18 ± 68.22	ND	ND	ND	ND	ND
131	twenty-two alkanes	ND	ND	ND	ND	12.19 ± 4.06	6.2 ± 1.54	ND	ND	ND	ND
132	*n*-triacontane	ND	ND	ND	ND	5.01 ± 0.98	ND	ND	ND	ND	ND
133	2-methylhexadecane	ND	ND	ND	ND	15.58 ± 2.37	4.97 ± 1.85	ND	ND	ND	ND
134	*n*-hexacosane	ND	ND	ND	ND	ND	ND	ND	ND	ND	ND
135	hexane	ND	ND	ND	ND	ND	ND	46.33 ± 2.13	ND	ND	ND
136	7-methyloctan-4-ol	ND	ND	ND	ND	ND	ND	ND	ND	8.57 ± 3.09	ND
137	*n*-tetracosane	ND	ND	ND	ND	ND	ND	ND	ND	ND	9.45 ± 1.42
-1	Alkenes	1.78 ± 0.03	4.72 ± 0.31	1.05 ± 0.15	2.58 ± 0.18	4.16 ± 0.46	1.66 ± 0.11	ND	4.44 ± 0.42	24.32 ± 0.73	3.94 ± 1.32
138	neophytadiene	1.78 ± 0.03	4.72 ± 0.31	1.05 ± 0.15	2.58 ± 0.18	1.38 ± 0.46	1.66 ± 0.11	ND	4.44 ± 0.42	ND	ND
139	3-[(*E*)-3-methyl-1-butenyl]-1-cyclohexene	ND	ND	ND	ND	1.27 ± 0.42	ND	ND	ND	ND	3.94 ± 1.32
140	squalene	ND	ND	ND	ND	1.5 ± 0.36	ND	ND	ND	ND	ND
141	trans-3-hexene	ND	ND	ND	ND	ND	ND	ND	ND	13.84 ± 0	ND
142	3-heptene, 2,3-dimethyl-, (*Z*)-(9ci)	ND	ND	ND	ND	ND	ND	ND	ND	5.25 ± 1	ND
143	1-octene, 6-methyl-	ND	ND	ND	ND	ND	ND	ND	ND	ND	2 ± 0.11
144	cyclohexane, 1-hexen-1-yl	ND	ND	ND	ND	ND	ND	ND	ND	5.23 ± 1.12	ND
	Others	ND	ND	2.61 ± 1.39	4.46 ± 0.99	10.33 ± 2.42	5.58 ± 0.85	10.94 ± 1.34	4.17 ± 1.21	9.9 ± 0.51	3.33 ± 0.35
145	2,3-dihydrothiophene	ND	ND	2.61 ± 1.39	2.42 ± 0.5	6.2 ± 2.41	5.58 ± 0.85	ND	4.17 ± 1.21	5.26 ± 1.26	ND
146	(6*E*,10*E*)-7,11,15-trimethyl-3-methylene-1,6,10,14-hexadecatetrene	ND	ND	ND	2.04 ± 0.86	ND	ND	ND	ND	ND	ND
147	1-phenyl-1,3,3	ND	ND	ND	ND	4.13 ± 0.99	ND	ND	ND	ND	ND
148	linoleoyl chloride	ND	ND	ND	ND	ND	ND	5.81 ± 1.34	ND	ND	ND
149	1-acetyl-2,2,4-trimethyl-4-phenyl-1,2,3,4-tetrahydroquinoline	ND	ND	ND	ND	ND	ND	5.13 ± 1.37	ND	ND	ND
150	(*S*)-1-bromo-2-methylbutane	ND	ND	ND	ND	ND	ND	ND	ND	2.26 ± 0.89	ND
151	3,4-dehydro-dl-proline	ND	ND	ND	ND	ND	ND	ND	ND	16.56 ± 2.59	ND
152	1,3,5-triazatricyclo[3.3.1.13,7]decane	ND	ND	ND	ND	ND	ND	ND	ND	5.94 ± 0.51	ND
153	1-octyne	ND	ND	ND	ND	ND	ND	ND	ND	3.96 ± 0.29	ND

**Table A3:** Volatile compounds with VIP scores ≥ 1.0 at 56 days and their dynamic changes from 28 days.

Var ID (CAS)	Compound Name	VIP (28 d)	VIP (56 d)	Trend (28 d → 56 d)	Interpretation
1599-47-9	hexanal dimethyl acetal	2.36	5.01	**↑↑↑**	Top marker
2363-88-4	(*E*,*E*)-2,4-Decadienal (=trans,trans-2,4-Decadienal)	2.61	3.47	**↑**	Key marker
66-25-1	Hexanal	2.58	3.28	**↑**	Key marker
112-39-0	Methyl palmitate (=Methyl hexadecanoate)	N/A	2.31	New	Late-stage marker
18824-63-0	1,1-dimethoxynonane (= Nonanal dimethyl acetal)	0.49	2.13	**↑↑↑**	Late-stage marker
106-70-7	Methyl hexanoate	1.83	2.11	**↑**	Stable marker
10022-28-3	Octanal dimethyl acetal (=1,1-Dimethoxyoctane)	N/A	2.04	New	Late-stage marker
142-62-1	Hexanoic acid	2.93	2.01	**↓** (High)	Stable marker
25152-84-5	(*E*)-2-Nonenal	1.42	1.80	**↑**	Late-stage marker
13481-95-3	Methyl 10-octadecenoate (=10-Octadecenoic acid, methyl ester)	1.06	1.72	**↑**	Stable marker
5187-23-5	5-Ethyl-1,3-dioxane-5-methanol	1.17	1.71	**↑**	Stable marker
2462-85-3	Methyl linoleate (=Methyl octadeca-9,12-dienoate)	1.59	1.67	**↑**	Stable marker
13170-00-8	Vinyl 2,2-dimethylbutanoate	1.32	1.65	**↑**	Stable marker
2548-87-0	(*E*)-2-Octenal	1.00	1.64	**↑**	Stable marker
112-61-8	Methyl stearate	0.89	1.40	**↑**	Late-stage marker
102-52-3	1,1,3,3-Tetramethoxypropane	1.06	1.40	**↑**	Stable marker
16339-12-1	*N*-Nitroso-*N*-methyl-*O*-methyl-hydroxylamine	N/A	1.30	New	Late-stage marker
64-19-7	Acetic acid	N/A	1.27	New	Late-stage marker
4744-10-9	Propionaldehyde dimethyl acetal	1.60	1.26	**↓**	Moderate marker
124-19-6	Nonanal	1.71	1.24	**↓**	Moderate marker
18829-55-5	(*E*)-2-Heptenal	0.90	1.22	**↑**	Late-stage marker
7779-41-1	Decanal dimethyl acetal	N/A	1.17	New	Late-stage marker
3913-81-3	(*E*)-2-Decenal	N/A	1.15	New	Late-stage marker
2883-98-9	α-Asarone	N/A	1.08	New	Late-stage marker
630-06-8	Hexatriacontane	2.93	1.05	**↓↓**	Early-stage marker
69668-82-2	Octa-3,5-dien-2-ol	0.80	1.03	**↑**	Borderline marker
4313-03-5	(*E*,*E*)-2,4-Heptadienal	1.83	0.99	**↓**	Marginally excluded

Note: N/A indicates either missing or unextractable 28-day data, or that the compound wasn’t a top VIP at 28 days but became significant by 56 days. The compound identified as α-asarone (CAS 2883-98-9) corresponds to the internal standard used for semi-quantification and is not an intrinsic volatile of the sample; its inclusion in the VIP list likely reflects co-elution or spectral interference. Arrows indicate the change trend of VIP (Variable Importance in Projection) values from 28 days to 56 days of oxidation: ↑↑↑, dramatic increase (ΔVIP > 2.0); ↑, slight increase (ΔVIP < 1.0); ↓, decrease; ↓↓, dramatic decrease (ΔVIP > 1.5).
