# Supplementary material for: Selective Inhibition of Lipid Oxidation by a Quercetagetin-Rich Extract from Tagetes erecta: A Chemometric Approach Using OPLS-DA and Selectivity Index Analysis
Source: Molecules. 2026 Aug 19;31(16):2891. doi: 10.3390/molecules31162891 (PMC13515886; doi:10.3390/molecules31162891)

PG-1-0d

<<Column>>
Name : HP-88
Thickness : 0.20um
Length : 100.0m
Inside Diameter : 0.25mm

Method

===== Analytical Line 1 =====

Column Oven Temp.	:120.0 °C	
Injection Temp.	:250.00 °C	
Injection Mode	:Split	
Column Flow	:1.04 mL/min	
Split Ratio	:20.0	
Oven Temp. Program		
Rate	Temperature(°C)	Hold Time(min)
-	120.0	1.00
10.00	175.0	10.00
5.00	210.0	5.00
5.00	230.0	15.00

[GCMS-QP2010]
IonSourceTemp :200.00 °C
Interface Temp. :220.00 °C
m/z scanning range：45～500m/z

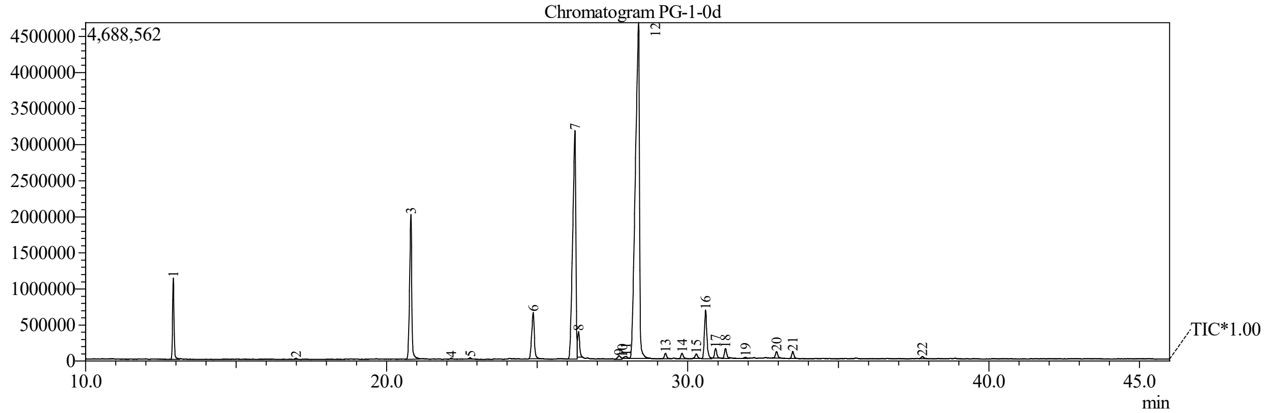

Peak Report TIC

Peak#	R.Time	Area	Area%	Height	Height%	A/H	Name
1	12.917	3786539	3.99	1124877	8.36	3.37	Undecanoic acid, methyl ester
2	16.991	76322	0.08	16934	0.13	4.51	Methyl tetradecanoate
3	20.807	10046122	10.58	2006383	14.91	5.01	Hexadecanoic acid, methyl ester
4	22.151	33565	0.04	10828	0.08	3.10	9-Hexadecenoic acid, methyl ester, (Z)-
5	22.776	67328	0.07	16456	0.12	4.09	Hexadecanoic acid, 15-methyl-, methyl ester
6	24.871	3966962	4.18	645620	4.80	6.14	Methyl stearate
7	26.253	22961158	24.19	3163257	23.51	7.26	9-Octadecenoic acid, methyl ester, (E)-
8	26.374	1664178	1.75	354039	2.63	4.70	11-Octadecenoic acid, methyl ester
9	27.716	350644	0.37	47772	0.36	7.34	9,12-Octadecadienoic acid (Z,Z)-, methyl ester
10	27.825	635	0.00	0	0.00	0.00	1,2,4-Oxadiazole-5-propanoic acid, 3-(4-amin
11	27.939	212017	0.22	26835	0.20	7.90	9,12-Octadecadienoic acid (Z,Z)-, methyl ester
12	28.365	43996727	46.35	4652785	34.58	9.46	9,12-Octadecadienoic acid (Z,Z)-, methyl ester
13	29.261	393147	0.41	75294	0.56	5.22	Methyl 18-methylnonadecanoate
14	29.812	408278	0.43	73244	0.54	5.57	9,12,15-Octadecatrienoic acid, methyl ester, (Z

Peak#	R.Time	Area	Area%	Height	Height%	A/H	Name
15	30.281	375536	0.40	63091	0.47	5.95	9,12,15-Octadecatrienoic acid, methyl ester, (Z
16	30.590	3888141	4.10	670817	4.99	5.80	9,12,15-Octadecatrienoic acid, methyl ester, (Z
17	30.922	678558	0.71	133230	0.99	5.09	Methyl 9-cis,11-trans-octadecadienoate
18	31.252	723869	0.76	134372	1.00	5.39	Methyl 10-trans,12-cis-octadecadienoate
19	31.914	96923	0.10	17395	0.13	5.57	9,11-Octadecadienoic acid, methyl ester, (E,E)
20	32.947	497927	0.52	90389	0.67	5.51	Cis-3-ethyl-endo-tricyclo[5.2.1.0(2.6)]decane
21	33.483	501082	0.53	98610	0.73	5.08	Docosanoic acid, methyl ester
22	37.785	189557	0.20	32576	0.24	5.82	Tetracosanoic acid, methyl ester
		94915215	100.00	13454804	100.00		

Library

<< Target >>
Line#:1 R.Time:12.917(Scan#:351) MassPeaks:394
RawMode:Averaged 12.908-12.925(350-352) BasePeak:74.00(335774)
BG Mode:Calc. from Peak Group 1 - Event 1

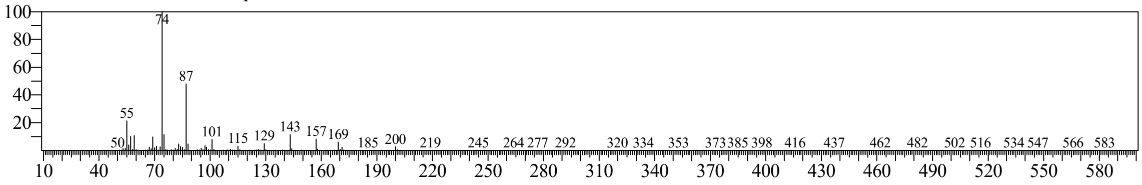

Hit#:1 Entry:18487 Library:NIST14s.lib
SI:95 Formula:C12H24O2 CAS:1731-86-8 MolWeight:200 RetIndex:1381
CompName:Undecanoic acid, methyl ester $$ Methyl undecanoate $$ Methyl undecylenate $$ n-Undecanoic acid methyl ester $$ Methyl ester of undecanoi

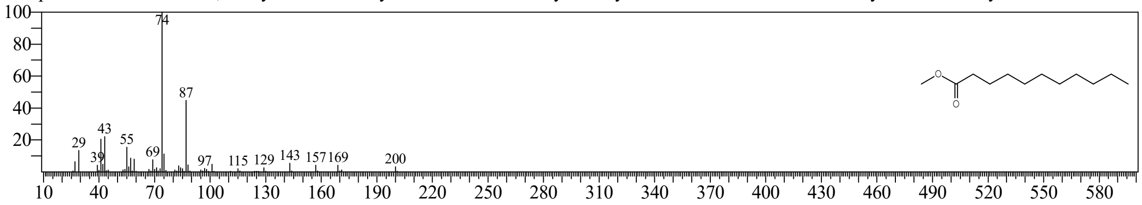

Hit#:2 Entry:21619 Library:NIST17s.lib
SI:95 Formula:C12H24O2 CAS:1731-86-8 MolWeight:200 RetIndex:1381
CompName:Undecanoic acid, methyl ester $$ Methyl undecanoate $$ Methyl undecylenate $$ n-Undecanoic acid methyl ester $$ Methyl ester of undecanoi

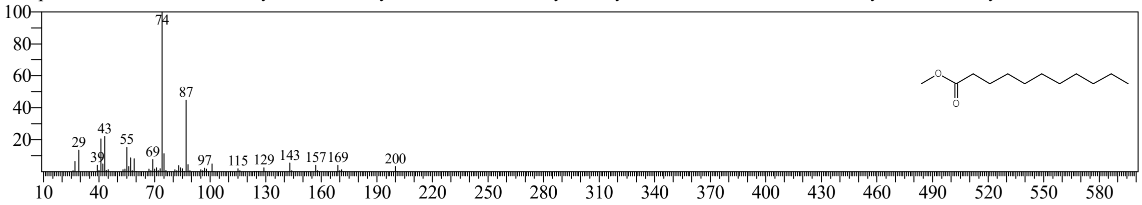

Hit#:3 Entry:21618 Library:NIST17s.lib
SI:95 Formula:C12H24O2 CAS:1731-86-8 MolWeight:200 RetIndex:1381
CompName:Undecanoic acid, methyl ester $$ Methyl undecanoate $$ Methyl undecylenate $$ n-Undecanoic acid methyl ester $$ Methyl ester of undecanoi

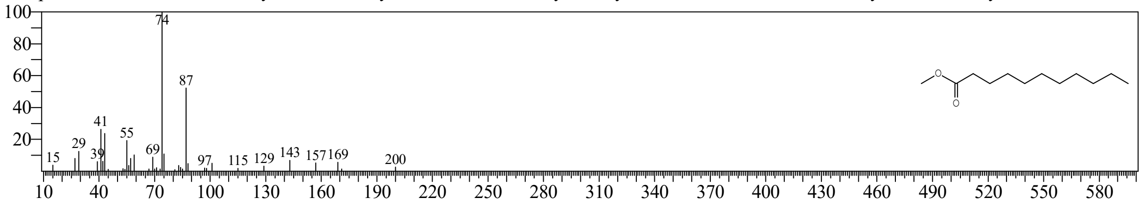

Hit#:4 Entry:49598 Library:NIST17-1.lib
SI:95 Formula:C12H24O2 CAS:1731-86-8 MolWeight:200 RetIndex:1381
CompName:Undecanoic acid, methyl ester $$ Methyl undecanoate $$ Methyl undecylenate $$ n-Undecanoic acid methyl ester $$ Methyl ester of undecanoi

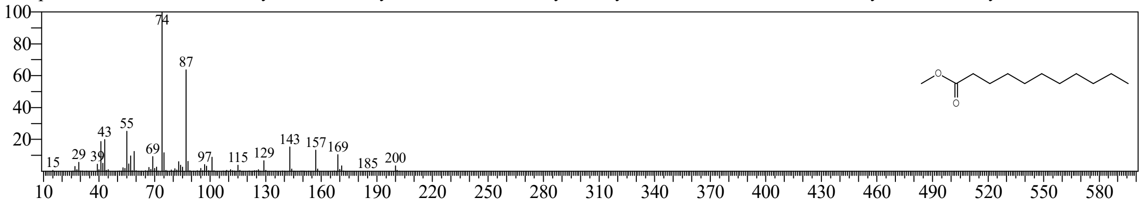

<< Target >>
Line#:1 R.Time:12.917(Scan#:351) MassPeaks:394
RawMode:Averaged 12.908-12.925(350-352) BasePeak:74.00(335774)
BG Mode:Calc. from Peak Group 1 - Event 1

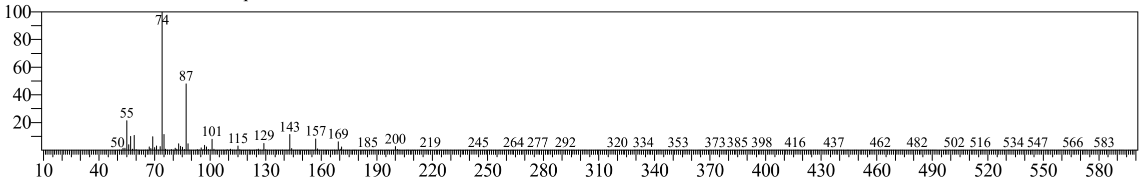

Hit#:5 Entry:46966 Library:NIST14.lib
SI:95 Formula:C12H24O2 CAS:1731-86-8 MolWeight:200 RetIndex:1381
CompName:Undecanoic acid, methyl ester $$ Methyl undecanoate $$ Methyl undecylenate $$ n-Undecanoic acid methyl ester $$ Methyl ester of undecanoi

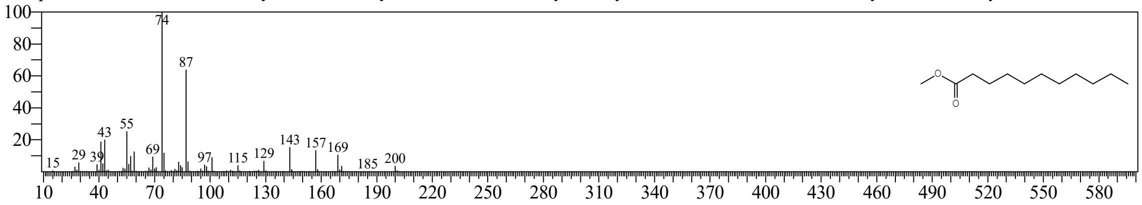

<< Target >>
Line#:2 R.Time:16.992(Scan#:840) MassPeaks:318
RawMode:Averaged 16.983-17.000(839-841) BasePeak:74.00(3607)
BG Mode:Calc. from Peak Group 1 - Event 1

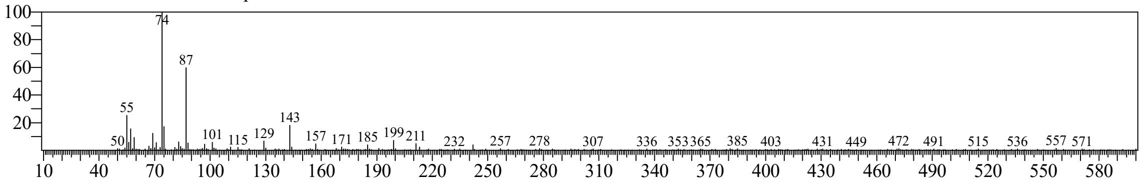

Hit#:1 Entry:80645 Library:NIST14.lib
SI:94 Formula:C15H30O2 CAS:124-10-7 MolWeight:242 RetIndex:1680
CompName:Methyl tetradecanoate $$ Tetradecanoic acid, methyl ester $$ Myristic acid, methyl ester $$ Metholeneat 2495 $$ Methyl myristate $$ Methyl r

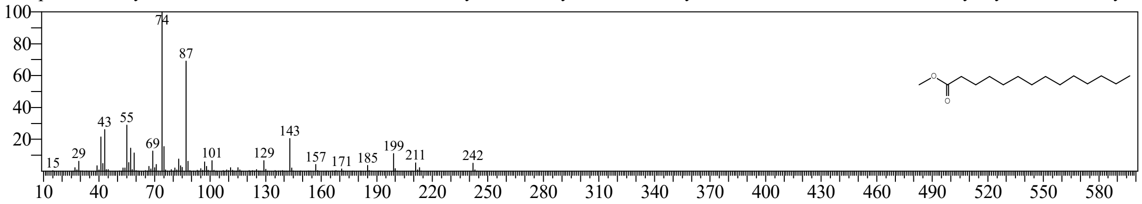

Hit#:2 Entry:86786 Library:NIST17-1.lib
SI:94 Formula:C15H30O2 CAS:124-10-7 MolWeight:242 RetIndex:1680
CompName:Methyl tetradecanoate $$ Tetradecanoic acid, methyl ester $$ Myristic acid, methyl ester $$ Metholeneat 2495 $$ Methyl myristate $$ Methyl r

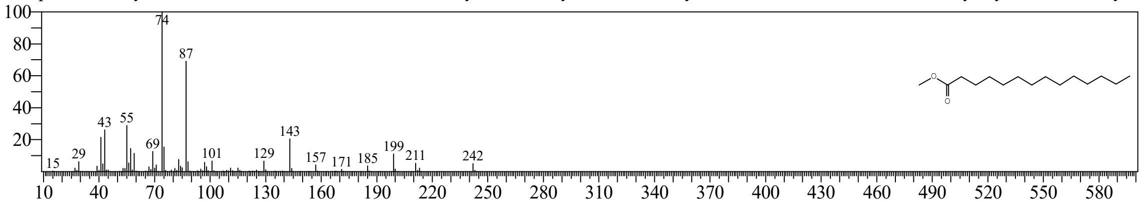

Hit#:3 Entry:27874 Library:NIST17s.lib
SI:93 Formula:C15H30O2 CAS:124-10-7 MolWeight:242 RetIndex:1680
CompName:Methyl tetradecanoate $$ Tetradecanoic acid, methyl ester $$ Myristic acid, methyl ester $$ Metholeneat 2495 $$ Methyl myristate $$ Methyl r

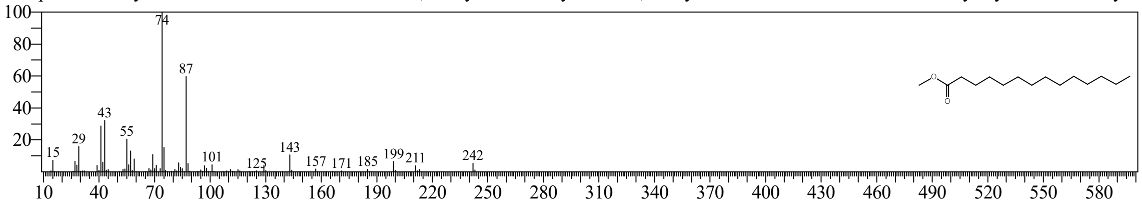

Hit#:4 Entry:23848 Library:NIST14s.lib
SI:93 Formula:C15H30O2 CAS:124-10-7 MolWeight:242 RetIndex:1680
CompName:Methyl tetradecanoate $$ Tetradecanoic acid, methyl ester $$ Myristic acid, methyl ester $$ Metholeneat 2495 $$ Methyl myristate $$ Methyl r

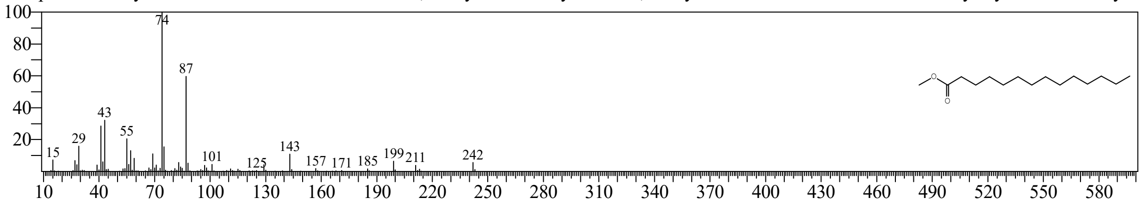

Hit#:5 Entry:27873 Library:NIST17s.lib
SI:93 Formula:C15H30O2 CAS:124-10-7 MolWeight:242 RetIndex:1680
CompName:Methyl tetradecanoate $$ Tetradecanoic acid, methyl ester $$ Myristic acid, methyl ester $$ Metholeneat 2495 $$ Methyl myristate $$ Methyl r

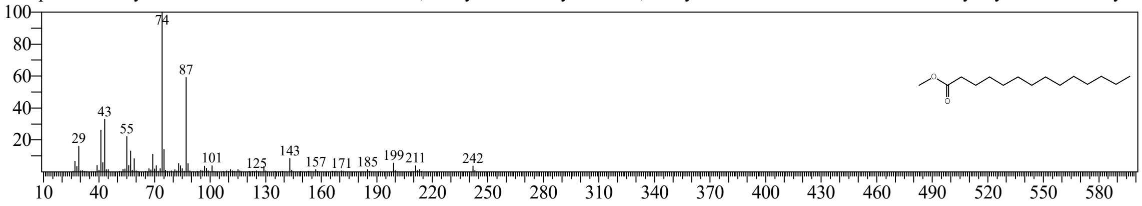

<< Target >>
Line#:3 R.Time:20.808(Scan#:1298) MassPeaks:370
RawMode:Averaged 20.800-20.817(1297-1299) BasePeak:74.00(473588)
BG Mode:Calc. from Peak Group 1 - Event 1

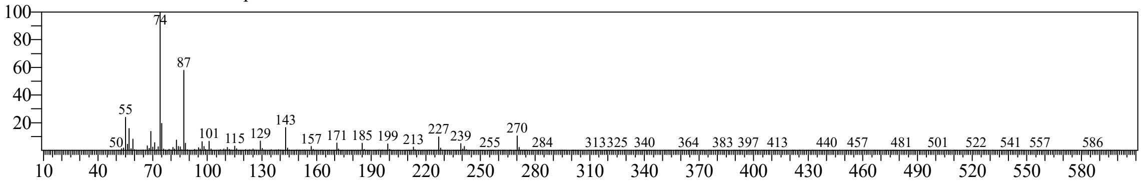

Hit#:1 Entry:26273 Library:NIST14s.lib
SI:97 Formula:C17H34O2 CAS:112-39-0 MolWeight:270 RetIndex:1878
CompName:Hexadecanoic acid, methyl ester $$ Palmitic acid, methyl ester $$ n-Hexadecanoic acid methyl ester $$ Metholene 2216 $$ Methyl hexadecano

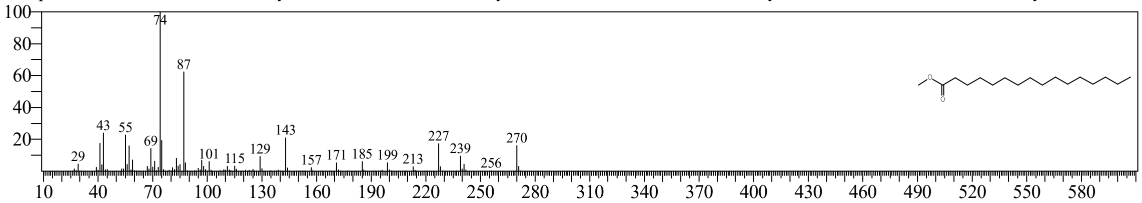

Hit#:2 Entry:113663 Library:NIST17-1.lib
SI:96 Formula:C17H34O2 CAS:112-39-0 MolWeight:270 RetIndex:1878
CompName:Hexadecanoic acid, methyl ester $$ Palmitic acid, methyl ester $$ n-Hexadecanoic acid methyl ester $$ Metholene 2216 $$ Methyl hexadecano

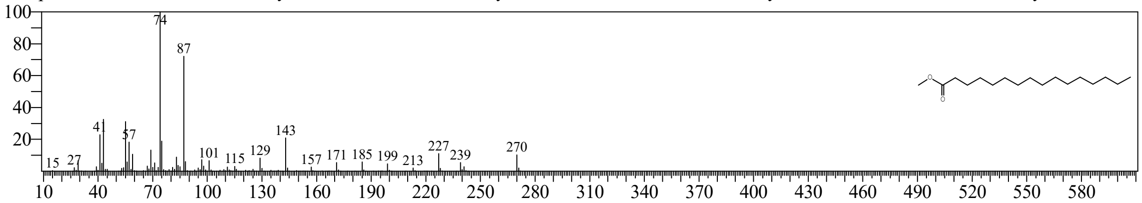

Hit#:3 Entry:104648 Library:NIST14.lib
SI:96 Formula:C17H34O2 CAS:112-39-0 MolWeight:270 RetIndex:1878
CompName:Hexadecanoic acid, methyl ester $$ Palmitic acid, methyl ester $$ n-Hexadecanoic acid methyl ester $$ Metholene 2216 $$ Methyl hexadecano

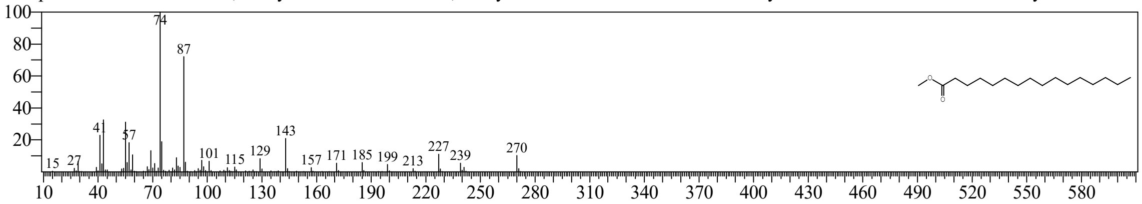

Hit#:4 Entry:30644 Library:NIST17s.lib
SI:96 Formula:C17H34O2 CAS:112-39-0 MolWeight:270 RetIndex:1878
CompName:Hexadecanoic acid, methyl ester $$ Palmitic acid, methyl ester $$ n-Hexadecanoic acid methyl ester $$ Metholene 2216 $$ Methyl hexadecano

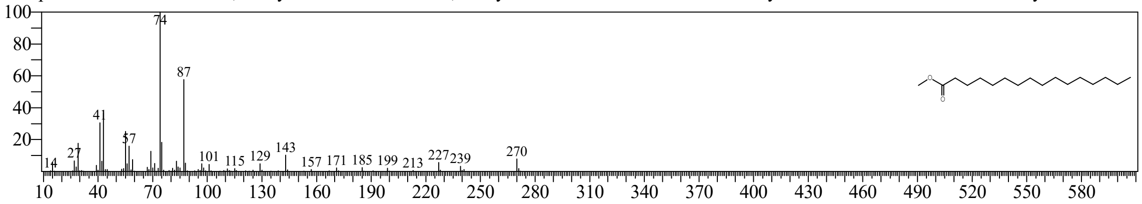

Hit#:5 Entry:26269 Library:NIST14s.lib
SI:96 Formula:C17H34O2 CAS:112-39-0 MolWeight:270 RetIndex:1878
CompName:Hexadecanoic acid, methyl ester $$ Palmitic acid, methyl ester $$ n-Hexadecanoic acid methyl ester $$ Metholene 2216 $$ Methyl hexadecano

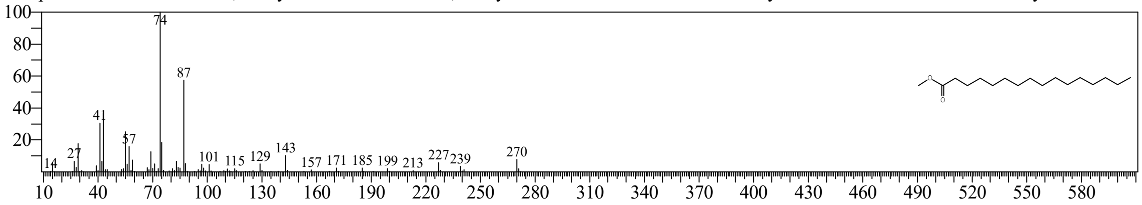

<< Target >>
Line#:4 R.Time:22.150(Scan#:1459) MassPeaks:295
RawMode:Averaged 22.142-22.158(1458-1460) BasePeak:55.05(1115)
BG Mode:Calc. from Peak Group 1 - Event 1

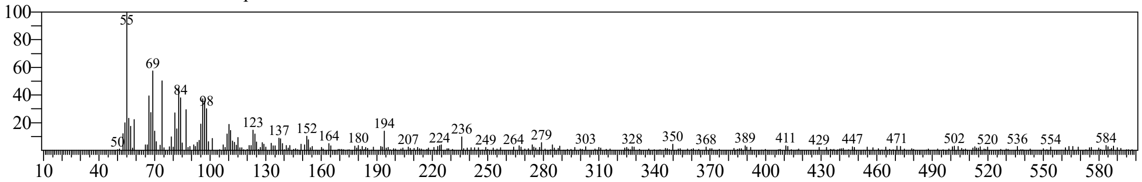

Hit#:1 Entry:26066 Library:NIST14s.lib
SI:91 Formula:C17H32O2 CAS:1120-25-8 MolWeight:268 RetIndex:1886
CompName:9-Hexadecenoic acid, methyl ester, (Z)- $$ Methyl palmitoleate $$ Methyl palmitoleinate $$ Palmitoleic acid, methyl ester $$ Methyl (9Z)-9-he:

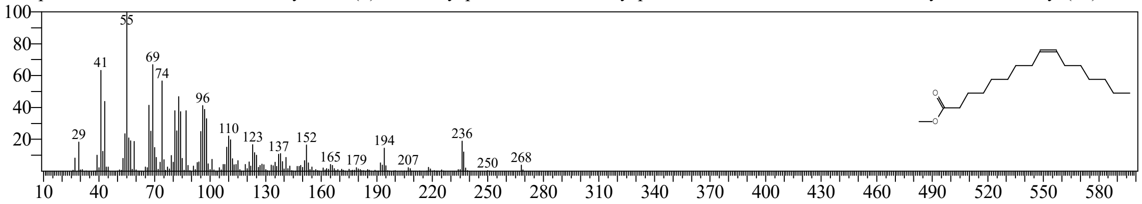

Hit#:2 Entry:30415 Library:NIST17s.lib
SI:91 Formula:C17H32O2 CAS:1120-25-8 MolWeight:268 RetIndex:1886
CompName:9-Hexadecenoic acid, methyl ester, (Z)- $$ Methyl palmitoleate $$ Methyl palmitoleinate $$ Palmitoleic acid, methyl ester $$ Methyl (9Z)-9-he:

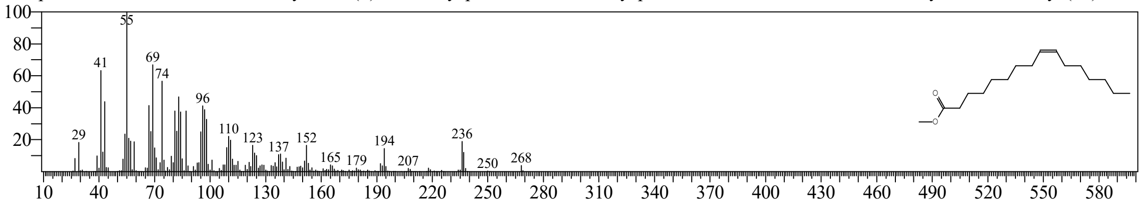

Hit#:3 Entry:111510 Library:NIST17-1.lib
SI:91 Formula:C17H32O2 CAS:56875-67-3 MolWeight:268 RetIndex:1886
CompName:7-Hexadecenoic acid, methyl ester, (Z)- $$ Methyl (7E)-7-hexadecenoate # $$

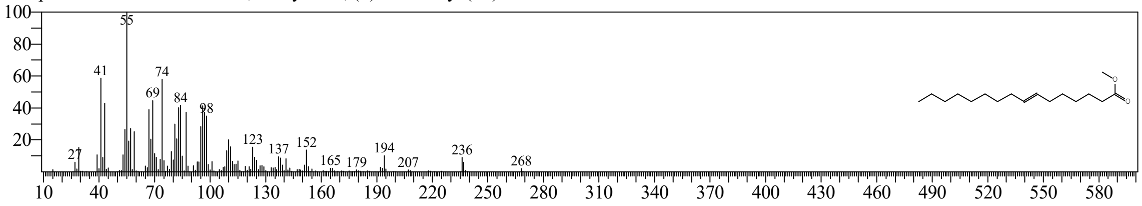

Hit#:4 Entry:30413 Library:NIST17s.lib
SI:91 Formula:C17H32O2 CAS:1120-25-8 MolWeight:268 RetIndex:1886
CompName:9-Hexadecenoic acid, methyl ester, (Z)- $$ Methyl palmitoleate $$ Methyl palmitoleinate $$ Palmitoleic acid, methyl ester $$ Methyl (9Z)-9-he:

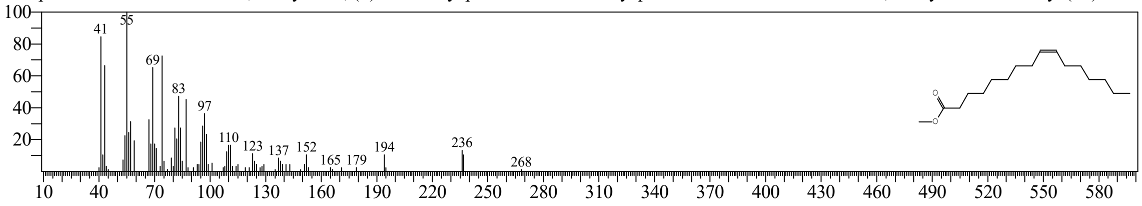

Hit#:5 Entry:26064 Library:NIST14s.lib
SI:91 Formula:C17H32O2 CAS:1120-25-8 MolWeight:268 RetIndex:1886
CompName:9-Hexadecenoic acid, methyl ester, (Z)- $$ Methyl palmitoleate $$ Methyl palmitoleinate $$ Palmitoleic acid, methyl ester $$ Methyl (9Z)-9-he:

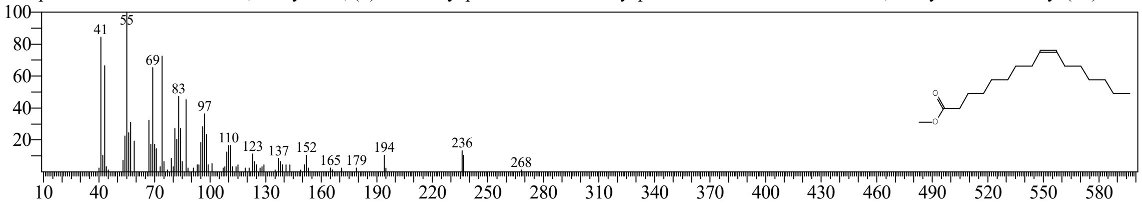

<< Target >>
Line#:5   R.Time:22.775(Scan#:1534) MassPeaks:321
RawMode:Averaged 22.767-22.783(1533-1535) BasePeak:74.00(2985)
BG Mode:Calc. from Peak Group 1 - Event 1

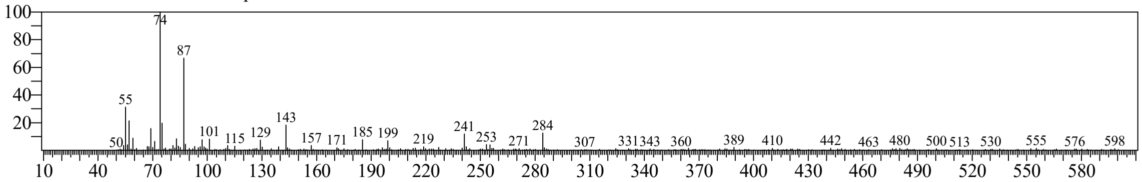

Hit#:1 Entry:31788 Library:NIST17s.lib
SI:92 Formula:C18H36O2 CAS:6929-04-0 MolWeight:284 RetIndex:1914
CompName:Hexadecanoic acid, 15-methyl-, methyl ester $$ Methyl isoheptadecanoate $$ Methyl 15-methylhexadecanoate $$

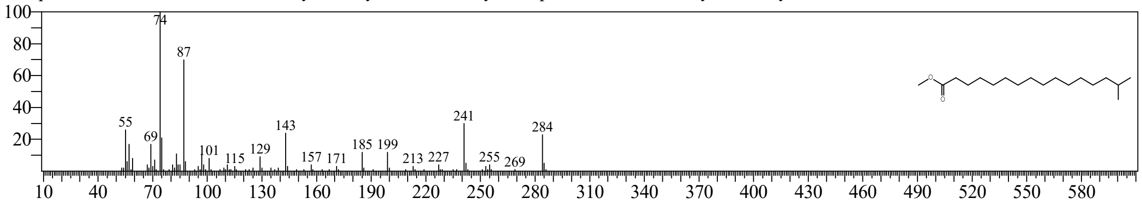

Hit#:2 Entry:27280 Library:NIST14s.lib
SI:92 Formula:C18H36O2 CAS:6929-04-0 MolWeight:284 RetIndex:1914
CompName:Hexadecanoic acid, 15-methyl-, methyl ester $$ Methyl isoheptadecanoate $$ Methyl 15-methylhexadecanoate $$

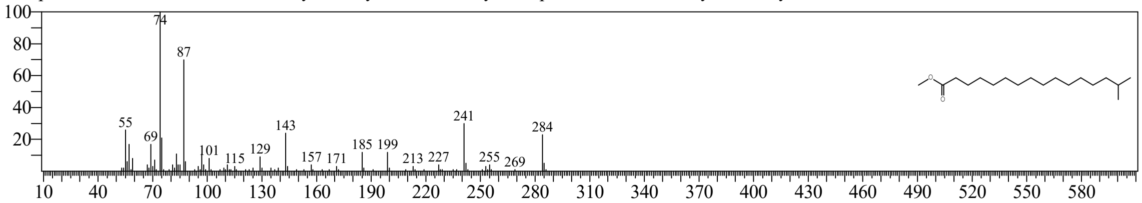

Hit#:3 Entry:127714 Library:NIST17-1.lib
SI:92 Formula:C18H36O2 CAS:6929-04-0 MolWeight:284 RetIndex:1914
CompName:Hexadecanoic acid, 15-methyl-, methyl ester $$ Methyl isoheptadecanoate $$ Methyl 15-methylhexadecanoate $$

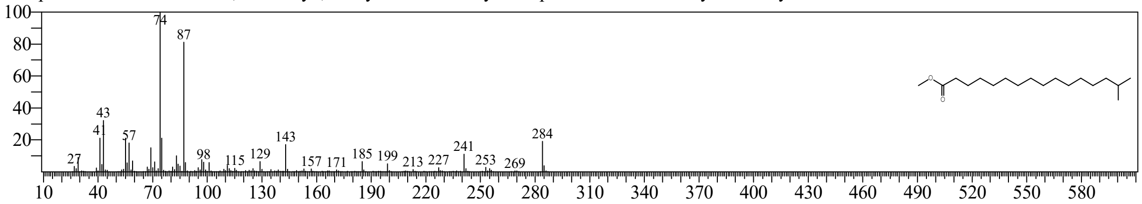

Hit#:4 Entry:27277 Library:NIST14s.lib
SI:92 Formula:C18H36O2 CAS:6929-04-0 MolWeight:284 RetIndex:1914
CompName:Hexadecanoic acid, 15-methyl-, methyl ester $$ Methyl isoheptadecanoate $$ Methyl 15-methylhexadecanoate $$

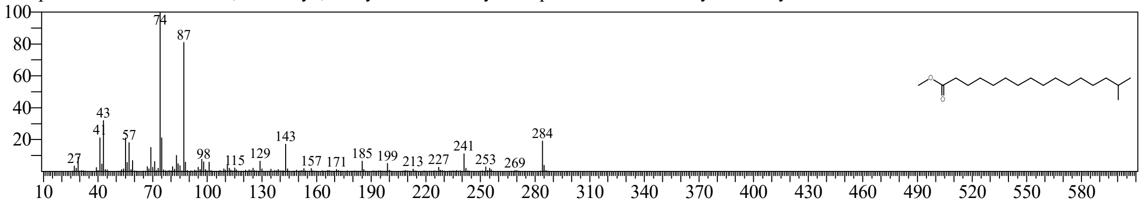

Hit#:5 Entry:27279 Library:NIST14s.lib
SI:92 Formula:C18H36O2 CAS:1731-92-6 MolWeight:284 RetIndex:1978
CompName:Heptadecanoic acid, methyl ester $$ Margaric acid methyl ester $$ Methyl heptadecanoate $$ Methyl margarate $$ n-Heptadecanoic acid methy

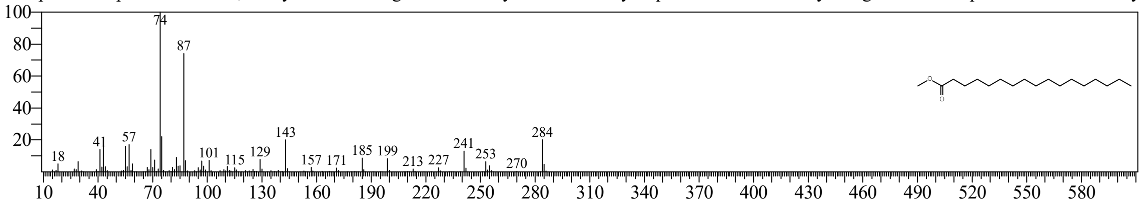

<< Target >>
Line#:6 R.Time:24.867(Scan#:1785) MassPeaks:339
RawMode:Averaged 24.858-24.875(1784-1786) BasePeak:74.00(133369)
BG Mode:Calc. from Peak Group 1 - Event 1

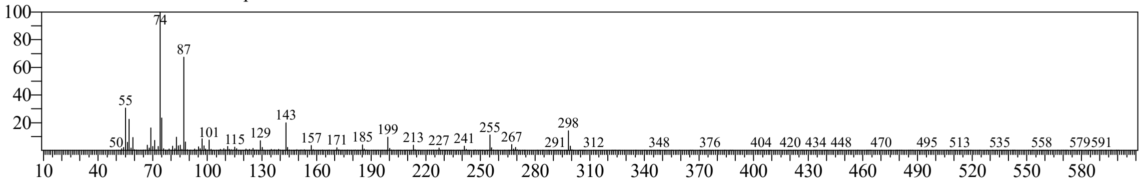

Hit#:1 Entry:129694 Library:NIST14.lib
SI:98 Formula:C19H38O2 CAS:112-61-8 MolWeight:298 RetIndex:2077
CompName:Methyl stearate $$ Octadecanoic acid, methyl ester $$ Stearic acid, methyl ester $$ n-Octadecanoic acid, methyl ester $$ Kemester 9718 $$ Me

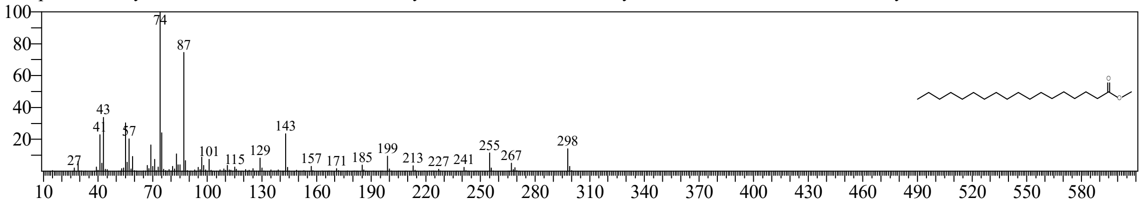

Hit#:2 Entry:141978 Library:NIST17-1.lib
SI:98 Formula:C19H38O2 CAS:112-61-8 MolWeight:298 RetIndex:2077
CompName:Methyl stearate $$ Octadecanoic acid, methyl ester $$ Stearic acid, methyl ester $$ n-Octadecanoic acid, methyl ester $$ Kemester 9718 $$ Me

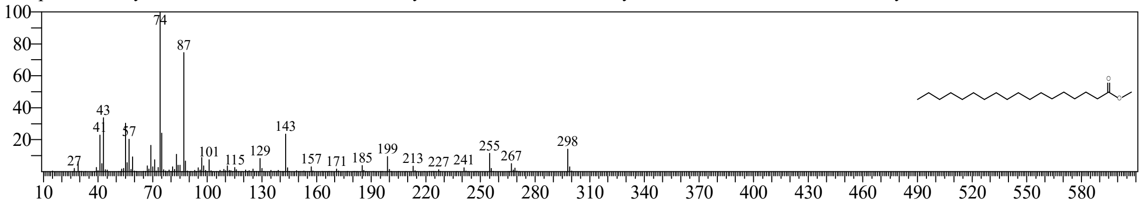

Hit#:3 Entry:28255 Library:NIST14s.lib
SI:97 Formula:C19H38O2 CAS:112-61-8 MolWeight:298 RetIndex:2077
CompName:Methyl stearate $$ Octadecanoic acid, methyl ester $$ Stearic acid, methyl ester $$ n-Octadecanoic acid, methyl ester $$ Kemester 9718 $$ Me

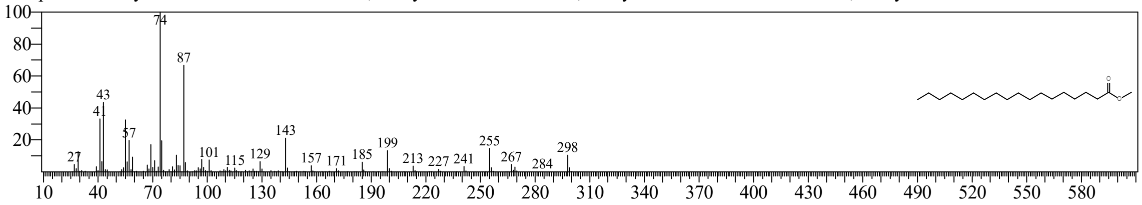

Hit#:4 Entry:32873 Library:NIST17s.lib
SI:97 Formula:C19H38O2 CAS:112-61-8 MolWeight:298 RetIndex:2077
CompName:Methyl stearate $$ Octadecanoic acid, methyl ester $$ Stearic acid, methyl ester $$ n-Octadecanoic acid, methyl ester $$ Kemester 9718 $$ Me

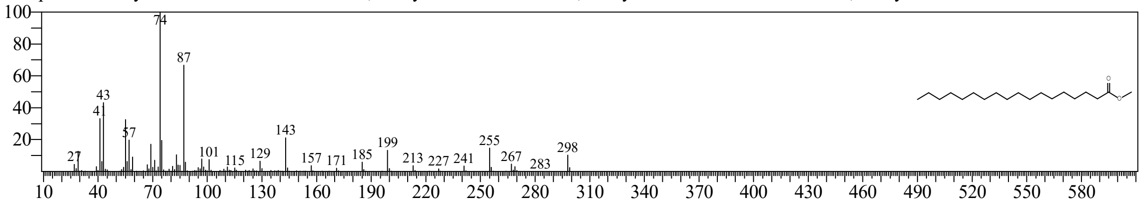

Hit#:5 Entry:28257 Library:NIST14s.lib
SI:96 Formula:C19H38O2 CAS:112-61-8 MolWeight:298 RetIndex:2077
CompName:Methyl stearate $$ Octadecanoic acid, methyl ester $$ Stearic acid, methyl ester $$ n-Octadecanoic acid, methyl ester $$ Kemester 9718 $$ Me

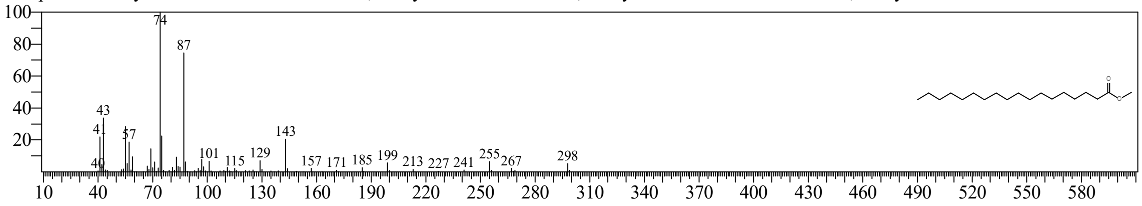

<< Target >>
Line#:7 R.Time:26.250(Scan#:1951) MassPeaks:405
RawMode:Averaged 26.242-26.258(1950-1952) BasePeak:55.05(232705)
BG Mode:Calc. from Peak Group 1 - Event 1

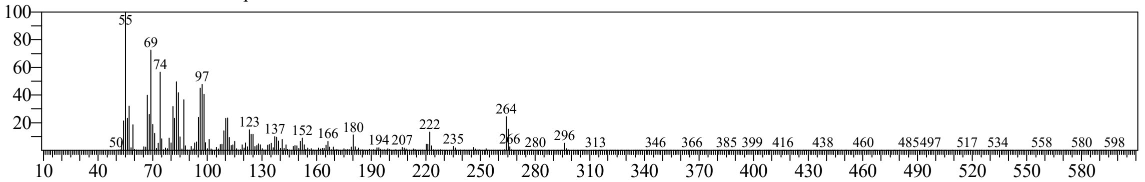

Hit#:1 Entry:28138 Library:NIST14s.lib
SI:97 Formula:C19H36O2 CAS:1937-62-8 MolWeight:296 RetIndex:2085
CompName:9-Octadecenoic acid, methyl ester, (E)- $$ Elaidic acid, methyl ester $$ Methyl elaidate $$ Methyl trans-9-octadecenoate $$ (E)-9-Octadecenoic

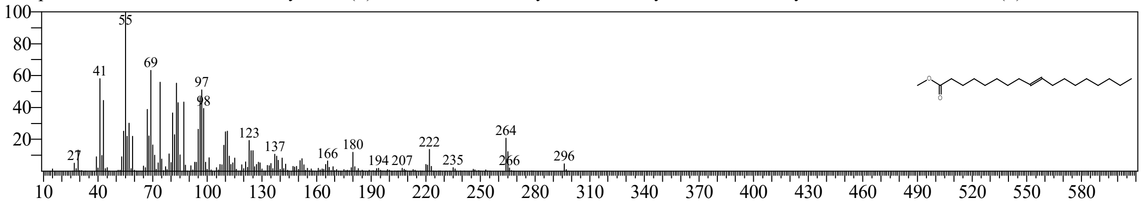

Hit#:2 Entry:32747 Library:NIST17s.lib
SI:97 Formula:C19H36O2 CAS:1937-62-8 MolWeight:296 RetIndex:2085
CompName:9-Octadecenoic acid, methyl ester, (E)- $$ Elaidic acid, methyl ester $$ Methyl elaidate $$ Methyl trans-9-octadecenoate $$ (E)-9-Octadecenoic

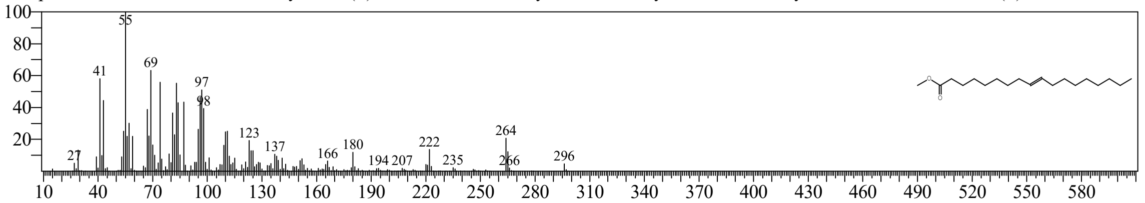

Hit#:3 Entry:139650 Library:NIST17-1.lib
SI:96 Formula:C19H36O2 CAS:112-62-9 MolWeight:296 RetIndex:2085
CompName:9-Octadecenoic acid (Z)-, methyl ester $$ Oleic acid, methyl ester $$ Emery oleic acid ester 2301 $$ Methyl cis-9-octadecenoate $$ Methyl ole

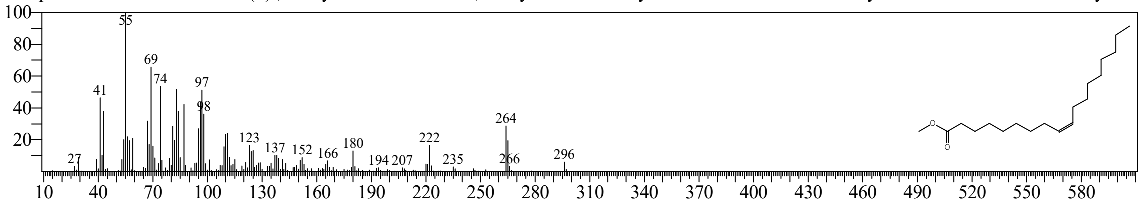

Hit#:4 Entry:127664 Library:NIST14.lib
SI:96 Formula:C19H36O2 CAS:112-62-9 MolWeight:296 RetIndex:2085
CompName:9-Octadecenoic acid (Z)-, methyl ester $$ Oleic acid, methyl ester $$ Emery oleic acid ester 2301 $$ Methyl cis-9-octadecenoate $$ Methyl ole

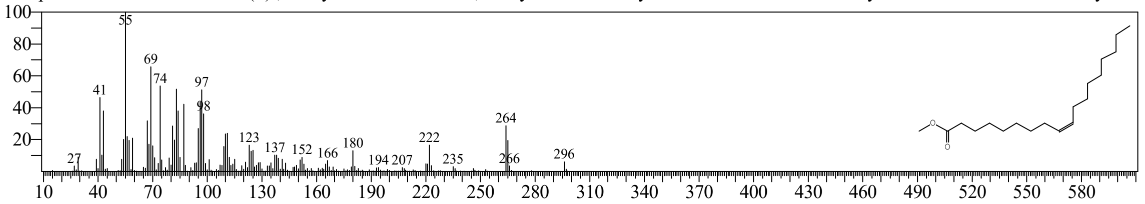

Hit#:5 Entry:127641 Library:NIST14.lib
SI:95 Formula:C19H36O2 CAS:2345-29-1 MolWeight:296 RetIndex:2085
CompName:8-Octadecenoic acid, methyl ester $$ Methyl 8-octadecenoate $$

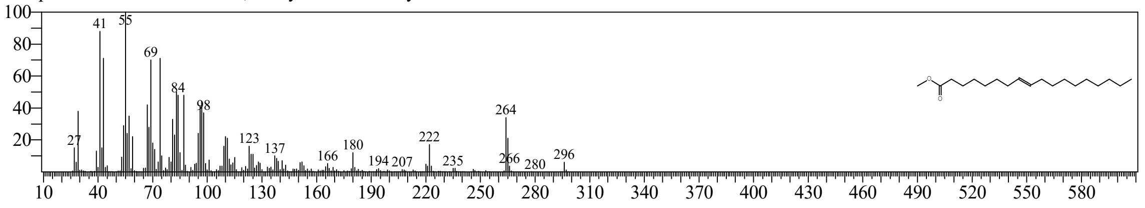

<< Target >>
Line#:8 R.Time:26.375(Scan#:1966) MassPeaks:374
RawMode:Averaged 26.367-26.383(1965-1967) BasePeak:55.05(19212)
BG Mode:Calc. from Peak Group 1 - Event 1

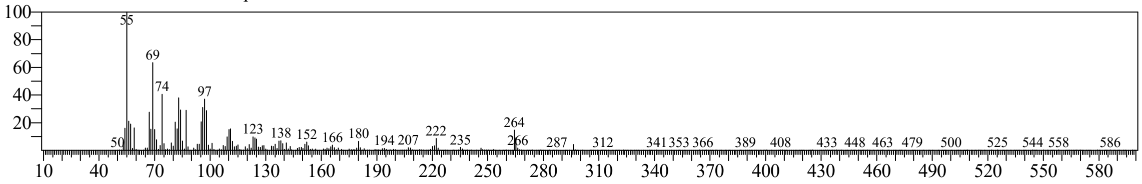

Hit#:1 Entry:32748 Library:NIST17s.lib
SI:95 Formula:C19H36O2 CAS:52380-33-3 MolWeight:296 RetIndex:2085
CompName:11-Octadecenoic acid, methyl ester $$ Methyl 11-octadecenoate $$ Octadec-11-enoic acid, methyl ester $$ Methyl (11E)-11-octadecenoate # $$

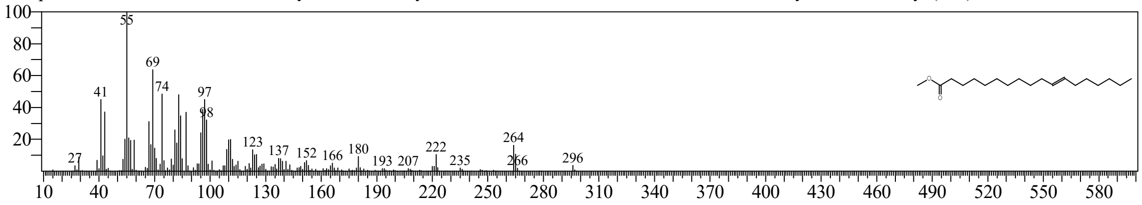

Hit#:2 Entry:28139 Library:NIST14s.lib
SI:95 Formula:C19H36O2 CAS:52380-33-3 MolWeight:296 RetIndex:2085
CompName:11-Octadecenoic acid, methyl ester $$ Methyl 11-octadecenoate $$ Octadec-11-enoic acid, methyl ester $$ Methyl (11E)-11-octadecenoate # $$

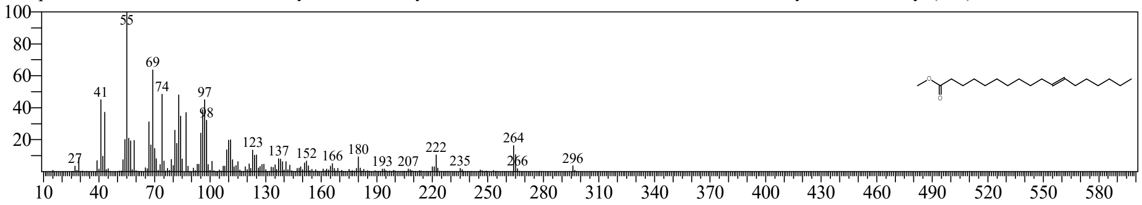

Hit#:3 Entry:127649 Library:NIST14.lib
SI:95 Formula:C19H36O2 CAS:56554-46-2 MolWeight:296 RetIndex:2085
CompName:12-Octadecenoic acid, methyl ester $$ Methyl (12E)-12-octadecenoate # $$

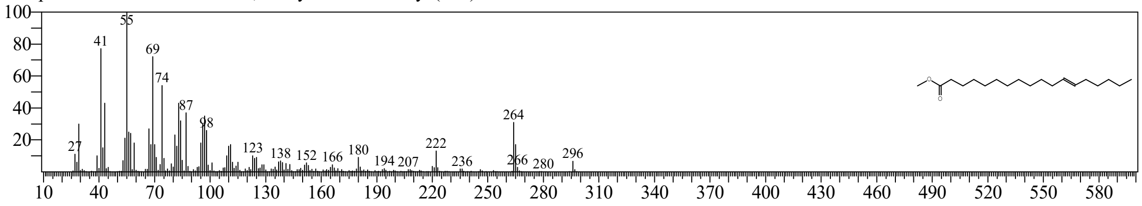

Hit#:4 Entry:127661 Library:NIST14.lib
SI:95 Formula:C19H36O2 CAS:0-00-0 MolWeight:296 RetIndex:2085
CompName:cis-13-Octadecenoic acid, methyl ester

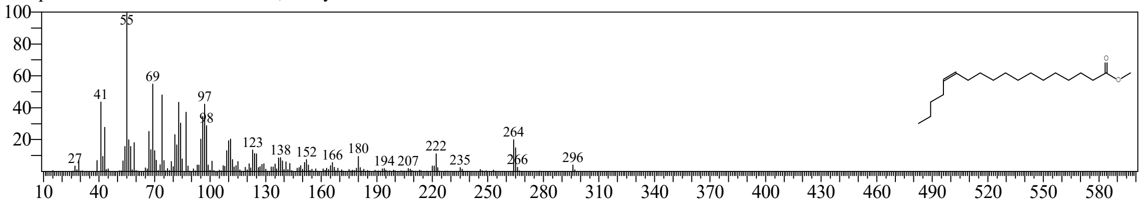

Hit#:5 Entry:139635 Library:NIST17-1.lib
SI:95 Formula:C19H36O2 CAS:56554-46-2 MolWeight:296 RetIndex:2085
CompName:12-Octadecenoic acid, methyl ester $$ Methyl (12E)-12-octadecenoate # $$

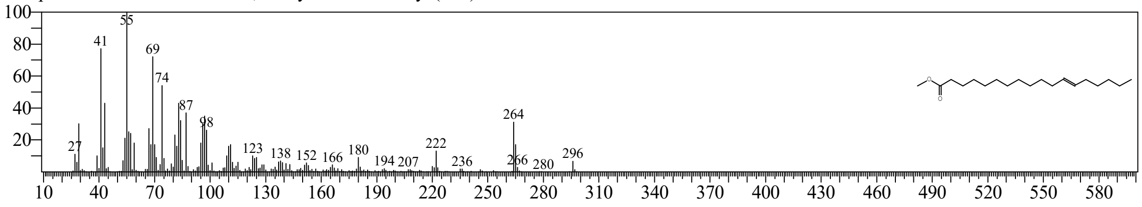

<< Target >>
Line#:9 R.Time:27.717(Scan#:2127) MassPeaks:342
RawMode:Averaged 27.708-27.725(2126-2128) BasePeak:67.00(3842)
BG Mode:Calc. from Peak Group 1 - Event 1

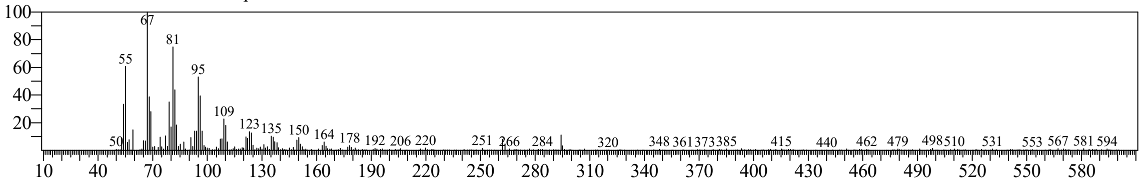

Hit#:1 Entry:27999 Library:NIST14s.lib
SI:96 Formula:C19H34O2 CAS:112-63-0 MolWeight:294 RetIndex:2093
CompName:9,12-Octadecadienoic acid (Z,Z)-, methyl ester $$ Linoleic acid, methyl ester $$ Methyl cis,cis-9,12-octadecadienoate $$ Methyl linoleate $$ M

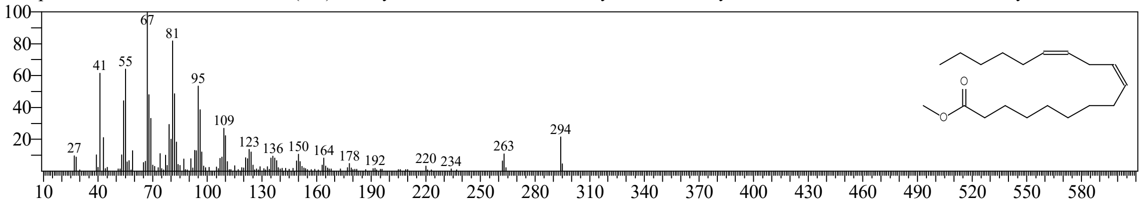

Hit#:2 Entry:32588 Library:NIST17s.lib
SI:96 Formula:C19H34O2 CAS:112-63-0 MolWeight:294 RetIndex:2093
CompName:9,12-Octadecadienoic acid (Z,Z)-, methyl ester $$ Linoleic acid, methyl ester $$ Methyl cis,cis-9,12-octadecadienoate $$ Methyl linoleate $$ M

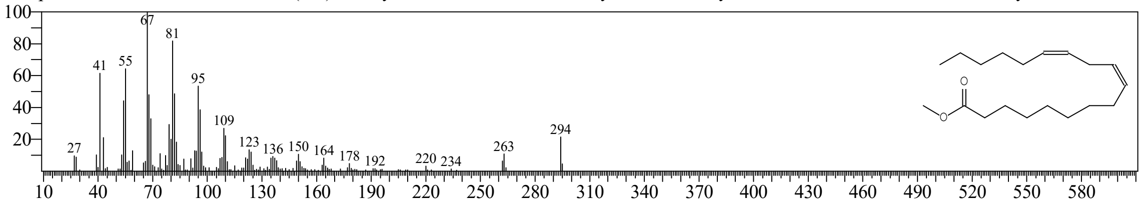

Hit#:3 Entry:32589 Library:NIST17s.lib
SI:96 Formula:C19H34O2 CAS:112-63-0 MolWeight:294 RetIndex:2093
CompName:9,12-Octadecadienoic acid (Z,Z)-, methyl ester $$ Linoleic acid, methyl ester $$ Methyl cis,cis-9,12-octadecadienoate $$ Methyl linoleate $$ M

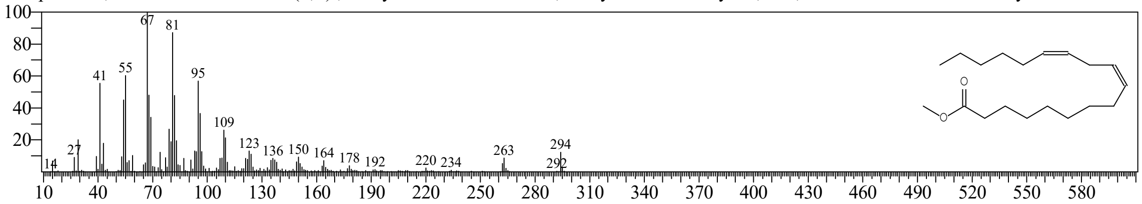

Hit#:4 Entry:28000 Library:NIST14s.lib
SI:96 Formula:C19H34O2 CAS:112-63-0 MolWeight:294 RetIndex:2093
CompName:9,12-Octadecadienoic acid (Z,Z)-, methyl ester $$ Linoleic acid, methyl ester $$ Methyl cis,cis-9,12-octadecadienoate $$ Methyl linoleate $$ M

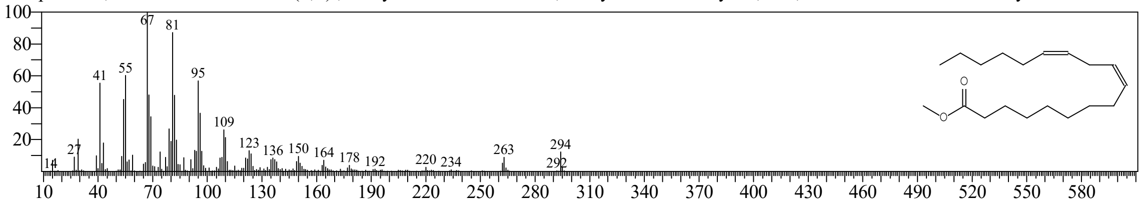

Hit#:5 Entry:125931 Library:NIST14.lib
SI:95 Formula:C19H34O2 CAS:2462-85-3 MolWeight:294 RetIndex:2093
CompName:9,12-Octadecadienoic acid, methyl ester $$ 9,12-Octadecenoic acid, methyl ester $$ Methyl octadeca-9,12-dienoate $$ Methyl 9,12-octadecadi

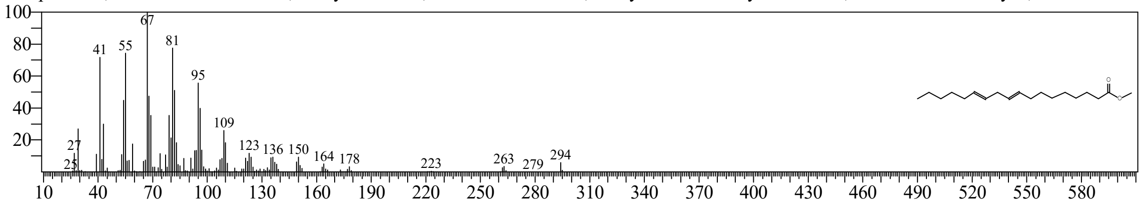

<< Target >>
Line#:10   R.Time:27.817(Scan#:2139) MassPeaks:267
RawMode:Averaged 27.817-27.825(2139-2140) BasePeak:55.10(127)
BG Mode:Calc. from Peak Group 1 - Event 1

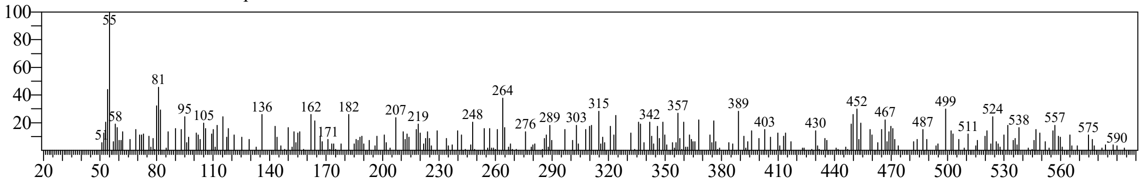

Hit#:1 Entry:83236 Library:NIST17-1.lib
SI:35 Formula:C8H9N5O4 CAS:0-00-0 MolWeight:239 RetIndex:2005
CompName:1,2,4-Oxadiazole-5-propanoic acid, 3-(4-amino-1,2,5-oxadiazol-3-yl)-, methyl ester

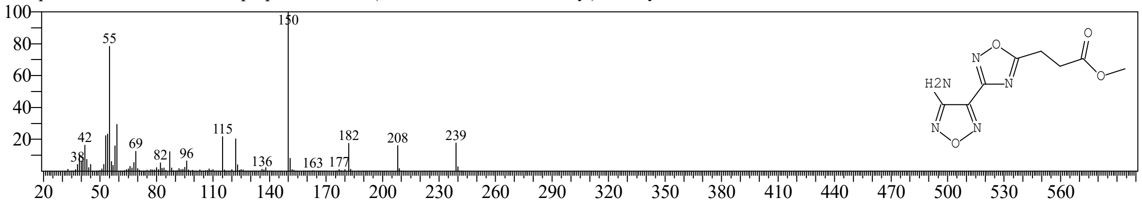

Hit#:2 Entry:77466 Library:NIST14.lib
SI:35 Formula:C8H9N5O4 CAS:0-00-0 MolWeight:239 RetIndex:2005
CompName:1,2,4-Oxadiazole-5-propanoic acid, 3-(4-amino-1,2,5-oxadiazol-3-yl)-, methyl ester

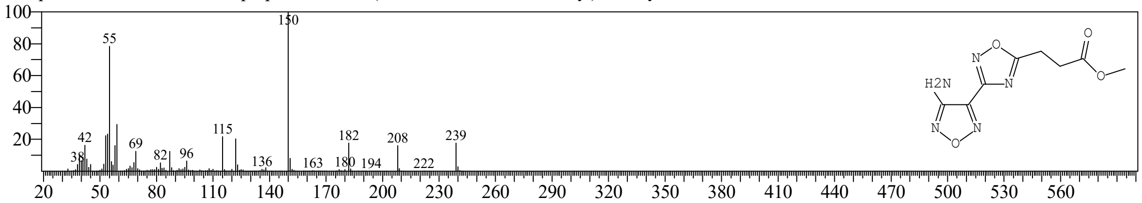

Hit#:3 Entry:44022 Library:NIST17-1.lib
SI:34 Formula:C13H23N CAS:6114-69-8 MolWeight:193 RetIndex:1692
CompName:Cyclohexanamine, N-cycloheptylidene- $$ N-Cycloheptylidenecyclohexanamine # $$

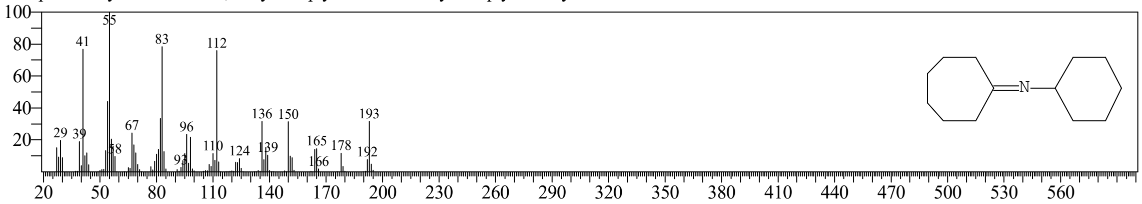

Hit#:4 Entry:41760 Library:NIST14.lib
SI:34 Formula:C13H23N CAS:6114-69-8 MolWeight:193 RetIndex:1692
CompName:Cyclohexanamine, N-cycloheptylidene- $$ N-Cycloheptylidenecyclohexanamine # $$

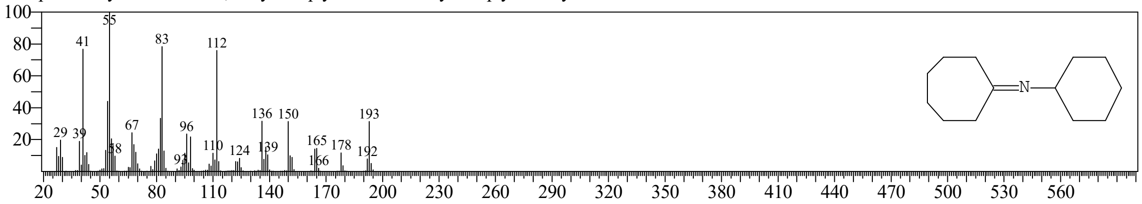

Hit#:5 Entry:138104 Library:NIST14.lib
SI:34 Formula:C16H27F3O2 CAS:159600-12-1 MolWeight:308 RetIndex:1621
CompName:14,14,14-Trifluoro-Z-11-tetradecen-1-ol acetate $$ (11Z)-14,14,14-Trifluoro-11-tetradecenyl acetate # $$

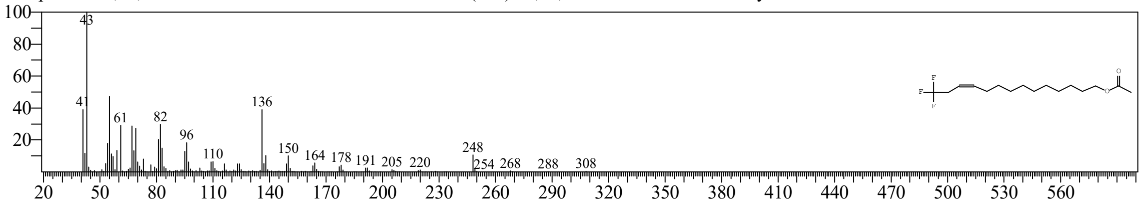

<< Target >>
Line#:11 R.Time:27.942(Scan#:2154) MassPeaks:330
RawMode:Averaged 27.933-27.950(2153-2155) BasePeak:67.00(2490)
BG Mode:Calc. from Peak Group 1 - Event 1

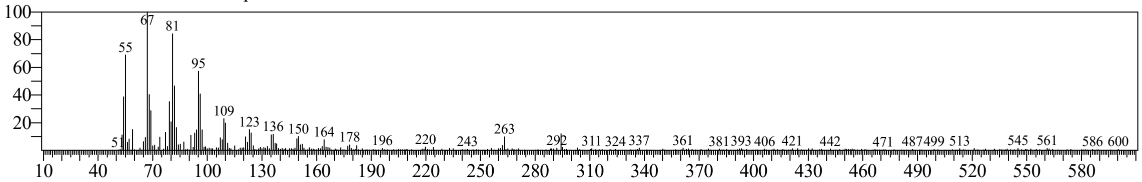

Hit#:1 Entry:27999 Library:NIST14s.lib
SI:96 Formula:C19H34O2 CAS:112-63-0 MolWeight:294 RetIndex:2093
CompName:9,12-Octadecadienoic acid (Z,Z)-, methyl ester $$ Linoleic acid, methyl ester $$ Methyl cis,cis-9,12-octadecadienoate $$ Methyl linoleate $$ M

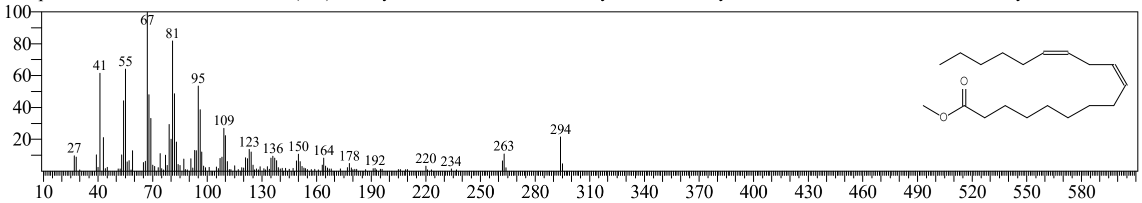

Hit#:2 Entry:32588 Library:NIST17s.lib
SI:96 Formula:C19H34O2 CAS:112-63-0 MolWeight:294 RetIndex:2093
CompName:9,12-Octadecadienoic acid (Z,Z)-, methyl ester $$ Linoleic acid, methyl ester $$ Methyl cis,cis-9,12-octadecadienoate $$ Methyl linoleate $$ M

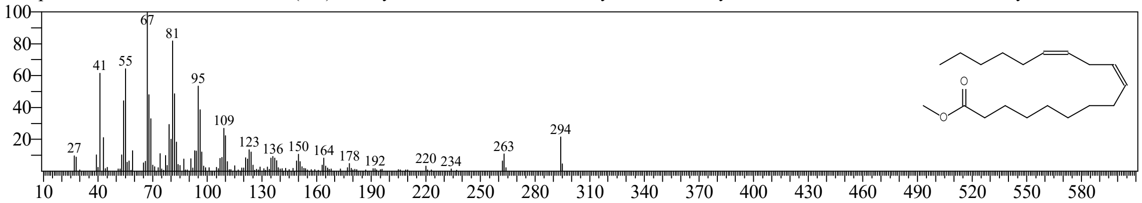

Hit#:3 Entry:137663 Library:NIST17-1.lib
SI:96 Formula:C19H34O2 CAS:2462-85-3 MolWeight:294 RetIndex:2093
CompName:9,12-Octadecadienoic acid, methyl ester $$ 9,12-Octadecenoic acid, methyl ester $$ Methyl octadeca-9,12-dienoate $$ Methyl 9,12-octadecadi

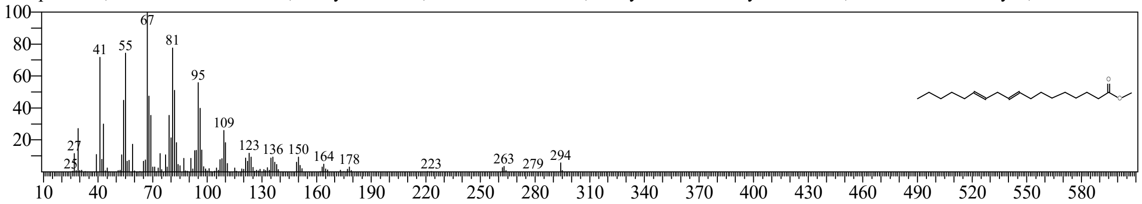

Hit#:4 Entry:125931 Library:NIST14.lib
SI:96 Formula:C19H34O2 CAS:2462-85-3 MolWeight:294 RetIndex:2093
CompName:9,12-Octadecadienoic acid, methyl ester $$ 9,12-Octadecenoic acid, methyl ester $$ Methyl octadeca-9,12-dienoate $$ Methyl 9,12-octadecadi

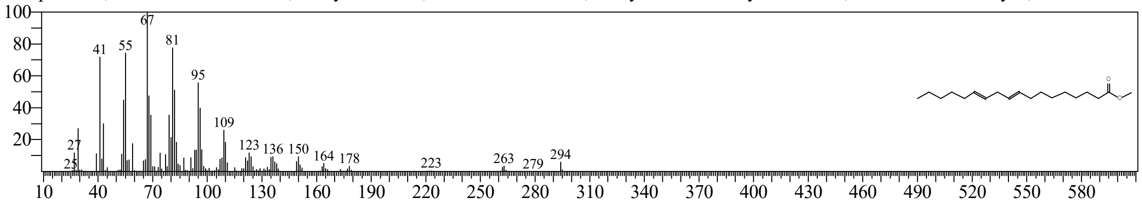

Hit#:5 Entry:32589 Library:NIST17s.lib
SI:96 Formula:C19H34O2 CAS:112-63-0 MolWeight:294 RetIndex:2093
CompName:9,12-Octadecadienoic acid (Z,Z)-, methyl ester $$ Linoleic acid, methyl ester $$ Methyl cis,cis-9,12-octadecadienoate $$ Methyl linoleate $$ M

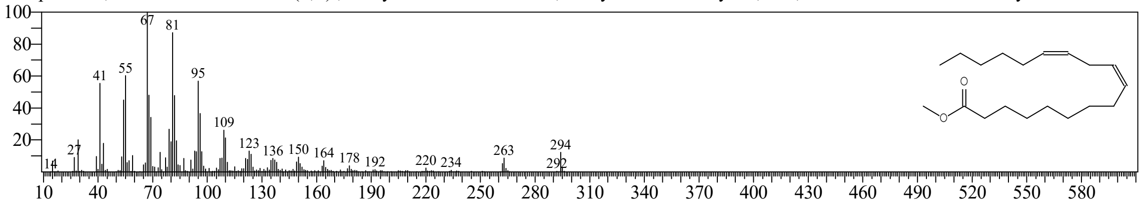

<< Target >>
Line#:12 R.Time:28.367(Scan#:2205) MassPeaks:401
RawMode:Averaged 28.358-28.375(2204-2206) BasePeak:67.05(448190)
BG Mode:Calc. from Peak Group 1 - Event 1

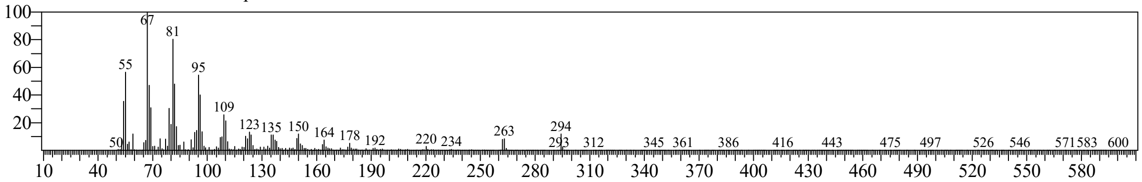

Hit#:1 Entry:32588 Library:NIST17s.lib
SI:97 Formula:C19H34O2 CAS:112-63-0 MolWeight:294 RetIndex:2093
CompName:9,12-Octadecadienoic acid (Z,Z)-, methyl ester $$ Linoleic acid, methyl ester $$ Methyl cis,cis-9,12-octadecadienoate $$ Methyl linoleate $$ M

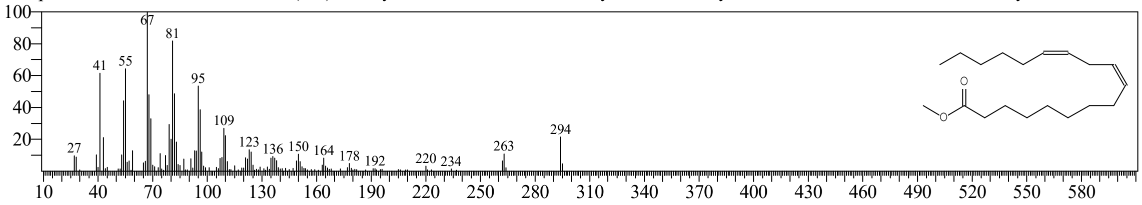

Hit#:2 Entry:27999 Library:NIST14s.lib
SI:97 Formula:C19H34O2 CAS:112-63-0 MolWeight:294 RetIndex:2093
CompName:9,12-Octadecadienoic acid (Z,Z)-, methyl ester $$ Linoleic acid, methyl ester $$ Methyl cis,cis-9,12-octadecadienoate $$ Methyl linoleate $$ M

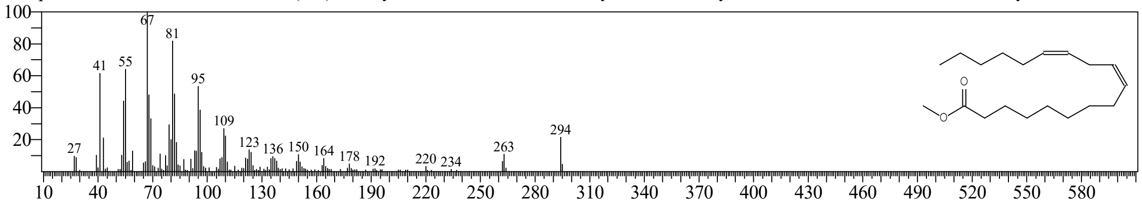

Hit#:3 Entry:32589 Library:NIST17s.lib
SI:97 Formula:C19H34O2 CAS:112-63-0 MolWeight:294 RetIndex:2093
CompName:9,12-Octadecadienoic acid (Z,Z)-, methyl ester $$ Linoleic acid, methyl ester $$ Methyl cis,cis-9,12-octadecadienoate $$ Methyl linoleate $$ M

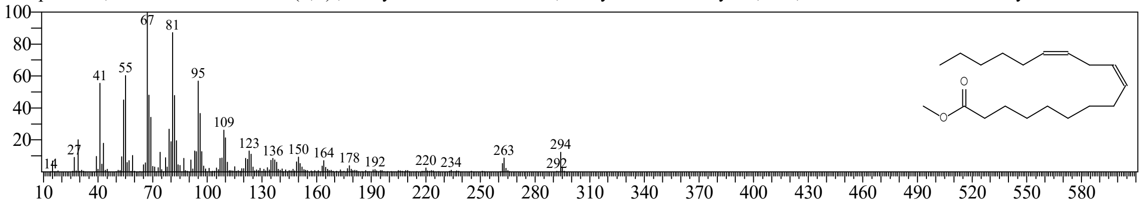

Hit#:4 Entry:28000 Library:NIST14s.lib
SI:97 Formula:C19H34O2 CAS:112-63-0 MolWeight:294 RetIndex:2093
CompName:9,12-Octadecadienoic acid (Z,Z)-, methyl ester $$ Linoleic acid, methyl ester $$ Methyl cis,cis-9,12-octadecadienoate $$ Methyl linoleate $$ M

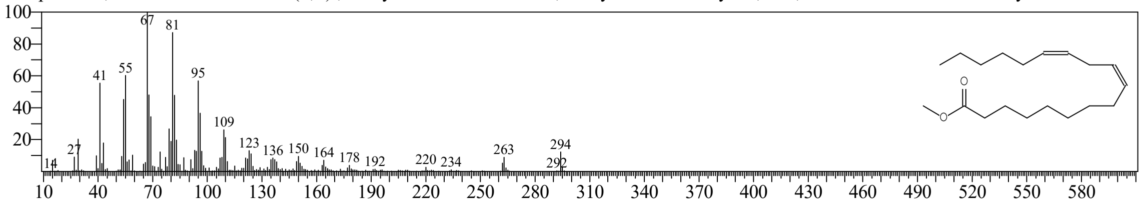

Hit#:5 Entry:137663 Library:NIST17-1.lib
SI:95 Formula:C19H34O2 CAS:2462-85-3 MolWeight:294 RetIndex:2093
CompName:9,12-Octadecadienoic acid, methyl ester $$ 9,12-Octadecenoic acid, methyl ester $$ Methyl octadeca-9,12-dienoate $$ Methyl 9,12-octadecadi

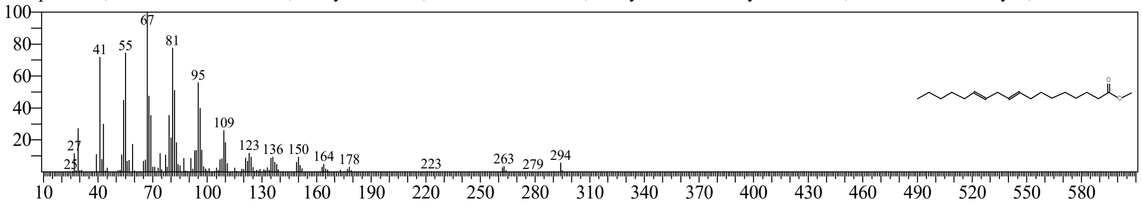

<< Target >>
Line#:13   R.Time:29.258(Scan#:2312) MassPeaks:295
RawMode:Averaged 29.250-29.267(2311-2313) BasePeak:74.00(13885)
BG Mode:Calc. from Peak Group 1 - Event 1

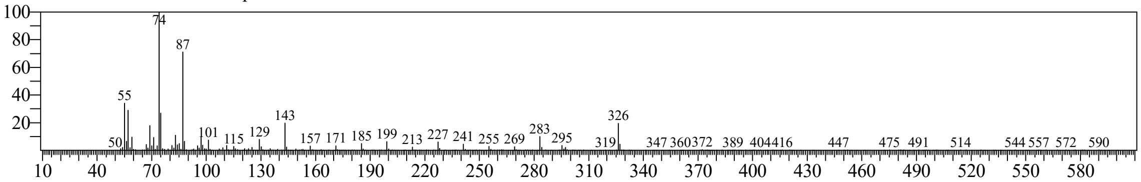

Hit#:1 Entry:170238 Library:NIST17-1.lib
SI:97 Formula:C21H42O2 CAS:0-00-0 MolWeight:326 RetIndex:2212
CompName:Methyl 18-methylnonadecanoate

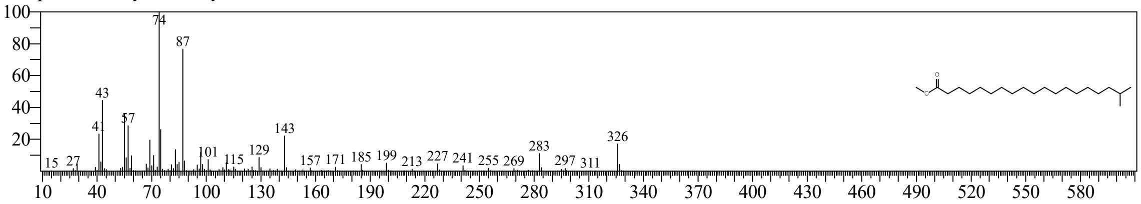

Hit#:2 Entry:154704 Library:NIST14.lib
SI:97 Formula:C21H42O2 CAS:0-00-0 MolWeight:326 RetIndex:2212
CompName:Methyl 18-methylnonadecanoate

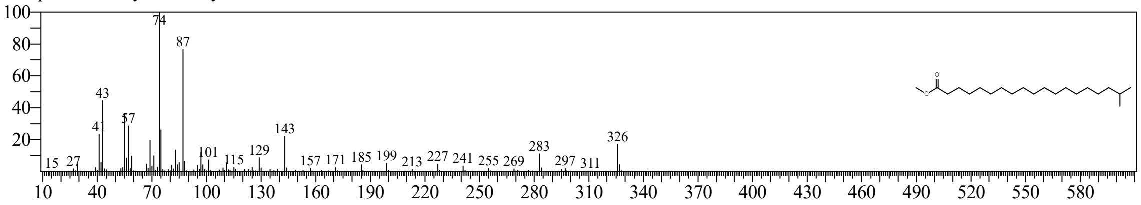

Hit#:3 Entry:34726 Library:NIST17s.lib
SI:96 Formula:C21H42O2 CAS:1120-28-1 MolWeight:326 RetIndex:2276
CompName:Eicosanoic acid, methyl ester $$ Methyl arachisate $$ Methyl eicosanoate $$ Arachidic acid methyl ester $$ Kemester 2050 $$ Methyl aracidat[illegible]

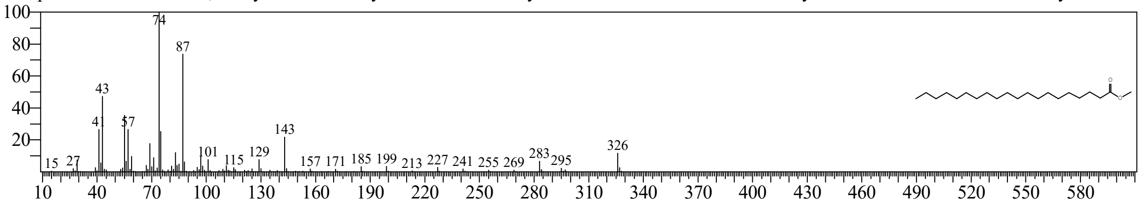

Hit#:4 Entry:29931 Library:NIST14s.lib
SI:93 Formula:C21H42O2 CAS:1120-28-1 MolWeight:326 RetIndex:2276
CompName:Eicosanoic acid, methyl ester $$ Methyl arachisate $$ Methyl eicosanoate $$ Arachidic acid methyl ester $$ Kemester 2050 $$ Methyl aracidat[illegible]

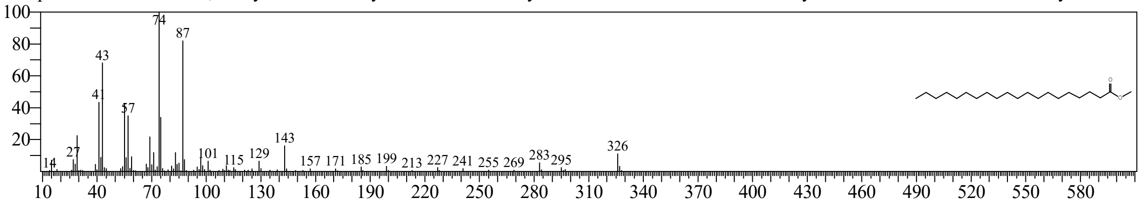

Hit#:5 Entry:34724 Library:NIST17s.lib
SI:93 Formula:C21H42O2 CAS:1120-28-1 MolWeight:326 RetIndex:2276
CompName:Eicosanoic acid, methyl ester $$ Methyl arachisate $$ Methyl eicosanoate $$ Arachidic acid methyl ester $$ Kemester 2050 $$ Methyl aracidat[illegible]

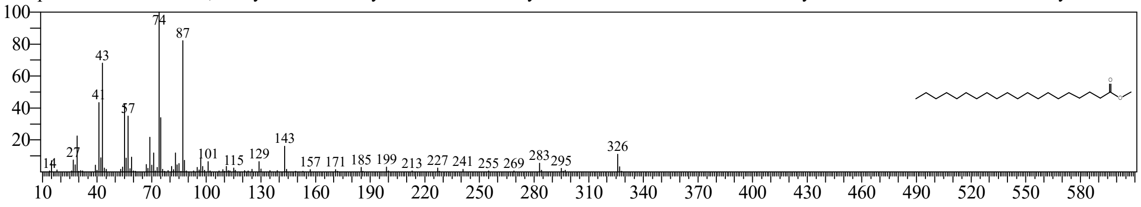

<< Target >>
Line#:14 R.Time:29.808(Scan#:2378) MassPeaks:353
RawMode:Averaged 29.800-29.817(2377-2379) BasePeak:79.05(8316)
BG Mode:Calc. from Peak Group 1 - Event 1

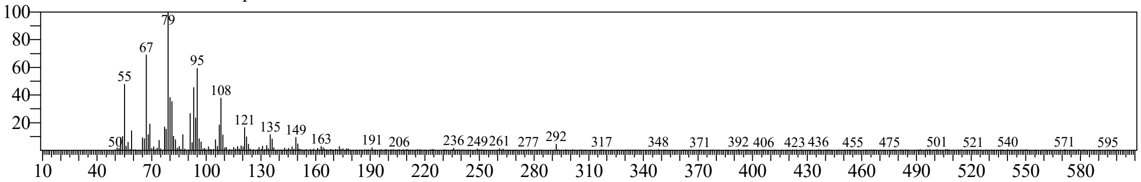

Hit#:1 Entry:32438 Library:NIST17s.lib
SI:95 Formula:C19H32O2 CAS:301-00-8 MolWeight:292 RetIndex:2101
CompName:9,12,15-Octadecatrienoic acid, methyl ester, (Z,Z,Z)- $$ Linolenic acid, methyl ester $$ Methyl all-cis-9,12,15-octadecatrienoate $$ Methyl lino

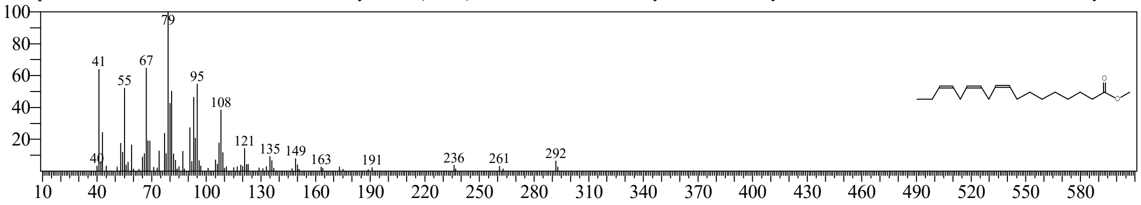

Hit#:2 Entry:135724 Library:NIST17-1.lib
SI:95 Formula:C19H32O2 CAS:301-00-8 MolWeight:292 RetIndex:2101
CompName:9,12,15-Octadecatrienoic acid, methyl ester, (Z,Z,Z)- $$ Linolenic acid, methyl ester $$ Methyl all-cis-9,12,15-octadecatrienoate $$ Methyl lino

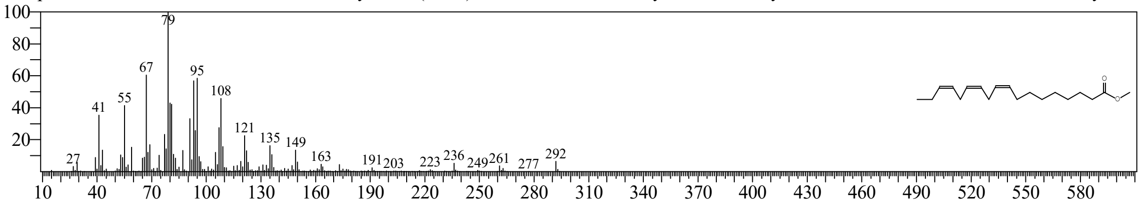

Hit#:3 Entry:124228 Library:NIST14.lib
SI:95 Formula:C19H32O2 CAS:301-00-8 MolWeight:292 RetIndex:2101
CompName:9,12,15-Octadecatrienoic acid, methyl ester, (Z,Z,Z)- $$ Linolenic acid, methyl ester $$ Methyl all-cis-9,12,15-octadecatrienoate $$ Methyl lino

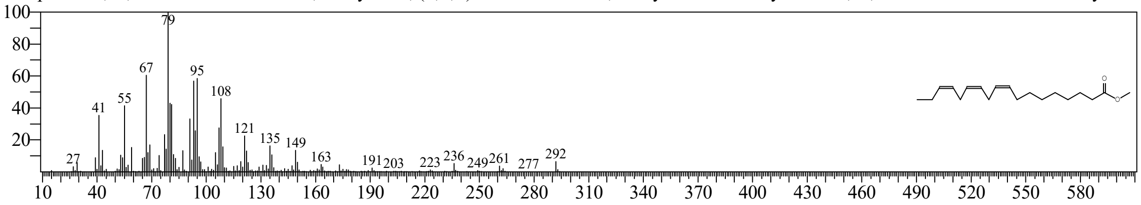

Hit#:4 Entry:27872 Library:NIST14s.lib
SI:95 Formula:C19H32O2 CAS:301-00-8 MolWeight:292 RetIndex:2101
CompName:9,12,15-Octadecatrienoic acid, methyl ester, (Z,Z,Z)- $$ Linolenic acid, methyl ester $$ Methyl all-cis-9,12,15-octadecatrienoate $$ Methyl lino

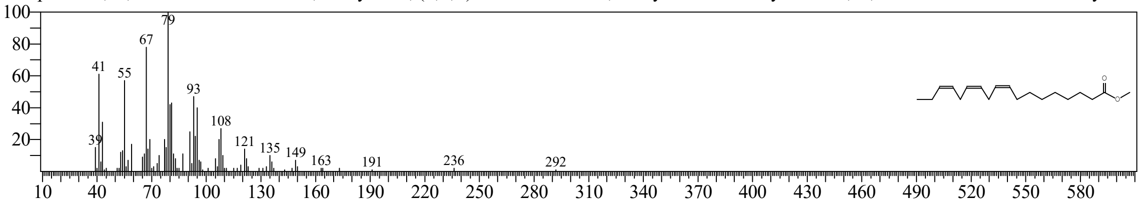

Hit#:5 Entry:32437 Library:NIST17s.lib
SI:95 Formula:C19H32O2 CAS:301-00-8 MolWeight:292 RetIndex:2101
CompName:9,12,15-Octadecatrienoic acid, methyl ester, (Z,Z,Z)- $$ Linolenic acid, methyl ester $$ Methyl all-cis-9,12,15-octadecatrienoate $$ Methyl lino

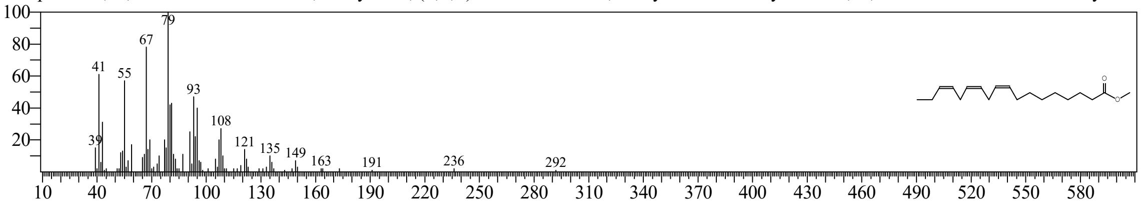

<< Target >>
Line#:15 R.Time:30.283(Scan#:2435) MassPeaks:325
RawMode:Averaged 30.275-30.292(2434-2436) BasePeak:79.05(6104)
BG Mode:Calc. from Peak Group 1 - Event 1

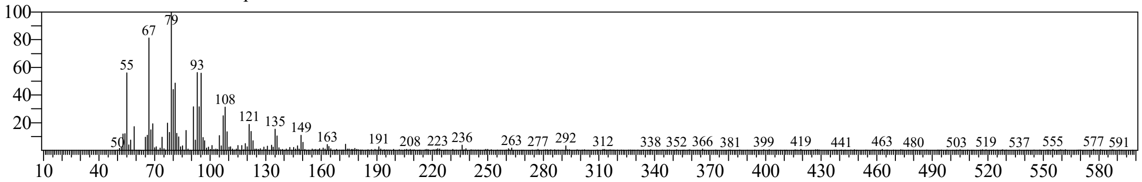

Hit#:1 Entry:135724 Library:NIST17-1.lib
SI:96 Formula:C19H32O2 CAS:301-00-8 MolWeight:292 RetIndex:2101
CompName:9,12,15-Octadecatrienoic acid, methyl ester, (Z,Z,Z)- $$ Linolenic acid, methyl ester $$ Methyl all-cis-9,12,15-octadecatrienoate $$ Methyl lino

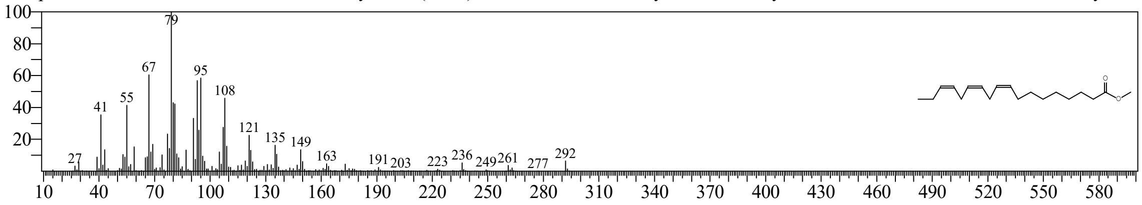

Hit#:2 Entry:124228 Library:NIST14.lib
SI:96 Formula:C19H32O2 CAS:301-00-8 MolWeight:292 RetIndex:2101
CompName:9,12,15-Octadecatrienoic acid, methyl ester, (Z,Z,Z)- $$ Linolenic acid, methyl ester $$ Methyl all-cis-9,12,15-octadecatrienoate $$ Methyl lino

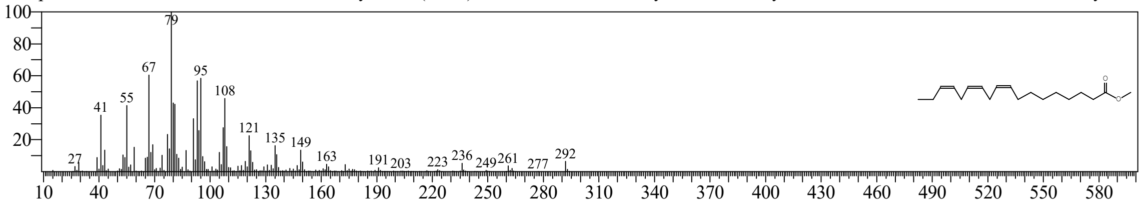

Hit#:3 Entry:32437 Library:NIST17s.lib
SI:95 Formula:C19H32O2 CAS:301-00-8 MolWeight:292 RetIndex:2101
CompName:9,12,15-Octadecatrienoic acid, methyl ester, (Z,Z,Z)- $$ Linolenic acid, methyl ester $$ Methyl all-cis-9,12,15-octadecatrienoate $$ Methyl lino

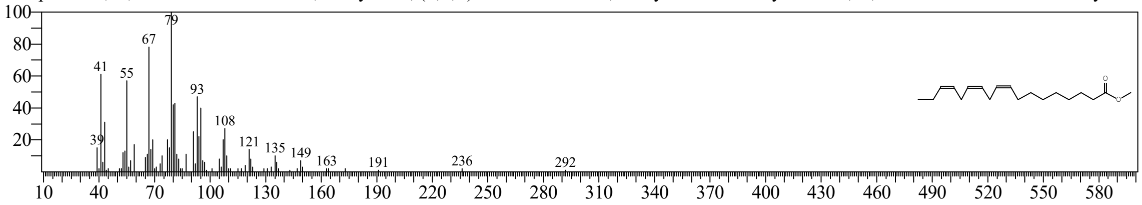

Hit#:4 Entry:27872 Library:NIST14s.lib
SI:95 Formula:C19H32O2 CAS:301-00-8 MolWeight:292 RetIndex:2101
CompName:9,12,15-Octadecatrienoic acid, methyl ester, (Z,Z,Z)- $$ Linolenic acid, methyl ester $$ Methyl all-cis-9,12,15-octadecatrienoate $$ Methyl lino

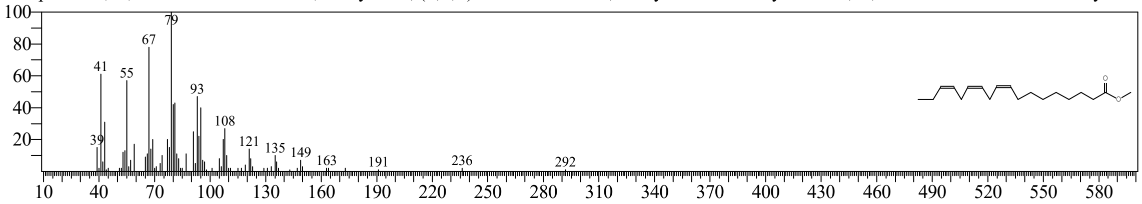

Hit#:5 Entry:121536 Library:NIST17-1.lib
SI:94 Formula:C18H30O2 CAS:0-00-0 MolWeight:278 RetIndex:2002
CompName:Methyl 8,11,14-heptadecatrienoate

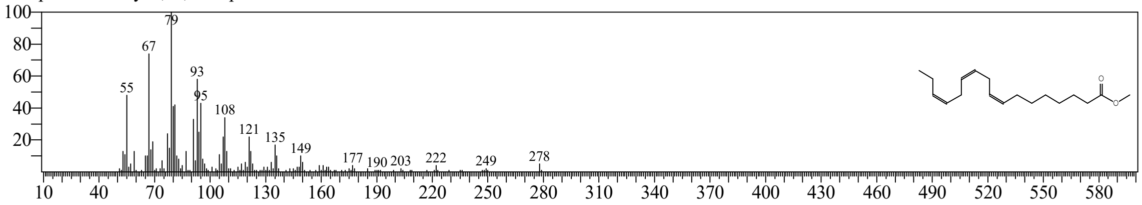

<< Target >>
Line#:16   R.Time:30.592(Scan#:2472) MassPeaks:379
RawMode:Averaged 30.583-30.600(2471-2473) BasePeak:79.05(68316)
BG Mode:Calc. from Peak Group 1 - Event 1

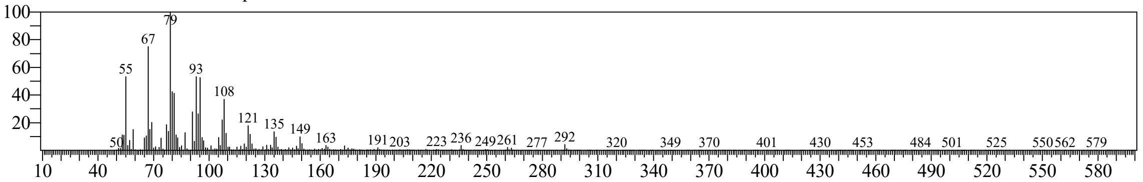

Hit#:1 Entry:135724 Library:NIST17-1.lib
SI:96 Formula:C19H32O2 CAS:301-00-8 MolWeight:292 RetIndex:2101
CompName:9,12,15-Octadecatrienoic acid, methyl ester, (Z,Z,Z)- $$ Linolenic acid, methyl ester $$ Methyl all-cis-9,12,15-octadecatrienoate $$ Methyl lino

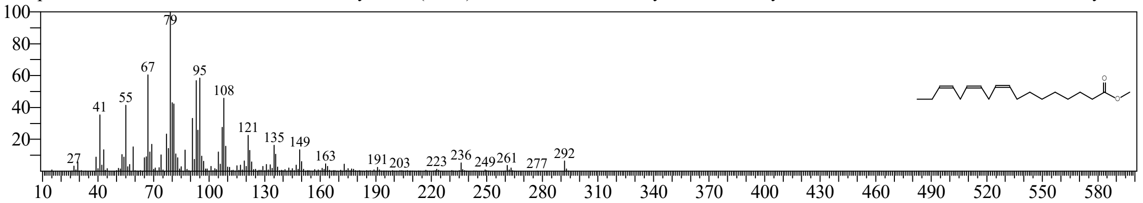

Hit#:2 Entry:124228 Library:NIST14.lib
SI:96 Formula:C19H32O2 CAS:301-00-8 MolWeight:292 RetIndex:2101
CompName:9,12,15-Octadecatrienoic acid, methyl ester, (Z,Z,Z)- $$ Linolenic acid, methyl ester $$ Methyl all-cis-9,12,15-octadecatrienoate $$ Methyl lino

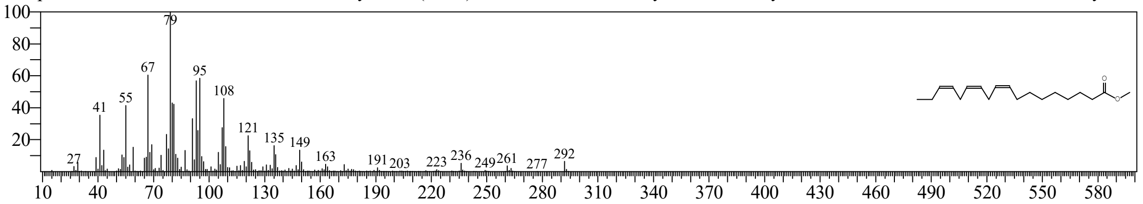

Hit#:3 Entry:32438 Library:NIST17s.lib
SI:95 Formula:C19H32O2 CAS:301-00-8 MolWeight:292 RetIndex:2101
CompName:9,12,15-Octadecatrienoic acid, methyl ester, (Z,Z,Z)- $$ Linolenic acid, methyl ester $$ Methyl all-cis-9,12,15-octadecatrienoate $$ Methyl lino

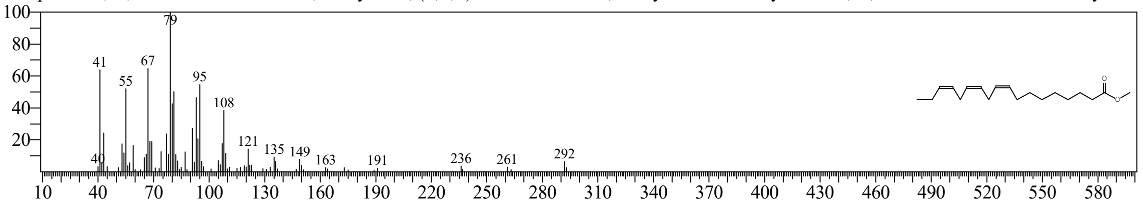

Hit#:4 Entry:27872 Library:NIST14s.lib
SI:95 Formula:C19H32O2 CAS:301-00-8 MolWeight:292 RetIndex:2101
CompName:9,12,15-Octadecatrienoic acid, methyl ester, (Z,Z,Z)- $$ Linolenic acid, methyl ester $$ Methyl all-cis-9,12,15-octadecatrienoate $$ Methyl lino

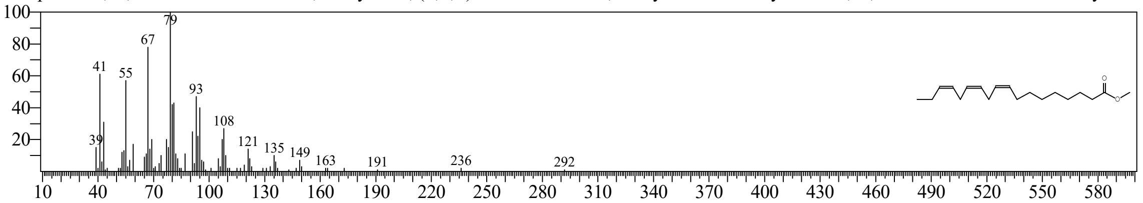

Hit#:5 Entry:32437 Library:NIST17s.lib
SI:95 Formula:C19H32O2 CAS:301-00-8 MolWeight:292 RetIndex:2101
CompName:9,12,15-Octadecatrienoic acid, methyl ester, (Z,Z,Z)- $$ Linolenic acid, methyl ester $$ Methyl all-cis-9,12,15-octadecatrienoate $$ Methyl lino

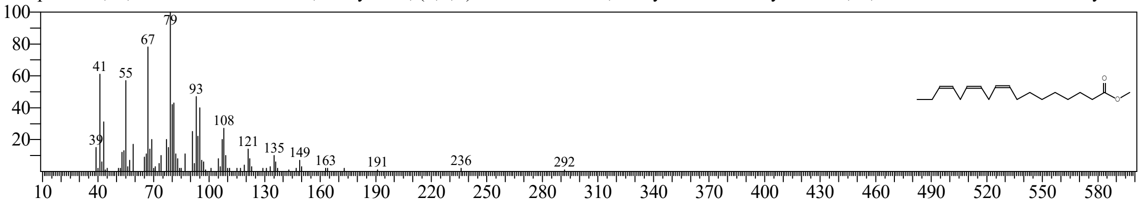

<< Target >>
Line#:17 R.Time:30.925(Scan#:2512) MassPeaks:371
RawMode:Averaged 30.917-30.933(2511-2513) BasePeak:67.05(14000)
BG Mode:Calc. from Peak Group 1 - Event 1

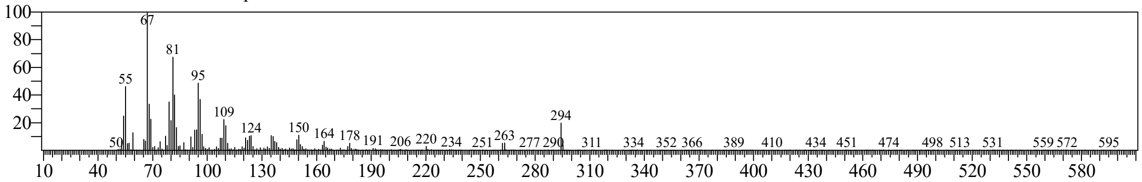

Hit#:1 Entry:137666 Library:NIST17-1.lib
SI:98 Formula:C19H34O2 CAS:13058-52-1 MolWeight:294 RetIndex:2093
CompName:Methyl 9-cis,11-trans-octadecadienoate

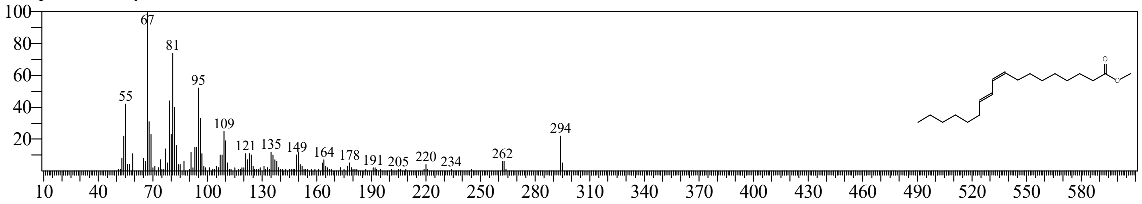

Hit#:2 Entry:125932 Library:NIST14.lib
SI:98 Formula:C19H34O2 CAS:0-00-0 MolWeight:294 RetIndex:2093
CompName:Methyl 9-cis,11-trans-octadecadienoate

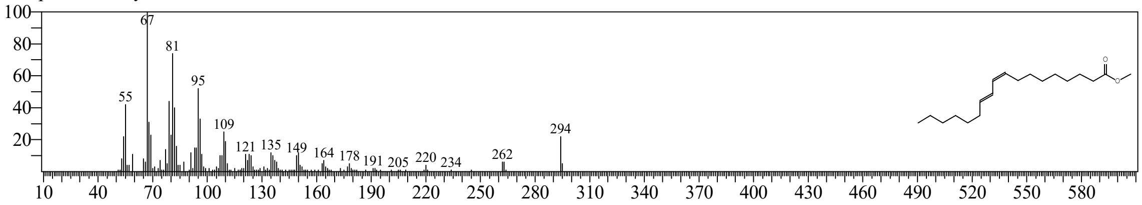

Hit#:3 Entry:125933 Library:NIST14.lib
SI:97 Formula:C19H34O2 CAS:0-00-0 MolWeight:294 RetIndex:2093
CompName:Methyl 10-trans,12-cis-octadecadienoate

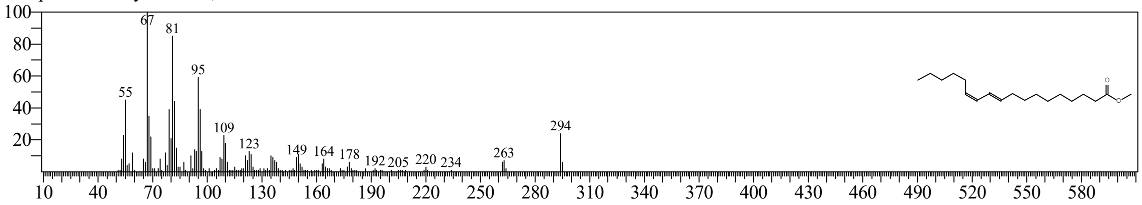

Hit#:4 Entry:137664 Library:NIST17-1.lib
SI:97 Formula:C19H34O2 CAS:0-00-0 MolWeight:294 RetIndex:2093
CompName:Methyl 10-trans,12-cis-octadecadienoate

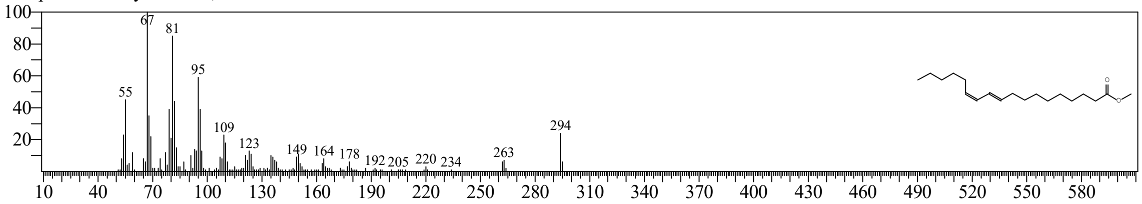

Hit#:5 Entry:32587 Library:NIST17s.lib
SI:95 Formula:C19H34O2 CAS:13038-47-6 MolWeight:294 RetIndex:2093
CompName:9,11-Octadecadienoic acid, methyl ester, (E,E)- $$ Methyl trans-9,trans-11-octadecadienoate $$ Methyl trans,trans-9,11-octadecadienoate $$ M

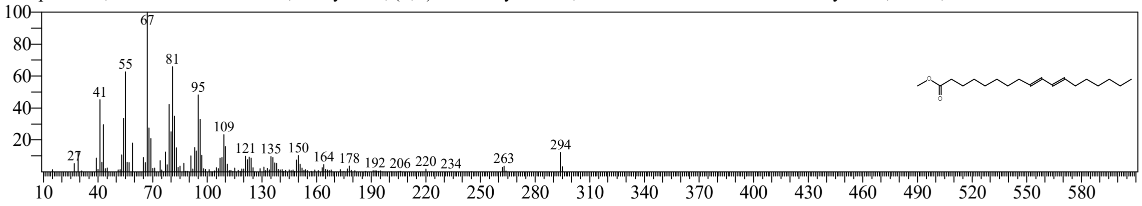

<< Target >>
Line#:18 R.Time:31.250(Scan#:2551) MassPeaks:355
RawMode:Averaged 31.242-31.258(2550-2552) BasePeak:67.05(15053)
BG Mode:Calc. from Peak Group 1 - Event 1

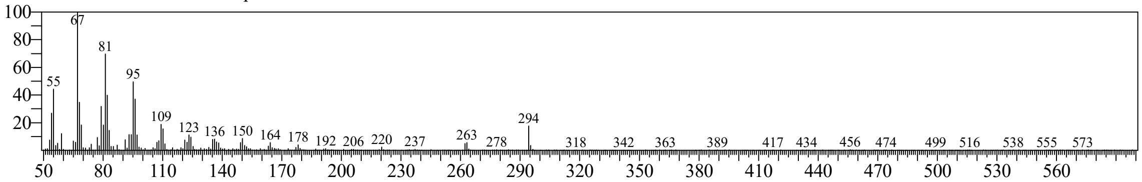

Hit#:1 Entry:125933 Library:NIST14.lib
SI:96 Formula:C19H34O2 CAS:0-00-0 MolWeight:294 RetIndex:2093
CompName:Methyl 10-trans,12-cis-octadecadienoate

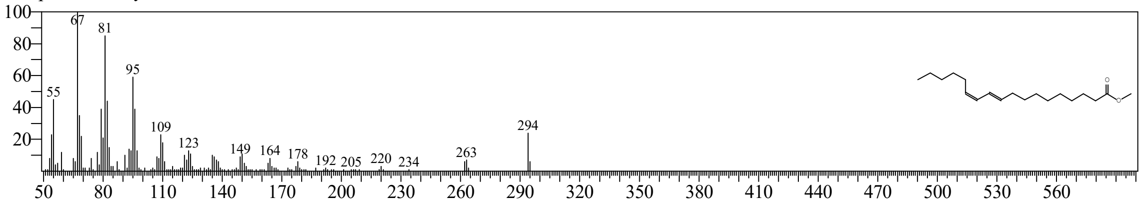

Hit#:2 Entry:137664 Library:NIST17-1.lib
SI:96 Formula:C19H34O2 CAS:0-00-0 MolWeight:294 RetIndex:2093
CompName:Methyl 10-trans,12-cis-octadecadienoate

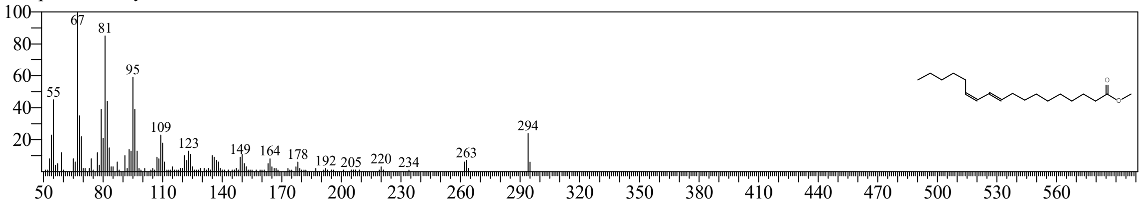

Hit#:3 Entry:125932 Library:NIST14.lib
SI:96 Formula:C19H34O2 CAS:0-00-0 MolWeight:294 RetIndex:2093
CompName:Methyl 9-cis,11-trans-octadecadienoate

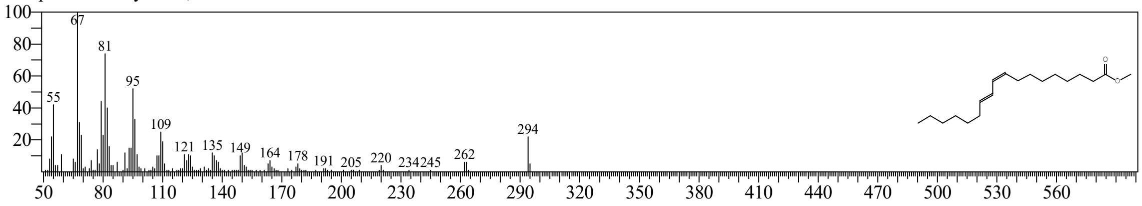

Hit#:4 Entry:137666 Library:NIST17-1.lib
SI:96 Formula:C19H34O2 CAS:13058-52-1 MolWeight:294 RetIndex:2093
CompName:Methyl 9-cis,11-trans-octadecadienoate

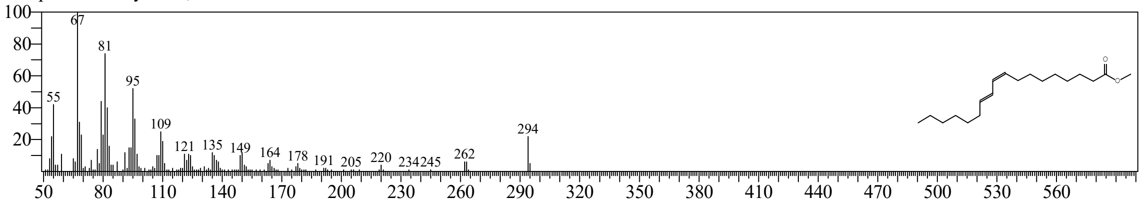

Hit#:5 Entry:32586 Library:NIST17s.lib
SI:95 Formula:C19H34O2 CAS:56554-61-1 MolWeight:294 RetIndex:2093
CompName:11,14-Octadecadienoic acid, methyl ester $$ Methyl (11E,14E)-11,14-octadecadienoate # $$

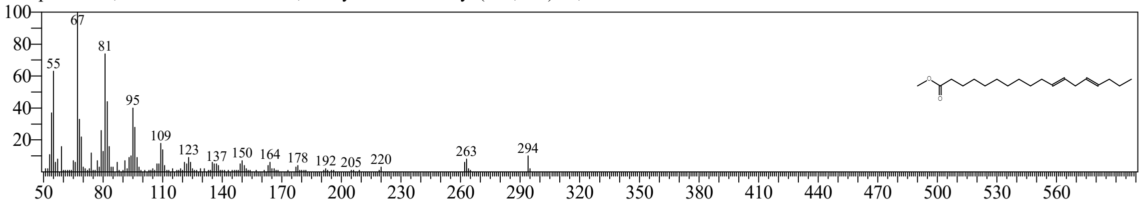

<< Target >>
Line#:19 R.Time:31.917(Scan#:2631) MassPeaks:356
RawMode:Averaged 31.908-31.925(2630-2632) BasePeak:67.00(1156)
BG Mode:Calc. from Peak Group 1 - Event 1

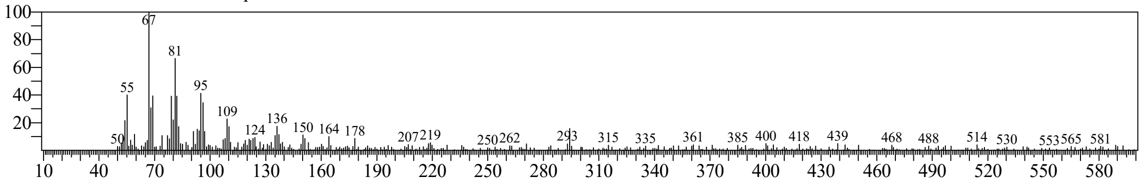

Hit#:1 Entry:32587 Library:NIST17s.lib
SI:91 Formula:C19H34O2 CAS:13038-47-6 MolWeight:294 RetIndex:2093
CompName:9,11-Octadecadienoic acid, methyl ester, (E,E)- $$ Methyl trans-9,trans-11-octadecadienoate $$ Methyl trans,trans-9,11-octadecadienoate $$ M

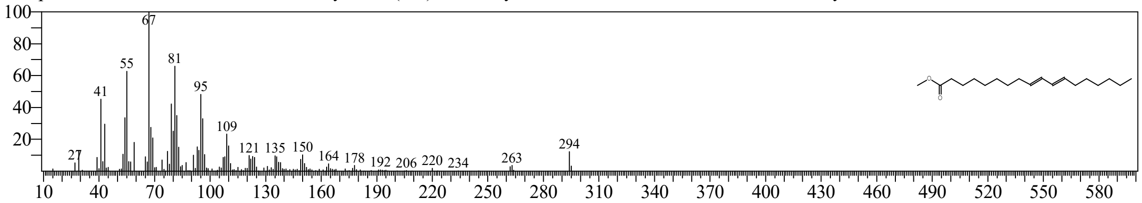

Hit#:2 Entry:137666 Library:NIST17-1.lib
SI:91 Formula:C19H34O2 CAS:13058-52-1 MolWeight:294 RetIndex:2093
CompName:Methyl 9-cis,11-trans-octadecadienoate

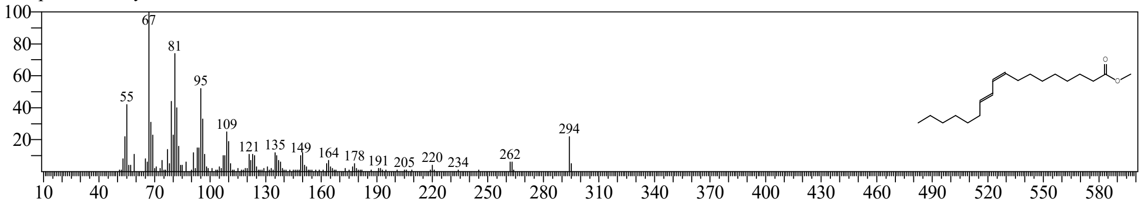

Hit#:3 Entry:125932 Library:NIST14.lib
SI:91 Formula:C19H34O2 CAS:0-00-0 MolWeight:294 RetIndex:2093
CompName:Methyl 9-cis,11-trans-octadecadienoate

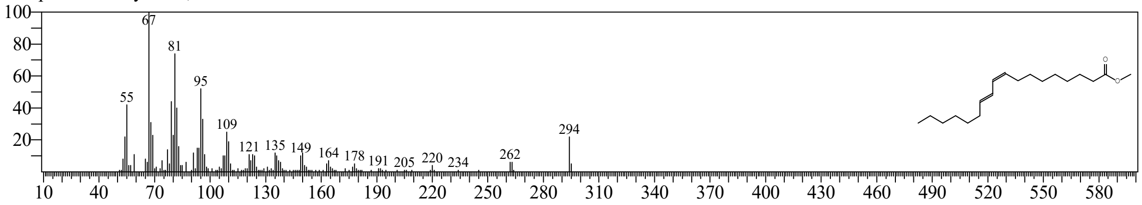

Hit#:4 Entry:125933 Library:NIST14.lib
SI:91 Formula:C19H34O2 CAS:0-00-0 MolWeight:294 RetIndex:2093
CompName:Methyl 10-trans,12-cis-octadecadienoate

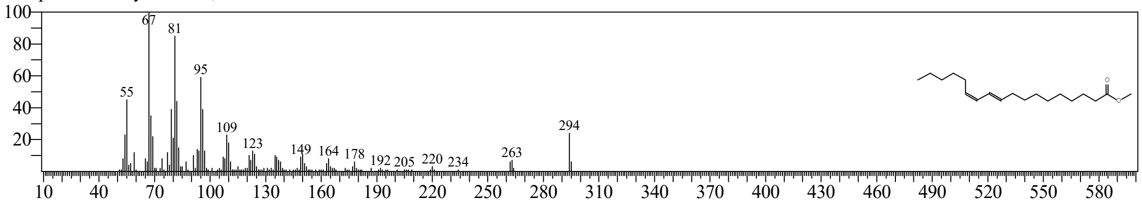

Hit#:5 Entry:137664 Library:NIST17-1.lib
SI:91 Formula:C19H34O2 CAS:0-00-0 MolWeight:294 RetIndex:2093
CompName:Methyl 10-trans,12-cis-octadecadienoate

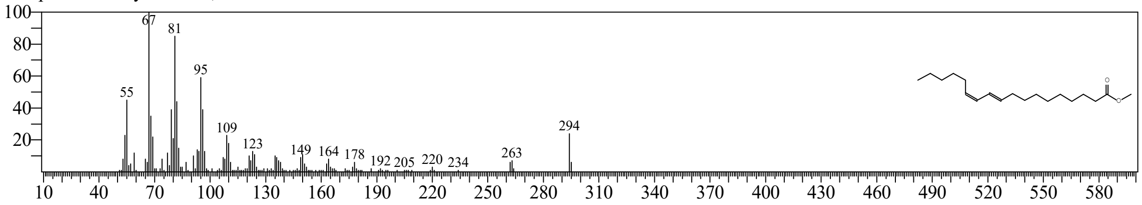

<< Target >>
Line#:20 R.Time:32.950(Scan#:2755) MassPeaks:352
RawMode:Averaged 32.942-32.958(2754-2756) BasePeak:95.10(16790)
BG Mode:Calc. from Peak Group 1 - Event 1

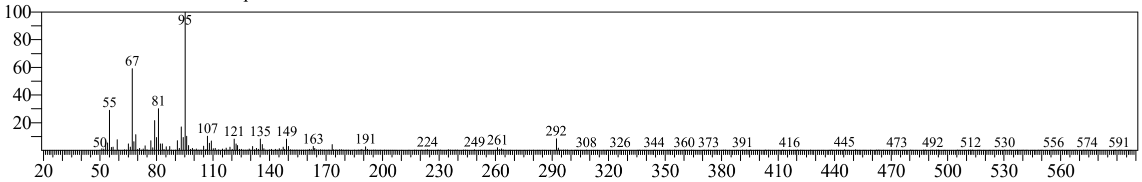

Hit#:1 Entry:24424 Library:NIST17-1.lib
SI:85 Formula:C12H20 CAS:0-00-0 MolWeight:164 RetIndex:1041
CompName:Cis-3-ethyl-endo-tricyclo[5.2.1.0(2.6)]decane

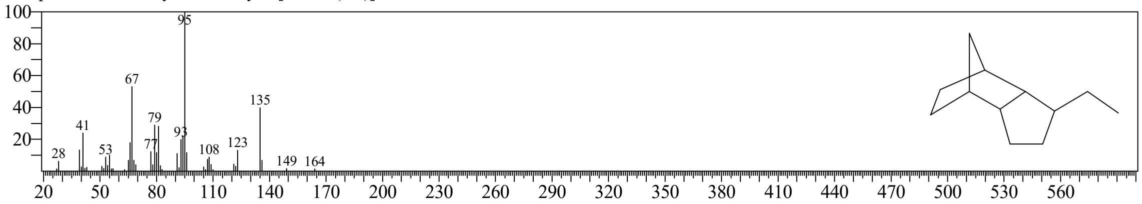

Hit#:2 Entry:23403 Library:NIST14.lib
SI:85 Formula:C12H20 CAS:0-00-0 MolWeight:164 RetIndex:1041
CompName:Cis-3-ethyl-endo-tricyclo[5.2.1.0(2.6)]decane

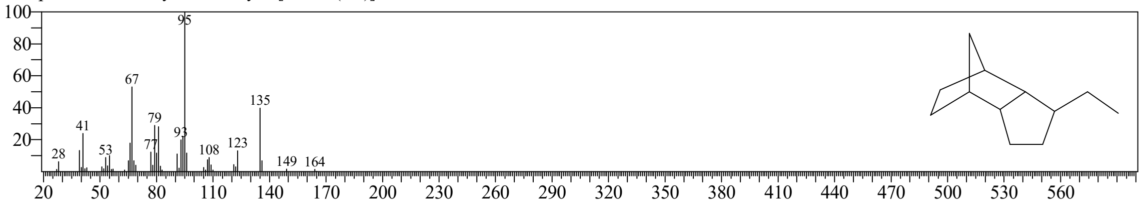

Hit#:3 Entry:16582 Library:NIST17-1.lib
SI:83 Formula:C11H18 CAS:0-00-0 MolWeight:150 RetIndex:1115
CompName:4-Cyclohexenyl-3-methylbutene

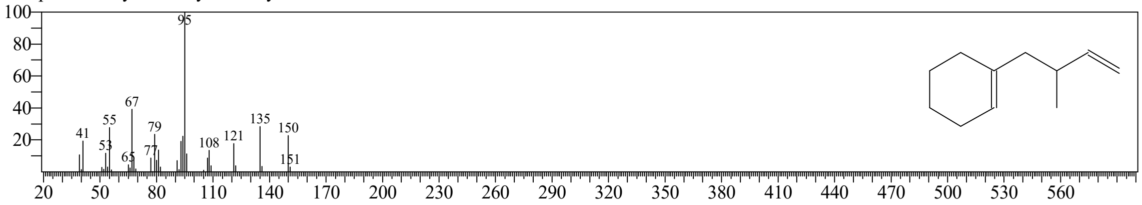

Hit#:4 Entry:16897 Library:NIST14.lib
SI:83 Formula:C10H16O CAS:60485-45-2 MolWeight:152 RetIndex:991
CompName:Santolina epoxide $$ 2-Methyl-2-(3-methyl-1-vinyl-2-butenyl)oxirane # $$

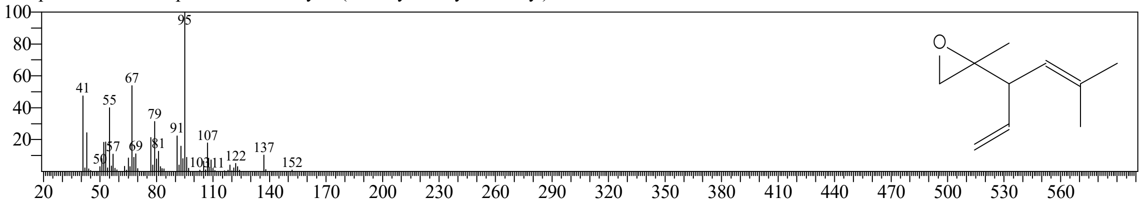

Hit#:5 Entry:17571 Library:NIST17-1.lib
SI:83 Formula:C10H16O CAS:60485-45-2 MolWeight:152 RetIndex:991
CompName:Santolina epoxide $$ 2-Methyl-2-(3-methyl-1-vinyl-2-butenyl)oxirane # $$

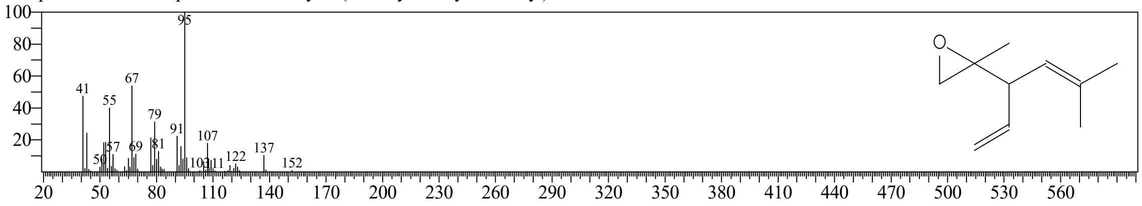

<< Target >>
Line#:21   R.Time:33.483(Scan#:2819) MassPeaks:314
RawMode:Averaged 33.475-33.492(2818-2820) BasePeak:74.00(16087)
BG Mode:Calc. from Peak Group 1 - Event 1

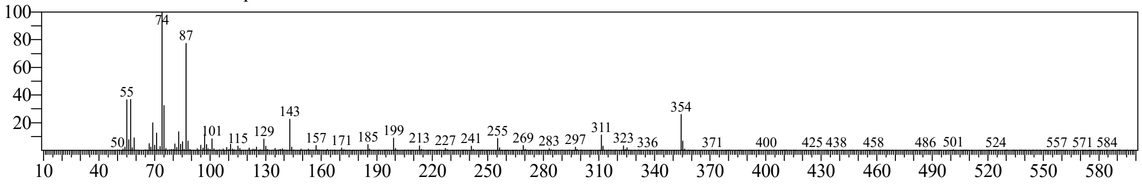

Hit#:1 Entry:196443 Library:NIST17-1.lib
SI:96 Formula:C23H46O2 CAS:929-77-1 MolWeight:354 RetIndex:2475
CompName:Docosanoic acid, methyl ester $$ Behenic acid, methyl ester $$ Methyl behenate $$ Methyl docosanoate $$ n-Docosanoic acid methyl ester $$

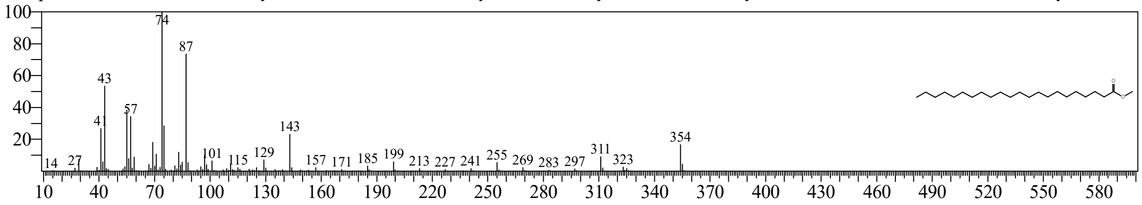

Hit#:2 Entry:178051 Library:NIST14.lib
SI:96 Formula:C23H46O2 CAS:929-77-1 MolWeight:354 RetIndex:2475
CompName:Docosanoic acid, methyl ester $$ Behenic acid, methyl ester $$ Methyl behenate $$ Methyl docosanoate $$ n-Docosanoic acid methyl ester $$

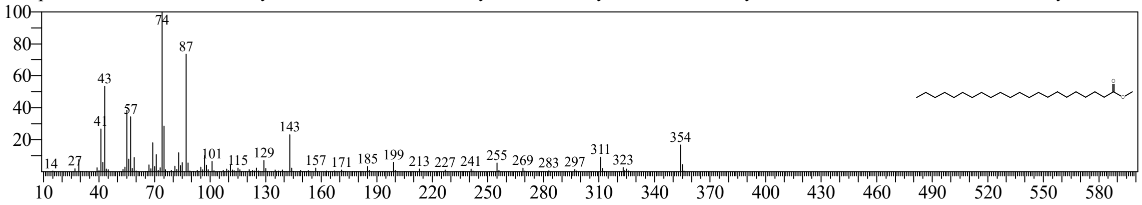

Hit#:3 Entry:196444 Library:NIST17-1.lib
SI:95 Formula:C23H46O2 CAS:0-00-0 MolWeight:354 RetIndex:2411
CompName:Methyl 20-methyl-heneicosanoate

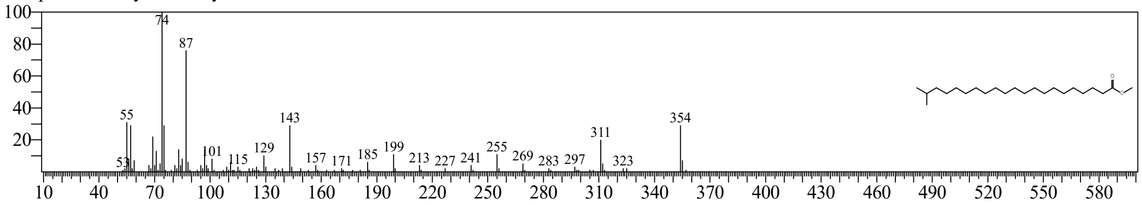

Hit#:4 Entry:178052 Library:NIST14.lib
SI:95 Formula:C23H46O2 CAS:0-00-0 MolWeight:354 RetIndex:2411
CompName:Methyl 20-methyl-heneicosanoate

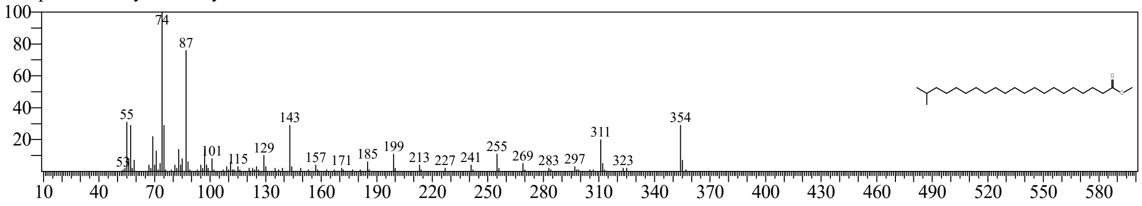

Hit#:5 Entry:31088 Library:NIST14s.lib
SI:95 Formula:C23H46O2 CAS:929-77-1 MolWeight:354 RetIndex:2475
CompName:Docosanoic acid, methyl ester $$ Behenic acid, methyl ester $$ Methyl behenate $$ Methyl docosanoate $$ n-Docosanoic acid methyl ester $$

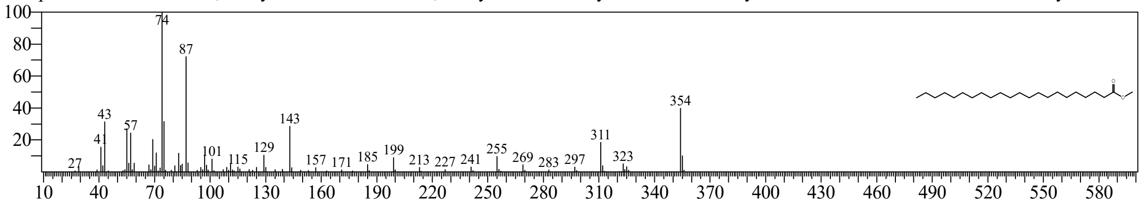

<< Target >>
Line#:22 R.Time:37.783(Scan#:3335) MassPeaks:347
RawMode:Averaged 37.775-37.792(3334-3336) BasePeak:74.00(4042)
BG Mode:Calc. from Peak Group 1 - Event 1

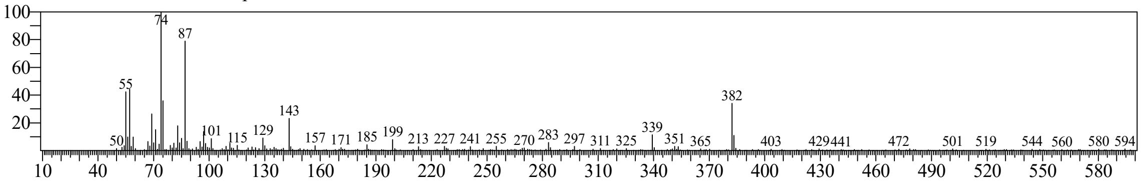

Hit#:1 Entry:31970 Library:NIST14s.lib
SI:94 Formula:C25H50O2 CAS:2442-49-1 MolWeight:382 RetIndex:2674
CompName:Tetracosanoic acid, methyl ester $$ Methyl lignocerate $$ Methyl tetracosanoate $$ Lignoceric acid methyl ester $$

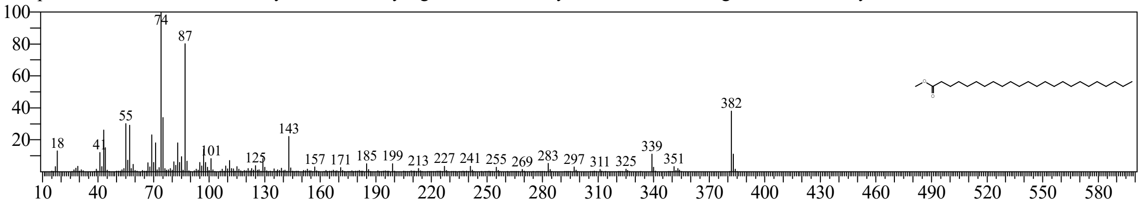

Hit#:2 Entry:37106 Library:NIST17s.lib
SI:94 Formula:C25H50O2 CAS:2442-49-1 MolWeight:382 RetIndex:2674
CompName:Tetracosanoic acid, methyl ester $$ Methyl lignocerate $$ Methyl tetracosanoate $$ Lignoceric acid methyl ester $$

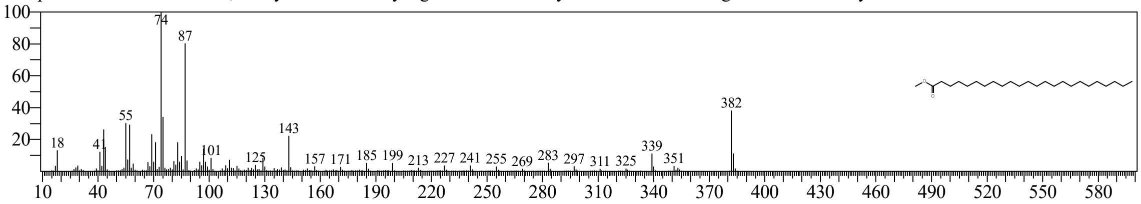

Hit#:3 Entry:31971 Library:NIST14s.lib
SI:93 Formula:C25H50O2 CAS:2442-49-1 MolWeight:382 RetIndex:2674
CompName:Tetracosanoic acid, methyl ester $$ Methyl lignocerate $$ Methyl tetracosanoate $$ Lignoceric acid methyl ester $$

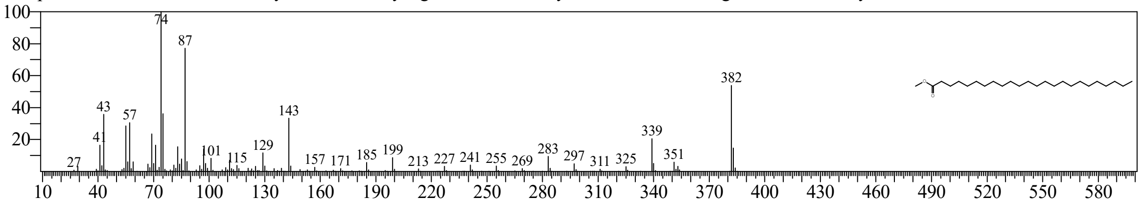

Hit#:4 Entry:37107 Library:NIST17s.lib
SI:93 Formula:C25H50O2 CAS:2442-49-1 MolWeight:382 RetIndex:2674
CompName:Tetracosanoic acid, methyl ester $$ Methyl lignocerate $$ Methyl tetracosanoate $$ Lignoceric acid methyl ester $$

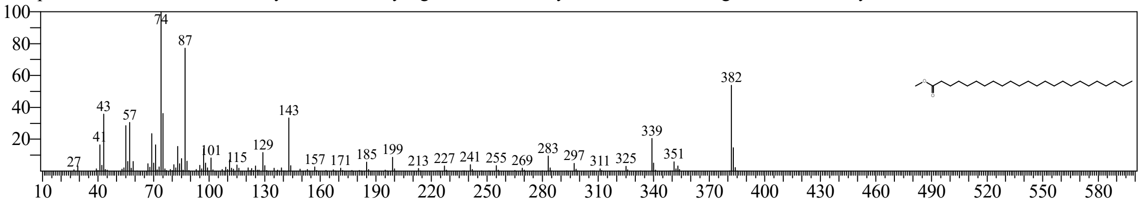

Hit#:5 Entry:37105 Library:NIST17s.lib
SI:89 Formula:C25H50O2 CAS:2442-49-1 MolWeight:382 RetIndex:2674
CompName:Tetracosanoic acid, methyl ester $$ Methyl lignocerate $$ Methyl tetracosanoate $$ Lignoceric acid methyl ester $$

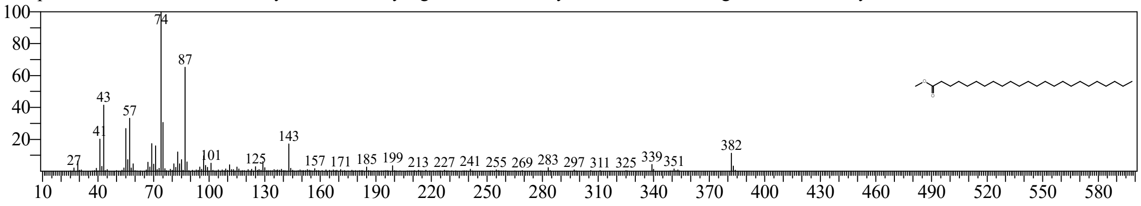

Supplement: Supplementary file 1 [file molecules-31-02891-s001.zip › 0d-PG-1.pdf]
