# Supplementary material for: Selective Inhibition of Lipid Oxidation by a Quercetagetin-Rich Extract from Tagetes erecta: A Chemometric Approach Using OPLS-DA and Selectivity Index Analysis
Source: Molecules. 2026 Aug 19;31(16):2891. doi: 10.3390/molecules31162891 (PMC13515886; doi:10.3390/molecules31162891)

# QG100-1-0d

<<Column>>
Name : HP-88
Thickness : 0.20um
Length : 100.0m
Inside Diameter : 0.25mm

## Method

===== Analytical Line 1 =====

| | | |
|---|---|---|
| Column Oven Temp. | :120.0 °C | |
| Injection Temp. | :250.00 °C | |
| Injection Mode | :Split | |
| Column Flow | :1.04 mL/min | |
| Split Ratio | :20.0 | |
| Oven Temp. Program | | |
| Rate | Temperature(°C) | Hold Time(min) |
| - | 120.0 | 1.00 |
| 10.00 | 175.0 | 10.00 |
| 5.00 | 210.0 | 5.00 |
| 5.00 | 230.0 | 15.00 |

[GCMS-QP2010]
IonSourceTemp :200.00 °C
Interface Temp. :220.00 °C
*m/z* scanning range：45～500m/z

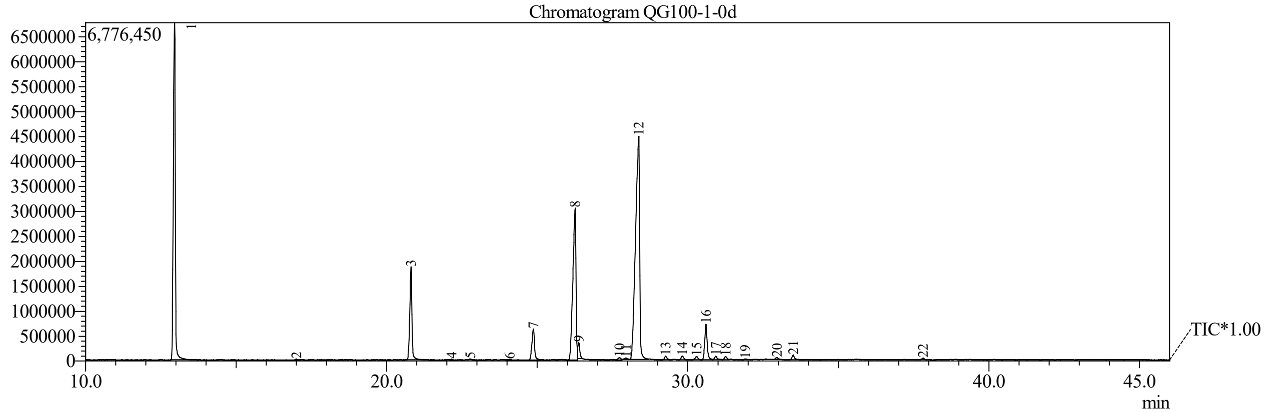


Peak Report TIC

| Peak# | R.Time | Area | Area% | Height | Height% | A/H | Name |
|---|---|---|---|---|---|---|---|
| 1 | 12.958 | 30439174 | 26.13 | 6749246 | 36.62 | 4.51 | Undecanoic acid, methyl ester |
| 2 | 17.003 | 90810 | 0.08 | 18496 | 0.10 | 4.91 | Methyl tetradecanoate |
| 3 | 20.812 | 9406717 | 8.08 | 1863947 | 10.11 | 5.05 | Hexadecanoic acid, methyl ester |
| 4 | 22.161 | 72728 | 0.06 | 15536 | 0.08 | 4.68 | 9-Hexadecenoic acid, methyl ester, (Z)- |
| 5 | 22.776 | 76314 | 0.07 | 15416 | 0.08 | 4.95 | Heptadecanoic acid, methyl ester |
| 6 | 24.085 | 65100 | 0.06 | 11865 | 0.06 | 5.49 | Cyclopropaneoctanoic acid, 2-hexyl-, methyl e |
| 7 | 24.874 | 3876554 | 3.33 | 619993 | 3.36 | 6.25 | Methyl stearate |
| 8 | 26.257 | 21608128 | 18.55 | 3044901 | 16.52 | 7.10 | 9-Octadecenoic acid, methyl ester, (E)- |
| 9 | 26.380 | 1477484 | 1.27 | 318796 | 1.73 | 4.63 | 11-Octadecenoic acid, methyl ester |
| 10 | 27.726 | 292940 | 0.25 | 45624 | 0.25 | 6.42 | 9,12-Octadecadienoic acid (Z,Z)-, methyl ester |
| 11 | 27.943 | 193144 | 0.17 | 27569 | 0.15 | 7.01 | 9,11-Octadecadienoic acid, methyl ester, (E,E) |
| 12 | 28.371 | 41978133 | 36.04 | 4472315 | 24.26 | 9.39 | 9,12-Octadecadienoic acid (Z,Z)-, methyl ester |
| 13 | 29.266 | 417914 | 0.36 | 73812 | 0.40 | 5.66 | Methyl 18-methylnonadecanoate |
| 14 | 29.817 | 389876 | 0.33 | 69386 | 0.38 | 5.62 | 11,14,17-Eicosatrienoic acid, methyl ester |

| Peak# | R.Time | Area | Area% | Height | Height% | A/H | Name |
|---|---|---|---|---|---|---|---|
| 15 | 30.294 | 402355 | 0.35 | 65261 | 0.35 | 6.17 | 9,12,15-Octadecatrienoic acid, methyl ester, (Z |
| 16 | 30.602 | 4044314 | 3.47 | 711983 | 3.86 | 5.68 | 9,12,15-Octadecatrienoic acid, methyl ester, (Z |
| 17 | 30.933 | 376494 | 0.32 | 68336 | 0.37 | 5.51 | Methyl 9-cis,11-trans-octadecadienoate |
| 18 | 31.258 | 331677 | 0.28 | 63775 | 0.35 | 5.20 | Methyl 10-trans,12-cis-octadecadienoate |
| 19 | 31.911 | 74085 | 0.06 | 14397 | 0.08 | 5.15 | Methyl 9-cis,11-trans-octadecadienoate |
| 20 | 32.956 | 217118 | 0.19 | 40028 | 0.22 | 5.42 | Cis-3-ethyl-endo-tricyclo[5.2.1.0(2.6)]decane |
| 21 | 33.499 | 501371 | 0.43 | 93612 | 0.51 | 5.36 | Docosanoic acid, methyl ester |
| 22 | 37.813 | 151588 | 0.13 | 28594 | 0.16 | 5.30 | Tetracosanoic acid, methyl ester |
| | | 116484018 | 100.00 | 18432888 | 100.00 | | |

Library

<< Target >>
Line#:1 R.Time:12.958(Scan#:356) MassPeaks:343
RawMode:Averaged 12.950-12.967(355-357) BasePeak:74.05(2097667)
BG Mode:Calc. from Peak Group 1 - Event 1

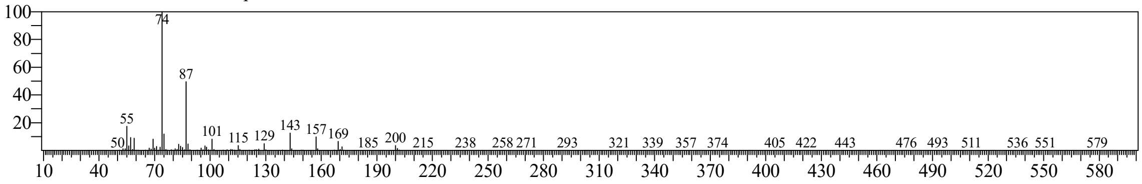


Hit#:1 Entry:21619 Library:NIST17s.lib
SI:95 Formula:C12H24O2 CAS:1731-86-8 MolWeight:200 RetIndex:1381
CompName:Undecanoic acid, methyl ester $$ Methyl undecanoate $$ Methyl undecylenate $$ n-Undecanoic acid methyl ester $$ Methyl ester of undecanoi

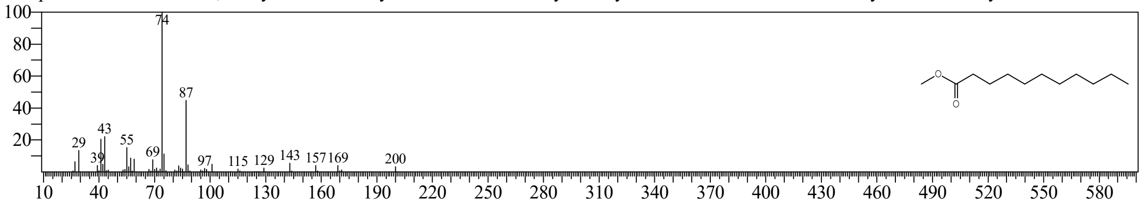


Hit#:2 Entry:18487 Library:NIST14s.lib
SI:95 Formula:C12H24O2 CAS:1731-86-8 MolWeight:200 RetIndex:1381
CompName:Undecanoic acid, methyl ester $$ Methyl undecanoate $$ Methyl undecylenate $$ n-Undecanoic acid methyl ester $$ Methyl ester of undecanoi

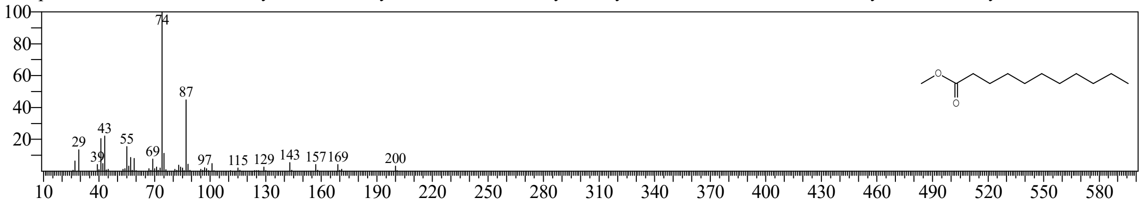


Hit#:3 Entry:18488 Library:NIST14s.lib
SI:95 Formula:C12H24O2 CAS:1731-86-8 MolWeight:200 RetIndex:1381
CompName:Undecanoic acid, methyl ester $$ Methyl undecanoate $$ Methyl undecylenate $$ n-Undecanoic acid methyl ester $$ Methyl ester of undecanoi

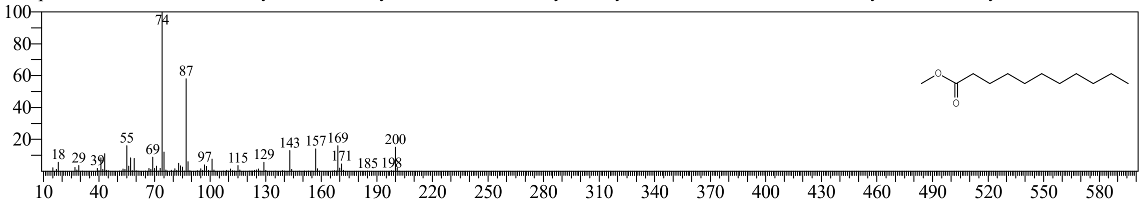


Hit#:4 Entry:21620 Library:NIST17s.lib
SI:95 Formula:C12H24O2 CAS:1731-86-8 MolWeight:200 RetIndex:1381
CompName:Undecanoic acid, methyl ester $$ Methyl undecanoate $$ Methyl undecylenate $$ n-Undecanoic acid methyl ester $$ Methyl ester of undecanoi

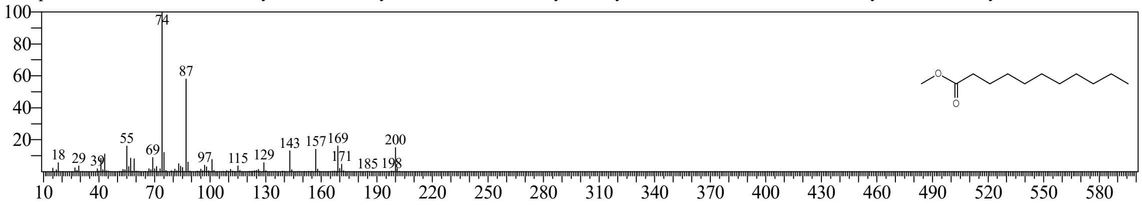

<< Target >>
Line#:1   R.Time:12.958(Scan#:356)  MassPeaks:343
RawMode:Averaged 12.950-12.967(355-357) BasePeak:74.05(2097667)
BG Mode:Calc. from Peak  Group 1 - Event 1

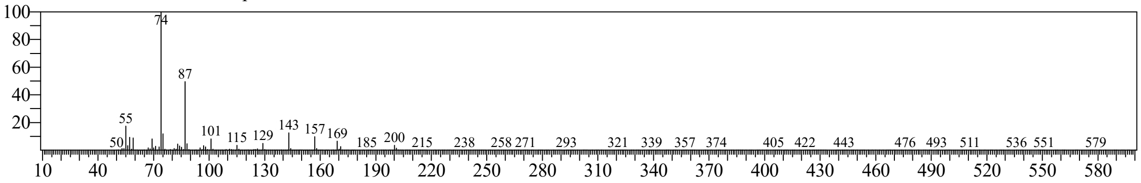


Hit#:5  Entry:21618  Library:NIST17s.lib
SI:95  Formula:C12H24O2  CAS:1731-86-8  MolWeight:200  RetIndex:1381
CompName:Undecanoic acid, methyl ester $$ Methyl undecanoate $$ Methyl undecylenate $$ n-Undecanoic acid methyl ester $$ Methyl ester of undecanoi

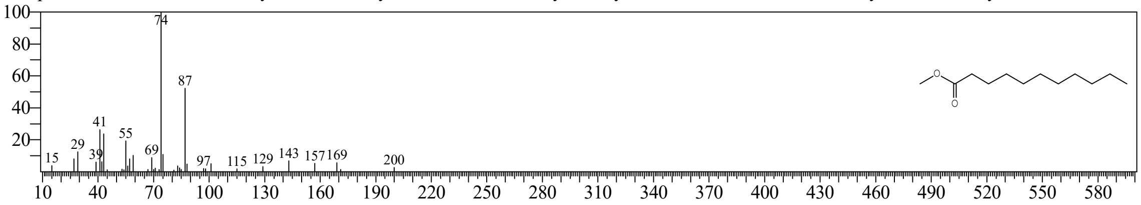

<< Target >>
Line#:2 R.Time:17.000(Scan#:841) MassPeaks:357
RawMode:Averaged 16.992-17.008(840-842) BasePeak:74.00(3444)
BG Mode:Calc. from Peak Group 1 - Event 1

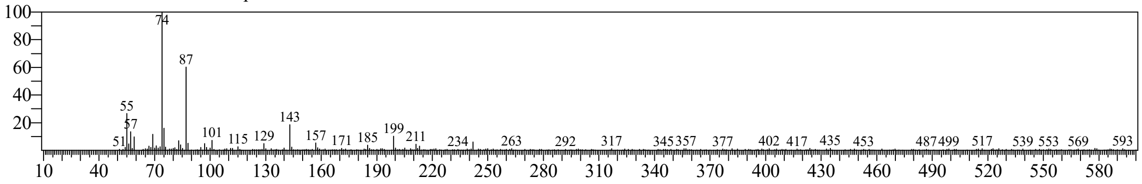


Hit#:1 Entry:80645 Library:NIST14.lib
SI:94 Formula:C15H30O2 CAS:124-10-7 MolWeight:242 RetIndex:1680
CompName:Methyl tetradecanoate $$ Tetradecanoic acid, methyl ester $$ Myristic acid, methyl ester $$ Metholeneat 2495 $$ Methyl myristate $$ Methyl r

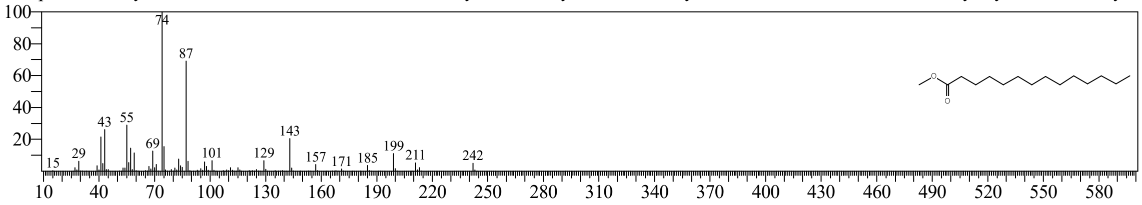


Hit#:2 Entry:86786 Library:NIST17-1.lib
SI:94 Formula:C15H30O2 CAS:124-10-7 MolWeight:242 RetIndex:1680
CompName:Methyl tetradecanoate $$ Tetradecanoic acid, methyl ester $$ Myristic acid, methyl ester $$ Metholeneat 2495 $$ Methyl myristate $$ Methyl r

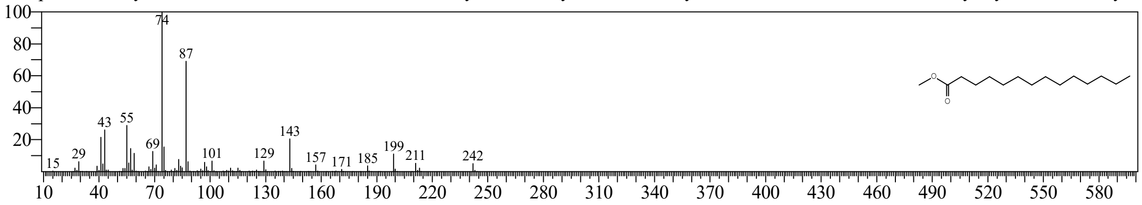


Hit#:3 Entry:27874 Library:NIST17s.lib
SI:94 Formula:C15H30O2 CAS:124-10-7 MolWeight:242 RetIndex:1680
CompName:Methyl tetradecanoate $$ Tetradecanoic acid, methyl ester $$ Myristic acid, methyl ester $$ Metholeneat 2495 $$ Methyl myristate $$ Methyl r

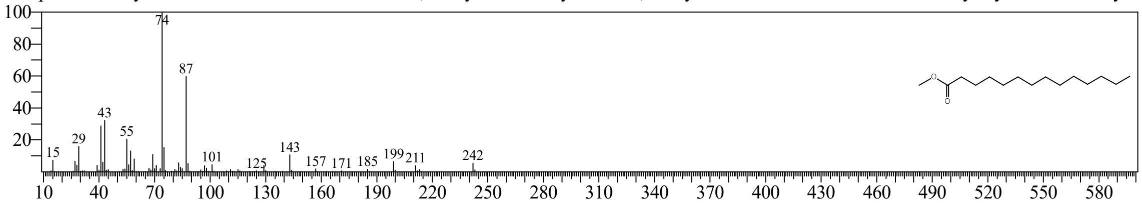


Hit#:4 Entry:23848 Library:NIST14s.lib
SI:94 Formula:C15H30O2 CAS:124-10-7 MolWeight:242 RetIndex:1680
CompName:Methyl tetradecanoate $$ Tetradecanoic acid, methyl ester $$ Myristic acid, methyl ester $$ Metholeneat 2495 $$ Methyl myristate $$ Methyl r

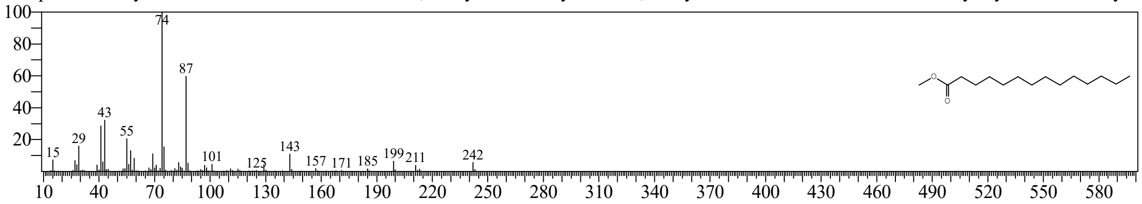


Hit#:5 Entry:27873 Library:NIST17s.lib
SI:94 Formula:C15H30O2 CAS:124-10-7 MolWeight:242 RetIndex:1680
CompName:Methyl tetradecanoate $$ Tetradecanoic acid, methyl ester $$ Myristic acid, methyl ester $$ Metholeneat 2495 $$ Methyl myristate $$ Methyl r

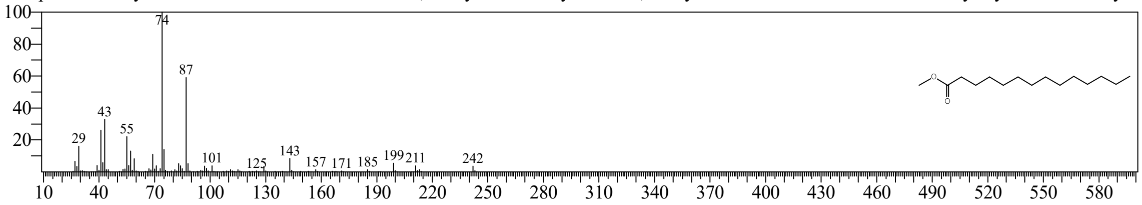

<< Target >>
Line#:3   R.Time:20.808(Scan#:1298)  MassPeaks:367
RawMode:Averaged 20.800-20.817(1297-1299) BasePeak:74.00(442745)
BG Mode:Calc. from Peak  Group 1 - Event 1

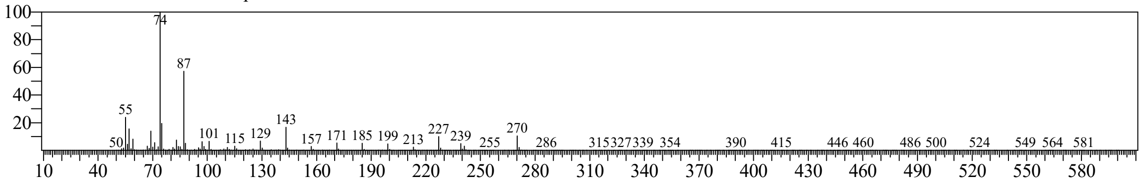


Hit#:1  Entry:26273  Library:NIST14s.lib
SI:97  Formula:C17H34O2  CAS:112-39-0  MolWeight:270  RetIndex:1878
CompName:Hexadecanoic acid, methyl ester $$ Palmitic acid, methyl ester $$ n-Hexadecanoic acid methyl ester $$ Metholene 2216 $$ Methyl hexadecano

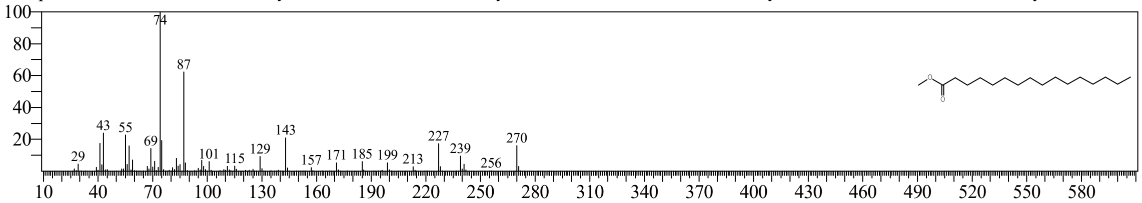


Hit#:2  Entry:113663  Library:NIST17-1.lib
SI:96  Formula:C17H34O2  CAS:112-39-0  MolWeight:270  RetIndex:1878
CompName:Hexadecanoic acid, methyl ester $$ Palmitic acid, methyl ester $$ n-Hexadecanoic acid methyl ester $$ Metholene 2216 $$ Methyl hexadecano

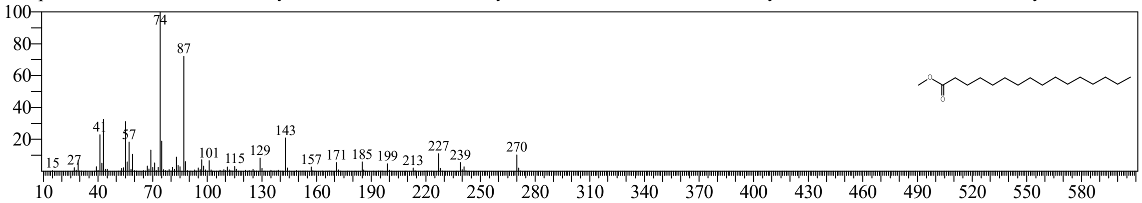


Hit#:3  Entry:104648  Library:NIST14.lib
SI:96  Formula:C17H34O2  CAS:112-39-0  MolWeight:270  RetIndex:1878
CompName:Hexadecanoic acid, methyl ester $$ Palmitic acid, methyl ester $$ n-Hexadecanoic acid methyl ester $$ Metholene 2216 $$ Methyl hexadecano

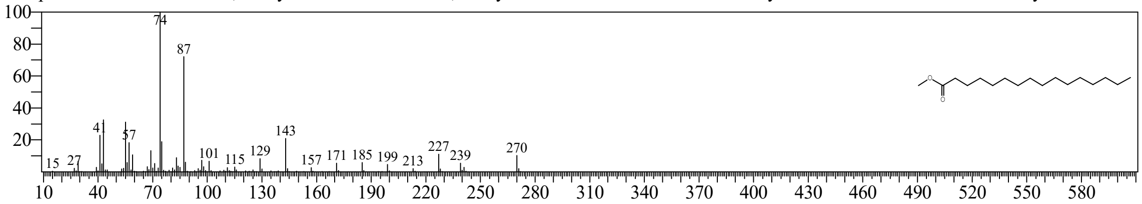


Hit#:4  Entry:30646  Library:NIST17s.lib
SI:96  Formula:C17H34O2  CAS:112-39-0  MolWeight:270  RetIndex:1878
CompName:Hexadecanoic acid, methyl ester $$ Palmitic acid, methyl ester $$ n-Hexadecanoic acid methyl ester $$ Metholene 2216 $$ Methyl hexadecano

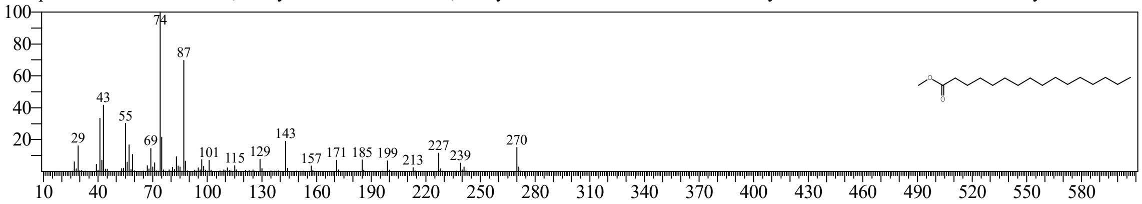


Hit#:5  Entry:26269  Library:NIST14s.lib
SI:96  Formula:C17H34O2  CAS:112-39-0  MolWeight:270  RetIndex:1878
CompName:Hexadecanoic acid, methyl ester $$ Palmitic acid, methyl ester $$ n-Hexadecanoic acid methyl ester $$ Metholene 2216 $$ Methyl hexadecano

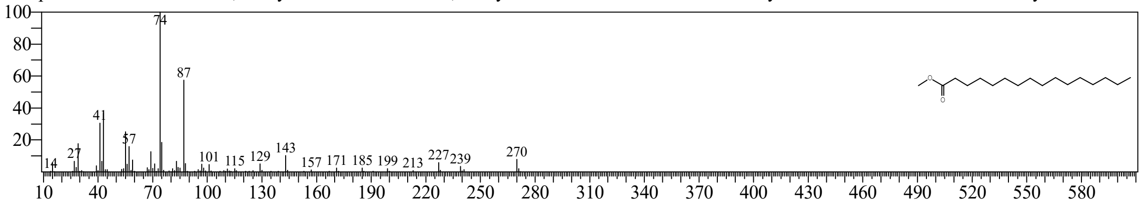

<< Target >>
Line#:4  R.Time:22.158(Scan#:1460)  MassPeaks:305
RawMode:Averaged 22.150-22.167(1459-1461) BasePeak:55.05(1311)
BG Mode:Calc. from Peak  Group 1 - Event 1

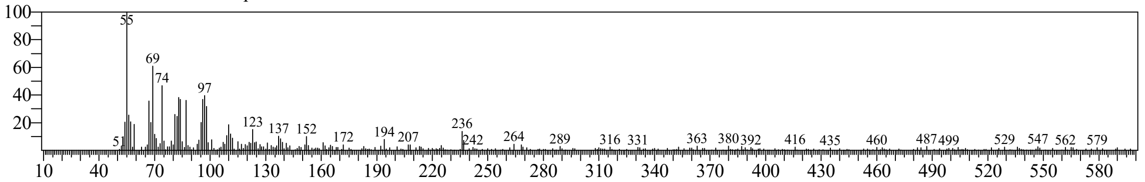


Hit#:1  Entry:26066  Library:NIST14s.lib
SI:94  Formula:C17H32O2  CAS:1120-25-8  MolWeight:268  RetIndex:1886
CompName:9-Hexadecenoic acid, methyl ester, (Z)- $$ Methyl palmitoleate $$ Methyl palmitoleinate $$ Palmitoleic acid, methyl ester $$ Methyl (9Z)-9-he:

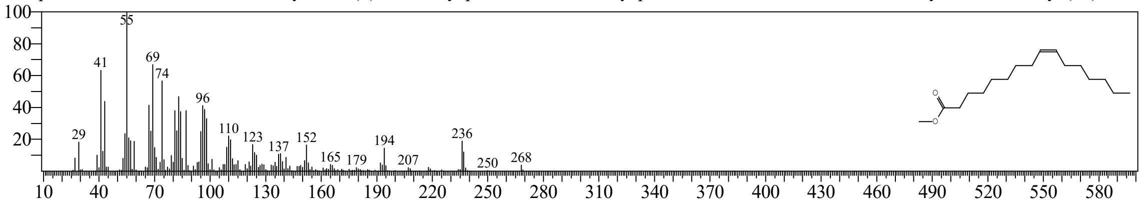


Hit#:2  Entry:30415  Library:NIST17s.lib
SI:94  Formula:C17H32O2  CAS:1120-25-8  MolWeight:268  RetIndex:1886
CompName:9-Hexadecenoic acid, methyl ester, (Z)- $$ Methyl palmitoleate $$ Methyl palmitoleinate $$ Palmitoleic acid, methyl ester $$ Methyl (9Z)-9-he:

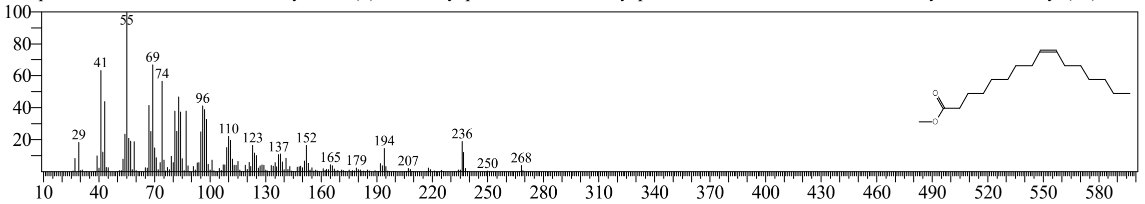


Hit#:3  Entry:111510  Library:NIST17-1.lib
SI:94  Formula:C17H32O2  CAS:56875-67-3  MolWeight:268  RetIndex:1886
CompName:7-Hexadecenoic acid, methyl ester, (Z)- $$ Methyl (7E)-7-hexadecenoate # $$

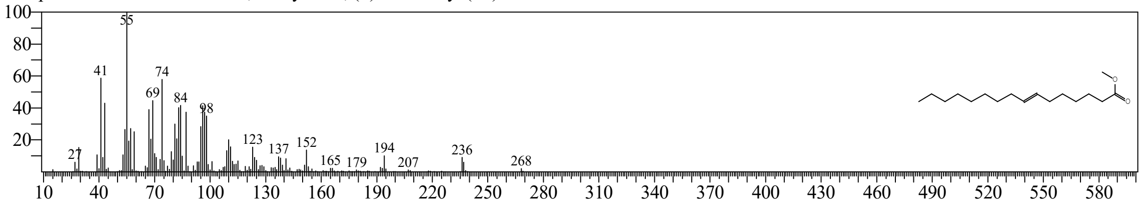


Hit#:4  Entry:26064  Library:NIST14s.lib
SI:93  Formula:C17H32O2  CAS:1120-25-8  MolWeight:268  RetIndex:1886
CompName:9-Hexadecenoic acid, methyl ester, (Z)- $$ Methyl palmitoleate $$ Methyl palmitoleinate $$ Palmitoleic acid, methyl ester $$ Methyl (9Z)-9-he:

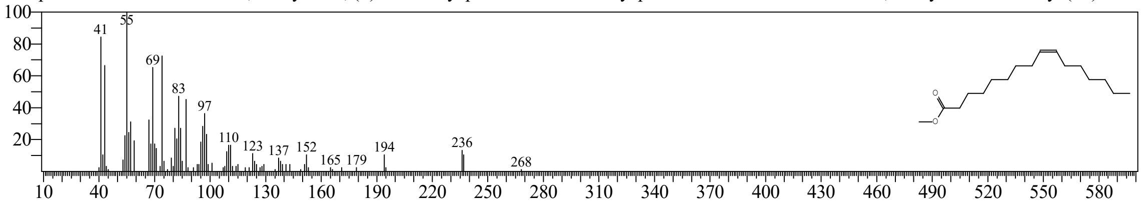


Hit#:5  Entry:30413  Library:NIST17s.lib
SI:93  Formula:C17H32O2  CAS:1120-25-8  MolWeight:268  RetIndex:1886
CompName:9-Hexadecenoic acid, methyl ester, (Z)- $$ Methyl palmitoleate $$ Methyl palmitoleinate $$ Palmitoleic acid, methyl ester $$ Methyl (9Z)-9-he:

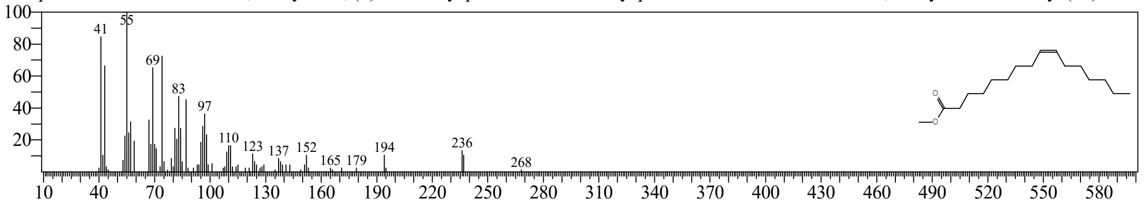

<< Target >>
Line#:5  R.Time:22.775(Scan#:1534)  MassPeaks:266
RawMode:Averaged 22.767-22.783(1533-1535) BasePeak:74.00(3164)
BG Mode:Calc. from Peak  Group 1 - Event 1

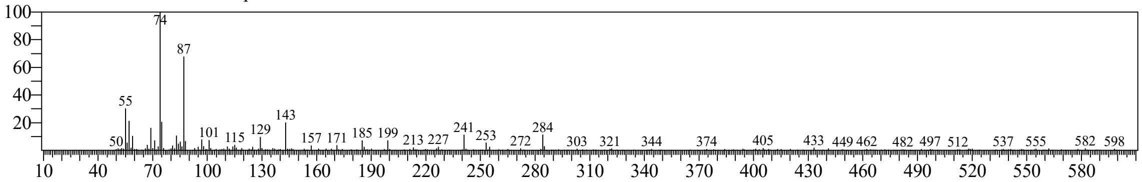


Hit#:1  Entry:31787  Library:NIST17s.lib
SI:93  Formula:C18H36O2  CAS:1731-92-6  MolWeight:284  RetIndex:1978
CompName:Heptadecanoic acid, methyl ester $$ Margaric acid methyl ester $$ Methyl heptadecanoate $$ Methyl margarate $$ n-Heptadecanoic acid methy

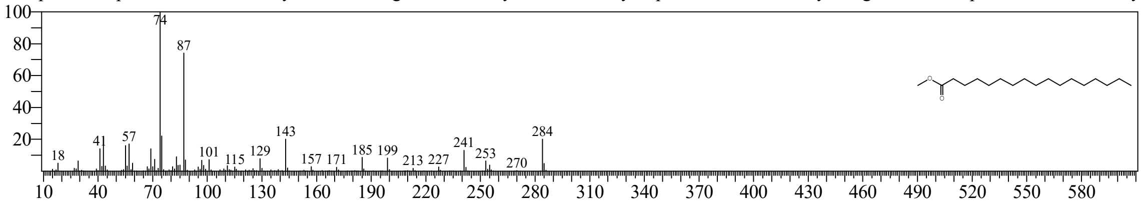


Hit#:2  Entry:27279  Library:NIST14s.lib
SI:93  Formula:C18H36O2  CAS:1731-92-6  MolWeight:284  RetIndex:1978
CompName:Heptadecanoic acid, methyl ester $$ Margaric acid methyl ester $$ Methyl heptadecanoate $$ Methyl margarate $$ n-Heptadecanoic acid methy

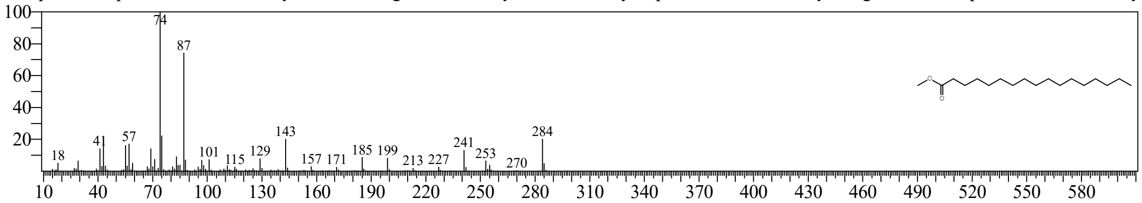


Hit#:3  Entry:27277  Library:NIST14s.lib
SI:92  Formula:C18H36O2  CAS:6929-04-0  MolWeight:284  RetIndex:1914
CompName:Hexadecanoic acid, 15-methyl-, methyl ester $$ Methyl isoheptadecanoate $$ Methyl 15-methylhexadecanoate $$

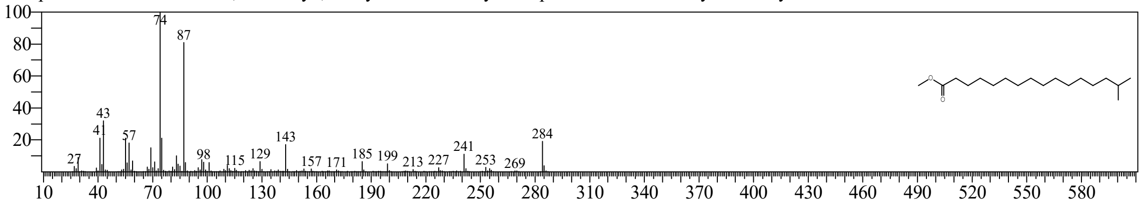


Hit#:4  Entry:127714  Library:NIST17-1.lib
SI:92  Formula:C18H36O2  CAS:6929-04-0  MolWeight:284  RetIndex:1914
CompName:Hexadecanoic acid, 15-methyl-, methyl ester $$ Methyl isoheptadecanoate $$ Methyl 15-methylhexadecanoate $$

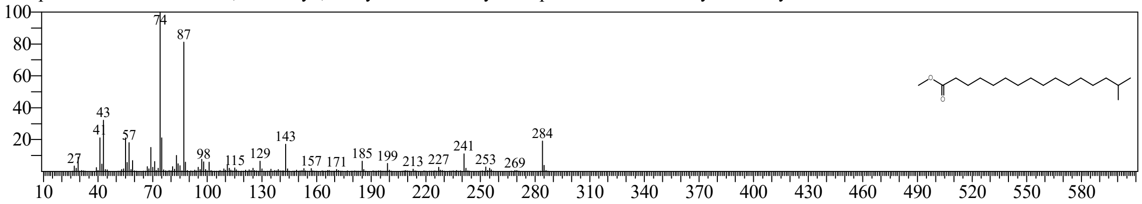


Hit#:5  Entry:27275  Library:NIST14s.lib
SI:92  Formula:C18H36O2  CAS:1731-92-6  MolWeight:284  RetIndex:1978
CompName:Heptadecanoic acid, methyl ester $$ Margaric acid methyl ester $$ Methyl heptadecanoate $$ Methyl margarate $$ n-Heptadecanoic acid methy

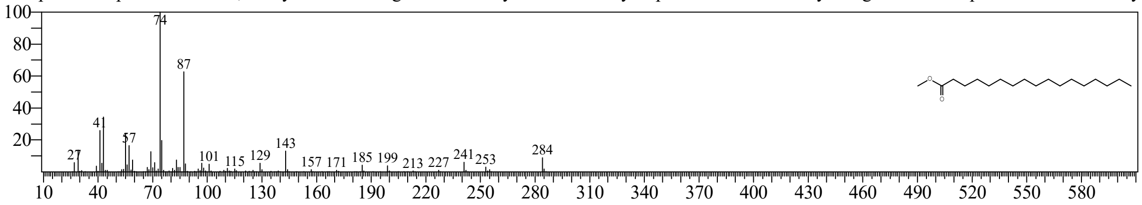

<< Target >>
Line#:6 R.Time:24.083(Scan#:1691) MassPeaks:341
RawMode:Averaged 24.075-24.092(1690-1692) BasePeak:55.00(715)
BG Mode:Calc. from Peak Group 1 - Event 1

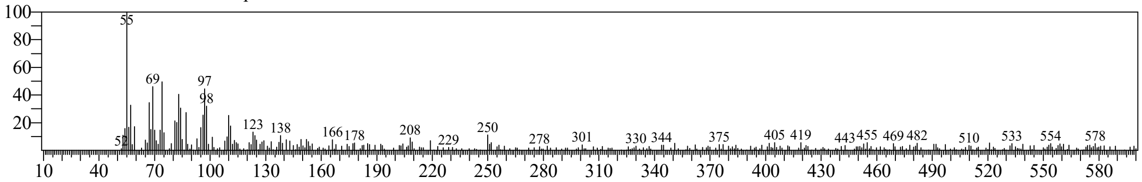


Hit#:1 Entry:31597 Library:NIST17s.lib
SI:88 Formula:C18H34O2 CAS:10152-61-1 MolWeight:282 RetIndex:1941
CompName:Cyclopropaneoctanoic acid, 2-hexyl-, methyl ester $$ Methyl 8-(2-hexylcyclopropyl)octanoate # $$

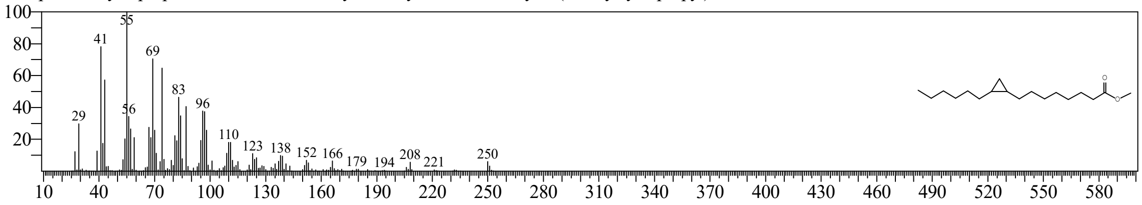


Hit#:2 Entry:27110 Library:NIST14s.lib
SI:88 Formula:C18H34O2 CAS:10152-61-1 MolWeight:282 RetIndex:1941
CompName:Cyclopropaneoctanoic acid, 2-hexyl-, methyl ester $$ Methyl 8-(2-hexylcyclopropyl)octanoate # $$

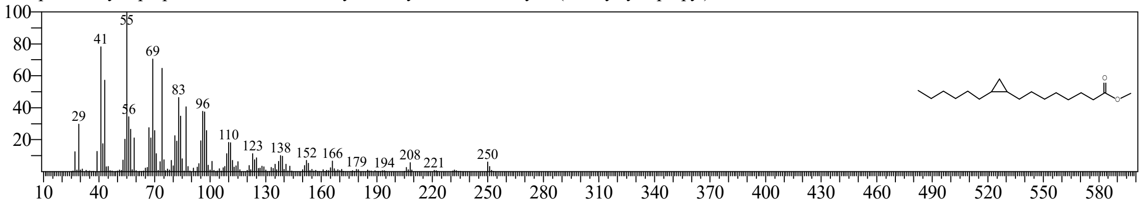


Hit#:3 Entry:152749 Library:NIST14.lib
SI:88 Formula:C21H40O2 CAS:2390-09-2 MolWeight:324 RetIndex:0
CompName:cis-Methyl 11-eicosenoate $$ 11-Eicosenoic acid, methyl ester, (Z)- $$

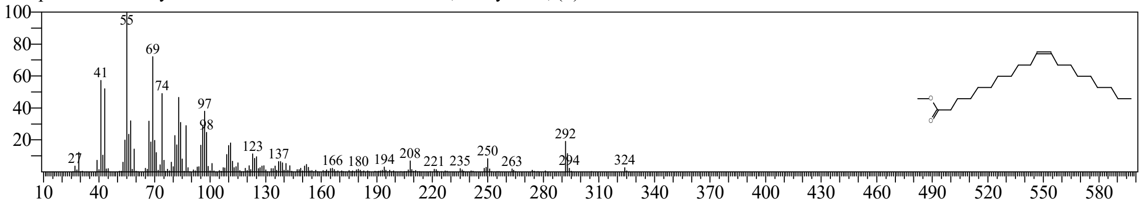


Hit#:4 Entry:125438 Library:NIST17-1.lib
SI:88 Formula:C18H34O2 CAS:0-00-0 MolWeight:282 RetIndex:1986
CompName:cis-10-Heptadecenoic acid, methyl ester

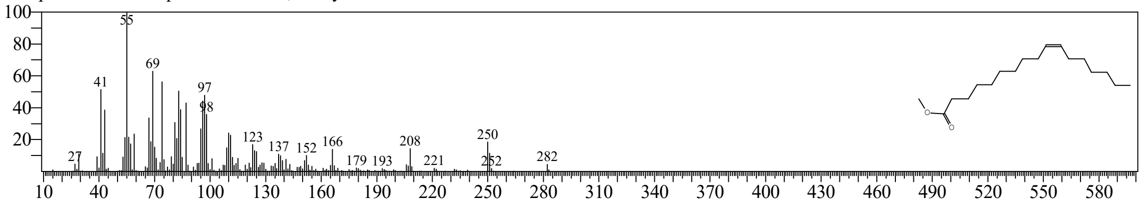


Hit#:5 Entry:115094 Library:NIST14.lib
SI:88 Formula:C18H34O2 CAS:0-00-0 MolWeight:282 RetIndex:1986
CompName:cis-10-Heptadecenoic acid, methyl ester

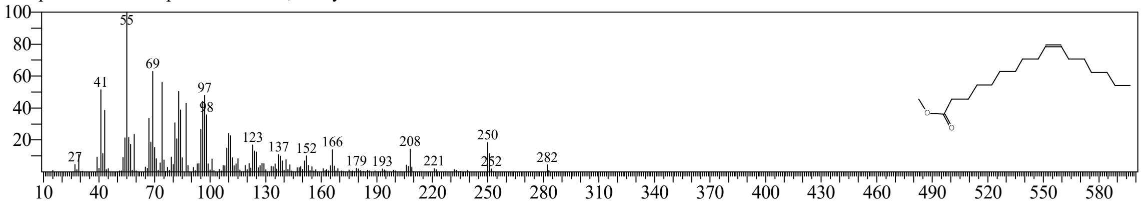

<< Target >>
Line#:7   R.Time:24.875(Scan#:1786)  MassPeaks:365
RawMode:Averaged 24.867-24.883(1785-1787) BasePeak:74.00(125680)
BG Mode:Calc. from Peak  Group 1 - Event 1

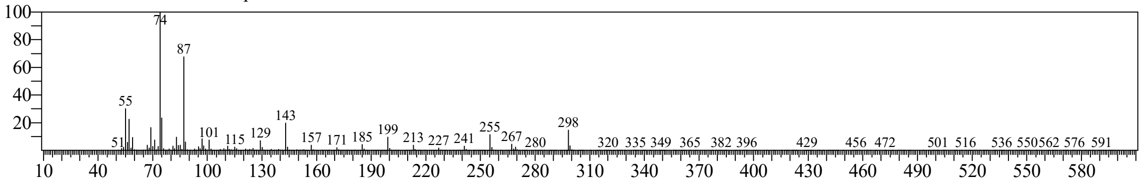


Hit#:1  Entry:129694  Library:NIST14.lib
SI:98  Formula:C19H38O2  CAS:112-61-8  MolWeight:298  RetIndex:2077
CompName:Methyl stearate $$ Octadecanoic acid, methyl ester $$ Stearic acid, methyl ester $$ n-Octadecanoic acid, methyl ester $$ Kemester 9718 $$ Me

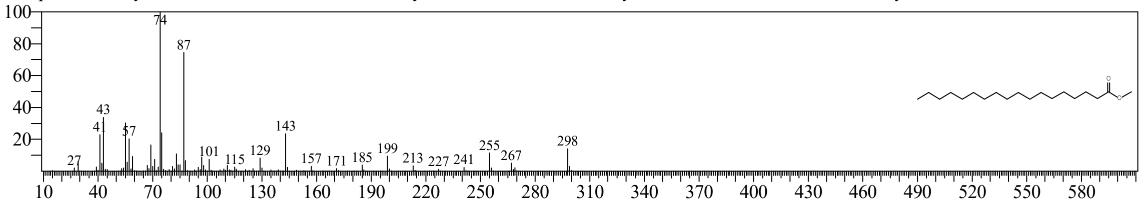


Hit#:2  Entry:141978  Library:NIST17-1.lib
SI:98  Formula:C19H38O2  CAS:112-61-8  MolWeight:298  RetIndex:2077
CompName:Methyl stearate $$ Octadecanoic acid, methyl ester $$ Stearic acid, methyl ester $$ n-Octadecanoic acid, methyl ester $$ Kemester 9718 $$ Me

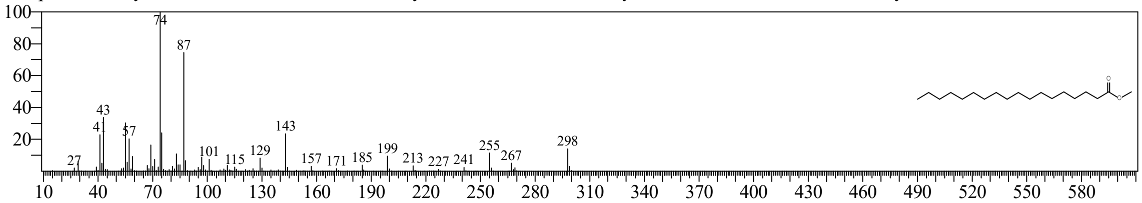


Hit#:3  Entry:28255  Library:NIST14s.lib
SI:97  Formula:C19H38O2  CAS:112-61-8  MolWeight:298  RetIndex:2077
CompName:Methyl stearate $$ Octadecanoic acid, methyl ester $$ Stearic acid, methyl ester $$ n-Octadecanoic acid, methyl ester $$ Kemester 9718 $$ Me

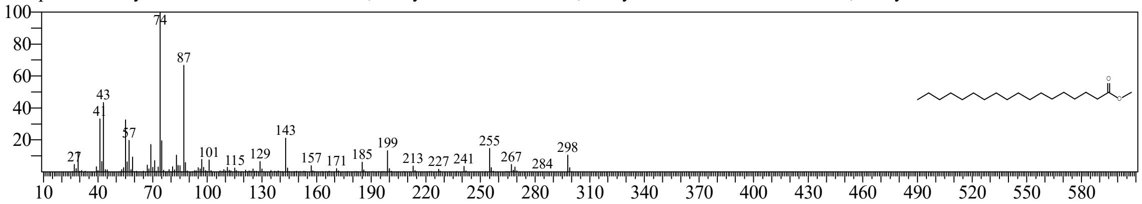


Hit#:4  Entry:32873  Library:NIST17s.lib
SI:97  Formula:C19H38O2  CAS:112-61-8  MolWeight:298  RetIndex:2077
CompName:Methyl stearate $$ Octadecanoic acid, methyl ester $$ Stearic acid, methyl ester $$ n-Octadecanoic acid, methyl ester $$ Kemester 9718 $$ Me

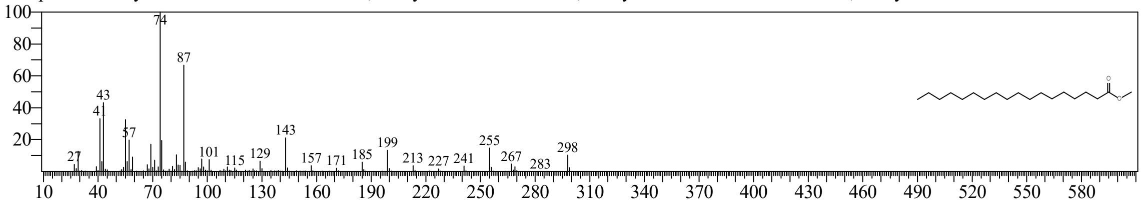


Hit#:5  Entry:28257  Library:NIST14s.lib
SI:96  Formula:C19H38O2  CAS:112-61-8  MolWeight:298  RetIndex:2077
CompName:Methyl stearate $$ Octadecanoic acid, methyl ester $$ Stearic acid, methyl ester $$ n-Octadecanoic acid, methyl ester $$ Kemester 9718 $$ Me

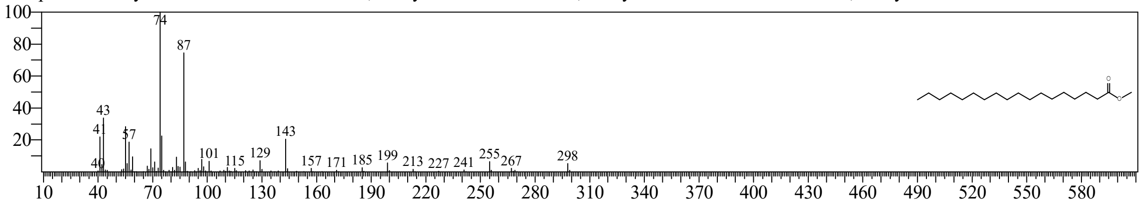

<< Target >>
Line#:8 R.Time:26.258(Scan#:1952) MassPeaks:374
RawMode:Averaged 26.250-26.267(1951-1953) BasePeak:55.05(224202)
BG Mode:Calc. from Peak Group 1 - Event 1

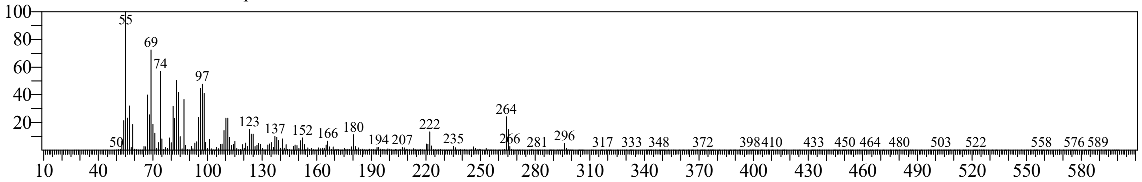


Hit#:1 Entry:32747 Library:NIST17s.lib
SI:96 Formula:C19H36O2 CAS:1937-62-8 MolWeight:296 RetIndex:2085
CompName:9-Octadecenoic acid, methyl ester, (E)- $$ Elaidic acid, methyl ester $$ Methyl elaidate $$ Methyl trans-9-octadecenoate $$ (E)-9-Octadecenoic

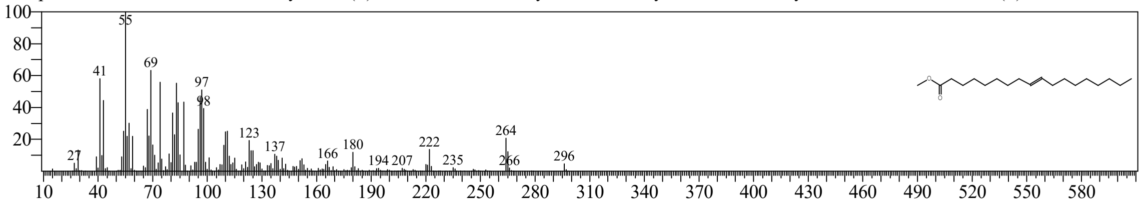


Hit#:2 Entry:28138 Library:NIST14s.lib
SI:96 Formula:C19H36O2 CAS:1937-62-8 MolWeight:296 RetIndex:2085
CompName:9-Octadecenoic acid, methyl ester, (E)- $$ Elaidic acid, methyl ester $$ Methyl elaidate $$ Methyl trans-9-octadecenoate $$ (E)-9-Octadecenoic

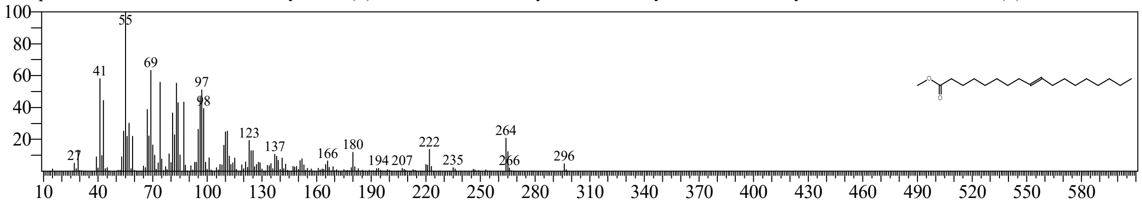


Hit#:3 Entry:127664 Library:NIST14.lib
SI:96 Formula:C19H36O2 CAS:112-62-9 MolWeight:296 RetIndex:2085
CompName:9-Octadecenoic acid (Z)-, methyl ester $$ Oleic acid, methyl ester $$ Emery oleic acid ester 2301 $$ Methyl cis-9-octadecenoate $$ Methyl ole

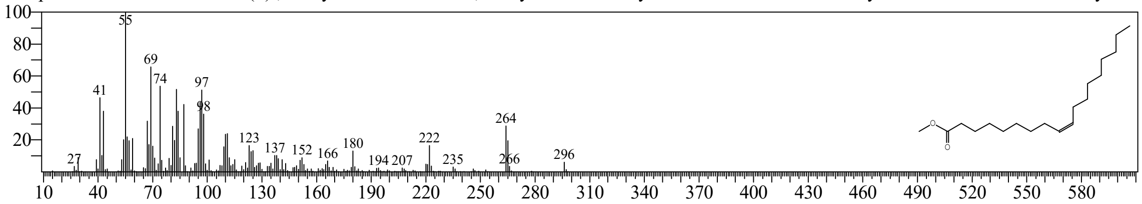


Hit#:4 Entry:139650 Library:NIST17-1.lib
SI:96 Formula:C19H36O2 CAS:112-62-9 MolWeight:296 RetIndex:2085
CompName:9-Octadecenoic acid (Z)-, methyl ester $$ Oleic acid, methyl ester $$ Emery oleic acid ester 2301 $$ Methyl cis-9-octadecenoate $$ Methyl ole

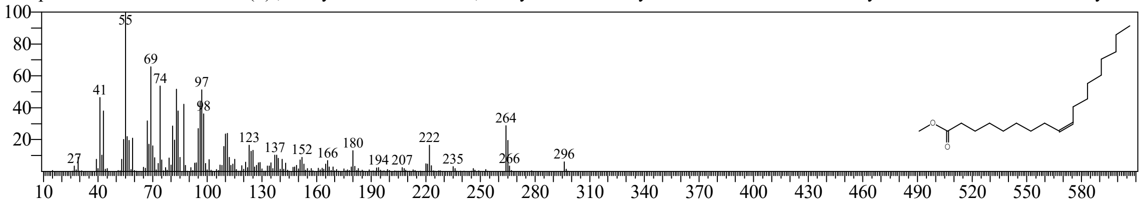


Hit#:5 Entry:32744 Library:NIST17s.lib
SI:95 Formula:C19H36O2 CAS:112-62-9 MolWeight:296 RetIndex:2085
CompName:9-Octadecenoic acid (Z)-, methyl ester $$ Oleic acid, methyl ester $$ Emery oleic acid ester 2301 $$ Methyl cis-9-octadecenoate $$ Methyl ole

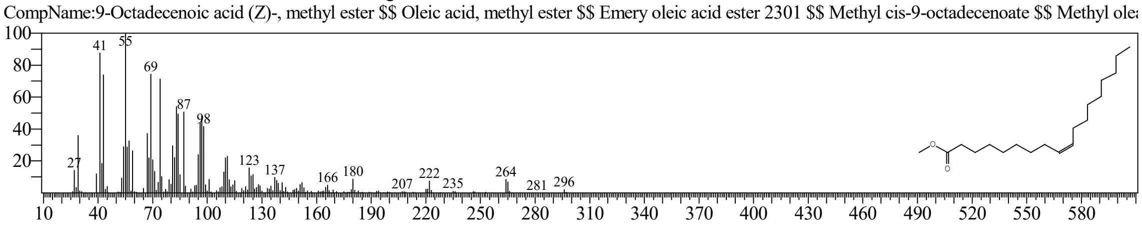

<< Target >>
Line#:9   R.Time:26.383(Scan#:1967)  MassPeaks:356
RawMode:Averaged 26.375-26.392(1966-1968) BasePeak:55.05(18378)
BG Mode:Calc. from Peak  Group 1 - Event 1

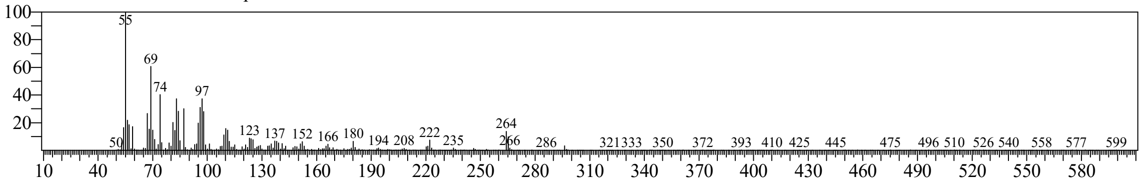


Hit#:1  Entry:32748  Library:NIST17s.lib
SI:95  Formula:C19H36O2  CAS:52380-33-3  MolWeight:296  RetIndex:2085
CompName:11-Octadecenoic acid, methyl ester $$ Methyl 11-octadecenoate $$ Octadec-11-enoic acid, methyl ester $$ Methyl (11E)-11-octadecenoate # $$

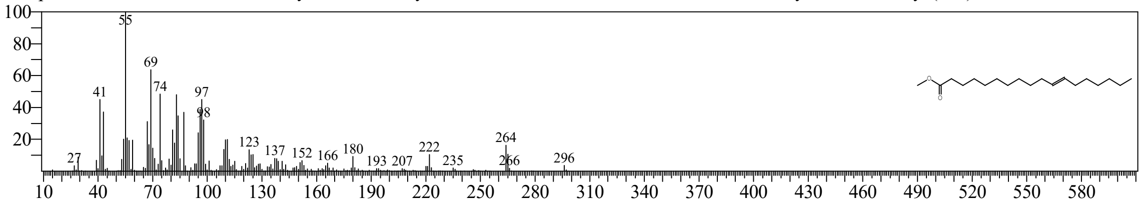


Hit#:2  Entry:28139  Library:NIST14s.lib
SI:95  Formula:C19H36O2  CAS:52380-33-3  MolWeight:296  RetIndex:2085
CompName:11-Octadecenoic acid, methyl ester $$ Methyl 11-octadecenoate $$ Octadec-11-enoic acid, methyl ester $$ Methyl (11E)-11-octadecenoate # $$

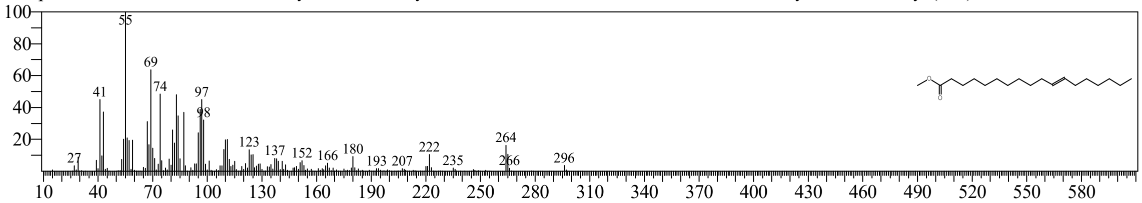


Hit#:3  Entry:127661  Library:NIST14.lib
SI:95  Formula:C19H36O2  CAS:0-00-0  MolWeight:296  RetIndex:2085
CompName:cis-13-Octadecenoic acid, methyl ester

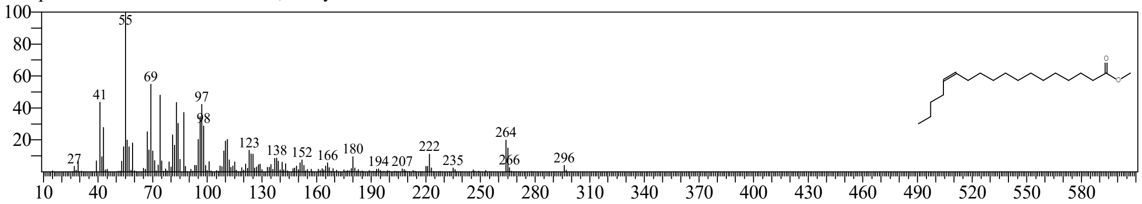


Hit#:4  Entry:139647  Library:NIST17-1.lib
SI:95  Formula:C19H36O2  CAS:0-00-0  MolWeight:296  RetIndex:2085
CompName:cis-13-Octadecenoic acid, methyl ester

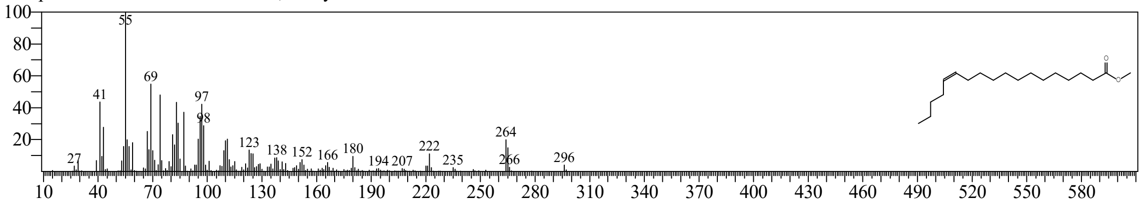


Hit#:5  Entry:127650  Library:NIST14.lib
SI:94  Formula:C19H36O2  CAS:13481-95-3  MolWeight:296  RetIndex:2085
CompName:10-Octadecenoic acid, methyl ester $$ Methyl 10-octadecenoate $$

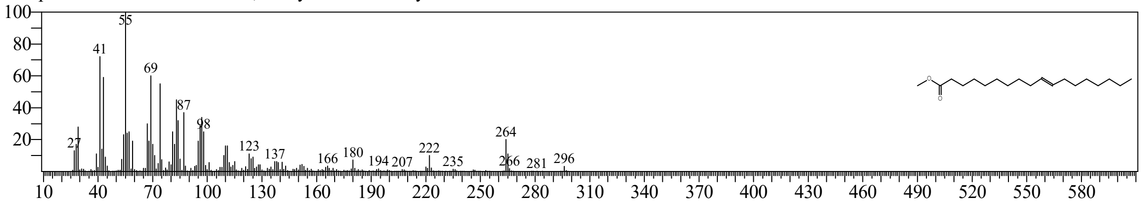

<< Target >>
Line#:10   R.Time:27.725(Scan#:2128)  MassPeaks:357
RawMode:Averaged 27.717-27.733(2127-2129) BasePeak:67.00(3628)
BG Mode:Calc. from Peak  Group 1 - Event 1

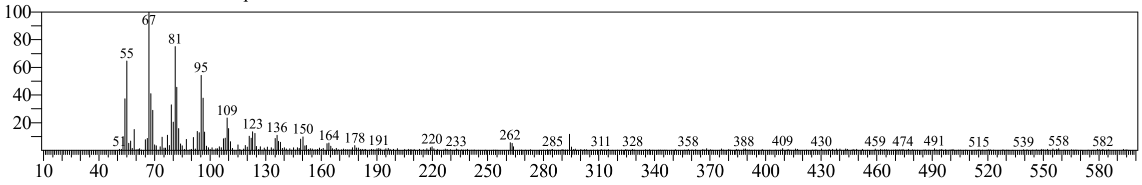


Hit#:1  Entry:27999  Library:NIST14s.lib
SI:96  Formula:C19H34O2  CAS:112-63-0  MolWeight:294  RetIndex:2093
CompName:9,12-Octadecadienoic acid (Z,Z)-, methyl ester $$ Linoleic acid, methyl ester $$ Methyl cis,cis-9,12-octadecadienoate $$ Methyl linoleate $$ M

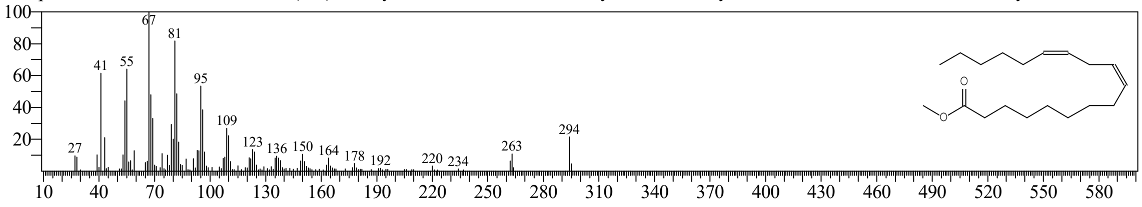


Hit#:2  Entry:32588  Library:NIST17s.lib
SI:96  Formula:C19H34O2  CAS:112-63-0  MolWeight:294  RetIndex:2093
CompName:9,12-Octadecadienoic acid (Z,Z)-, methyl ester $$ Linoleic acid, methyl ester $$ Methyl cis,cis-9,12-octadecadienoate $$ Methyl linoleate $$ M

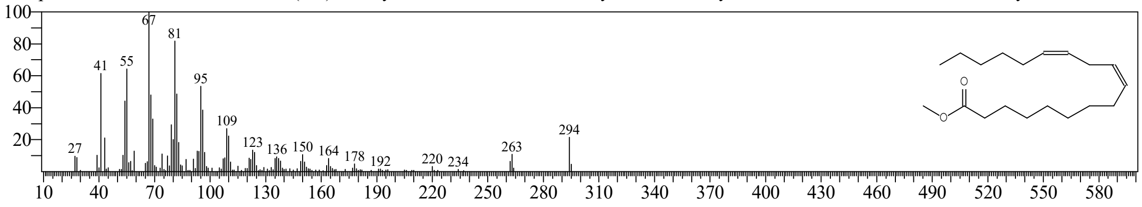


Hit#:3  Entry:125931  Library:NIST14.lib
SI:96  Formula:C19H34O2  CAS:2462-85-3  MolWeight:294  RetIndex:2093
CompName:9,12-Octadecadienoic acid, methyl ester $$ 9,12-Octadecenoic acid, methyl ester $$ Methyl octadeca-9,12-dienoate $$ Methyl 9,12-octadecadi

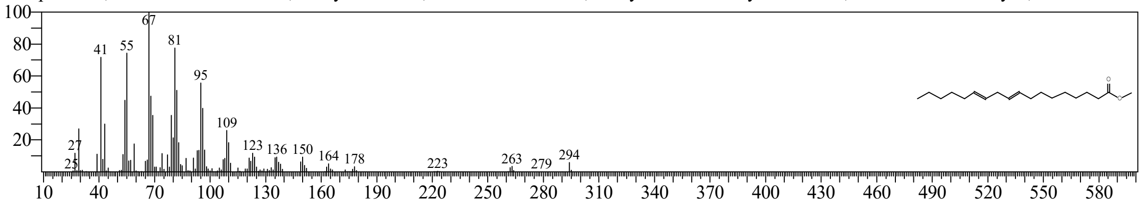


Hit#:4  Entry:137663  Library:NIST17-1.lib
SI:96  Formula:C19H34O2  CAS:2462-85-3  MolWeight:294  RetIndex:2093
CompName:9,12-Octadecadienoic acid, methyl ester $$ 9,12-Octadecenoic acid, methyl ester $$ Methyl octadeca-9,12-dienoate $$ Methyl 9,12-octadecadi

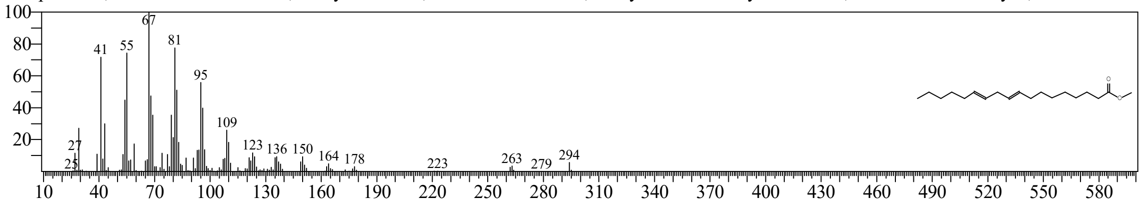


Hit#:5  Entry:32589  Library:NIST17s.lib
SI:95  Formula:C19H34O2  CAS:112-63-0  MolWeight:294  RetIndex:2093
CompName:9,12-Octadecadienoic acid (Z,Z)-, methyl ester $$ Linoleic acid, methyl ester $$ Methyl cis,cis-9,12-octadecadienoate $$ Methyl linoleate $$ M

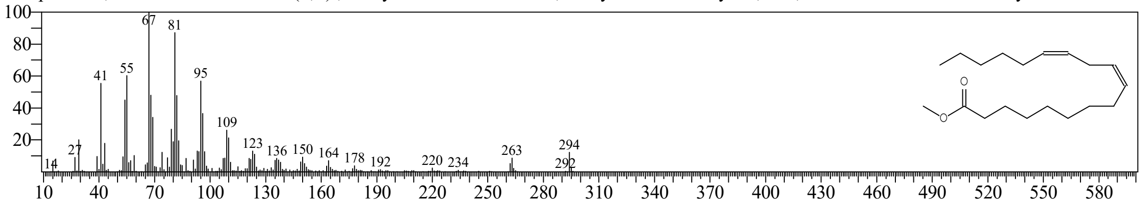

<< Target >>
Line#:11   R.Time:27.942(Scan#:2154)  MassPeaks:349
RawMode:Averaged 27.933-27.950(2153-2155) BasePeak:67.00(2505)
BG Mode:Calc. from Peak  Group 1 - Event 1

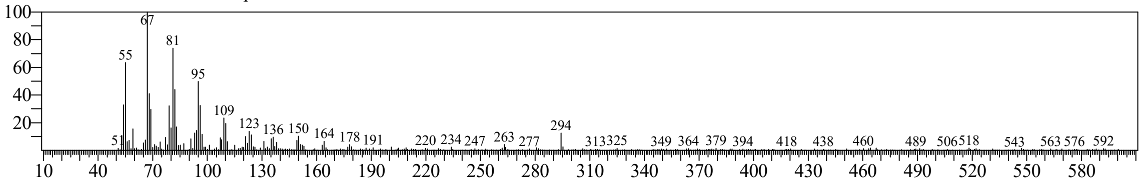


Hit#:1  Entry:32587  Library:NIST17s.lib
SI:95  Formula:C19H34O2  CAS:13038-47-6  MolWeight:294  RetIndex:2093
CompName:9,11-Octadecadienoic acid, methyl ester, (E,E)- $$ Methyl trans-9,trans-11-octadecadienoate $$ Methyl trans,trans-9,11-octadecadienoate $$ M

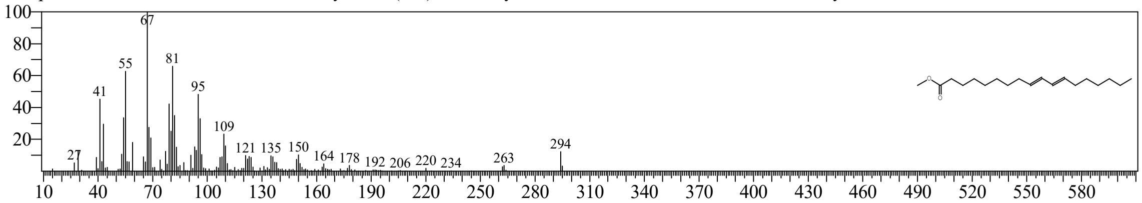


Hit#:2  Entry:27999  Library:NIST14s.lib
SI:95  Formula:C19H34O2  CAS:112-63-0  MolWeight:294  RetIndex:2093
CompName:9,12-Octadecadienoic acid (Z,Z)-, methyl ester $$ Linoleic acid, methyl ester $$ Methyl cis,cis-9,12-octadecadienoate $$ Methyl linoleate $$ M

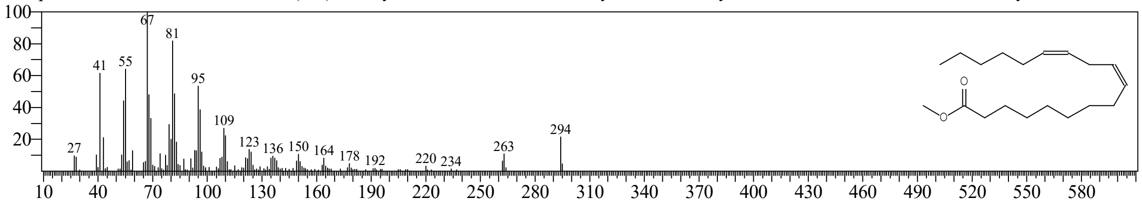


Hit#:3  Entry:32588  Library:NIST17s.lib
SI:95  Formula:C19H34O2  CAS:112-63-0  MolWeight:294  RetIndex:2093
CompName:9,12-Octadecadienoic acid (Z,Z)-, methyl ester $$ Linoleic acid, methyl ester $$ Methyl cis,cis-9,12-octadecadienoate $$ Methyl linoleate $$ M

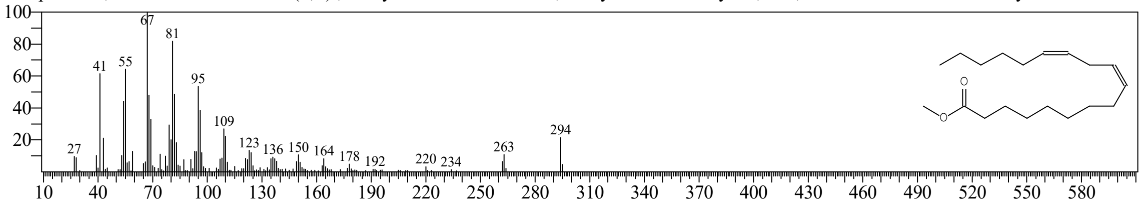


Hit#:4  Entry:137663  Library:NIST17-1.lib
SI:94  Formula:C19H34O2  CAS:2462-85-3  MolWeight:294  RetIndex:2093
CompName:9,12-Octadecadienoic acid, methyl ester $$ 9,12-Octadecenoic acid, methyl ester $$ Methyl octadeca-9,12-dienoate $$ Methyl 9,12-octadecadi

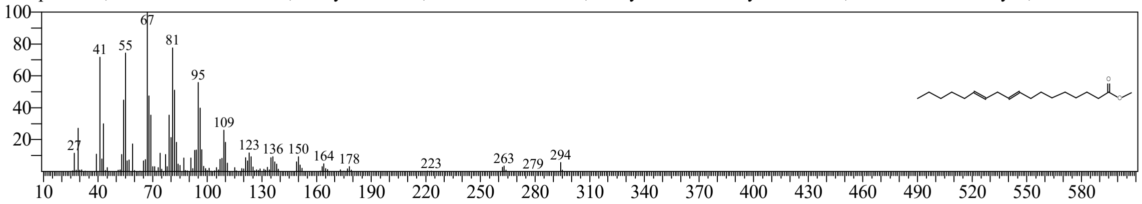


Hit#:5  Entry:125931  Library:NIST14.lib
SI:94  Formula:C19H34O2  CAS:2462-85-3  MolWeight:294  RetIndex:2093
CompName:9,12-Octadecadienoic acid, methyl ester $$ 9,12-Octadecenoic acid, methyl ester $$ Methyl octadeca-9,12-dienoate $$ Methyl 9,12-octadecadi

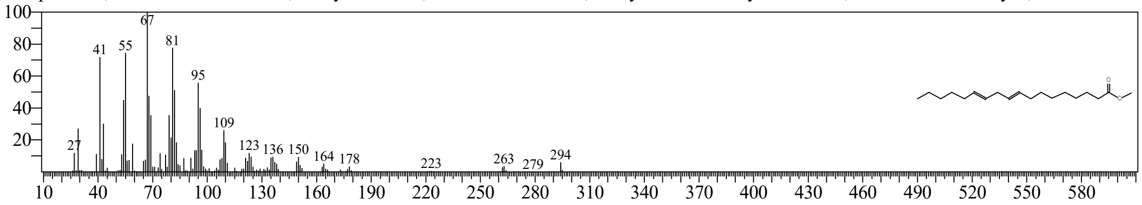

<< Target >>
Line#:12   R.Time:28.375(Scan#:2206)  MassPeaks:388
RawMode:Averaged 28.367-28.383(2205-2207) BasePeak:67.05(430232)
BG Mode:Calc. from Peak  Group 1 - Event 1

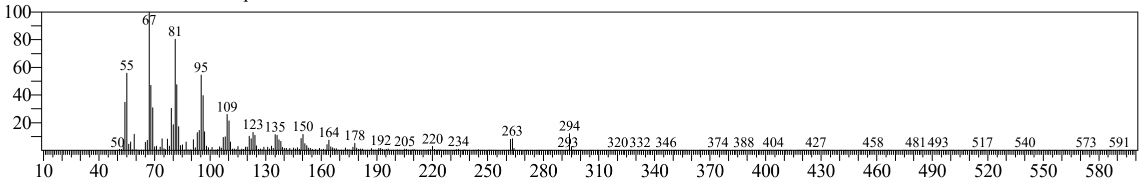


Hit#:1  Entry:32588  Library:NIST17s.lib
SI:97  Formula:C19H34O2  CAS:112-63-0  MolWeight:294  RetIndex:2093
CompName:9,12-Octadecadienoic acid (Z,Z)-, methyl ester $$ Linoleic acid, methyl ester $$ Methyl cis,cis-9,12-octadecadienoate $$ Methyl linoleate $$ M

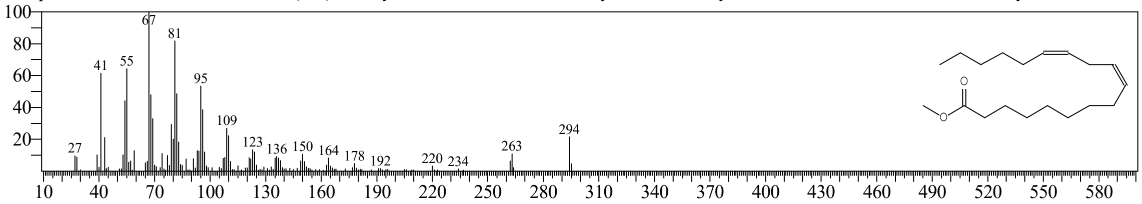


Hit#:2  Entry:27999  Library:NIST14s.lib
SI:97  Formula:C19H34O2  CAS:112-63-0  MolWeight:294  RetIndex:2093
CompName:9,12-Octadecadienoic acid (Z,Z)-, methyl ester $$ Linoleic acid, methyl ester $$ Methyl cis,cis-9,12-octadecadienoate $$ Methyl linoleate $$ M

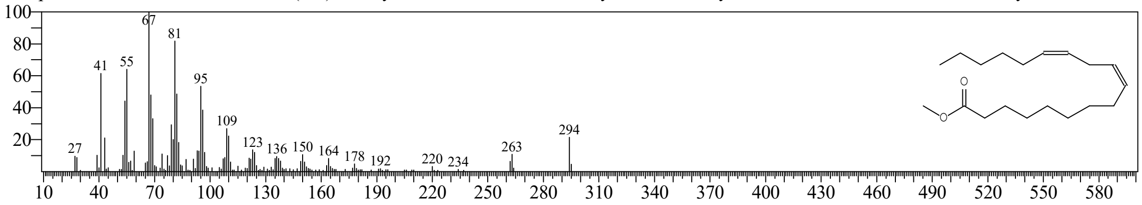


Hit#:3  Entry:32589  Library:NIST17s.lib
SI:97  Formula:C19H34O2  CAS:112-63-0  MolWeight:294  RetIndex:2093
CompName:9,12-Octadecadienoic acid (Z,Z)-, methyl ester $$ Linoleic acid, methyl ester $$ Methyl cis,cis-9,12-octadecadienoate $$ Methyl linoleate $$ M

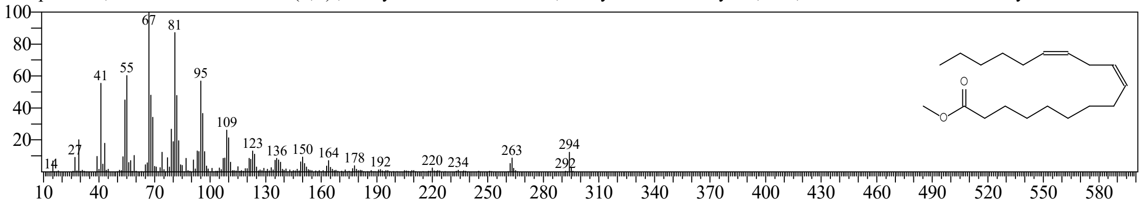


Hit#:4  Entry:28000  Library:NIST14s.lib
SI:97  Formula:C19H34O2  CAS:112-63-0  MolWeight:294  RetIndex:2093
CompName:9,12-Octadecadienoic acid (Z,Z)-, methyl ester $$ Linoleic acid, methyl ester $$ Methyl cis,cis-9,12-octadecadienoate $$ Methyl linoleate $$ M

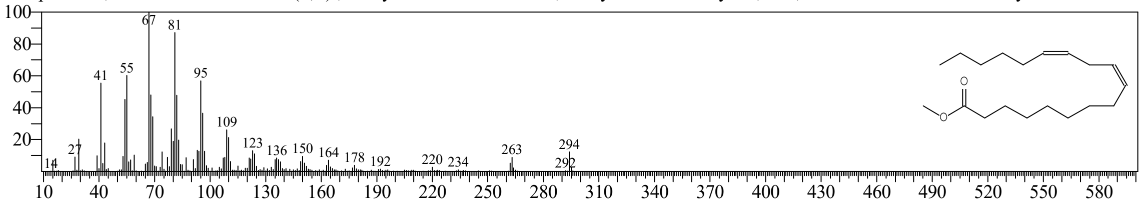


Hit#:5  Entry:137664  Library:NIST17-1.lib
SI:95  Formula:C19H34O2  CAS:0-00-0  MolWeight:294  RetIndex:2093
CompName:Methyl 10-trans,12-cis-octadecadienoate

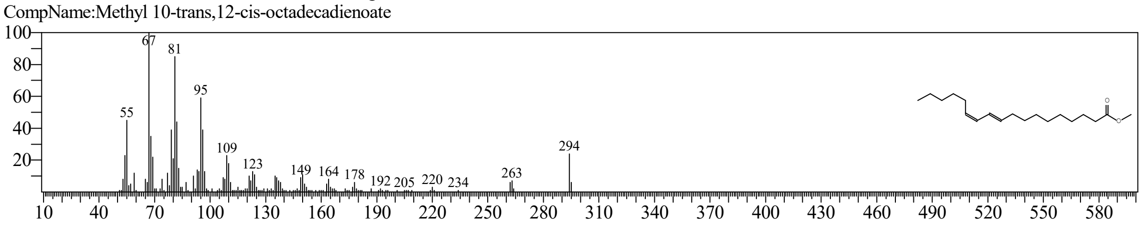

<< Target >>
Line#:13 R.Time:29.267(Scan#:2313) MassPeaks:346
RawMode:Averaged 29.258-29.275(2312-2314) BasePeak:74.00(12650)
BG Mode:Calc. from Peak Group 1 - Event 1

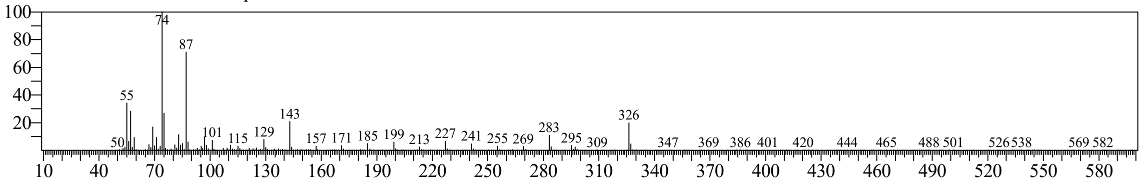


Hit#:1 Entry:154704 Library:NIST14.lib
SI:97 Formula:C21H42O2 CAS:0-00-0 MolWeight:326 RetIndex:2212
CompName:Methyl 18-methylnonadecanoate

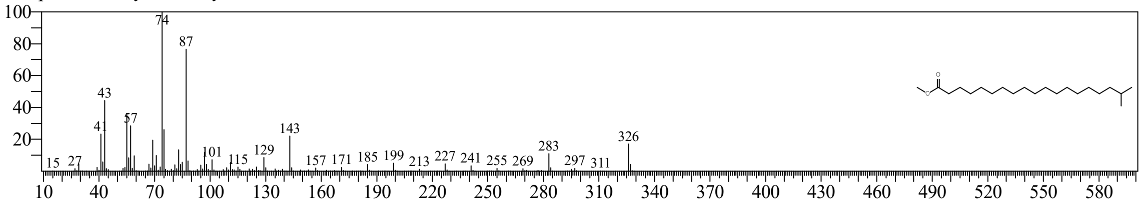


Hit#:2 Entry:170238 Library:NIST17-1.lib
SI:97 Formula:C21H42O2 CAS:0-00-0 MolWeight:326 RetIndex:2212
CompName:Methyl 18-methylnonadecanoate

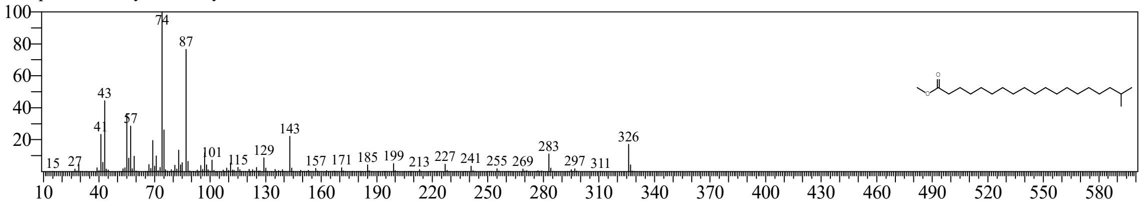


Hit#:3 Entry:34726 Library:NIST17s.lib
SI:96 Formula:C21H42O2 CAS:1120-28-1 MolWeight:326 RetIndex:2276
CompName:Eicosanoic acid, methyl ester $$ Methyl arachisate $$ Methyl eicosanoate $$ Arachidic acid methyl ester $$ Kemester 2050 $$ Methyl aracidat

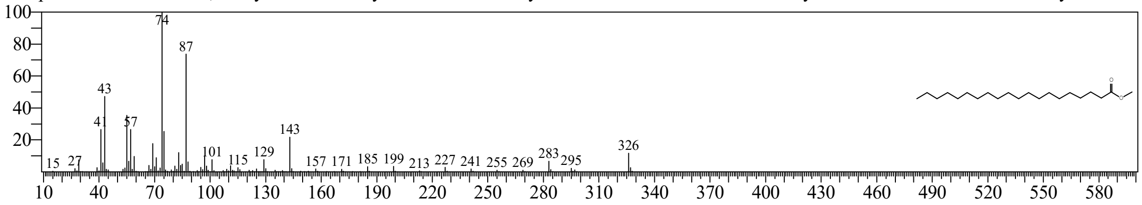


Hit#:4 Entry:34724 Library:NIST17s.lib
SI:93 Formula:C21H42O2 CAS:1120-28-1 MolWeight:326 RetIndex:2276
CompName:Eicosanoic acid, methyl ester $$ Methyl arachisate $$ Methyl eicosanoate $$ Arachidic acid methyl ester $$ Kemester 2050 $$ Methyl aracidat

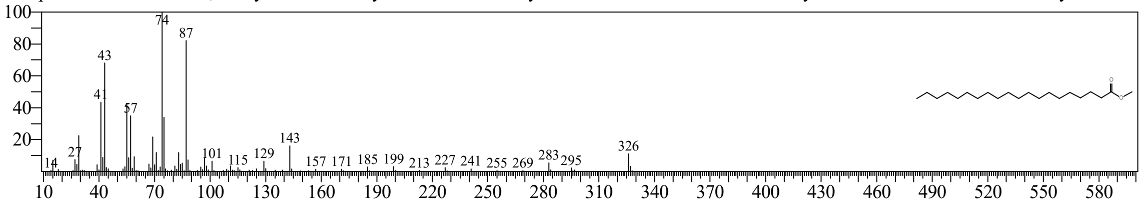


Hit#:5 Entry:29931 Library:NIST14s.lib
SI:93 Formula:C21H42O2 CAS:1120-28-1 MolWeight:326 RetIndex:2276
CompName:Eicosanoic acid, methyl ester $$ Methyl arachisate $$ Methyl eicosanoate $$ Arachidic acid methyl ester $$ Kemester 2050 $$ Methyl aracidat

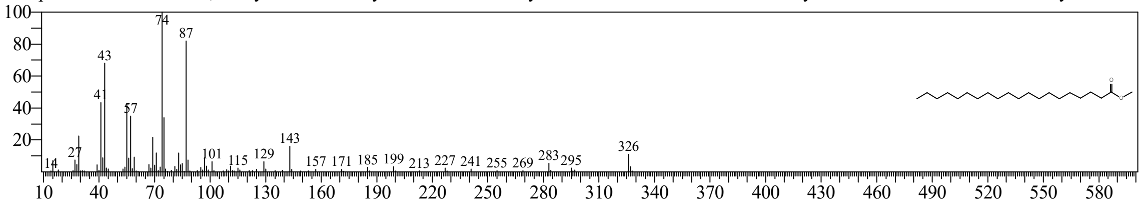

<< Target >>
Line#:14  R.Time:29.817(Scan#:2379)  MassPeaks:339
RawMode:Averaged 29.808-29.825(2378-2380) BasePeak:79.05(7923)
BG Mode:Calc. from Peak  Group 1 - Event 1

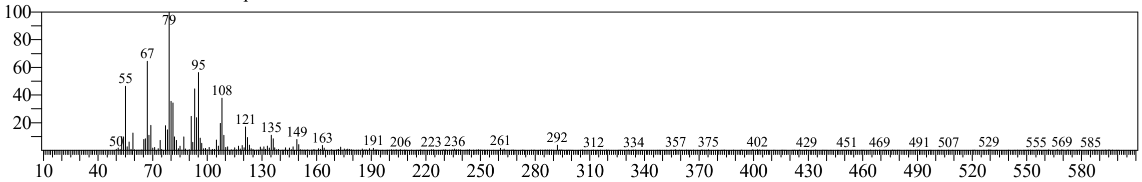


Hit#:1  Entry:29609  Library:NIST14s.lib
SI:95  Formula:C21H36O2  CAS:55682-88-7  MolWeight:320  RetIndex:2300
CompName:11,14,17-Eicosatrienoic acid, methyl ester $$ Methyl 11,14,17-icosatrienoate $$ Methyl 11,14,17-eicosatrienoate $$

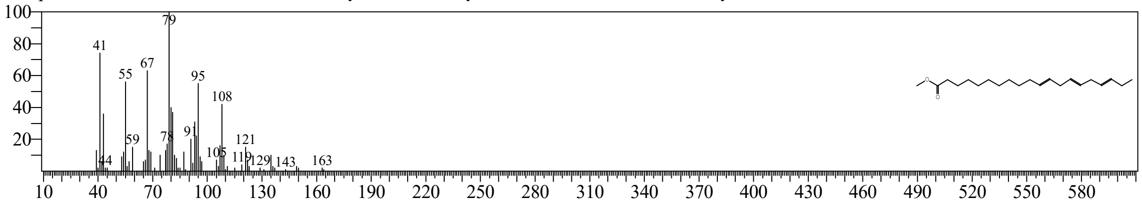


Hit#:2  Entry:34362  Library:NIST17s.lib
SI:95  Formula:C21H36O2  CAS:55682-88-7  MolWeight:320  RetIndex:2300
CompName:11,14,17-Eicosatrienoic acid, methyl ester $$ Methyl 11,14,17-icosatrienoate $$ Methyl 11,14,17-eicosatrienoate $$

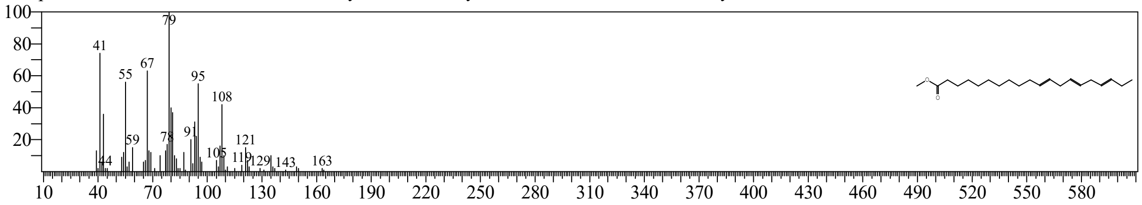


Hit#:3  Entry:32438  Library:NIST17s.lib
SI:95  Formula:C19H32O2  CAS:301-00-8  MolWeight:292  RetIndex:2101
CompName:9,12,15-Octadecatrienoic acid, methyl ester, (Z,Z,Z)- $$ Linolenic acid, methyl ester $$ Methyl all-cis-9,12,15-octadecatrienoate $$ Methyl lino

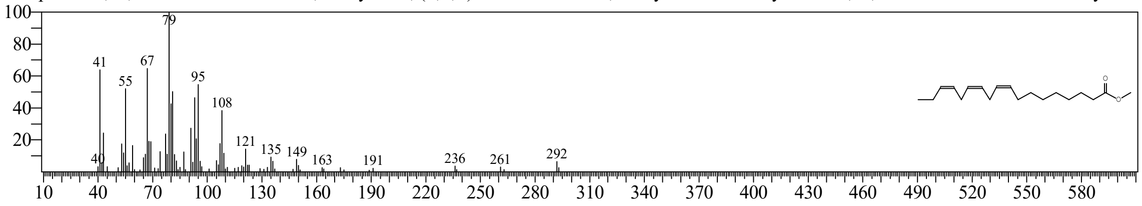


Hit#:4  Entry:135724  Library:NIST17-1.lib
SI:95  Formula:C19H32O2  CAS:301-00-8  MolWeight:292  RetIndex:2101
CompName:9,12,15-Octadecatrienoic acid, methyl ester, (Z,Z,Z)- $$ Linolenic acid, methyl ester $$ Methyl all-cis-9,12,15-octadecatrienoate $$ Methyl lino

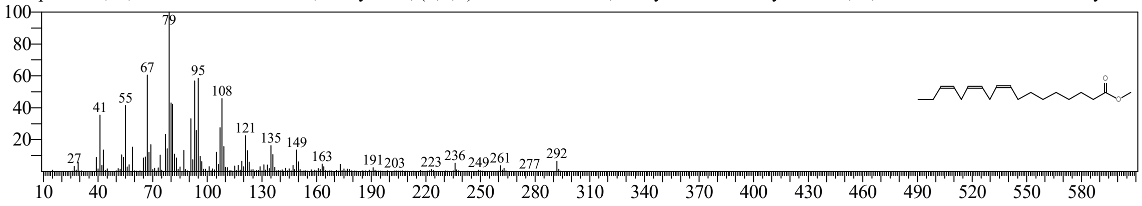


Hit#:5  Entry:124228  Library:NIST14.lib
SI:95  Formula:C19H32O2  CAS:301-00-8  MolWeight:292  RetIndex:2101
CompName:9,12,15-Octadecatrienoic acid, methyl ester, (Z,Z,Z)- $$ Linolenic acid, methyl ester $$ Methyl all-cis-9,12,15-octadecatrienoate $$ Methyl lino

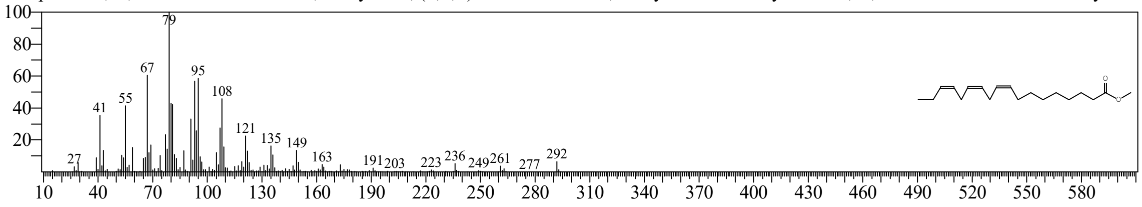

<< Target >>
Line#:15 R.Time:30.292(Scan#:2436) MassPeaks:362
RawMode:Averaged 30.283-30.300(2435-2437) BasePeak:79.05(6089)
BG Mode:Calc. from Peak Group 1 - Event 1

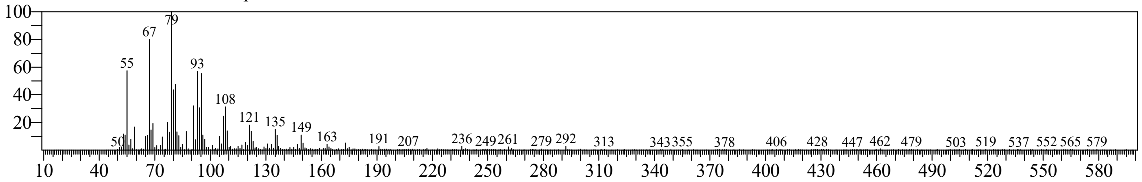


Hit#:1 Entry:135724 Library:NIST17-1.lib
SI:95 Formula:C19H32O2 CAS:301-00-8 MolWeight:292 RetIndex:2101
CompName:9,12,15-Octadecatrienoic acid, methyl ester, (Z,Z,Z)- $$ Linolenic acid, methyl ester $$ Methyl all-cis-9,12,15-octadecatrienoate $$ Methyl lino

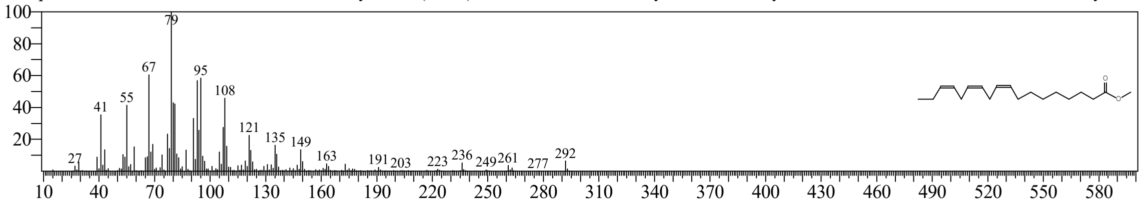


Hit#:2 Entry:124228 Library:NIST14.lib
SI:95 Formula:C19H32O2 CAS:301-00-8 MolWeight:292 RetIndex:2101
CompName:9,12,15-Octadecatrienoic acid, methyl ester, (Z,Z,Z)- $$ Linolenic acid, methyl ester $$ Methyl all-cis-9,12,15-octadecatrienoate $$ Methyl lino

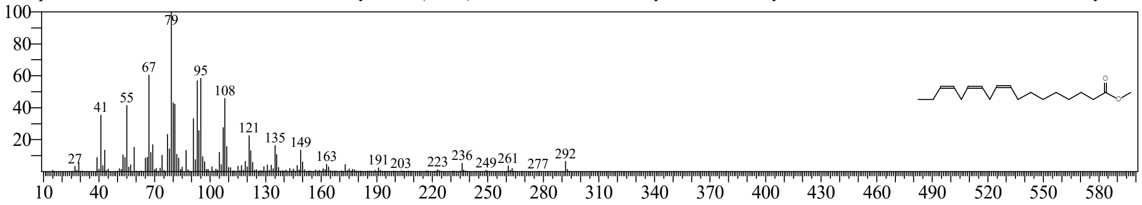


Hit#:3 Entry:27872 Library:NIST14s.lib
SI:95 Formula:C19H32O2 CAS:301-00-8 MolWeight:292 RetIndex:2101
CompName:9,12,15-Octadecatrienoic acid, methyl ester, (Z,Z,Z)- $$ Linolenic acid, methyl ester $$ Methyl all-cis-9,12,15-octadecatrienoate $$ Methyl lino

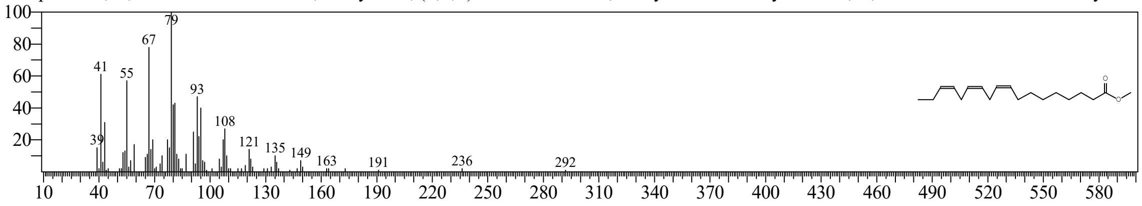


Hit#:4 Entry:32437 Library:NIST17s.lib
SI:95 Formula:C19H32O2 CAS:301-00-8 MolWeight:292 RetIndex:2101
CompName:9,12,15-Octadecatrienoic acid, methyl ester, (Z,Z,Z)- $$ Linolenic acid, methyl ester $$ Methyl all-cis-9,12,15-octadecatrienoate $$ Methyl lino

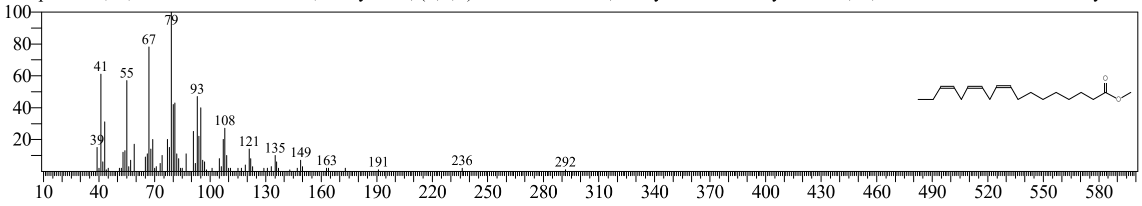


Hit#:5 Entry:111654 Library:NIST14.lib
SI:94 Formula:C18H30O2 CAS:0-00-0 MolWeight:278 RetIndex:2002
CompName:Methyl 8,11,14-heptadecatrienoate

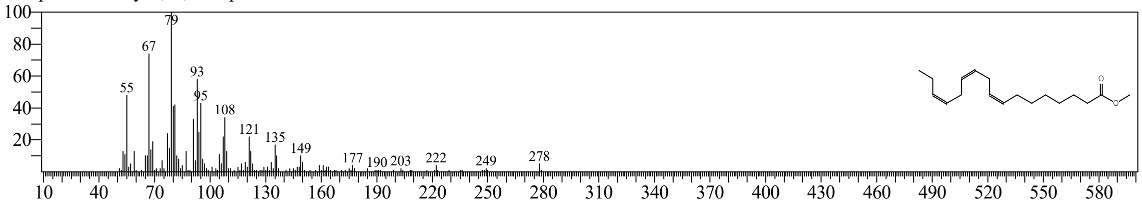

<< Target >>
Line#:16   R.Time:30.600(Scan#:2473)  MassPeaks:402
RawMode:Averaged 30.592-30.608(2472-2474) BasePeak:79.05(71729)
BG Mode:Calc. from Peak  Group 1 - Event 1

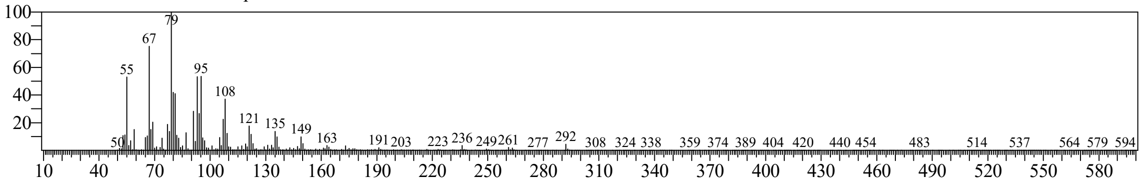


Hit#:1  Entry:124228  Library:NIST14.lib
SI:96  Formula:C19H32O2  CAS:301-00-8  MolWeight:292  RetIndex:2101
CompName:9,12,15-Octadecatrienoic acid, methyl ester, (Z,Z,Z)- $$ Linolenic acid, methyl ester $$ Methyl all-cis-9,12,15-octadecatrienoate $$ Methyl lino

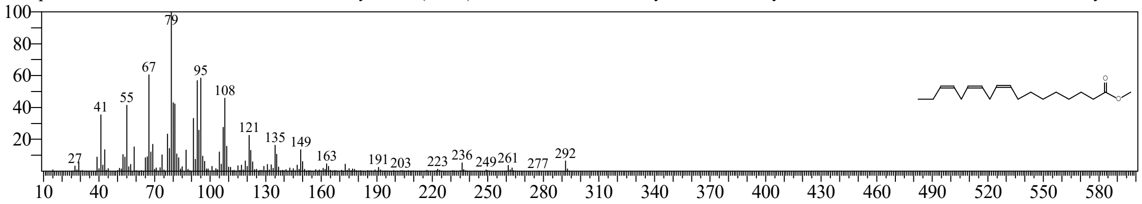


Hit#:2  Entry:135724  Library:NIST17-1.lib
SI:96  Formula:C19H32O2  CAS:301-00-8  MolWeight:292  RetIndex:2101
CompName:9,12,15-Octadecatrienoic acid, methyl ester, (Z,Z,Z)- $$ Linolenic acid, methyl ester $$ Methyl all-cis-9,12,15-octadecatrienoate $$ Methyl lino

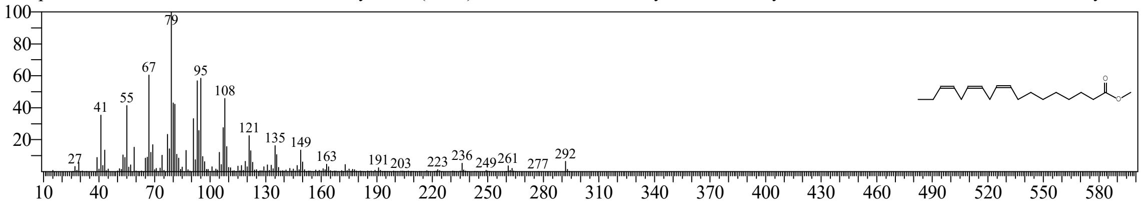


Hit#:3  Entry:32438  Library:NIST17s.lib
SI:95  Formula:C19H32O2  CAS:301-00-8  MolWeight:292  RetIndex:2101
CompName:9,12,15-Octadecatrienoic acid, methyl ester, (Z,Z,Z)- $$ Linolenic acid, methyl ester $$ Methyl all-cis-9,12,15-octadecatrienoate $$ Methyl lino

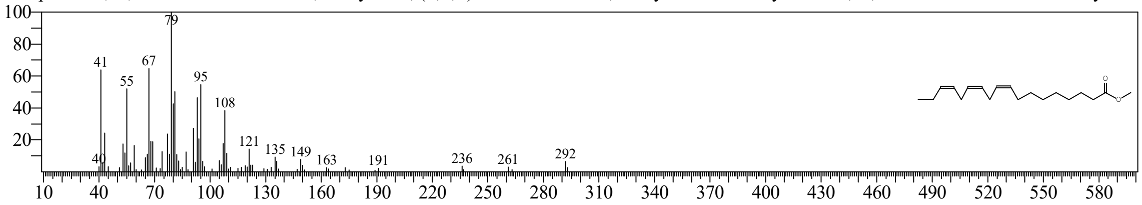


Hit#:4  Entry:27872  Library:NIST14s.lib
SI:95  Formula:C19H32O2  CAS:301-00-8  MolWeight:292  RetIndex:2101
CompName:9,12,15-Octadecatrienoic acid, methyl ester, (Z,Z,Z)- $$ Linolenic acid, methyl ester $$ Methyl all-cis-9,12,15-octadecatrienoate $$ Methyl lino

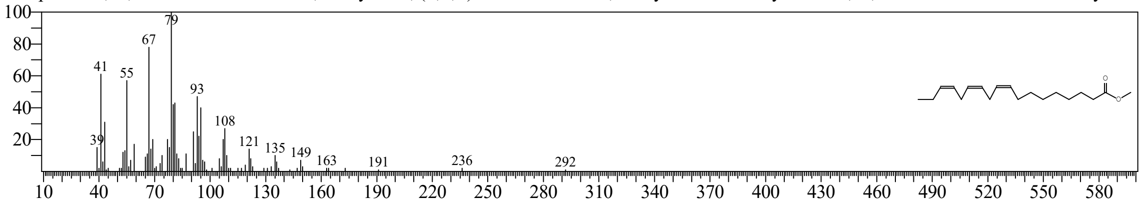


Hit#:5  Entry:111654  Library:NIST14.lib
SI:95  Formula:C18H30O2  CAS:0-00-0  MolWeight:278  RetIndex:2002
CompName:Methyl 8,11,14-heptadecatrienoate

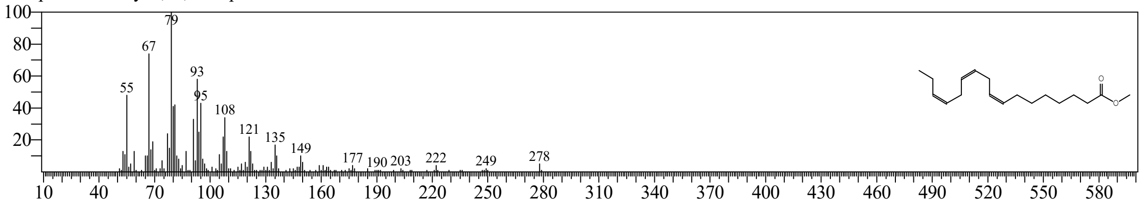

<< Target >>
Line#:17 R.Time:30.933(Scan#:2513) MassPeaks:372
RawMode:Averaged 30.925-30.942(2512-2514) BasePeak:67.00(6435)
BG Mode:Calc. from Peak Group 1 - Event 1

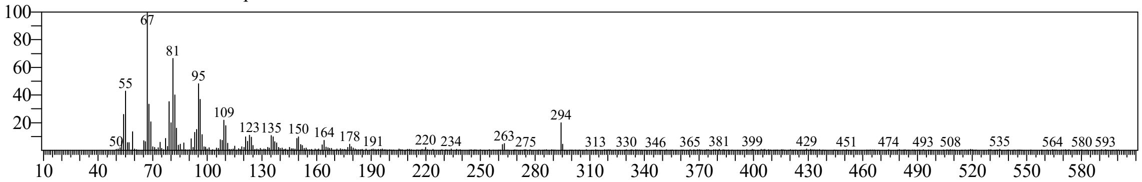


Hit#:1 Entry:137666 Library:NIST17-1.lib
SI:96 Formula:C19H34O2 CAS:13058-52-1 MolWeight:294 RetIndex:2093
CompName:Methyl 9-cis,11-trans-octadecadienoate

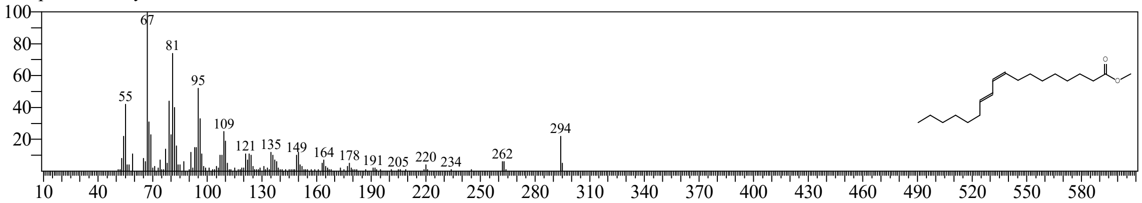


Hit#:2 Entry:125932 Library:NIST14.lib
SI:96 Formula:C19H34O2 CAS:0-00-0 MolWeight:294 RetIndex:2093
CompName:Methyl 9-cis,11-trans-octadecadienoate

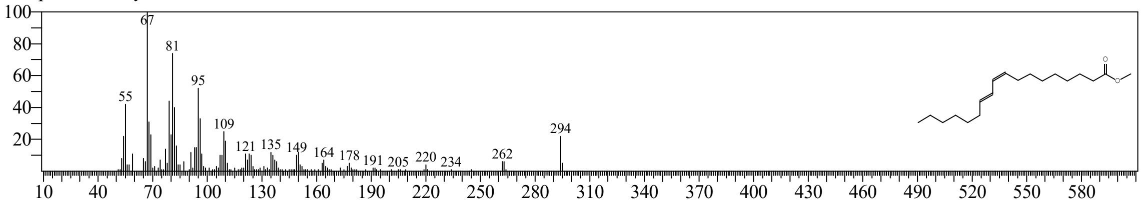


Hit#:3 Entry:125933 Library:NIST14.lib
SI:96 Formula:C19H34O2 CAS:0-00-0 MolWeight:294 RetIndex:2093
CompName:Methyl 10-trans,12-cis-octadecadienoate

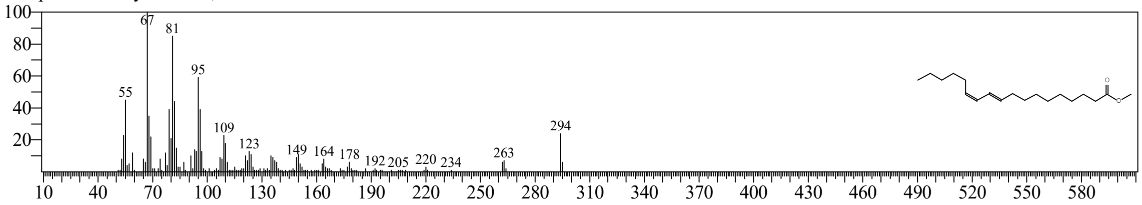


Hit#:4 Entry:137664 Library:NIST17-1.lib
SI:96 Formula:C19H34O2 CAS:0-00-0 MolWeight:294 RetIndex:2093
CompName:Methyl 10-trans,12-cis-octadecadienoate

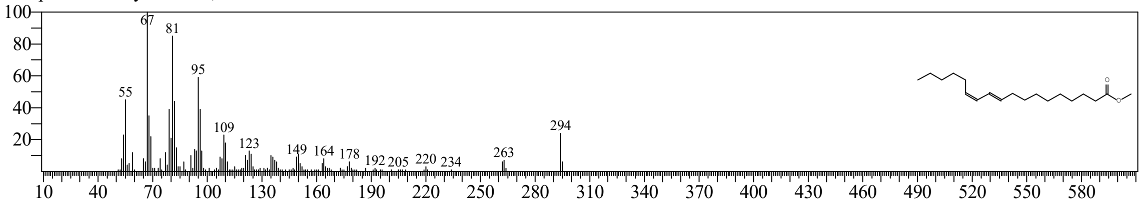


Hit#:5 Entry:32587 Library:NIST17s.lib
SI:95 Formula:C19H34O2 CAS:13038-47-6 MolWeight:294 RetIndex:2093
CompName:9,11-Octadecadienoic acid, methyl ester, (E,E)- $$ Methyl trans-9,trans-11-octadecadienoate $$ Methyl trans,trans-9,11-octadecadienoate $$ M

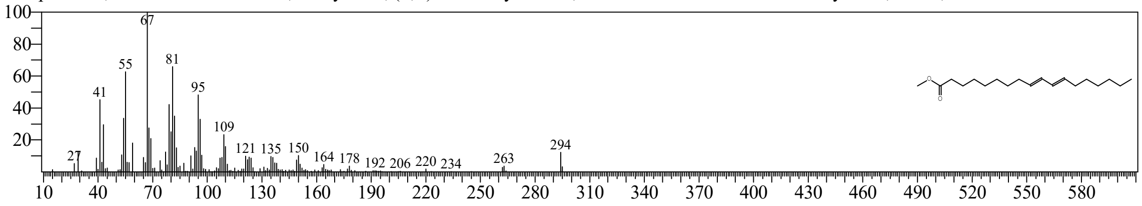

<< Target >>
Line#:18 R.Time:31.258(Scan#:2552) MassPeaks:376
RawMode:Averaged 31.250-31.267(2551-2553) BasePeak:67.05(6808)
BG Mode:Calc. from Peak Group 1 - Event 1

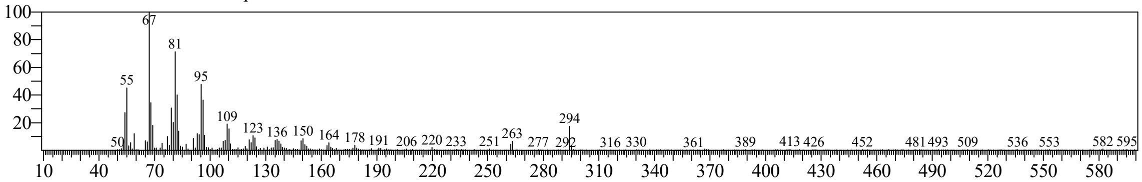


Hit#:1 Entry:125933 Library:NIST14.lib
SI:96 Formula:C19H34O2 CAS:0-00-0 MolWeight:294 RetIndex:2093
CompName:Methyl 10-trans,12-cis-octadecadienoate

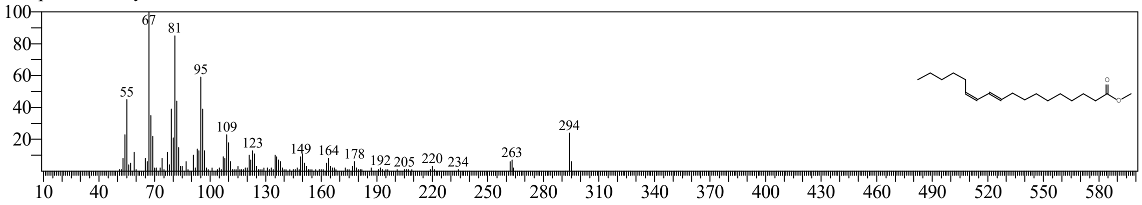


Hit#:2 Entry:137664 Library:NIST17-1.lib
SI:96 Formula:C19H34O2 CAS:0-00-0 MolWeight:294 RetIndex:2093
CompName:Methyl 10-trans,12-cis-octadecadienoate

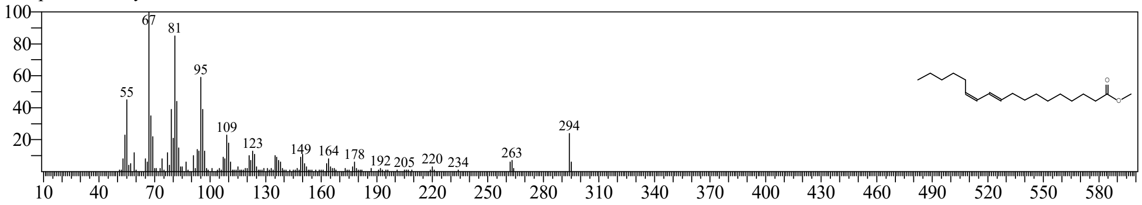


Hit#:3 Entry:125932 Library:NIST14.lib
SI:96 Formula:C19H34O2 CAS:0-00-0 MolWeight:294 RetIndex:2093
CompName:Methyl 9-cis,11-trans-octadecadienoate

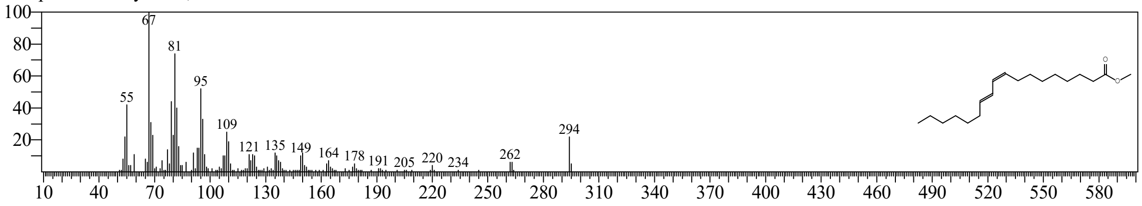


Hit#:4 Entry:137666 Library:NIST17-1.lib
SI:96 Formula:C19H34O2 CAS:13058-52-1 MolWeight:294 RetIndex:2093
CompName:Methyl 9-cis,11-trans-octadecadienoate

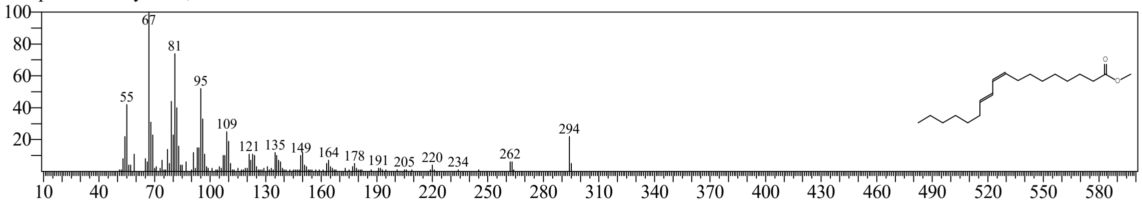


Hit#:5 Entry:32587 Library:NIST17s.lib
SI:95 Formula:C19H34O2 CAS:13038-47-6 MolWeight:294 RetIndex:2093
CompName:9,11-Octadecadienoic acid, methyl ester, (E,E)- $$ Methyl trans-9,trans-11-octadecadienoate $$ Methyl trans,trans-9,11-octadecadienoate $$ M

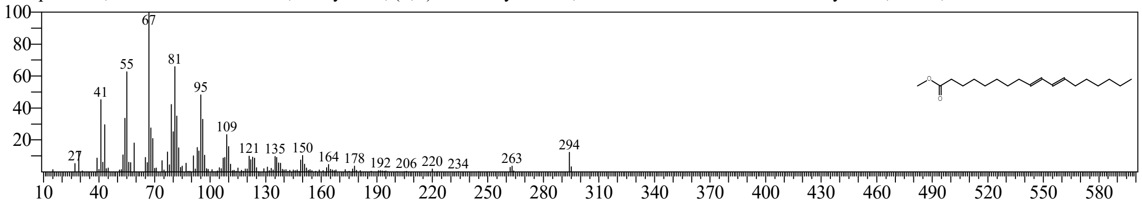

<< Target >>
Line#:19   R.Time:31.908(Scan#:2630)  MassPeaks:365
RawMode:Averaged 31.900-31.917(2629-2631) BasePeak:67.05(977)
BG Mode:Calc. from Peak  Group 1 - Event 1

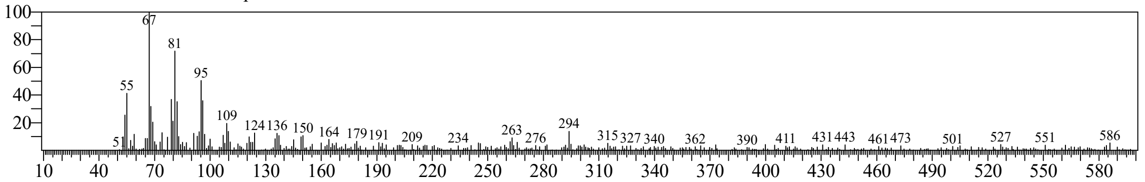


Hit#:1  Entry:125932  Library:NIST14.lib
SI:91  Formula:C19H34O2  CAS:0-00-0  MolWeight:294  RetIndex:2093
CompName:Methyl 9-cis,11-trans-octadecadienoate

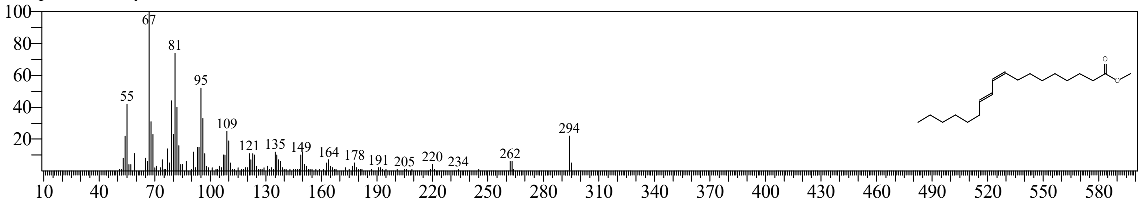


Hit#:2  Entry:137666  Library:NIST17-1.lib
SI:91  Formula:C19H34O2  CAS:13058-52-1  MolWeight:294  RetIndex:2093
CompName:Methyl 9-cis,11-trans-octadecadienoate

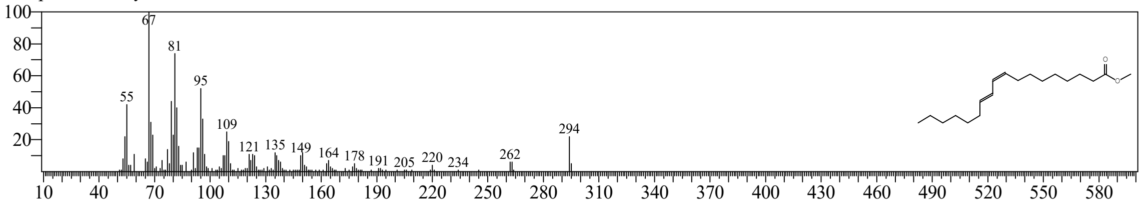


Hit#:3  Entry:137664  Library:NIST17-1.lib
SI:91  Formula:C19H34O2  CAS:0-00-0  MolWeight:294  RetIndex:2093
CompName:Methyl 10-trans,12-cis-octadecadienoate

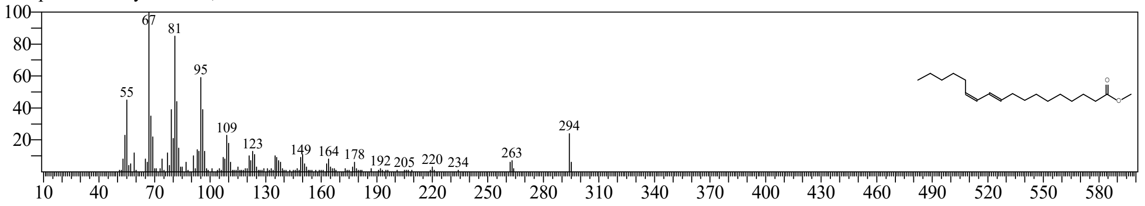


Hit#:4  Entry:125933  Library:NIST14.lib
SI:91  Formula:C19H34O2  CAS:0-00-0  MolWeight:294  RetIndex:2093
CompName:Methyl 10-trans,12-cis-octadecadienoate

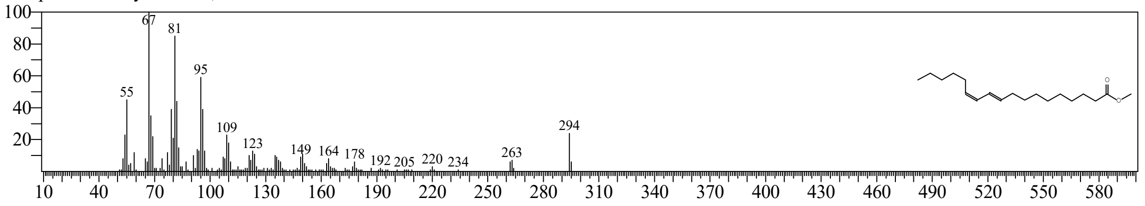


Hit#:5  Entry:32587  Library:NIST17s.lib
SI:90  Formula:C19H34O2  CAS:13038-47-6  MolWeight:294  RetIndex:2093
CompName:9,11-Octadecadienoic acid, methyl ester, (E,E)- $$ Methyl trans-9,trans-11-octadecadienoate $$ Methyl trans,trans-9,11-octadecadienoate $$ M

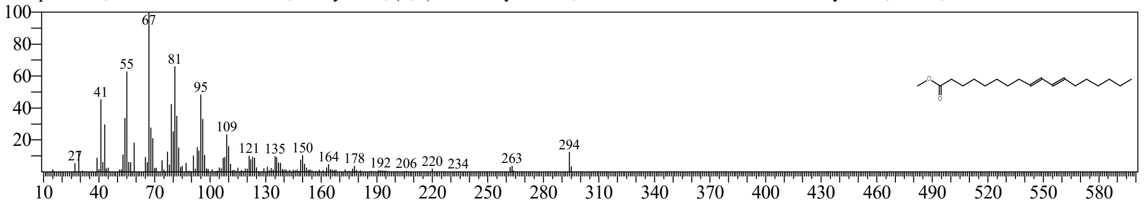

<< Target >>
Line#:20 R.Time:32.958(Scan#:2756) MassPeaks:313
RawMode:Averaged 32.950-32.967(2755-2757) BasePeak:95.05(7671)
BG Mode:Calc. from Peak Group 1 - Event 1

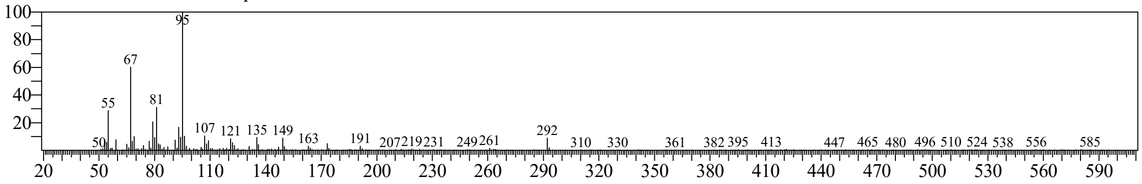


Hit#:1 Entry:24424 Library:NIST17-1.lib
SI:85 Formula:C12H20 CAS:0-00-0 MolWeight:164 RetIndex:1041
CompName:Cis-3-ethyl-endo-tricyclo[5.2.1.0(2.6)]decane

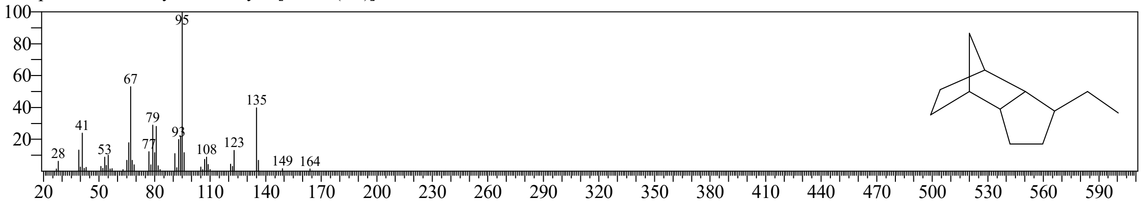


Hit#:2 Entry:23403 Library:NIST14.lib
SI:85 Formula:C12H20 CAS:0-00-0 MolWeight:164 RetIndex:1041
CompName:Cis-3-ethyl-endo-tricyclo[5.2.1.0(2.6)]decane

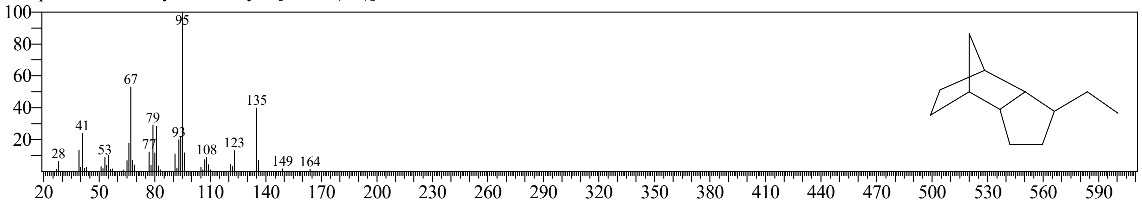


Hit#:3 Entry:16582 Library:NIST17-1.lib
SI:84 Formula:C11H18 CAS:0-00-0 MolWeight:150 RetIndex:1115
CompName:4-Cyclohexenyl-3-methylbutene

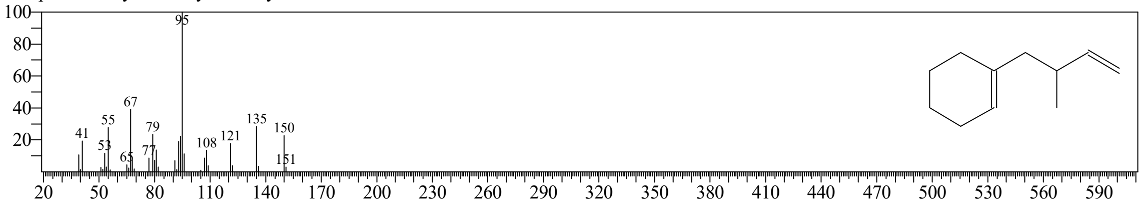


Hit#:4 Entry:16897 Library:NIST14.lib
SI:82 Formula:C10H16O CAS:60485-45-2 MolWeight:152 RetIndex:991
CompName:Santolina epoxide $$ 2-Methyl-2-(3-methyl-1-vinyl-2-butenyl)oxirane # $$

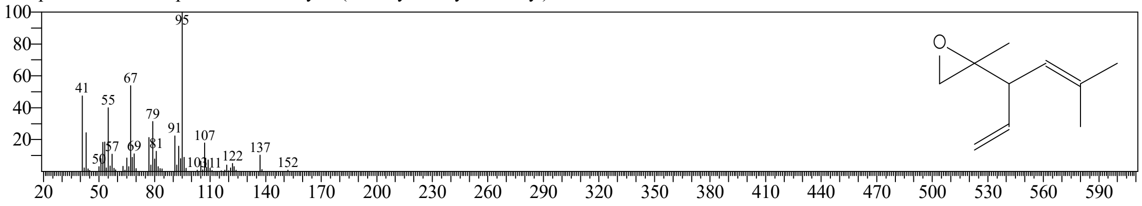


Hit#:5 Entry:17571 Library:NIST17-1.lib
SI:82 Formula:C10H16O CAS:60485-45-2 MolWeight:152 RetIndex:991
CompName:Santolina epoxide $$ 2-Methyl-2-(3-methyl-1-vinyl-2-butenyl)oxirane # $$

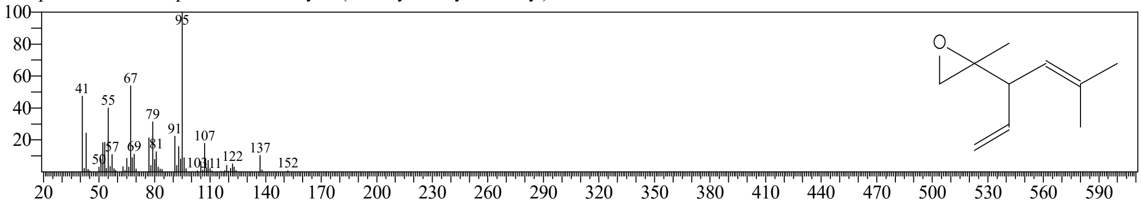

<< Target >>
Line#:21   R.Time:33.500(Scan#:2821)  MassPeaks:374
RawMode:Averaged 33.492-33.508(2820-2822) BasePeak:74.00(14818)
BG Mode:Calc. from Peak  Group 1 - Event 1

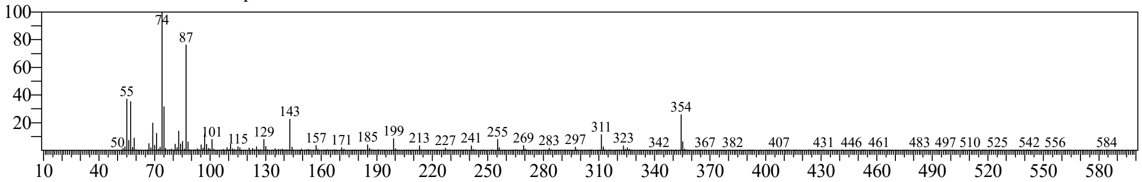


Hit#:1  Entry:178051  Library:NIST14.lib
SI:96  Formula:C23H46O2  CAS:929-77-1  MolWeight:354  RetIndex:2475
CompName:Docosanoic acid, methyl ester $$ Behenic acid, methyl ester $$ Methyl behenate $$ Methyl docosanoate $$ n-Docosanoic acid methyl ester $$

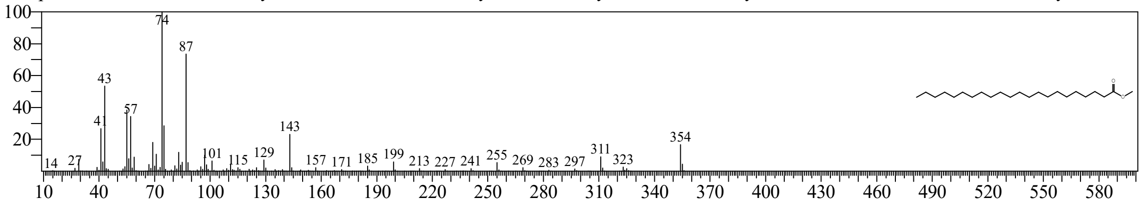


Hit#:2  Entry:196443  Library:NIST17-1.lib
SI:96  Formula:C23H46O2  CAS:929-77-1  MolWeight:354  RetIndex:2475
CompName:Docosanoic acid, methyl ester $$ Behenic acid, methyl ester $$ Methyl behenate $$ Methyl docosanoate $$ n-Docosanoic acid methyl ester $$

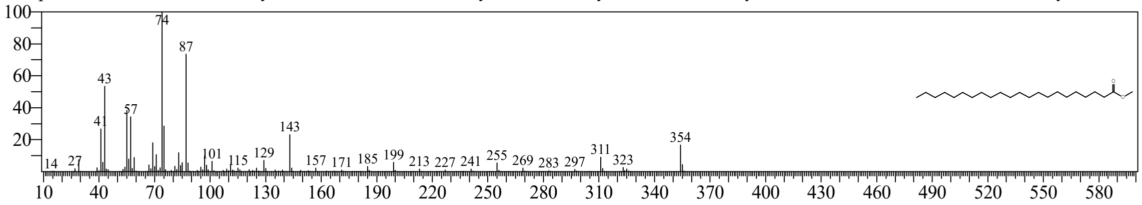


Hit#:3  Entry:178052  Library:NIST14.lib
SI:95  Formula:C23H46O2  CAS:0-00-0  MolWeight:354  RetIndex:2411
CompName:Methyl 20-methyl-heneicosanoate

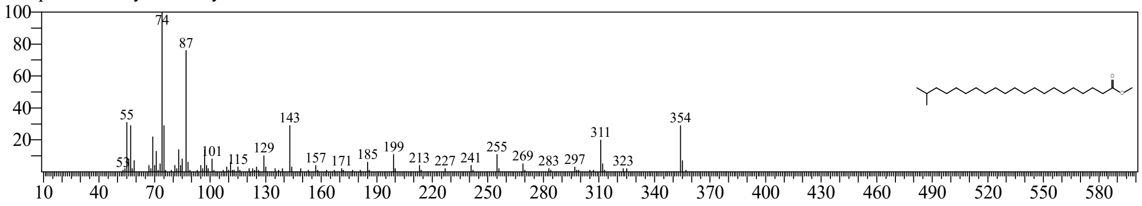


Hit#:4  Entry:196444  Library:NIST17-1.lib
SI:95  Formula:C23H46O2  CAS:0-00-0  MolWeight:354  RetIndex:2411
CompName:Methyl 20-methyl-heneicosanoate

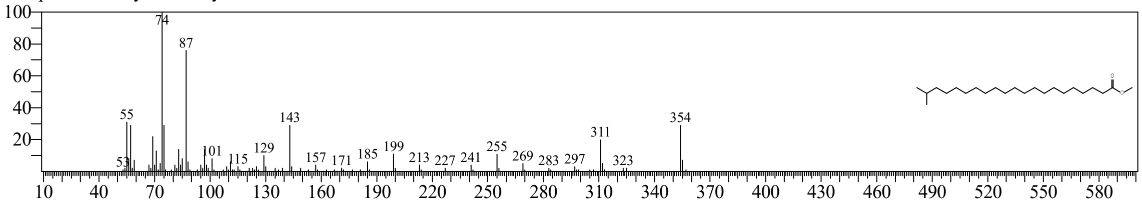


Hit#:5  Entry:31088  Library:NIST14s.lib
SI:95  Formula:C23H46O2  CAS:929-77-1  MolWeight:354  RetIndex:2475
CompName:Docosanoic acid, methyl ester $$ Behenic acid, methyl ester $$ Methyl behenate $$ Methyl docosanoate $$ n-Docosanoic acid methyl ester $$

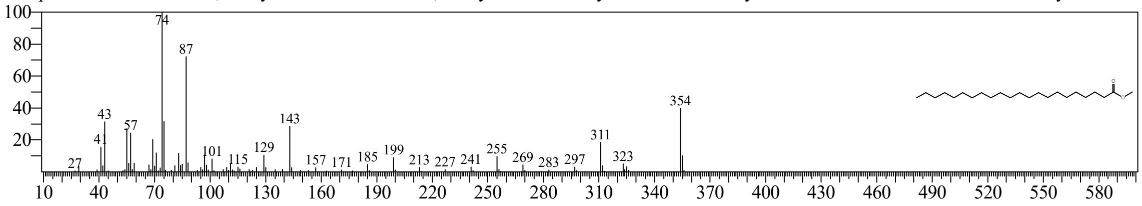

<< Target >>
Line#:22 R.Time:37.817(Scan#:3339) MassPeaks:350
RawMode:Averaged 37.808-37.825(3338-3340) BasePeak:74.05(3452)
BG Mode:Calc. from Peak Group 1 - Event 1

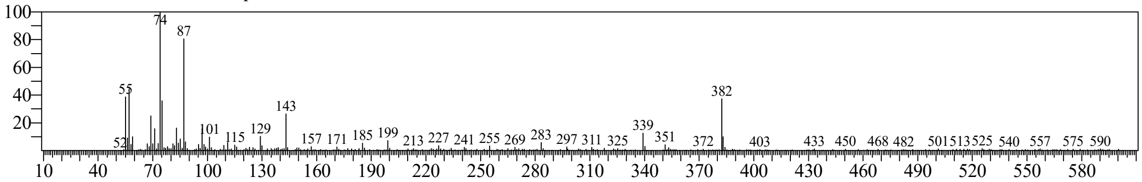


Hit#:1 Entry:31970 Library:NIST14s.lib
SI:94 Formula:C25H50O2 CAS:2442-49-1 MolWeight:382 RetIndex:2674
CompName:Tetracosanoic acid, methyl ester $$ Methyl lignocerate $$ Methyl tetracosanoate $$ Lignoceric acid methyl ester $$

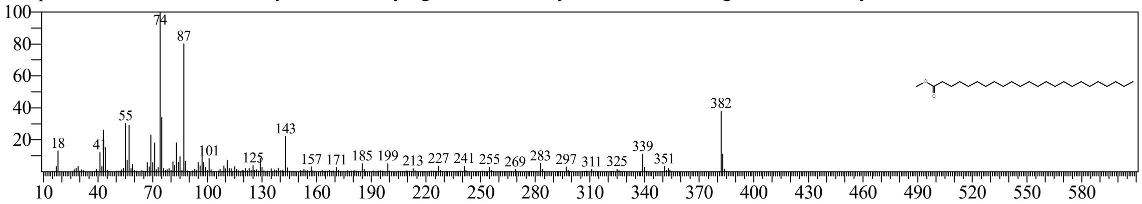


Hit#:2 Entry:37106 Library:NIST17s.lib
SI:94 Formula:C25H50O2 CAS:2442-49-1 MolWeight:382 RetIndex:2674
CompName:Tetracosanoic acid, methyl ester $$ Methyl lignocerate $$ Methyl tetracosanoate $$ Lignoceric acid methyl ester $$

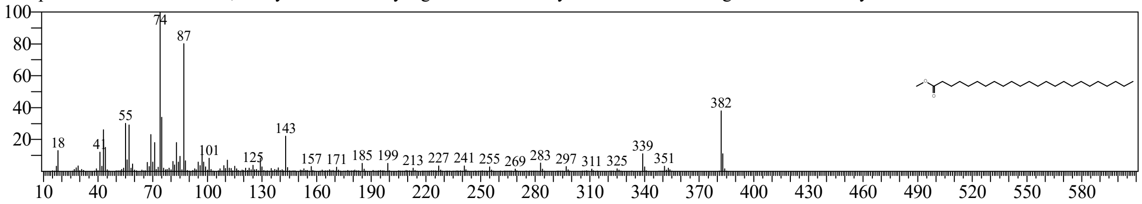


Hit#:3 Entry:31971 Library:NIST14s.lib
SI:93 Formula:C25H50O2 CAS:2442-49-1 MolWeight:382 RetIndex:2674
CompName:Tetracosanoic acid, methyl ester $$ Methyl lignocerate $$ Methyl tetracosanoate $$ Lignoceric acid methyl ester $$

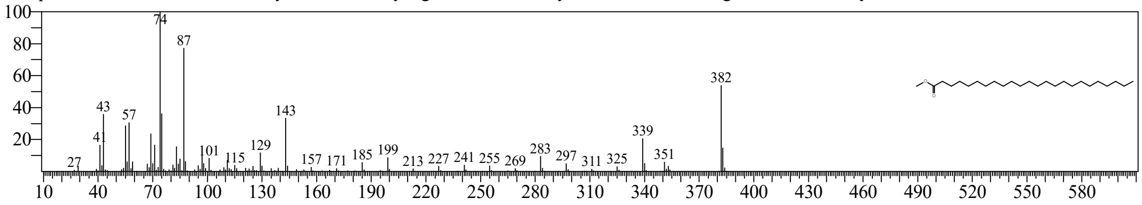


Hit#:4 Entry:37107 Library:NIST17s.lib
SI:93 Formula:C25H50O2 CAS:2442-49-1 MolWeight:382 RetIndex:2674
CompName:Tetracosanoic acid, methyl ester $$ Methyl lignocerate $$ Methyl tetracosanoate $$ Lignoceric acid methyl ester $$

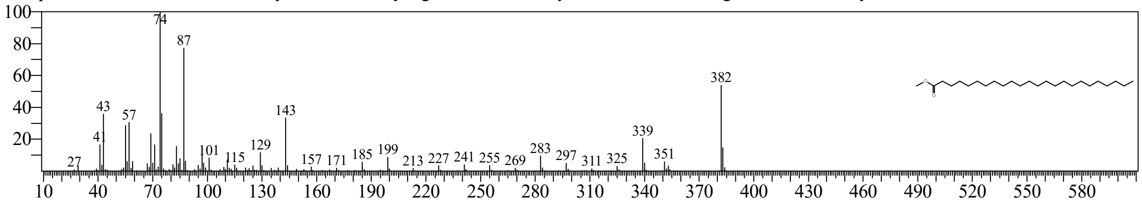


Hit#:5 Entry:37105 Library:NIST17s.lib
SI:90 Formula:C25H50O2 CAS:2442-49-1 MolWeight:382 RetIndex:2674
CompName:Tetracosanoic acid, methyl ester $$ Methyl lignocerate $$ Methyl tetracosanoate $$ Lignoceric acid methyl ester $$

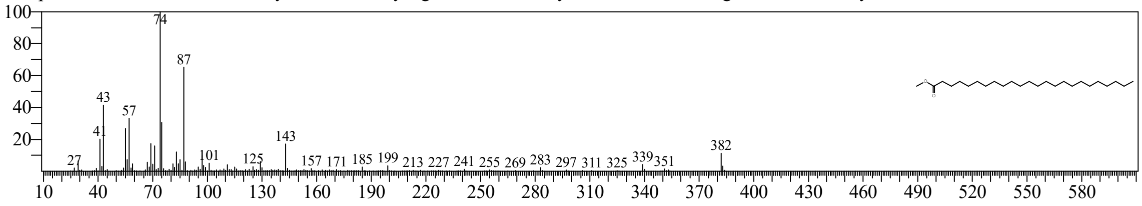

Supplement: Supplementary file 1 [file molecules-31-02891-s001.zip › 0d-QG100-1.pdf]
