# Supplementary material for: Selective Inhibition of Lipid Oxidation by a Quercetagetin-Rich Extract from Tagetes erecta: A Chemometric Approach Using OPLS-DA and Selectivity Index Analysis
Source: Molecules. 2026 Aug 19;31(16):2891. doi: 10.3390/molecules31162891 (PMC13515886; doi:10.3390/molecules31162891)

# QG100-2-0d

<<Column>>
Name : HP-88
Thickness : 0.20um
Length : 100.0m
Inside Diameter : 0.25mm

## Method

===== Analytical Line 1 =====

| | | |
|---|---|---|
| Column Oven Temp. | :120.0 °C | |
| Injection Temp. | :250.00 °C | |
| Injection Mode | :Split | |
| Column Flow | :1.04 mL/min | |
| Split Ratio | :20.0 | |
| Oven Temp. Program | | |
| Rate | Temperature(°C) | Hold Time(min) |
| - | 120.0 | 1.00 |
| 10.00 | 175.0 | 10.00 |
| 5.00 | 210.0 | 5.00 |
| 5.00 | 230.0 | 15.00 |

[GCMS-QP2010]
IonSourceTemp :200.00 °C
Interface Temp. :220.00 °C
*m/z* scanning range：45～500m/z

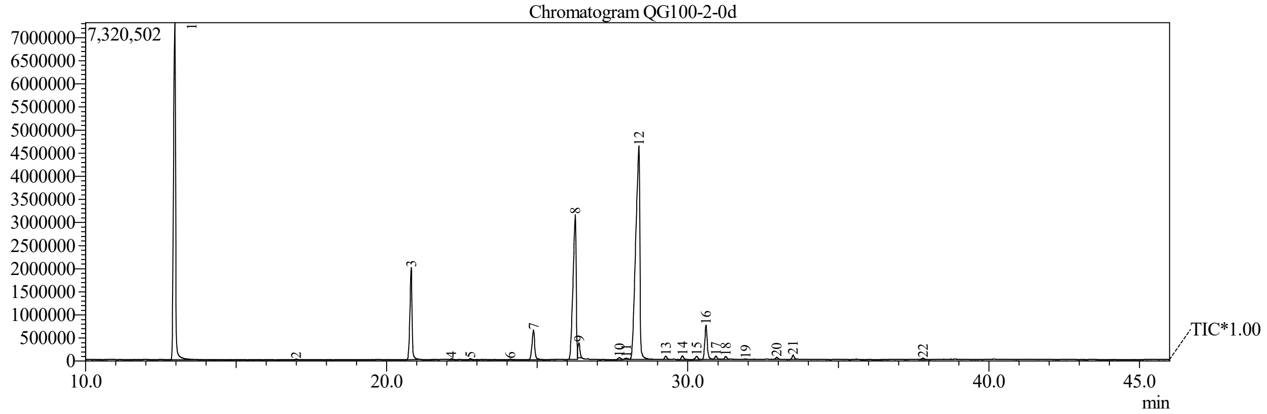


Peak Report TIC

| Peak# | R.Time | Area | Area% | Height | Height% | A/H | Name |
|---|---|---|---|---|---|---|---|
| 1 | 12.967 | 35838193 | 28.49 | 7288622 | 37.53 | 4.92 | Undecanoic acid, methyl ester |
| 2 | 16.996 | 64220 | 0.05 | 15187 | 0.08 | 4.23 | Methyl tetradecanoate |
| 3 | 20.817 | 9859242 | 7.84 | 1993402 | 10.26 | 4.95 | Hexadecanoic acid, methyl ester |
| 4 | 22.150 | 79562 | 0.06 | 16396 | 0.08 | 4.85 | 9-Hexadecenoic acid, methyl ester, (Z)- |
| 5 | 22.777 | 89556 | 0.07 | 18327 | 0.09 | 4.89 | Heptadecanoic acid, methyl ester |
| 6 | 24.102 | 49760 | 0.04 | 10951 | 0.06 | 4.54 | cis-10-Heptadecenoic acid, methyl ester |
| 7 | 24.876 | 4012352 | 3.19 | 645049 | 3.32 | 6.22 | Methyl stearate |
| 8 | 26.264 | 22707932 | 18.05 | 3136671 | 16.15 | 7.24 | 9-Octadecenoic acid, methyl ester, (E)- |
| 9 | 26.385 | 1442807 | 1.15 | 327474 | 1.69 | 4.41 | 11-Octadecenoic acid, methyl ester |
| 10 | 27.733 | 308660 | 0.25 | 41503 | 0.21 | 7.44 | 9,12-Octadecadienoic acid, methyl ester |
| 11 | 27.958 | 193395 | 0.15 | 27650 | 0.14 | 6.99 | 9,12-Octadecadienoic acid (Z,Z)-, methyl ester |
| 12 | 28.379 | 43879635 | 34.88 | 4617656 | 23.78 | 9.50 | 9,12-Octadecadienoic acid (Z,Z)-, methyl ester |
| 13 | 29.271 | 369282 | 0.29 | 71604 | 0.37 | 5.16 | Methyl 18-methylnonadecanoate |
| 14 | 29.821 | 453227 | 0.36 | 78855 | 0.41 | 5.75 | 9,12,15-Octadecatrienoic acid, methyl ester, (Z |

| Peak# | R.Time | Area | Area% | Height | Height% | A/H | Name |
|---|---|---|---|---|---|---|---|
| 15 | 30.298 | 446092 | 0.35 | 71068 | 0.37 | 6.28 | 9,12,15-Octadecatrienoic acid, methyl ester, (Z |
| 16 | 30.606 | 4308825 | 3.43 | 745608 | 3.84 | 5.78 | 9,12,15-Octadecatrienoic acid, methyl ester, (Z |
| 17 | 30.935 | 344184 | 0.27 | 64632 | 0.33 | 5.33 | Methyl 9-cis,11-trans-octadecadienoate |
| 18 | 31.263 | 315015 | 0.25 | 61255 | 0.32 | 5.14 | Methyl 10-trans,12-cis-octadecadienoate |
| 19 | 31.915 | 55999 | 0.04 | 12524 | 0.06 | 4.47 | Methyl 9-cis,11-trans-octadecadienoate |
| 20 | 32.953 | 262230 | 0.21 | 48030 | 0.25 | 5.46 | Cis-3-ethyl-endo-tricyclo[5.2.1.0(2.6)]decane |
| 21 | 33.498 | 510893 | 0.41 | 98343 | 0.51 | 5.20 | Docosanoic acid, methyl ester |
| 22 | 37.813 | 201003 | 0.16 | 30591 | 0.16 | 6.57 | Tetracosanoic acid, methyl ester |
| | | 125792064 | 100.00 | 19421398 | 100.00 | | |

Library

<< Target >>
Line#:1 R.Time:12.967(Scan#:357) MassPeaks:347
RawMode:Averaged 12.958-12.975(356-358) BasePeak:74.05(2269765)
BG Mode:Calc. from Peak Group 1 - Event 1

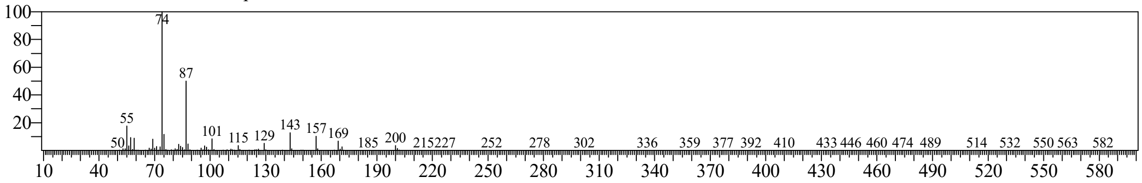


Hit#:1 Entry:21619 Library:NIST17s.lib
SI:95 Formula:C12H24O2 CAS:1731-86-8 MolWeight:200 RetIndex:1381
CompName:Undecanoic acid, methyl ester $$ Methyl undecanoate $$ Methyl undecylenate $$ n-Undecanoic acid methyl ester $$ Methyl ester of undecanoi

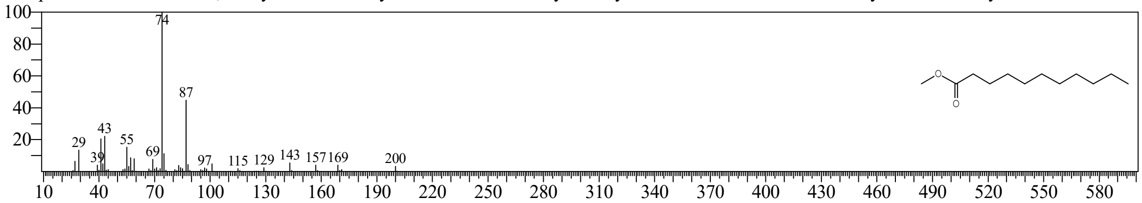


Hit#:2 Entry:18487 Library:NIST14s.lib
SI:95 Formula:C12H24O2 CAS:1731-86-8 MolWeight:200 RetIndex:1381
CompName:Undecanoic acid, methyl ester $$ Methyl undecanoate $$ Methyl undecylenate $$ n-Undecanoic acid methyl ester $$ Methyl ester of undecanoi

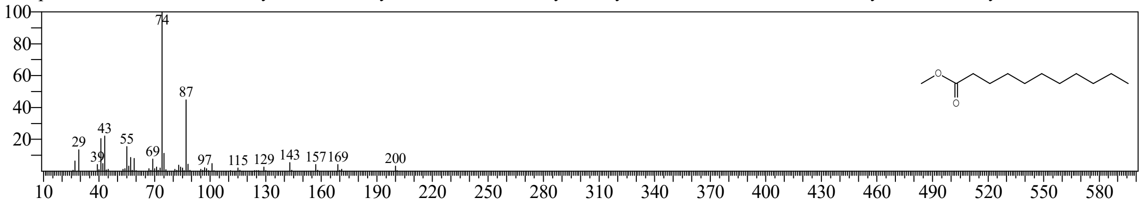


Hit#:3 Entry:18488 Library:NIST14s.lib
SI:95 Formula:C12H24O2 CAS:1731-86-8 MolWeight:200 RetIndex:1381
CompName:Undecanoic acid, methyl ester $$ Methyl undecanoate $$ Methyl undecylenate $$ n-Undecanoic acid methyl ester $$ Methyl ester of undecanoi

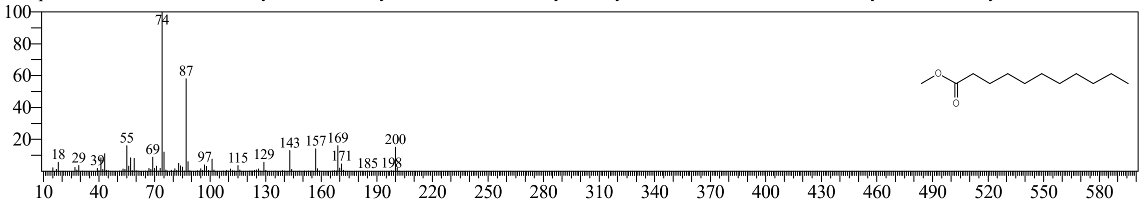


Hit#:4 Entry:21620 Library:NIST17s.lib
SI:95 Formula:C12H24O2 CAS:1731-86-8 MolWeight:200 RetIndex:1381
CompName:Undecanoic acid, methyl ester $$ Methyl undecanoate $$ Methyl undecylenate $$ n-Undecanoic acid methyl ester $$ Methyl ester of undecanoi

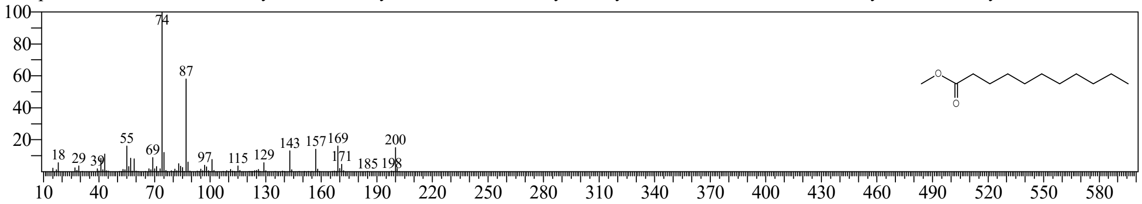

<< Target >>
Line#:1 R.Time:12.967(Scan#:357) MassPeaks:347
RawMode:Averaged 12.958-12.975(356-358) BasePeak:74.05(2269765)
BG Mode:Calc. from Peak Group 1 - Event 1

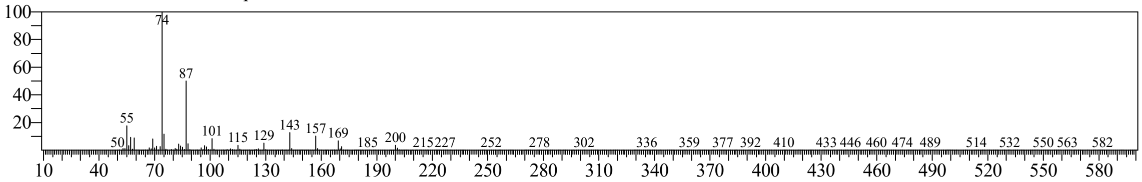


Hit#:5 Entry:46966 Library:NIST14.lib
SI:95 Formula:C12H24O2 CAS:1731-86-8 MolWeight:200 RetIndex:1381
CompName:Undecanoic acid, methyl ester $$ Methyl undecanoate $$ Methyl undecylenate $$ n-Undecanoic acid methyl ester $$ Methyl ester of undecanoi

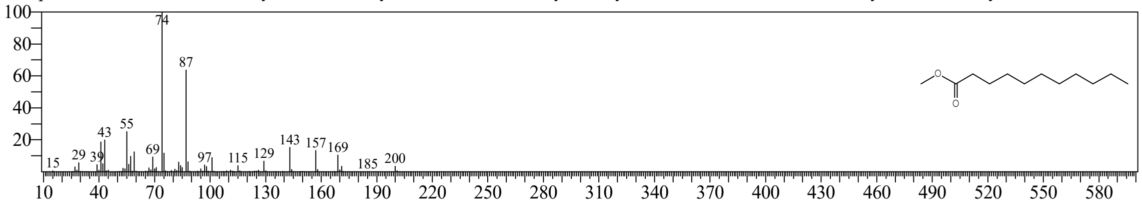

<< Target >>
Line#:2 R.Time:17.000(Scan#:841) MassPeaks:289
RawMode:Averaged 16.992-17.008(840-842) BasePeak:74.00(3562)
BG Mode:Calc. from Peak Group 1 - Event 1

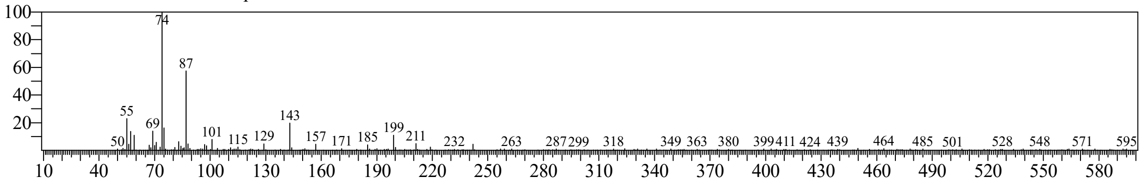


Hit#:1 Entry:86786 Library:NIST17-1.lib
SI:93 Formula:C15H30O2 CAS:124-10-7 MolWeight:242 RetIndex:1680
CompName:Methyl tetradecanoate $$ Tetradecanoic acid, methyl ester $$ Myristic acid, methyl ester $$ Metholeneat 2495 $$ Methyl myristate $$ Methyl r

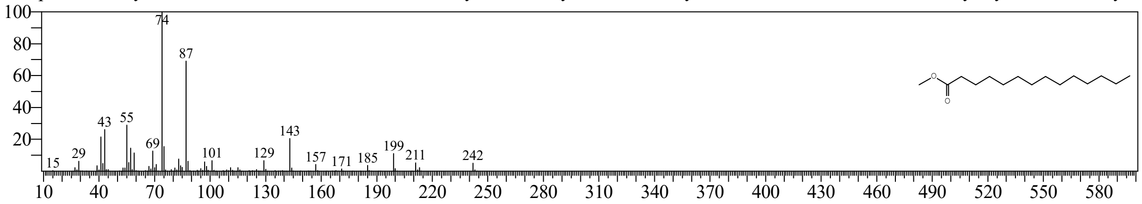


Hit#:2 Entry:80645 Library:NIST14.lib
SI:93 Formula:C15H30O2 CAS:124-10-7 MolWeight:242 RetIndex:1680
CompName:Methyl tetradecanoate $$ Tetradecanoic acid, methyl ester $$ Myristic acid, methyl ester $$ Metholeneat 2495 $$ Methyl myristate $$ Methyl r

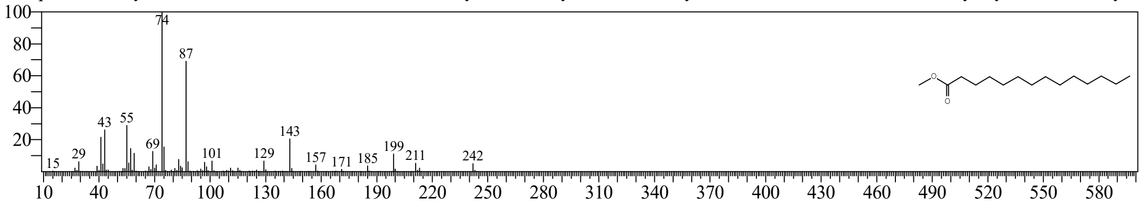


Hit#:3 Entry:27874 Library:NIST17s.lib
SI:93 Formula:C15H30O2 CAS:124-10-7 MolWeight:242 RetIndex:1680
CompName:Methyl tetradecanoate $$ Tetradecanoic acid, methyl ester $$ Myristic acid, methyl ester $$ Metholeneat 2495 $$ Methyl myristate $$ Methyl r

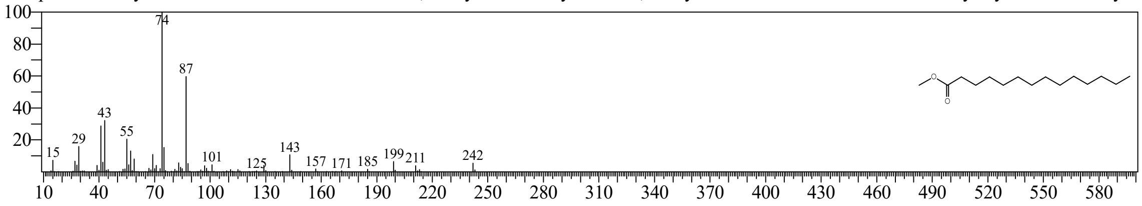


Hit#:4 Entry:23848 Library:NIST14s.lib
SI:93 Formula:C15H30O2 CAS:124-10-7 MolWeight:242 RetIndex:1680
CompName:Methyl tetradecanoate $$ Tetradecanoic acid, methyl ester $$ Myristic acid, methyl ester $$ Metholeneat 2495 $$ Methyl myristate $$ Methyl r

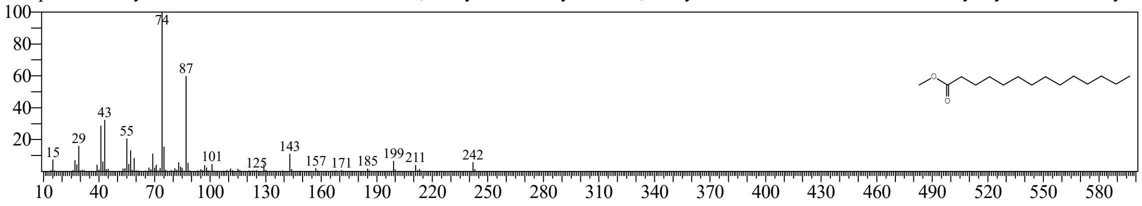


Hit#:5 Entry:27873 Library:NIST17s.lib
SI:92 Formula:C15H30O2 CAS:124-10-7 MolWeight:242 RetIndex:1680
CompName:Methyl tetradecanoate $$ Tetradecanoic acid, methyl ester $$ Myristic acid, methyl ester $$ Metholeneat 2495 $$ Methyl myristate $$ Methyl r

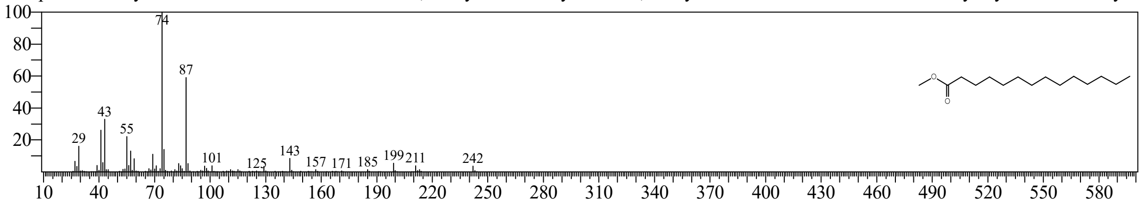

<< Target >>
Line#:3  R.Time:20.817(Scan#:1299)  MassPeaks:373
RawMode:Averaged 20.808-20.825(1298-1300) BasePeak:74.00(472151)
BG Mode:Calc. from Peak  Group 1 - Event 1

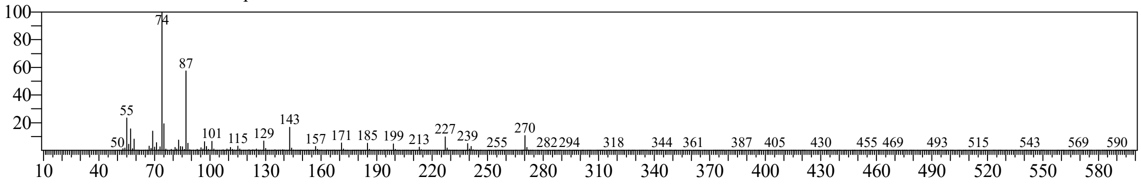


Hit#:1  Entry:26273  Library:NIST14s.lib
SI:97  Formula:C17H34O2  CAS:112-39-0  MolWeight:270  RetIndex:1878
CompName:Hexadecanoic acid, methyl ester $$ Palmitic acid, methyl ester $$ n-Hexadecanoic acid methyl ester $$ Metholene 2216 $$ Methyl hexadecano

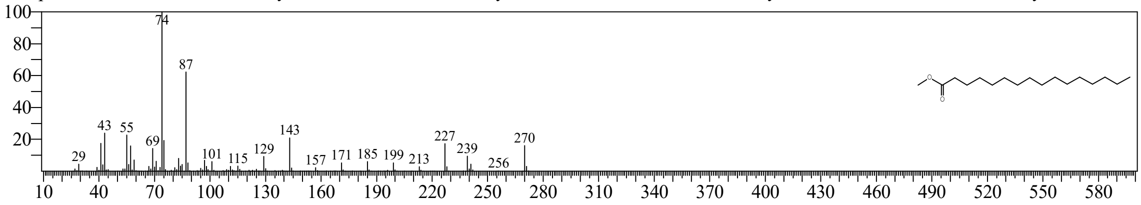


Hit#:2  Entry:104648  Library:NIST14.lib
SI:96  Formula:C17H34O2  CAS:112-39-0  MolWeight:270  RetIndex:1878
CompName:Hexadecanoic acid, methyl ester $$ Palmitic acid, methyl ester $$ n-Hexadecanoic acid methyl ester $$ Metholene 2216 $$ Methyl hexadecano

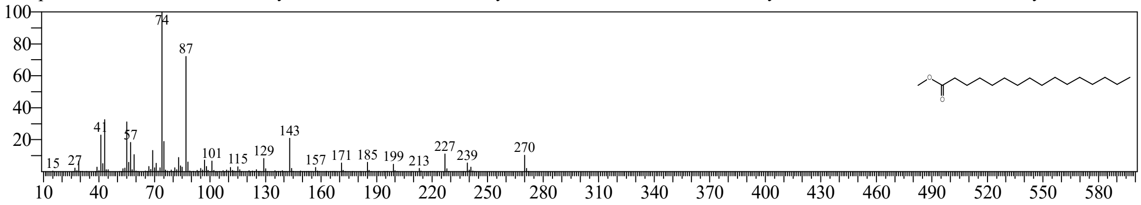


Hit#:3  Entry:113663  Library:NIST17-1.lib
SI:96  Formula:C17H34O2  CAS:112-39-0  MolWeight:270  RetIndex:1878
CompName:Hexadecanoic acid, methyl ester $$ Palmitic acid, methyl ester $$ n-Hexadecanoic acid methyl ester $$ Metholene 2216 $$ Methyl hexadecano

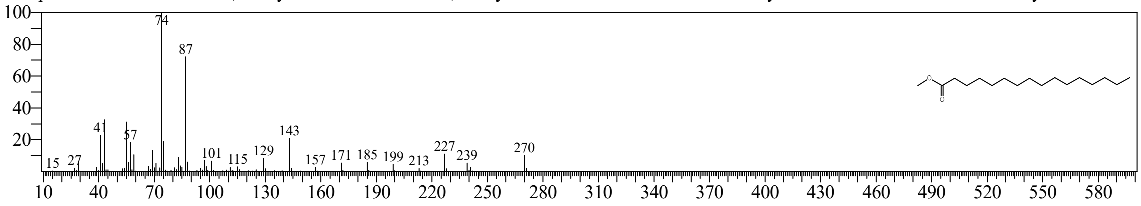


Hit#:4  Entry:26269  Library:NIST14s.lib
SI:96  Formula:C17H34O2  CAS:112-39-0  MolWeight:270  RetIndex:1878
CompName:Hexadecanoic acid, methyl ester $$ Palmitic acid, methyl ester $$ n-Hexadecanoic acid methyl ester $$ Metholene 2216 $$ Methyl hexadecano

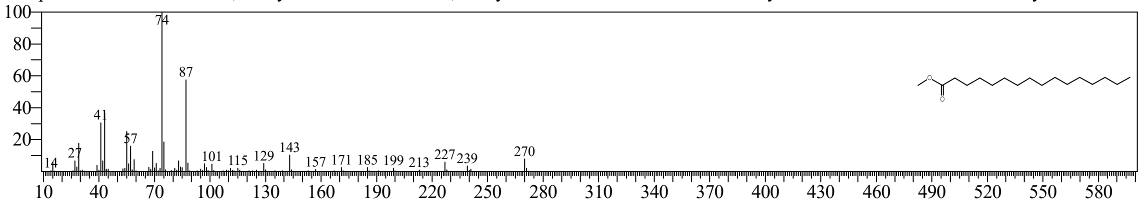


Hit#:5  Entry:30644  Library:NIST17s.lib
SI:96  Formula:C17H34O2  CAS:112-39-0  MolWeight:270  RetIndex:1878
CompName:Hexadecanoic acid, methyl ester $$ Palmitic acid, methyl ester $$ n-Hexadecanoic acid methyl ester $$ Metholene 2216 $$ Methyl hexadecano

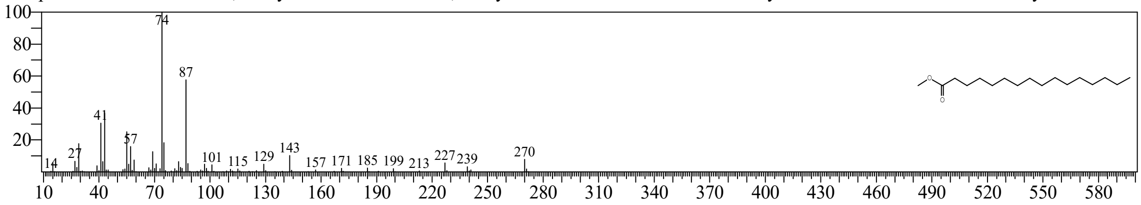

<< Target >>
Line#:4  R.Time:22.150(Scan#:1459)  MassPeaks:303
RawMode:Averaged 22.142-22.158(1458-1460) BasePeak:55.00(1323)
BG Mode:Calc. from Peak  Group 1 - Event 1

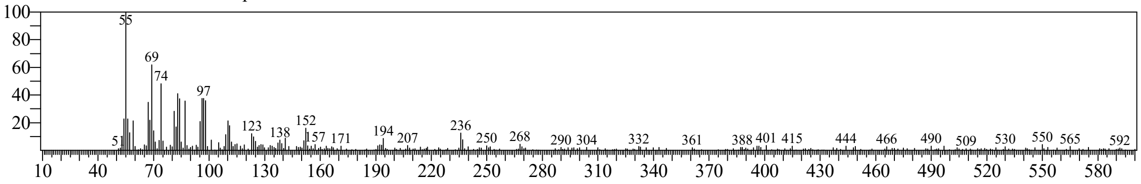


Hit#:1  Entry:30415  Library:NIST17s.lib
SI:93  Formula:C17H32O2  CAS:1120-25-8  MolWeight:268  RetIndex:1886
CompName:9-Hexadecenoic acid, methyl ester, (Z)- $$ Methyl palmitoleate $$ Methyl palmitoleinate $$ Palmitoleic acid, methyl ester $$ Methyl (9Z)-9-he

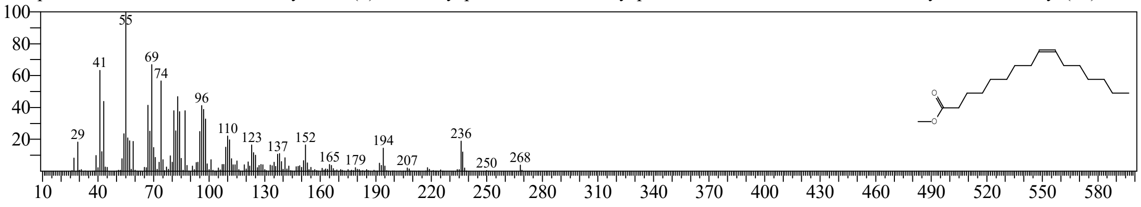


Hit#:2  Entry:26066  Library:NIST14s.lib
SI:93  Formula:C17H32O2  CAS:1120-25-8  MolWeight:268  RetIndex:1886
CompName:9-Hexadecenoic acid, methyl ester, (Z)- $$ Methyl palmitoleate $$ Methyl palmitoleinate $$ Palmitoleic acid, methyl ester $$ Methyl (9Z)-9-he

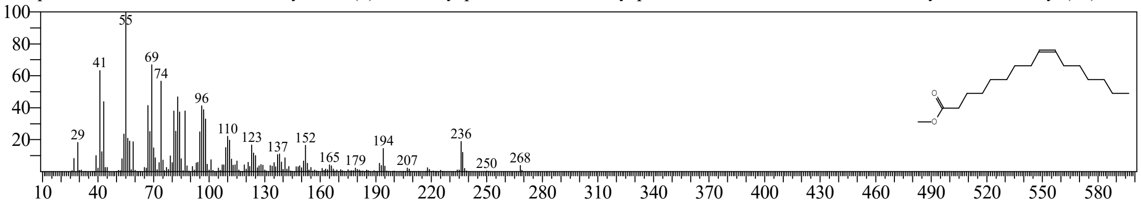


Hit#:3  Entry:111510  Library:NIST17-1.lib
SI:92  Formula:C17H32O2  CAS:56875-67-3  MolWeight:268  RetIndex:1886
CompName:7-Hexadecenoic acid, methyl ester, (Z)- $$ Methyl (7E)-7-hexadecenoate # $$

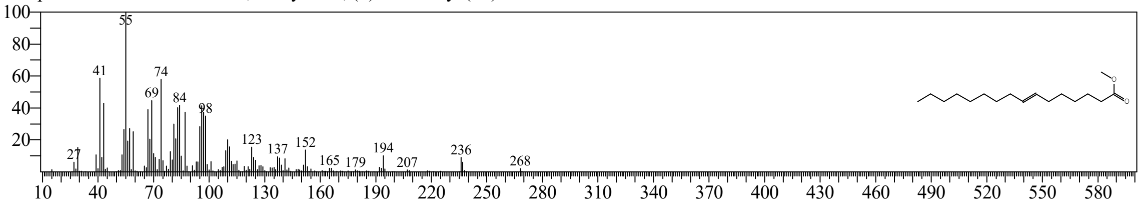


Hit#:4  Entry:30413  Library:NIST17s.lib
SI:92  Formula:C17H32O2  CAS:1120-25-8  MolWeight:268  RetIndex:1886
CompName:9-Hexadecenoic acid, methyl ester, (Z)- $$ Methyl palmitoleate $$ Methyl palmitoleinate $$ Palmitoleic acid, methyl ester $$ Methyl (9Z)-9-he

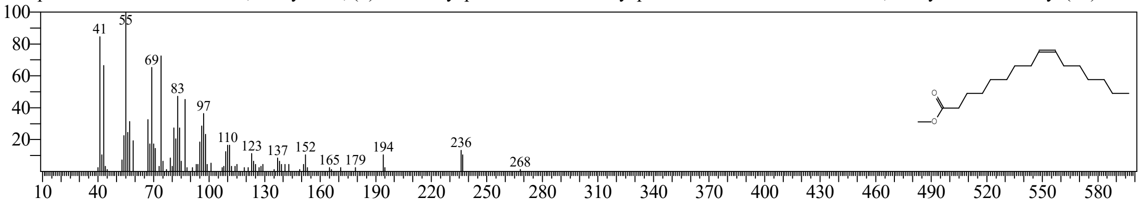


Hit#:5  Entry:26064  Library:NIST14s.lib
SI:92  Formula:C17H32O2  CAS:1120-25-8  MolWeight:268  RetIndex:1886
CompName:9-Hexadecenoic acid, methyl ester, (Z)- $$ Methyl palmitoleate $$ Methyl palmitoleinate $$ Palmitoleic acid, methyl ester $$ Methyl (9Z)-9-he

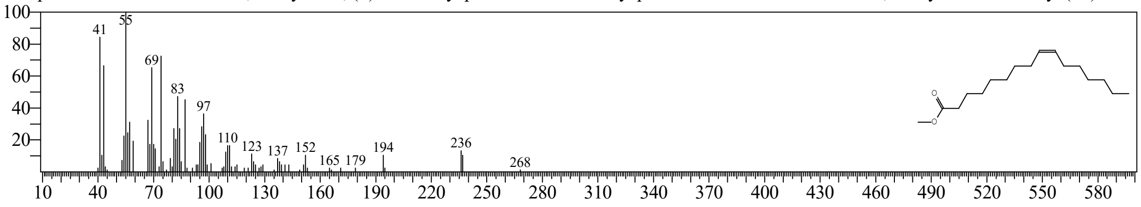

<< Target >>
Line#:5  R.Time:22.775(Scan#:1534)  MassPeaks:334
RawMode:Averaged 22.767-22.783(1533-1535) BasePeak:74.05(3385)
BG Mode:Calc. from Peak  Group 1 - Event 1

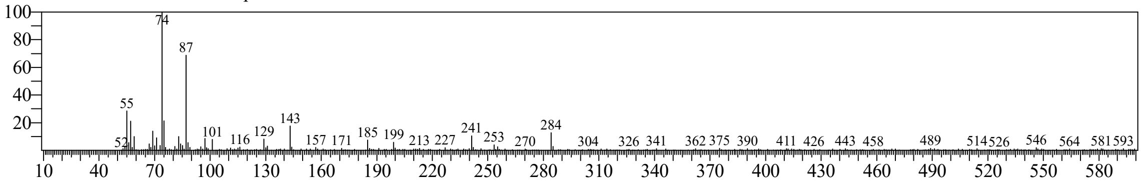


Hit#:1  Entry:27274  Library:NIST14s.lib
SI:94  Formula:C18H36O2  CAS:1731-92-6  MolWeight:284  RetIndex:1978
CompName:Heptadecanoic acid, methyl ester $$ Margaric acid methyl ester $$ Methyl heptadecanoate $$ Methyl margarate $$ n-Heptadecanoic acid methy

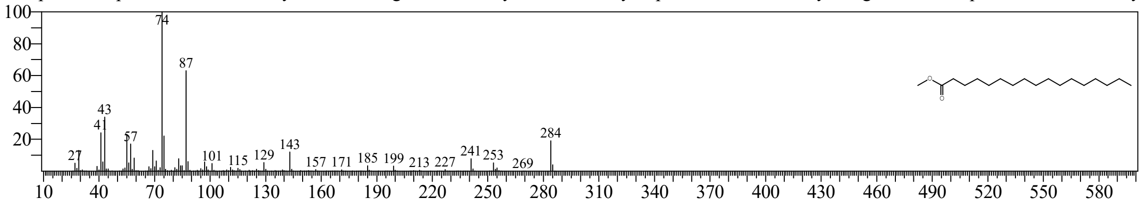


Hit#:2  Entry:31783  Library:NIST17s.lib
SI:94  Formula:C18H36O2  CAS:1731-92-6  MolWeight:284  RetIndex:1978
CompName:Heptadecanoic acid, methyl ester $$ Margaric acid methyl ester $$ Methyl heptadecanoate $$ Methyl margarate $$ n-Heptadecanoic acid methy

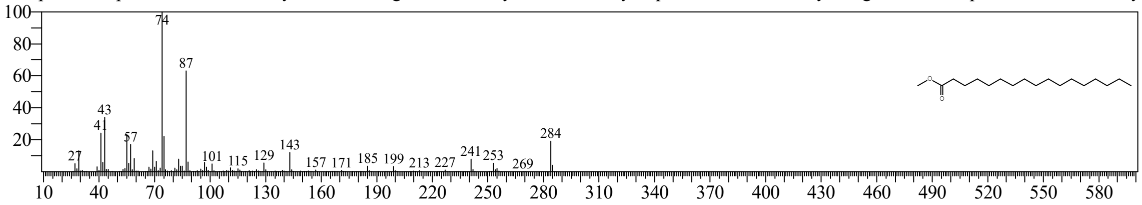


Hit#:3  Entry:27277  Library:NIST14s.lib
SI:93  Formula:C18H36O2  CAS:6929-04-0  MolWeight:284  RetIndex:1914
CompName:Hexadecanoic acid, 15-methyl-, methyl ester $$ Methyl isoheptadecanoate $$ Methyl 15-methylhexadecanoate $$

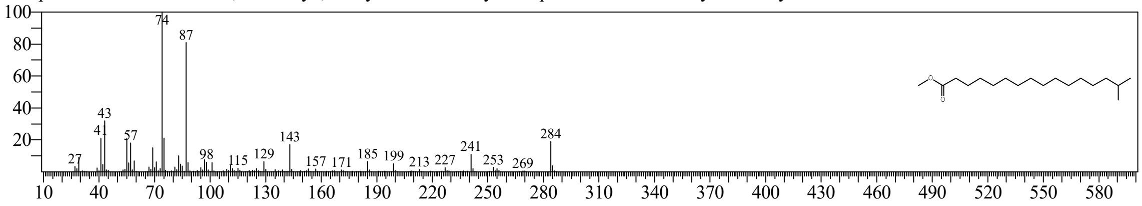


Hit#:4  Entry:127714  Library:NIST17-1.lib
SI:93  Formula:C18H36O2  CAS:6929-04-0  MolWeight:284  RetIndex:1914
CompName:Hexadecanoic acid, 15-methyl-, methyl ester $$ Methyl isoheptadecanoate $$ Methyl 15-methylhexadecanoate $$

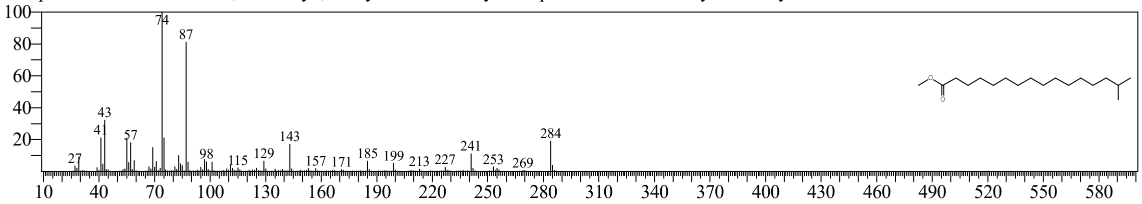


Hit#:5  Entry:27279  Library:NIST14s.lib
SI:93  Formula:C18H36O2  CAS:1731-92-6  MolWeight:284  RetIndex:1978
CompName:Heptadecanoic acid, methyl ester $$ Margaric acid methyl ester $$ Methyl heptadecanoate $$ Methyl margarate $$ n-Heptadecanoic acid methy

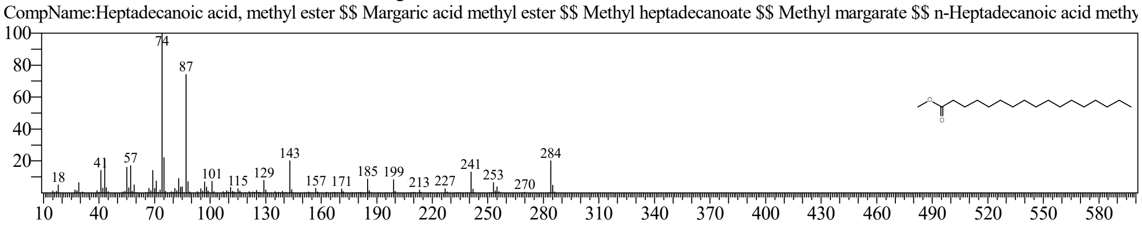

<< Target >>
Line#:6 R.Time:24.100(Scan#:1693) MassPeaks:356
RawMode:Averaged 24.092-24.108(1692-1694) BasePeak:55.05(587)
BG Mode:Calc. from Peak Group 1 - Event 1

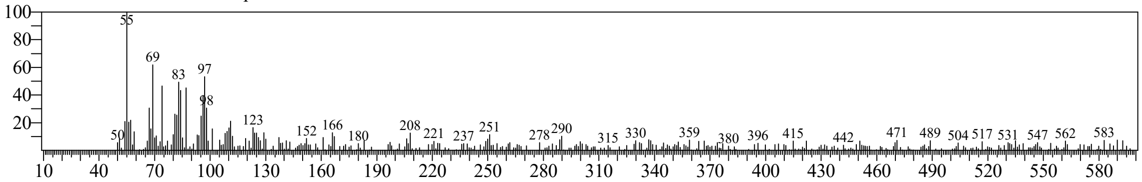


Hit#:1 Entry:115094 Library:NIST14.lib
SI:86 Formula:C18H34O2 CAS:0-00-0 MolWeight:282 RetIndex:1986
CompName:cis-10-Heptadecenoic acid, methyl ester

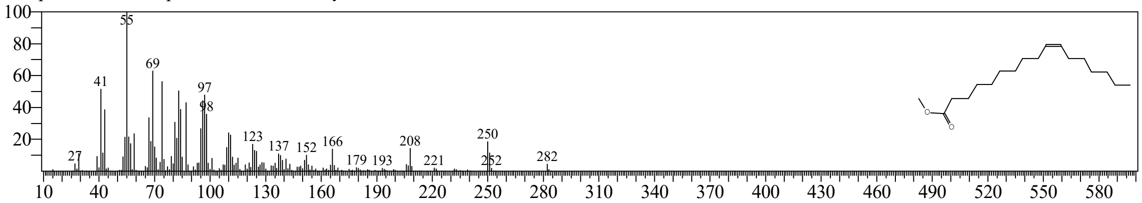


Hit#:2 Entry:125438 Library:NIST17-1.lib
SI:86 Formula:C18H34O2 CAS:0-00-0 MolWeight:282 RetIndex:1986
CompName:cis-10-Heptadecenoic acid, methyl ester

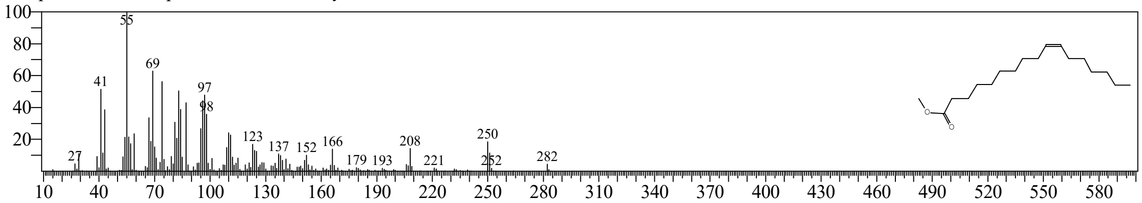


Hit#:3 Entry:115095 Library:NIST14.lib
SI:85 Formula:C18H34O2 CAS:0-00-0 MolWeight:282 RetIndex:1986
CompName:Methyl 8-heptadecenoate

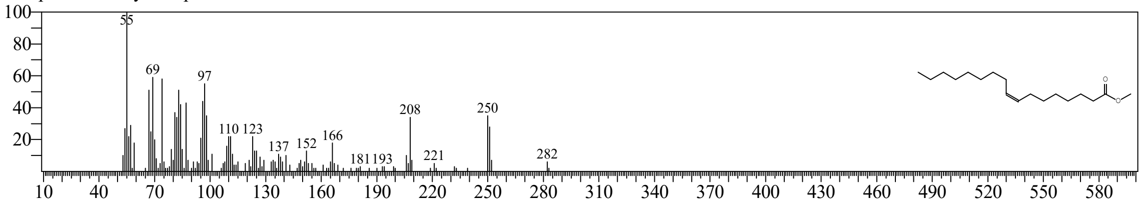


Hit#:4 Entry:125439 Library:NIST17-1.lib
SI:85 Formula:C18H34O2 CAS:0-00-0 MolWeight:282 RetIndex:1986
CompName:Methyl 8-heptadecenoate

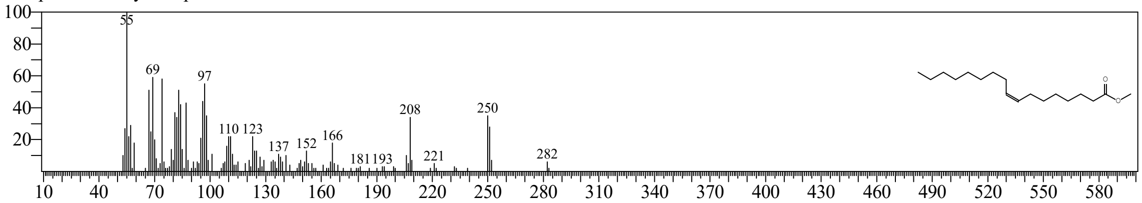


Hit#:5 Entry:168029 Library:NIST17-1.lib
SI:84 Formula:C21H40O2 CAS:2390-09-2 MolWeight:324 RetIndex:2284
CompName:cis-Methyl 11-eicosenoate $$ 11-Eicosenoic acid, methyl ester, (Z)- $$

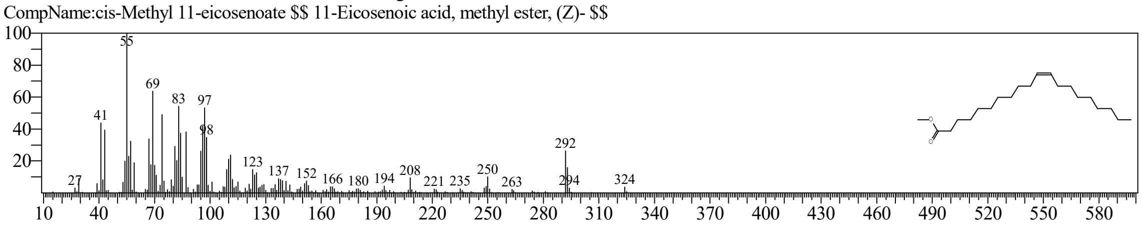

<< Target >>
Line#:7 R.Time:24.875(Scan#:1786) MassPeaks:386
RawMode:Averaged 24.867-24.883(1785-1787) BasePeak:74.00(131134)
BG Mode:Calc. from Peak Group 1 - Event 1

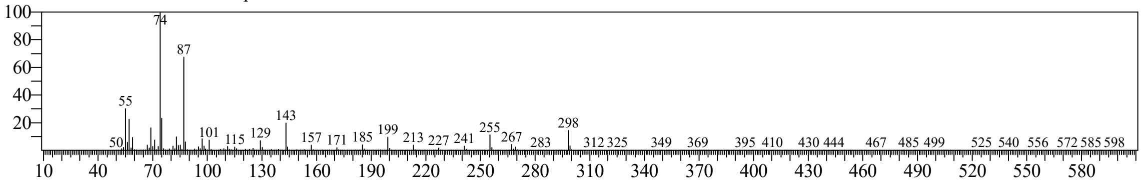


Hit#:1 Entry:129694 Library:NIST14.lib
SI:98 Formula:C19H38O2 CAS:112-61-8 MolWeight:298 RetIndex:2077
CompName:Methyl stearate $$ Octadecanoic acid, methyl ester $$ Stearic acid, methyl ester $$ n-Octadecanoic acid, methyl ester $$ Kemester 9718 $$ Me

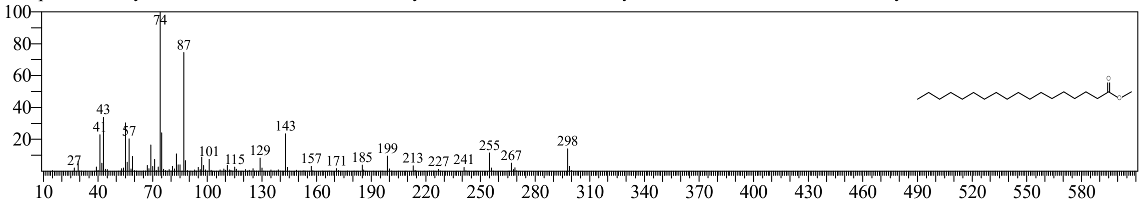


Hit#:2 Entry:141978 Library:NIST17-1.lib
SI:98 Formula:C19H38O2 CAS:112-61-8 MolWeight:298 RetIndex:2077
CompName:Methyl stearate $$ Octadecanoic acid, methyl ester $$ Stearic acid, methyl ester $$ n-Octadecanoic acid, methyl ester $$ Kemester 9718 $$ Me

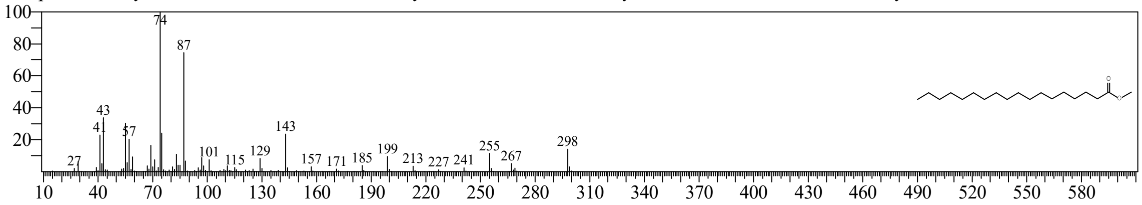


Hit#:3 Entry:28255 Library:NIST14s.lib
SI:97 Formula:C19H38O2 CAS:112-61-8 MolWeight:298 RetIndex:2077
CompName:Methyl stearate $$ Octadecanoic acid, methyl ester $$ Stearic acid, methyl ester $$ n-Octadecanoic acid, methyl ester $$ Kemester 9718 $$ Me

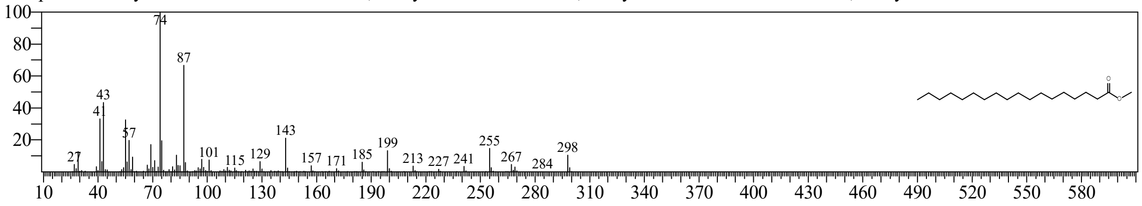


Hit#:4 Entry:32873 Library:NIST17s.lib
SI:97 Formula:C19H38O2 CAS:112-61-8 MolWeight:298 RetIndex:2077
CompName:Methyl stearate $$ Octadecanoic acid, methyl ester $$ Stearic acid, methyl ester $$ n-Octadecanoic acid, methyl ester $$ Kemester 9718 $$ Me

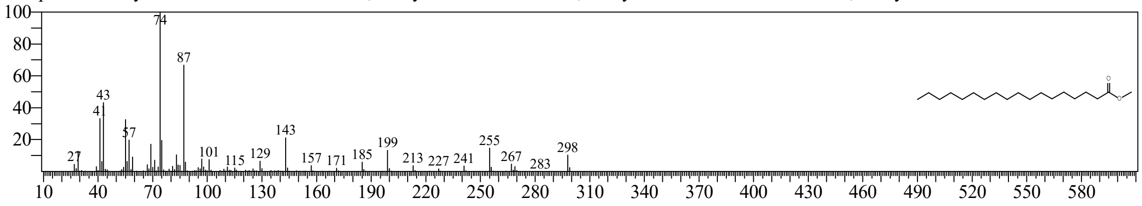


Hit#:5 Entry:28257 Library:NIST14s.lib
SI:96 Formula:C19H38O2 CAS:112-61-8 MolWeight:298 RetIndex:2077
CompName:Methyl stearate $$ Octadecanoic acid, methyl ester $$ Stearic acid, methyl ester $$ n-Octadecanoic acid, methyl ester $$ Kemester 9718 $$ Me

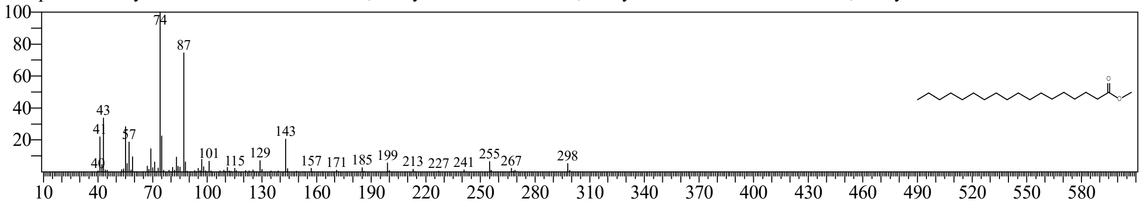

<< Target >>
Line#:8  R.Time:26.267(Scan#:1953)  MassPeaks:424
RawMode:Averaged 26.258-26.275(1952-1954) BasePeak:55.05(228451)
BG Mode:Calc. from Peak  Group 1 - Event 1

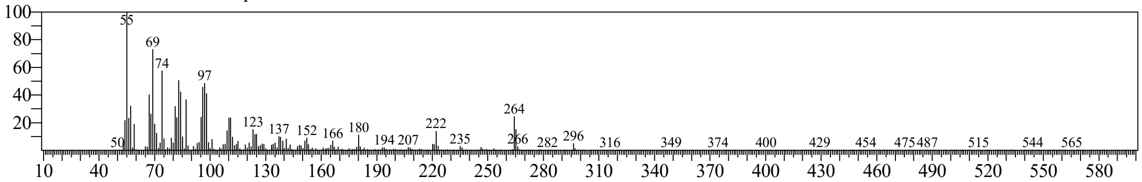


Hit#:1  Entry:32747  Library:NIST17s.lib
SI:97  Formula:C19H36O2  CAS:1937-62-8  MolWeight:296  RetIndex:2085
CompName:9-Octadecenoic acid, methyl ester, (E)- $$ Elaidic acid, methyl ester $$ Methyl elaidate $$ Methyl trans-9-octadecenoate $$ (E)-9-Octadecenoic

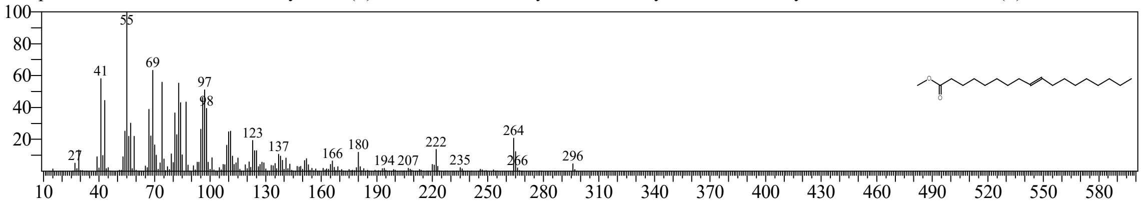


Hit#:2  Entry:28138  Library:NIST14s.lib
SI:97  Formula:C19H36O2  CAS:1937-62-8  MolWeight:296  RetIndex:2085
CompName:9-Octadecenoic acid, methyl ester, (E)- $$ Elaidic acid, methyl ester $$ Methyl elaidate $$ Methyl trans-9-octadecenoate $$ (E)-9-Octadecenoic

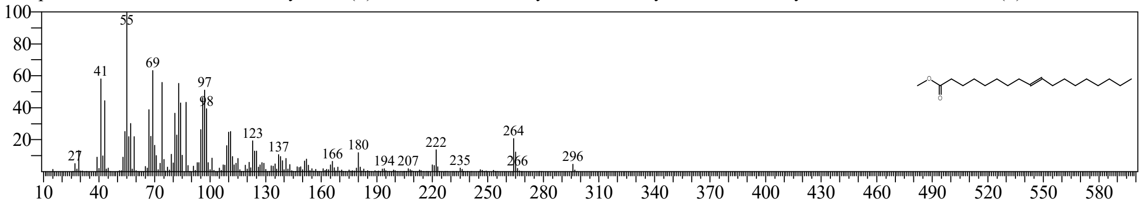


Hit#:3  Entry:127664  Library:NIST14.lib
SI:96  Formula:C19H36O2  CAS:112-62-9  MolWeight:296  RetIndex:2085
CompName:9-Octadecenoic acid (Z)-, methyl ester $$ Oleic acid, methyl ester $$ Emery oleic acid ester 2301 $$ Methyl cis-9-octadecenoate $$ Methyl ole

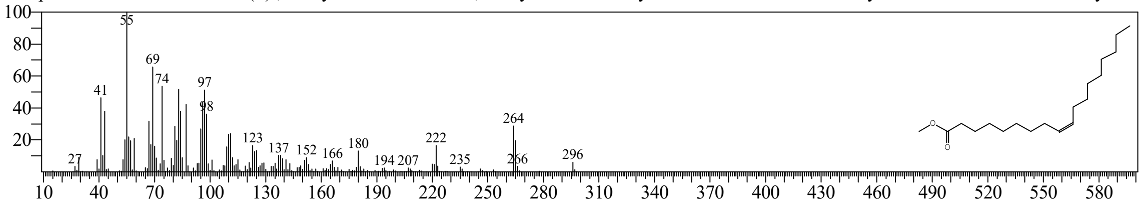


Hit#:4  Entry:139650  Library:NIST17-1.lib
SI:96  Formula:C19H36O2  CAS:112-62-9  MolWeight:296  RetIndex:2085
CompName:9-Octadecenoic acid (Z)-, methyl ester $$ Oleic acid, methyl ester $$ Emery oleic acid ester 2301 $$ Methyl cis-9-octadecenoate $$ Methyl ole

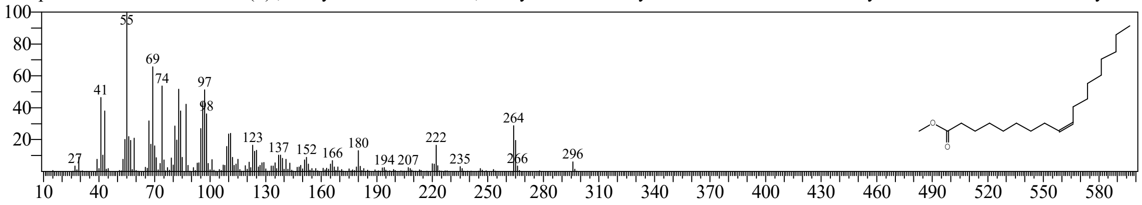


Hit#:5  Entry:32744  Library:NIST17s.lib
SI:95  Formula:C19H36O2  CAS:112-62-9  MolWeight:296  RetIndex:2085
CompName:9-Octadecenoic acid (Z)-, methyl ester $$ Oleic acid, methyl ester $$ Emery oleic acid ester 2301 $$ Methyl cis-9-octadecenoate $$ Methyl ole

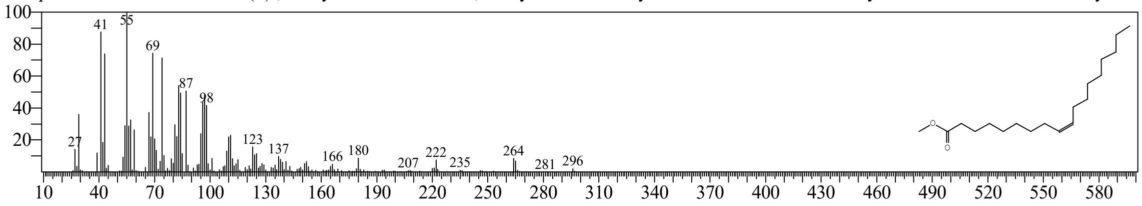

<< Target >>
Line#:9  R.Time:26.383(Scan#:1967)  MassPeaks:335
RawMode:Averaged 26.375-26.392(1966-1968) BasePeak:55.05(17699)
BG Mode:Calc. from Peak  Group 1 - Event 1

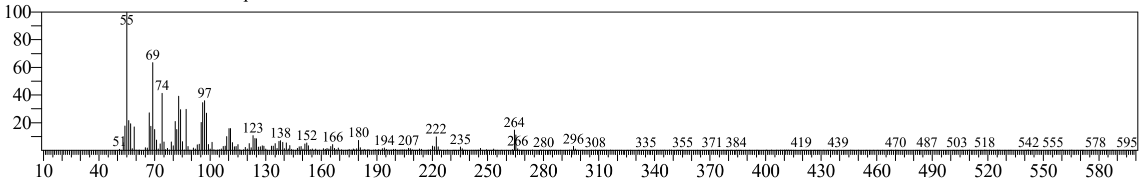


Hit#:1  Entry:32748  Library:NIST17s.lib
SI:95  Formula:C19H36O2  CAS:52380-33-3  MolWeight:296  RetIndex:2085
CompName:11-Octadecenoic acid, methyl ester $$ Methyl 11-octadecenoate $$ Octadec-11-enoic acid, methyl ester $$ Methyl (11E)-11-octadecenoate # $$

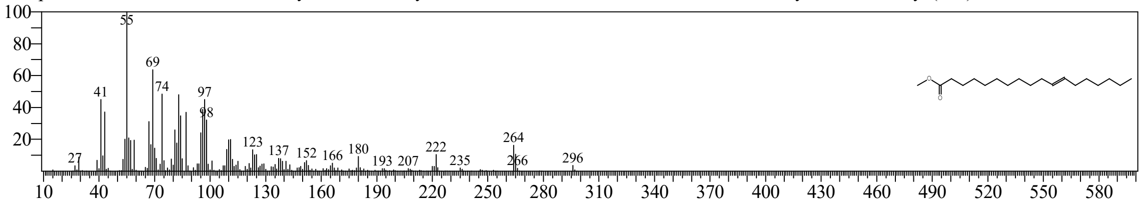


Hit#:2  Entry:28139  Library:NIST14s.lib
SI:95  Formula:C19H36O2  CAS:52380-33-3  MolWeight:296  RetIndex:2085
CompName:11-Octadecenoic acid, methyl ester $$ Methyl 11-octadecenoate $$ Octadec-11-enoic acid, methyl ester $$ Methyl (11E)-11-octadecenoate # $$

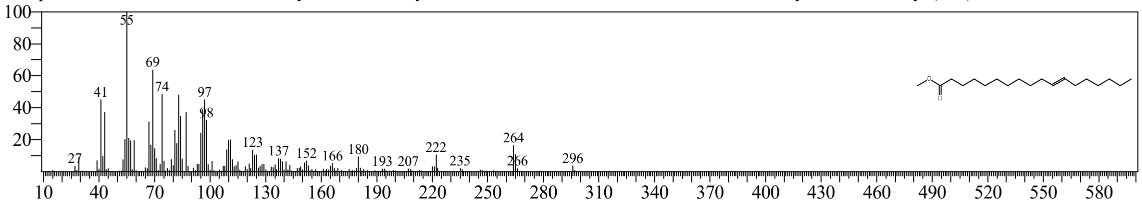


Hit#:3  Entry:139636  Library:NIST17-1.lib
SI:95  Formula:C19H36O2  CAS:13481-95-3  MolWeight:296  RetIndex:2085
CompName:10-Octadecenoic acid, methyl ester $$ Methyl 10-octadecenoate $$

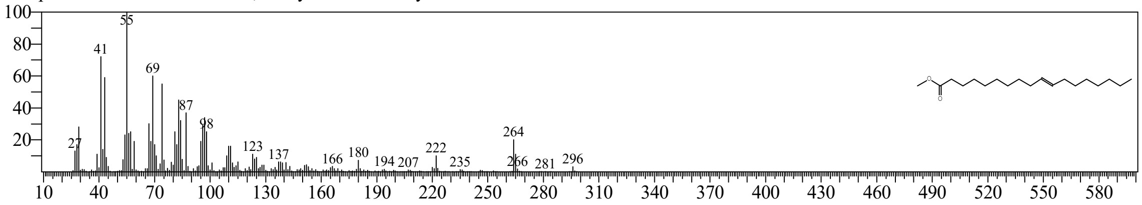


Hit#:4  Entry:127650  Library:NIST14.lib
SI:95  Formula:C19H36O2  CAS:13481-95-3  MolWeight:296  RetIndex:2085
CompName:10-Octadecenoic acid, methyl ester $$ Methyl 10-octadecenoate $$

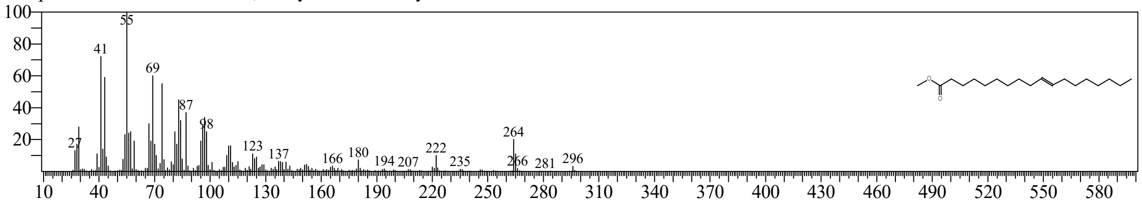


Hit#:5  Entry:127661  Library:NIST14.lib
SI:95  Formula:C19H36O2  CAS:0-00-0  MolWeight:296  RetIndex:2085
CompName:cis-13-Octadecenoic acid, methyl ester

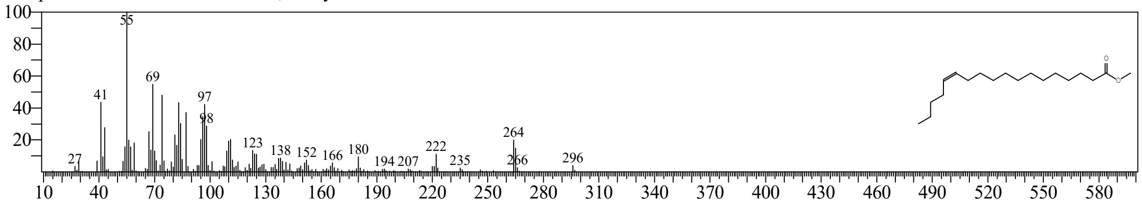

<< Target >>
Line#:10 R.Time:27.733(Scan#:2129) MassPeaks:315
RawMode:Averaged 27.725-27.742(2128-2130) BasePeak:67.05(3860)
BG Mode:Calc. from Peak Group 1 - Event 1

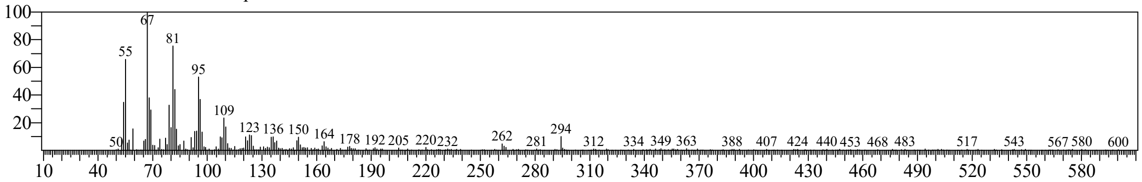


Hit#:1 Entry:137663 Library:NIST17-1.lib
SI:96 Formula:C19H34O2 CAS:2462-85-3 MolWeight:294 RetIndex:2093
CompName:9,12-Octadecadienoic acid, methyl ester $$ 9,12-Octadecenoic acid, methyl ester $$ Methyl octadeca-9,12-dienoate $$ Methyl 9,12-octadecadi

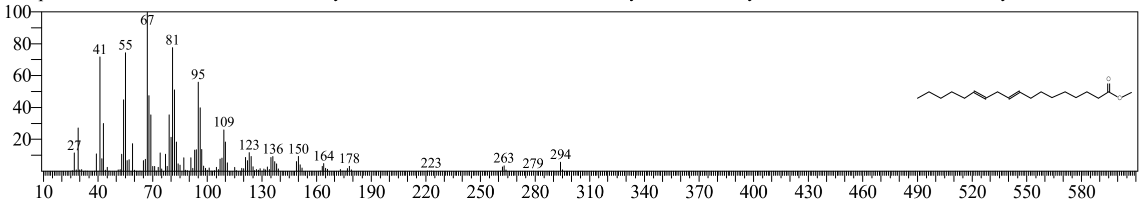


Hit#:2 Entry:125931 Library:NIST14.lib
SI:96 Formula:C19H34O2 CAS:2462-85-3 MolWeight:294 RetIndex:2093
CompName:9,12-Octadecadienoic acid, methyl ester $$ 9,12-Octadecenoic acid, methyl ester $$ Methyl octadeca-9,12-dienoate $$ Methyl 9,12-octadecadi

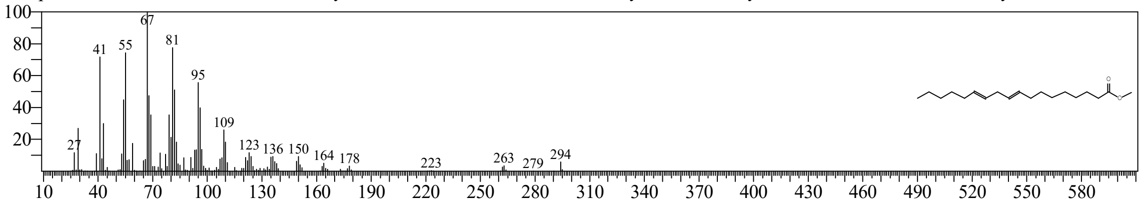


Hit#:3 Entry:32587 Library:NIST17s.lib
SI:96 Formula:C19H34O2 CAS:13038-47-6 MolWeight:294 RetIndex:2093
CompName:9,11-Octadecadienoic acid, methyl ester, (E,E)- $$ Methyl trans-9,trans-11-octadecadienoate $$ Methyl trans,trans-9,11-octadecadienoate $$ M

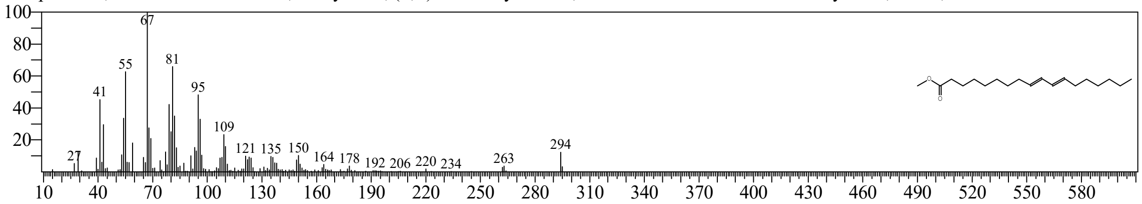


Hit#:4 Entry:27999 Library:NIST14s.lib
SI:95 Formula:C19H34O2 CAS:112-63-0 MolWeight:294 RetIndex:2093
CompName:9,12-Octadecadienoic acid (Z,Z)-, methyl ester $$ Linoleic acid, methyl ester $$ Methyl cis,cis-9,12-octadecadienoate $$ Methyl linoleate $$ M

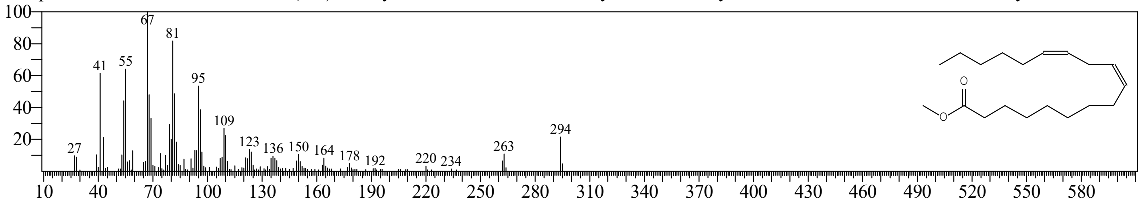


Hit#:5 Entry:32588 Library:NIST17s.lib
SI:95 Formula:C19H34O2 CAS:112-63-0 MolWeight:294 RetIndex:2093
CompName:9,12-Octadecadienoic acid (Z,Z)-, methyl ester $$ Linoleic acid, methyl ester $$ Methyl cis,cis-9,12-octadecadienoate $$ Methyl linoleate $$ M

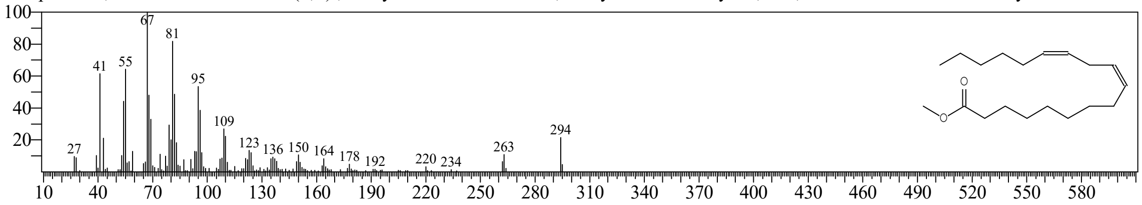

<< Target >>
Line#:11 R.Time:27.958(Scan#:2156) MassPeaks:345
RawMode:Averaged 27.950-27.967(2155-2157) BasePeak:67.05(2415)
BG Mode:Calc. from Peak Group 1 - Event 1

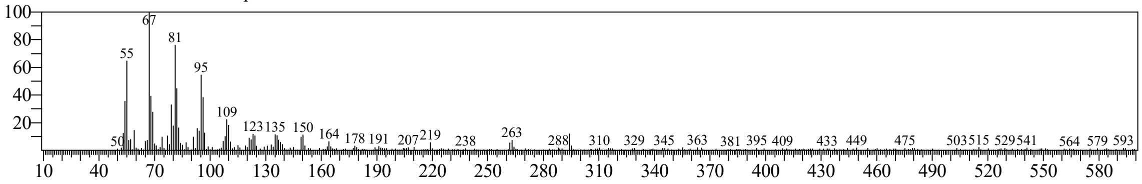


Hit#:1 Entry:27999 Library:NIST14s.lib
SI:95 Formula:C19H34O2 CAS:112-63-0 MolWeight:294 RetIndex:2093
CompName:9,12-Octadecadienoic acid (Z,Z)-, methyl ester $$ Linoleic acid, methyl ester $$ Methyl cis,cis-9,12-octadecadienoate $$ Methyl linoleate $$ M

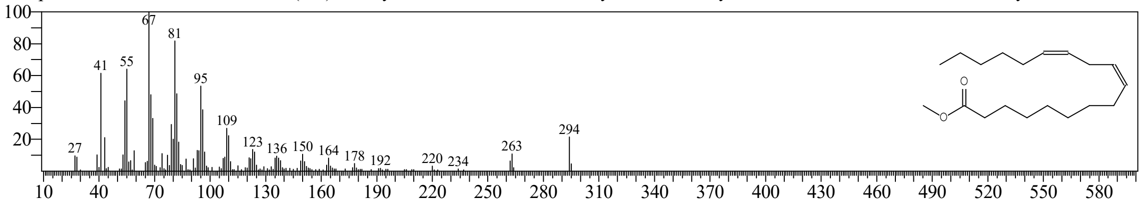


Hit#:2 Entry:32588 Library:NIST17s.lib
SI:95 Formula:C19H34O2 CAS:112-63-0 MolWeight:294 RetIndex:2093
CompName:9,12-Octadecadienoic acid (Z,Z)-, methyl ester $$ Linoleic acid, methyl ester $$ Methyl cis,cis-9,12-octadecadienoate $$ Methyl linoleate $$ M

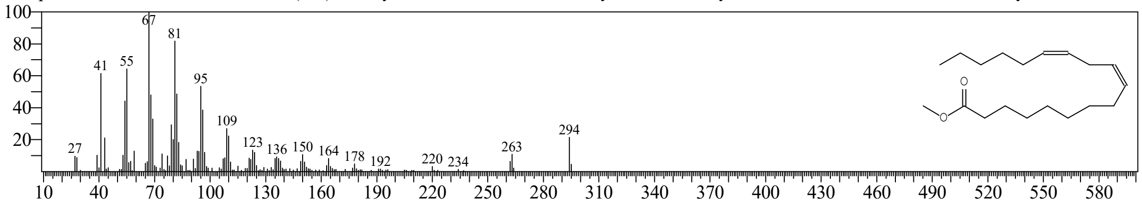


Hit#:3 Entry:125931 Library:NIST14.lib
SI:95 Formula:C19H34O2 CAS:2462-85-3 MolWeight:294 RetIndex:2093
CompName:9,12-Octadecadienoic acid, methyl ester $$ 9,12-Octadecenoic acid, methyl ester $$ Methyl octadeca-9,12-dienoate $$ Methyl 9,12-octadecadi

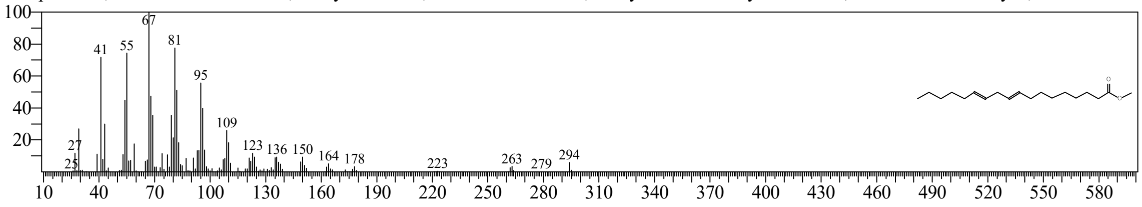


Hit#:4 Entry:137663 Library:NIST17-1.lib
SI:95 Formula:C19H34O2 CAS:2462-85-3 MolWeight:294 RetIndex:2093
CompName:9,12-Octadecadienoic acid, methyl ester $$ 9,12-Octadecenoic acid, methyl ester $$ Methyl octadeca-9,12-dienoate $$ Methyl 9,12-octadecadi

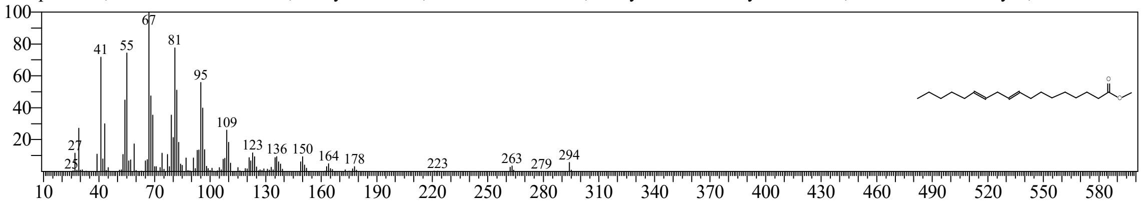


Hit#:5 Entry:32589 Library:NIST17s.lib
SI:95 Formula:C19H34O2 CAS:112-63-0 MolWeight:294 RetIndex:2093
CompName:9,12-Octadecadienoic acid (Z,Z)-, methyl ester $$ Linoleic acid, methyl ester $$ Methyl cis,cis-9,12-octadecadienoate $$ Methyl linoleate $$ M

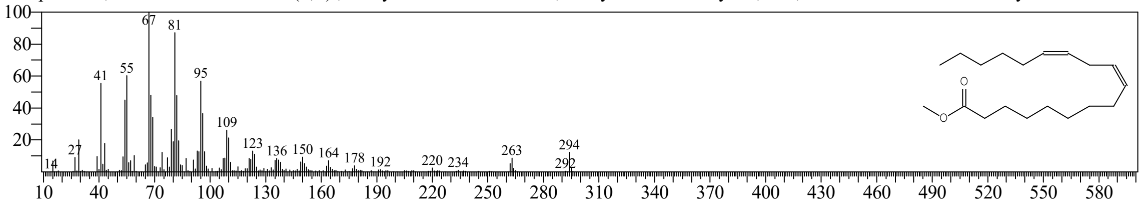

<< Target >>
Line#:12 R.Time:28.375(Scan#:2206) MassPeaks:390
RawMode:Averaged 28.367-28.383(2205-2207) BasePeak:67.05(444478)
BG Mode:Calc. from Peak Group 1 - Event 1

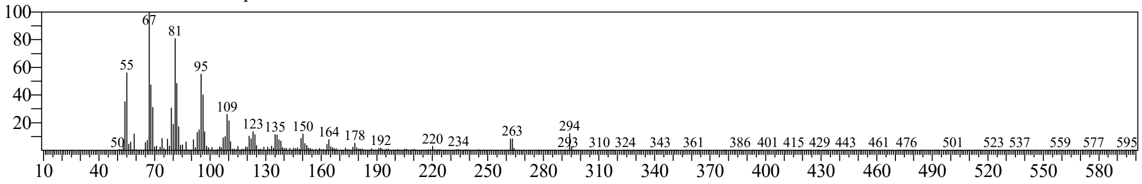


Hit#:1 Entry:32588 Library:NIST17s.lib
SI:97 Formula:C19H34O2 CAS:112-63-0 MolWeight:294 RetIndex:2093
CompName:9,12-Octadecadienoic acid (Z,Z)-, methyl ester $$ Linoleic acid, methyl ester $$ Methyl cis,cis-9,12-octadecadienoate $$ Methyl linoleate $$ M

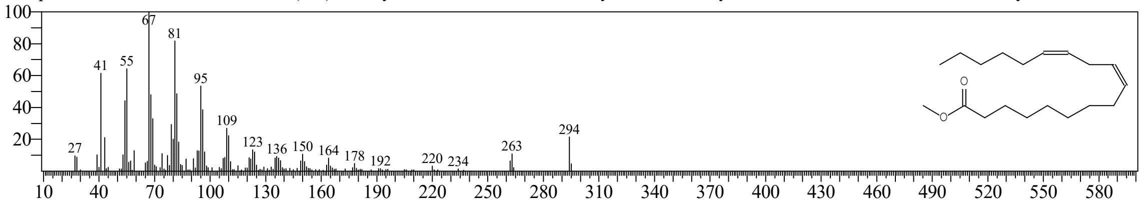


Hit#:2 Entry:27999 Library:NIST14s.lib
SI:97 Formula:C19H34O2 CAS:112-63-0 MolWeight:294 RetIndex:2093
CompName:9,12-Octadecadienoic acid (Z,Z)-, methyl ester $$ Linoleic acid, methyl ester $$ Methyl cis,cis-9,12-octadecadienoate $$ Methyl linoleate $$ M

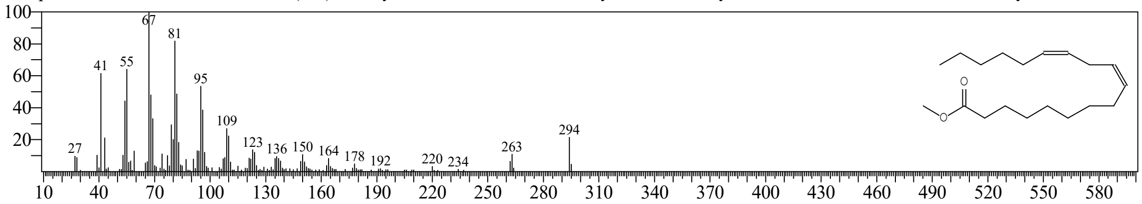


Hit#:3 Entry:32589 Library:NIST17s.lib
SI:97 Formula:C19H34O2 CAS:112-63-0 MolWeight:294 RetIndex:2093
CompName:9,12-Octadecadienoic acid (Z,Z)-, methyl ester $$ Linoleic acid, methyl ester $$ Methyl cis,cis-9,12-octadecadienoate $$ Methyl linoleate $$ M

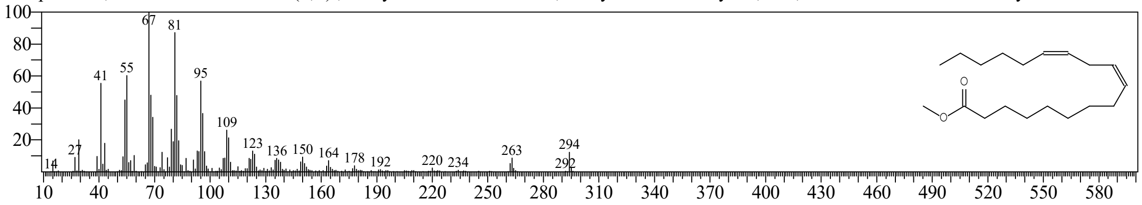


Hit#:4 Entry:28000 Library:NIST14s.lib
SI:97 Formula:C19H34O2 CAS:112-63-0 MolWeight:294 RetIndex:2093
CompName:9,12-Octadecadienoic acid (Z,Z)-, methyl ester $$ Linoleic acid, methyl ester $$ Methyl cis,cis-9,12-octadecadienoate $$ Methyl linoleate $$ M

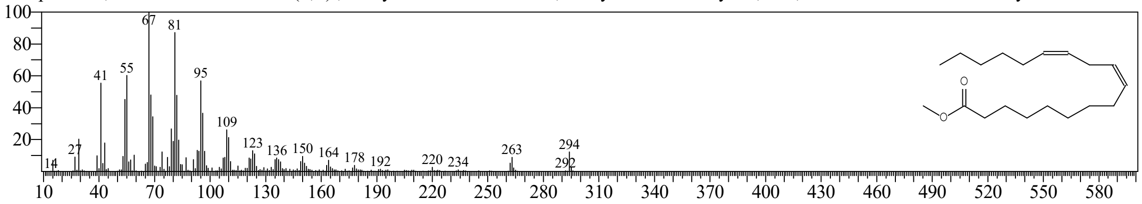


Hit#:5 Entry:137664 Library:NIST17-1.lib
SI:95 Formula:C19H34O2 CAS:0-00-0 MolWeight:294 RetIndex:2093
CompName:Methyl 10-trans,12-cis-octadecadienoate

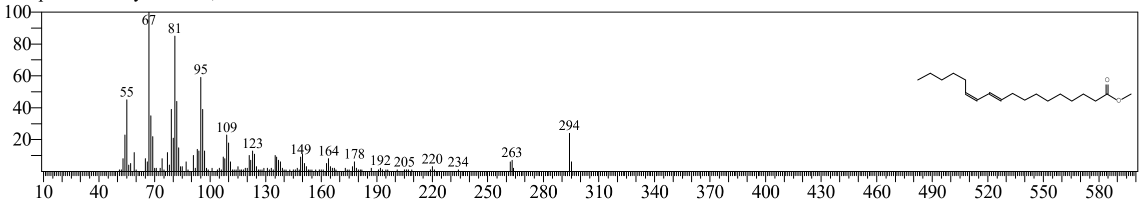

<< Target >>
Line#:13   R.Time:29.275(Scan#:2314)  MassPeaks:358
RawMode:Averaged 29.267-29.283(2313-2315) BasePeak:74.00(12887)
BG Mode:Calc. from Peak  Group 1 - Event 1

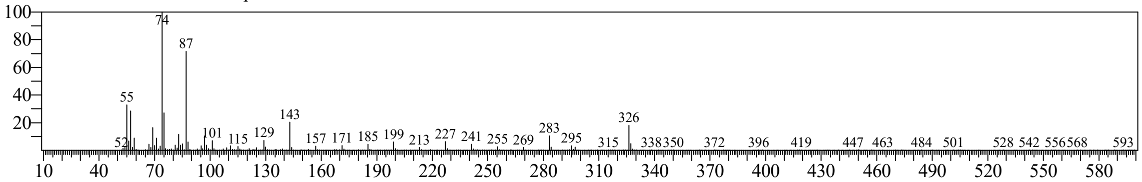


Hit#:1  Entry:170238  Library:NIST17-1.lib
SI:97  Formula:C21H42O2  CAS:0-00-0  MolWeight:326  RetIndex:2212
CompName:Methyl 18-methylnonadecanoate

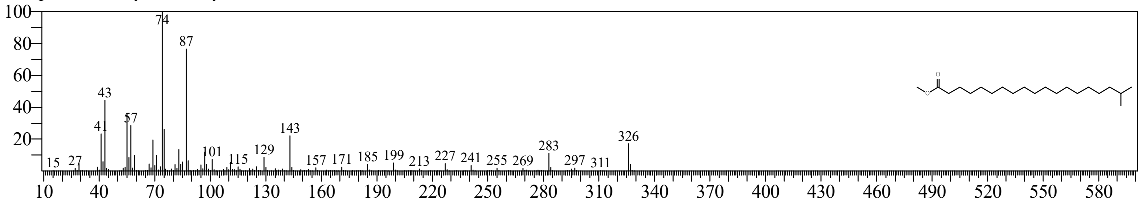


Hit#:2  Entry:154704  Library:NIST14.lib
SI:97  Formula:C21H42O2  CAS:0-00-0  MolWeight:326  RetIndex:2212
CompName:Methyl 18-methylnonadecanoate

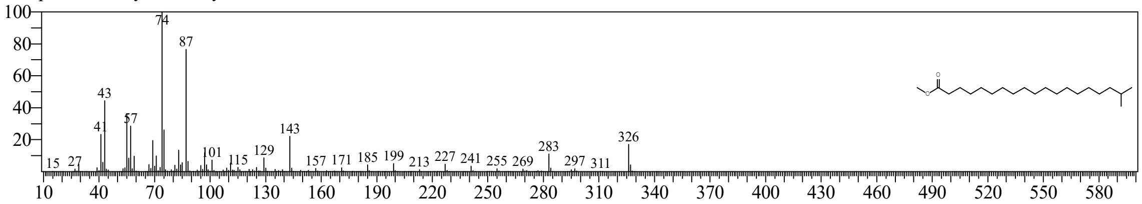


Hit#:3  Entry:34726  Library:NIST17s.lib
SI:96  Formula:C21H42O2  CAS:1120-28-1  MolWeight:326  RetIndex:2276
CompName:Eicosanoic acid, methyl ester $$ Methyl arachisate $$ Methyl eicosanoate $$ Arachidic acid methyl ester $$ Kemester 2050 $$ Methyl aracidat

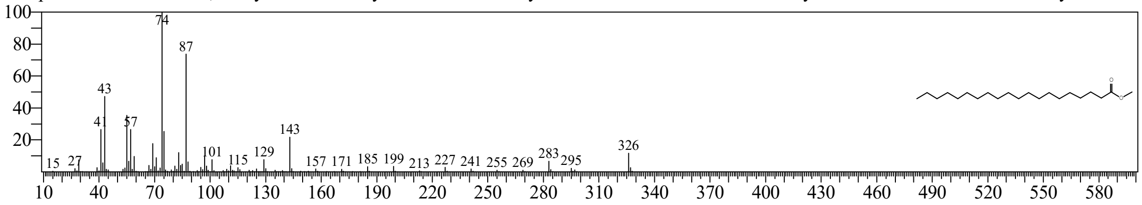


Hit#:4  Entry:29931  Library:NIST14s.lib
SI:93  Formula:C21H42O2  CAS:1120-28-1  MolWeight:326  RetIndex:2276
CompName:Eicosanoic acid, methyl ester $$ Methyl arachisate $$ Methyl eicosanoate $$ Arachidic acid methyl ester $$ Kemester 2050 $$ Methyl aracidat

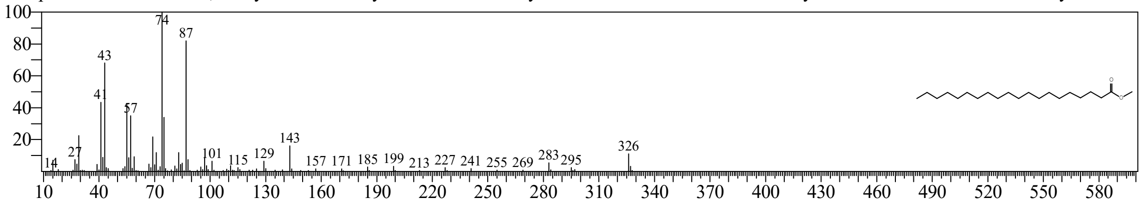


Hit#:5  Entry:34724  Library:NIST17s.lib
SI:93  Formula:C21H42O2  CAS:1120-28-1  MolWeight:326  RetIndex:2276
CompName:Eicosanoic acid, methyl ester $$ Methyl arachisate $$ Methyl eicosanoate $$ Arachidic acid methyl ester $$ Kemester 2050 $$ Methyl aracidat

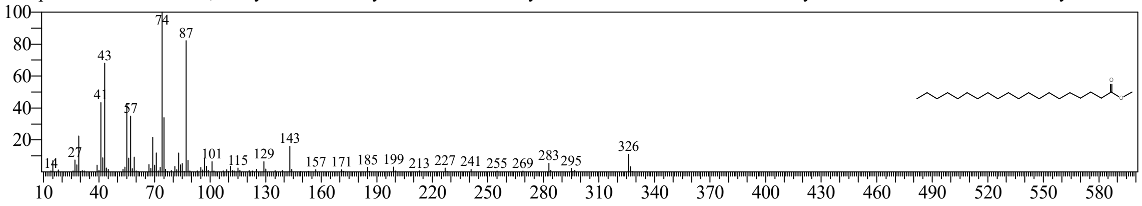

<< Target >>
Line#:14 R.Time:29.825(Scan#:2380) MassPeaks:380
RawMode:Averaged 29.817-29.833(2379-2381) BasePeak:79.05(8406)
BG Mode:Calc. from Peak Group 1 - Event 1

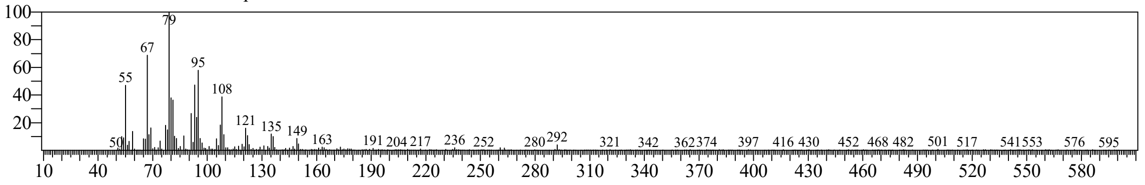


Hit#:1 Entry:32438 Library:NIST17s.lib
SI:95 Formula:C19H32O2 CAS:301-00-8 MolWeight:292 RetIndex:2101
CompName:9,12,15-Octadecatrienoic acid, methyl ester, (Z,Z,Z)- $$ Linolenic acid, methyl ester $$ Methyl all-cis-9,12,15-octadecatrienoate $$ Methyl lino

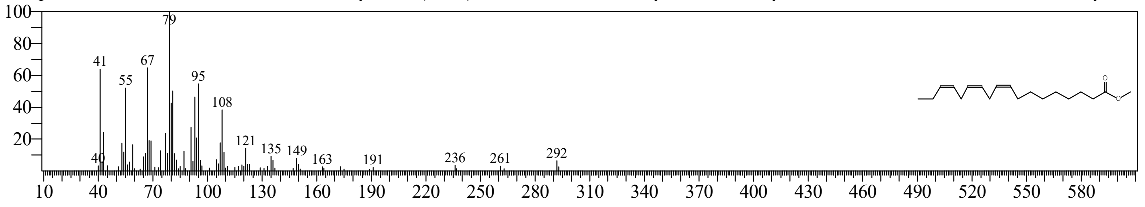


Hit#:2 Entry:135724 Library:NIST17-1.lib
SI:95 Formula:C19H32O2 CAS:301-00-8 MolWeight:292 RetIndex:2101
CompName:9,12,15-Octadecatrienoic acid, methyl ester, (Z,Z,Z)- $$ Linolenic acid, methyl ester $$ Methyl all-cis-9,12,15-octadecatrienoate $$ Methyl lino

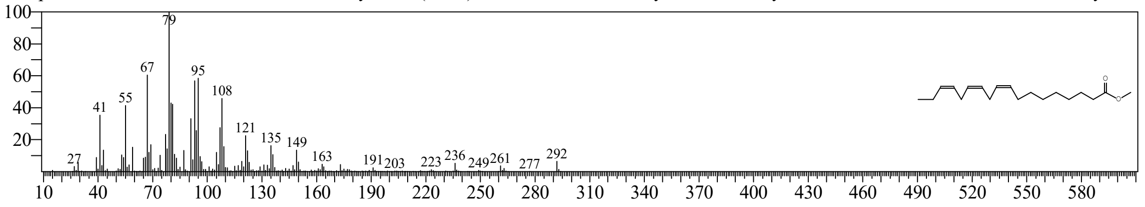


Hit#:3 Entry:124228 Library:NIST14.lib
SI:95 Formula:C19H32O2 CAS:301-00-8 MolWeight:292 RetIndex:2101
CompName:9,12,15-Octadecatrienoic acid, methyl ester, (Z,Z,Z)- $$ Linolenic acid, methyl ester $$ Methyl all-cis-9,12,15-octadecatrienoate $$ Methyl lino

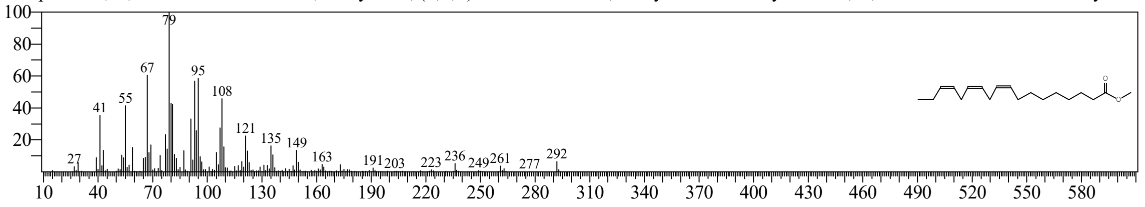


Hit#:4 Entry:27872 Library:NIST14s.lib
SI:95 Formula:C19H32O2 CAS:301-00-8 MolWeight:292 RetIndex:2101
CompName:9,12,15-Octadecatrienoic acid, methyl ester, (Z,Z,Z)- $$ Linolenic acid, methyl ester $$ Methyl all-cis-9,12,15-octadecatrienoate $$ Methyl lino

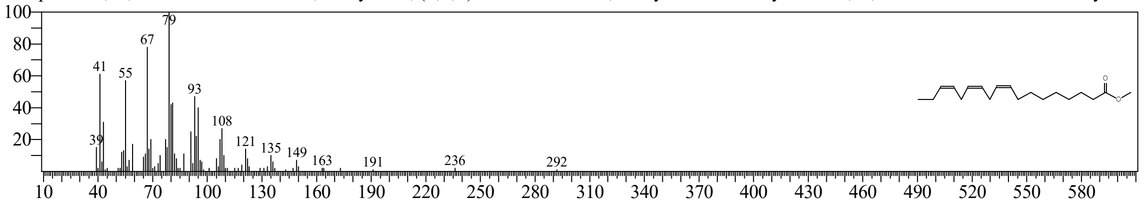


Hit#:5 Entry:32437 Library:NIST17s.lib
SI:95 Formula:C19H32O2 CAS:301-00-8 MolWeight:292 RetIndex:2101
CompName:9,12,15-Octadecatrienoic acid, methyl ester, (Z,Z,Z)- $$ Linolenic acid, methyl ester $$ Methyl all-cis-9,12,15-octadecatrienoate $$ Methyl lino

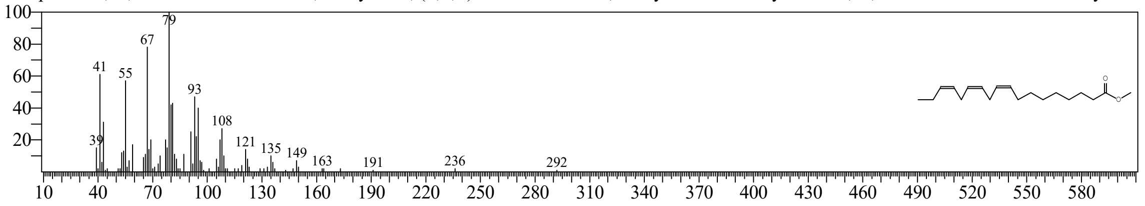

<< Target >>
Line#:15 R.Time:30.300(Scan#:2437) MassPeaks:351
RawMode:Averaged 30.292-30.308(2436-2438) BasePeak:79.05(6336)
BG Mode:Calc. from Peak Group 1 - Event 1

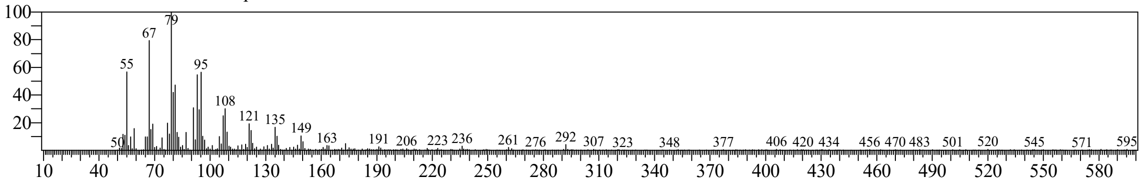


Hit#:1 Entry:135724 Library:NIST17-1.lib
SI:96 Formula:C19H32O2 CAS:301-00-8 MolWeight:292 RetIndex:2101
CompName:9,12,15-Octadecatrienoic acid, methyl ester, (Z,Z,Z)- $$ Linolenic acid, methyl ester $$ Methyl all-cis-9,12,15-octadecatrienoate $$ Methyl lino

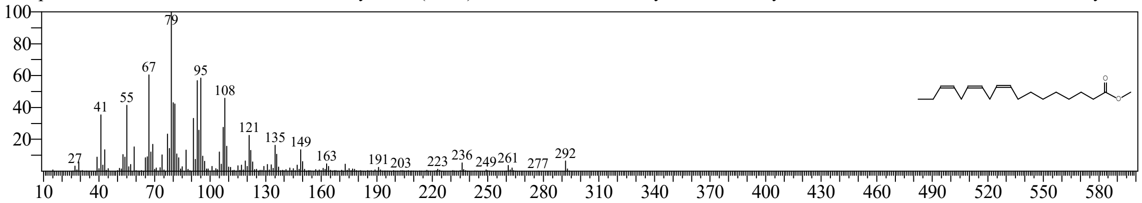


Hit#:2 Entry:124228 Library:NIST14.lib
SI:96 Formula:C19H32O2 CAS:301-00-8 MolWeight:292 RetIndex:2101
CompName:9,12,15-Octadecatrienoic acid, methyl ester, (Z,Z,Z)- $$ Linolenic acid, methyl ester $$ Methyl all-cis-9,12,15-octadecatrienoate $$ Methyl lino

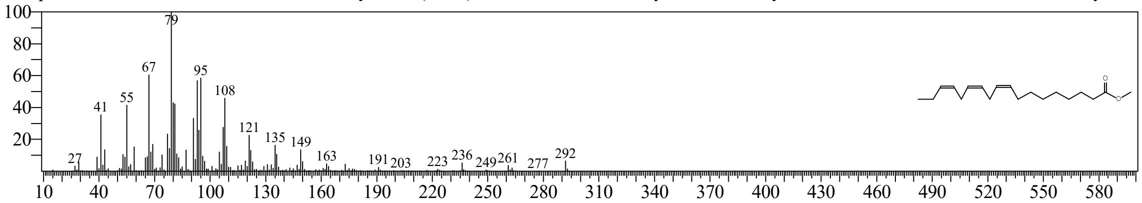


Hit#:3 Entry:111654 Library:NIST14.lib
SI:94 Formula:C18H30O2 CAS:0-00-0 MolWeight:278 RetIndex:2002
CompName:Methyl 8,11,14-heptadecatrienoate

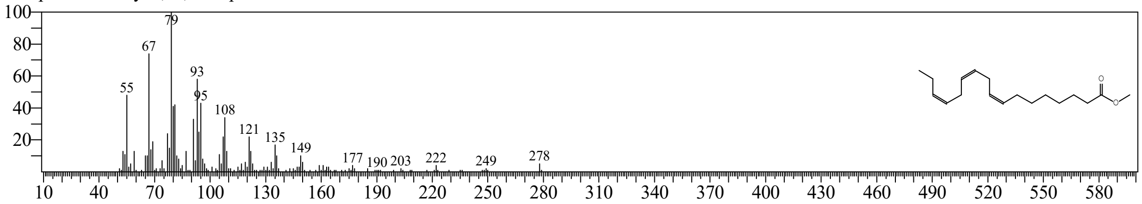


Hit#:4 Entry:121536 Library:NIST17-1.lib
SI:94 Formula:C18H30O2 CAS:0-00-0 MolWeight:278 RetIndex:2002
CompName:Methyl 8,11,14-heptadecatrienoate

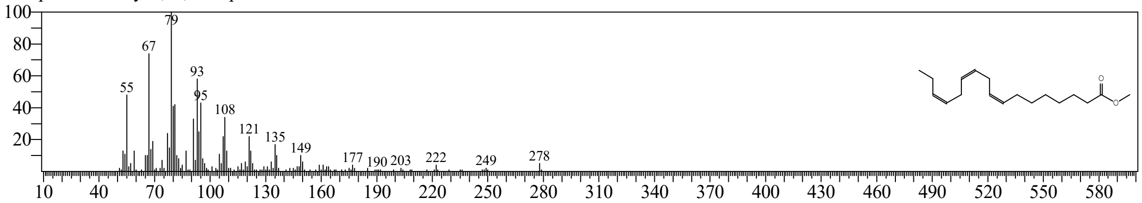


Hit#:5 Entry:27872 Library:NIST14s.lib
SI:94 Formula:C19H32O2 CAS:301-00-8 MolWeight:292 RetIndex:2101
CompName:9,12,15-Octadecatrienoic acid, methyl ester, (Z,Z,Z)- $$ Linolenic acid, methyl ester $$ Methyl all-cis-9,12,15-octadecatrienoate $$ Methyl lino

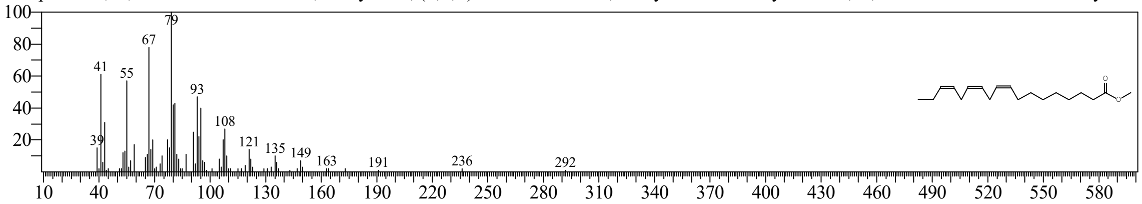

<< Target >>
Line#:16 R.Time:30.608(Scan#:2474) MassPeaks:389
RawMode:Averaged 30.600-30.617(2473-2475) BasePeak:79.05(75087)
BG Mode:Calc. from Peak Group 1 - Event 1

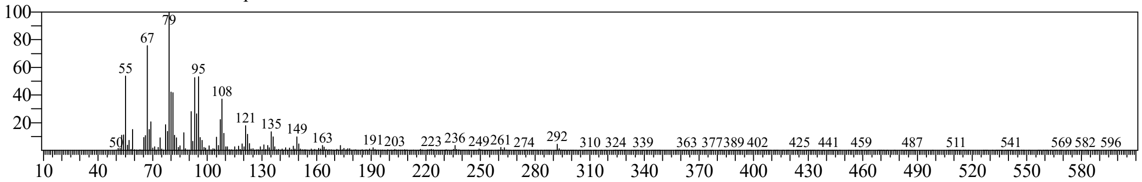


Hit#:1 Entry:124228 Library:NIST14.lib
SI:96 Formula:C19H32O2 CAS:301-00-8 MolWeight:292 RetIndex:2101
CompName:9,12,15-Octadecatrienoic acid, methyl ester, (Z,Z,Z)- $$ Linolenic acid, methyl ester $$ Methyl all-cis-9,12,15-octadecatrienoate $$ Methyl lino

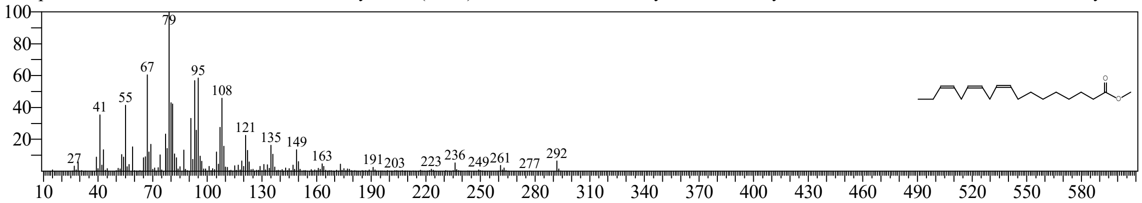


Hit#:2 Entry:135724 Library:NIST17-1.lib
SI:96 Formula:C19H32O2 CAS:301-00-8 MolWeight:292 RetIndex:2101
CompName:9,12,15-Octadecatrienoic acid, methyl ester, (Z,Z,Z)- $$ Linolenic acid, methyl ester $$ Methyl all-cis-9,12,15-octadecatrienoate $$ Methyl lino

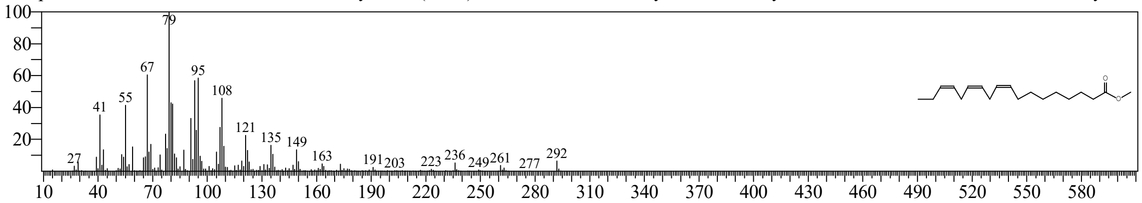


Hit#:3 Entry:32438 Library:NIST17s.lib
SI:95 Formula:C19H32O2 CAS:301-00-8 MolWeight:292 RetIndex:2101
CompName:9,12,15-Octadecatrienoic acid, methyl ester, (Z,Z,Z)- $$ Linolenic acid, methyl ester $$ Methyl all-cis-9,12,15-octadecatrienoate $$ Methyl lino

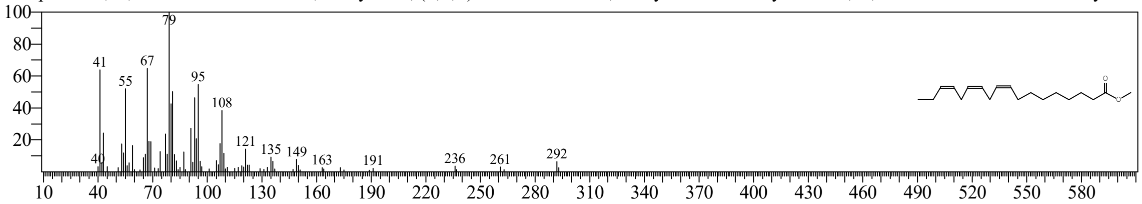


Hit#:4 Entry:27872 Library:NIST14s.lib
SI:95 Formula:C19H32O2 CAS:301-00-8 MolWeight:292 RetIndex:2101
CompName:9,12,15-Octadecatrienoic acid, methyl ester, (Z,Z,Z)- $$ Linolenic acid, methyl ester $$ Methyl all-cis-9,12,15-octadecatrienoate $$ Methyl lino

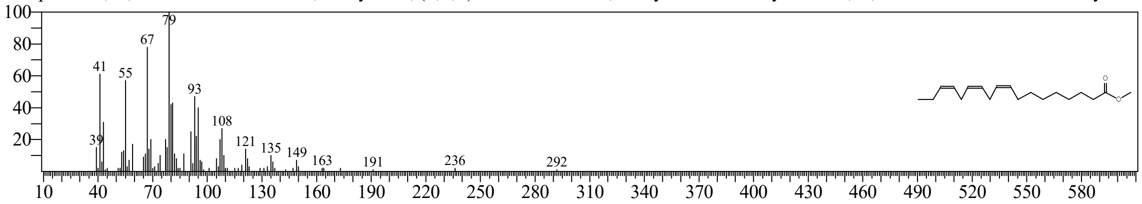


Hit#:5 Entry:32437 Library:NIST17s.lib
SI:95 Formula:C19H32O2 CAS:301-00-8 MolWeight:292 RetIndex:2101
CompName:9,12,15-Octadecatrienoic acid, methyl ester, (Z,Z,Z)- $$ Linolenic acid, methyl ester $$ Methyl all-cis-9,12,15-octadecatrienoate $$ Methyl lino

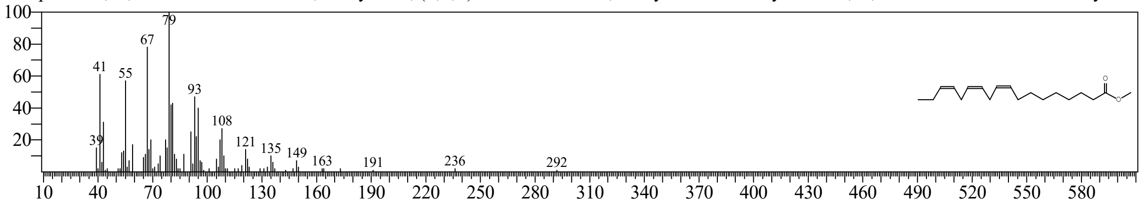

<< Target >>
Line#:17 R.Time:30.933(Scan#:2513) MassPeaks:362
RawMode:Averaged 30.925-30.942(2512-2514) BasePeak:67.05(6763)
BG Mode:Calc. from Peak Group 1 - Event 1

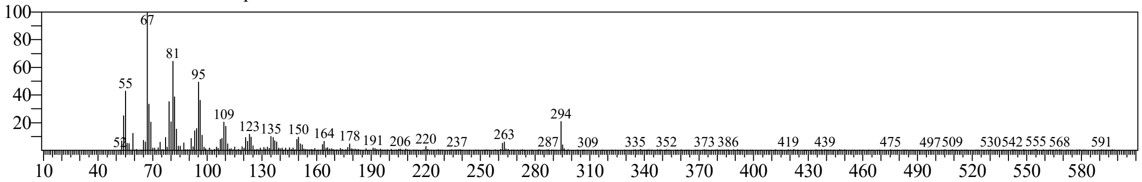


Hit#:1 Entry:137666 Library:NIST17-1.lib
SI:97 Formula:C19H34O2 CAS:13058-52-1 MolWeight:294 RetIndex:2093
CompName:Methyl 9-cis,11-trans-octadecadienoate

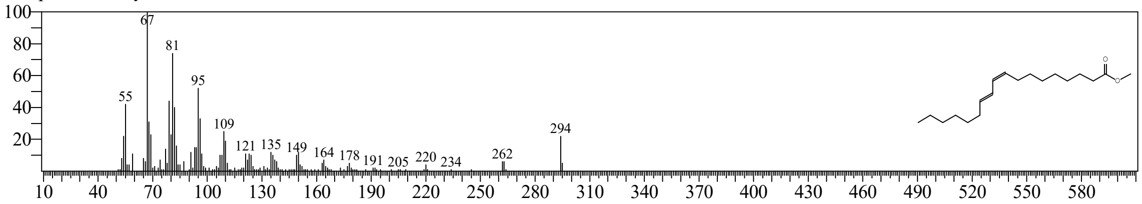


Hit#:2 Entry:125932 Library:NIST14.lib
SI:97 Formula:C19H34O2 CAS:0-00-0 MolWeight:294 RetIndex:2093
CompName:Methyl 9-cis,11-trans-octadecadienoate

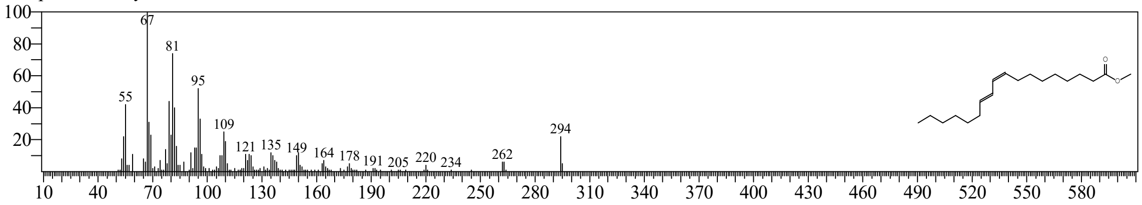


Hit#:3 Entry:125933 Library:NIST14.lib
SI:97 Formula:C19H34O2 CAS:0-00-0 MolWeight:294 RetIndex:2093
CompName:Methyl 10-trans,12-cis-octadecadienoate

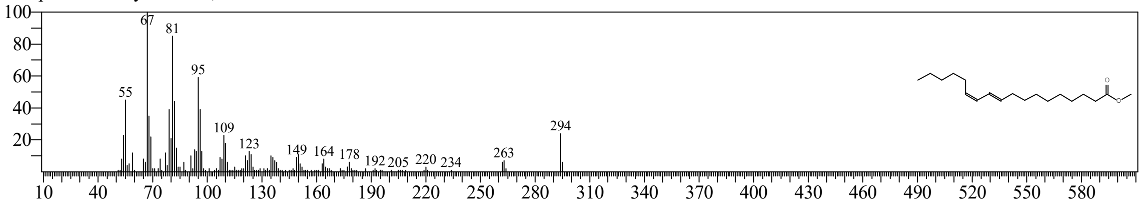


Hit#:4 Entry:137664 Library:NIST17-1.lib
SI:97 Formula:C19H34O2 CAS:0-00-0 MolWeight:294 RetIndex:2093
CompName:Methyl 10-trans,12-cis-octadecadienoate

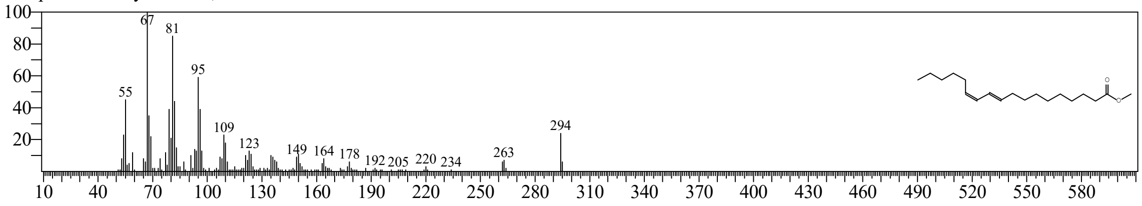


Hit#:5 Entry:32587 Library:NIST17s.lib
SI:95 Formula:C19H34O2 CAS:13038-47-6 MolWeight:294 RetIndex:2093
CompName:9,11-Octadecadienoic acid, methyl ester, (E,E)- $$ Methyl trans-9,trans-11-octadecadienoate $$ Methyl trans,trans-9,11-octadecadienoate $$ M

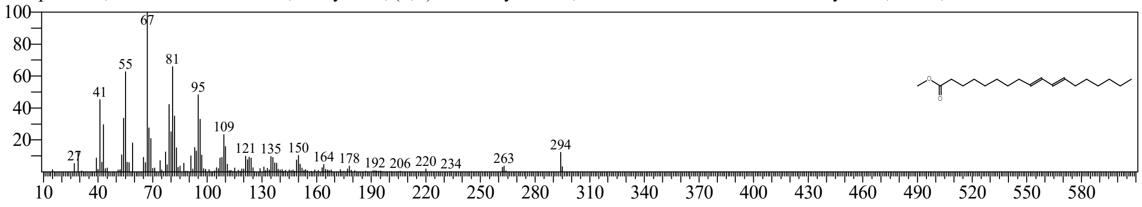

<< Target >>
Line#:18 R.Time:31.267(Scan#:2553) MassPeaks:316
RawMode:Averaged 31.258-31.275(2552-2554) BasePeak:67.05(7098)
BG Mode:Calc. from Peak Group 1 - Event 1

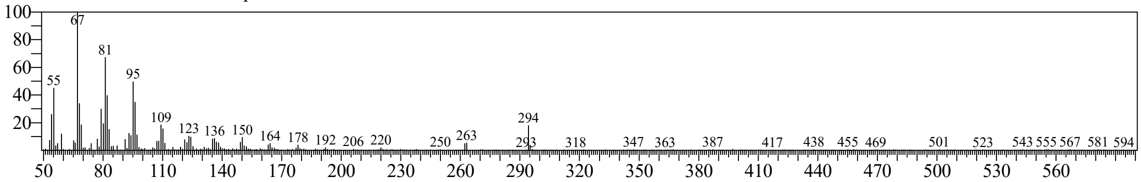


Hit#:1 Entry:125933 Library:NIST14.lib
SI:96 Formula:C19H34O2 CAS:0-00-0 MolWeight:294 RetIndex:2093
CompName:Methyl 10-trans,12-cis-octadecadienoate

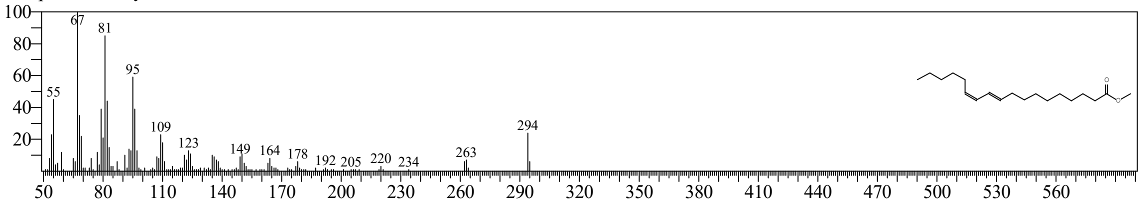


Hit#:2 Entry:137664 Library:NIST17-1.lib
SI:96 Formula:C19H34O2 CAS:0-00-0 MolWeight:294 RetIndex:2093
CompName:Methyl 10-trans,12-cis-octadecadienoate

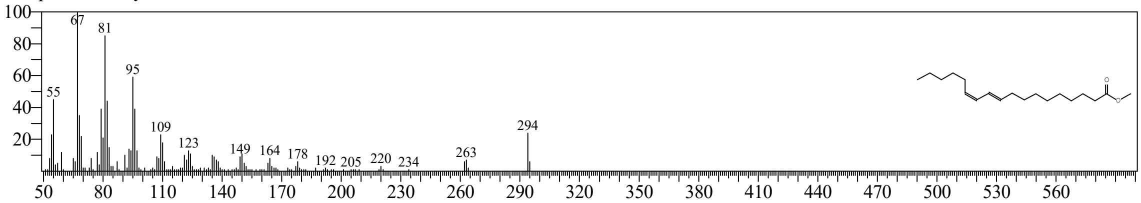


Hit#:3 Entry:125932 Library:NIST14.lib
SI:95 Formula:C19H34O2 CAS:0-00-0 MolWeight:294 RetIndex:2093
CompName:Methyl 9-cis,11-trans-octadecadienoate

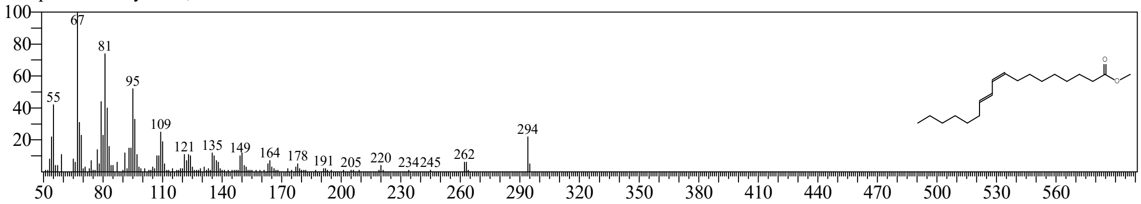


Hit#:4 Entry:137666 Library:NIST17-1.lib
SI:95 Formula:C19H34O2 CAS:13058-52-1 MolWeight:294 RetIndex:2093
CompName:Methyl 9-cis,11-trans-octadecadienoate

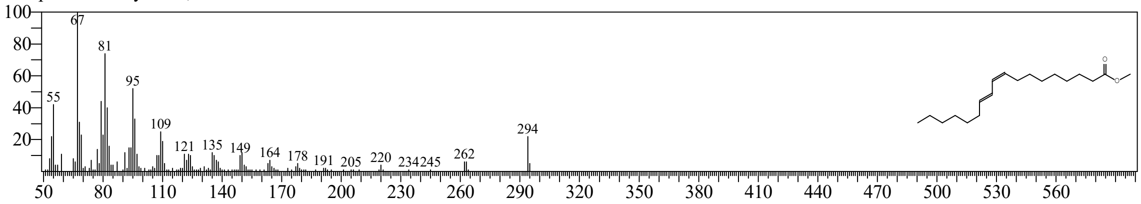


Hit#:5 Entry:28003 Library:NIST14s.lib
SI:95 Formula:C19H34O2 CAS:57156-97-5 MolWeight:294 RetIndex:2093
CompName:12,15-Octadecadienoic acid, methyl ester $$ Methyl (12E,15E)-12,15-octadecadienoate # $$

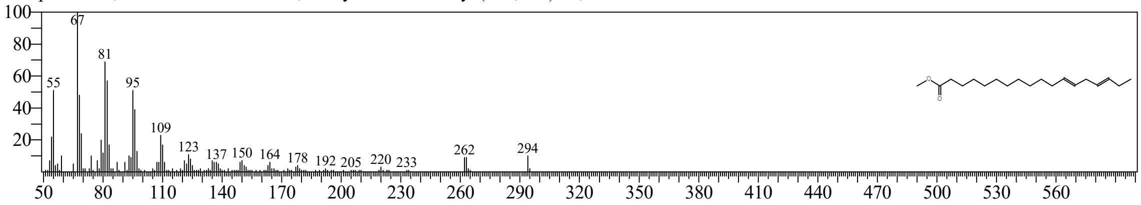

<< Target >>
Line#:19 R.Time:31.917(Scan#:2631) MassPeaks:340
RawMode:Averaged 31.908-31.925(2630-2632) BasePeak:67.05(949)
BG Mode:Calc. from Peak Group 1 - Event 1

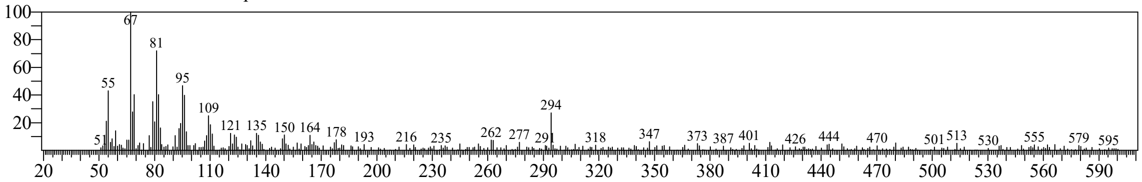


Hit#:1 Entry:125932 Library:NIST14.lib
SI:91 Formula:C19H34O2 CAS:0-00-0 MolWeight:294 RetIndex:2093
CompName:Methyl 9-cis,11-trans-octadecadienoate

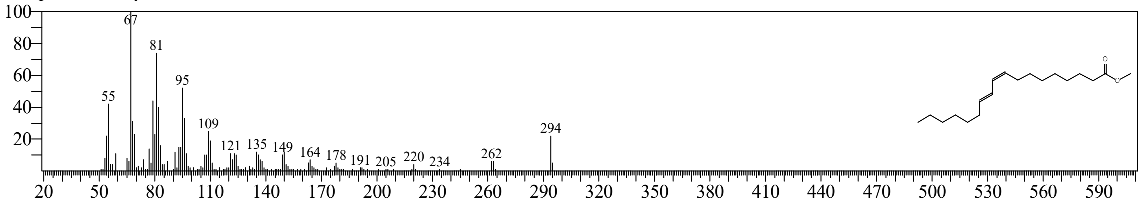


Hit#:2 Entry:137666 Library:NIST17-1.lib
SI:91 Formula:C19H34O2 CAS:13058-52-1 MolWeight:294 RetIndex:2093
CompName:Methyl 9-cis,11-trans-octadecadienoate

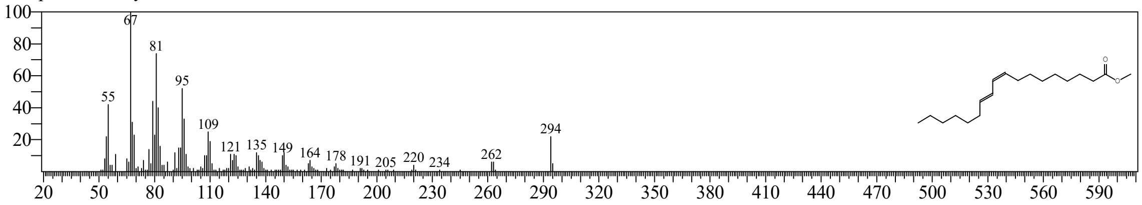


Hit#:3 Entry:137664 Library:NIST17-1.lib
SI:91 Formula:C19H34O2 CAS:0-00-0 MolWeight:294 RetIndex:2093
CompName:Methyl 10-trans,12-cis-octadecadienoate

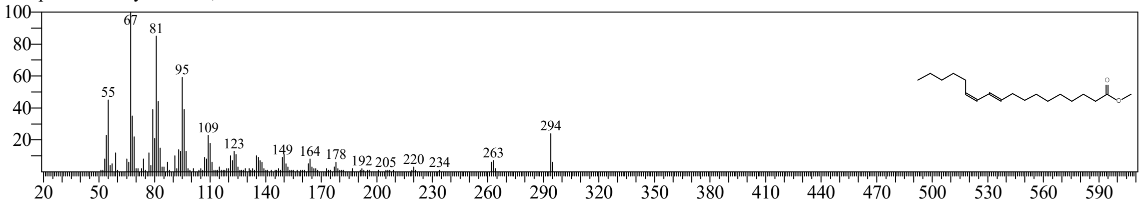


Hit#:4 Entry:125933 Library:NIST14.lib
SI:91 Formula:C19H34O2 CAS:0-00-0 MolWeight:294 RetIndex:2093
CompName:Methyl 10-trans,12-cis-octadecadienoate

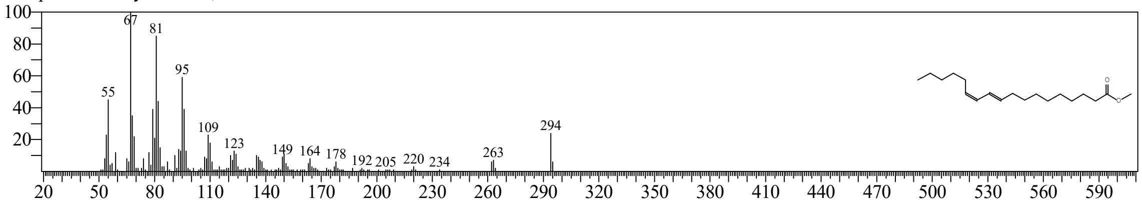


Hit#:5 Entry:27997 Library:NIST14s.lib
SI:90 Formula:C19H34O2 CAS:13038-47-6 MolWeight:294 RetIndex:2093
CompName:9,11-Octadecadienoic acid, methyl ester, (E,E)- $$ Methyl trans-9,trans-11-octadecadienoate $$ Methyl trans,trans-9,11-octadecadienoate $$ M

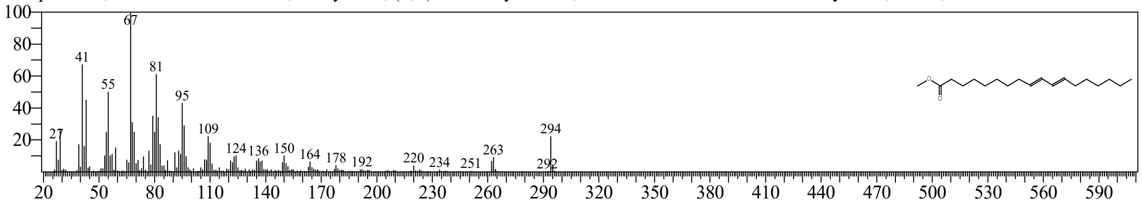

<< Target >>
Line#:20 R.Time:32.950(Scan#:2755) MassPeaks:403
RawMode:Averaged 32.942-32.958(2754-2756) BasePeak:95.10(7756)
BG Mode:Calc. from Peak Group 1 - Event 1

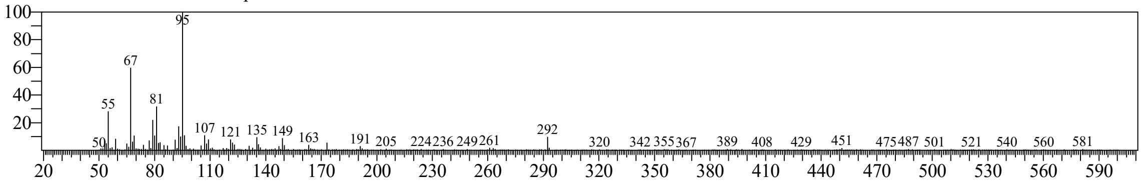


Hit#:1 Entry:24424 Library:NIST17-1.lib
SI:84 Formula:C12H20 CAS:0-00-0 MolWeight:164 RetIndex:1041
CompName:Cis-3-ethyl-endo-tricyclo[5.2.1.0(2.6)]decane

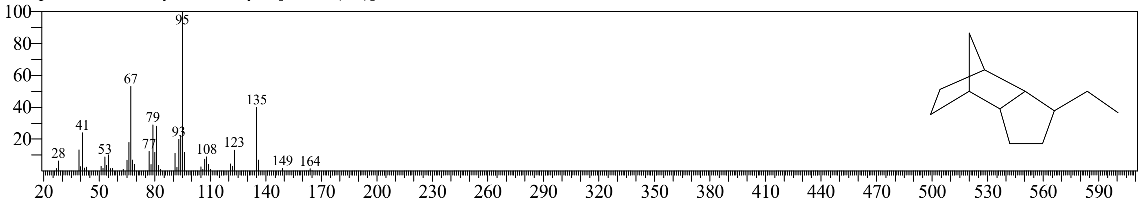


Hit#:2 Entry:23403 Library:NIST14.lib
SI:84 Formula:C12H20 CAS:0-00-0 MolWeight:164 RetIndex:1041
CompName:Cis-3-ethyl-endo-tricyclo[5.2.1.0(2.6)]decane

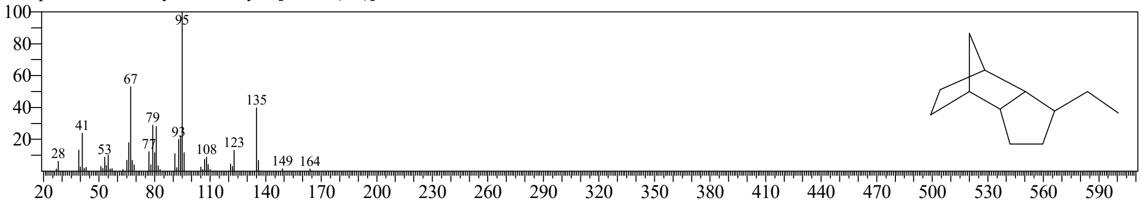


Hit#:3 Entry:16582 Library:NIST17-1.lib
SI:83 Formula:C11H18 CAS:0-00-0 MolWeight:150 RetIndex:1115
CompName:4-Cyclohexenyl-3-methylbutene

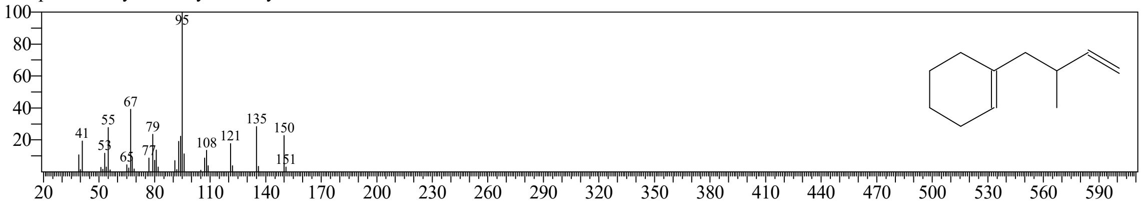


Hit#:4 Entry:16897 Library:NIST14.lib
SI:82 Formula:C10H16O CAS:60485-45-2 MolWeight:152 RetIndex:991
CompName:Santolina epoxide $$ 2-Methyl-2-(3-methyl-1-vinyl-2-butenyl)oxirane # $$

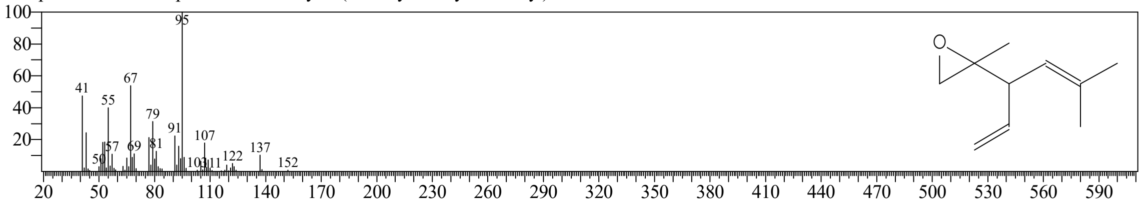


Hit#:5 Entry:17571 Library:NIST17-1.lib
SI:82 Formula:C10H16O CAS:60485-45-2 MolWeight:152 RetIndex:991
CompName:Santolina epoxide $$ 2-Methyl-2-(3-methyl-1-vinyl-2-butenyl)oxirane # $$

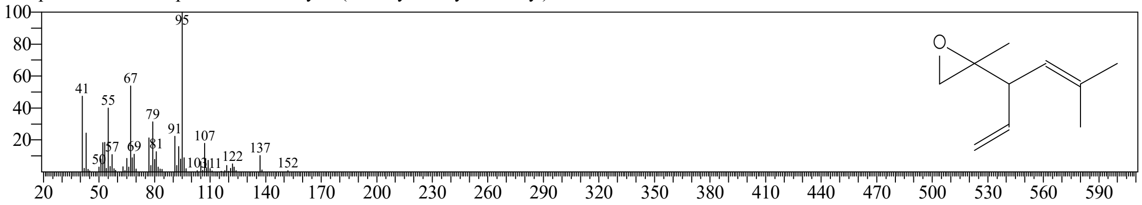

<< Target >>
Line#:21 R.Time:33.500(Scan#:2821) MassPeaks:364
RawMode:Averaged 33.492-33.508(2820-2822) BasePeak:74.00(15338)
BG Mode:Calc. from Peak Group 1 - Event 1

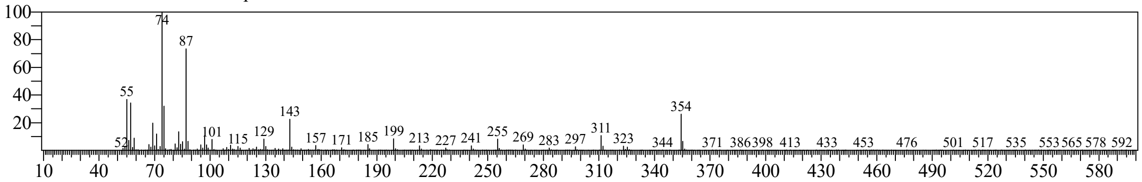


Hit#:1 Entry:196443 Library:NIST17-1.lib
SI:96 Formula:C23H46O2 CAS:929-77-1 MolWeight:354 RetIndex:2475
CompName:Docosanoic acid, methyl ester $$ Behenic acid, methyl ester $$ Methyl behenate $$ Methyl docosanoate $$ n-Docosanoic acid methyl ester $$

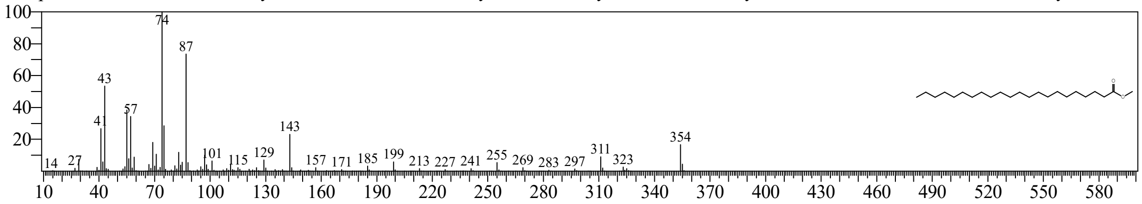


Hit#:2 Entry:178051 Library:NIST14.lib
SI:96 Formula:C23H46O2 CAS:929-77-1 MolWeight:354 RetIndex:2475
CompName:Docosanoic acid, methyl ester $$ Behenic acid, methyl ester $$ Methyl behenate $$ Methyl docosanoate $$ n-Docosanoic acid methyl ester $$

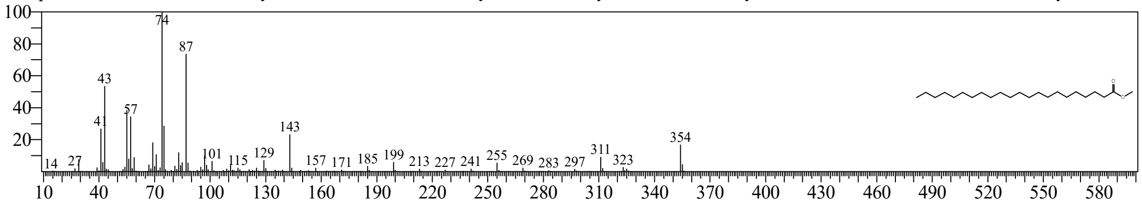


Hit#:3 Entry:196444 Library:NIST17-1.lib
SI:95 Formula:C23H46O2 CAS:0-00-0 MolWeight:354 RetIndex:2411
CompName:Methyl 20-methyl-heneicosanoate

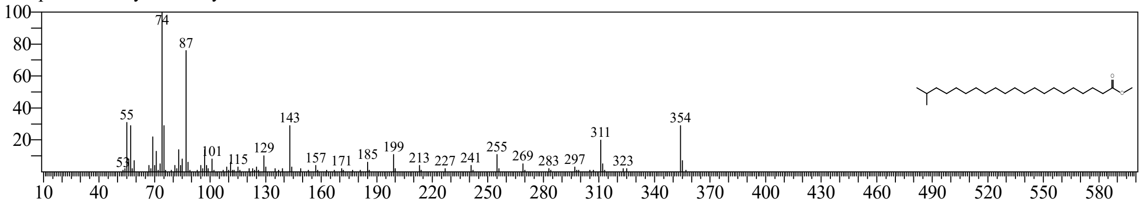


Hit#:4 Entry:178052 Library:NIST14.lib
SI:95 Formula:C23H46O2 CAS:0-00-0 MolWeight:354 RetIndex:2411
CompName:Methyl 20-methyl-heneicosanoate

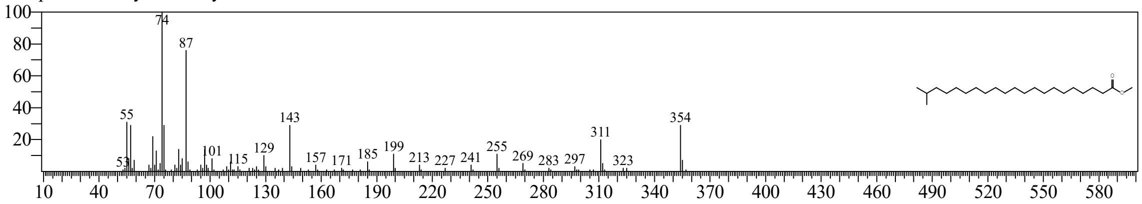


Hit#:5 Entry:31088 Library:NIST14s.lib
SI:95 Formula:C23H46O2 CAS:929-77-1 MolWeight:354 RetIndex:2475
CompName:Docosanoic acid, methyl ester $$ Behenic acid, methyl ester $$ Methyl behenate $$ Methyl docosanoate $$ n-Docosanoic acid methyl ester $$

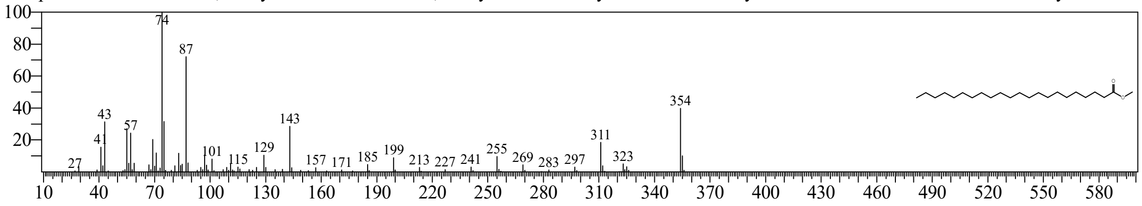

<< Target >>
Line#:22 R.Time:37.817(Scan#:3339) MassPeaks:328
RawMode:Averaged 37.808-37.825(3338-3340) BasePeak:74.00(3691)
BG Mode:Calc. from Peak Group 1 - Event 1

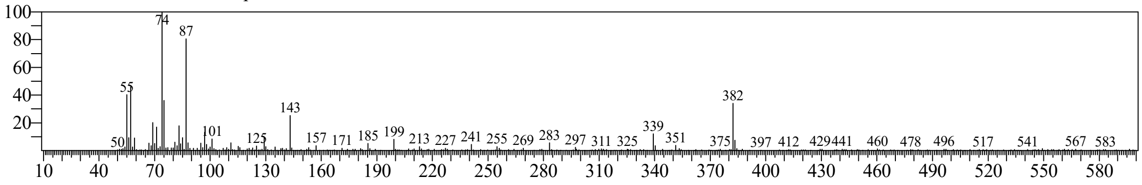


Hit#:1 Entry:31970 Library:NIST14s.lib
SI:95 Formula:C25H50O2 CAS:2442-49-1 MolWeight:382 RetIndex:2674
CompName:Tetracosanoic acid, methyl ester $$ Methyl lignocerate $$ Methyl tetracosanoate $$ Lignoceric acid methyl ester $$

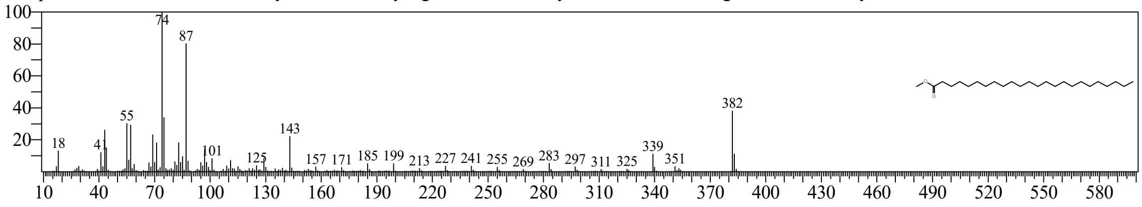


Hit#:2 Entry:37106 Library:NIST17s.lib
SI:95 Formula:C25H50O2 CAS:2442-49-1 MolWeight:382 RetIndex:2674
CompName:Tetracosanoic acid, methyl ester $$ Methyl lignocerate $$ Methyl tetracosanoate $$ Lignoceric acid methyl ester $$

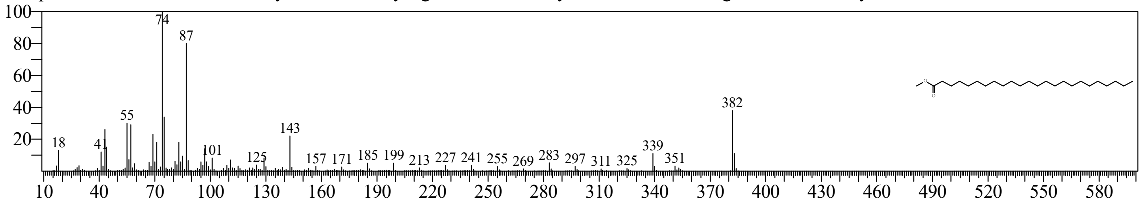


Hit#:3 Entry:31971 Library:NIST14s.lib
SI:92 Formula:C25H50O2 CAS:2442-49-1 MolWeight:382 RetIndex:2674
CompName:Tetracosanoic acid, methyl ester $$ Methyl lignocerate $$ Methyl tetracosanoate $$ Lignoceric acid methyl ester $$

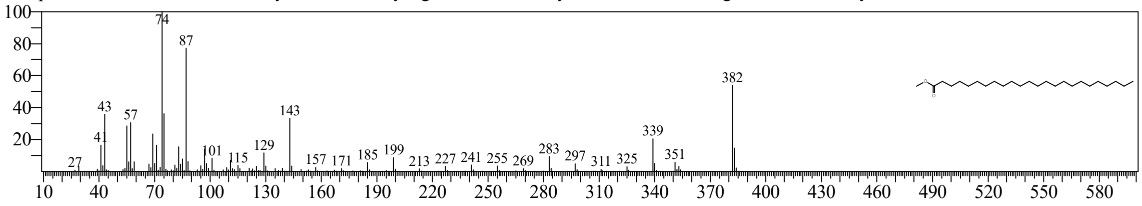


Hit#:4 Entry:37107 Library:NIST17s.lib
SI:92 Formula:C25H50O2 CAS:2442-49-1 MolWeight:382 RetIndex:2674
CompName:Tetracosanoic acid, methyl ester $$ Methyl lignocerate $$ Methyl tetracosanoate $$ Lignoceric acid methyl ester $$

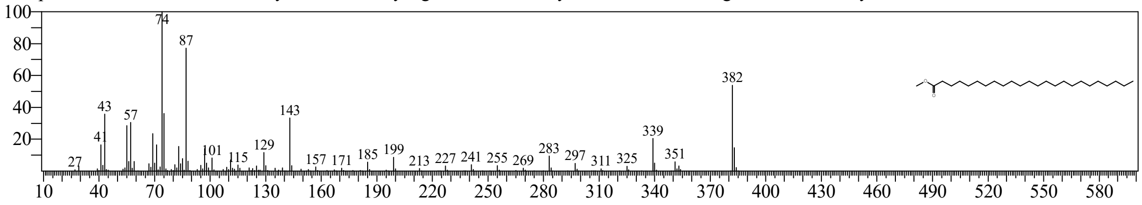


Hit#:5 Entry:37105 Library:NIST17s.lib
SI:91 Formula:C25H50O2 CAS:2442-49-1 MolWeight:382 RetIndex:2674
CompName:Tetracosanoic acid, methyl ester $$ Methyl lignocerate $$ Methyl tetracosanoate $$ Lignoceric acid methyl ester $$

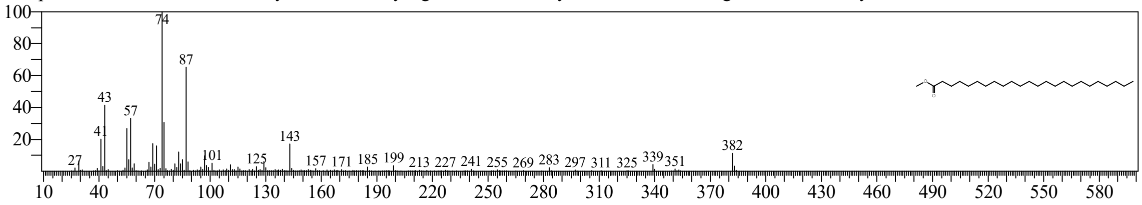

Supplement: Supplementary file 1 [file molecules-31-02891-s001.zip › 0d-QG100-2.pdf]
