# Supplementary material for: Selective Inhibition of Lipid Oxidation by a Quercetagetin-Rich Extract from Tagetes erecta: A Chemometric Approach Using OPLS-DA and Selectivity Index Analysis
Source: Molecules. 2026 Aug 19;31(16):2891. doi: 10.3390/molecules31162891 (PMC13515886; doi:10.3390/molecules31162891)

CK-2-14d

<<Column>>
Name : HP-88
Thickness : 0.20um
Length : 100.0m
Inside Diameter : 0.25mm

Method

===== Analytical Line 1 =====

| | | |
|---|---|---|
| Column Oven Temp. | :120.0 °C | |
| Injection Temp. | :250.00 °C | |
| Injection Mode | :Split | |
| Column Flow | :1.04 mL/min | |
| Split Ratio | :20.0 | |
| Oven Temp. Program | | |
| Rate | Temperature(°C) | Hold Time(min) |
| - | 120.0 | 1.00 |
| 10.00 | 175.0 | 10.00 |
| 5.00 | 210.0 | 5.00 |
| 5.00 | 230.0 | 15.00 |

[GCMS-QP2010]
IonSourceTemp :200.00 °C
Interface Temp. :220.00 °C
*m/z* scanning range：45～500m/z

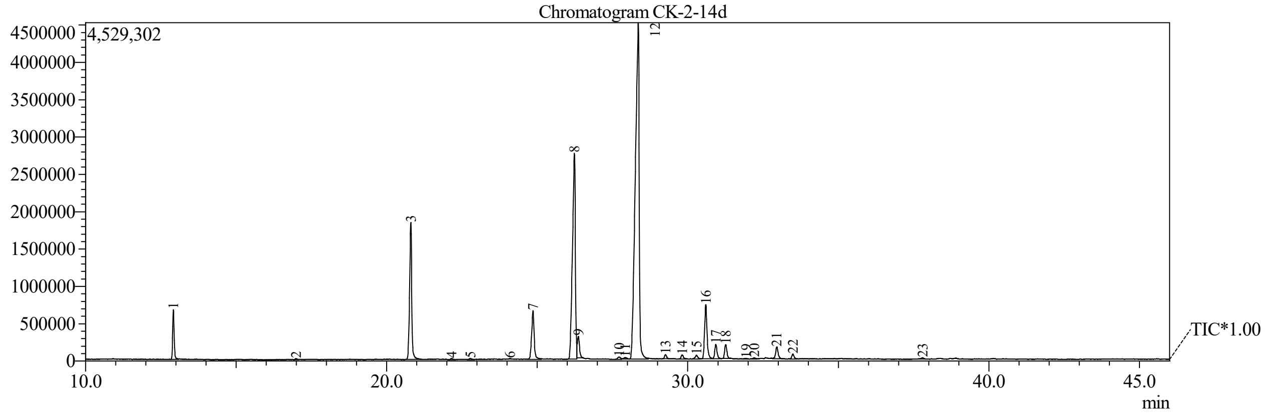


Peak Report TIC

| Peak# | R.Time | Area | Area% | Height | Height% | A/H | Name |
|---|---|---|---|---|---|---|---|
| 1 | 12.919 | 2310805 | 2.74 | 666450 | 5.40 | 3.47 | Undecanoic acid, methyl ester |
| 2 | 16.990 | 67780 | 0.08 | 14076 | 0.11 | 4.82 | Methyl tetradecanoate |
| 3 | 20.805 | 9072971 | 10.74 | 1834912 | 14.86 | 4.94 | Hexadecanoic acid, methyl ester |
| 4 | 22.158 | 80752 | 0.10 | 17758 | 0.14 | 4.55 | 7-Hexadecenoic acid, methyl ester, (Z)- |
| 5 | 22.781 | 112240 | 0.13 | 18487 | 0.15 | 6.07 | Heptadecanoic acid, methyl ester |
| 6 | 24.092 | 42125 | 0.05 | 9651 | 0.08 | 4.36 | Methyl 9-heptadecenoate or 9-17:1 |
| 7 | 24.861 | 3844552 | 4.55 | 651100 | 5.27 | 5.90 | Methyl stearate |
| 8 | 26.236 | 18303696 | 21.67 | 2757696 | 22.33 | 6.64 | 9-Octadecenoic acid, methyl ester, (E)- |
| 9 | 26.367 | 1423676 | 1.69 | 286734 | 2.32 | 4.97 | cis-13-Octadecenoic acid, methyl ester |
| 10 | 27.724 | 212632 | 0.25 | 31765 | 0.26 | 6.69 | 9,12-Octadecadienoic acid, methyl ester |
| 11 | 27.926 | 155488 | 0.18 | 21297 | 0.17 | 7.30 | 9,12-Octadecadienoic acid (Z,Z)-, methyl estei |
| 12 | 28.355 | 40243103 | 47.64 | 4502525 | 36.46 | 8.94 | 9,12-Octadecadienoic acid (Z,Z)-, methyl estei |
| 13 | 29.258 | 279918 | 0.33 | 54738 | 0.44 | 5.11 | Methyl 18-methylnonadecanoate |
| 14 | 29.812 | 312003 | 0.37 | 54792 | 0.44 | 5.69 | 11,14,17-Eicosatrienoic acid, methyl ester |

| Peak# | R.Time | Area | Area% | Height | Height% | A/H | Name |
|---|---|---|---|---|---|---|---|
| 15 | 30.289 | 327626 | 0.39 | 52255 | 0.42 | 6.27 | 9,12,15-Octadecatrienoic acid, methyl ester, (Z |
| 16 | 30.597 | 4179450 | 4.95 | 726571 | 5.88 | 5.75 | 9,12,15-Octadecatrienoic acid, methyl ester, (Z |
| 17 | 30.931 | 994819 | 1.18 | 190750 | 1.54 | 5.22 | Methyl 9-cis,11-trans-octadecadienoate |
| 18 | 31.259 | 976282 | 1.16 | 187343 | 1.52 | 5.21 | Methyl 10-trans,12-cis-octadecadienoate |
| 19 | 31.940 | 46120 | 0.05 | 8946 | 0.07 | 5.16 | 9,11-Octadecadienoic acid, methyl ester, (E,E) |
| 20 | 32.206 | 82958 | 0.10 | 14589 | 0.12 | 5.69 | Methyl 6-cis,9-cis,11-trans-octadecatrienoate |
| 21 | 32.955 | 938388 | 1.11 | 160359 | 1.30 | 5.85 | Cis-3-ethyl-endo-tricyclo[5.2.1.0(2.6)]decane |
| 22 | 33.491 | 343361 | 0.41 | 67248 | 0.54 | 5.11 | Docosanoic acid, methyl ester |
| 23 | 37.799 | 116250 | 0.14 | 18651 | 0.15 | 6.23 | Tetracosanoic acid, methyl ester |
| | | 84466995 | 100.00 | 12348693 | 100.00 | | |

Library

<< Target >>

Line#:1 R.Time:12.917(Scan#:351) MassPeaks:389

RawMode:Averaged 12.908-12.925(350-352) BasePeak:74.00(192889)

BG Mode:Calc. from Peak Group 1 - Event 1

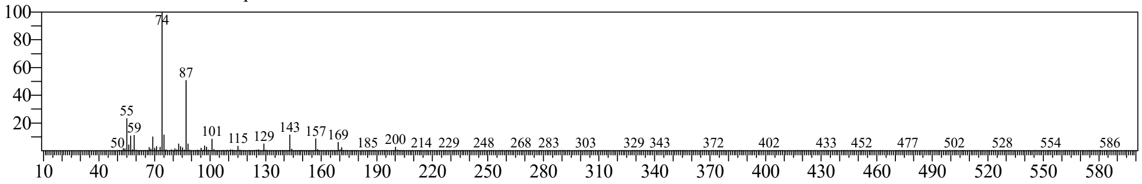

Hit#:1 Entry:49598 Library:NIST17-1.lib

SI:95 Formula:C12H24O2 CAS:1731-86-8 MolWeight:200 RetIndex:1381

CompName:Undecanoic acid, methyl ester $$ Methyl undecanoate $$ Methyl undecylenate $$ n-Undecanoic acid methyl ester $$ Methyl ester of undecanoi

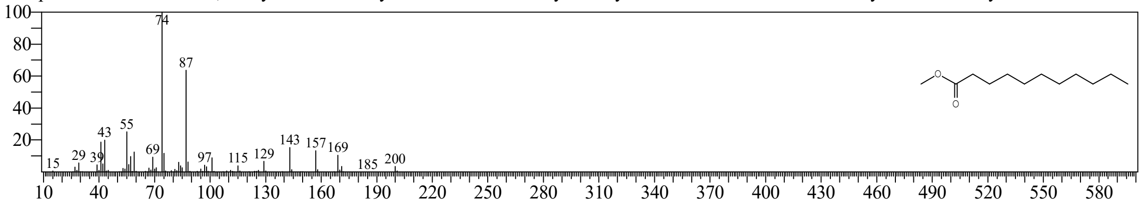

Hit#:2 Entry:46966 Library:NIST14.lib

SI:95 Formula:C12H24O2 CAS:1731-86-8 MolWeight:200 RetIndex:1381

CompName:Undecanoic acid, methyl ester $$ Methyl undecanoate $$ Methyl undecylenate $$ n-Undecanoic acid methyl ester $$ Methyl ester of undecanoi

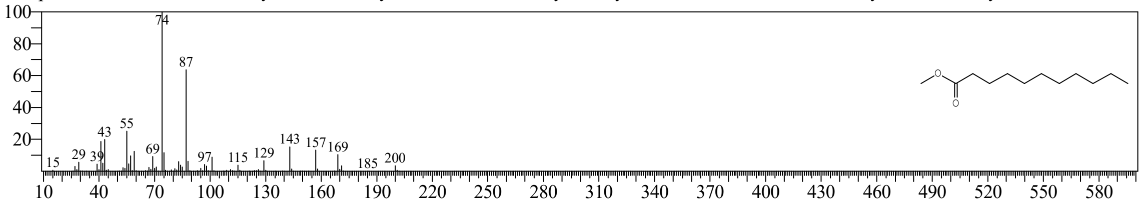

Hit#:3 Entry:21618 Library:NIST17s.lib

SI:95 Formula:C12H24O2 CAS:1731-86-8 MolWeight:200 RetIndex:1381

CompName:Undecanoic acid, methyl ester $$ Methyl undecanoate $$ Methyl undecylenate $$ n-Undecanoic acid methyl ester $$ Methyl ester of undecanoi

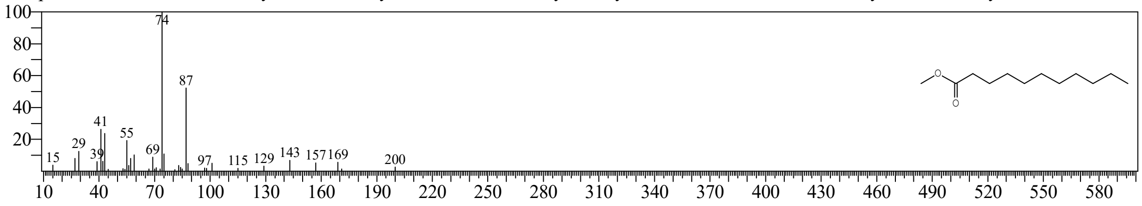

Hit#:4 Entry:21619 Library:NIST17s.lib

SI:95 Formula:C12H24O2 CAS:1731-86-8 MolWeight:200 RetIndex:1381

CompName:Undecanoic acid, methyl ester $$ Methyl undecanoate $$ Methyl undecylenate $$ n-Undecanoic acid methyl ester $$ Methyl ester of undecanoi

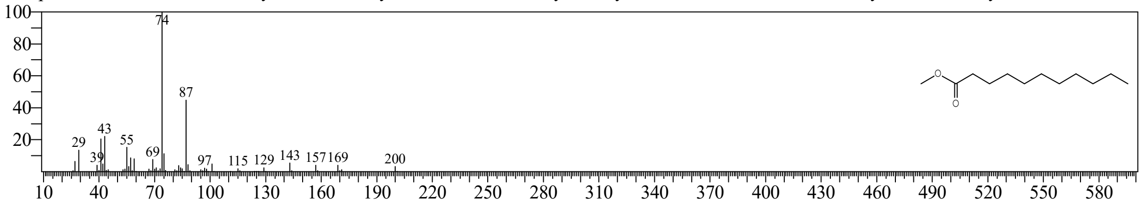

<< Target >>
Line#:1   R.Time:12.917(Scan#:351)  MassPeaks:389
RawMode:Averaged 12.908-12.925(350-352) BasePeak:74.00(192889)
BG Mode:Calc. from Peak  Group 1 - Event 1

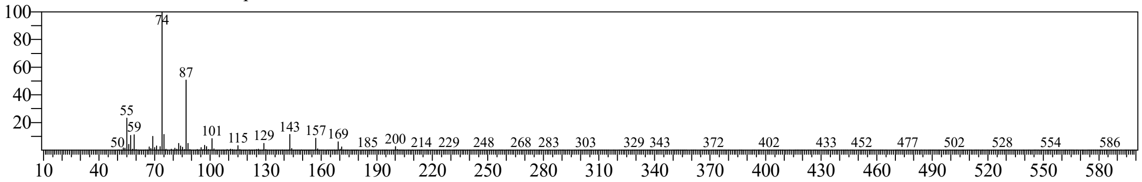


Hit#:5  Entry:18487  Library:NIST14s.lib
SI:95  Formula:C12H24O2  CAS:1731-86-8  MolWeight:200  RetIndex:1381
CompName:Undecanoic acid, methyl ester $$ Methyl undecanoate $$ Methyl undecylenate $$ n-Undecanoic acid methyl ester $$ Methyl ester of undecanoi

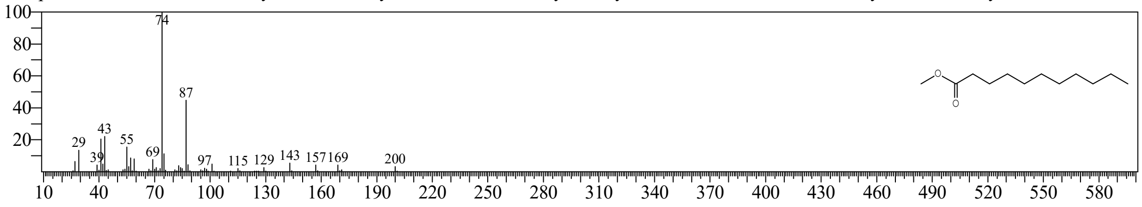

<< Target >>
Line#:2   R.Time:16.992(Scan#:840)  MassPeaks:338
RawMode:Averaged 16.983-17.000(839-841) BasePeak:74.00(2853)
BG Mode:Calc. from Peak  Group 1 - Event 1

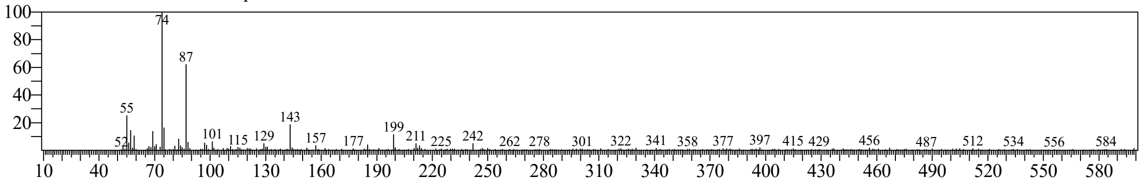


Hit#:1  Entry:80645  Library:NIST14.lib
SI:94  Formula:C15H30O2  CAS:124-10-7  MolWeight:242  RetIndex:1680
CompName:Methyl tetradecanoate $$ Tetradecanoic acid, methyl ester $$ Myristic acid, methyl ester $$ Metholeneat 2495 $$ Methyl myristate $$ Methyl r

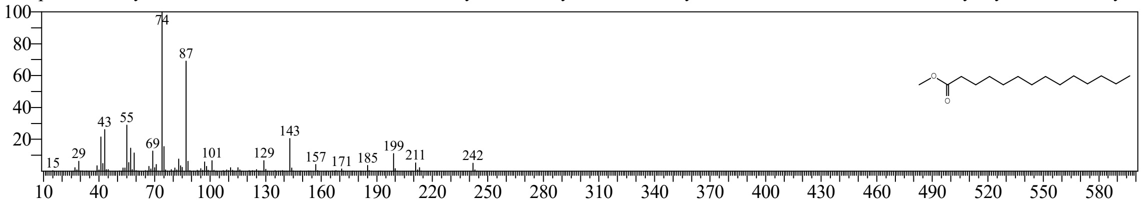


Hit#:2  Entry:86786  Library:NIST17-1.lib
SI:94  Formula:C15H30O2  CAS:124-10-7  MolWeight:242  RetIndex:1680
CompName:Methyl tetradecanoate $$ Tetradecanoic acid, methyl ester $$ Myristic acid, methyl ester $$ Metholeneat 2495 $$ Methyl myristate $$ Methyl r

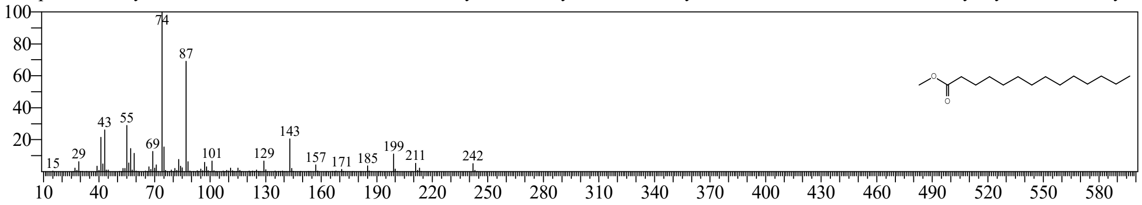


Hit#:3  Entry:27874  Library:NIST17s.lib
SI:92  Formula:C15H30O2  CAS:124-10-7  MolWeight:242  RetIndex:1680
CompName:Methyl tetradecanoate $$ Tetradecanoic acid, methyl ester $$ Myristic acid, methyl ester $$ Metholeneat 2495 $$ Methyl myristate $$ Methyl r

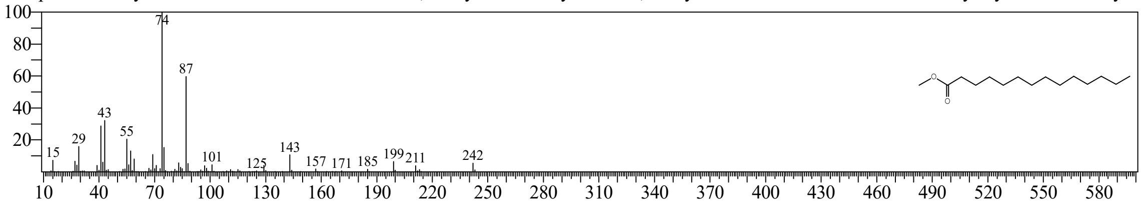


Hit#:4  Entry:23848  Library:NIST14s.lib
SI:92  Formula:C15H30O2  CAS:124-10-7  MolWeight:242  RetIndex:1680
CompName:Methyl tetradecanoate $$ Tetradecanoic acid, methyl ester $$ Myristic acid, methyl ester $$ Metholeneat 2495 $$ Methyl myristate $$ Methyl r

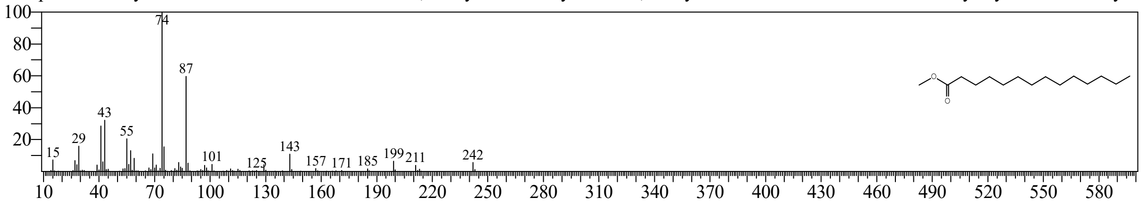


Hit#:5  Entry:27873  Library:NIST17s.lib
SI:92  Formula:C15H30O2  CAS:124-10-7  MolWeight:242  RetIndex:1680
CompName:Methyl tetradecanoate $$ Tetradecanoic acid, methyl ester $$ Myristic acid, methyl ester $$ Metholeneat 2495 $$ Methyl myristate $$ Methyl r

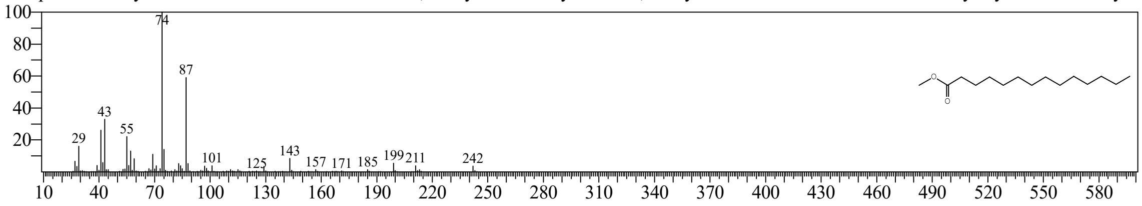

<< Target >>
Line#:3 R.Time:20.808(Scan#:1298) MassPeaks:412
RawMode:Averaged 20.800-20.817(1297-1299) BasePeak:74.00(425048)
BG Mode:Calc. from Peak Group 1 - Event 1

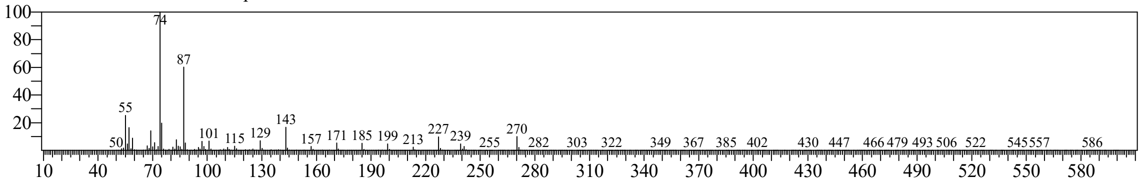

Hit#:1 Entry:113663 Library:NIST17-1.lib
SI:97 Formula:C17H34O2 CAS:112-39-0 MolWeight:270 RetIndex:1878
CompName:Hexadecanoic acid, methyl ester $$ Palmitic acid, methyl ester $$ n-Hexadecanoic acid methyl ester $$ Metholene 2216 $$ Methyl hexadecano

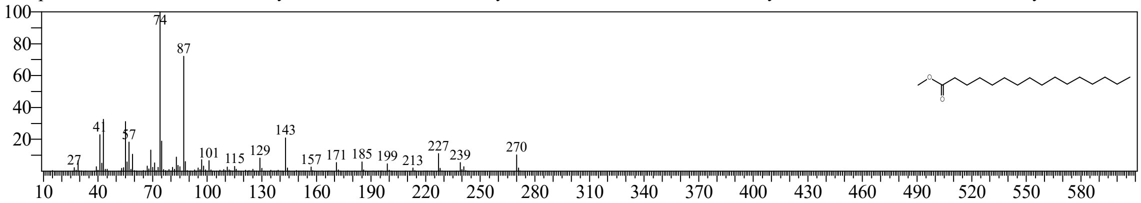

Hit#:2 Entry:104648 Library:NIST14.lib
SI:97 Formula:C17H34O2 CAS:112-39-0 MolWeight:270 RetIndex:1878
CompName:Hexadecanoic acid, methyl ester $$ Palmitic acid, methyl ester $$ n-Hexadecanoic acid methyl ester $$ Metholene 2216 $$ Methyl hexadecano

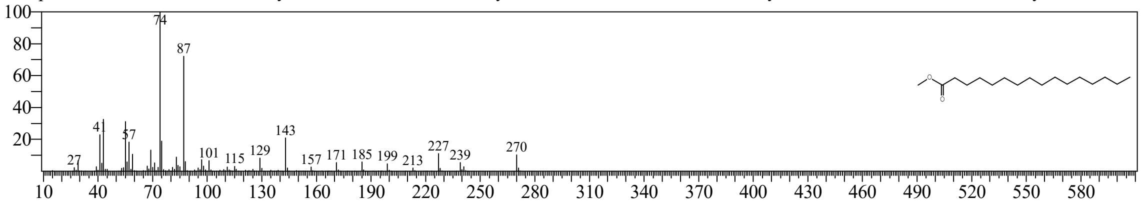

Hit#:3 Entry:26273 Library:NIST14s.lib
SI:96 Formula:C17H34O2 CAS:112-39-0 MolWeight:270 RetIndex:1878
CompName:Hexadecanoic acid, methyl ester $$ Palmitic acid, methyl ester $$ n-Hexadecanoic acid methyl ester $$ Metholene 2216 $$ Methyl hexadecano

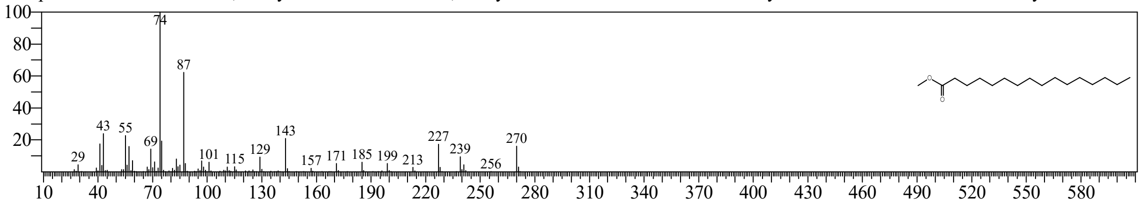

Hit#:4 Entry:26271 Library:NIST14s.lib
SI:96 Formula:C17H34O2 CAS:112-39-0 MolWeight:270 RetIndex:1878
CompName:Hexadecanoic acid, methyl ester $$ Palmitic acid, methyl ester $$ n-Hexadecanoic acid methyl ester $$ Metholene 2216 $$ Methyl hexadecano

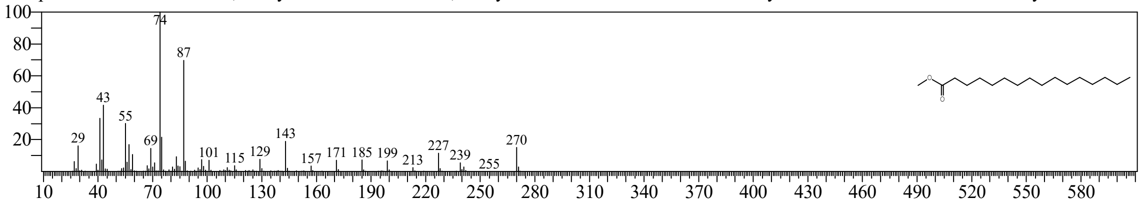

Hit#:5 Entry:30646 Library:NIST17s.lib
SI:96 Formula:C17H34O2 CAS:112-39-0 MolWeight:270 RetIndex:1878
CompName:Hexadecanoic acid, methyl ester $$ Palmitic acid, methyl ester $$ n-Hexadecanoic acid methyl ester $$ Metholene 2216 $$ Methyl hexadecano

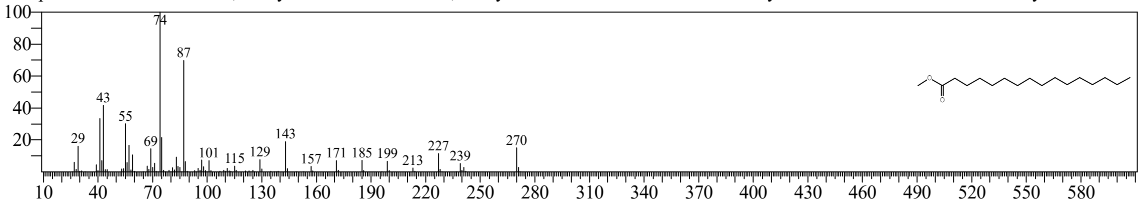

<< Target >>
Line#:4  R.Time:22.158(Scan#:1460)  MassPeaks:377
RawMode:Averaged 22.150-22.167(1459-1461) BasePeak:55.05(1244)
BG Mode:Calc. from Peak  Group 1 - Event 1

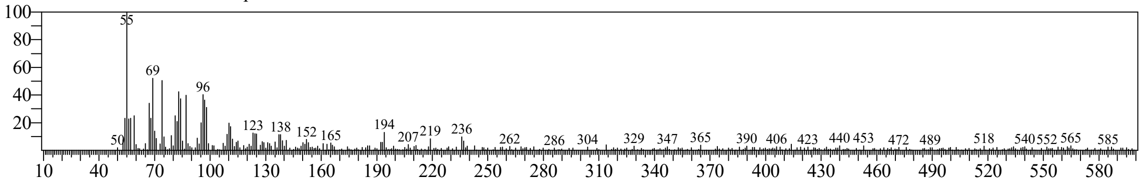

Hit#:1  Entry:111510  Library:NIST17-1.lib
SI:92  Formula:C17H32O2  CAS:56875-67-3  MolWeight:268  RetIndex:1886
CompName:7-Hexadecenoic acid, methyl ester, (Z)- $$ Methyl (7E)-7-hexadecenoate # $$

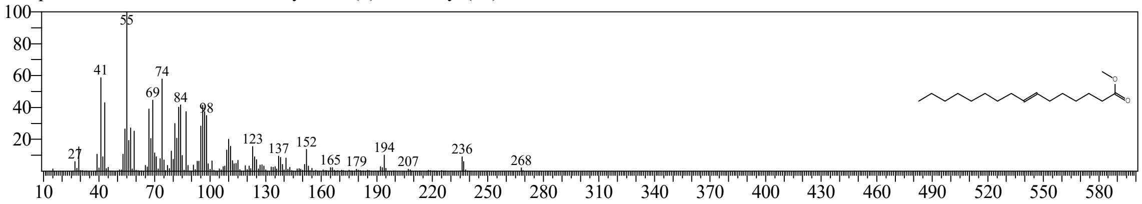

Hit#:2  Entry:30415  Library:NIST17s.lib
SI:92  Formula:C17H32O2  CAS:1120-25-8  MolWeight:268  RetIndex:1886
CompName:9-Hexadecenoic acid, methyl ester, (Z)- $$ Methyl palmitoleate $$ Methyl palmitoleinate $$ Palmitoleic acid, methyl ester $$ Methyl (9Z)-9-he:

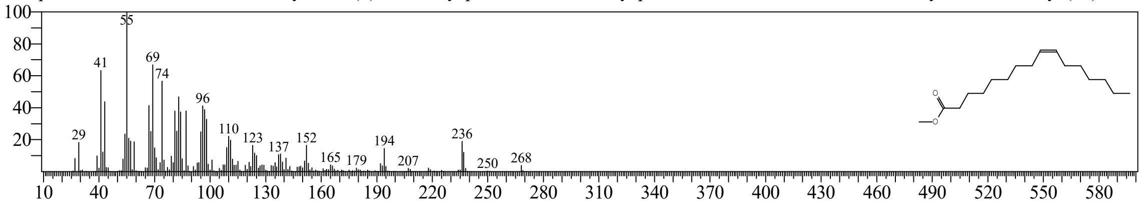

Hit#:3  Entry:26066  Library:NIST14s.lib
SI:92  Formula:C17H32O2  CAS:1120-25-8  MolWeight:268  RetIndex:1886
CompName:9-Hexadecenoic acid, methyl ester, (Z)- $$ Methyl palmitoleate $$ Methyl palmitoleinate $$ Palmitoleic acid, methyl ester $$ Methyl (9Z)-9-he:

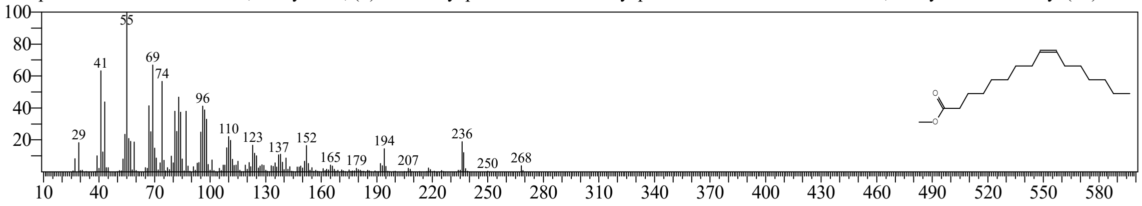

Hit#:4  Entry:30413  Library:NIST17s.lib
SI:91  Formula:C17H32O2  CAS:1120-25-8  MolWeight:268  RetIndex:1886
CompName:9-Hexadecenoic acid, methyl ester, (Z)- $$ Methyl palmitoleate $$ Methyl palmitoleinate $$ Palmitoleic acid, methyl ester $$ Methyl (9Z)-9-he:

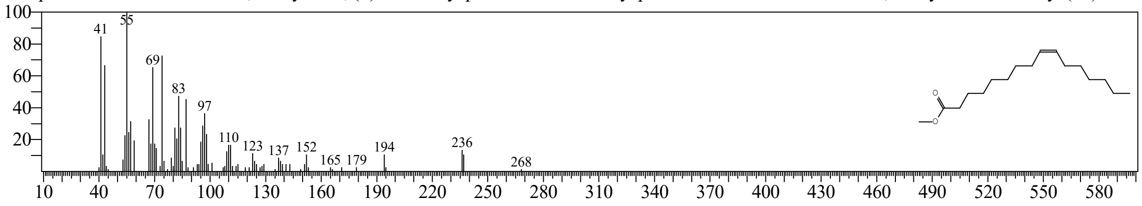

Hit#:5  Entry:26064  Library:NIST14s.lib
SI:91  Formula:C17H32O2  CAS:1120-25-8  MolWeight:268  RetIndex:1886
CompName:9-Hexadecenoic acid, methyl ester, (Z)- $$ Methyl palmitoleate $$ Methyl palmitoleinate $$ Palmitoleic acid, methyl ester $$ Methyl (9Z)-9-he:

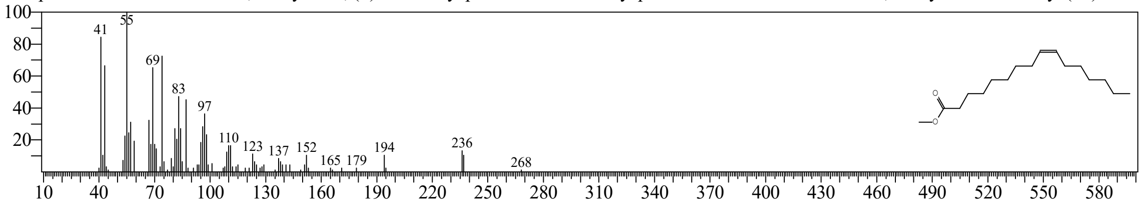

<< Target >>
Line#:5  R.Time:22.783(Scan#:1535)  MassPeaks:309
RawMode:Averaged 22.775-22.792(1534-1536) BasePeak:74.00(3716)
BG Mode:Calc. from Peak  Group 1 - Event 1

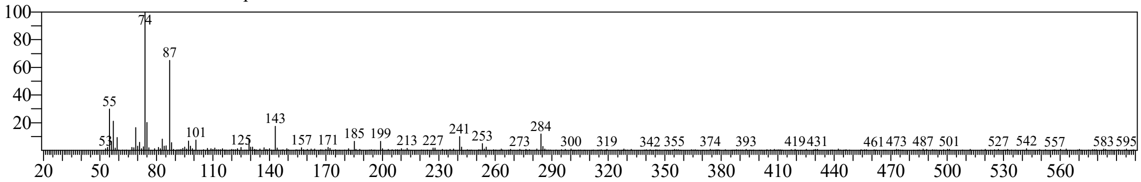


Hit#:1  Entry:27274  Library:NIST14s.lib
SI:94  Formula:C18H36O2  CAS:1731-92-6  MolWeight:284  RetIndex:1978
CompName:Heptadecanoic acid, methyl ester $$ Margaric acid methyl ester $$ Methyl heptadecanoate $$ Methyl margarate $$ n-Heptadecanoic acid methy

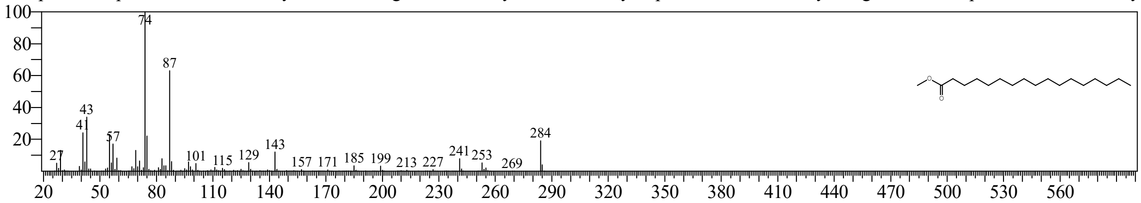


Hit#:2  Entry:27275  Library:NIST14s.lib
SI:94  Formula:C18H36O2  CAS:1731-92-6  MolWeight:284  RetIndex:1978
CompName:Heptadecanoic acid, methyl ester $$ Margaric acid methyl ester $$ Methyl heptadecanoate $$ Methyl margarate $$ n-Heptadecanoic acid methy

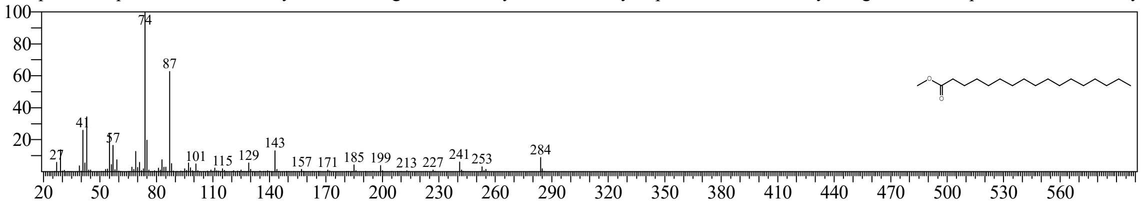


Hit#:3  Entry:31784  Library:NIST17s.lib
SI:94  Formula:C18H36O2  CAS:1731-92-6  MolWeight:284  RetIndex:1978
CompName:Heptadecanoic acid, methyl ester $$ Margaric acid methyl ester $$ Methyl heptadecanoate $$ Methyl margarate $$ n-Heptadecanoic acid methy

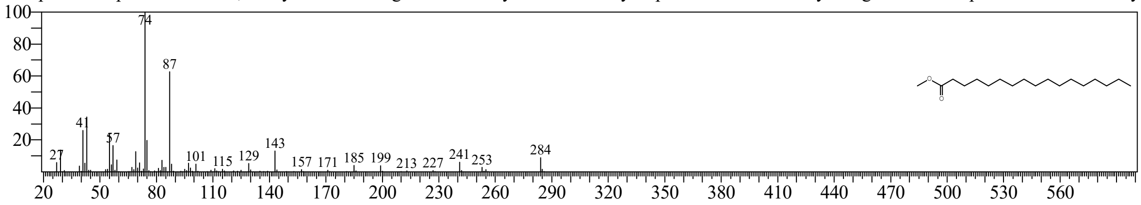


Hit#:4  Entry:31783  Library:NIST17s.lib
SI:94  Formula:C18H36O2  CAS:1731-92-6  MolWeight:284  RetIndex:1978
CompName:Heptadecanoic acid, methyl ester $$ Margaric acid methyl ester $$ Methyl heptadecanoate $$ Methyl margarate $$ n-Heptadecanoic acid methy

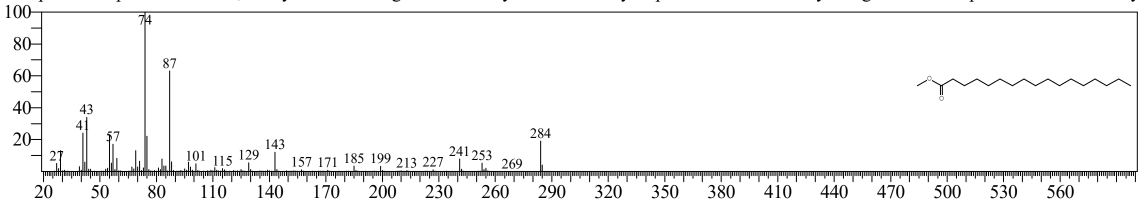


Hit#:5  Entry:127714  Library:NIST17-1.lib
SI:93  Formula:C18H36O2  CAS:6929-04-0  MolWeight:284  RetIndex:1914
CompName:Hexadecanoic acid, 15-methyl-, methyl ester $$ Methyl isoheptadecanoate $$ Methyl 15-methylhexadecanoate $$

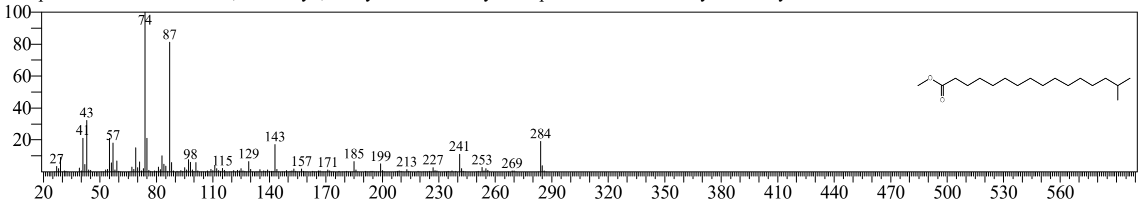

<< Target >>
Line#:6 R.Time:24.092(Scan#:1692) MassPeaks:332
RawMode:Averaged 24.083-24.100(1691-1693) BasePeak:55.05(707)
BG Mode:Calc. from Peak Group 1 - Event 1

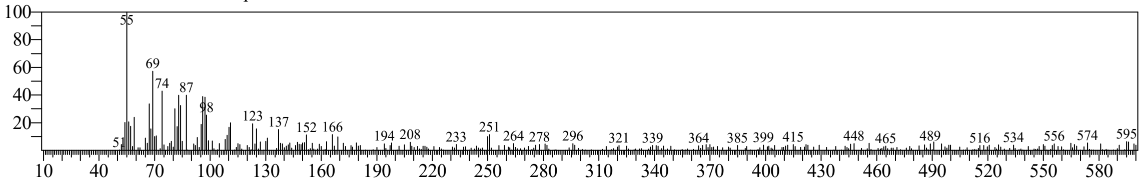


Hit#:1 Entry:125440 Library:NIST17-1.lib
SI:87 Formula:C18H34O2 CAS:0-00-0 MolWeight:282 RetIndex:1986
CompName:Methyl 9-heptadecenoate or 9-17:1

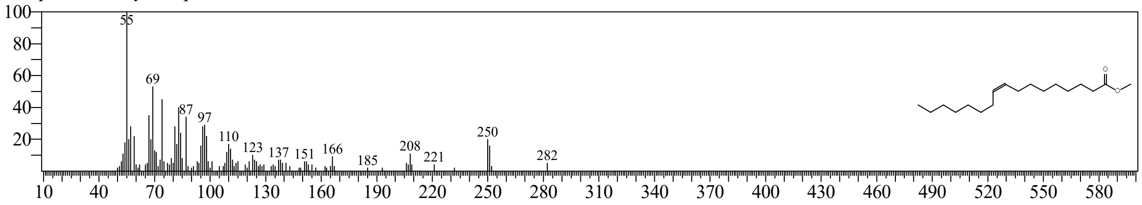


Hit#:2 Entry:115096 Library:NIST14.lib
SI:87 Formula:C18H34O2 CAS:0-00-0 MolWeight:282 RetIndex:1986
CompName:Methyl 9-heptadecenoate or 9-17:1

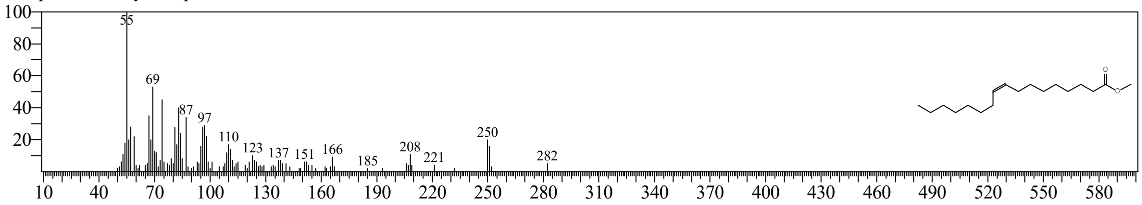


Hit#:3 Entry:115094 Library:NIST14.lib
SI:87 Formula:C18H34O2 CAS:0-00-0 MolWeight:282 RetIndex:1986
CompName:cis-10-Heptadecenoic acid, methyl ester

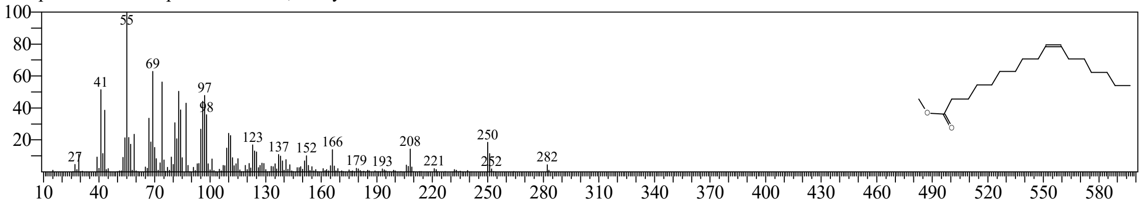


Hit#:4 Entry:125438 Library:NIST17-1.lib
SI:87 Formula:C18H34O2 CAS:0-00-0 MolWeight:282 RetIndex:1986
CompName:cis-10-Heptadecenoic acid, methyl ester

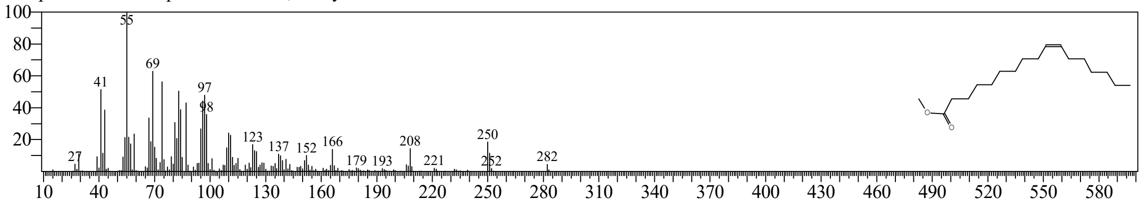


Hit#:5 Entry:31597 Library:NIST17s.lib
SI:85 Formula:C18H34O2 CAS:10152-61-1 MolWeight:282 RetIndex:1941
CompName:Cyclopropaneoctanoic acid, 2-hexyl-, methyl ester $$ Methyl 8-(2-hexylcyclopropyl)octanoate # $$

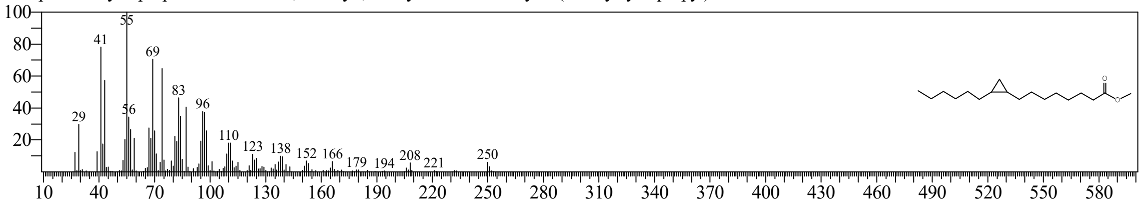

<< Target >>
Line#:7 R.Time:24.858(Scan#:1784) MassPeaks:400
RawMode:Averaged 24.850-24.867(1783-1785) BasePeak:74.00(129724)
BG Mode:Calc. from Peak Group 1 - Event 1

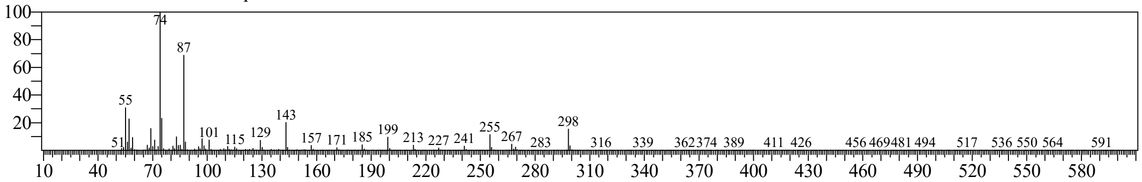


Hit#:1 Entry:129694 Library:NIST14.lib
SI:98 Formula:C19H38O2 CAS:112-61-8 MolWeight:298 RetIndex:2077
CompName:Methyl stearate $$ Octadecanoic acid, methyl ester $$ Stearic acid, methyl ester $$ n-Octadecanoic acid, methyl ester $$ Kemester 9718 $$ Me

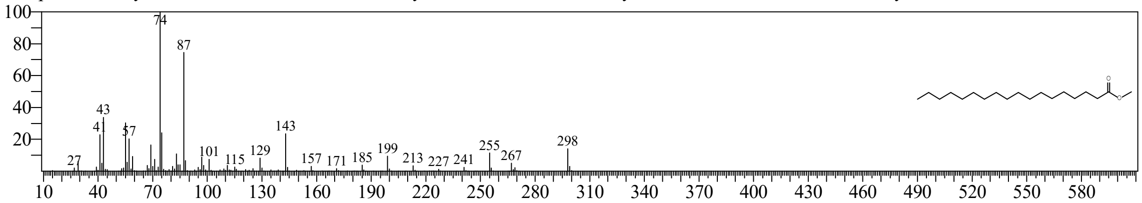


Hit#:2 Entry:141978 Library:NIST17-1.lib
SI:98 Formula:C19H38O2 CAS:112-61-8 MolWeight:298 RetIndex:2077
CompName:Methyl stearate $$ Octadecanoic acid, methyl ester $$ Stearic acid, methyl ester $$ n-Octadecanoic acid, methyl ester $$ Kemester 9718 $$ Me

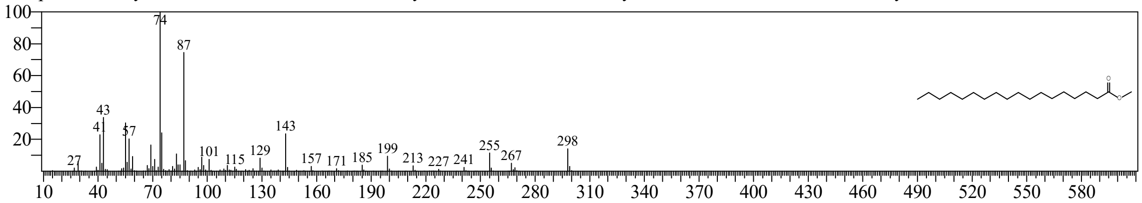


Hit#:3 Entry:28255 Library:NIST14s.lib
SI:97 Formula:C19H38O2 CAS:112-61-8 MolWeight:298 RetIndex:2077
CompName:Methyl stearate $$ Octadecanoic acid, methyl ester $$ Stearic acid, methyl ester $$ n-Octadecanoic acid, methyl ester $$ Kemester 9718 $$ Me

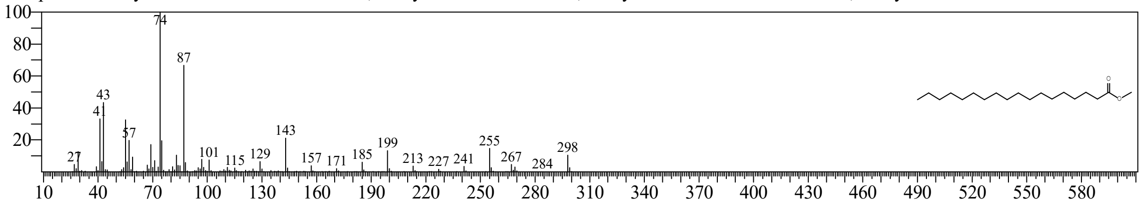


Hit#:4 Entry:32873 Library:NIST17s.lib
SI:97 Formula:C19H38O2 CAS:112-61-8 MolWeight:298 RetIndex:2077
CompName:Methyl stearate $$ Octadecanoic acid, methyl ester $$ Stearic acid, methyl ester $$ n-Octadecanoic acid, methyl ester $$ Kemester 9718 $$ Me

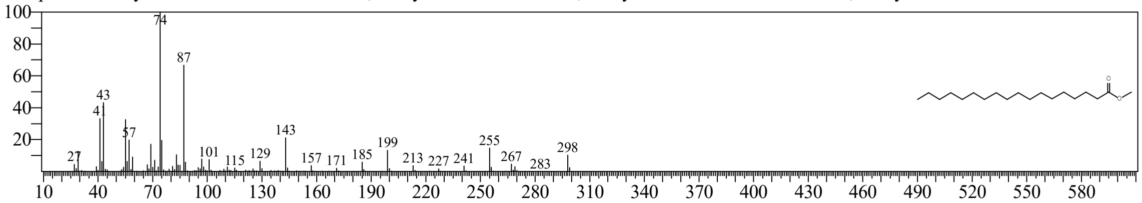


Hit#:5 Entry:28257 Library:NIST14s.lib
SI:96 Formula:C19H38O2 CAS:112-61-8 MolWeight:298 RetIndex:2077
CompName:Methyl stearate $$ Octadecanoic acid, methyl ester $$ Stearic acid, methyl ester $$ n-Octadecanoic acid, methyl ester $$ Kemester 9718 $$ Me

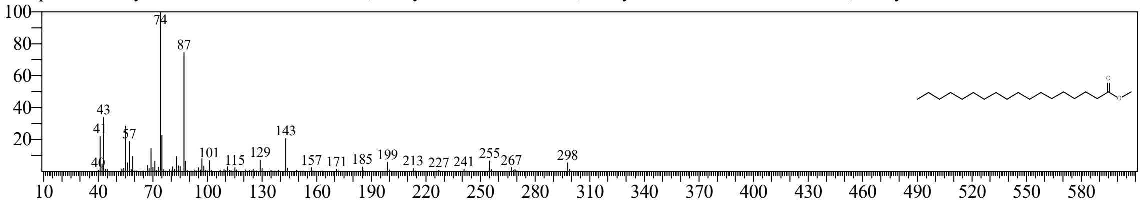

<< Target >>
Line#:8 R.Time:26.233(Scan#:1949) MassPeaks:416
RawMode:Averaged 26.225-26.242(1948-1950) BasePeak:55.05(212115)
BG Mode:Calc. from Peak Group 1 - Event 1

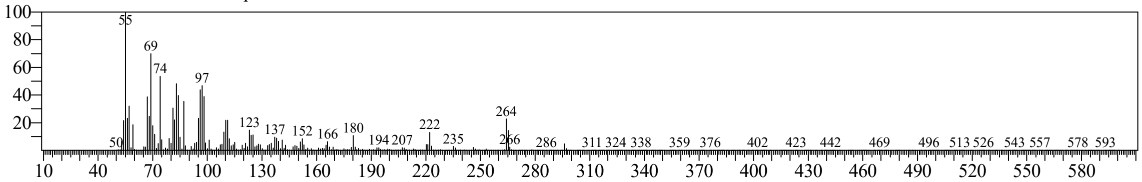


Hit#:1 Entry:32747 Library:NIST17s.lib
SI:96 Formula:C19H36O2 CAS:1937-62-8 MolWeight:296 RetIndex:2085
CompName:9-Octadecenoic acid, methyl ester, (E)- $$ Elaidic acid, methyl ester $$ Methyl elaidate $$ Methyl trans-9-octadecenoate $$ (E)-9-Octadecenoic

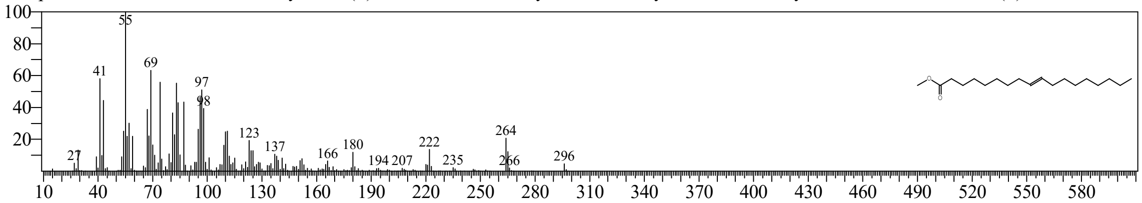


Hit#:2 Entry:28138 Library:NIST14s.lib
SI:96 Formula:C19H36O2 CAS:1937-62-8 MolWeight:296 RetIndex:2085
CompName:9-Octadecenoic acid, methyl ester, (E)- $$ Elaidic acid, methyl ester $$ Methyl elaidate $$ Methyl trans-9-octadecenoate $$ (E)-9-Octadecenoic

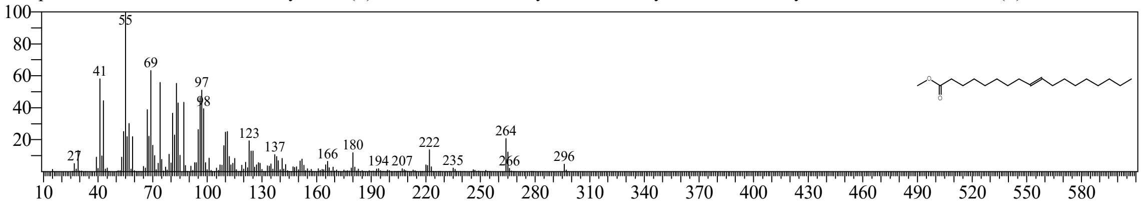


Hit#:3 Entry:28139 Library:NIST14s.lib
SI:96 Formula:C19H36O2 CAS:52380-33-3 MolWeight:296 RetIndex:2085
CompName:11-Octadecenoic acid, methyl ester $$ Methyl 11-octadecenoate $$ Octadec-11-enoic acid, methyl ester $$ Methyl (11E)-11-octadecenoate # $$

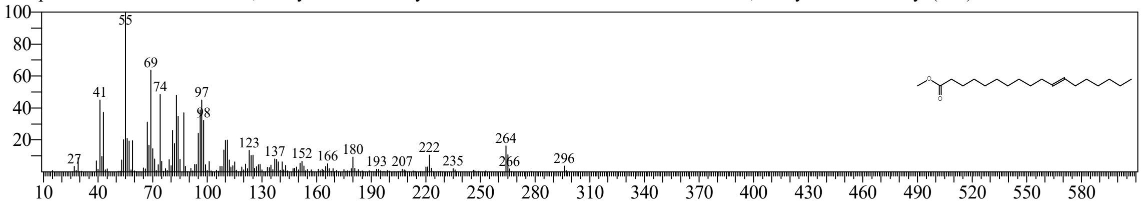


Hit#:4 Entry:32748 Library:NIST17s.lib
SI:96 Formula:C19H36O2 CAS:52380-33-3 MolWeight:296 RetIndex:2085
CompName:11-Octadecenoic acid, methyl ester $$ Methyl 11-octadecenoate $$ Octadec-11-enoic acid, methyl ester $$ Methyl (11E)-11-octadecenoate # $$

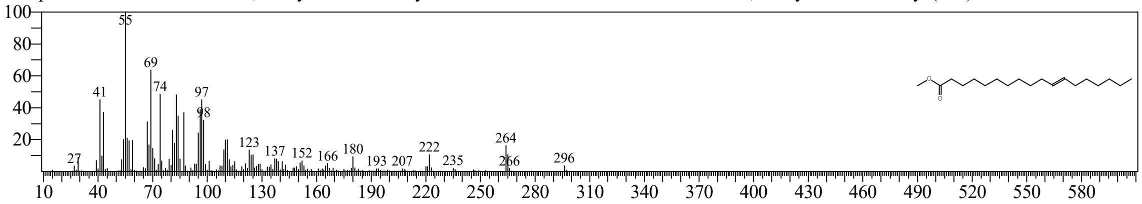


Hit#:5 Entry:139650 Library:NIST17-1.lib
SI:96 Formula:C19H36O2 CAS:112-62-9 MolWeight:296 RetIndex:2085
CompName:9-Octadecenoic acid (Z)-, methyl ester $$ Oleic acid, methyl ester $$ Emery oleic acid ester 2301 $$ Methyl cis-9-octadecenoate $$ Methyl olea

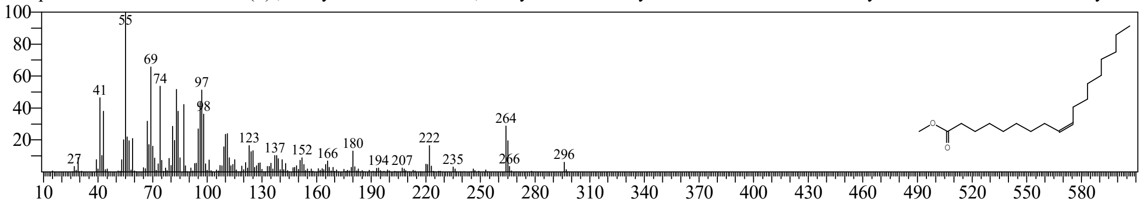

<< Target >>
Line#:9 R.Time:26.367(Scan#:1965) MassPeaks:350
RawMode:Averaged 26.358-26.375(1964-1966) BasePeak:55.05(15745)
BG Mode:Calc. from Peak Group 1 - Event 1

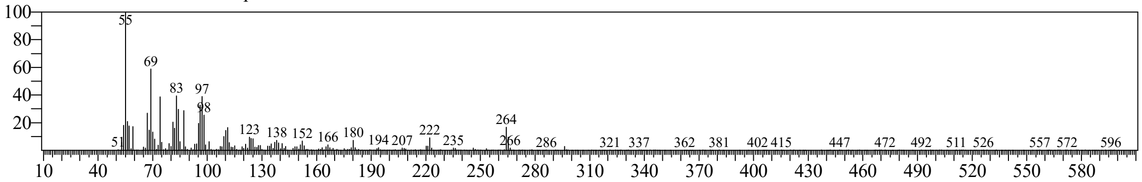


Hit#:1 Entry:127661 Library:NIST14.lib
SI:95 Formula:C19H36O2 CAS:0-00-0 MolWeight:296 RetIndex:2085
CompName:cis-13-Octadecenoic acid, methyl ester

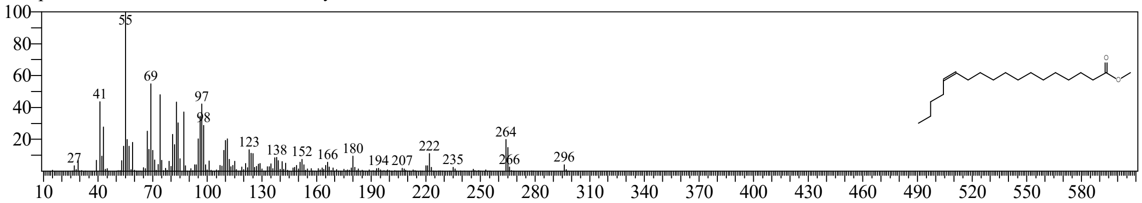


Hit#:2 Entry:28139 Library:NIST14s.lib
SI:95 Formula:C19H36O2 CAS:52380-33-3 MolWeight:296 RetIndex:2085
CompName:11-Octadecenoic acid, methyl ester $$ Methyl 11-octadecenoate $$ Octadec-11-enoic acid, methyl ester $$ Methyl (11E)-11-octadecenoate # $$

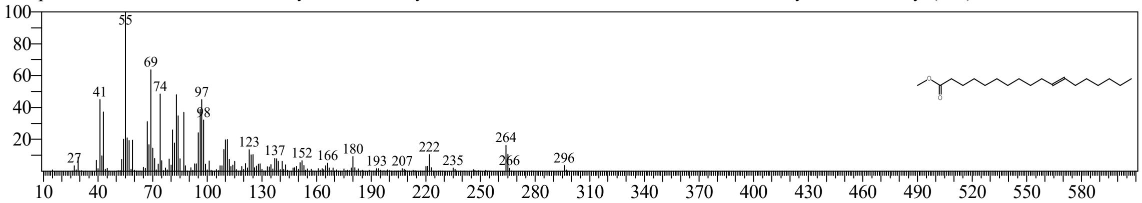


Hit#:3 Entry:32748 Library:NIST17s.lib
SI:95 Formula:C19H36O2 CAS:52380-33-3 MolWeight:296 RetIndex:2085
CompName:11-Octadecenoic acid, methyl ester $$ Methyl 11-octadecenoate $$ Octadec-11-enoic acid, methyl ester $$ Methyl (11E)-11-octadecenoate # $$

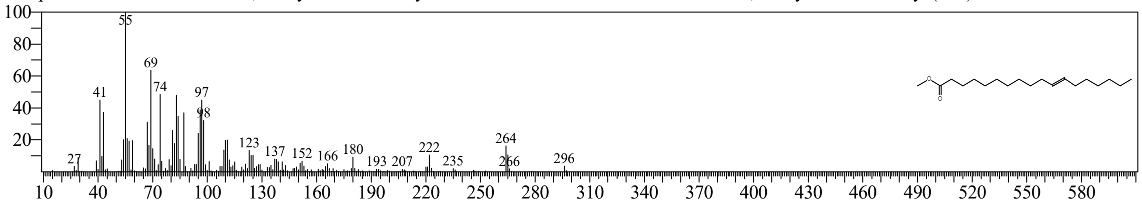


Hit#:4 Entry:139647 Library:NIST17-1.lib
SI:95 Formula:C19H36O2 CAS:0-00-0 MolWeight:296 RetIndex:2085
CompName:cis-13-Octadecenoic acid, methyl ester

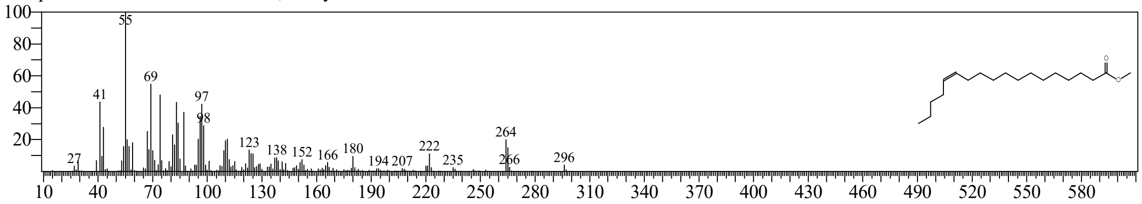


Hit#:5 Entry:127650 Library:NIST14.lib
SI:94 Formula:C19H36O2 CAS:13481-95-3 MolWeight:296 RetIndex:2085
CompName:10-Octadecenoic acid, methyl ester $$ Methyl 10-octadecenoate $$

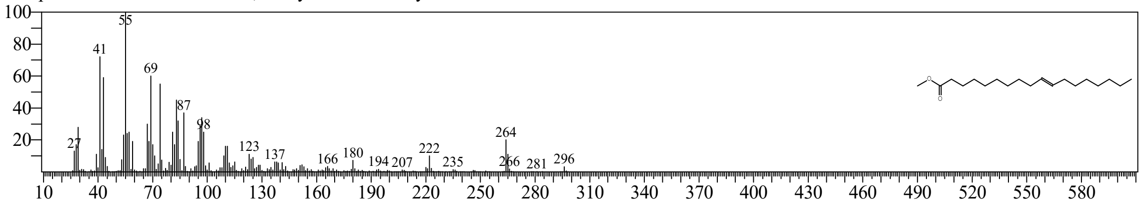

<< Target >>
Line#:10 R.Time:27.725(Scan#:2128) MassPeaks:370
RawMode:Averaged 27.717-27.733(2127-2129) BasePeak:67.05(2198)
BG Mode:Calc. from Peak Group 1 - Event 1

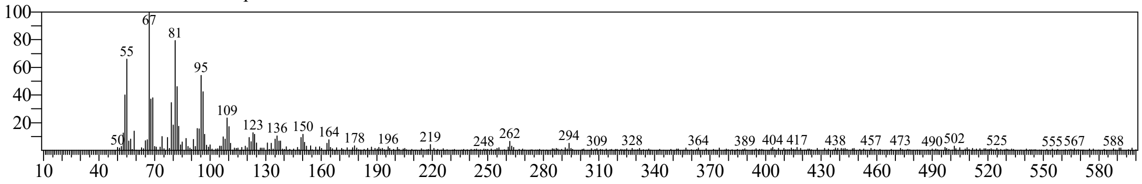


Hit#:1 Entry:125931 Library:NIST14.lib
SI:95 Formula:C19H34O2 CAS:2462-85-3 MolWeight:294 RetIndex:2093
CompName:9,12-Octadecadienoic acid, methyl ester $$ 9,12-Octadecenoic acid, methyl ester $$ Methyl octadeca-9,12-dienoate $$ Methyl 9,12-octadecadi

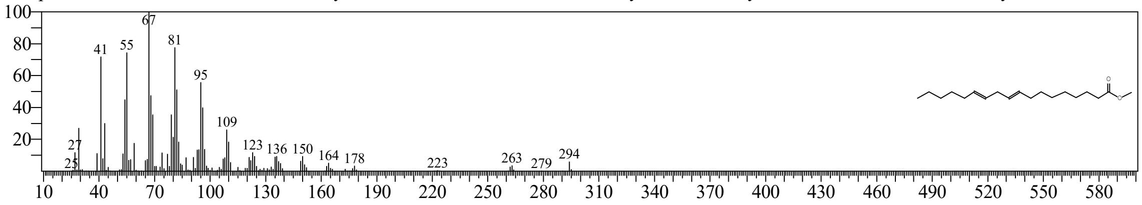


Hit#:2 Entry:137663 Library:NIST17-1.lib
SI:95 Formula:C19H34O2 CAS:2462-85-3 MolWeight:294 RetIndex:2093
CompName:9,12-Octadecadienoic acid, methyl ester $$ 9,12-Octadecenoic acid, methyl ester $$ Methyl octadeca-9,12-dienoate $$ Methyl 9,12-octadecadi

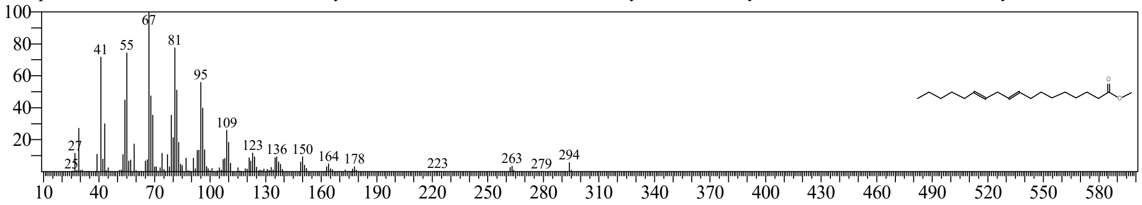


Hit#:3 Entry:32588 Library:NIST17s.lib
SI:95 Formula:C19H34O2 CAS:112-63-0 MolWeight:294 RetIndex:2093
CompName:9,12-Octadecadienoic acid (Z,Z)-, methyl ester $$ Linoleic acid, methyl ester $$ Methyl cis,cis-9,12-octadecadienoate $$ Methyl linoleate $$ M

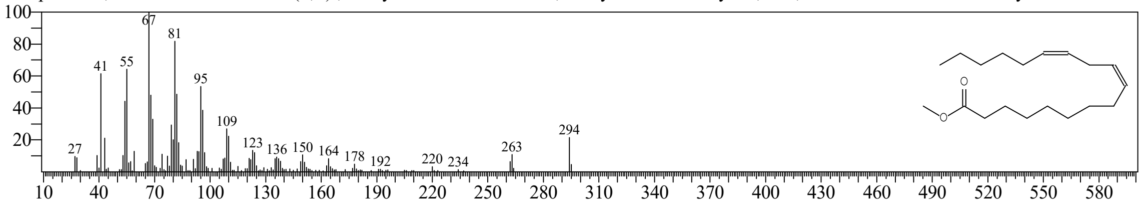


Hit#:4 Entry:27999 Library:NIST14s.lib
SI:95 Formula:C19H34O2 CAS:112-63-0 MolWeight:294 RetIndex:2093
CompName:9,12-Octadecadienoic acid (Z,Z)-, methyl ester $$ Linoleic acid, methyl ester $$ Methyl cis,cis-9,12-octadecadienoate $$ Methyl linoleate $$ M

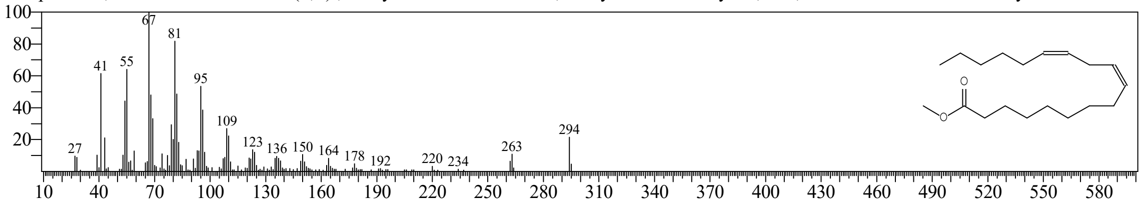


Hit#:5 Entry:32589 Library:NIST17s.lib
SI:95 Formula:C19H34O2 CAS:112-63-0 MolWeight:294 RetIndex:2093
CompName:9,12-Octadecadienoic acid (Z,Z)-, methyl ester $$ Linoleic acid, methyl ester $$ Methyl cis,cis-9,12-octadecadienoate $$ Methyl linoleate $$ M

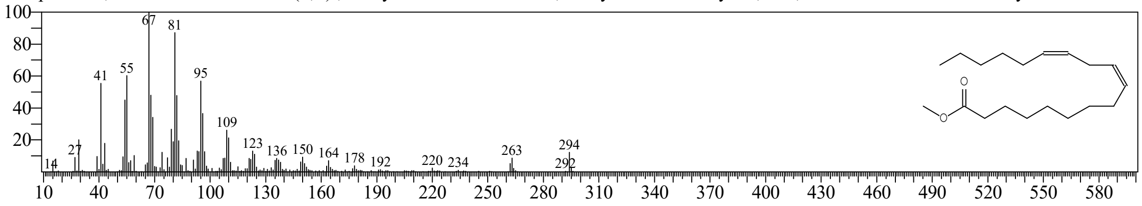

<< Target >>
Line#:11   R.Time:27.925(Scan#:2152)  MassPeaks:358
RawMode:Averaged 27.917-27.933(2151-2153) BasePeak:67.00(1590)
BG Mode:Calc. from Peak  Group 1 - Event 1

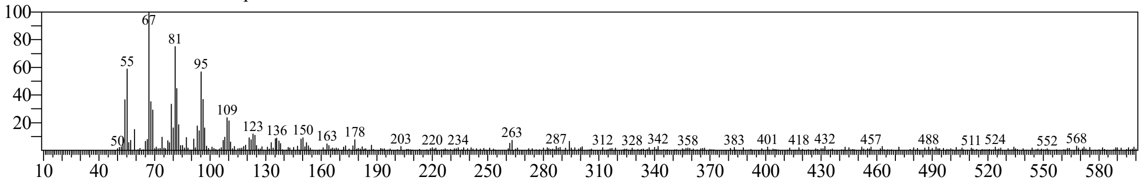


Hit#:1  Entry:32589  Library:NIST17s.lib
SI:94  Formula:C19H34O2  CAS:112-63-0  MolWeight:294  RetIndex:2093
CompName:9,12-Octadecadienoic acid (Z,Z)-, methyl ester $$ Linoleic acid, methyl ester $$ Methyl cis,cis-9,12-octadecadienoate $$ Methyl linoleate $$ M

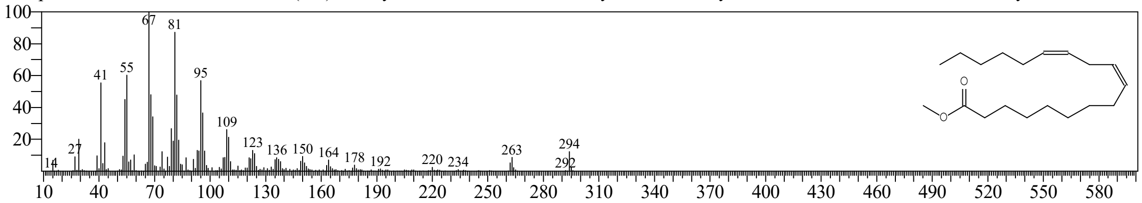


Hit#:2  Entry:28000  Library:NIST14s.lib
SI:94  Formula:C19H34O2  CAS:112-63-0  MolWeight:294  RetIndex:2093
CompName:9,12-Octadecadienoic acid (Z,Z)-, methyl ester $$ Linoleic acid, methyl ester $$ Methyl cis,cis-9,12-octadecadienoate $$ Methyl linoleate $$ M

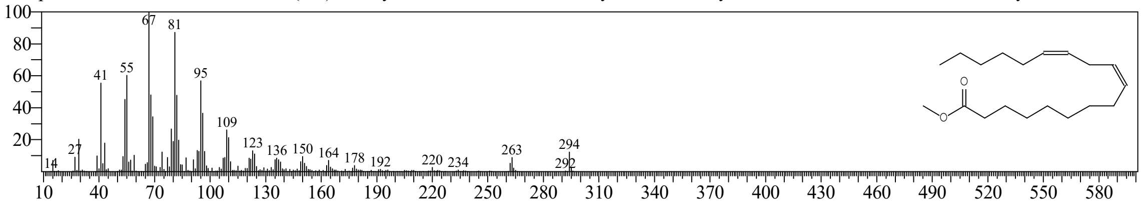


Hit#:3  Entry:27999  Library:NIST14s.lib
SI:94  Formula:C19H34O2  CAS:112-63-0  MolWeight:294  RetIndex:2093
CompName:9,12-Octadecadienoic acid (Z,Z)-, methyl ester $$ Linoleic acid, methyl ester $$ Methyl cis,cis-9,12-octadecadienoate $$ Methyl linoleate $$ M

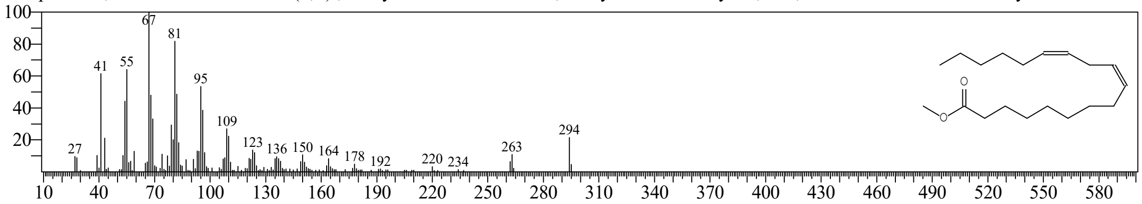


Hit#:4  Entry:32588  Library:NIST17s.lib
SI:94  Formula:C19H34O2  CAS:112-63-0  MolWeight:294  RetIndex:2093
CompName:9,12-Octadecadienoic acid (Z,Z)-, methyl ester $$ Linoleic acid, methyl ester $$ Methyl cis,cis-9,12-octadecadienoate $$ Methyl linoleate $$ M

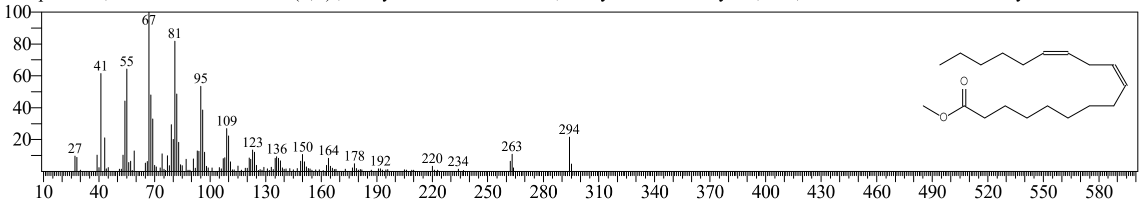


Hit#:5  Entry:125931  Library:NIST14.lib
SI:94  Formula:C19H34O2  CAS:2462-85-3  MolWeight:294  RetIndex:2093
CompName:9,12-Octadecadienoic acid, methyl ester $$ 9,12-Octadecenoic acid, methyl ester $$ Methyl octadeca-9,12-dienoate $$ Methyl 9,12-octadecadi

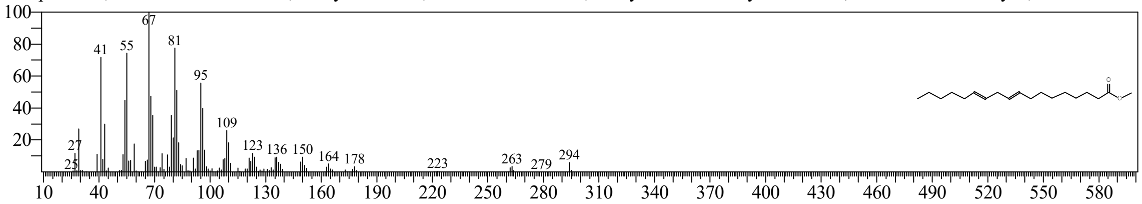

<< Target >>
Line#:12 R.Time:28.358(Scan#:2204) MassPeaks:357
RawMode:Averaged 28.350-28.367(2203-2205) BasePeak:67.05(438831)
BG Mode:Calc. from Peak Group 1 - Event 1

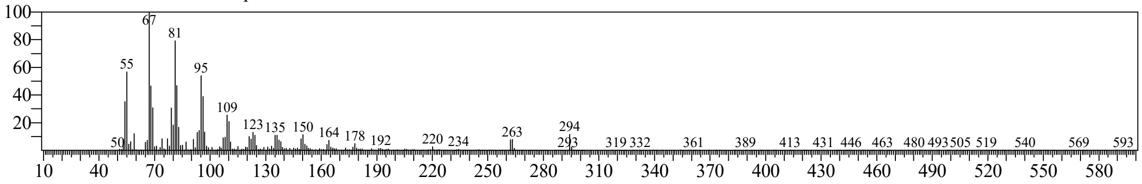


Hit#:1 Entry:32588 Library:NIST17s.lib
SI:97 Formula:C19H34O2 CAS:112-63-0 MolWeight:294 RetIndex:2093
CompName:9,12-Octadecadienoic acid (Z,Z)-, methyl ester $$ Linoleic acid, methyl ester $$ Methyl cis,cis-9,12-octadecadienoate $$ Methyl linoleate $$ M

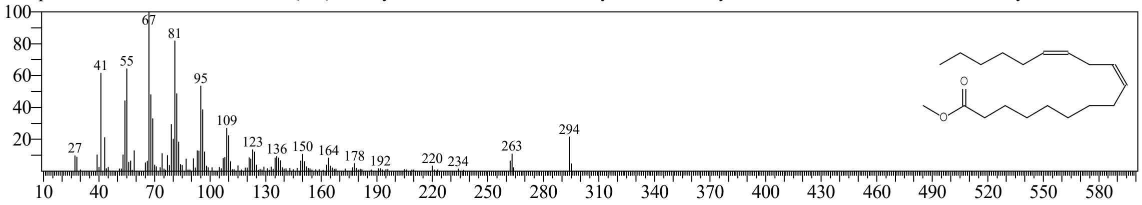


Hit#:2 Entry:27999 Library:NIST14s.lib
SI:97 Formula:C19H34O2 CAS:112-63-0 MolWeight:294 RetIndex:2093
CompName:9,12-Octadecadienoic acid (Z,Z)-, methyl ester $$ Linoleic acid, methyl ester $$ Methyl cis,cis-9,12-octadecadienoate $$ Methyl linoleate $$ M

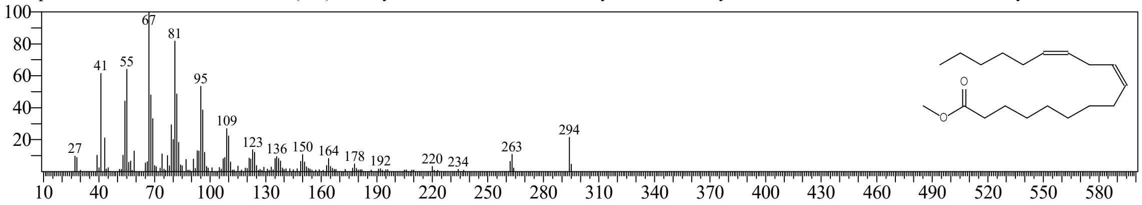


Hit#:3 Entry:32589 Library:NIST17s.lib
SI:97 Formula:C19H34O2 CAS:112-63-0 MolWeight:294 RetIndex:2093
CompName:9,12-Octadecadienoic acid (Z,Z)-, methyl ester $$ Linoleic acid, methyl ester $$ Methyl cis,cis-9,12-octadecadienoate $$ Methyl linoleate $$ M

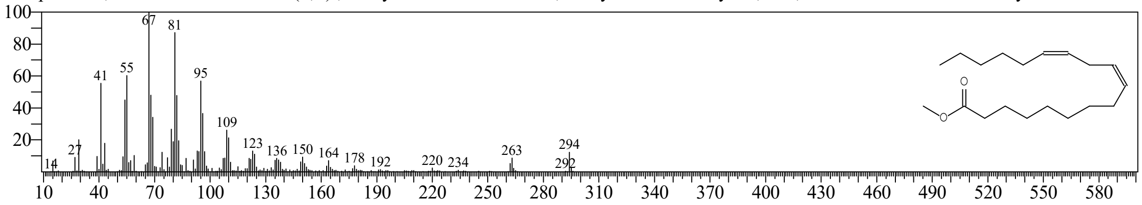


Hit#:4 Entry:28000 Library:NIST14s.lib
SI:97 Formula:C19H34O2 CAS:112-63-0 MolWeight:294 RetIndex:2093
CompName:9,12-Octadecadienoic acid (Z,Z)-, methyl ester $$ Linoleic acid, methyl ester $$ Methyl cis,cis-9,12-octadecadienoate $$ Methyl linoleate $$ M

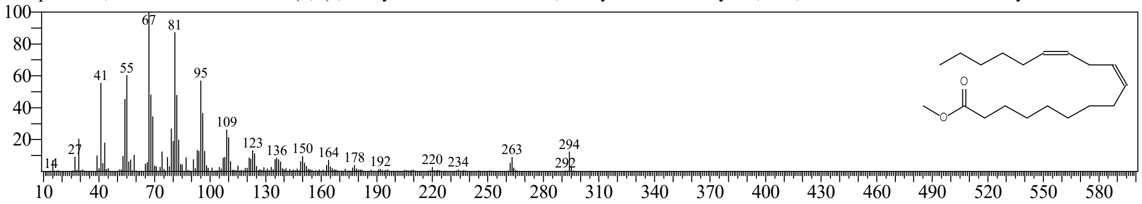


Hit#:5 Entry:137664 Library:NIST17-1.lib
SI:95 Formula:C19H34O2 CAS:0-00-0 MolWeight:294 RetIndex:2093
CompName:Methyl 10-trans,12-cis-octadecadienoate

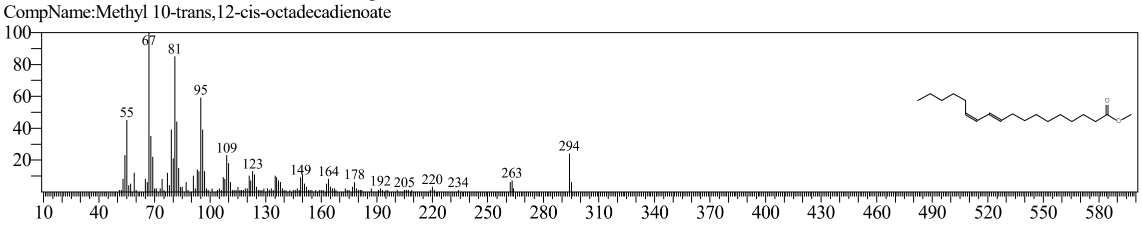

<< Target >>
Line#:13 R.Time:29.258(Scan#:2312) MassPeaks:307
RawMode:Averaged 29.250-29.267(2311-2313) BasePeak:74.00(10086)
BG Mode:Calc. from Peak Group 1 - Event 1

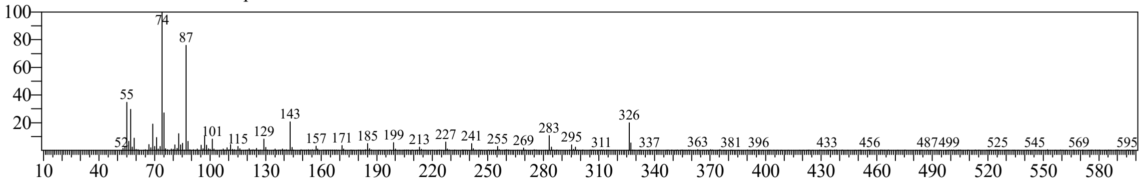


Hit#:1 Entry:154704 Library:NIST14.lib
SI:97 Formula:C21H42O2 CAS:0-00-0 MolWeight:326 RetIndex:2212
CompName:Methyl 18-methylnonadecanoate

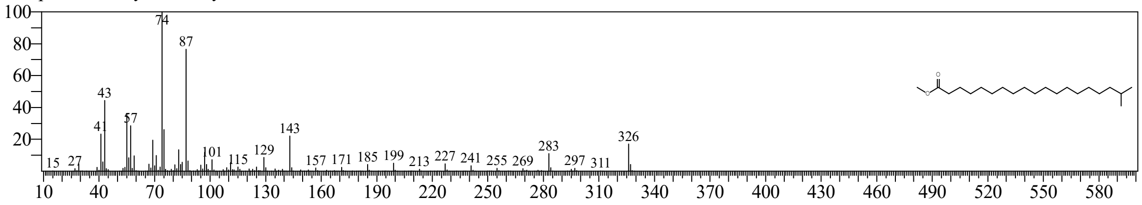


Hit#:2 Entry:170238 Library:NIST17-1.lib
SI:97 Formula:C21H42O2 CAS:0-00-0 MolWeight:326 RetIndex:2212
CompName:Methyl 18-methylnonadecanoate

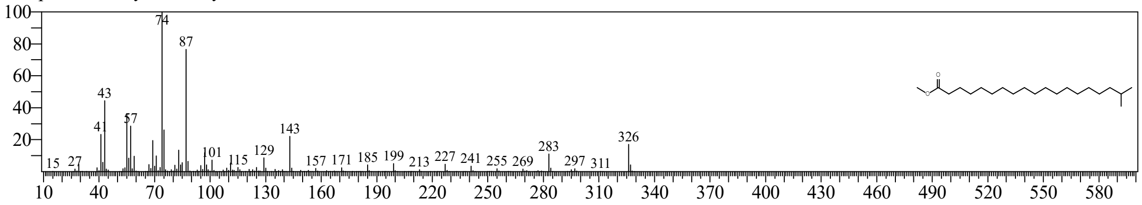


Hit#:3 Entry:34726 Library:NIST17s.lib
SI:96 Formula:C21H42O2 CAS:1120-28-1 MolWeight:326 RetIndex:2276
CompName:Eicosanoic acid, methyl ester $$ Methyl arachisate $$ Methyl eicosanoate $$ Arachidic acid methyl ester $$ Kemester 2050 $$ Methyl aracidat

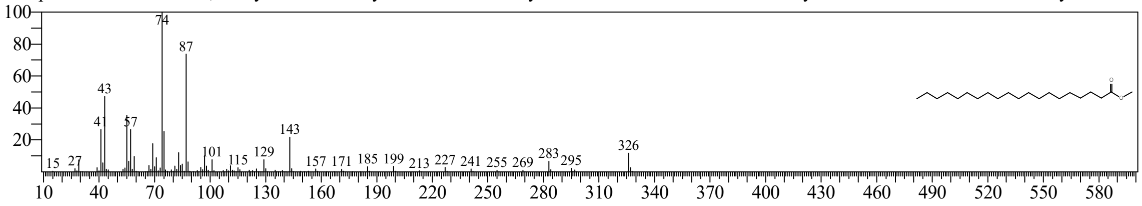


Hit#:4 Entry:34724 Library:NIST17s.lib
SI:93 Formula:C21H42O2 CAS:1120-28-1 MolWeight:326 RetIndex:2276
CompName:Eicosanoic acid, methyl ester $$ Methyl arachisate $$ Methyl eicosanoate $$ Arachidic acid methyl ester $$ Kemester 2050 $$ Methyl aracidat

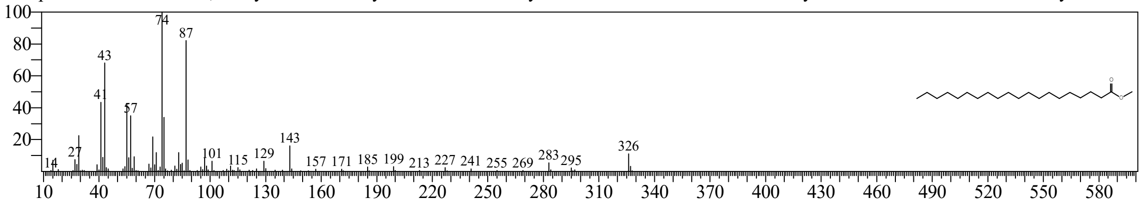


Hit#:5 Entry:29931 Library:NIST14s.lib
SI:93 Formula:C21H42O2 CAS:1120-28-1 MolWeight:326 RetIndex:2276
CompName:Eicosanoic acid, methyl ester $$ Methyl arachisate $$ Methyl eicosanoate $$ Arachidic acid methyl ester $$ Kemester 2050 $$ Methyl aracidat

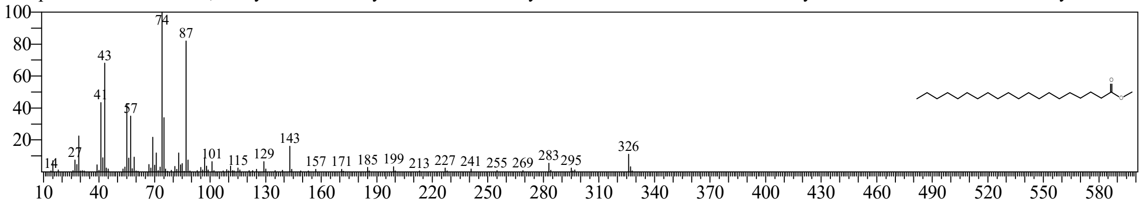

<< Target >>
Line#:14  R.Time:29.808(Scan#:2378)  MassPeaks:300
RawMode:Averaged 29.800-29.817(2377-2379) BasePeak:79.05(6273)
BG Mode:Calc. from Peak  Group 1 - Event 1

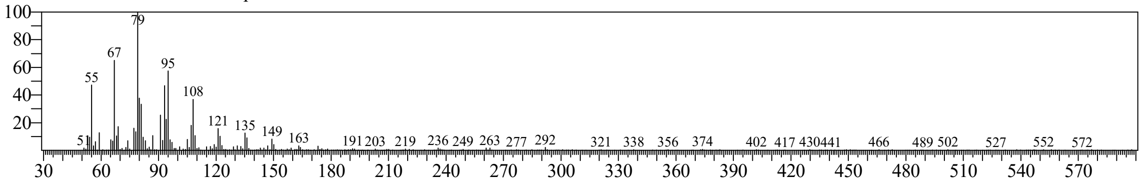


Hit#:1  Entry:29609  Library:NIST14s.lib
SI:94  Formula:C21H36O2  CAS:55682-88-7  MolWeight:320  RetIndex:2300
CompName:11,14,17-Eicosatrienoic acid, methyl ester $$ Methyl 11,14,17-icosatrienoate $$ Methyl 11,14,17-eicosatrienoate $$

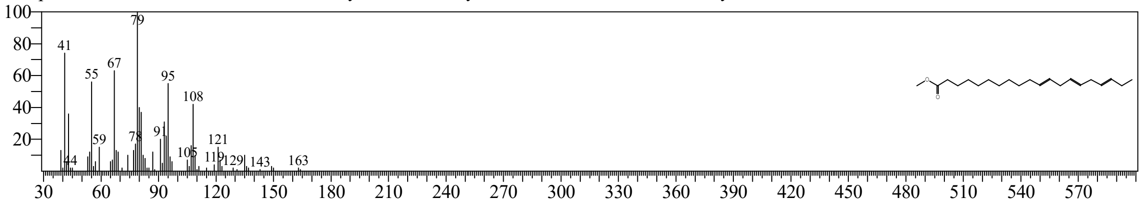


Hit#:2  Entry:34362  Library:NIST17s.lib
SI:94  Formula:C21H36O2  CAS:55682-88-7  MolWeight:320  RetIndex:2300
CompName:11,14,17-Eicosatrienoic acid, methyl ester $$ Methyl 11,14,17-icosatrienoate $$ Methyl 11,14,17-eicosatrienoate $$

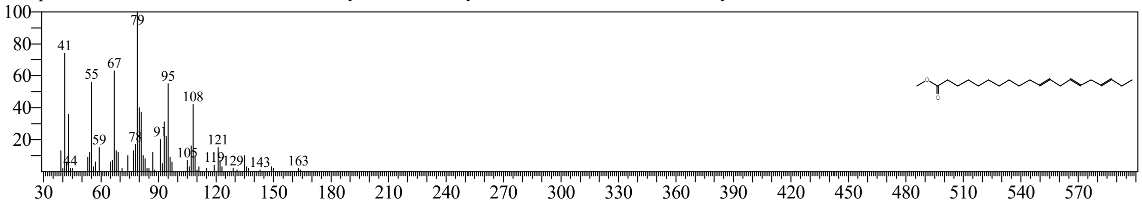


Hit#:3  Entry:27872  Library:NIST14s.lib
SI:94  Formula:C19H32O2  CAS:301-00-8  MolWeight:292  RetIndex:2101
CompName:9,12,15-Octadecatrienoic acid, methyl ester, (Z,Z,Z)- $$ Linolenic acid, methyl ester $$ Methyl all-cis-9,12,15-octadecatrienoate $$ Methyl lino

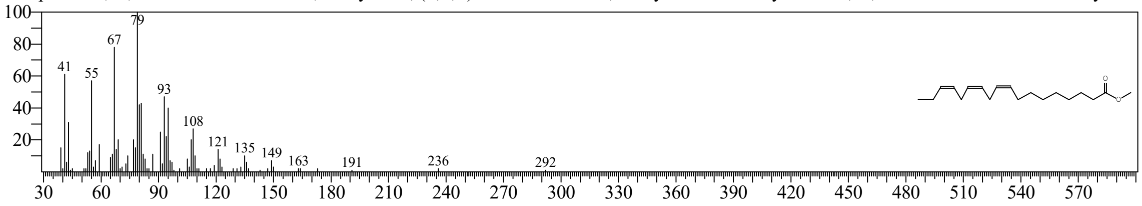


Hit#:4  Entry:32437  Library:NIST17s.lib
SI:94  Formula:C19H32O2  CAS:301-00-8  MolWeight:292  RetIndex:2101
CompName:9,12,15-Octadecatrienoic acid, methyl ester, (Z,Z,Z)- $$ Linolenic acid, methyl ester $$ Methyl all-cis-9,12,15-octadecatrienoate $$ Methyl lino

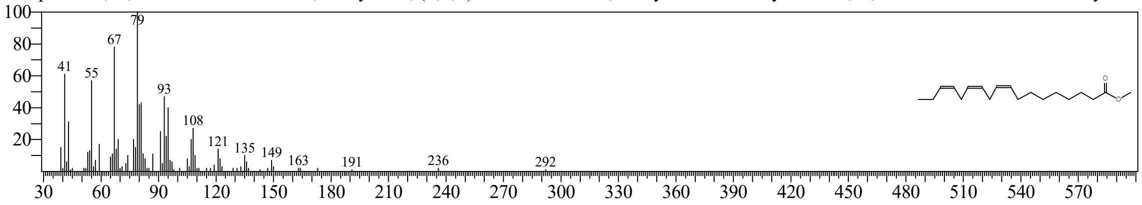


Hit#:5  Entry:32438  Library:NIST17s.lib
SI:94  Formula:C19H32O2  CAS:301-00-8  MolWeight:292  RetIndex:2101
CompName:9,12,15-Octadecatrienoic acid, methyl ester, (Z,Z,Z)- $$ Linolenic acid, methyl ester $$ Methyl all-cis-9,12,15-octadecatrienoate $$ Methyl lino

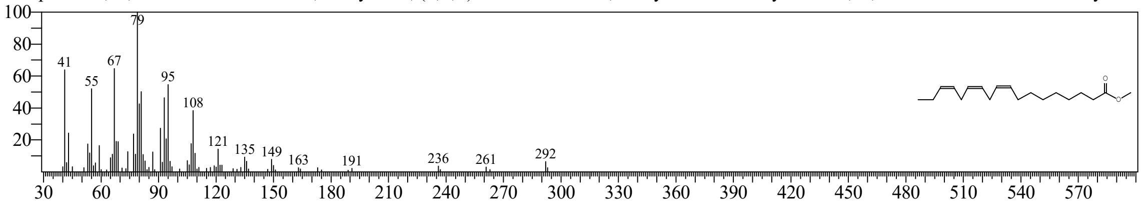

<< Target >>
Line#:15 R.Time:30.292(Scan#:2436) MassPeaks:378
RawMode:Averaged 30.283-30.300(2435-2437) BasePeak:79.00(4592)
BG Mode:Calc. from Peak Group 1 - Event 1

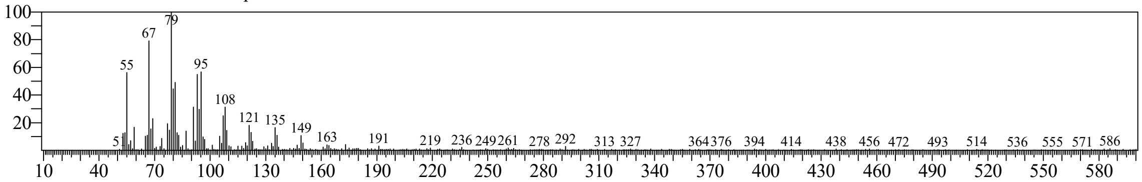


Hit#:1 Entry:124228 Library:NIST14.lib
SI:95 Formula:C19H32O2 CAS:301-00-8 MolWeight:292 RetIndex:2101
CompName:9,12,15-Octadecatrienoic acid, methyl ester, (Z,Z,Z)- $$ Linolenic acid, methyl ester $$ Methyl all-cis-9,12,15-octadecatrienoate $$ Methyl lino

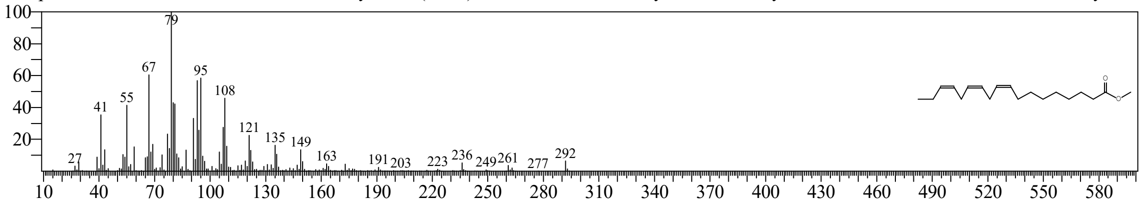


Hit#:2 Entry:135724 Library:NIST17-1.lib
SI:95 Formula:C19H32O2 CAS:301-00-8 MolWeight:292 RetIndex:2101
CompName:9,12,15-Octadecatrienoic acid, methyl ester, (Z,Z,Z)- $$ Linolenic acid, methyl ester $$ Methyl all-cis-9,12,15-octadecatrienoate $$ Methyl lino

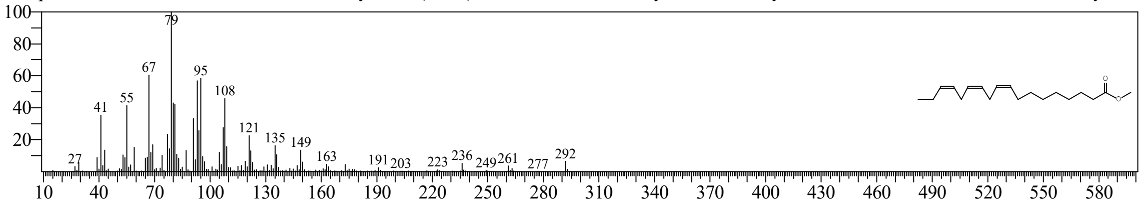


Hit#:3 Entry:121536 Library:NIST17-1.lib
SI:94 Formula:C18H30O2 CAS:0-00-0 MolWeight:278 RetIndex:2002
CompName:Methyl 8,11,14-heptadecatrienoate

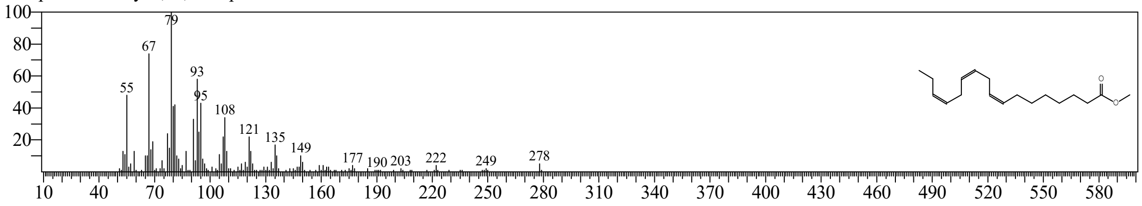


Hit#:4 Entry:111654 Library:NIST14.lib
SI:94 Formula:C18H30O2 CAS:0-00-0 MolWeight:278 RetIndex:2002
CompName:Methyl 8,11,14-heptadecatrienoate

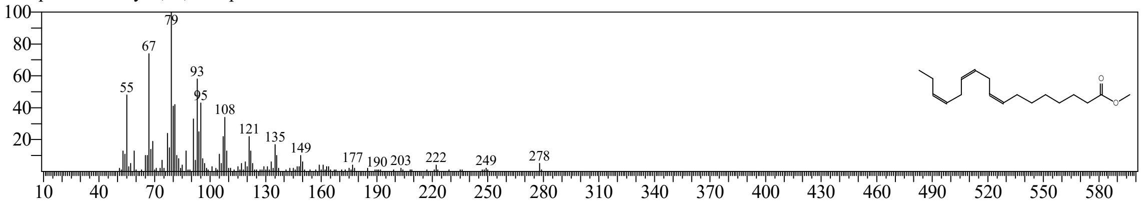


Hit#:5 Entry:32437 Library:NIST17s.lib
SI:94 Formula:C19H32O2 CAS:301-00-8 MolWeight:292 RetIndex:2101
CompName:9,12,15-Octadecatrienoic acid, methyl ester, (Z,Z,Z)- $$ Linolenic acid, methyl ester $$ Methyl all-cis-9,12,15-octadecatrienoate $$ Methyl lino

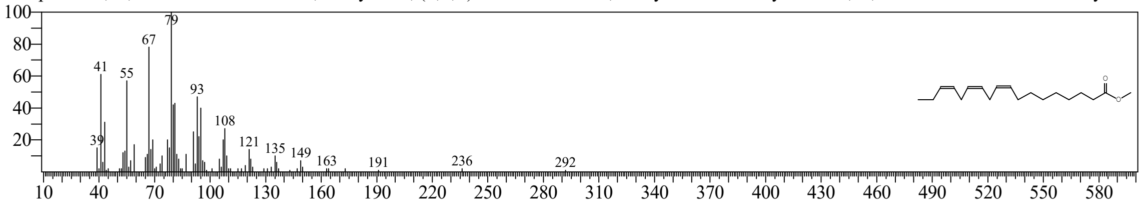

<< Target >>
Line#:16 R.Time:30.600(Scan#:2473) MassPeaks:421
RawMode:Averaged 30.592-30.608(2472-2474) BasePeak:79.05(74721)
BG Mode:Calc. from Peak Group 1 - Event 1

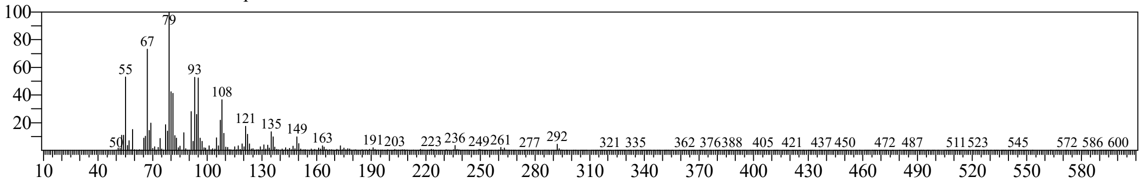


Hit#:1 Entry:135724 Library:NIST17-1.lib
SI:96 Formula:C19H32O2 CAS:301-00-8 MolWeight:292 RetIndex:2101
CompName:9,12,15-Octadecatrienoic acid, methyl ester, (Z,Z,Z)- $$ Linolenic acid, methyl ester $$ Methyl all-cis-9,12,15-octadecatrienoate $$ Methyl lino

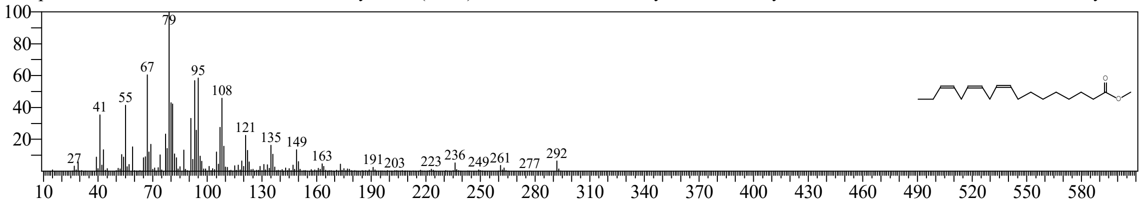


Hit#:2 Entry:124228 Library:NIST14.lib
SI:96 Formula:C19H32O2 CAS:301-00-8 MolWeight:292 RetIndex:2101
CompName:9,12,15-Octadecatrienoic acid, methyl ester, (Z,Z,Z)- $$ Linolenic acid, methyl ester $$ Methyl all-cis-9,12,15-octadecatrienoate $$ Methyl lino

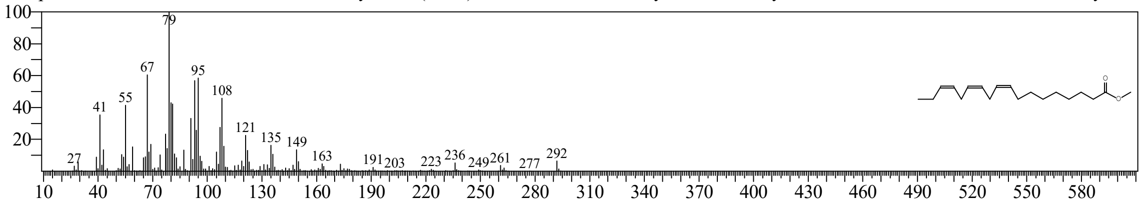


Hit#:3 Entry:32438 Library:NIST17s.lib
SI:95 Formula:C19H32O2 CAS:301-00-8 MolWeight:292 RetIndex:2101
CompName:9,12,15-Octadecatrienoic acid, methyl ester, (Z,Z,Z)- $$ Linolenic acid, methyl ester $$ Methyl all-cis-9,12,15-octadecatrienoate $$ Methyl lino

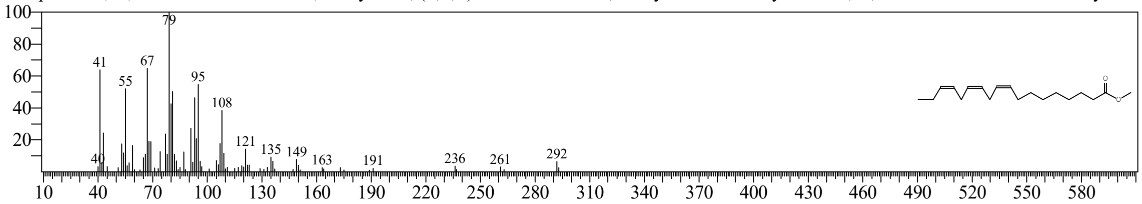


Hit#:4 Entry:27872 Library:NIST14s.lib
SI:95 Formula:C19H32O2 CAS:301-00-8 MolWeight:292 RetIndex:2101
CompName:9,12,15-Octadecatrienoic acid, methyl ester, (Z,Z,Z)- $$ Linolenic acid, methyl ester $$ Methyl all-cis-9,12,15-octadecatrienoate $$ Methyl lino

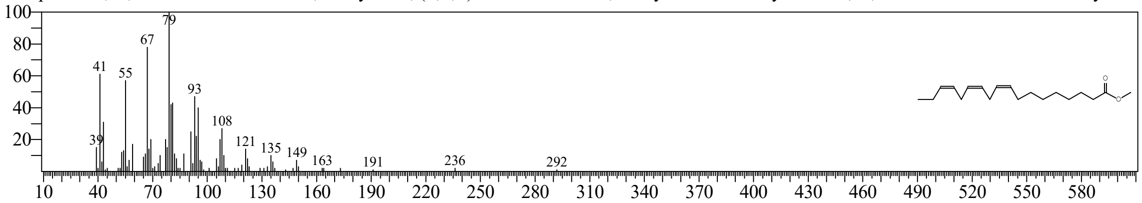


Hit#:5 Entry:121536 Library:NIST17-1.lib
SI:95 Formula:C18H30O2 CAS:0-00-0 MolWeight:278 RetIndex:2002
CompName:Methyl 8,11,14-heptadecatrienoate

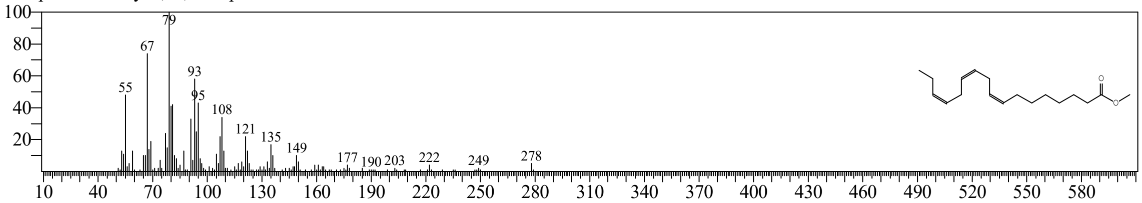

<< Target >>
Line#:17  R.Time:30.933(Scan#:2513)  MassPeaks:401
RawMode:Averaged 30.925-30.942(2512-2514) BasePeak:67.00(19766)
BG Mode:Calc. from Peak  Group 1 - Event 1

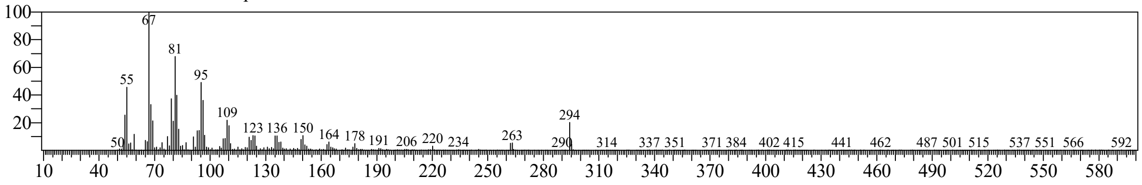


Hit#:1  Entry:125932  Library:NIST14.lib
SI:98  Formula:C19H34O2  CAS:0-00-0  MolWeight:294  RetIndex:2093
CompName:Methyl 9-cis,11-trans-octadecadienoate

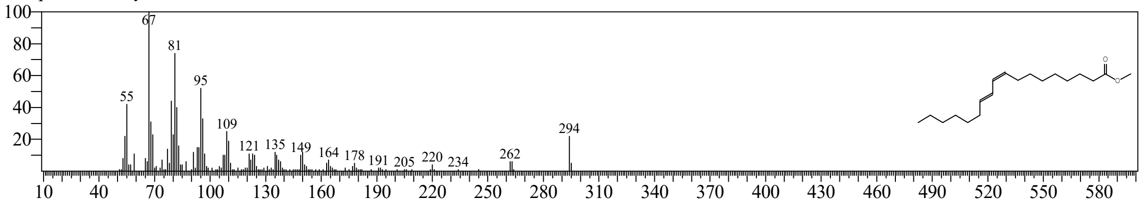


Hit#:2  Entry:137666  Library:NIST17-1.lib
SI:98  Formula:C19H34O2  CAS:13058-52-1  MolWeight:294  RetIndex:2093
CompName:Methyl 9-cis,11-trans-octadecadienoate

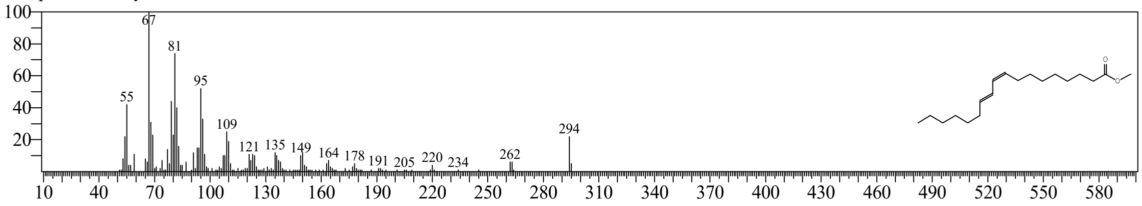


Hit#:3  Entry:137664  Library:NIST17-1.lib
SI:97  Formula:C19H34O2  CAS:0-00-0  MolWeight:294  RetIndex:2093
CompName:Methyl 10-trans,12-cis-octadecadienoate

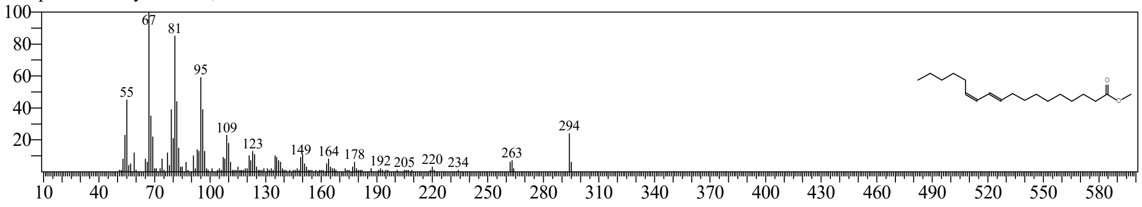


Hit#:4  Entry:125933  Library:NIST14.lib
SI:97  Formula:C19H34O2  CAS:0-00-0  MolWeight:294  RetIndex:2093
CompName:Methyl 10-trans,12-cis-octadecadienoate

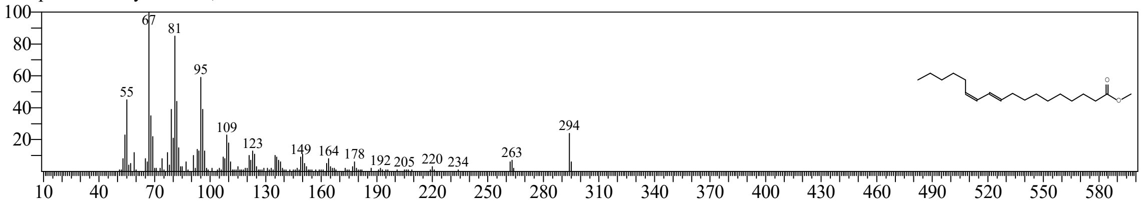


Hit#:5  Entry:32587  Library:NIST17s.lib
SI:96  Formula:C19H34O2  CAS:13038-47-6  MolWeight:294  RetIndex:2093
CompName:9,11-Octadecadienoic acid, methyl ester, (E,E)- $$ Methyl trans-9,trans-11-octadecadienoate $$ Methyl trans,trans-9,11-octadecadienoate $$ M

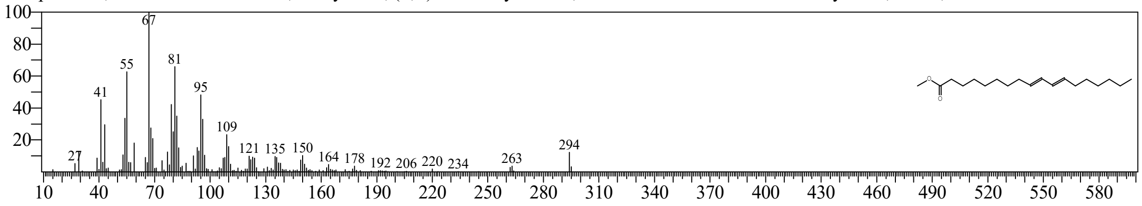

<< Target >>
Line#:18   R.Time:31.258(Scan#:2552)  MassPeaks:350
RawMode:Averaged 31.250-31.267(2551-2553) BasePeak:67.05(21155)
BG Mode:Calc. from Peak  Group 1 - Event 1

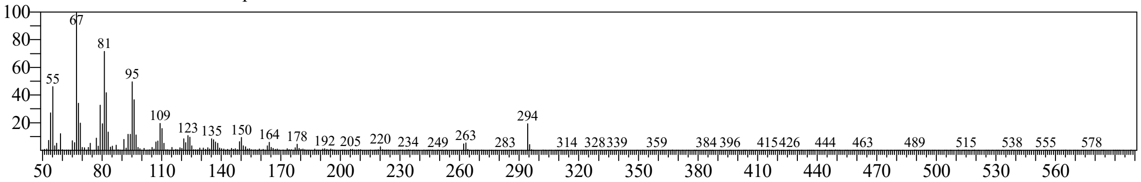


Hit#:1  Entry:125933  Library:NIST14.lib
SI:96  Formula:C19H34O2  CAS:0-00-0  MolWeight:294  RetIndex:2093
CompName:Methyl 10-trans,12-cis-octadecadienoate

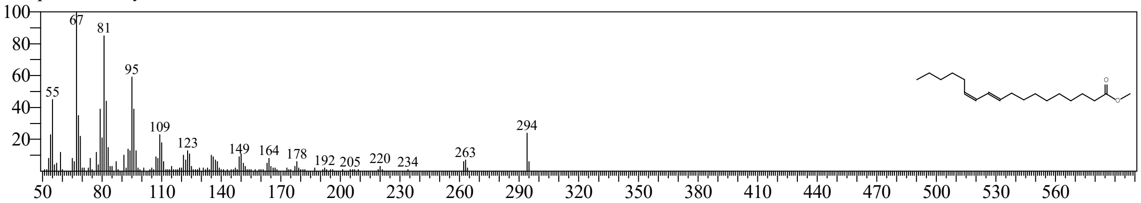


Hit#:2  Entry:137664  Library:NIST17-1.lib
SI:96  Formula:C19H34O2  CAS:0-00-0  MolWeight:294  RetIndex:2093
CompName:Methyl 10-trans,12-cis-octadecadienoate

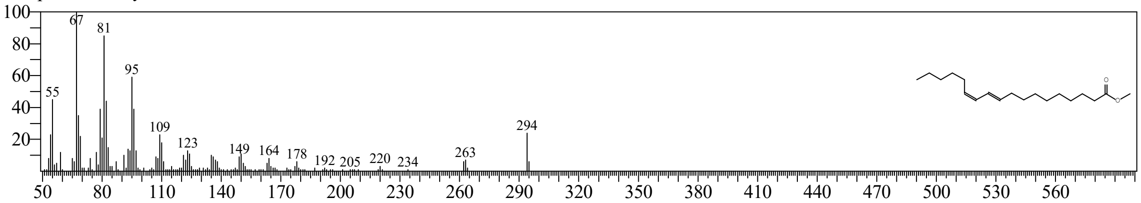


Hit#:3  Entry:125932  Library:NIST14.lib
SI:96  Formula:C19H34O2  CAS:0-00-0  MolWeight:294  RetIndex:2093
CompName:Methyl 9-cis,11-trans-octadecadienoate

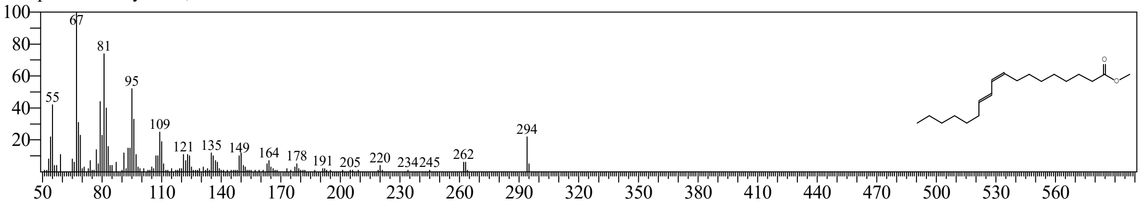


Hit#:4  Entry:137666  Library:NIST17-1.lib
SI:96  Formula:C19H34O2  CAS:13058-52-1  MolWeight:294  RetIndex:2093
CompName:Methyl 9-cis,11-trans-octadecadienoate

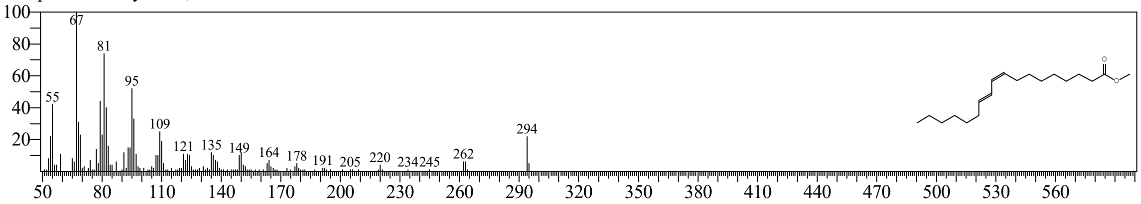


Hit#:5  Entry:28003  Library:NIST14s.lib
SI:95  Formula:C19H34O2  CAS:57156-97-5  MolWeight:294  RetIndex:2093
CompName:12,15-Octadecadienoic acid, methyl ester $$ Methyl (12E,15E)-12,15-octadecadienoate # $$

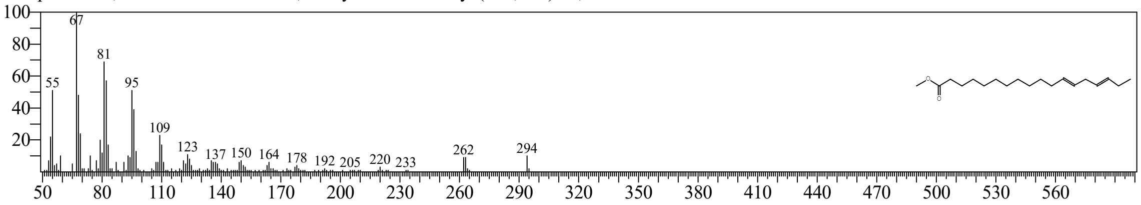

<< Target >>
Line#:19  R.Time:31.942(Scan#:2634)  MassPeaks:283
RawMode:Averaged 31.933-31.950(2633-2635) BasePeak:67.00(659)
BG Mode:Calc. from Peak  Group 1 - Event 1

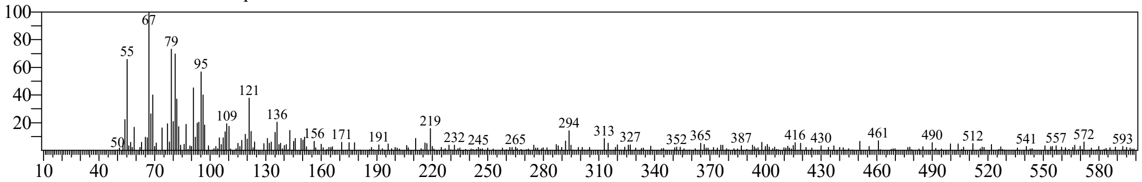


Hit#:1  Entry:32587  Library:NIST17s.lib
SI:83  Formula:C19H34O2  CAS:13038-47-6  MolWeight:294  RetIndex:2093
CompName:9,11-Octadecadienoic acid, methyl ester, (E,E)- $$ Methyl trans-9,trans-11-octadecadienoate $$ Methyl trans,trans-9,11-octadecadienoate $$ M

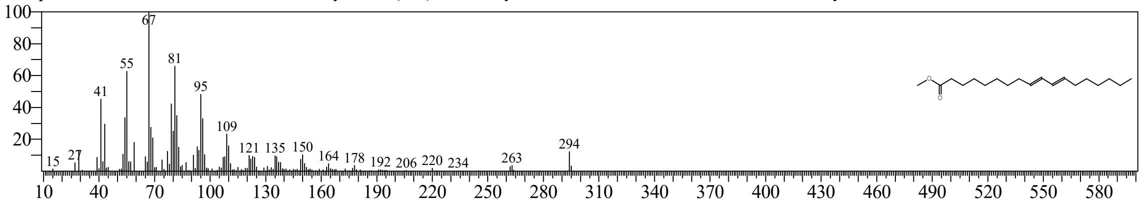


Hit#:2  Entry:32440  Library:NIST17s.lib
SI:83  Formula:C19H32O2  CAS:301-00-8  MolWeight:292  RetIndex:2101
CompName:9,12,15-Octadecatrienoic acid, methyl ester, (Z,Z,Z)- $$ Linolenic acid, methyl ester $$ Methyl all-cis-9,12,15-octadecatrienoate $$ Methyl lino

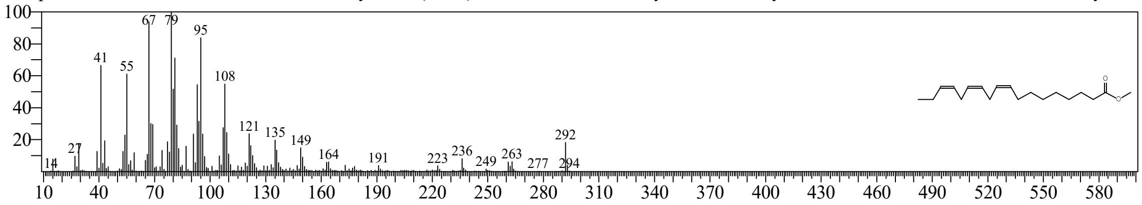


Hit#:3  Entry:27874  Library:NIST14s.lib
SI:83  Formula:C19H32O2  CAS:301-00-8  MolWeight:292  RetIndex:2101
CompName:9,12,15-Octadecatrienoic acid, methyl ester, (Z,Z,Z)- $$ Linolenic acid, methyl ester $$ Methyl all-cis-9,12,15-octadecatrienoate $$ Methyl lino

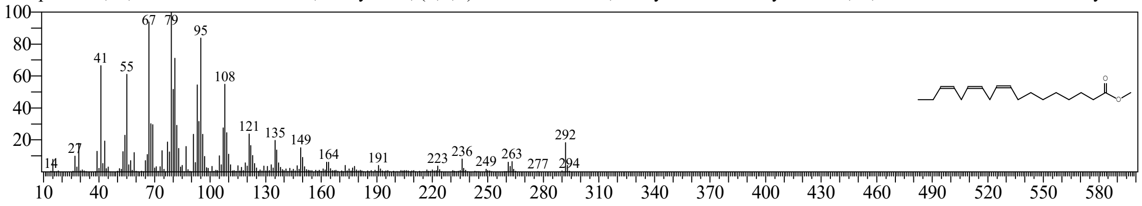


Hit#:4  Entry:137663  Library:NIST17-1.lib
SI:83  Formula:C19H34O2  CAS:2462-85-3  MolWeight:294  RetIndex:2093
CompName:9,12-Octadecadienoic acid, methyl ester $$ 9,12-Octadecenoic acid, methyl ester $$ Methyl octadeca-9,12-dienoate $$ Methyl 9,12-octadecadi

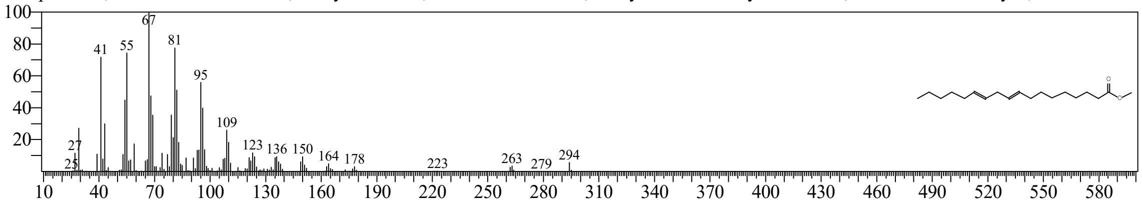


Hit#:5  Entry:125931  Library:NIST14.lib
SI:83  Formula:C19H34O2  CAS:2462-85-3  MolWeight:294  RetIndex:2093
CompName:9,12-Octadecadienoic acid, methyl ester $$ 9,12-Octadecenoic acid, methyl ester $$ Methyl octadeca-9,12-dienoate $$ Methyl 9,12-octadecadi

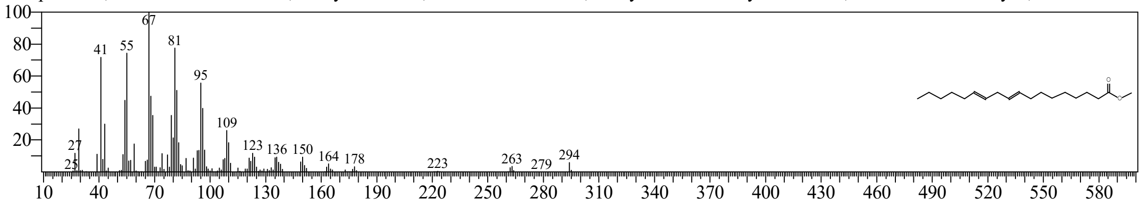

<< Target >>
Line#:20 R.Time:32.208(Scan#:2666) MassPeaks:328
RawMode:Averaged 32.200-32.217(2665-2667) BasePeak:79.05(1015)
BG Mode:Calc. from Peak Group 1 - Event 1

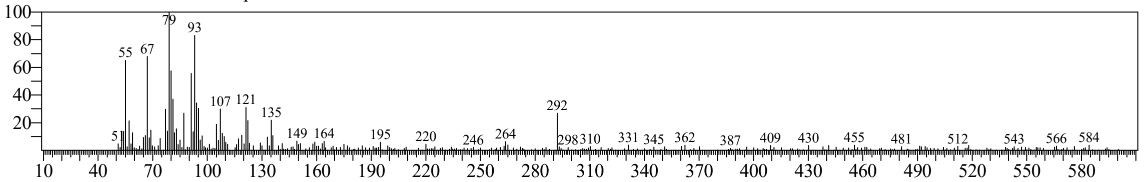


Hit#:1 Entry:135722 Library:NIST17-1.lib
SI:90 Formula:C19H32O2 CAS:0-00-0 MolWeight:292 RetIndex:2101
CompName:Methyl 6-cis,9-cis,11-trans-octadecatrienoate

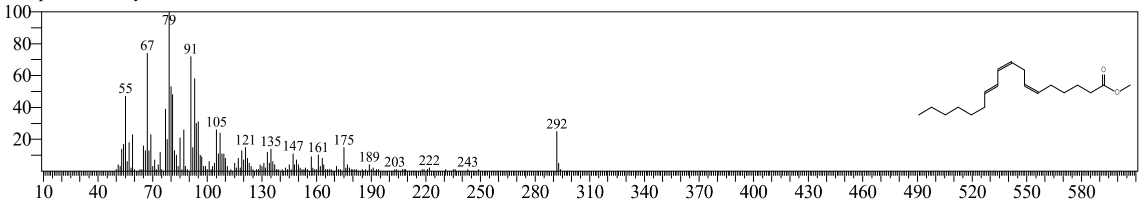


Hit#:2 Entry:124226 Library:NIST14.lib
SI:90 Formula:C19H32O2 CAS:0-00-0 MolWeight:292 RetIndex:2101
CompName:Methyl 6-cis,9-cis,11-trans-octadecatrienoate

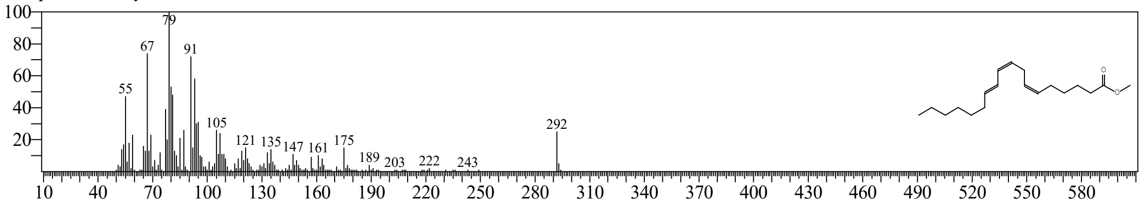


Hit#:3 Entry:124225 Library:NIST14.lib
SI:89 Formula:C19H32O2 CAS:0-00-0 MolWeight:292 RetIndex:2101
CompName:.gamma.-Linolenic acid, methyl ester

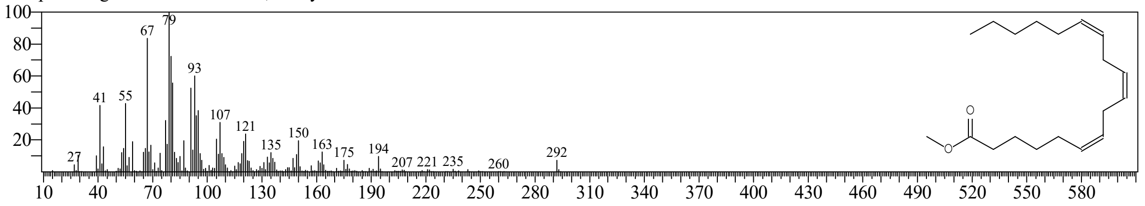


Hit#:4 Entry:135721 Library:NIST17-1.lib
SI:89 Formula:C19H32O2 CAS:16326-32-2 MolWeight:292 RetIndex:2101
CompName:Methyl .gamma.-linolenate

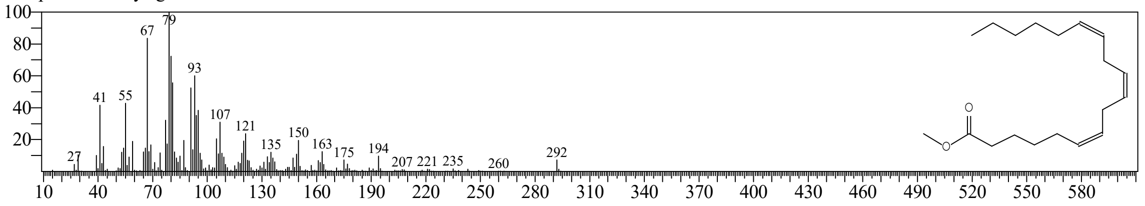


Hit#:5 Entry:111654 Library:NIST14.lib
SI:88 Formula:C18H30O2 CAS:0-00-0 MolWeight:278 RetIndex:2002
CompName:Methyl 8,11,14-heptadecatrienoate

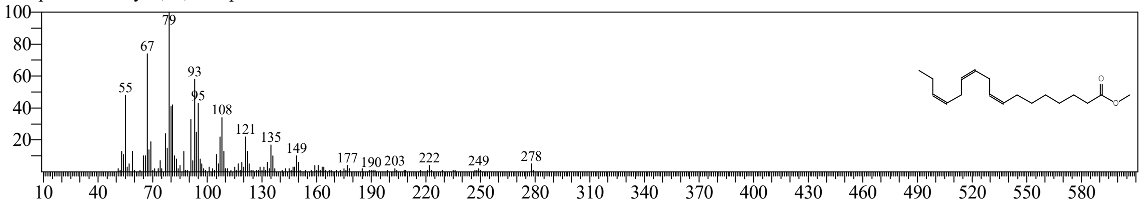

<< Target >>
Line#:21 R.Time:32.958(Scan#:2756) MassPeaks:372
RawMode:Averaged 32.950-32.967(2755-2757) BasePeak:95.10(30331)
BG Mode:Calc. from Peak Group 1 - Event 1

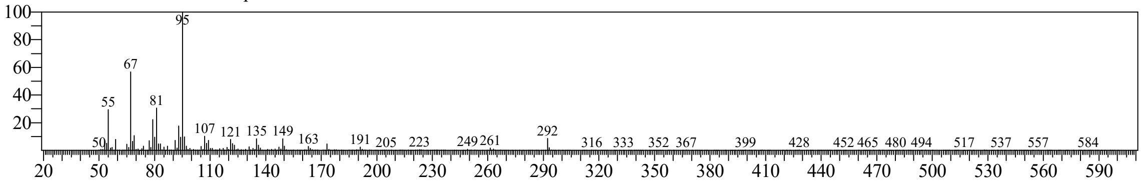


Hit#:1 Entry:24424 Library:NIST17-1.lib
SI:85 Formula:C12H20 CAS:0-00-0 MolWeight:164 RetIndex:1041
CompName:Cis-3-ethyl-endo-tricyclo[5.2.1.0(2.6)]decane

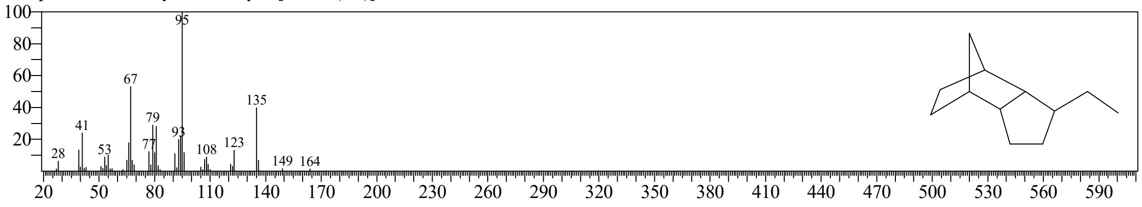


Hit#:2 Entry:23403 Library:NIST14.lib
SI:85 Formula:C12H20 CAS:0-00-0 MolWeight:164 RetIndex:1041
CompName:Cis-3-ethyl-endo-tricyclo[5.2.1.0(2.6)]decane

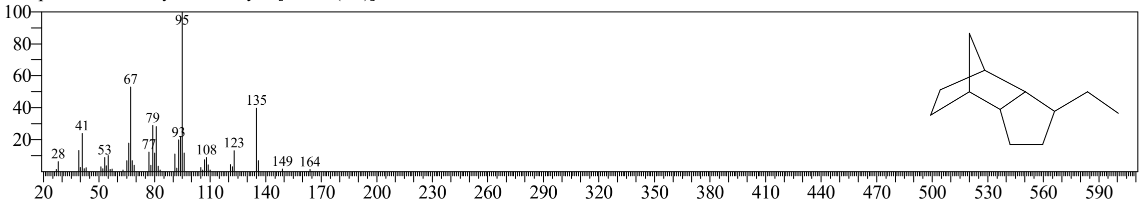


Hit#:3 Entry:16897 Library:NIST14.lib
SI:84 Formula:C10H16O CAS:60485-45-2 MolWeight:152 RetIndex:991
CompName:Santolina epoxide $$ 2-Methyl-2-(3-methyl-1-vinyl-2-butenyl)oxirane # $$

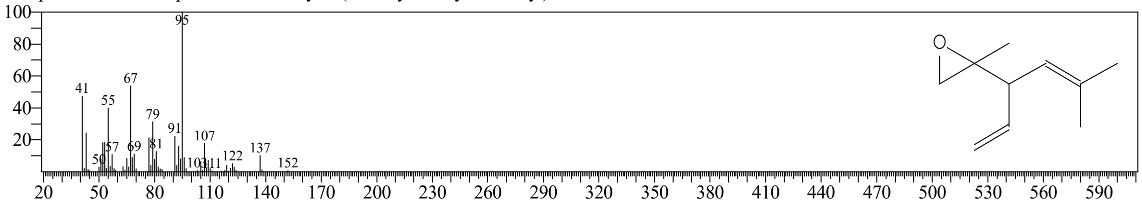


Hit#:4 Entry:17571 Library:NIST17-1.lib
SI:84 Formula:C10H16O CAS:60485-45-2 MolWeight:152 RetIndex:991
CompName:Santolina epoxide $$ 2-Methyl-2-(3-methyl-1-vinyl-2-butenyl)oxirane # $$

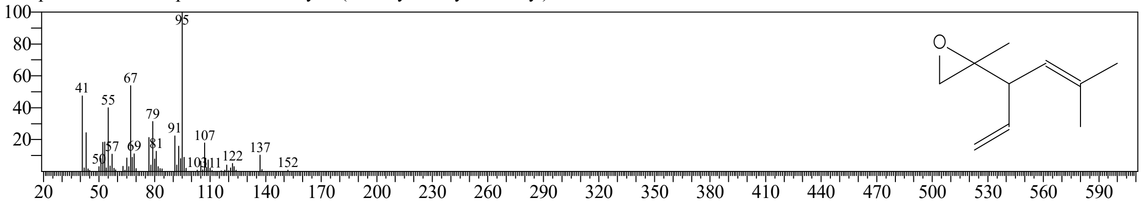


Hit#:5 Entry:16582 Library:NIST17-1.lib
SI:84 Formula:C11H18 CAS:0-00-0 MolWeight:150 RetIndex:1115
CompName:4-Cyclohexenyl-3-methylbutene

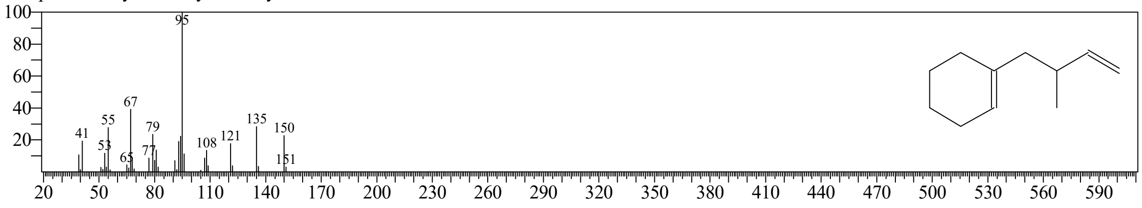

<< Target >>
Line#:22 R.Time:33.492(Scan#:2820) MassPeaks:338
RawMode:Averaged 33.483-33.500(2819-2821) BasePeak:74.00(10537)
BG Mode:Calc. from Peak Group 1 - Event 1

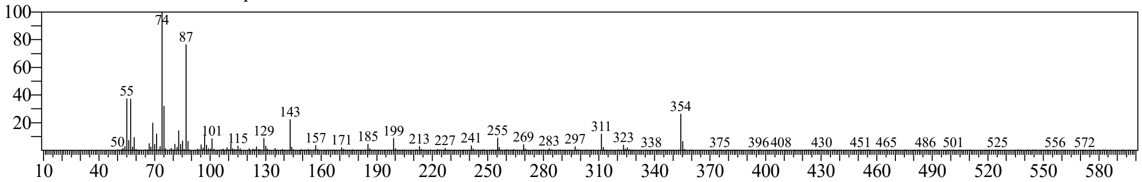


Hit#:1 Entry:196443 Library:NIST17-1.lib
SI:96 Formula:C23H46O2 CAS:929-77-1 MolWeight:354 RetIndex:2475
CompName:Docosanoic acid, methyl ester $$ Behenic acid, methyl ester $$ Methyl behenate $$ Methyl docosanoate $$ n-Docosanoic acid methyl ester $$

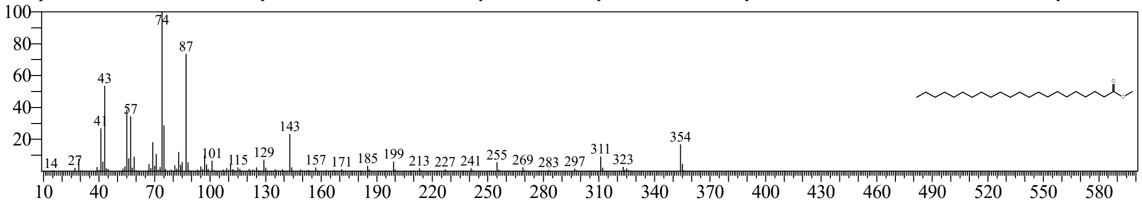


Hit#:2 Entry:178051 Library:NIST14.lib
SI:96 Formula:C23H46O2 CAS:929-77-1 MolWeight:354 RetIndex:2475
CompName:Docosanoic acid, methyl ester $$ Behenic acid, methyl ester $$ Methyl behenate $$ Methyl docosanoate $$ n-Docosanoic acid methyl ester $$

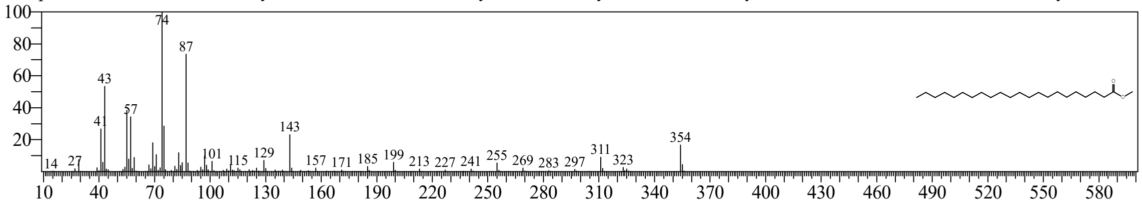


Hit#:3 Entry:196444 Library:NIST17-1.lib
SI:95 Formula:C23H46O2 CAS:0-00-0 MolWeight:354 RetIndex:2411
CompName:Methyl 20-methyl-heneicosanoate

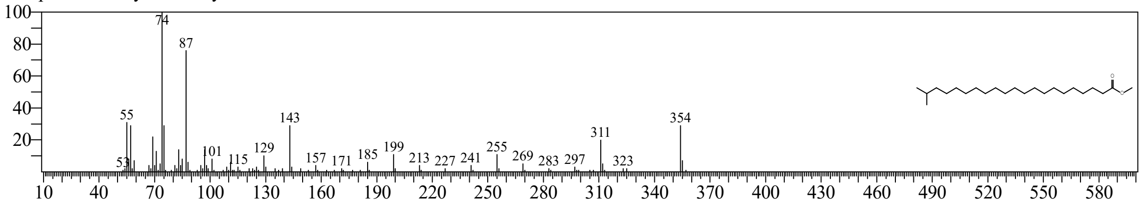


Hit#:4 Entry:178052 Library:NIST14.lib
SI:95 Formula:C23H46O2 CAS:0-00-0 MolWeight:354 RetIndex:2411
CompName:Methyl 20-methyl-heneicosanoate

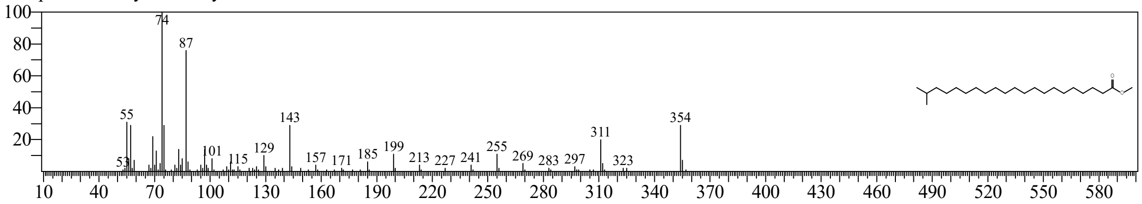


Hit#:5 Entry:31088 Library:NIST14s.lib
SI:95 Formula:C23H46O2 CAS:929-77-1 MolWeight:354 RetIndex:2475
CompName:Docosanoic acid, methyl ester $$ Behenic acid, methyl ester $$ Methyl behenate $$ Methyl docosanoate $$ n-Docosanoic acid methyl ester $$

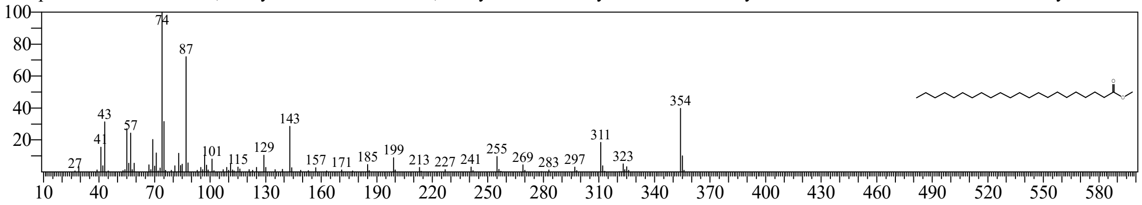

<< Target >>
Line#:23  R.Time:37.800(Scan#:3337)  MassPeaks:310
RawMode:Averaged 37.792-37.808(3336-3338) BasePeak:74.00(2517)
BG Mode:Calc. from Peak  Group 1 - Event 1

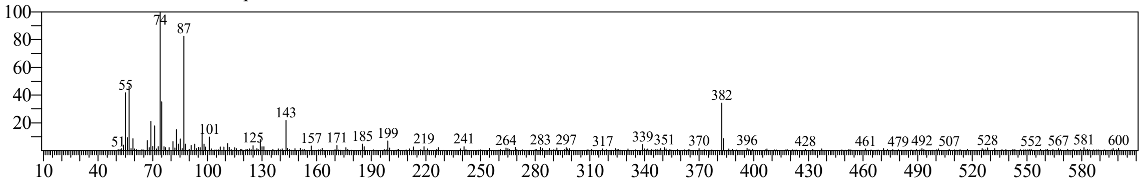


Hit#:1  Entry:31970  Library:NIST14s.lib
SI:91  Formula:C25H50O2  CAS:2442-49-1  MolWeight:382  RetIndex:2674
CompName:Tetracosanoic acid, methyl ester $$ Methyl lignocerate $$ Methyl tetracosanoate $$ Lignoceric acid methyl ester $$

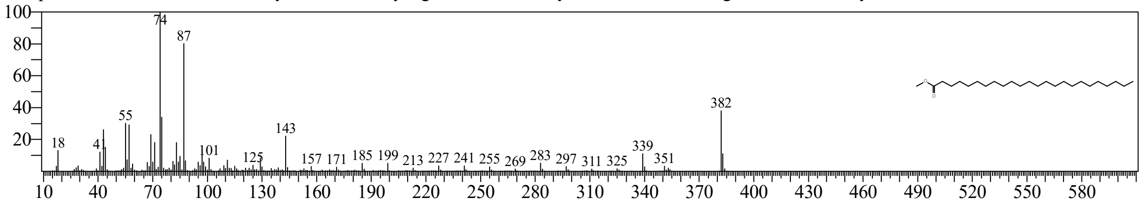


Hit#:2  Entry:37106  Library:NIST17s.lib
SI:91  Formula:C25H50O2  CAS:2442-49-1  MolWeight:382  RetIndex:2674
CompName:Tetracosanoic acid, methyl ester $$ Methyl lignocerate $$ Methyl tetracosanoate $$ Lignoceric acid methyl ester $$

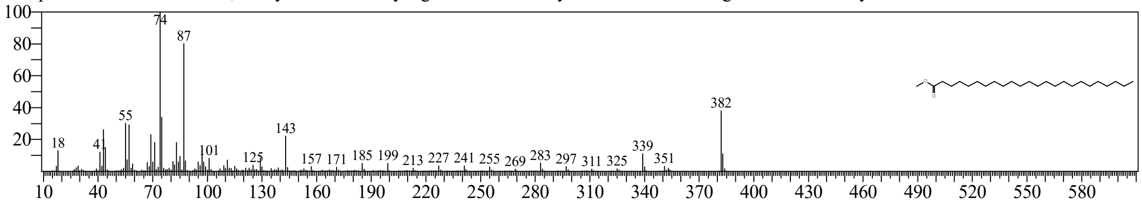


Hit#:3  Entry:37105  Library:NIST17s.lib
SI:89  Formula:C25H50O2  CAS:2442-49-1  MolWeight:382  RetIndex:2674
CompName:Tetracosanoic acid, methyl ester $$ Methyl lignocerate $$ Methyl tetracosanoate $$ Lignoceric acid methyl ester $$

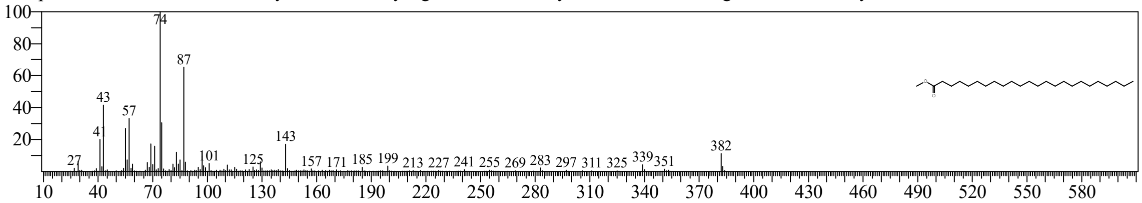


Hit#:4  Entry:31969  Library:NIST14s.lib
SI:89  Formula:C25H50O2  CAS:2442-49-1  MolWeight:382  RetIndex:2674
CompName:Tetracosanoic acid, methyl ester $$ Methyl lignocerate $$ Methyl tetracosanoate $$ Lignoceric acid methyl ester $$

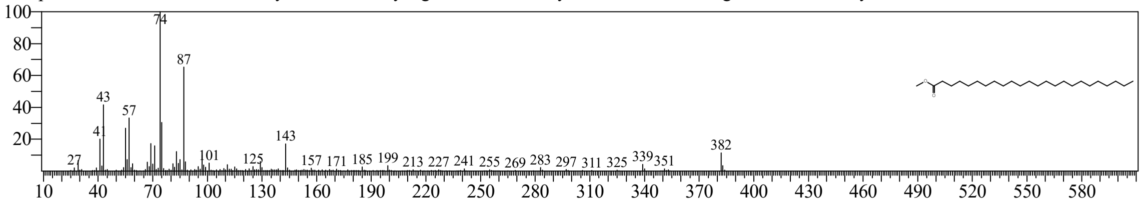


Hit#:5  Entry:196723  Library:NIST14.lib
SI:88  Formula:C25H50O2  CAS:2442-49-1  MolWeight:382  RetIndex:2674
CompName:Tetracosanoic acid, methyl ester $$ Methyl lignocerate $$ Methyl tetracosanoate $$ Lignoceric acid methyl ester $$

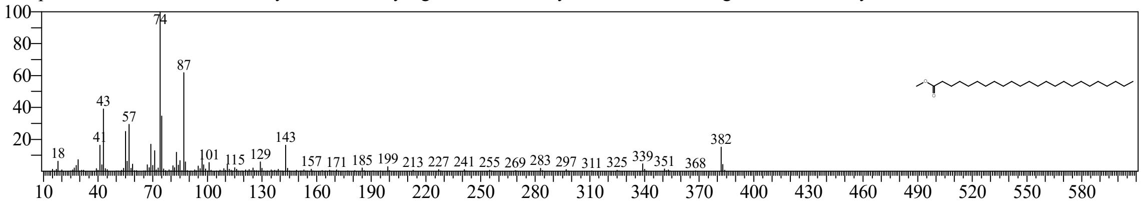

Supplement: Supplementary file 1 [file molecules-31-02891-s001.zip › 14d-CK-2.pdf]
