# Supplementary material for: Selective Inhibition of Lipid Oxidation by a Quercetagetin-Rich Extract from Tagetes erecta: A Chemometric Approach Using OPLS-DA and Selectivity Index Analysis
Source: Molecules. 2026 Aug 19;31(16):2891. doi: 10.3390/molecules31162891 (PMC13515886; doi:10.3390/molecules31162891)

PG-3-14d

<<Column>>
Name : HP-88
Thickness : 0.20um
Length : 100.0m
Inside Diameter : 0.25mm

Method

===== Analytical Line 1 =====

Column Oven Temp.	:120.0 °C	
Injection Temp.	:250.00 °C	
Injection Mode	:Split	
Column Flow	:1.04 mL/min	
Split Ratio	:20.0	
Oven Temp. Program		
Rate	Temperature(°C)	Hold Time(min)
-	120.0	1.00
10.00	175.0	10.00
5.00	210.0	5.00
5.00	230.0	15.00

[GCMS-QP2010]
IonSourceTemp :200.00 °C
Interface Temp. :220.00 °C
m/z scanning range：45～500m/z

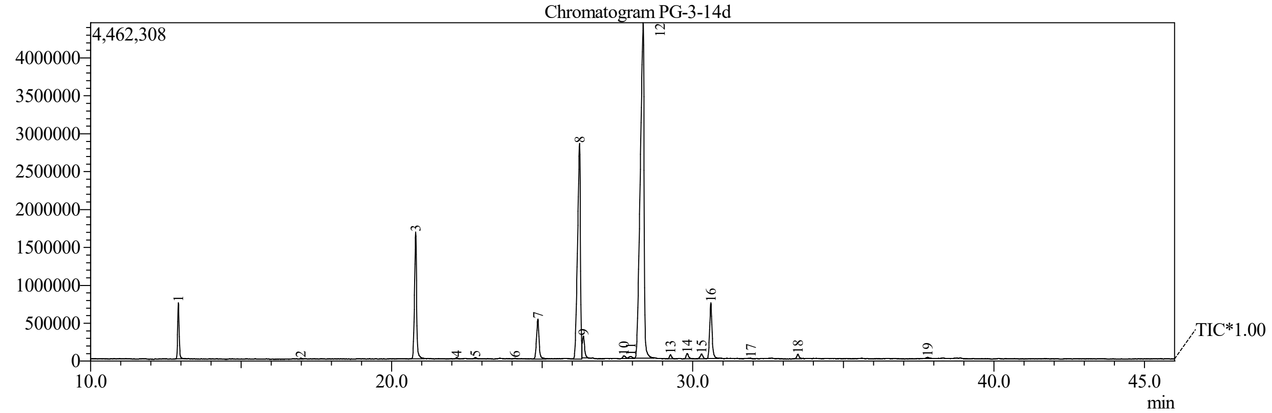

Peak Report TIC

Peak#	R.Time	Area	Area%	Height	Height%	A/H	Name
1	12.919	2578613	3.23	738464	6.36	3.49	Undecanoic acid, methyl ester
2	16.983	51575	0.06	12201	0.11	4.23	Methyl tetradecanoate
3	20.801	8179971	10.25	1675143	14.42	4.88	Hexadecanoic acid, methyl ester
4	22.161	77511	0.10	17071	0.15	4.54	7-Hexadecenoic acid, methyl ester, (Z)-
5	22.778	89459	0.11	16462	0.14	5.43	Heptadecanoic acid, methyl ester
6	24.094	42540	0.05	8179	0.07	5.20	cis-10-Heptadecenoic acid, methyl ester
7	24.856	3111546	3.90	527015	4.54	5.90	Methyl stearate
8	26.239	18858830	23.63	2842737	24.48	6.63	9-Octadecenoic acid, methyl ester, (E)-
9	26.368	1468237	1.84	288573	2.48	5.09	cis-13-Octadecenoic acid, methyl ester
10	27.710	227374	0.28	35970	0.31	6.32	9,12-Octadecadienoic acid (Z,Z)-, methyl este
11	27.940	173806	0.22	24658	0.21	7.05	9,12-Octadecadienoic acid, methyl ester
12	28.353	39248947	49.19	4422166	38.08	8.88	9,12-Octadecadienoic acid (Z,Z)-, methyl este
13	29.259	242369	0.30	51575	0.44	4.70	Methyl 18-methylnonadecanoate
14	29.814	412434	0.52	70210	0.60	5.87	9,12,15-Octadecatrienoic acid, methyl ester, (Z

Peak#	R.Time	Area	Area%	Height	Height%	A/H	Name
15	30.296	382385	0.48	62718	0.54	6.10	9,12,15-Octadecatrienoic acid, methyl ester, (Z
16	30.600	4189296	5.25	737079	6.35	5.68	9,12,15-Octadecatrienoic acid, methyl ester, (Z
17	31.925	29033	0.04	6748	0.06	4.30	9,11-Octadecadienoic acid, methyl ester, (E,E)
18	33.490	343363	0.43	61814	0.53	5.55	Docosanoic acid, methyl ester
19	37.799	88062	0.11	15200	0.13	5.79	Tetracosanoic acid, methyl ester
		79795351	100.00	11613983	100.00		

Library

<< Target >>
Line#:1 R.Time:12.917(Scan#:351) MassPeaks:312
RawMode:Averaged 12.908-12.925(350-352) BasePeak:74.00(216988)
BG Mode:Calc. from Peak Group 1 - Event 1

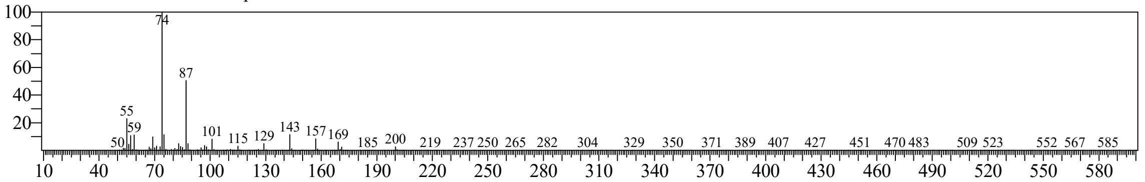

Hit#:1 Entry:46966 Library:NIST14.lib
SI:95 Formula:C12H24O2 CAS:1731-86-8 MolWeight:200 RetIndex:1381
CompName:Undecanoic acid, methyl ester $$ Methyl undecanoate $$ Methyl undecylenate $$ n-Undecanoic acid methyl ester $$ Methyl ester of undecanoi

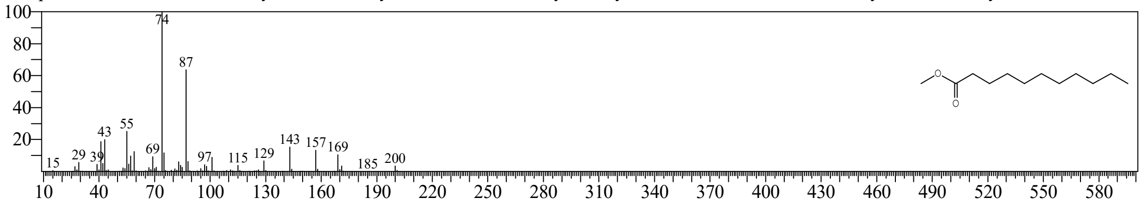

Hit#:2 Entry:49598 Library:NIST17-1.lib
SI:95 Formula:C12H24O2 CAS:1731-86-8 MolWeight:200 RetIndex:1381
CompName:Undecanoic acid, methyl ester $$ Methyl undecanoate $$ Methyl undecylenate $$ n-Undecanoic acid methyl ester $$ Methyl ester of undecanoi

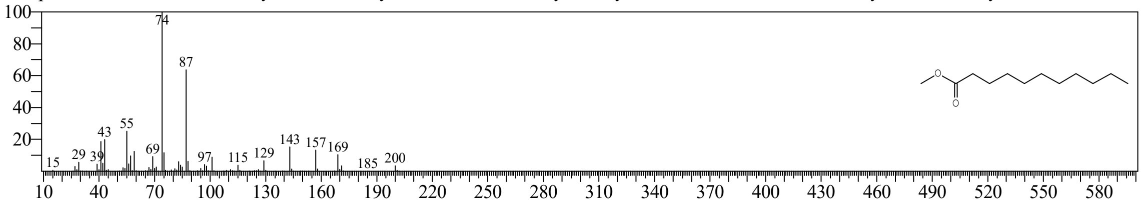

Hit#:3 Entry:21618 Library:NIST17s.lib
SI:95 Formula:C12H24O2 CAS:1731-86-8 MolWeight:200 RetIndex:1381
CompName:Undecanoic acid, methyl ester $$ Methyl undecanoate $$ Methyl undecylenate $$ n-Undecanoic acid methyl ester $$ Methyl ester of undecanoi

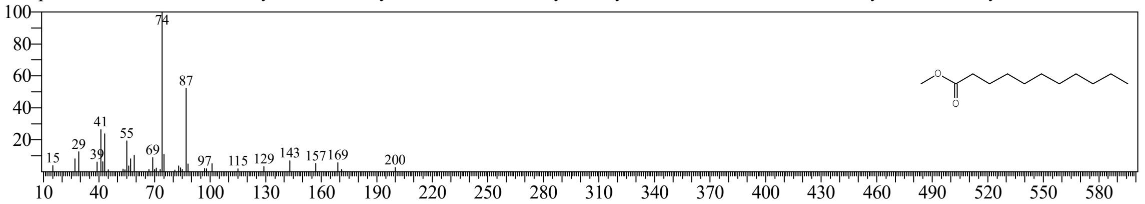

Hit#:4 Entry:18487 Library:NIST14s.lib
SI:95 Formula:C12H24O2 CAS:1731-86-8 MolWeight:200 RetIndex:1381
CompName:Undecanoic acid, methyl ester $$ Methyl undecanoate $$ Methyl undecylenate $$ n-Undecanoic acid methyl ester $$ Methyl ester of undecanoi

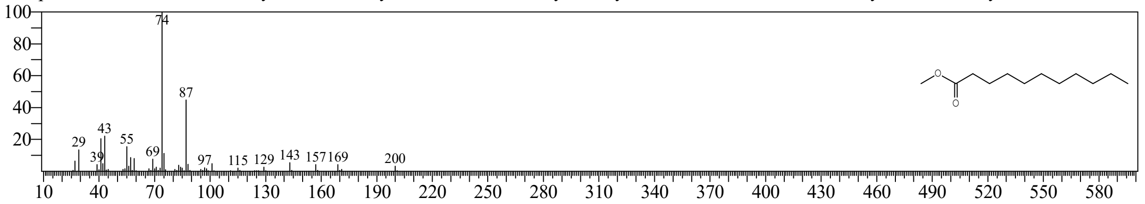

Hit#:5 Entry:21619 Library:NIST17s.lib
SI:95 Formula:C12H24O2 CAS:1731-86-8 MolWeight:200 RetIndex:1381
CompName:Undecanoic acid, methyl ester $$ Methyl undecanoate $$ Methyl undecylenate $$ n-Undecanoic acid methyl ester $$ Methyl ester of undecanoi

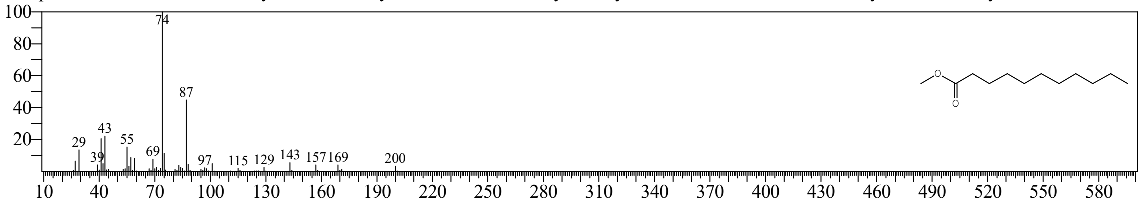

<< Target >>
Line#:2 R.Time:16.983(Scan#:839) MassPeaks:306
RawMode:Averaged 16.975-16.992(838-840) BasePeak:74.00(2463)
BG Mode:Calc. from Peak Group 1 - Event 1

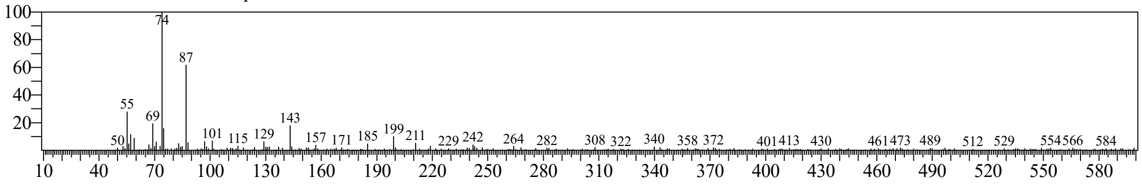

Hit#:1 Entry:80645 Library:NIST14.lib
SI:90 Formula:C15H30O2 CAS:124-10-7 MolWeight:242 RetIndex:1680
CompName:Methyl tetradecanoate $$ Tetradecanoic acid, methyl ester $$ Myristic acid, methyl ester $$ Metholeneat 2495 $$ Methyl myristate $$ Methyl r

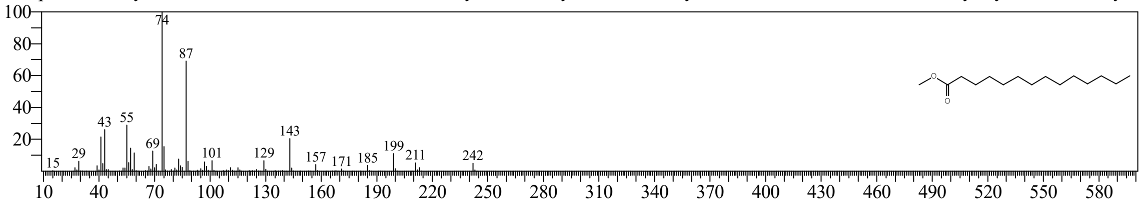

Hit#:2 Entry:86786 Library:NIST17-1.lib
SI:90 Formula:C15H30O2 CAS:124-10-7 MolWeight:242 RetIndex:1680
CompName:Methyl tetradecanoate $$ Tetradecanoic acid, methyl ester $$ Myristic acid, methyl ester $$ Metholeneat 2495 $$ Methyl myristate $$ Methyl r

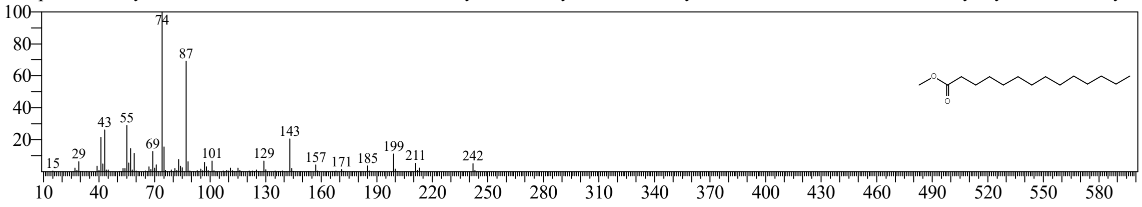

Hit#:3 Entry:27875 Library:NIST17s.lib
SI:89 Formula:C15H30O2 CAS:124-10-7 MolWeight:242 RetIndex:1680
CompName:Methyl tetradecanoate $$ Tetradecanoic acid, methyl ester $$ Myristic acid, methyl ester $$ Metholeneat 2495 $$ Methyl myristate $$ Methyl r

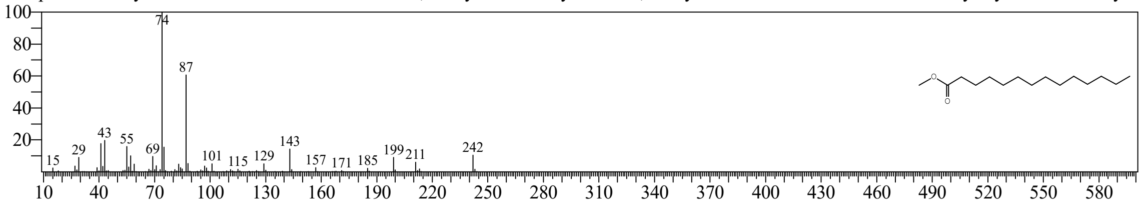

Hit#:4 Entry:23849 Library:NIST14s.lib
SI:89 Formula:C15H30O2 CAS:124-10-7 MolWeight:242 RetIndex:1680
CompName:Methyl tetradecanoate $$ Tetradecanoic acid, methyl ester $$ Myristic acid, methyl ester $$ Metholeneat 2495 $$ Methyl myristate $$ Methyl r

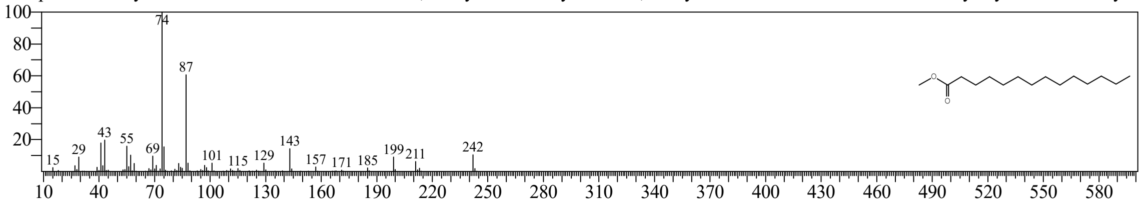

Hit#:5 Entry:86785 Library:NIST17-1.lib
SI:89 Formula:C15H30O2 CAS:5129-58-8 MolWeight:242 RetIndex:1615
CompName:Tridecanoic acid, 12-methyl-, methyl ester $$ Methyl isomyristate $$ Methyl 12-methyltridecanoate $$

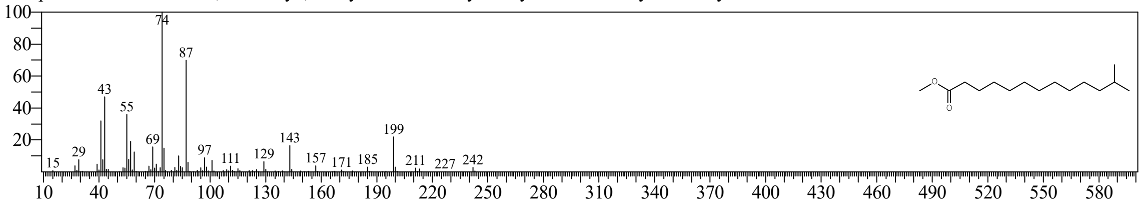

<< Target >>
Line#:3 R.Time:20.800(Scan#:1297) MassPeaks:351
RawMode:Averaged 20.792-20.808(1296-1298) BasePeak:74.00(387677)
BG Mode:Calc. from Peak Group 1 - Event 1

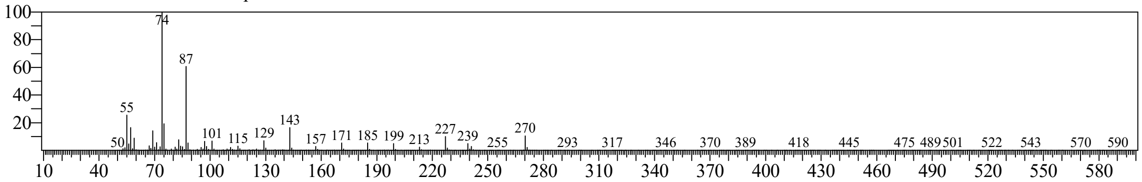

Hit#:1 Entry:113663 Library:NIST17-1.lib
SI:97 Formula:C17H34O2 CAS:112-39-0 MolWeight:270 RetIndex:1878
CompName:Hexadecanoic acid, methyl ester $$ Palmitic acid, methyl ester $$ n-Hexadecanoic acid methyl ester $$ Metholene 2216 $$ Methyl hexadecano

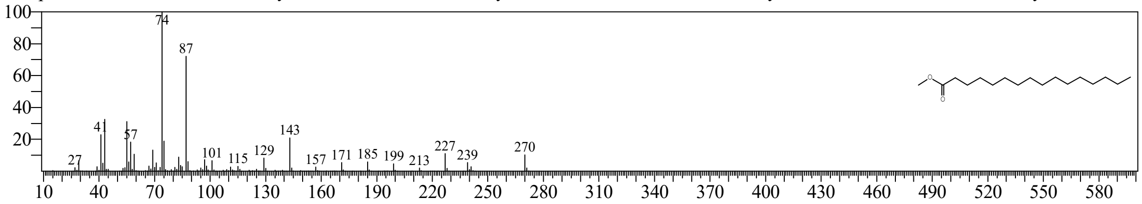

Hit#:2 Entry:104648 Library:NIST14.lib
SI:97 Formula:C17H34O2 CAS:112-39-0 MolWeight:270 RetIndex:1878
CompName:Hexadecanoic acid, methyl ester $$ Palmitic acid, methyl ester $$ n-Hexadecanoic acid methyl ester $$ Metholene 2216 $$ Methyl hexadecano

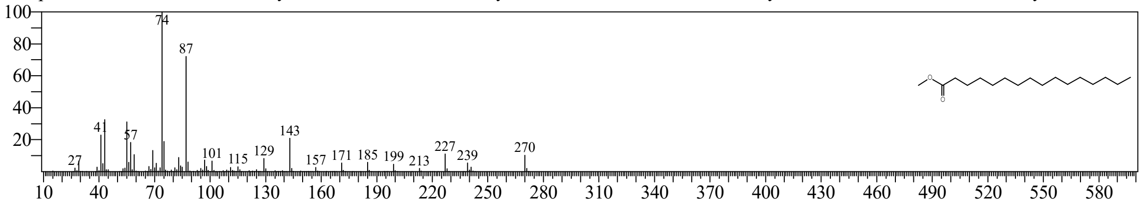

Hit#:3 Entry:26273 Library:NIST14s.lib
SI:96 Formula:C17H34O2 CAS:112-39-0 MolWeight:270 RetIndex:1878
CompName:Hexadecanoic acid, methyl ester $$ Palmitic acid, methyl ester $$ n-Hexadecanoic acid methyl ester $$ Metholene 2216 $$ Methyl hexadecano

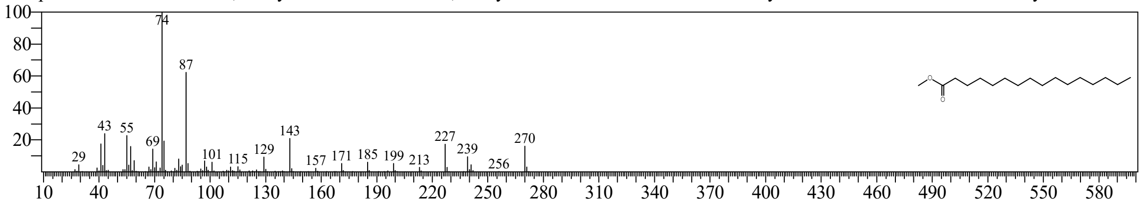

Hit#:4 Entry:26271 Library:NIST14s.lib
SI:96 Formula:C17H34O2 CAS:112-39-0 MolWeight:270 RetIndex:1878
CompName:Hexadecanoic acid, methyl ester $$ Palmitic acid, methyl ester $$ n-Hexadecanoic acid methyl ester $$ Metholene 2216 $$ Methyl hexadecano

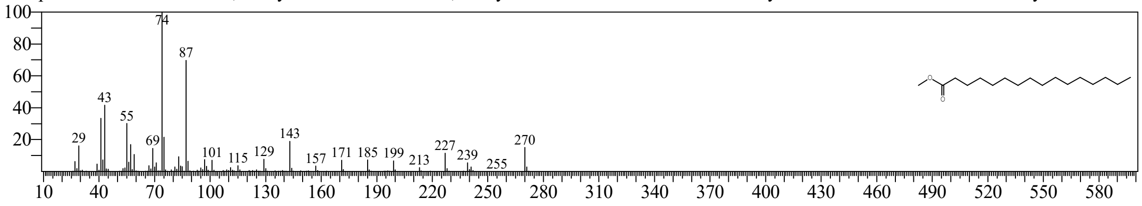

Hit#:5 Entry:30646 Library:NIST17s.lib
SI:96 Formula:C17H34O2 CAS:112-39-0 MolWeight:270 RetIndex:1878
CompName:Hexadecanoic acid, methyl ester $$ Palmitic acid, methyl ester $$ n-Hexadecanoic acid methyl ester $$ Metholene 2216 $$ Methyl hexadecano

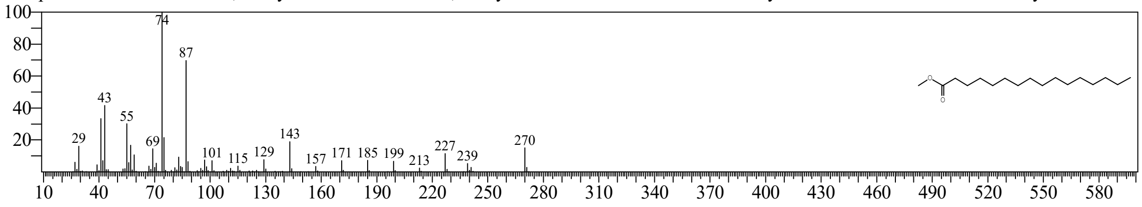

<< Target >>
Line#:4 R.Time:22.158(Scan#:1460) MassPeaks:326
RawMode:Averaged 22.150-22.167(1459-1461) BasePeak:55.05(1331)
BG Mode:Calc. from Peak Group 1 - Event 1

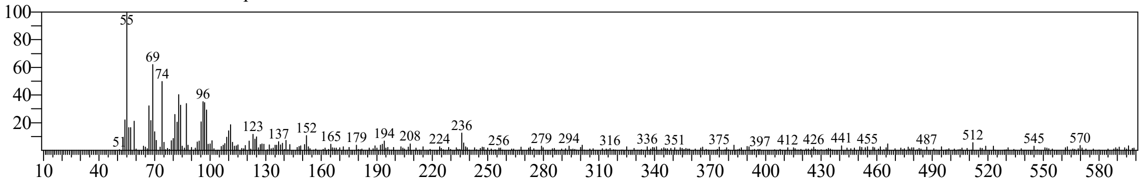

Hit#:1 Entry:111510 Library:NIST17-1.lib
SI:91 Formula:C17H32O2 CAS:56875-67-3 MolWeight:268 RetIndex:1886
CompName:7-Hexadecenoic acid, methyl ester, (Z)- $$ Methyl (7E)-7-hexadecenoate # $$

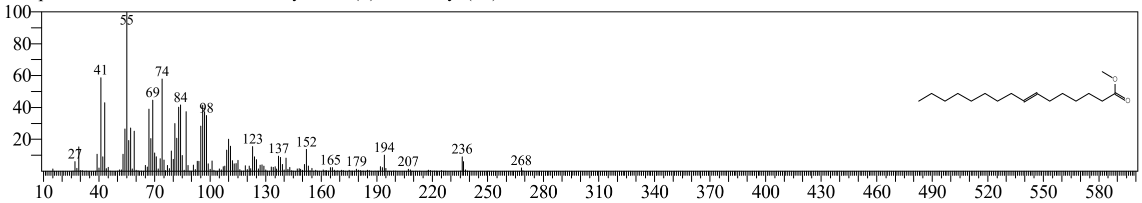

Hit#:2 Entry:30415 Library:NIST17s.lib
SI:91 Formula:C17H32O2 CAS:1120-25-8 MolWeight:268 RetIndex:1886
CompName:9-Hexadecenoic acid, methyl ester, (Z)- $$ Methyl palmitoleate $$ Methyl palmitoleinate $$ Palmitoleic acid, methyl ester $$ Methyl (9Z)-9-he

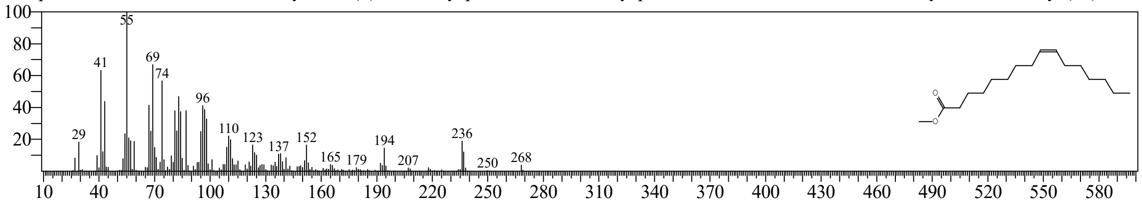

Hit#:3 Entry:26066 Library:NIST14s.lib
SI:91 Formula:C17H32O2 CAS:1120-25-8 MolWeight:268 RetIndex:1886
CompName:9-Hexadecenoic acid, methyl ester, (Z)- $$ Methyl palmitoleate $$ Methyl palmitoleinate $$ Palmitoleic acid, methyl ester $$ Methyl (9Z)-9-he

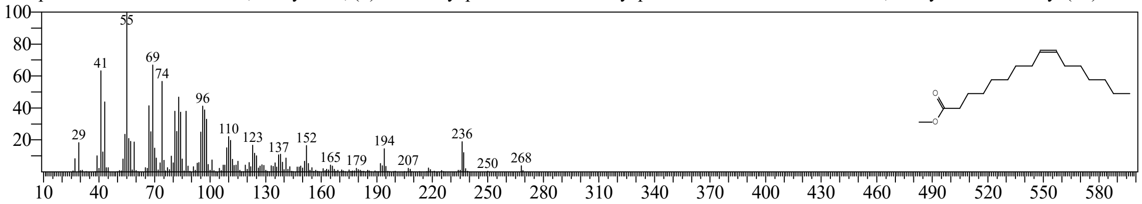

Hit#:4 Entry:26064 Library:NIST14s.lib
SI:91 Formula:C17H32O2 CAS:1120-25-8 MolWeight:268 RetIndex:1886
CompName:9-Hexadecenoic acid, methyl ester, (Z)- $$ Methyl palmitoleate $$ Methyl palmitoleinate $$ Palmitoleic acid, methyl ester $$ Methyl (9Z)-9-he

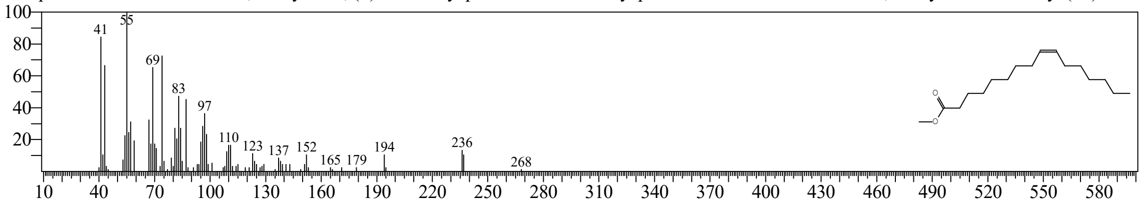

Hit#:5 Entry:30413 Library:NIST17s.lib
SI:91 Formula:C17H32O2 CAS:1120-25-8 MolWeight:268 RetIndex:1886
CompName:9-Hexadecenoic acid, methyl ester, (Z)- $$ Methyl palmitoleate $$ Methyl palmitoleinate $$ Palmitoleic acid, methyl ester $$ Methyl (9Z)-9-he

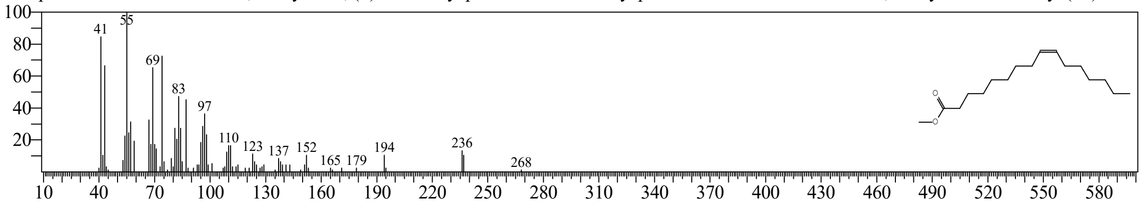

<< Target >>
Line#:5 R.Time:22.775(Scan#:1534) MassPeaks:344
RawMode:Averaged 22.767-22.783(1533-1535) BasePeak:74.00(2692)
BG Mode:Calc. from Peak Group 1 - Event 1

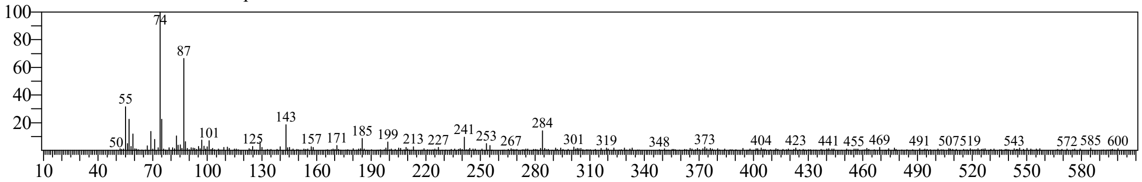

Hit#:1 Entry:27279 Library:NIST14s.lib
SI:92 Formula:C18H36O2 CAS:1731-92-6 MolWeight:284 RetIndex:1978
CompName:Heptadecanoic acid, methyl ester $$ Margaric acid methyl ester $$ Methyl heptadecanoate $$ Methyl margarate $$ n-Heptadecanoic acid methy

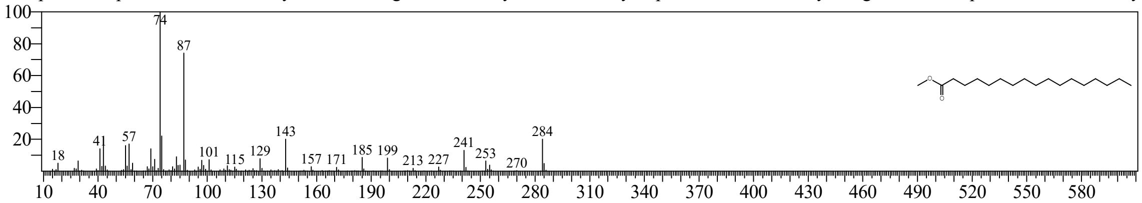

Hit#:2 Entry:31787 Library:NIST17s.lib
SI:92 Formula:C18H36O2 CAS:1731-92-6 MolWeight:284 RetIndex:1978
CompName:Heptadecanoic acid, methyl ester $$ Margaric acid methyl ester $$ Methyl heptadecanoate $$ Methyl margarate $$ n-Heptadecanoic acid methy

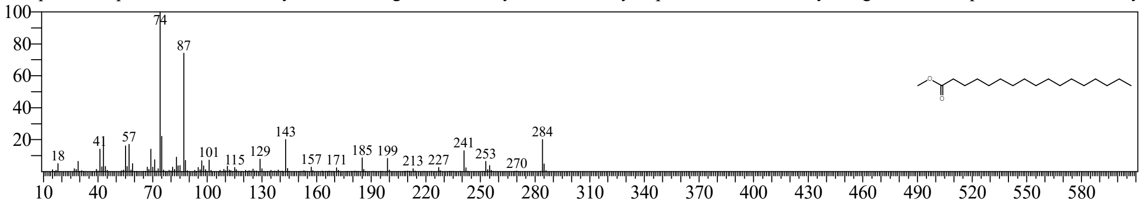

Hit#:3 Entry:27275 Library:NIST14s.lib
SI:91 Formula:C18H36O2 CAS:1731-92-6 MolWeight:284 RetIndex:1978
CompName:Heptadecanoic acid, methyl ester $$ Margaric acid methyl ester $$ Methyl heptadecanoate $$ Methyl margarate $$ n-Heptadecanoic acid methy

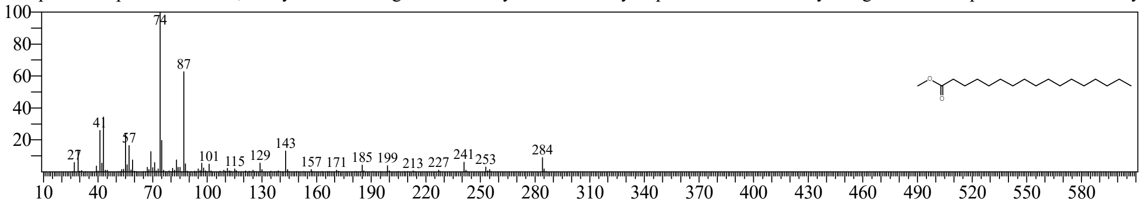

Hit#:4 Entry:31784 Library:NIST17s.lib
SI:91 Formula:C18H36O2 CAS:1731-92-6 MolWeight:284 RetIndex:1978
CompName:Heptadecanoic acid, methyl ester $$ Margaric acid methyl ester $$ Methyl heptadecanoate $$ Methyl margarate $$ n-Heptadecanoic acid methy

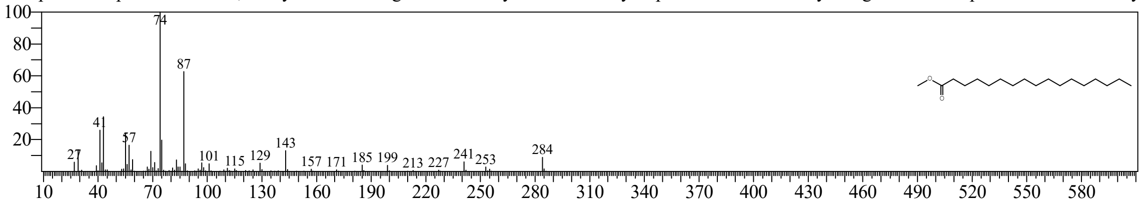

Hit#:5 Entry:127714 Library:NIST17-1.lib
SI:91 Formula:C18H36O2 CAS:6929-04-0 MolWeight:284 RetIndex:1914
CompName:Hexadecanoic acid, 15-methyl-, methyl ester $$ Methyl isoheptadecanoate $$ Methyl 15-methylhexadecanoate $$

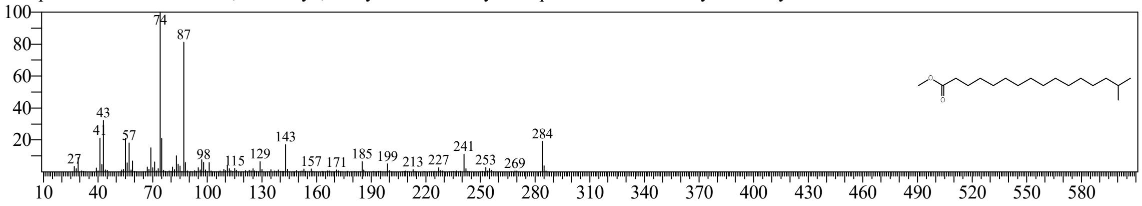

<< Target >>
Line#:6 R.Time:24.092(Scan#:1692) MassPeaks:341
RawMode:Averaged 24.083-24.100(1691-1693) BasePeak:55.05(634)
BG Mode:Calc. from Peak Group 1 - Event 1

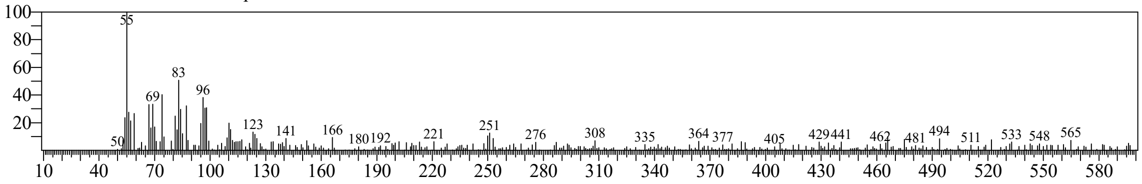

Hit#:1 Entry:115094 Library:NIST14.lib
SI:86 Formula:C18H34O2 CAS:0-00-0 MolWeight:282 RetIndex:1986
CompName:cis-10-Heptadecenoic acid, methyl ester

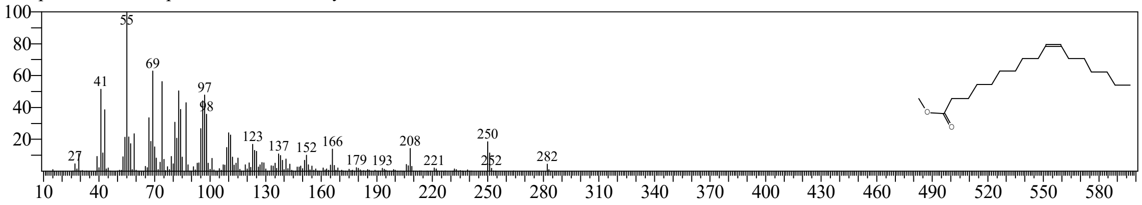

Hit#:2 Entry:125438 Library:NIST17-1.lib
SI:86 Formula:C18H34O2 CAS:0-00-0 MolWeight:282 RetIndex:1986
CompName:cis-10-Heptadecenoic acid, methyl ester

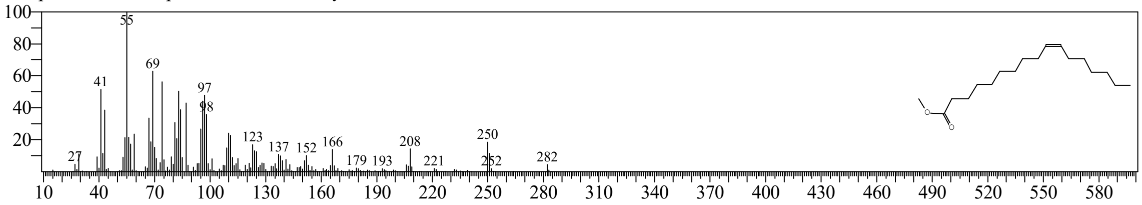

Hit#:3 Entry:115096 Library:NIST14.lib
SI:85 Formula:C18H34O2 CAS:0-00-0 MolWeight:282 RetIndex:1986
CompName:Methyl 9-heptadecenoate or 9-17:1

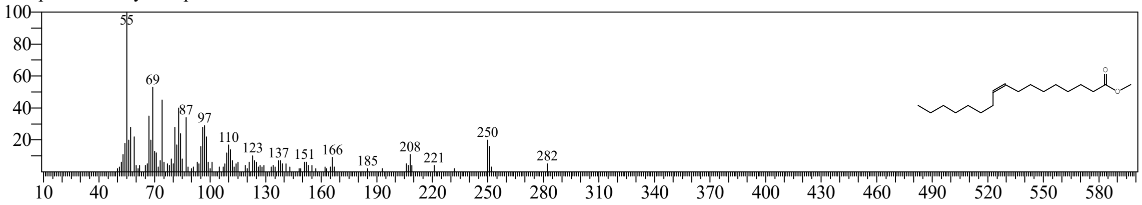

Hit#:4 Entry:125440 Library:NIST17-1.lib
SI:85 Formula:C18H34O2 CAS:0-00-0 MolWeight:282 RetIndex:1986
CompName:Methyl 9-heptadecenoate or 9-17:1

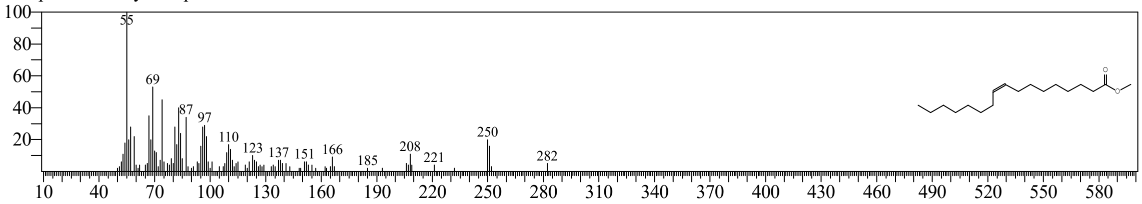

Hit#:5 Entry:27110 Library:NIST14s.lib
SI:85 Formula:C18H34O2 CAS:10152-61-1 MolWeight:282 RetIndex:1941
CompName:Cyclopropaneoctanoic acid, 2-hexyl-, methyl ester $$ Methyl 8-(2-hexylcyclopropyl)octanoate # $$

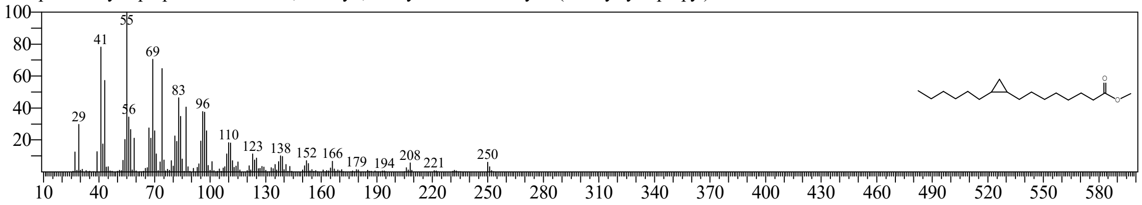

<< Target >>
Line#:7 R.Time:24.858(Scan#:1784) MassPeaks:371
RawMode:Averaged 24.850-24.867(1783-1785) BasePeak:74.00(105995)
BG Mode:Calc. from Peak Group 1 - Event 1

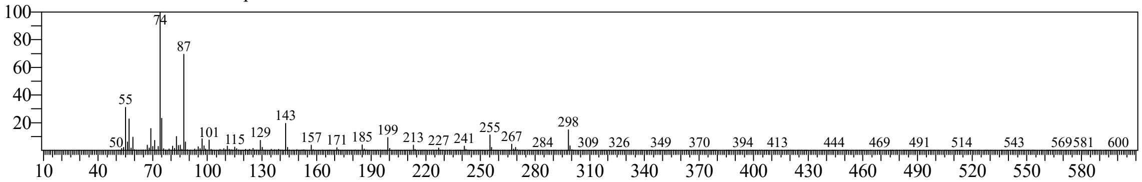

Hit#:1 Entry:129694 Library:NIST14.lib
SI:98 Formula:C19H38O2 CAS:112-61-8 MolWeight:298 RetIndex:2077
CompName:Methyl stearate $$ Octadecanoic acid, methyl ester $$ Stearic acid, methyl ester $$ n-Octadecanoic acid, methyl ester $$ Kemester 9718 $$ Me

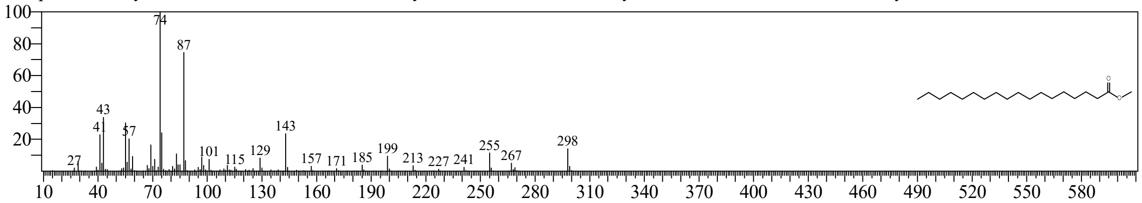

Hit#:2 Entry:141978 Library:NIST17-1.lib
SI:98 Formula:C19H38O2 CAS:112-61-8 MolWeight:298 RetIndex:2077
CompName:Methyl stearate $$ Octadecanoic acid, methyl ester $$ Stearic acid, methyl ester $$ n-Octadecanoic acid, methyl ester $$ Kemester 9718 $$ Me

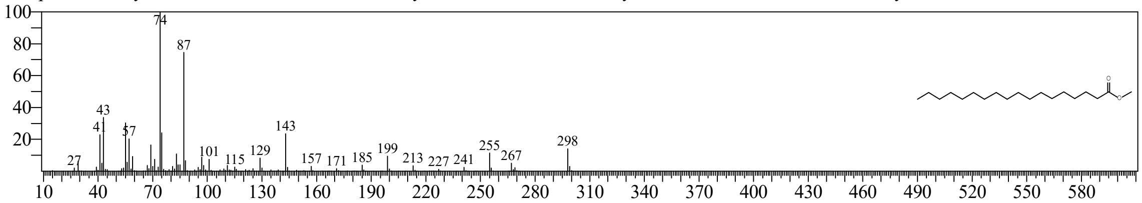

Hit#:3 Entry:28255 Library:NIST14s.lib
SI:97 Formula:C19H38O2 CAS:112-61-8 MolWeight:298 RetIndex:2077
CompName:Methyl stearate $$ Octadecanoic acid, methyl ester $$ Stearic acid, methyl ester $$ n-Octadecanoic acid, methyl ester $$ Kemester 9718 $$ Me

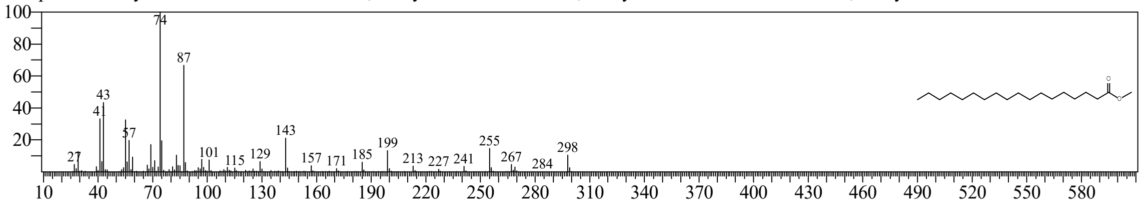

Hit#:4 Entry:32873 Library:NIST17s.lib
SI:97 Formula:C19H38O2 CAS:112-61-8 MolWeight:298 RetIndex:2077
CompName:Methyl stearate $$ Octadecanoic acid, methyl ester $$ Stearic acid, methyl ester $$ n-Octadecanoic acid, methyl ester $$ Kemester 9718 $$ Me

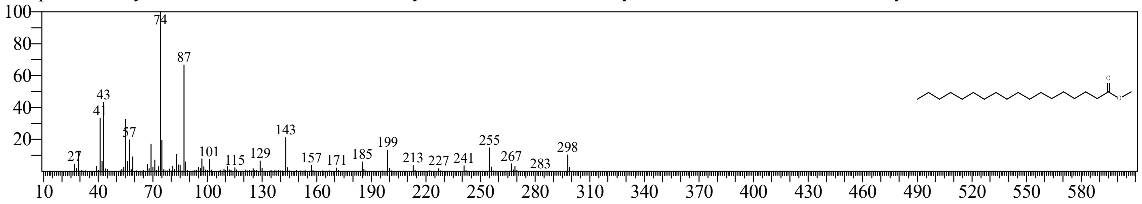

Hit#:5 Entry:28257 Library:NIST14s.lib
SI:97 Formula:C19H38O2 CAS:112-61-8 MolWeight:298 RetIndex:2077
CompName:Methyl stearate $$ Octadecanoic acid, methyl ester $$ Stearic acid, methyl ester $$ n-Octadecanoic acid, methyl ester $$ Kemester 9718 $$ Me

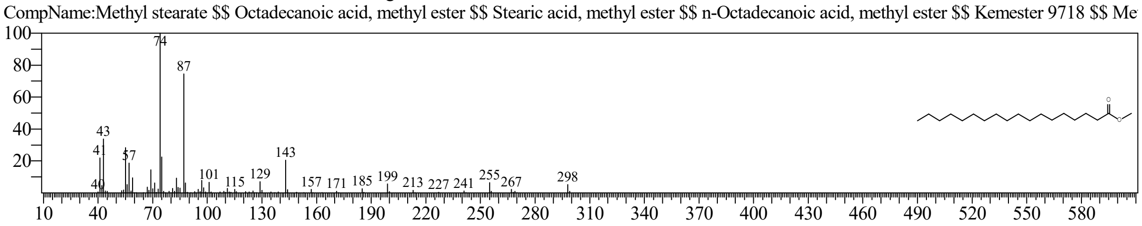

<< Target >>
Line#:8 R.Time:26.242(Scan#:1950) MassPeaks:393
RawMode:Averaged 26.233-26.250(1949-1951) BasePeak:55.05(217691)
BG Mode:Calc. from Peak Group 1 - Event 1

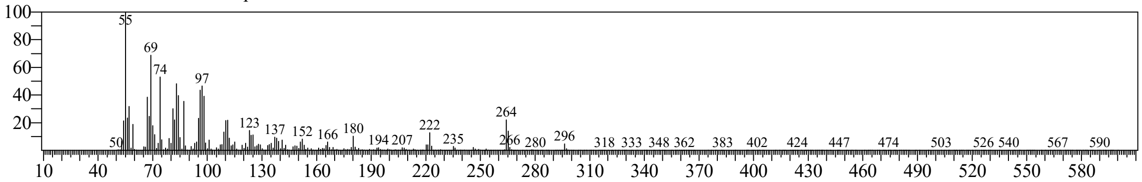

Hit#:1 Entry:32747 Library:NIST17s.lib
SI:96 Formula:C19H36O2 CAS:1937-62-8 MolWeight:296 RetIndex:2085
CompName:9-Octadecenoic acid, methyl ester, (E)- $$ Elaidic acid, methyl ester $$ Methyl elaidate $$ Methyl trans-9-octadecenoate $$ (E)-9-Octadecenoic

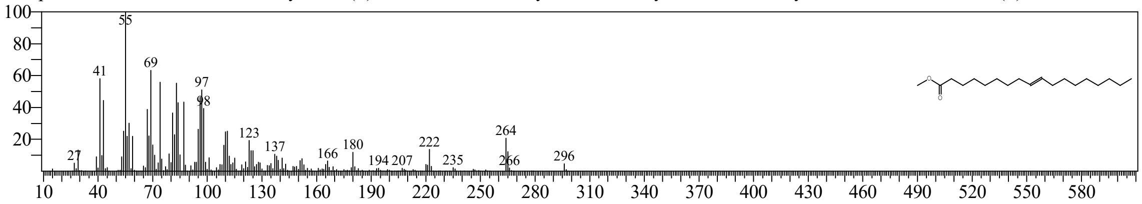

Hit#:2 Entry:28138 Library:NIST14s.lib
SI:96 Formula:C19H36O2 CAS:1937-62-8 MolWeight:296 RetIndex:2085
CompName:9-Octadecenoic acid, methyl ester, (E)- $$ Elaidic acid, methyl ester $$ Methyl elaidate $$ Methyl trans-9-octadecenoate $$ (E)-9-Octadecenoic

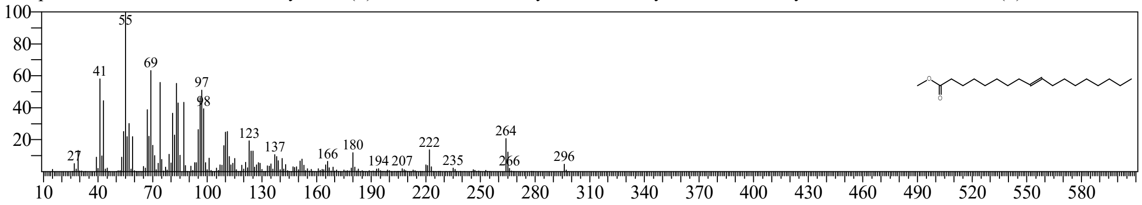

Hit#:3 Entry:28139 Library:NIST14s.lib
SI:96 Formula:C19H36O2 CAS:52380-33-3 MolWeight:296 RetIndex:2085
CompName:11-Octadecenoic acid, methyl ester $$ Methyl 11-octadecenoate $$ Octadec-11-enoic acid, methyl ester $$ Methyl (11E)-11-octadecenoate # $$

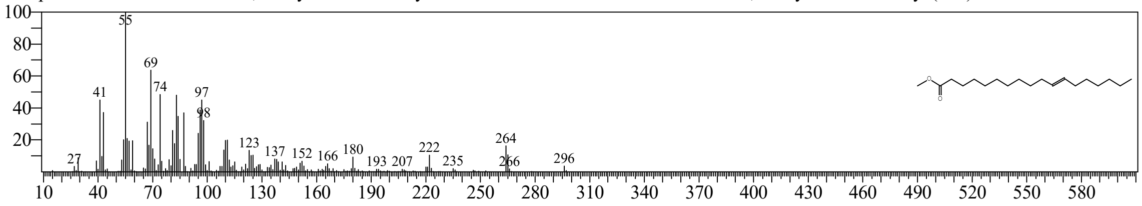

Hit#:4 Entry:32748 Library:NIST17s.lib
SI:96 Formula:C19H36O2 CAS:52380-33-3 MolWeight:296 RetIndex:2085
CompName:11-Octadecenoic acid, methyl ester $$ Methyl 11-octadecenoate $$ Octadec-11-enoic acid, methyl ester $$ Methyl (11E)-11-octadecenoate # $$

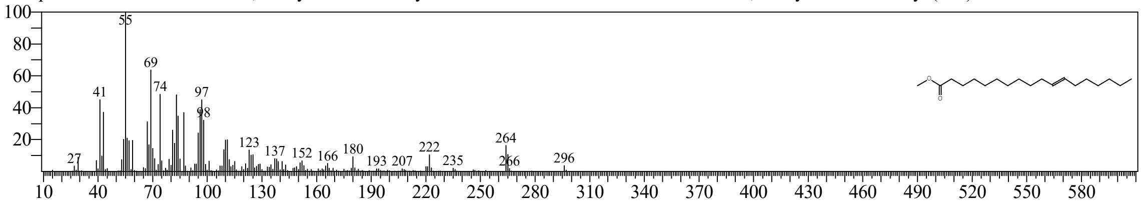

Hit#:5 Entry:127664 Library:NIST14.lib
SI:96 Formula:C19H36O2 CAS:112-62-9 MolWeight:296 RetIndex:2085
CompName:9-Octadecenoic acid (Z)-, methyl ester $$ Oleic acid, methyl ester $$ Emery oleic acid ester 2301 $$ Methyl cis-9-octadecenoate $$ Methyl ole

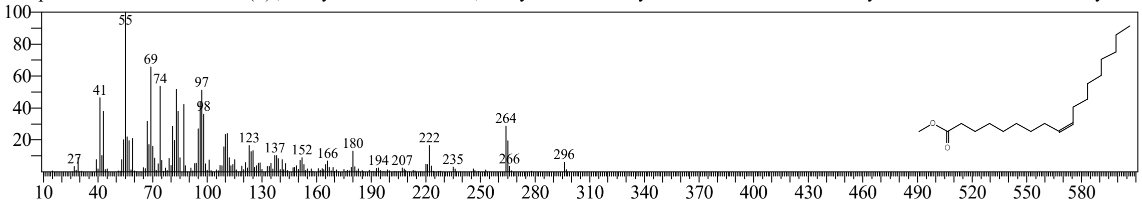

<< Target >>
Line#:9   R.Time:26.367(Scan#:1965) MassPeaks:372
RawMode:Averaged 26.358-26.375(1964-1966) BasePeak:55.05(14864)
BG Mode:Calc. from Peak Group 1 - Event 1

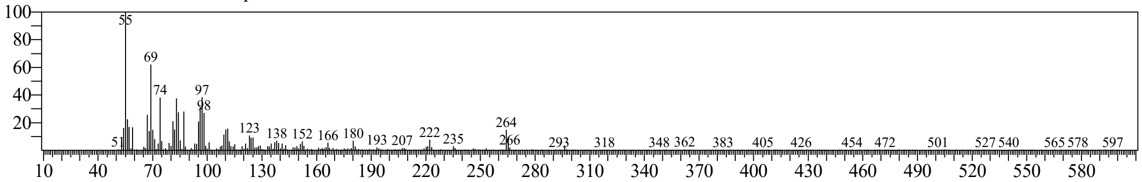

Hit#:1 Entry:139647 Library:NIST17-1.lib
SI:95 Formula:C19H36O2 CAS:0-00-0 MolWeight:296 RetIndex:2085
CompName:cis-13-Octadecenoic acid, methyl ester

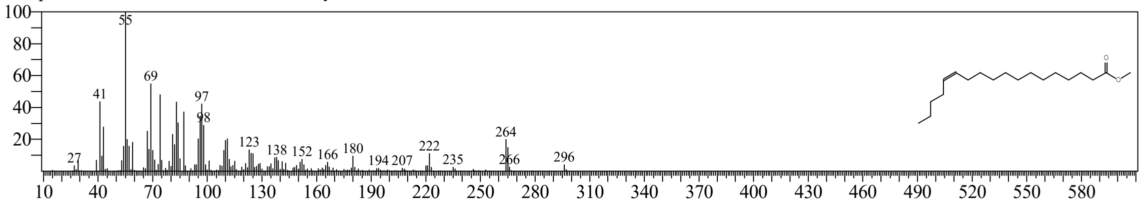

Hit#:2 Entry:127661 Library:NIST14.lib
SI:95 Formula:C19H36O2 CAS:0-00-0 MolWeight:296 RetIndex:2085
CompName:cis-13-Octadecenoic acid, methyl ester

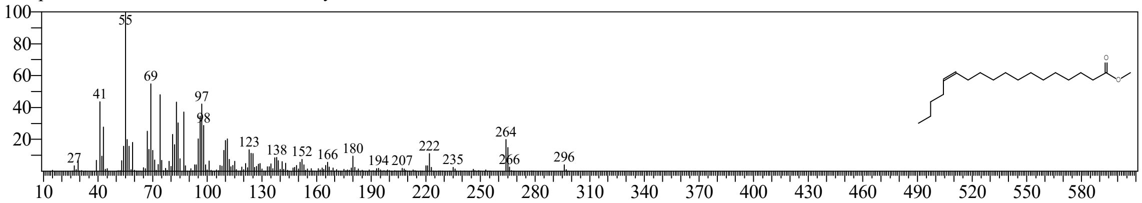

Hit#:3 Entry:139644 Library:NIST17-1.lib
SI:95 Formula:C19H36O2 CAS:0-00-0 MolWeight:296 RetIndex:2085
CompName:trans-13-Octadecenoic acid, methyl ester

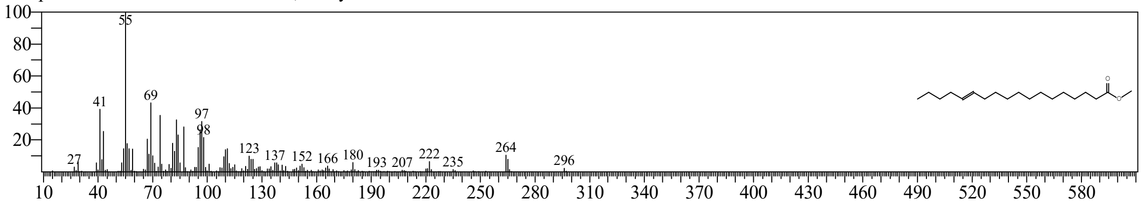

Hit#:4 Entry:127658 Library:NIST14.lib
SI:95 Formula:C19H36O2 CAS:0-00-0 MolWeight:296 RetIndex:2085
CompName:trans-13-Octadecenoic acid, methyl ester

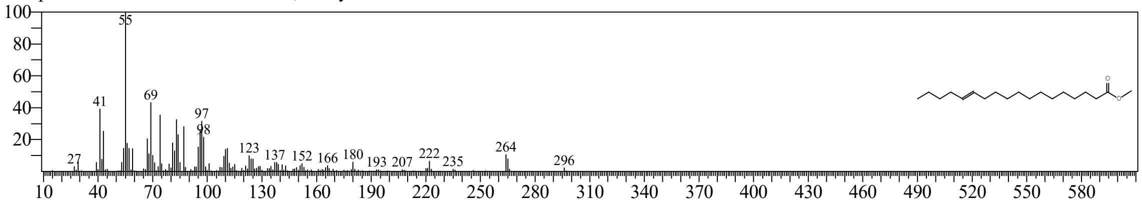

Hit#:5 Entry:32748 Library:NIST17s.lib
SI:95 Formula:C19H36O2 CAS:52380-33-3 MolWeight:296 RetIndex:2085
CompName:11-Octadecenoic acid, methyl ester $$ Methyl 11-octadecenoate $$ Octadec-11-enoic acid, methyl ester $$ Methyl (11E)-11-octadecenoate # $$

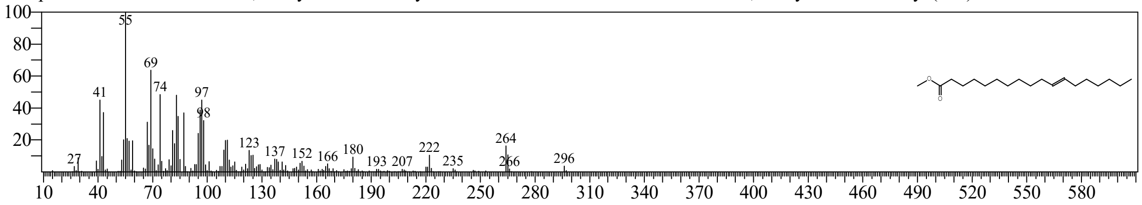

<< Target >>
Line#:10 R.Time:27.708(Scan#:2126) MassPeaks:348
RawMode:Averaged 27.700-27.717(2125-2127) BasePeak:67.05(3134)
BG Mode:Calc. from Peak Group 1 - Event 1

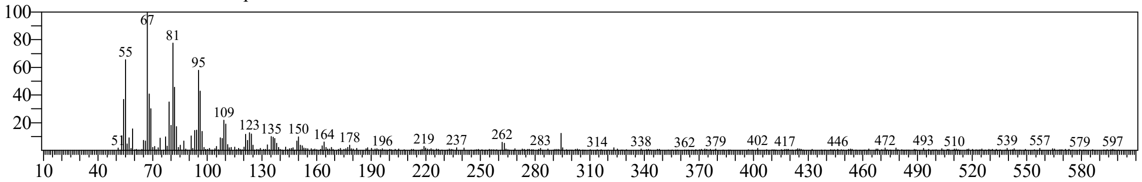

Hit#:1 Entry:32588 Library:NIST17s.lib
SI:96 Formula:C19H34O2 CAS:112-63-0 MolWeight:294 RetIndex:2093
CompName:9,12-Octadecadienoic acid (Z,Z)-, methyl ester $$ Linoleic acid, methyl ester $$ Methyl cis,cis-9,12-octadecadienoate $$ Methyl linoleate $$ M

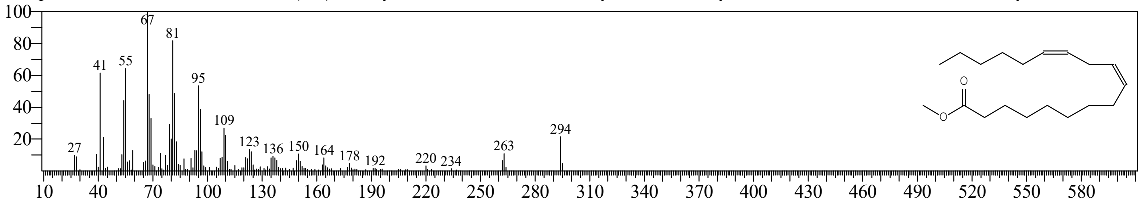

Hit#:2 Entry:27999 Library:NIST14s.lib
SI:96 Formula:C19H34O2 CAS:112-63-0 MolWeight:294 RetIndex:2093
CompName:9,12-Octadecadienoic acid (Z,Z)-, methyl ester $$ Linoleic acid, methyl ester $$ Methyl cis,cis-9,12-octadecadienoate $$ Methyl linoleate $$ M

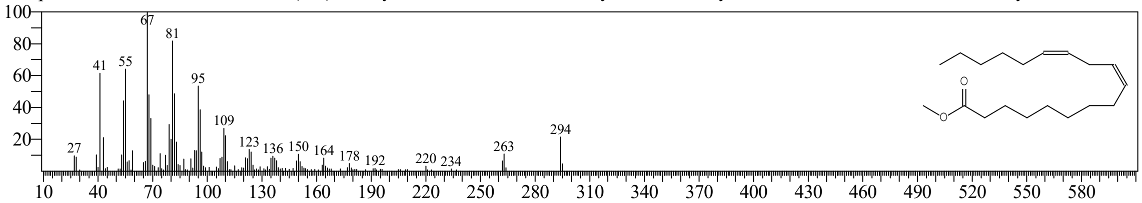

Hit#:3 Entry:32589 Library:NIST17s.lib
SI:96 Formula:C19H34O2 CAS:112-63-0 MolWeight:294 RetIndex:2093
CompName:9,12-Octadecadienoic acid (Z,Z)-, methyl ester $$ Linoleic acid, methyl ester $$ Methyl cis,cis-9,12-octadecadienoate $$ Methyl linoleate $$ M

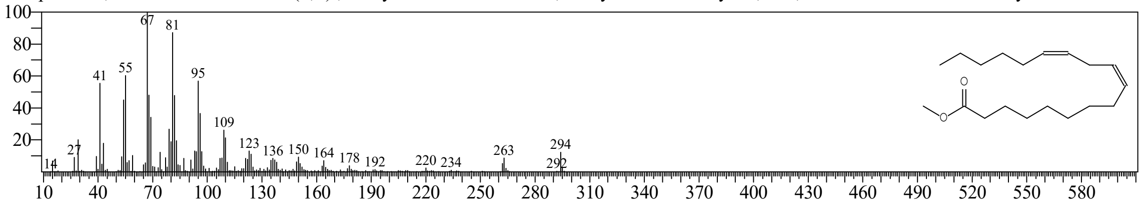

Hit#:4 Entry:28000 Library:NIST14s.lib
SI:96 Formula:C19H34O2 CAS:112-63-0 MolWeight:294 RetIndex:2093
CompName:9,12-Octadecadienoic acid (Z,Z)-, methyl ester $$ Linoleic acid, methyl ester $$ Methyl cis,cis-9,12-octadecadienoate $$ Methyl linoleate $$ M

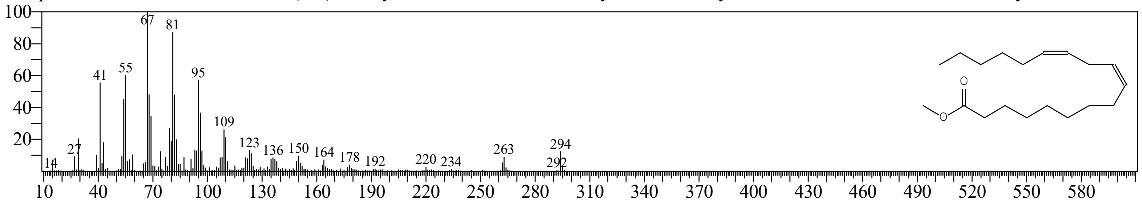

Hit#:5 Entry:137663 Library:NIST17-1.lib
SI:96 Formula:C19H34O2 CAS:2462-85-3 MolWeight:294 RetIndex:2093
CompName:9,12-Octadecadienoic acid, methyl ester $$ 9,12-Octadecenoic acid, methyl ester $$ Methyl octadeca-9,12-dienoate $$ Methyl 9,12-octadecadi

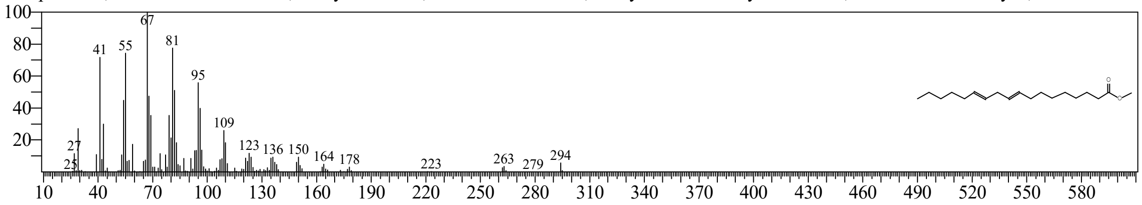

<< Target >>
Line#:11 R.Time:27.942(Scan#:2154) MassPeaks:296
RawMode:Averaged 27.933-27.950(2153-2155) BasePeak:67.05(2129)
BG Mode:Calc. from Peak Group 1 - Event 1

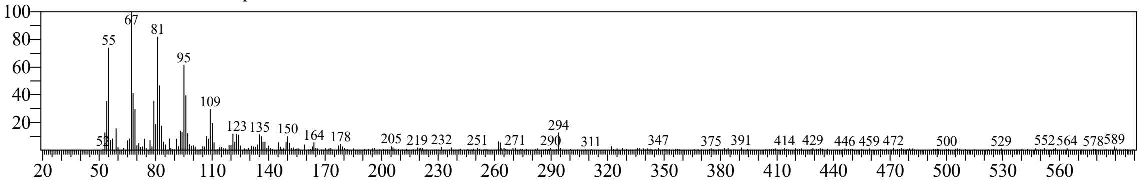

Hit#:1 Entry:137663 Library:NIST17-1.lib
SI:96 Formula:C19H34O2 CAS:2462-85-3 MolWeight:294 RetIndex:2093
CompName:9,12-Octadecadienoic acid, methyl ester $$ 9,12-Octadecenoic acid, methyl ester $$ Methyl octadeca-9,12-dienoate $$ Methyl 9,12-octadecadi

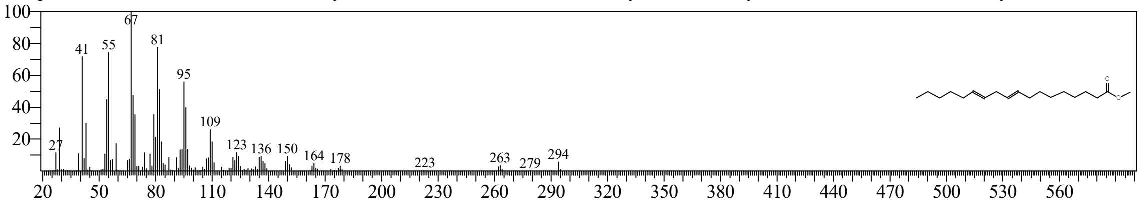

Hit#:2 Entry:125931 Library:NIST14.lib
SI:96 Formula:C19H34O2 CAS:2462-85-3 MolWeight:294 RetIndex:2093
CompName:9,12-Octadecadienoic acid, methyl ester $$ 9,12-Octadecenoic acid, methyl ester $$ Methyl octadeca-9,12-dienoate $$ Methyl 9,12-octadecadi

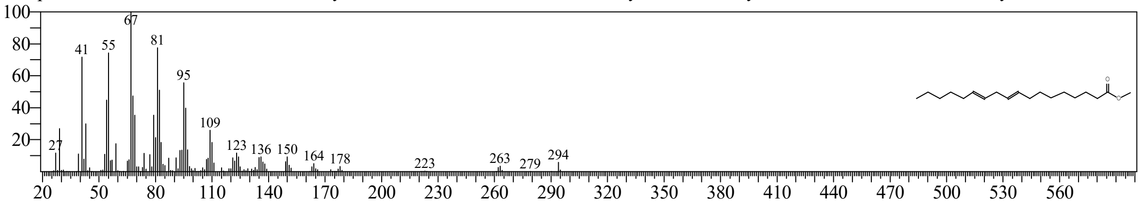

Hit#:3 Entry:27999 Library:NIST14s.lib
SI:96 Formula:C19H34O2 CAS:112-63-0 MolWeight:294 RetIndex:2093
CompName:9,12-Octadecadienoic acid (Z,Z)-, methyl ester $$ Linoleic acid, methyl ester $$ Methyl cis,cis-9,12-octadecadienoate $$ Methyl linoleate $$ M

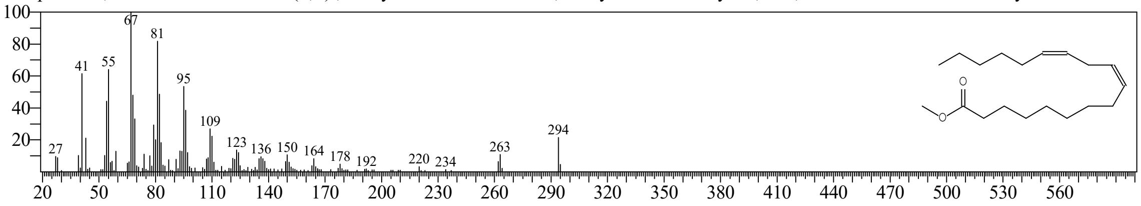

Hit#:4 Entry:32588 Library:NIST17s.lib
SI:96 Formula:C19H34O2 CAS:112-63-0 MolWeight:294 RetIndex:2093
CompName:9,12-Octadecadienoic acid (Z,Z)-, methyl ester $$ Linoleic acid, methyl ester $$ Methyl cis,cis-9,12-octadecadienoate $$ Methyl linoleate $$ M

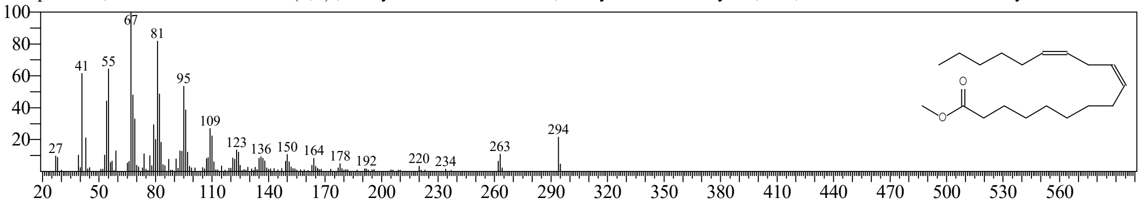

Hit#:5 Entry:28001 Library:NIST14s.lib
SI:95 Formula:C19H34O2 CAS:112-63-0 MolWeight:294 RetIndex:2093
CompName:9,12-Octadecadienoic acid (Z,Z)-, methyl ester $$ Linoleic acid, methyl ester $$ Methyl cis,cis-9,12-octadecadienoate $$ Methyl linoleate $$ M

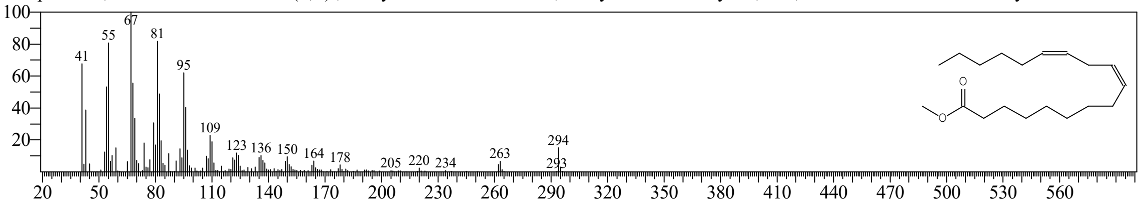

<< Target >>
Line#:12 R.Time:28.350(Scan#:2203) MassPeaks:336
RawMode:Averaged 28.342-28.358(2202-2204) BasePeak:67.05(434619)
BG Mode:Calc. from Peak Group 1 - Event 1

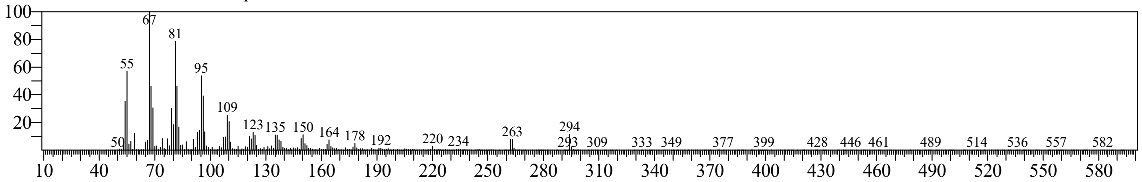

Hit#:1 Entry:32588 Library:NIST17s.lib
SI:97 Formula:C19H34O2 CAS:112-63-0 MolWeight:294 RetIndex:2093
CompName:9,12-Octadecadienoic acid (Z,Z)-, methyl ester $$ Linoleic acid, methyl ester $$ Methyl cis,cis-9,12-octadecadienoate $$ Methyl linoleate $$ M

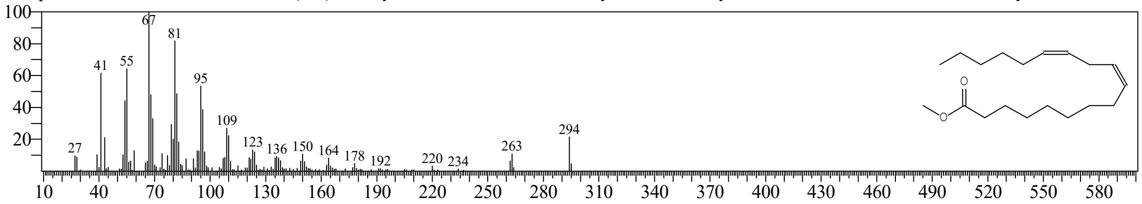

Hit#:2 Entry:27999 Library:NIST14s.lib
SI:97 Formula:C19H34O2 CAS:112-63-0 MolWeight:294 RetIndex:2093
CompName:9,12-Octadecadienoic acid (Z,Z)-, methyl ester $$ Linoleic acid, methyl ester $$ Methyl cis,cis-9,12-octadecadienoate $$ Methyl linoleate $$ M

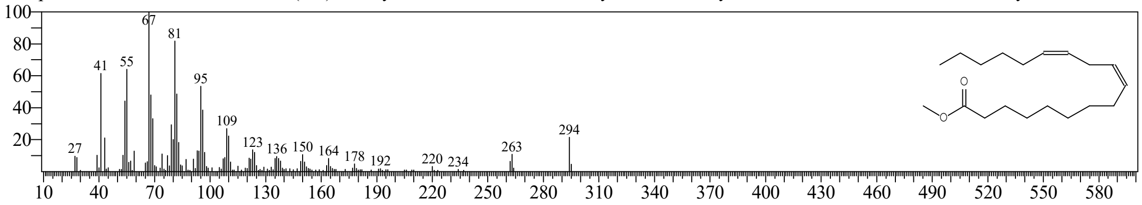

Hit#:3 Entry:32589 Library:NIST17s.lib
SI:96 Formula:C19H34O2 CAS:112-63-0 MolWeight:294 RetIndex:2093
CompName:9,12-Octadecadienoic acid (Z,Z)-, methyl ester $$ Linoleic acid, methyl ester $$ Methyl cis,cis-9,12-octadecadienoate $$ Methyl linoleate $$ M

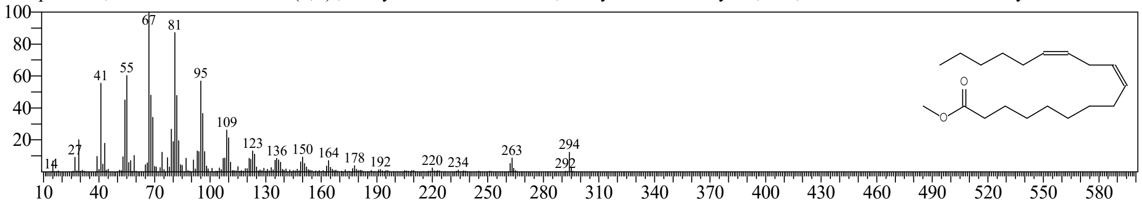

Hit#:4 Entry:28000 Library:NIST14s.lib
SI:96 Formula:C19H34O2 CAS:112-63-0 MolWeight:294 RetIndex:2093
CompName:9,12-Octadecadienoic acid (Z,Z)-, methyl ester $$ Linoleic acid, methyl ester $$ Methyl cis,cis-9,12-octadecadienoate $$ Methyl linoleate $$ M

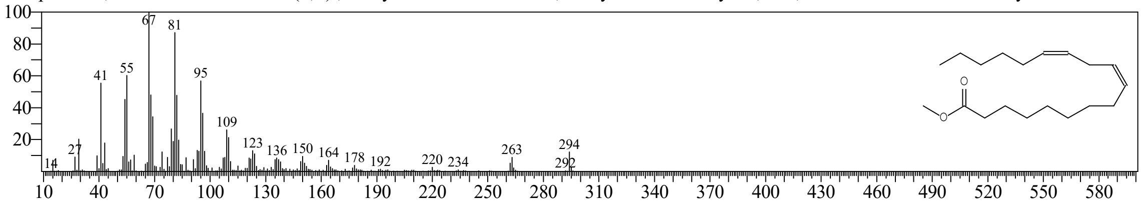

Hit#:5 Entry:125933 Library:NIST14.lib
SI:95 Formula:C19H34O2 CAS:0-00-0 MolWeight:294 RetIndex:2093
CompName:Methyl 10-trans,12-cis-octadecadienoate

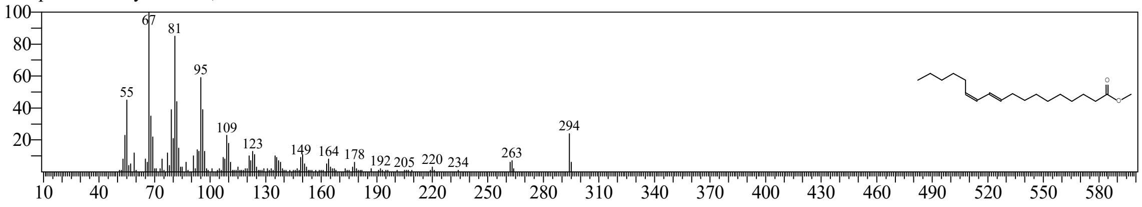

<< Target >>
Line#:13   R.Time:29.258(Scan#:2312) MassPeaks:296
RawMode:Averaged 29.250-29.267(2311-2313) BasePeak:74.00(9090)
BG Mode:Calc. from Peak Group 1 - Event 1

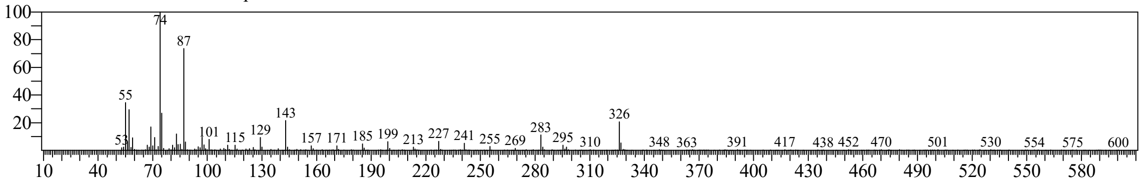

Hit#:1 Entry:170238 Library:NIST17-1.lib
SI:97 Formula:C21H42O2 CAS:0-00-0 MolWeight:326 RetIndex:2212
CompName:Methyl 18-methylnonadecanoate

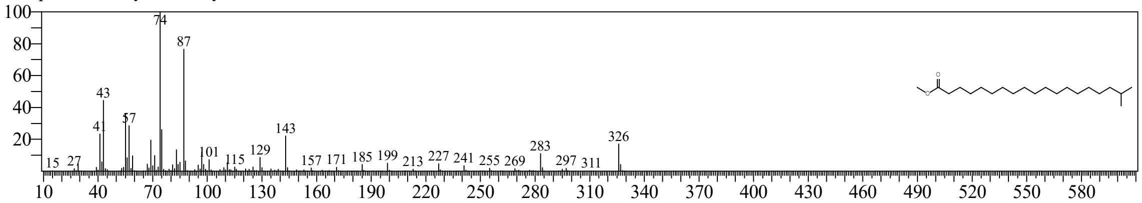

Hit#:2 Entry:154704 Library:NIST14.lib
SI:97 Formula:C21H42O2 CAS:0-00-0 MolWeight:326 RetIndex:2212
CompName:Methyl 18-methylnonadecanoate

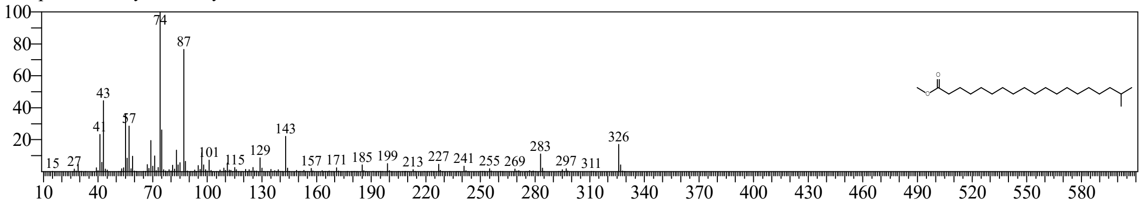

Hit#:3 Entry:34726 Library:NIST17s.lib
SI:96 Formula:C21H42O2 CAS:1120-28-1 MolWeight:326 RetIndex:2276
CompName:Eicosanoic acid, methyl ester $$ Methyl arachisate $$ Methyl eicosanoate $$ Arachidic acid methyl ester $$ Kemester 2050 $$ Methyl aracidat

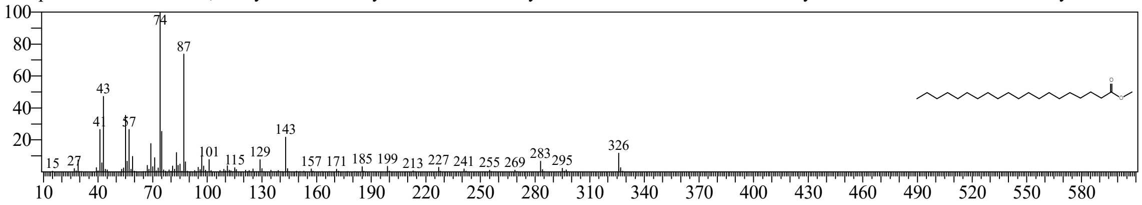

Hit#:4 Entry:34724 Library:NIST17s.lib
SI:93 Formula:C21H42O2 CAS:1120-28-1 MolWeight:326 RetIndex:2276
CompName:Eicosanoic acid, methyl ester $$ Methyl arachisate $$ Methyl eicosanoate $$ Arachidic acid methyl ester $$ Kemester 2050 $$ Methyl aracidat

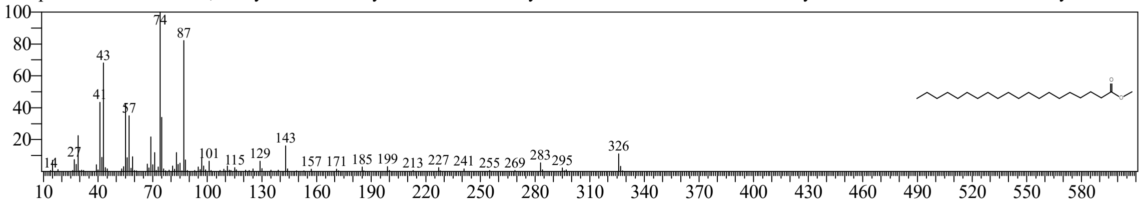

Hit#:5 Entry:29931 Library:NIST14s.lib
SI:93 Formula:C21H42O2 CAS:1120-28-1 MolWeight:326 RetIndex:2276
CompName:Eicosanoic acid, methyl ester $$ Methyl arachisate $$ Methyl eicosanoate $$ Arachidic acid methyl ester $$ Kemester 2050 $$ Methyl aracidat

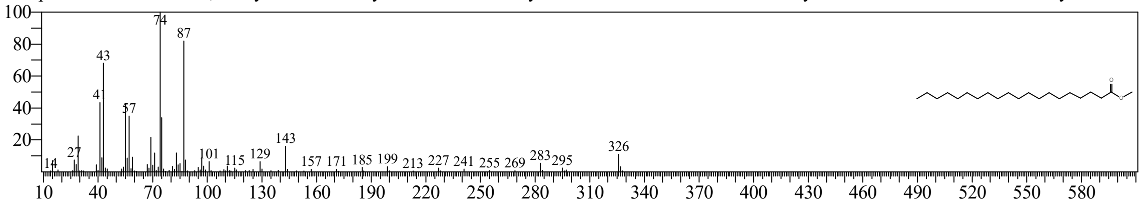

<< Target >>
Line#:14 R.Time:29.817(Scan#:2379) MassPeaks:353
RawMode:Averaged 29.808-29.825(2378-2380) BasePeak:79.05(7868)
BG Mode:Calc. from Peak Group 1 - Event 1

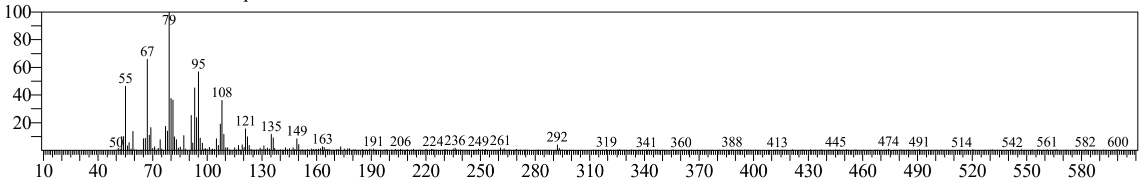

Hit#:1 Entry:32438 Library:NIST17s.lib
SI:95 Formula:C19H32O2 CAS:301-00-8 MolWeight:292 RetIndex:2101
CompName:9,12,15-Octadecatrienoic acid, methyl ester, (Z,Z,Z)- $$ Linolenic acid, methyl ester $$ Methyl all-cis-9,12,15-octadecatrienoate $$ Methyl lino

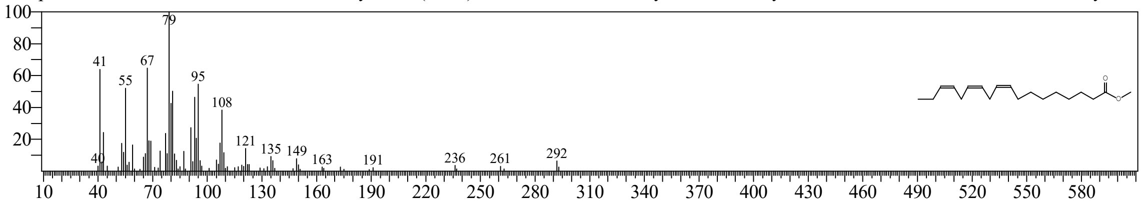

Hit#:2 Entry:29609 Library:NIST14s.lib
SI:95 Formula:C21H36O2 CAS:55682-88-7 MolWeight:320 RetIndex:2300
CompName:11,14,17-Eicosatrienoic acid, methyl ester $$ Methyl 11,14,17-icosatrienoate $$ Methyl 11,14,17-eicosatrienoate $$

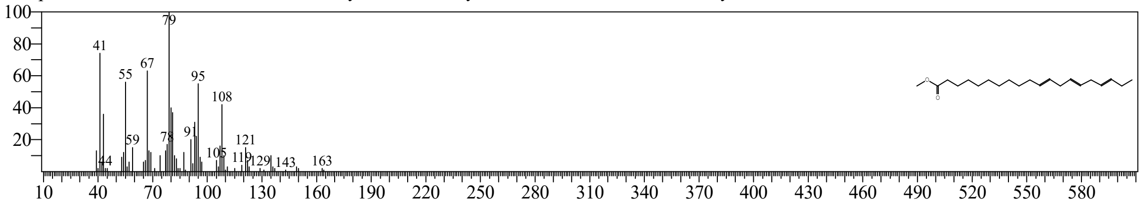

Hit#:3 Entry:34362 Library:NIST17s.lib
SI:95 Formula:C21H36O2 CAS:55682-88-7 MolWeight:320 RetIndex:2300
CompName:11,14,17-Eicosatrienoic acid, methyl ester $$ Methyl 11,14,17-icosatrienoate $$ Methyl 11,14,17-eicosatrienoate $$

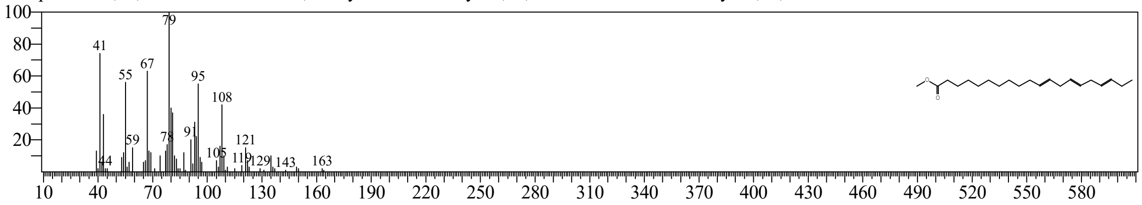

Hit#:4 Entry:124228 Library:NIST14.lib
SI:95 Formula:C19H32O2 CAS:301-00-8 MolWeight:292 RetIndex:2101
CompName:9,12,15-Octadecatrienoic acid, methyl ester, (Z,Z,Z)- $$ Linolenic acid, methyl ester $$ Methyl all-cis-9,12,15-octadecatrienoate $$ Methyl lino

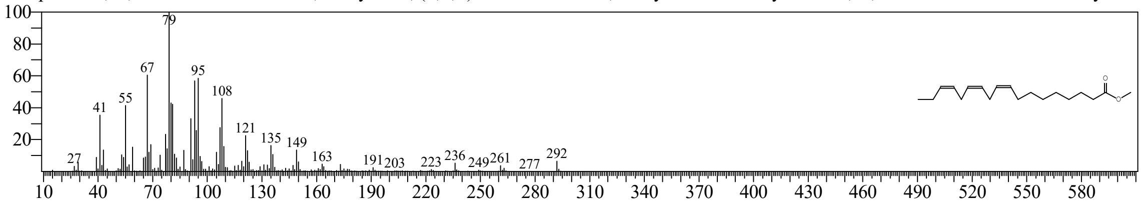

Hit#:5 Entry:135724 Library:NIST17-1.lib
SI:95 Formula:C19H32O2 CAS:301-00-8 MolWeight:292 RetIndex:2101
CompName:9,12,15-Octadecatrienoic acid, methyl ester, (Z,Z,Z)- $$ Linolenic acid, methyl ester $$ Methyl all-cis-9,12,15-octadecatrienoate $$ Methyl lino

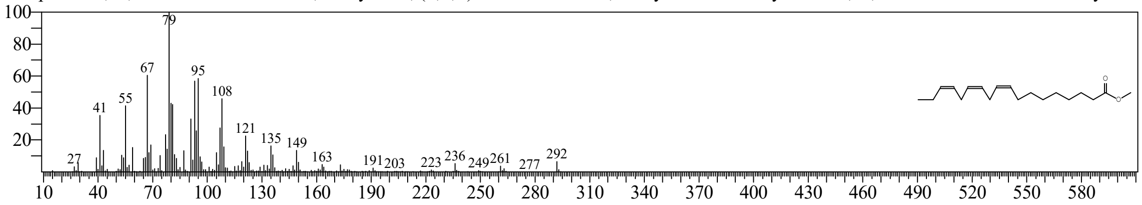

<< Target >>
Line#:15 R.Time:30.300(Scan#:2437) MassPeaks:378
RawMode:Averaged 30.292-30.308(2436-2438) BasePeak:79.05(5408)
BG Mode:Calc. from Peak Group 1 - Event 1

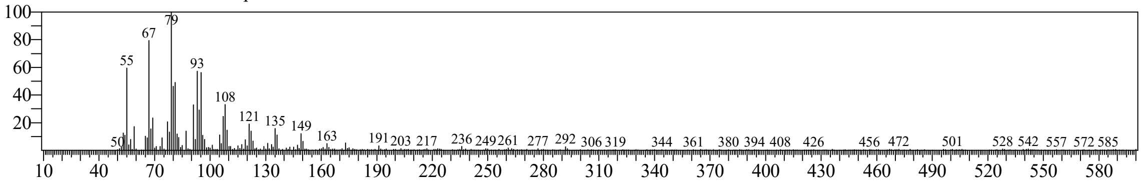

Hit#:1 Entry:124228 Library:NIST14.lib
SI:95 Formula:C19H32O2 CAS:301-00-8 MolWeight:292 RetIndex:2101
CompName:9,12,15-Octadecatrienoic acid, methyl ester, (Z,Z,Z)- $$ Linolenic acid, methyl ester $$ Methyl all-cis-9,12,15-octadecatrienoate $$ Methyl lino

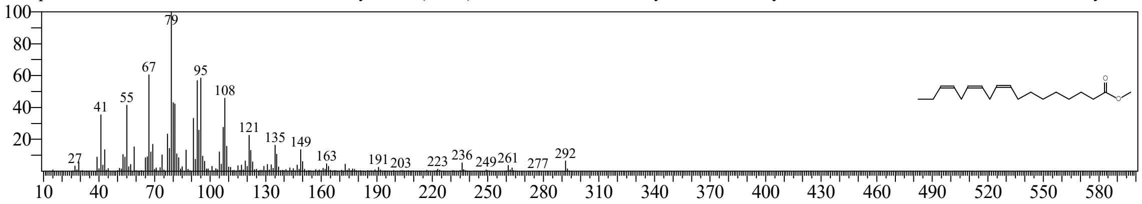

Hit#:2 Entry:135724 Library:NIST17-1.lib
SI:95 Formula:C19H32O2 CAS:301-00-8 MolWeight:292 RetIndex:2101
CompName:9,12,15-Octadecatrienoic acid, methyl ester, (Z,Z,Z)- $$ Linolenic acid, methyl ester $$ Methyl all-cis-9,12,15-octadecatrienoate $$ Methyl lino

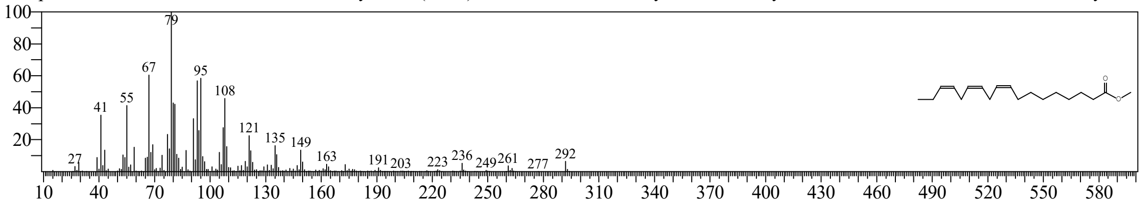

Hit#:3 Entry:121536 Library:NIST17-1.lib
SI:94 Formula:C18H30O2 CAS:0-00-0 MolWeight:278 RetIndex:2002
CompName:Methyl 8,11,14-heptadecatrienoate

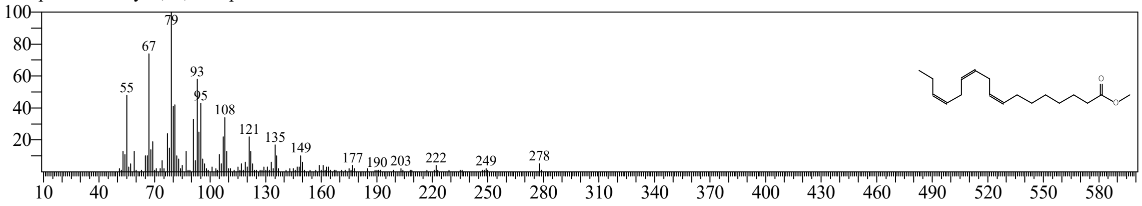

Hit#:4 Entry:111654 Library:NIST14.lib
SI:94 Formula:C18H30O2 CAS:0-00-0 MolWeight:278 RetIndex:2002
CompName:Methyl 8,11,14-heptadecatrienoate

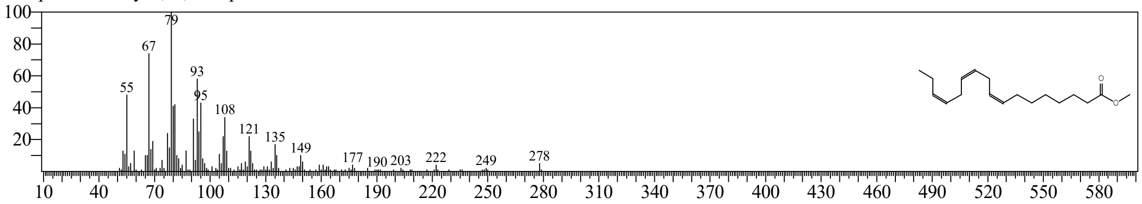

Hit#:5 Entry:27872 Library:NIST14s.lib
SI:94 Formula:C19H32O2 CAS:301-00-8 MolWeight:292 RetIndex:2101
CompName:9,12,15-Octadecatrienoic acid, methyl ester, (Z,Z,Z)- $$ Linolenic acid, methyl ester $$ Methyl all-cis-9,12,15-octadecatrienoate $$ Methyl lino

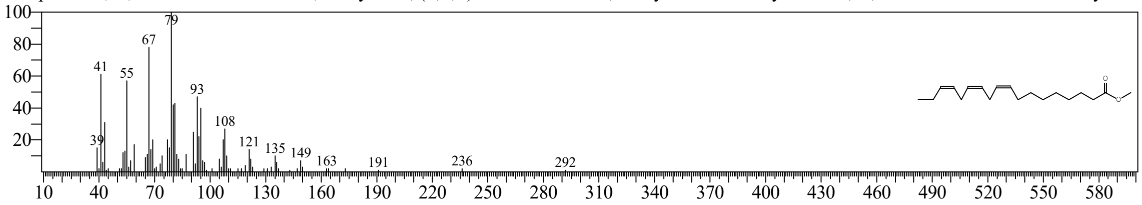

<< Target >>
Line#:16   R.Time:30.600(Scan#:2473) MassPeaks:349
RawMode:Averaged 30.592-30.608(2472-2474) BasePeak:79.05(75482)
BG Mode:Calc. from Peak Group 1 - Event 1

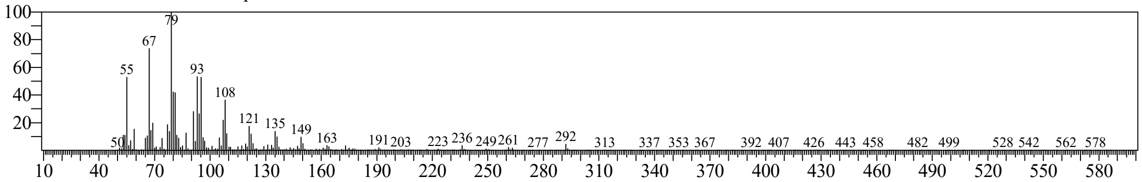

Hit#:1 Entry:124228 Library:NIST14.lib
SI:96 Formula:C19H32O2 CAS:301-00-8 MolWeight:292 RetIndex:2101
CompName:9,12,15-Octadecatrienoic acid, methyl ester, (Z,Z,Z)- $$ Linolenic acid, methyl ester $$ Methyl all-cis-9,12,15-octadecatrienoate $$ Methyl lino

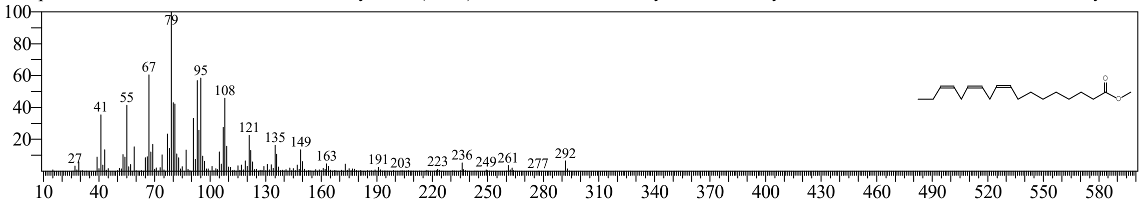

Hit#:2 Entry:135724 Library:NIST17-1.lib
SI:96 Formula:C19H32O2 CAS:301-00-8 MolWeight:292 RetIndex:2101
CompName:9,12,15-Octadecatrienoic acid, methyl ester, (Z,Z,Z)- $$ Linolenic acid, methyl ester $$ Methyl all-cis-9,12,15-octadecatrienoate $$ Methyl lino

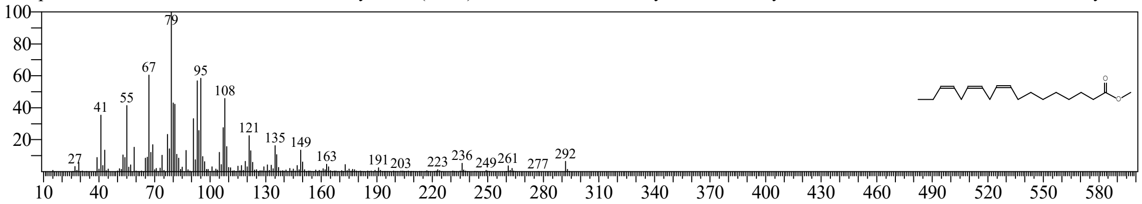

Hit#:3 Entry:32438 Library:NIST17s.lib
SI:95 Formula:C19H32O2 CAS:301-00-8 MolWeight:292 RetIndex:2101
CompName:9,12,15-Octadecatrienoic acid, methyl ester, (Z,Z,Z)- $$ Linolenic acid, methyl ester $$ Methyl all-cis-9,12,15-octadecatrienoate $$ Methyl lino

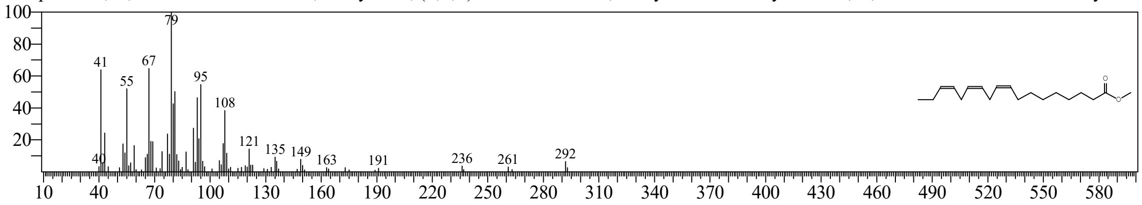

Hit#:4 Entry:111654 Library:NIST14.lib
SI:95 Formula:C18H30O2 CAS:0-00-0 MolWeight:278 RetIndex:2002
CompName:Methyl 8,11,14-heptadecatrienoate

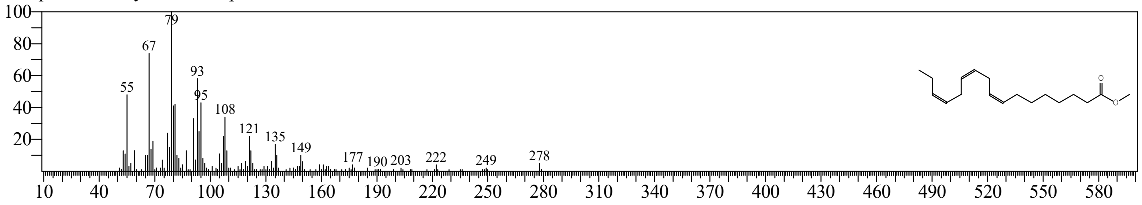

Hit#:5 Entry:121536 Library:NIST17-1.lib
SI:95 Formula:C18H30O2 CAS:0-00-0 MolWeight:278 RetIndex:2002
CompName:Methyl 8,11,14-heptadecatrienoate

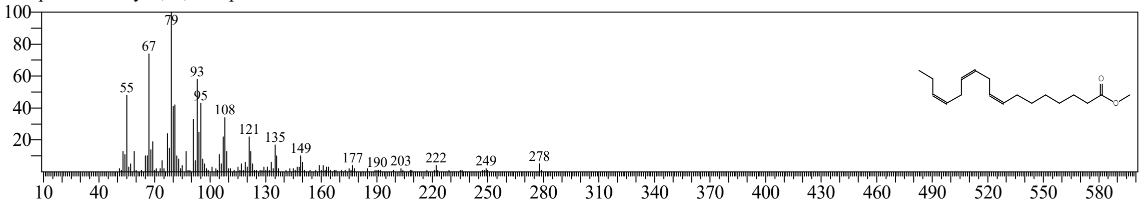

<< Target >>
Line#:17 R.Time:31.925(Scan#:2632) MassPeaks:289
RawMode:Averaged 31.917-31.933(2631-2633) BasePeak:67.05(706)
BG Mode:Calc. from Peak Group 1 - Event 1

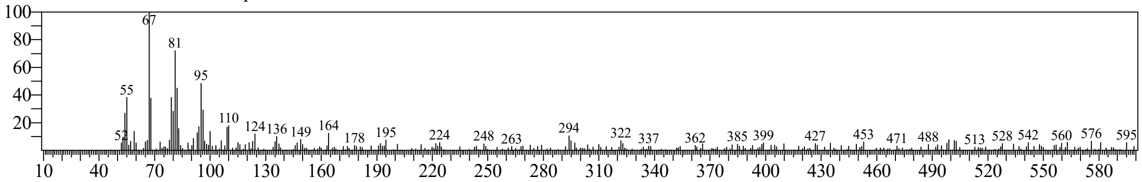

Hit#:1 Entry:32587 Library:NIST17s.lib
SI:82 Formula:C19H34O2 CAS:13038-47-6 MolWeight:294 RetIndex:2093
CompName:9,11-Octadecadienoic acid, methyl ester, (E,E)- $$ Methyl trans-9,trans-11-octadecadienoate $$ Methyl trans,trans-9,11-octadecadienoate $$ M

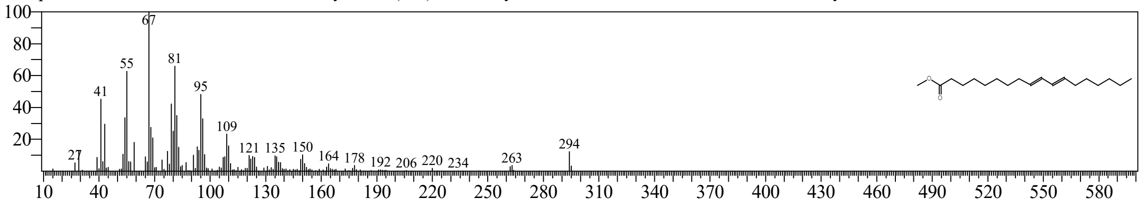

Hit#:2 Entry:137666 Library:NIST17-1.lib
SI:81 Formula:C19H34O2 CAS:13058-52-1 MolWeight:294 RetIndex:2093
CompName:Methyl 9-cis,11-trans-octadecadienoate

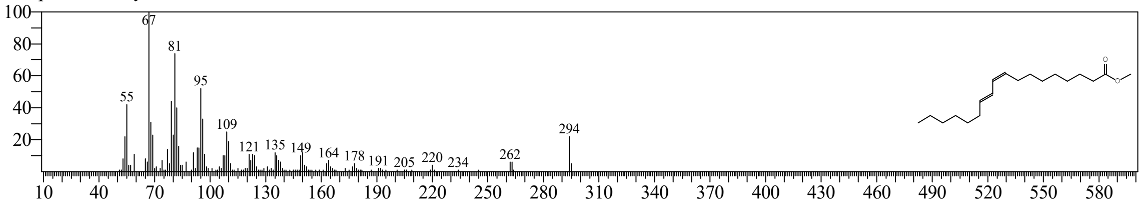

Hit#:3 Entry:125933 Library:NIST14.lib
SI:81 Formula:C19H34O2 CAS:0-00-0 MolWeight:294 RetIndex:2093
CompName:Methyl 10-trans,12-cis-octadecadienoate

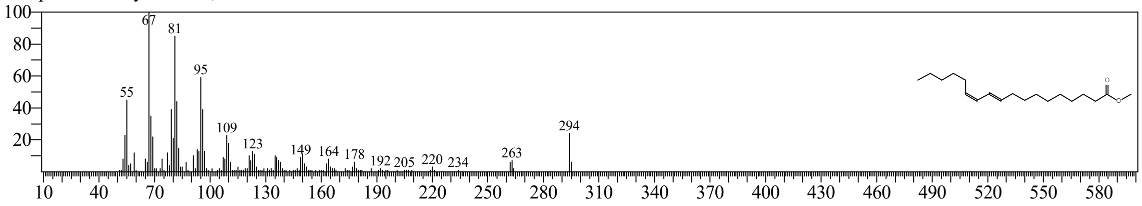

Hit#:4 Entry:125932 Library:NIST14.lib
SI:81 Formula:C19H34O2 CAS:0-00-0 MolWeight:294 RetIndex:2093
CompName:Methyl 9-cis,11-trans-octadecadienoate

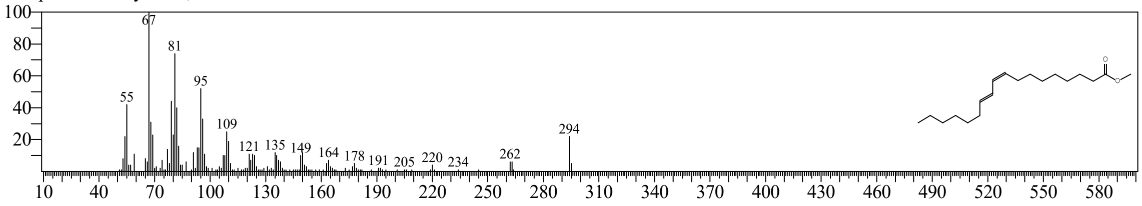

Hit#:5 Entry:137664 Library:NIST17-1.lib
SI:81 Formula:C19H34O2 CAS:0-00-0 MolWeight:294 RetIndex:2093
CompName:Methyl 10-trans,12-cis-octadecadienoate

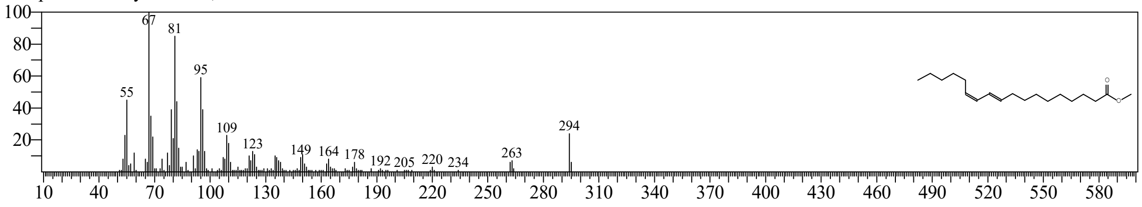

<< Target >>
Line#:18 R.Time:33.492(Scan#:2820) MassPeaks:343
RawMode:Averaged 33.483-33.500(2819-2821) BasePeak:74.00(9408)
BG Mode:Calc. from Peak Group 1 - Event 1

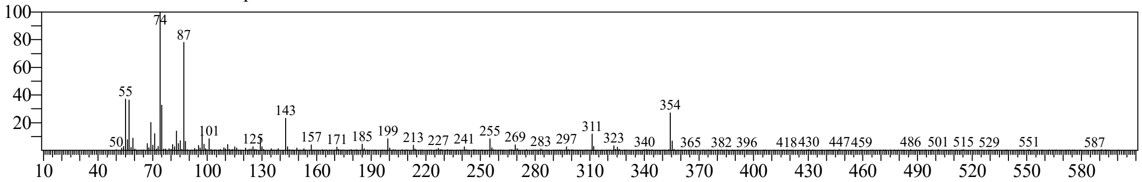

Hit#:1 Entry:196443 Library:NIST17-1.lib
SI:96 Formula:C23H46O2 CAS:929-77-1 MolWeight:354 RetIndex:2475
CompName:Docosanoic acid, methyl ester $$ Behenic acid, methyl ester $$ Methyl behenate $$ Methyl docosanoate $$ n-Docosanoic acid methyl ester $$

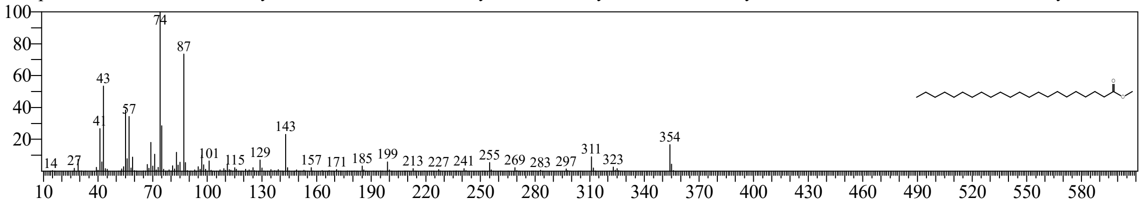

Hit#:2 Entry:178051 Library:NIST14.lib
SI:96 Formula:C23H46O2 CAS:929-77-1 MolWeight:354 RetIndex:2475
CompName:Docosanoic acid, methyl ester $$ Behenic acid, methyl ester $$ Methyl behenate $$ Methyl docosanoate $$ n-Docosanoic acid methyl ester $$

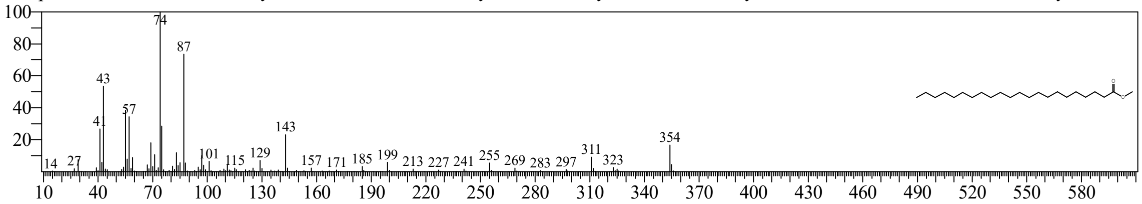

Hit#:3 Entry:196444 Library:NIST17-1.lib
SI:95 Formula:C23H46O2 CAS:0-00-0 MolWeight:354 RetIndex:2411
CompName:Methyl 20-methyl-heneicosanoate

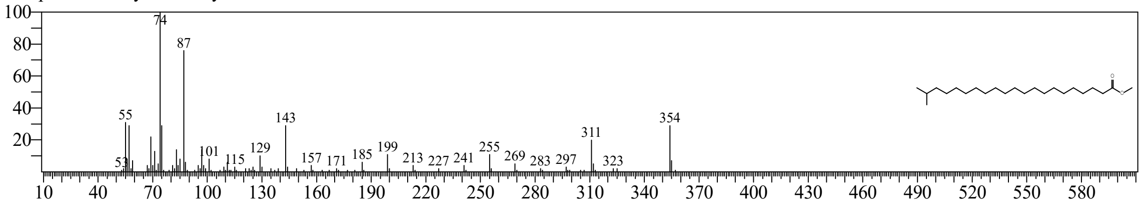

Hit#:4 Entry:178052 Library:NIST14.lib
SI:95 Formula:C23H46O2 CAS:0-00-0 MolWeight:354 RetIndex:2411
CompName:Methyl 20-methyl-heneicosanoate

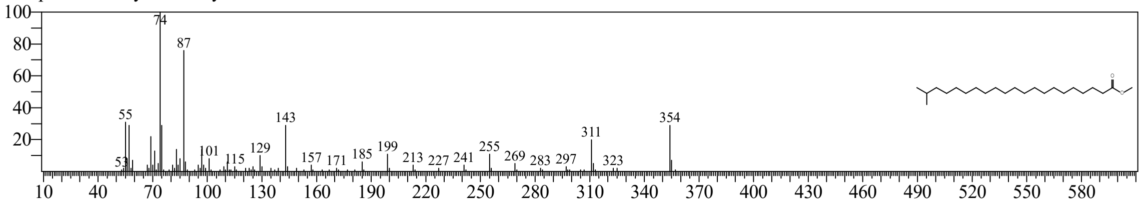

Hit#:5 Entry:31088 Library:NIST14s.lib
SI:95 Formula:C23H46O2 CAS:929-77-1 MolWeight:354 RetIndex:2475
CompName:Docosanoic acid, methyl ester $$ Behenic acid, methyl ester $$ Methyl behenate $$ Methyl docosanoate $$ n-Docosanoic acid methyl ester $$

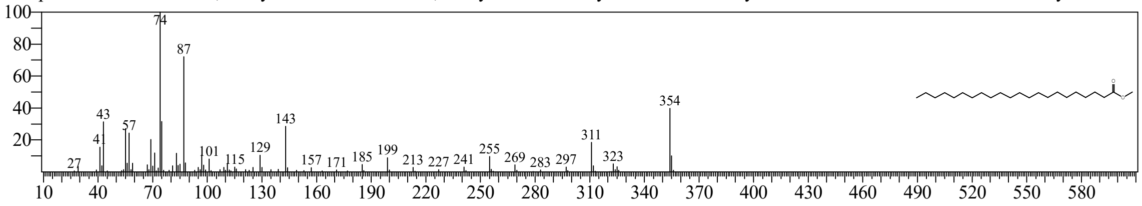

<< Target >>
Line#:19 R.Time:37.800(Scan#:3337) MassPeaks:293
RawMode:Averaged 37.792-37.808(3336-3338) BasePeak:74.05(2004)
BG Mode:Calc. from Peak Group 1 - Event 1

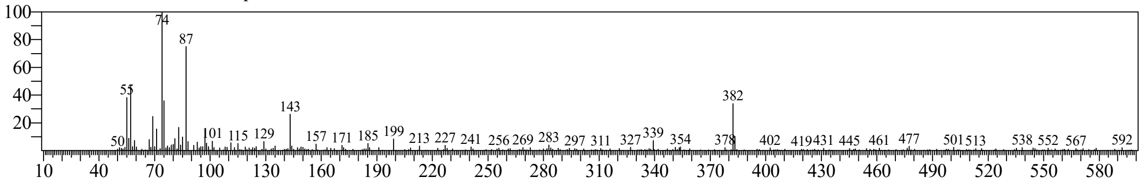

Hit#:1 Entry:37106 Library:NIST17s.lib
SI:91 Formula:C25H50O2 CAS:2442-49-1 MolWeight:382 RetIndex:2674
CompName:Tetracosanoic acid, methyl ester $$ Methyl lignocerate $$ Methyl tetracosanoate $$ Lignoceric acid methyl ester $$

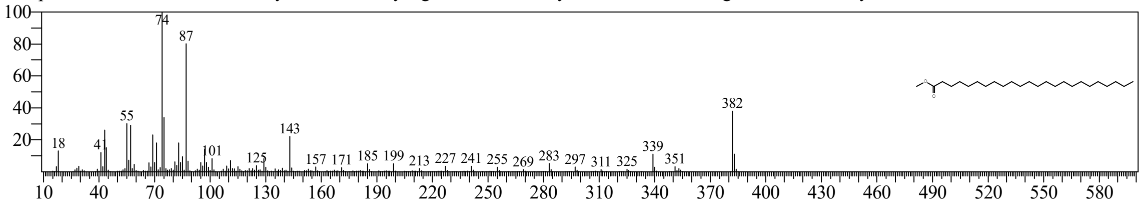

Hit#:2 Entry:31970 Library:NIST14s.lib
SI:91 Formula:C25H50O2 CAS:2442-49-1 MolWeight:382 RetIndex:2674
CompName:Tetracosanoic acid, methyl ester $$ Methyl lignocerate $$ Methyl tetracosanoate $$ Lignoceric acid methyl ester $$

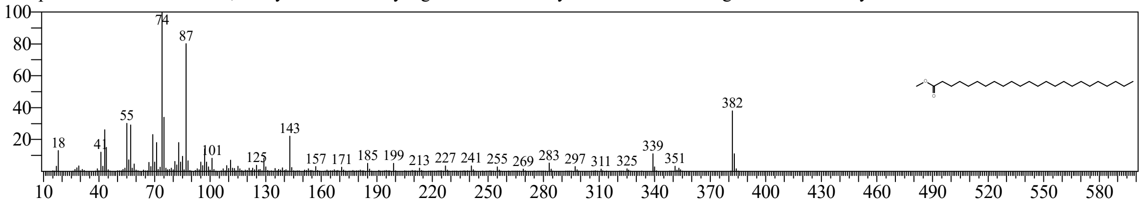

Hit#:3 Entry:37107 Library:NIST17s.lib
SI:88 Formula:C25H50O2 CAS:2442-49-1 MolWeight:382 RetIndex:2674
CompName:Tetracosanoic acid, methyl ester $$ Methyl lignocerate $$ Methyl tetracosanoate $$ Lignoceric acid methyl ester $$

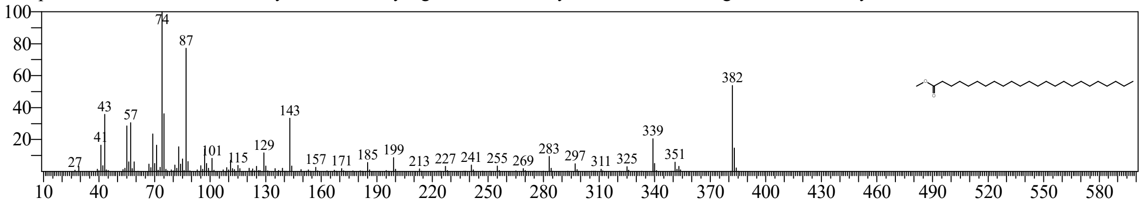

Hit#:4 Entry:31971 Library:NIST14s.lib
SI:88 Formula:C25H50O2 CAS:2442-49-1 MolWeight:382 RetIndex:2674
CompName:Tetracosanoic acid, methyl ester $$ Methyl lignocerate $$ Methyl tetracosanoate $$ Lignoceric acid methyl ester $$

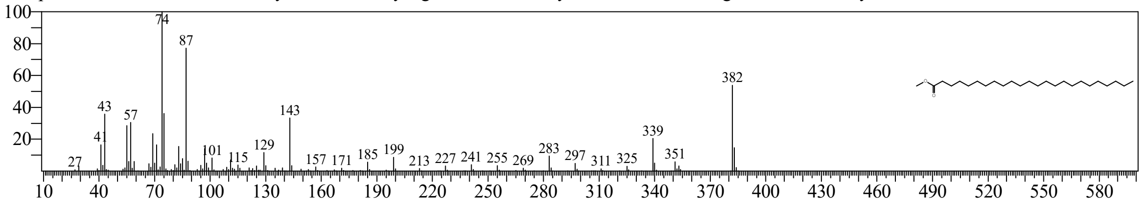

Hit#:5 Entry:31969 Library:NIST14s.lib
SI:88 Formula:C25H50O2 CAS:2442-49-1 MolWeight:382 RetIndex:2674
CompName:Tetracosanoic acid, methyl ester $$ Methyl lignocerate $$ Methyl tetracosanoate $$ Lignoceric acid methyl ester $$

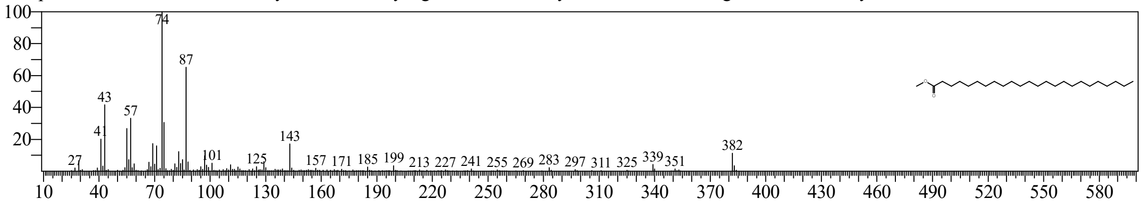

Supplement: Supplementary file 1 [file molecules-31-02891-s001.zip › 14d-PG-3.pdf]
