# Supplementary material for: Selective Inhibition of Lipid Oxidation by a Quercetagetin-Rich Extract from Tagetes erecta: A Chemometric Approach Using OPLS-DA and Selectivity Index Analysis
Source: Molecules. 2026 Aug 19;31(16):2891. doi: 10.3390/molecules31162891 (PMC13515886; doi:10.3390/molecules31162891)

QG100-1-14d

<<Column>>
Name : HP-88
Thickness : 0.20um
Length : 100.0m
Inside Diameter : 0.25mm

Method

===== Analytical Line 1 =====

Column Oven Temp.	:120.0 °C	
Injection Temp.	:250.00 °C	
Injection Mode	:Split	
Column Flow	:1.04 mL/min	
Split Ratio	:20.0	
Oven Temp. Program		
Rate	Temperature(°C)	Hold Time(min)
-	120.0	1.00
10.00	175.0	10.00
5.00	210.0	5.00
5.00	230.0	15.00

[GCMS-QP2010]
IonSourceTemp :200.00 °C
Interface Temp. :220.00 °C
m/z scanning range：45～500m/z

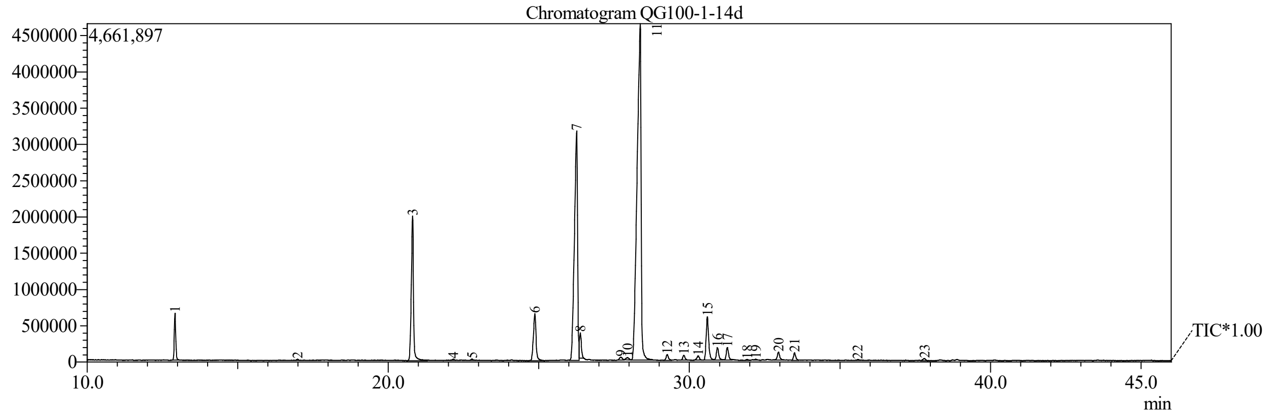

Peak Report TIC

Peak#	R.Time	Area	Area%	Height	Height%	A/H	Name
1	12.921	2206944	2.40	646948	4.98	3.41	Undecanoic acid, methyl ester
2	16.991	59926	0.07	16222	0.12	3.69	Methyl tetradecanoate
3	20.809	10099086	10.98	1994462	15.36	5.06	Hexadecanoic acid, methyl ester
4	22.162	64721	0.07	14513	0.11	4.46	9-Hexadecenoic acid, methyl ester, (Z)-
5	22.784	88746	0.10	17246	0.13	5.15	Heptadecanoic acid, methyl ester
6	24.871	3956998	4.30	648753	5.00	6.10	Methyl stearate
7	26.256	22707315	24.69	3161886	24.35	7.18	9-Octadecenoic acid, methyl ester, (E)-
8	26.377	1543748	1.68	348065	2.68	4.44	cis-13-Octadecenoic acid, methyl ester
9	27.726	287896	0.31	43300	0.33	6.65	9,12-Octadecadienoic acid (Z,Z)-, methyl ester
10	27.943	229649	0.25	32909	0.25	6.98	9,12-Octadecadienoic acid (Z,Z)-, methyl ester
11	28.366	42777629	46.51	4633061	35.69	9.23	9,12-Octadecadienoic acid (Z,Z)-, methyl ester
12	29.264	442173	0.48	80887	0.62	5.47	Methyl 18-methylnonadecanoate
13	29.817	335098	0.36	67437	0.52	4.97	9,12,15-Octadecatrienoic acid, methyl ester, (Z
14	30.291	360575	0.39	60789	0.47	5.93	9,12,15-Octadecatrienoic acid, methyl ester, (Z

Peak#	R.Time	Area	Area%	Height	Height%	A/H	Name
15	30.596	3480305	3.78	597015	4.60	5.83	9,12,15-Octadecatrienoic acid, methyl ester, (Z
16	30.929	920593	1.00	168630	1.30	5.46	Methyl 9-cis,11-trans-octadecadienoate
17	31.261	925850	1.01	170937	1.32	5.42	Methyl 10-trans,12-cis-octadecadienoate
18	31.918	74631	0.08	14538	0.11	5.13	9,11-Octadecadienoic acid, methyl ester, (E,E)
19	32.203	55723	0.06	14000	0.11	3.98	Methyl 9.cis.,11.trans.t,13.trans.-octadecatrien
20	32.956	613080	0.67	108389	0.83	5.66	Cis-3-ethyl-endo-tricyclo[5.2.1.0(2.6)]decane
21	33.490	507863	0.55	101844	0.78	4.99	Docosanoic acid, methyl ester
22	35.591	54124	0.06	11233	0.09	4.82	Tricosanoic acid, methyl ester
23	37.807	180899	0.20	29495	0.23	6.13	Tetracosanoic acid, methyl ester
		91973572	100.00	12982559	100.00		

Library

<< Target >>
Line#:1 R.Time:12.917(Scan#:351) MassPeaks:314
RawMode:Averaged 12.908-12.925(350-352) BasePeak:74.00(191937)
BG Mode:Calc. from Peak Group 1 - Event 1

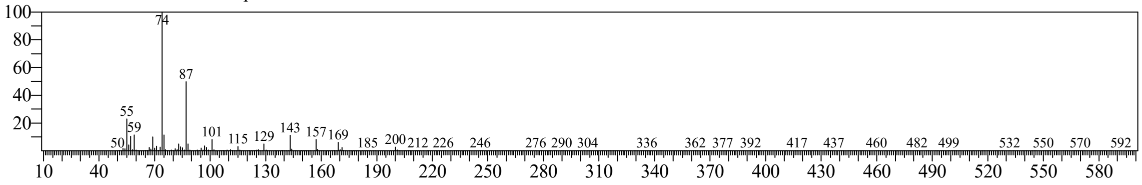

Hit#:1 Entry:46966 Library:NIST14.lib
SI:95 Formula:C12H24O2 CAS:1731-86-8 MolWeight:200 RetIndex:1381
CompName:Undecanoic acid, methyl ester $$ Methyl undecanoate $$ Methyl undecylenate $$ n-Undecanoic acid methyl ester $$ Methyl ester of undecanoi

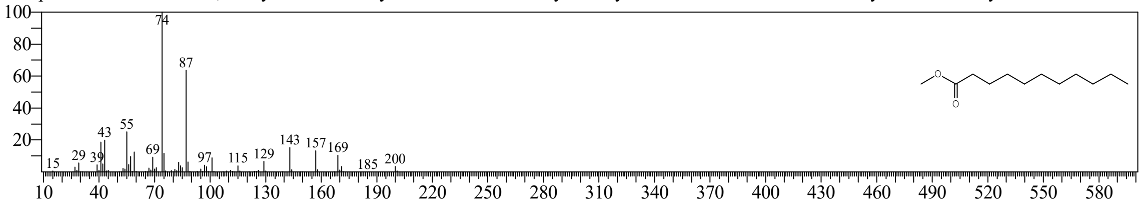

Hit#:2 Entry:49598 Library:NIST17-1.lib
SI:95 Formula:C12H24O2 CAS:1731-86-8 MolWeight:200 RetIndex:1381
CompName:Undecanoic acid, methyl ester $$ Methyl undecanoate $$ Methyl undecylenate $$ n-Undecanoic acid methyl ester $$ Methyl ester of undecanoi

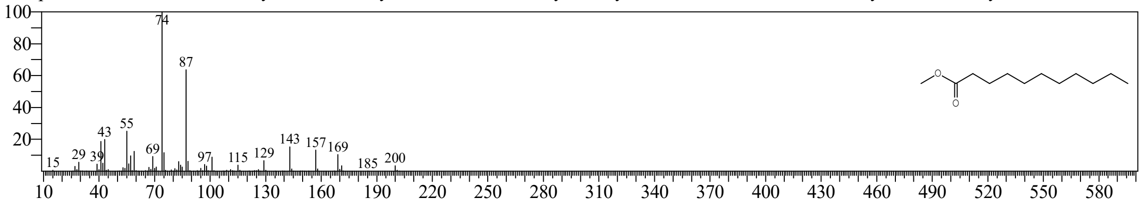

Hit#:3 Entry:21618 Library:NIST17s.lib
SI:95 Formula:C12H24O2 CAS:1731-86-8 MolWeight:200 RetIndex:1381
CompName:Undecanoic acid, methyl ester $$ Methyl undecanoate $$ Methyl undecylenate $$ n-Undecanoic acid methyl ester $$ Methyl ester of undecanoi

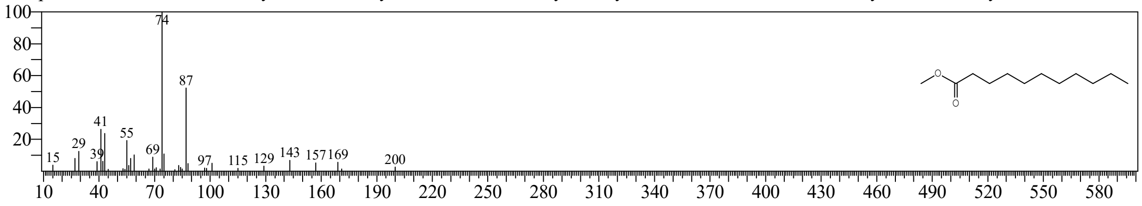

Hit#:4 Entry:18487 Library:NIST14s.lib
SI:95 Formula:C12H24O2 CAS:1731-86-8 MolWeight:200 RetIndex:1381
CompName:Undecanoic acid, methyl ester $$ Methyl undecanoate $$ Methyl undecylenate $$ n-Undecanoic acid methyl ester $$ Methyl ester of undecanoi

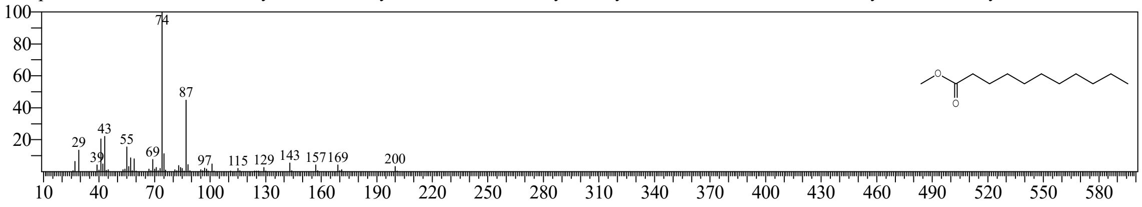

<< Target >>
Line#:1   R.Time:12.917(Scan#:351) MassPeaks:314
RawMode:Averaged 12.908-12.925(350-352) BasePeak:74.00(191937)
BG Mode:Calc. from Peak Group 1 - Event 1

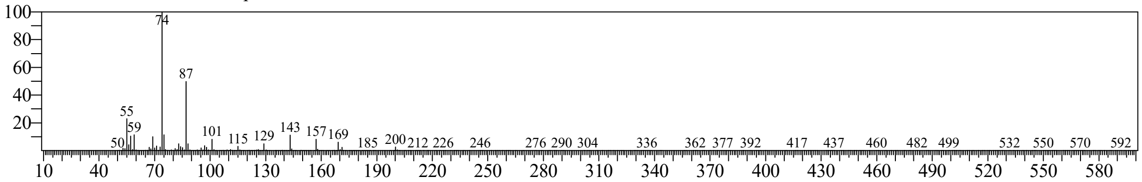

Hit#:5 Entry:21619 Library:NIST17s.lib
SI:95 Formula:C12H24O2 CAS:1731-86-8 MolWeight:200 RetIndex:1381
CompName:Undecanoic acid, methyl ester $$ Methyl undecanoate $$ Methyl undecylenate $$ n-Undecanoic acid methyl ester $$ Methyl ester of undecanoi

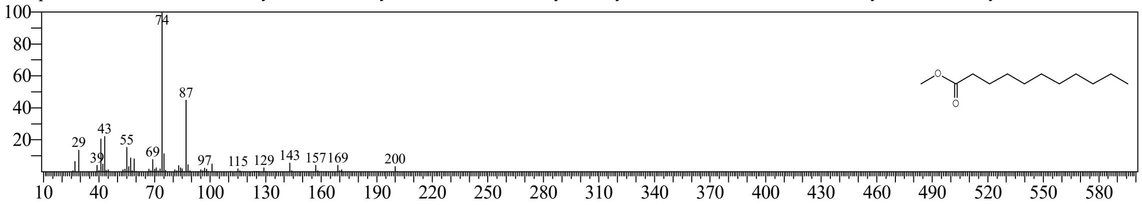

<< Target >>
Line#:2 R.Time:16.992(Scan#:840) MassPeaks:329
RawMode:Averaged 16.983-17.000(839-841) BasePeak:74.00(3319)
BG Mode:Calc. from Peak Group 1 - Event 1

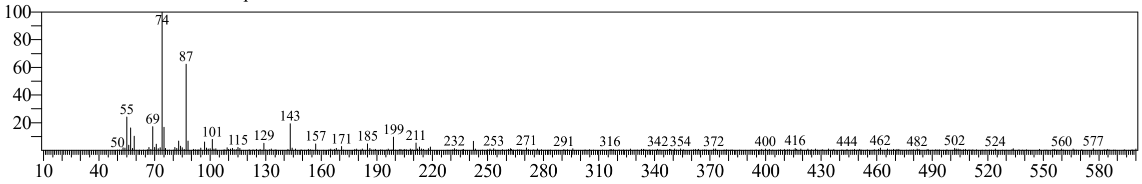

Hit#:1 Entry:86786 Library:NIST17-1.lib
SI:94 Formula:C15H30O2 CAS:124-10-7 MolWeight:242 RetIndex:1680
CompName:Methyl tetradecanoate $$ Tetradecanoic acid, methyl ester $$ Myristic acid, methyl ester $$ Metholeneat 2495 $$ Methyl myristate $$ Methyl r

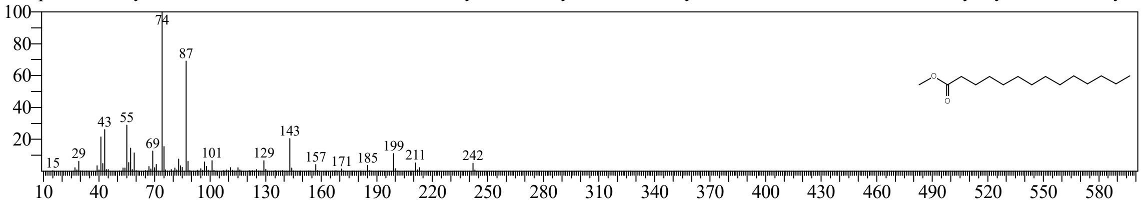

Hit#:2 Entry:80645 Library:NIST14.lib
SI:94 Formula:C15H30O2 CAS:124-10-7 MolWeight:242 RetIndex:1680
CompName:Methyl tetradecanoate $$ Tetradecanoic acid, methyl ester $$ Myristic acid, methyl ester $$ Metholeneat 2495 $$ Methyl myristate $$ Methyl r

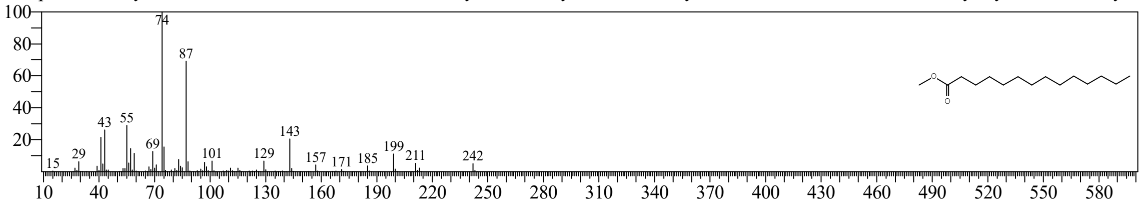

Hit#:3 Entry:27874 Library:NIST17s.lib
SI:92 Formula:C15H30O2 CAS:124-10-7 MolWeight:242 RetIndex:1680
CompName:Methyl tetradecanoate $$ Tetradecanoic acid, methyl ester $$ Myristic acid, methyl ester $$ Metholeneat 2495 $$ Methyl myristate $$ Methyl r

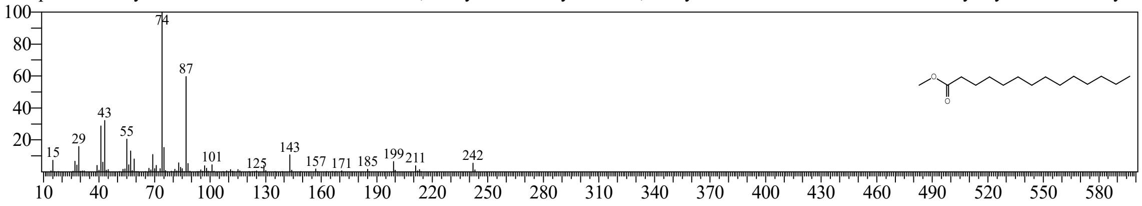

Hit#:4 Entry:23848 Library:NIST14s.lib
SI:92 Formula:C15H30O2 CAS:124-10-7 MolWeight:242 RetIndex:1680
CompName:Methyl tetradecanoate $$ Tetradecanoic acid, methyl ester $$ Myristic acid, methyl ester $$ Metholeneat 2495 $$ Methyl myristate $$ Methyl r

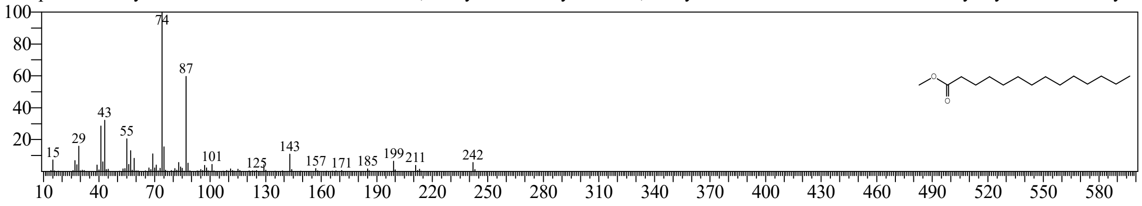

Hit#:5 Entry:27873 Library:NIST17s.lib
SI:92 Formula:C15H30O2 CAS:124-10-7 MolWeight:242 RetIndex:1680
CompName:Methyl tetradecanoate $$ Tetradecanoic acid, methyl ester $$ Myristic acid, methyl ester $$ Metholeneat 2495 $$ Methyl myristate $$ Methyl r

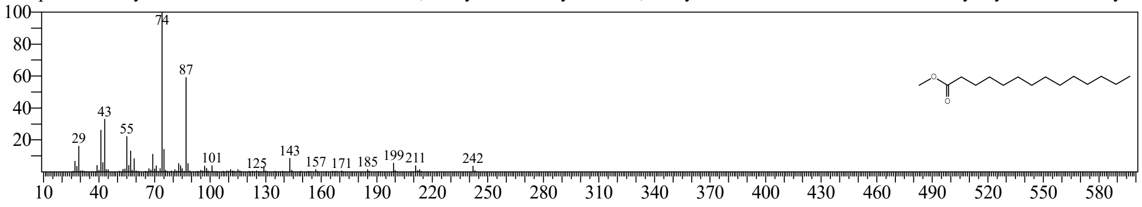

<< Target >>
Line#:3 R.Time:20.808(Scan#:1298) MassPeaks:391
RawMode:Averaged 20.800-20.817(1297-1299) BasePeak:74.00(465920)
BG Mode:Calc. from Peak Group 1 - Event 1

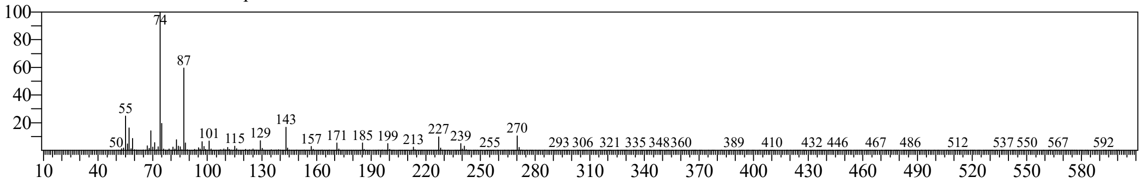

Hit#:1 Entry:113663 Library:NIST17-1.lib
SI:97 Formula:C17H34O2 CAS:112-39-0 MolWeight:270 RetIndex:1878
CompName:Hexadecanoic acid, methyl ester $$ Palmitic acid, methyl ester $$ n-Hexadecanoic acid methyl ester $$ Metholene 2216 $$ Methyl hexadecano

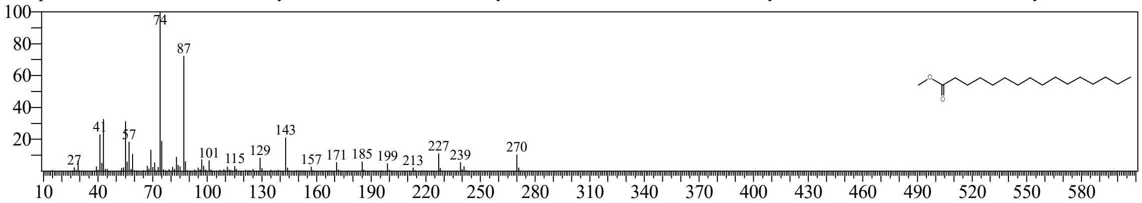

Hit#:2 Entry:104648 Library:NIST14.lib
SI:97 Formula:C17H34O2 CAS:112-39-0 MolWeight:270 RetIndex:1878
CompName:Hexadecanoic acid, methyl ester $$ Palmitic acid, methyl ester $$ n-Hexadecanoic acid methyl ester $$ Metholene 2216 $$ Methyl hexadecano

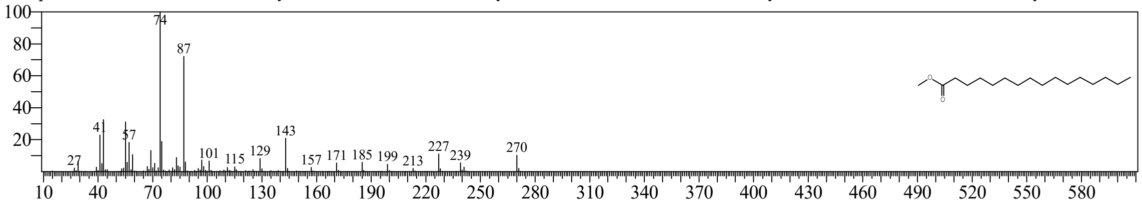

Hit#:3 Entry:26273 Library:NIST14s.lib
SI:97 Formula:C17H34O2 CAS:112-39-0 MolWeight:270 RetIndex:1878
CompName:Hexadecanoic acid, methyl ester $$ Palmitic acid, methyl ester $$ n-Hexadecanoic acid methyl ester $$ Metholene 2216 $$ Methyl hexadecano

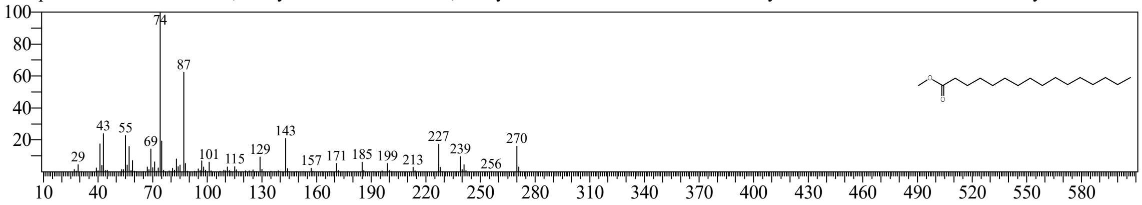

Hit#:4 Entry:26271 Library:NIST14s.lib
SI:96 Formula:C17H34O2 CAS:112-39-0 MolWeight:270 RetIndex:1878
CompName:Hexadecanoic acid, methyl ester $$ Palmitic acid, methyl ester $$ n-Hexadecanoic acid methyl ester $$ Metholene 2216 $$ Methyl hexadecano

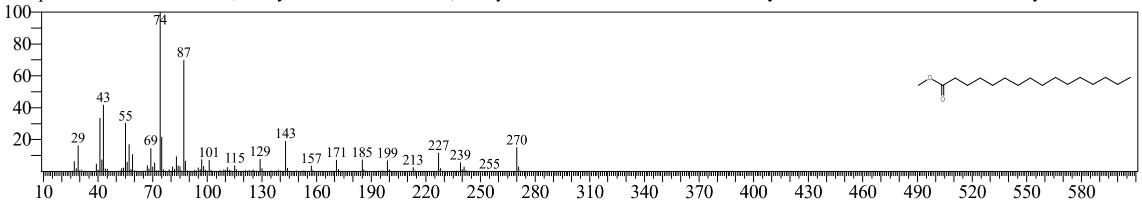

Hit#:5 Entry:30646 Library:NIST17s.lib
SI:96 Formula:C17H34O2 CAS:112-39-0 MolWeight:270 RetIndex:1878
CompName:Hexadecanoic acid, methyl ester $$ Palmitic acid, methyl ester $$ n-Hexadecanoic acid methyl ester $$ Metholene 2216 $$ Methyl hexadecano

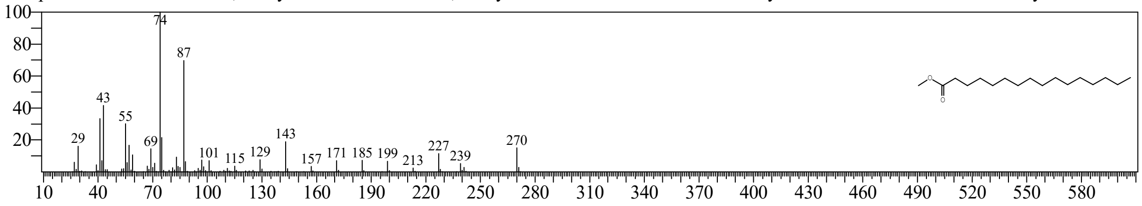

<< Target >>
Line#:4 R.Time:22.158(Scan#:1460) MassPeaks:297
RawMode:Averaged 22.150-22.167(1459-1461) BasePeak:55.05(1486)
BG Mode:Calc. from Peak Group 1 - Event 1

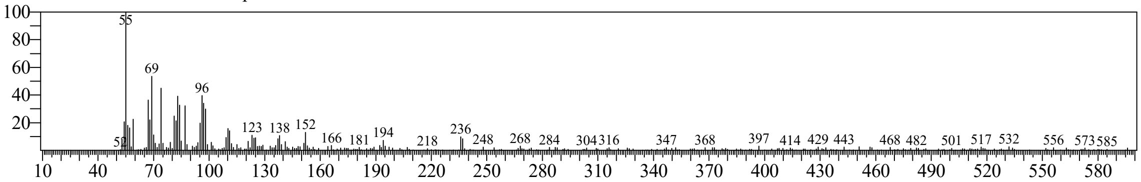

Hit#:1 Entry:26066 Library:NIST14s.lib
SI:94 Formula:C17H32O2 CAS:1120-25-8 MolWeight:268 RetIndex:1886
CompName:9-Hexadecenoic acid, methyl ester, (Z)- $$ Methyl palmitoleate $$ Methyl palmitoleinate $$ Palmitoleic acid, methyl ester $$ Methyl (9Z)-9-he:

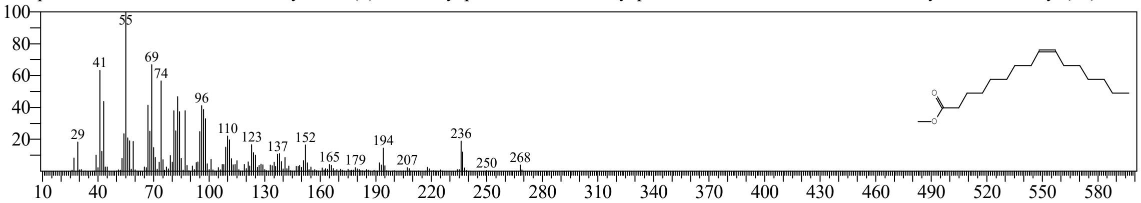

Hit#:2 Entry:30415 Library:NIST17s.lib
SI:94 Formula:C17H32O2 CAS:1120-25-8 MolWeight:268 RetIndex:1886
CompName:9-Hexadecenoic acid, methyl ester, (Z)- $$ Methyl palmitoleate $$ Methyl palmitoleinate $$ Palmitoleic acid, methyl ester $$ Methyl (9Z)-9-he:

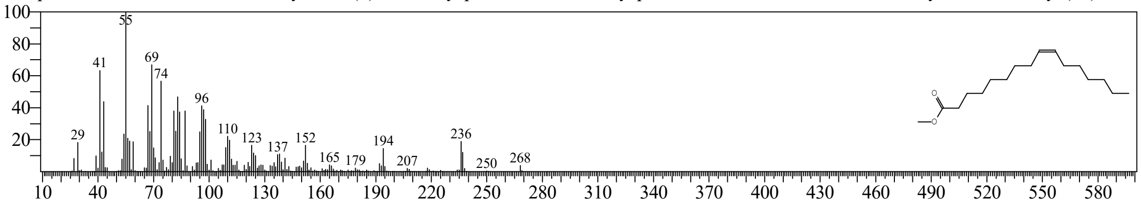

Hit#:3 Entry:111510 Library:NIST17-1.lib
SI:93 Formula:C17H32O2 CAS:56875-67-3 MolWeight:268 RetIndex:1886
CompName:7-Hexadecenoic acid, methyl ester, (Z)- $$ Methyl (7E)-7-hexadecenoate # $$

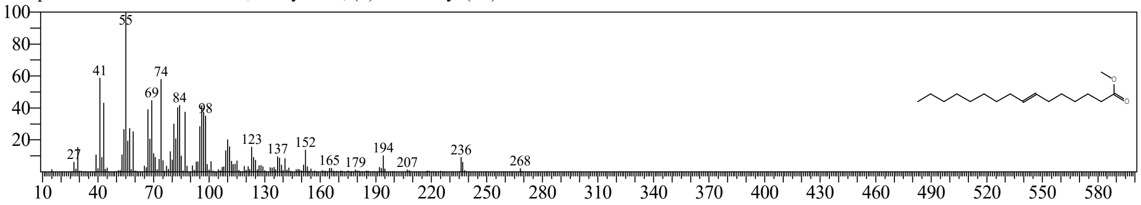

Hit#:4 Entry:26064 Library:NIST14s.lib
SI:93 Formula:C17H32O2 CAS:1120-25-8 MolWeight:268 RetIndex:1886
CompName:9-Hexadecenoic acid, methyl ester, (Z)- $$ Methyl palmitoleate $$ Methyl palmitoleinate $$ Palmitoleic acid, methyl ester $$ Methyl (9Z)-9-he:

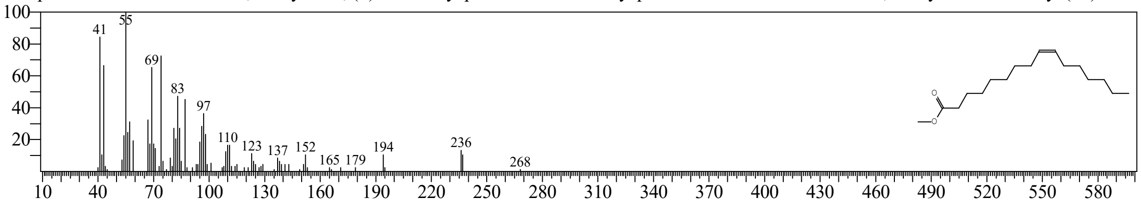

Hit#:5 Entry:30413 Library:NIST17s.lib
SI:93 Formula:C17H32O2 CAS:1120-25-8 MolWeight:268 RetIndex:1886
CompName:9-Hexadecenoic acid, methyl ester, (Z)- $$ Methyl palmitoleate $$ Methyl palmitoleinate $$ Palmitoleic acid, methyl ester $$ Methyl (9Z)-9-he:

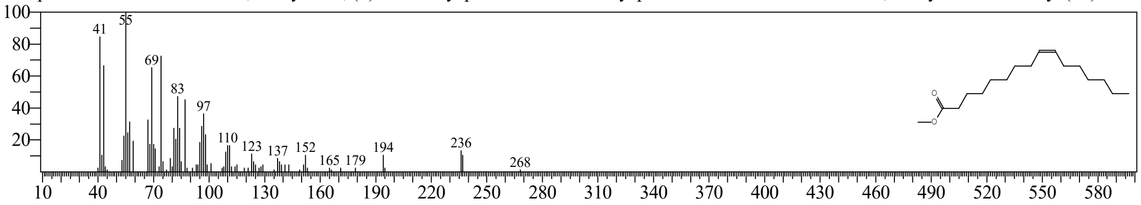

<< Target >>
Line#:5 R.Time:22.783(Scan#:1535) MassPeaks:329
RawMode:Averaged 22.775-22.792(1534-1536) BasePeak:74.00(3375)
BG Mode:Calc. from Peak Group 1 - Event 1

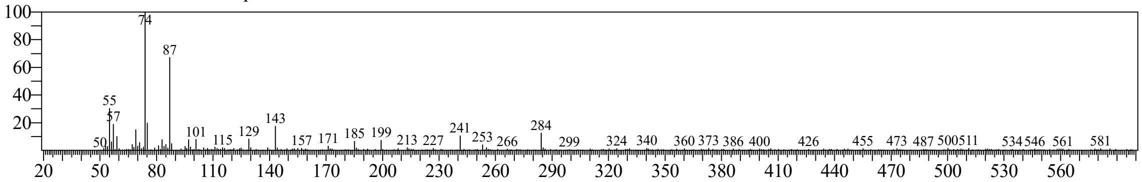

Hit#:1 Entry:31784 Library:NIST17s.lib
SI:94 Formula:C18H36O2 CAS:1731-92-6 MolWeight:284 RetIndex:1978
CompName:Heptadecanoic acid, methyl ester $$ Margaric acid methyl ester $$ Methyl heptadecanoate $$ Methyl margarate $$ n-Heptadecanoic acid methy

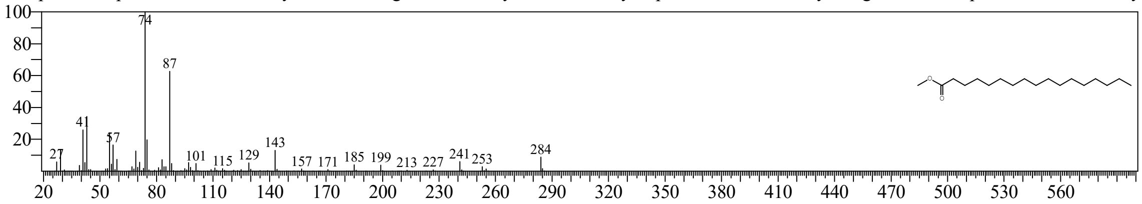

Hit#:2 Entry:27275 Library:NIST14s.lib
SI:94 Formula:C18H36O2 CAS:1731-92-6 MolWeight:284 RetIndex:1978
CompName:Heptadecanoic acid, methyl ester $$ Margaric acid methyl ester $$ Methyl heptadecanoate $$ Methyl margarate $$ n-Heptadecanoic acid methy

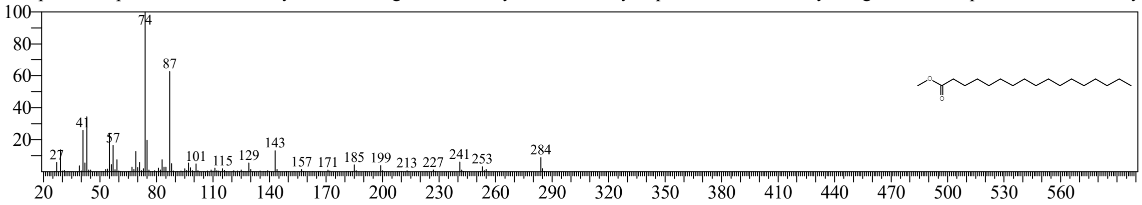

Hit#:3 Entry:27277 Library:NIST14s.lib
SI:93 Formula:C18H36O2 CAS:6929-04-0 MolWeight:284 RetIndex:1914
CompName:Hexadecanoic acid, 15-methyl-, methyl ester $$ Methyl isoheptadecanoate $$ Methyl 15-methylhexadecanoate $$

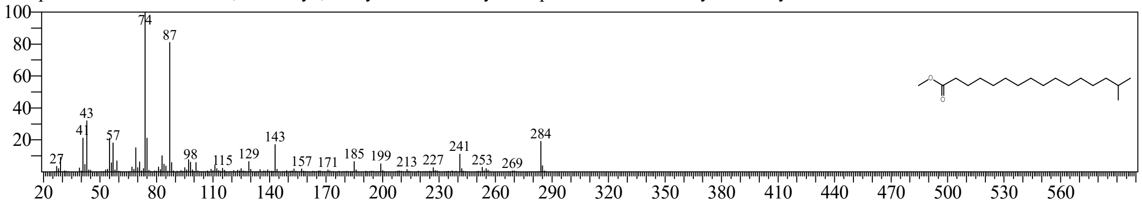

Hit#:4 Entry:127714 Library:NIST17-1.lib
SI:93 Formula:C18H36O2 CAS:6929-04-0 MolWeight:284 RetIndex:1914
CompName:Hexadecanoic acid, 15-methyl-, methyl ester $$ Methyl isoheptadecanoate $$ Methyl 15-methylhexadecanoate $$

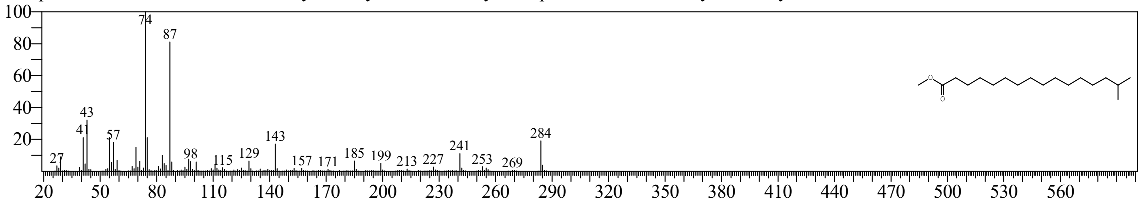

Hit#:5 Entry:31783 Library:NIST17s.lib
SI:93 Formula:C18H36O2 CAS:1731-92-6 MolWeight:284 RetIndex:1978
CompName:Heptadecanoic acid, methyl ester $$ Margaric acid methyl ester $$ Methyl heptadecanoate $$ Methyl margarate $$ n-Heptadecanoic acid methy

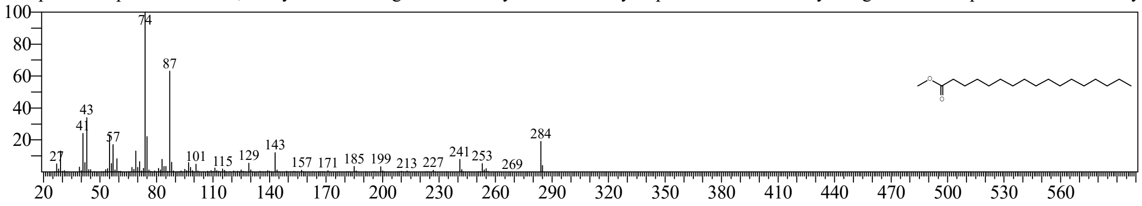

<< Target >>
Line#:6 R.Time:24.867(Scan#:1785) MassPeaks:326
RawMode:Averaged 24.858-24.875(1784-1786) BasePeak:74.00(131762)
BG Mode:Calc. from Peak Group 1 - Event 1

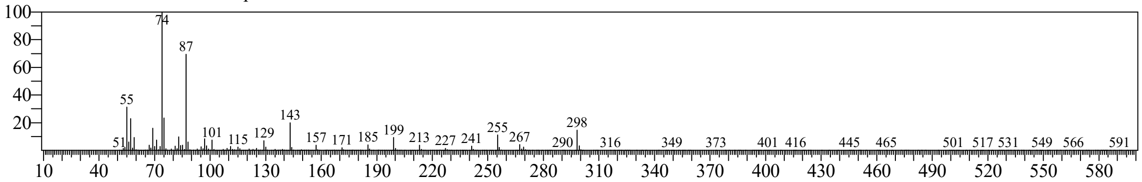

Hit#:1 Entry:129694 Library:NIST14.lib
SI:98 Formula:C19H38O2 CAS:112-61-8 MolWeight:298 RetIndex:2077
CompName:Methyl stearate $$ Octadecanoic acid, methyl ester $$ Stearic acid, methyl ester $$ n-Octadecanoic acid, methyl ester $$ Kemester 9718 $$ Me

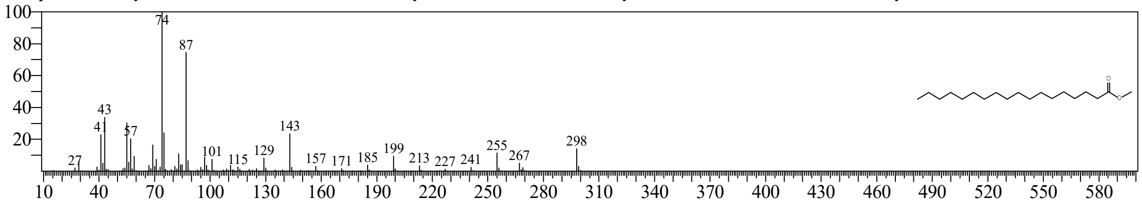

Hit#:2 Entry:141978 Library:NIST17-1.lib
SI:98 Formula:C19H38O2 CAS:112-61-8 MolWeight:298 RetIndex:2077
CompName:Methyl stearate $$ Octadecanoic acid, methyl ester $$ Stearic acid, methyl ester $$ n-Octadecanoic acid, methyl ester $$ Kemester 9718 $$ Me

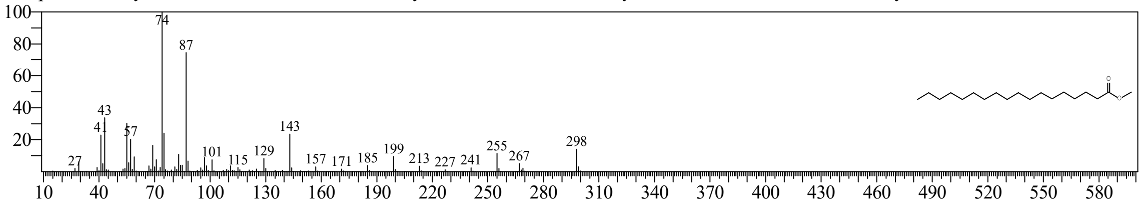

Hit#:3 Entry:28255 Library:NIST14s.lib
SI:97 Formula:C19H38O2 CAS:112-61-8 MolWeight:298 RetIndex:2077
CompName:Methyl stearate $$ Octadecanoic acid, methyl ester $$ Stearic acid, methyl ester $$ n-Octadecanoic acid, methyl ester $$ Kemester 9718 $$ Me

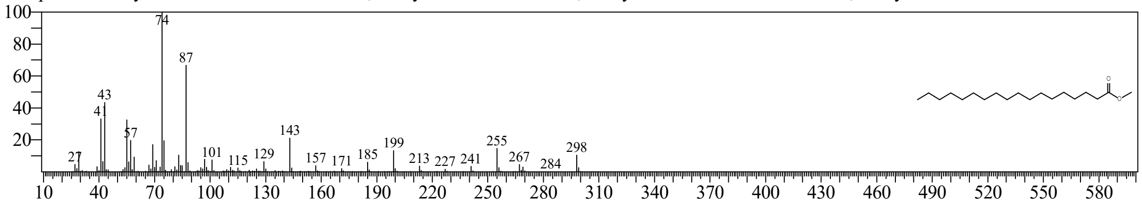

Hit#:4 Entry:32873 Library:NIST17s.lib
SI:97 Formula:C19H38O2 CAS:112-61-8 MolWeight:298 RetIndex:2077
CompName:Methyl stearate $$ Octadecanoic acid, methyl ester $$ Stearic acid, methyl ester $$ n-Octadecanoic acid, methyl ester $$ Kemester 9718 $$ Me

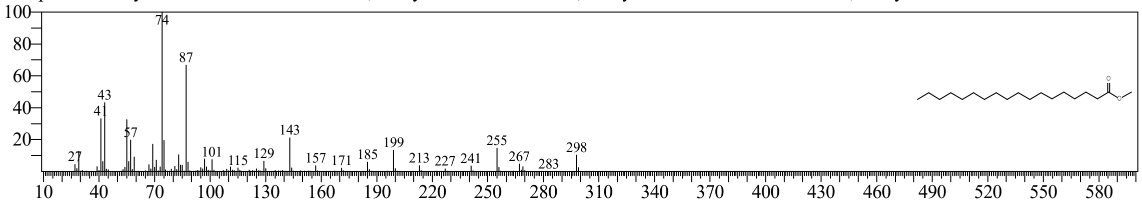

Hit#:5 Entry:28257 Library:NIST14s.lib
SI:96 Formula:C19H38O2 CAS:112-61-8 MolWeight:298 RetIndex:2077
CompName:Methyl stearate $$ Octadecanoic acid, methyl ester $$ Stearic acid, methyl ester $$ n-Octadecanoic acid, methyl ester $$ Kemester 9718 $$ Me

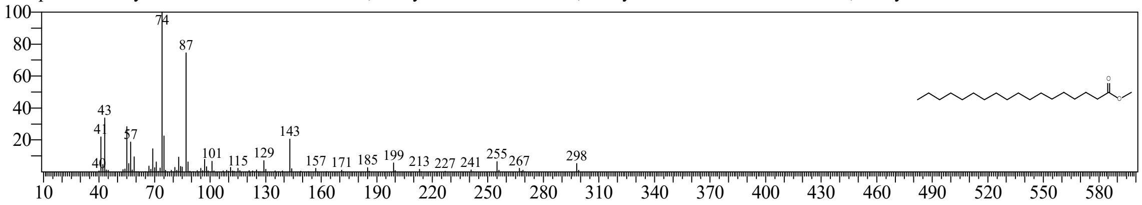

<< Target >>
Line#:7 R.Time:26.258(Scan#:1952) MassPeaks:363
RawMode:Averaged 26.250-26.267(1951-1953) BasePeak:55.05(239767)
BG Mode:Calc. from Peak Group 1 - Event 1

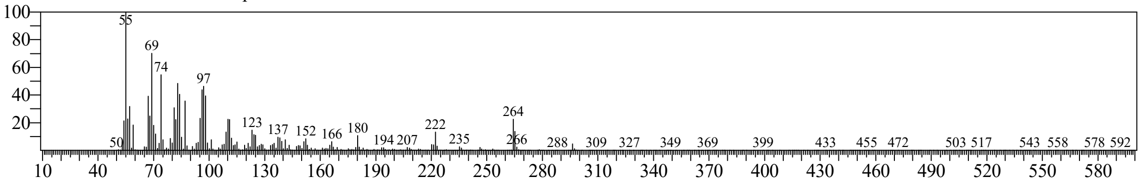

Hit#:1 Entry:32747 Library:NIST17s.lib
SI:97 Formula:C19H36O2 CAS:1937-62-8 MolWeight:296 RetIndex:2085
CompName:9-Octadecenoic acid, methyl ester, (E)- $$ Elaidic acid, methyl ester $$ Methyl elaidate $$ Methyl trans-9-octadecenoate $$ (E)-9-Octadecenoi

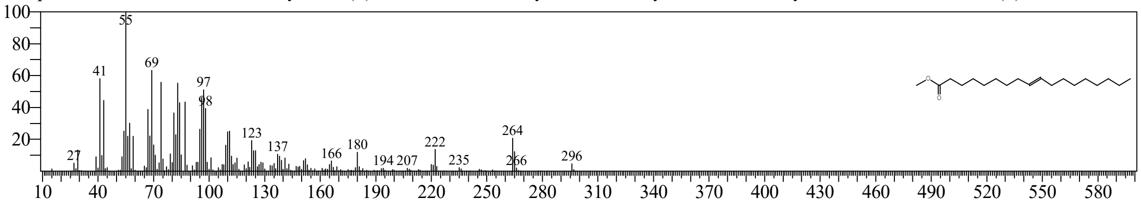

Hit#:2 Entry:28138 Library:NIST14s.lib
SI:97 Formula:C19H36O2 CAS:1937-62-8 MolWeight:296 RetIndex:2085
CompName:9-Octadecenoic acid, methyl ester, (E)- $$ Elaidic acid, methyl ester $$ Methyl elaidate $$ Methyl trans-9-octadecenoate $$ (E)-9-Octadecenoi

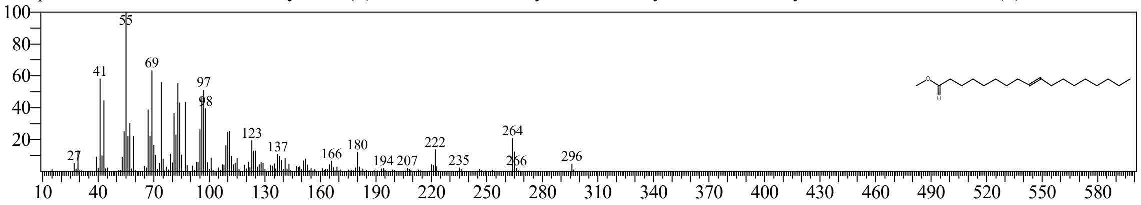

Hit#:3 Entry:28139 Library:NIST14s.lib
SI:96 Formula:C19H36O2 CAS:52380-33-3 MolWeight:296 RetIndex:2085
CompName:11-Octadecenoic acid, methyl ester $$ Methyl 11-octadecenoate $$ Octadec-11-enoic acid, methyl ester $$ Methyl (11E)-11-octadecenoate # $$

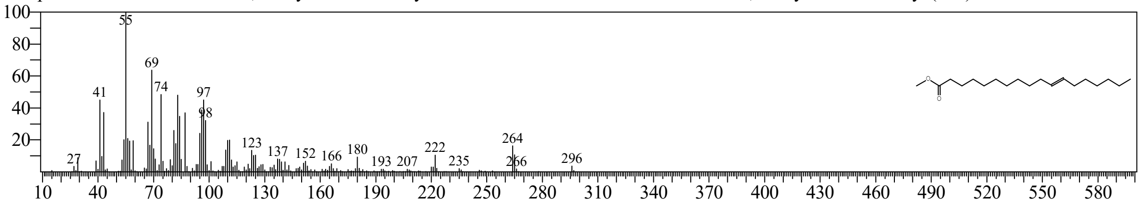

Hit#:4 Entry:127664 Library:NIST14.lib
SI:96 Formula:C19H36O2 CAS:112-62-9 MolWeight:296 RetIndex:2085
CompName:9-Octadecenoic acid (Z)-, methyl ester $$ Oleic acid, methyl ester $$ Emery oleic acid ester 2301 $$ Methyl cis-9-octadecenoate $$ Methyl ole

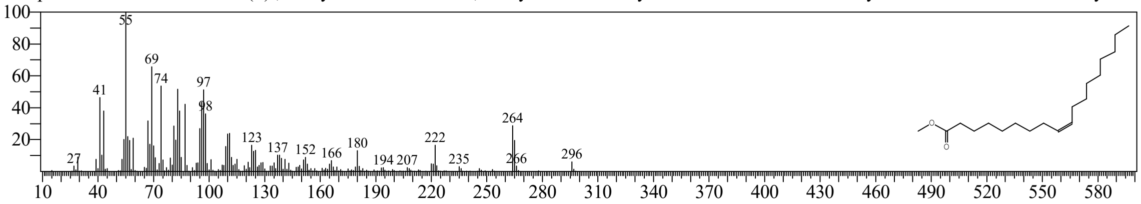

Hit#:5 Entry:32748 Library:NIST17s.lib
SI:96 Formula:C19H36O2 CAS:52380-33-3 MolWeight:296 RetIndex:2085
CompName:11-Octadecenoic acid, methyl ester $$ Methyl 11-octadecenoate $$ Octadec-11-enoic acid, methyl ester $$ Methyl (11E)-11-octadecenoate # $$

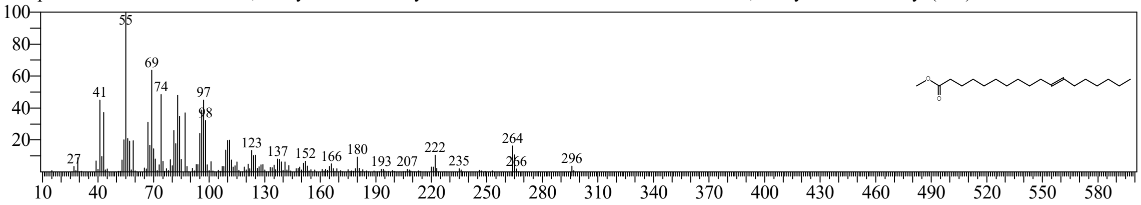

<< Target >>
Line#:8 R.Time:26.375(Scan#:1966) MassPeaks:383
RawMode:Averaged 26.367-26.383(1965-1967) BasePeak:55.05(18624)
BG Mode:Calc. from Peak Group 1 - Event 1

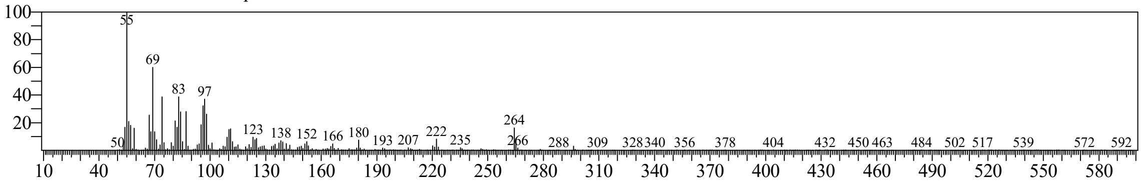

Hit#:1 Entry:139647 Library:NIST17-1.lib
SI:95 Formula:C19H36O2 CAS:0-00-0 MolWeight:296 RetIndex:2085
CompName:cis-13-Octadecenoic acid, methyl ester

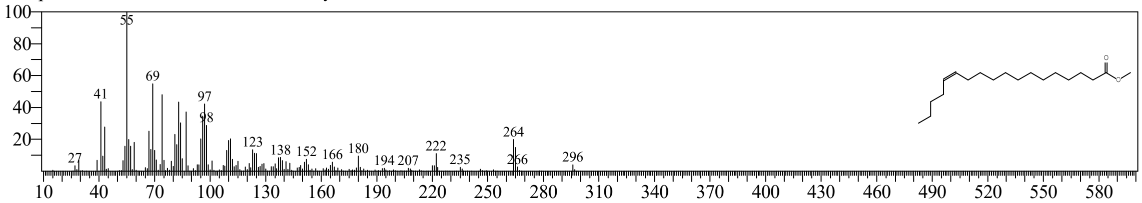

Hit#:2 Entry:127661 Library:NIST14.lib
SI:95 Formula:C19H36O2 CAS:0-00-0 MolWeight:296 RetIndex:2085
CompName:cis-13-Octadecenoic acid, methyl ester

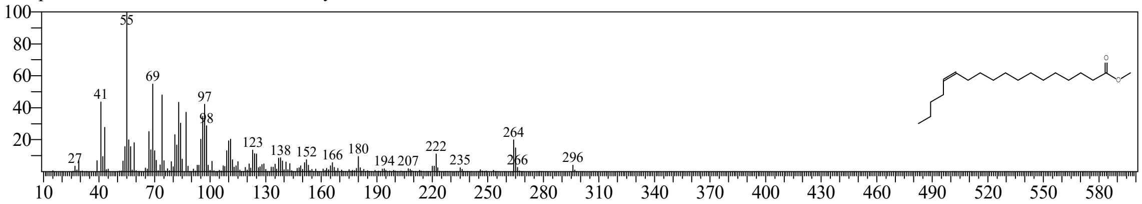

Hit#:3 Entry:32748 Library:NIST17s.lib
SI:95 Formula:C19H36O2 CAS:52380-33-3 MolWeight:296 RetIndex:2085
CompName:11-Octadecenoic acid, methyl ester $$ Methyl 11-octadecenoate $$ Octadec-11-enoic acid, methyl ester $$ Methyl (11E)-11-octadecenoate # $$

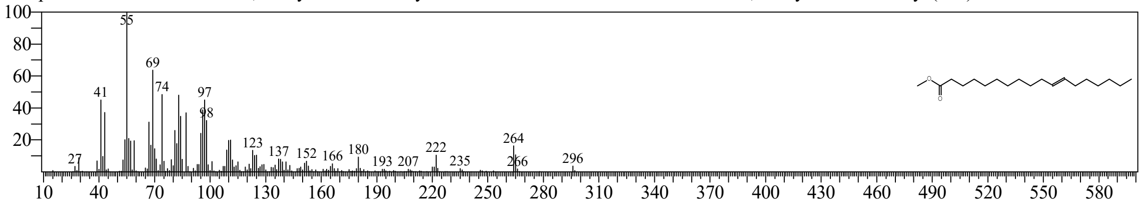

Hit#:4 Entry:28139 Library:NIST14s.lib
SI:95 Formula:C19H36O2 CAS:52380-33-3 MolWeight:296 RetIndex:2085
CompName:11-Octadecenoic acid, methyl ester $$ Methyl 11-octadecenoate $$ Octadec-11-enoic acid, methyl ester $$ Methyl (11E)-11-octadecenoate # $$

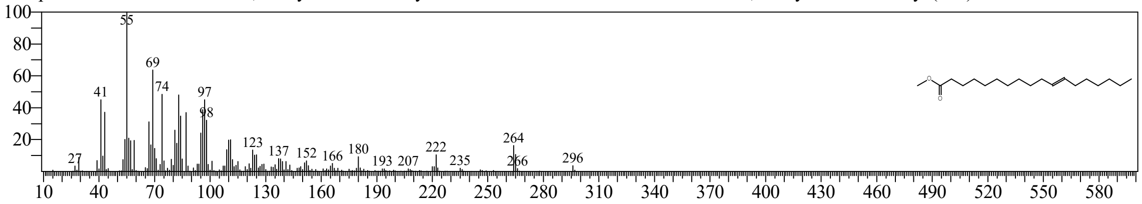

Hit#:5 Entry:127658 Library:NIST14.lib
SI:95 Formula:C19H36O2 CAS:0-00-0 MolWeight:296 RetIndex:2085
CompName:trans-13-Octadecenoic acid, methyl ester

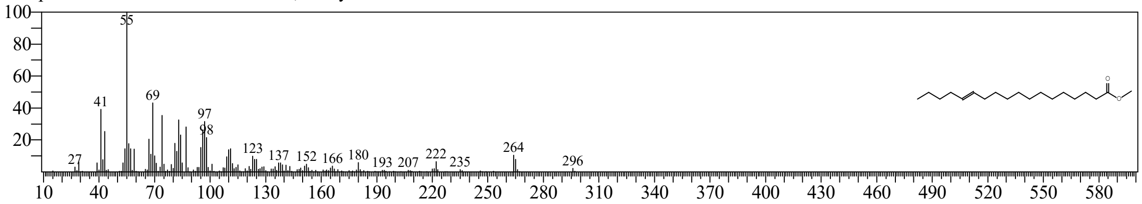

<< Target >>
Line#:9 R.Time:27.725(Scan#:2128) MassPeaks:331
RawMode:Averaged 27.717-27.733(2127-2129) BasePeak:67.05(3882)
BG Mode:Calc. from Peak Group 1 - Event 1

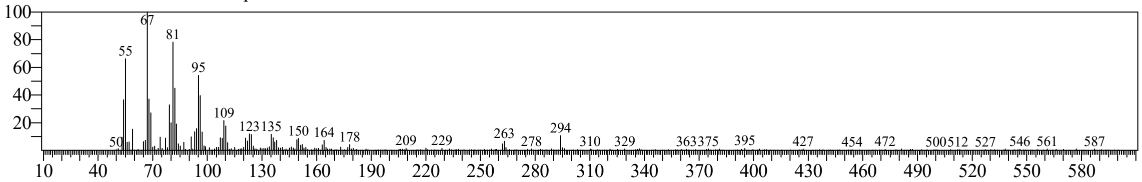

Hit#:1 Entry:27999 Library:NIST14s.lib
SI:96 Formula:C19H34O2 CAS:112-63-0 MolWeight:294 RetIndex:2093
CompName:9,12-Octadecadienoic acid (Z,Z)-, methyl ester $$ Linoleic acid, methyl ester $$ Methyl cis,cis-9,12-octadecadienoate $$ Methyl linoleate $$ M

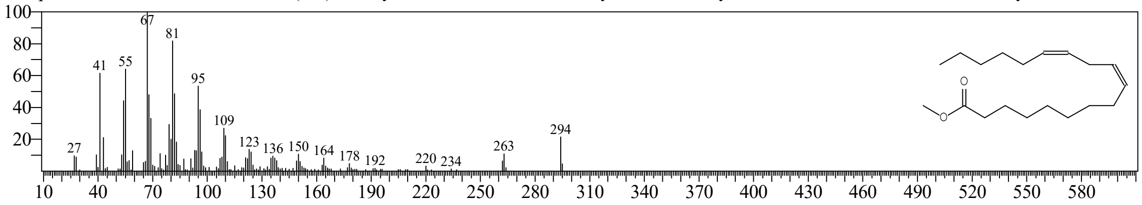

Hit#:2 Entry:137663 Library:NIST17-1.lib
SI:96 Formula:C19H34O2 CAS:2462-85-3 MolWeight:294 RetIndex:2093
CompName:9,12-Octadecadienoic acid, methyl ester $$ 9,12-Octadecenoic acid, methyl ester $$ Methyl octadeca-9,12-dienoate $$ Methyl 9,12-octadecadi

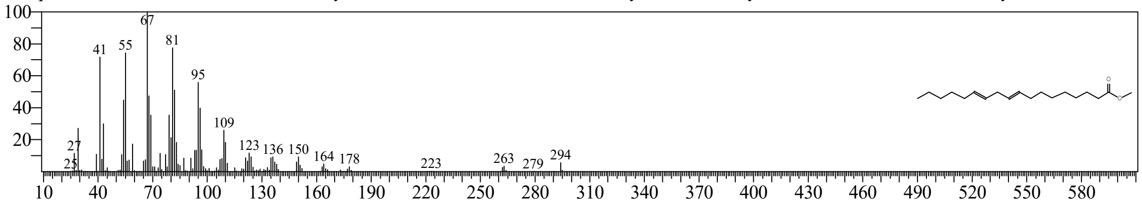

Hit#:3 Entry:32588 Library:NIST17s.lib
SI:96 Formula:C19H34O2 CAS:112-63-0 MolWeight:294 RetIndex:2093
CompName:9,12-Octadecadienoic acid (Z,Z)-, methyl ester $$ Linoleic acid, methyl ester $$ Methyl cis,cis-9,12-octadecadienoate $$ Methyl linoleate $$ M

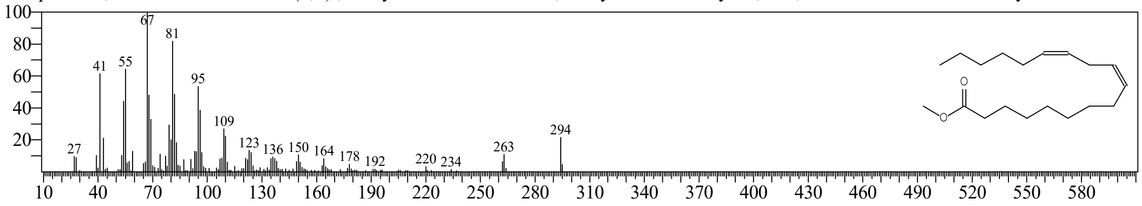

Hit#:4 Entry:125931 Library:NIST14.lib
SI:96 Formula:C19H34O2 CAS:2462-85-3 MolWeight:294 RetIndex:2093
CompName:9,12-Octadecadienoic acid, methyl ester $$ 9,12-Octadecenoic acid, methyl ester $$ Methyl octadeca-9,12-dienoate $$ Methyl 9,12-octadecadi

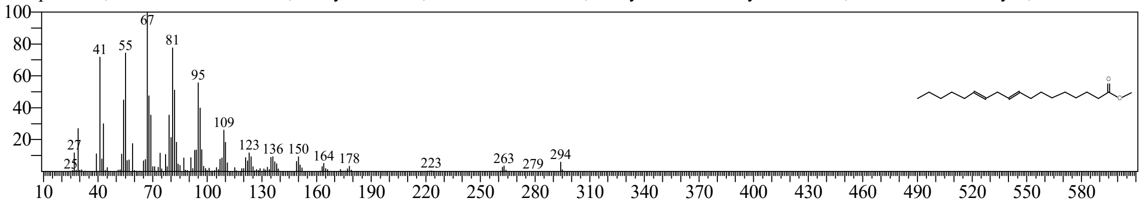

Hit#:5 Entry:32589 Library:NIST17s.lib
SI:96 Formula:C19H34O2 CAS:112-63-0 MolWeight:294 RetIndex:2093
CompName:9,12-Octadecadienoic acid (Z,Z)-, methyl ester $$ Linoleic acid, methyl ester $$ Methyl cis,cis-9,12-octadecadienoate $$ Methyl linoleate $$ M

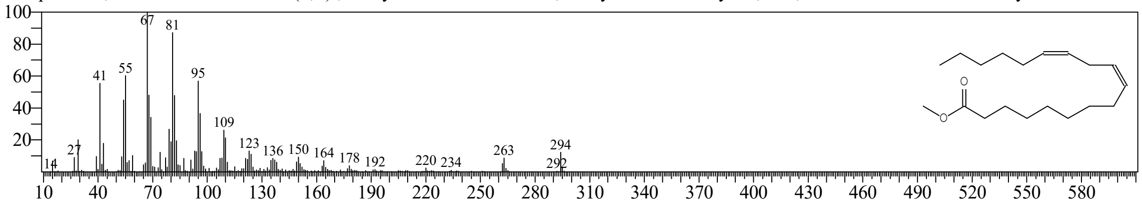

<< Target >>
Line#:10   R.Time:27.942(Scan#:2154) MassPeaks:356
RawMode:Averaged 27.933-27.950(2153-2155) BasePeak:67.05(2682)
BG Mode:Calc. from Peak Group 1 - Event 1

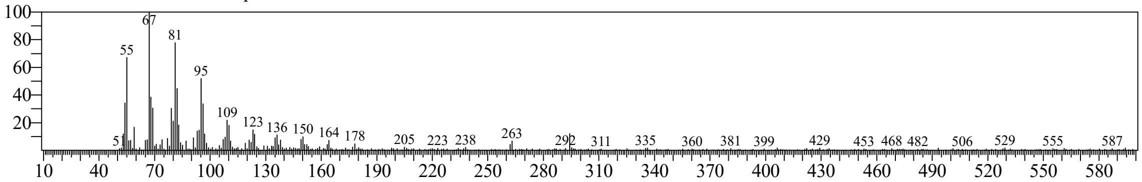

Hit#:1 Entry:27999 Library:NIST14s.lib
SI:95 Formula:C19H34O2 CAS:112-63-0 MolWeight:294 RetIndex:2093
CompName:9,12-Octadecadienoic acid (Z,Z)-, methyl ester $$ Linoleic acid, methyl ester $$ Methyl cis,cis-9,12-octadecadienoate $$ Methyl linoleate $$ M

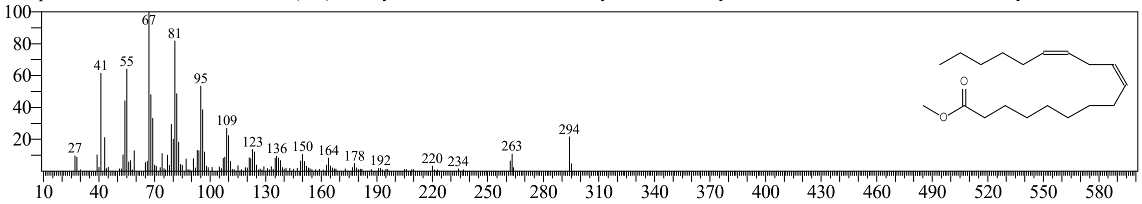

Hit#:2 Entry:32588 Library:NIST17s.lib
SI:95 Formula:C19H34O2 CAS:112-63-0 MolWeight:294 RetIndex:2093
CompName:9,12-Octadecadienoic acid (Z,Z)-, methyl ester $$ Linoleic acid, methyl ester $$ Methyl cis,cis-9,12-octadecadienoate $$ Methyl linoleate $$ M

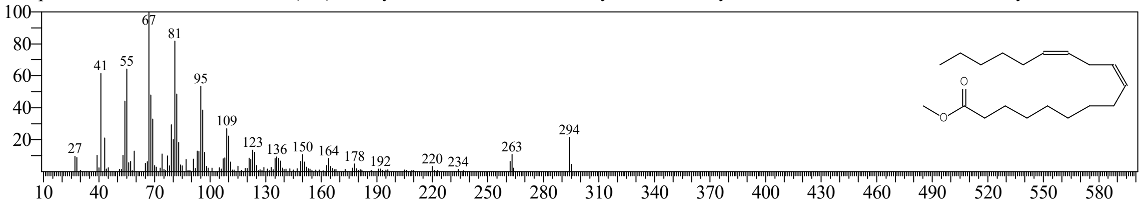

Hit#:3 Entry:125931 Library:NIST14.lib
SI:95 Formula:C19H34O2 CAS:2462-85-3 MolWeight:294 RetIndex:2093
CompName:9,12-Octadecadienoic acid, methyl ester $$ 9,12-Octadecenoic acid, methyl ester $$ Methyl octadeca-9,12-dienoate $$ Methyl 9,12-octadecadi

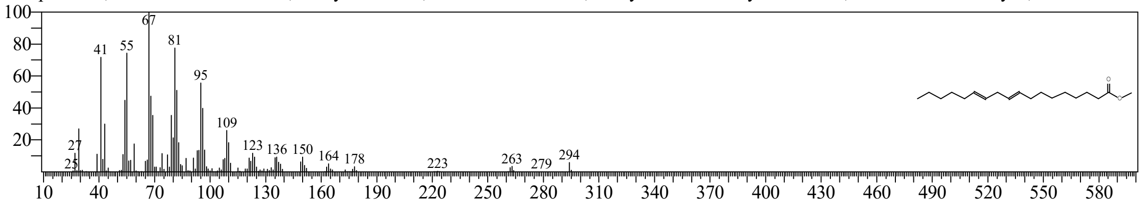

Hit#:4 Entry:137663 Library:NIST17-1.lib
SI:95 Formula:C19H34O2 CAS:2462-85-3 MolWeight:294 RetIndex:2093
CompName:9,12-Octadecadienoic acid, methyl ester $$ 9,12-Octadecenoic acid, methyl ester $$ Methyl octadeca-9,12-dienoate $$ Methyl 9,12-octadecadi

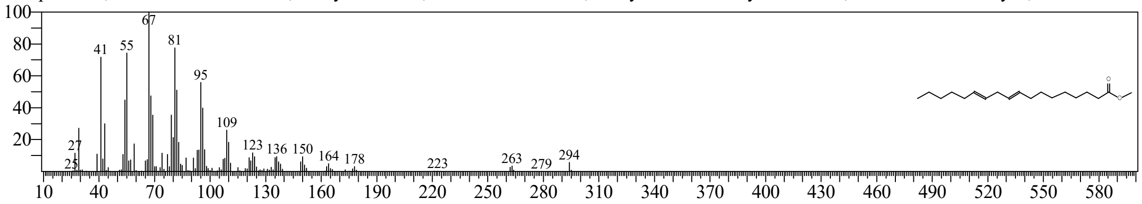

Hit#:5 Entry:32589 Library:NIST17s.lib
SI:94 Formula:C19H34O2 CAS:112-63-0 MolWeight:294 RetIndex:2093
CompName:9,12-Octadecadienoic acid (Z,Z)-, methyl ester $$ Linoleic acid, methyl ester $$ Methyl cis,cis-9,12-octadecadienoate $$ Methyl linoleate $$ M

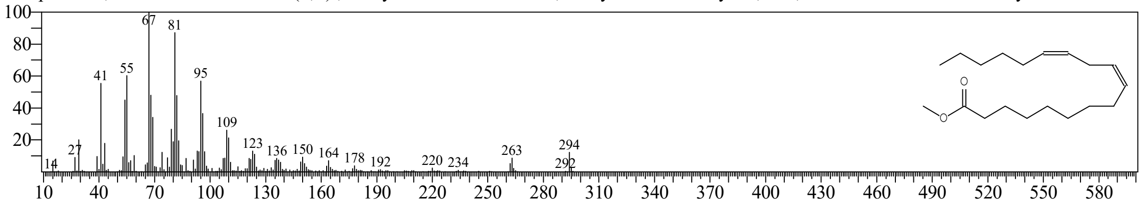

<< Target >>
Line#:11 R.Time:28.367(Scan#:2205) MassPeaks:410
RawMode:Averaged 28.358-28.375(2204-2206) BasePeak:67.05(453163)
BG Mode:Calc. from Peak Group 1 - Event 1

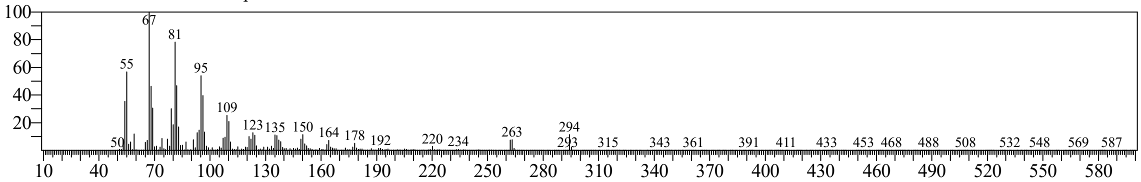

Hit#:1 Entry:32588 Library:NIST17s.lib
SI:97 Formula:C19H34O2 CAS:112-63-0 MolWeight:294 RetIndex:2093
CompName:9,12-Octadecadienoic acid (Z,Z)-, methyl ester $$ Linoleic acid, methyl ester $$ Methyl cis,cis-9,12-octadecadienoate $$ Methyl linoleate $$ M

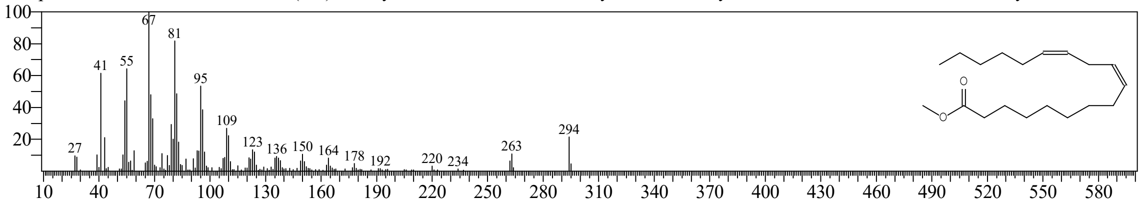

Hit#:2 Entry:27999 Library:NIST14s.lib
SI:97 Formula:C19H34O2 CAS:112-63-0 MolWeight:294 RetIndex:2093
CompName:9,12-Octadecadienoic acid (Z,Z)-, methyl ester $$ Linoleic acid, methyl ester $$ Methyl cis,cis-9,12-octadecadienoate $$ Methyl linoleate $$ M

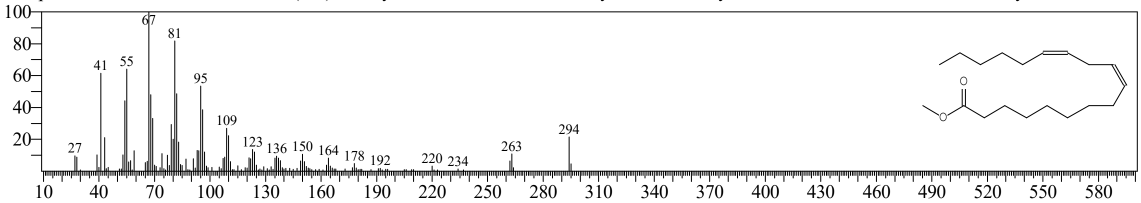

Hit#:3 Entry:32589 Library:NIST17s.lib
SI:97 Formula:C19H34O2 CAS:112-63-0 MolWeight:294 RetIndex:2093
CompName:9,12-Octadecadienoic acid (Z,Z)-, methyl ester $$ Linoleic acid, methyl ester $$ Methyl cis,cis-9,12-octadecadienoate $$ Methyl linoleate $$ M

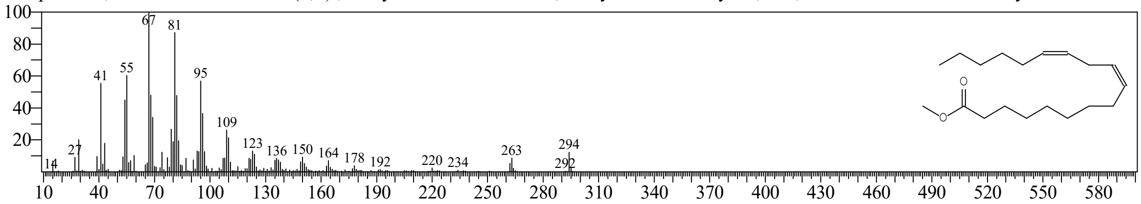

Hit#:4 Entry:28000 Library:NIST14s.lib
SI:97 Formula:C19H34O2 CAS:112-63-0 MolWeight:294 RetIndex:2093
CompName:9,12-Octadecadienoic acid (Z,Z)-, methyl ester $$ Linoleic acid, methyl ester $$ Methyl cis,cis-9,12-octadecadienoate $$ Methyl linoleate $$ M

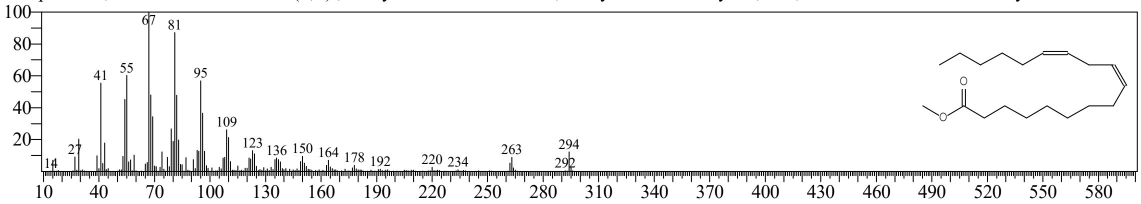

Hit#:5 Entry:137663 Library:NIST17-1.lib
SI:95 Formula:C19H34O2 CAS:2462-85-3 MolWeight:294 RetIndex:2093
CompName:9,12-Octadecadienoic acid, methyl ester $$ 9,12-Octadecenoic acid, methyl ester $$ Methyl octadeca-9,12-dienoate $$ Methyl 9,12-octadecadi

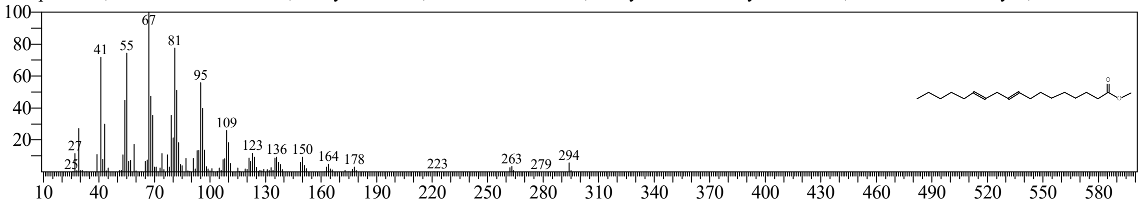

<< Target >>
Line#:12   R.Time:29.267(Scan#:2313) MassPeaks:405
RawMode:Averaged 29.258-29.275(2312-2314) BasePeak:74.00(13491)
BG Mode:Calc. from Peak Group 1 - Event 1

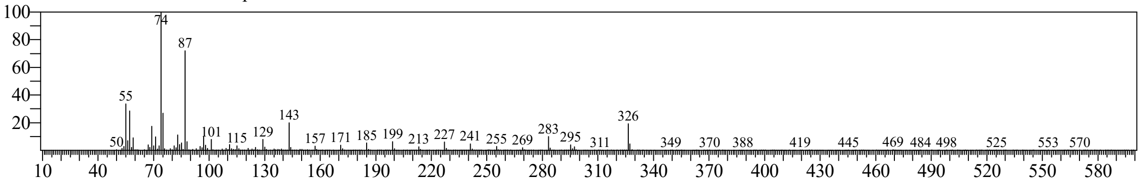

Hit#:1 Entry:154704 Library:NIST14.lib
SI:97 Formula:C21H42O2 CAS:0-00-0 MolWeight:326 RetIndex:2212
CompName:Methyl 18-methylnonadecanoate

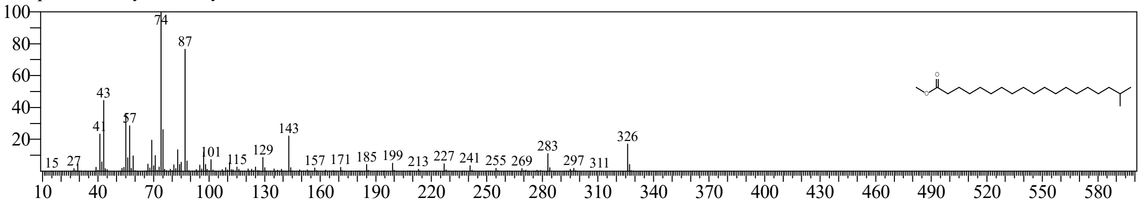

Hit#:2 Entry:170238 Library:NIST17-1.lib
SI:97 Formula:C21H42O2 CAS:0-00-0 MolWeight:326 RetIndex:2212
CompName:Methyl 18-methylnonadecanoate

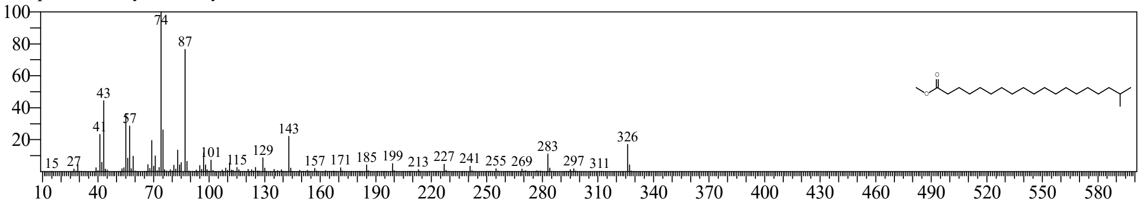

Hit#:3 Entry:34726 Library:NIST17s.lib
SI:96 Formula:C21H42O2 CAS:1120-28-1 MolWeight:326 RetIndex:2276
CompName:Eicosanoic acid, methyl ester $$ Methyl arachisate $$ Methyl eicosanoate $$ Arachidic acid methyl ester $$ Kemester 2050 $$ Methyl aracidat

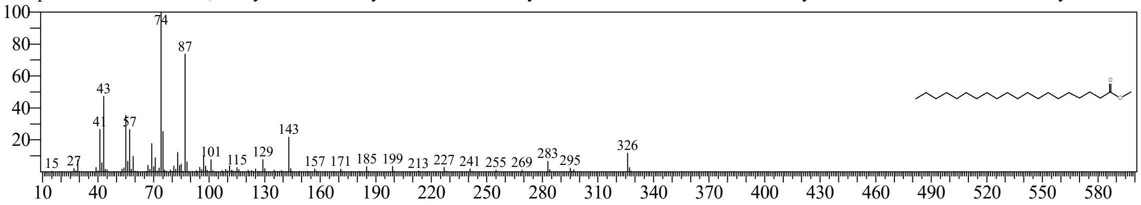

Hit#:4 Entry:34724 Library:NIST17s.lib
SI:93 Formula:C21H42O2 CAS:1120-28-1 MolWeight:326 RetIndex:2276
CompName:Eicosanoic acid, methyl ester $$ Methyl arachisate $$ Methyl eicosanoate $$ Arachidic acid methyl ester $$ Kemester 2050 $$ Methyl aracidat

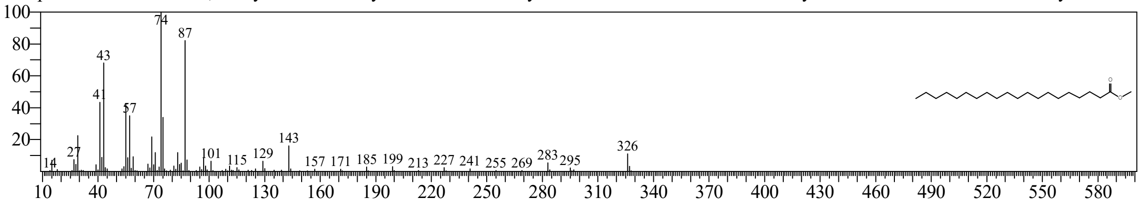

Hit#:5 Entry:29931 Library:NIST14s.lib
SI:93 Formula:C21H42O2 CAS:1120-28-1 MolWeight:326 RetIndex:2276
CompName:Eicosanoic acid, methyl ester $$ Methyl arachisate $$ Methyl eicosanoate $$ Arachidic acid methyl ester $$ Kemester 2050 $$ Methyl aracidat

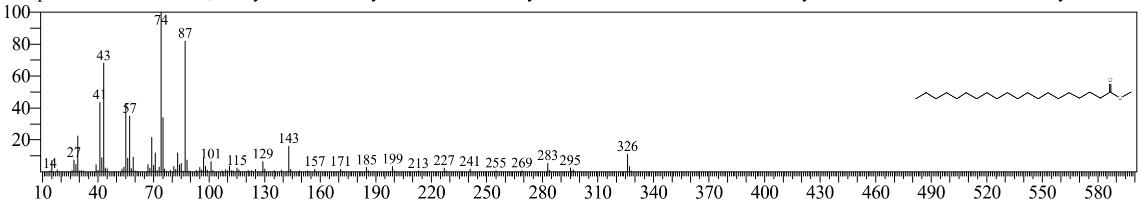

<< Target >>
Line#:13 R.Time:29.817(Scan#:2379) MassPeaks:332
RawMode:Averaged 29.808-29.825(2378-2380) BasePeak:79.05(7304)
BG Mode:Calc. from Peak Group 1 - Event 1

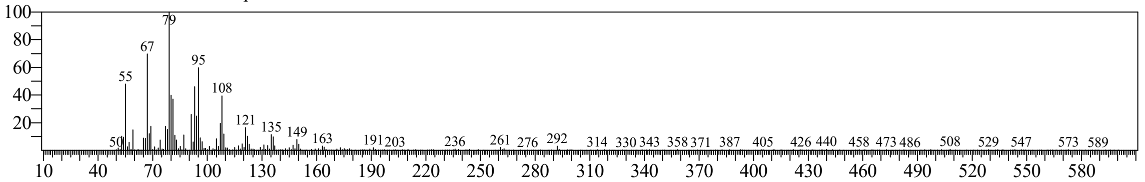

Hit#:1 Entry:135724 Library:NIST17-1.lib
SI:95 Formula:C19H32O2 CAS:301-00-8 MolWeight:292 RetIndex:2101
CompName:9,12,15-Octadecatrienoic acid, methyl ester, (Z,Z,Z)- $$ Linolenic acid, methyl ester $$ Methyl all-cis-9,12,15-octadecatrienoate $$ Methyl lino

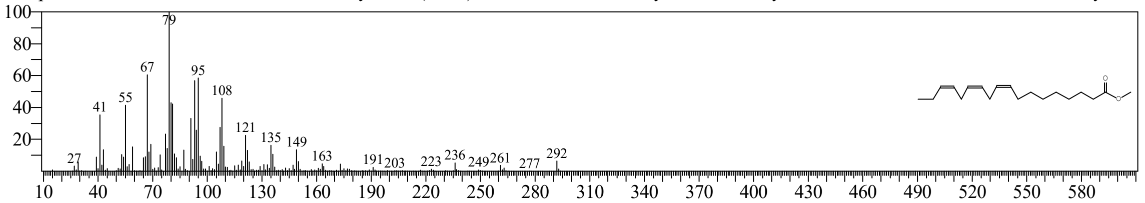

Hit#:2 Entry:124228 Library:NIST14.lib
SI:95 Formula:C19H32O2 CAS:301-00-8 MolWeight:292 RetIndex:2101
CompName:9,12,15-Octadecatrienoic acid, methyl ester, (Z,Z,Z)- $$ Linolenic acid, methyl ester $$ Methyl all-cis-9,12,15-octadecatrienoate $$ Methyl lino

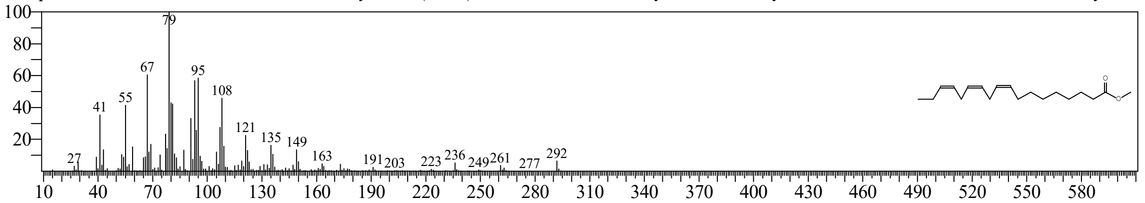

Hit#:3 Entry:27872 Library:NIST14s.lib
SI:95 Formula:C19H32O2 CAS:301-00-8 MolWeight:292 RetIndex:2101
CompName:9,12,15-Octadecatrienoic acid, methyl ester, (Z,Z,Z)- $$ Linolenic acid, methyl ester $$ Methyl all-cis-9,12,15-octadecatrienoate $$ Methyl lino

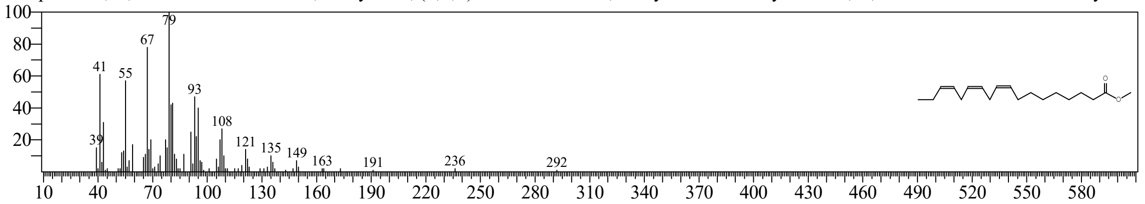

Hit#:4 Entry:32437 Library:NIST17s.lib
SI:95 Formula:C19H32O2 CAS:301-00-8 MolWeight:292 RetIndex:2101
CompName:9,12,15-Octadecatrienoic acid, methyl ester, (Z,Z,Z)- $$ Linolenic acid, methyl ester $$ Methyl all-cis-9,12,15-octadecatrienoate $$ Methyl lino

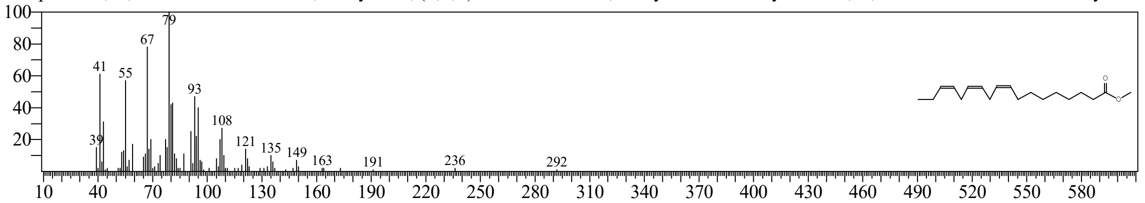

Hit#:5 Entry:32438 Library:NIST17s.lib
SI:95 Formula:C19H32O2 CAS:301-00-8 MolWeight:292 RetIndex:2101
CompName:9,12,15-Octadecatrienoic acid, methyl ester, (Z,Z,Z)- $$ Linolenic acid, methyl ester $$ Methyl all-cis-9,12,15-octadecatrienoate $$ Methyl lino

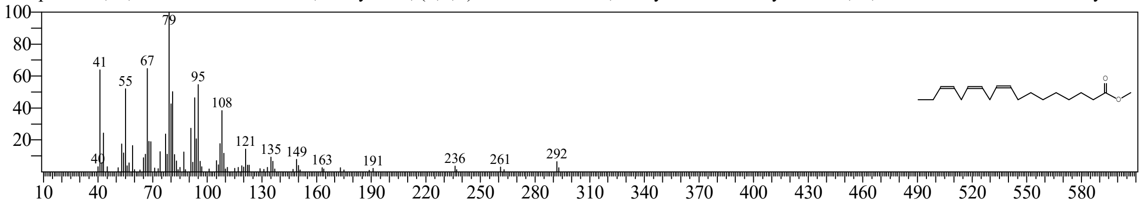

<< Target >>
Line#:14 R.Time:30.292(Scan#:2436) MassPeaks:346
RawMode:Averaged 30.283-30.300(2435-2437) BasePeak:79.05(5796)
BG Mode:Calc. from Peak Group 1 - Event 1

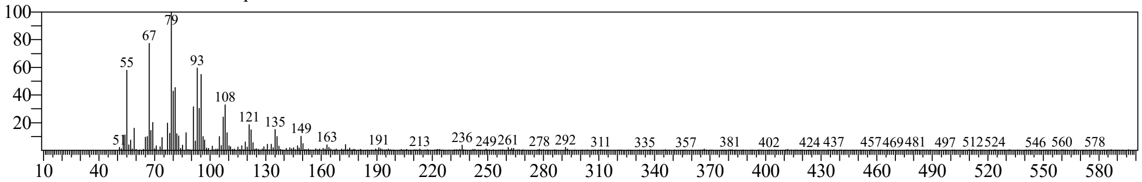

Hit#:1 Entry:124228 Library:NIST14.lib
SI:96 Formula:C19H32O2 CAS:301-00-8 MolWeight:292 RetIndex:2101
CompName:9,12,15-Octadecatrienoic acid, methyl ester, (Z,Z,Z)- $$ Linolenic acid, methyl ester $$ Methyl all-cis-9,12,15-octadecatrienoate $$ Methyl lino

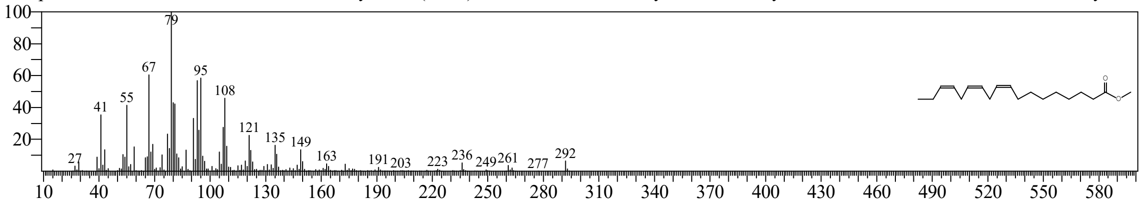

Hit#:2 Entry:135724 Library:NIST17-1.lib
SI:96 Formula:C19H32O2 CAS:301-00-8 MolWeight:292 RetIndex:2101
CompName:9,12,15-Octadecatrienoic acid, methyl ester, (Z,Z,Z)- $$ Linolenic acid, methyl ester $$ Methyl all-cis-9,12,15-octadecatrienoate $$ Methyl lino

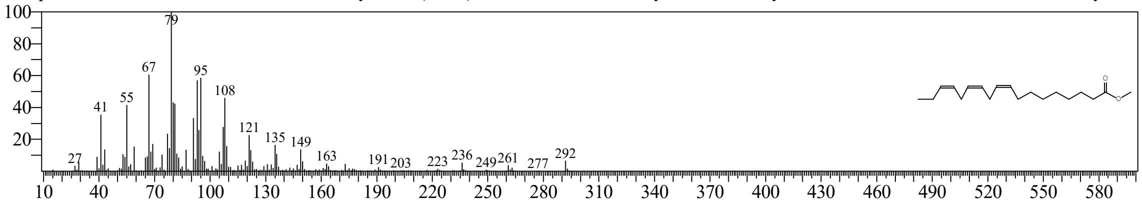

Hit#:3 Entry:27872 Library:NIST14s.lib
SI:95 Formula:C19H32O2 CAS:301-00-8 MolWeight:292 RetIndex:2101
CompName:9,12,15-Octadecatrienoic acid, methyl ester, (Z,Z,Z)- $$ Linolenic acid, methyl ester $$ Methyl all-cis-9,12,15-octadecatrienoate $$ Methyl lino

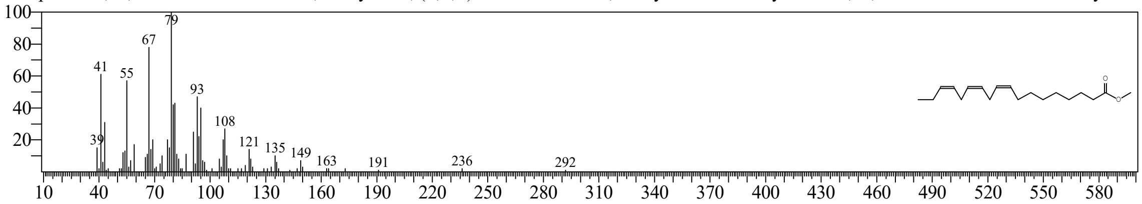

Hit#:4 Entry:111654 Library:NIST14.lib
SI:95 Formula:C18H30O2 CAS:0-00-0 MolWeight:278 RetIndex:2002
CompName:Methyl 8,11,14-heptadecatrienoate

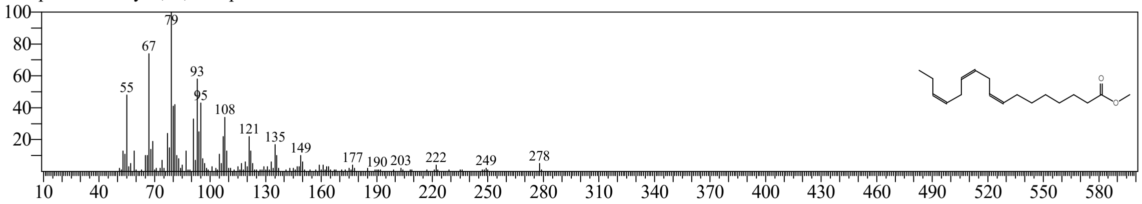

Hit#:5 Entry:32437 Library:NIST17s.lib
SI:95 Formula:C19H32O2 CAS:301-00-8 MolWeight:292 RetIndex:2101
CompName:9,12,15-Octadecatrienoic acid, methyl ester, (Z,Z,Z)- $$ Linolenic acid, methyl ester $$ Methyl all-cis-9,12,15-octadecatrienoate $$ Methyl lino

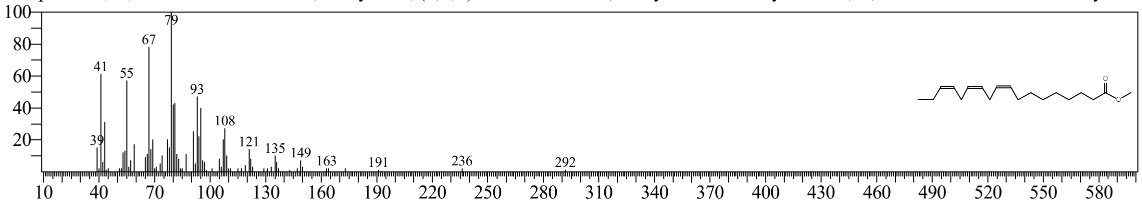

<< Target >>
Line#:15   R.Time:30.600(Scan#:2473) MassPeaks:380
RawMode:Averaged 30.592-30.608(2472-2474) BasePeak:79.05(61758)
BG Mode:Calc. from Peak Group 1 - Event 1

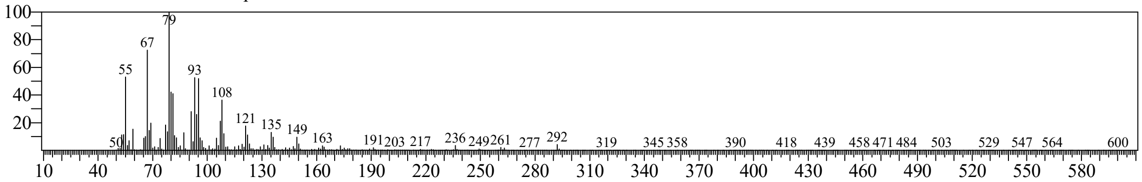

Hit#:1 Entry:135724 Library:NIST17-1.lib
SI:96 Formula:C19H32O2 CAS:301-00-8 MolWeight:292 RetIndex:2101
CompName:9,12,15-Octadecatrienoic acid, methyl ester, (Z,Z,Z)- $$ Linolenic acid, methyl ester $$ Methyl all-cis-9,12,15-octadecatrienoate $$ Methyl lino

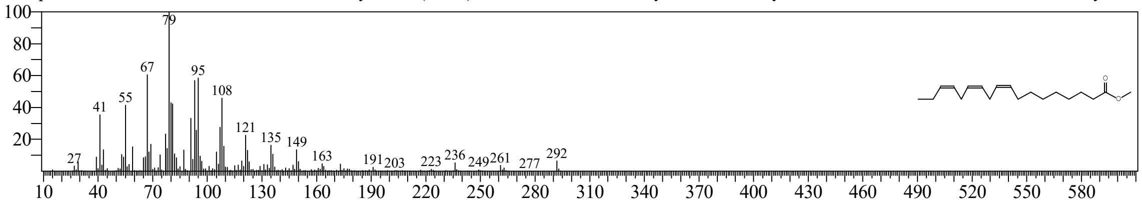

Hit#:2 Entry:124228 Library:NIST14.lib
SI:96 Formula:C19H32O2 CAS:301-00-8 MolWeight:292 RetIndex:2101
CompName:9,12,15-Octadecatrienoic acid, methyl ester, (Z,Z,Z)- $$ Linolenic acid, methyl ester $$ Methyl all-cis-9,12,15-octadecatrienoate $$ Methyl lino

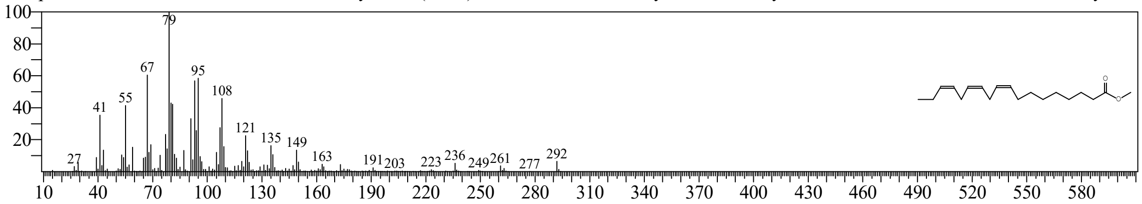

Hit#:3 Entry:32438 Library:NIST17s.lib
SI:95 Formula:C19H32O2 CAS:301-00-8 MolWeight:292 RetIndex:2101
CompName:9,12,15-Octadecatrienoic acid, methyl ester, (Z,Z,Z)- $$ Linolenic acid, methyl ester $$ Methyl all-cis-9,12,15-octadecatrienoate $$ Methyl lino

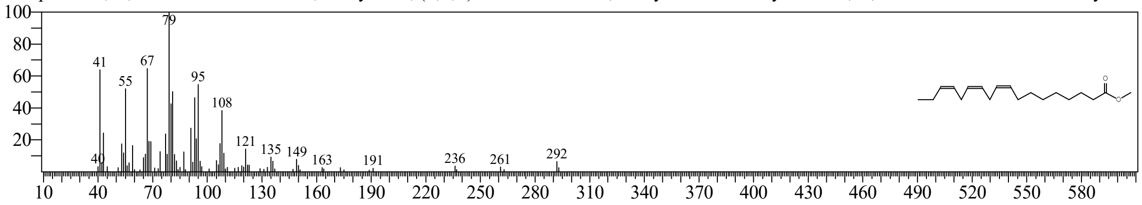

Hit#:4 Entry:27872 Library:NIST14s.lib
SI:95 Formula:C19H32O2 CAS:301-00-8 MolWeight:292 RetIndex:2101
CompName:9,12,15-Octadecatrienoic acid, methyl ester, (Z,Z,Z)- $$ Linolenic acid, methyl ester $$ Methyl all-cis-9,12,15-octadecatrienoate $$ Methyl lino

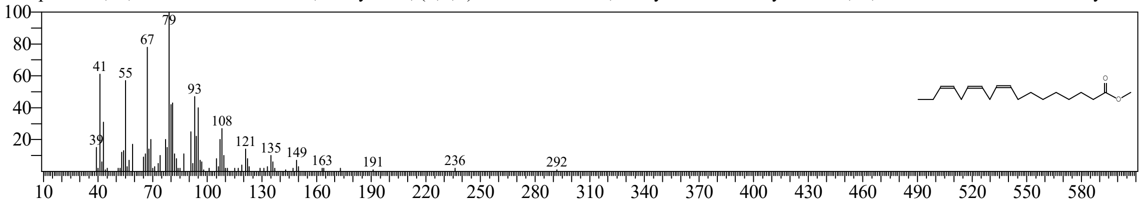

Hit#:5 Entry:32437 Library:NIST17s.lib
SI:95 Formula:C19H32O2 CAS:301-00-8 MolWeight:292 RetIndex:2101
CompName:9,12,15-Octadecatrienoic acid, methyl ester, (Z,Z,Z)- $$ Linolenic acid, methyl ester $$ Methyl all-cis-9,12,15-octadecatrienoate $$ Methyl lino

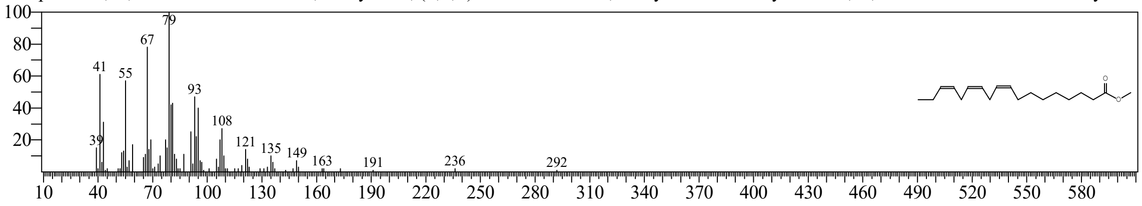

<< Target >>
Line#:16 R.Time:30.925(Scan#:2512) MassPeaks:381
RawMode:Averaged 30.917-30.933(2511-2513) BasePeak:67.05(17426)
BG Mode:Calc. from Peak Group 1 - Event 1

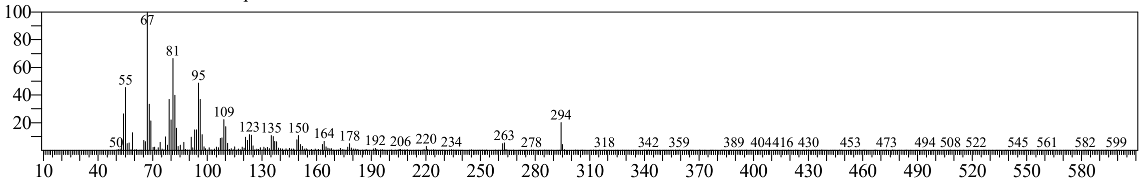

Hit#:1 Entry:137666 Library:NIST17-1.lib
SI:97 Formula:C19H34O2 CAS:13058-52-1 MolWeight:294 RetIndex:2093
CompName:Methyl 9-cis,11-trans-octadecadienoate

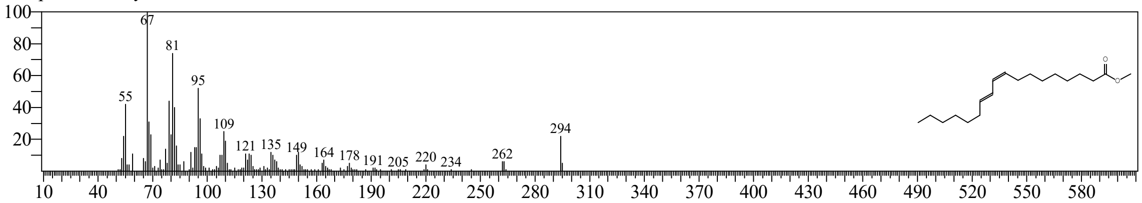

Hit#:2 Entry:125932 Library:NIST14.lib
SI:97 Formula:C19H34O2 CAS:0-00-0 MolWeight:294 RetIndex:2093
CompName:Methyl 9-cis,11-trans-octadecadienoate

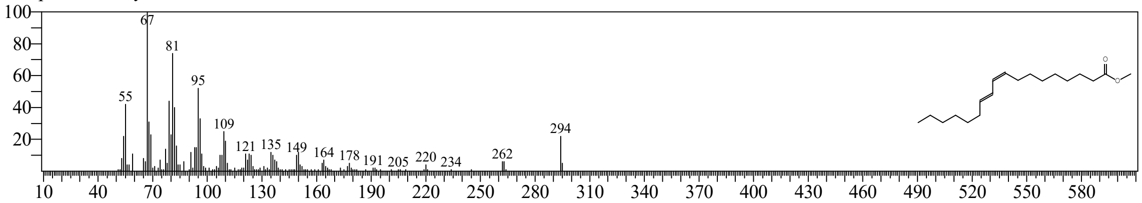

Hit#:3 Entry:125933 Library:NIST14.lib
SI:97 Formula:C19H34O2 CAS:0-00-0 MolWeight:294 RetIndex:2093
CompName:Methyl 10-trans,12-cis-octadecadienoate

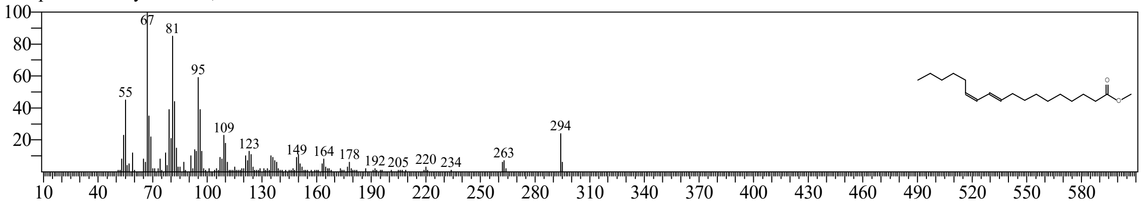

Hit#:4 Entry:137664 Library:NIST17-1.lib
SI:97 Formula:C19H34O2 CAS:0-00-0 MolWeight:294 RetIndex:2093
CompName:Methyl 10-trans,12-cis-octadecadienoate

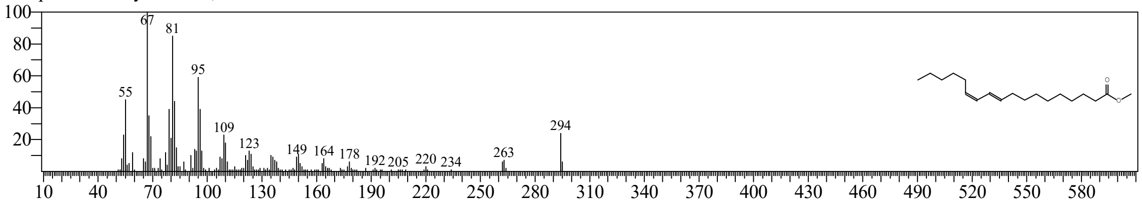

Hit#:5 Entry:32587 Library:NIST17s.lib
SI:96 Formula:C19H34O2 CAS:13038-47-6 MolWeight:294 RetIndex:2093
CompName:9,11-Octadecadienoic acid, methyl ester, (E,E)- $$ Methyl trans-9,trans-11-octadecadienoate $$ Methyl trans,trans-9,11-octadecadienoate $$ M

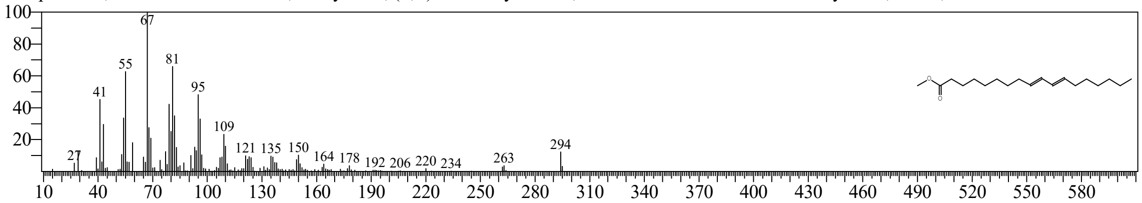

<< Target >>
Line#:17 R.Time:31.258(Scan#:2552) MassPeaks:389
RawMode:Averaged 31.250-31.267(2551-2553) BasePeak:67.05(19000)
BG Mode:Calc. from Peak Group 1 - Event 1

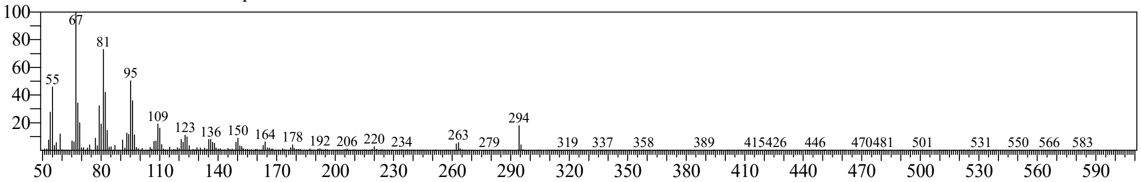

Hit#:1 Entry:125933 Library:NIST14.lib
SI:96 Formula:C19H34O2 CAS:0-00-0 MolWeight:294 RetIndex:2093
CompName:Methyl 10-trans,12-cis-octadecadienoate

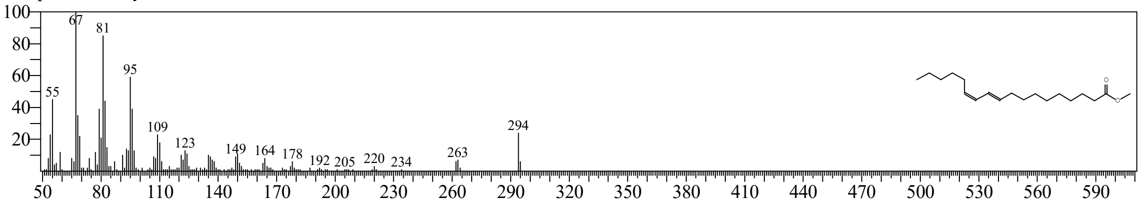

Hit#:2 Entry:137664 Library:NIST17-1.lib
SI:96 Formula:C19H34O2 CAS:0-00-0 MolWeight:294 RetIndex:2093
CompName:Methyl 10-trans,12-cis-octadecadienoate

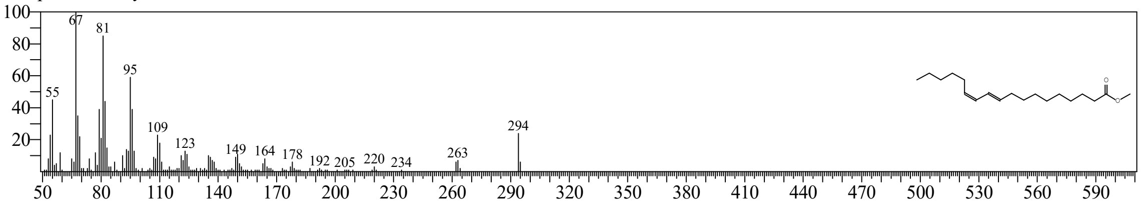

Hit#:3 Entry:125932 Library:NIST14.lib
SI:96 Formula:C19H34O2 CAS:0-00-0 MolWeight:294 RetIndex:2093
CompName:Methyl 9-cis,11-trans-octadecadienoate

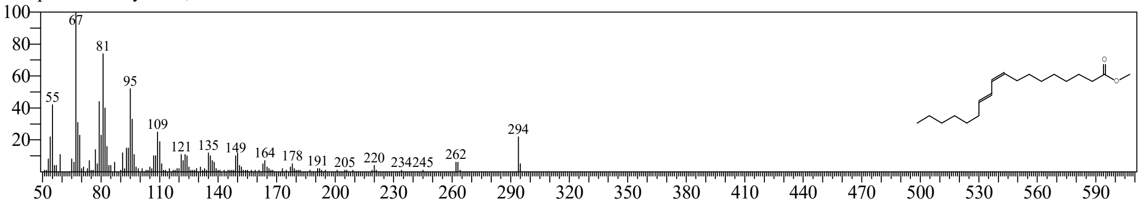

Hit#:4 Entry:137666 Library:NIST17-1.lib
SI:96 Formula:C19H34O2 CAS:13058-52-1 MolWeight:294 RetIndex:2093
CompName:Methyl 9-cis,11-trans-octadecadienoate

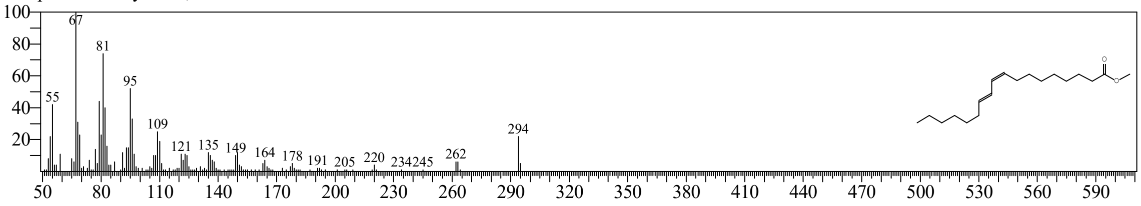

Hit#:5 Entry:28003 Library:NIST14s.lib
SI:95 Formula:C19H34O2 CAS:57156-97-5 MolWeight:294 RetIndex:2093
CompName:12,15-Octadecadienoic acid, methyl ester $$ Methyl (12E,15E)-12,15-octadecadienoate # $$

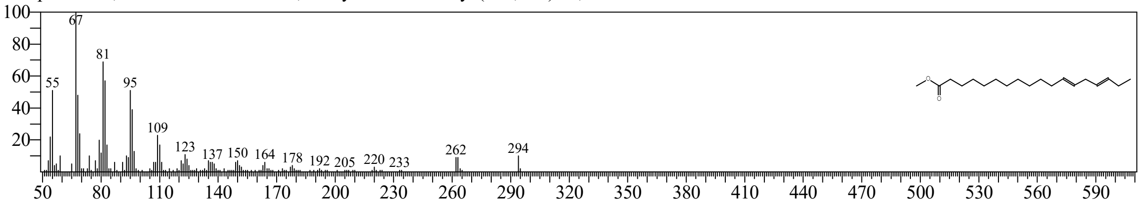

<< Target >>
Line#:18   R.Time:31.917(Scan#:2631) MassPeaks:352
RawMode:Averaged 31.908-31.925(2630-2632) BasePeak:67.05(1036)
BG Mode:Calc. from Peak Group 1 - Event 1

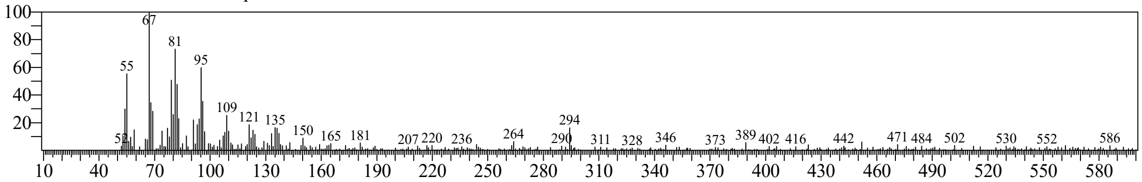

Hit#:1 Entry:32587 Library:NIST17s.lib
SI:90 Formula:C19H34O2 CAS:13038-47-6 MolWeight:294 RetIndex:2093
CompName:9,11-Octadecadienoic acid, methyl ester, (E,E)- $$ Methyl trans-9,trans-11-octadecadienoate $$ Methyl trans,trans-9,11-octadecadienoate $$ M

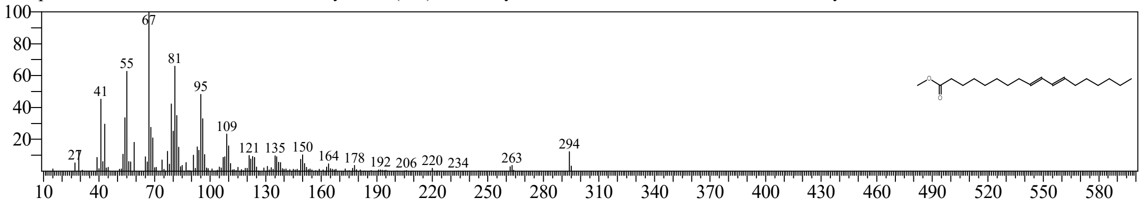

Hit#:2 Entry:125935 Library:NIST14.lib
SI:90 Formula:C19H34O2 CAS:112-63-0 MolWeight:294 RetIndex:2093
CompName:9,12-Octadecadienoic acid (Z,Z)-, methyl ester $$ Linoleic acid, methyl ester $$ Methyl cis,cis-9,12-octadecadienoate $$ Methyl linoleate $$ M

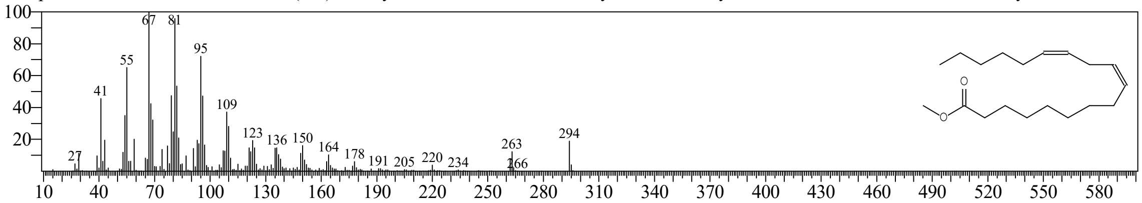

Hit#:3 Entry:137663 Library:NIST17-1.lib
SI:90 Formula:C19H34O2 CAS:2462-85-3 MolWeight:294 RetIndex:2093
CompName:9,12-Octadecadienoic acid, methyl ester $$ 9,12-Octadecenoic acid, methyl ester $$ Methyl octadeca-9,12-dienoate $$ Methyl 9,12-octadecadi

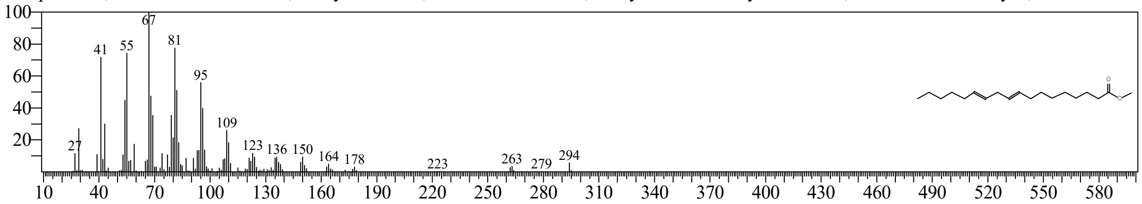

Hit#:4 Entry:137667 Library:NIST17-1.lib
SI:90 Formula:C19H34O2 CAS:112-63-0 MolWeight:294 RetIndex:2093
CompName:9,12-Octadecadienoic acid (Z,Z)-, methyl ester $$ Linoleic acid, methyl ester $$ Methyl cis,cis-9,12-octadecadienoate $$ Methyl linoleate $$ M

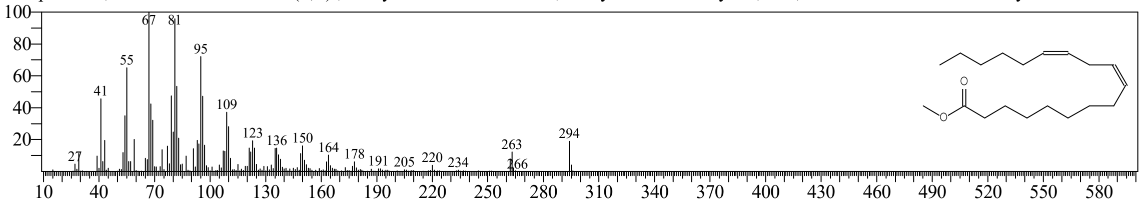

Hit#:5 Entry:125931 Library:NIST14.lib
SI:90 Formula:C19H34O2 CAS:2462-85-3 MolWeight:294 RetIndex:2093
CompName:9,12-Octadecadienoic acid, methyl ester $$ 9,12-Octadecenoic acid, methyl ester $$ Methyl octadeca-9,12-dienoate $$ Methyl 9,12-octadecadi

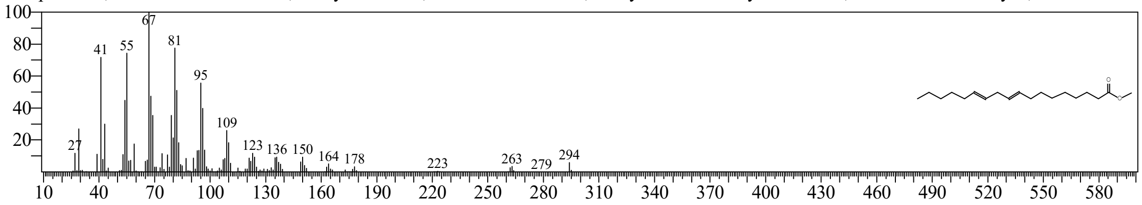

<< Target >>
Line#:19 R.Time:32.200(Scan#:2665) MassPeaks:362
RawMode:Averaged 32.192-32.208(2664-2666) BasePeak:79.05(583)
BG Mode:Calc. from Peak Group 1 - Event 1

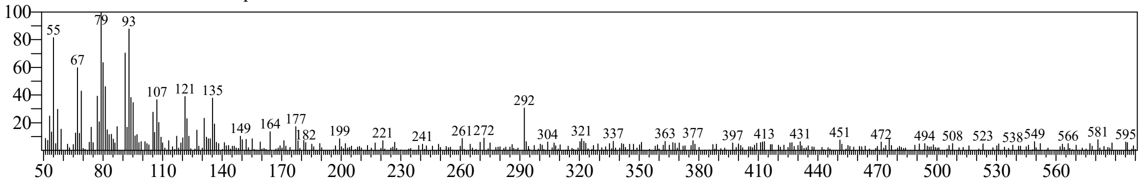

Hit#:1 Entry:135726 Library:NIST17-1.lib
SI:85 Formula:C19H32O2 CAS:0-00-0 MolWeight:292 RetIndex:2101
CompName:Methyl 9.cis.,11.trans.t,13.trans.-octadecatrienoate

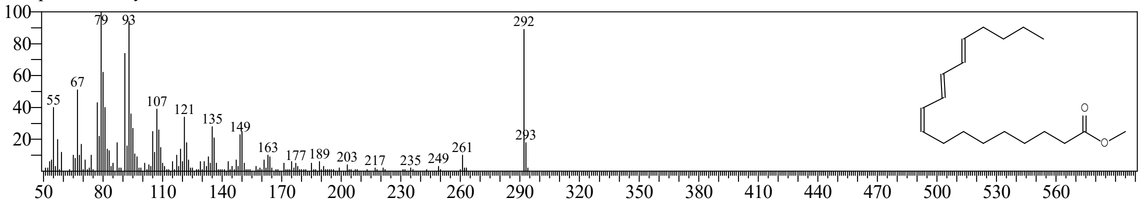

Hit#:2 Entry:124230 Library:NIST14.lib
SI:85 Formula:C19H32O2 CAS:0-00-0 MolWeight:292 RetIndex:2101
CompName:Methyl 9.cis.,11.trans.t,13.trans.-octadecatrienoate

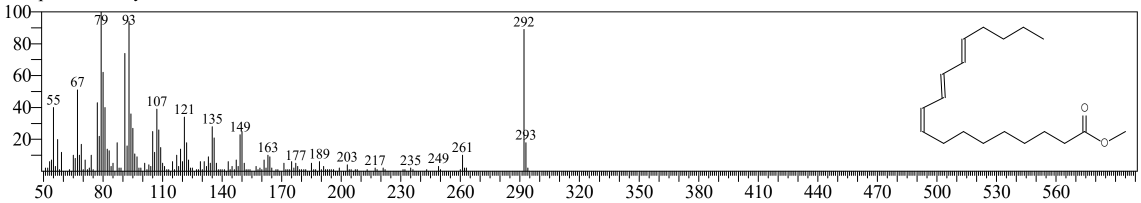

Hit#:3 Entry:164437 Library:NIST17-1.lib
SI:83 Formula:C21H36O2 CAS:0-00-0 MolWeight:320 RetIndex:2301
CompName:Methyl eicos-11-en-14-ynoate

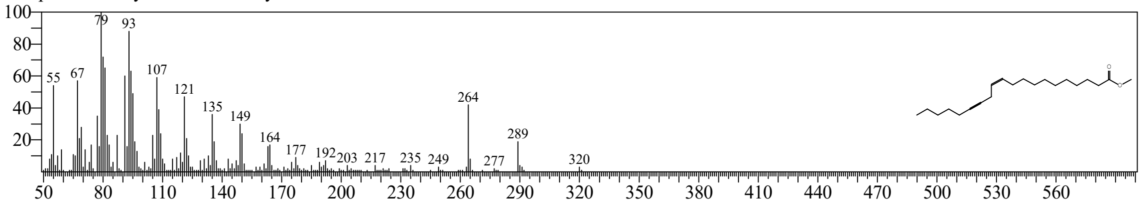

Hit#:4 Entry:149536 Library:NIST14.lib
SI:83 Formula:C21H36O2 CAS:0-00-0 MolWeight:320 RetIndex:2301
CompName:Methyl eicos-11-en-14-ynoate

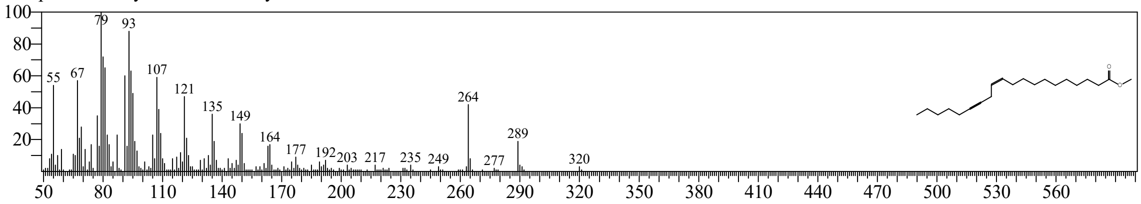

Hit#:5 Entry:124226 Library:NIST14.lib
SI:82 Formula:C19H32O2 CAS:0-00-0 MolWeight:292 RetIndex:2101
CompName:Methyl 6-cis,9-cis,11-trans-octadecatrienoate

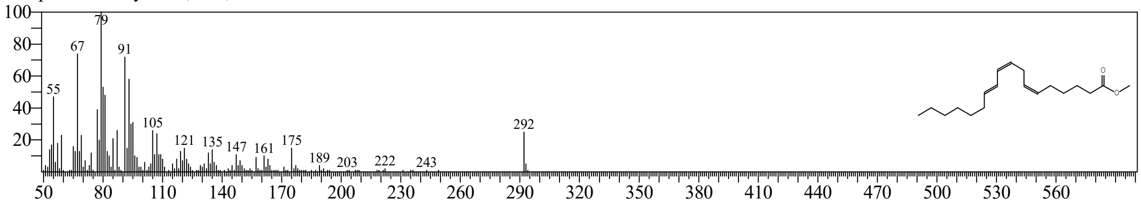

<< Target >>
Line#:20 R.Time:32.958(Scan#:2756) MassPeaks:391
RawMode:Averaged 32.950-32.967(2755-2757) BasePeak:95.10(20393)
BG Mode:Calc. from Peak Group 1 - Event 1

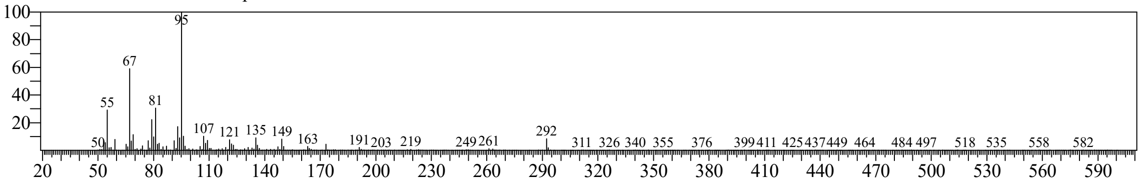

Hit#:1 Entry:23403 Library:NIST14.lib
SI:86 Formula:C12H20 CAS:0-00-0 MolWeight:164 RetIndex:1041
CompName:Cis-3-ethyl-endo-tricyclo[5.2.1.0(2.6)]decane

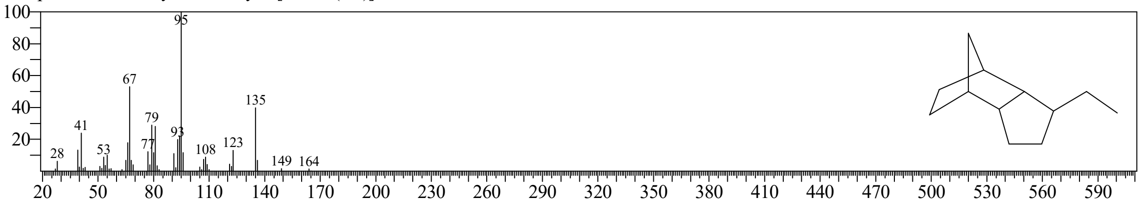

Hit#:2 Entry:24424 Library:NIST17-1.lib
SI:86 Formula:C12H20 CAS:0-00-0 MolWeight:164 RetIndex:1041
CompName:Cis-3-ethyl-endo-tricyclo[5.2.1.0(2.6)]decane

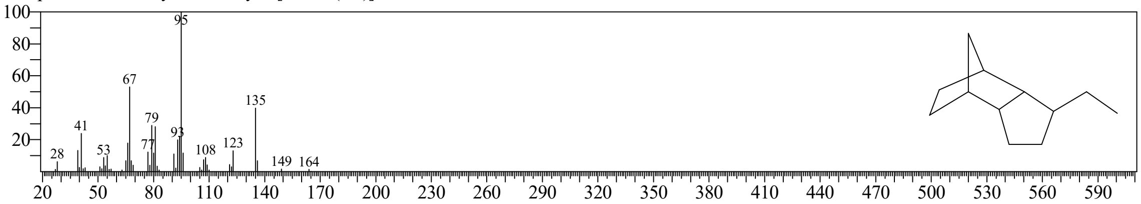

Hit#:3 Entry:16582 Library:NIST17-1.lib
SI:84 Formula:C11H18 CAS:0-00-0 MolWeight:150 RetIndex:1115
CompName:4-Cyclohexenyl-3-methylbutene

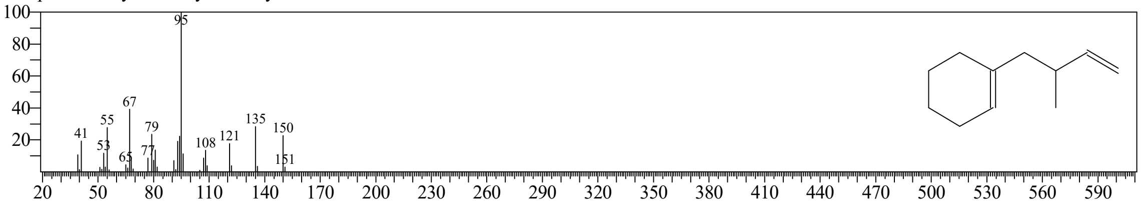

Hit#:4 Entry:17571 Library:NIST17-1.lib
SI:83 Formula:C10H16O CAS:60485-45-2 MolWeight:152 RetIndex:991
CompName:Santolina epoxide $$ 2-Methyl-2-(3-methyl-1-vinyl-2-butenyl)oxirane # $$

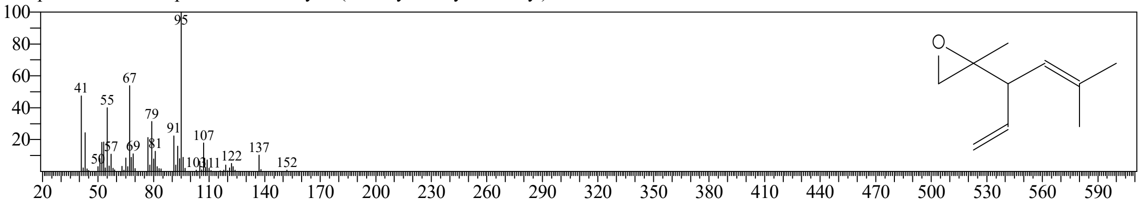

Hit#:5 Entry:16897 Library:NIST14.lib
SI:83 Formula:C10H16O CAS:60485-45-2 MolWeight:152 RetIndex:991
CompName:Santolina epoxide $$ 2-Methyl-2-(3-methyl-1-vinyl-2-butenyl)oxirane # $$

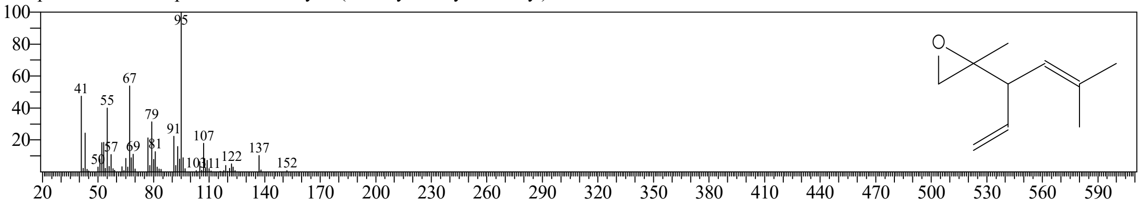

<< Target >>
Line#:21 R.Time:33.492(Scan#:2820) MassPeaks:399
RawMode:Averaged 33.483-33.500(2819-2821) BasePeak:74.00(15735)
BG Mode:Calc. from Peak Group 1 - Event 1

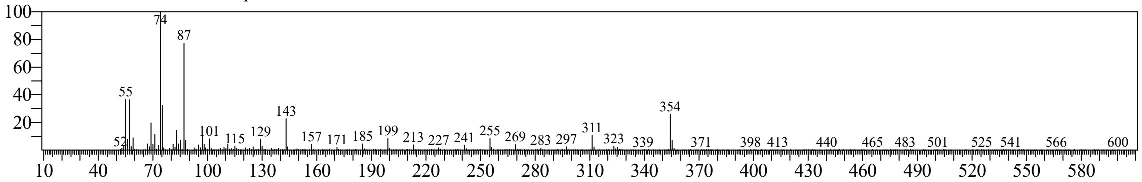

Hit#:1 Entry:196443 Library:NIST17-1.lib
SI:96 Formula:C23H46O2 CAS:929-77-1 MolWeight:354 RetIndex:2475
CompName:Docosanoic acid, methyl ester $$ Behenic acid, methyl ester $$ Methyl behenate $$ Methyl docosanoate $$ n-Docosanoic acid methyl ester $$ [illegible]

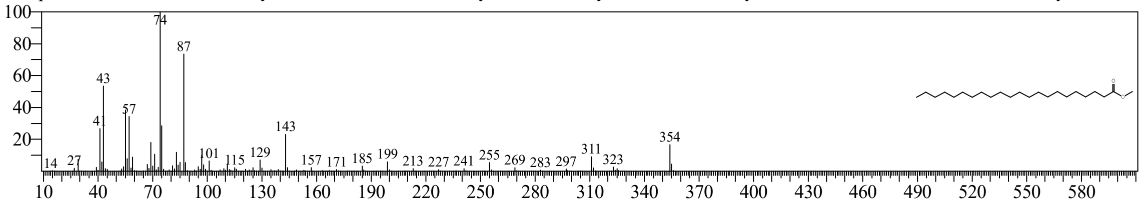

Hit#:2 Entry:178051 Library:NIST14.lib
SI:96 Formula:C23H46O2 CAS:929-77-1 MolWeight:354 RetIndex:2475
CompName:Docosanoic acid, methyl ester $$ Behenic acid, methyl ester $$ Methyl behenate $$ Methyl docosanoate $$ n-Docosanoic acid methyl ester $$ [illegible]

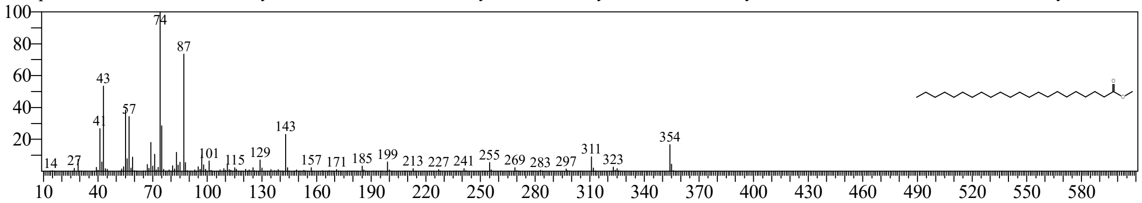

Hit#:3 Entry:196444 Library:NIST17-1.lib
SI:95 Formula:C23H46O2 CAS:0-00-0 MolWeight:354 RetIndex:2411
CompName:Methyl 20-methyl-heneicosanoate

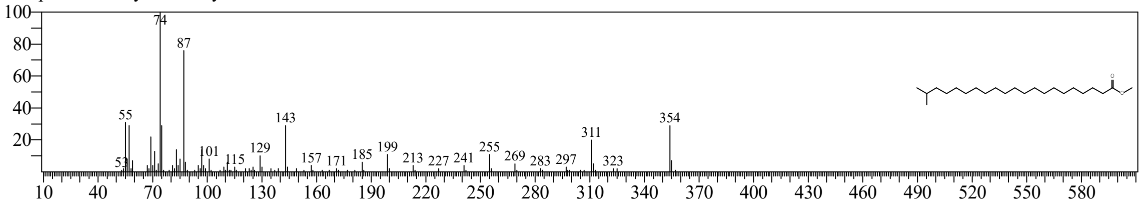

Hit#:4 Entry:178052 Library:NIST14.lib
SI:95 Formula:C23H46O2 CAS:0-00-0 MolWeight:354 RetIndex:2411
CompName:Methyl 20-methyl-heneicosanoate

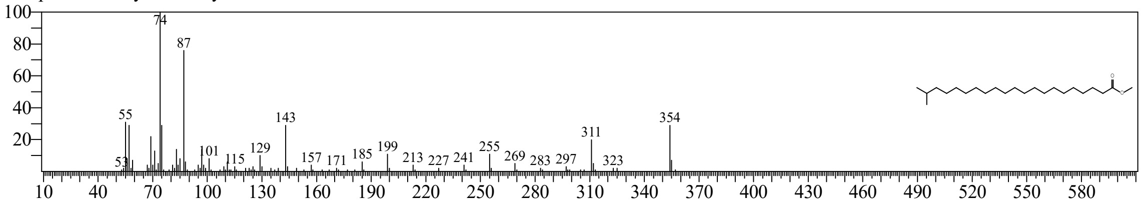

Hit#:5 Entry:31088 Library:NIST14s.lib
SI:94 Formula:C23H46O2 CAS:929-77-1 MolWeight:354 RetIndex:2475
CompName:Docosanoic acid, methyl ester $$ Behenic acid, methyl ester $$ Methyl behenate $$ Methyl docosanoate $$ n-Docosanoic acid methyl ester $$ [illegible]

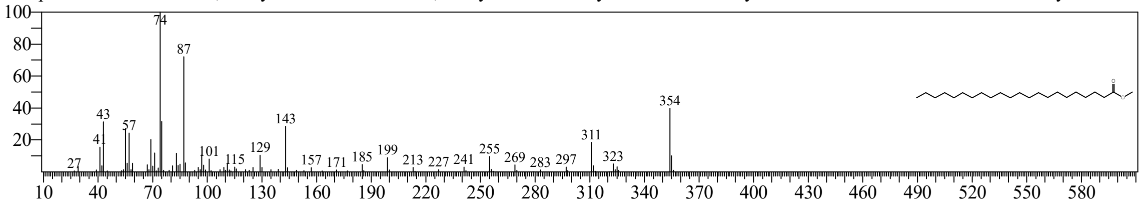

<< Target >>
Line#:22 R.Time:35.592(Scan#:3072) MassPeaks:378
RawMode:Averaged 35.583-35.600(3071-3073) BasePeak:74.05(1245)
BG Mode:Calc. from Peak Group 1 - Event 1

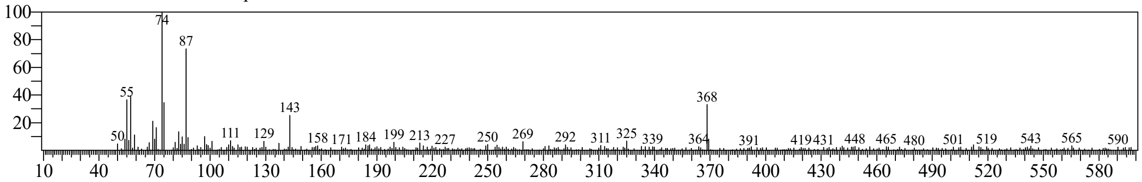

Hit#:1 Entry:36674 Library:NIST17s.lib
SI:88 Formula:C24H48O2 CAS:2433-97-8 MolWeight:368 RetIndex:2574
CompName:Tricosanoic acid, methyl ester $$ Methyl tricosanoate $$

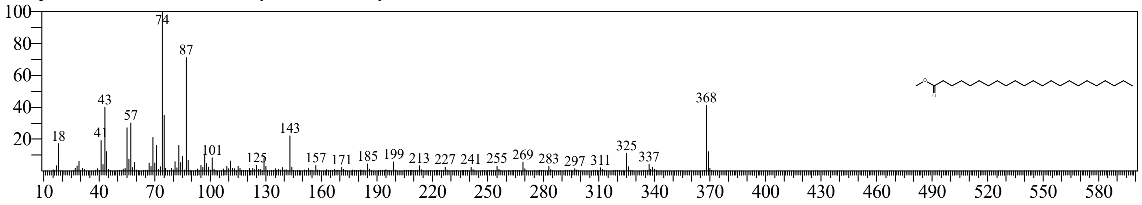

Hit#:2 Entry:31595 Library:NIST14s.lib
SI:88 Formula:C24H48O2 CAS:2433-97-8 MolWeight:368 RetIndex:2574
CompName:Tricosanoic acid, methyl ester $$ Methyl tricosanoate $$

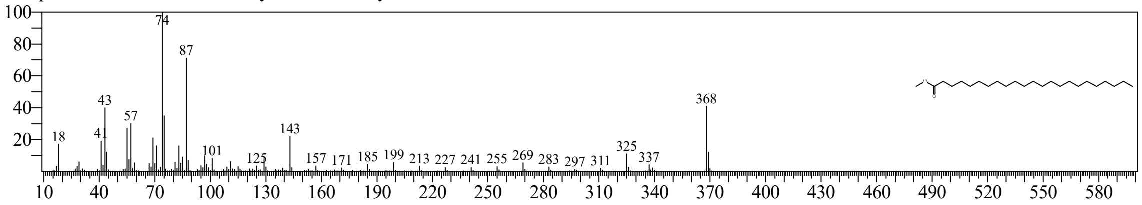

Hit#:3 Entry:187843 Library:NIST14.lib
SI:87 Formula:C24H48O2 CAS:2433-97-8 MolWeight:368 RetIndex:2574
CompName:Tricosanoic acid, methyl ester $$ Methyl tricosanoate $$

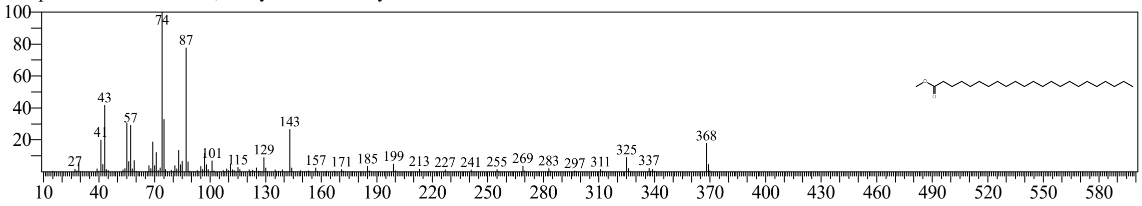

Hit#:4 Entry:207309 Library:NIST17-1.lib
SI:87 Formula:C24H48O2 CAS:2433-97-8 MolWeight:368 RetIndex:2574
CompName:Tricosanoic acid, methyl ester $$ Methyl tricosanoate $$

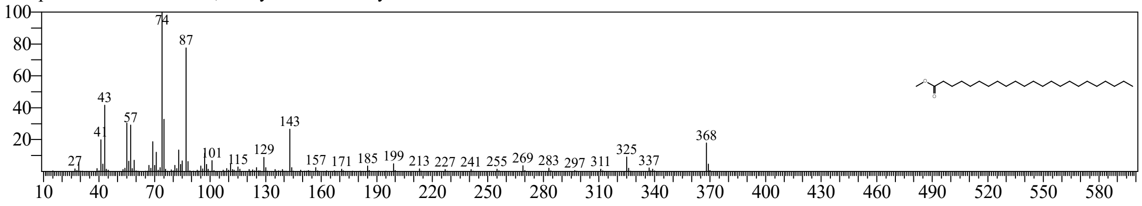

Hit#:5 Entry:31594 Library:NIST14s.lib
SI:86 Formula:C24H48O2 CAS:2433-97-8 MolWeight:368 RetIndex:2574
CompName:Tricosanoic acid, methyl ester $$ Methyl tricosanoate $$

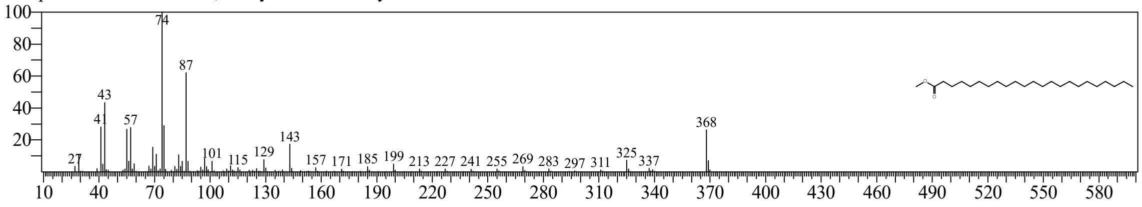

<< Target >>
Line#:23 R.Time:37.808(Scan#:3338) MassPeaks:352
RawMode:Averaged 37.800-37.817(3337-3339) BasePeak:74.00(3890)
BG Mode:Calc. from Peak Group 1 - Event 1

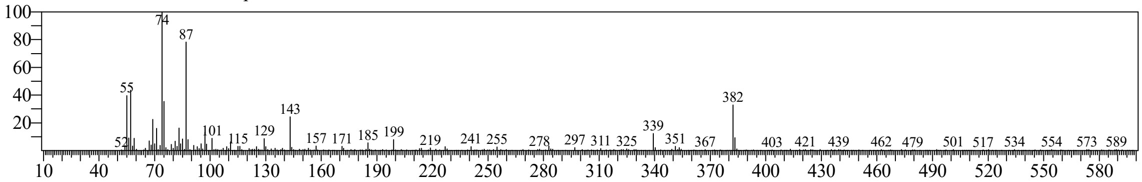

Hit#:1 Entry:31970 Library:NIST14s.lib
SI:94 Formula:C25H50O2 CAS:2442-49-1 MolWeight:382 RetIndex:2674
CompName:Tetracosanoic acid, methyl ester $$ Methyl lignocerate $$ Methyl tetracosanoate $$ Lignoceric acid methyl ester $$

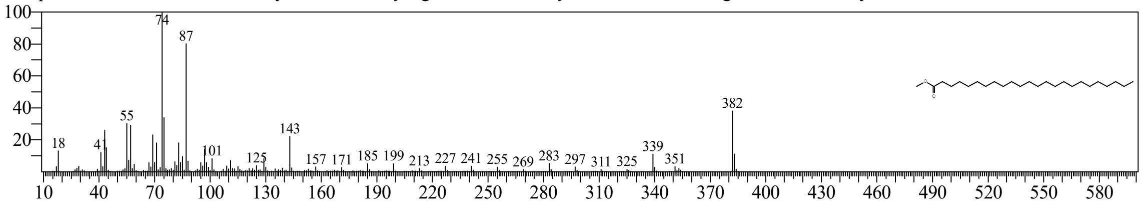

Hit#:2 Entry:37106 Library:NIST17s.lib
SI:94 Formula:C25H50O2 CAS:2442-49-1 MolWeight:382 RetIndex:2674
CompName:Tetracosanoic acid, methyl ester $$ Methyl lignocerate $$ Methyl tetracosanoate $$ Lignoceric acid methyl ester $$

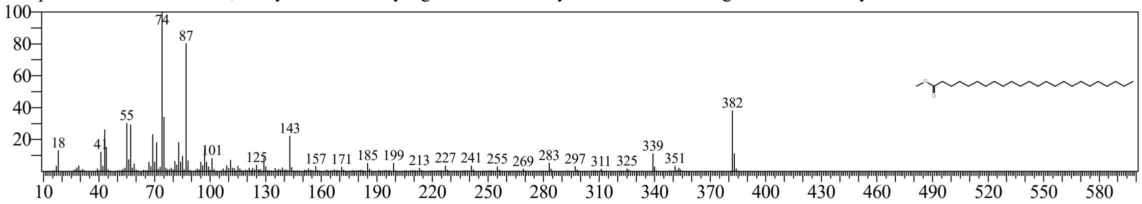

Hit#:3 Entry:31971 Library:NIST14s.lib
SI:92 Formula:C25H50O2 CAS:2442-49-1 MolWeight:382 RetIndex:2674
CompName:Tetracosanoic acid, methyl ester $$ Methyl lignocerate $$ Methyl tetracosanoate $$ Lignoceric acid methyl ester $$

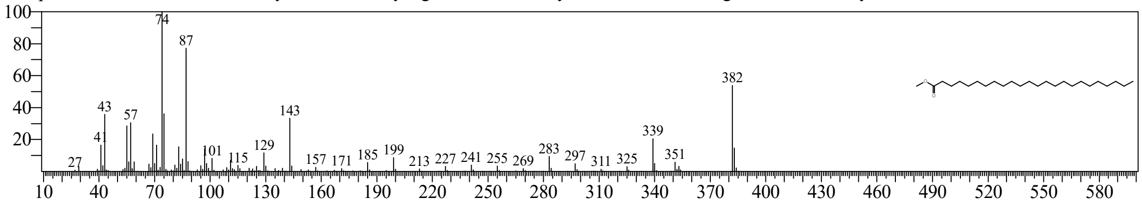

Hit#:4 Entry:37107 Library:NIST17s.lib
SI:92 Formula:C25H50O2 CAS:2442-49-1 MolWeight:382 RetIndex:2674
CompName:Tetracosanoic acid, methyl ester $$ Methyl lignocerate $$ Methyl tetracosanoate $$ Lignoceric acid methyl ester $$

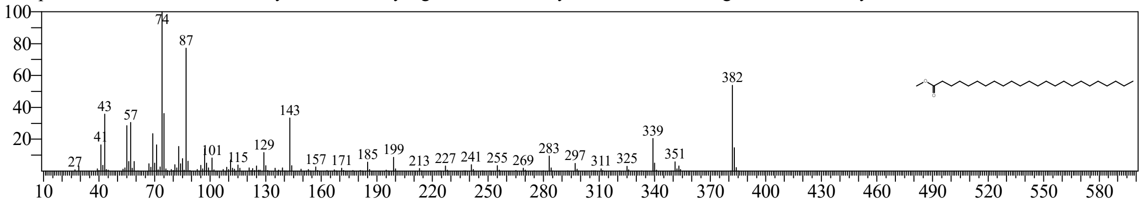

Hit#:5 Entry:37105 Library:NIST17s.lib
SI:91 Formula:C25H50O2 CAS:2442-49-1 MolWeight:382 RetIndex:2674
CompName:Tetracosanoic acid, methyl ester $$ Methyl lignocerate $$ Methyl tetracosanoate $$ Lignoceric acid methyl ester $$

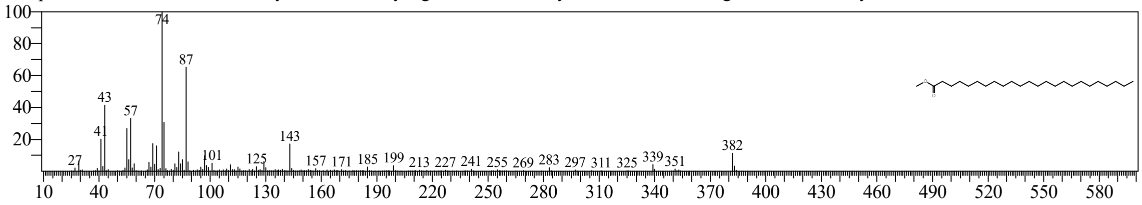

Supplement: Supplementary file 1 [file molecules-31-02891-s001.zip › 14d-QG100-1.pdf]
