# Supplementary material for: Selective Inhibition of Lipid Oxidation by a Quercetagetin-Rich Extract from Tagetes erecta: A Chemometric Approach Using OPLS-DA and Selectivity Index Analysis
Source: Molecules. 2026 Aug 19;31(16):2891. doi: 10.3390/molecules31162891 (PMC13515886; doi:10.3390/molecules31162891)

QG200-1-14d

<<Column>>
Name : HP-88
Thickness : 0.20um
Length : 100.0m
Inside Diameter : 0.25mm

Method

===== Analytical Line 1 =====

| | | |
|---|---|---|
| Column Oven Temp. | :120.0 °C | |
| Injection Temp. | :250.00 °C | |
| Injection Mode | :Split | |
| Column Flow | :1.04 mL/min | |
| Split Ratio | :20.0 | |
| Oven Temp. Program | | |
| Rate | Temperature(°C) | Hold Time(min) |
| - | 120.0 | 1.00 |
| 10.00 | 175.0 | 10.00 |
| 5.00 | 210.0 | 5.00 |
| 5.00 | 230.0 | 15.00 |

[GCMS-QP2010]
IonSourceTemp :200.00 °C
Interface Temp. :220.00 °C
*m/z* scanning range：45～500m/z

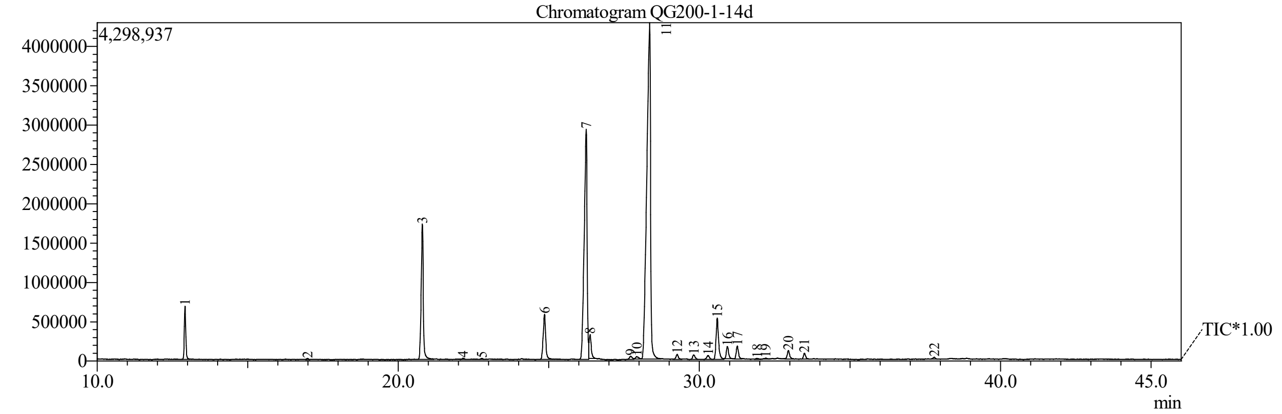


Peak Report TIC

| Peak# | R.Time | Area | Area% | Height | Height% | A/H | Name |
|---|---|---|---|---|---|---|---|
| 1 | 12.921 | 2313210 | 2.90 | 673145 | 5.69 | 3.44 | Undecanoic acid, methyl ester |
| 2 | 16.987 | 69058 | 0.09 | 14978 | 0.13 | 4.61 | Methyl tetradecanoate |
| 3 | 20.803 | 8419778 | 10.54 | 1719957 | 14.54 | 4.90 | Hexadecanoic acid, methyl ester |
| 4 | 22.149 | 69234 | 0.09 | 15826 | 0.13 | 4.37 | 9-Hexadecenoic acid, methyl ester, (Z)- |
| 5 | 22.779 | 52981 | 0.07 | 13288 | 0.11 | 3.99 | Heptadecanoic acid, methyl ester |
| 6 | 24.858 | 3379206 | 4.23 | 573422 | 4.85 | 5.89 | Methyl stearate |
| 7 | 26.241 | 19515997 | 24.44 | 2925331 | 24.73 | 6.67 | 9-Octadecenoic acid, methyl ester, (E)- |
| 8 | 26.371 | 1455797 | 1.82 | 300378 | 2.54 | 4.85 | 11-Octadecenoic acid, methyl ester |
| 9 | 27.716 | 270094 | 0.34 | 39885 | 0.34 | 6.77 | 9,12-Octadecadienoic acid (Z,Z)-, methyl estei |
| 10 | 27.918 | 209686 | 0.26 | 30391 | 0.26 | 6.90 | 9,12-Octadecadienoic acid (Z,Z)-, methyl estei |
| 11 | 28.348 | 37118311 | 46.48 | 4272075 | 36.12 | 8.69 | 9,12-Octadecadienoic acid (Z,Z)-, methyl estei |
| 12 | 29.259 | 329188 | 0.41 | 62440 | 0.53 | 5.27 | Methyl 18-methylnonadecanoate |
| 13 | 29.813 | 320693 | 0.40 | 57893 | 0.49 | 5.54 | 11,14,17-Eicosatrienoic acid, methyl ester |
| 14 | 30.291 | 313876 | 0.39 | 50815 | 0.43 | 6.18 | 9,12,15-Octadecatrienoic acid, methyl ester, (Z |

| Peak# | R.Time | Area | Area% | Height | Height% | A/H | Name |
|---|---|---|---|---|---|---|---|
| 15 | 30.593 | 3042629 | 3.81 | 524856 | 4.44 | 5.80 | 9,12,15-Octadecatrienoic acid, methyl ester, (Z |
| 16 | 30.930 | 828490 | 1.04 | 154982 | 1.31 | 5.35 | Methyl 9-cis,11-trans-octadecadienoate |
| 17 | 31.258 | 853773 | 1.07 | 165284 | 1.40 | 5.17 | Methyl 10-trans,12-cis-octadecadienoate |
| 18 | 31.917 | 74916 | 0.09 | 13000 | 0.11 | 5.76 | Methyl 9-cis,11-trans-octadecadienoate |
| 19 | 32.194 | 89262 | 0.11 | 12251 | 0.10 | 7.29 | 7,10,13-Eicosatrienoic acid, methyl ester |
| 20 | 32.953 | 595072 | 0.75 | 107535 | 0.91 | 5.53 | Cis-3-ethyl-endo-tricyclo[5.2.1.0(2.6)]decane |
| 21 | 33.486 | 406616 | 0.51 | 77233 | 0.65 | 5.26 | Docosanoic acid, methyl ester |
| 22 | 37.799 | 131519 | 0.16 | 23652 | 0.20 | 5.56 | Tetracosanoic acid, methyl ester |
| | | 79859386 | 100.00 | 11828617 | 100.00 | | |

Library

<< Target >>
Line#:1 R.Time:12.917(Scan#:351) MassPeaks:293
RawMode:Averaged 12.908-12.925(350-352) BasePeak:74.00(197377)
BG Mode:Calc. from Peak Group 1 - Event 1

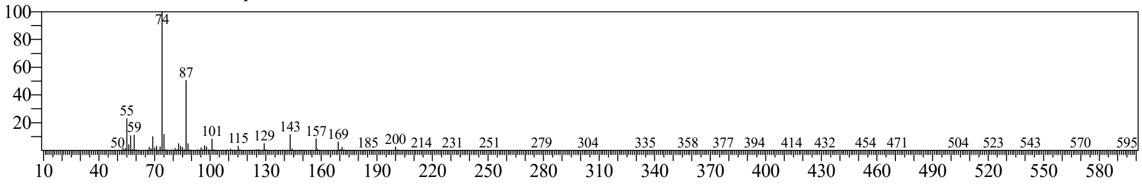


Hit#:1 Entry:49598 Library:NIST17-1.lib
SI:95 Formula:C12H24O2 CAS:1731-86-8 MolWeight:200 RetIndex:1381
CompName:Undecanoic acid, methyl ester $$ Methyl undecanoate $$ Methyl undecylenate $$ n-Undecanoic acid methyl ester $$ Methyl ester of undecanoi

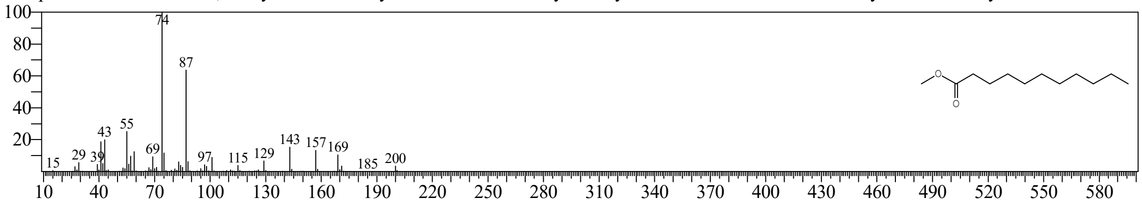


Hit#:2 Entry:46966 Library:NIST14.lib
SI:95 Formula:C12H24O2 CAS:1731-86-8 MolWeight:200 RetIndex:1381
CompName:Undecanoic acid, methyl ester $$ Methyl undecanoate $$ Methyl undecylenate $$ n-Undecanoic acid methyl ester $$ Methyl ester of undecanoi

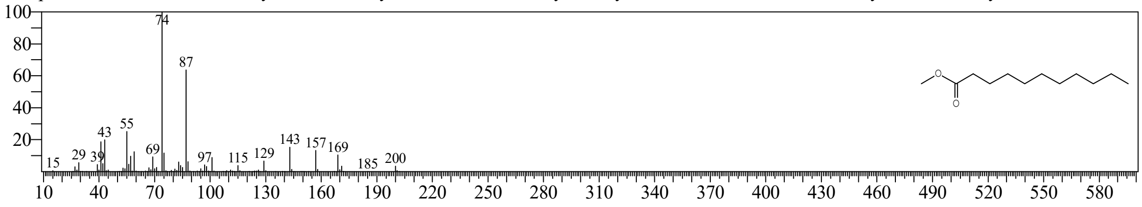


Hit#:3 Entry:21618 Library:NIST17s.lib
SI:95 Formula:C12H24O2 CAS:1731-86-8 MolWeight:200 RetIndex:1381
CompName:Undecanoic acid, methyl ester $$ Methyl undecanoate $$ Methyl undecylenate $$ n-Undecanoic acid methyl ester $$ Methyl ester of undecanoi

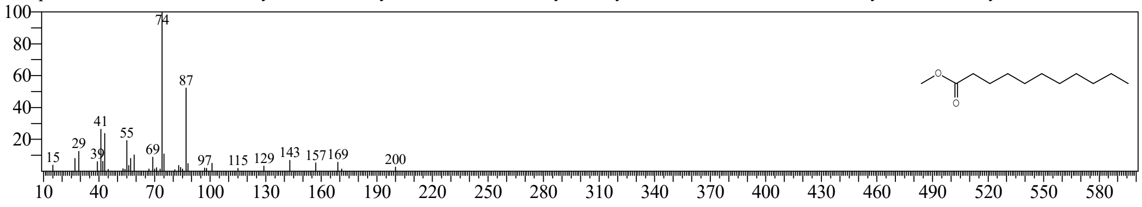


Hit#:4 Entry:21619 Library:NIST17s.lib
SI:94 Formula:C12H24O2 CAS:1731-86-8 MolWeight:200 RetIndex:1381
CompName:Undecanoic acid, methyl ester $$ Methyl undecanoate $$ Methyl undecylenate $$ n-Undecanoic acid methyl ester $$ Methyl ester of undecanoi

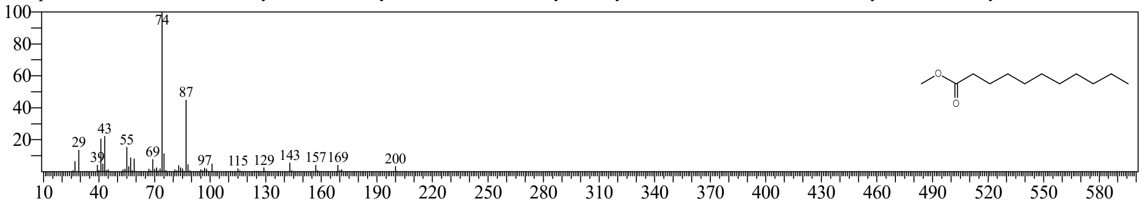

<< Target >>
Line#:1 R.Time:12.917(Scan#:351) MassPeaks:293
RawMode:Averaged 12.908-12.925(350-352) BasePeak:74.00(197377)
BG Mode:Calc. from Peak Group 1 - Event 1

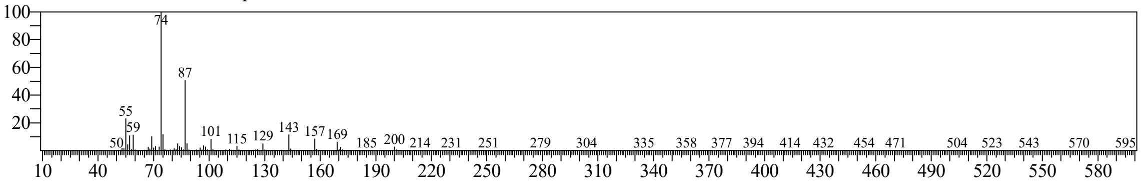


Hit#:5 Entry:18487 Library:NIST14s.lib
SI:94 Formula:C12H24O2 CAS:1731-86-8 MolWeight:200 RetIndex:1381
CompName:Undecanoic acid, methyl ester $$ Methyl undecanoate $$ Methyl undecylenate $$ n-Undecanoic acid methyl ester $$ Methyl ester of undecanoi

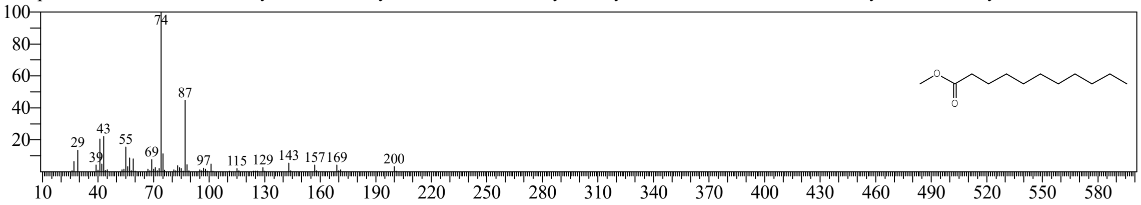

<< Target >>
Line#:2 R.Time:16.983(Scan#:839) MassPeaks:307
RawMode:Averaged 16.975-16.992(838-840) BasePeak:74.00(2878)
BG Mode:Calc. from Peak Group 1 - Event 1

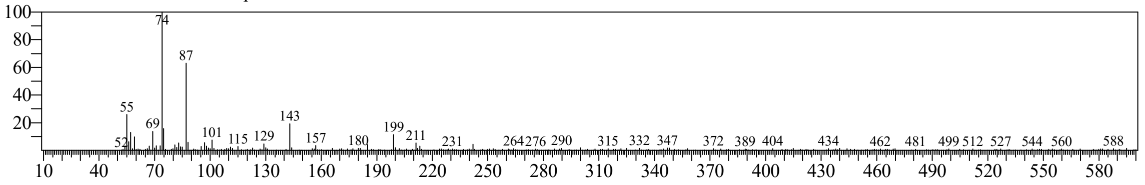


Hit#:1 Entry:80645 Library:NIST14.lib
SI:94 Formula:C15H30O2 CAS:124-10-7 MolWeight:242 RetIndex:1680
CompName:Methyl tetradecanoate $$ Tetradecanoic acid, methyl ester $$ Myristic acid, methyl ester $$ Metholeneat 2495 $$ Methyl myristate $$ Methyl r

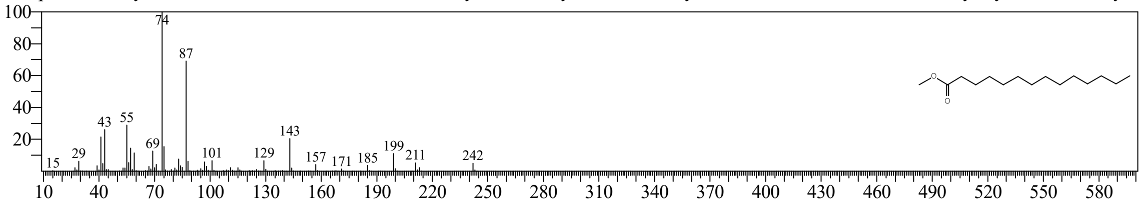


Hit#:2 Entry:86786 Library:NIST17-1.lib
SI:94 Formula:C15H30O2 CAS:124-10-7 MolWeight:242 RetIndex:1680
CompName:Methyl tetradecanoate $$ Tetradecanoic acid, methyl ester $$ Myristic acid, methyl ester $$ Metholeneat 2495 $$ Methyl myristate $$ Methyl r

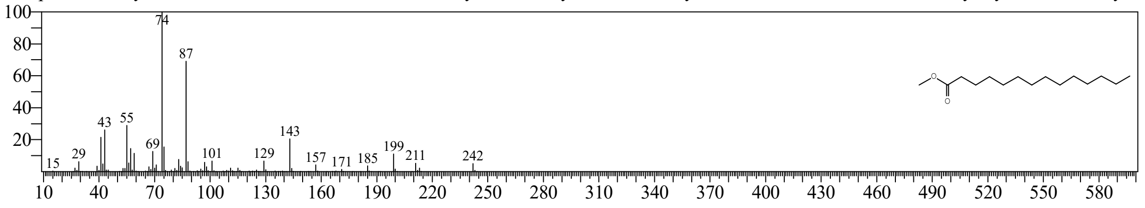


Hit#:3 Entry:23848 Library:NIST14s.lib
SI:92 Formula:C15H30O2 CAS:124-10-7 MolWeight:242 RetIndex:1680
CompName:Methyl tetradecanoate $$ Tetradecanoic acid, methyl ester $$ Myristic acid, methyl ester $$ Metholeneat 2495 $$ Methyl myristate $$ Methyl r

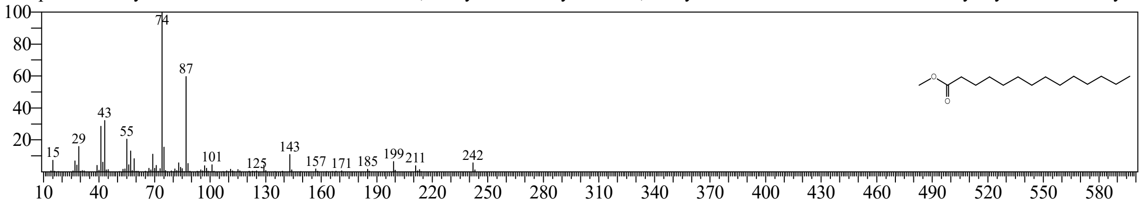


Hit#:4 Entry:27874 Library:NIST17s.lib
SI:92 Formula:C15H30O2 CAS:124-10-7 MolWeight:242 RetIndex:1680
CompName:Methyl tetradecanoate $$ Tetradecanoic acid, methyl ester $$ Myristic acid, methyl ester $$ Metholeneat 2495 $$ Methyl myristate $$ Methyl r

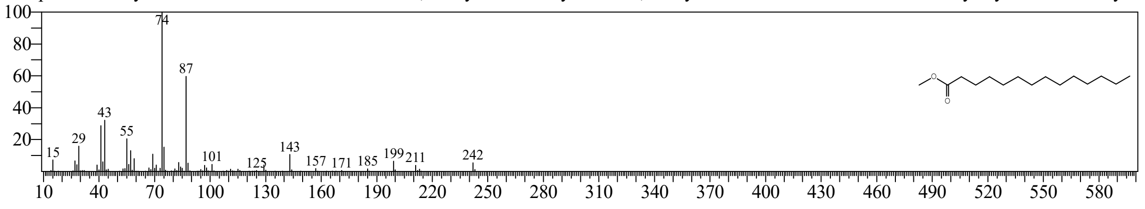


Hit#:5 Entry:27875 Library:NIST17s.lib
SI:92 Formula:C15H30O2 CAS:124-10-7 MolWeight:242 RetIndex:1680
CompName:Methyl tetradecanoate $$ Tetradecanoic acid, methyl ester $$ Myristic acid, methyl ester $$ Metholeneat 2495 $$ Methyl myristate $$ Methyl r

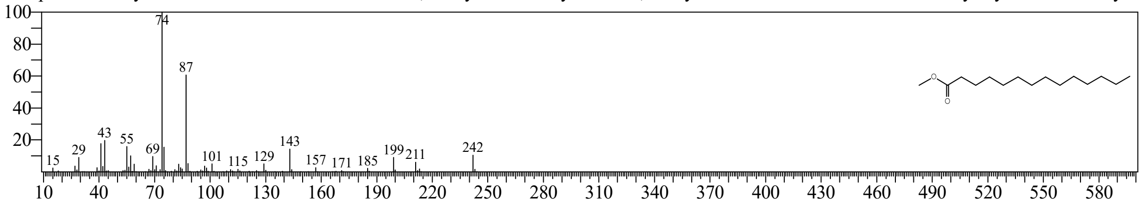

<< Target >>
Line#:3  R.Time:20.800(Scan#:1297)  MassPeaks:394
RawMode:Averaged 20.792-20.808(1296-1298) BasePeak:74.00(398650)
BG Mode:Calc. from Peak  Group 1 - Event 1

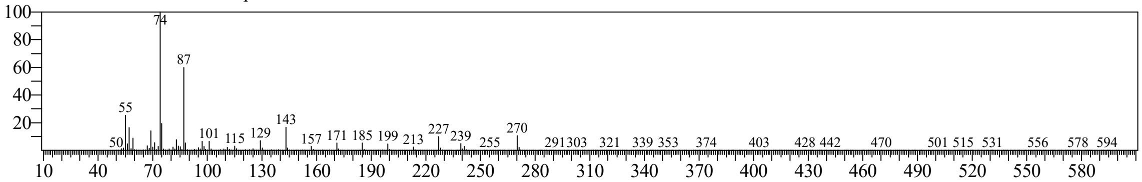


Hit#:1  Entry:113663  Library:NIST17-1.lib
SI:97  Formula:C17H34O2  CAS:112-39-0  MolWeight:270  RetIndex:1878
CompName:Hexadecanoic acid, methyl ester $$ Palmitic acid, methyl ester $$ n-Hexadecanoic acid methyl ester $$ Metholene 2216 $$ Methyl hexadecano

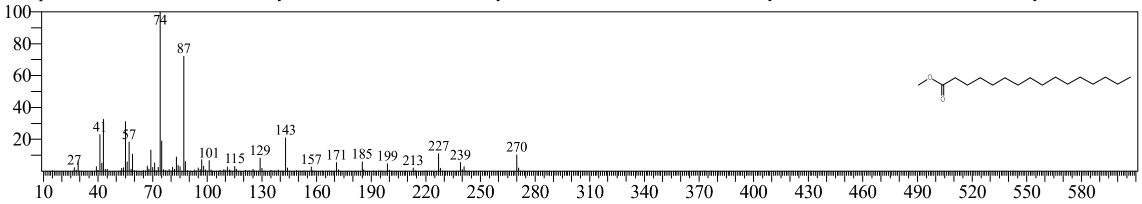


Hit#:2  Entry:104648  Library:NIST14.lib
SI:97  Formula:C17H34O2  CAS:112-39-0  MolWeight:270  RetIndex:1878
CompName:Hexadecanoic acid, methyl ester $$ Palmitic acid, methyl ester $$ n-Hexadecanoic acid methyl ester $$ Metholene 2216 $$ Methyl hexadecano

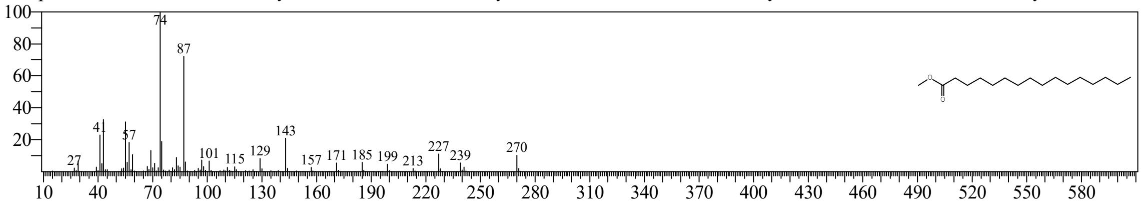


Hit#:3  Entry:26273  Library:NIST14s.lib
SI:96  Formula:C17H34O2  CAS:112-39-0  MolWeight:270  RetIndex:1878
CompName:Hexadecanoic acid, methyl ester $$ Palmitic acid, methyl ester $$ n-Hexadecanoic acid methyl ester $$ Metholene 2216 $$ Methyl hexadecano

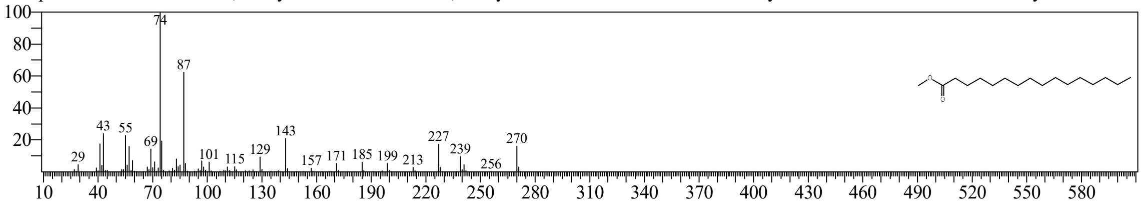


Hit#:4  Entry:26271  Library:NIST14s.lib
SI:96  Formula:C17H34O2  CAS:112-39-0  MolWeight:270  RetIndex:1878
CompName:Hexadecanoic acid, methyl ester $$ Palmitic acid, methyl ester $$ n-Hexadecanoic acid methyl ester $$ Metholene 2216 $$ Methyl hexadecano

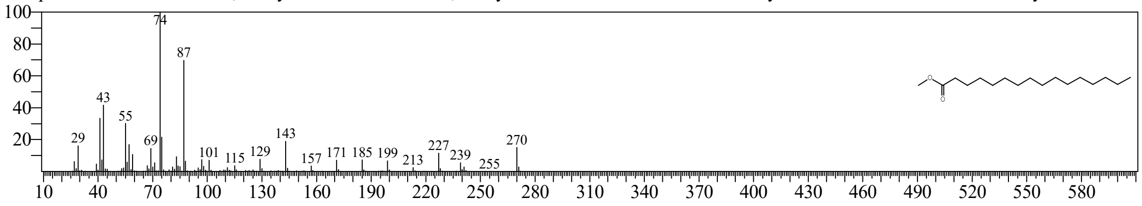


Hit#:5  Entry:30646  Library:NIST17s.lib
SI:96  Formula:C17H34O2  CAS:112-39-0  MolWeight:270  RetIndex:1878
CompName:Hexadecanoic acid, methyl ester $$ Palmitic acid, methyl ester $$ n-Hexadecanoic acid methyl ester $$ Metholene 2216 $$ Methyl hexadecano

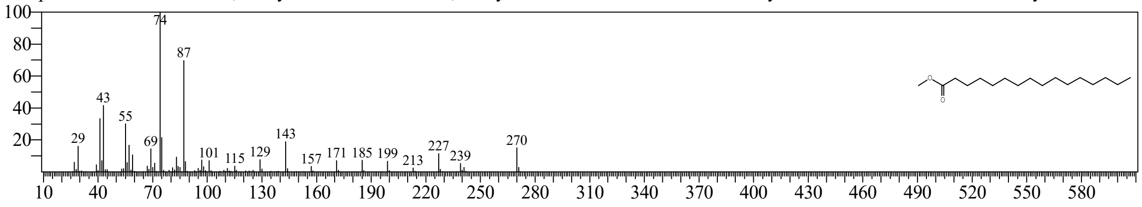

<< Target >>
Line#:4  R.Time:22.150(Scan#:1459)  MassPeaks:350
RawMode:Averaged 22.142-22.158(1458-1460) BasePeak:55.05(1181)
BG Mode:Calc. from Peak  Group 1 - Event 1

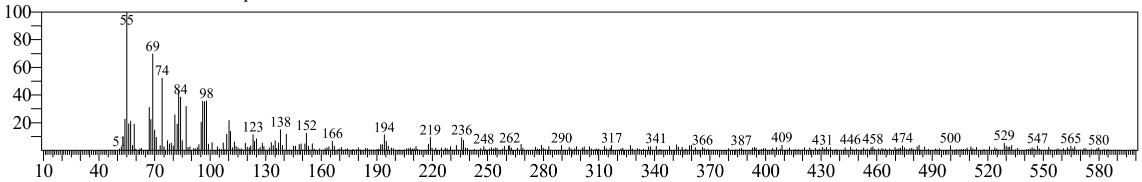


Hit#:1  Entry:26066  Library:NIST14s.lib
SI:90  Formula:C17H32O2  CAS:1120-25-8  MolWeight:268  RetIndex:1886
CompName:9-Hexadecenoic acid, methyl ester, (Z)- $$ Methyl palmitoleate $$ Methyl palmitoleinate $$ Palmitoleic acid, methyl ester $$ Methyl (9Z)-9-he:

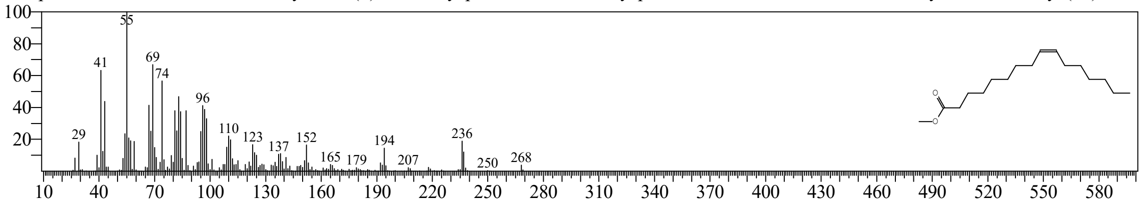


Hit#:2  Entry:30415  Library:NIST17s.lib
SI:90  Formula:C17H32O2  CAS:1120-25-8  MolWeight:268  RetIndex:1886
CompName:9-Hexadecenoic acid, methyl ester, (Z)- $$ Methyl palmitoleate $$ Methyl palmitoleinate $$ Palmitoleic acid, methyl ester $$ Methyl (9Z)-9-he:

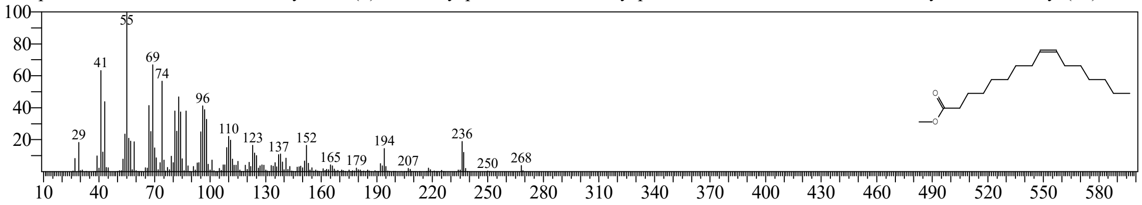


Hit#:3  Entry:111510  Library:NIST17-1.lib
SI:89  Formula:C17H32O2  CAS:56875-67-3  MolWeight:268  RetIndex:1886
CompName:7-Hexadecenoic acid, methyl ester, (Z)- $$ Methyl (7E)-7-hexadecenoate # $$

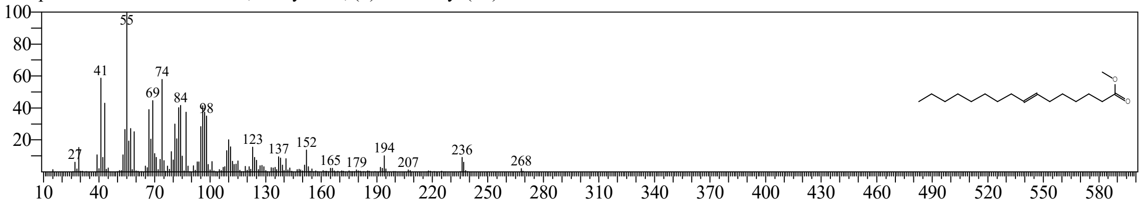


Hit#:4  Entry:26064  Library:NIST14s.lib
SI:89  Formula:C17H32O2  CAS:1120-25-8  MolWeight:268  RetIndex:1886
CompName:9-Hexadecenoic acid, methyl ester, (Z)- $$ Methyl palmitoleate $$ Methyl palmitoleinate $$ Palmitoleic acid, methyl ester $$ Methyl (9Z)-9-he:

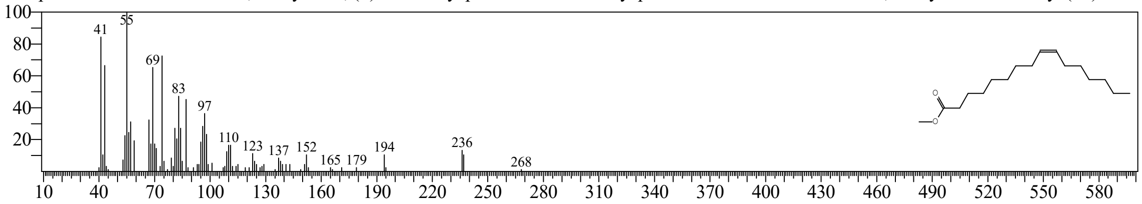


Hit#:5  Entry:30413  Library:NIST17s.lib
SI:89  Formula:C17H32O2  CAS:1120-25-8  MolWeight:268  RetIndex:1886
CompName:9-Hexadecenoic acid, methyl ester, (Z)- $$ Methyl palmitoleate $$ Methyl palmitoleinate $$ Palmitoleic acid, methyl ester $$ Methyl (9Z)-9-he:

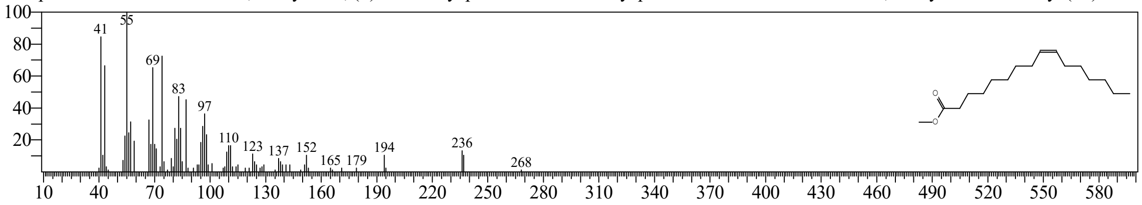

<< Target >>
Line#:5 R.Time:22.775(Scan#:1534) MassPeaks:299
RawMode:Averaged 22.767-22.783(1533-1535) BasePeak:74.00(2784)
BG Mode:Calc. from Peak Group 1 - Event 1

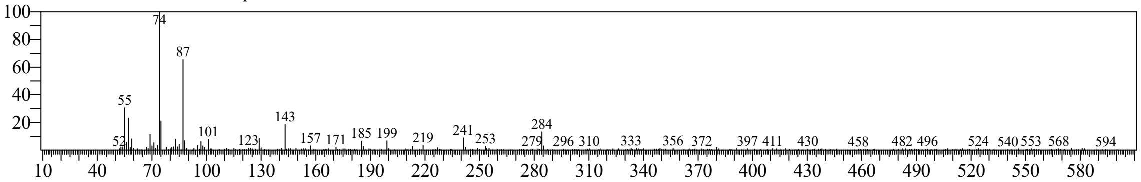


Hit#:1 Entry:27274 Library:NIST14s.lib
SI:92 Formula:C18H36O2 CAS:1731-92-6 MolWeight:284 RetIndex:1978
CompName:Heptadecanoic acid, methyl ester $$ Margaric acid methyl ester $$ Methyl heptadecanoate $$ Methyl margarate $$ n-Heptadecanoic acid methy

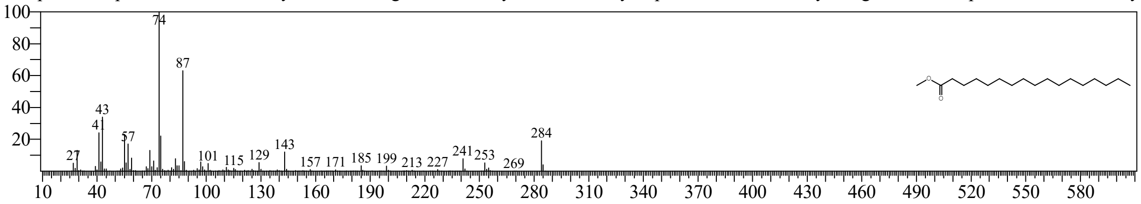


Hit#:2 Entry:31783 Library:NIST17s.lib
SI:92 Formula:C18H36O2 CAS:1731-92-6 MolWeight:284 RetIndex:1978
CompName:Heptadecanoic acid, methyl ester $$ Margaric acid methyl ester $$ Methyl heptadecanoate $$ Methyl margarate $$ n-Heptadecanoic acid methy

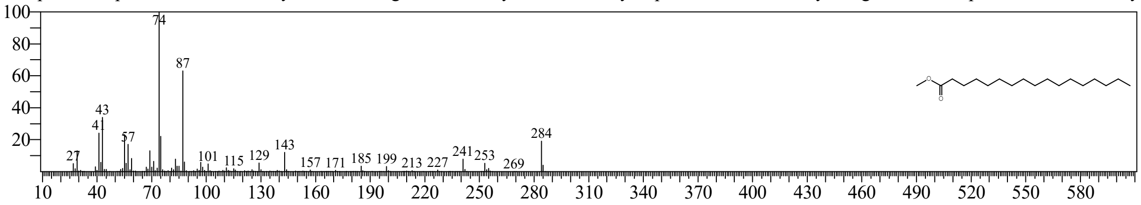


Hit#:3 Entry:31784 Library:NIST17s.lib
SI:91 Formula:C18H36O2 CAS:1731-92-6 MolWeight:284 RetIndex:1978
CompName:Heptadecanoic acid, methyl ester $$ Margaric acid methyl ester $$ Methyl heptadecanoate $$ Methyl margarate $$ n-Heptadecanoic acid methy

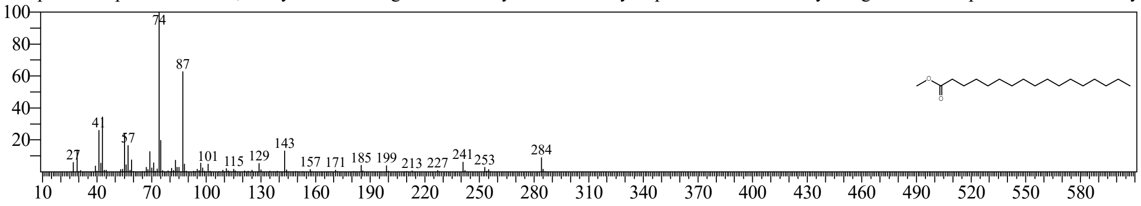


Hit#:4 Entry:27275 Library:NIST14s.lib
SI:91 Formula:C18H36O2 CAS:1731-92-6 MolWeight:284 RetIndex:1978
CompName:Heptadecanoic acid, methyl ester $$ Margaric acid methyl ester $$ Methyl heptadecanoate $$ Methyl margarate $$ n-Heptadecanoic acid methy

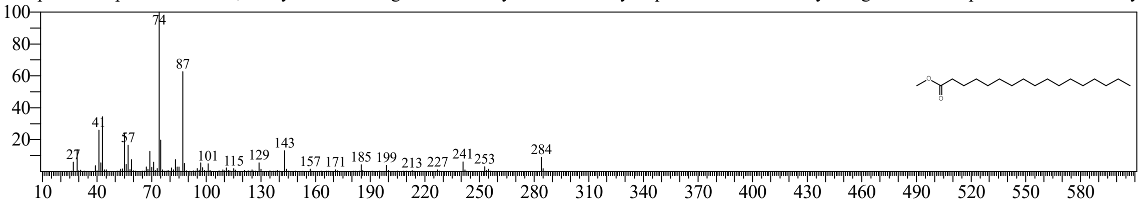


Hit#:5 Entry:27279 Library:NIST14s.lib
SI:91 Formula:C18H36O2 CAS:1731-92-6 MolWeight:284 RetIndex:1978
CompName:Heptadecanoic acid, methyl ester $$ Margaric acid methyl ester $$ Methyl heptadecanoate $$ Methyl margarate $$ n-Heptadecanoic acid methy

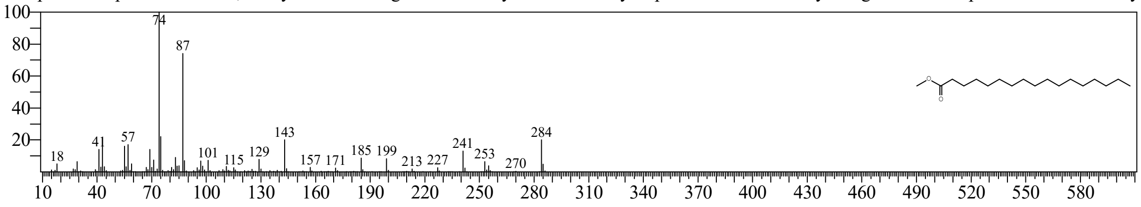

<< Target >>
Line#:6  R.Time:24.858(Scan#:1784)  MassPeaks:362
RawMode:Averaged 24.850-24.867(1783-1785) BasePeak:74.00(115905)
BG Mode:Calc. from Peak  Group 1 - Event 1

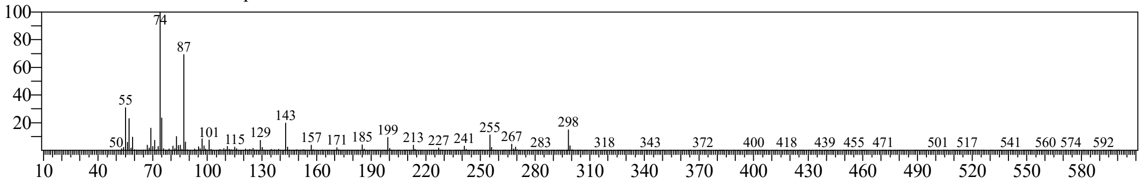


Hit#:1  Entry:129694  Library:NIST14.lib
SI:98  Formula:C19H38O2  CAS:112-61-8  MolWeight:298  RetIndex:2077
CompName:Methyl stearate $$ Octadecanoic acid, methyl ester $$ Stearic acid, methyl ester $$ n-Octadecanoic acid, methyl ester $$ Kemester 9718 $$ Me

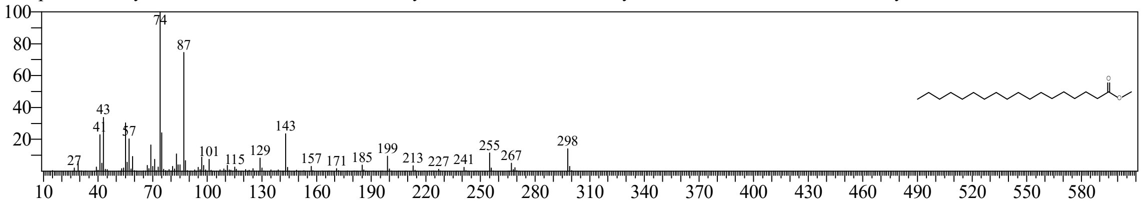


Hit#:2  Entry:141978  Library:NIST17-1.lib
SI:98  Formula:C19H38O2  CAS:112-61-8  MolWeight:298  RetIndex:2077
CompName:Methyl stearate $$ Octadecanoic acid, methyl ester $$ Stearic acid, methyl ester $$ n-Octadecanoic acid, methyl ester $$ Kemester 9718 $$ Me

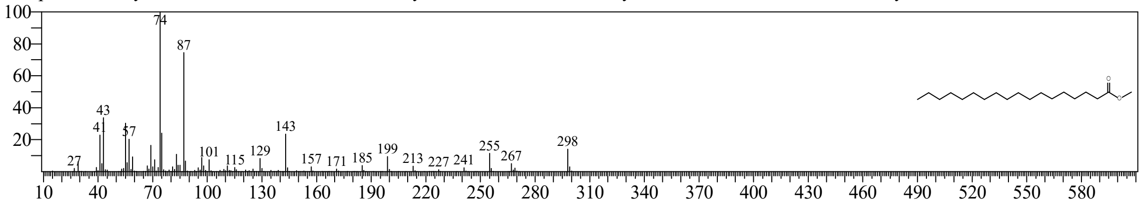


Hit#:3  Entry:28255  Library:NIST14s.lib
SI:97  Formula:C19H38O2  CAS:112-61-8  MolWeight:298  RetIndex:2077
CompName:Methyl stearate $$ Octadecanoic acid, methyl ester $$ Stearic acid, methyl ester $$ n-Octadecanoic acid, methyl ester $$ Kemester 9718 $$ Me

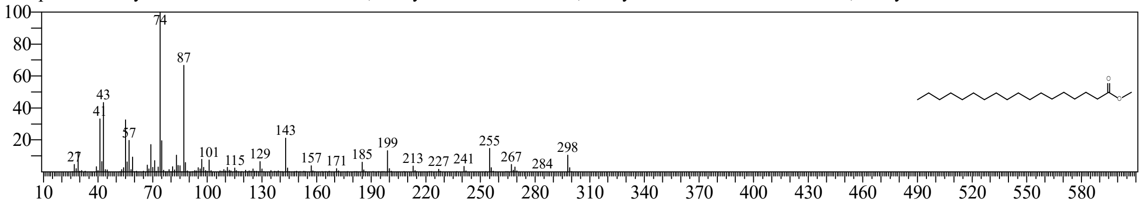


Hit#:4  Entry:32873  Library:NIST17s.lib
SI:97  Formula:C19H38O2  CAS:112-61-8  MolWeight:298  RetIndex:2077
CompName:Methyl stearate $$ Octadecanoic acid, methyl ester $$ Stearic acid, methyl ester $$ n-Octadecanoic acid, methyl ester $$ Kemester 9718 $$ Me

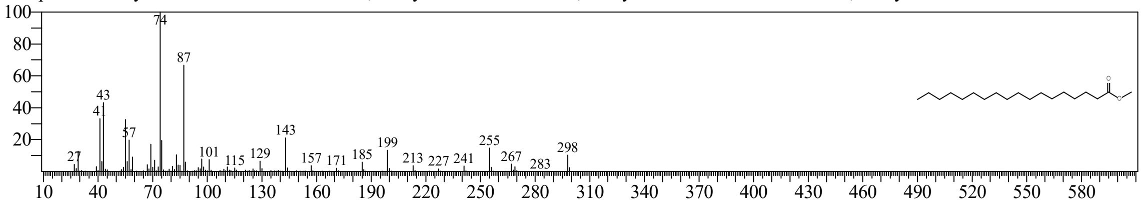


Hit#:5  Entry:28257  Library:NIST14s.lib
SI:96  Formula:C19H38O2  CAS:112-61-8  MolWeight:298  RetIndex:2077
CompName:Methyl stearate $$ Octadecanoic acid, methyl ester $$ Stearic acid, methyl ester $$ n-Octadecanoic acid, methyl ester $$ Kemester 9718 $$ Me

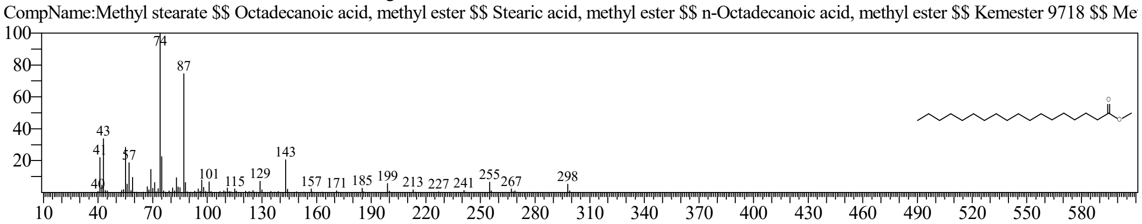

<< Target >>
Line#:7 R.Time:26.242(Scan#:1950) MassPeaks:385
RawMode:Averaged 26.233-26.250(1949-1951) BasePeak:55.05(223584)
BG Mode:Calc. from Peak Group 1 - Event 1

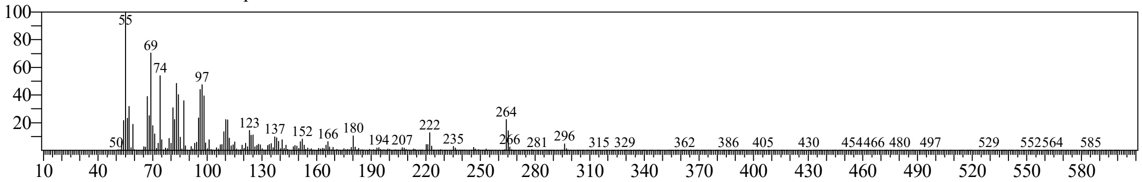


Hit#:1 Entry:32747 Library:NIST17s.lib
SI:97 Formula:C19H36O2 CAS:1937-62-8 MolWeight:296 RetIndex:2085
CompName:9-Octadecenoic acid, methyl ester, (E)- $$ Elaidic acid, methyl ester $$ Methyl elaidate $$ Methyl trans-9-octadecenoate $$ (E)-9-Octadecenoic

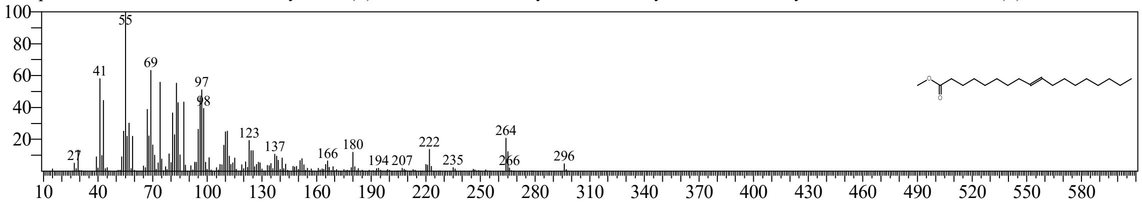


Hit#:2 Entry:28138 Library:NIST14s.lib
SI:97 Formula:C19H36O2 CAS:1937-62-8 MolWeight:296 RetIndex:2085
CompName:9-Octadecenoic acid, methyl ester, (E)- $$ Elaidic acid, methyl ester $$ Methyl elaidate $$ Methyl trans-9-octadecenoate $$ (E)-9-Octadecenoic

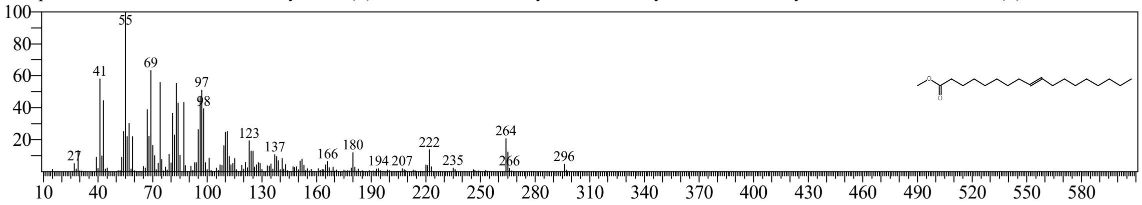


Hit#:3 Entry:28139 Library:NIST14s.lib
SI:96 Formula:C19H36O2 CAS:52380-33-3 MolWeight:296 RetIndex:2085
CompName:11-Octadecenoic acid, methyl ester $$ Methyl 11-octadecenoate $$ Octadec-11-enoic acid, methyl ester $$ Methyl (11E)-11-octadecenoate # $$

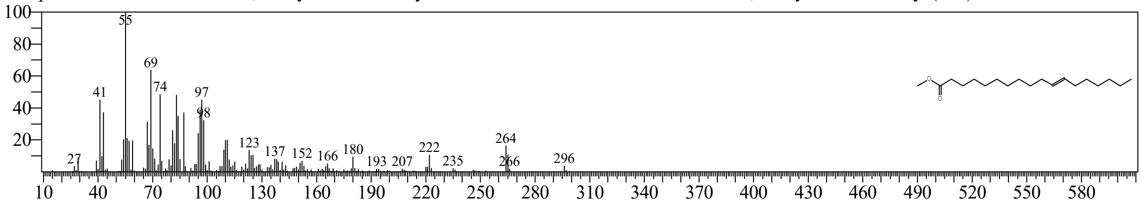


Hit#:4 Entry:32748 Library:NIST17s.lib
SI:96 Formula:C19H36O2 CAS:52380-33-3 MolWeight:296 RetIndex:2085
CompName:11-Octadecenoic acid, methyl ester $$ Methyl 11-octadecenoate $$ Octadec-11-enoic acid, methyl ester $$ Methyl (11E)-11-octadecenoate # $$

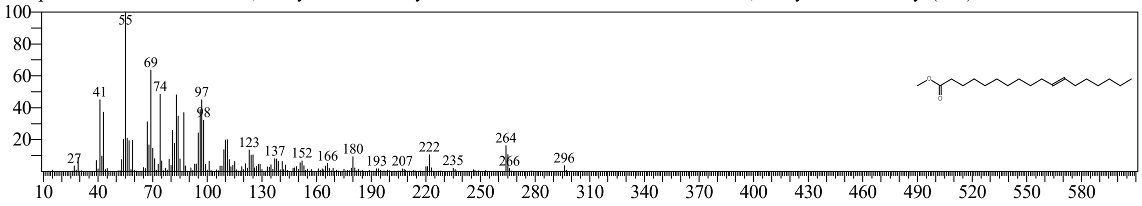


Hit#:5 Entry:127664 Library:NIST14.lib
SI:96 Formula:C19H36O2 CAS:112-62-9 MolWeight:296 RetIndex:2085
CompName:9-Octadecenoic acid (Z)-, methyl ester $$ Oleic acid, methyl ester $$ Emery oleic acid ester 2301 $$ Methyl cis-9-octadecenoate $$ Methyl ole

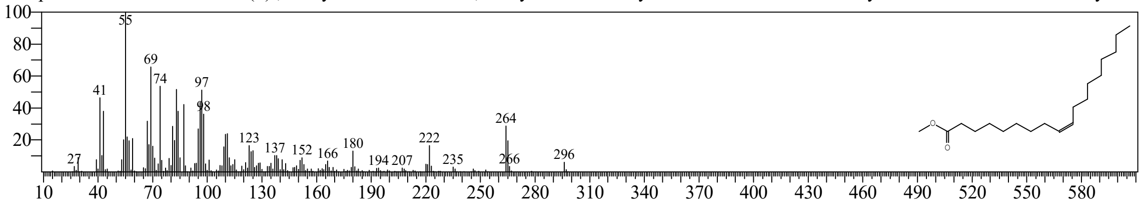

<< Target >>
Line#:8  R.Time:26.367(Scan#:1965)  MassPeaks:341
RawMode:Averaged 26.358-26.375(1964-1966) BasePeak:55.05(14373)
BG Mode:Calc. from Peak  Group 1 - Event 1

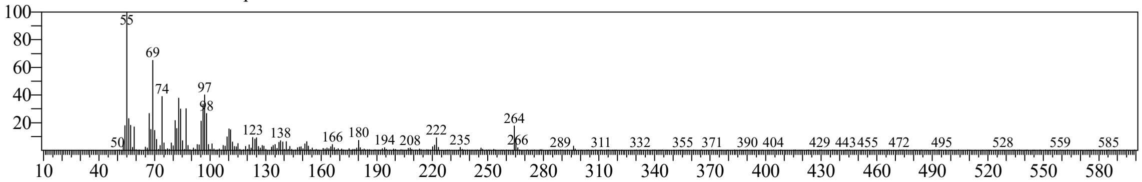

Hit#:1  Entry:32748  Library:NIST17s.lib
SI:95  Formula:C19H36O2  CAS:52380-33-3  MolWeight:296  RetIndex:2085
CompName:11-Octadecenoic acid, methyl ester $$ Methyl 11-octadecenoate $$ Octadec-11-enoic acid, methyl ester $$ Methyl (11E)-11-octadecenoate # $$

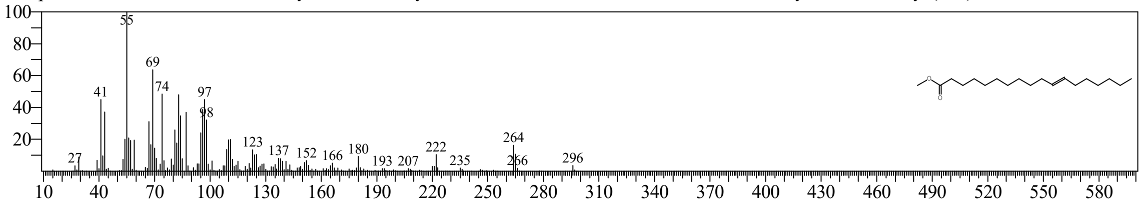

Hit#:2  Entry:28139  Library:NIST14s.lib
SI:95  Formula:C19H36O2  CAS:52380-33-3  MolWeight:296  RetIndex:2085
CompName:11-Octadecenoic acid, methyl ester $$ Methyl 11-octadecenoate $$ Octadec-11-enoic acid, methyl ester $$ Methyl (11E)-11-octadecenoate # $$

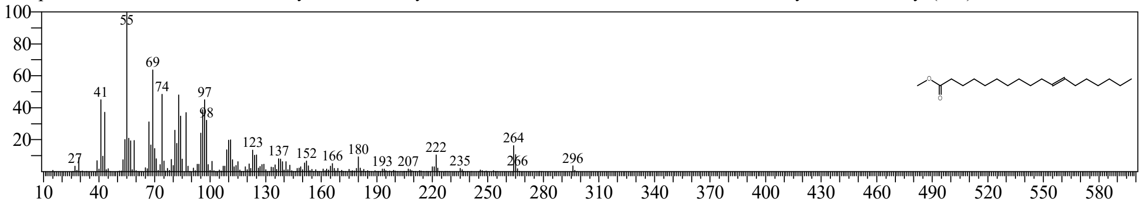

Hit#:3  Entry:139647  Library:NIST17-1.lib
SI:95  Formula:C19H36O2  CAS:0-00-0  MolWeight:296  RetIndex:2085
CompName:cis-13-Octadecenoic acid, methyl ester

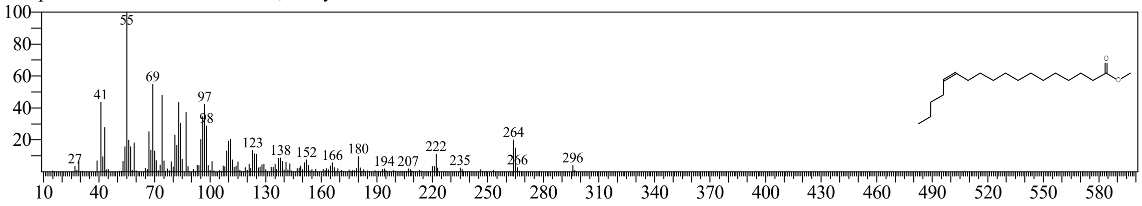

Hit#:4  Entry:127661  Library:NIST14.lib
SI:95  Formula:C19H36O2  CAS:0-00-0  MolWeight:296  RetIndex:2085
CompName:cis-13-Octadecenoic acid, methyl ester

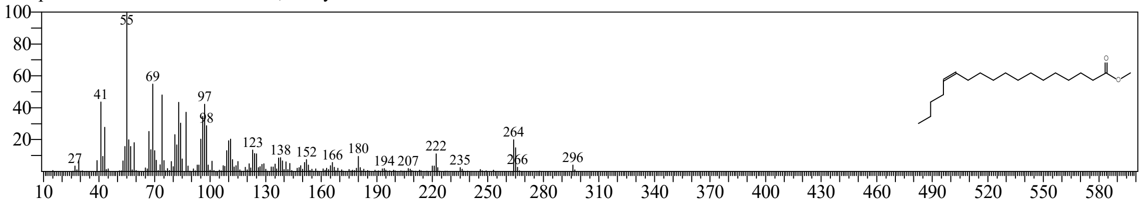

Hit#:5  Entry:127649  Library:NIST14.lib
SI:94  Formula:C19H36O2  CAS:56554-46-2  MolWeight:296  RetIndex:2085
CompName:12-Octadecenoic acid, methyl ester $$ Methyl (12E)-12-octadecenoate # $$

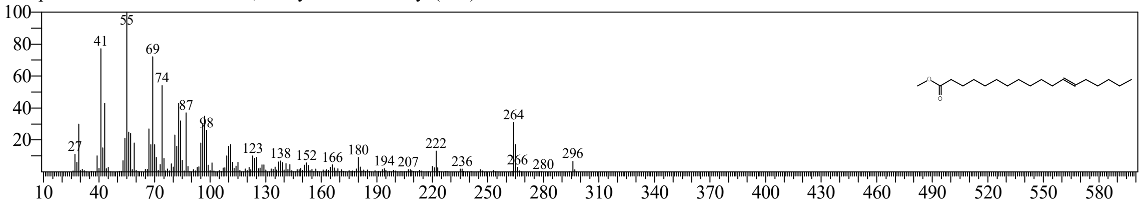

<< Target >>
Line#:9 R.Time:27.717(Scan#:2127) MassPeaks:336
RawMode:Averaged 27.708-27.725(2126-2128) BasePeak:67.00(3363)
BG Mode:Calc. from Peak Group 1 - Event 1

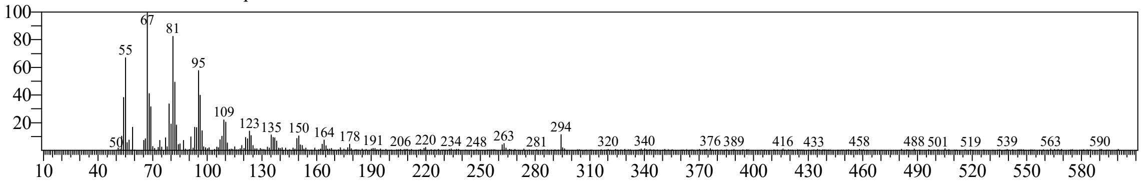


Hit#:1 Entry:32588 Library:NIST17s.lib
SI:96 Formula:C19H34O2 CAS:112-63-0 MolWeight:294 RetIndex:2093
CompName:9,12-Octadecadienoic acid (Z,Z)-, methyl ester $$ Linoleic acid, methyl ester $$ Methyl cis,cis-9,12-octadecadienoate $$ Methyl linoleate $$ M

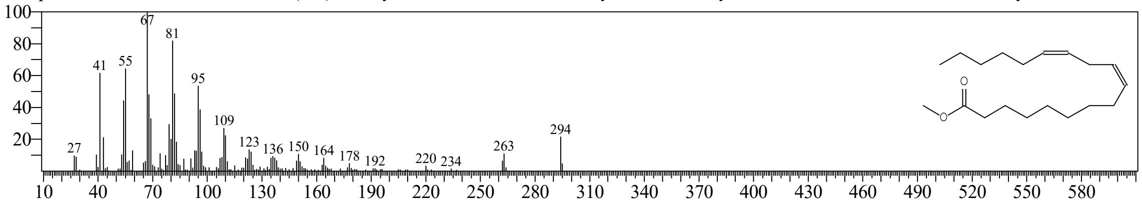


Hit#:2 Entry:27999 Library:NIST14s.lib
SI:96 Formula:C19H34O2 CAS:112-63-0 MolWeight:294 RetIndex:2093
CompName:9,12-Octadecadienoic acid (Z,Z)-, methyl ester $$ Linoleic acid, methyl ester $$ Methyl cis,cis-9,12-octadecadienoate $$ Methyl linoleate $$ M

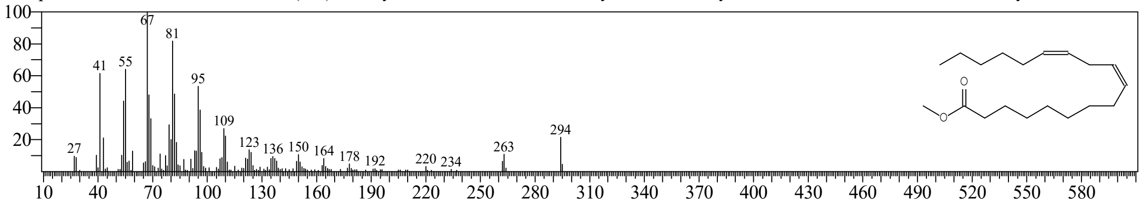


Hit#:3 Entry:137663 Library:NIST17-1.lib
SI:96 Formula:C19H34O2 CAS:2462-85-3 MolWeight:294 RetIndex:2093
CompName:9,12-Octadecadienoic acid, methyl ester $$ 9,12-Octadecenoic acid, methyl ester $$ Methyl octadeca-9,12-dienoate $$ Methyl 9,12-octadecadi

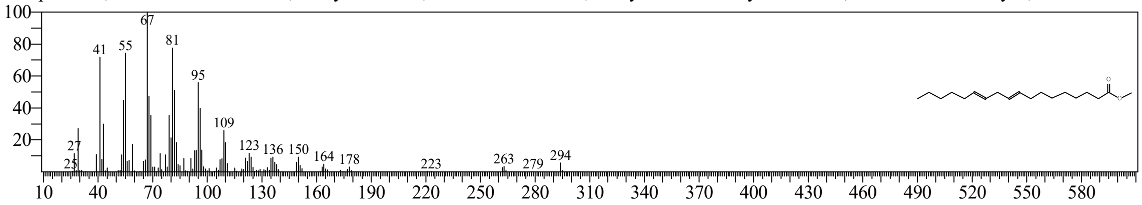


Hit#:4 Entry:125931 Library:NIST14.lib
SI:96 Formula:C19H34O2 CAS:2462-85-3 MolWeight:294 RetIndex:2093
CompName:9,12-Octadecadienoic acid, methyl ester $$ 9,12-Octadecenoic acid, methyl ester $$ Methyl octadeca-9,12-dienoate $$ Methyl 9,12-octadecadi

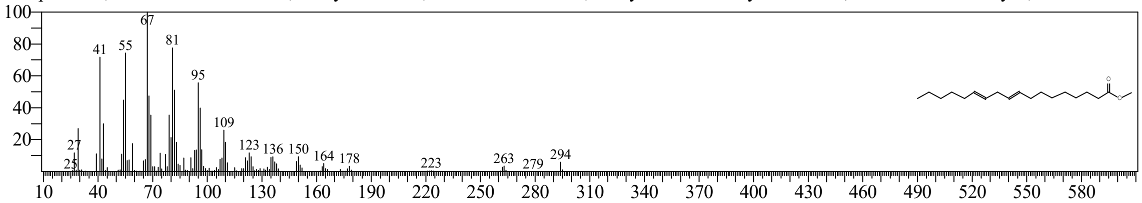


Hit#:5 Entry:32589 Library:NIST17s.lib
SI:96 Formula:C19H34O2 CAS:112-63-0 MolWeight:294 RetIndex:2093
CompName:9,12-Octadecadienoic acid (Z,Z)-, methyl ester $$ Linoleic acid, methyl ester $$ Methyl cis,cis-9,12-octadecadienoate $$ Methyl linoleate $$ M

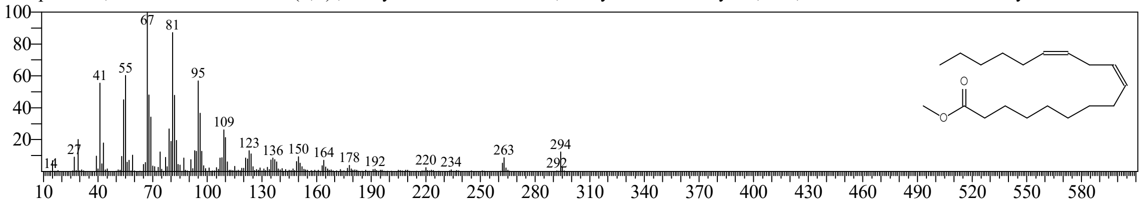

<< Target >>
Line#:10 R.Time:27.917(Scan#:2151) MassPeaks:381
RawMode:Averaged 27.908-27.925(2150-2152) BasePeak:67.05(2296)
BG Mode:Calc. from Peak Group 1 - Event 1

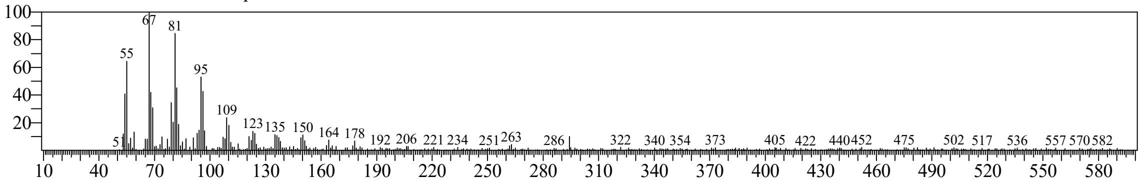


Hit#:1 Entry:32588 Library:NIST17s.lib
SI:96 Formula:C19H34O2 CAS:112-63-0 MolWeight:294 RetIndex:2093
CompName:9,12-Octadecadienoic acid (Z,Z)-, methyl ester $$ Linoleic acid, methyl ester $$ Methyl cis,cis-9,12-octadecadienoate $$ Methyl linoleate $$ M

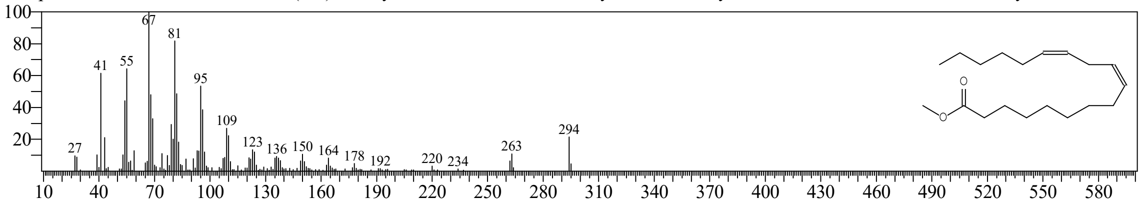


Hit#:2 Entry:27999 Library:NIST14s.lib
SI:96 Formula:C19H34O2 CAS:112-63-0 MolWeight:294 RetIndex:2093
CompName:9,12-Octadecadienoic acid (Z,Z)-, methyl ester $$ Linoleic acid, methyl ester $$ Methyl cis,cis-9,12-octadecadienoate $$ Methyl linoleate $$ M

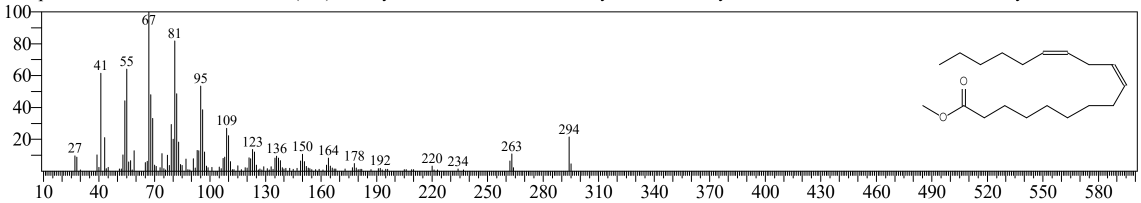


Hit#:3 Entry:137663 Library:NIST17-1.lib
SI:95 Formula:C19H34O2 CAS:2462-85-3 MolWeight:294 RetIndex:2093
CompName:9,12-Octadecadienoic acid, methyl ester $$ 9,12-Octadecenoic acid, methyl ester $$ Methyl octadeca-9,12-dienoate $$ Methyl 9,12-octadecadi

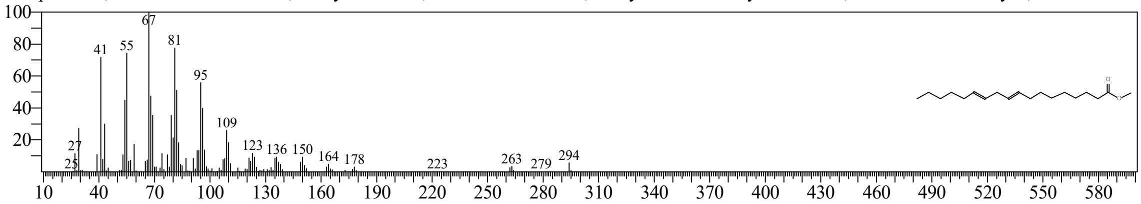


Hit#:4 Entry:125931 Library:NIST14.lib
SI:95 Formula:C19H34O2 CAS:2462-85-3 MolWeight:294 RetIndex:2093
CompName:9,12-Octadecadienoic acid, methyl ester $$ 9,12-Octadecenoic acid, methyl ester $$ Methyl octadeca-9,12-dienoate $$ Methyl 9,12-octadecadi

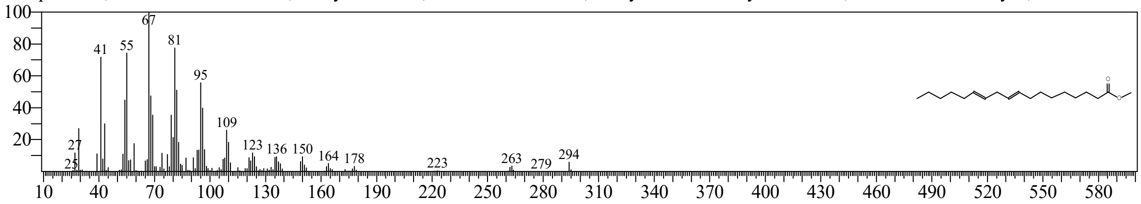


Hit#:5 Entry:32589 Library:NIST17s.lib
SI:95 Formula:C19H34O2 CAS:112-63-0 MolWeight:294 RetIndex:2093
CompName:9,12-Octadecadienoic acid (Z,Z)-, methyl ester $$ Linoleic acid, methyl ester $$ Methyl cis,cis-9,12-octadecadienoate $$ Methyl linoleate $$ M

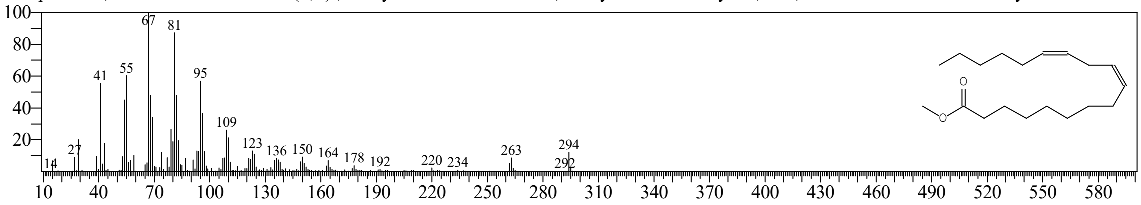

<< Target >>
Line#:11 R.Time:28.350(Scan#:2203) MassPeaks:421
RawMode:Averaged 28.342-28.358(2202-2204) BasePeak:67.05(419181)
BG Mode:Calc. from Peak Group 1 - Event 1

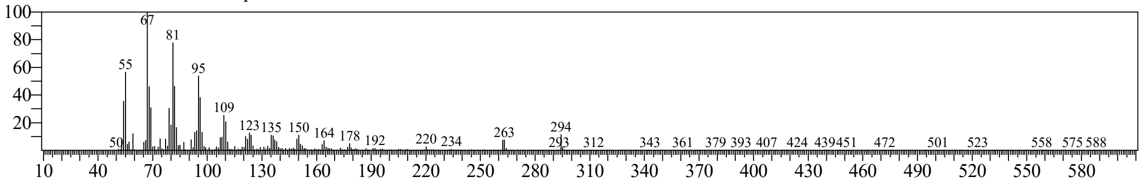


Hit#:1 Entry:32588 Library:NIST17s.lib
SI:97 Formula:C19H34O2 CAS:112-63-0 MolWeight:294 RetIndex:2093
CompName:9,12-Octadecadienoic acid (Z,Z)-, methyl ester $$ Linoleic acid, methyl ester $$ Methyl cis,cis-9,12-octadecadienoate $$ Methyl linoleate $$ M

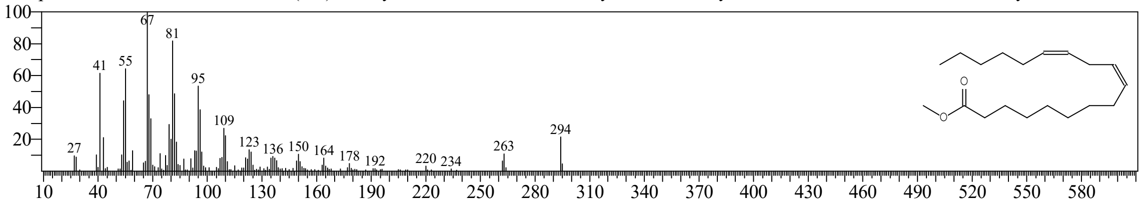


Hit#:2 Entry:27999 Library:NIST14s.lib
SI:97 Formula:C19H34O2 CAS:112-63-0 MolWeight:294 RetIndex:2093
CompName:9,12-Octadecadienoic acid (Z,Z)-, methyl ester $$ Linoleic acid, methyl ester $$ Methyl cis,cis-9,12-octadecadienoate $$ Methyl linoleate $$ M

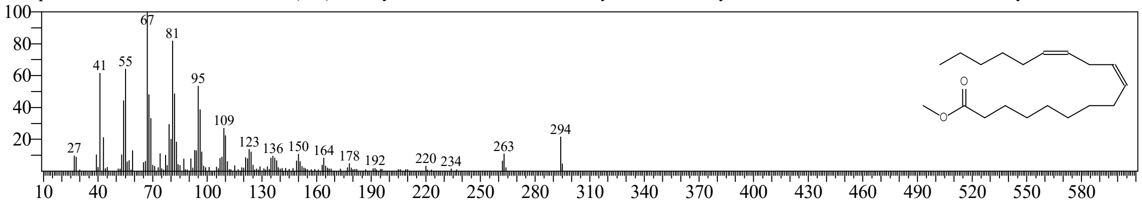


Hit#:3 Entry:32589 Library:NIST17s.lib
SI:96 Formula:C19H34O2 CAS:112-63-0 MolWeight:294 RetIndex:2093
CompName:9,12-Octadecadienoic acid (Z,Z)-, methyl ester $$ Linoleic acid, methyl ester $$ Methyl cis,cis-9,12-octadecadienoate $$ Methyl linoleate $$ M

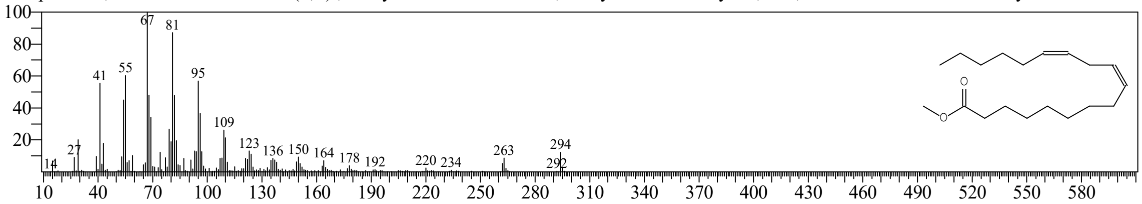


Hit#:4 Entry:28000 Library:NIST14s.lib
SI:96 Formula:C19H34O2 CAS:112-63-0 MolWeight:294 RetIndex:2093
CompName:9,12-Octadecadienoic acid (Z,Z)-, methyl ester $$ Linoleic acid, methyl ester $$ Methyl cis,cis-9,12-octadecadienoate $$ Methyl linoleate $$ M

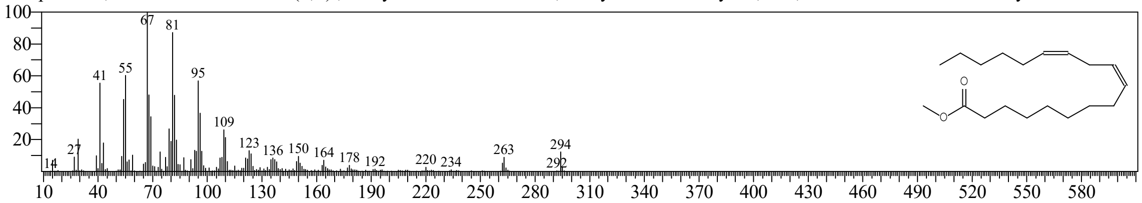


Hit#:5 Entry:125933 Library:NIST14.lib
SI:96 Formula:C19H34O2 CAS:0-00-0 MolWeight:294 RetIndex:2093
CompName:Methyl 10-trans,12-cis-octadecadienoate

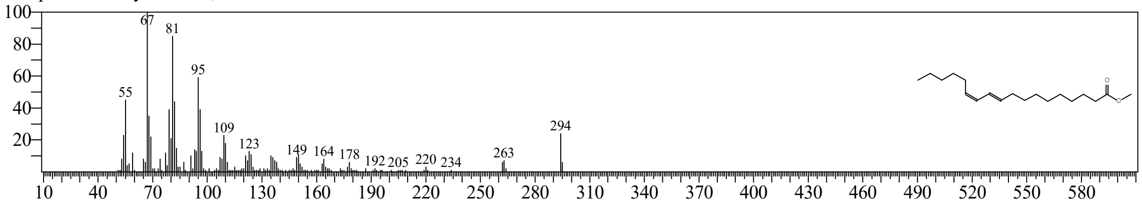

<< Target >>
Line#:12   R.Time:29.258(Scan#:2312)  MassPeaks:351
RawMode:Averaged 29.250-29.267(2311-2313) BasePeak:74.00(10571)
BG Mode:Calc. from Peak  Group 1 - Event 1

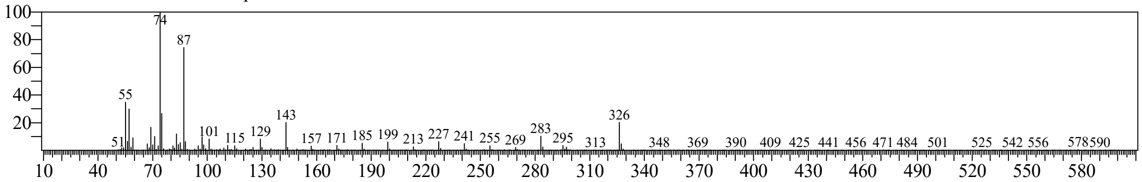


Hit#:1  Entry:170238  Library:NIST17-1.lib
SI:97  Formula:C21H42O2  CAS:0-00-0  MolWeight:326  RetIndex:2212
CompName:Methyl 18-methylnonadecanoate

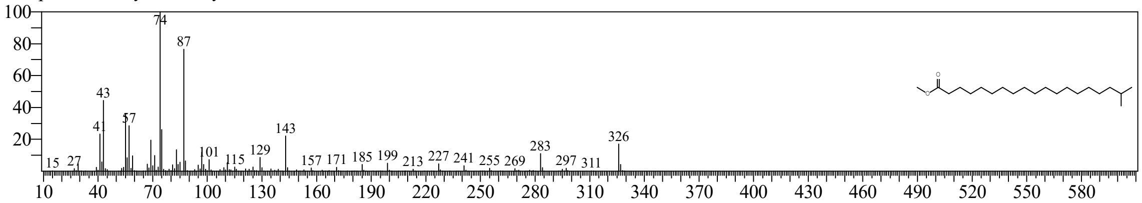


Hit#:2  Entry:154704  Library:NIST14.lib
SI:97  Formula:C21H42O2  CAS:0-00-0  MolWeight:326  RetIndex:2212
CompName:Methyl 18-methylnonadecanoate

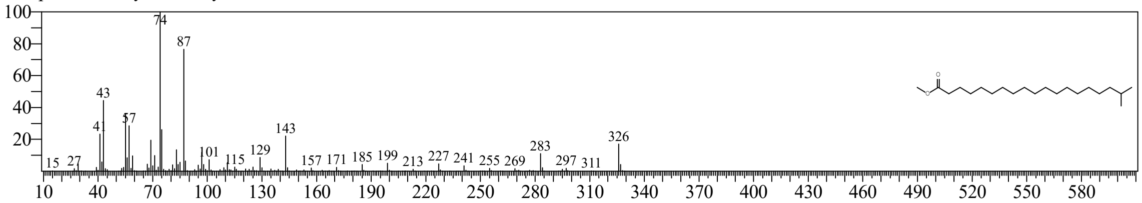


Hit#:3  Entry:34726  Library:NIST17s.lib
SI:96  Formula:C21H42O2  CAS:1120-28-1  MolWeight:326  RetIndex:2276
CompName:Eicosanoic acid, methyl ester $$ Methyl arachisate $$ Methyl eicosanoate $$ Arachidic acid methyl ester $$ Kemester 2050 $$ Methyl aracidat

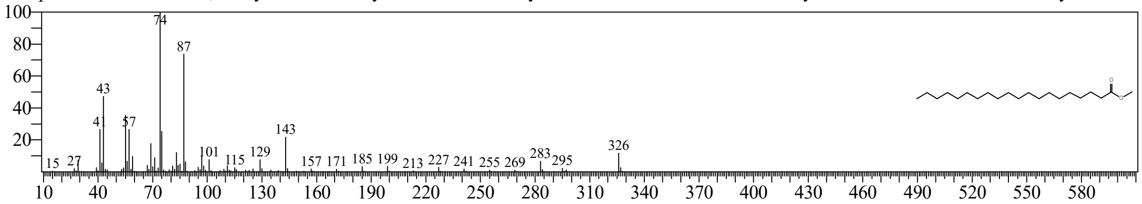


Hit#:4  Entry:29931  Library:NIST14s.lib
SI:93  Formula:C21H42O2  CAS:1120-28-1  MolWeight:326  RetIndex:2276
CompName:Eicosanoic acid, methyl ester $$ Methyl arachisate $$ Methyl eicosanoate $$ Arachidic acid methyl ester $$ Kemester 2050 $$ Methyl aracidat

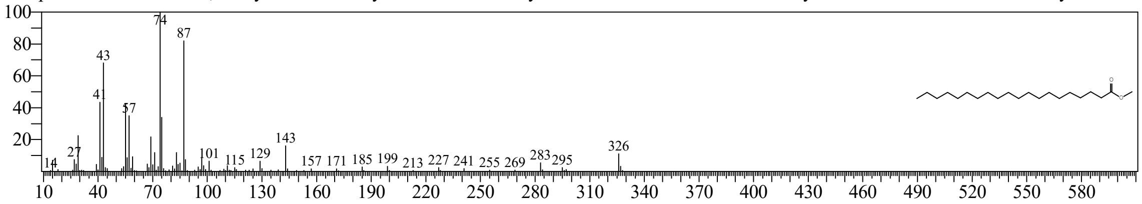


Hit#:5  Entry:34724  Library:NIST17s.lib
SI:93  Formula:C21H42O2  CAS:1120-28-1  MolWeight:326  RetIndex:2276
CompName:Eicosanoic acid, methyl ester $$ Methyl arachisate $$ Methyl eicosanoate $$ Arachidic acid methyl ester $$ Kemester 2050 $$ Methyl aracidat

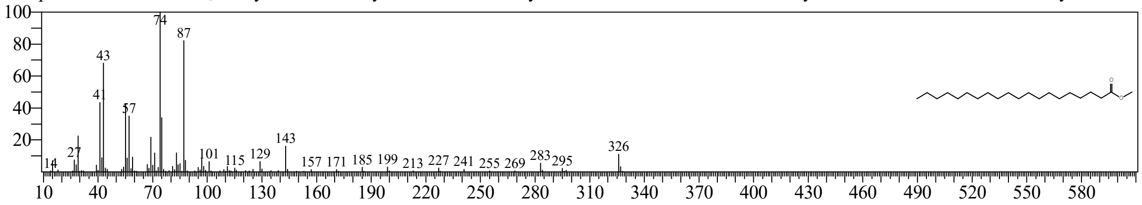

<< Target >>
Line#:13 R.Time:29.817(Scan#:2379) MassPeaks:348
RawMode:Averaged 29.808-29.825(2378-2380) BasePeak:79.05(6393)
BG Mode:Calc. from Peak Group 1 - Event 1

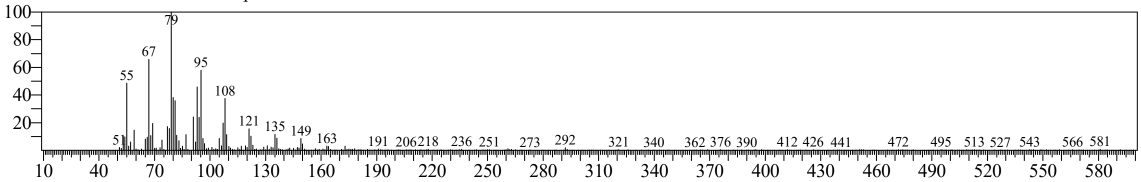

Hit#:1 Entry:29609 Library:NIST14s.lib
SI:95 Formula:C21H36O2 CAS:55682-88-7 MolWeight:320 RetIndex:2300
CompName:11,14,17-Eicosatrienoic acid, methyl ester $$ Methyl 11,14,17-icosatrienoate $$ Methyl 11,14,17-eicosatrienoate $$

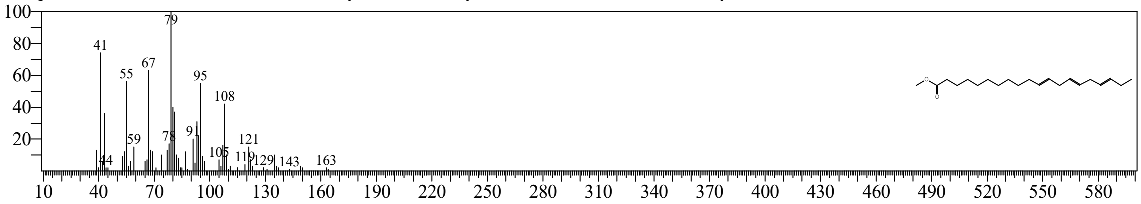

Hit#:2 Entry:34362 Library:NIST17s.lib
SI:95 Formula:C21H36O2 CAS:55682-88-7 MolWeight:320 RetIndex:2300
CompName:11,14,17-Eicosatrienoic acid, methyl ester $$ Methyl 11,14,17-icosatrienoate $$ Methyl 11,14,17-eicosatrienoate $$

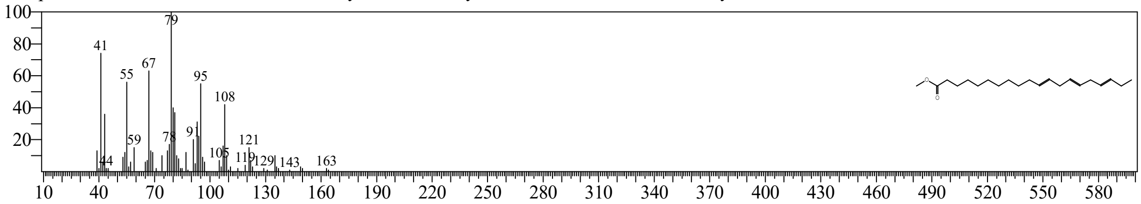

Hit#:3 Entry:32438 Library:NIST17s.lib
SI:95 Formula:C19H32O2 CAS:301-00-8 MolWeight:292 RetIndex:2101
CompName:9,12,15-Octadecatrienoic acid, methyl ester, (Z,Z,Z)- $$ Linolenic acid, methyl ester $$ Methyl all-cis-9,12,15-octadecatrienoate $$ Methyl lino

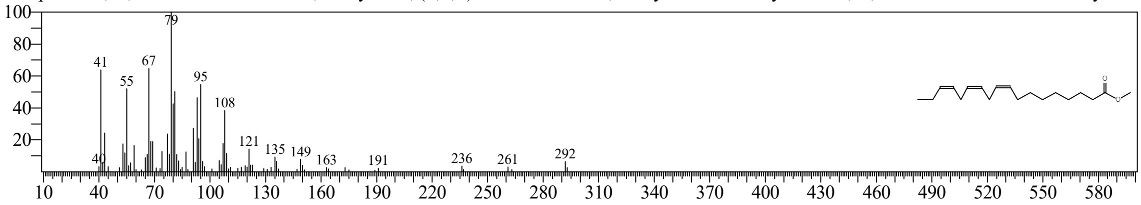

Hit#:4 Entry:32437 Library:NIST17s.lib
SI:95 Formula:C19H32O2 CAS:301-00-8 MolWeight:292 RetIndex:2101
CompName:9,12,15-Octadecatrienoic acid, methyl ester, (Z,Z,Z)- $$ Linolenic acid, methyl ester $$ Methyl all-cis-9,12,15-octadecatrienoate $$ Methyl lino

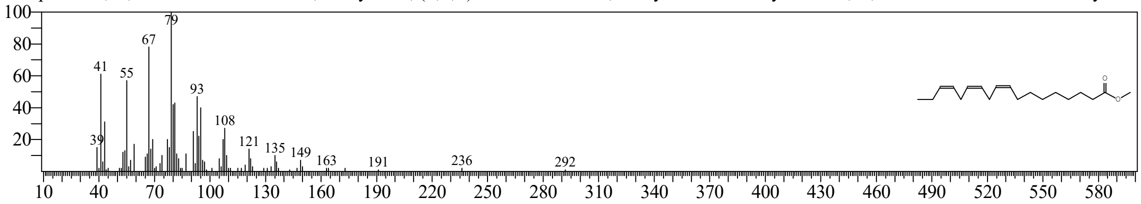

Hit#:5 Entry:124228 Library:NIST14.lib
SI:95 Formula:C19H32O2 CAS:301-00-8 MolWeight:292 RetIndex:2101
CompName:9,12,15-Octadecatrienoic acid, methyl ester, (Z,Z,Z)- $$ Linolenic acid, methyl ester $$ Methyl all-cis-9,12,15-octadecatrienoate $$ Methyl lino

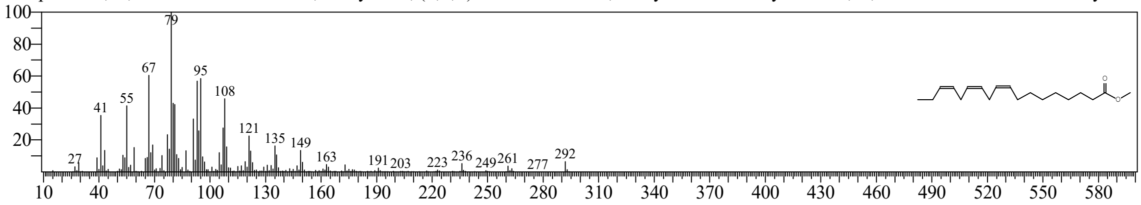

<< Target >>
Line#:14 R.Time:30.292(Scan#:2436) MassPeaks:322
RawMode:Averaged 30.283-30.300(2435-2437) BasePeak:79.05(4979)
BG Mode:Calc. from Peak Group 1 - Event 1

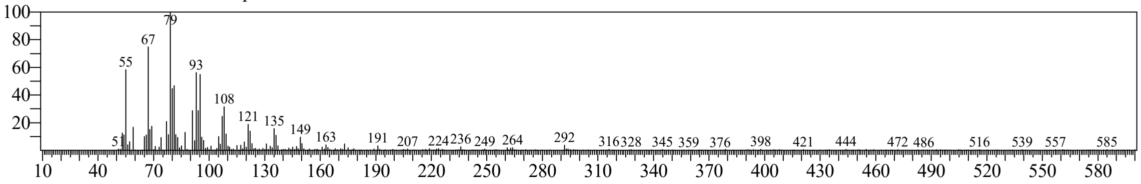


Hit#:1 Entry:135724 Library:NIST17-1.lib
SI:96 Formula:C19H32O2 CAS:301-00-8 MolWeight:292 RetIndex:2101
CompName:9,12,15-Octadecatrienoic acid, methyl ester, (Z,Z,Z)- $$ Linolenic acid, methyl ester $$ Methyl all-cis-9,12,15-octadecatrienoate $$ Methyl lino

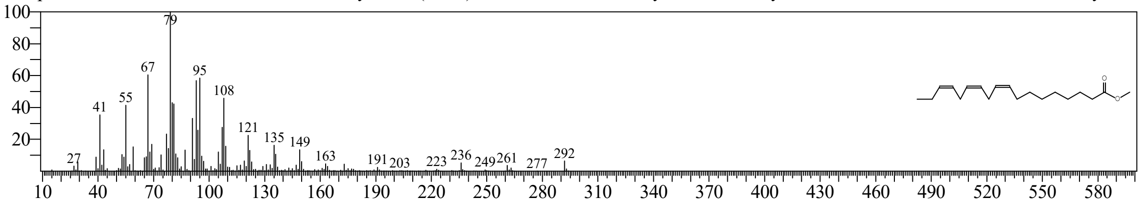


Hit#:2 Entry:124228 Library:NIST14.lib
SI:96 Formula:C19H32O2 CAS:301-00-8 MolWeight:292 RetIndex:2101
CompName:9,12,15-Octadecatrienoic acid, methyl ester, (Z,Z,Z)- $$ Linolenic acid, methyl ester $$ Methyl all-cis-9,12,15-octadecatrienoate $$ Methyl lino

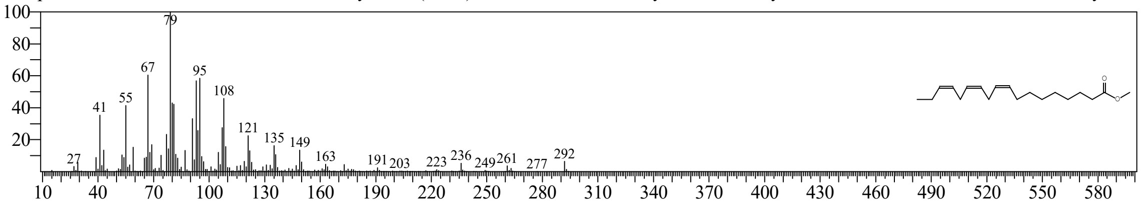


Hit#:3 Entry:111654 Library:NIST14.lib
SI:95 Formula:C18H30O2 CAS:0-00-0 MolWeight:278 RetIndex:2002
CompName:Methyl 8,11,14-heptadecatrienoate

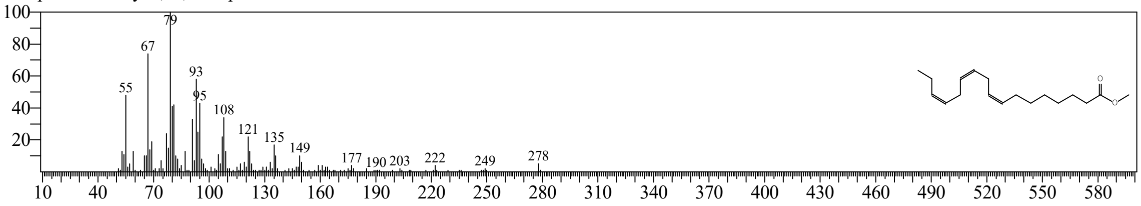


Hit#:4 Entry:32437 Library:NIST17s.lib
SI:95 Formula:C19H32O2 CAS:301-00-8 MolWeight:292 RetIndex:2101
CompName:9,12,15-Octadecatrienoic acid, methyl ester, (Z,Z,Z)- $$ Linolenic acid, methyl ester $$ Methyl all-cis-9,12,15-octadecatrienoate $$ Methyl lino

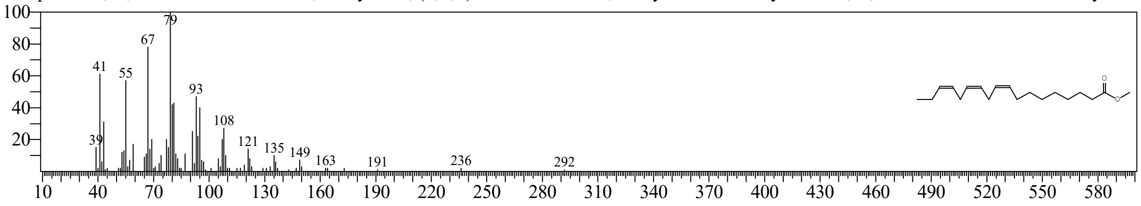


Hit#:5 Entry:27872 Library:NIST14s.lib
SI:95 Formula:C19H32O2 CAS:301-00-8 MolWeight:292 RetIndex:2101
CompName:9,12,15-Octadecatrienoic acid, methyl ester, (Z,Z,Z)- $$ Linolenic acid, methyl ester $$ Methyl all-cis-9,12,15-octadecatrienoate $$ Methyl lino

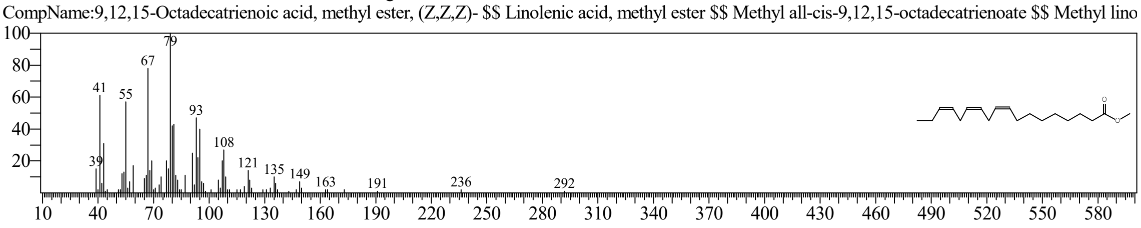

<< Target >>
Line#:15 R.Time:30.592(Scan#:2472) MassPeaks:396
RawMode:Averaged 30.583-30.600(2471-2473) BasePeak:79.05(53920)
BG Mode:Calc. from Peak Group 1 - Event 1

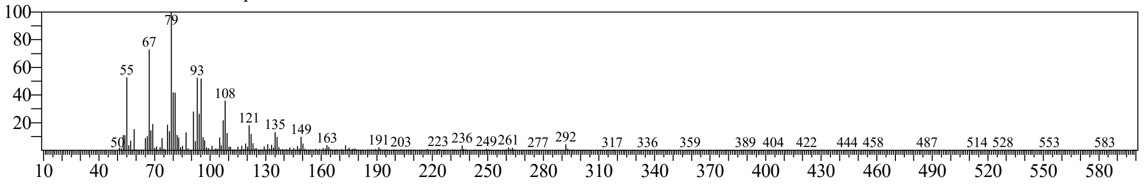


Hit#:1 Entry:135724 Library:NIST17-1.lib
SI:96 Formula:C19H32O2 CAS:301-00-8 MolWeight:292 RetIndex:2101
CompName:9,12,15-Octadecatrienoic acid, methyl ester, (Z,Z,Z)- $$ Linolenic acid, methyl ester $$ Methyl all-cis-9,12,15-octadecatrienoate $$ Methyl lino

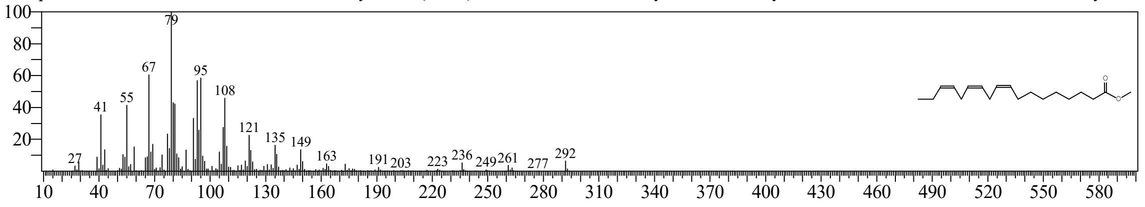


Hit#:2 Entry:124228 Library:NIST14.lib
SI:96 Formula:C19H32O2 CAS:301-00-8 MolWeight:292 RetIndex:2101
CompName:9,12,15-Octadecatrienoic acid, methyl ester, (Z,Z,Z)- $$ Linolenic acid, methyl ester $$ Methyl all-cis-9,12,15-octadecatrienoate $$ Methyl lino

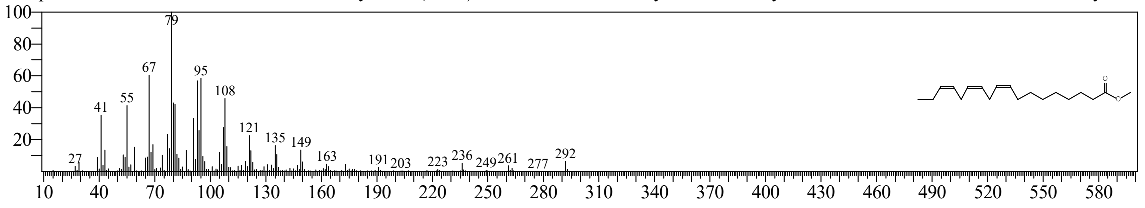


Hit#:3 Entry:32438 Library:NIST17s.lib
SI:95 Formula:C19H32O2 CAS:301-00-8 MolWeight:292 RetIndex:2101
CompName:9,12,15-Octadecatrienoic acid, methyl ester, (Z,Z,Z)- $$ Linolenic acid, methyl ester $$ Methyl all-cis-9,12,15-octadecatrienoate $$ Methyl lino

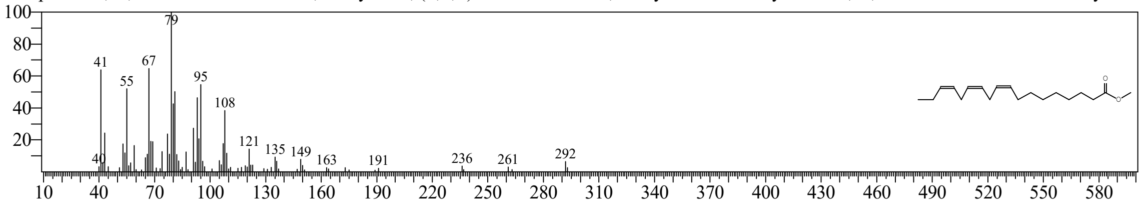


Hit#:4 Entry:27872 Library:NIST14s.lib
SI:95 Formula:C19H32O2 CAS:301-00-8 MolWeight:292 RetIndex:2101
CompName:9,12,15-Octadecatrienoic acid, methyl ester, (Z,Z,Z)- $$ Linolenic acid, methyl ester $$ Methyl all-cis-9,12,15-octadecatrienoate $$ Methyl lino

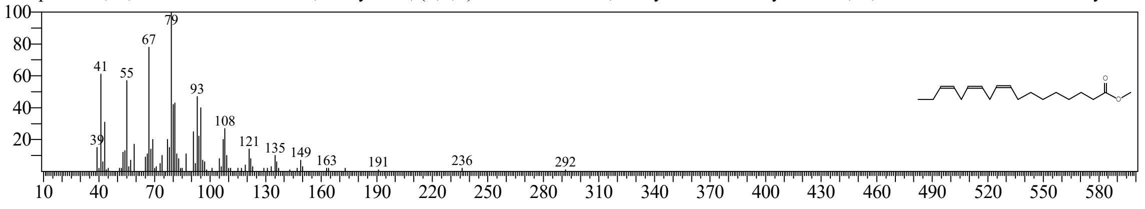


Hit#:5 Entry:121536 Library:NIST17-1.lib
SI:95 Formula:C18H30O2 CAS:0-00-0 MolWeight:278 RetIndex:2002
CompName:Methyl 8,11,14-heptadecatrienoate

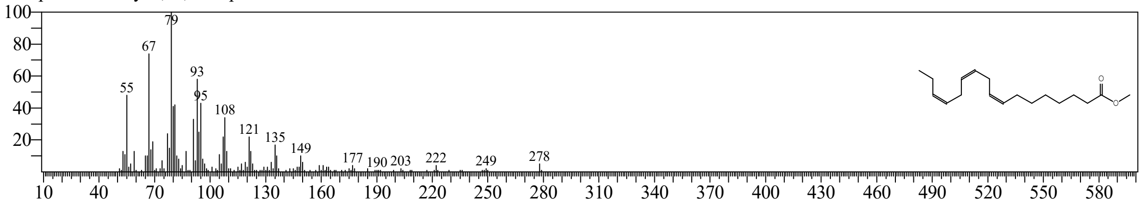

<< Target >>
Line#:16 R.Time:30.933(Scan#:2513) MassPeaks:350
RawMode:Averaged 30.925-30.942(2512-2514) BasePeak:67.05(16551)
BG Mode:Calc. from Peak Group 1 - Event 1

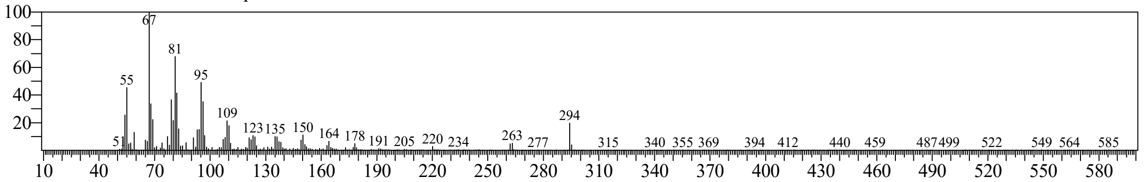


Hit#:1 Entry:125932 Library:NIST14.lib
SI:97 Formula:C19H34O2 CAS:0-00-0 MolWeight:294 RetIndex:2093
CompName:Methyl 9-cis,11-trans-octadecadienoate

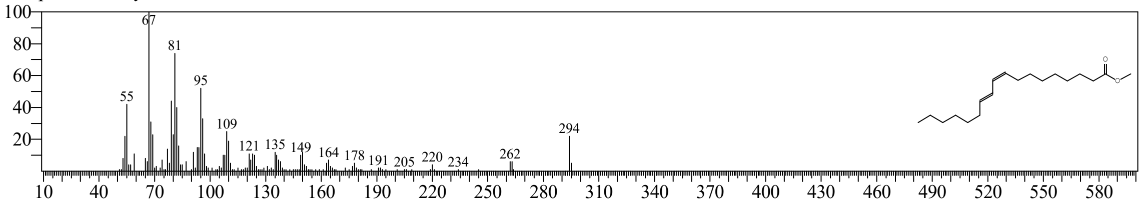


Hit#:2 Entry:137666 Library:NIST17-1.lib
SI:97 Formula:C19H34O2 CAS:13058-52-1 MolWeight:294 RetIndex:2093
CompName:Methyl 9-cis,11-trans-octadecadienoate

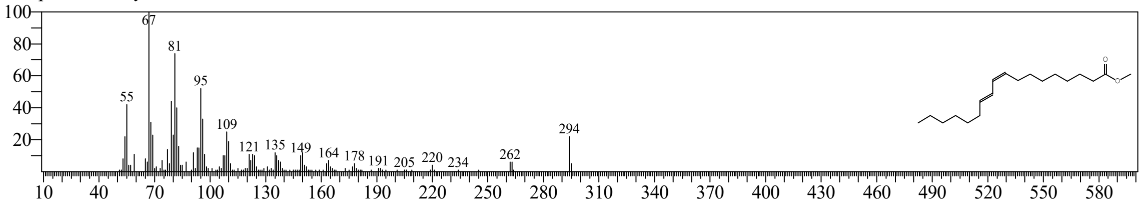


Hit#:3 Entry:137664 Library:NIST17-1.lib
SI:97 Formula:C19H34O2 CAS:0-00-0 MolWeight:294 RetIndex:2093
CompName:Methyl 10-trans,12-cis-octadecadienoate

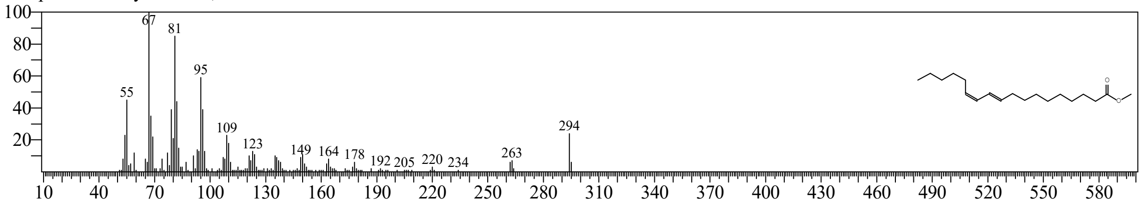


Hit#:4 Entry:125933 Library:NIST14.lib
SI:97 Formula:C19H34O2 CAS:0-00-0 MolWeight:294 RetIndex:2093
CompName:Methyl 10-trans,12-cis-octadecadienoate

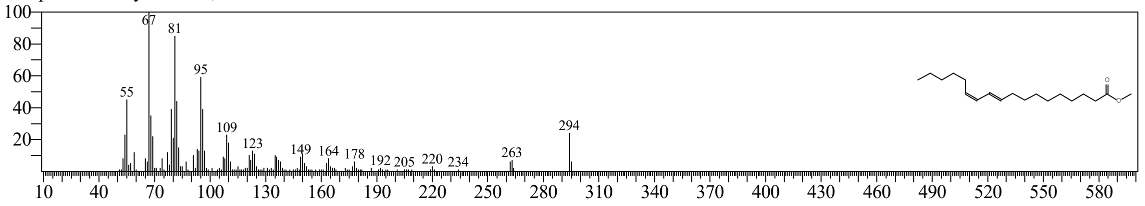


Hit#:5 Entry:32587 Library:NIST17s.lib
SI:96 Formula:C19H34O2 CAS:13038-47-6 MolWeight:294 RetIndex:2093
CompName:9,11-Octadecadienoic acid, methyl ester, (E,E)- $$ Methyl trans-9,trans-11-octadecadienoate $$ Methyl trans,trans-9,11-octadecadienoate $$ M

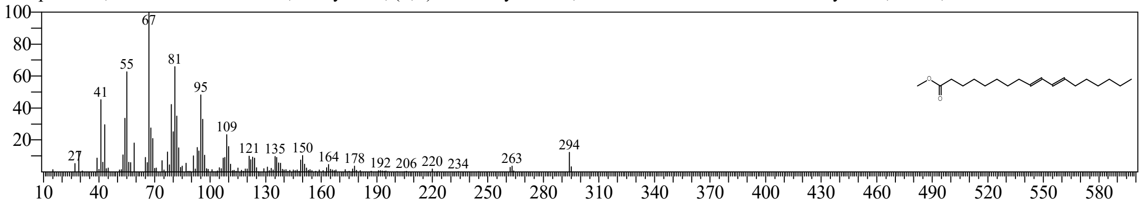

<< Target >>
Line#:17  R.Time:31.258(Scan#:2552)  MassPeaks:406
RawMode:Averaged 31.250-31.267(2551-2553) BasePeak:67.05(18000)
BG Mode:Calc. from Peak  Group 1 - Event 1

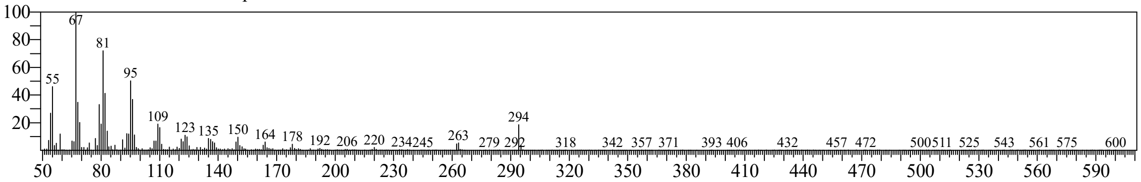


Hit#:1  Entry:125933  Library:NIST14.lib
SI:97  Formula:C19H34O2  CAS:0-00-0  MolWeight:294  RetIndex:2093
CompName:Methyl 10-trans,12-cis-octadecadienoate

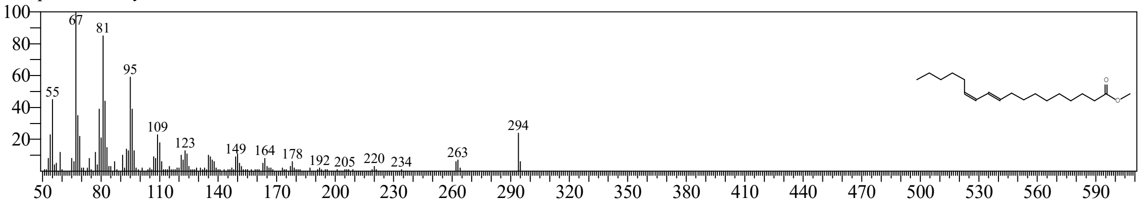


Hit#:2  Entry:137664  Library:NIST17-1.lib
SI:97  Formula:C19H34O2  CAS:0-00-0  MolWeight:294  RetIndex:2093
CompName:Methyl 10-trans,12-cis-octadecadienoate

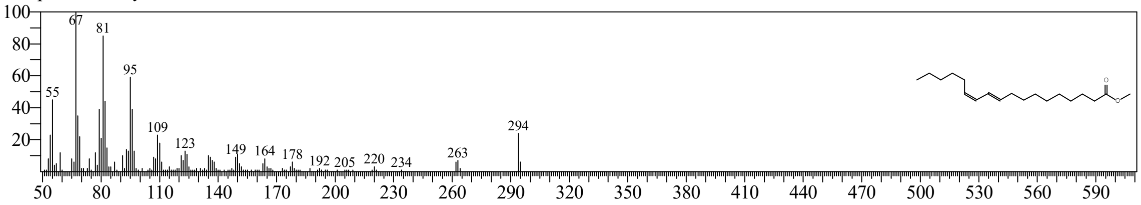


Hit#:3  Entry:125932  Library:NIST14.lib
SI:96  Formula:C19H34O2  CAS:0-00-0  MolWeight:294  RetIndex:2093
CompName:Methyl 9-cis,11-trans-octadecadienoate

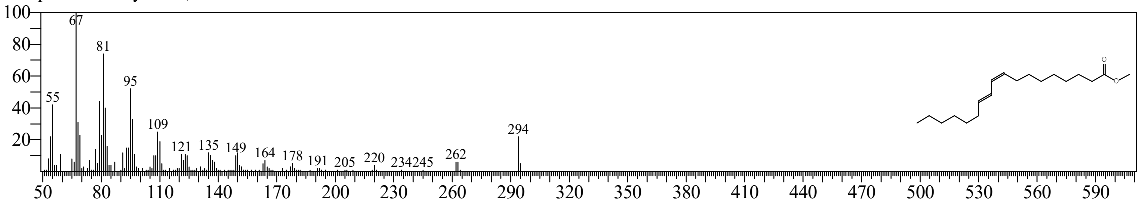


Hit#:4  Entry:137666  Library:NIST17-1.lib
SI:96  Formula:C19H34O2  CAS:13058-52-1  MolWeight:294  RetIndex:2093
CompName:Methyl 9-cis,11-trans-octadecadienoate

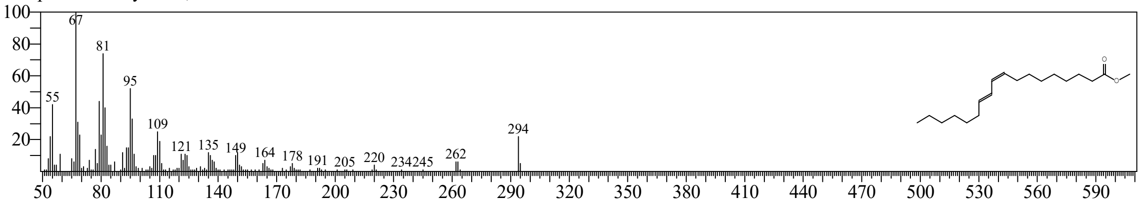


Hit#:5  Entry:32592  Library:NIST17s.lib
SI:95  Formula:C19H34O2  CAS:57156-97-5  MolWeight:294  RetIndex:2093
CompName:12,15-Octadecadienoic acid, methyl ester $$ Methyl (12E,15E)-12,15-octadecadienoate # $$

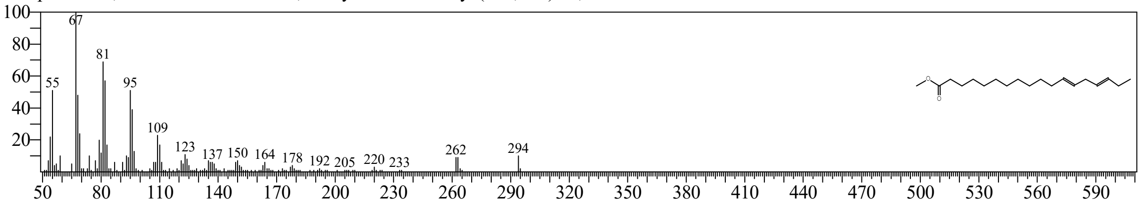

<< Target >>
Line#:18   R.Time:31.917(Scan#:2631)  MassPeaks:310
RawMode:Averaged 31.908-31.925(2630-2632) BasePeak:67.10(1069)
BG Mode:Calc. from Peak  Group 1 - Event 1

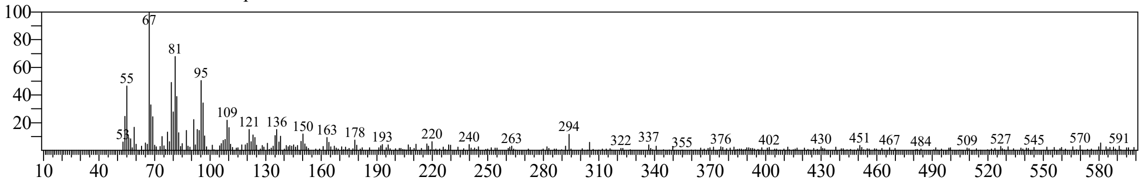


Hit#:1  Entry:125932  Library:NIST14.lib
SI:93  Formula:C19H34O2  CAS:0-00-0  MolWeight:294  RetIndex:2093
CompName:Methyl 9-cis,11-trans-octadecadienoate

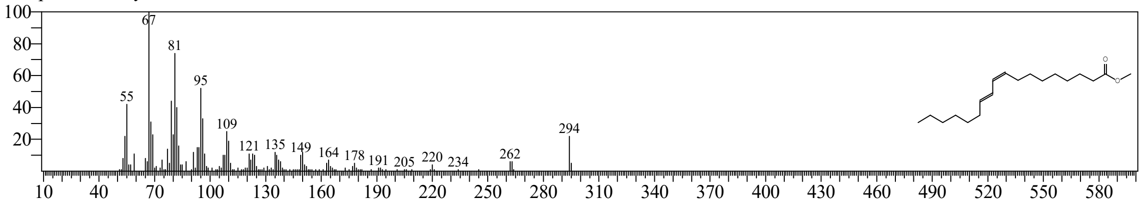


Hit#:2  Entry:137666  Library:NIST17-1.lib
SI:93  Formula:C19H34O2  CAS:13058-52-1  MolWeight:294  RetIndex:2093
CompName:Methyl 9-cis,11-trans-octadecadienoate

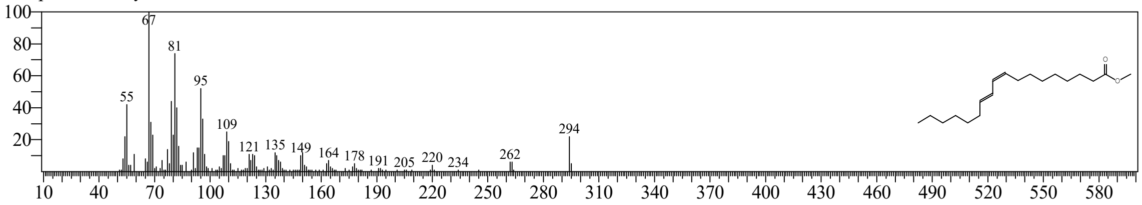


Hit#:3  Entry:32587  Library:NIST17s.lib
SI:92  Formula:C19H34O2  CAS:13038-47-6  MolWeight:294  RetIndex:2093
CompName:9,11-Octadecadienoic acid, methyl ester, (E,E)- $$ Methyl trans-9,trans-11-octadecadienoate $$ Methyl trans,trans-9,11-octadecadienoate $$ M

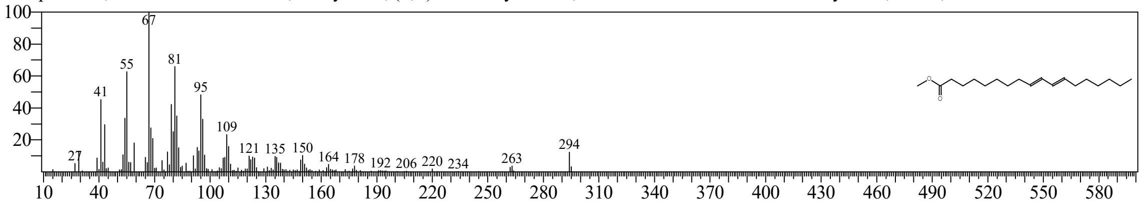


Hit#:4  Entry:137664  Library:NIST17-1.lib
SI:91  Formula:C19H34O2  CAS:0-00-0  MolWeight:294  RetIndex:2093
CompName:Methyl 10-trans,12-cis-octadecadienoate

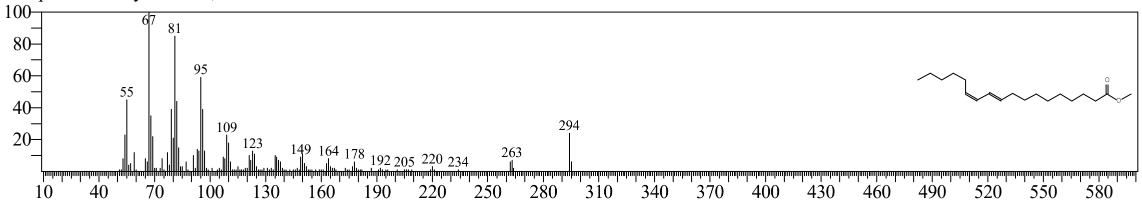


Hit#:5  Entry:125933  Library:NIST14.lib
SI:91  Formula:C19H34O2  CAS:0-00-0  MolWeight:294  RetIndex:2093
CompName:Methyl 10-trans,12-cis-octadecadienoate

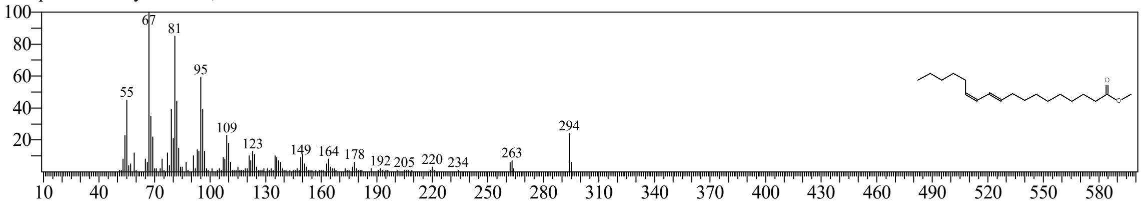

<< Target >>
Line#:19 R.Time:32.192(Scan#:2664) MassPeaks:346
RawMode:Averaged 32.183-32.200(2663-2665) BasePeak:79.05(701)
BG Mode:Calc. from Peak Group 1 - Event 1

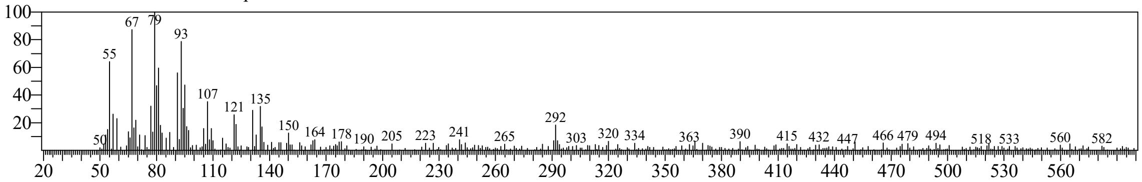


Hit#:1 Entry:164425 Library:NIST17-1.lib
SI:87 Formula:C21H36O2 CAS:30223-51-9 MolWeight:320 RetIndex:2300
CompName:7,10,13-Eicosatrienoic acid, methyl ester $$ Methyl eicosa-7,10,13-trienoate $$ Methyl (7E,10E,13E)-7,10,13-icosatrienoate # $$

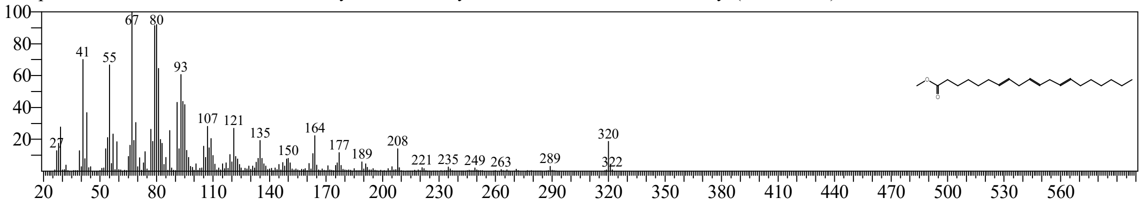


Hit#:2 Entry:149524 Library:NIST14.lib
SI:87 Formula:C21H36O2 CAS:30223-51-9 MolWeight:320 RetIndex:2300
CompName:7,10,13-Eicosatrienoic acid, methyl ester $$ Methyl eicosa-7,10,13-trienoate $$ Methyl (7E,10E,13E)-7,10,13-icosatrienoate # $$

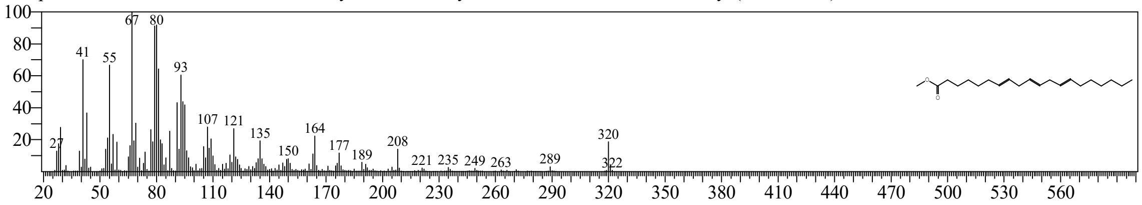


Hit#:3 Entry:29611 Library:NIST14s.lib
SI:87 Formula:C21H36O2 CAS:17364-32-8 MolWeight:320 RetIndex:2300
CompName:8,11,14-Eicosatrienoic acid, methyl ester $$ Methyl (8E,11E,14E)-8,11,14-icosatrienoate # $$

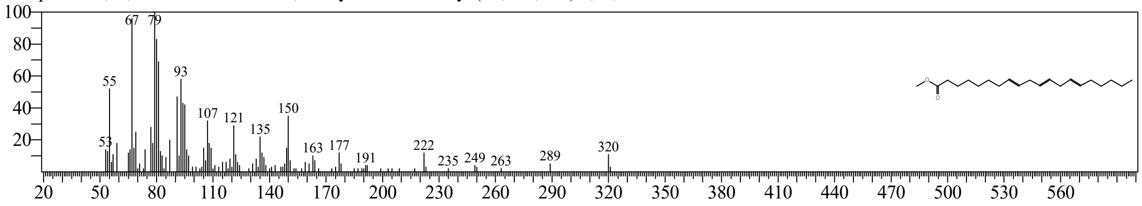


Hit#:4 Entry:34364 Library:NIST17s.lib
SI:87 Formula:C21H36O2 CAS:17364-32-8 MolWeight:320 RetIndex:2300
CompName:8,11,14-Eicosatrienoic acid, methyl ester $$ Methyl (8E,11E,14E)-8,11,14-icosatrienoate # $$

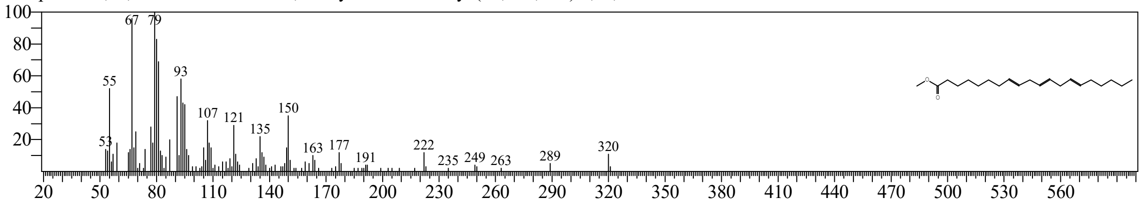


Hit#:5 Entry:164434 Library:NIST17-1.lib
SI:87 Formula:C21H36O2 CAS:21061-10-9 MolWeight:320 RetIndex:2300
CompName:8,11,14-Eicosatrienoic acid, methyl ester, (Z,Z,Z)- $$ Methyl dihomo-.gamma.-linolenate $$ Methyl (8Z,11Z,14Z)-8,11,14-icosatrienoate # $$ I

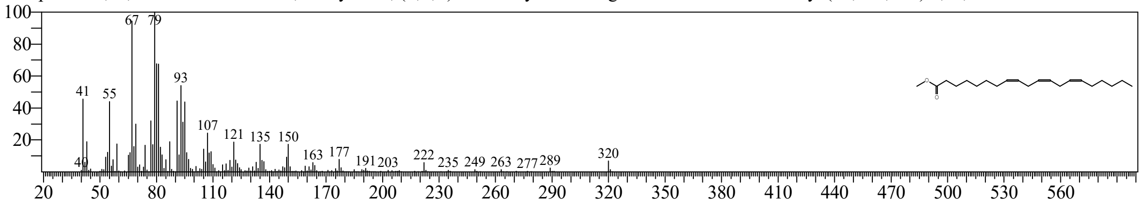

<< Target >>
Line#:20  R.Time:32.950(Scan#:2755)  MassPeaks:393
RawMode:Averaged 32.942-32.958(2754-2756) BasePeak:95.05(19520)
BG Mode:Calc. from Peak  Group 1 - Event 1

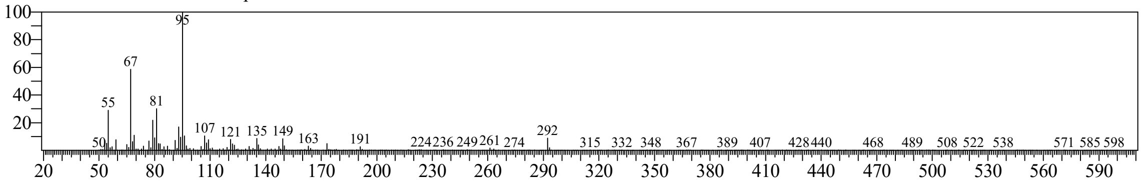


Hit#:1  Entry:24424  Library:NIST17-1.lib
SI:85  Formula:C12H20  CAS:0-00-0  MolWeight:164  RetIndex:1041
CompName:Cis-3-ethyl-endo-tricyclo[5.2.1.0(2.6)]decane

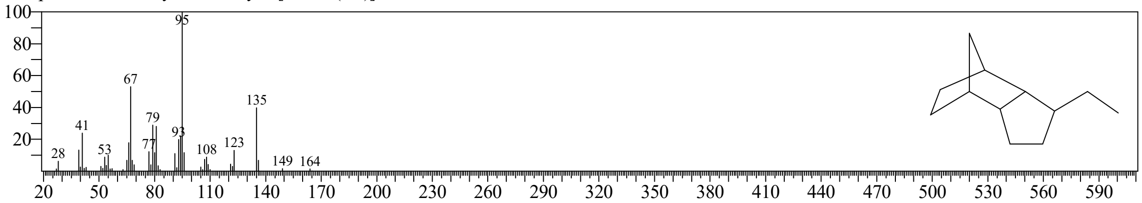


Hit#:2  Entry:23403  Library:NIST14.lib
SI:85  Formula:C12H20  CAS:0-00-0  MolWeight:164  RetIndex:1041
CompName:Cis-3-ethyl-endo-tricyclo[5.2.1.0(2.6)]decane

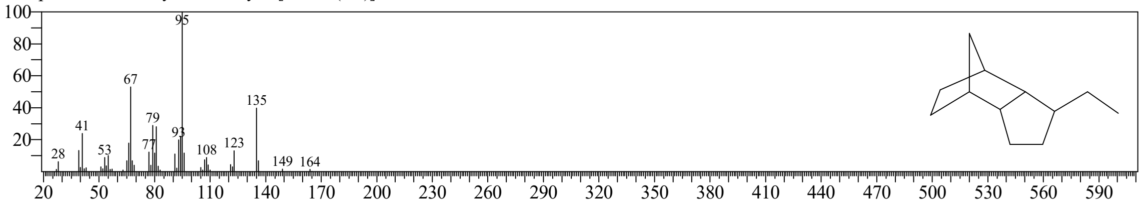


Hit#:3  Entry:16582  Library:NIST17-1.lib
SI:84  Formula:C11H18  CAS:0-00-0  MolWeight:150  RetIndex:1115
CompName:4-Cyclohexenyl-3-methylbutene

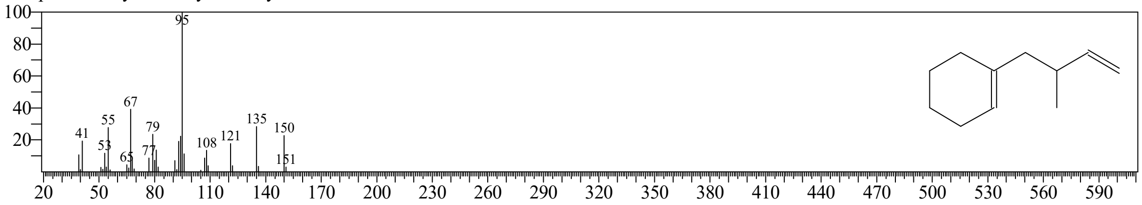


Hit#:4  Entry:17571  Library:NIST17-1.lib
SI:83  Formula:C10H16O  CAS:60485-45-2  MolWeight:152  RetIndex:991
CompName:Santolina epoxide $$ 2-Methyl-2-(3-methyl-1-vinyl-2-butenyl)oxirane # $$

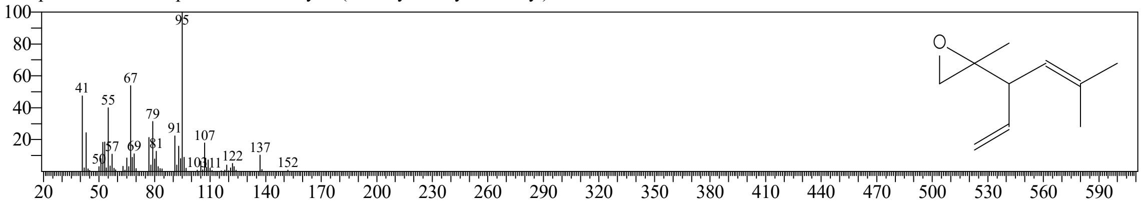


Hit#:5  Entry:16897  Library:NIST14.lib
SI:83  Formula:C10H16O  CAS:60485-45-2  MolWeight:152  RetIndex:991
CompName:Santolina epoxide $$ 2-Methyl-2-(3-methyl-1-vinyl-2-butenyl)oxirane # $$

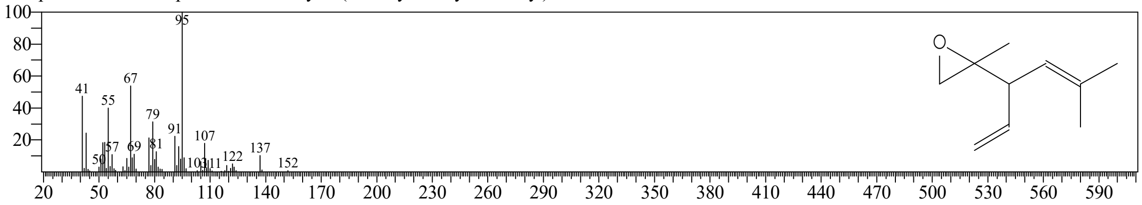

<< Target >>
Line#:21  R.Time:33.483(Scan#:2819)  MassPeaks:358
RawMode:Averaged 33.475-33.492(2818-2820) BasePeak:74.00(12109)
BG Mode:Calc. from Peak  Group 1 - Event 1

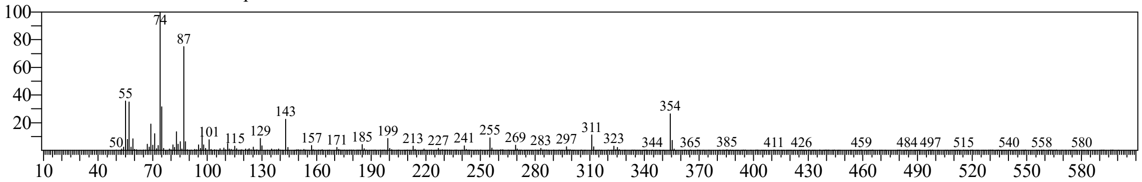


Hit#:1  Entry:196443  Library:NIST17-1.lib
SI:96  Formula:C23H46O2  CAS:929-77-1  MolWeight:354  RetIndex:2475
CompName:Docosanoic acid, methyl ester $$ Behenic acid, methyl ester $$ Methyl behenate $$ Methyl docosanoate $$ n-Docosanoic acid methyl ester $$

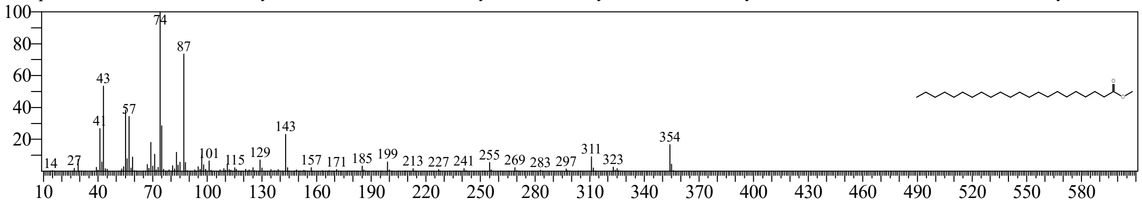


Hit#:2  Entry:178051  Library:NIST14.lib
SI:96  Formula:C23H46O2  CAS:929-77-1  MolWeight:354  RetIndex:2475
CompName:Docosanoic acid, methyl ester $$ Behenic acid, methyl ester $$ Methyl behenate $$ Methyl docosanoate $$ n-Docosanoic acid methyl ester $$

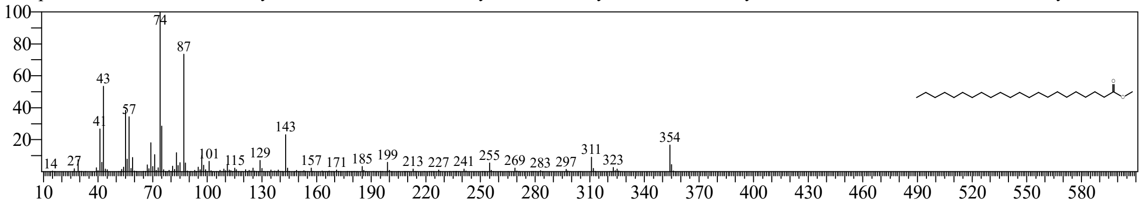


Hit#:3  Entry:196444  Library:NIST17-1.lib
SI:95  Formula:C23H46O2  CAS:0-00-0  MolWeight:354  RetIndex:2411
CompName:Methyl 20-methyl-heneicosanoate

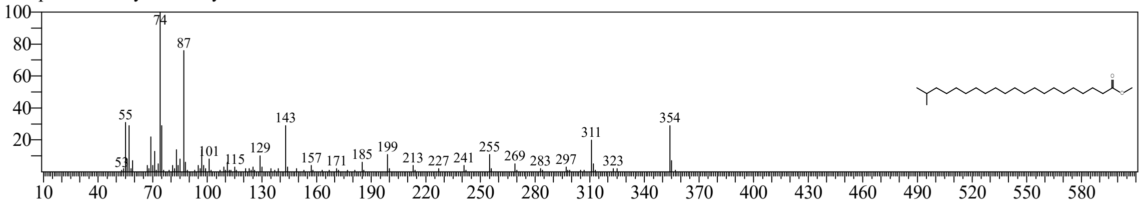


Hit#:4  Entry:178052  Library:NIST14.lib
SI:95  Formula:C23H46O2  CAS:0-00-0  MolWeight:354  RetIndex:2411
CompName:Methyl 20-methyl-heneicosanoate

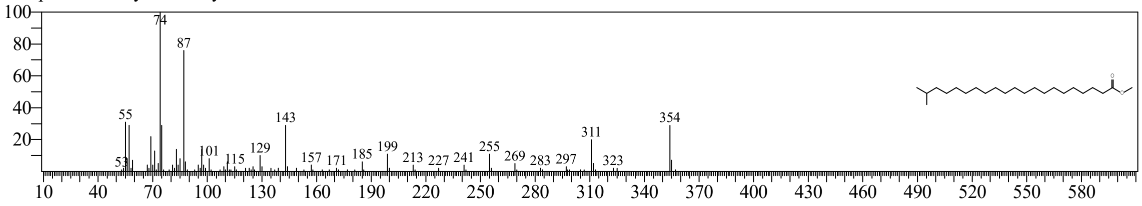


Hit#:5  Entry:31088  Library:NIST14s.lib
SI:95  Formula:C23H46O2  CAS:929-77-1  MolWeight:354  RetIndex:2475
CompName:Docosanoic acid, methyl ester $$ Behenic acid, methyl ester $$ Methyl behenate $$ Methyl docosanoate $$ n-Docosanoic acid methyl ester $$

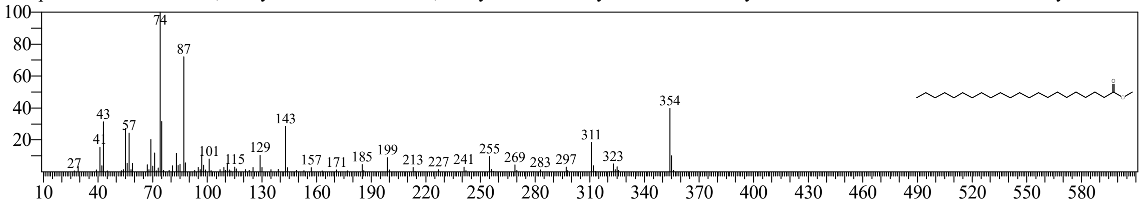

<< Target >>
Line#:22 R.Time:37.800(Scan#:3337) MassPeaks:319
RawMode:Averaged 37.792-37.808(3336-3338) BasePeak:74.00(3002)
BG Mode:Calc. from Peak Group 1 - Event 1

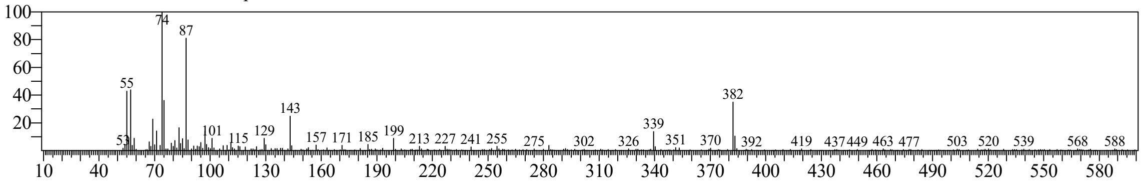


Hit#:1 Entry:37106 Library:NIST17s.lib
SI:92 Formula:C25H50O2 CAS:2442-49-1 MolWeight:382 RetIndex:2674
CompName:Tetracosanoic acid, methyl ester $$ Methyl lignocerate $$ Methyl tetracosanoate $$ Lignoceric acid methyl ester $$

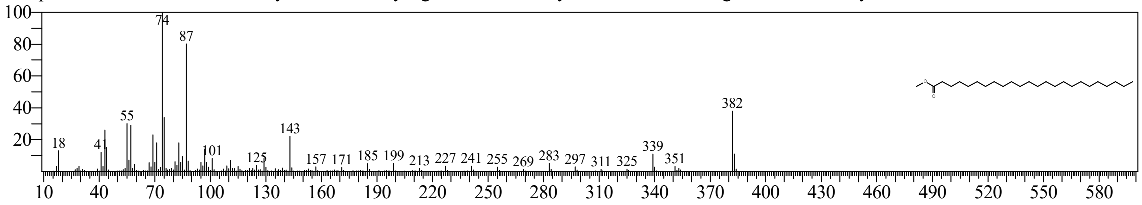


Hit#:2 Entry:31970 Library:NIST14s.lib
SI:92 Formula:C25H50O2 CAS:2442-49-1 MolWeight:382 RetIndex:2674
CompName:Tetracosanoic acid, methyl ester $$ Methyl lignocerate $$ Methyl tetracosanoate $$ Lignoceric acid methyl ester $$

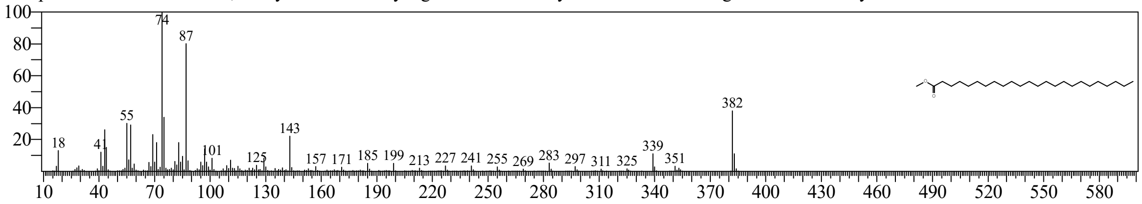


Hit#:3 Entry:37107 Library:NIST17s.lib
SI:91 Formula:C25H50O2 CAS:2442-49-1 MolWeight:382 RetIndex:2674
CompName:Tetracosanoic acid, methyl ester $$ Methyl lignocerate $$ Methyl tetracosanoate $$ Lignoceric acid methyl ester $$

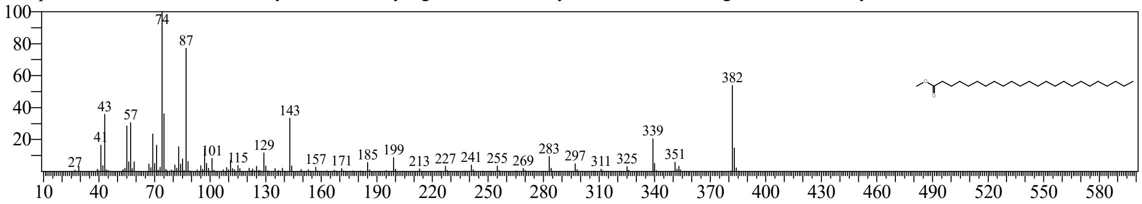


Hit#:4 Entry:31971 Library:NIST14s.lib
SI:91 Formula:C25H50O2 CAS:2442-49-1 MolWeight:382 RetIndex:2674
CompName:Tetracosanoic acid, methyl ester $$ Methyl lignocerate $$ Methyl tetracosanoate $$ Lignoceric acid methyl ester $$

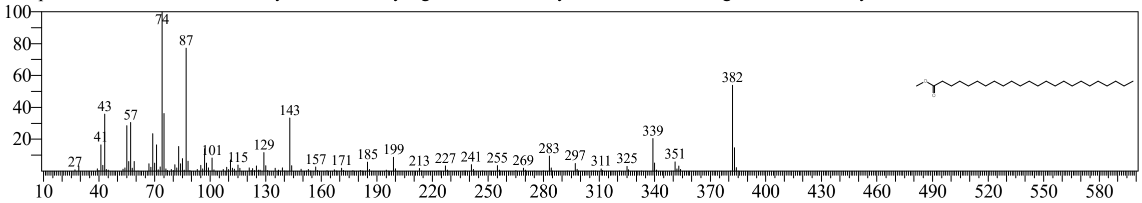


Hit#:5 Entry:31969 Library:NIST14s.lib
SI:89 Formula:C25H50O2 CAS:2442-49-1 MolWeight:382 RetIndex:2674
CompName:Tetracosanoic acid, methyl ester $$ Methyl lignocerate $$ Methyl tetracosanoate $$ Lignoceric acid methyl ester $$

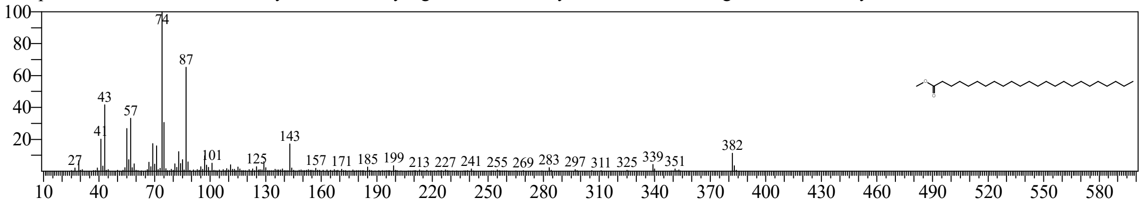

Supplement: Supplementary file 1 [file molecules-31-02891-s001.zip › 14d-QG200-1.pdf]
