# Supplementary material for: Selective Inhibition of Lipid Oxidation by a Quercetagetin-Rich Extract from Tagetes erecta: A Chemometric Approach Using OPLS-DA and Selectivity Index Analysis
Source: Molecules. 2026 Aug 19;31(16):2891. doi: 10.3390/molecules31162891 (PMC13515886; doi:10.3390/molecules31162891)

# CK-3-28d

<<Column>>
Name : HP-88
Thickness : 0.20um
Length : 100.0m
Inside Diameter : 0.25mm

## Method

===== Analytical Line 1 =====

| | | |
|---|---|---|
| Column Oven Temp. | :120.0 °C | |
| Injection Temp. | :250.00 °C | |
| Injection Mode | :Split | |
| Column Flow | :1.04 mL/min | |
| Split Ratio | :20.0 | |
| Oven Temp. Program | | |
| Rate | Temperature(°C) | Hold Time(min) |
| - | 120.0 | 1.00 |
| 10.00 | 175.0 | 10.00 |
| 5.00 | 210.0 | 5.00 |
| 5.00 | 230.0 | 15.00 |

[GCMS-QP2010]
IonSourceTemp :200.00 °C
Interface Temp. :220.00 °C
*m/z* scanning range：45～500m/z

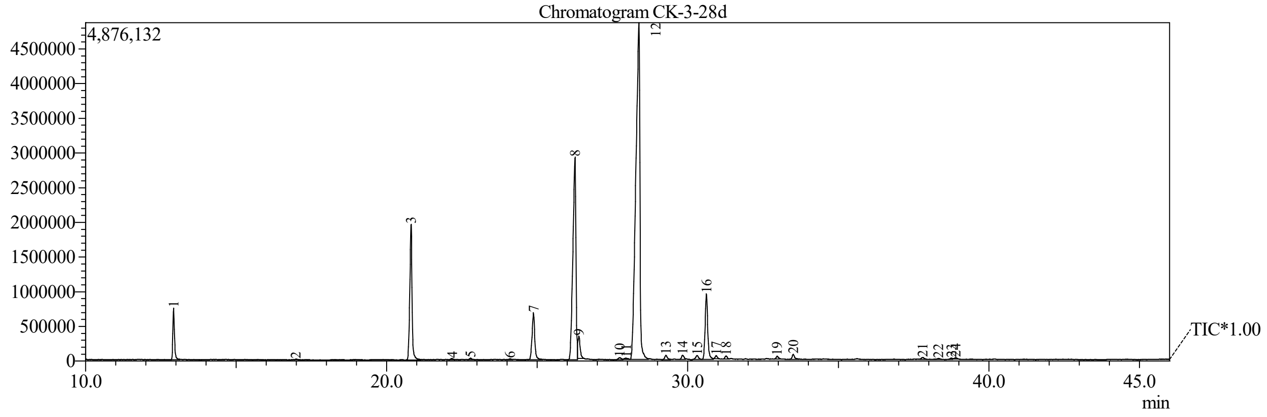


Peak Report TIC

| Peak# | R.Time | Area | Area% | Height | Height% | A/H | Name |
|---|---|---|---|---|---|---|---|
| 1 | 12.926 | 2789876 | 2.98 | 743255 | 5.72 | 3.75 | Undecanoic acid, methyl ester |
| 2 | 16.986 | 58440 | 0.06 | 13083 | 0.10 | 4.47 | Methyl tetradecanoate |
| 3 | 20.813 | 10093836 | 10.79 | 1954478 | 15.04 | 5.16 | Hexadecanoic acid, methyl ester |
| 4 | 22.176 | 69430 | 0.07 | 15321 | 0.12 | 4.53 | 7-Hexadecenoic acid, methyl ester, (Z)- |
| 5 | 22.787 | 111685 | 0.12 | 20785 | 0.16 | 5.37 | Heptadecanoic acid, methyl ester |
| 6 | 24.097 | 56012 | 0.06 | 10555 | 0.08 | 5.31 | Methyl 9-heptadecenoate or 9-17:1 |
| 7 | 24.875 | 4171084 | 4.46 | 677000 | 5.21 | 6.16 | Methyl stearate |
| 8 | 26.255 | 20431349 | 21.84 | 2921810 | 22.48 | 6.99 | 9-Octadecenoic acid, methyl ester, (E)- |
| 9 | 26.385 | 1615704 | 1.73 | 322890 | 2.48 | 5.00 | trans-13-Octadecenoic acid, methyl ester |
| 10 | 27.739 | 200841 | 0.21 | 28607 | 0.22 | 7.02 | 9,12-Octadecadienoic acid, methyl ester |
| 11 | 27.948 | 159506 | 0.17 | 19996 | 0.15 | 7.98 | 9,12-Octadecadienoic acid, methyl ester |
| 12 | 28.379 | 45447784 | 48.59 | 4852626 | 37.34 | 9.37 | 9,12-Octadecadienoic acid (Z,Z)-, methyl ester |
| 13 | 29.275 | 372438 | 0.40 | 62085 | 0.48 | 6.00 | Methyl 18-methylnonadecanoate |
| 14 | 29.825 | 358653 | 0.38 | 62073 | 0.48 | 5.78 | 9,12,15-Octadecatrienoic acid, methyl ester, (Z |

| Peak# | R.Time | Area | Area% | Height | Height% | A/H | Name |
|---|---|---|---|---|---|---|---|
| 15 | 30.309 | 367281 | 0.39 | 56983 | 0.44 | 6.45 | 9,12,15-Octadecatrienoic acid, methyl ester, (Z |
| 16 | 30.618 | 5649637 | 6.04 | 947598 | 7.29 | 5.96 | 9,12,15-Octadecatrienoic acid, methyl ester, (Z |
| 17 | 30.942 | 200402 | 0.21 | 44532 | 0.34 | 4.50 | Methyl 9-cis,11-trans-octadecadienoate |
| 18 | 31.272 | 251423 | 0.27 | 45492 | 0.35 | 5.53 | Methyl 10-trans,12-cis-octadecadienoate |
| 19 | 32.966 | 235315 | 0.25 | 44014 | 0.34 | 5.35 | Cis-3-ethyl-endo-tricyclo[5.2.1.0(2.6)]decane |
| 20 | 33.502 | 426350 | 0.46 | 71619 | 0.55 | 5.95 | Docosanoic acid, methyl ester |
| 21 | 37.794 | 185610 | 0.20 | 27060 | 0.21 | 6.86 | Tetracosanoic acid, methyl ester |
| 22 | 38.324 | 56833 | 0.06 | 12004 | 0.09 | 4.73 | Methyl 9.cis.,11.trans.t,13.trans.-octadecatrien |
| 23 | 38.759 | 106001 | 0.11 | 20413 | 0.16 | 5.19 | Methyl 9.cis.,11.trans.t,13.trans.-octadecatrien |
| 24 | 38.895 | 115845 | 0.12 | 21503 | 0.17 | 5.39 | Methyl 9.cis.,11.trans.t,13.trans.-octadecatrien |
| | | 93531335 | 100.00 | 12995782 | 100.00 | | |

Library

<< Target >>
Line#:1 R.Time:12.925(Scan#:352) MassPeaks:371
RawMode:Averaged 12.917-12.933(351-353) BasePeak:74.00(219249)
BG Mode:Calc. from Peak Group 1 - Event 1

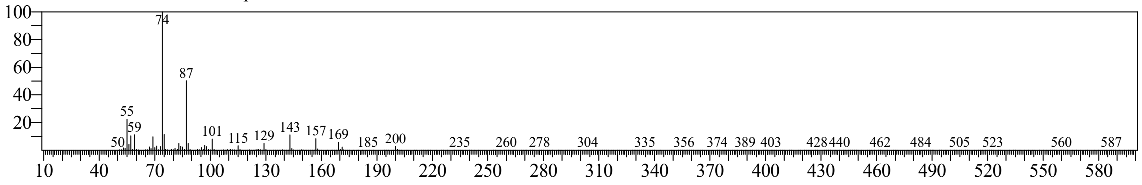


Hit#:1 Entry:46966 Library:NIST14.lib
SI:95 Formula:C12H24O2 CAS:1731-86-8 MolWeight:200 RetIndex:1381
CompName:Undecanoic acid, methyl ester $$ Methyl undecanoate $$ Methyl undecylenate $$ n-Undecanoic acid methyl ester $$ Methyl ester of undecanoi

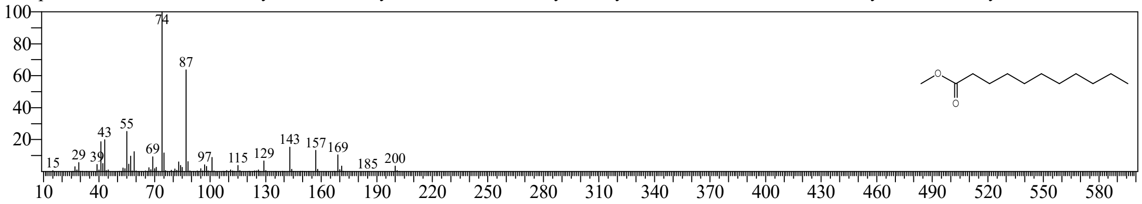


Hit#:2 Entry:49598 Library:NIST17-1.lib
SI:95 Formula:C12H24O2 CAS:1731-86-8 MolWeight:200 RetIndex:1381
CompName:Undecanoic acid, methyl ester $$ Methyl undecanoate $$ Methyl undecylenate $$ n-Undecanoic acid methyl ester $$ Methyl ester of undecanoi

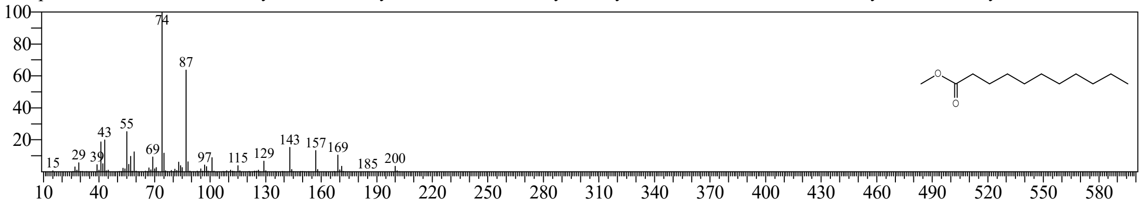


Hit#:3 Entry:21618 Library:NIST17s.lib
SI:95 Formula:C12H24O2 CAS:1731-86-8 MolWeight:200 RetIndex:1381
CompName:Undecanoic acid, methyl ester $$ Methyl undecanoate $$ Methyl undecylenate $$ n-Undecanoic acid methyl ester $$ Methyl ester of undecanoi

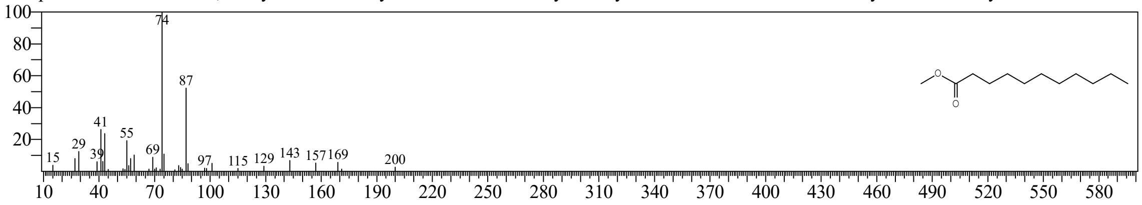


Hit#:4 Entry:18487 Library:NIST14s.lib
SI:95 Formula:C12H24O2 CAS:1731-86-8 MolWeight:200 RetIndex:1381
CompName:Undecanoic acid, methyl ester $$ Methyl undecanoate $$ Methyl undecylenate $$ n-Undecanoic acid methyl ester $$ Methyl ester of undecanoi

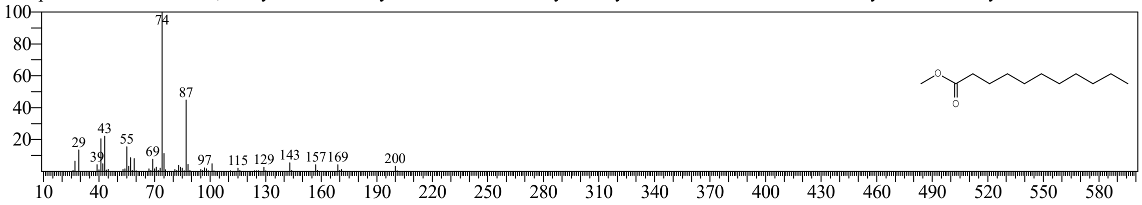

<< Target >>
Line#:1   R.Time:12.925(Scan#:352)  MassPeaks:371
RawMode:Averaged 12.917-12.933(351-353) BasePeak:74.00(219249)
BG Mode:Calc. from Peak  Group 1 - Event 1

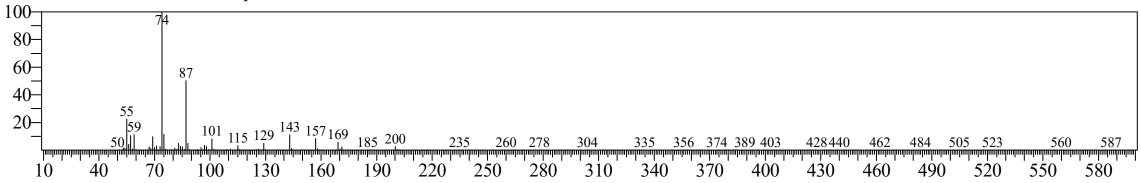


Hit#:5  Entry:21619  Library:NIST17s.lib
SI:95  Formula:C12H24O2  CAS:1731-86-8  MolWeight:200  RetIndex:1381
CompName:Undecanoic acid, methyl ester $$ Methyl undecanoate $$ Methyl undecylenate $$ n-Undecanoic acid methyl ester $$ Methyl ester of undecanoi

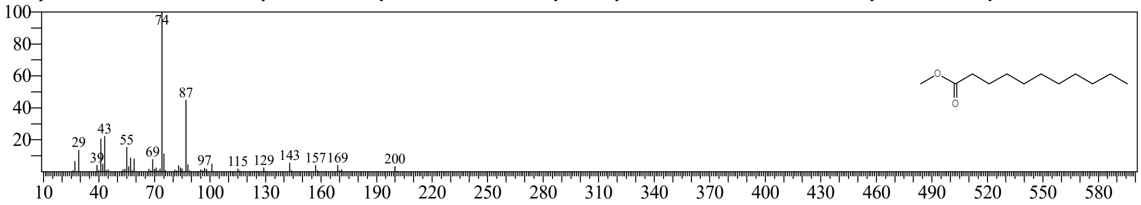

<< Target >>
Line#:2 R.Time:16.983(Scan#:839) MassPeaks:285
RawMode:Averaged 16.975-16.992(838-840) BasePeak:74.05(2519)
BG Mode:Calc. from Peak Group 1 - Event 1

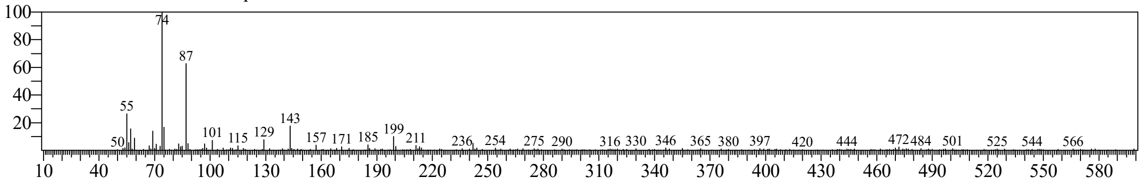


Hit#:1 Entry:86786 Library:NIST17-1.lib
SI:93 Formula:C15H30O2 CAS:124-10-7 MolWeight:242 RetIndex:1680
CompName:Methyl tetradecanoate $$ Tetradecanoic acid, methyl ester $$ Myristic acid, methyl ester $$ Metholeneat 2495 $$ Methyl myristate $$ Methyl r

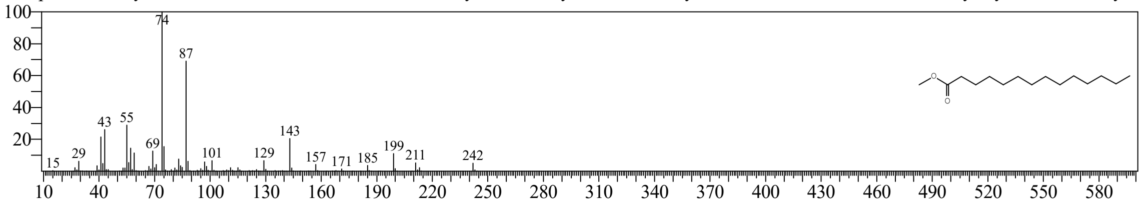


Hit#:2 Entry:80645 Library:NIST14.lib
SI:93 Formula:C15H30O2 CAS:124-10-7 MolWeight:242 RetIndex:1680
CompName:Methyl tetradecanoate $$ Tetradecanoic acid, methyl ester $$ Myristic acid, methyl ester $$ Metholeneat 2495 $$ Methyl myristate $$ Methyl r

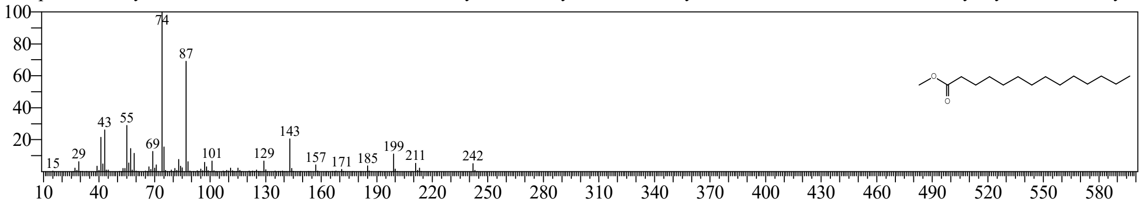


Hit#:3 Entry:23848 Library:NIST14s.lib
SI:92 Formula:C15H30O2 CAS:124-10-7 MolWeight:242 RetIndex:1680
CompName:Methyl tetradecanoate $$ Tetradecanoic acid, methyl ester $$ Myristic acid, methyl ester $$ Metholeneat 2495 $$ Methyl myristate $$ Methyl r

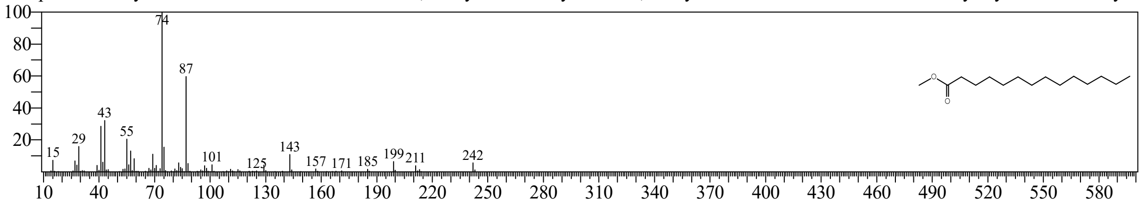


Hit#:4 Entry:27874 Library:NIST17s.lib
SI:92 Formula:C15H30O2 CAS:124-10-7 MolWeight:242 RetIndex:1680
CompName:Methyl tetradecanoate $$ Tetradecanoic acid, methyl ester $$ Myristic acid, methyl ester $$ Metholeneat 2495 $$ Methyl myristate $$ Methyl r

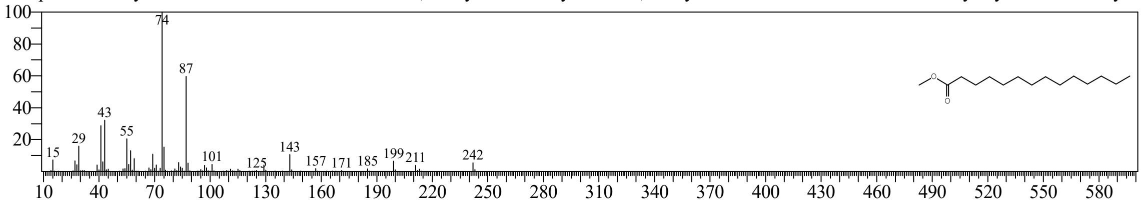


Hit#:5 Entry:27875 Library:NIST17s.lib
SI:91 Formula:C15H30O2 CAS:124-10-7 MolWeight:242 RetIndex:1680
CompName:Methyl tetradecanoate $$ Tetradecanoic acid, methyl ester $$ Myristic acid, methyl ester $$ Metholeneat 2495 $$ Methyl myristate $$ Methyl r

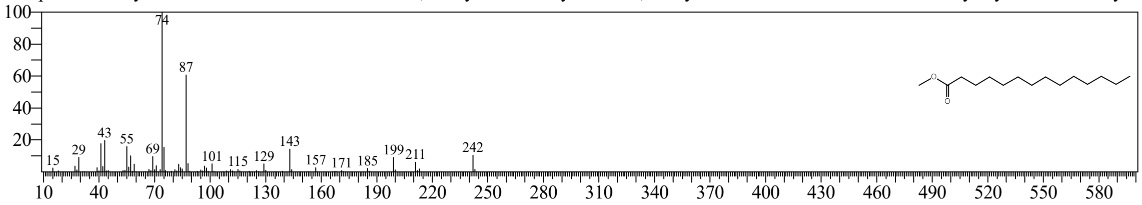

<< Target >>
Line#:3  R.Time:20.817(Scan#:1299)  MassPeaks:386
RawMode:Averaged 20.808-20.825(1298-1300) BasePeak:74.00(452341)
BG Mode:Calc. from Peak  Group 1 - Event 1

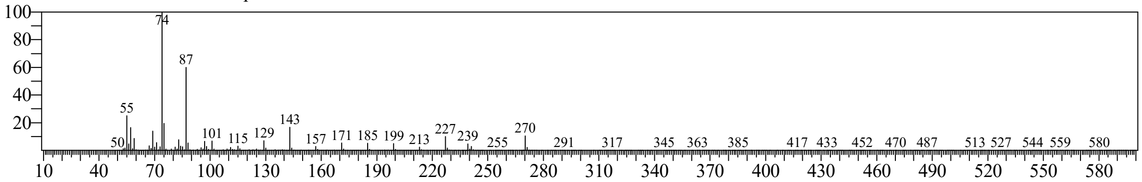


Hit#:1  Entry:113663  Library:NIST17-1.lib
SI:97  Formula:C17H34O2  CAS:112-39-0  MolWeight:270  RetIndex:1878
CompName:Hexadecanoic acid, methyl ester $$ Palmitic acid, methyl ester $$ n-Hexadecanoic acid methyl ester $$ Metholene 2216 $$ Methyl hexadecano

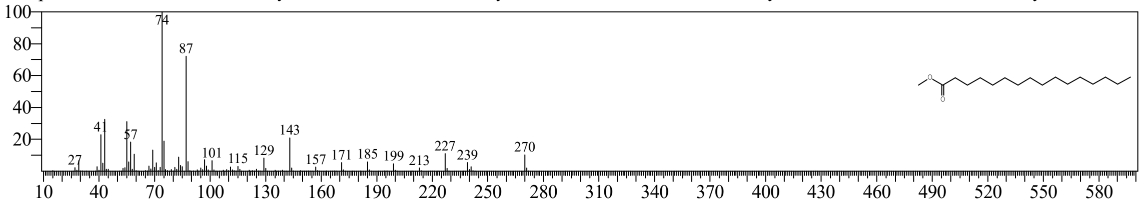


Hit#:2  Entry:104648  Library:NIST14.lib
SI:97  Formula:C17H34O2  CAS:112-39-0  MolWeight:270  RetIndex:1878
CompName:Hexadecanoic acid, methyl ester $$ Palmitic acid, methyl ester $$ n-Hexadecanoic acid methyl ester $$ Metholene 2216 $$ Methyl hexadecano

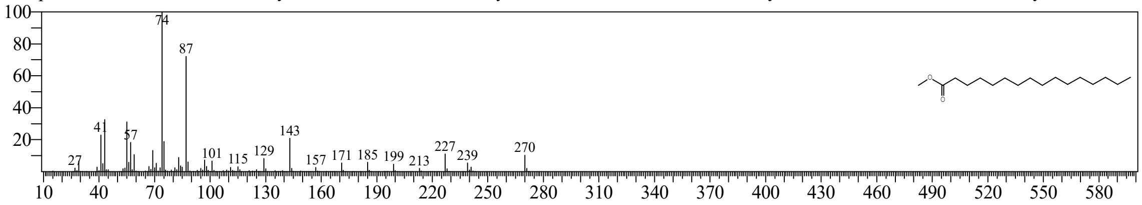


Hit#:3  Entry:26273  Library:NIST14s.lib
SI:97  Formula:C17H34O2  CAS:112-39-0  MolWeight:270  RetIndex:1878
CompName:Hexadecanoic acid, methyl ester $$ Palmitic acid, methyl ester $$ n-Hexadecanoic acid methyl ester $$ Metholene 2216 $$ Methyl hexadecano

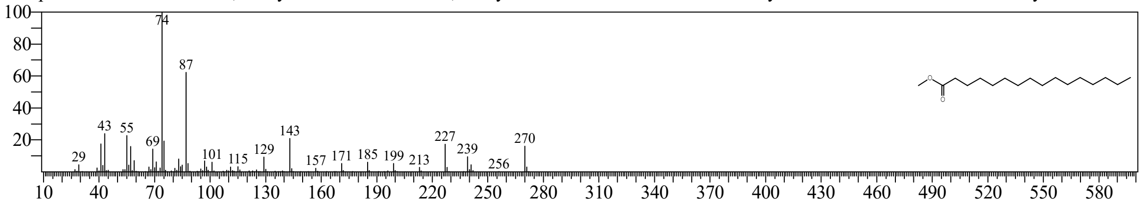


Hit#:4  Entry:26271  Library:NIST14s.lib
SI:96  Formula:C17H34O2  CAS:112-39-0  MolWeight:270  RetIndex:1878
CompName:Hexadecanoic acid, methyl ester $$ Palmitic acid, methyl ester $$ n-Hexadecanoic acid methyl ester $$ Metholene 2216 $$ Methyl hexadecano

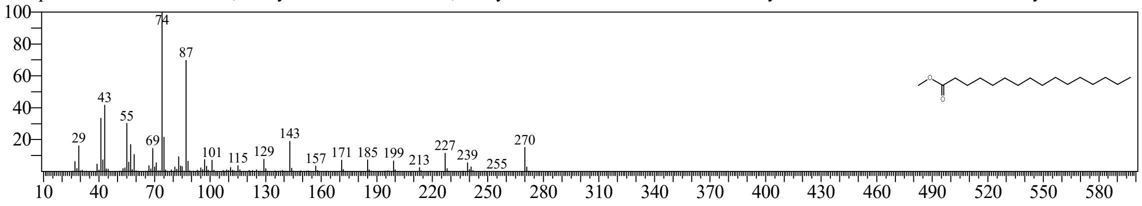


Hit#:5  Entry:30646  Library:NIST17s.lib
SI:96  Formula:C17H34O2  CAS:112-39-0  MolWeight:270  RetIndex:1878
CompName:Hexadecanoic acid, methyl ester $$ Palmitic acid, methyl ester $$ n-Hexadecanoic acid methyl ester $$ Metholene 2216 $$ Methyl hexadecano

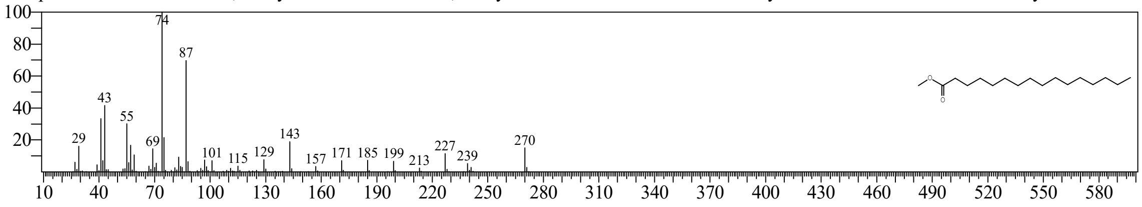

<< Target >>
Line#:4  R.Time:22.175(Scan#:1462)  MassPeaks:314
RawMode:Averaged 22.167-22.183(1461-1463) BasePeak:55.05(1197)
BG Mode:Calc. from Peak  Group 1 - Event 1

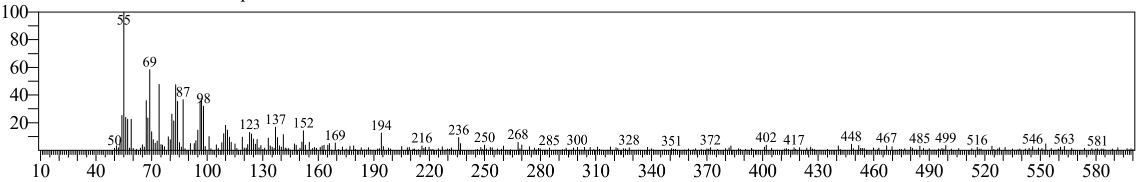


Hit#:1  Entry:111510  Library:NIST17-1.lib
SI:92  Formula:C17H32O2  CAS:56875-67-3  MolWeight:268  RetIndex:1886
CompName:7-Hexadecenoic acid, methyl ester, (Z)- $$ Methyl (7E)-7-hexadecenoate # $$

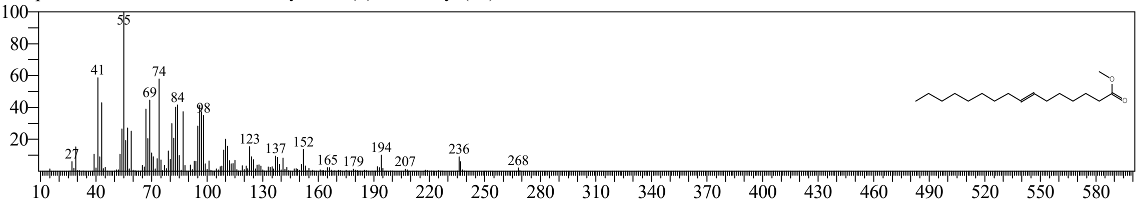


Hit#:2  Entry:30415  Library:NIST17s.lib
SI:91  Formula:C17H32O2  CAS:1120-25-8  MolWeight:268  RetIndex:1886
CompName:9-Hexadecenoic acid, methyl ester, (Z)- $$ Methyl palmitoleate $$ Methyl palmitoleinate $$ Palmitoleic acid, methyl ester $$ Methyl (9Z)-9-he:

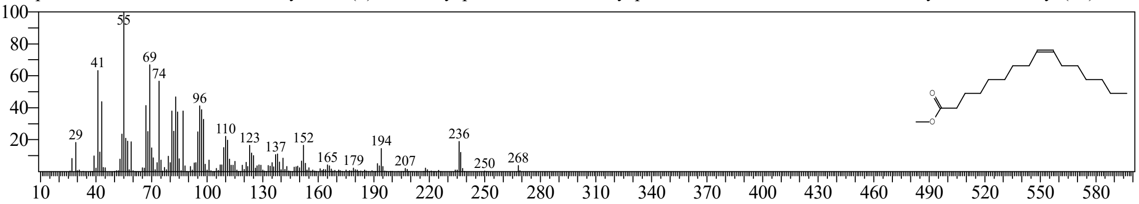


Hit#:3  Entry:26066  Library:NIST14s.lib
SI:91  Formula:C17H32O2  CAS:1120-25-8  MolWeight:268  RetIndex:1886
CompName:9-Hexadecenoic acid, methyl ester, (Z)- $$ Methyl palmitoleate $$ Methyl palmitoleinate $$ Palmitoleic acid, methyl ester $$ Methyl (9Z)-9-he:

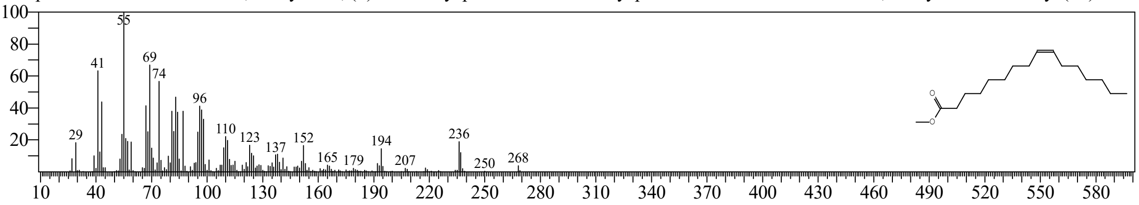


Hit#:4  Entry:30412  Library:NIST17s.lib
SI:90  Formula:C17H32O2  CAS:56875-67-3  MolWeight:268  RetIndex:1886
CompName:7-Hexadecenoic acid, methyl ester, (Z)- $$ Methyl (7E)-7-hexadecenoate # $$

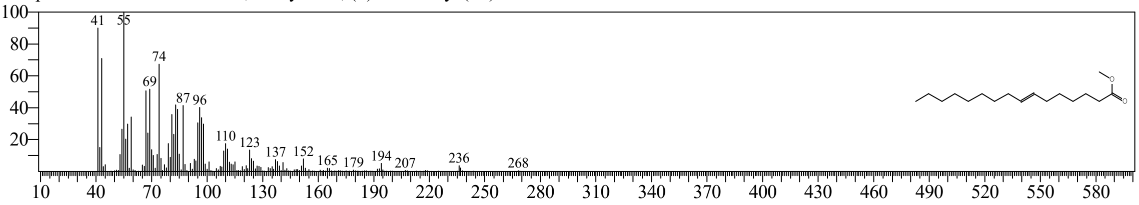


Hit#:5  Entry:26063  Library:NIST14s.lib
SI:90  Formula:C17H32O2  CAS:56875-67-3  MolWeight:268  RetIndex:1886
CompName:7-Hexadecenoic acid, methyl ester, (Z)- $$ Methyl (7E)-7-hexadecenoate # $$

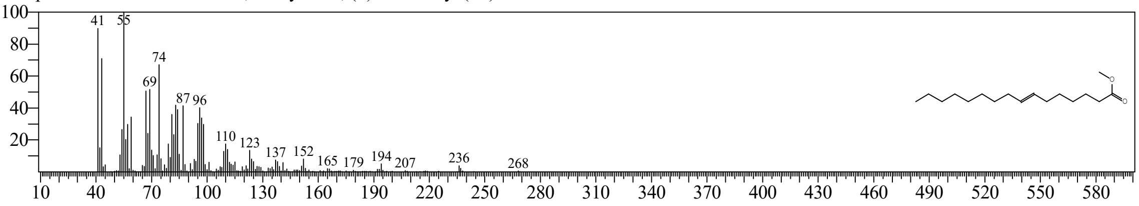

<< Target >>
Line#:5  R.Time:22.783(Scan#:1535)  MassPeaks:336
RawMode:Averaged 22.775-22.792(1534-1536) BasePeak:74.00(3742)
BG Mode:Calc. from Peak  Group 1 - Event 1

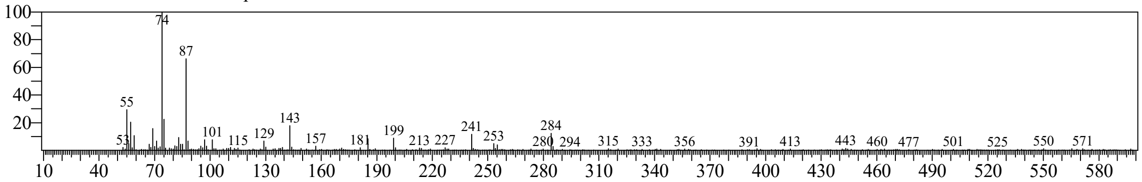


Hit#:1  Entry:27279  Library:NIST14s.lib
SI:94  Formula:C18H36O2  CAS:1731-92-6  MolWeight:284  RetIndex:1978
CompName:Heptadecanoic acid, methyl ester $$ Margaric acid methyl ester $$ Methyl heptadecanoate $$ Methyl margarate $$ n-Heptadecanoic acid methy

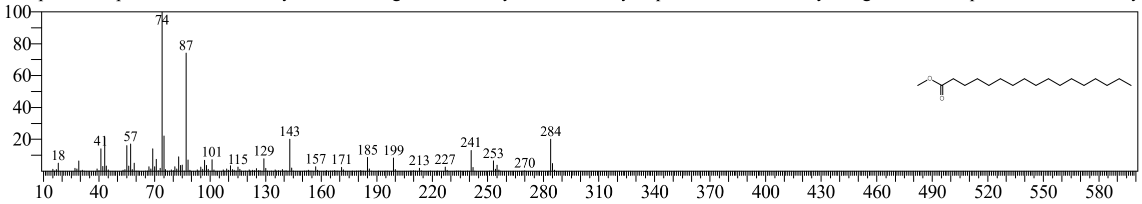


Hit#:2  Entry:31787  Library:NIST17s.lib
SI:94  Formula:C18H36O2  CAS:1731-92-6  MolWeight:284  RetIndex:1978
CompName:Heptadecanoic acid, methyl ester $$ Margaric acid methyl ester $$ Methyl heptadecanoate $$ Methyl margarate $$ n-Heptadecanoic acid methy

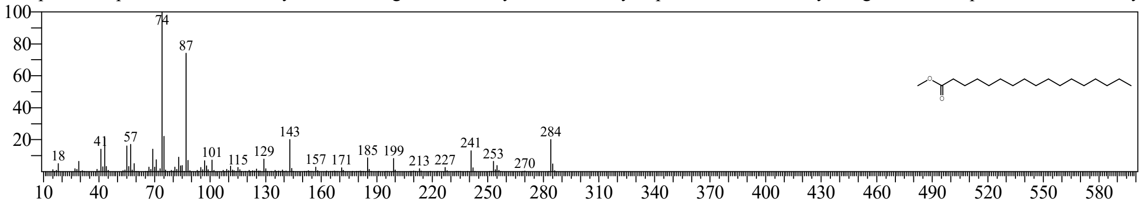


Hit#:3  Entry:31783  Library:NIST17s.lib
SI:93  Formula:C18H36O2  CAS:1731-92-6  MolWeight:284  RetIndex:1978
CompName:Heptadecanoic acid, methyl ester $$ Margaric acid methyl ester $$ Methyl heptadecanoate $$ Methyl margarate $$ n-Heptadecanoic acid methy

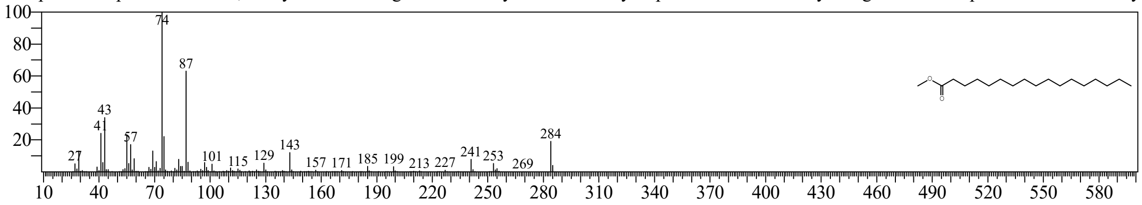


Hit#:4  Entry:27274  Library:NIST14s.lib
SI:93  Formula:C18H36O2  CAS:1731-92-6  MolWeight:284  RetIndex:1978
CompName:Heptadecanoic acid, methyl ester $$ Margaric acid methyl ester $$ Methyl heptadecanoate $$ Methyl margarate $$ n-Heptadecanoic acid methy

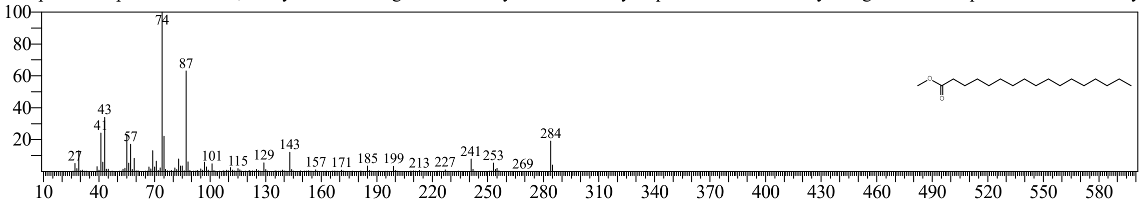


Hit#:5  Entry:27275  Library:NIST14s.lib
SI:93  Formula:C18H36O2  CAS:1731-92-6  MolWeight:284  RetIndex:1978
CompName:Heptadecanoic acid, methyl ester $$ Margaric acid methyl ester $$ Methyl heptadecanoate $$ Methyl margarate $$ n-Heptadecanoic acid methy

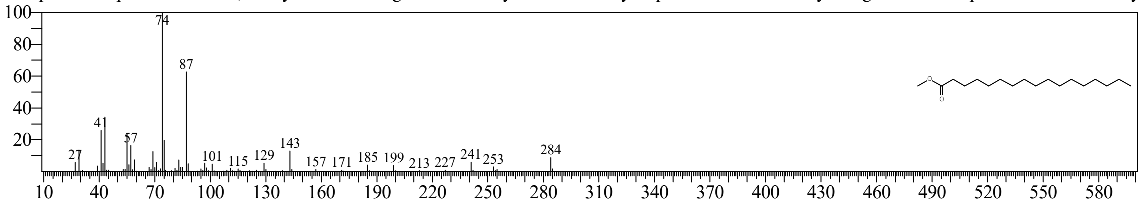

<< Target >>
Line#:6  R.Time:24.100(Scan#:1693)  MassPeaks:316
RawMode:Averaged 24.092-24.108(1692-1694) BasePeak:55.00(710)
BG Mode:Calc. from Peak  Group 1 - Event 1

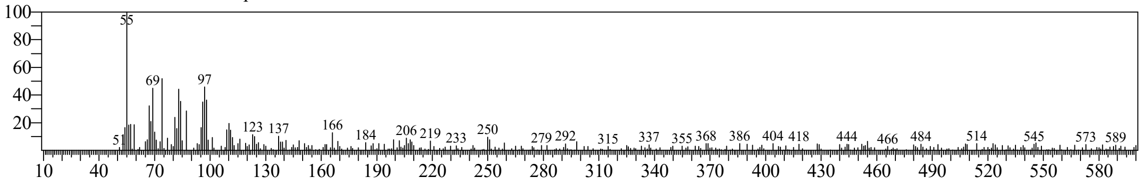


Hit#:1  Entry:115096  Library:NIST14.lib
SI:87  Formula:C18H34O2  CAS:0-00-0  MolWeight:282  RetIndex:1986
CompName:Methyl 9-heptadecenoate or 9-17:1

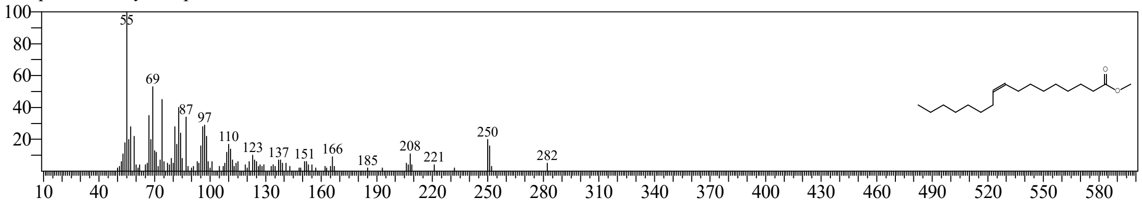


Hit#:2  Entry:125440  Library:NIST17-1.lib
SI:87  Formula:C18H34O2  CAS:0-00-0  MolWeight:282  RetIndex:1986
CompName:Methyl 9-heptadecenoate or 9-17:1

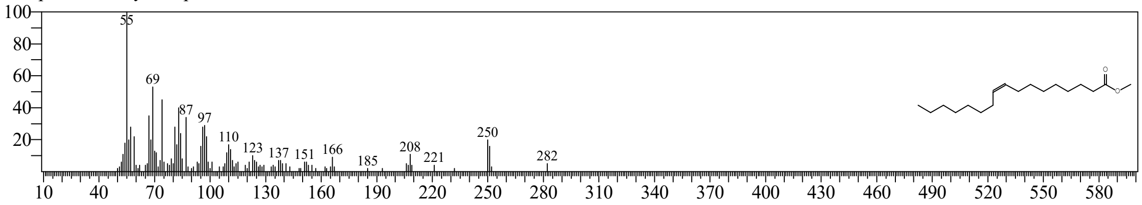


Hit#:3  Entry:125438  Library:NIST17-1.lib
SI:87  Formula:C18H34O2  CAS:0-00-0  MolWeight:282  RetIndex:1986
CompName:cis-10-Heptadecenoic acid, methyl ester

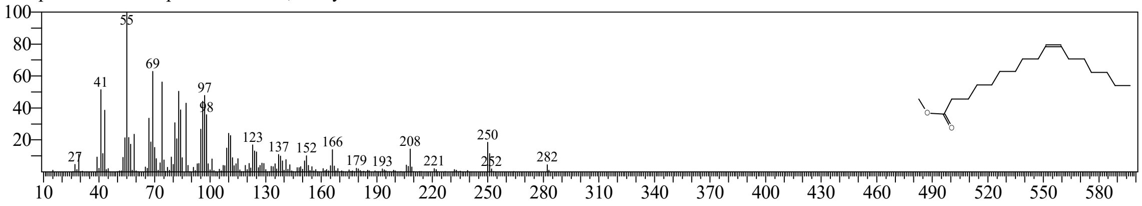


Hit#:4  Entry:115094  Library:NIST14.lib
SI:87  Formula:C18H34O2  CAS:0-00-0  MolWeight:282  RetIndex:1986
CompName:cis-10-Heptadecenoic acid, methyl ester

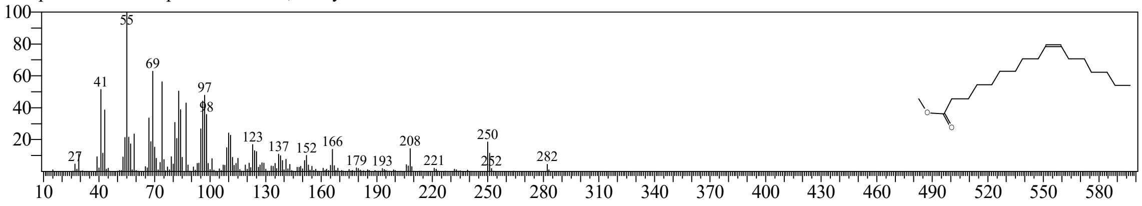


Hit#:5  Entry:31597  Library:NIST17s.lib
SI:86  Formula:C18H34O2  CAS:10152-61-1  MolWeight:282  RetIndex:1941
CompName:Cyclopropaneoctanoic acid, 2-hexyl-, methyl ester $$ Methyl 8-(2-hexylcyclopropyl)octanoate # $$

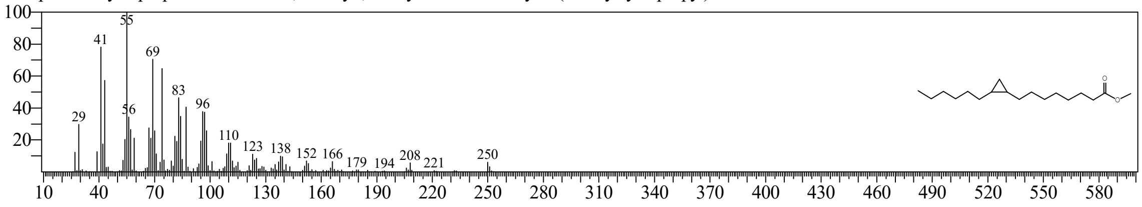

<< Target >>
Line#:7   R.Time:24.875(Scan#:1786)  MassPeaks:336
RawMode:Averaged 24.867-24.883(1785-1787) BasePeak:74.00(136394)
BG Mode:Calc. from Peak  Group 1 - Event 1

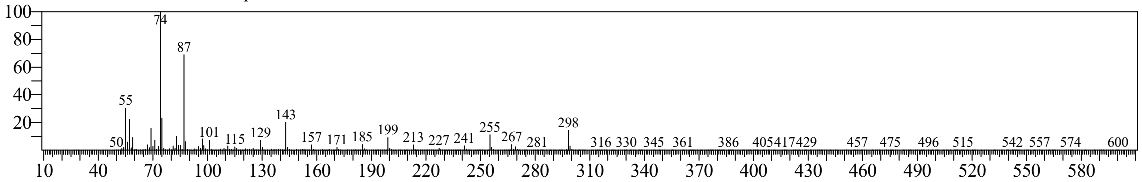


Hit#:1  Entry:129694  Library:NIST14.lib
SI:98  Formula:C19H38O2  CAS:112-61-8  MolWeight:298  RetIndex:2077
CompName:Methyl stearate $$ Octadecanoic acid, methyl ester $$ Stearic acid, methyl ester $$ n-Octadecanoic acid, methyl ester $$ Kemester 9718 $$ Me

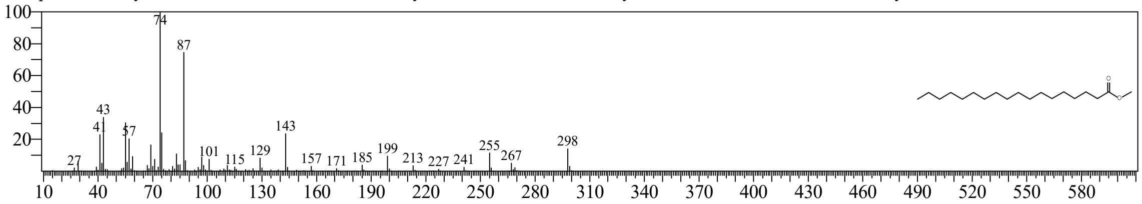

Hit#:2  Entry:141978  Library:NIST17-1.lib
SI:98  Formula:C19H38O2  CAS:112-61-8  MolWeight:298  RetIndex:2077
CompName:Methyl stearate $$ Octadecanoic acid, methyl ester $$ Stearic acid, methyl ester $$ n-Octadecanoic acid, methyl ester $$ Kemester 9718 $$ Me

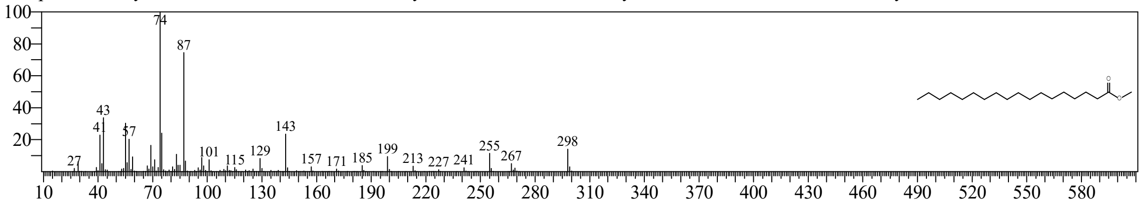

Hit#:3  Entry:28255  Library:NIST14s.lib
SI:97  Formula:C19H38O2  CAS:112-61-8  MolWeight:298  RetIndex:2077
CompName:Methyl stearate $$ Octadecanoic acid, methyl ester $$ Stearic acid, methyl ester $$ n-Octadecanoic acid, methyl ester $$ Kemester 9718 $$ Me

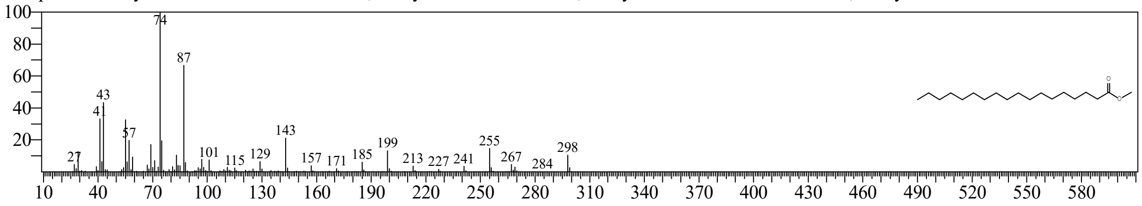

Hit#:4  Entry:28257  Library:NIST14s.lib
SI:97  Formula:C19H38O2  CAS:112-61-8  MolWeight:298  RetIndex:2077
CompName:Methyl stearate $$ Octadecanoic acid, methyl ester $$ Stearic acid, methyl ester $$ n-Octadecanoic acid, methyl ester $$ Kemester 9718 $$ Me

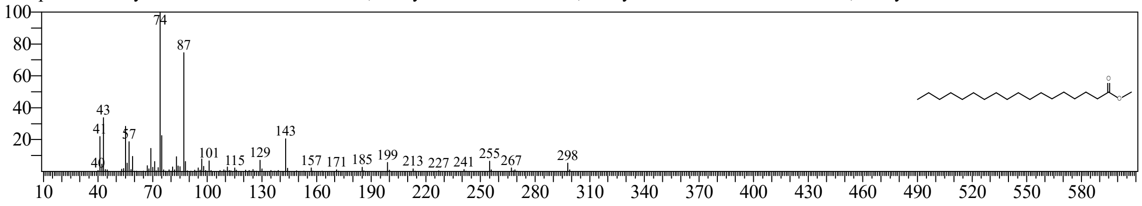

Hit#:5  Entry:32875  Library:NIST17s.lib
SI:97  Formula:C19H38O2  CAS:112-61-8  MolWeight:298  RetIndex:2077
CompName:Methyl stearate $$ Octadecanoic acid, methyl ester $$ Stearic acid, methyl ester $$ n-Octadecanoic acid, methyl ester $$ Kemester 9718 $$ Me

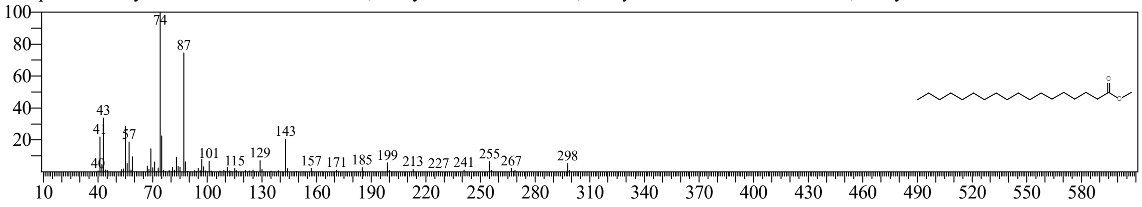

<< Target >>
Line#:8  R.Time:26.258(Scan#:1952)  MassPeaks:425
RawMode:Averaged 26.250-26.267(1951-1953) BasePeak:55.05(220564)
BG Mode:Calc. from Peak  Group 1 - Event 1

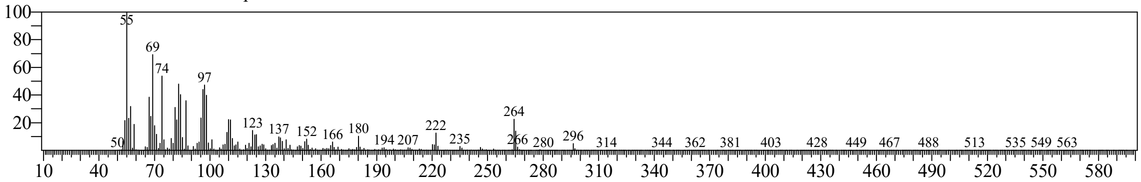


Hit#:1  Entry:32747  Library:NIST17s.lib
SI:97  Formula:C19H36O2  CAS:1937-62-8  MolWeight:296  RetIndex:2085
CompName:9-Octadecenoic acid, methyl ester, (E)- $$ Elaidic acid, methyl ester $$ Methyl elaidate $$ Methyl trans-9-octadecenoate $$ (E)-9-Octadecenoic

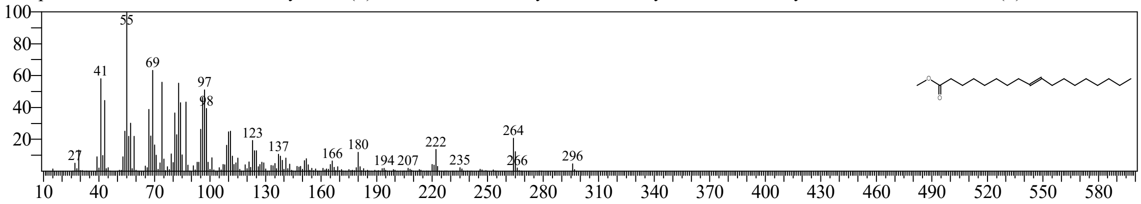


Hit#:2  Entry:28138  Library:NIST14s.lib
SI:97  Formula:C19H36O2  CAS:1937-62-8  MolWeight:296  RetIndex:2085
CompName:9-Octadecenoic acid, methyl ester, (E)- $$ Elaidic acid, methyl ester $$ Methyl elaidate $$ Methyl trans-9-octadecenoate $$ (E)-9-Octadecenoic

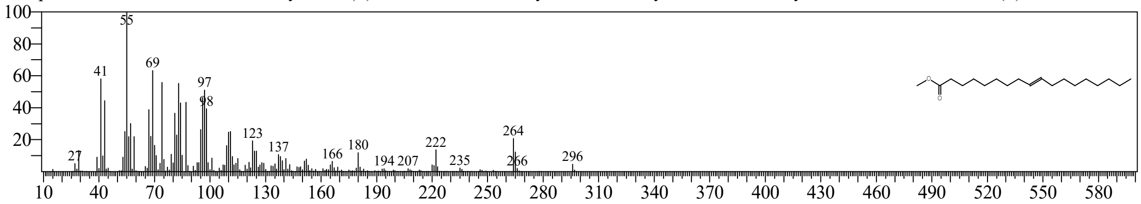


Hit#:3  Entry:28139  Library:NIST14s.lib
SI:96  Formula:C19H36O2  CAS:52380-33-3  MolWeight:296  RetIndex:2085
CompName:11-Octadecenoic acid, methyl ester $$ Methyl 11-octadecenoate $$ Octadec-11-enoic acid, methyl ester $$ Methyl (11E)-11-octadecenoate # $$

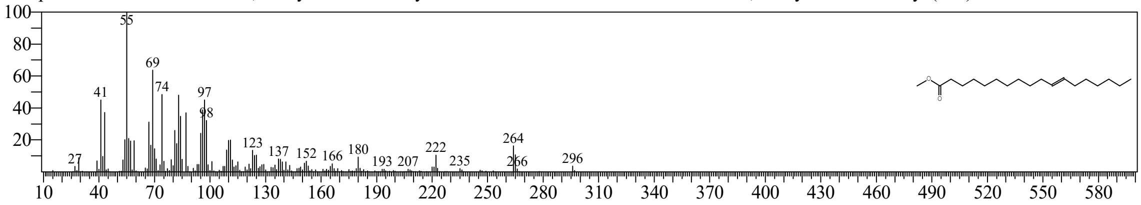


Hit#:4  Entry:32748  Library:NIST17s.lib
SI:96  Formula:C19H36O2  CAS:52380-33-3  MolWeight:296  RetIndex:2085
CompName:11-Octadecenoic acid, methyl ester $$ Methyl 11-octadecenoate $$ Octadec-11-enoic acid, methyl ester $$ Methyl (11E)-11-octadecenoate # $$

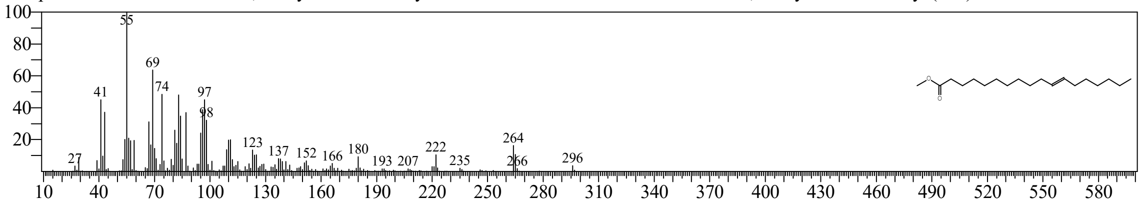


Hit#:5  Entry:139650  Library:NIST17-1.lib
SI:96  Formula:C19H36O2  CAS:112-62-9  MolWeight:296  RetIndex:2085
CompName:9-Octadecenoic acid (Z)-, methyl ester $$ Oleic acid, methyl ester $$ Emery oleic acid ester 2301 $$ Methyl cis-9-octadecenoate $$ Methyl olea

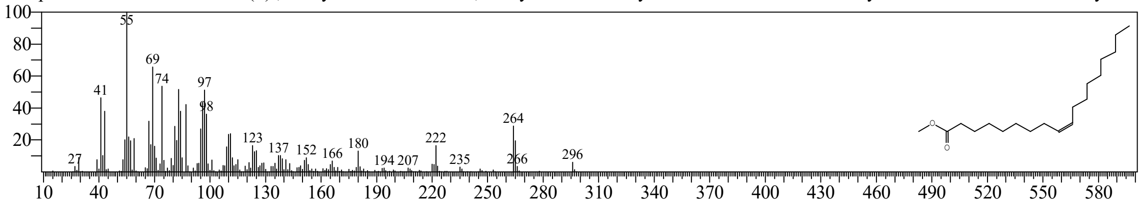

<< Target >>
Line#:9   R.Time:26.383(Scan#:1967)  MassPeaks:355
RawMode:Averaged 26.375-26.392(1966-1968) BasePeak:55.05(14840)
BG Mode:Calc. from Peak  Group 1 - Event 1

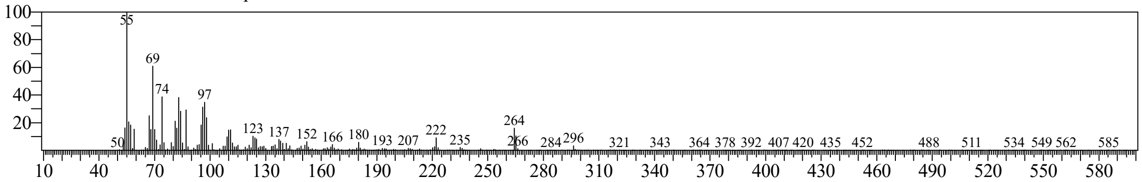


Hit#:1  Entry:139644  Library:NIST17-1.lib
SI:95  Formula:C19H36O2  CAS:0-00-0  MolWeight:296  RetIndex:2085
CompName:trans-13-Octadecenoic acid, methyl ester

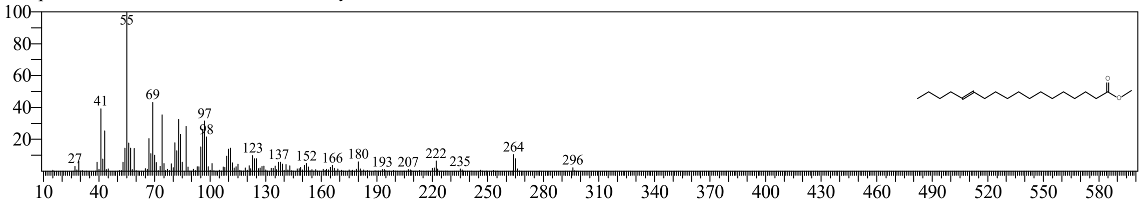


Hit#:2  Entry:127658  Library:NIST14.lib
SI:95  Formula:C19H36O2  CAS:0-00-0  MolWeight:296  RetIndex:2085
CompName:trans-13-Octadecenoic acid, methyl ester

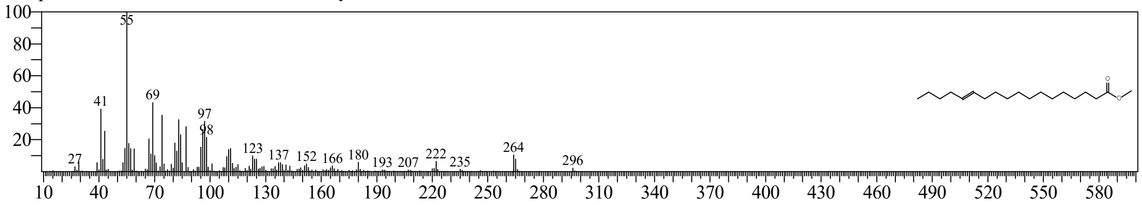


Hit#:3  Entry:127661  Library:NIST14.lib
SI:95  Formula:C19H36O2  CAS:0-00-0  MolWeight:296  RetIndex:2085
CompName:cis-13-Octadecenoic acid, methyl ester

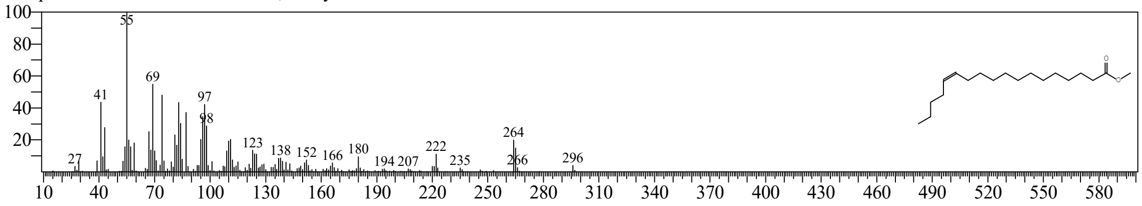


Hit#:4  Entry:28139  Library:NIST14s.lib
SI:95  Formula:C19H36O2  CAS:52380-33-3  MolWeight:296  RetIndex:2085
CompName:11-Octadecenoic acid, methyl ester $$ Methyl 11-octadecenoate $$ Octadec-11-enoic acid, methyl ester $$ Methyl (11E)-11-octadecenoate # $$

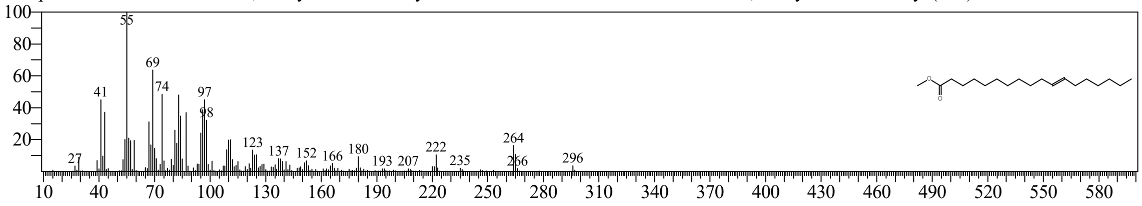


Hit#:5  Entry:127650  Library:NIST14.lib
SI:95  Formula:C19H36O2  CAS:13481-95-3  MolWeight:296  RetIndex:2085
CompName:10-Octadecenoic acid, methyl ester $$ Methyl 10-octadecenoate $$

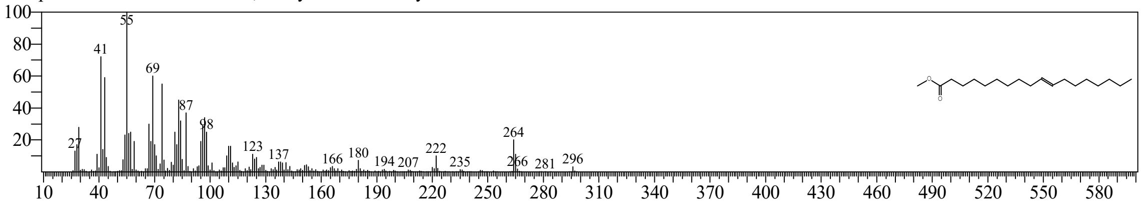

<< Target >>
Line#:10   R.Time:27.742(Scan#:2130)  MassPeaks:384
RawMode:Averaged 27.733-27.750(2129-2131) BasePeak:67.05(2304)
BG Mode:Calc. from Peak  Group 1 - Event 1

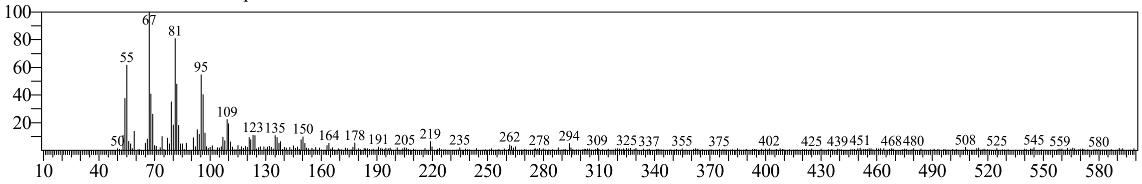


Hit#:1  Entry:137663  Library:NIST17-1.lib
SI:96  Formula:C19H34O2  CAS:2462-85-3  MolWeight:294  RetIndex:2093
CompName:9,12-Octadecadienoic acid, methyl ester $$ 9,12-Octadecenoic acid, methyl ester $$ Methyl octadeca-9,12-dienoate $$ Methyl 9,12-octadecadi

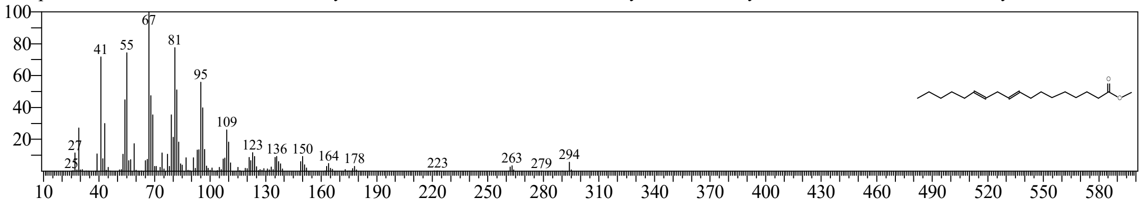


Hit#:2  Entry:125931  Library:NIST14.lib
SI:96  Formula:C19H34O2  CAS:2462-85-3  MolWeight:294  RetIndex:2093
CompName:9,12-Octadecadienoic acid, methyl ester $$ 9,12-Octadecenoic acid, methyl ester $$ Methyl octadeca-9,12-dienoate $$ Methyl 9,12-octadecadi

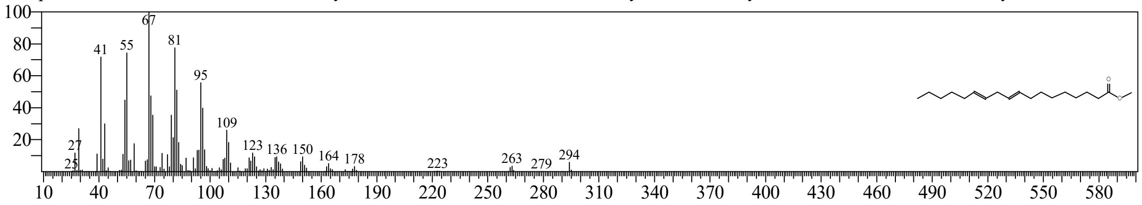


Hit#:3  Entry:32588  Library:NIST17s.lib
SI:95  Formula:C19H34O2  CAS:112-63-0  MolWeight:294  RetIndex:2093
CompName:9,12-Octadecadienoic acid (Z,Z)-, methyl ester $$ Linoleic acid, methyl ester $$ Methyl cis,cis-9,12-octadecadienoate $$ Methyl linoleate $$ M

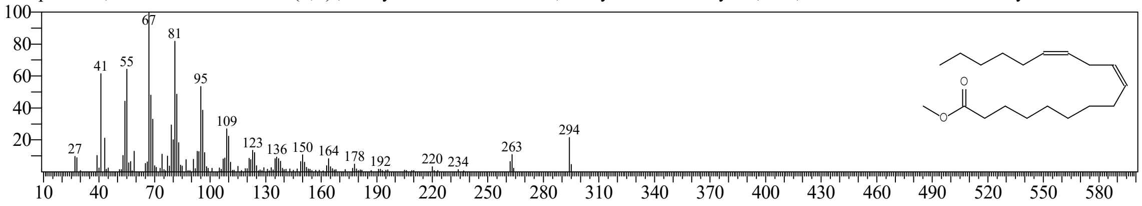


Hit#:4  Entry:27999  Library:NIST14s.lib
SI:95  Formula:C19H34O2  CAS:112-63-0  MolWeight:294  RetIndex:2093
CompName:9,12-Octadecadienoic acid (Z,Z)-, methyl ester $$ Linoleic acid, methyl ester $$ Methyl cis,cis-9,12-octadecadienoate $$ Methyl linoleate $$ M

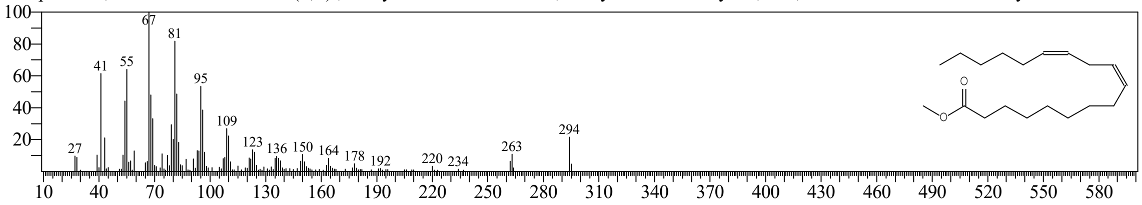


Hit#:5  Entry:32589  Library:NIST17s.lib
SI:95  Formula:C19H34O2  CAS:112-63-0  MolWeight:294  RetIndex:2093
CompName:9,12-Octadecadienoic acid (Z,Z)-, methyl ester $$ Linoleic acid, methyl ester $$ Methyl cis,cis-9,12-octadecadienoate $$ Methyl linoleate $$ M

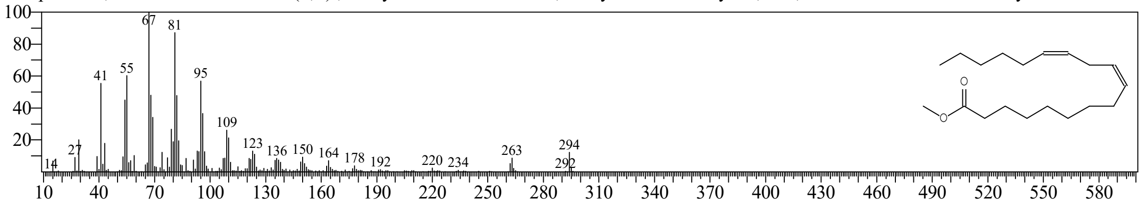

<< Target >>
Line#:11   R.Time:27.950(Scan#:2155)  MassPeaks:359
RawMode:Averaged 27.942-27.958(2154-2156) BasePeak:67.00(1472)
BG Mode:Calc. from Peak  Group 1 - Event 1

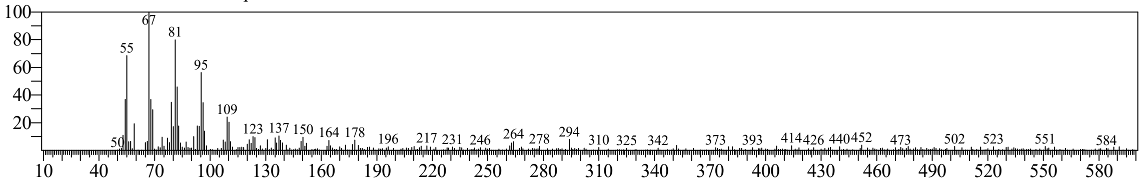


Hit#:1  Entry:125931  Library:NIST14.lib
SI:95  Formula:C19H34O2  CAS:2462-85-3  MolWeight:294  RetIndex:2093
CompName:9,12-Octadecadienoic acid, methyl ester $$ 9,12-Octadecenoic acid, methyl ester $$ Methyl octadeca-9,12-dienoate $$ Methyl 9,12-octadecadi

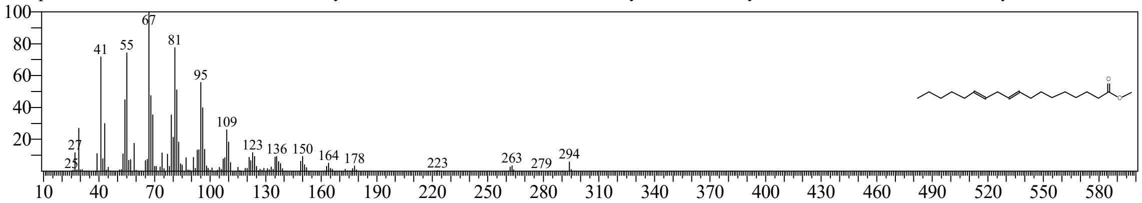


Hit#:2  Entry:137663  Library:NIST17-1.lib
SI:95  Formula:C19H34O2  CAS:2462-85-3  MolWeight:294  RetIndex:2093
CompName:9,12-Octadecadienoic acid, methyl ester $$ 9,12-Octadecenoic acid, methyl ester $$ Methyl octadeca-9,12-dienoate $$ Methyl 9,12-octadecadi

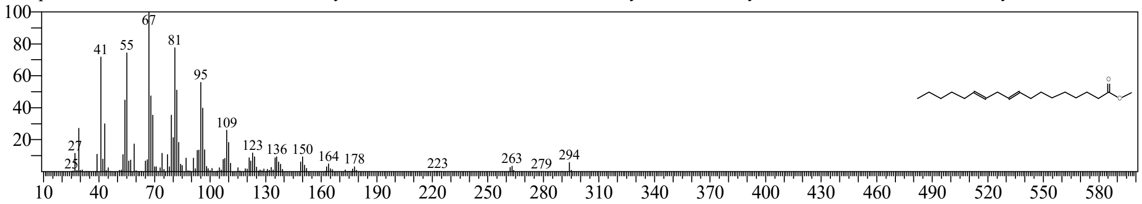


Hit#:3  Entry:32588  Library:NIST17s.lib
SI:94  Formula:C19H34O2  CAS:112-63-0  MolWeight:294  RetIndex:2093
CompName:9,12-Octadecadienoic acid (Z,Z)-, methyl ester $$ Linoleic acid, methyl ester $$ Methyl cis,cis-9,12-octadecadienoate $$ Methyl linoleate $$ M

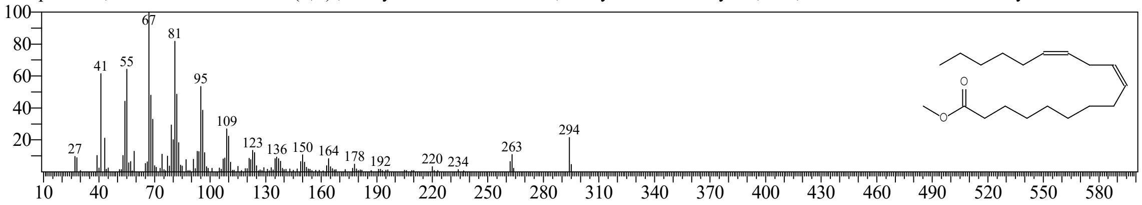


Hit#:4  Entry:27999  Library:NIST14s.lib
SI:94  Formula:C19H34O2  CAS:112-63-0  MolWeight:294  RetIndex:2093
CompName:9,12-Octadecadienoic acid (Z,Z)-, methyl ester $$ Linoleic acid, methyl ester $$ Methyl cis,cis-9,12-octadecadienoate $$ Methyl linoleate $$ M

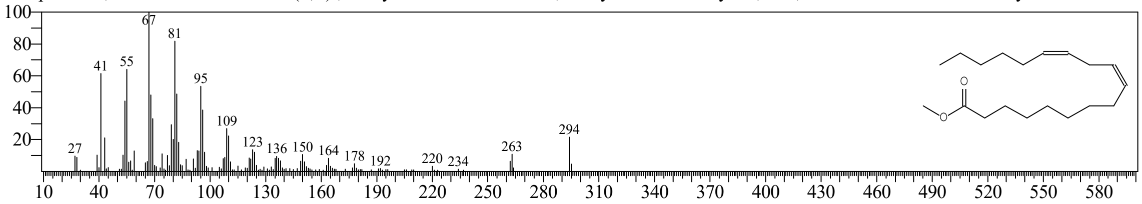


Hit#:5  Entry:32589  Library:NIST17s.lib
SI:94  Formula:C19H34O2  CAS:112-63-0  MolWeight:294  RetIndex:2093
CompName:9,12-Octadecadienoic acid (Z,Z)-, methyl ester $$ Linoleic acid, methyl ester $$ Methyl cis,cis-9,12-octadecadienoate $$ Methyl linoleate $$ M

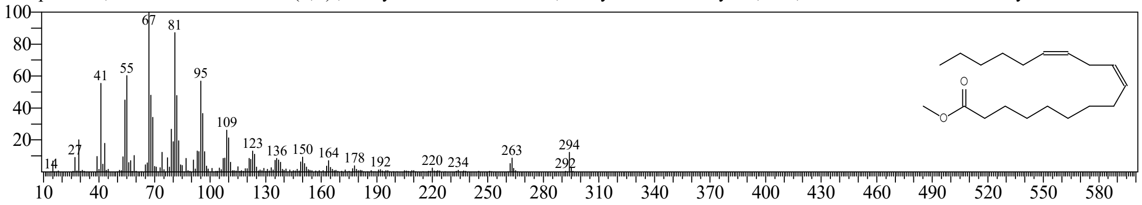

<< Target >>
Line#:12   R.Time:28.375(Scan#:2206)  MassPeaks:418
RawMode:Averaged 28.367-28.383(2205-2207) BasePeak:67.05(471273)
BG Mode:Calc. from Peak  Group 1 - Event 1

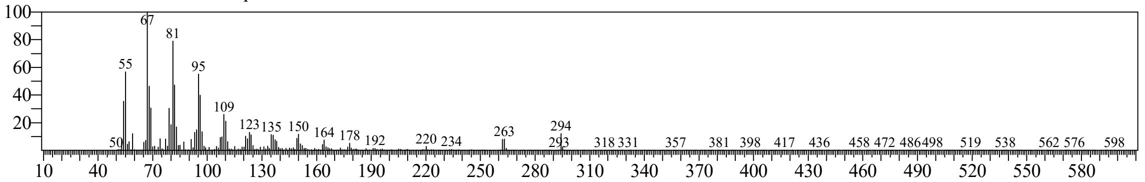


Hit#:1  Entry:32588  Library:NIST17s.lib
SI:97  Formula:C19H34O2  CAS:112-63-0  MolWeight:294  RetIndex:2093
CompName:9,12-Octadecadienoic acid (Z,Z)-, methyl ester $$ Linoleic acid, methyl ester $$ Methyl cis,cis-9,12-octadecadienoate $$ Methyl linoleate $$ M

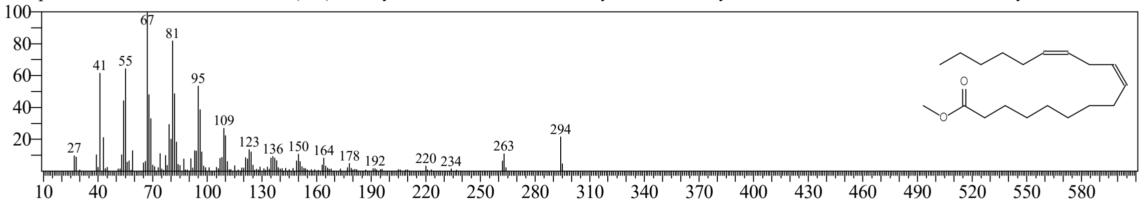


Hit#:2  Entry:27999  Library:NIST14s.lib
SI:97  Formula:C19H34O2  CAS:112-63-0  MolWeight:294  RetIndex:2093
CompName:9,12-Octadecadienoic acid (Z,Z)-, methyl ester $$ Linoleic acid, methyl ester $$ Methyl cis,cis-9,12-octadecadienoate $$ Methyl linoleate $$ M

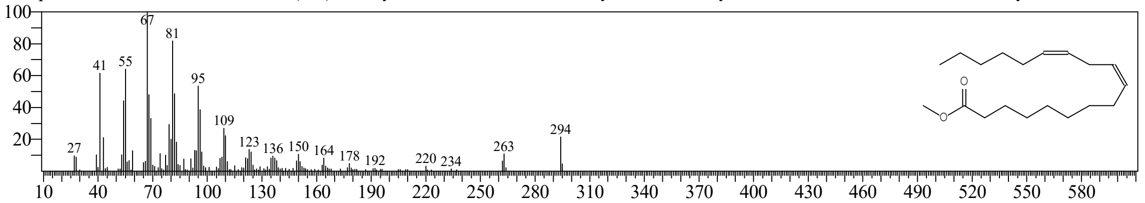


Hit#:3  Entry:32589  Library:NIST17s.lib
SI:97  Formula:C19H34O2  CAS:112-63-0  MolWeight:294  RetIndex:2093
CompName:9,12-Octadecadienoic acid (Z,Z)-, methyl ester $$ Linoleic acid, methyl ester $$ Methyl cis,cis-9,12-octadecadienoate $$ Methyl linoleate $$ M

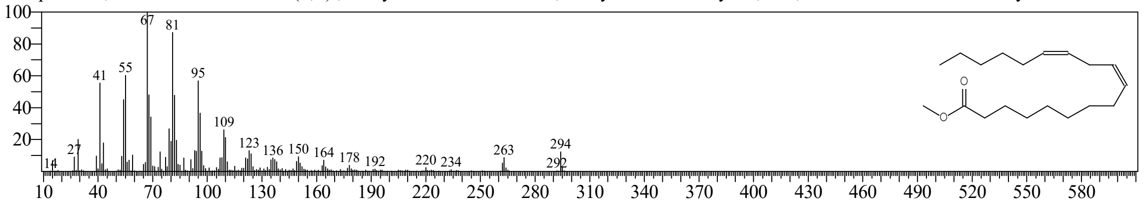


Hit#:4  Entry:28000  Library:NIST14s.lib
SI:97  Formula:C19H34O2  CAS:112-63-0  MolWeight:294  RetIndex:2093
CompName:9,12-Octadecadienoic acid (Z,Z)-, methyl ester $$ Linoleic acid, methyl ester $$ Methyl cis,cis-9,12-octadecadienoate $$ Methyl linoleate $$ M

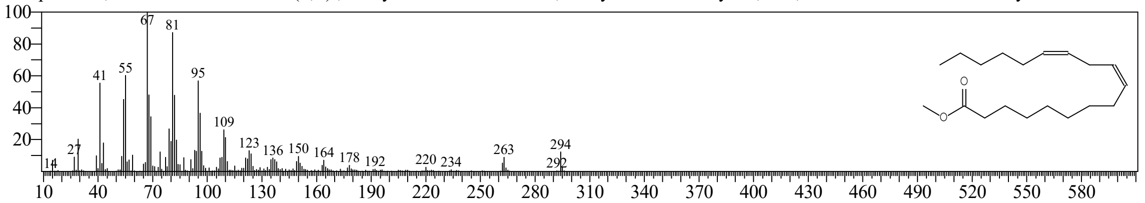


Hit#:5  Entry:137664  Library:NIST17-1.lib
SI:95  Formula:C19H34O2  CAS:0-00-0  MolWeight:294  RetIndex:2093
CompName:Methyl 10-trans,12-cis-octadecadienoate

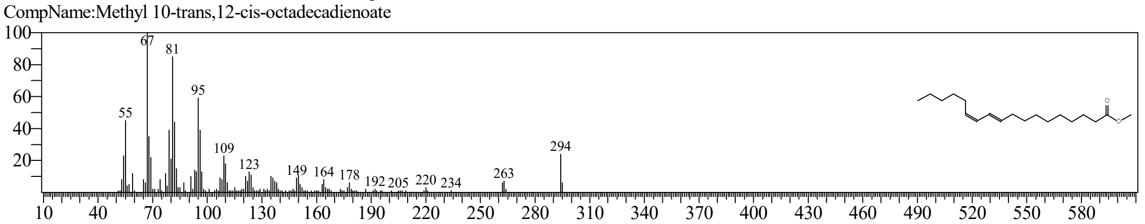

<< Target >>
Line#:13 R.Time:29.275(Scan#:2314) MassPeaks:314
RawMode:Averaged 29.267-29.283(2313-2315) BasePeak:74.00(10878)
BG Mode:Calc. from Peak Group 1 - Event 1

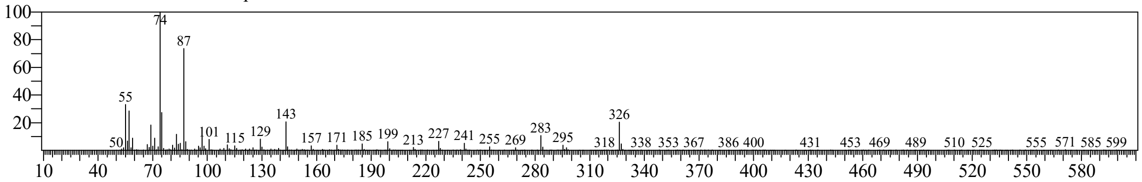


Hit#:1 Entry:154704 Library:NIST14.lib
SI:97 Formula:C21H42O2 CAS:0-00-0 MolWeight:326 RetIndex:2212
CompName:Methyl 18-methylnonadecanoate

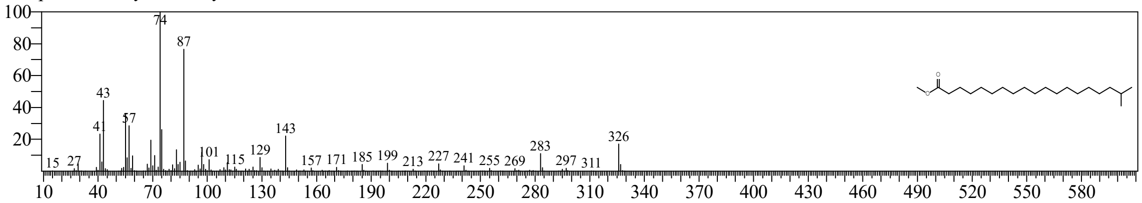


Hit#:2 Entry:170238 Library:NIST17-1.lib
SI:97 Formula:C21H42O2 CAS:0-00-0 MolWeight:326 RetIndex:2212
CompName:Methyl 18-methylnonadecanoate

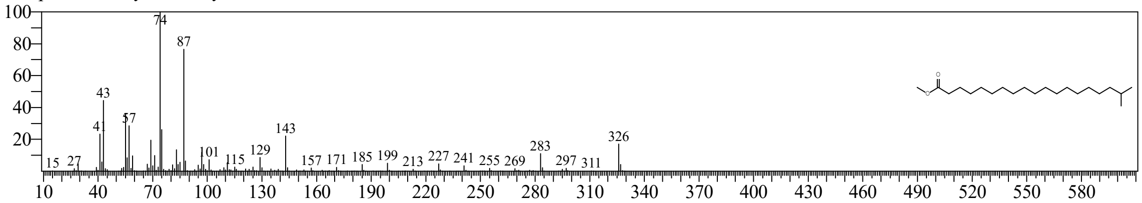


Hit#:3 Entry:34726 Library:NIST17s.lib
SI:96 Formula:C21H42O2 CAS:1120-28-1 MolWeight:326 RetIndex:2276
CompName:Eicosanoic acid, methyl ester $$ Methyl arachisate $$ Methyl eicosanoate $$ Arachidic acid methyl ester $$ Kemester 2050 $$ Methyl aracidat

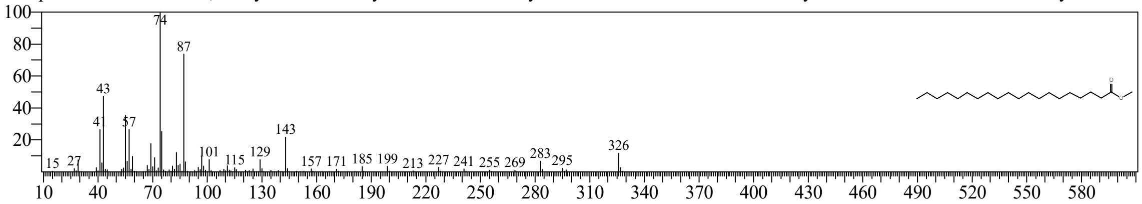


Hit#:4 Entry:34724 Library:NIST17s.lib
SI:93 Formula:C21H42O2 CAS:1120-28-1 MolWeight:326 RetIndex:2276
CompName:Eicosanoic acid, methyl ester $$ Methyl arachisate $$ Methyl eicosanoate $$ Arachidic acid methyl ester $$ Kemester 2050 $$ Methyl aracidat

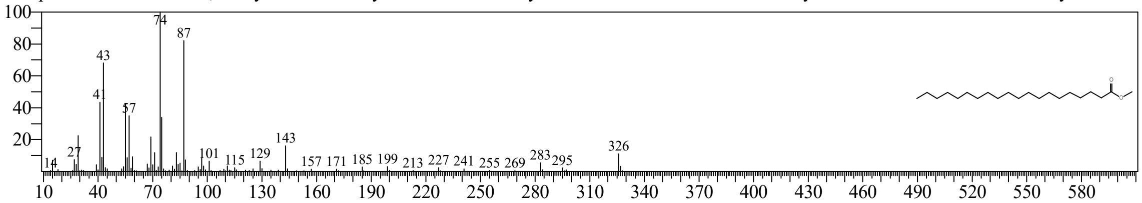


Hit#:5 Entry:29931 Library:NIST14s.lib
SI:93 Formula:C21H42O2 CAS:1120-28-1 MolWeight:326 RetIndex:2276
CompName:Eicosanoic acid, methyl ester $$ Methyl arachisate $$ Methyl eicosanoate $$ Arachidic acid methyl ester $$ Kemester 2050 $$ Methyl aracidat

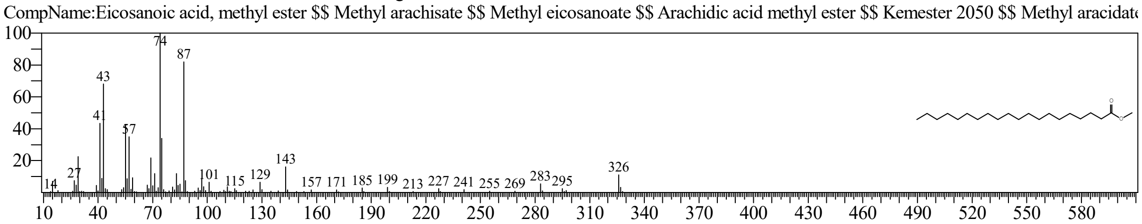

<< Target >>
Line#:14  R.Time:29.825(Scan#:2380)  MassPeaks:355
RawMode:Averaged 29.817-29.833(2379-2381) BasePeak:79.05(6454)
BG Mode:Calc. from Peak  Group 1 - Event 1

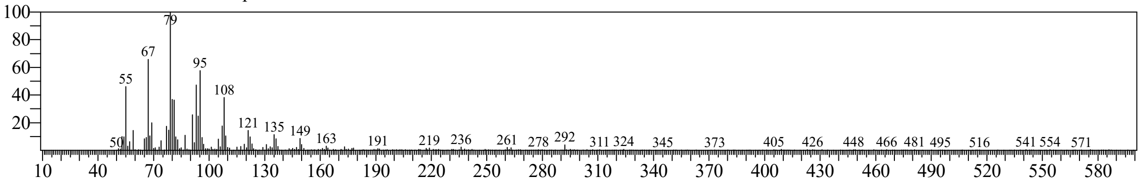


Hit#:1  Entry:135724  Library:NIST17-1.lib
SI:95  Formula:C19H32O2  CAS:301-00-8  MolWeight:292  RetIndex:2101
CompName:9,12,15-Octadecatrienoic acid, methyl ester, (Z,Z,Z)- $$ Linolenic acid, methyl ester $$ Methyl all-cis-9,12,15-octadecatrienoate $$ Methyl lino

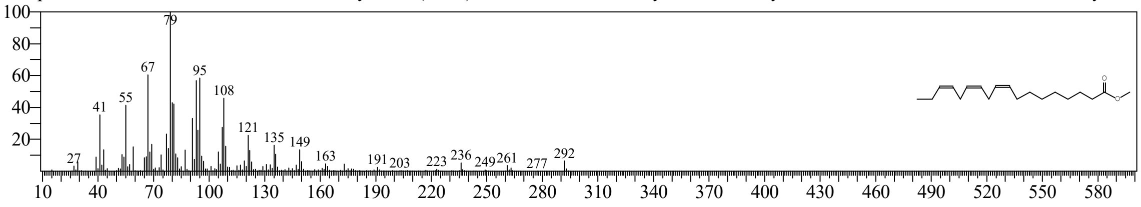


Hit#:2  Entry:124228  Library:NIST14.lib
SI:95  Formula:C19H32O2  CAS:301-00-8  MolWeight:292  RetIndex:2101
CompName:9,12,15-Octadecatrienoic acid, methyl ester, (Z,Z,Z)- $$ Linolenic acid, methyl ester $$ Methyl all-cis-9,12,15-octadecatrienoate $$ Methyl lino

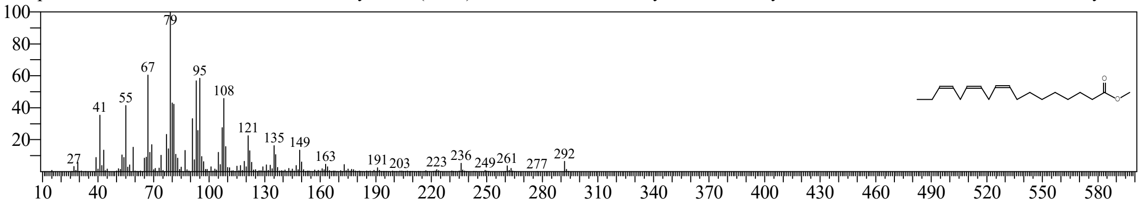


Hit#:3  Entry:32437  Library:NIST17s.lib
SI:95  Formula:C19H32O2  CAS:301-00-8  MolWeight:292  RetIndex:2101
CompName:9,12,15-Octadecatrienoic acid, methyl ester, (Z,Z,Z)- $$ Linolenic acid, methyl ester $$ Methyl all-cis-9,12,15-octadecatrienoate $$ Methyl lino

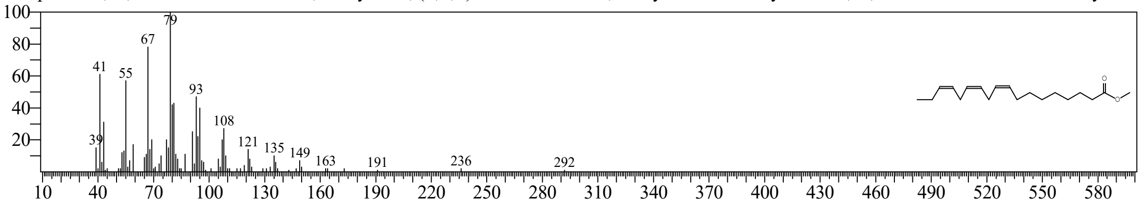


Hit#:4  Entry:27872  Library:NIST14s.lib
SI:95  Formula:C19H32O2  CAS:301-00-8  MolWeight:292  RetIndex:2101
CompName:9,12,15-Octadecatrienoic acid, methyl ester, (Z,Z,Z)- $$ Linolenic acid, methyl ester $$ Methyl all-cis-9,12,15-octadecatrienoate $$ Methyl lino

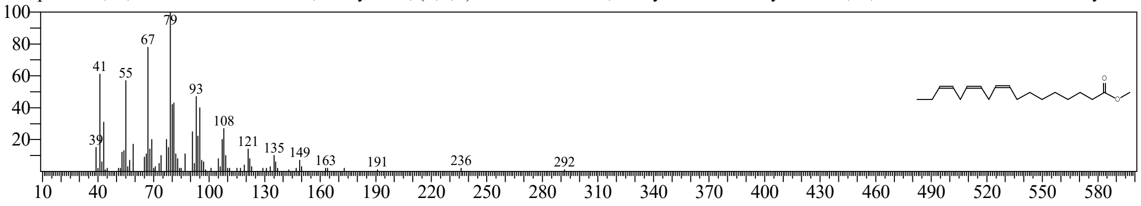


Hit#:5  Entry:32438  Library:NIST17s.lib
SI:95  Formula:C19H32O2  CAS:301-00-8  MolWeight:292  RetIndex:2101
CompName:9,12,15-Octadecatrienoic acid, methyl ester, (Z,Z,Z)- $$ Linolenic acid, methyl ester $$ Methyl all-cis-9,12,15-octadecatrienoate $$ Methyl lino

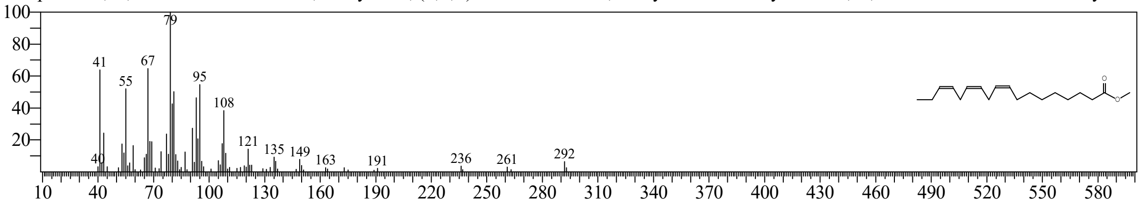

<< Target >>
Line#:15  R.Time:30.308(Scan#:2438)  MassPeaks:379
RawMode:Averaged 30.300-30.317(2437-2439) BasePeak:79.05(5075)
BG Mode:Calc. from Peak  Group 1 - Event 1

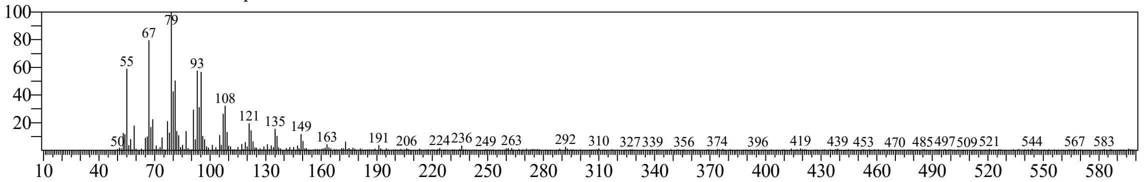


Hit#:1  Entry:124228  Library:NIST14.lib
SI:95  Formula:C19H32O2  CAS:301-00-8  MolWeight:292  RetIndex:2101
CompName:9,12,15-Octadecatrienoic acid, methyl ester, (Z,Z,Z)- $$ Linolenic acid, methyl ester $$ Methyl all-cis-9,12,15-octadecatrienoate $$ Methyl lino

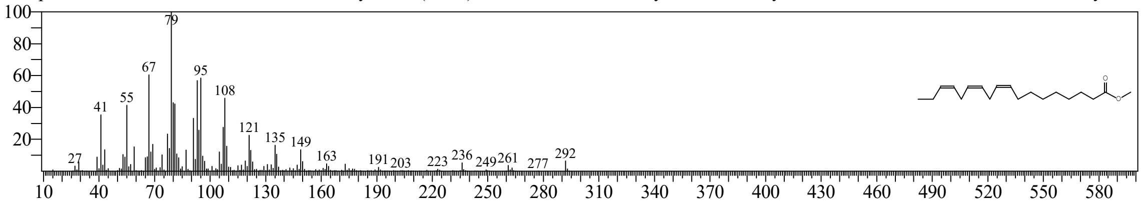


Hit#:2  Entry:135724  Library:NIST17-1.lib
SI:95  Formula:C19H32O2  CAS:301-00-8  MolWeight:292  RetIndex:2101
CompName:9,12,15-Octadecatrienoic acid, methyl ester, (Z,Z,Z)- $$ Linolenic acid, methyl ester $$ Methyl all-cis-9,12,15-octadecatrienoate $$ Methyl lino

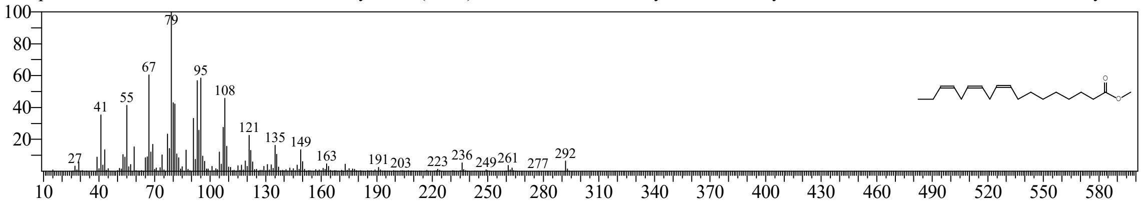


Hit#:3  Entry:121536  Library:NIST17-1.lib
SI:94  Formula:C18H30O2  CAS:0-00-0  MolWeight:278  RetIndex:2002
CompName:Methyl 8,11,14-heptadecatrienoate

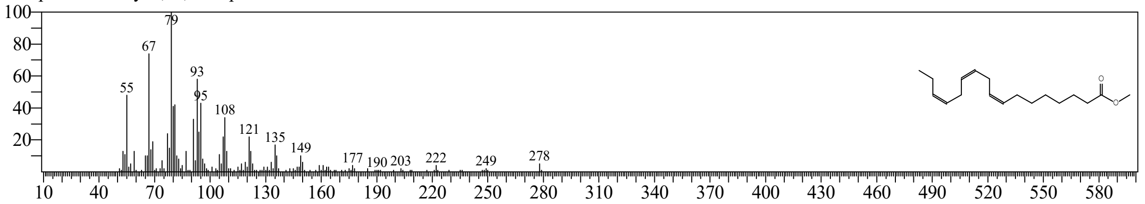


Hit#:4  Entry:111654  Library:NIST14.lib
SI:94  Formula:C18H30O2  CAS:0-00-0  MolWeight:278  RetIndex:2002
CompName:Methyl 8,11,14-heptadecatrienoate

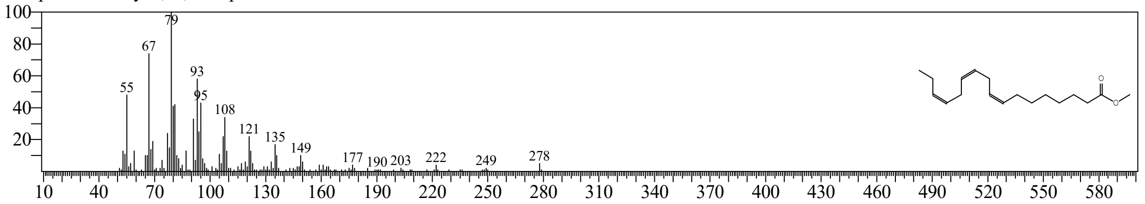


Hit#:5  Entry:32437  Library:NIST17s.lib
SI:94  Formula:C19H32O2  CAS:301-00-8  MolWeight:292  RetIndex:2101
CompName:9,12,15-Octadecatrienoic acid, methyl ester, (Z,Z,Z)- $$ Linolenic acid, methyl ester $$ Methyl all-cis-9,12,15-octadecatrienoate $$ Methyl lino

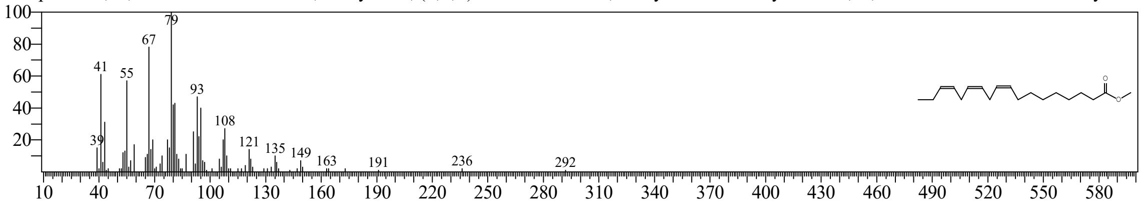

<< Target >>
Line#:16 R.Time:30.617(Scan#:2475) MassPeaks:407
RawMode:Averaged 30.608-30.625(2474-2476) BasePeak:79.05(96633)
BG Mode:Calc. from Peak Group 1 - Event 1

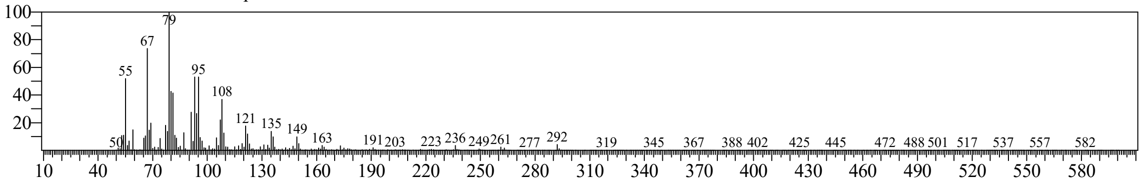


Hit#:1 Entry:124228 Library:NIST14.lib
SI:96 Formula:C19H32O2 CAS:301-00-8 MolWeight:292 RetIndex:2101
CompName:9,12,15-Octadecatrienoic acid, methyl ester, (Z,Z,Z)- $$ Linolenic acid, methyl ester $$ Methyl all-cis-9,12,15-octadecatrienoate $$ Methyl lino

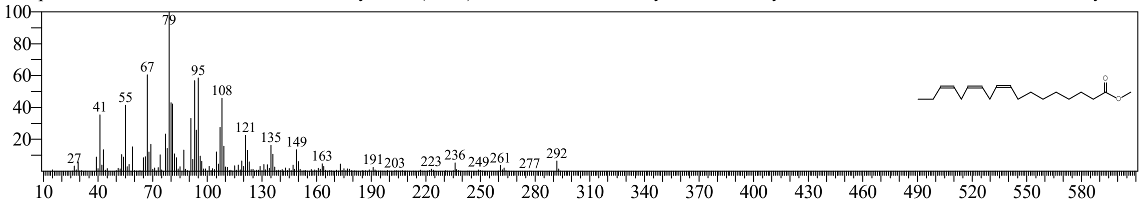


Hit#:2 Entry:135724 Library:NIST17-1.lib
SI:96 Formula:C19H32O2 CAS:301-00-8 MolWeight:292 RetIndex:2101
CompName:9,12,15-Octadecatrienoic acid, methyl ester, (Z,Z,Z)- $$ Linolenic acid, methyl ester $$ Methyl all-cis-9,12,15-octadecatrienoate $$ Methyl lino

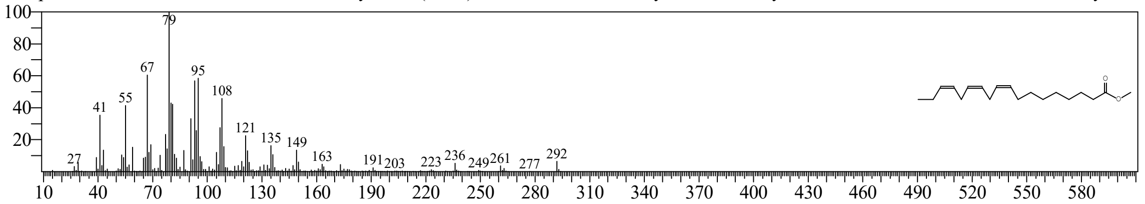


Hit#:3 Entry:32438 Library:NIST17s.lib
SI:95 Formula:C19H32O2 CAS:301-00-8 MolWeight:292 RetIndex:2101
CompName:9,12,15-Octadecatrienoic acid, methyl ester, (Z,Z,Z)- $$ Linolenic acid, methyl ester $$ Methyl all-cis-9,12,15-octadecatrienoate $$ Methyl lino

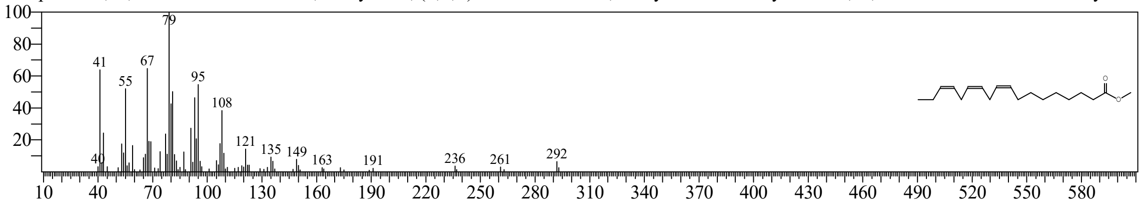


Hit#:4 Entry:111654 Library:NIST14.lib
SI:95 Formula:C18H30O2 CAS:0-00-0 MolWeight:278 RetIndex:2002
CompName:Methyl 8,11,14-heptadecatrienoate

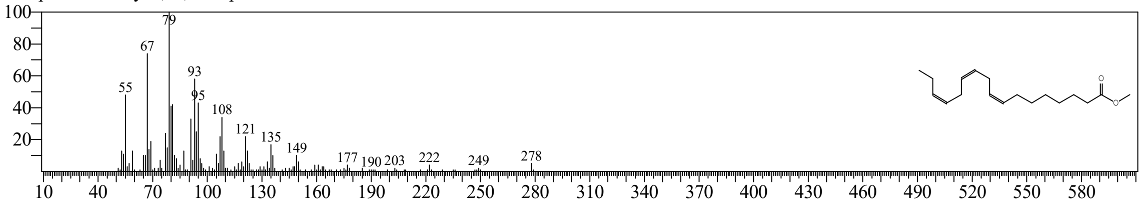


Hit#:5 Entry:121536 Library:NIST17-1.lib
SI:95 Formula:C18H30O2 CAS:0-00-0 MolWeight:278 RetIndex:2002
CompName:Methyl 8,11,14-heptadecatrienoate

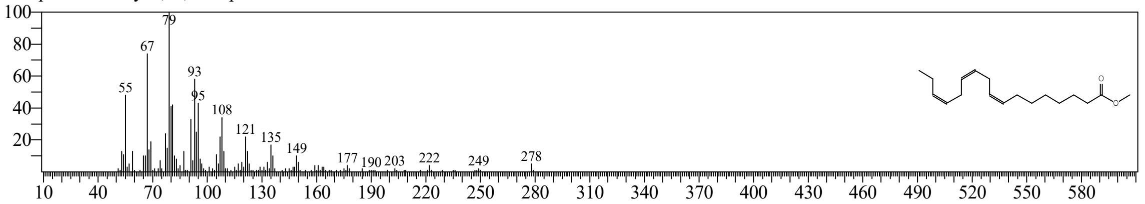

<< Target >>
Line#:17  R.Time:30.942(Scan#:2514)  MassPeaks:342
RawMode:Averaged 30.933-30.950(2513-2515) BasePeak:67.05(4323)
BG Mode:Calc. from Peak  Group 1 - Event 1

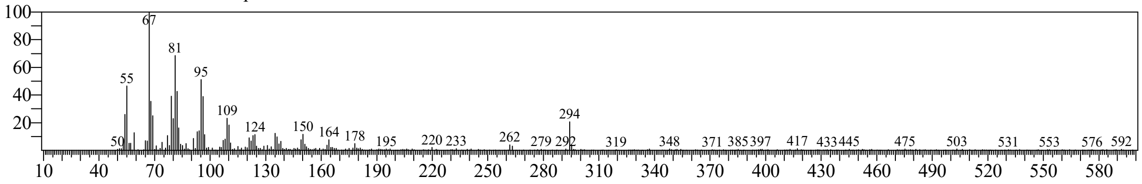


Hit#:1  Entry:137666  Library:NIST17-1.lib
SI:97  Formula:C19H34O2  CAS:13058-52-1  MolWeight:294  RetIndex:2093
CompName:Methyl 9-cis,11-trans-octadecadienoate

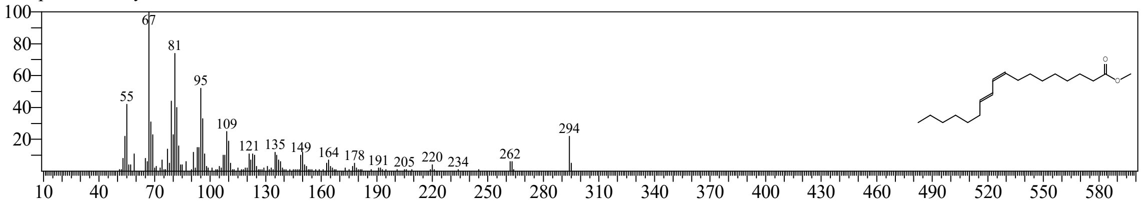


Hit#:2  Entry:137664  Library:NIST17-1.lib
SI:97  Formula:C19H34O2  CAS:0-00-0  MolWeight:294  RetIndex:2093
CompName:Methyl 10-trans,12-cis-octadecadienoate

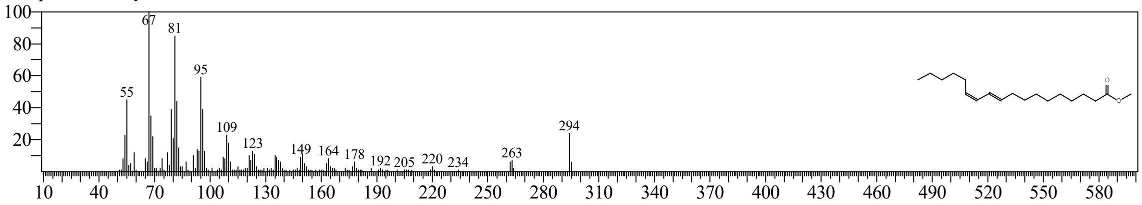


Hit#:3  Entry:125933  Library:NIST14.lib
SI:97  Formula:C19H34O2  CAS:0-00-0  MolWeight:294  RetIndex:2093
CompName:Methyl 10-trans,12-cis-octadecadienoate

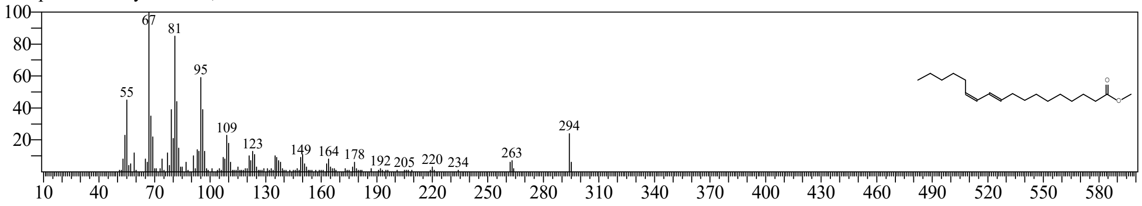


Hit#:4  Entry:125932  Library:NIST14.lib
SI:97  Formula:C19H34O2  CAS:0-00-0  MolWeight:294  RetIndex:2093
CompName:Methyl 9-cis,11-trans-octadecadienoate

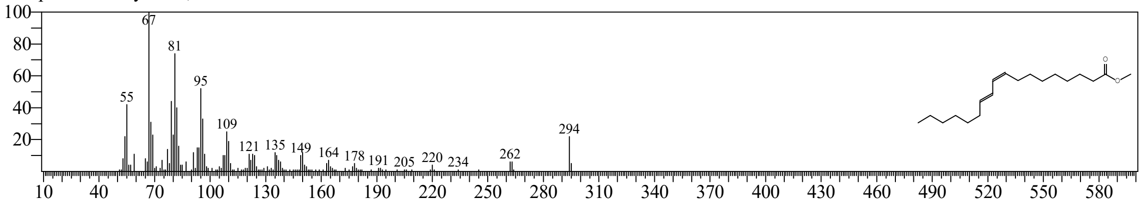


Hit#:5  Entry:32587  Library:NIST17s.lib
SI:95  Formula:C19H34O2  CAS:13038-47-6  MolWeight:294  RetIndex:2093
CompName:9,11-Octadecadienoic acid, methyl ester, (E,E)- $$ Methyl trans-9,trans-11-octadecadienoate $$ Methyl trans,trans-9,11-octadecadienoate $$ M

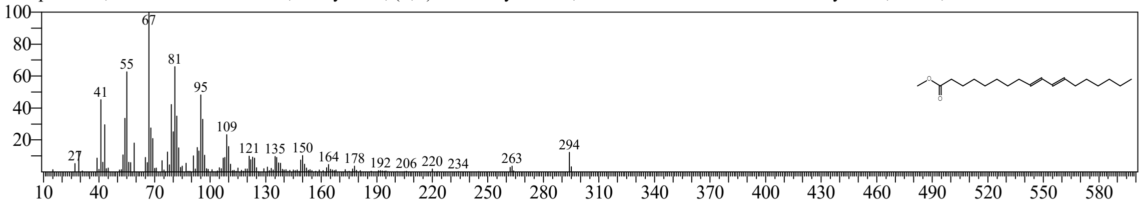

<< Target >>
Line#:18 R.Time:31.275(Scan#:2554) MassPeaks:314
RawMode:Averaged 31.267-31.283(2553-2555) BasePeak:67.05(5030)
BG Mode:Calc. from Peak Group 1 - Event 1

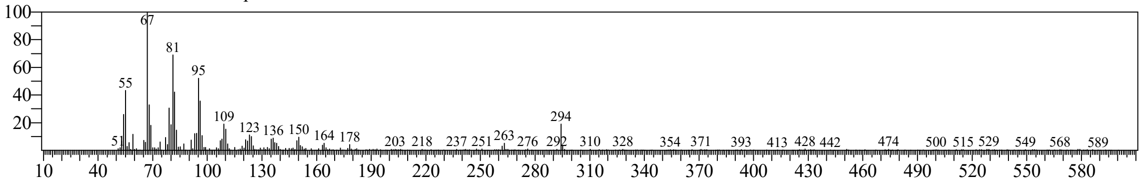


Hit#:1 Entry:125933 Library:NIST14.lib
SI:96 Formula:C19H34O2 CAS:0-00-0 MolWeight:294 RetIndex:2093
CompName:Methyl 10-trans,12-cis-octadecadienoate

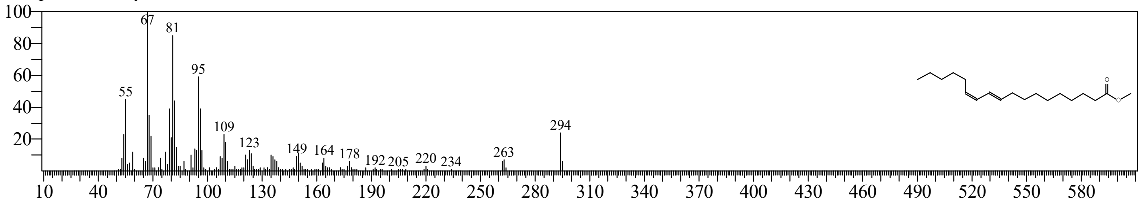


Hit#:2 Entry:137664 Library:NIST17-1.lib
SI:96 Formula:C19H34O2 CAS:0-00-0 MolWeight:294 RetIndex:2093
CompName:Methyl 10-trans,12-cis-octadecadienoate

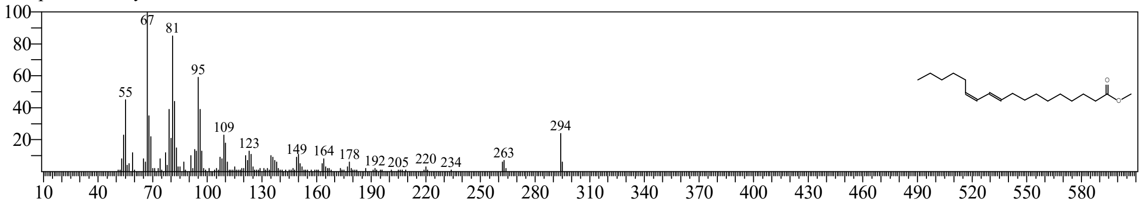


Hit#:3 Entry:125932 Library:NIST14.lib
SI:96 Formula:C19H34O2 CAS:0-00-0 MolWeight:294 RetIndex:2093
CompName:Methyl 9-cis,11-trans-octadecadienoate

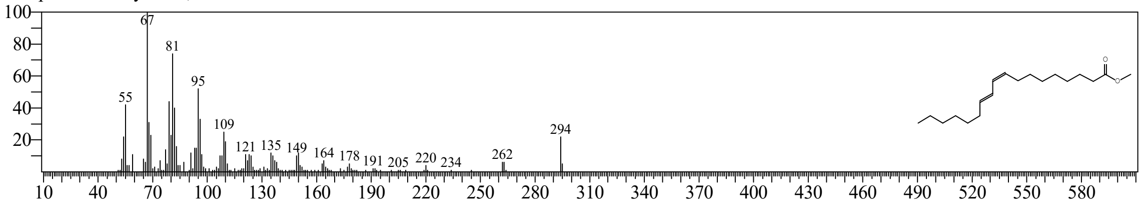


Hit#:4 Entry:137666 Library:NIST17-1.lib
SI:96 Formula:C19H34O2 CAS:13058-52-1 MolWeight:294 RetIndex:2093
CompName:Methyl 9-cis,11-trans-octadecadienoate

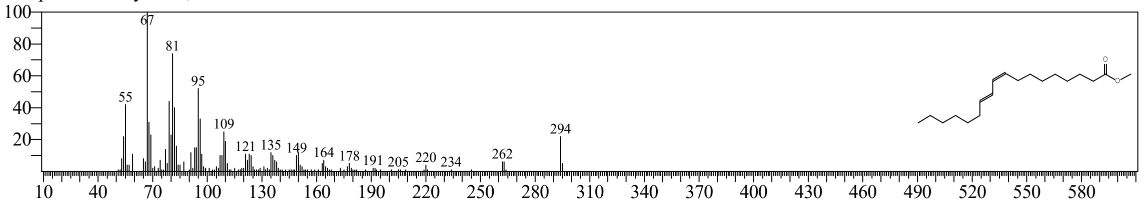


Hit#:5 Entry:32587 Library:NIST17s.lib
SI:94 Formula:C19H34O2 CAS:13038-47-6 MolWeight:294 RetIndex:2093
CompName:9,11-Octadecadienoic acid, methyl ester, (E,E)- $$ Methyl trans-9,trans-11-octadecadienoate $$ Methyl trans,trans-9,11-octadecadienoate $$ M

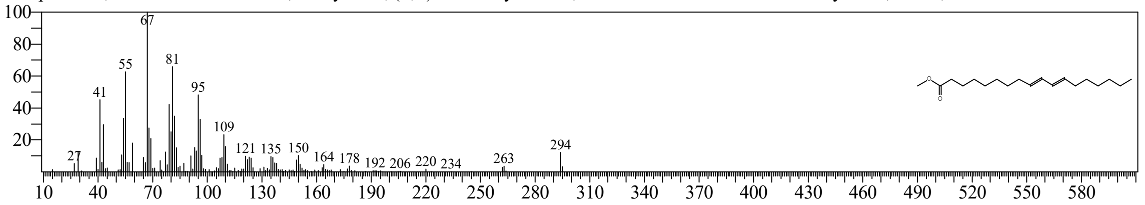

<< Target >>
Line#:19   R.Time:32.967(Scan#:2757)  MassPeaks:382
RawMode:Averaged 32.958-32.975(2756-2758) BasePeak:95.10(7422)
BG Mode:Calc. from Peak  Group 1 - Event 1

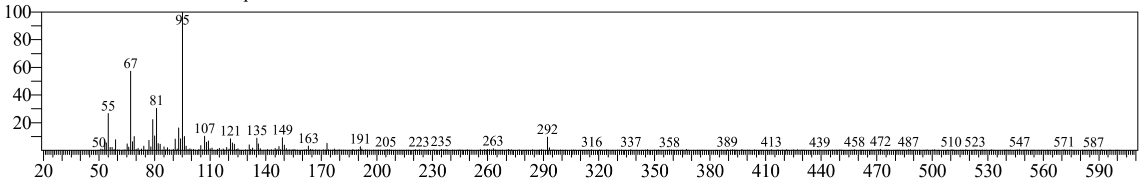


Hit#:1  Entry:24424  Library:NIST17-1.lib
SI:85  Formula:C12H20  CAS:0-00-0  MolWeight:164  RetIndex:1041
CompName:Cis-3-ethyl-endo-tricyclo[5.2.1.0(2.6)]decane

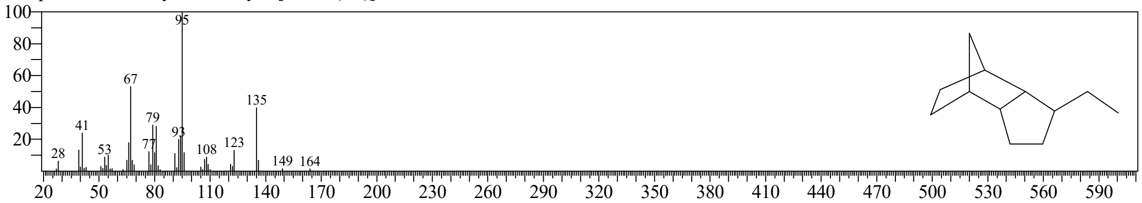


Hit#:2  Entry:23403  Library:NIST14.lib
SI:85  Formula:C12H20  CAS:0-00-0  MolWeight:164  RetIndex:1041
CompName:Cis-3-ethyl-endo-tricyclo[5.2.1.0(2.6)]decane

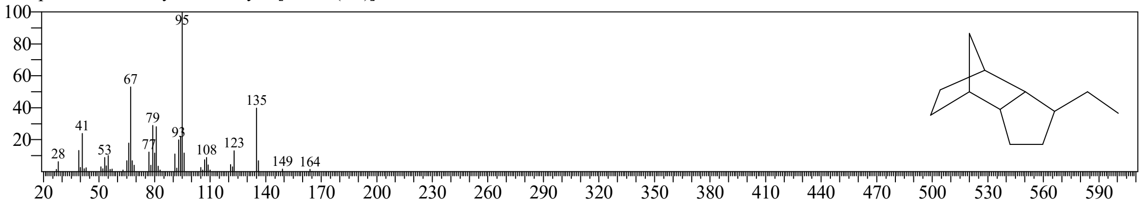


Hit#:3  Entry:16582  Library:NIST17-1.lib
SI:83  Formula:C11H18  CAS:0-00-0  MolWeight:150  RetIndex:1115
CompName:4-Cyclohexenyl-3-methylbutene

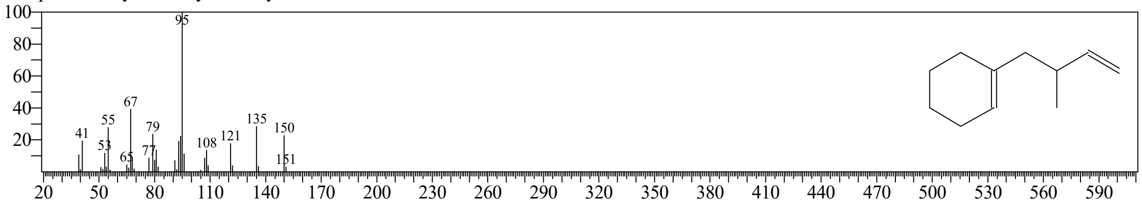


Hit#:4  Entry:16897  Library:NIST14.lib
SI:82  Formula:C10H16O  CAS:60485-45-2  MolWeight:152  RetIndex:991
CompName:Santolina epoxide $$ 2-Methyl-2-(3-methyl-1-vinyl-2-butenyl)oxirane # $$

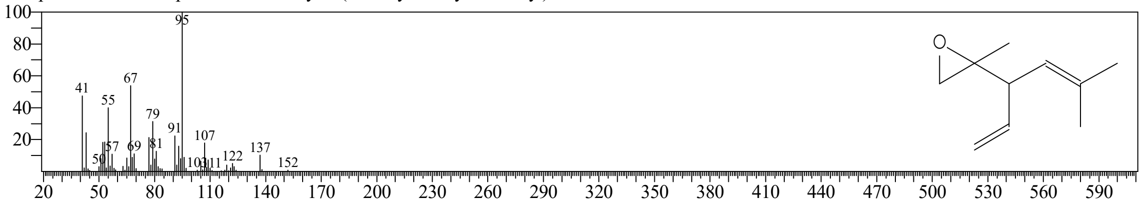


Hit#:5  Entry:17571  Library:NIST17-1.lib
SI:82  Formula:C10H16O  CAS:60485-45-2  MolWeight:152  RetIndex:991
CompName:Santolina epoxide $$ 2-Methyl-2-(3-methyl-1-vinyl-2-butenyl)oxirane # $$

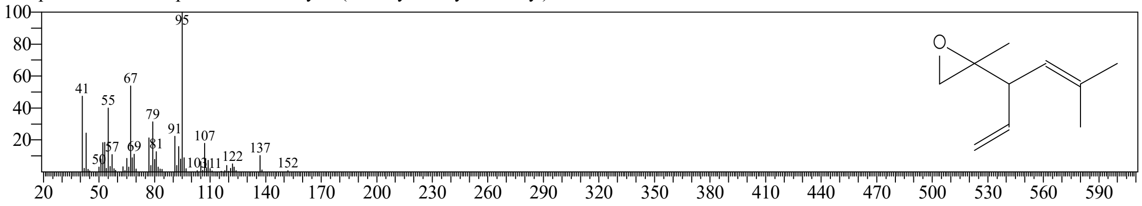

<< Target >>
Line#:20 R.Time:33.500(Scan#:2821) MassPeaks:327
RawMode:Averaged 33.492-33.508(2820-2822) BasePeak:74.00(11526)
BG Mode:Calc. from Peak Group 1 - Event 1

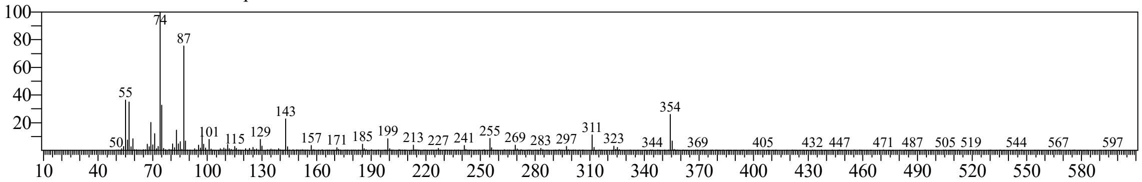

Hit#:1 Entry:178051 Library:NIST14.lib
SI:96 Formula:C23H46O2 CAS:929-77-1 MolWeight:354 RetIndex:2475
CompName:Docosanoic acid, methyl ester $$ Behenic acid, methyl ester $$ Methyl behenate $$ Methyl docosanoate $$ n-Docosanoic acid methyl ester $$

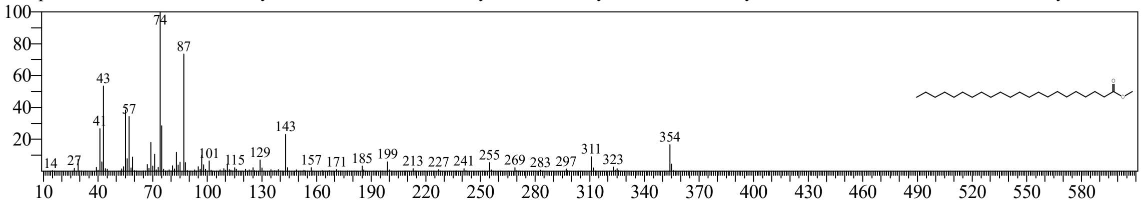

Hit#:2 Entry:196443 Library:NIST17-1.lib
SI:96 Formula:C23H46O2 CAS:929-77-1 MolWeight:354 RetIndex:2475
CompName:Docosanoic acid, methyl ester $$ Behenic acid, methyl ester $$ Methyl behenate $$ Methyl docosanoate $$ n-Docosanoic acid methyl ester $$

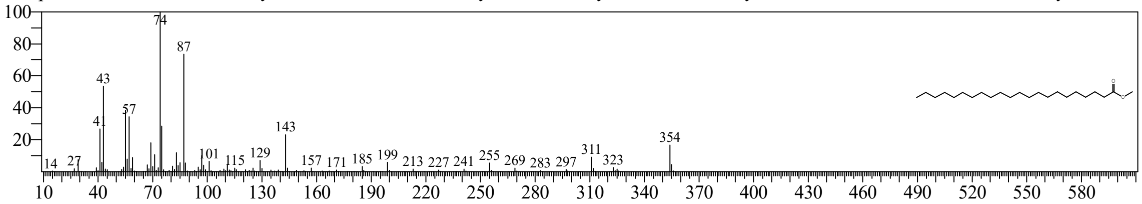

Hit#:3 Entry:178052 Library:NIST14.lib
SI:95 Formula:C23H46O2 CAS:0-00-0 MolWeight:354 RetIndex:2411
CompName:Methyl 20-methyl-heneicosanoate

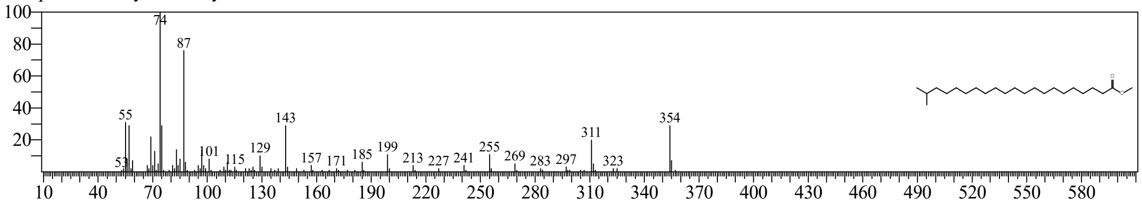

Hit#:4 Entry:196444 Library:NIST17-1.lib
SI:95 Formula:C23H46O2 CAS:0-00-0 MolWeight:354 RetIndex:2411
CompName:Methyl 20-methyl-heneicosanoate

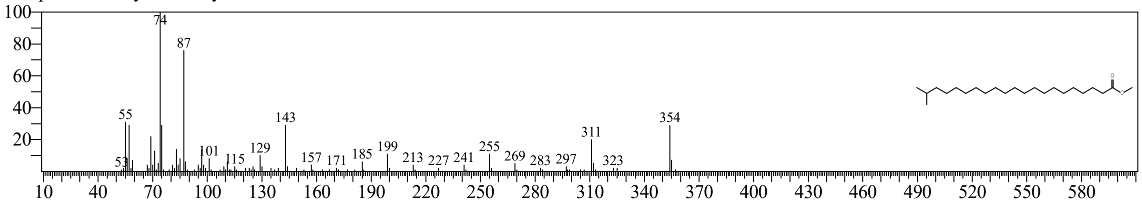

Hit#:5 Entry:31088 Library:NIST14s.lib
SI:95 Formula:C23H46O2 CAS:929-77-1 MolWeight:354 RetIndex:2475
CompName:Docosanoic acid, methyl ester $$ Behenic acid, methyl ester $$ Methyl behenate $$ Methyl docosanoate $$ n-Docosanoic acid methyl ester $$

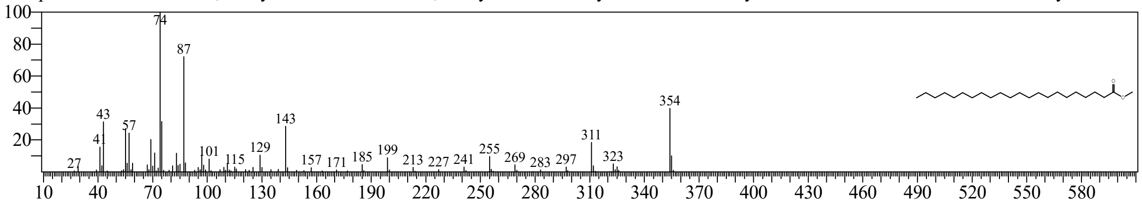

<< Target >>
Line#:21 R.Time:37.792(Scan#:3336) MassPeaks:362
RawMode:Averaged 37.783-37.800(3335-3337) BasePeak:74.05(2445)
BG Mode:Calc. from Peak Group 1 - Event 1

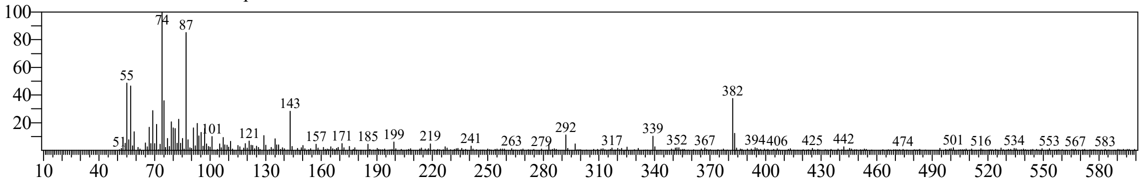


Hit#:1 Entry:31970 Library:NIST14s.lib
SI:82 Formula:C25H50O2 CAS:2442-49-1 MolWeight:382 RetIndex:2674
CompName:Tetracosanoic acid, methyl ester $$ Methyl lignocerate $$ Methyl tetracosanoate $$ Lignoceric acid methyl ester $$

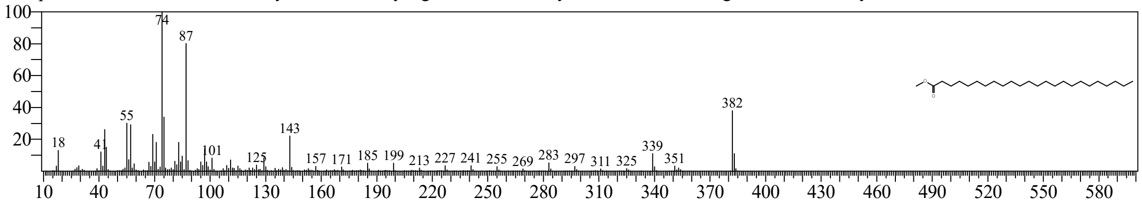


Hit#:2 Entry:37106 Library:NIST17s.lib
SI:82 Formula:C25H50O2 CAS:2442-49-1 MolWeight:382 RetIndex:2674
CompName:Tetracosanoic acid, methyl ester $$ Methyl lignocerate $$ Methyl tetracosanoate $$ Lignoceric acid methyl ester $$

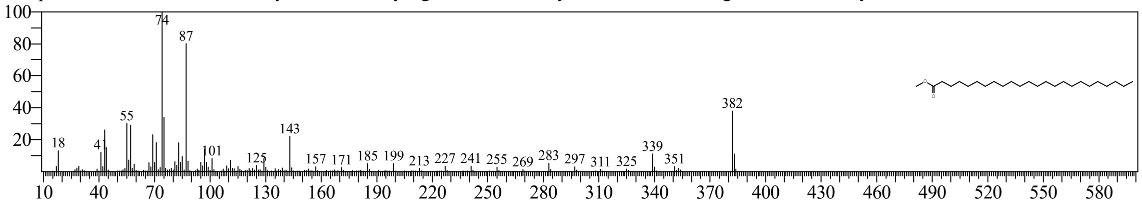


Hit#:3 Entry:31971 Library:NIST14s.lib
SI:80 Formula:C25H50O2 CAS:2442-49-1 MolWeight:382 RetIndex:2674
CompName:Tetracosanoic acid, methyl ester $$ Methyl lignocerate $$ Methyl tetracosanoate $$ Lignoceric acid methyl ester $$

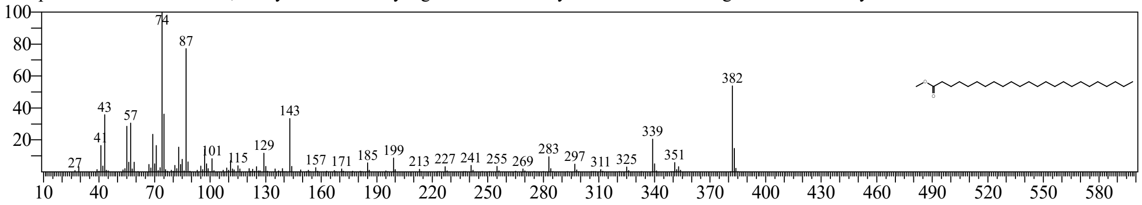


Hit#:4 Entry:37107 Library:NIST17s.lib
SI:80 Formula:C25H50O2 CAS:2442-49-1 MolWeight:382 RetIndex:2674
CompName:Tetracosanoic acid, methyl ester $$ Methyl lignocerate $$ Methyl tetracosanoate $$ Lignoceric acid methyl ester $$

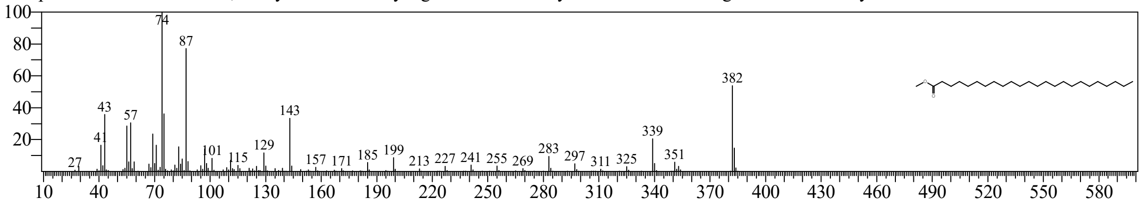


Hit#:5 Entry:167027 Library:NIST14.lib
SI:78 Formula:C22H44O2 CAS:0-00-0 MolWeight:340 RetIndex:2311
CompName:Methyl 18-methylicosanoate

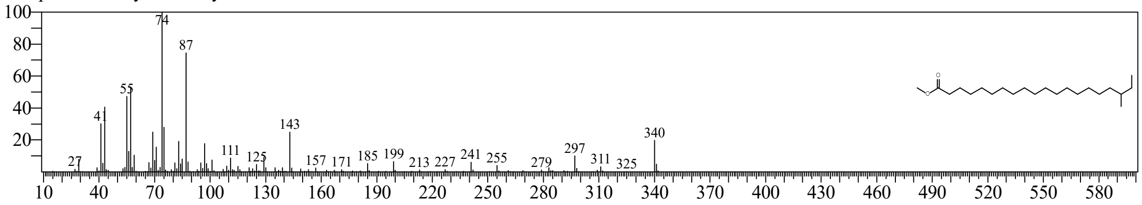

<< Target >>
Line#:22 R.Time:38.325(Scan#:3400) MassPeaks:327
RawMode:Averaged 38.317-38.333(3399-3401) BasePeak:79.05(708)
BG Mode:Calc. from Peak Group 1 - Event 1

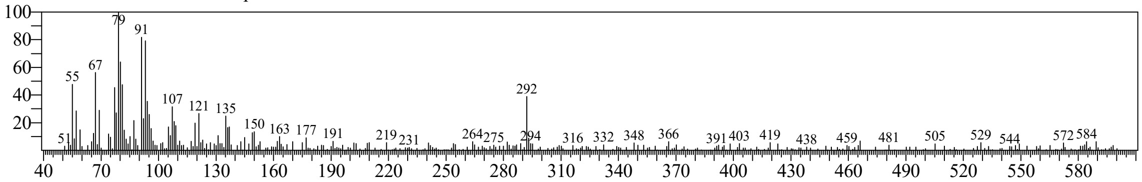


Hit#:1 Entry:135726 Library:NIST17-1.lib
SI:87 Formula:C19H32O2 CAS:0-00-0 MolWeight:292 RetIndex:2101
CompName:Methyl 9.cis.,11.trans.t,13.trans.-octadecatrienoate

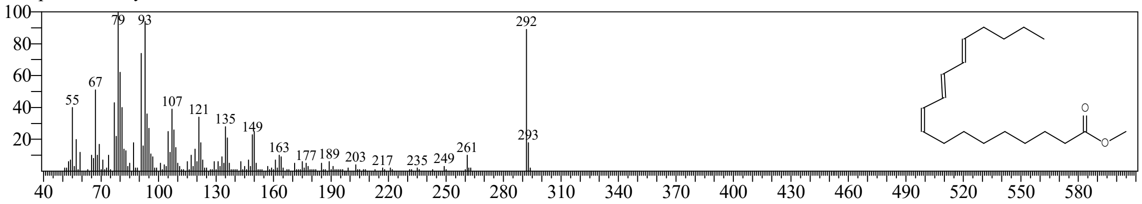


Hit#:2 Entry:124230 Library:NIST14.lib
SI:87 Formula:C19H32O2 CAS:0-00-0 MolWeight:292 RetIndex:2101
CompName:Methyl 9.cis.,11.trans.t,13.trans.-octadecatrienoate

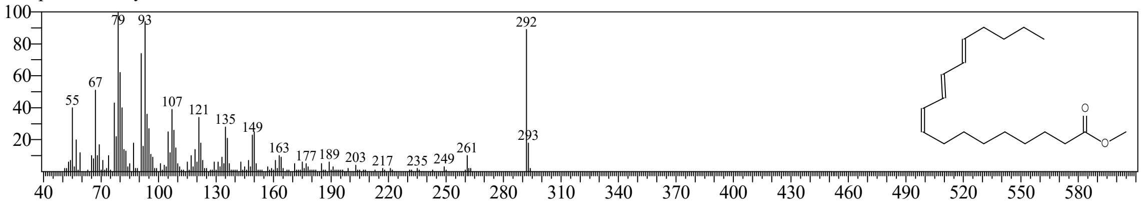


Hit#:3 Entry:164434 Library:NIST17-1.lib
SI:85 Formula:C21H36O2 CAS:21061-10-9 MolWeight:320 RetIndex:2300
CompName:8,11,14-Eicosatrienoic acid, methyl ester, (Z,Z,Z)- $$ Methyl dihomo-.gamma.-linolenate $$ Methyl (8Z,11Z,14Z)-8,11,14-icosatrienoate # $$ I

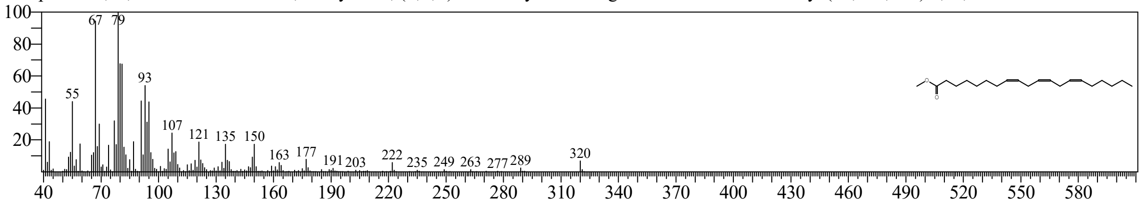


Hit#:4 Entry:29611 Library:NIST14s.lib
SI:84 Formula:C21H36O2 CAS:17364-32-8 MolWeight:320 RetIndex:2300
CompName:8,11,14-Eicosatrienoic acid, methyl ester $$ Methyl (8E,11E,14E)-8,11,14-icosatrienoate # $$

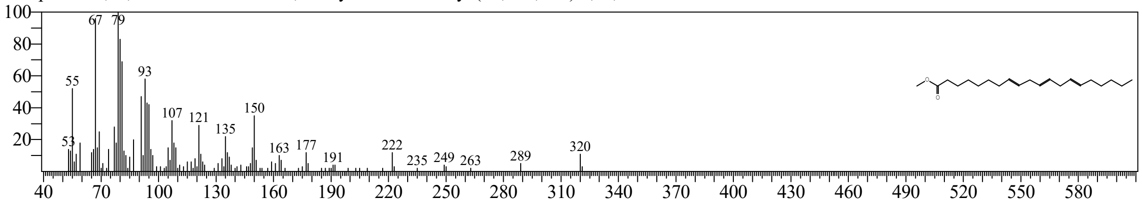


Hit#:5 Entry:34364 Library:NIST17s.lib
SI:84 Formula:C21H36O2 CAS:17364-32-8 MolWeight:320 RetIndex:2300
CompName:8,11,14-Eicosatrienoic acid, methyl ester $$ Methyl (8E,11E,14E)-8,11,14-icosatrienoate # $$

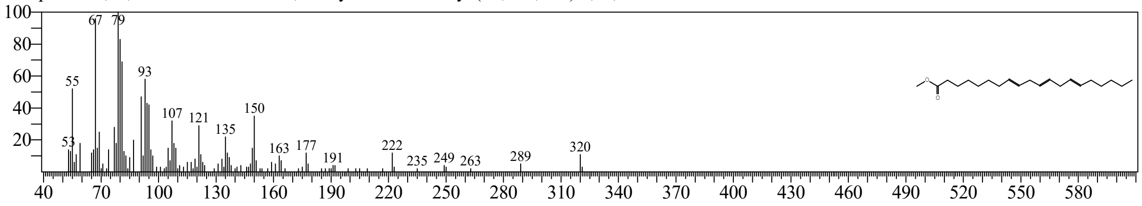

<< Target >>
Line#:23 R.Time:38.758(Scan#:3452) MassPeaks:323
RawMode:Averaged 38.750-38.767(3451-3453) BasePeak:79.05(749)
BG Mode:Calc. from Peak Group 1 - Event 1

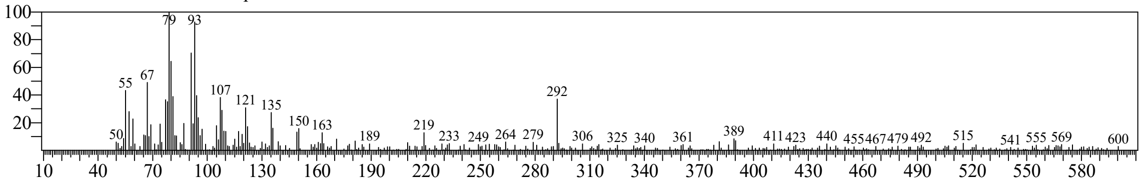


Hit#:1 Entry:135726 Library:NIST17-1.lib
SI:90 Formula:C19H32O2 CAS:0-00-0 MolWeight:292 RetIndex:2101
CompName:Methyl 9.cis.,11.trans.t,13.trans.-octadecatrienoate

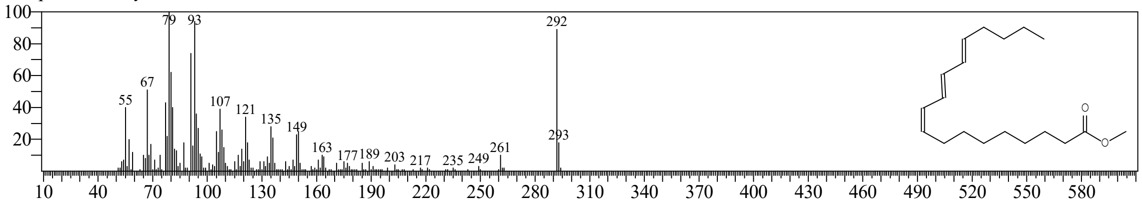


Hit#:2 Entry:124230 Library:NIST14.lib
SI:90 Formula:C19H32O2 CAS:0-00-0 MolWeight:292 RetIndex:2101
CompName:Methyl 9.cis.,11.trans.t,13.trans.-octadecatrienoate

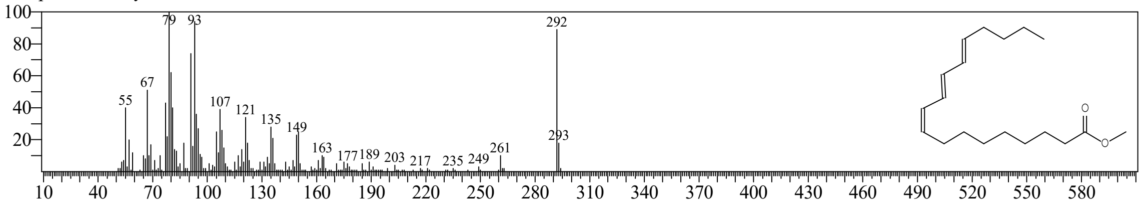


Hit#:3 Entry:135721 Library:NIST17-1.lib
SI:85 Formula:C19H32O2 CAS:16326-32-2 MolWeight:292 RetIndex:2101
CompName:Methyl .gamma.-linolenate

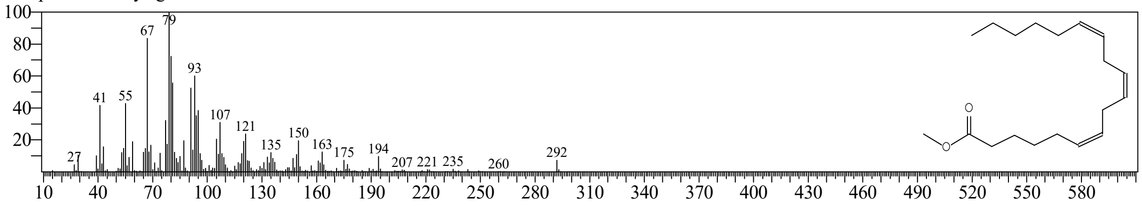


Hit#:4 Entry:124225 Library:NIST14.lib
SI:85 Formula:C19H32O2 CAS:0-00-0 MolWeight:292 RetIndex:2101
CompName:.gamma.-Linolenic acid, methyl ester

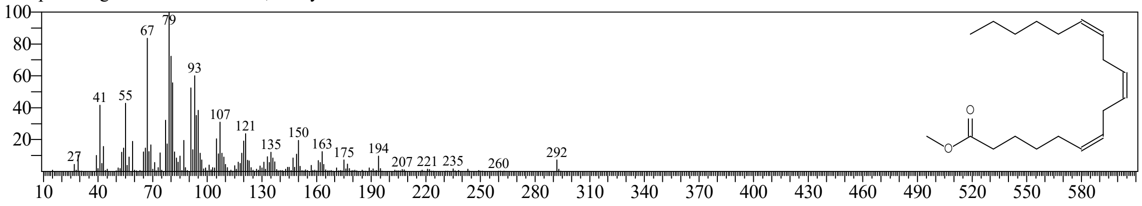


Hit#:5 Entry:124226 Library:NIST14.lib
SI:85 Formula:C19H32O2 CAS:0-00-0 MolWeight:292 RetIndex:2101
CompName:Methyl 6-cis,9-cis,11-trans-octadecatrienoate

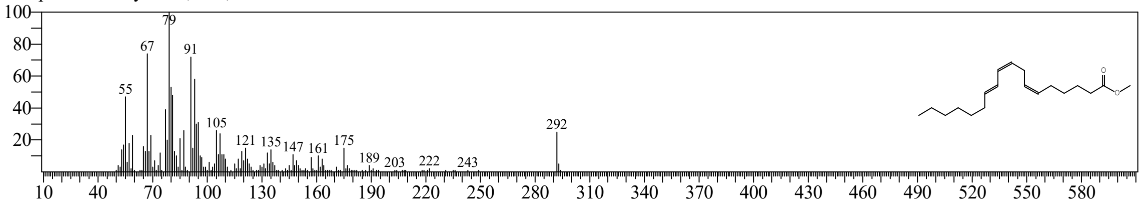

<< Target >>
Line#:24 R.Time:38.892(Scan#:3468) MassPeaks:374
RawMode:Averaged 38.883-38.900(3467-3469) BasePeak:79.05(839)
BG Mode:Calc. from Peak Group 1 - Event 1

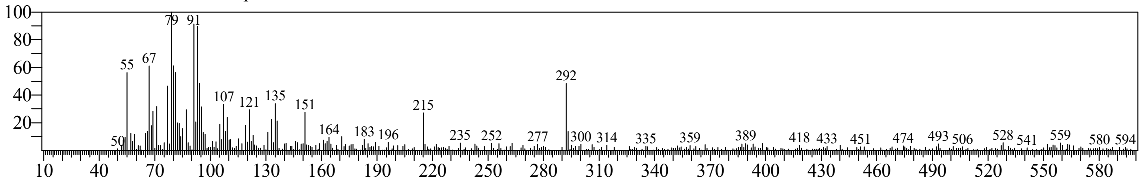


Hit#:1 Entry:135726 Library:NIST17-1.lib
SI:84 Formula:C19H32O2 CAS:0-00-0 MolWeight:292 RetIndex:2101
CompName:Methyl 9.cis.,11.trans.t,13.trans.-octadecatrienoate

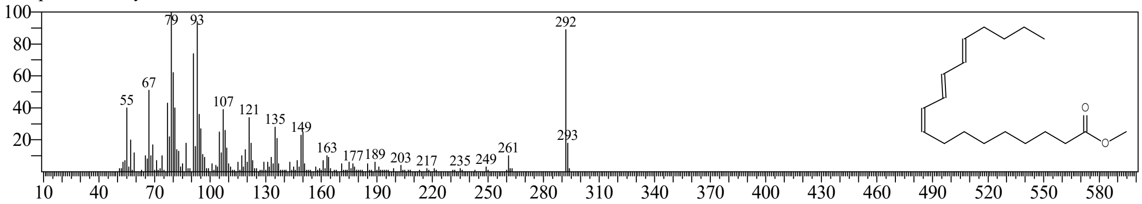


Hit#:2 Entry:124230 Library:NIST14.lib
SI:84 Formula:C19H32O2 CAS:0-00-0 MolWeight:292 RetIndex:2101
CompName:Methyl 9.cis.,11.trans.t,13.trans.-octadecatrienoate

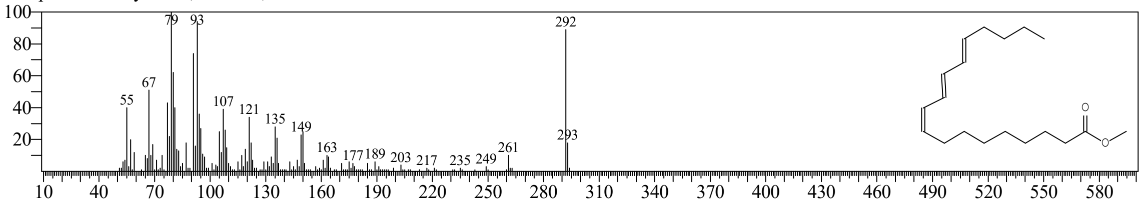


Hit#:3 Entry:124226 Library:NIST14.lib
SI:82 Formula:C19H32O2 CAS:0-00-0 MolWeight:292 RetIndex:2101
CompName:Methyl 6-cis,9-cis,11-trans-octadecatrienoate

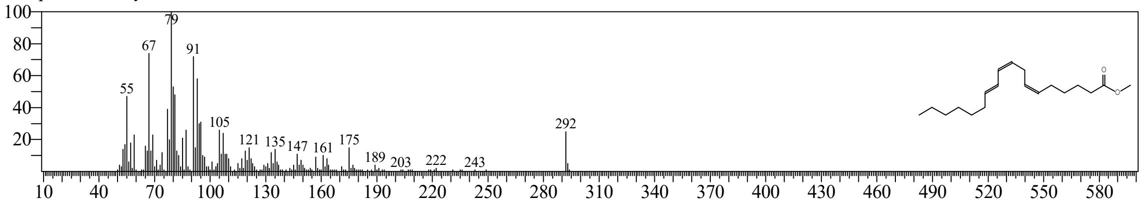


Hit#:4 Entry:135722 Library:NIST17-1.lib
SI:82 Formula:C19H32O2 CAS:0-00-0 MolWeight:292 RetIndex:2101
CompName:Methyl 6-cis,9-cis,11-trans-octadecatrienoate

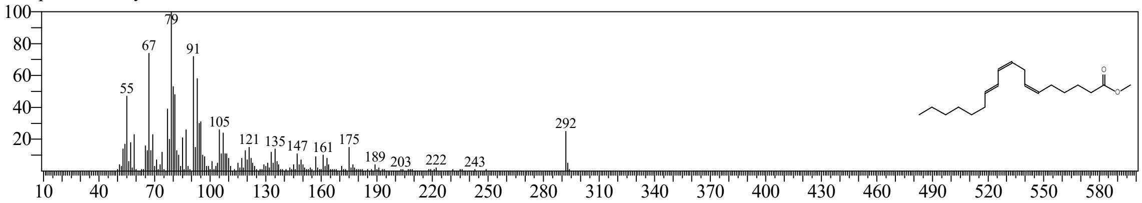


Hit#:5 Entry:164431 Library:NIST17-1.lib
SI:82 Formula:C21H36O2 CAS:0-00-0 MolWeight:320 RetIndex:2300
CompName:cis-5,8,11-Eicosatrienoic acid, methyl ester

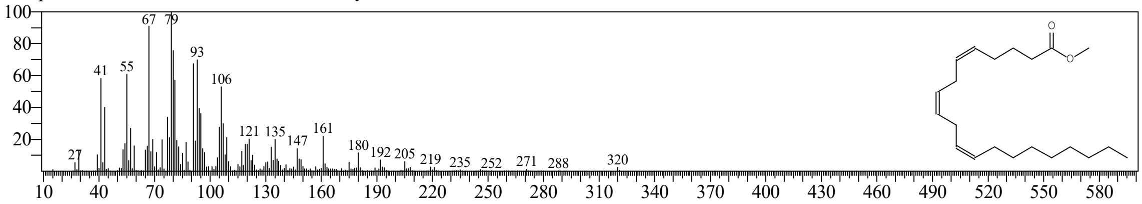

Supplement: Supplementary file 1 [file molecules-31-02891-s001.zip › 28d-CK-3.pdf]
