# Supplementary material for: Selective Inhibition of Lipid Oxidation by a Quercetagetin-Rich Extract from Tagetes erecta: A Chemometric Approach Using OPLS-DA and Selectivity Index Analysis
Source: Molecules. 2026 Aug 19;31(16):2891. doi: 10.3390/molecules31162891 (PMC13515886; doi:10.3390/molecules31162891)

# QG200-1-28d

<<Column>>
Name : HP-88
Thickness : 0.20um
Length : 100.0m
Inside Diameter : 0.25mm

## Method

===== Analytical Line 1 =====

| | | |
|---|---|---|
| Column Oven Temp. | :120.0 °C | |
| Injection Temp. | :250.00 °C | |
| Injection Mode | :Split | |
| Column Flow | :1.04 mL/min | |
| Split Ratio | :20.0 | |
| Oven Temp. Program | | |
| Rate | Temperature(°C) | Hold Time(min) |
| - | 120.0 | 1.00 |
| 10.00 | 175.0 | 10.00 |
| 5.00 | 210.0 | 5.00 |
| 5.00 | 230.0 | 15.00 |

[GCMS-QP2010]
IonSourceTemp :200.00 °C
Interface Temp. :220.00 °C
*m/z* scanning range：45～500m/z

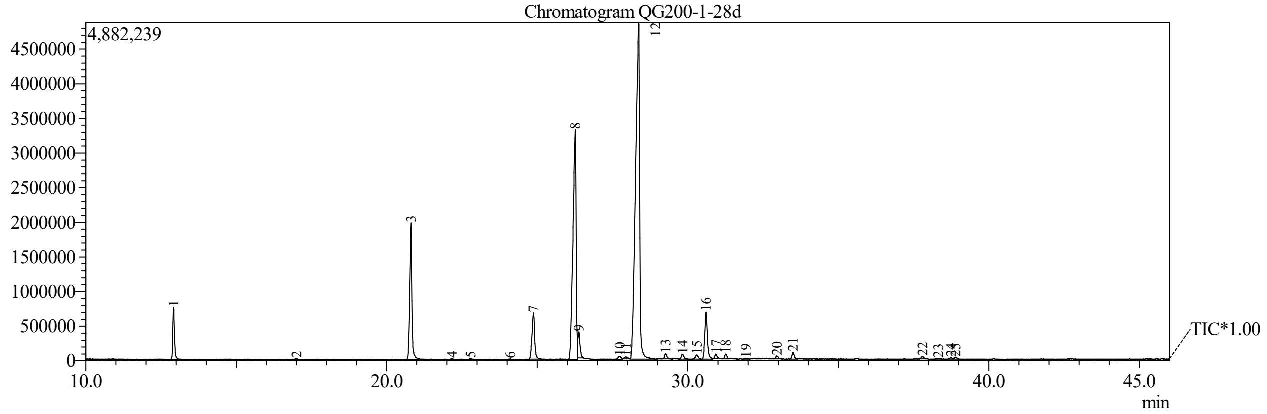


Peak Report TIC

| Peak# | R.Time | Area | Area% | Height | Height% | A/H | Name |
|---|---|---|---|---|---|---|---|
| 1 | 12.918 | 2827930 | 2.92 | 753992 | 5.65 | 3.75 | Undecanoic acid, methyl ester |
| 2 | 16.996 | 59345 | 0.06 | 14351 | 0.11 | 4.14 | Methyl tetradecanoate |
| 3 | 20.808 | 10465573 | 10.81 | 1973904 | 14.80 | 5.30 | Hexadecanoic acid, methyl ester |
| 4 | 22.171 | 80588 | 0.08 | 14247 | 0.11 | 5.66 | 9-Hexadecenoic acid, methyl ester, (Z)- |
| 5 | 22.784 | 96358 | 0.10 | 18407 | 0.14 | 5.23 | Heptadecanoic acid, methyl ester |
| 6 | 24.093 | 45358 | 0.05 | 9411 | 0.07 | 4.82 | Methyl 9-heptadecenoate or 9-17:1 |
| 7 | 24.875 | 4236657 | 4.37 | 673168 | 5.05 | 6.29 | Methyl stearate |
| 8 | 26.261 | 24108661 | 24.90 | 3319828 | 24.89 | 7.26 | 9-Octadecenoic acid, methyl ester, (E)- |
| 9 | 26.384 | 1721527 | 1.78 | 371112 | 2.78 | 4.64 | 11-Octadecenoic acid, methyl ester |
| 10 | 27.738 | 309422 | 0.32 | 44333 | 0.33 | 6.98 | 9,12-Octadecadienoic acid (Z,Z)-, methyl estei |
| 11 | 27.939 | 216782 | 0.22 | 28967 | 0.22 | 7.48 | 9,12-Octadecadienoic acid (Z,Z)-, methyl estei |
| 12 | 28.369 | 45142925 | 46.62 | 4855498 | 36.40 | 9.30 | 9,12-Octadecadienoic acid (Z,Z)-, methyl estei |
| 13 | 29.264 | 414756 | 0.43 | 74376 | 0.56 | 5.58 | Methyl 18-methylnonadecanoate |
| 14 | 29.822 | 388383 | 0.40 | 69704 | 0.52 | 5.57 | 9,12,15-Octadecatrienoic acid, methyl ester, (Z |

| Peak# | R.Time | Area | Area% | Height | Height% | A/H | Name |
|---|---|---|---|---|---|---|---|
| 15 | 30.299 | 409528 | 0.42 | 60345 | 0.45 | 6.79 | 9,12,15-Octadecatrienoic acid, methyl ester, (Z |
| 16 | 30.605 | 4158122 | 4.29 | 675693 | 5.07 | 6.15 | 9,12,15-Octadecatrienoic acid, methyl ester, (Z |
| 17 | 30.936 | 338113 | 0.35 | 63635 | 0.48 | 5.31 | Methyl 9-cis,11-trans-octadecadienoate |
| 18 | 31.261 | 348282 | 0.36 | 66879 | 0.50 | 5.21 | Methyl 10-trans,12-cis-octadecadienoate |
| 19 | 31.935 | 91018 | 0.09 | 13187 | 0.10 | 6.90 | Methyl 10-trans,12-cis-octadecadienoate |
| 20 | 32.956 | 227593 | 0.24 | 40957 | 0.31 | 5.56 | Cis-3-ethyl-endo-tricyclo[5.2.1.0(2.6)]decane |
| 21 | 33.493 | 545970 | 0.56 | 101230 | 0.76 | 5.39 | Docosanoic acid, methyl ester |
| 22 | 37.798 | 196200 | 0.20 | 32472 | 0.24 | 6.04 | Tetracosanoic acid, methyl ester |
| 23 | 38.324 | 101137 | 0.10 | 12997 | 0.10 | 7.78 | Methyl 9.cis.,11.trans.t,13.trans.-octadecatrien |
| 24 | 38.743 | 118854 | 0.12 | 22453 | 0.17 | 5.29 | Methyl 9.cis.,11.trans.t,13.trans.-octadecatrien |
| 25 | 38.893 | 189296 | 0.20 | 27755 | 0.21 | 6.82 | Methyl 9.cis.,11.trans.t,13.trans.-octadecatrien |
| | | 96838378 | 100.00 | 13338901 | 100.00 | | |

Library

<< Target >>

Line#:1 R.Time:12.917(Scan#:351) MassPeaks:326

RawMode:Averaged 12.908-12.925(350-352) BasePeak:74.00(220912)

BG Mode:Calc. from Peak Group 1 - Event 1

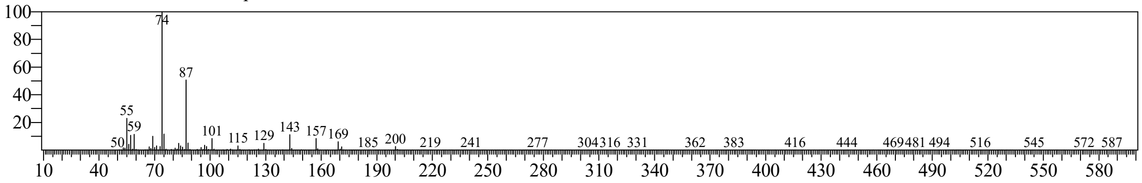

Hit#:1 Entry:46966 Library:NIST14.lib

SI:95 Formula:C12H24O2 CAS:1731-86-8 MolWeight:200 RetIndex:1381

CompName:Undecanoic acid, methyl ester $$ Methyl undecanoate $$ Methyl undecylenate $$ n-Undecanoic acid methyl ester $$ Methyl ester of undecanoi

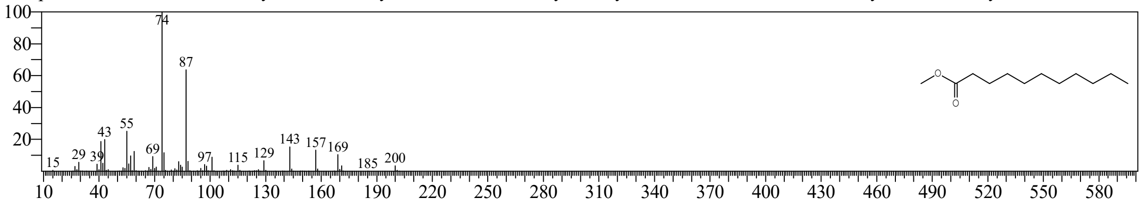

Hit#:2 Entry:49598 Library:NIST17-1.lib

SI:95 Formula:C12H24O2 CAS:1731-86-8 MolWeight:200 RetIndex:1381

CompName:Undecanoic acid, methyl ester $$ Methyl undecanoate $$ Methyl undecylenate $$ n-Undecanoic acid methyl ester $$ Methyl ester of undecanoi

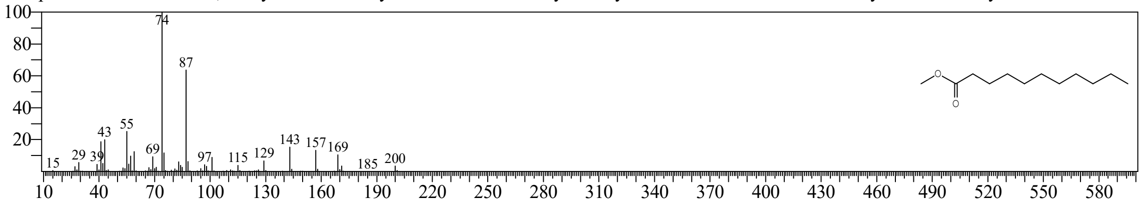

Hit#:3 Entry:21618 Library:NIST17s.lib

SI:95 Formula:C12H24O2 CAS:1731-86-8 MolWeight:200 RetIndex:1381

CompName:Undecanoic acid, methyl ester $$ Methyl undecanoate $$ Methyl undecylenate $$ n-Undecanoic acid methyl ester $$ Methyl ester of undecanoi

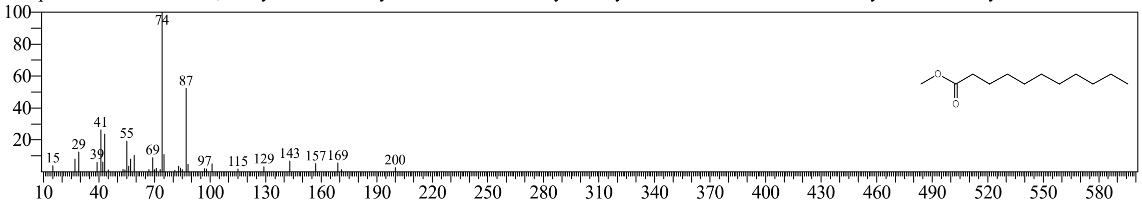

Hit#:4 Entry:18487 Library:NIST14s.lib

SI:95 Formula:C12H24O2 CAS:1731-86-8 MolWeight:200 RetIndex:1381

CompName:Undecanoic acid, methyl ester $$ Methyl undecanoate $$ Methyl undecylenate $$ n-Undecanoic acid methyl ester $$ Methyl ester of undecanoi

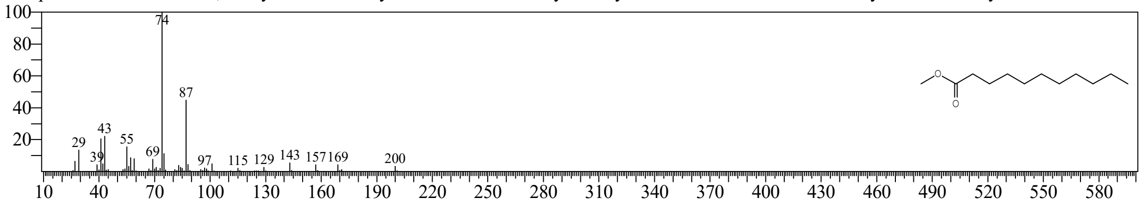

<< Target >>
Line#:1   R.Time:12.917(Scan#:351)  MassPeaks:326
RawMode:Averaged 12.908-12.925(350-352) BasePeak:74.00(220912)
BG Mode:Calc. from Peak  Group 1 - Event 1

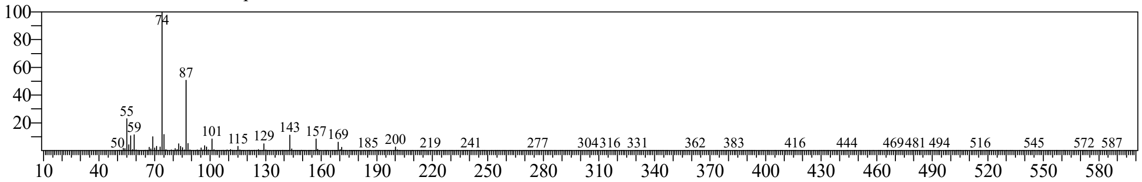


Hit#:5  Entry:21619  Library:NIST17s.lib
SI:95  Formula:C12H24O2  CAS:1731-86-8  MolWeight:200  RetIndex:1381
CompName:Undecanoic acid, methyl ester $$ Methyl undecanoate $$ Methyl undecylenate $$ n-Undecanoic acid methyl ester $$ Methyl ester of undecanoi

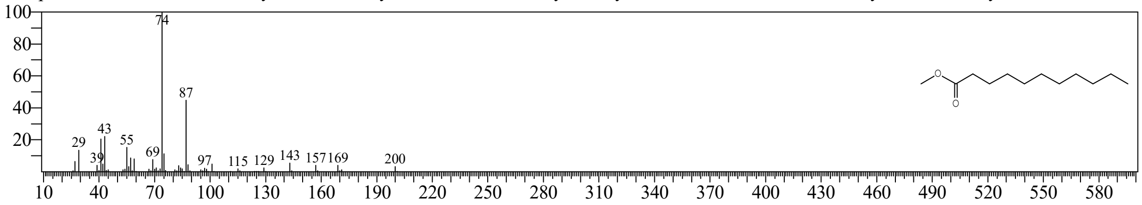

<< Target >>
Line#:2 R.Time:17.000(Scan#:841) MassPeaks:345
RawMode:Averaged 16.992-17.008(840-842) BasePeak:74.00(2782)
BG Mode:Calc. from Peak Group 1 - Event 1

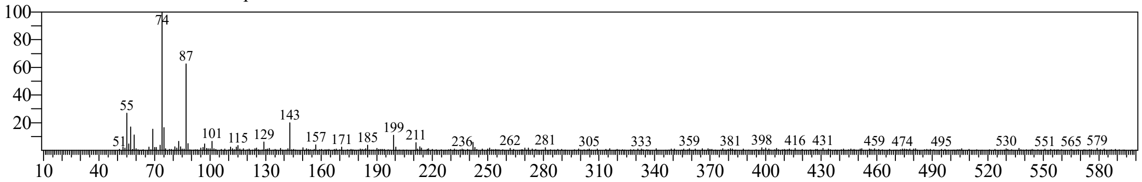


Hit#:1 Entry:80645 Library:NIST14.lib
SI:93 Formula:C15H30O2 CAS:124-10-7 MolWeight:242 RetIndex:1680
CompName:Methyl tetradecanoate $$ Tetradecanoic acid, methyl ester $$ Myristic acid, methyl ester $$ Metholeneat 2495 $$ Methyl myristate $$ Methyl r

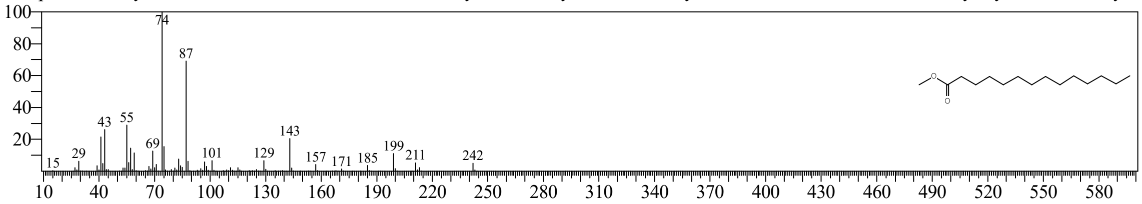


Hit#:2 Entry:86786 Library:NIST17-1.lib
SI:93 Formula:C15H30O2 CAS:124-10-7 MolWeight:242 RetIndex:1680
CompName:Methyl tetradecanoate $$ Tetradecanoic acid, methyl ester $$ Myristic acid, methyl ester $$ Metholeneat 2495 $$ Methyl myristate $$ Methyl r

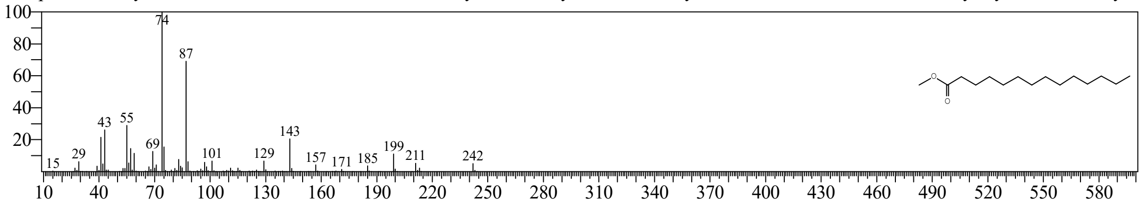


Hit#:3 Entry:27874 Library:NIST17s.lib
SI:91 Formula:C15H30O2 CAS:124-10-7 MolWeight:242 RetIndex:1680
CompName:Methyl tetradecanoate $$ Tetradecanoic acid, methyl ester $$ Myristic acid, methyl ester $$ Metholeneat 2495 $$ Methyl myristate $$ Methyl r

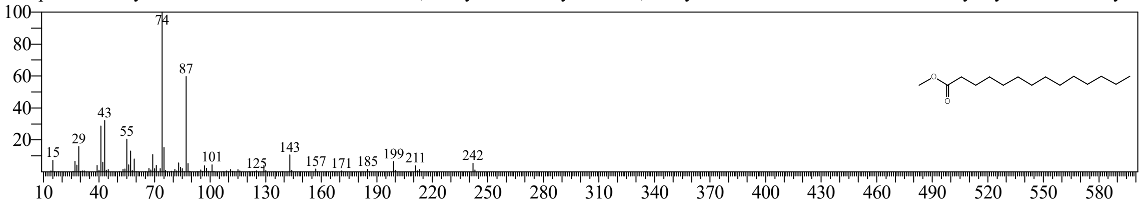


Hit#:4 Entry:23848 Library:NIST14s.lib
SI:91 Formula:C15H30O2 CAS:124-10-7 MolWeight:242 RetIndex:1680
CompName:Methyl tetradecanoate $$ Tetradecanoic acid, methyl ester $$ Myristic acid, methyl ester $$ Metholeneat 2495 $$ Methyl myristate $$ Methyl r

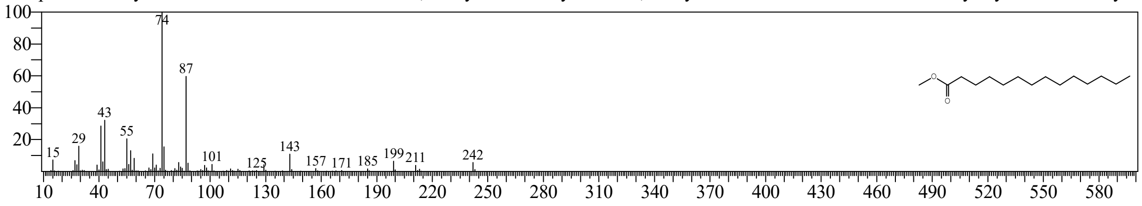


Hit#:5 Entry:80644 Library:NIST14.lib
SI:91 Formula:C15H30O2 CAS:5129-58-8 MolWeight:242 RetIndex:1615
CompName:Tridecanoic acid, 12-methyl-, methyl ester $$ Methyl isomyristate $$ Methyl 12-methyltridecanoate $$

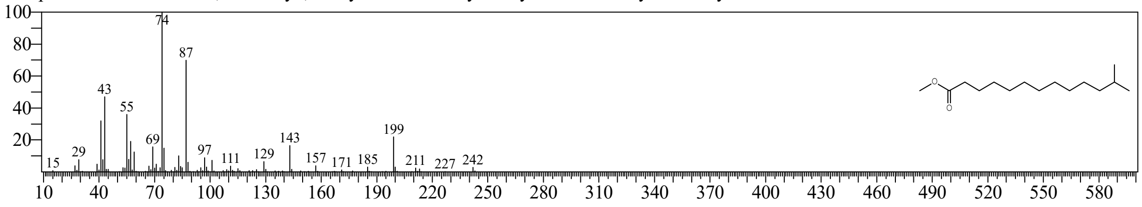

<< Target >>
Line#:3 R.Time:20.808(Scan#:1298) MassPeaks:384
RawMode:Averaged 20.800-20.817(1297-1299) BasePeak:74.00(459231)
BG Mode:Calc. from Peak Group 1 - Event 1

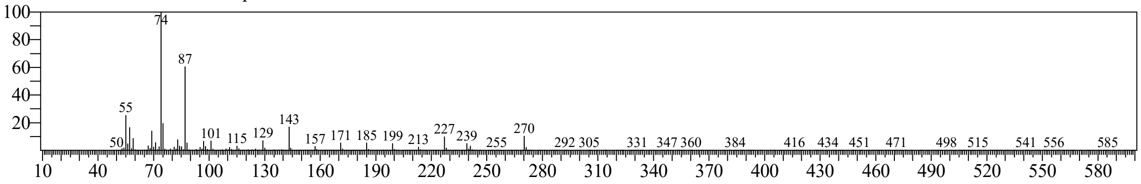


Hit#:1 Entry:113663 Library:NIST17-1.lib
SI:97 Formula:C17H34O2 CAS:112-39-0 MolWeight:270 RetIndex:1878
CompName:Hexadecanoic acid, methyl ester $$ Palmitic acid, methyl ester $$ n-Hexadecanoic acid methyl ester $$ Metholene 2216 $$ Methyl hexadecano

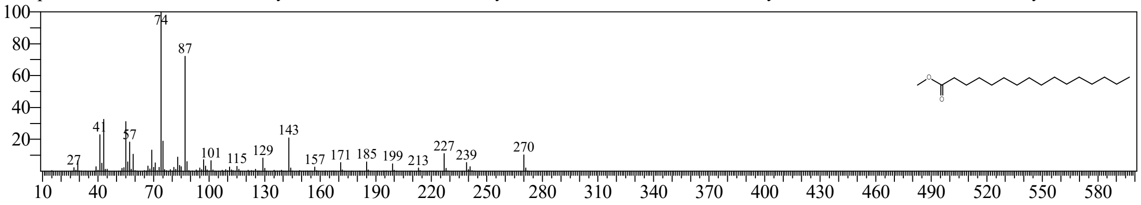


Hit#:2 Entry:104648 Library:NIST14.lib
SI:97 Formula:C17H34O2 CAS:112-39-0 MolWeight:270 RetIndex:1878
CompName:Hexadecanoic acid, methyl ester $$ Palmitic acid, methyl ester $$ n-Hexadecanoic acid methyl ester $$ Metholene 2216 $$ Methyl hexadecano

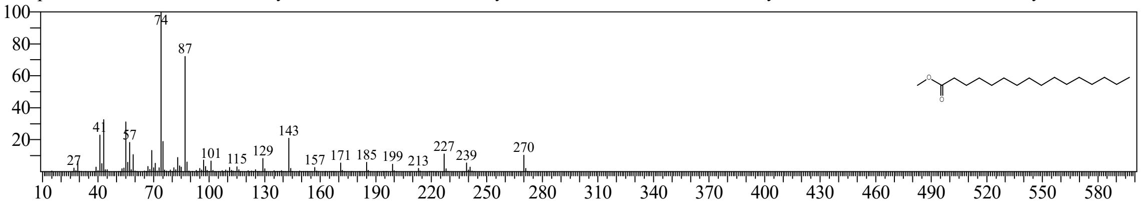


Hit#:3 Entry:26273 Library:NIST14s.lib
SI:97 Formula:C17H34O2 CAS:112-39-0 MolWeight:270 RetIndex:1878
CompName:Hexadecanoic acid, methyl ester $$ Palmitic acid, methyl ester $$ n-Hexadecanoic acid methyl ester $$ Metholene 2216 $$ Methyl hexadecano

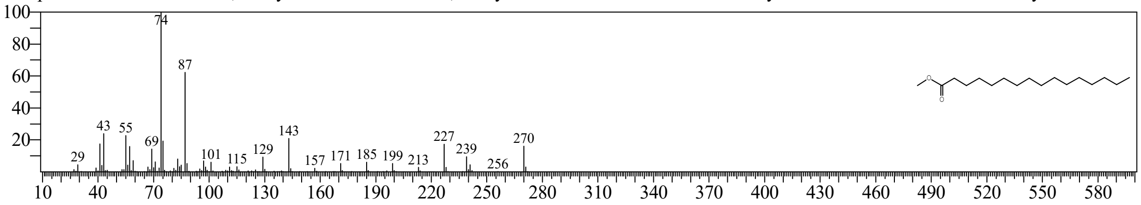


Hit#:4 Entry:26271 Library:NIST14s.lib
SI:96 Formula:C17H34O2 CAS:112-39-0 MolWeight:270 RetIndex:1878
CompName:Hexadecanoic acid, methyl ester $$ Palmitic acid, methyl ester $$ n-Hexadecanoic acid methyl ester $$ Metholene 2216 $$ Methyl hexadecano

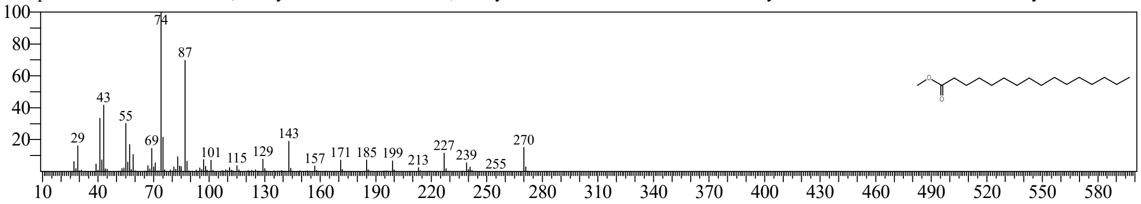


Hit#:5 Entry:30646 Library:NIST17s.lib
SI:96 Formula:C17H34O2 CAS:112-39-0 MolWeight:270 RetIndex:1878
CompName:Hexadecanoic acid, methyl ester $$ Palmitic acid, methyl ester $$ n-Hexadecanoic acid methyl ester $$ Metholene 2216 $$ Methyl hexadecano

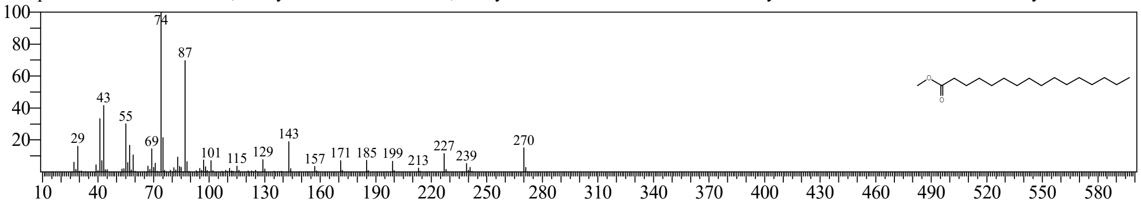

<< Target >>
Line#:4 R.Time:22.167(Scan#:1461) MassPeaks:273
RawMode:Averaged 22.158-22.175(1460-1462) BasePeak:55.05(1388)
BG Mode:Calc. from Peak Group 1 - Event 1

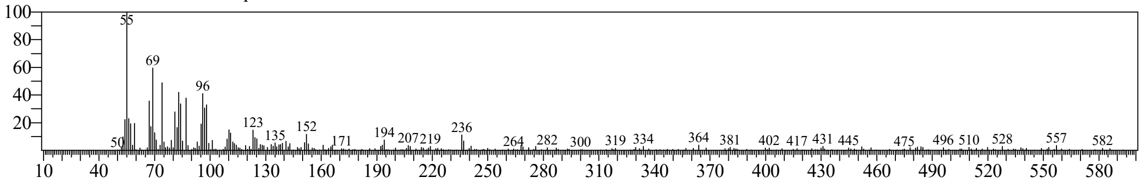


Hit#:1 Entry:30413 Library:NIST17s.lib
SI:93 Formula:C17H32O2 CAS:1120-25-8 MolWeight:268 RetIndex:1886
CompName:9-Hexadecenoic acid, methyl ester, (Z)- $$ Methyl palmitoleate $$ Methyl palmitoleinate $$ Palmitoleic acid, methyl ester $$ Methyl (9Z)-9-he:

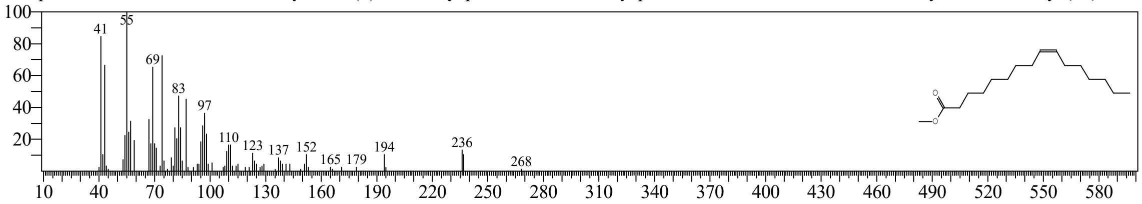


Hit#:2 Entry:26064 Library:NIST14s.lib
SI:93 Formula:C17H32O2 CAS:1120-25-8 MolWeight:268 RetIndex:1886
CompName:9-Hexadecenoic acid, methyl ester, (Z)- $$ Methyl palmitoleate $$ Methyl palmitoleinate $$ Palmitoleic acid, methyl ester $$ Methyl (9Z)-9-he:

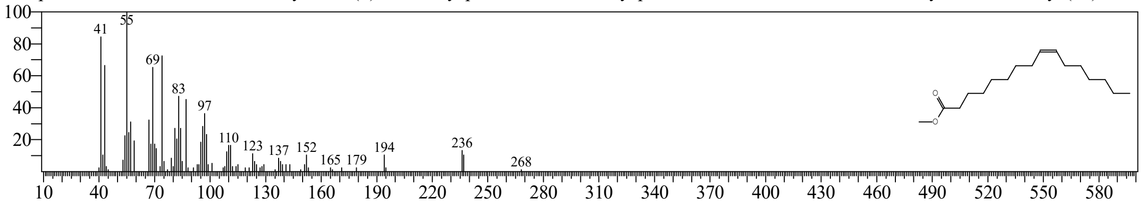


Hit#:3 Entry:26066 Library:NIST14s.lib
SI:93 Formula:C17H32O2 CAS:1120-25-8 MolWeight:268 RetIndex:1886
CompName:9-Hexadecenoic acid, methyl ester, (Z)- $$ Methyl palmitoleate $$ Methyl palmitoleinate $$ Palmitoleic acid, methyl ester $$ Methyl (9Z)-9-he:

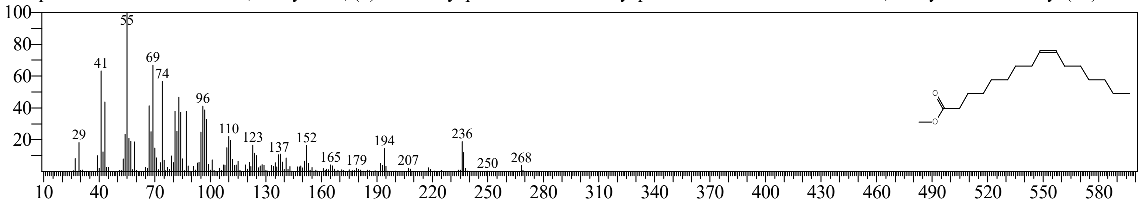


Hit#:4 Entry:30415 Library:NIST17s.lib
SI:93 Formula:C17H32O2 CAS:1120-25-8 MolWeight:268 RetIndex:1886
CompName:9-Hexadecenoic acid, methyl ester, (Z)- $$ Methyl palmitoleate $$ Methyl palmitoleinate $$ Palmitoleic acid, methyl ester $$ Methyl (9Z)-9-he:

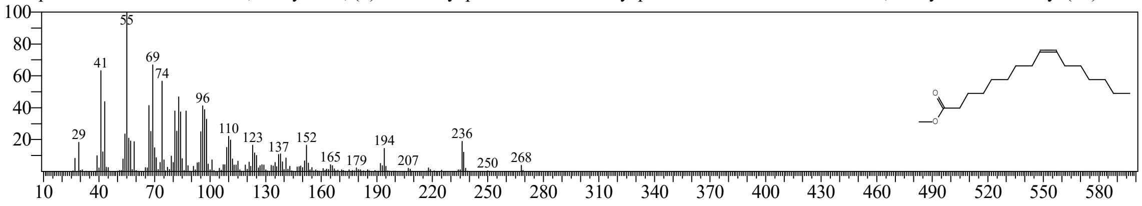


Hit#:5 Entry:111510 Library:NIST17-1.lib
SI:92 Formula:C17H32O2 CAS:56875-67-3 MolWeight:268 RetIndex:1886
CompName:7-Hexadecenoic acid, methyl ester, (Z)- $$ Methyl (7E)-7-hexadecenoate # $$

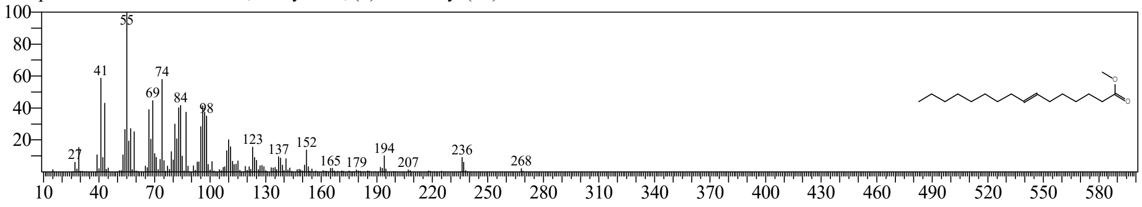

<< Target >>
Line#:5 R.Time:22.783(Scan#:1535) MassPeaks:317
RawMode:Averaged 22.775-22.792(1534-1536) BasePeak:74.05(3357)
BG Mode:Calc. from Peak Group 1 - Event 1

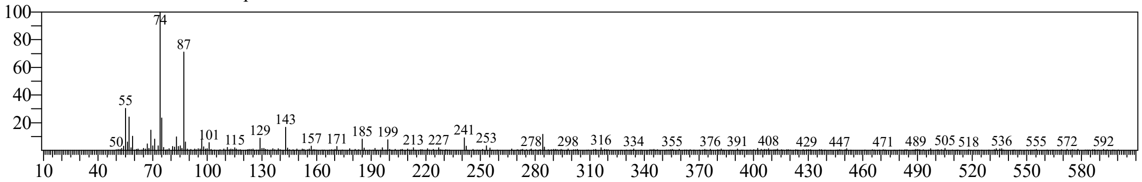

Hit#:1 Entry:27274 Library:NIST14s.lib
SI:93 Formula:C18H36O2 CAS:1731-92-6 MolWeight:284 RetIndex:1978
CompName:Heptadecanoic acid, methyl ester $$ Margaric acid methyl ester $$ Methyl heptadecanoate $$ Methyl margarate $$ n-Heptadecanoic acid methy

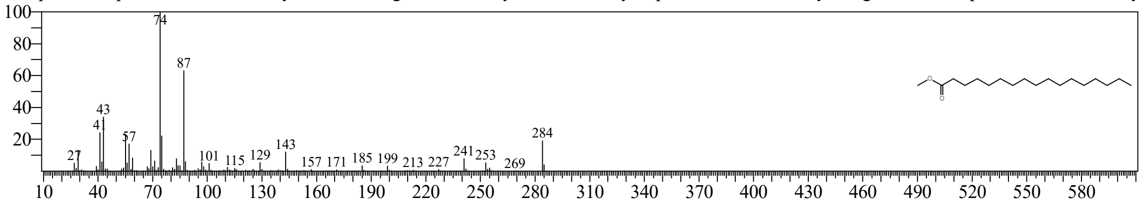

Hit#:2 Entry:31783 Library:NIST17s.lib
SI:93 Formula:C18H36O2 CAS:1731-92-6 MolWeight:284 RetIndex:1978
CompName:Heptadecanoic acid, methyl ester $$ Margaric acid methyl ester $$ Methyl heptadecanoate $$ Methyl margarate $$ n-Heptadecanoic acid methy

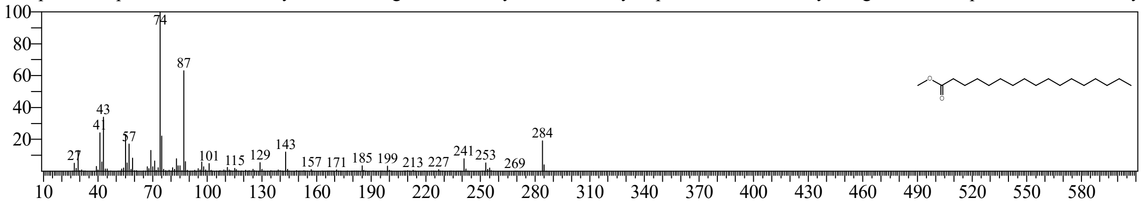

Hit#:3 Entry:27279 Library:NIST14s.lib
SI:93 Formula:C18H36O2 CAS:1731-92-6 MolWeight:284 RetIndex:1978
CompName:Heptadecanoic acid, methyl ester $$ Margaric acid methyl ester $$ Methyl heptadecanoate $$ Methyl margarate $$ n-Heptadecanoic acid methy

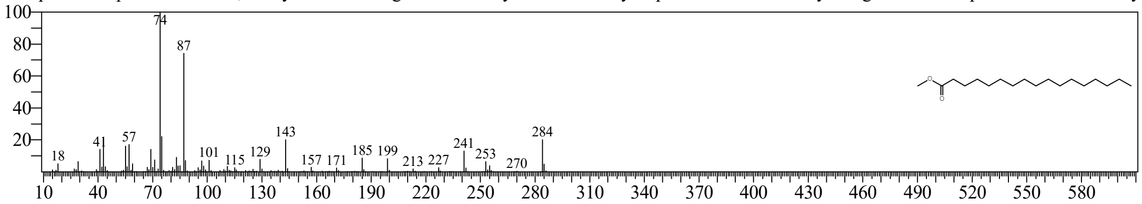

Hit#:4 Entry:31787 Library:NIST17s.lib
SI:93 Formula:C18H36O2 CAS:1731-92-6 MolWeight:284 RetIndex:1978
CompName:Heptadecanoic acid, methyl ester $$ Margaric acid methyl ester $$ Methyl heptadecanoate $$ Methyl margarate $$ n-Heptadecanoic acid methy

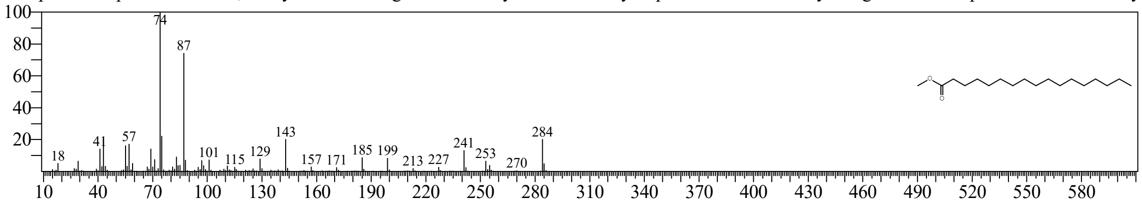

Hit#:5 Entry:31784 Library:NIST17s.lib
SI:92 Formula:C18H36O2 CAS:1731-92-6 MolWeight:284 RetIndex:1978
CompName:Heptadecanoic acid, methyl ester $$ Margaric acid methyl ester $$ Methyl heptadecanoate $$ Methyl margarate $$ n-Heptadecanoic acid methy

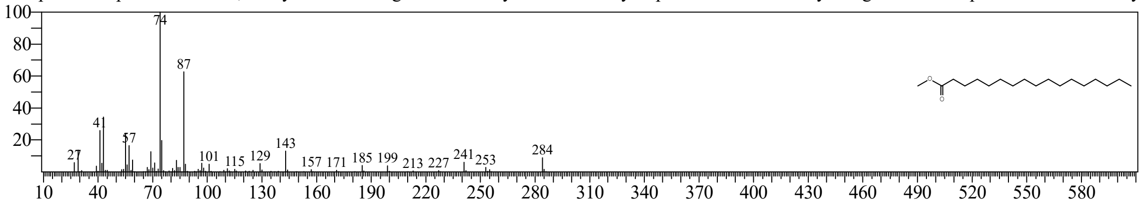

<< Target >>
Line#:6  R.Time:24.092(Scan#:1692)  MassPeaks:332
RawMode:Averaged 24.083-24.100(1691-1693) BasePeak:55.00(738)
BG Mode:Calc. from Peak  Group 1 - Event 1

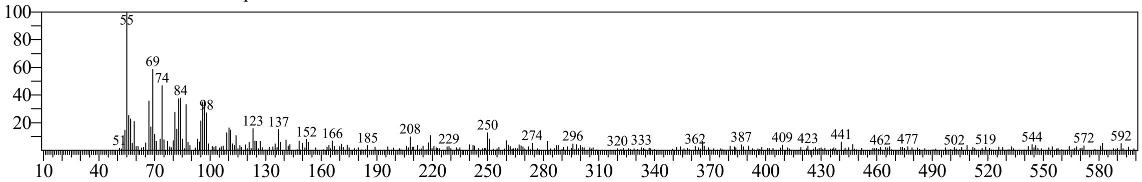


Hit#:1  Entry:125440  Library:NIST17-1.lib
SI:89  Formula:C18H34O2  CAS:0-00-0  MolWeight:282  RetIndex:1986
CompName:Methyl 9-heptadecenoate or 9-17:1

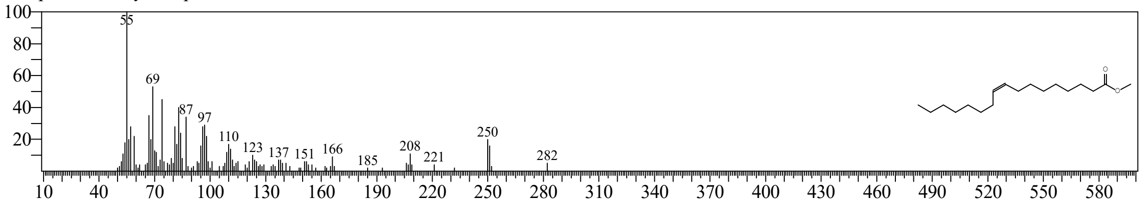


Hit#:2  Entry:115096  Library:NIST14.lib
SI:89  Formula:C18H34O2  CAS:0-00-0  MolWeight:282  RetIndex:1986
CompName:Methyl 9-heptadecenoate or 9-17:1

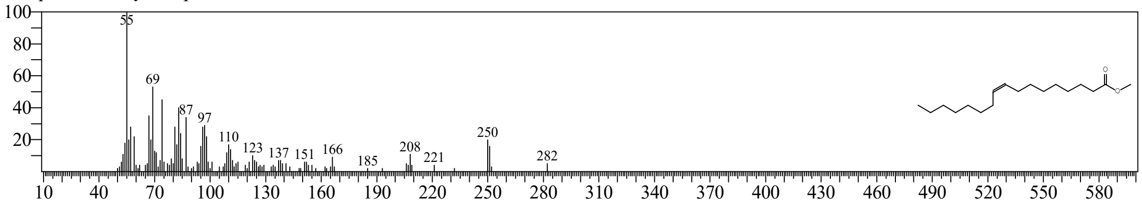


Hit#:3  Entry:31597  Library:NIST17s.lib
SI:88  Formula:C18H34O2  CAS:10152-61-1  MolWeight:282  RetIndex:1941
CompName:Cyclopropaneoctanoic acid, 2-hexyl-, methyl ester $$ Methyl 8-(2-hexylcyclopropyl)octanoate # $$

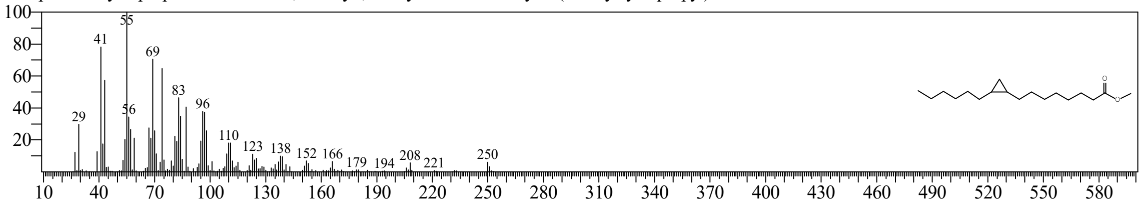


Hit#:4  Entry:27110  Library:NIST14s.lib
SI:88  Formula:C18H34O2  CAS:10152-61-1  MolWeight:282  RetIndex:1941
CompName:Cyclopropaneoctanoic acid, 2-hexyl-, methyl ester $$ Methyl 8-(2-hexylcyclopropyl)octanoate # $$

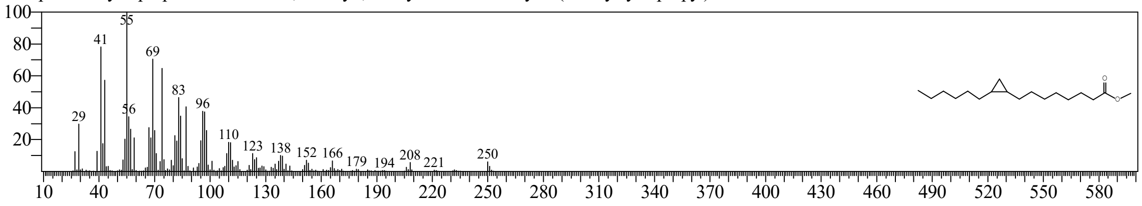


Hit#:5  Entry:115094  Library:NIST14.lib
SI:87  Formula:C18H34O2  CAS:0-00-0  MolWeight:282  RetIndex:1986
CompName:cis-10-Heptadecenoic acid, methyl ester

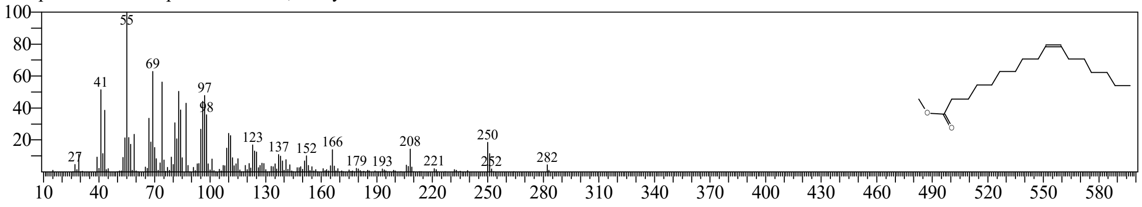

<< Target >>
Line#:7  R.Time:24.875(Scan#:1786)  MassPeaks:324
RawMode:Averaged 24.867-24.883(1785-1787) BasePeak:74.00(137083)
BG Mode:Calc. from Peak  Group 1 - Event 1

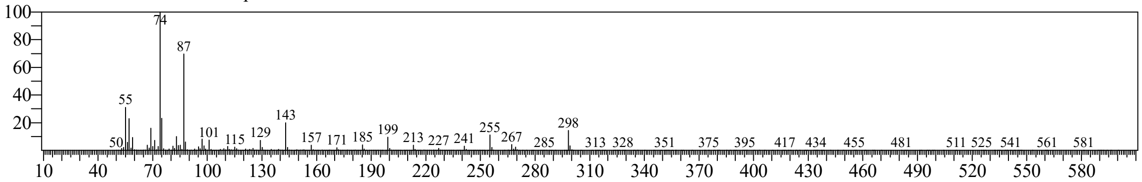


Hit#:1  Entry:129694  Library:NIST14.lib
SI:98  Formula:C19H38O2  CAS:112-61-8  MolWeight:298  RetIndex:2077
CompName:Methyl stearate $$ Octadecanoic acid, methyl ester $$ Stearic acid, methyl ester $$ n-Octadecanoic acid, methyl ester $$ Kemester 9718 $$ Me

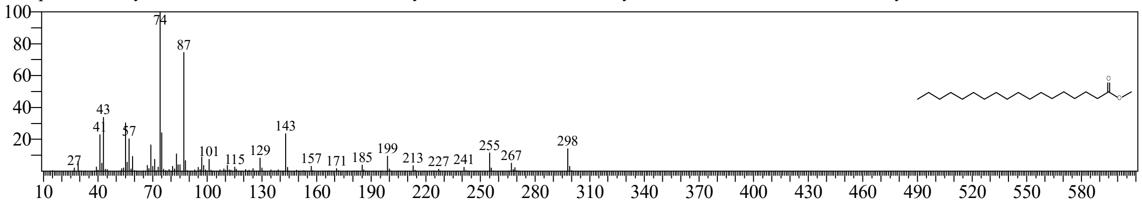


Hit#:2  Entry:141978  Library:NIST17-1.lib
SI:98  Formula:C19H38O2  CAS:112-61-8  MolWeight:298  RetIndex:2077
CompName:Methyl stearate $$ Octadecanoic acid, methyl ester $$ Stearic acid, methyl ester $$ n-Octadecanoic acid, methyl ester $$ Kemester 9718 $$ Me

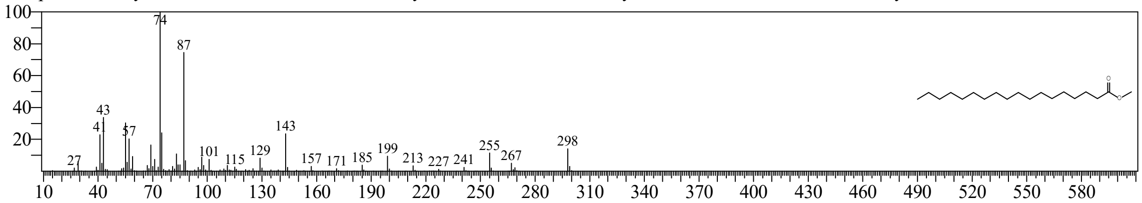


Hit#:3  Entry:28255  Library:NIST14s.lib
SI:97  Formula:C19H38O2  CAS:112-61-8  MolWeight:298  RetIndex:2077
CompName:Methyl stearate $$ Octadecanoic acid, methyl ester $$ Stearic acid, methyl ester $$ n-Octadecanoic acid, methyl ester $$ Kemester 9718 $$ Me

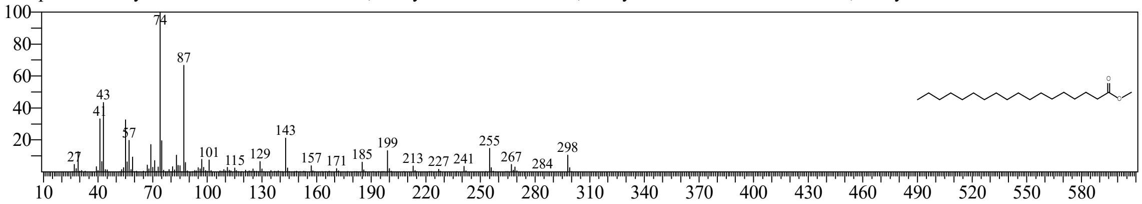


Hit#:4  Entry:32873  Library:NIST17s.lib
SI:97  Formula:C19H38O2  CAS:112-61-8  MolWeight:298  RetIndex:2077
CompName:Methyl stearate $$ Octadecanoic acid, methyl ester $$ Stearic acid, methyl ester $$ n-Octadecanoic acid, methyl ester $$ Kemester 9718 $$ Me

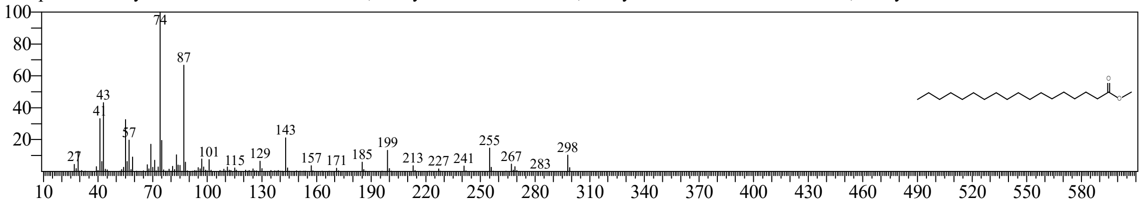


Hit#:5  Entry:28257  Library:NIST14s.lib
SI:96  Formula:C19H38O2  CAS:112-61-8  MolWeight:298  RetIndex:2077
CompName:Methyl stearate $$ Octadecanoic acid, methyl ester $$ Stearic acid, methyl ester $$ n-Octadecanoic acid, methyl ester $$ Kemester 9718 $$ Me

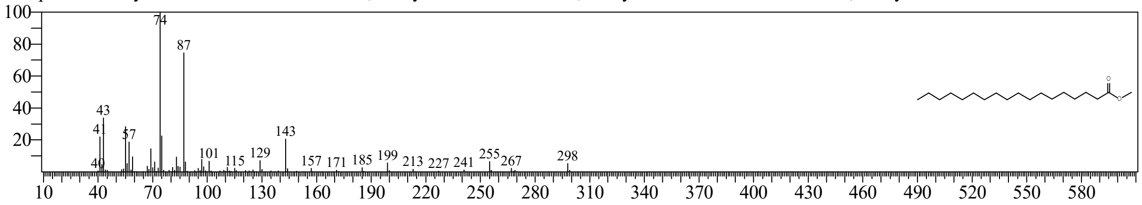

<< Target >>
Line#:8 R.Time:26.258(Scan#:1952) MassPeaks:427
RawMode:Averaged 26.250-26.267(1951-1953) BasePeak:55.05(249625)
BG Mode:Calc. from Peak Group 1 - Event 1

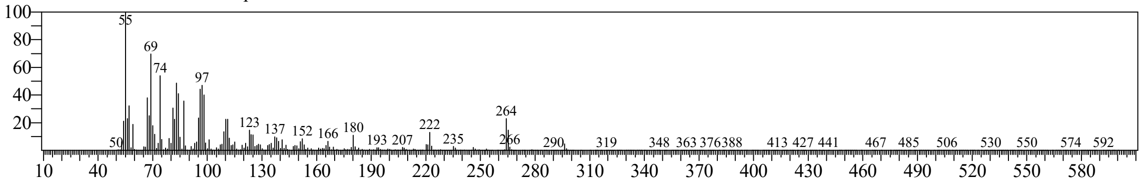


Hit#:1 Entry:32747 Library:NIST17s.lib
SI:97 Formula:C19H36O2 CAS:1937-62-8 MolWeight:296 RetIndex:2085
CompName:9-Octadecenoic acid, methyl ester, (E)- $$ Elaidic acid, methyl ester $$ Methyl elaidate $$ Methyl trans-9-octadecenoate $$ (E)-9-Octadecenoic

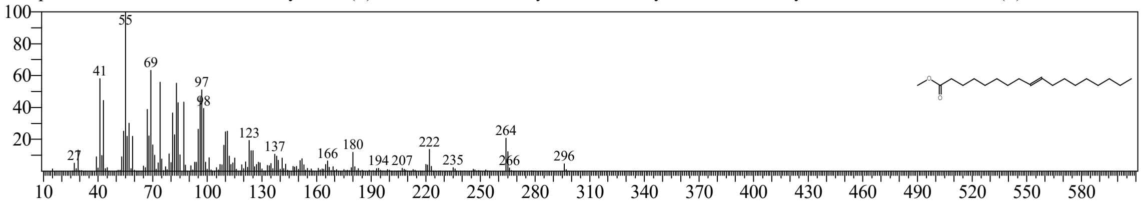


Hit#:2 Entry:28138 Library:NIST14s.lib
SI:97 Formula:C19H36O2 CAS:1937-62-8 MolWeight:296 RetIndex:2085
CompName:9-Octadecenoic acid, methyl ester, (E)- $$ Elaidic acid, methyl ester $$ Methyl elaidate $$ Methyl trans-9-octadecenoate $$ (E)-9-Octadecenoic

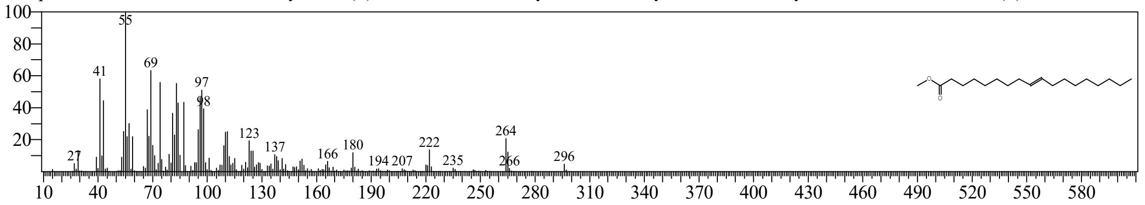


Hit#:3 Entry:28139 Library:NIST14s.lib
SI:96 Formula:C19H36O2 CAS:52380-33-3 MolWeight:296 RetIndex:2085
CompName:11-Octadecenoic acid, methyl ester $$ Methyl 11-octadecenoate $$ Octadec-11-enoic acid, methyl ester $$ Methyl (11E)-11-octadecenoate # $$

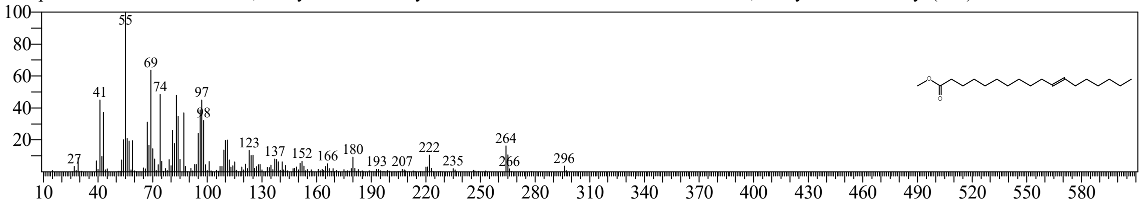


Hit#:4 Entry:127664 Library:NIST14.lib
SI:96 Formula:C19H36O2 CAS:112-62-9 MolWeight:296 RetIndex:2085
CompName:9-Octadecenoic acid (Z)-, methyl ester $$ Oleic acid, methyl ester $$ Emery oleic acid ester 2301 $$ Methyl cis-9-octadecenoate $$ Methyl ole

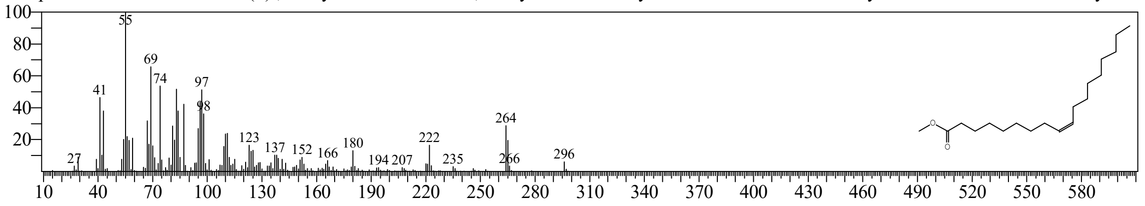


Hit#:5 Entry:139650 Library:NIST17-1.lib
SI:96 Formula:C19H36O2 CAS:112-62-9 MolWeight:296 RetIndex:2085
CompName:9-Octadecenoic acid (Z)-, methyl ester $$ Oleic acid, methyl ester $$ Emery oleic acid ester 2301 $$ Methyl cis-9-octadecenoate $$ Methyl ole

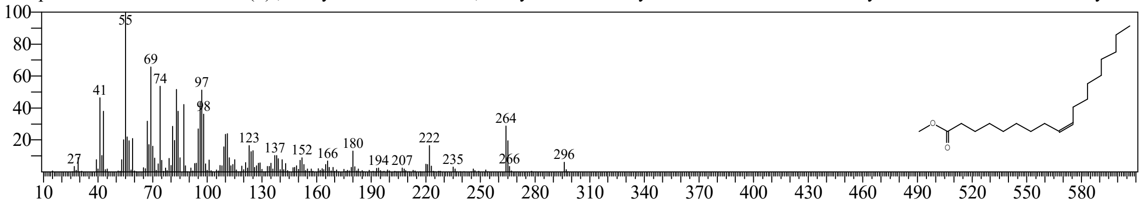

<< Target >>
Line#:9  R.Time:26.383(Scan#:1967)  MassPeaks:364
RawMode:Averaged 26.375-26.392(1966-1968) BasePeak:55.05(17146)
BG Mode:Calc. from Peak  Group 1 - Event 1

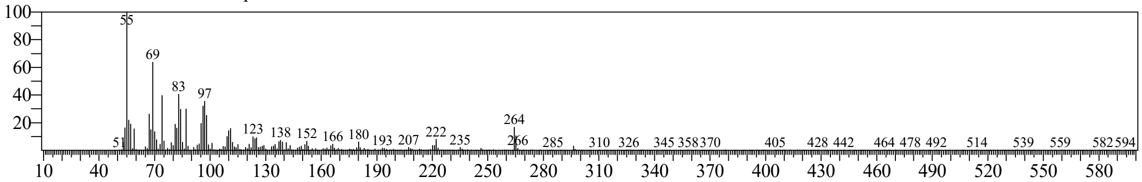


Hit#:1  Entry:32748  Library:NIST17s.lib
SI:95  Formula:C19H36O2  CAS:52380-33-3  MolWeight:296  RetIndex:2085
CompName:11-Octadecenoic acid, methyl ester $$ Methyl 11-octadecenoate $$ Octadec-11-enoic acid, methyl ester $$ Methyl (11E)-11-octadecenoate # $$

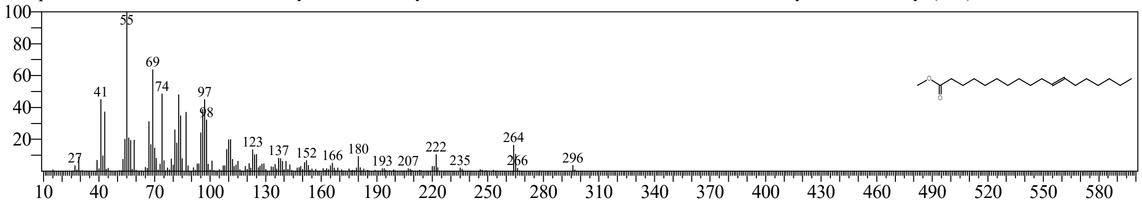


Hit#:2  Entry:28139  Library:NIST14s.lib
SI:95  Formula:C19H36O2  CAS:52380-33-3  MolWeight:296  RetIndex:2085
CompName:11-Octadecenoic acid, methyl ester $$ Methyl 11-octadecenoate $$ Octadec-11-enoic acid, methyl ester $$ Methyl (11E)-11-octadecenoate # $$

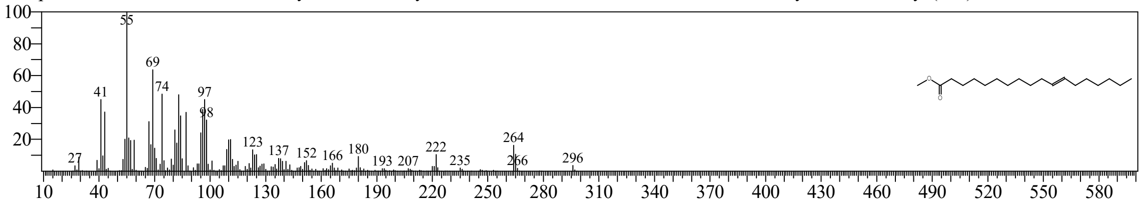


Hit#:3  Entry:127650  Library:NIST14.lib
SI:95  Formula:C19H36O2  CAS:13481-95-3  MolWeight:296  RetIndex:2085
CompName:10-Octadecenoic acid, methyl ester $$ Methyl 10-octadecenoate $$

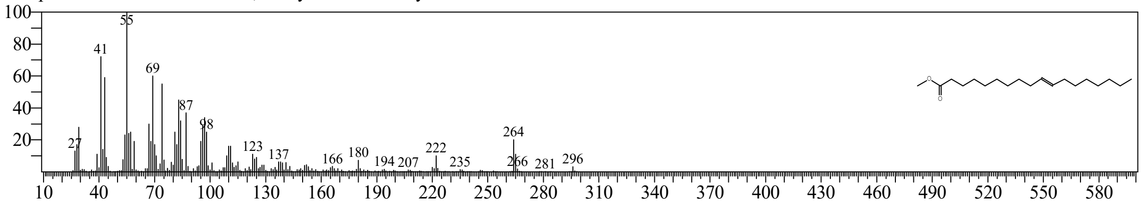


Hit#:4  Entry:139647  Library:NIST17-1.lib
SI:95  Formula:C19H36O2  CAS:0-00-0  MolWeight:296  RetIndex:2085
CompName:cis-13-Octadecenoic acid, methyl ester

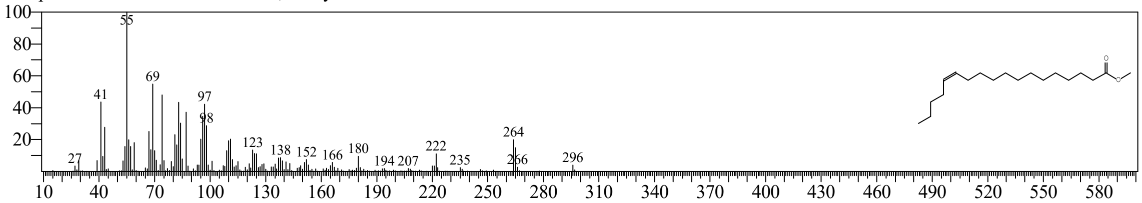


Hit#:5  Entry:127661  Library:NIST14.lib
SI:95  Formula:C19H36O2  CAS:0-00-0  MolWeight:296  RetIndex:2085
CompName:cis-13-Octadecenoic acid, methyl ester

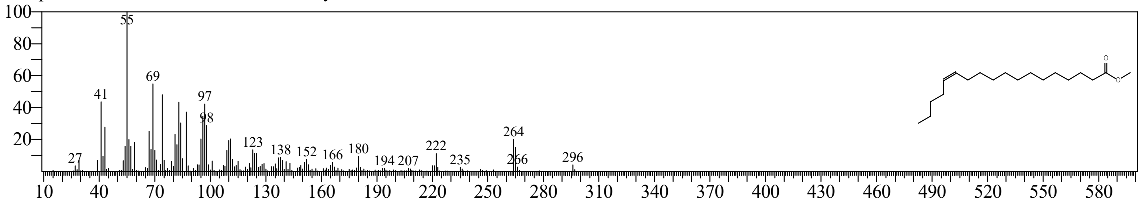

<< Target >>
Line#:10   R.Time:27.742(Scan#:2130)   MassPeaks:360
RawMode:Averaged 27.733-27.750(2129-2131) BasePeak:67.05(3373)
BG Mode:Calc. from Peak  Group 1 - Event 1

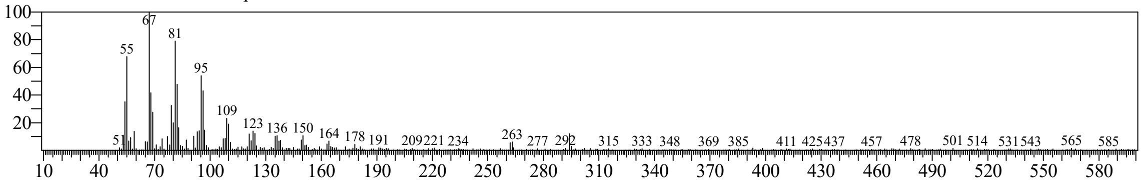


Hit#:1  Entry:27999  Library:NIST14s.lib
SI:96  Formula:C19H34O2  CAS:112-63-0  MolWeight:294  RetIndex:2093
CompName:9,12-Octadecadienoic acid (Z,Z)-, methyl ester $$ Linoleic acid, methyl ester $$ Methyl cis,cis-9,12-octadecadienoate $$ Methyl linoleate $$ M

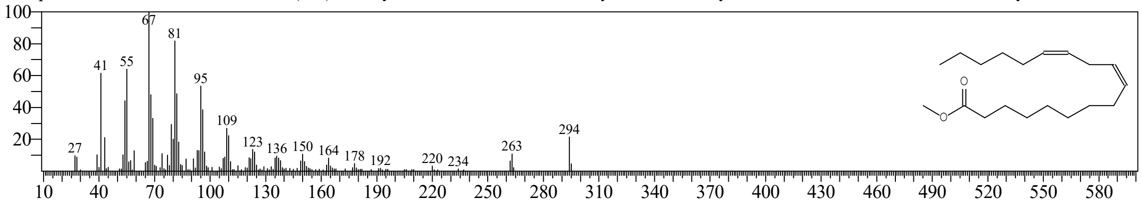


Hit#:2  Entry:32588  Library:NIST17s.lib
SI:96  Formula:C19H34O2  CAS:112-63-0  MolWeight:294  RetIndex:2093
CompName:9,12-Octadecadienoic acid (Z,Z)-, methyl ester $$ Linoleic acid, methyl ester $$ Methyl cis,cis-9,12-octadecadienoate $$ Methyl linoleate $$ M

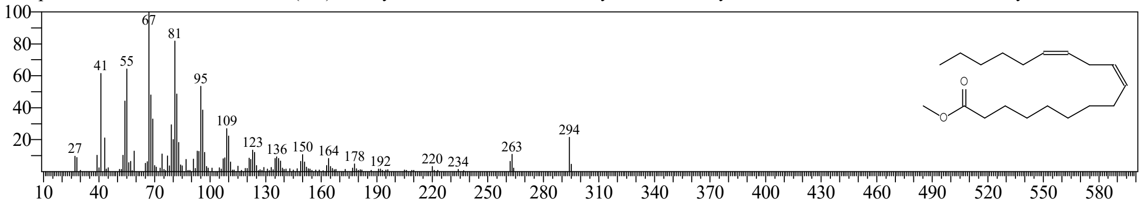


Hit#:3  Entry:32589  Library:NIST17s.lib
SI:96  Formula:C19H34O2  CAS:112-63-0  MolWeight:294  RetIndex:2093
CompName:9,12-Octadecadienoic acid (Z,Z)-, methyl ester $$ Linoleic acid, methyl ester $$ Methyl cis,cis-9,12-octadecadienoate $$ Methyl linoleate $$ M

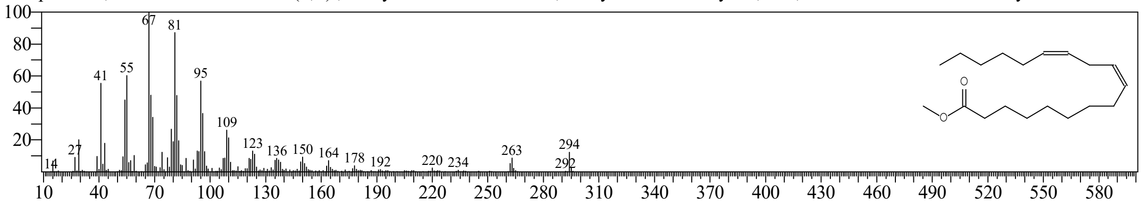


Hit#:4  Entry:28000  Library:NIST14s.lib
SI:96  Formula:C19H34O2  CAS:112-63-0  MolWeight:294  RetIndex:2093
CompName:9,12-Octadecadienoic acid (Z,Z)-, methyl ester $$ Linoleic acid, methyl ester $$ Methyl cis,cis-9,12-octadecadienoate $$ Methyl linoleate $$ M

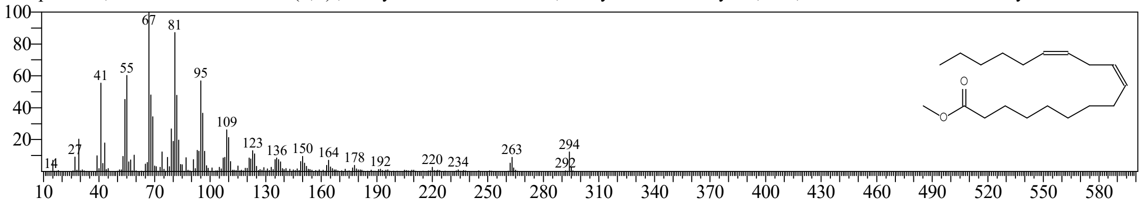


Hit#:5  Entry:137663  Library:NIST17-1.lib
SI:96  Formula:C19H34O2  CAS:2462-85-3  MolWeight:294  RetIndex:2093
CompName:9,12-Octadecadienoic acid, methyl ester $$ 9,12-Octadecenoic acid, methyl ester $$ Methyl octadeca-9,12-dienoate $$ Methyl 9,12-octadecadi

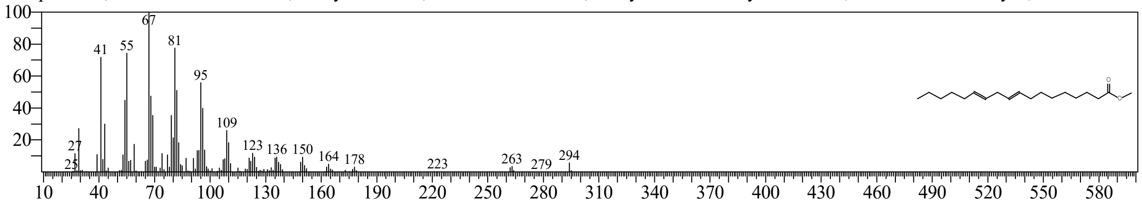

<< Target >>
Line#:11 R.Time:27.942(Scan#:2154) MassPeaks:363
RawMode:Averaged 27.933-27.950(2153-2155) BasePeak:67.05(2357)
BG Mode:Calc. from Peak Group 1 - Event 1

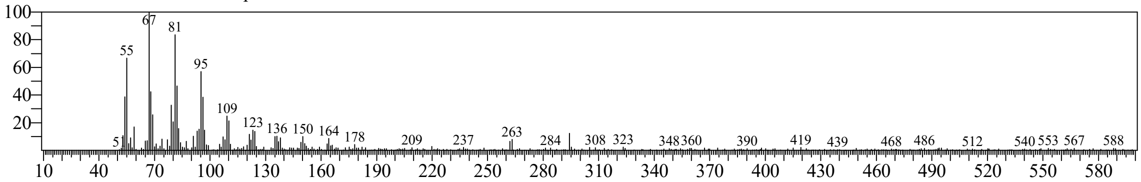


Hit#:1 Entry:27999 Library:NIST14s.lib
SI:96 Formula:C19H34O2 CAS:112-63-0 MolWeight:294 RetIndex:2093
CompName:9,12-Octadecadienoic acid (Z,Z)-, methyl ester $$ Linoleic acid, methyl ester $$ Methyl cis,cis-9,12-octadecadienoate $$ Methyl linoleate $$ M

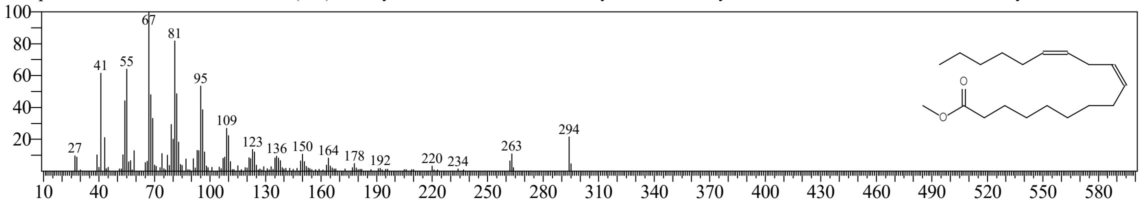


Hit#:2 Entry:32588 Library:NIST17s.lib
SI:96 Formula:C19H34O2 CAS:112-63-0 MolWeight:294 RetIndex:2093
CompName:9,12-Octadecadienoic acid (Z,Z)-, methyl ester $$ Linoleic acid, methyl ester $$ Methyl cis,cis-9,12-octadecadienoate $$ Methyl linoleate $$ M

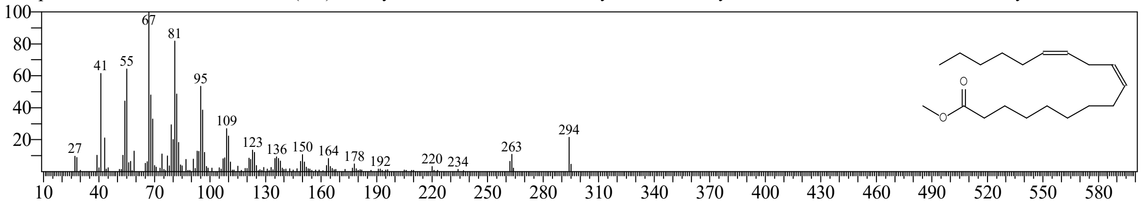


Hit#:3 Entry:32589 Library:NIST17s.lib
SI:96 Formula:C19H34O2 CAS:112-63-0 MolWeight:294 RetIndex:2093
CompName:9,12-Octadecadienoic acid (Z,Z)-, methyl ester $$ Linoleic acid, methyl ester $$ Methyl cis,cis-9,12-octadecadienoate $$ Methyl linoleate $$ M

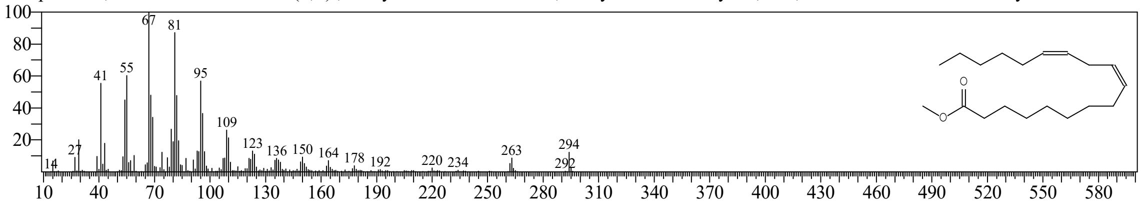


Hit#:4 Entry:28000 Library:NIST14s.lib
SI:96 Formula:C19H34O2 CAS:112-63-0 MolWeight:294 RetIndex:2093
CompName:9,12-Octadecadienoic acid (Z,Z)-, methyl ester $$ Linoleic acid, methyl ester $$ Methyl cis,cis-9,12-octadecadienoate $$ Methyl linoleate $$ M

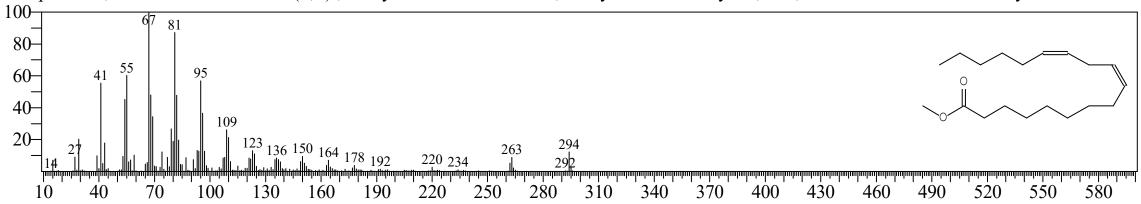


Hit#:5 Entry:137663 Library:NIST17-1.lib
SI:96 Formula:C19H34O2 CAS:2462-85-3 MolWeight:294 RetIndex:2093
CompName:9,12-Octadecadienoic acid, methyl ester $$ 9,12-Octadecenoic acid, methyl ester $$ Methyl octadeca-9,12-dienoate $$ Methyl 9,12-octadecadi

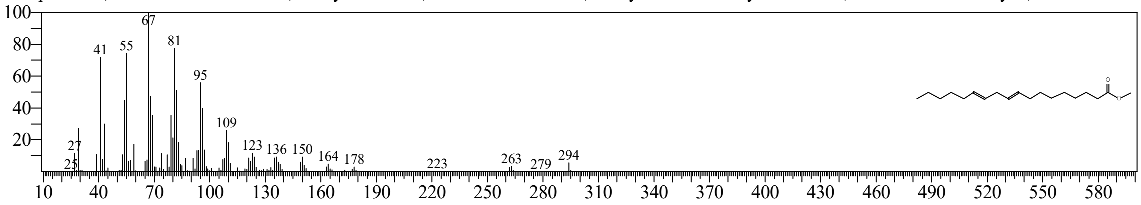

<< Target >>
Line#:12   R.Time:28.367(Scan#:2205)  MassPeaks:420
RawMode:Averaged 28.358-28.375(2204-2206) BasePeak:67.05(472049)
BG Mode:Calc. from Peak  Group 1 - Event 1

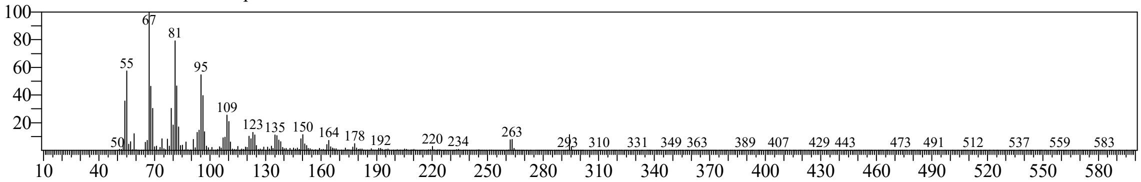


Hit#:1  Entry:32588  Library:NIST17s.lib
SI:97  Formula:C19H34O2  CAS:112-63-0  MolWeight:294  RetIndex:2093
CompName:9,12-Octadecadienoic acid (Z,Z)-, methyl ester $$ Linoleic acid, methyl ester $$ Methyl cis,cis-9,12-octadecadienoate $$ Methyl linoleate $$ M

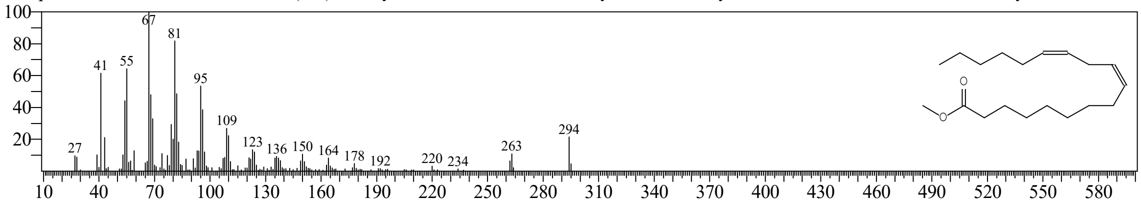


Hit#:2  Entry:27999  Library:NIST14s.lib
SI:97  Formula:C19H34O2  CAS:112-63-0  MolWeight:294  RetIndex:2093
CompName:9,12-Octadecadienoic acid (Z,Z)-, methyl ester $$ Linoleic acid, methyl ester $$ Methyl cis,cis-9,12-octadecadienoate $$ Methyl linoleate $$ M

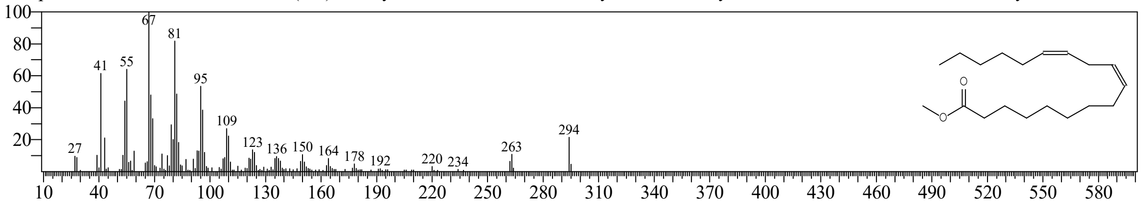


Hit#:3  Entry:32589  Library:NIST17s.lib
SI:97  Formula:C19H34O2  CAS:112-63-0  MolWeight:294  RetIndex:2093
CompName:9,12-Octadecadienoic acid (Z,Z)-, methyl ester $$ Linoleic acid, methyl ester $$ Methyl cis,cis-9,12-octadecadienoate $$ Methyl linoleate $$ M

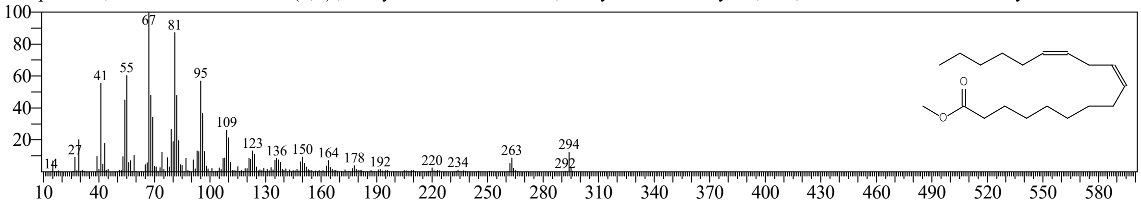


Hit#:4  Entry:28000  Library:NIST14s.lib
SI:97  Formula:C19H34O2  CAS:112-63-0  MolWeight:294  RetIndex:2093
CompName:9,12-Octadecadienoic acid (Z,Z)-, methyl ester $$ Linoleic acid, methyl ester $$ Methyl cis,cis-9,12-octadecadienoate $$ Methyl linoleate $$ M

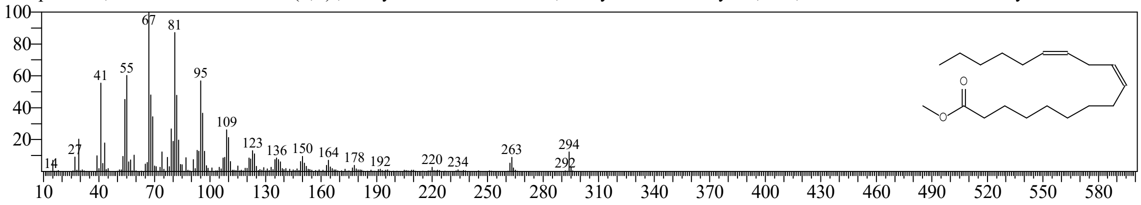


Hit#:5  Entry:137664  Library:NIST17-1.lib
SI:95  Formula:C19H34O2  CAS:0-00-0  MolWeight:294  RetIndex:2093
CompName:Methyl 10-trans,12-cis-octadecadienoate

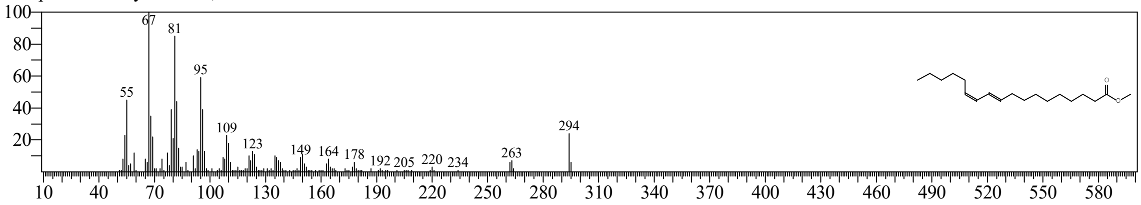

<< Target >>
Line#:13 R.Time:29.267(Scan#:2313) MassPeaks:308
RawMode:Averaged 29.258-29.275(2312-2314) BasePeak:74.00(13715)
BG Mode:Calc. from Peak Group 1 - Event 1

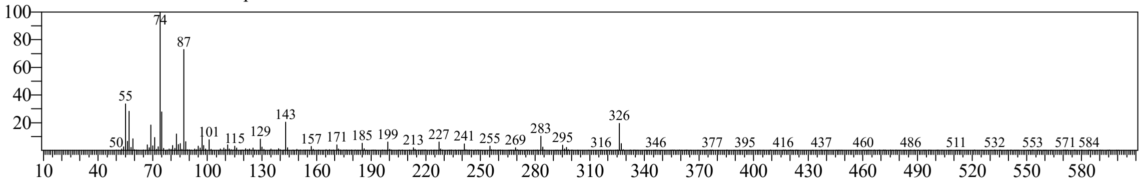


Hit#:1 Entry:154704 Library:NIST14.lib
SI:97 Formula:C21H42O2 CAS:0-00-0 MolWeight:326 RetIndex:2212
CompName:Methyl 18-methylnonadecanoate

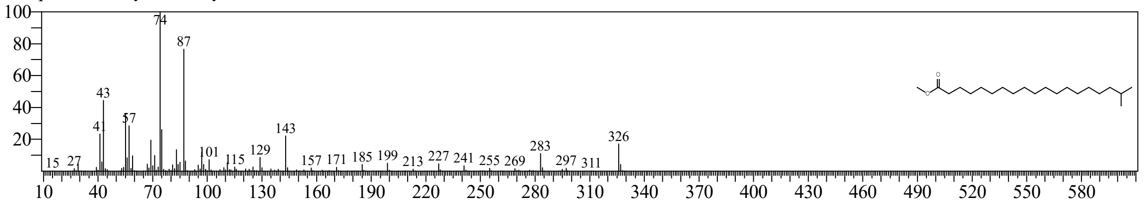


Hit#:2 Entry:170238 Library:NIST17-1.lib
SI:97 Formula:C21H42O2 CAS:0-00-0 MolWeight:326 RetIndex:2212
CompName:Methyl 18-methylnonadecanoate

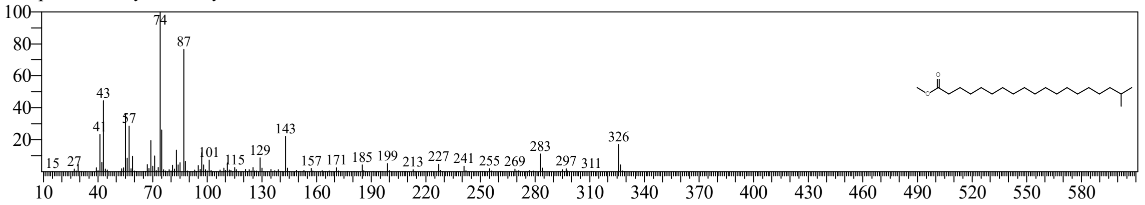


Hit#:3 Entry:34726 Library:NIST17s.lib
SI:96 Formula:C21H42O2 CAS:1120-28-1 MolWeight:326 RetIndex:2276
CompName:Eicosanoic acid, methyl ester $$ Methyl arachisate $$ Methyl eicosanoate $$ Arachidic acid methyl ester $$ Kemester 2050 $$ Methyl aracidat

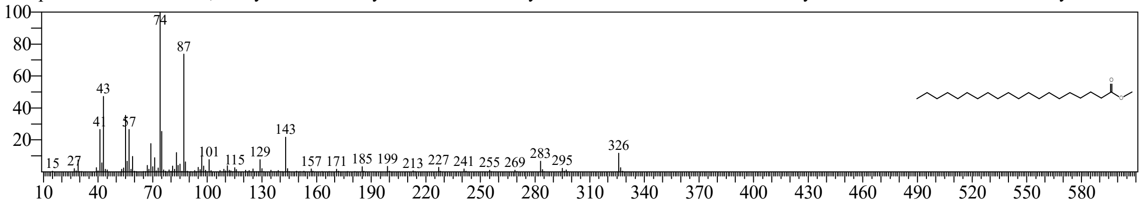


Hit#:4 Entry:34724 Library:NIST17s.lib
SI:93 Formula:C21H42O2 CAS:1120-28-1 MolWeight:326 RetIndex:2276
CompName:Eicosanoic acid, methyl ester $$ Methyl arachisate $$ Methyl eicosanoate $$ Arachidic acid methyl ester $$ Kemester 2050 $$ Methyl aracidat

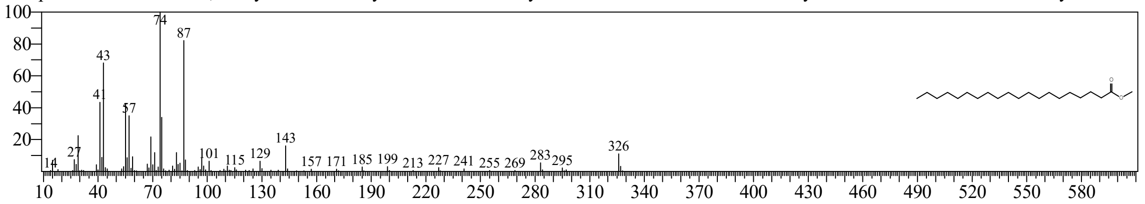


Hit#:5 Entry:29931 Library:NIST14s.lib
SI:93 Formula:C21H42O2 CAS:1120-28-1 MolWeight:326 RetIndex:2276
CompName:Eicosanoic acid, methyl ester $$ Methyl arachisate $$ Methyl eicosanoate $$ Arachidic acid methyl ester $$ Kemester 2050 $$ Methyl aracidat

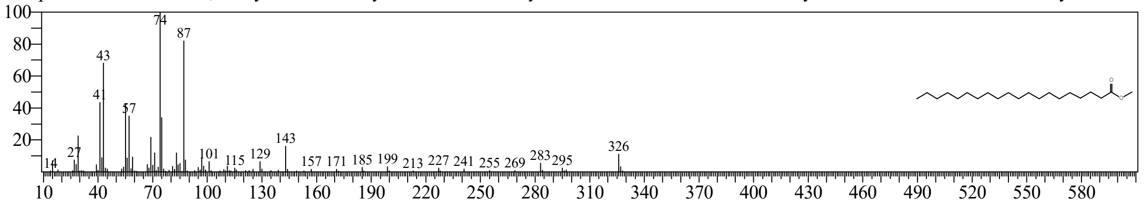

<< Target >>
Line#:14 R.Time:29.825(Scan#:2380) MassPeaks:336
RawMode:Averaged 29.817-29.833(2379-2381) BasePeak:79.05(7947)
BG Mode:Calc. from Peak Group 1 - Event 1

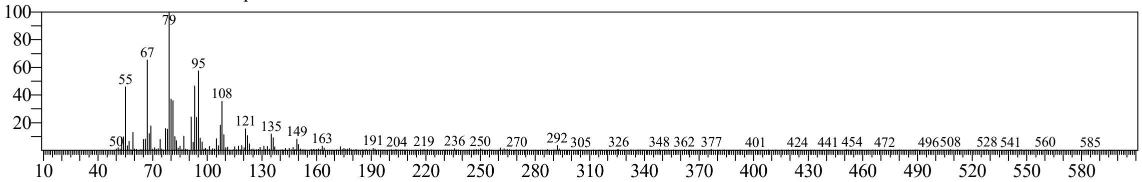


Hit#:1 Entry:135724 Library:NIST17-1.lib
SI:95 Formula:C19H32O2 CAS:301-00-8 MolWeight:292 RetIndex:2101
CompName:9,12,15-Octadecatrienoic acid, methyl ester, (Z,Z,Z)- $$ Linolenic acid, methyl ester $$ Methyl all-cis-9,12,15-octadecatrienoate $$ Methyl lino

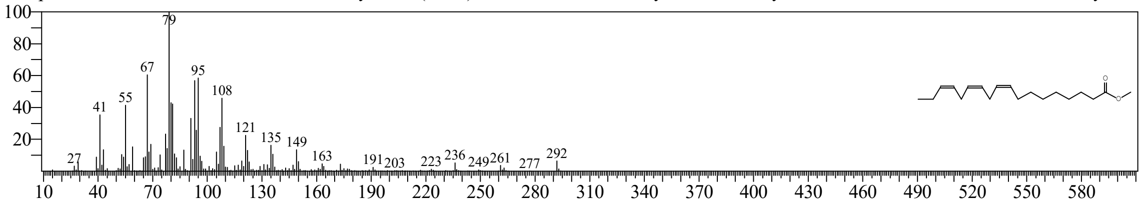


Hit#:2 Entry:124228 Library:NIST14.lib
SI:95 Formula:C19H32O2 CAS:301-00-8 MolWeight:292 RetIndex:2101
CompName:9,12,15-Octadecatrienoic acid, methyl ester, (Z,Z,Z)- $$ Linolenic acid, methyl ester $$ Methyl all-cis-9,12,15-octadecatrienoate $$ Methyl lino

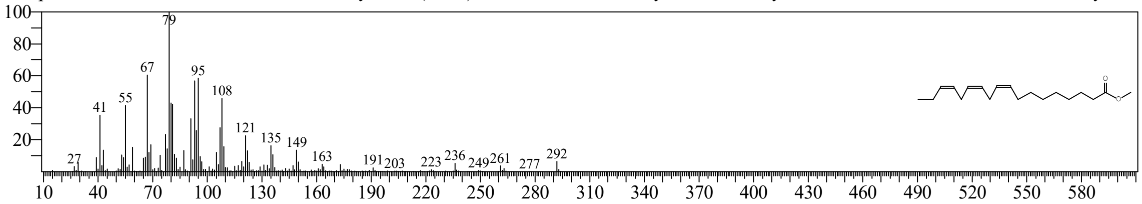


Hit#:3 Entry:32438 Library:NIST17s.lib
SI:95 Formula:C19H32O2 CAS:301-00-8 MolWeight:292 RetIndex:2101
CompName:9,12,15-Octadecatrienoic acid, methyl ester, (Z,Z,Z)- $$ Linolenic acid, methyl ester $$ Methyl all-cis-9,12,15-octadecatrienoate $$ Methyl lino

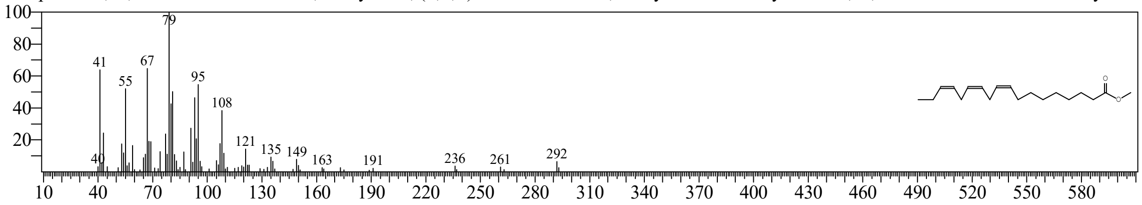


Hit#:4 Entry:27872 Library:NIST14s.lib
SI:94 Formula:C19H32O2 CAS:301-00-8 MolWeight:292 RetIndex:2101
CompName:9,12,15-Octadecatrienoic acid, methyl ester, (Z,Z,Z)- $$ Linolenic acid, methyl ester $$ Methyl all-cis-9,12,15-octadecatrienoate $$ Methyl lino

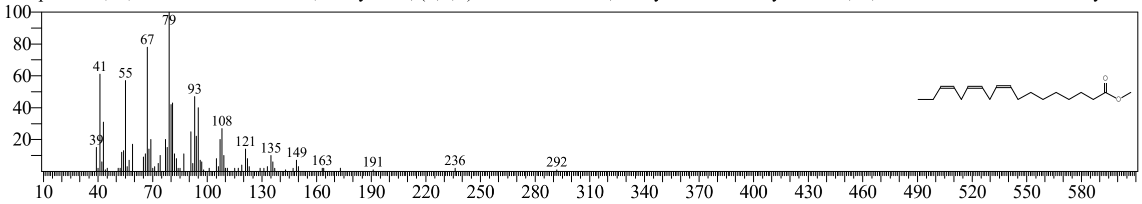


Hit#:5 Entry:32437 Library:NIST17s.lib
SI:94 Formula:C19H32O2 CAS:301-00-8 MolWeight:292 RetIndex:2101
CompName:9,12,15-Octadecatrienoic acid, methyl ester, (Z,Z,Z)- $$ Linolenic acid, methyl ester $$ Methyl all-cis-9,12,15-octadecatrienoate $$ Methyl lino

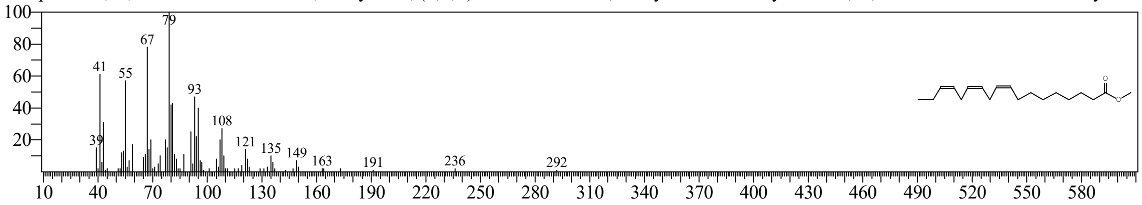

<< Target >>
Line#:15 R.Time:30.300(Scan#:2437) MassPeaks:325
RawMode:Averaged 30.292-30.308(2436-2438) BasePeak:79.05(5822)
BG Mode:Calc. from Peak Group 1 - Event 1

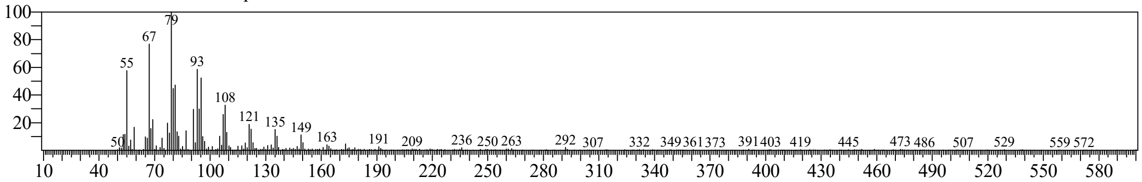


Hit#:1 Entry:135724 Library:NIST17-1.lib
SI:95 Formula:C19H32O2 CAS:301-00-8 MolWeight:292 RetIndex:2101
CompName:9,12,15-Octadecatrienoic acid, methyl ester, (Z,Z,Z)- $$ Linolenic acid, methyl ester $$ Methyl all-cis-9,12,15-octadecatrienoate $$ Methyl lino

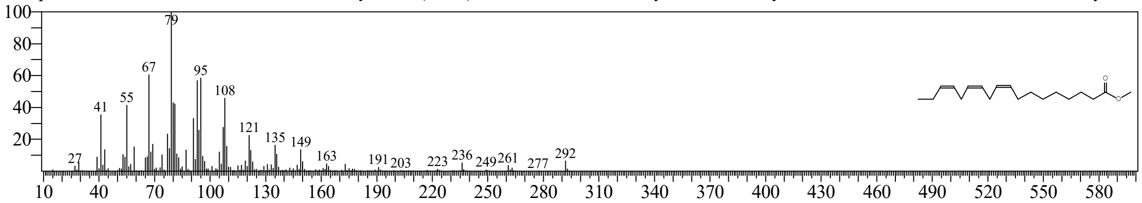


Hit#:2 Entry:124228 Library:NIST14.lib
SI:95 Formula:C19H32O2 CAS:301-00-8 MolWeight:292 RetIndex:2101
CompName:9,12,15-Octadecatrienoic acid, methyl ester, (Z,Z,Z)- $$ Linolenic acid, methyl ester $$ Methyl all-cis-9,12,15-octadecatrienoate $$ Methyl lino

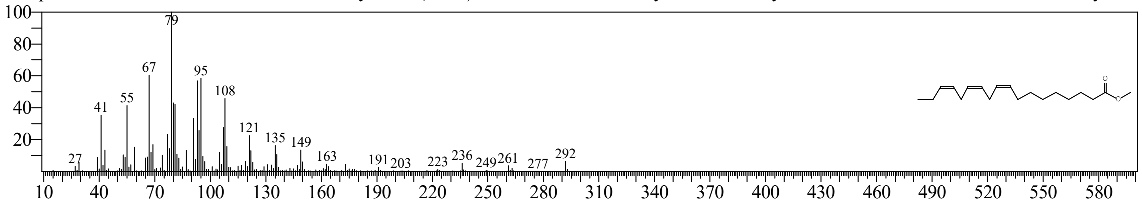


Hit#:3 Entry:27872 Library:NIST14s.lib
SI:95 Formula:C19H32O2 CAS:301-00-8 MolWeight:292 RetIndex:2101
CompName:9,12,15-Octadecatrienoic acid, methyl ester, (Z,Z,Z)- $$ Linolenic acid, methyl ester $$ Methyl all-cis-9,12,15-octadecatrienoate $$ Methyl lino

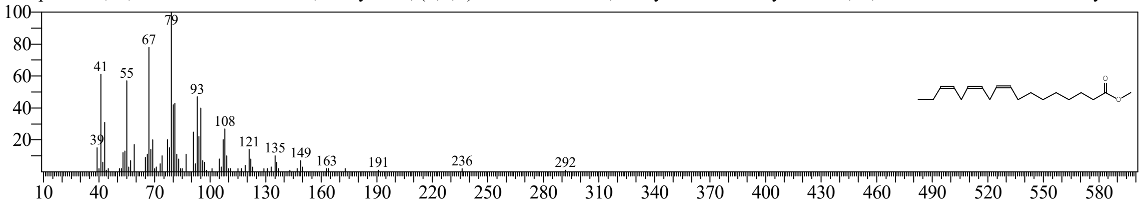


Hit#:4 Entry:32437 Library:NIST17s.lib
SI:95 Formula:C19H32O2 CAS:301-00-8 MolWeight:292 RetIndex:2101
CompName:9,12,15-Octadecatrienoic acid, methyl ester, (Z,Z,Z)- $$ Linolenic acid, methyl ester $$ Methyl all-cis-9,12,15-octadecatrienoate $$ Methyl lino

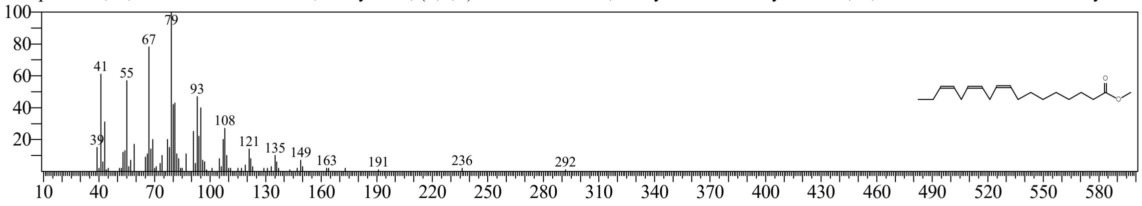


Hit#:5 Entry:121536 Library:NIST17-1.lib
SI:94 Formula:C18H30O2 CAS:0-00-0 MolWeight:278 RetIndex:2002
CompName:Methyl 8,11,14-heptadecatrienoate

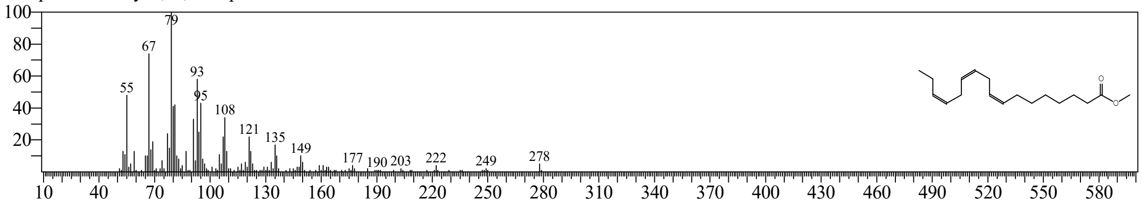

<< Target >>
Line#:16 R.Time:30.608(Scan#:2474) MassPeaks:360
RawMode:Averaged 30.600-30.617(2473-2475) BasePeak:79.05(68984)
BG Mode:Calc. from Peak Group 1 - Event 1

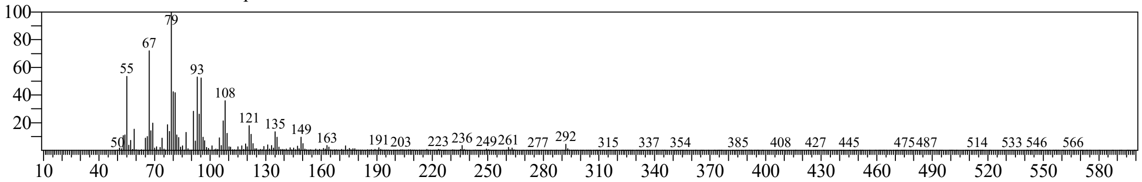


Hit#:1 Entry:124228 Library:NIST14.lib
SI:96 Formula:C19H32O2 CAS:301-00-8 MolWeight:292 RetIndex:2101
CompName:9,12,15-Octadecatrienoic acid, methyl ester, (Z,Z,Z)- $$ Linolenic acid, methyl ester $$ Methyl all-cis-9,12,15-octadecatrienoate $$ Methyl lino

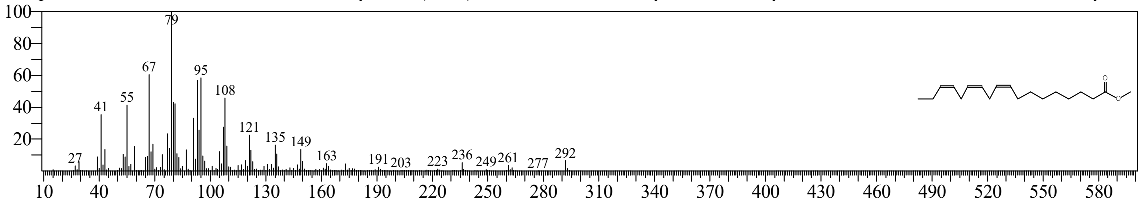


Hit#:2 Entry:135724 Library:NIST17-1.lib
SI:96 Formula:C19H32O2 CAS:301-00-8 MolWeight:292 RetIndex:2101
CompName:9,12,15-Octadecatrienoic acid, methyl ester, (Z,Z,Z)- $$ Linolenic acid, methyl ester $$ Methyl all-cis-9,12,15-octadecatrienoate $$ Methyl lino

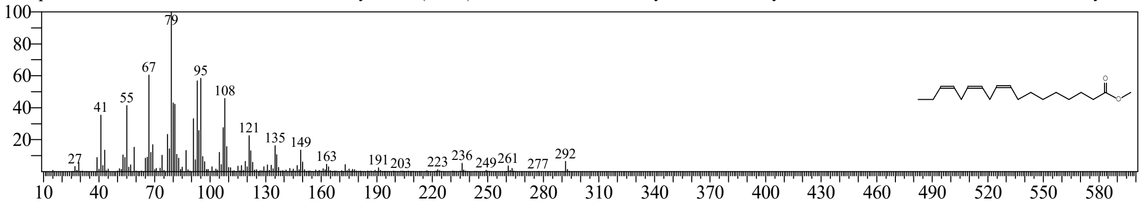


Hit#:3 Entry:32438 Library:NIST17s.lib
SI:95 Formula:C19H32O2 CAS:301-00-8 MolWeight:292 RetIndex:2101
CompName:9,12,15-Octadecatrienoic acid, methyl ester, (Z,Z,Z)- $$ Linolenic acid, methyl ester $$ Methyl all-cis-9,12,15-octadecatrienoate $$ Methyl lino

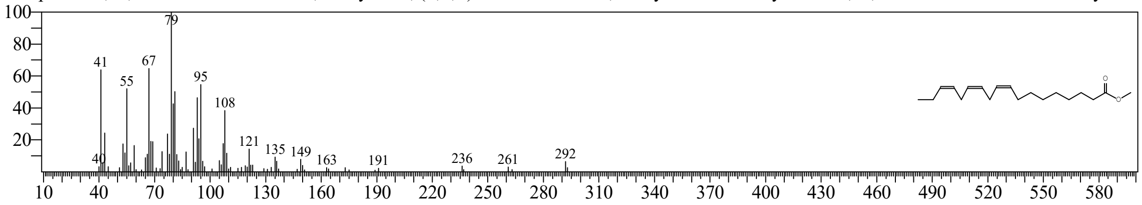


Hit#:4 Entry:27872 Library:NIST14s.lib
SI:95 Formula:C19H32O2 CAS:301-00-8 MolWeight:292 RetIndex:2101
CompName:9,12,15-Octadecatrienoic acid, methyl ester, (Z,Z,Z)- $$ Linolenic acid, methyl ester $$ Methyl all-cis-9,12,15-octadecatrienoate $$ Methyl lino

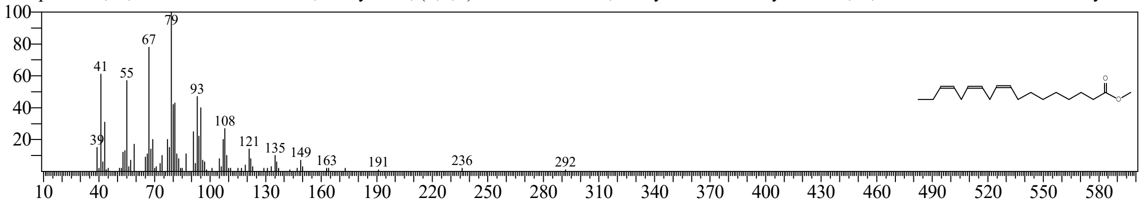


Hit#:5 Entry:32437 Library:NIST17s.lib
SI:95 Formula:C19H32O2 CAS:301-00-8 MolWeight:292 RetIndex:2101
CompName:9,12,15-Octadecatrienoic acid, methyl ester, (Z,Z,Z)- $$ Linolenic acid, methyl ester $$ Methyl all-cis-9,12,15-octadecatrienoate $$ Methyl lino

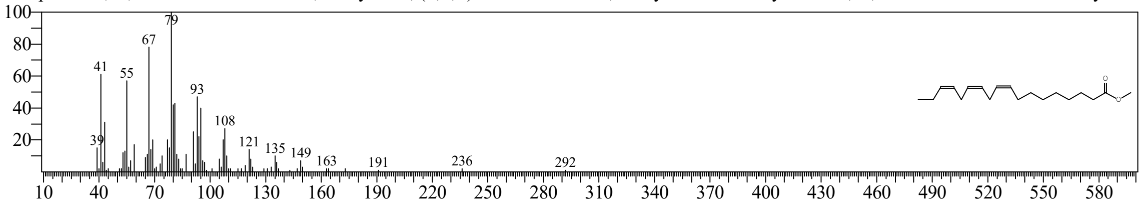

<< Target >>
Line#:17 R.Time:30.933(Scan#:2513) MassPeaks:341
RawMode:Averaged 30.925-30.942(2512-2514) BasePeak:67.00(6633)
BG Mode:Calc. from Peak Group 1 - Event 1

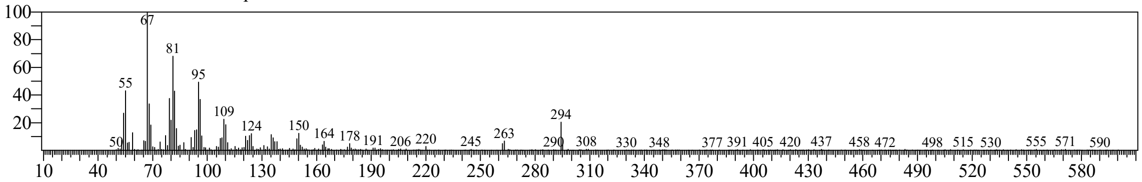


Hit#:1 Entry:137666 Library:NIST17-1.lib
SI:97 Formula:C19H34O2 CAS:13058-52-1 MolWeight:294 RetIndex:2093
CompName:Methyl 9-cis,11-trans-octadecadienoate

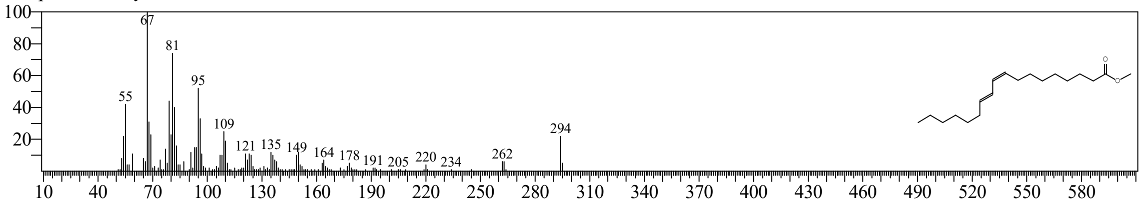


Hit#:2 Entry:125932 Library:NIST14.lib
SI:97 Formula:C19H34O2 CAS:0-00-0 MolWeight:294 RetIndex:2093
CompName:Methyl 9-cis,11-trans-octadecadienoate

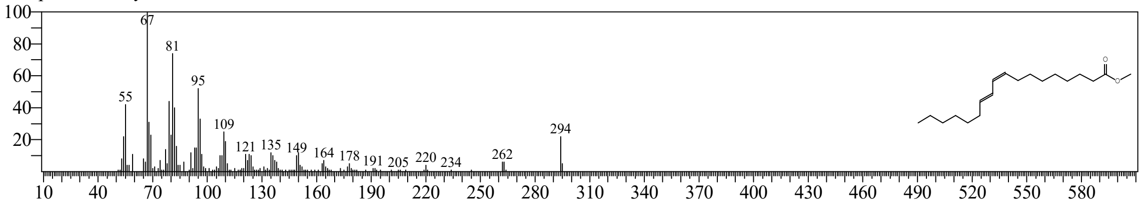


Hit#:3 Entry:125933 Library:NIST14.lib
SI:97 Formula:C19H34O2 CAS:0-00-0 MolWeight:294 RetIndex:2093
CompName:Methyl 10-trans,12-cis-octadecadienoate

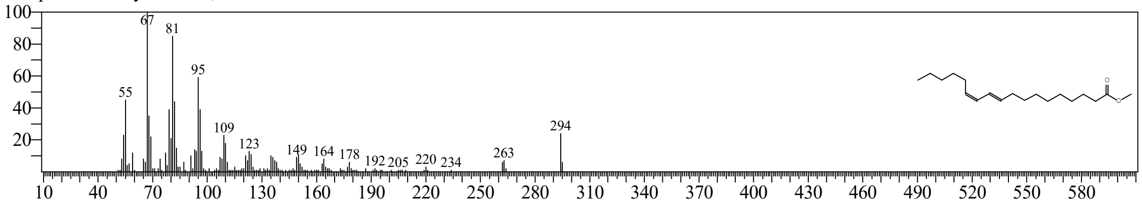


Hit#:4 Entry:137664 Library:NIST17-1.lib
SI:97 Formula:C19H34O2 CAS:0-00-0 MolWeight:294 RetIndex:2093
CompName:Methyl 10-trans,12-cis-octadecadienoate

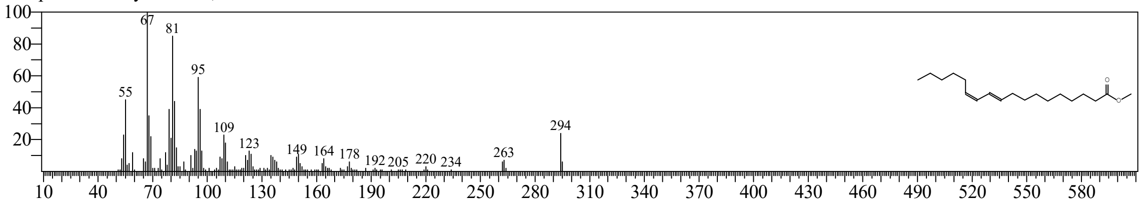


Hit#:5 Entry:32587 Library:NIST17s.lib
SI:95 Formula:C19H34O2 CAS:13038-47-6 MolWeight:294 RetIndex:2093
CompName:9,11-Octadecadienoic acid, methyl ester, (E,E)- $$ Methyl trans-9,trans-11-octadecadienoate $$ Methyl trans,trans-9,11-octadecadienoate $$ M

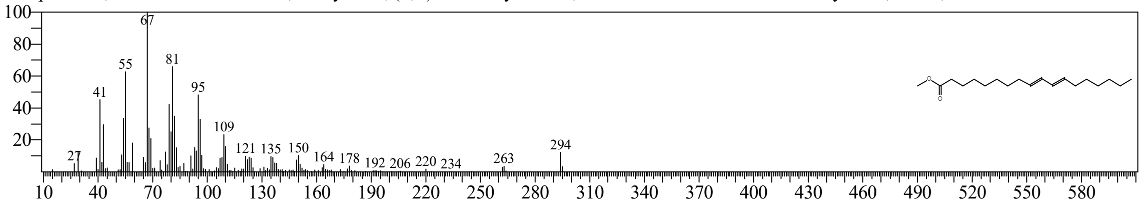

<< Target >>
Line#:18 R.Time:31.258(Scan#:2552) MassPeaks:388
RawMode:Averaged 31.250-31.267(2551-2553) BasePeak:67.05(6920)
BG Mode:Calc. from Peak Group 1 - Event 1

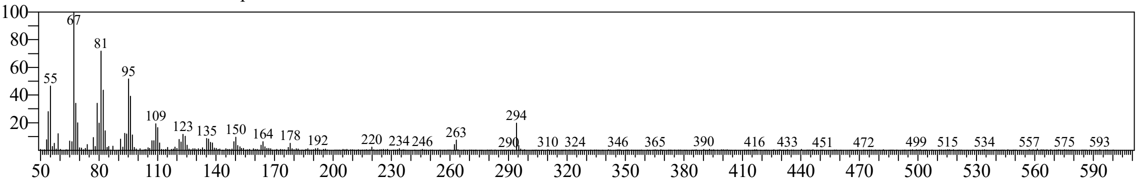


Hit#:1 Entry:125933 Library:NIST14.lib
SI:96 Formula:C19H34O2 CAS:0-00-0 MolWeight:294 RetIndex:2093
CompName:Methyl 10-trans,12-cis-octadecadienoate

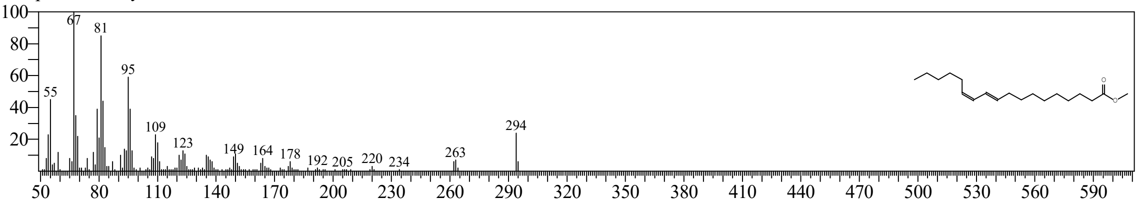


Hit#:2 Entry:137664 Library:NIST17-1.lib
SI:96 Formula:C19H34O2 CAS:0-00-0 MolWeight:294 RetIndex:2093
CompName:Methyl 10-trans,12-cis-octadecadienoate

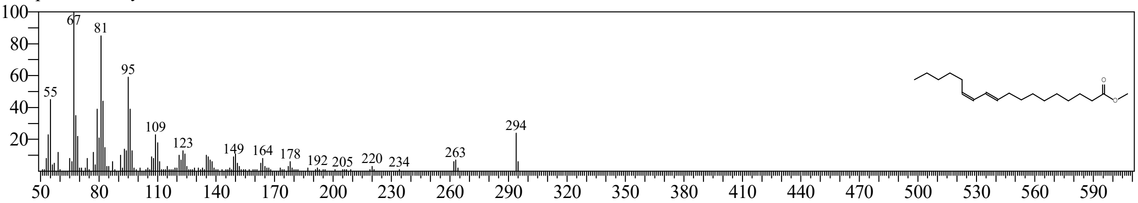


Hit#:3 Entry:125932 Library:NIST14.lib
SI:96 Formula:C19H34O2 CAS:0-00-0 MolWeight:294 RetIndex:2093
CompName:Methyl 9-cis,11-trans-octadecadienoate

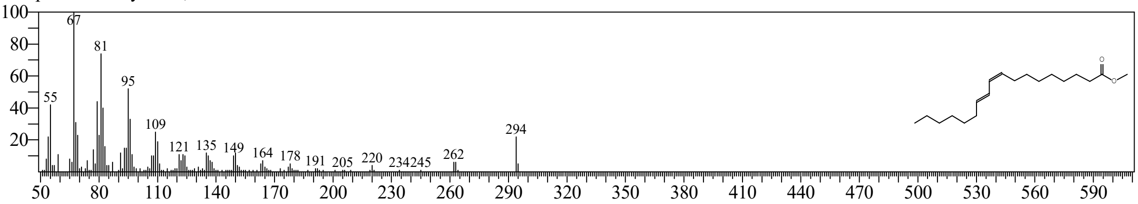


Hit#:4 Entry:137666 Library:NIST17-1.lib
SI:96 Formula:C19H34O2 CAS:13058-52-1 MolWeight:294 RetIndex:2093
CompName:Methyl 9-cis,11-trans-octadecadienoate

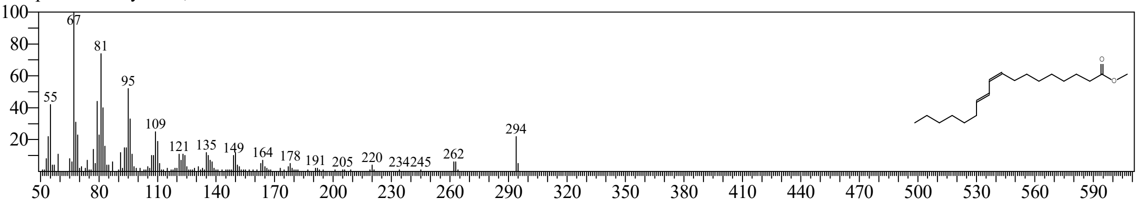


Hit#:5 Entry:28003 Library:NIST14s.lib
SI:94 Formula:C19H34O2 CAS:57156-97-5 MolWeight:294 RetIndex:2093
CompName:12,15-Octadecadienoic acid, methyl ester $$ Methyl (12E,15E)-12,15-octadecadienoate # $$

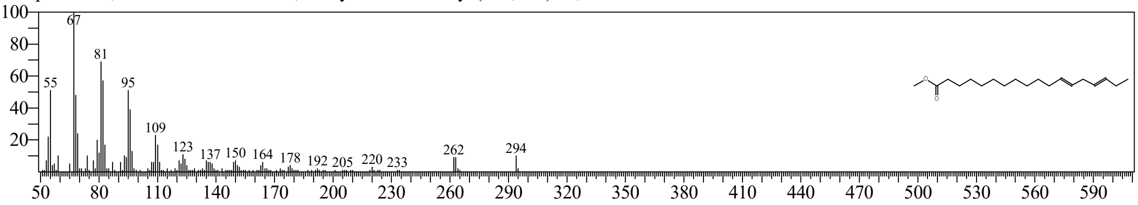

<< Target >>
Line#:19   R.Time:31.933(Scan#:2633)  MassPeaks:374
RawMode:Averaged 31.925-31.942(2632-2634) BasePeak:67.00(1022)
BG Mode:Calc. from Peak  Group 1 - Event 1

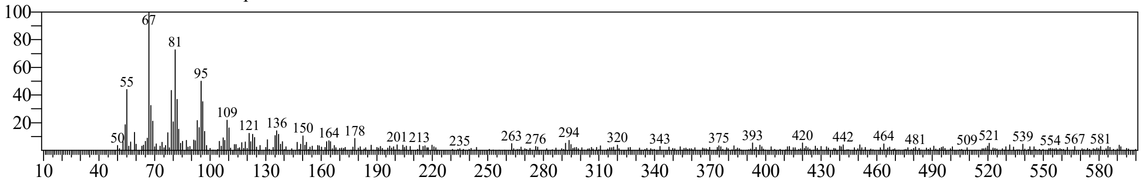


Hit#:1  Entry:125933  Library:NIST14.lib
SI:90  Formula:C19H34O2  CAS:0-00-0  MolWeight:294  RetIndex:2093
CompName:Methyl 10-trans,12-cis-octadecadienoate

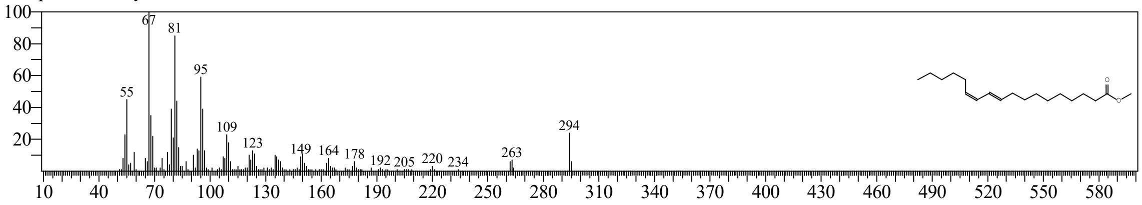


Hit#:2  Entry:125932  Library:NIST14.lib
SI:90  Formula:C19H34O2  CAS:0-00-0  MolWeight:294  RetIndex:2093
CompName:Methyl 9-cis,11-trans-octadecadienoate

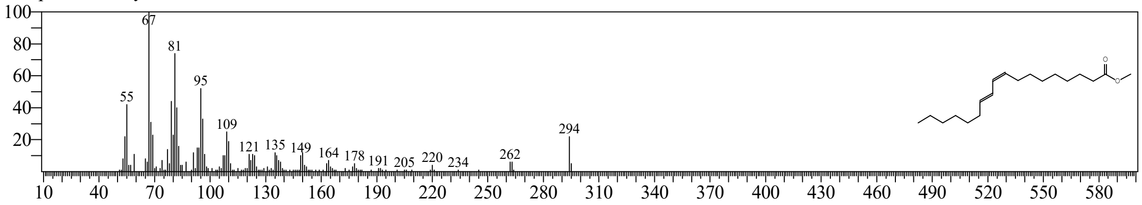


Hit#:3  Entry:137664  Library:NIST17-1.lib
SI:90  Formula:C19H34O2  CAS:0-00-0  MolWeight:294  RetIndex:2093
CompName:Methyl 10-trans,12-cis-octadecadienoate

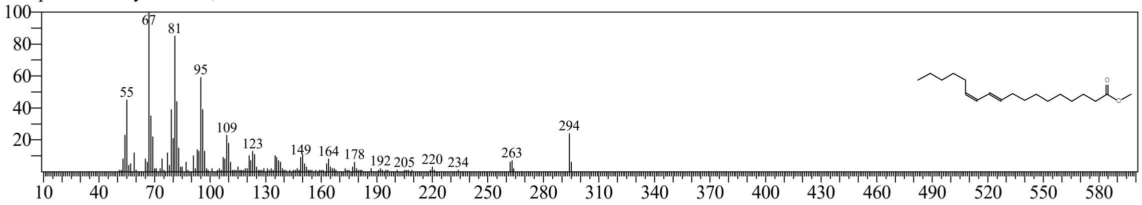


Hit#:4  Entry:137666  Library:NIST17-1.lib
SI:90  Formula:C19H34O2  CAS:13058-52-1  MolWeight:294  RetIndex:2093
CompName:Methyl 9-cis,11-trans-octadecadienoate

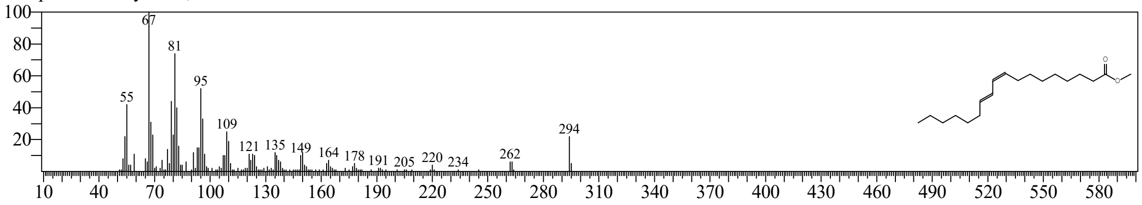


Hit#:5  Entry:32587  Library:NIST17s.lib
SI:90  Formula:C19H34O2  CAS:13038-47-6  MolWeight:294  RetIndex:2093
CompName:9,11-Octadecadienoic acid, methyl ester, (E,E)- $$ Methyl trans-9,trans-11-octadecadienoate $$ Methyl trans,trans-9,11-octadecadienoate $$ M

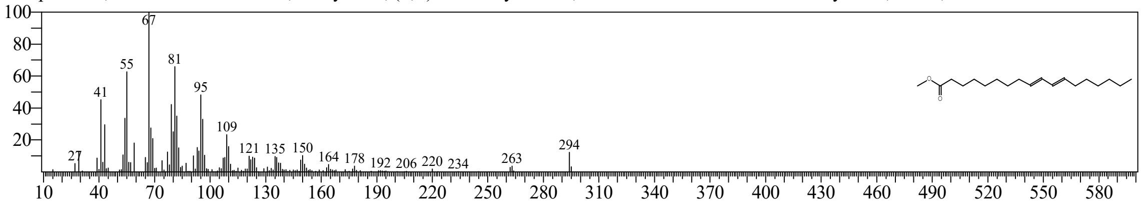

<< Target >>
Line#:20  R.Time:32.958(Scan#:2756)  MassPeaks:317
RawMode:Averaged 32.950-32.967(2755-2757) BasePeak:95.05(8123)
BG Mode:Calc. from Peak  Group 1 - Event 1

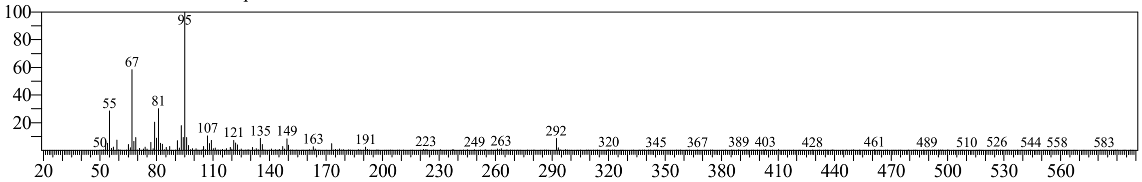


Hit#:1  Entry:24424  Library:NIST17-1.lib
SI:85  Formula:C12H20  CAS:0-00-0  MolWeight:164  RetIndex:1041
CompName:Cis-3-ethyl-endo-tricyclo[5.2.1.0(2.6)]decane

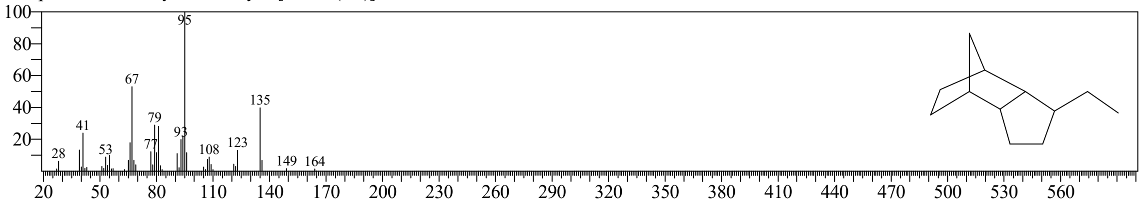


Hit#:2  Entry:23403  Library:NIST14.lib
SI:85  Formula:C12H20  CAS:0-00-0  MolWeight:164  RetIndex:1041
CompName:Cis-3-ethyl-endo-tricyclo[5.2.1.0(2.6)]decane

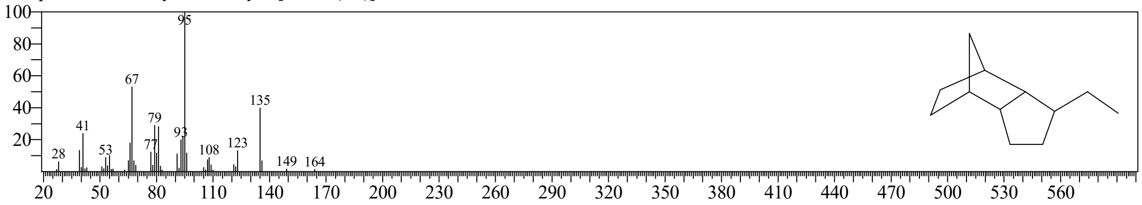


Hit#:3  Entry:16582  Library:NIST17-1.lib
SI:84  Formula:C11H18  CAS:0-00-0  MolWeight:150  RetIndex:1115
CompName:4-Cyclohexenyl-3-methylbutene

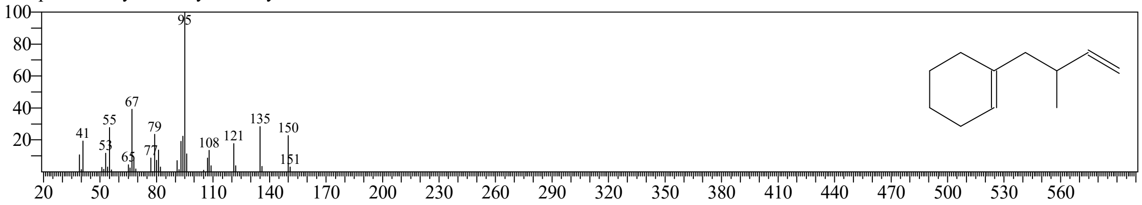


Hit#:4  Entry:17571  Library:NIST17-1.lib
SI:83  Formula:C10H16O  CAS:60485-45-2  MolWeight:152  RetIndex:991
CompName:Santolina epoxide $$ 2-Methyl-2-(3-methyl-1-vinyl-2-butenyl)oxirane # $$

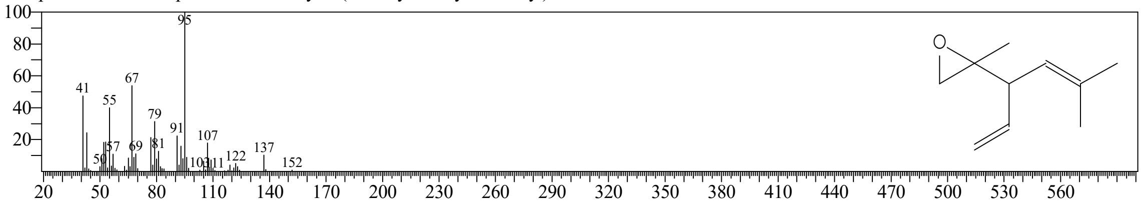


Hit#:5  Entry:16897  Library:NIST14.lib
SI:83  Formula:C10H16O  CAS:60485-45-2  MolWeight:152  RetIndex:991
CompName:Santolina epoxide $$ 2-Methyl-2-(3-methyl-1-vinyl-2-butenyl)oxirane # $$

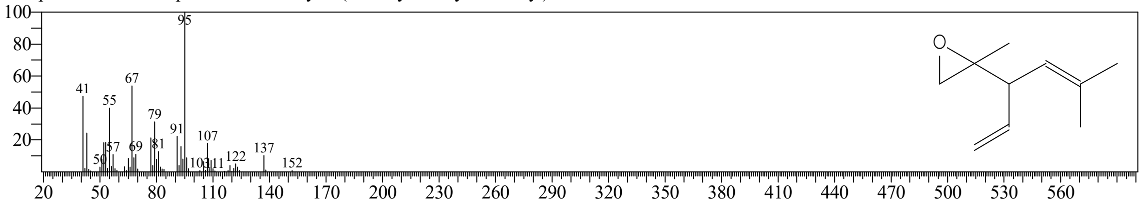

<< Target >>
Line#:21   R.Time:33.492(Scan#:2820)  MassPeaks:360
RawMode:Averaged 33.483-33.500(2819-2821) BasePeak:74.00(15976)
BG Mode:Calc. from Peak  Group 1 - Event 1

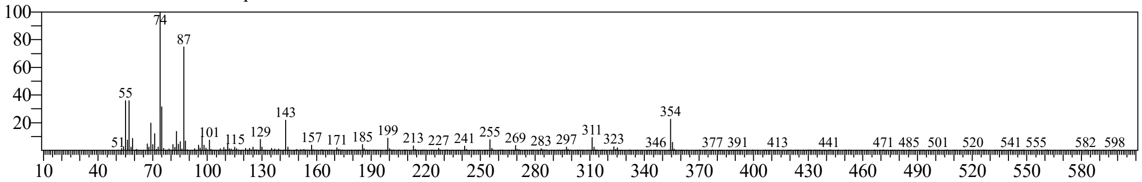

Hit#:1  Entry:196443  Library:NIST17-1.lib
SI:97  Formula:C23H46O2  CAS:929-77-1  MolWeight:354  RetIndex:2475
CompName:Docosanoic acid, methyl ester $$ Behenic acid, methyl ester $$ Methyl behenate $$ Methyl docosanoate $$ n-Docosanoic acid methyl ester $$

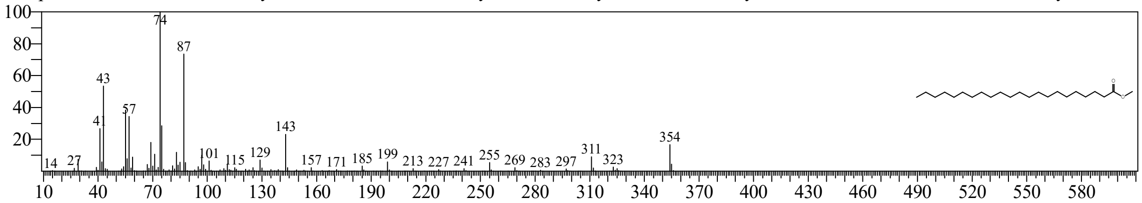

Hit#:2  Entry:178051  Library:NIST14.lib
SI:97  Formula:C23H46O2  CAS:929-77-1  MolWeight:354  RetIndex:2475
CompName:Docosanoic acid, methyl ester $$ Behenic acid, methyl ester $$ Methyl behenate $$ Methyl docosanoate $$ n-Docosanoic acid methyl ester $$

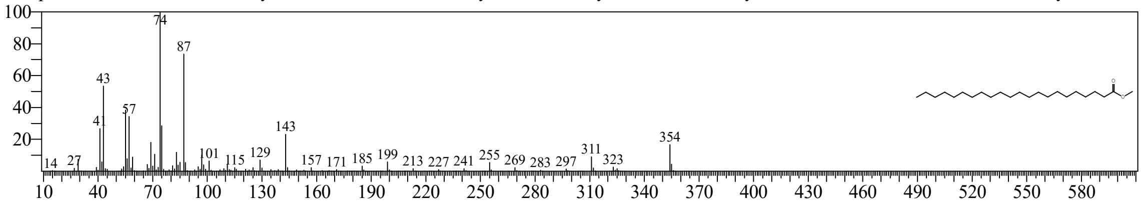

Hit#:3  Entry:196444  Library:NIST17-1.lib
SI:95  Formula:C23H46O2  CAS:0-00-0  MolWeight:354  RetIndex:2411
CompName:Methyl 20-methyl-heneicosanoate

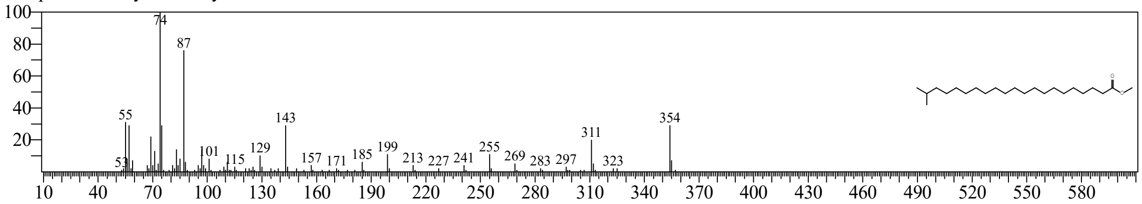

Hit#:4  Entry:178052  Library:NIST14.lib
SI:95  Formula:C23H46O2  CAS:0-00-0  MolWeight:354  RetIndex:2411
CompName:Methyl 20-methyl-heneicosanoate

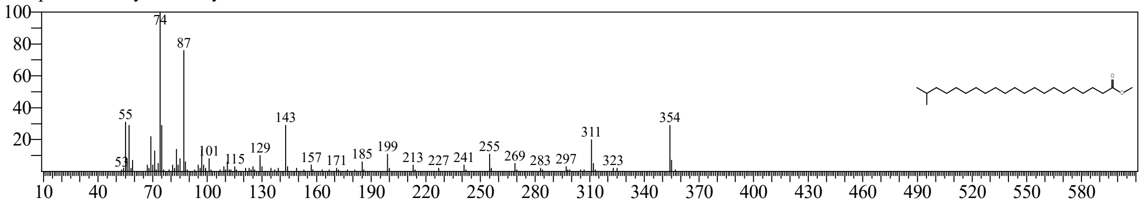

Hit#:5  Entry:31088  Library:NIST14s.lib
SI:94  Formula:C23H46O2  CAS:929-77-1  MolWeight:354  RetIndex:2475
CompName:Docosanoic acid, methyl ester $$ Behenic acid, methyl ester $$ Methyl behenate $$ Methyl docosanoate $$ n-Docosanoic acid methyl ester $$

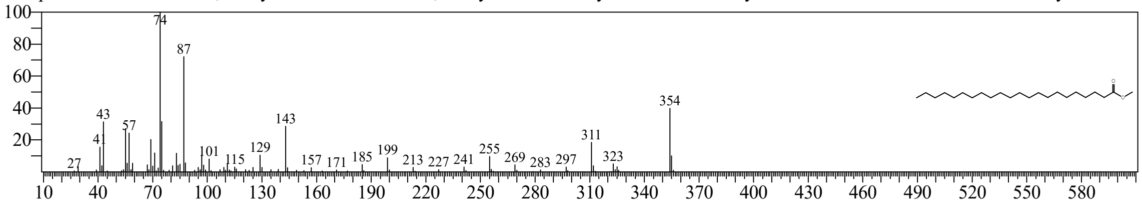

<< Target >>
Line#:22 R.Time:37.800(Scan#:3337) MassPeaks:333
RawMode:Averaged 37.792-37.808(3336-3338) BasePeak:74.00(4175)
BG Mode:Calc. from Peak Group 1 - Event 1

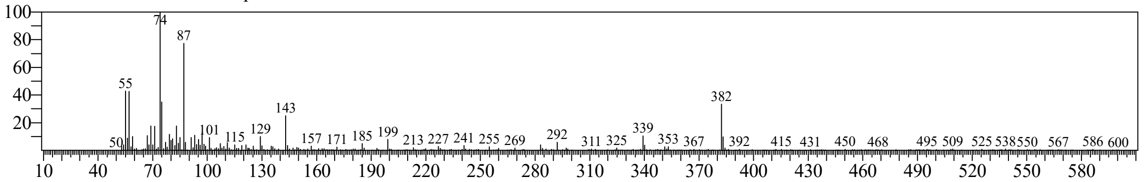


Hit#:1 Entry:37106 Library:NIST17s.lib
SI:89 Formula:C25H50O2 CAS:2442-49-1 MolWeight:382 RetIndex:2674
CompName:Tetracosanoic acid, methyl ester $$ Methyl lignocerate $$ Methyl tetracosanoate $$ Lignoceric acid methyl ester $$

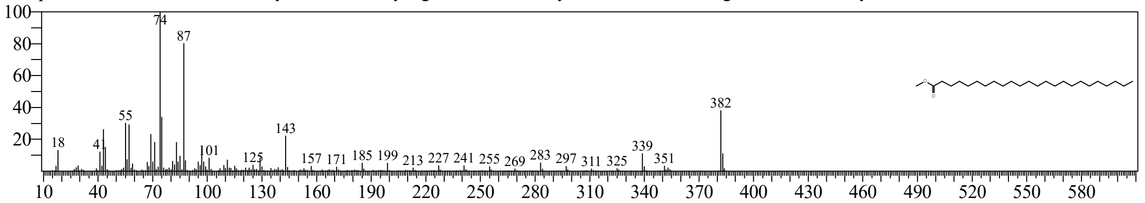


Hit#:2 Entry:31970 Library:NIST14s.lib
SI:89 Formula:C25H50O2 CAS:2442-49-1 MolWeight:382 RetIndex:2674
CompName:Tetracosanoic acid, methyl ester $$ Methyl lignocerate $$ Methyl tetracosanoate $$ Lignoceric acid methyl ester $$

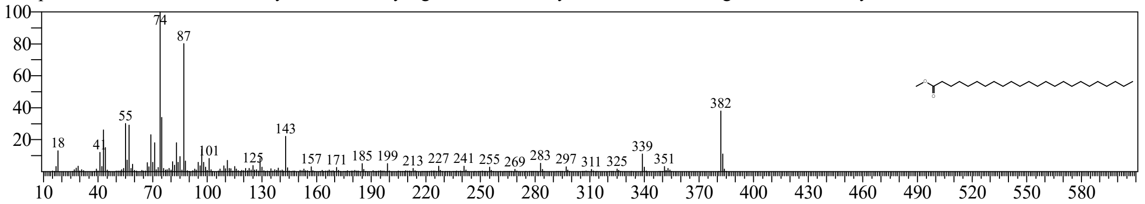


Hit#:3 Entry:37107 Library:NIST17s.lib
SI:86 Formula:C25H50O2 CAS:2442-49-1 MolWeight:382 RetIndex:2674
CompName:Tetracosanoic acid, methyl ester $$ Methyl lignocerate $$ Methyl tetracosanoate $$ Lignoceric acid methyl ester $$

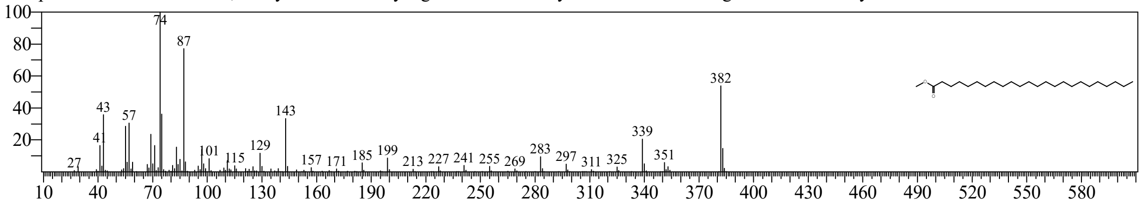


Hit#:4 Entry:31971 Library:NIST14s.lib
SI:86 Formula:C25H50O2 CAS:2442-49-1 MolWeight:382 RetIndex:2674
CompName:Tetracosanoic acid, methyl ester $$ Methyl lignocerate $$ Methyl tetracosanoate $$ Lignoceric acid methyl ester $$

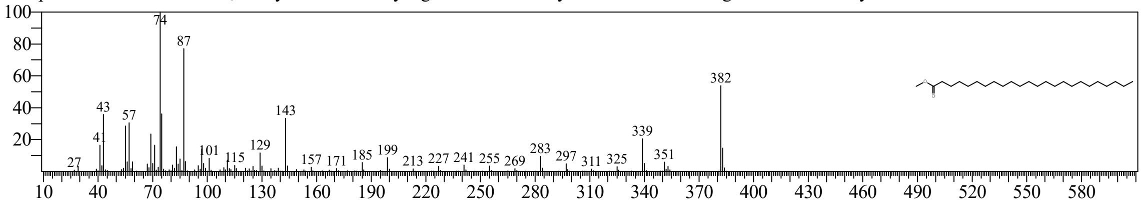


Hit#:5 Entry:217200 Library:NIST17-1.lib
SI:85 Formula:C25H50O2 CAS:2442-49-1 MolWeight:382 RetIndex:2674
CompName:Tetracosanoic acid, methyl ester $$ Methyl lignocerate $$ Methyl tetracosanoate $$ Lignoceric acid methyl ester $$

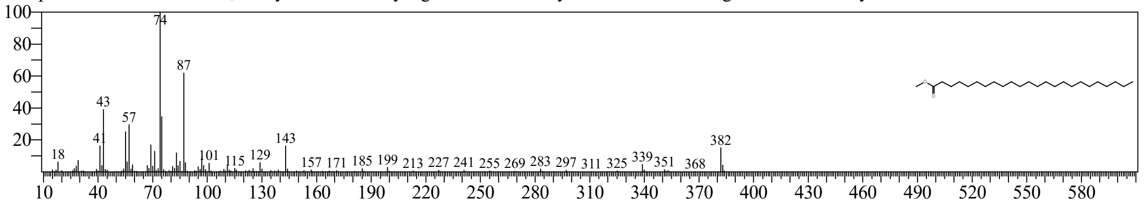

<< Target >>
Line#:23 R.Time:38.325(Scan#:3400) MassPeaks:341
RawMode:Averaged 38.317-38.333(3399-3401) BasePeak:79.00(864)
BG Mode:Calc. from Peak Group 1 - Event 1

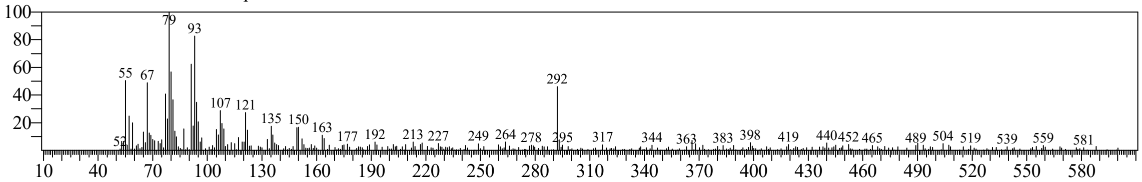


Hit#:1 Entry:135726 Library:NIST17-1.lib
SI:90 Formula:C19H32O2 CAS:0-00-0 MolWeight:292 RetIndex:2101
CompName:Methyl 9.cis.,11.trans.t,13.trans.-octadecatrienoate

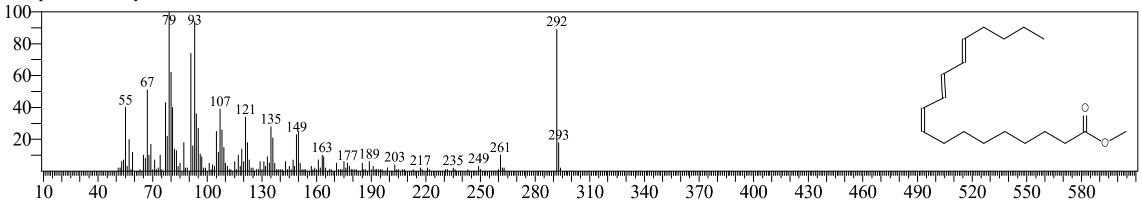


Hit#:2 Entry:124230 Library:NIST14.lib
SI:90 Formula:C19H32O2 CAS:0-00-0 MolWeight:292 RetIndex:2101
CompName:Methyl 9.cis.,11.trans.t,13.trans.-octadecatrienoate

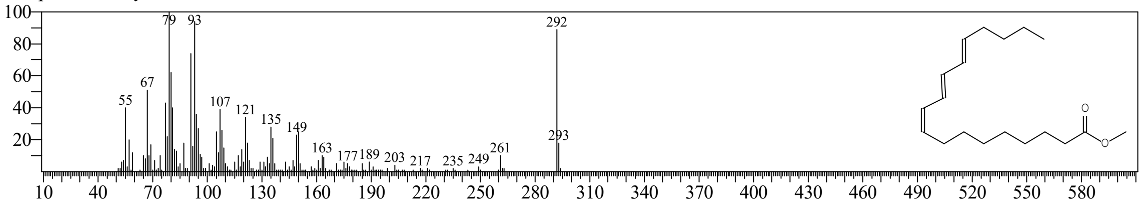


Hit#:3 Entry:135721 Library:NIST17-1.lib
SI:86 Formula:C19H32O2 CAS:16326-32-2 MolWeight:292 RetIndex:2101
CompName:Methyl .gamma.-linolenate

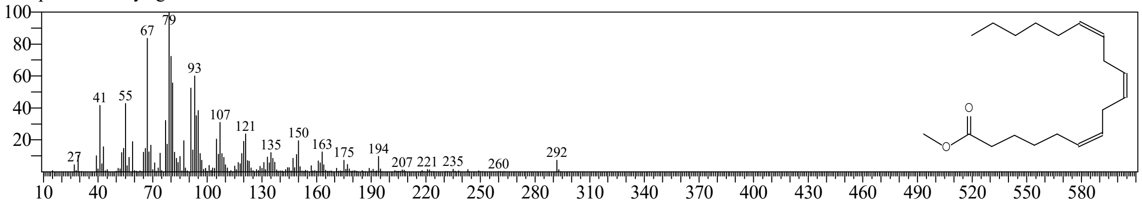


Hit#:4 Entry:124225 Library:NIST14.lib
SI:86 Formula:C19H32O2 CAS:0-00-0 MolWeight:292 RetIndex:2101
CompName:.gamma.-Linolenic acid, methyl ester

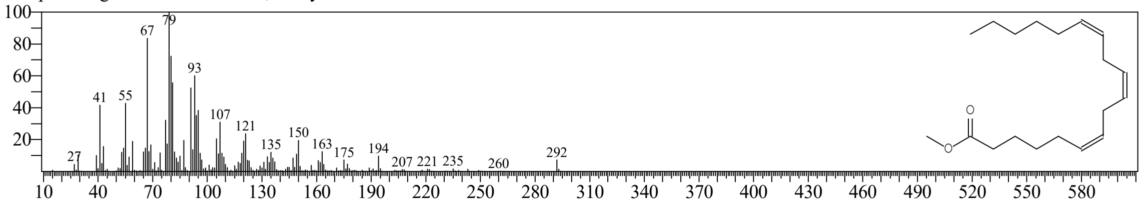


Hit#:5 Entry:124228 Library:NIST14.lib
SI:85 Formula:C19H32O2 CAS:301-00-8 MolWeight:292 RetIndex:2101
CompName:9,12,15-Octadecatrienoic acid, methyl ester, (Z,Z,Z)- $$ Linolenic acid, methyl ester $$ Methyl all-cis-9,12,15-octadecatrienoate $$ Methyl lino

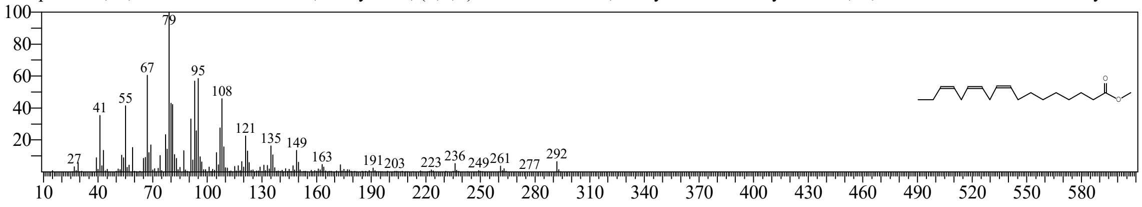

<< Target >>
Line#:24 R.Time:38.742(Scan#:3450) MassPeaks:358
RawMode:Averaged 38.733-38.750(3449-3451) BasePeak:79.05(824)
BG Mode:Calc. from Peak Group 1 - Event 1

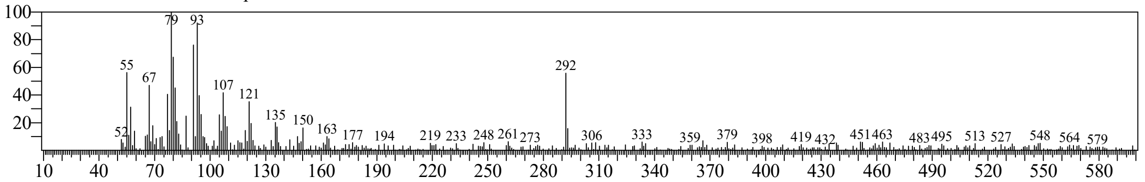


Hit#:1 Entry:124230 Library:NIST14.lib
SI:92 Formula:C19H32O2 CAS:0-00-0 MolWeight:292 RetIndex:2101
CompName:Methyl 9.cis.,11.trans.t,13.trans.-octadecatrienoate

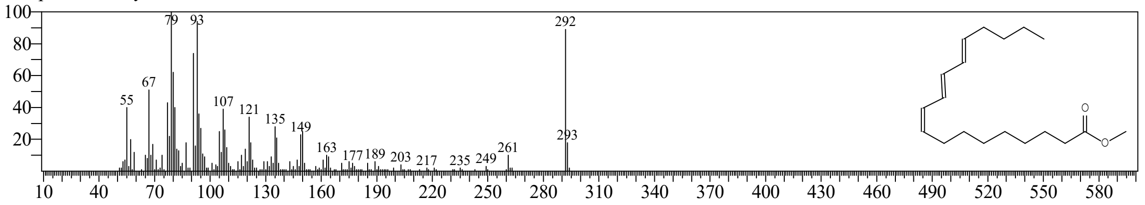


Hit#:2 Entry:135726 Library:NIST17-1.lib
SI:92 Formula:C19H32O2 CAS:0-00-0 MolWeight:292 RetIndex:2101
CompName:Methyl 9.cis.,11.trans.t,13.trans.-octadecatrienoate

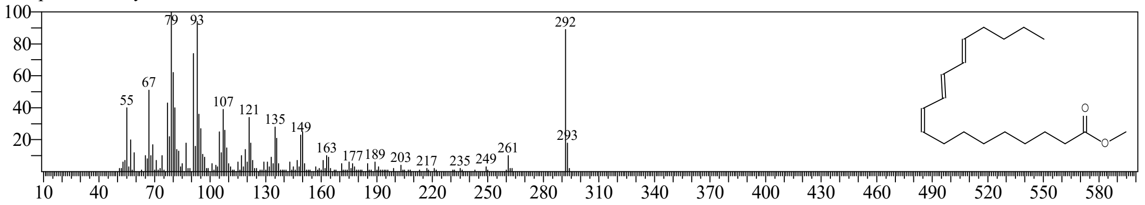


Hit#:3 Entry:124225 Library:NIST14.lib
SI:84 Formula:C19H32O2 CAS:0-00-0 MolWeight:292 RetIndex:2101
CompName:.gamma.-Linolenic acid, methyl ester

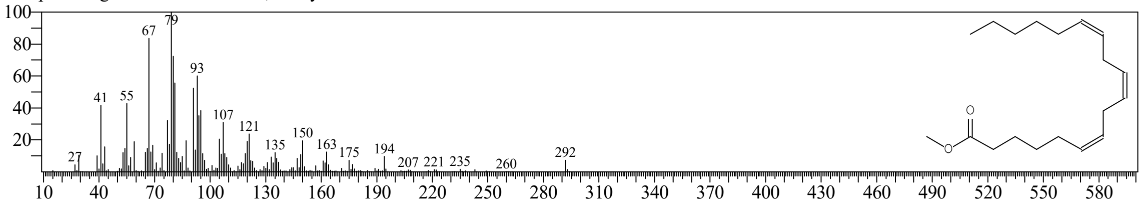


Hit#:4 Entry:135721 Library:NIST17-1.lib
SI:84 Formula:C19H32O2 CAS:16326-32-2 MolWeight:292 RetIndex:2101
CompName:Methyl .gamma.-linolenate

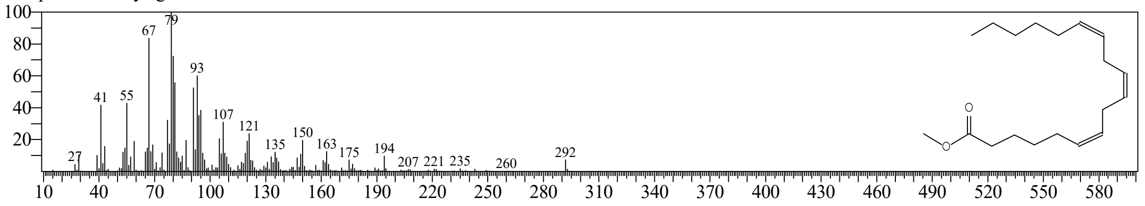


Hit#:5 Entry:124226 Library:NIST14.lib
SI:84 Formula:C19H32O2 CAS:0-00-0 MolWeight:292 RetIndex:2101
CompName:Methyl 6-cis,9-cis,11-trans-octadecatrienoate

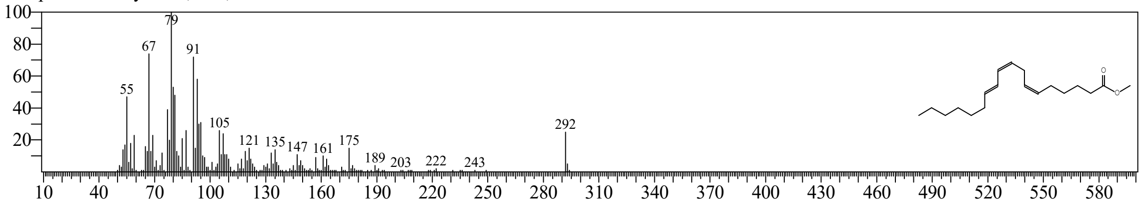

<< Target >>
Line#:25   R.Time:38.892(Scan#:3468)  MassPeaks:338
RawMode:Averaged 38.883-38.900(3467-3469) BasePeak:79.10(1064)
BG Mode:Calc. from Peak  Group 1 - Event 1

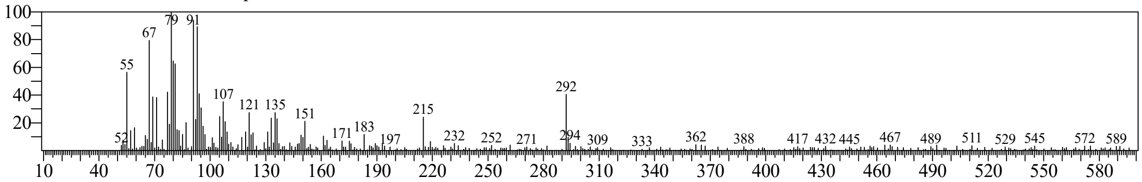


Hit#:1  Entry:124230  Library:NIST14.lib
SI:88  Formula:C19H32O2  CAS:0-00-0  MolWeight:292  RetIndex:2101
CompName:Methyl 9.cis.,11.trans.t,13.trans.-octadecatrienoate

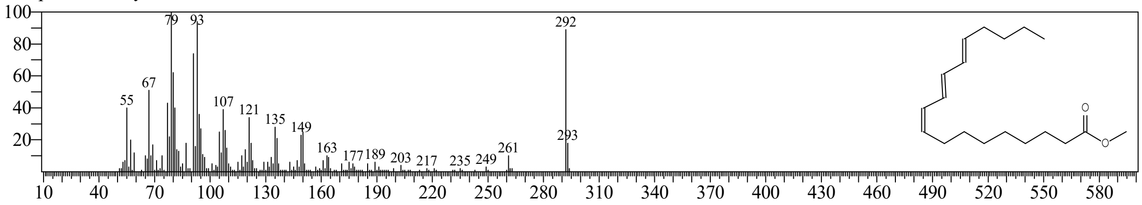


Hit#:2  Entry:135726  Library:NIST17-1.lib
SI:88  Formula:C19H32O2  CAS:0-00-0  MolWeight:292  RetIndex:2101
CompName:Methyl 9.cis.,11.trans.t,13.trans.-octadecatrienoate

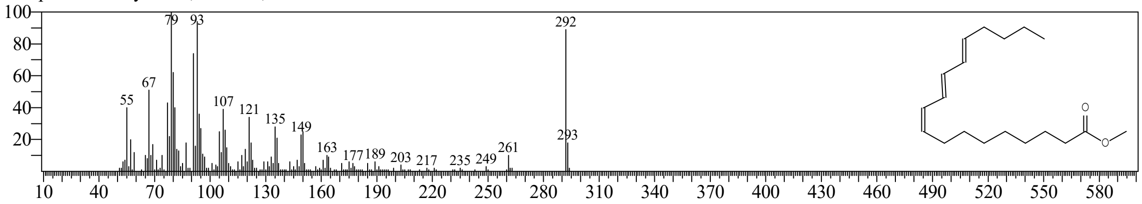


Hit#:3  Entry:135722  Library:NIST17-1.lib
SI:85  Formula:C19H32O2  CAS:0-00-0  MolWeight:292  RetIndex:2101
CompName:Methyl 6-cis,9-cis,11-trans-octadecatrienoate

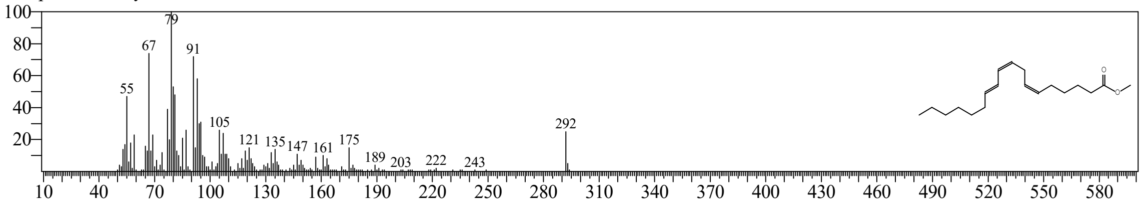


Hit#:4  Entry:124226  Library:NIST14.lib
SI:85  Formula:C19H32O2  CAS:0-00-0  MolWeight:292  RetIndex:2101
CompName:Methyl 6-cis,9-cis,11-trans-octadecatrienoate

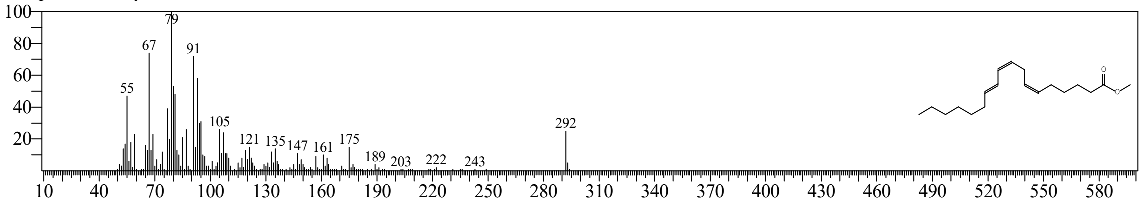


Hit#:5  Entry:149530  Library:NIST14.lib
SI:84  Formula:C21H36O2  CAS:0-00-0  MolWeight:320  RetIndex:2300
CompName:cis-5,8,11-Eicosatrienoic acid, methyl ester

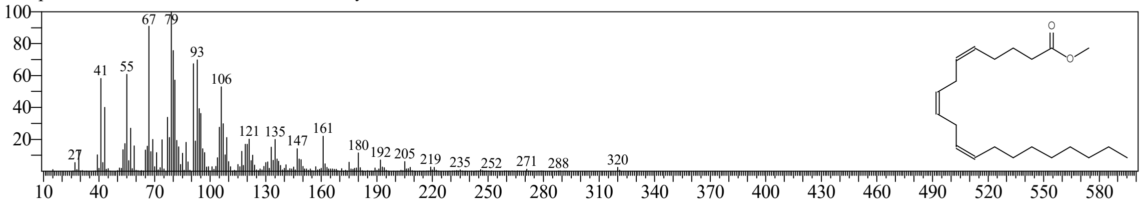

Supplement: Supplementary file 1 [file molecules-31-02891-s001.zip › 28d-QG200-1.pdf]
