# Supplementary material for: Selective Inhibition of Lipid Oxidation by a Quercetagetin-Rich Extract from Tagetes erecta: A Chemometric Approach Using OPLS-DA and Selectivity Index Analysis
Source: Molecules. 2026 Aug 19;31(16):2891. doi: 10.3390/molecules31162891 (PMC13515886; doi:10.3390/molecules31162891)

# QG200-2-28d

<<Column>>
Name : HP-88
Thickness : 0.20um
Length : 100.0m
Inside Diameter : 0.25mm

## Method

===== Analytical Line 1 =====

| | | |
|---|---|---|
| Column Oven Temp. | :120.0 °C | |
| Injection Temp. | :250.00 °C | |
| Injection Mode | :Split | |
| Column Flow | :1.04 mL/min | |
| Split Ratio | :20.0 | |
| Oven Temp. Program | | |
| Rate | Temperature(°C) | Hold Time(min) |
| - | 120.0 | 1.00 |
| 10.00 | 175.0 | 10.00 |
| 5.00 | 210.0 | 5.00 |
| 5.00 | 230.0 | 15.00 |

[GCMS-QP2010]
IonSourceTemp :200.00 °C
Interface Temp. :220.00 °C
*m/z* scanning range：45～500m/z

Chromatogram QG200-2-28d

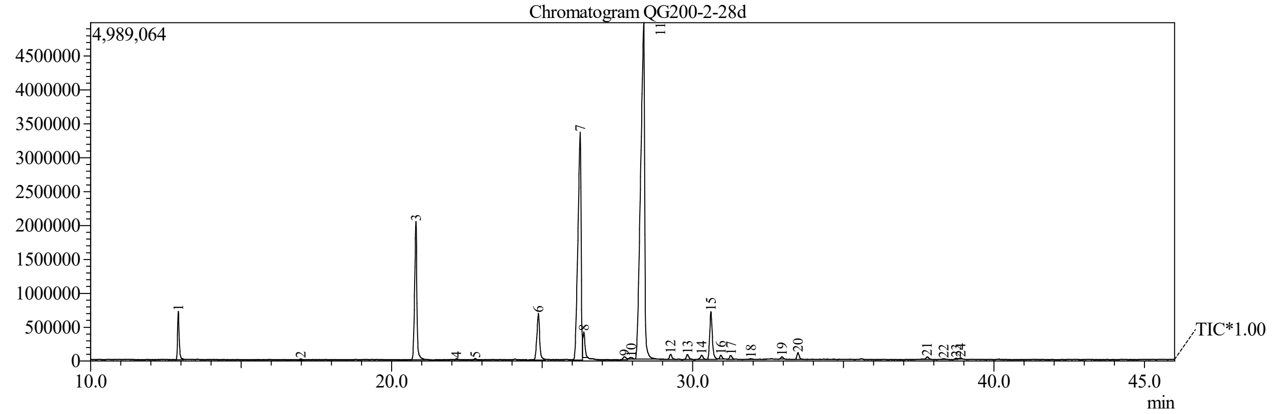


Peak Report TIC

| Peak# | R.Time | Area | Area% | Height | Height% | A/H | Name |
|---|---|---|---|---|---|---|---|
| 1 | 12.917 | 2641343 | 2.69 | 710997 | 5.26 | 3.71 | Undecanoic acid, methyl ester |
| 2 | 16.983 | 74814 | 0.08 | 16217 | 0.12 | 4.61 | Methyl tetradecanoate |
| 3 | 20.808 | 10578809 | 10.76 | 2041027 | 15.11 | 5.18 | Hexadecanoic acid, methyl ester |
| 4 | 22.150 | 63584 | 0.06 | 14154 | 0.10 | 4.49 | 9-Hexadecenoic acid, methyl ester, (Z)- |
| 5 | 22.775 | 100703 | 0.10 | 19002 | 0.14 | 5.30 | Heptadecanoic acid, methyl ester |
| 6 | 24.875 | 4396705 | 4.47 | 686396 | 5.08 | 6.41 | Methyl stearate |
| 7 | 26.262 | 24700164 | 25.12 | 3360393 | 24.87 | 7.35 | 9-Octadecenoic acid, methyl ester, (E)- |
| 8 | 26.383 | 1722988 | 1.75 | 376704 | 2.79 | 4.57 | 11-Octadecenoic acid, methyl ester |
| 9 | 27.738 | 289416 | 0.29 | 41498 | 0.31 | 6.97 | 9,12-Octadecadienoic acid, methyl ester |
| 10 | 27.948 | 200366 | 0.20 | 26975 | 0.20 | 7.43 | 9,12-Octadecadienoic acid (Z,Z)-, methyl ester |
| 11 | 28.372 | 46136986 | 46.92 | 4960116 | 36.71 | 9.30 | 9,12-Octadecadienoic acid (Z,Z)-, methyl ester |
| 12 | 29.263 | 435493 | 0.44 | 75973 | 0.56 | 5.73 | Methyl 18-methylnonadecanoate |
| 13 | 29.823 | 432217 | 0.44 | 72883 | 0.54 | 5.93 | 9,12,15-Octadecatrienoic acid, methyl ester, (Z |
| 14 | 30.297 | 381712 | 0.39 | 60190 | 0.45 | 6.34 | 9,12,15-Octadecatrienoic acid, methyl ester, (Z |

| Peak# | R.Time | Area | Area% | Height | Height% | A/H | Name |
|---|---|---|---|---|---|---|---|
| 15 | 30.603 | 4245476 | 4.32 | 702363 | 5.20 | 6.04 | 9,12,15-Octadecatrienoic acid, methyl ester, (Z |
| 16 | 30.929 | 276082 | 0.28 | 53846 | 0.40 | 5.13 | Methyl 10-trans,12-cis-octadecadienoate |
| 17 | 31.268 | 328922 | 0.33 | 57878 | 0.43 | 5.68 | Methyl 10-trans,12-cis-octadecadienoate |
| 18 | 31.928 | 68714 | 0.07 | 11859 | 0.09 | 5.79 | Methyl 10-trans,12-cis-octadecadienoate |
| 19 | 32.956 | 196735 | 0.20 | 36616 | 0.27 | 5.37 | Cis-3-ethyl-endo-tricyclo[5.2.1.0(2.6)]decane |
| 20 | 33.492 | 576397 | 0.59 | 101365 | 0.75 | 5.69 | Docosanoic acid, methyl ester |
| 21 | 37.782 | 223435 | 0.23 | 35814 | 0.27 | 6.24 | Tetracosanoic acid, methyl ester |
| 22 | 38.331 | 66620 | 0.07 | 10168 | 0.08 | 6.55 | Methyl 9.cis.,11.trans.t,13.trans.-octadecatrien |
| 23 | 38.748 | 98913 | 0.10 | 18293 | 0.14 | 5.41 | Methyl 9.cis.,11.trans.t,13.trans.-octadecatrien |
| 24 | 38.897 | 99312 | 0.10 | 19563 | 0.14 | 5.08 | Methyl 9.cis.,11.trans.t,13.trans.-octadecatrien |
| | | 98335906 | 100.00 | 13510290 | 100.00 | | |

Library

<< Target >>

Line#:1 R.Time:12.917(Scan#:351) MassPeaks:332

RawMode:Averaged 12.908-12.925(350-352) BasePeak:74.00(208741)

BG Mode:Calc. from Peak Group 1 - Event 1

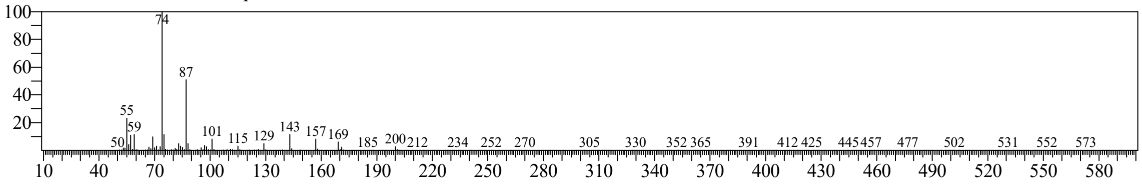

Hit#:1 Entry:49598 Library:NIST17-1.lib

SI:95 Formula:C12H24O2 CAS:1731-86-8 MolWeight:200 RetIndex:1381

CompName:Undecanoic acid, methyl ester $$ Methyl undecanoate $$ Methyl undecylenate $$ n-Undecanoic acid methyl ester $$ Methyl ester of undecanoi

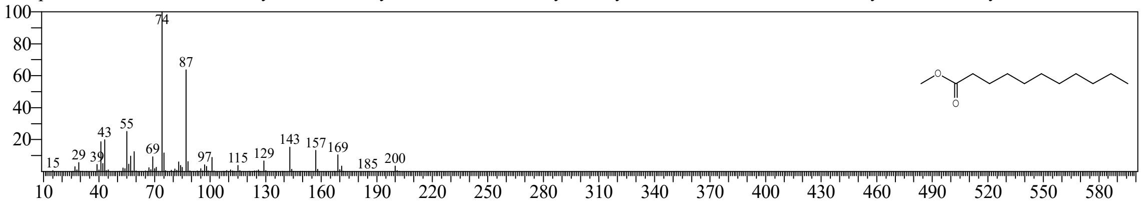

Hit#:2 Entry:46966 Library:NIST14.lib

SI:95 Formula:C12H24O2 CAS:1731-86-8 MolWeight:200 RetIndex:1381

CompName:Undecanoic acid, methyl ester $$ Methyl undecanoate $$ Methyl undecylenate $$ n-Undecanoic acid methyl ester $$ Methyl ester of undecanoi

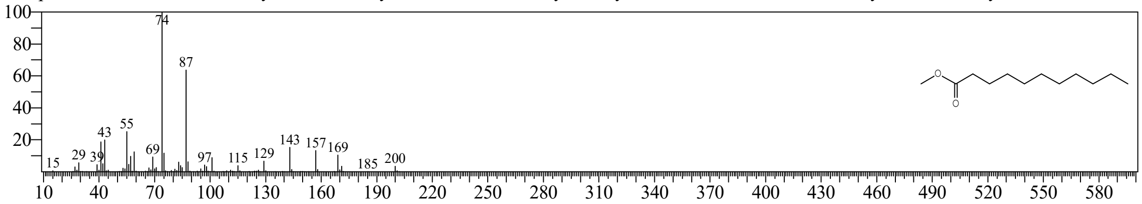

Hit#:3 Entry:21618 Library:NIST17s.lib

SI:95 Formula:C12H24O2 CAS:1731-86-8 MolWeight:200 RetIndex:1381

CompName:Undecanoic acid, methyl ester $$ Methyl undecanoate $$ Methyl undecylenate $$ n-Undecanoic acid methyl ester $$ Methyl ester of undecanoi

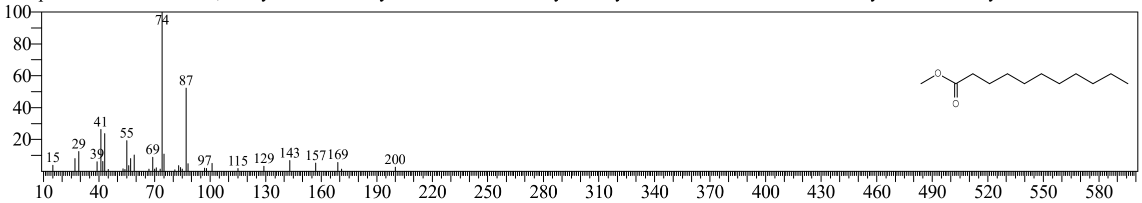

Hit#:4 Entry:21619 Library:NIST17s.lib

SI:95 Formula:C12H24O2 CAS:1731-86-8 MolWeight:200 RetIndex:1381

CompName:Undecanoic acid, methyl ester $$ Methyl undecanoate $$ Methyl undecylenate $$ n-Undecanoic acid methyl ester $$ Methyl ester of undecanoi

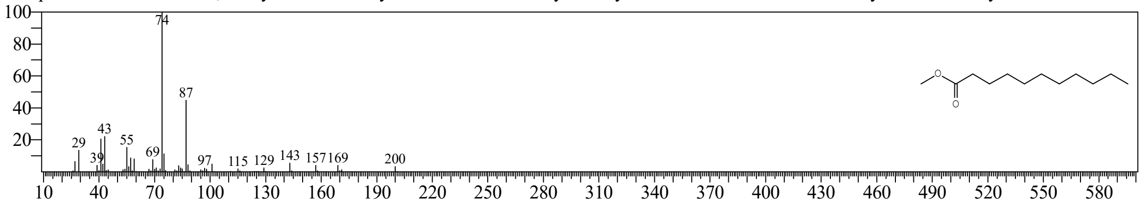

<< Target >>
Line#:1 R.Time:12.917(Scan#:351) MassPeaks:332
RawMode:Averaged 12.908-12.925(350-352) BasePeak:74.00(208741)
BG Mode:Calc. from Peak Group 1 - Event 1

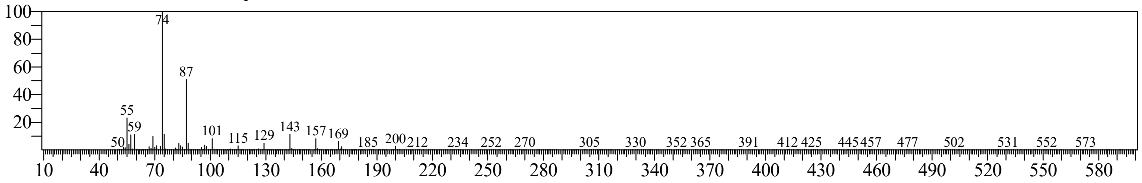


Hit#:5 Entry:18487 Library:NIST14s.lib
SI:95 Formula:C12H24O2 CAS:1731-86-8 MolWeight:200 RetIndex:1381
CompName:Undecanoic acid, methyl ester $$ Methyl undecanoate $$ Methyl undecylenate $$ n-Undecanoic acid methyl ester $$ Methyl ester of undecanoi

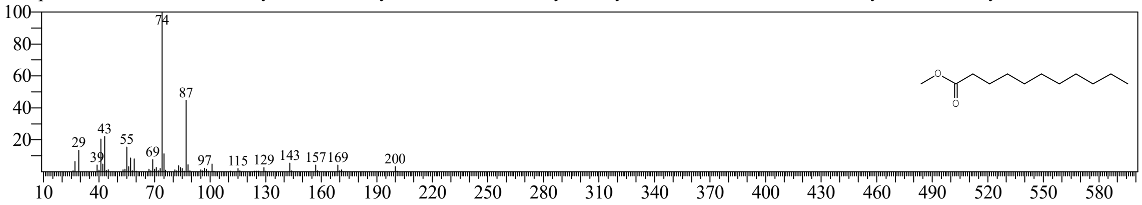

<< Target >>
Line#:2 R.Time:16.983(Scan#:839) MassPeaks:334
RawMode:Averaged 16.975-16.992(838-840) BasePeak:74.00(2845)
BG Mode:Calc. from Peak Group 1 - Event 1

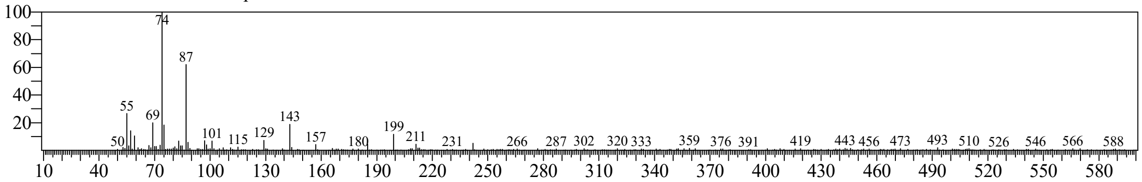


Hit#:1 Entry:80645 Library:NIST14.lib
SI:93 Formula:C15H30O2 CAS:124-10-7 MolWeight:242 RetIndex:1680
CompName:Methyl tetradecanoate $$ Tetradecanoic acid, methyl ester $$ Myristic acid, methyl ester $$ Metholeneat 2495 $$ Methyl myristate $$ Methyl r

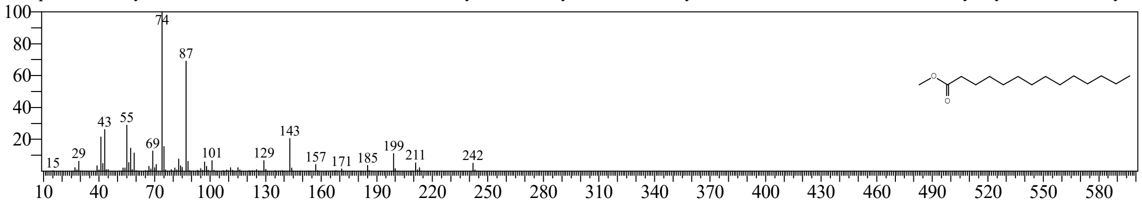


Hit#:2 Entry:86786 Library:NIST17-1.lib
SI:93 Formula:C15H30O2 CAS:124-10-7 MolWeight:242 RetIndex:1680
CompName:Methyl tetradecanoate $$ Tetradecanoic acid, methyl ester $$ Myristic acid, methyl ester $$ Metholeneat 2495 $$ Methyl myristate $$ Methyl r

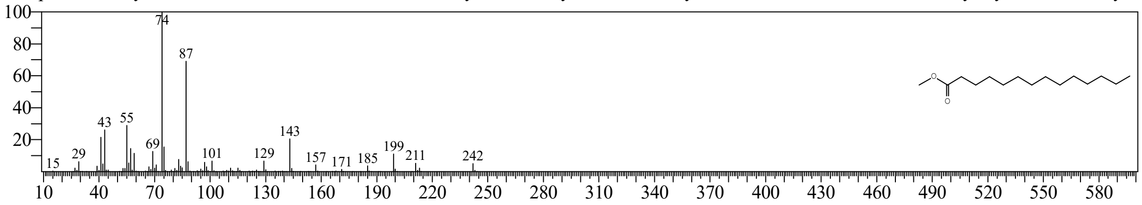


Hit#:3 Entry:27874 Library:NIST17s.lib
SI:92 Formula:C15H30O2 CAS:124-10-7 MolWeight:242 RetIndex:1680
CompName:Methyl tetradecanoate $$ Tetradecanoic acid, methyl ester $$ Myristic acid, methyl ester $$ Metholeneat 2495 $$ Methyl myristate $$ Methyl r

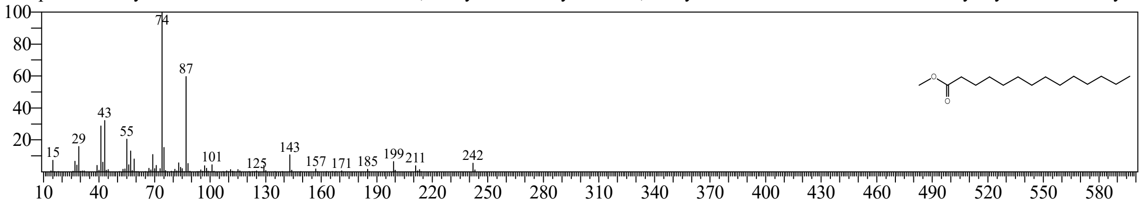


Hit#:4 Entry:23848 Library:NIST14s.lib
SI:92 Formula:C15H30O2 CAS:124-10-7 MolWeight:242 RetIndex:1680
CompName:Methyl tetradecanoate $$ Tetradecanoic acid, methyl ester $$ Myristic acid, methyl ester $$ Metholeneat 2495 $$ Methyl myristate $$ Methyl r

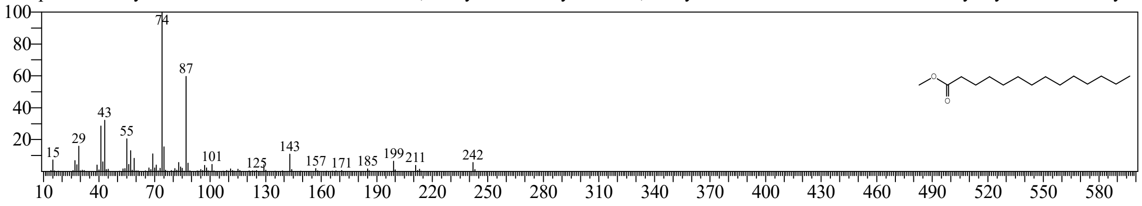


Hit#:5 Entry:23851 Library:NIST14s.lib
SI:92 Formula:C15H30O2 CAS:5129-58-8 MolWeight:242 RetIndex:1615
CompName:Tridecanoic acid, 12-methyl-, methyl ester $$ Methyl isomyristate $$ Methyl 12-methyltridecanoate $$

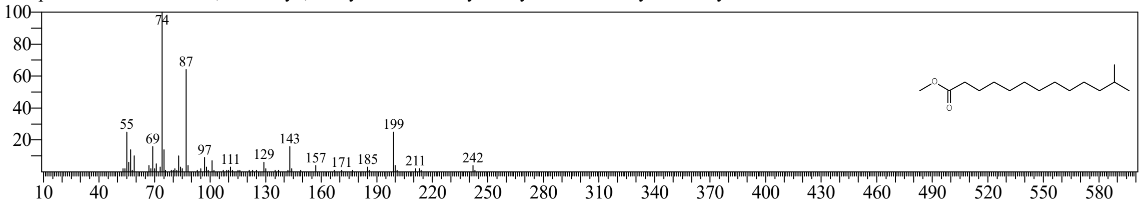

<< Target >>
Line#:3 R.Time:20.808(Scan#:1298) MassPeaks:391
RawMode:Averaged 20.800-20.817(1297-1299) BasePeak:74.00(473450)
BG Mode:Calc. from Peak Group 1 - Event 1

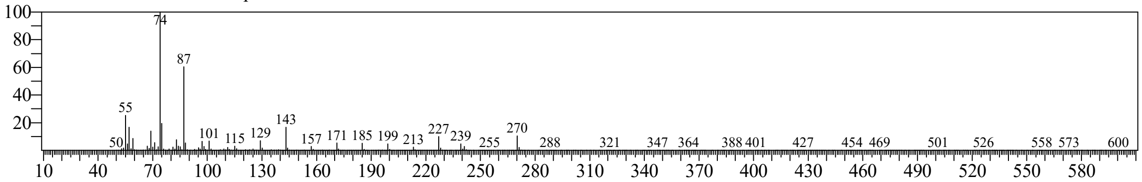


Hit#:1 Entry:113663 Library:NIST17-1.lib
SI:97 Formula:C17H34O2 CAS:112-39-0 MolWeight:270 RetIndex:1878
CompName:Hexadecanoic acid, methyl ester $$ Palmitic acid, methyl ester $$ n-Hexadecanoic acid methyl ester $$ Metholene 2216 $$ Methyl hexadecano

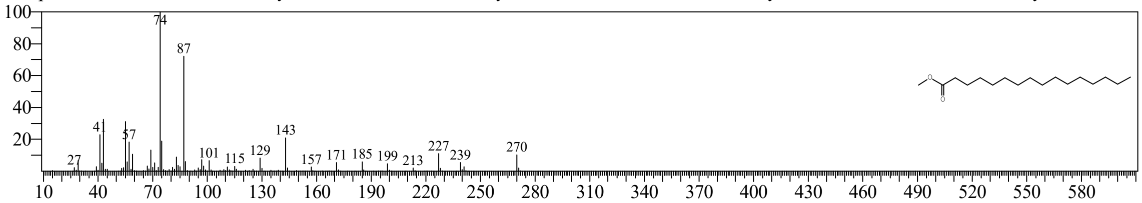


Hit#:2 Entry:104648 Library:NIST14.lib
SI:97 Formula:C17H34O2 CAS:112-39-0 MolWeight:270 RetIndex:1878
CompName:Hexadecanoic acid, methyl ester $$ Palmitic acid, methyl ester $$ n-Hexadecanoic acid methyl ester $$ Metholene 2216 $$ Methyl hexadecano

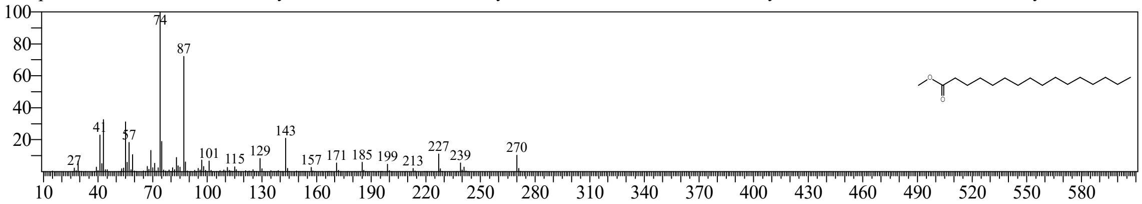


Hit#:3 Entry:26273 Library:NIST14s.lib
SI:97 Formula:C17H34O2 CAS:112-39-0 MolWeight:270 RetIndex:1878
CompName:Hexadecanoic acid, methyl ester $$ Palmitic acid, methyl ester $$ n-Hexadecanoic acid methyl ester $$ Metholene 2216 $$ Methyl hexadecano

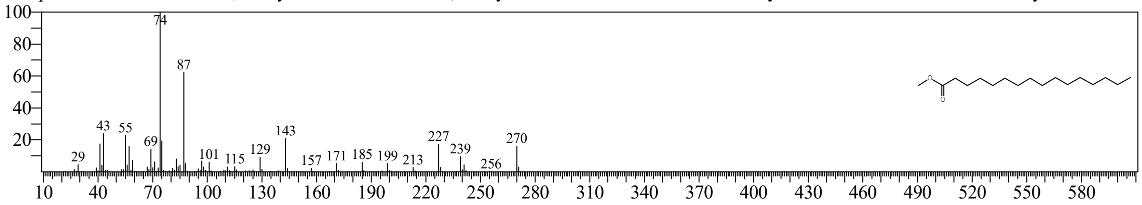


Hit#:4 Entry:26271 Library:NIST14s.lib
SI:96 Formula:C17H34O2 CAS:112-39-0 MolWeight:270 RetIndex:1878
CompName:Hexadecanoic acid, methyl ester $$ Palmitic acid, methyl ester $$ n-Hexadecanoic acid methyl ester $$ Metholene 2216 $$ Methyl hexadecano

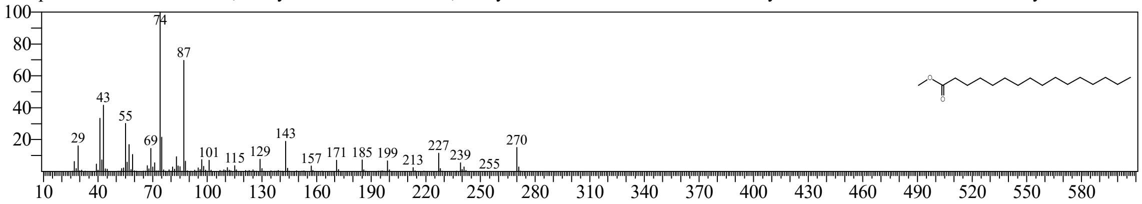


Hit#:5 Entry:30646 Library:NIST17s.lib
SI:96 Formula:C17H34O2 CAS:112-39-0 MolWeight:270 RetIndex:1878
CompName:Hexadecanoic acid, methyl ester $$ Palmitic acid, methyl ester $$ n-Hexadecanoic acid methyl ester $$ Metholene 2216 $$ Methyl hexadecano

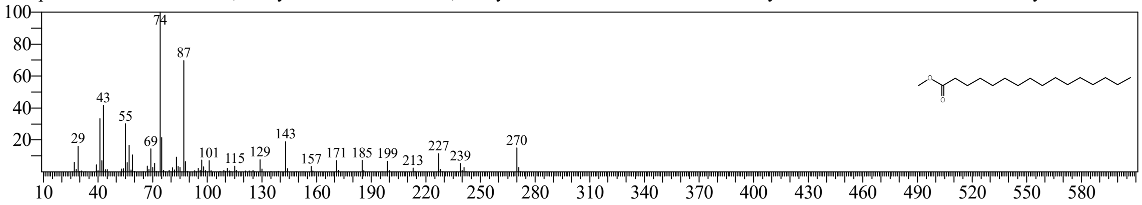

<< Target >>
Line#:4 R.Time:22.150(Scan#:1459) MassPeaks:306
RawMode:Averaged 22.142-22.158(1458-1460) BasePeak:55.05(1232)
BG Mode:Calc. from Peak Group 1 - Event 1

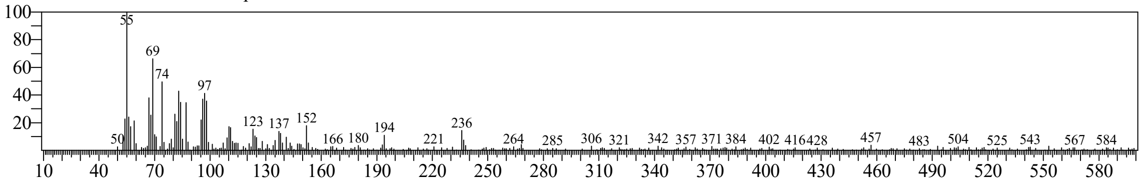


Hit#:1 Entry:30415 Library:NIST17s.lib
SI:93 Formula:C17H32O2 CAS:1120-25-8 MolWeight:268 RetIndex:1886
CompName:9-Hexadecenoic acid, methyl ester, (Z)- $$ Methyl palmitoleate $$ Methyl palmitoleinate $$ Palmitoleic acid, methyl ester $$ Methyl (9Z)-9-he:

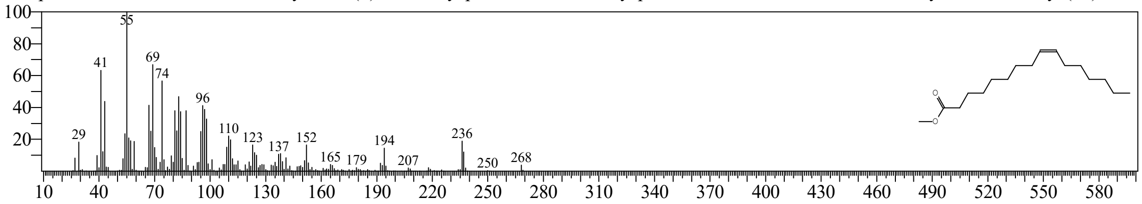


Hit#:2 Entry:26066 Library:NIST14s.lib
SI:93 Formula:C17H32O2 CAS:1120-25-8 MolWeight:268 RetIndex:1886
CompName:9-Hexadecenoic acid, methyl ester, (Z)- $$ Methyl palmitoleate $$ Methyl palmitoleinate $$ Palmitoleic acid, methyl ester $$ Methyl (9Z)-9-he:

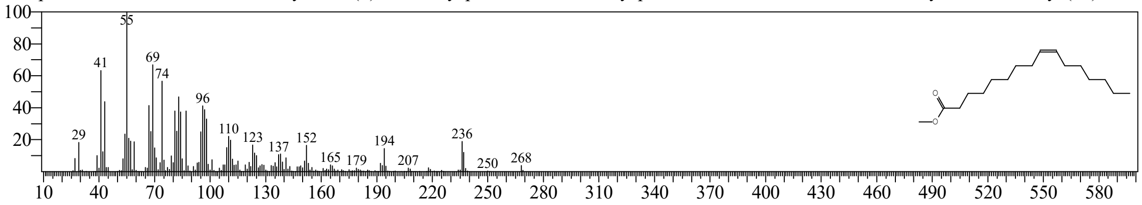


Hit#:3 Entry:111510 Library:NIST17-1.lib
SI:91 Formula:C17H32O2 CAS:56875-67-3 MolWeight:268 RetIndex:1886
CompName:7-Hexadecenoic acid, methyl ester, (Z)- $$ Methyl (7E)-7-hexadecenoate # $$

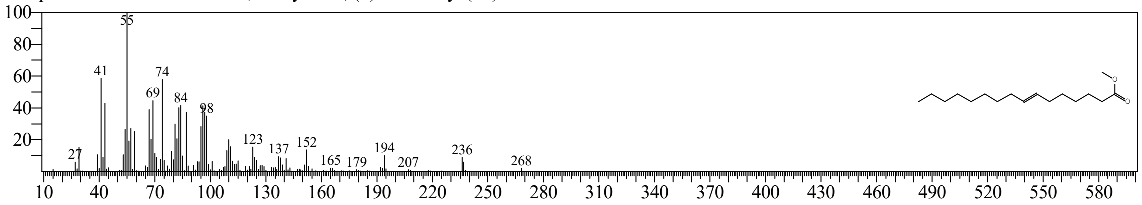


Hit#:4 Entry:30413 Library:NIST17s.lib
SI:91 Formula:C17H32O2 CAS:1120-25-8 MolWeight:268 RetIndex:1886
CompName:9-Hexadecenoic acid, methyl ester, (Z)- $$ Methyl palmitoleate $$ Methyl palmitoleinate $$ Palmitoleic acid, methyl ester $$ Methyl (9Z)-9-he:

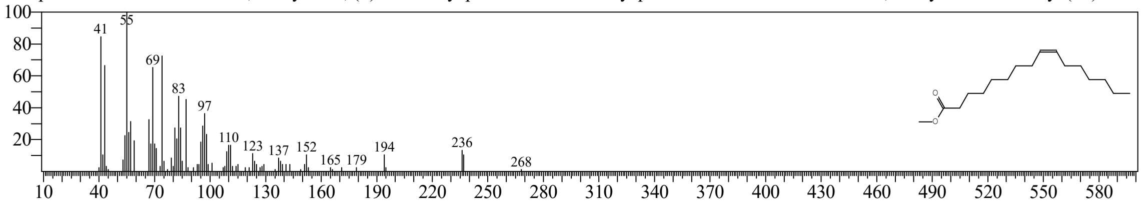


Hit#:5 Entry:26064 Library:NIST14s.lib
SI:91 Formula:C17H32O2 CAS:1120-25-8 MolWeight:268 RetIndex:1886
CompName:9-Hexadecenoic acid, methyl ester, (Z)- $$ Methyl palmitoleate $$ Methyl palmitoleinate $$ Palmitoleic acid, methyl ester $$ Methyl (9Z)-9-he:

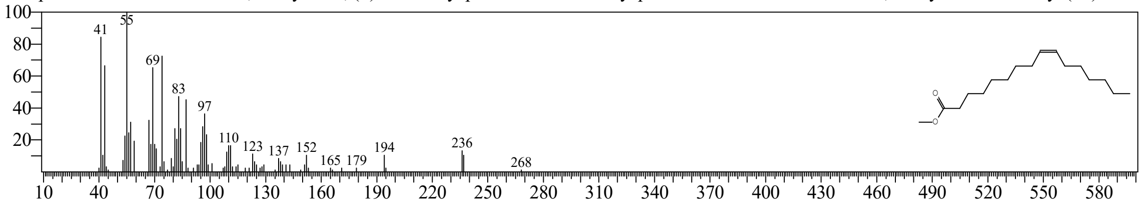

<< Target >>
Line#:5  R.Time:22.775(Scan#:1534)  MassPeaks:335
RawMode:Averaged 22.767-22.783(1533-1535) BasePeak:74.05(3431)
BG Mode:Calc. from Peak  Group 1 - Event 1

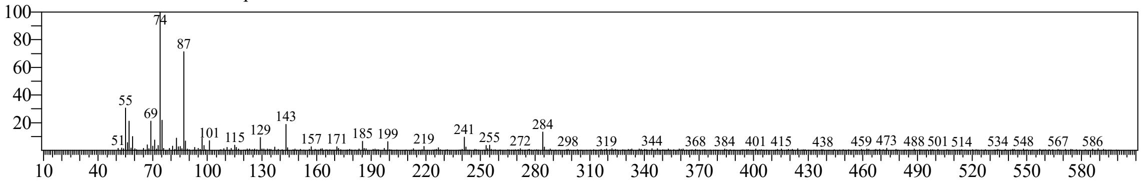


Hit#:1  Entry:27279  Library:NIST14s.lib
SI:93  Formula:C18H36O2  CAS:1731-92-6  MolWeight:284  RetIndex:1978
CompName:Heptadecanoic acid, methyl ester $$ Margaric acid methyl ester $$ Methyl heptadecanoate $$ Methyl margarate $$ n-Heptadecanoic acid methy

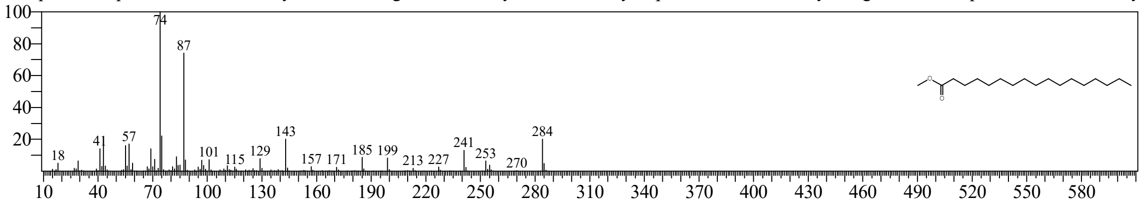


Hit#:2  Entry:31787  Library:NIST17s.lib
SI:93  Formula:C18H36O2  CAS:1731-92-6  MolWeight:284  RetIndex:1978
CompName:Heptadecanoic acid, methyl ester $$ Margaric acid methyl ester $$ Methyl heptadecanoate $$ Methyl margarate $$ n-Heptadecanoic acid methy

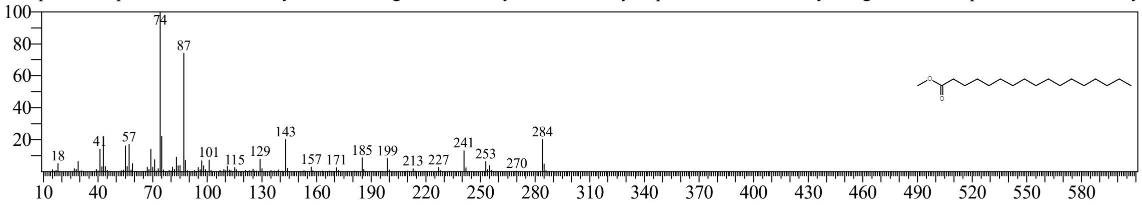


Hit#:3  Entry:127714  Library:NIST17-1.lib
SI:92  Formula:C18H36O2  CAS:6929-04-0  MolWeight:284  RetIndex:1914
CompName:Hexadecanoic acid, 15-methyl-, methyl ester $$ Methyl isoheptadecanoate $$ Methyl 15-methylhexadecanoate $$

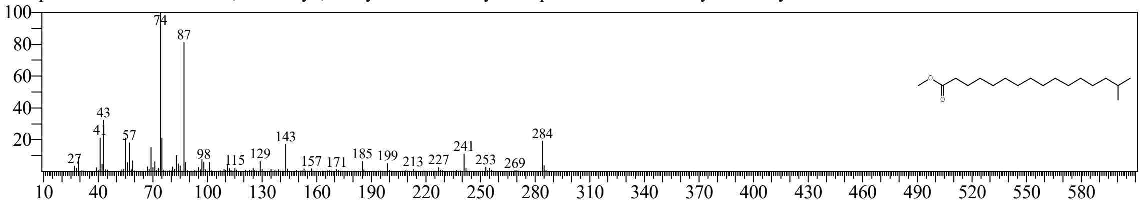


Hit#:4  Entry:27277  Library:NIST14s.lib
SI:92  Formula:C18H36O2  CAS:6929-04-0  MolWeight:284  RetIndex:1914
CompName:Hexadecanoic acid, 15-methyl-, methyl ester $$ Methyl isoheptadecanoate $$ Methyl 15-methylhexadecanoate $$

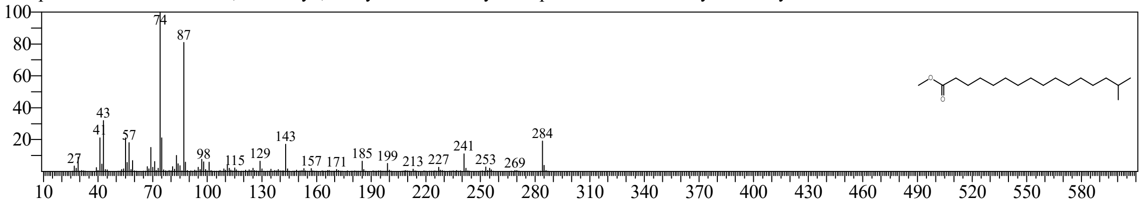


Hit#:5  Entry:31783  Library:NIST17s.lib
SI:92  Formula:C18H36O2  CAS:1731-92-6  MolWeight:284  RetIndex:1978
CompName:Heptadecanoic acid, methyl ester $$ Margaric acid methyl ester $$ Methyl heptadecanoate $$ Methyl margarate $$ n-Heptadecanoic acid methy

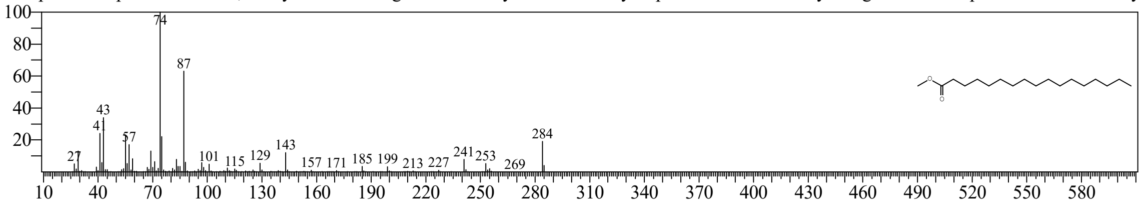

<< Target >>
Line#:6  R.Time:24.875(Scan#:1786)  MassPeaks:367
RawMode:Averaged 24.867-24.883(1785-1787) BasePeak:74.00(138281)
BG Mode:Calc. from Peak  Group 1 - Event 1

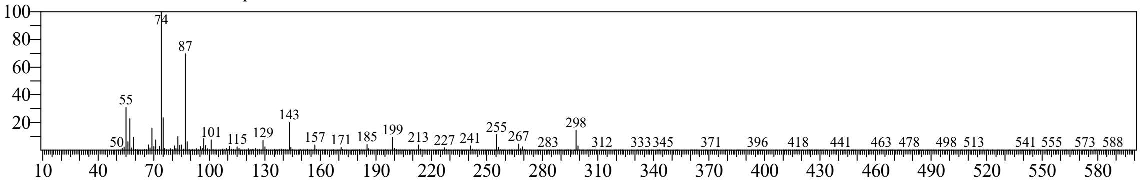


Hit#:1  Entry:129694  Library:NIST14.lib
SI:98  Formula:C19H38O2  CAS:112-61-8  MolWeight:298  RetIndex:2077
CompName:Methyl stearate $$ Octadecanoic acid, methyl ester $$ Stearic acid, methyl ester $$ n-Octadecanoic acid, methyl ester $$ Kemester 9718 $$ Me

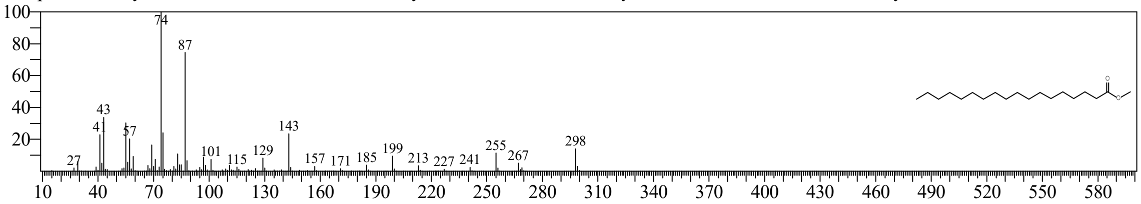


Hit#:2  Entry:141978  Library:NIST17-1.lib
SI:98  Formula:C19H38O2  CAS:112-61-8  MolWeight:298  RetIndex:2077
CompName:Methyl stearate $$ Octadecanoic acid, methyl ester $$ Stearic acid, methyl ester $$ n-Octadecanoic acid, methyl ester $$ Kemester 9718 $$ Me

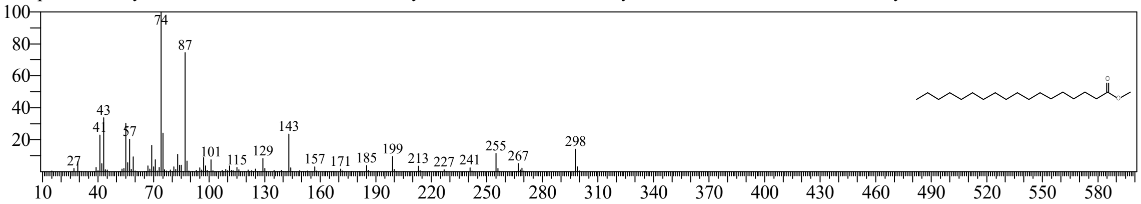


Hit#:3  Entry:28255  Library:NIST14s.lib
SI:97  Formula:C19H38O2  CAS:112-61-8  MolWeight:298  RetIndex:2077
CompName:Methyl stearate $$ Octadecanoic acid, methyl ester $$ Stearic acid, methyl ester $$ n-Octadecanoic acid, methyl ester $$ Kemester 9718 $$ Me

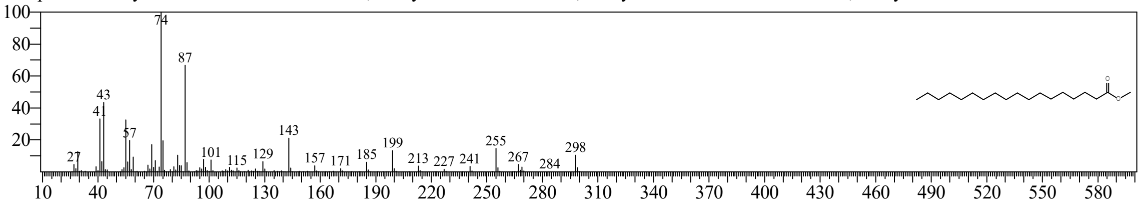


Hit#:4  Entry:32873  Library:NIST17s.lib
SI:97  Formula:C19H38O2  CAS:112-61-8  MolWeight:298  RetIndex:2077
CompName:Methyl stearate $$ Octadecanoic acid, methyl ester $$ Stearic acid, methyl ester $$ n-Octadecanoic acid, methyl ester $$ Kemester 9718 $$ Me

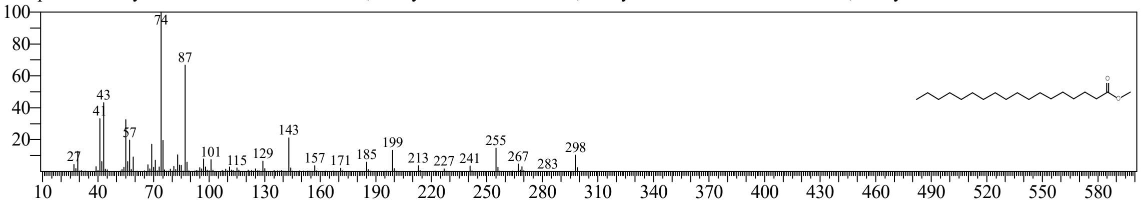


Hit#:5  Entry:28257  Library:NIST14s.lib
SI:97  Formula:C19H38O2  CAS:112-61-8  MolWeight:298  RetIndex:2077
CompName:Methyl stearate $$ Octadecanoic acid, methyl ester $$ Stearic acid, methyl ester $$ n-Octadecanoic acid, methyl ester $$ Kemester 9718 $$ Me

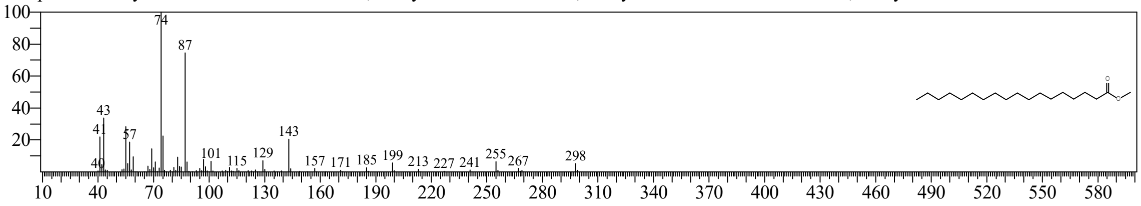

<< Target >>
Line#:7 R.Time:26.258(Scan#:1952) MassPeaks:406
RawMode:Averaged 26.250-26.267(1951-1953) BasePeak:55.05(252078)
BG Mode:Calc. from Peak Group 1 - Event 1

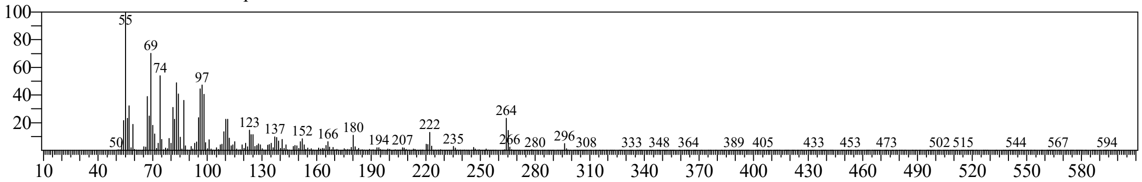


Hit#:1 Entry:32747 Library:NIST17s.lib
SI:97 Formula:C19H36O2 CAS:1937-62-8 MolWeight:296 RetIndex:2085
CompName:9-Octadecenoic acid, methyl ester, (E)- $$ Elaidic acid, methyl ester $$ Methyl elaidate $$ Methyl trans-9-octadecenoate $$ (E)-9-Octadecenoic

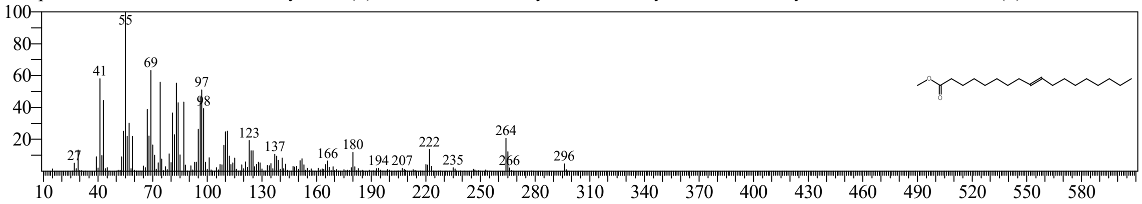


Hit#:2 Entry:28138 Library:NIST14s.lib
SI:97 Formula:C19H36O2 CAS:1937-62-8 MolWeight:296 RetIndex:2085
CompName:9-Octadecenoic acid, methyl ester, (E)- $$ Elaidic acid, methyl ester $$ Methyl elaidate $$ Methyl trans-9-octadecenoate $$ (E)-9-Octadecenoic

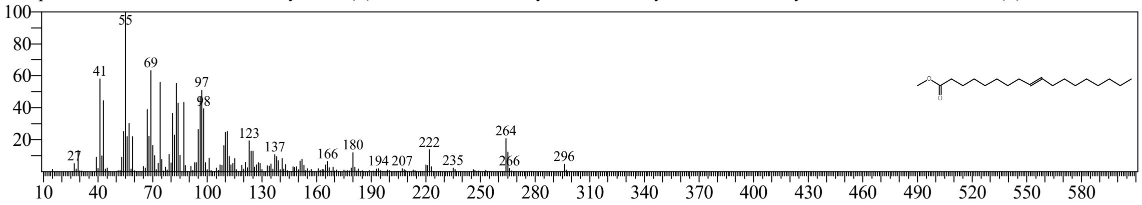


Hit#:3 Entry:127664 Library:NIST14.lib
SI:96 Formula:C19H36O2 CAS:112-62-9 MolWeight:296 RetIndex:2085
CompName:9-Octadecenoic acid (Z)-, methyl ester $$ Oleic acid, methyl ester $$ Emery oleic acid ester 2301 $$ Methyl cis-9-octadecenoate $$ Methyl ole

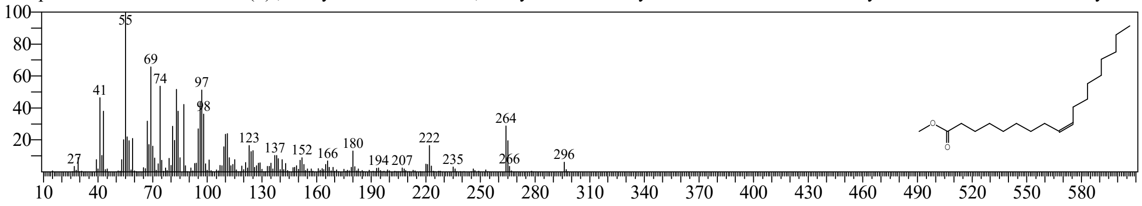


Hit#:4 Entry:139650 Library:NIST17-1.lib
SI:96 Formula:C19H36O2 CAS:112-62-9 MolWeight:296 RetIndex:2085
CompName:9-Octadecenoic acid (Z)-, methyl ester $$ Oleic acid, methyl ester $$ Emery oleic acid ester 2301 $$ Methyl cis-9-octadecenoate $$ Methyl ole

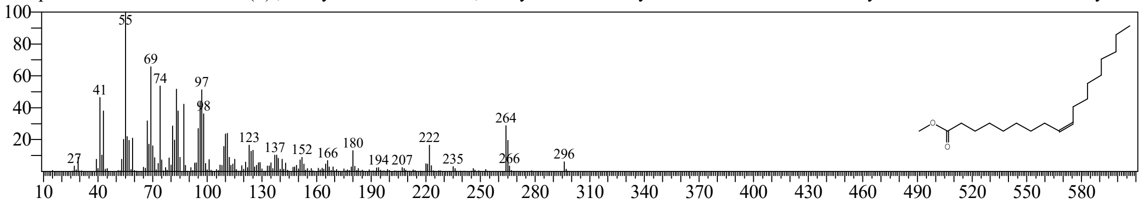


Hit#:5 Entry:28139 Library:NIST14s.lib
SI:96 Formula:C19H36O2 CAS:52380-33-3 MolWeight:296 RetIndex:2085
CompName:11-Octadecenoic acid, methyl ester $$ Methyl 11-octadecenoate $$ Octadec-11-enoic acid, methyl ester $$ Methyl (11E)-11-octadecenoate # $$

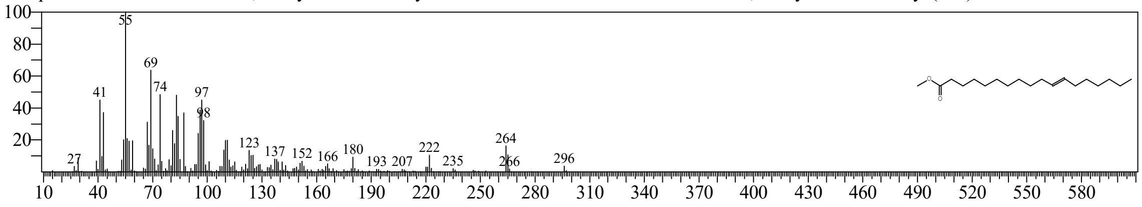

<< Target >>
Line#:8 R.Time:26.383(Scan#:1967) MassPeaks:360
RawMode:Averaged 26.375-26.392(1966-1968) BasePeak:55.05(17670)
BG Mode:Calc. from Peak Group 1 - Event 1

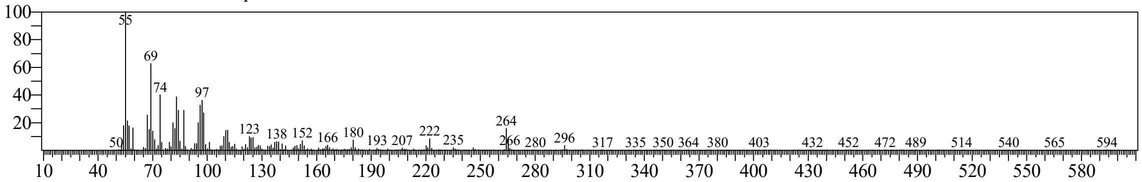


Hit#:1 Entry:32748 Library:NIST17s.lib
SI:95 Formula:C19H36O2 CAS:52380-33-3 MolWeight:296 RetIndex:2085
CompName:11-Octadecenoic acid, methyl ester $$ Methyl 11-octadecenoate $$ Octadec-11-enoic acid, methyl ester $$ Methyl (11E)-11-octadecenoate # $$

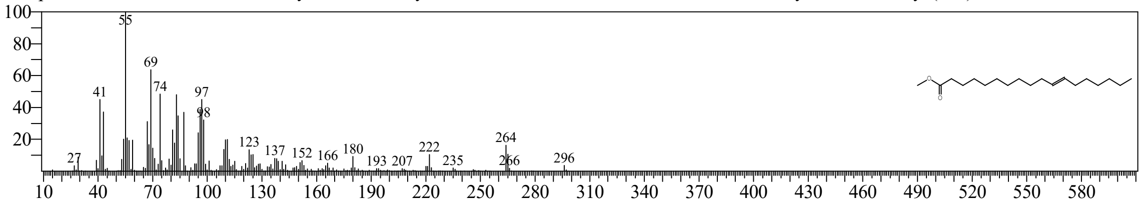


Hit#:2 Entry:28139 Library:NIST14s.lib
SI:95 Formula:C19H36O2 CAS:52380-33-3 MolWeight:296 RetIndex:2085
CompName:11-Octadecenoic acid, methyl ester $$ Methyl 11-octadecenoate $$ Octadec-11-enoic acid, methyl ester $$ Methyl (11E)-11-octadecenoate # $$

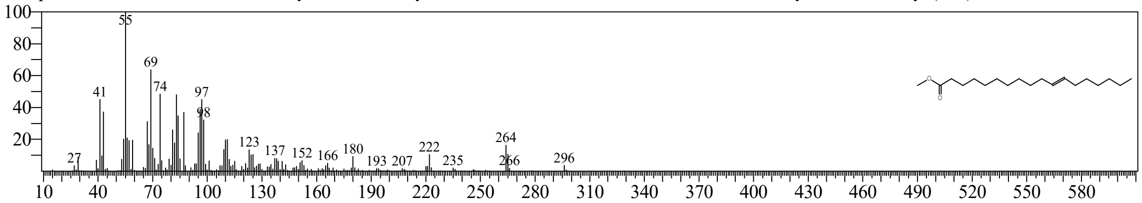


Hit#:3 Entry:127661 Library:NIST14.lib
SI:95 Formula:C19H36O2 CAS:0-00-0 MolWeight:296 RetIndex:2085
CompName:cis-13-Octadecenoic acid, methyl ester

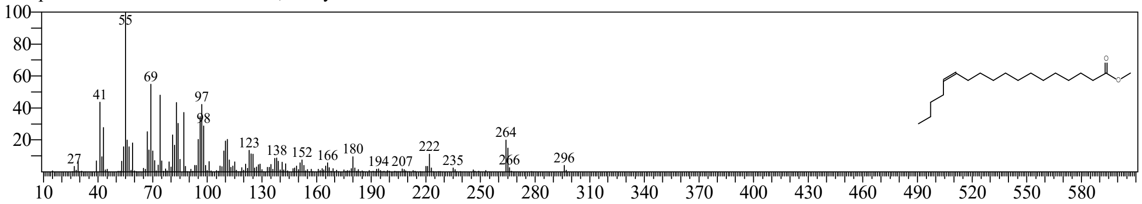


Hit#:4 Entry:139647 Library:NIST17-1.lib
SI:95 Formula:C19H36O2 CAS:0-00-0 MolWeight:296 RetIndex:2085
CompName:cis-13-Octadecenoic acid, methyl ester

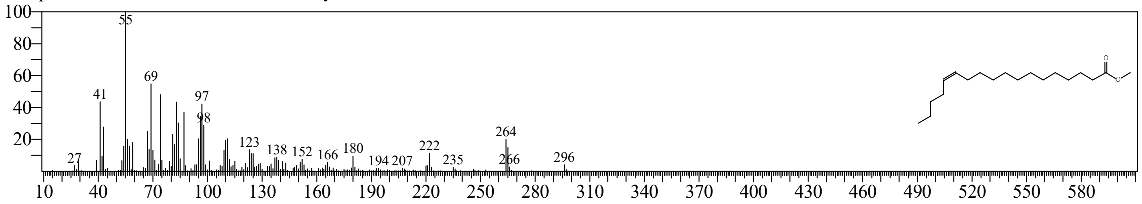


Hit#:5 Entry:127658 Library:NIST14.lib
SI:94 Formula:C19H36O2 CAS:0-00-0 MolWeight:296 RetIndex:2085
CompName:trans-13-Octadecenoic acid, methyl ester

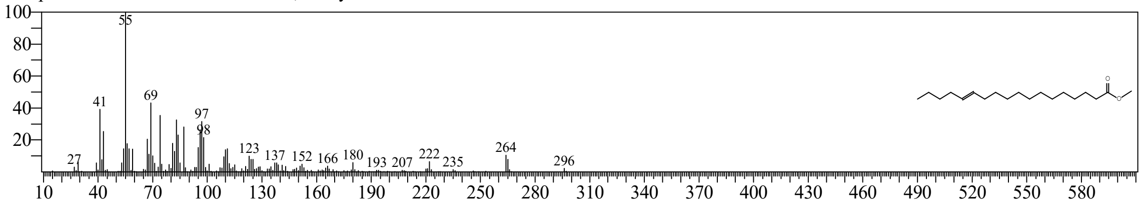

<< Target >>
Line#:9 R.Time:27.742(Scan#:2130) MassPeaks:349
RawMode:Averaged 27.733-27.750(2129-2131) BasePeak:67.05(3465)
BG Mode:Calc. from Peak Group 1 - Event 1

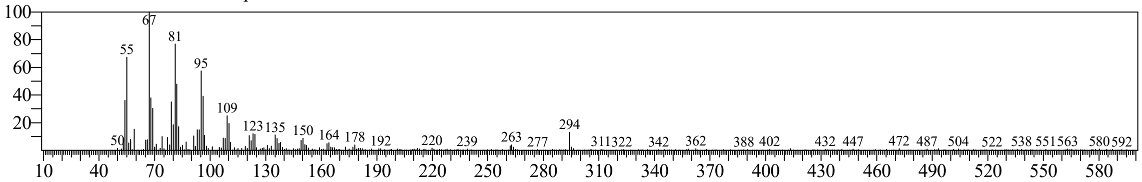


Hit#:1 Entry:137663 Library:NIST17-1.lib
SI:96 Formula:C19H34O2 CAS:2462-85-3 MolWeight:294 RetIndex:2093
CompName:9,12-Octadecadienoic acid, methyl ester $$ 9,12-Octadecenoic acid, methyl ester $$ Methyl octadeca-9,12-dienoate $$ Methyl 9,12-octadecadi

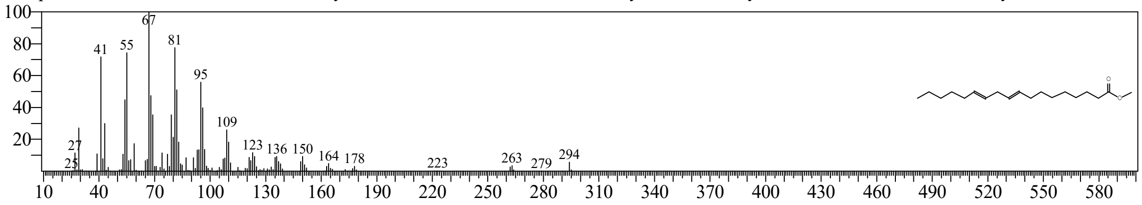


Hit#:2 Entry:125931 Library:NIST14.lib
SI:96 Formula:C19H34O2 CAS:2462-85-3 MolWeight:294 RetIndex:2093
CompName:9,12-Octadecadienoic acid, methyl ester $$ 9,12-Octadecenoic acid, methyl ester $$ Methyl octadeca-9,12-dienoate $$ Methyl 9,12-octadecadi

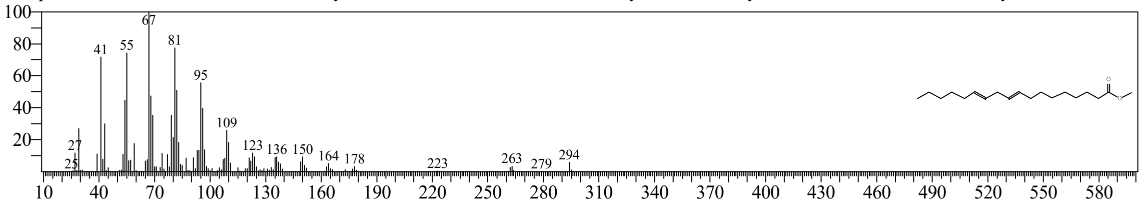


Hit#:3 Entry:32588 Library:NIST17s.lib
SI:96 Formula:C19H34O2 CAS:112-63-0 MolWeight:294 RetIndex:2093
CompName:9,12-Octadecadienoic acid (Z,Z)-, methyl ester $$ Linoleic acid, methyl ester $$ Methyl cis,cis-9,12-octadecadienoate $$ Methyl linoleate $$ M

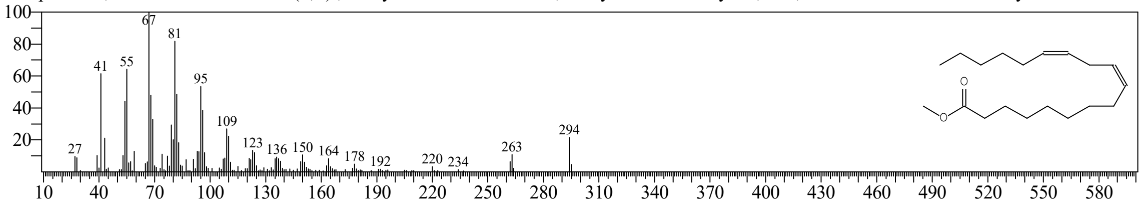


Hit#:4 Entry:27999 Library:NIST14s.lib
SI:96 Formula:C19H34O2 CAS:112-63-0 MolWeight:294 RetIndex:2093
CompName:9,12-Octadecadienoic acid (Z,Z)-, methyl ester $$ Linoleic acid, methyl ester $$ Methyl cis,cis-9,12-octadecadienoate $$ Methyl linoleate $$ M

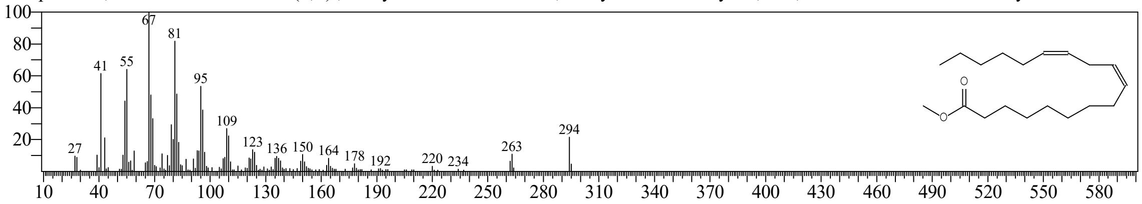


Hit#:5 Entry:32589 Library:NIST17s.lib
SI:95 Formula:C19H34O2 CAS:112-63-0 MolWeight:294 RetIndex:2093
CompName:9,12-Octadecadienoic acid (Z,Z)-, methyl ester $$ Linoleic acid, methyl ester $$ Methyl cis,cis-9,12-octadecadienoate $$ Methyl linoleate $$ M

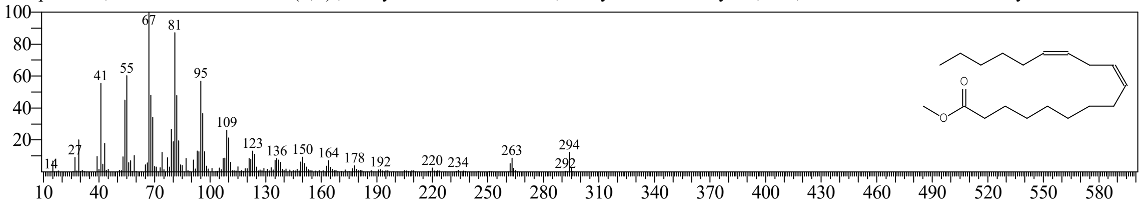

<< Target >>
Line#:10 R.Time:27.950(Scan#:2155) MassPeaks:357
RawMode:Averaged 27.942-27.958(2154-2156) BasePeak:67.05(2314)
BG Mode:Calc. from Peak Group 1 - Event 1

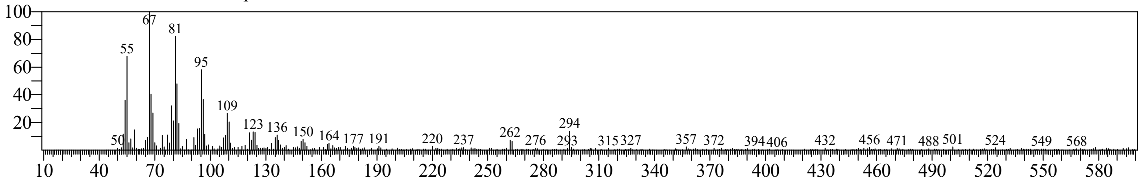


Hit#:1 Entry:32588 Library:NIST17s.lib
SI:96 Formula:C19H34O2 CAS:112-63-0 MolWeight:294 RetIndex:2093
CompName:9,12-Octadecadienoic acid (Z,Z)-, methyl ester $$ Linoleic acid, methyl ester $$ Methyl cis,cis-9,12-octadecadienoate $$ Methyl linoleate $$ M

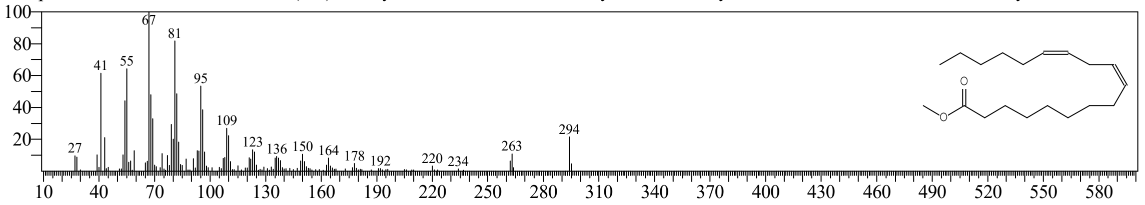


Hit#:2 Entry:27999 Library:NIST14s.lib
SI:96 Formula:C19H34O2 CAS:112-63-0 MolWeight:294 RetIndex:2093
CompName:9,12-Octadecadienoic acid (Z,Z)-, methyl ester $$ Linoleic acid, methyl ester $$ Methyl cis,cis-9,12-octadecadienoate $$ Methyl linoleate $$ M

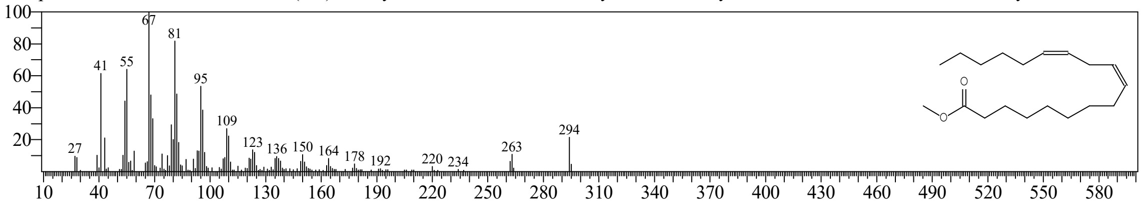


Hit#:3 Entry:32589 Library:NIST17s.lib
SI:96 Formula:C19H34O2 CAS:112-63-0 MolWeight:294 RetIndex:2093
CompName:9,12-Octadecadienoic acid (Z,Z)-, methyl ester $$ Linoleic acid, methyl ester $$ Methyl cis,cis-9,12-octadecadienoate $$ Methyl linoleate $$ M

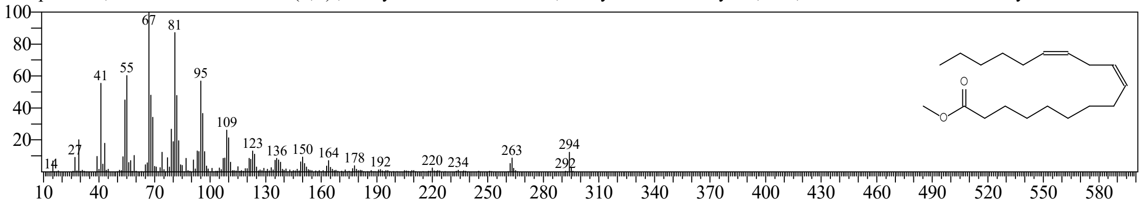


Hit#:4 Entry:28000 Library:NIST14s.lib
SI:96 Formula:C19H34O2 CAS:112-63-0 MolWeight:294 RetIndex:2093
CompName:9,12-Octadecadienoic acid (Z,Z)-, methyl ester $$ Linoleic acid, methyl ester $$ Methyl cis,cis-9,12-octadecadienoate $$ Methyl linoleate $$ M

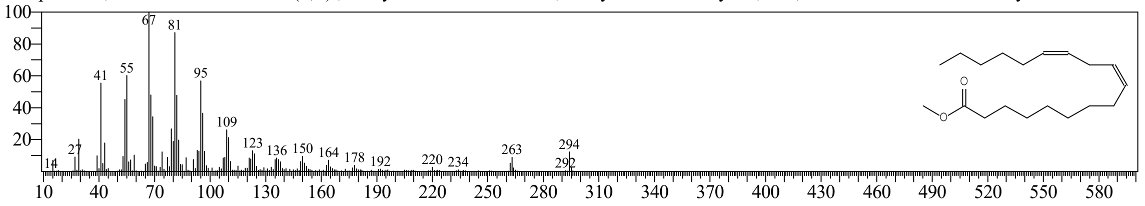


Hit#:5 Entry:125931 Library:NIST14.lib
SI:95 Formula:C19H34O2 CAS:2462-85-3 MolWeight:294 RetIndex:2093
CompName:9,12-Octadecadienoic acid, methyl ester $$ 9,12-Octadecenoic acid, methyl ester $$ Methyl octadeca-9,12-dienoate $$ Methyl 9,12-octadecadi

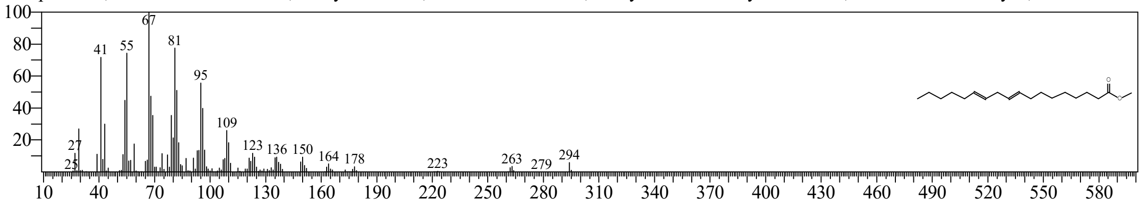

<< Target >>
Line#:11 R.Time:28.375(Scan#:2206) MassPeaks:393
RawMode:Averaged 28.367-28.383(2205-2207) BasePeak:67.05(474184)
BG Mode:Calc. from Peak Group 1 - Event 1

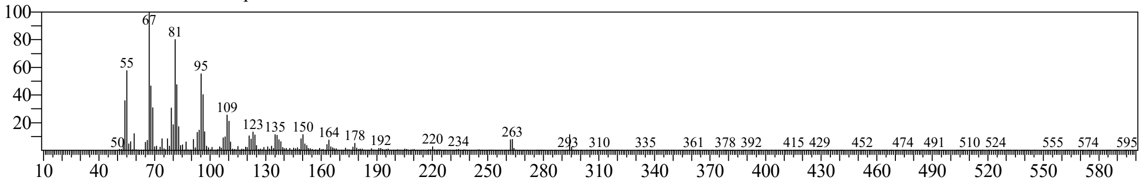

Hit#:1 Entry:32588 Library:NIST17s.lib
SI:97 Formula:C19H34O2 CAS:112-63-0 MolWeight:294 RetIndex:2093
CompName:9,12-Octadecadienoic acid (Z,Z)-, methyl ester $$ Linoleic acid, methyl ester $$ Methyl cis,cis-9,12-octadecadienoate $$ Methyl linoleate $$ M

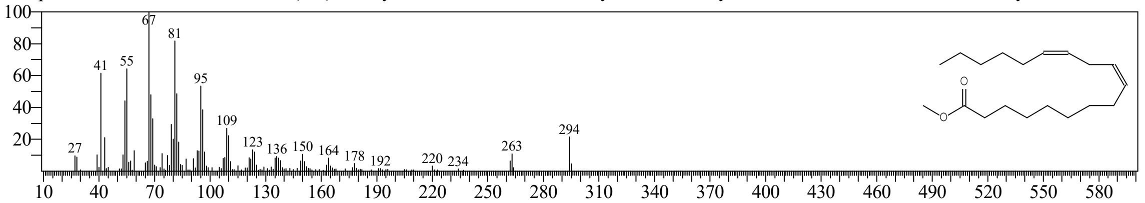

Hit#:2 Entry:27999 Library:NIST14s.lib
SI:97 Formula:C19H34O2 CAS:112-63-0 MolWeight:294 RetIndex:2093
CompName:9,12-Octadecadienoic acid (Z,Z)-, methyl ester $$ Linoleic acid, methyl ester $$ Methyl cis,cis-9,12-octadecadienoate $$ Methyl linoleate $$ M

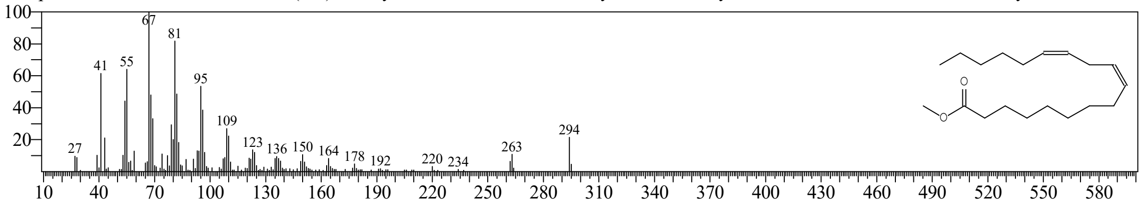

Hit#:3 Entry:32589 Library:NIST17s.lib
SI:97 Formula:C19H34O2 CAS:112-63-0 MolWeight:294 RetIndex:2093
CompName:9,12-Octadecadienoic acid (Z,Z)-, methyl ester $$ Linoleic acid, methyl ester $$ Methyl cis,cis-9,12-octadecadienoate $$ Methyl linoleate $$ M

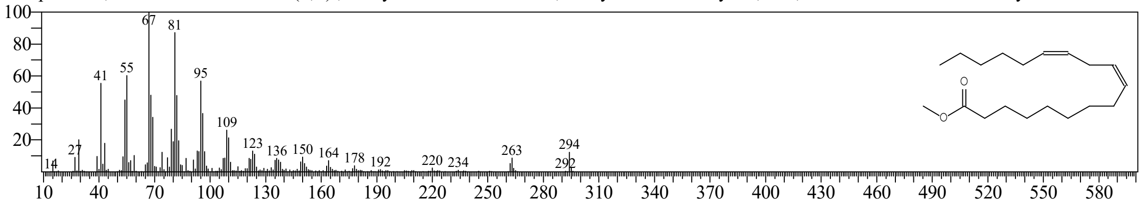

Hit#:4 Entry:28000 Library:NIST14s.lib
SI:97 Formula:C19H34O2 CAS:112-63-0 MolWeight:294 RetIndex:2093
CompName:9,12-Octadecadienoic acid (Z,Z)-, methyl ester $$ Linoleic acid, methyl ester $$ Methyl cis,cis-9,12-octadecadienoate $$ Methyl linoleate $$ M

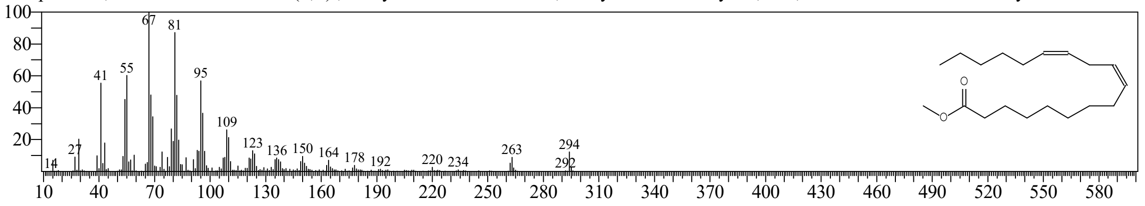

Hit#:5 Entry:137663 Library:NIST17-1.lib
SI:95 Formula:C19H34O2 CAS:2462-85-3 MolWeight:294 RetIndex:2093
CompName:9,12-Octadecadienoic acid, methyl ester $$ 9,12-Octadecenoic acid, methyl ester $$ Methyl octadeca-9,12-dienoate $$ Methyl 9,12-octadecadi

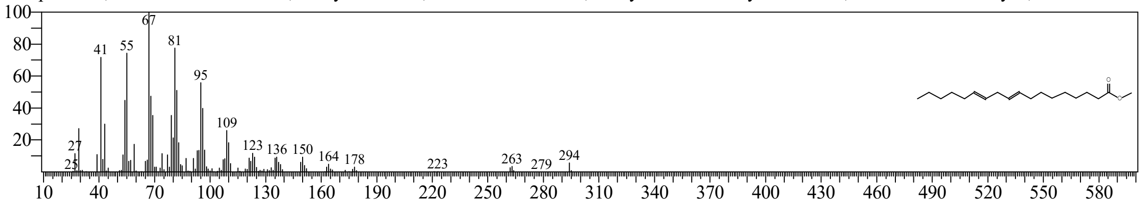

<< Target >>
Line#:12 R.Time:29.267(Scan#:2313) MassPeaks:306
RawMode:Averaged 29.258-29.275(2312-2314) BasePeak:74.00(14099)
BG Mode:Calc. from Peak Group 1 - Event 1

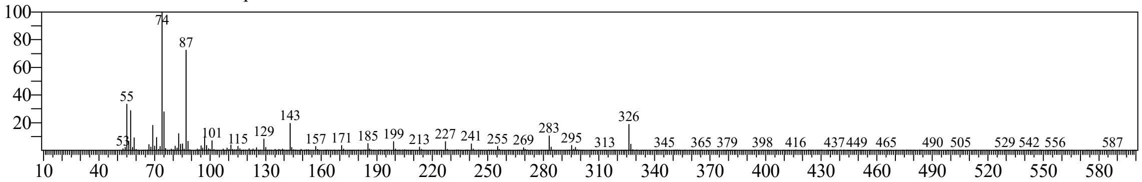


Hit#:1 Entry:154704 Library:NIST14.lib
SI:97 Formula:C21H42O2 CAS:0-00-0 MolWeight:326 RetIndex:2212
CompName:Methyl 18-methylnonadecanoate

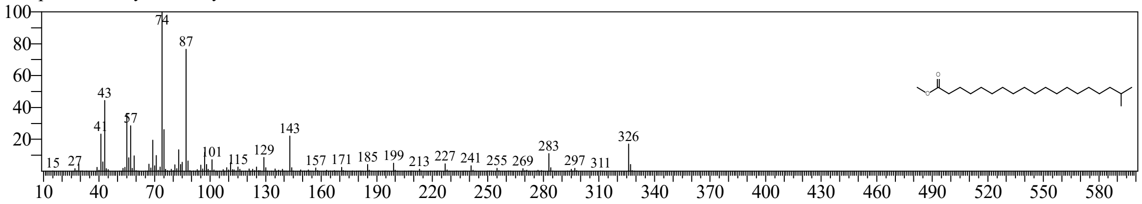


Hit#:2 Entry:170238 Library:NIST17-1.lib
SI:97 Formula:C21H42O2 CAS:0-00-0 MolWeight:326 RetIndex:2212
CompName:Methyl 18-methylnonadecanoate

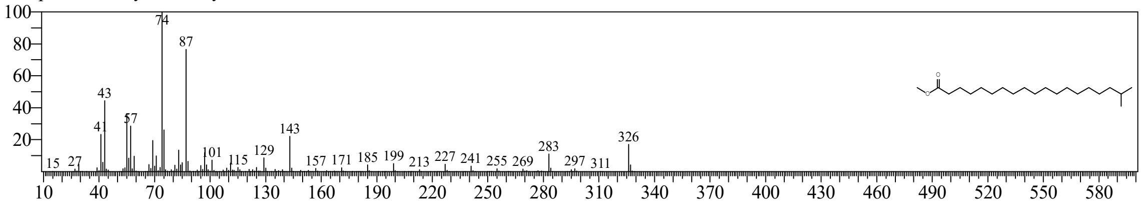


Hit#:3 Entry:34726 Library:NIST17s.lib
SI:96 Formula:C21H42O2 CAS:1120-28-1 MolWeight:326 RetIndex:2276
CompName:Eicosanoic acid, methyl ester $$ Methyl arachisate $$ Methyl eicosanoate $$ Arachidic acid methyl ester $$ Kemester 2050 $$ Methyl aracidat

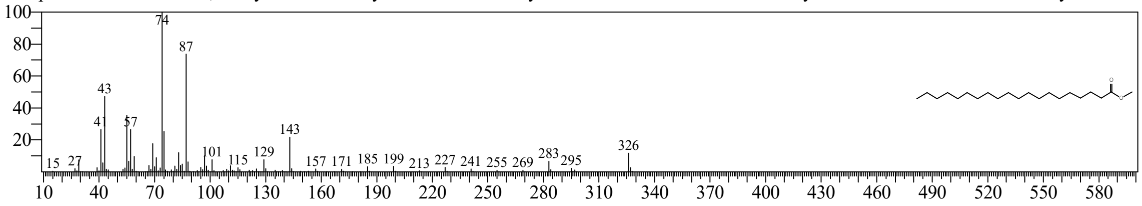


Hit#:4 Entry:34724 Library:NIST17s.lib
SI:94 Formula:C21H42O2 CAS:1120-28-1 MolWeight:326 RetIndex:2276
CompName:Eicosanoic acid, methyl ester $$ Methyl arachisate $$ Methyl eicosanoate $$ Arachidic acid methyl ester $$ Kemester 2050 $$ Methyl aracidat

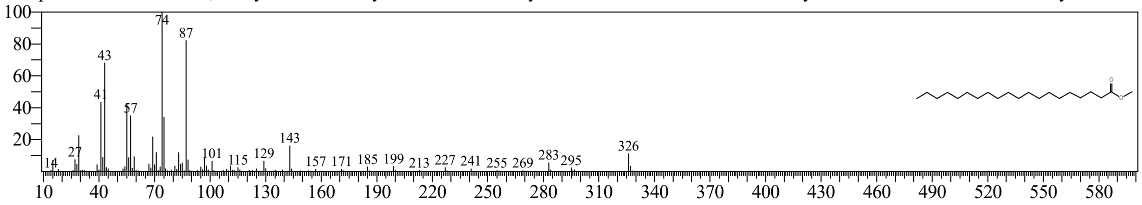


Hit#:5 Entry:29931 Library:NIST14s.lib
SI:94 Formula:C21H42O2 CAS:1120-28-1 MolWeight:326 RetIndex:2276
CompName:Eicosanoic acid, methyl ester $$ Methyl arachisate $$ Methyl eicosanoate $$ Arachidic acid methyl ester $$ Kemester 2050 $$ Methyl aracidat

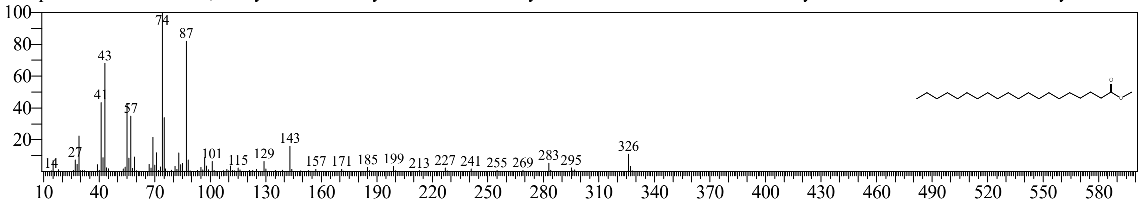

<< Target >>
Line#:13 R.Time:29.825(Scan#:2380) MassPeaks:335
RawMode:Averaged 29.817-29.833(2379-2381) BasePeak:79.05(8210)
BG Mode:Calc. from Peak Group 1 - Event 1

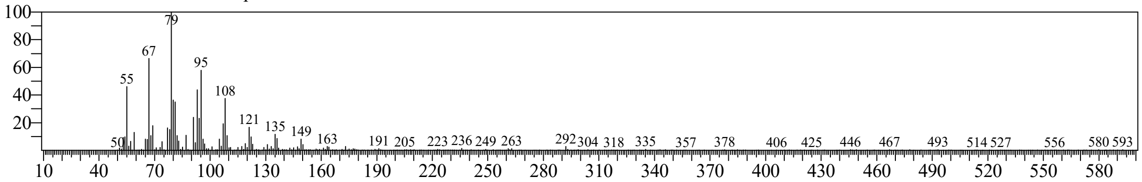


Hit#:1 Entry:135724 Library:NIST17-1.lib
SI:95 Formula:C19H32O2 CAS:301-00-8 MolWeight:292 RetIndex:2101
CompName:9,12,15-Octadecatrienoic acid, methyl ester, (Z,Z,Z)- $$ Linolenic acid, methyl ester $$ Methyl all-cis-9,12,15-octadecatrienoate $$ Methyl lino

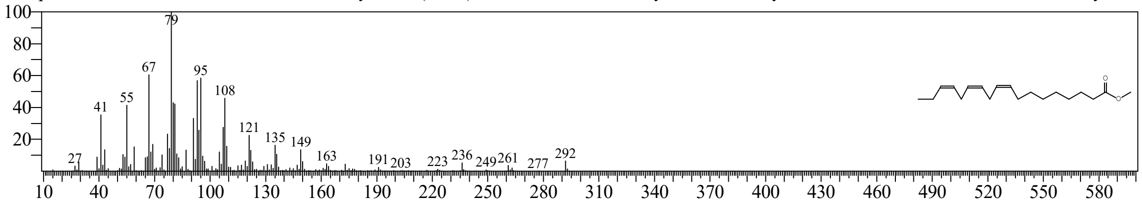


Hit#:2 Entry:124228 Library:NIST14.lib
SI:95 Formula:C19H32O2 CAS:301-00-8 MolWeight:292 RetIndex:2101
CompName:9,12,15-Octadecatrienoic acid, methyl ester, (Z,Z,Z)- $$ Linolenic acid, methyl ester $$ Methyl all-cis-9,12,15-octadecatrienoate $$ Methyl lino

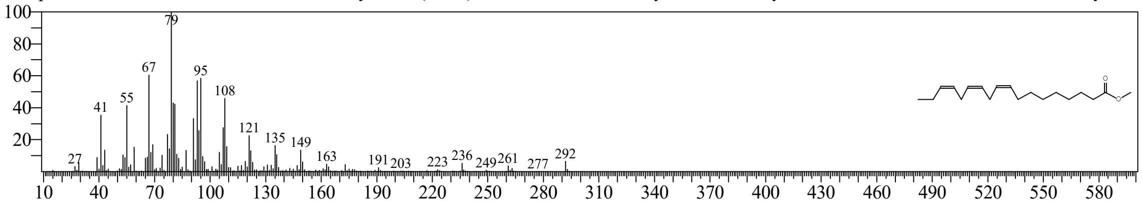


Hit#:3 Entry:32438 Library:NIST17s.lib
SI:94 Formula:C19H32O2 CAS:301-00-8 MolWeight:292 RetIndex:2101
CompName:9,12,15-Octadecatrienoic acid, methyl ester, (Z,Z,Z)- $$ Linolenic acid, methyl ester $$ Methyl all-cis-9,12,15-octadecatrienoate $$ Methyl lino

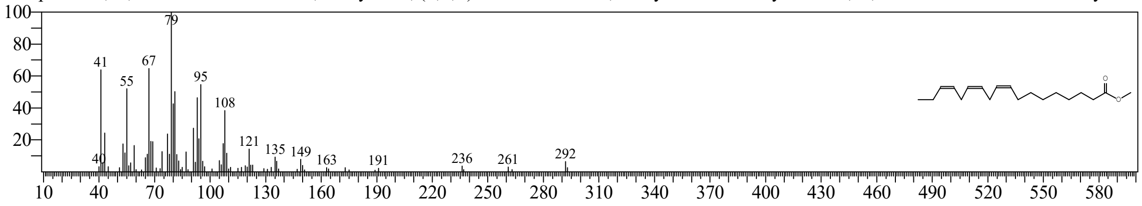


Hit#:4 Entry:29609 Library:NIST14s.lib
SI:94 Formula:C21H36O2 CAS:55682-88-7 MolWeight:320 RetIndex:2300
CompName:11,14,17-Eicosatrienoic acid, methyl ester $$ Methyl 11,14,17-icosatrienoate $$ Methyl 11,14,17-eicosatrienoate $$

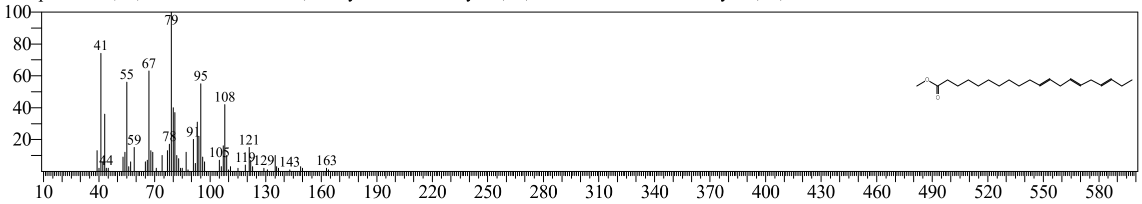


Hit#:5 Entry:34362 Library:NIST17s.lib
SI:94 Formula:C21H36O2 CAS:55682-88-7 MolWeight:320 RetIndex:2300
CompName:11,14,17-Eicosatrienoic acid, methyl ester $$ Methyl 11,14,17-icosatrienoate $$ Methyl 11,14,17-eicosatrienoate $$

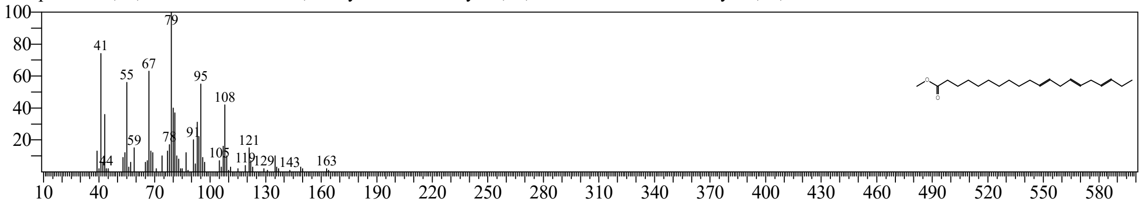

<< Target >>
Line#:14 R.Time:30.300(Scan#:2437) MassPeaks:310
RawMode:Averaged 30.292-30.308(2436-2438) BasePeak:79.05(6078)
BG Mode:Calc. from Peak Group 1 - Event 1

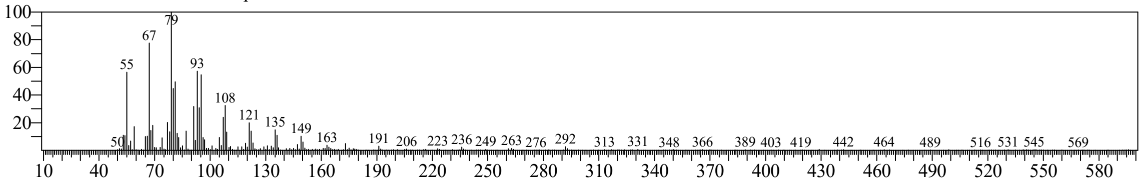


Hit#:1 Entry:124228 Library:NIST14.lib
SI:95 Formula:C19H32O2 CAS:301-00-8 MolWeight:292 RetIndex:2101
CompName:9,12,15-Octadecatrienoic acid, methyl ester, (Z,Z,Z)- $$ Linolenic acid, methyl ester $$ Methyl all-cis-9,12,15-octadecatrienoate $$ Methyl lino

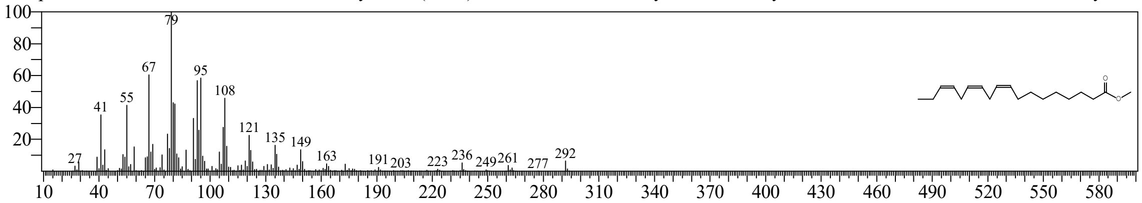


Hit#:2 Entry:135724 Library:NIST17-1.lib
SI:95 Formula:C19H32O2 CAS:301-00-8 MolWeight:292 RetIndex:2101
CompName:9,12,15-Octadecatrienoic acid, methyl ester, (Z,Z,Z)- $$ Linolenic acid, methyl ester $$ Methyl all-cis-9,12,15-octadecatrienoate $$ Methyl lino

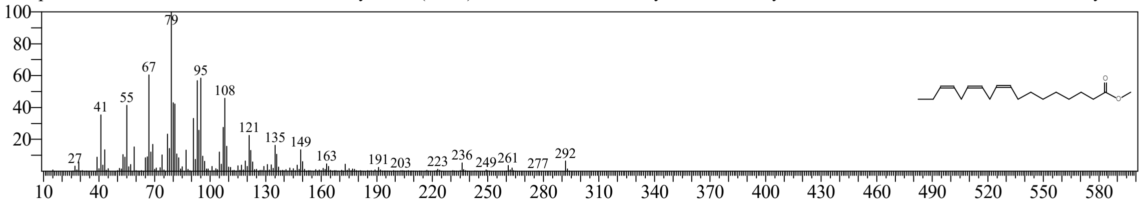


Hit#:3 Entry:32437 Library:NIST17s.lib
SI:94 Formula:C19H32O2 CAS:301-00-8 MolWeight:292 RetIndex:2101
CompName:9,12,15-Octadecatrienoic acid, methyl ester, (Z,Z,Z)- $$ Linolenic acid, methyl ester $$ Methyl all-cis-9,12,15-octadecatrienoate $$ Methyl lino

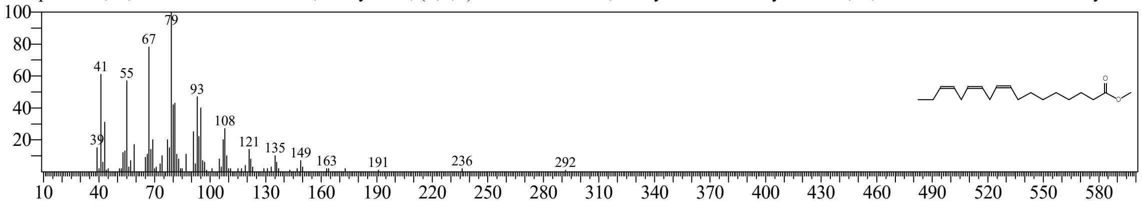


Hit#:4 Entry:111654 Library:NIST14.lib
SI:94 Formula:C18H30O2 CAS:0-00-0 MolWeight:278 RetIndex:2002
CompName:Methyl 8,11,14-heptadecatrienoate

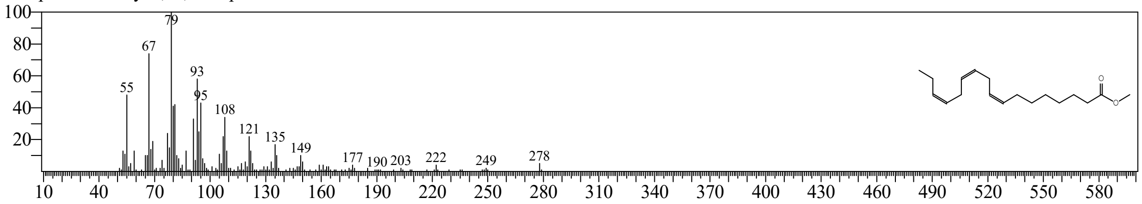


Hit#:5 Entry:27872 Library:NIST14s.lib
SI:94 Formula:C19H32O2 CAS:301-00-8 MolWeight:292 RetIndex:2101
CompName:9,12,15-Octadecatrienoic acid, methyl ester, (Z,Z,Z)- $$ Linolenic acid, methyl ester $$ Methyl all-cis-9,12,15-octadecatrienoate $$ Methyl lino

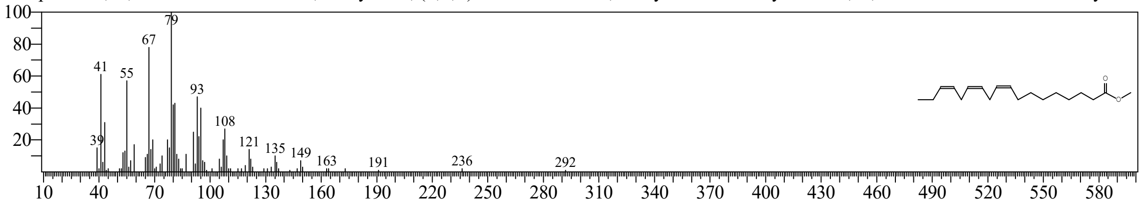

<< Target >>
Line#:15 R.Time:30.600(Scan#:2473) MassPeaks:387
RawMode:Averaged 30.592-30.608(2472-2474) BasePeak:79.05(72002)
BG Mode:Calc. from Peak Group 1 - Event 1

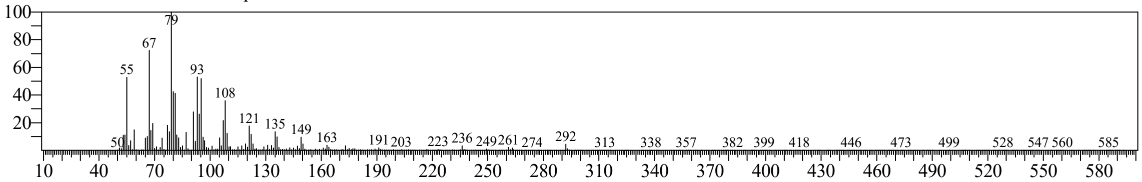


Hit#:1 Entry:135724 Library:NIST17-1.lib
SI:96 Formula:C19H32O2 CAS:301-00-8 MolWeight:292 RetIndex:2101
CompName:9,12,15-Octadecatrienoic acid, methyl ester, (Z,Z,Z)- $$ Linolenic acid, methyl ester $$ Methyl all-cis-9,12,15-octadecatrienoate $$ Methyl lino

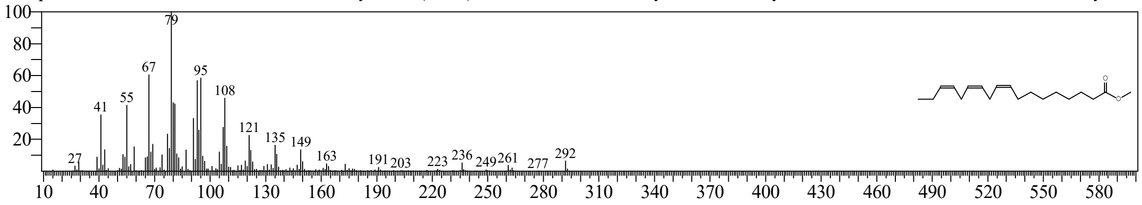


Hit#:2 Entry:124228 Library:NIST14.lib
SI:96 Formula:C19H32O2 CAS:301-00-8 MolWeight:292 RetIndex:2101
CompName:9,12,15-Octadecatrienoic acid, methyl ester, (Z,Z,Z)- $$ Linolenic acid, methyl ester $$ Methyl all-cis-9,12,15-octadecatrienoate $$ Methyl lino

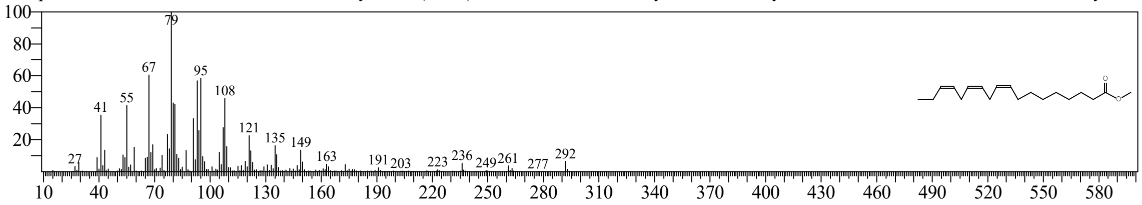


Hit#:3 Entry:32438 Library:NIST17s.lib
SI:95 Formula:C19H32O2 CAS:301-00-8 MolWeight:292 RetIndex:2101
CompName:9,12,15-Octadecatrienoic acid, methyl ester, (Z,Z,Z)- $$ Linolenic acid, methyl ester $$ Methyl all-cis-9,12,15-octadecatrienoate $$ Methyl lino

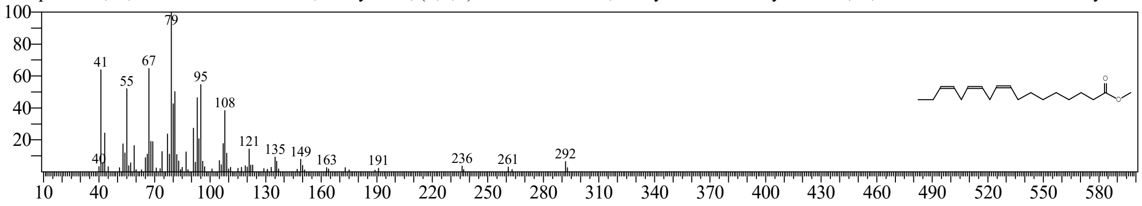


Hit#:4 Entry:27872 Library:NIST14s.lib
SI:95 Formula:C19H32O2 CAS:301-00-8 MolWeight:292 RetIndex:2101
CompName:9,12,15-Octadecatrienoic acid, methyl ester, (Z,Z,Z)- $$ Linolenic acid, methyl ester $$ Methyl all-cis-9,12,15-octadecatrienoate $$ Methyl lino

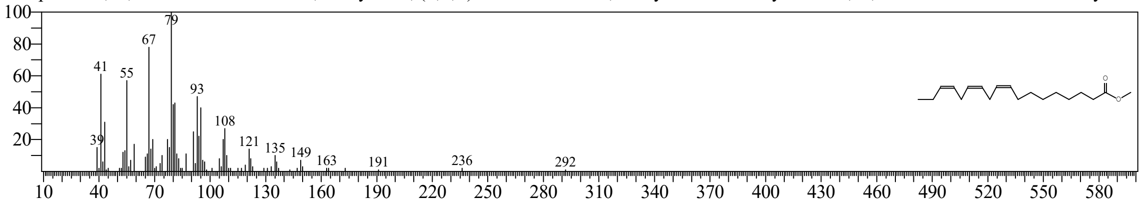


Hit#:5 Entry:121536 Library:NIST17-1.lib
SI:95 Formula:C18H30O2 CAS:0-00-0 MolWeight:278 RetIndex:2002
CompName:Methyl 8,11,14-heptadecatrienoate

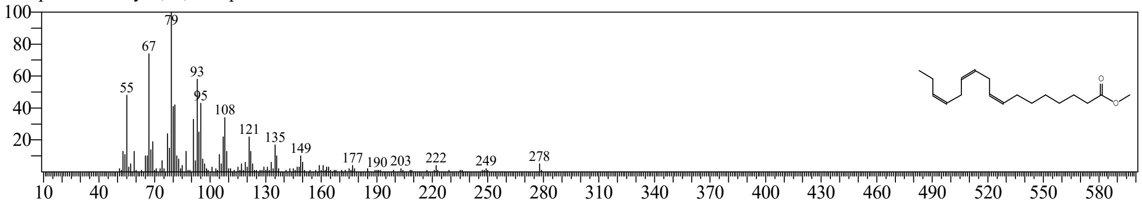

<< Target >>
Line#:16 R.Time:30.925(Scan#:2512) MassPeaks:328
RawMode:Averaged 30.917-30.933(2511-2513) BasePeak:67.05(5471)
BG Mode:Calc. from Peak Group 1 - Event 1

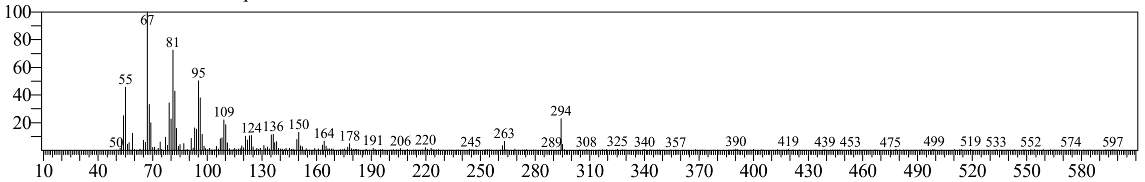


Hit#:1 Entry:125933 Library:NIST14.lib
SI:97 Formula:C19H34O2 CAS:0-00-0 MolWeight:294 RetIndex:2093
CompName:Methyl 10-trans,12-cis-octadecadienoate

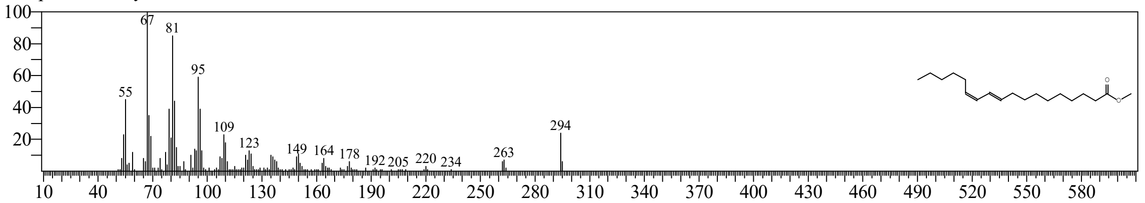


Hit#:2 Entry:125932 Library:NIST14.lib
SI:97 Formula:C19H34O2 CAS:0-00-0 MolWeight:294 RetIndex:2093
CompName:Methyl 9-cis,11-trans-octadecadienoate

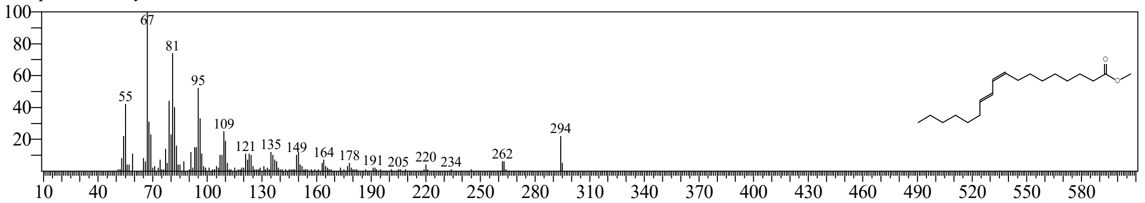


Hit#:3 Entry:137664 Library:NIST17-1.lib
SI:97 Formula:C19H34O2 CAS:0-00-0 MolWeight:294 RetIndex:2093
CompName:Methyl 10-trans,12-cis-octadecadienoate

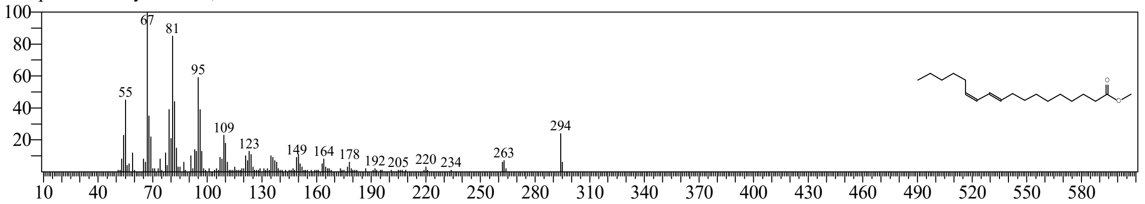


Hit#:4 Entry:137666 Library:NIST17-1.lib
SI:97 Formula:C19H34O2 CAS:13058-52-1 MolWeight:294 RetIndex:2093
CompName:Methyl 9-cis,11-trans-octadecadienoate

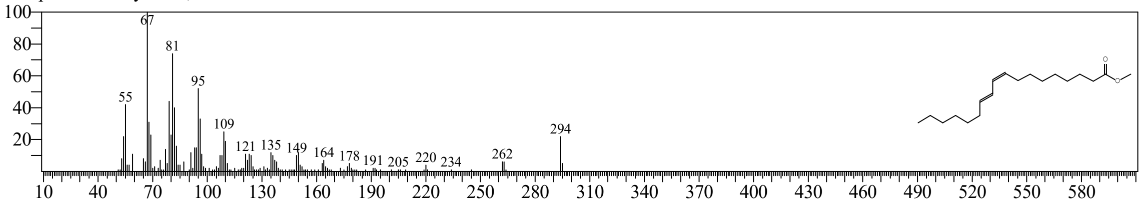


Hit#:5 Entry:32587 Library:NIST17s.lib
SI:94 Formula:C19H34O2 CAS:13038-47-6 MolWeight:294 RetIndex:2093
CompName:9,11-Octadecadienoic acid, methyl ester, (E,E)- $$ Methyl trans-9,trans-11-octadecadienoate $$ Methyl trans,trans-9,11-octadecadienoate $$ M

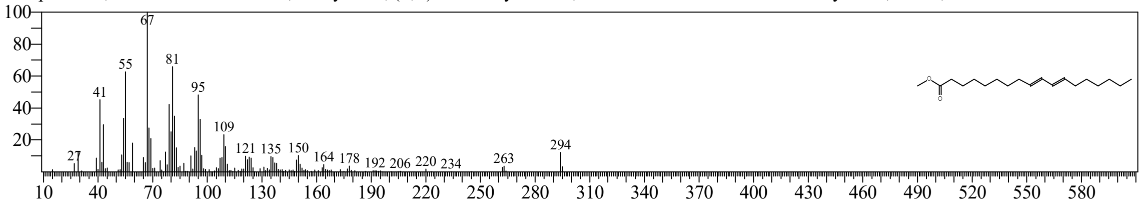

<< Target >>
Line#:17 R.Time:31.267(Scan#:2553) MassPeaks:345
RawMode:Averaged 31.258-31.275(2552-2554) BasePeak:67.05(6142)
BG Mode:Calc. from Peak Group 1 - Event 1

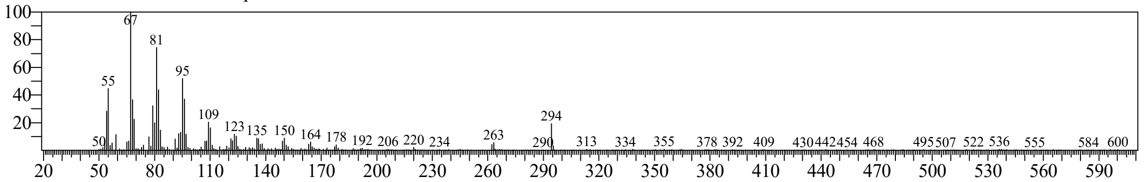


Hit#:1 Entry:125933 Library:NIST14.lib
SI:96 Formula:C19H34O2 CAS:0-00-0 MolWeight:294 RetIndex:2093
CompName:Methyl 10-trans,12-cis-octadecadienoate

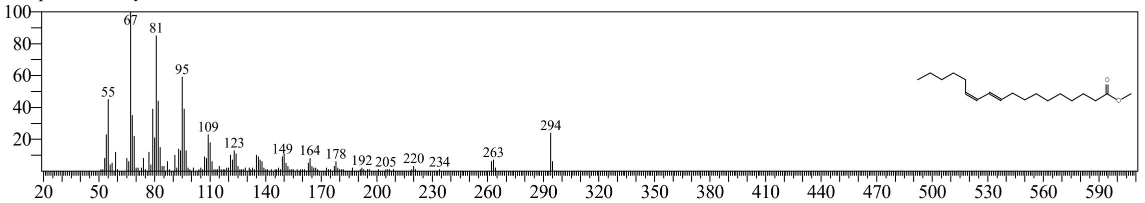


Hit#:2 Entry:137664 Library:NIST17-1.lib
SI:96 Formula:C19H34O2 CAS:0-00-0 MolWeight:294 RetIndex:2093
CompName:Methyl 10-trans,12-cis-octadecadienoate

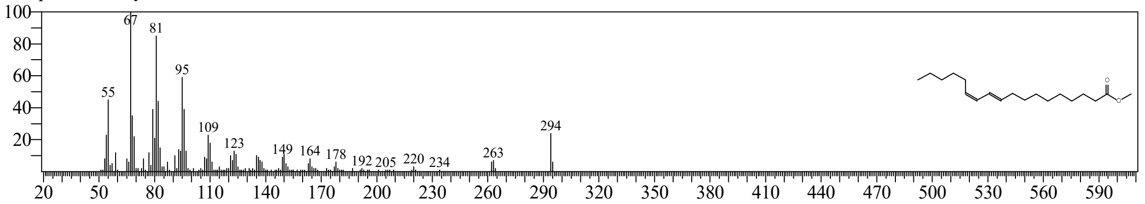


Hit#:3 Entry:125932 Library:NIST14.lib
SI:96 Formula:C19H34O2 CAS:0-00-0 MolWeight:294 RetIndex:2093
CompName:Methyl 9-cis,11-trans-octadecadienoate

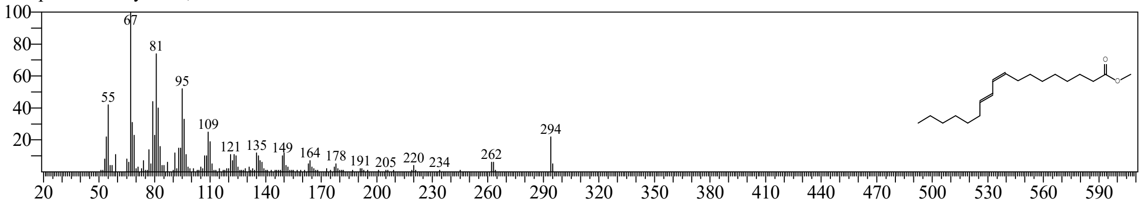


Hit#:4 Entry:137666 Library:NIST17-1.lib
SI:96 Formula:C19H34O2 CAS:13058-52-1 MolWeight:294 RetIndex:2093
CompName:Methyl 9-cis,11-trans-octadecadienoate

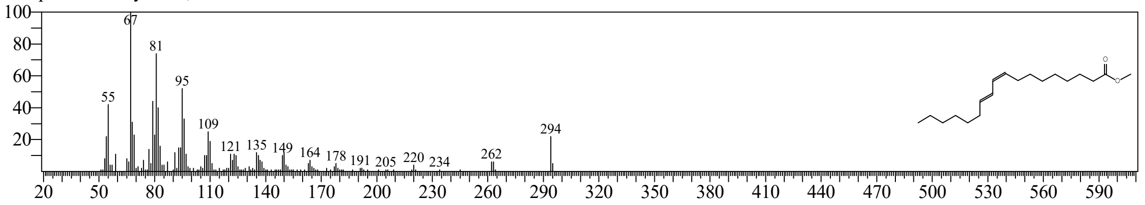


Hit#:5 Entry:32588 Library:NIST17s.lib
SI:94 Formula:C19H34O2 CAS:112-63-0 MolWeight:294 RetIndex:2093
CompName:9,12-Octadecadienoic acid (Z,Z)-, methyl ester $$ Linoleic acid, methyl ester $$ Methyl cis,cis-9,12-octadecadienoate $$ Methyl linoleate $$ M

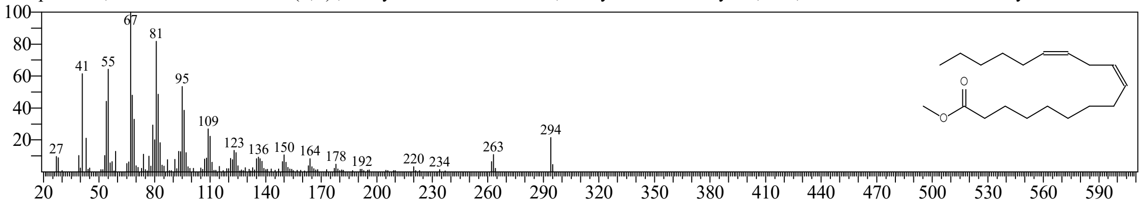

<< Target >>
Line#:18   R.Time:31.925(Scan#:2632)  MassPeaks:311
RawMode:Averaged 31.917-31.933(2631-2633) BasePeak:67.05(905)
BG Mode:Calc. from Peak  Group 1 - Event 1

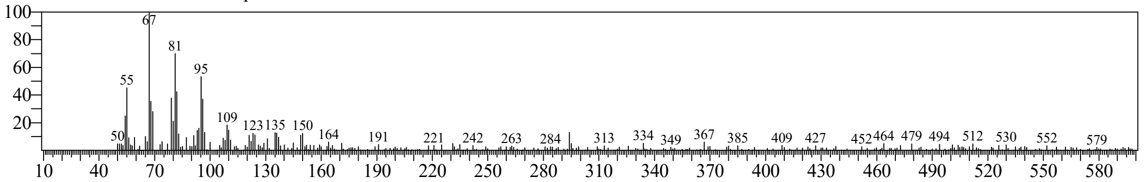


Hit#:1  Entry:137664  Library:NIST17-1.lib
SI:90  Formula:C19H34O2  CAS:0-00-0  MolWeight:294  RetIndex:2093
CompName:Methyl 10-trans,12-cis-octadecadienoate

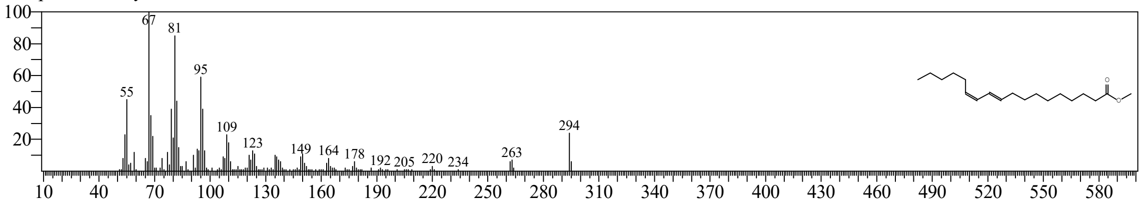


Hit#:2  Entry:125933  Library:NIST14.lib
SI:90  Formula:C19H34O2  CAS:0-00-0  MolWeight:294  RetIndex:2093
CompName:Methyl 10-trans,12-cis-octadecadienoate

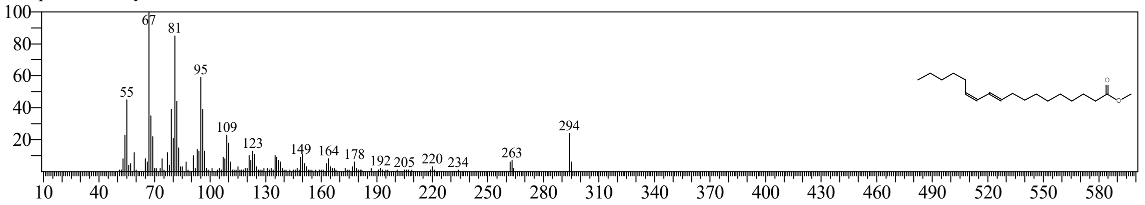


Hit#:3  Entry:137666  Library:NIST17-1.lib
SI:90  Formula:C19H34O2  CAS:13058-52-1  MolWeight:294  RetIndex:2093
CompName:Methyl 9-cis,11-trans-octadecadienoate

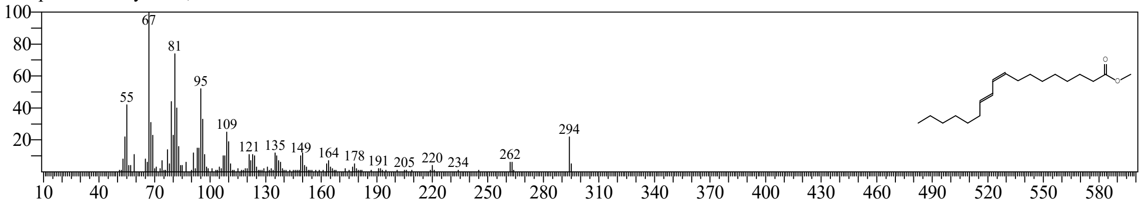


Hit#:4  Entry:125932  Library:NIST14.lib
SI:90  Formula:C19H34O2  CAS:0-00-0  MolWeight:294  RetIndex:2093
CompName:Methyl 9-cis,11-trans-octadecadienoate

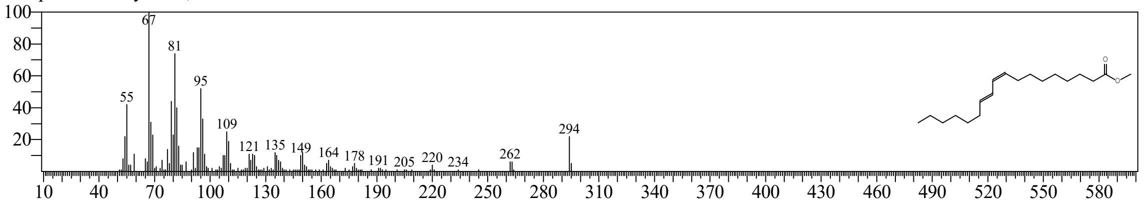


Hit#:5  Entry:32587  Library:NIST17s.lib
SI:90  Formula:C19H34O2  CAS:13038-47-6  MolWeight:294  RetIndex:2093
CompName:9,11-Octadecadienoic acid, methyl ester, (E,E)- $$ Methyl trans-9,trans-11-octadecadienoate $$ Methyl trans,trans-9,11-octadecadienoate $$ M

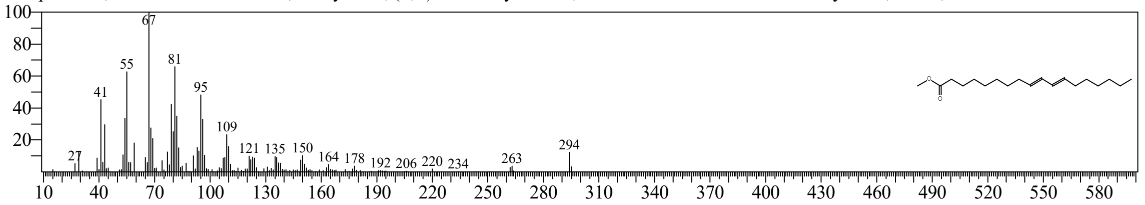

<< Target >>
Line#:19   R.Time:32.958(Scan#:2756)  MassPeaks:336
RawMode:Averaged 32.950-32.967(2755-2757) BasePeak:95.10(6860)
BG Mode:Calc. from Peak  Group 1 - Event 1

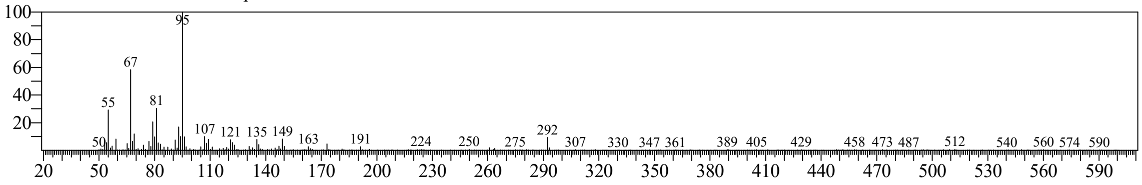


Hit#:1  Entry:24424  Library:NIST17-1.lib
SI:83  Formula:C12H20  CAS:0-00-0  MolWeight:164  RetIndex:1041
CompName:Cis-3-ethyl-endo-tricyclo[5.2.1.0(2.6)]decane

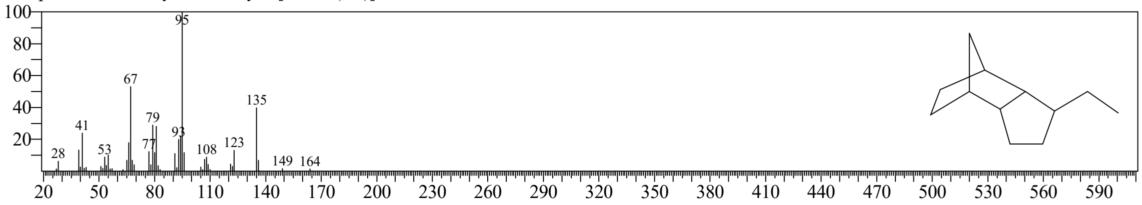


Hit#:2  Entry:23403  Library:NIST14.lib
SI:83  Formula:C12H20  CAS:0-00-0  MolWeight:164  RetIndex:1041
CompName:Cis-3-ethyl-endo-tricyclo[5.2.1.0(2.6)]decane

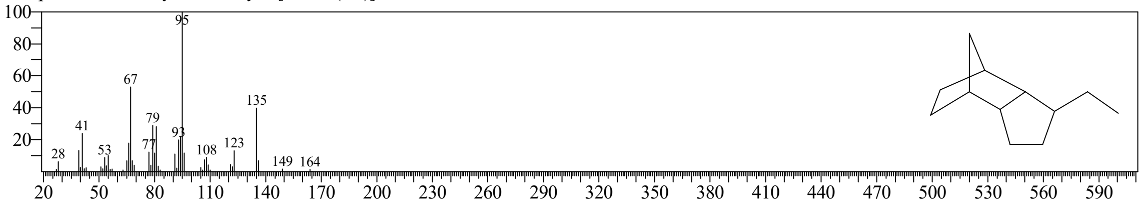


Hit#:3  Entry:16582  Library:NIST17-1.lib
SI:83  Formula:C11H18  CAS:0-00-0  MolWeight:150  RetIndex:1115
CompName:4-Cyclohexenyl-3-methylbutene

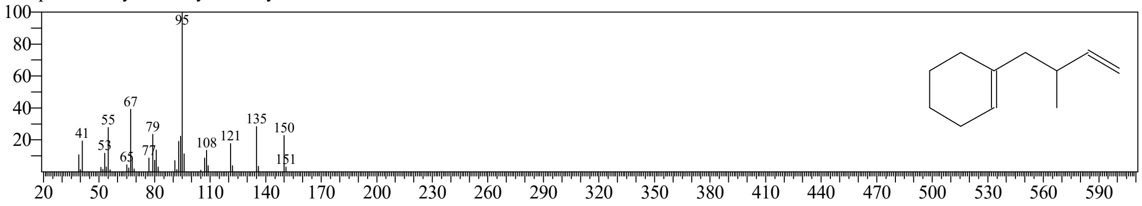


Hit#:4  Entry:16897  Library:NIST14.lib
SI:82  Formula:C10H16O  CAS:60485-45-2  MolWeight:152  RetIndex:991
CompName:Santolina epoxide $$ 2-Methyl-2-(3-methyl-1-vinyl-2-butenyl)oxirane # $$

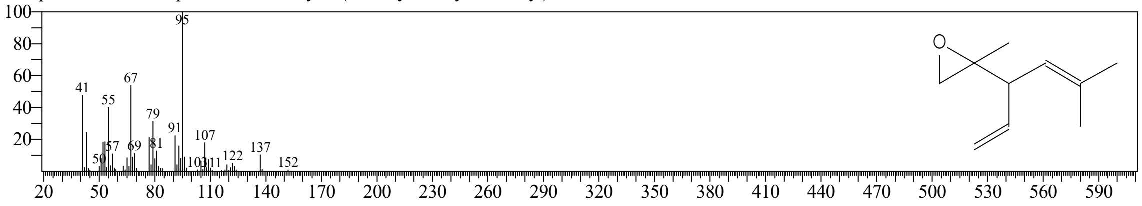


Hit#:5  Entry:17571  Library:NIST17-1.lib
SI:82  Formula:C10H16O  CAS:60485-45-2  MolWeight:152  RetIndex:991
CompName:Santolina epoxide $$ 2-Methyl-2-(3-methyl-1-vinyl-2-butenyl)oxirane # $$

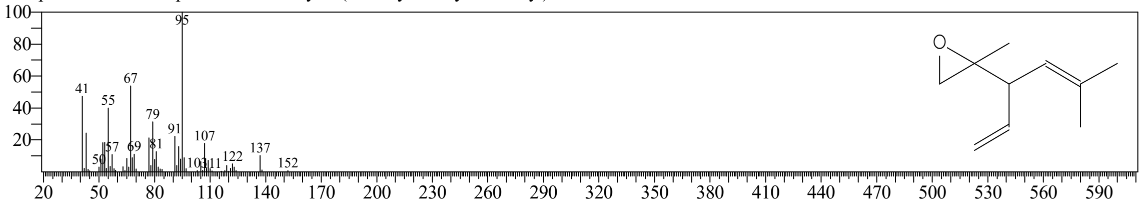

<< Target >>
Line#:20 R.Time:33.492(Scan#:2820) MassPeaks:366
RawMode:Averaged 33.483-33.500(2819-2821) BasePeak:74.00(16400)
BG Mode:Calc. from Peak Group 1 - Event 1

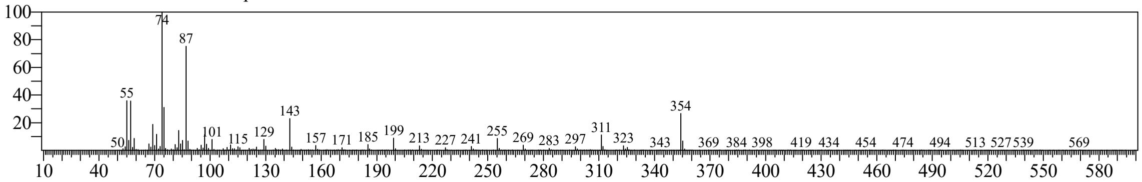


Hit#:1 Entry:196443 Library:NIST17-1.lib
SI:96 Formula:C23H46O2 CAS:929-77-1 MolWeight:354 RetIndex:2475
CompName:Docosanoic acid, methyl ester $$ Behenic acid, methyl ester $$ Methyl behenate $$ Methyl docosanoate $$ n-Docosanoic acid methyl ester $$

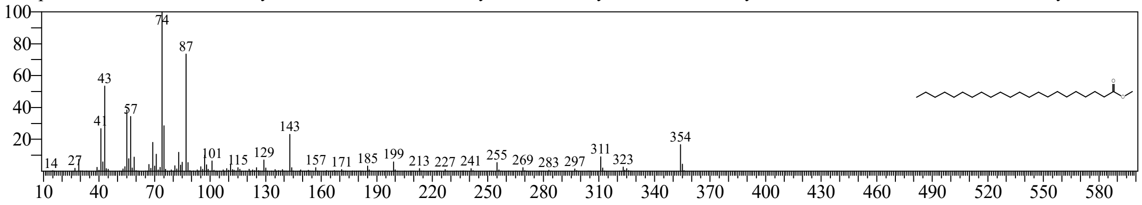


Hit#:2 Entry:178051 Library:NIST14.lib
SI:96 Formula:C23H46O2 CAS:929-77-1 MolWeight:354 RetIndex:2475
CompName:Docosanoic acid, methyl ester $$ Behenic acid, methyl ester $$ Methyl behenate $$ Methyl docosanoate $$ n-Docosanoic acid methyl ester $$

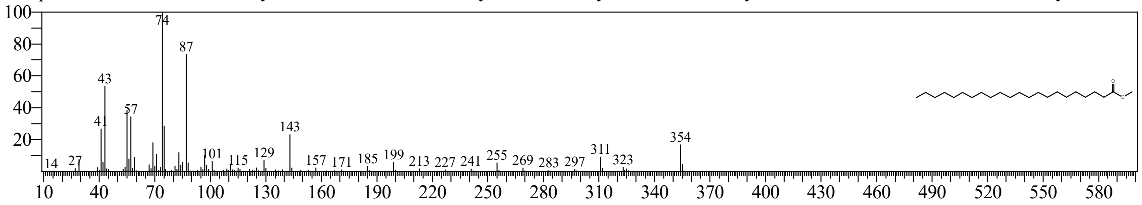


Hit#:3 Entry:196444 Library:NIST17-1.lib
SI:95 Formula:C23H46O2 CAS:0-00-0 MolWeight:354 RetIndex:2411
CompName:Methyl 20-methyl-heneicosanoate

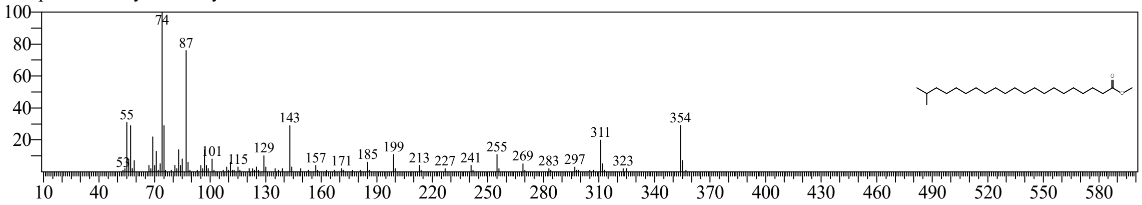


Hit#:4 Entry:178052 Library:NIST14.lib
SI:95 Formula:C23H46O2 CAS:0-00-0 MolWeight:354 RetIndex:2411
CompName:Methyl 20-methyl-heneicosanoate

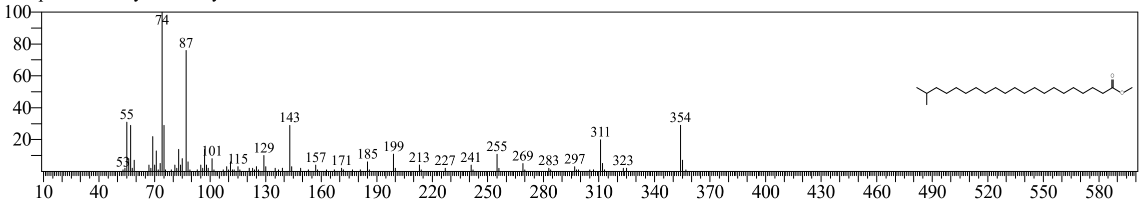


Hit#:5 Entry:31088 Library:NIST14s.lib
SI:95 Formula:C23H46O2 CAS:929-77-1 MolWeight:354 RetIndex:2475
CompName:Docosanoic acid, methyl ester $$ Behenic acid, methyl ester $$ Methyl behenate $$ Methyl docosanoate $$ n-Docosanoic acid methyl ester $$

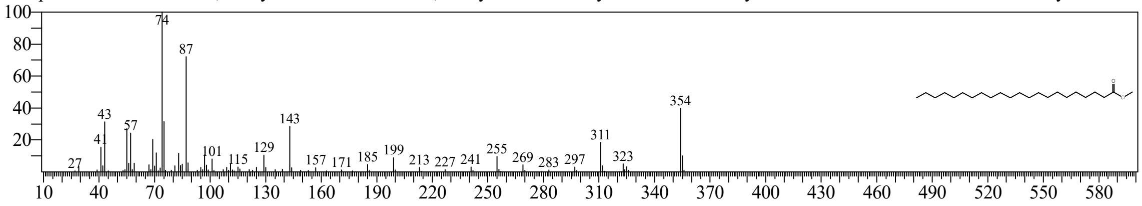

<< Target >>
Line#:21   R.Time:37.783(Scan#:3335)  MassPeaks:351
RawMode:Averaged 37.775-37.792(3334-3336) BasePeak:74.00(3981)
BG Mode:Calc. from Peak  Group 1 - Event 1

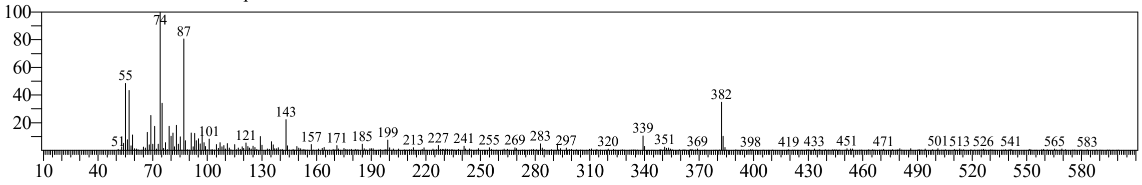

Hit#:1  Entry:37106  Library:NIST17s.lib
SI:87  Formula:C25H50O2  CAS:2442-49-1  MolWeight:382  RetIndex:2674
CompName:Tetracosanoic acid, methyl ester $$ Methyl lignocerate $$ Methyl tetracosanoate $$ Lignoceric acid methyl ester $$

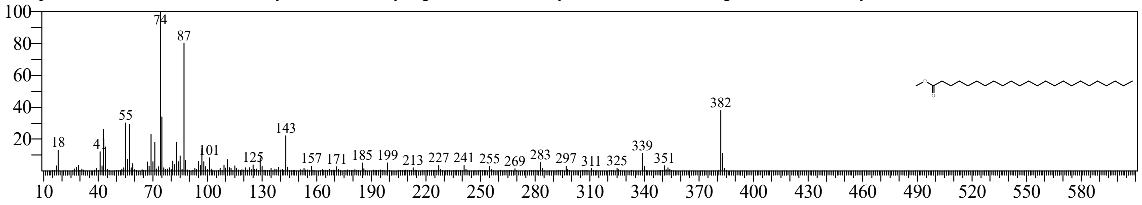

Hit#:2  Entry:31970  Library:NIST14s.lib
SI:87  Formula:C25H50O2  CAS:2442-49-1  MolWeight:382  RetIndex:2674
CompName:Tetracosanoic acid, methyl ester $$ Methyl lignocerate $$ Methyl tetracosanoate $$ Lignoceric acid methyl ester $$

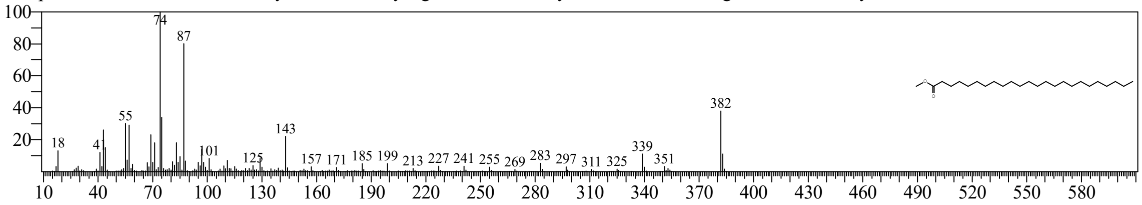

Hit#:3  Entry:37107  Library:NIST17s.lib
SI:84  Formula:C25H50O2  CAS:2442-49-1  MolWeight:382  RetIndex:2674
CompName:Tetracosanoic acid, methyl ester $$ Methyl lignocerate $$ Methyl tetracosanoate $$ Lignoceric acid methyl ester $$

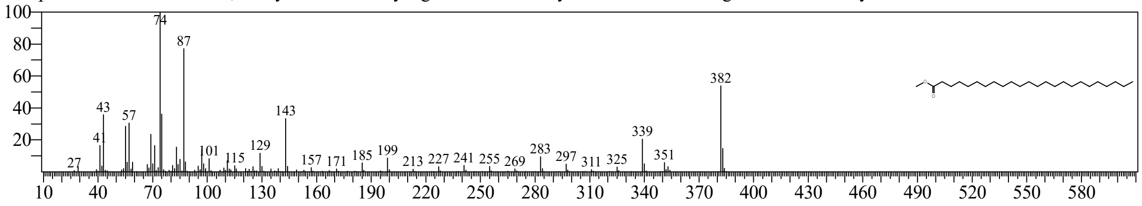

Hit#:4  Entry:31971  Library:NIST14s.lib
SI:84  Formula:C25H50O2  CAS:2442-49-1  MolWeight:382  RetIndex:2674
CompName:Tetracosanoic acid, methyl ester $$ Methyl lignocerate $$ Methyl tetracosanoate $$ Lignoceric acid methyl ester $$

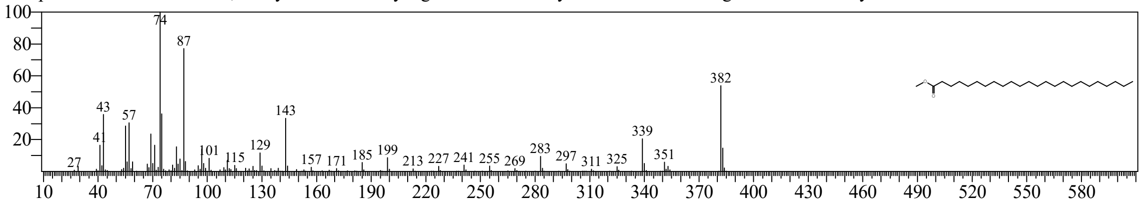

Hit#:5  Entry:31969  Library:NIST14s.lib
SI:83  Formula:C25H50O2  CAS:2442-49-1  MolWeight:382  RetIndex:2674
CompName:Tetracosanoic acid, methyl ester $$ Methyl lignocerate $$ Methyl tetracosanoate $$ Lignoceric acid methyl ester $$

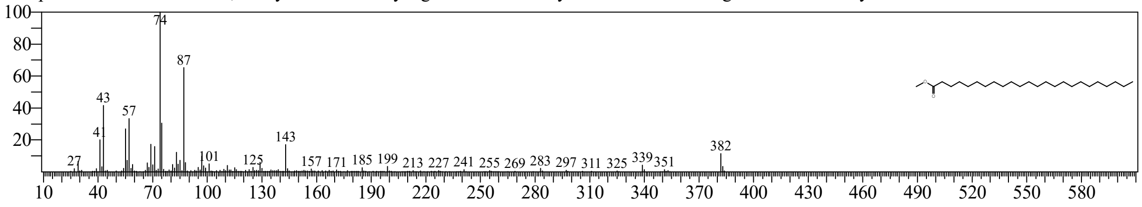

<< Target >>
Line#:22 R.Time:38.333(Scan#:3401) MassPeaks:318
RawMode:Averaged 38.325-38.342(3400-3402) BasePeak:79.00(598)
BG Mode:Calc. from Peak Group 1 - Event 1

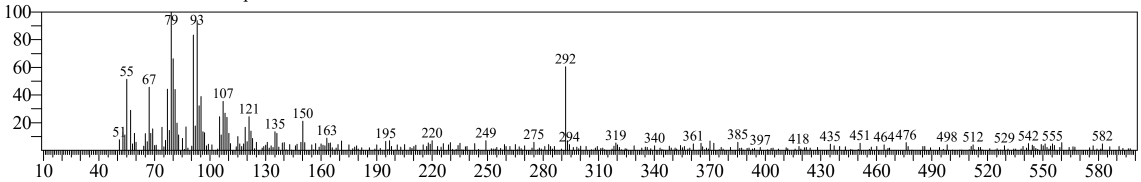


Hit#:1 Entry:124230 Library:NIST14.lib
SI:89 Formula:C19H32O2 CAS:0-00-0 MolWeight:292 RetIndex:2101
CompName:Methyl 9.cis.,11.trans.t,13.trans.-octadecatrienoate

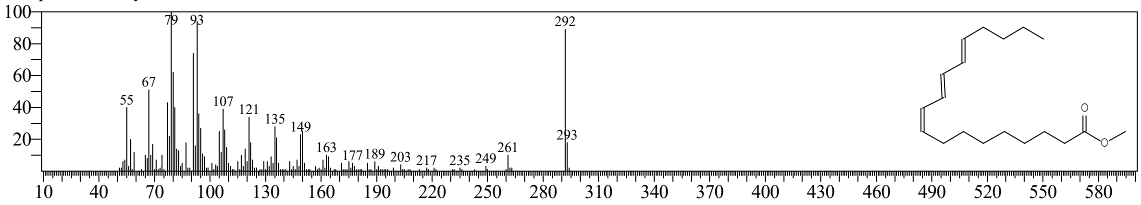


Hit#:2 Entry:135726 Library:NIST17-1.lib
SI:89 Formula:C19H32O2 CAS:0-00-0 MolWeight:292 RetIndex:2101
CompName:Methyl 9.cis.,11.trans.t,13.trans.-octadecatrienoate

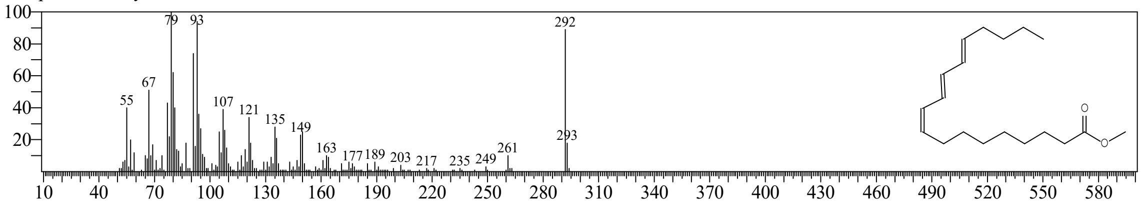


Hit#:3 Entry:124225 Library:NIST14.lib
SI:86 Formula:C19H32O2 CAS:0-00-0 MolWeight:292 RetIndex:2101
CompName:.gamma.-Linolenic acid, methyl ester

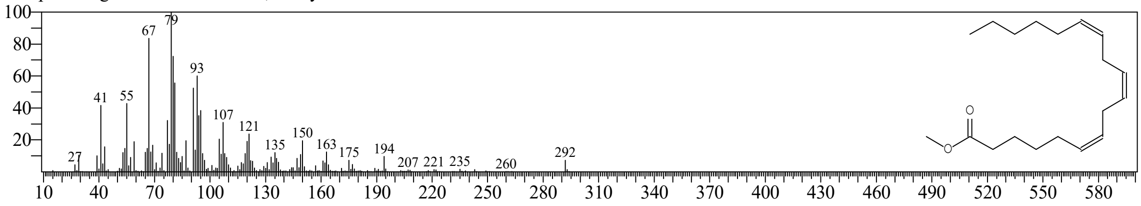


Hit#:4 Entry:135721 Library:NIST17-1.lib
SI:86 Formula:C19H32O2 CAS:16326-32-2 MolWeight:292 RetIndex:2101
CompName:Methyl .gamma.-linolenate

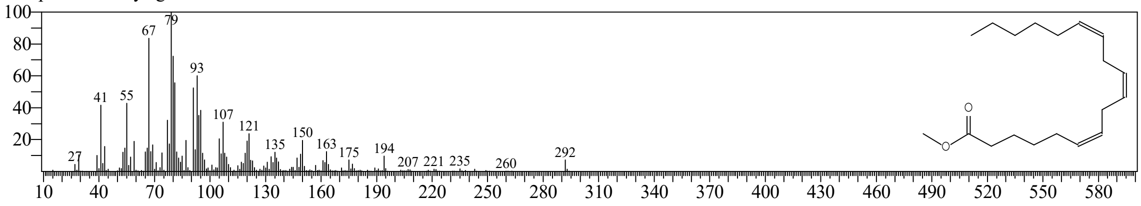


Hit#:5 Entry:135722 Library:NIST17-1.lib
SI:85 Formula:C19H32O2 CAS:0-00-0 MolWeight:292 RetIndex:2101
CompName:Methyl 6-cis,9-cis,11-trans-octadecatrienoate

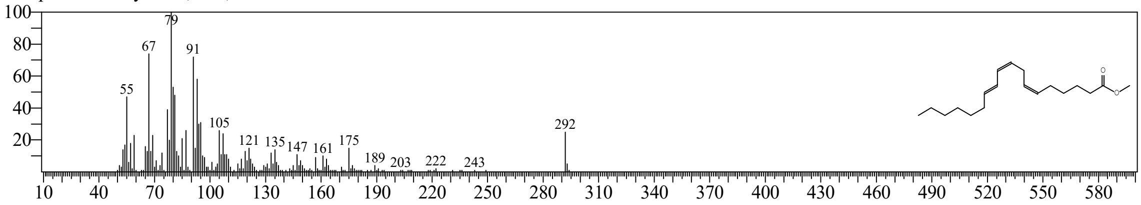

<< Target >>
Line#:23 R.Time:38.750(Scan#:3451) MassPeaks:310
RawMode:Averaged 38.742-38.758(3450-3452) BasePeak:93.10(874)
BG Mode:Calc. from Peak Group 1 - Event 1

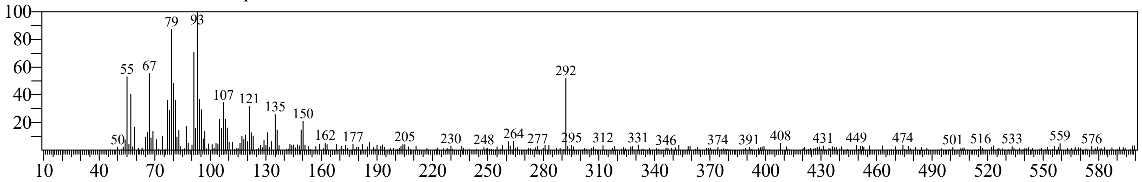


Hit#:1 Entry:124230 Library:NIST14.lib
SI:91 Formula:C19H32O2 CAS:0-00-0 MolWeight:292 RetIndex:2101
CompName:Methyl 9.cis.,11.trans.t,13.trans.-octadecatrienoate

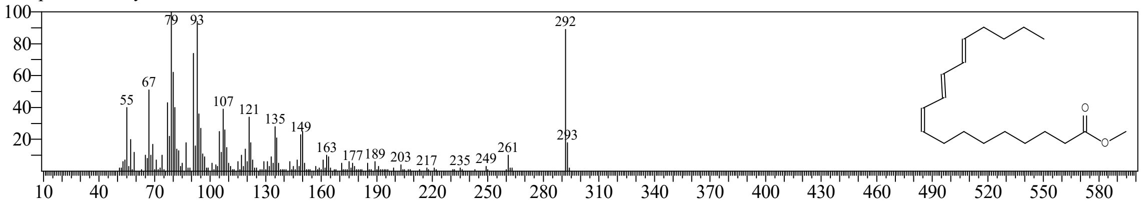


Hit#:2 Entry:135726 Library:NIST17-1.lib
SI:91 Formula:C19H32O2 CAS:0-00-0 MolWeight:292 RetIndex:2101
CompName:Methyl 9.cis.,11.trans.t,13.trans.-octadecatrienoate

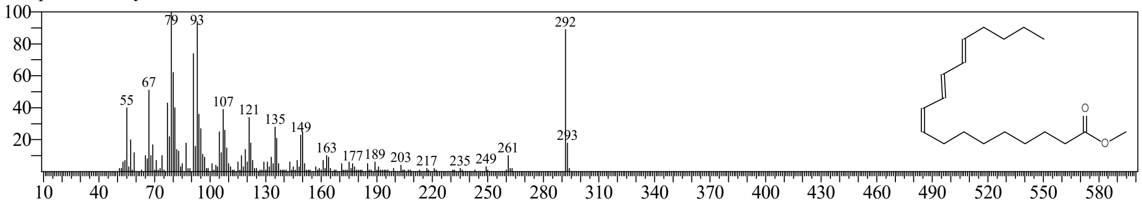


Hit#:3 Entry:135724 Library:NIST17-1.lib
SI:86 Formula:C19H32O2 CAS:301-00-8 MolWeight:292 RetIndex:2101
CompName:9,12,15-Octadecatrienoic acid, methyl ester, (Z,Z,Z)- $$ Linolenic acid, methyl ester $$ Methyl all-cis-9,12,15-octadecatrienoate $$ Methyl lino

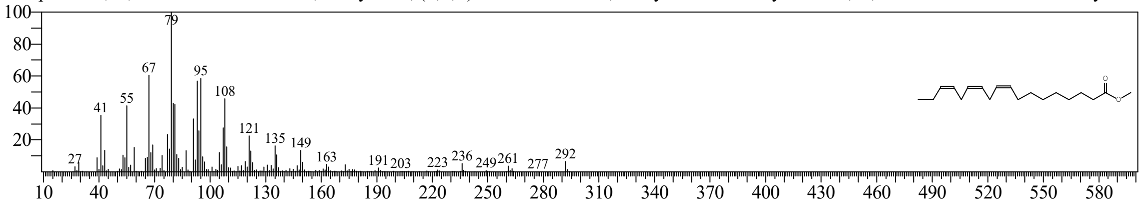


Hit#:4 Entry:124228 Library:NIST14.lib
SI:86 Formula:C19H32O2 CAS:301-00-8 MolWeight:292 RetIndex:2101
CompName:9,12,15-Octadecatrienoic acid, methyl ester, (Z,Z,Z)- $$ Linolenic acid, methyl ester $$ Methyl all-cis-9,12,15-octadecatrienoate $$ Methyl lino

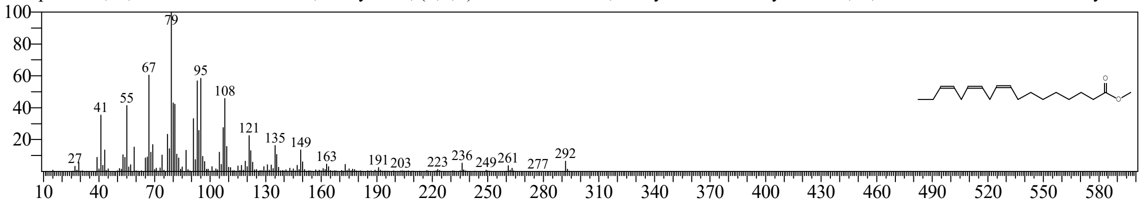


Hit#:5 Entry:135722 Library:NIST17-1.lib
SI:86 Formula:C19H32O2 CAS:0-00-0 MolWeight:292 RetIndex:2101
CompName:Methyl 6-cis,9-cis,11-trans-octadecatrienoate

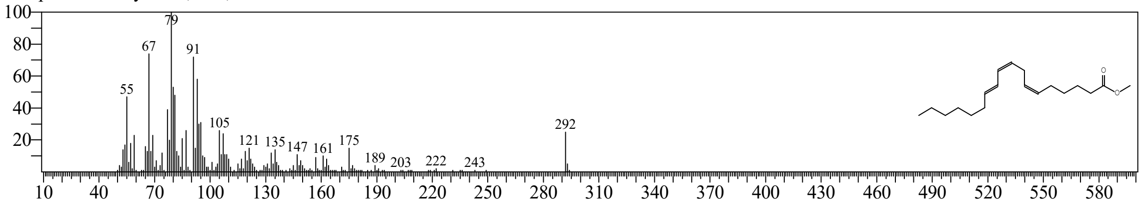

<< Target >>
Line#:24   R.Time:38.900(Scan#:3469)  MassPeaks:346
RawMode:Averaged 38.892-38.908(3468-3470) BasePeak:79.05(875)
BG Mode:Calc. from Peak  Group 1 - Event 1

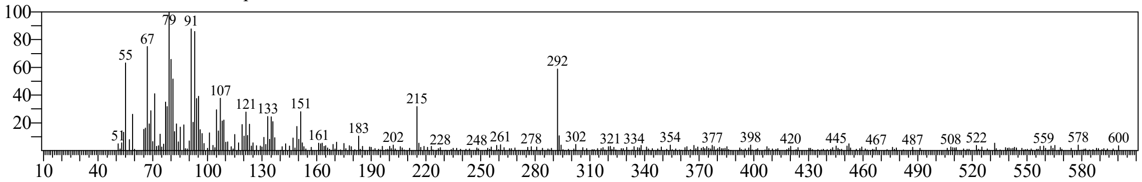


Hit#:1  Entry:135726  Library:NIST17-1.lib
SI:86  Formula:C19H32O2  CAS:0-00-0  MolWeight:292  RetIndex:2101
CompName:Methyl 9.cis.,11.trans.t,13.trans.-octadecatrienoate

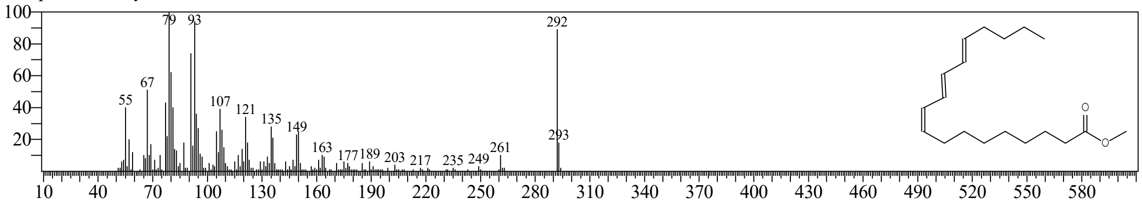


Hit#:2  Entry:124230  Library:NIST14.lib
SI:86  Formula:C19H32O2  CAS:0-00-0  MolWeight:292  RetIndex:2101
CompName:Methyl 9.cis.,11.trans.t,13.trans.-octadecatrienoate

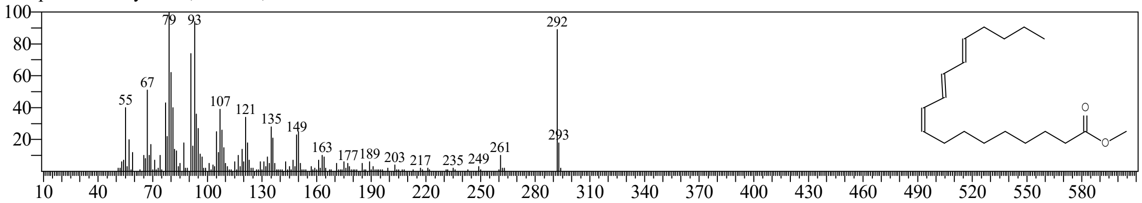


Hit#:3  Entry:124226  Library:NIST14.lib
SI:84  Formula:C19H32O2  CAS:0-00-0  MolWeight:292  RetIndex:2101
CompName:Methyl 6-cis,9-cis,11-trans-octadecatrienoate

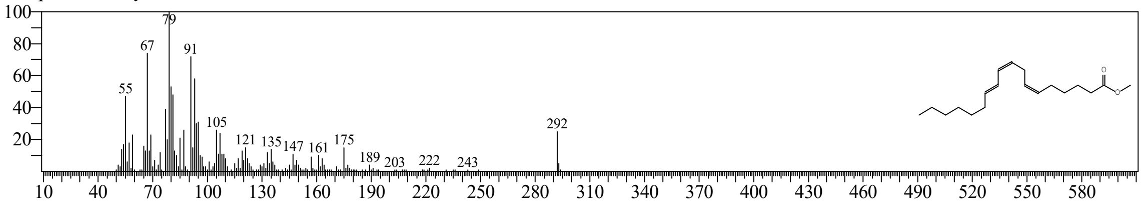


Hit#:4  Entry:135722  Library:NIST17-1.lib
SI:84  Formula:C19H32O2  CAS:0-00-0  MolWeight:292  RetIndex:2101
CompName:Methyl 6-cis,9-cis,11-trans-octadecatrienoate

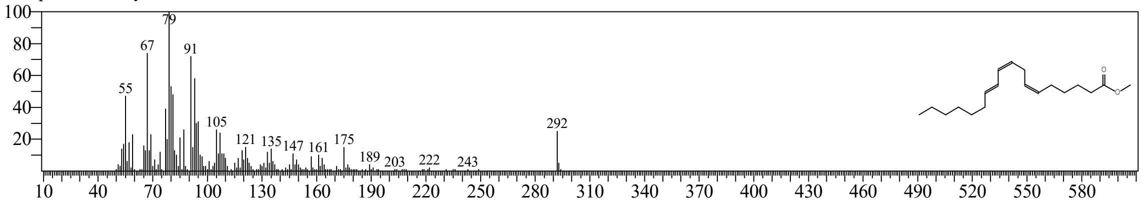


Hit#:5  Entry:149530  Library:NIST14.lib
SI:84  Formula:C21H36O2  CAS:0-00-0  MolWeight:320  RetIndex:2300
CompName:cis-5,8,11-Eicosatrienoic acid, methyl ester

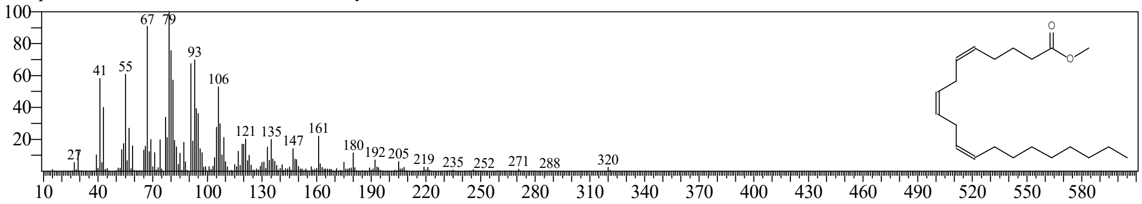

Supplement: Supplementary file 1 [file molecules-31-02891-s001.zip › 28d-QG200-2.pdf]
