# Supplementary material for: Selective Inhibition of Lipid Oxidation by a Quercetagetin-Rich Extract from Tagetes erecta: A Chemometric Approach Using OPLS-DA and Selectivity Index Analysis
Source: Molecules. 2026 Aug 19;31(16):2891. doi: 10.3390/molecules31162891 (PMC13515886; doi:10.3390/molecules31162891)

QG200-3-28d

<<Column>>
Name : HP-88
Thickness : 0.20um
Length : 100.0m
Inside Diameter : 0.25mm

Method

===== Analytical Line 1 =====

Column Oven Temp.	:120.0 °C	
Injection Temp.	:250.00 °C	
Injection Mode	:Split	
Column Flow	:1.04 mL/min	
Split Ratio	:20.0	
Oven Temp. Program		
Rate	Temperature(°C)	Hold Time(min)
-	120.0	1.00
10.00	175.0	10.00
5.00	210.0	5.00
5.00	230.0	15.00

[GCMS-QP2010]
IonSourceTemp :200.00 °C
Interface Temp. :220.00 °C
m/z scanning range：45～500m/z

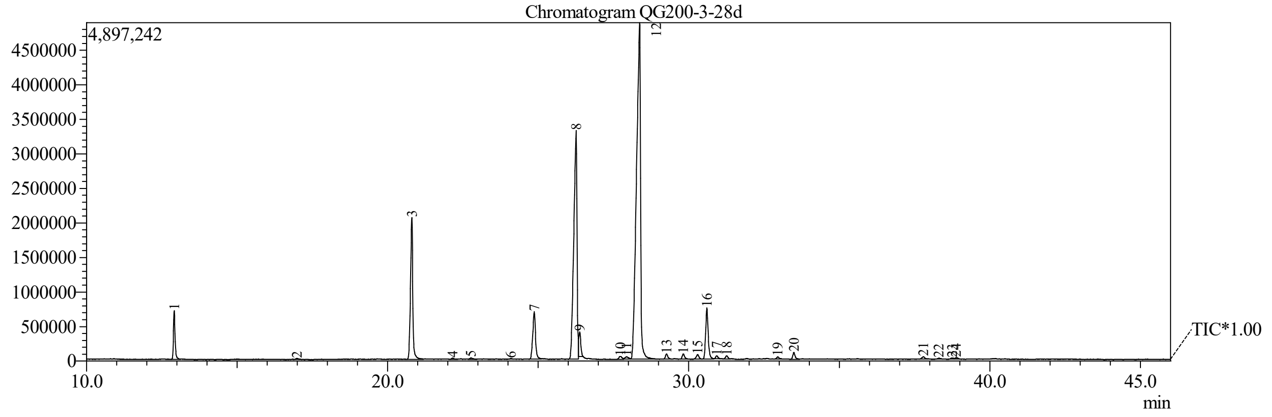

Peak Report TIC

Peak#	R.Time	Area	Area%	Height	Height%	A/H	Name
1	12.915	2624480	2.68	703859	5.26	3.73	Undecanoic acid, methyl ester
2	16.988	102416	0.10	17731	0.13	5.78	Methyl tetradecanoate
3	20.806	10820837	11.07	2051330	15.33	5.28	Hexadecanoic acid, methyl ester
4	22.162	113238	0.12	18476	0.14	6.13	7-Hexadecenoic acid, methyl ester, (Z)-
5	22.768	142908	0.15	21215	0.16	6.74	Heptadecanoic acid, methyl ester
6	24.093	45302	0.05	10758	0.08	4.21	cis-10-Heptadecenoic acid, methyl ester
7	24.870	4407738	4.51	685260	5.12	6.43	Methyl stearate
8	26.259	24322045	24.88	3308035	24.73	7.35	9-Octadecenoic acid, methyl ester, (E)-
9	26.379	1552155	1.59	355906	2.66	4.36	11-Octadecenoic acid, methyl ester
10	27.728	319616	0.33	43542	0.33	7.34	9,12-Octadecadienoic acid (Z,Z)-, methyl estei
11	27.934	232056	0.24	30885	0.23	7.51	9,12-Octadecadienoic acid, methyl ester
12	28.369	45755745	46.81	4869189	36.40	9.40	9,12-Octadecadienoic acid (Z,Z)-, methyl estei
13	29.261	441016	0.45	76297	0.57	5.78	Methyl 18-methylnonadecanoate
14	29.818	386232	0.40	73993	0.55	5.22	11,14,17-Eicosatrienoic acid, methyl ester

Peak#	R.Time	Area	Area%	Height	Height%	A/H	Name
15	30.294	416798	0.43	67465	0.50	6.18	9,12,15-Octadecatrienoic acid, methyl ester, (Z
16	30.601	4341941	4.44	738585	5.52	5.88	9,12,15-Octadecatrienoic acid, methyl ester, (Z
17	30.927	210017	0.21	41568	0.31	5.05	Methyl 9-cis,11-trans-octadecadienoate
18	31.257	240383	0.25	46510	0.35	5.17	Methyl 10-trans,12-cis-octadecadienoate
19	32.953	171038	0.17	31236	0.23	5.48	Cis-3-ethyl-endo-tricyclo[5.2.1.0(2.6)]decane
20	33.490	552451	0.57	99739	0.75	5.54	Docosanoic acid, methyl ester
21	37.785	213669	0.22	32938	0.25	6.49	Tetracosanoic acid, methyl ester
22	38.300	78811	0.08	12879	0.10	6.12	Methyl 9.cis.,11.trans.t,13.trans.-octadecatrien
23	38.742	115264	0.12	18243	0.14	6.32	Methyl 9.cis.,11.trans.t,13.trans.-octadecatrien
24	38.875	150480	0.15	21352	0.16	7.05	Methyl 9.cis.,11.trans.t,13.trans.-octadecatrien
		97756636	100.00	13376991	100.00		

Library

<< Target >>
Line#:1 R.Time:12.917(Scan#:351) MassPeaks:295
RawMode:Averaged 12.908-12.925(350-352) BasePeak:74.00(205912)
BG Mode:Calc. from Peak Group 1 - Event 1

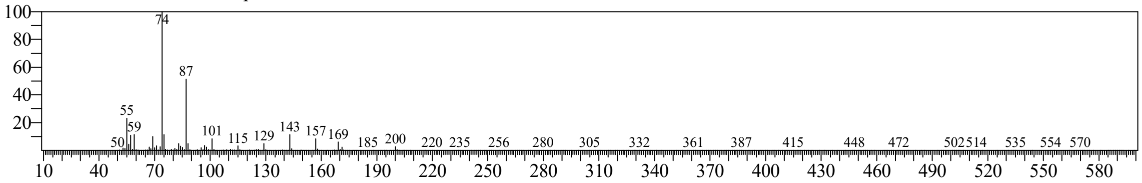

Hit#:1 Entry:49598 Library:NIST17-1.lib
SI:95 Formula:C12H24O2 CAS:1731-86-8 MolWeight:200 RetIndex:1381
CompName:Undecanoic acid, methyl ester $$ Methyl undecanoate $$ Methyl undecylenate $$ n-Undecanoic acid methyl ester $$ Methyl ester of undecanoi

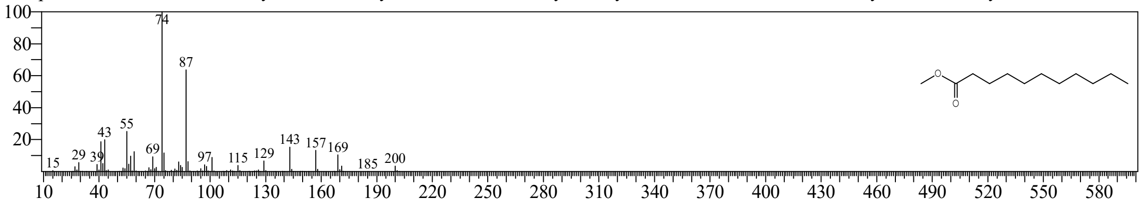

Hit#:2 Entry:46966 Library:NIST14.lib
SI:95 Formula:C12H24O2 CAS:1731-86-8 MolWeight:200 RetIndex:1381
CompName:Undecanoic acid, methyl ester $$ Methyl undecanoate $$ Methyl undecylenate $$ n-Undecanoic acid methyl ester $$ Methyl ester of undecanoi

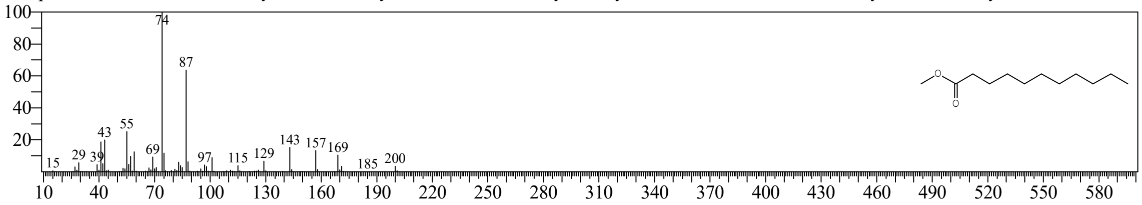

Hit#:3 Entry:21618 Library:NIST17s.lib
SI:95 Formula:C12H24O2 CAS:1731-86-8 MolWeight:200 RetIndex:1381
CompName:Undecanoic acid, methyl ester $$ Methyl undecanoate $$ Methyl undecylenate $$ n-Undecanoic acid methyl ester $$ Methyl ester of undecanoi

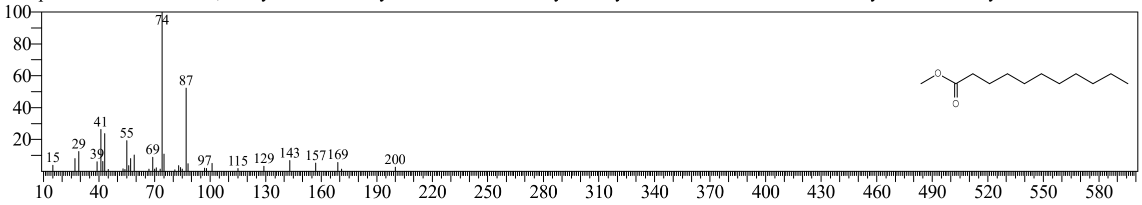

Hit#:4 Entry:21619 Library:NIST17s.lib
SI:94 Formula:C12H24O2 CAS:1731-86-8 MolWeight:200 RetIndex:1381
CompName:Undecanoic acid, methyl ester $$ Methyl undecanoate $$ Methyl undecylenate $$ n-Undecanoic acid methyl ester $$ Methyl ester of undecanoi

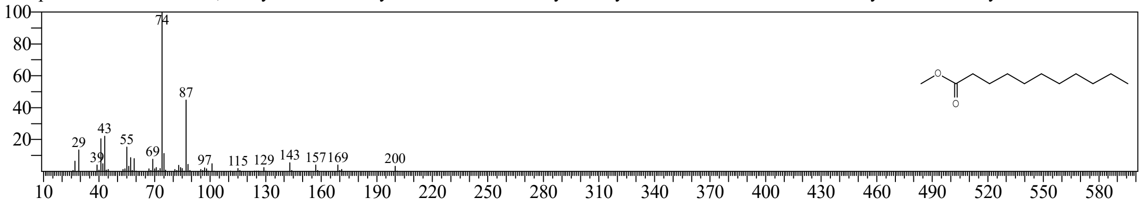

<< Target >>
Line#:1 R.Time:12.917(Scan#:351) MassPeaks:295
RawMode:Averaged 12.908-12.925(350-352) BasePeak:74.00(205912)
BG Mode:Calc. from Peak Group 1 - Event 1

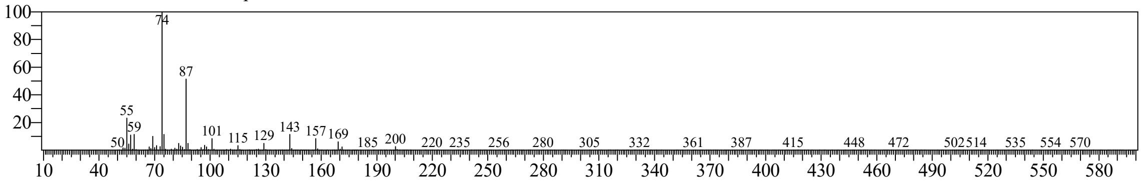

Hit#:5 Entry:18487 Library:NIST14s.lib
SI:94 Formula:C12H24O2 CAS:1731-86-8 MolWeight:200 RetIndex:1381
CompName:Undecanoic acid, methyl ester $$ Methyl undecanoate $$ Methyl undecylenate $$ n-Undecanoic acid methyl ester $$ Methyl ester of undecanoi

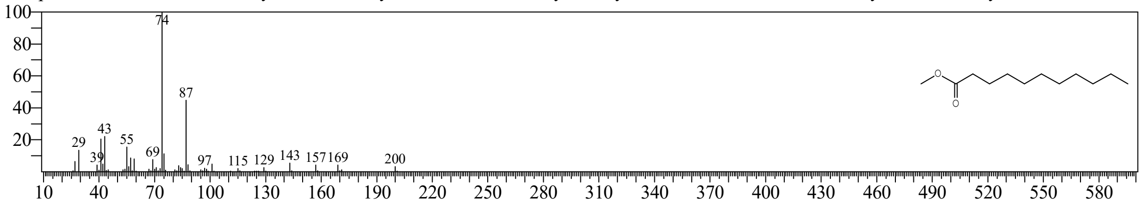

<< Target >>
Line#:2 R.Time:16.992(Scan#:840) MassPeaks:343
RawMode:Averaged 16.983-17.000(839-841) BasePeak:74.00(3429)
BG Mode:Calc. from Peak Group 1 - Event 1

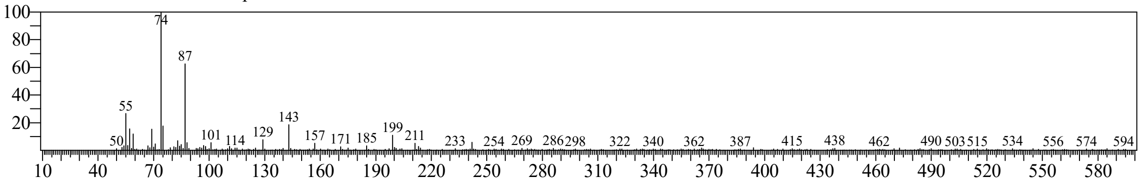

Hit#:1 Entry:86786 Library:NIST17-1.lib
SI:95 Formula:C15H30O2 CAS:124-10-7 MolWeight:242 RetIndex:1680
CompName:Methyl tetradecanoate $$ Tetradecanoic acid, methyl ester $$ Myristic acid, methyl ester $$ Metholeneat 2495 $$ Methyl myristate $$ Methyl [illegible]

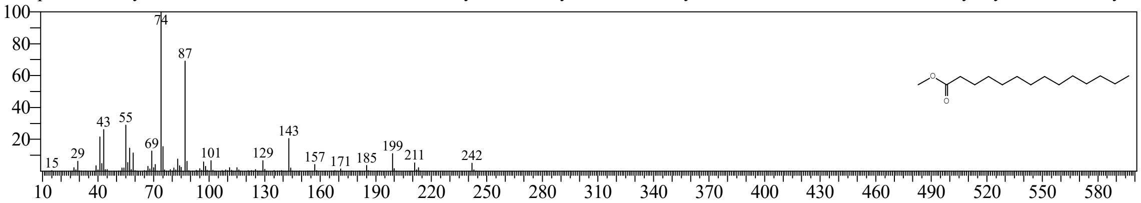

Hit#:2 Entry:80645 Library:NIST14.lib
SI:95 Formula:C15H30O2 CAS:124-10-7 MolWeight:242 RetIndex:1680
CompName:Methyl tetradecanoate $$ Tetradecanoic acid, methyl ester $$ Myristic acid, methyl ester $$ Metholeneat 2495 $$ Methyl myristate $$ Methyl [illegible]

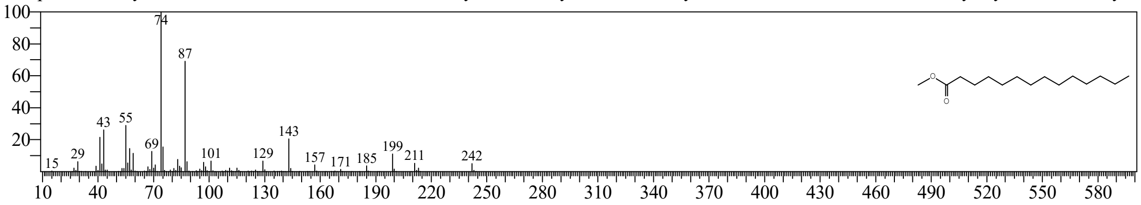

Hit#:3 Entry:86785 Library:NIST17-1.lib
SI:92 Formula:C15H30O2 CAS:5129-58-8 MolWeight:242 RetIndex:1615
CompName:Tridecanoic acid, 12-methyl-, methyl ester $$ Methyl isomyristate $$ Methyl 12-methyltridecanoate $$

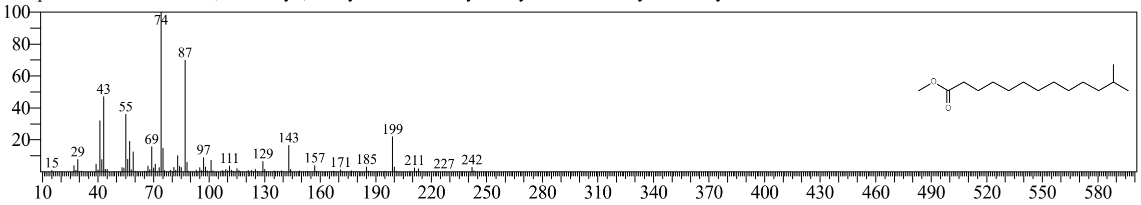

Hit#:4 Entry:80644 Library:NIST14.lib
SI:92 Formula:C15H30O2 CAS:5129-58-8 MolWeight:242 RetIndex:1615
CompName:Tridecanoic acid, 12-methyl-, methyl ester $$ Methyl isomyristate $$ Methyl 12-methyltridecanoate $$

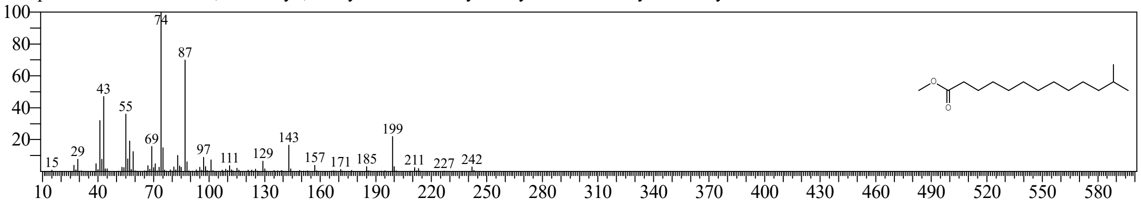

Hit#:5 Entry:23848 Library:NIST14s.lib
SI:91 Formula:C15H30O2 CAS:124-10-7 MolWeight:242 RetIndex:1680
CompName:Methyl tetradecanoate $$ Tetradecanoic acid, methyl ester $$ Myristic acid, methyl ester $$ Metholeneat 2495 $$ Methyl myristate $$ Methyl [illegible]

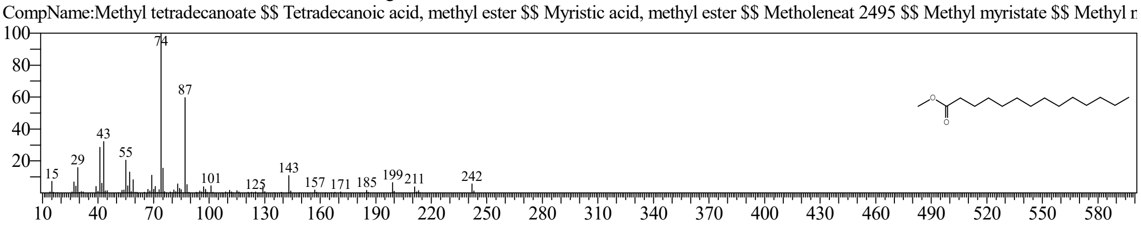

<< Target >>
Line#:3 R.Time:20.808(Scan#:1298) MassPeaks:381
RawMode:Averaged 20.800-20.817(1297-1299) BasePeak:74.00(473449)
BG Mode:Calc. from Peak Group 1 - Event 1

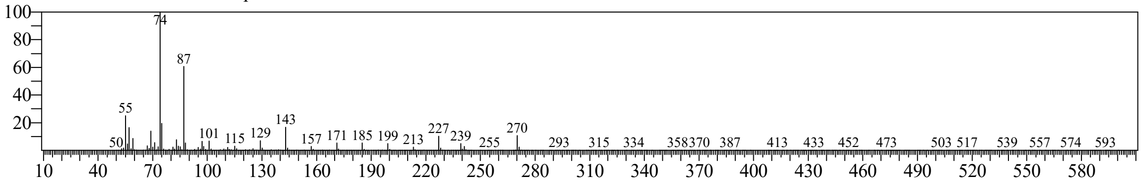

Hit#:1 Entry:113663 Library:NIST17-1.lib
SI:97 Formula:C17H34O2 CAS:112-39-0 MolWeight:270 RetIndex:1878
CompName:Hexadecanoic acid, methyl ester $$ Palmitic acid, methyl ester $$ n-Hexadecanoic acid methyl ester $$ Metholene 2216 $$ Methyl hexadecano

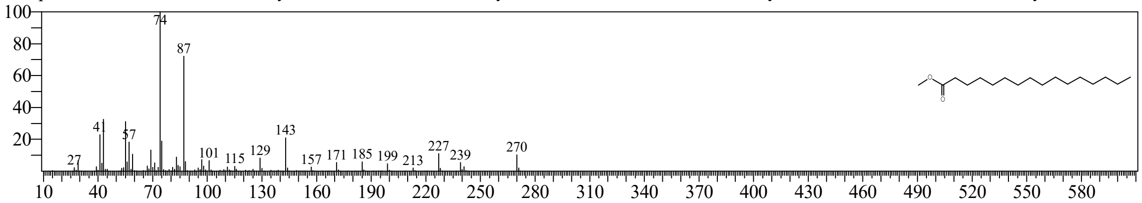

Hit#:2 Entry:104648 Library:NIST14.lib
SI:97 Formula:C17H34O2 CAS:112-39-0 MolWeight:270 RetIndex:1878
CompName:Hexadecanoic acid, methyl ester $$ Palmitic acid, methyl ester $$ n-Hexadecanoic acid methyl ester $$ Metholene 2216 $$ Methyl hexadecano

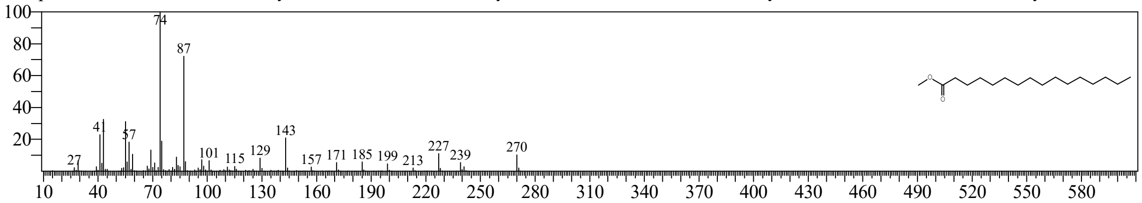

Hit#:3 Entry:26273 Library:NIST14s.lib
SI:97 Formula:C17H34O2 CAS:112-39-0 MolWeight:270 RetIndex:1878
CompName:Hexadecanoic acid, methyl ester $$ Palmitic acid, methyl ester $$ n-Hexadecanoic acid methyl ester $$ Metholene 2216 $$ Methyl hexadecano

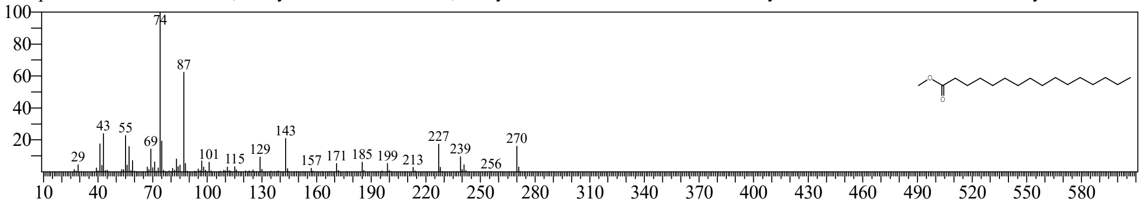

Hit#:4 Entry:26271 Library:NIST14s.lib
SI:96 Formula:C17H34O2 CAS:112-39-0 MolWeight:270 RetIndex:1878
CompName:Hexadecanoic acid, methyl ester $$ Palmitic acid, methyl ester $$ n-Hexadecanoic acid methyl ester $$ Metholene 2216 $$ Methyl hexadecano

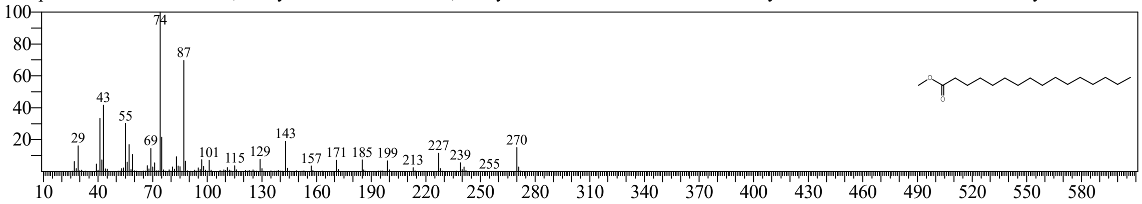

Hit#:5 Entry:30646 Library:NIST17s.lib
SI:96 Formula:C17H34O2 CAS:112-39-0 MolWeight:270 RetIndex:1878
CompName:Hexadecanoic acid, methyl ester $$ Palmitic acid, methyl ester $$ n-Hexadecanoic acid methyl ester $$ Metholene 2216 $$ Methyl hexadecano

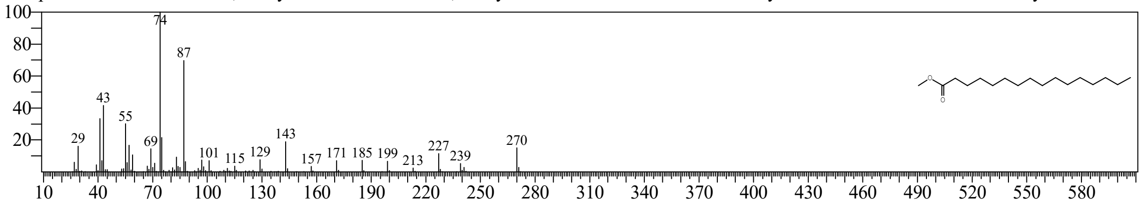

<< Target >>
Line#:4 R.Time:22.158(Scan#:1460) MassPeaks:352
RawMode:Averaged 22.150-22.167(1459-1461) BasePeak:55.05(1505)
BG Mode:Calc. from Peak Group 1 - Event 1

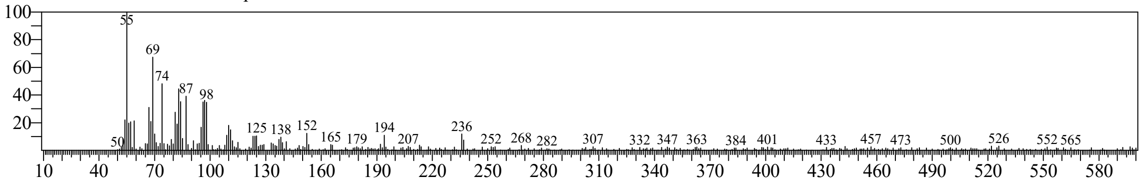

Hit#:1 Entry:111510 Library:NIST17-1.lib
SI:94 Formula:C17H32O2 CAS:56875-67-3 MolWeight:268 RetIndex:1886
CompName:7-Hexadecenoic acid, methyl ester, (Z)- $$ Methyl (7E)-7-hexadecenoate # $$

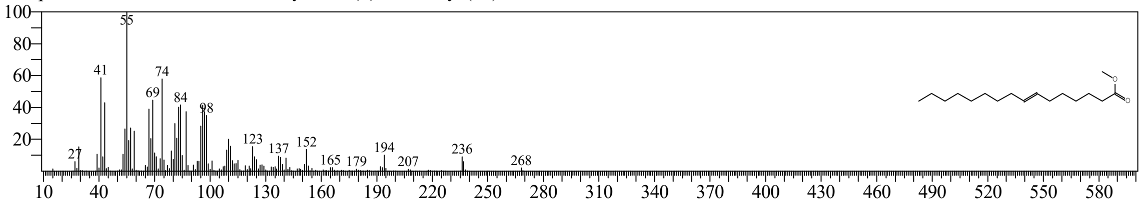

Hit#:2 Entry:30415 Library:NIST17s.lib
SI:93 Formula:C17H32O2 CAS:1120-25-8 MolWeight:268 RetIndex:1886
CompName:9-Hexadecenoic acid, methyl ester, (Z)- $$ Methyl palmitoleate $$ Methyl palmitoleinate $$ Palmitoleic acid, methyl ester $$ Methyl (9Z)-9-he:

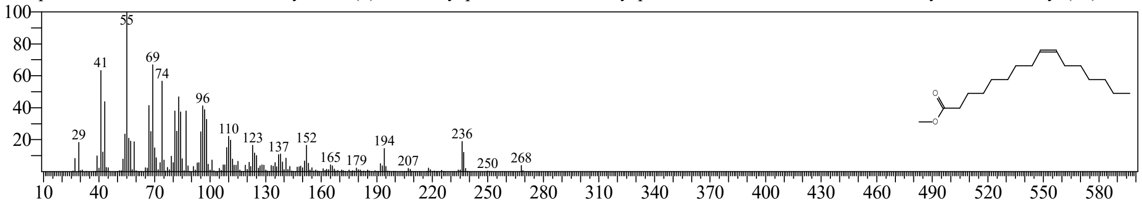

Hit#:3 Entry:26066 Library:NIST14s.lib
SI:93 Formula:C17H32O2 CAS:1120-25-8 MolWeight:268 RetIndex:1886
CompName:9-Hexadecenoic acid, methyl ester, (Z)- $$ Methyl palmitoleate $$ Methyl palmitoleinate $$ Palmitoleic acid, methyl ester $$ Methyl (9Z)-9-he:

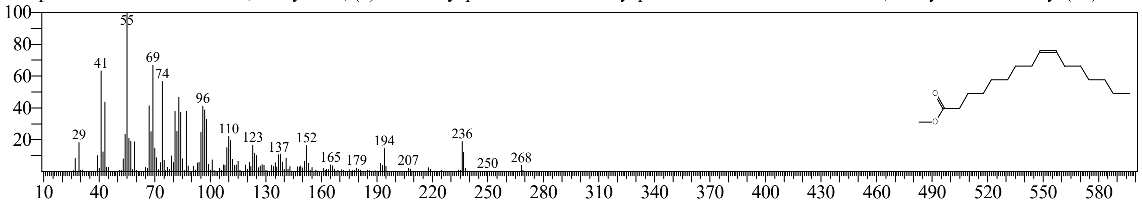

Hit#:4 Entry:30413 Library:NIST17s.lib
SI:93 Formula:C17H32O2 CAS:1120-25-8 MolWeight:268 RetIndex:1886
CompName:9-Hexadecenoic acid, methyl ester, (Z)- $$ Methyl palmitoleate $$ Methyl palmitoleinate $$ Palmitoleic acid, methyl ester $$ Methyl (9Z)-9-he:

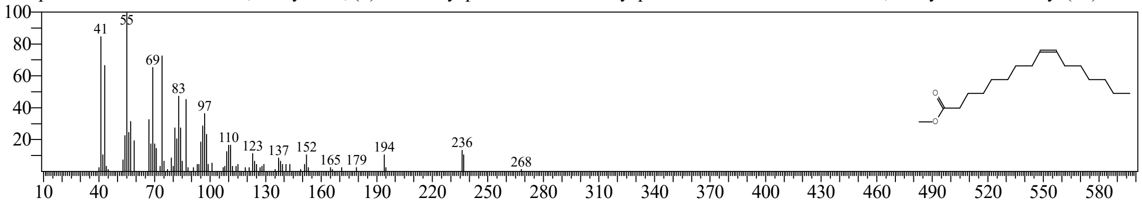

Hit#:5 Entry:26064 Library:NIST14s.lib
SI:93 Formula:C17H32O2 CAS:1120-25-8 MolWeight:268 RetIndex:1886
CompName:9-Hexadecenoic acid, methyl ester, (Z)- $$ Methyl palmitoleate $$ Methyl palmitoleinate $$ Palmitoleic acid, methyl ester $$ Methyl (9Z)-9-he:

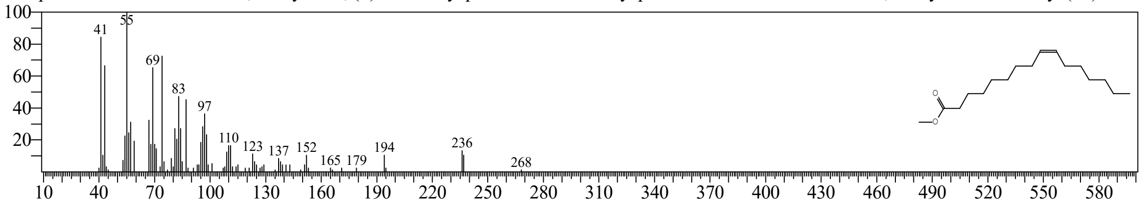

<< Target >>
Line#:5 R.Time:22.767(Scan#:1533) MassPeaks:335
RawMode:Averaged 22.758-22.775(1532-1534) BasePeak:74.00(3491)
BG Mode:Calc. from Peak Group 1 - Event 1

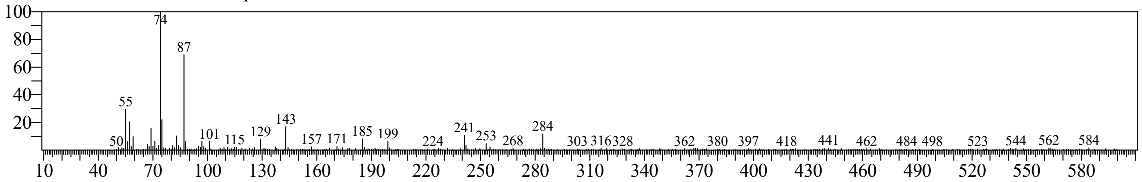

Hit#:1 Entry:27274 Library:NIST14s.lib
SI:93 Formula:C18H36O2 CAS:1731-92-6 MolWeight:284 RetIndex:1978
CompName:Heptadecanoic acid, methyl ester $$ Margaric acid methyl ester $$ Methyl heptadecanoate $$ Methyl margarate $$ n-Heptadecanoic acid methy

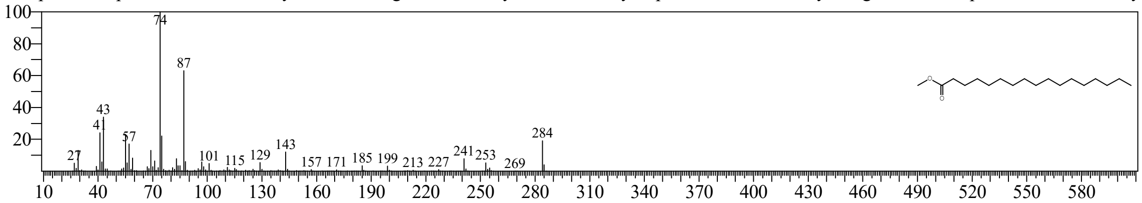

Hit#:2 Entry:31783 Library:NIST17s.lib
SI:93 Formula:C18H36O2 CAS:1731-92-6 MolWeight:284 RetIndex:1978
CompName:Heptadecanoic acid, methyl ester $$ Margaric acid methyl ester $$ Methyl heptadecanoate $$ Methyl margarate $$ n-Heptadecanoic acid methy

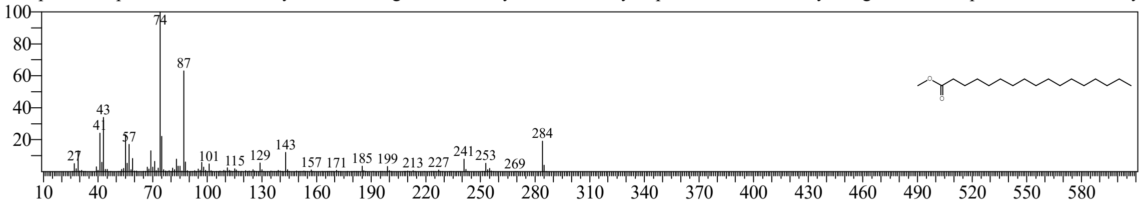

Hit#:3 Entry:31784 Library:NIST17s.lib
SI:92 Formula:C18H36O2 CAS:1731-92-6 MolWeight:284 RetIndex:1978
CompName:Heptadecanoic acid, methyl ester $$ Margaric acid methyl ester $$ Methyl heptadecanoate $$ Methyl margarate $$ n-Heptadecanoic acid methy

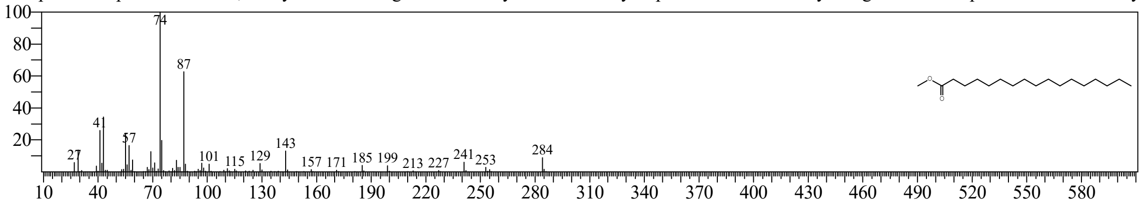

Hit#:4 Entry:27275 Library:NIST14s.lib
SI:92 Formula:C18H36O2 CAS:1731-92-6 MolWeight:284 RetIndex:1978
CompName:Heptadecanoic acid, methyl ester $$ Margaric acid methyl ester $$ Methyl heptadecanoate $$ Methyl margarate $$ n-Heptadecanoic acid methy

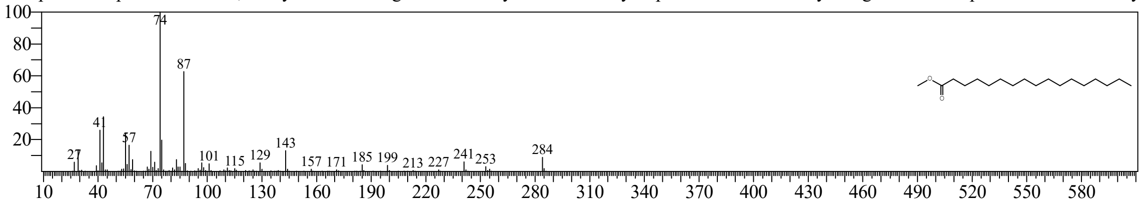

Hit#:5 Entry:27279 Library:NIST14s.lib
SI:92 Formula:C18H36O2 CAS:1731-92-6 MolWeight:284 RetIndex:1978
CompName:Heptadecanoic acid, methyl ester $$ Margaric acid methyl ester $$ Methyl heptadecanoate $$ Methyl margarate $$ n-Heptadecanoic acid methy

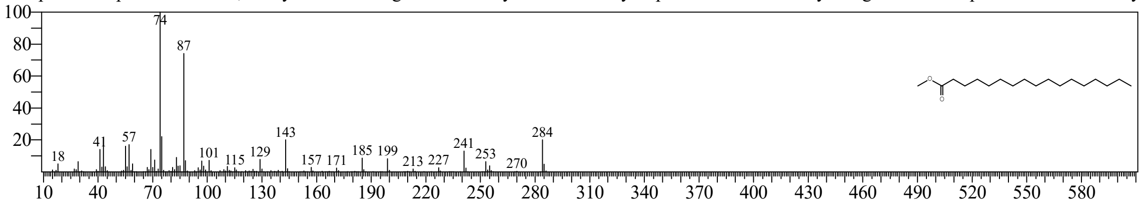

<< Target >>
Line#:6 R.Time:24.092(Scan#:1692) MassPeaks:310
RawMode:Averaged 24.083-24.100(1691-1693) BasePeak:55.10(788)
BG Mode:Calc. from Peak Group 1 - Event 1

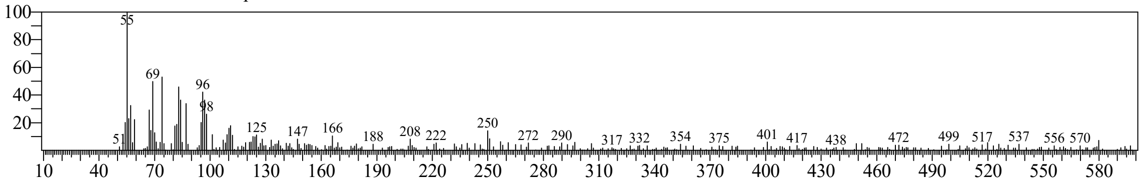

Hit#:1 Entry:125438 Library:NIST17-1.lib
SI:88 Formula:C18H34O2 CAS:0-00-0 MolWeight:282 RetIndex:1986
CompName:cis-10-Heptadecenoic acid, methyl ester

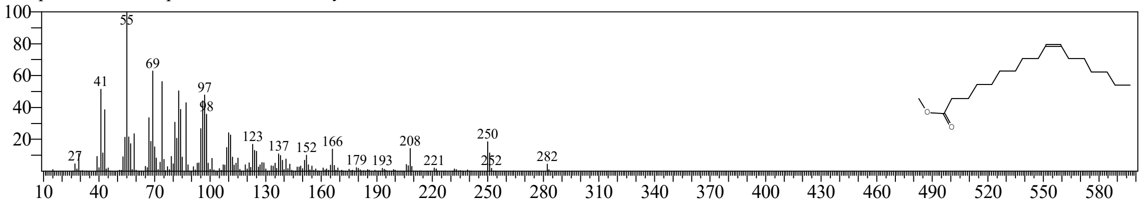

Hit#:2 Entry:115094 Library:NIST14.lib
SI:88 Formula:C18H34O2 CAS:0-00-0 MolWeight:282 RetIndex:1986
CompName:cis-10-Heptadecenoic acid, methyl ester

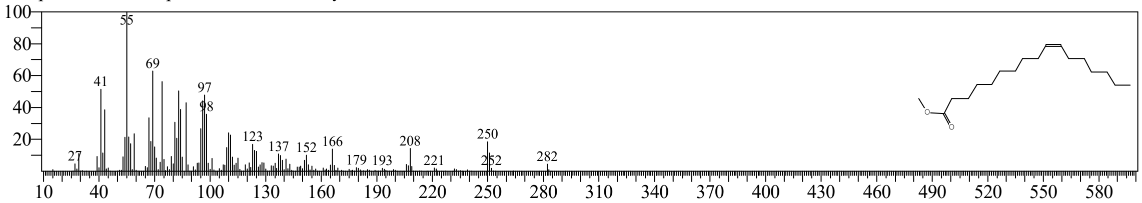

Hit#:3 Entry:31597 Library:NIST17s.lib
SI:87 Formula:C18H34O2 CAS:10152-61-1 MolWeight:282 RetIndex:1941
CompName:Cyclopropaneoctanoic acid, 2-hexyl-, methyl ester $$ Methyl 8-(2-hexylcyclopropyl)octanoate # $$

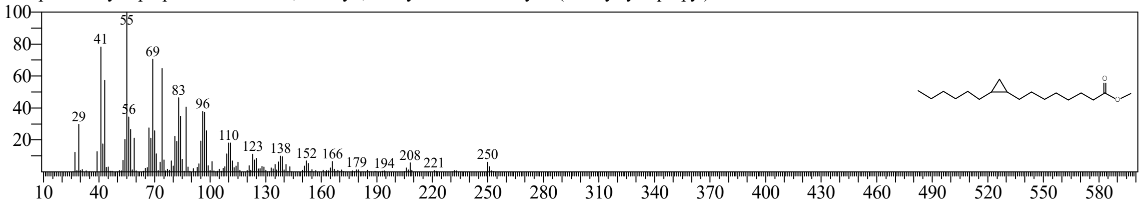

Hit#:4 Entry:27110 Library:NIST14s.lib
SI:87 Formula:C18H34O2 CAS:10152-61-1 MolWeight:282 RetIndex:1941
CompName:Cyclopropaneoctanoic acid, 2-hexyl-, methyl ester $$ Methyl 8-(2-hexylcyclopropyl)octanoate # $$

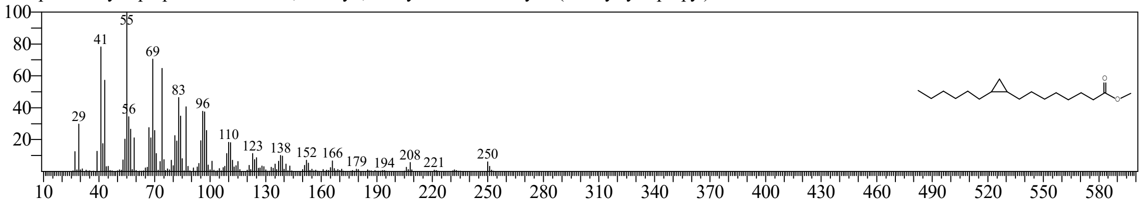

Hit#:5 Entry:125440 Library:NIST17-1.lib
SI:87 Formula:C18H34O2 CAS:0-00-0 MolWeight:282 RetIndex:1986
CompName:Methyl 9-heptadecenoate or 9-17:1

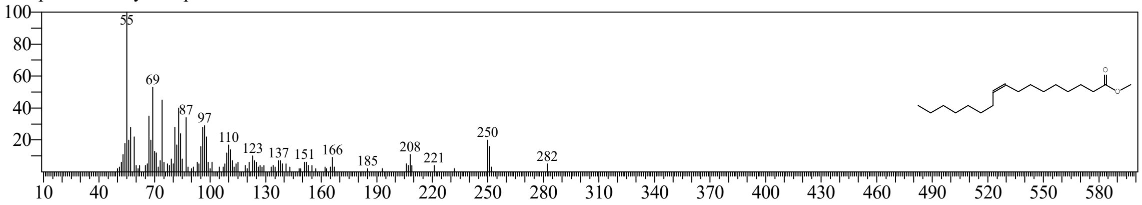

<< Target >>
Line#:7 R.Time:24.867(Scan#:1785) MassPeaks:367
RawMode:Averaged 24.858-24.875(1784-1786) BasePeak:74.00(137298)
BG Mode:Calc. from Peak Group 1 - Event 1

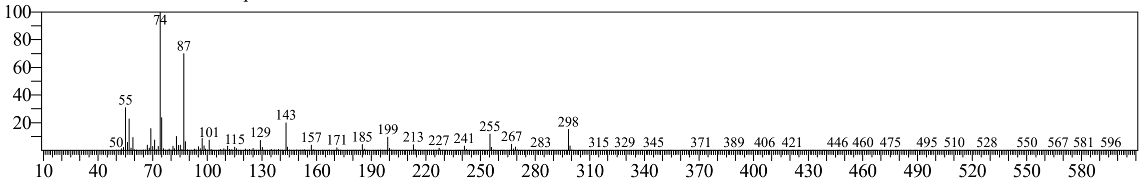

Hit#:1 Entry:129694 Library:NIST14.lib
SI:98 Formula:C19H38O2 CAS:112-61-8 MolWeight:298 RetIndex:2077
CompName:Methyl stearate $$ Octadecanoic acid, methyl ester $$ Stearic acid, methyl ester $$ n-Octadecanoic acid, methyl ester $$ Kemester 9718 $$ Me

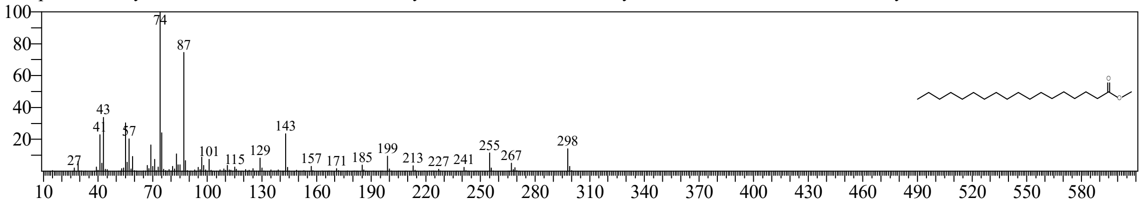

Hit#:2 Entry:141978 Library:NIST17-1.lib
SI:98 Formula:C19H38O2 CAS:112-61-8 MolWeight:298 RetIndex:2077
CompName:Methyl stearate $$ Octadecanoic acid, methyl ester $$ Stearic acid, methyl ester $$ n-Octadecanoic acid, methyl ester $$ Kemester 9718 $$ Me

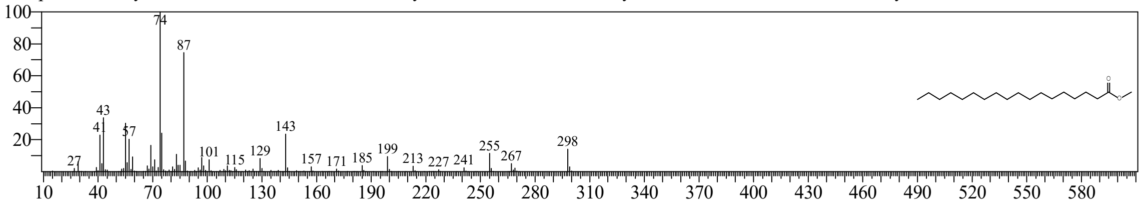

Hit#:3 Entry:32873 Library:NIST17s.lib
SI:97 Formula:C19H38O2 CAS:112-61-8 MolWeight:298 RetIndex:2077
CompName:Methyl stearate $$ Octadecanoic acid, methyl ester $$ Stearic acid, methyl ester $$ n-Octadecanoic acid, methyl ester $$ Kemester 9718 $$ Me

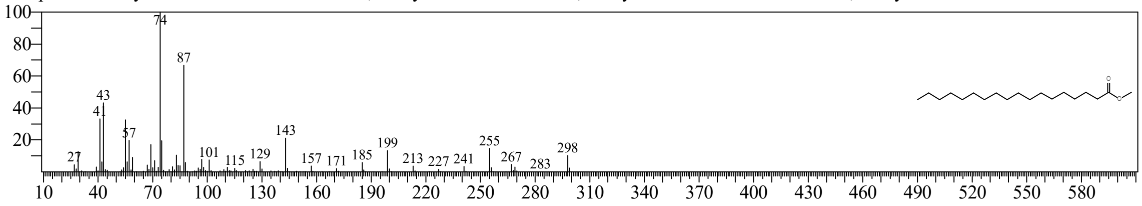

Hit#:4 Entry:28255 Library:NIST14s.lib
SI:97 Formula:C19H38O2 CAS:112-61-8 MolWeight:298 RetIndex:2077
CompName:Methyl stearate $$ Octadecanoic acid, methyl ester $$ Stearic acid, methyl ester $$ n-Octadecanoic acid, methyl ester $$ Kemester 9718 $$ Me

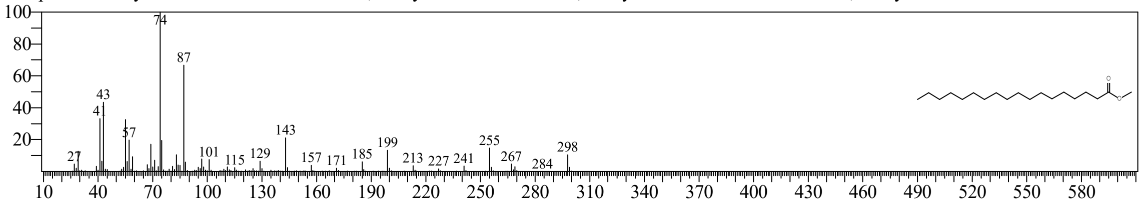

Hit#:5 Entry:28257 Library:NIST14s.lib
SI:96 Formula:C19H38O2 CAS:112-61-8 MolWeight:298 RetIndex:2077
CompName:Methyl stearate $$ Octadecanoic acid, methyl ester $$ Stearic acid, methyl ester $$ n-Octadecanoic acid, methyl ester $$ Kemester 9718 $$ Me

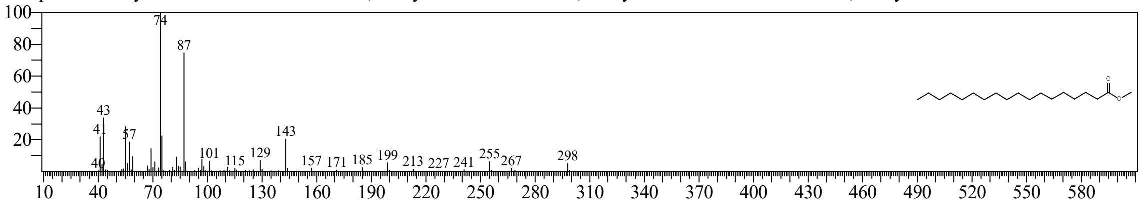

<< Target >>
Line#:8 R.Time:26.258(Scan#:1952) MassPeaks:381
RawMode:Averaged 26.250-26.267(1951-1953) BasePeak:55.05(248428)
BG Mode:Calc. from Peak Group 1 - Event 1

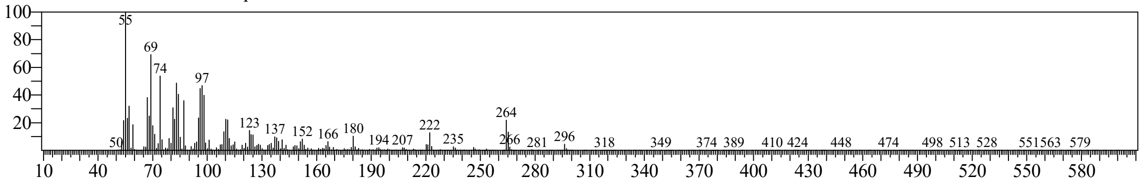

Hit#:1 Entry:32747 Library:NIST17s.lib
SI:97 Formula:C19H36O2 CAS:1937-62-8 MolWeight:296 RetIndex:2085
CompName:9-Octadecenoic acid, methyl ester, (E)- $$ Elaidic acid, methyl ester $$ Methyl elaidate $$ Methyl trans-9-octadecenoate $$ (E)-9-Octadecenoic

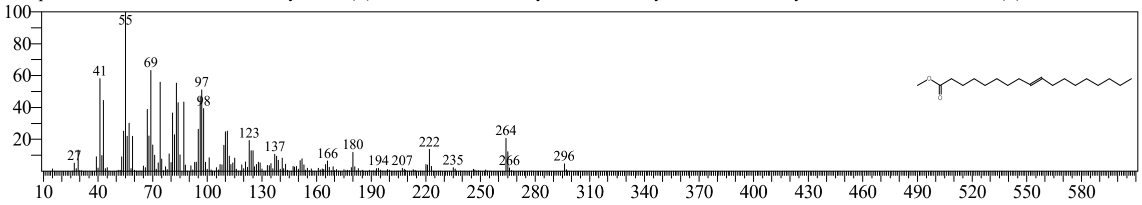

Hit#:2 Entry:28138 Library:NIST14s.lib
SI:97 Formula:C19H36O2 CAS:1937-62-8 MolWeight:296 RetIndex:2085
CompName:9-Octadecenoic acid, methyl ester, (E)- $$ Elaidic acid, methyl ester $$ Methyl elaidate $$ Methyl trans-9-octadecenoate $$ (E)-9-Octadecenoic

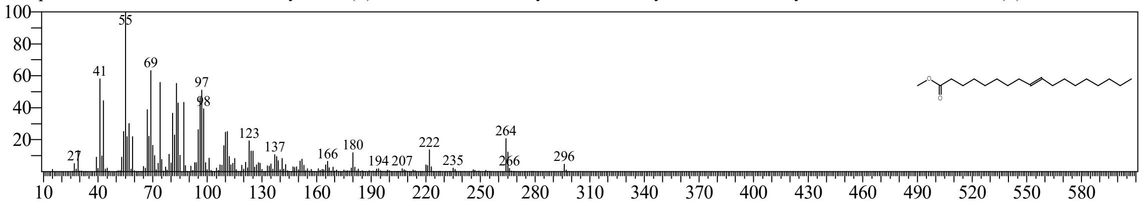

Hit#:3 Entry:28139 Library:NIST14s.lib
SI:96 Formula:C19H36O2 CAS:52380-33-3 MolWeight:296 RetIndex:2085
CompName:11-Octadecenoic acid, methyl ester $$ Methyl 11-octadecenoate $$ Octadec-11-enoic acid, methyl ester $$ Methyl (11E)-11-octadecenoate # $$

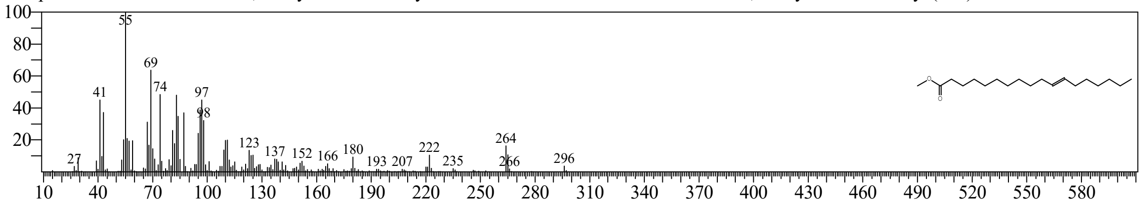

Hit#:4 Entry:32748 Library:NIST17s.lib
SI:96 Formula:C19H36O2 CAS:52380-33-3 MolWeight:296 RetIndex:2085
CompName:11-Octadecenoic acid, methyl ester $$ Methyl 11-octadecenoate $$ Octadec-11-enoic acid, methyl ester $$ Methyl (11E)-11-octadecenoate # $$

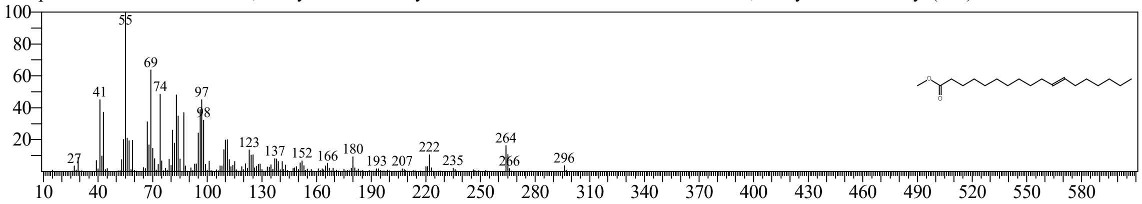

Hit#:5 Entry:139650 Library:NIST17-1.lib
SI:96 Formula:C19H36O2 CAS:112-62-9 MolWeight:296 RetIndex:2085
CompName:9-Octadecenoic acid (Z)-, methyl ester $$ Oleic acid, methyl ester $$ Emery oleic acid ester 2301 $$ Methyl cis-9-octadecenoate $$ Methyl ole

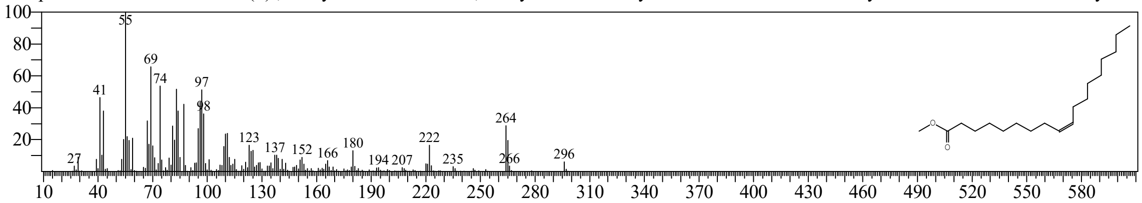

<< Target >>
Line#:9 R.Time:26.375(Scan#:1966) MassPeaks:356
RawMode:Averaged 26.367-26.383(1965-1967) BasePeak:55.05(16281)
BG Mode:Calc. from Peak Group 1 - Event 1

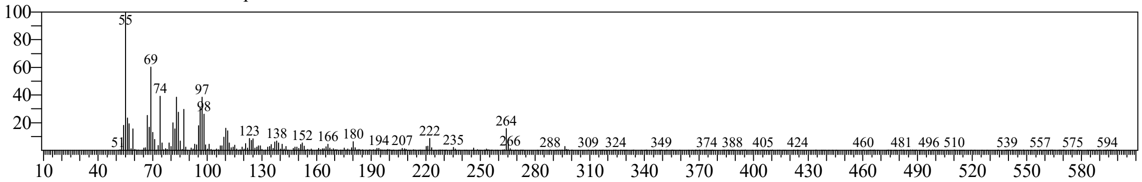

Hit#:1 Entry:32748 Library:NIST17s.lib
SI:95 Formula:C19H36O2 CAS:52380-33-3 MolWeight:296 RetIndex:2085
CompName:11-Octadecenoic acid, methyl ester $$ Methyl 11-octadecenoate $$ Octadec-11-enoic acid, methyl ester $$ Methyl (11E)-11-octadecenoate # $$

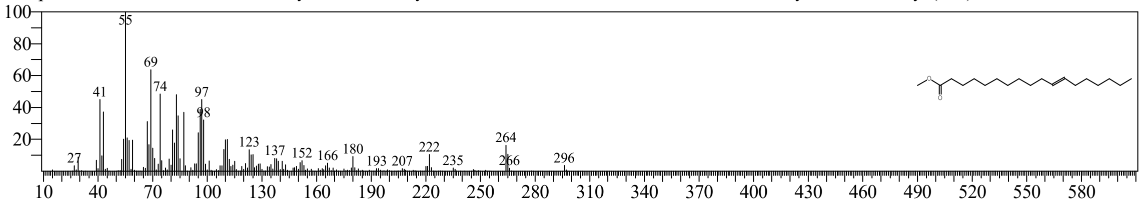

Hit#:2 Entry:28139 Library:NIST14s.lib
SI:95 Formula:C19H36O2 CAS:52380-33-3 MolWeight:296 RetIndex:2085
CompName:11-Octadecenoic acid, methyl ester $$ Methyl 11-octadecenoate $$ Octadec-11-enoic acid, methyl ester $$ Methyl (11E)-11-octadecenoate # $$

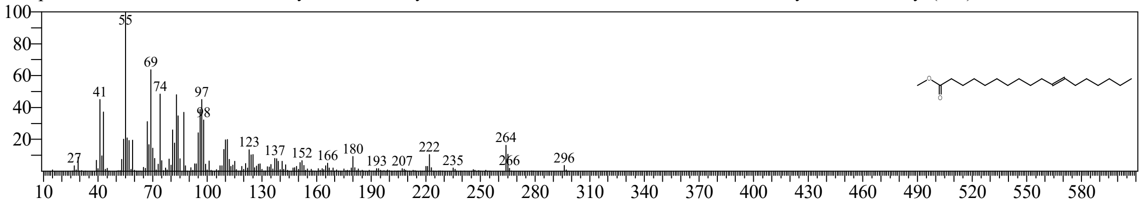

Hit#:3 Entry:139636 Library:NIST17-1.lib
SI:95 Formula:C19H36O2 CAS:13481-95-3 MolWeight:296 RetIndex:2085
CompName:10-Octadecenoic acid, methyl ester $$ Methyl 10-octadecenoate $$

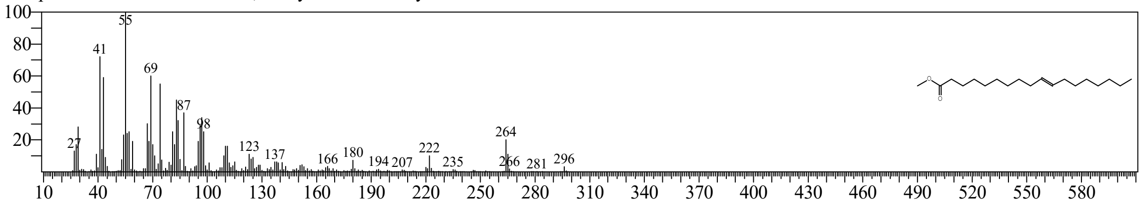

Hit#:4 Entry:127650 Library:NIST14.lib
SI:95 Formula:C19H36O2 CAS:13481-95-3 MolWeight:296 RetIndex:2085
CompName:10-Octadecenoic acid, methyl ester $$ Methyl 10-octadecenoate $$

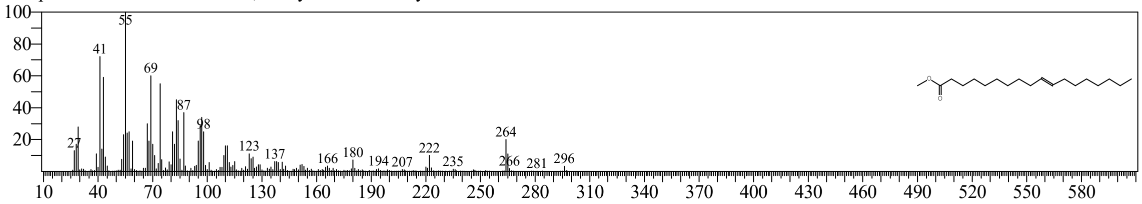

Hit#:5 Entry:127658 Library:NIST14.lib
SI:94 Formula:C19H36O2 CAS:0-00-0 MolWeight:296 RetIndex:2085
CompName:trans-13-Octadecenoic acid, methyl ester

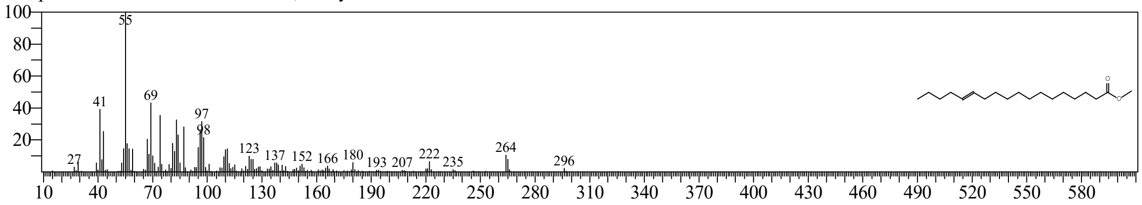

<< Target >>
Line#:10 R.Time:27.725(Scan#:2128) MassPeaks:339
RawMode:Averaged 27.717-27.733(2127-2129) BasePeak:67.00(3594)
BG Mode:Calc. from Peak Group 1 - Event 1

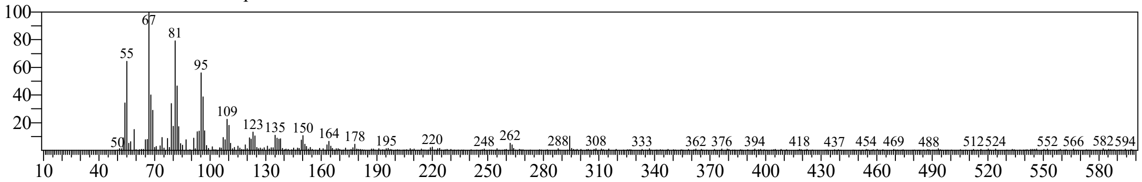

Hit#:1 Entry:27999 Library:NIST14s.lib
SI:96 Formula:C19H34O2 CAS:112-63-0 MolWeight:294 RetIndex:2093
CompName:9,12-Octadecadienoic acid (Z,Z)-, methyl ester $$ Linoleic acid, methyl ester $$ Methyl cis,cis-9,12-octadecadienoate $$ Methyl linoleate $$ M

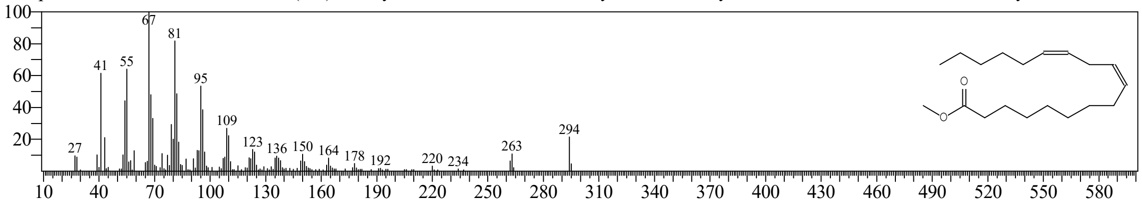

Hit#:2 Entry:32588 Library:NIST17s.lib
SI:96 Formula:C19H34O2 CAS:112-63-0 MolWeight:294 RetIndex:2093
CompName:9,12-Octadecadienoic acid (Z,Z)-, methyl ester $$ Linoleic acid, methyl ester $$ Methyl cis,cis-9,12-octadecadienoate $$ Methyl linoleate $$ M

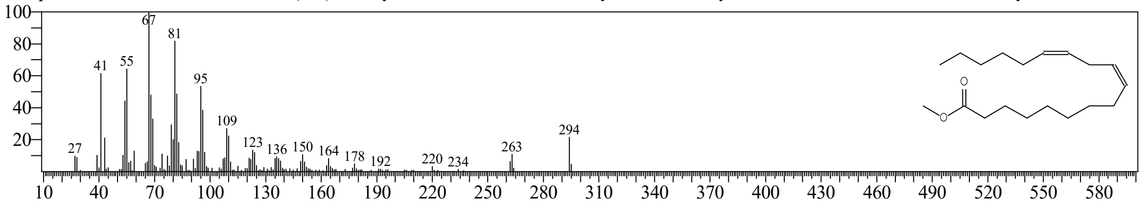

Hit#:3 Entry:125931 Library:NIST14.lib
SI:96 Formula:C19H34O2 CAS:2462-85-3 MolWeight:294 RetIndex:2093
CompName:9,12-Octadecadienoic acid, methyl ester $$ 9,12-Octadecenoic acid, methyl ester $$ Methyl octadeca-9,12-dienoate $$ Methyl 9,12-octadecadi

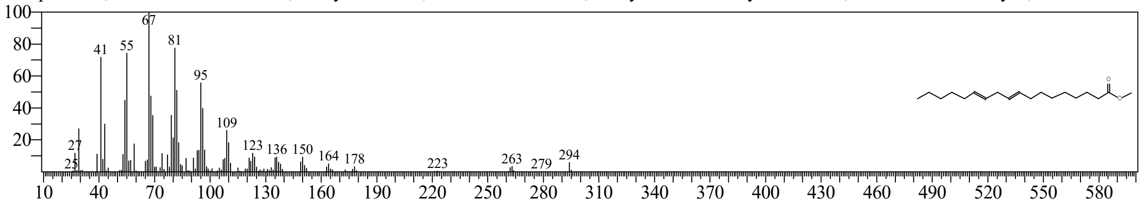

Hit#:4 Entry:137663 Library:NIST17-1.lib
SI:96 Formula:C19H34O2 CAS:2462-85-3 MolWeight:294 RetIndex:2093
CompName:9,12-Octadecadienoic acid, methyl ester $$ 9,12-Octadecenoic acid, methyl ester $$ Methyl octadeca-9,12-dienoate $$ Methyl 9,12-octadecadi

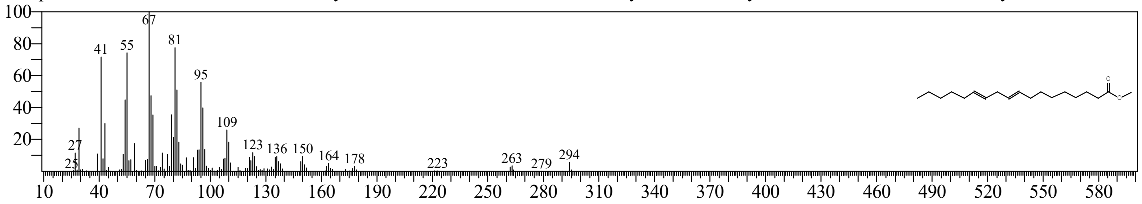

Hit#:5 Entry:32589 Library:NIST17s.lib
SI:96 Formula:C19H34O2 CAS:112-63-0 MolWeight:294 RetIndex:2093
CompName:9,12-Octadecadienoic acid (Z,Z)-, methyl ester $$ Linoleic acid, methyl ester $$ Methyl cis,cis-9,12-octadecadienoate $$ Methyl linoleate $$ M

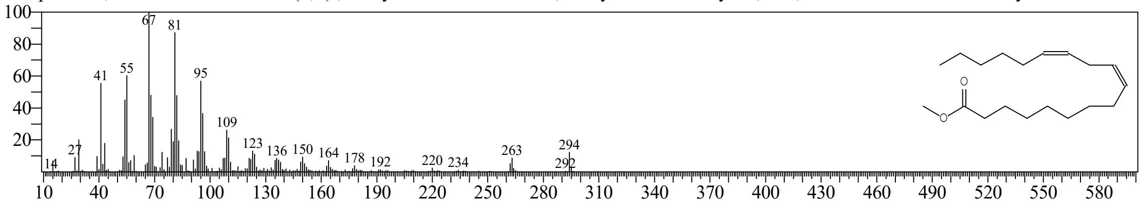

<< Target >>
Line#:11 R.Time:27.933(Scan#:2153) MassPeaks:361
RawMode:Averaged 27.925-27.942(2152-2154) BasePeak:67.05(2269)
BG Mode:Calc. from Peak Group 1 - Event 1

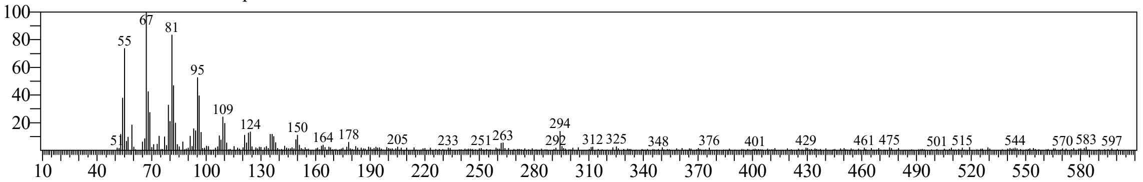

Hit#:1 Entry:137663 Library:NIST17-1.lib
SI:96 Formula:C19H34O2 CAS:2462-85-3 MolWeight:294 RetIndex:2093
CompName:9,12-Octadecadienoic acid, methyl ester $$ 9,12-Octadecenoic acid, methyl ester $$ Methyl octadeca-9,12-dienoate $$ Methyl 9,12-octadecadi

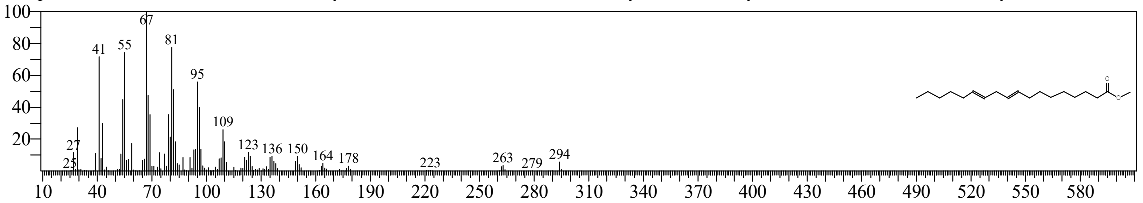

Hit#:2 Entry:125931 Library:NIST14.lib
SI:96 Formula:C19H34O2 CAS:2462-85-3 MolWeight:294 RetIndex:2093
CompName:9,12-Octadecadienoic acid, methyl ester $$ 9,12-Octadecenoic acid, methyl ester $$ Methyl octadeca-9,12-dienoate $$ Methyl 9,12-octadecadi

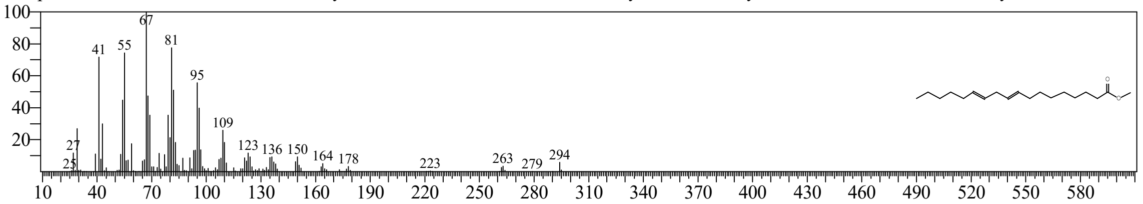

Hit#:3 Entry:32588 Library:NIST17s.lib
SI:96 Formula:C19H34O2 CAS:112-63-0 MolWeight:294 RetIndex:2093
CompName:9,12-Octadecadienoic acid (Z,Z)-, methyl ester $$ Linoleic acid, methyl ester $$ Methyl cis,cis-9,12-octadecadienoate $$ Methyl linoleate $$ M

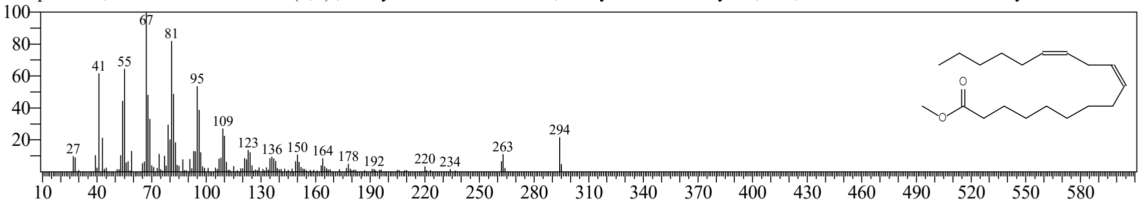

Hit#:4 Entry:27999 Library:NIST14s.lib
SI:96 Formula:C19H34O2 CAS:112-63-0 MolWeight:294 RetIndex:2093
CompName:9,12-Octadecadienoic acid (Z,Z)-, methyl ester $$ Linoleic acid, methyl ester $$ Methyl cis,cis-9,12-octadecadienoate $$ Methyl linoleate $$ M

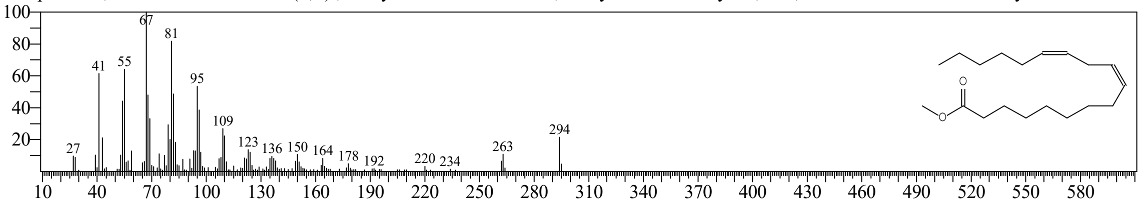

Hit#:5 Entry:32589 Library:NIST17s.lib
SI:95 Formula:C19H34O2 CAS:112-63-0 MolWeight:294 RetIndex:2093
CompName:9,12-Octadecadienoic acid (Z,Z)-, methyl ester $$ Linoleic acid, methyl ester $$ Methyl cis,cis-9,12-octadecadienoate $$ Methyl linoleate $$ M

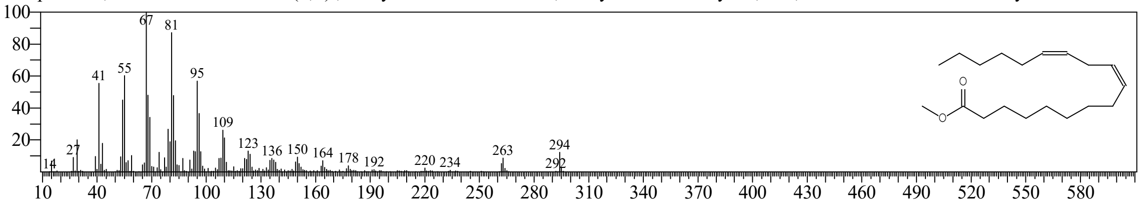

<< Target >>
Line#:12   R.Time:28.367(Scan#:2205) MassPeaks:408
RawMode:Averaged 28.358-28.375(2204-2206) BasePeak:67.05(471045)
BG Mode:Calc. from Peak Group 1 - Event 1

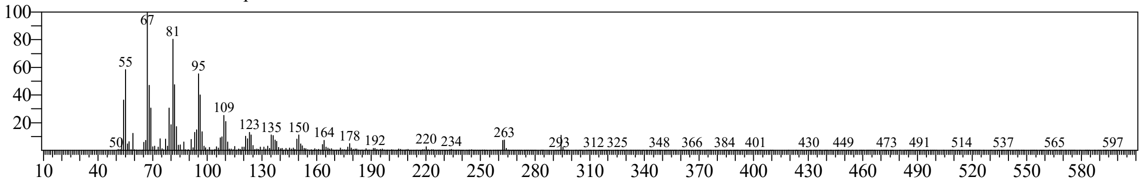

Hit#:1 Entry:32588 Library:NIST17s.lib
SI:97 Formula:C19H34O2 CAS:112-63-0 MolWeight:294 RetIndex:2093
CompName:9,12-Octadecadienoic acid (Z,Z)-, methyl ester $$ Linoleic acid, methyl ester $$ Methyl cis,cis-9,12-octadecadienoate $$ Methyl linoleate $$ M

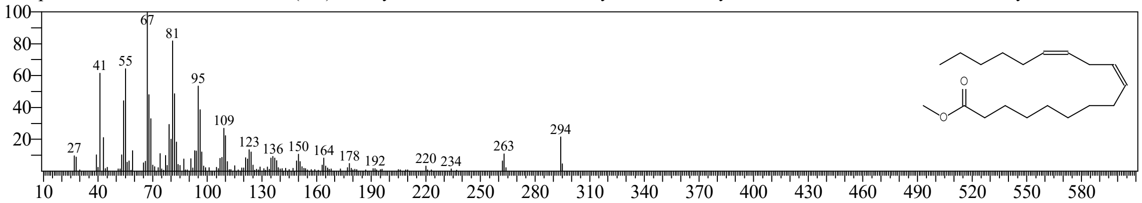

Hit#:2 Entry:27999 Library:NIST14s.lib
SI:97 Formula:C19H34O2 CAS:112-63-0 MolWeight:294 RetIndex:2093
CompName:9,12-Octadecadienoic acid (Z,Z)-, methyl ester $$ Linoleic acid, methyl ester $$ Methyl cis,cis-9,12-octadecadienoate $$ Methyl linoleate $$ M

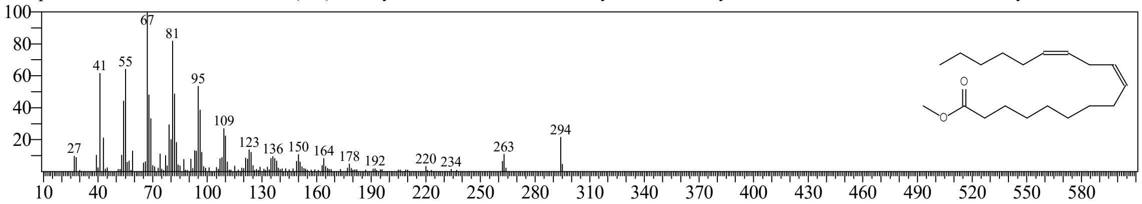

Hit#:3 Entry:32589 Library:NIST17s.lib
SI:97 Formula:C19H34O2 CAS:112-63-0 MolWeight:294 RetIndex:2093
CompName:9,12-Octadecadienoic acid (Z,Z)-, methyl ester $$ Linoleic acid, methyl ester $$ Methyl cis,cis-9,12-octadecadienoate $$ Methyl linoleate $$ M

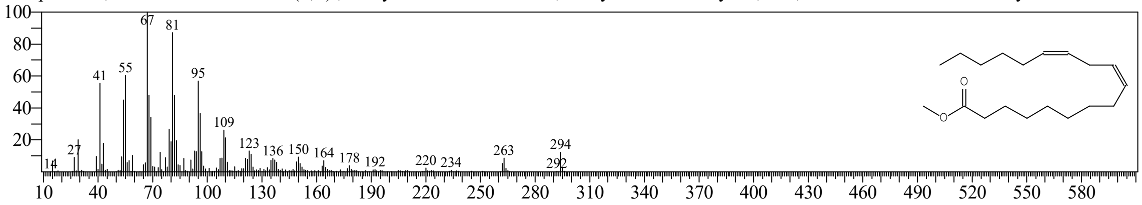

Hit#:4 Entry:28000 Library:NIST14s.lib
SI:97 Formula:C19H34O2 CAS:112-63-0 MolWeight:294 RetIndex:2093
CompName:9,12-Octadecadienoic acid (Z,Z)-, methyl ester $$ Linoleic acid, methyl ester $$ Methyl cis,cis-9,12-octadecadienoate $$ Methyl linoleate $$ M

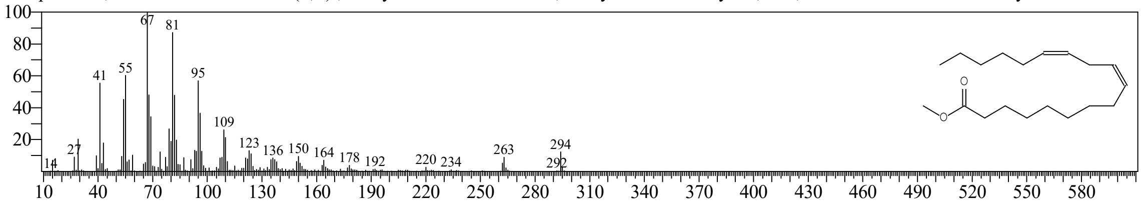

Hit#:5 Entry:125931 Library:NIST14.lib
SI:96 Formula:C19H34O2 CAS:2462-85-3 MolWeight:294 RetIndex:2093
CompName:9,12-Octadecadienoic acid, methyl ester $$ 9,12-Octadecenoic acid, methyl ester $$ Methyl octadeca-9,12-dienoate $$ Methyl 9,12-octadecadi

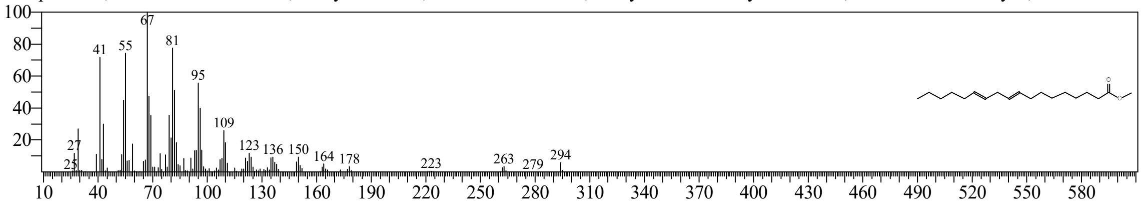

<< Target >>
Line#:13   R.Time:29.258(Scan#:2312) MassPeaks:339
RawMode:Averaged 29.250-29.267(2311-2313) BasePeak:74.00(13758)
BG Mode:Calc. from Peak Group 1 - Event 1

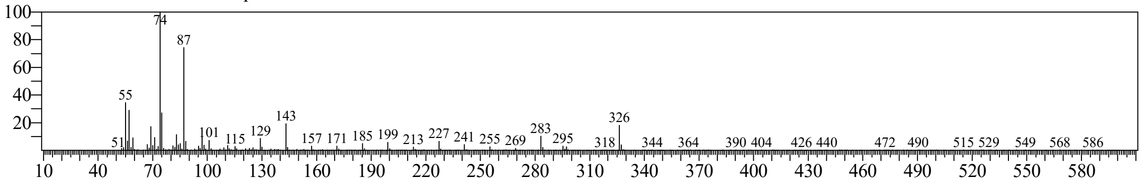

Hit#:1 Entry:154704 Library:NIST14.lib
SI:97 Formula:C21H42O2 CAS:0-00-0 MolWeight:326 RetIndex:2212
CompName:Methyl 18-methylnonadecanoate

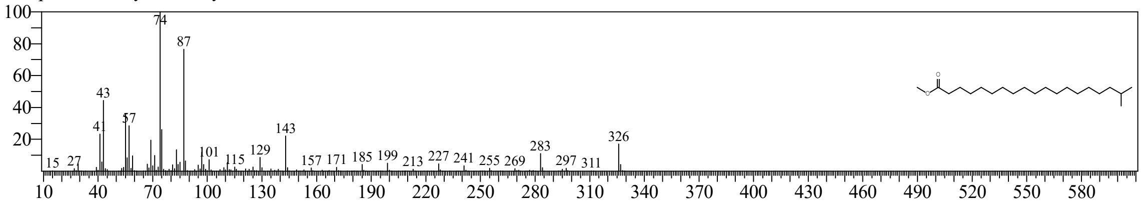

Hit#:2 Entry:170238 Library:NIST17-1.lib
SI:97 Formula:C21H42O2 CAS:0-00-0 MolWeight:326 RetIndex:2212
CompName:Methyl 18-methylnonadecanoate

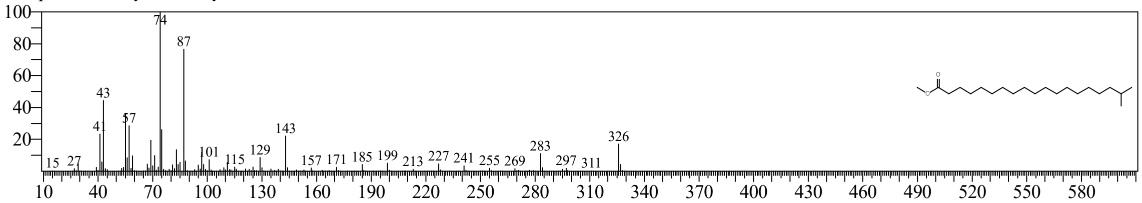

Hit#:3 Entry:34726 Library:NIST17s.lib
SI:97 Formula:C21H42O2 CAS:1120-28-1 MolWeight:326 RetIndex:2276
CompName:Eicosanoic acid, methyl ester $$ Methyl arachisate $$ Methyl eicosanoate $$ Arachidic acid methyl ester $$ Kemester 2050 $$ Methyl aracidat

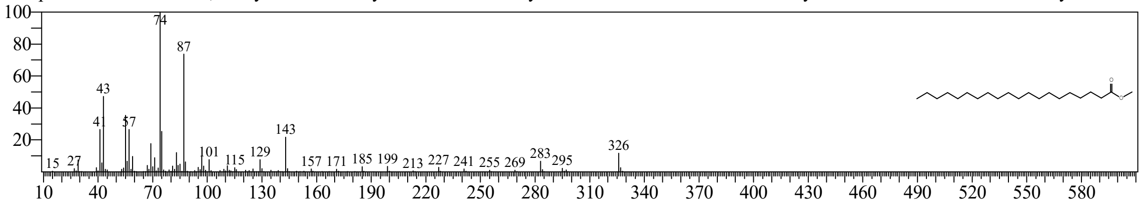

Hit#:4 Entry:34724 Library:NIST17s.lib
SI:94 Formula:C21H42O2 CAS:1120-28-1 MolWeight:326 RetIndex:2276
CompName:Eicosanoic acid, methyl ester $$ Methyl arachisate $$ Methyl eicosanoate $$ Arachidic acid methyl ester $$ Kemester 2050 $$ Methyl aracidat

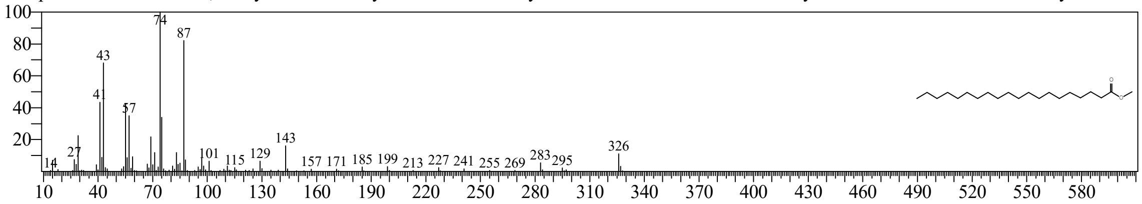

Hit#:5 Entry:29931 Library:NIST14s.lib
SI:94 Formula:C21H42O2 CAS:1120-28-1 MolWeight:326 RetIndex:2276
CompName:Eicosanoic acid, methyl ester $$ Methyl arachisate $$ Methyl eicosanoate $$ Arachidic acid methyl ester $$ Kemester 2050 $$ Methyl aracidat

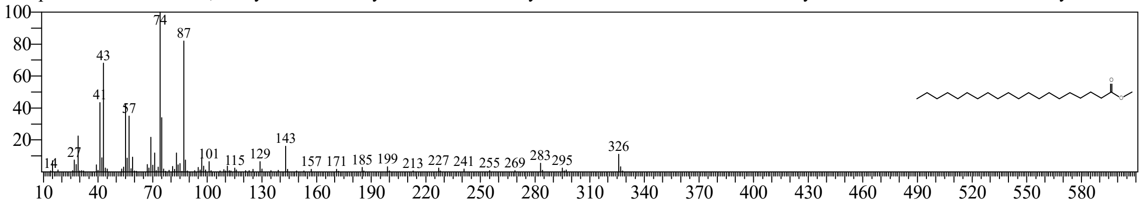

<< Target >>
Line#:14 R.Time:29.817(Scan#:2379) MassPeaks:407
RawMode:Averaged 29.808-29.825(2378-2380) BasePeak:79.05(7875)
BG Mode:Calc. from Peak Group 1 - Event 1

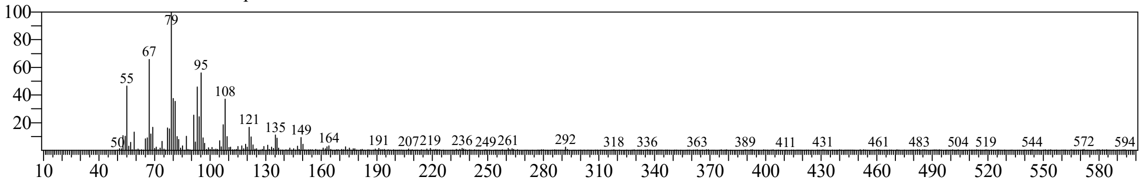

Hit#:1 Entry:29609 Library:NIST14s.lib
SI:95 Formula:C21H36O2 CAS:55682-88-7 MolWeight:320 RetIndex:2300
CompName:11,14,17-Eicosatrienoic acid, methyl ester $$ Methyl 11,14,17-icosatrienoate $$ Methyl 11,14,17-eicosatrienoate $$

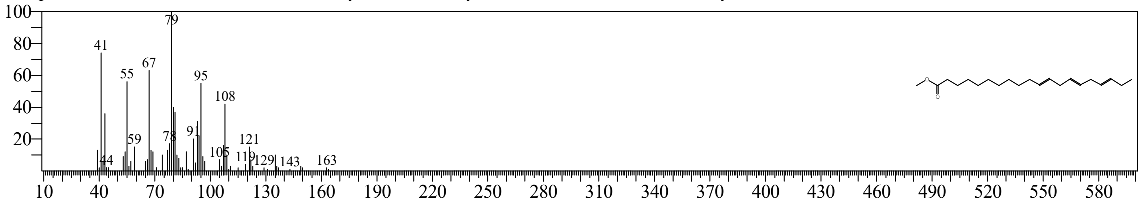

Hit#:2 Entry:34362 Library:NIST17s.lib
SI:95 Formula:C21H36O2 CAS:55682-88-7 MolWeight:320 RetIndex:2300
CompName:11,14,17-Eicosatrienoic acid, methyl ester $$ Methyl 11,14,17-icosatrienoate $$ Methyl 11,14,17-eicosatrienoate $$

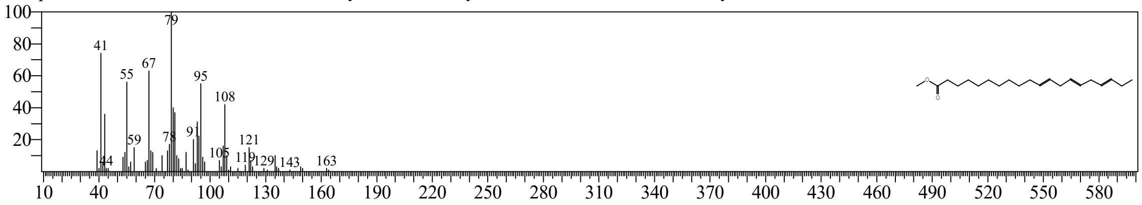

Hit#:3 Entry:32437 Library:NIST17s.lib
SI:94 Formula:C19H32O2 CAS:301-00-8 MolWeight:292 RetIndex:2101
CompName:9,12,15-Octadecatrienoic acid, methyl ester, (Z,Z,Z)- $$ Linolenic acid, methyl ester $$ Methyl all-cis-9,12,15-octadecatrienoate $$ Methyl lino

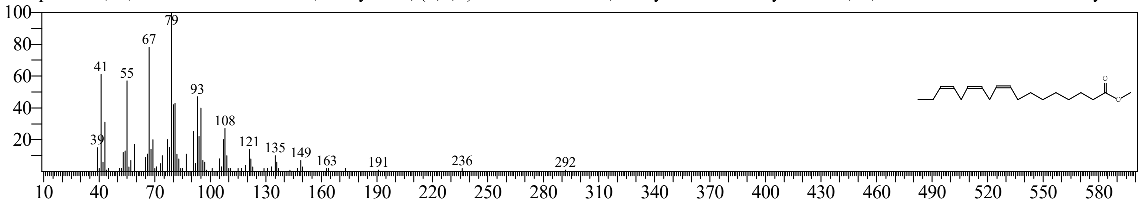

Hit#:4 Entry:27872 Library:NIST14s.lib
SI:94 Formula:C19H32O2 CAS:301-00-8 MolWeight:292 RetIndex:2101
CompName:9,12,15-Octadecatrienoic acid, methyl ester, (Z,Z,Z)- $$ Linolenic acid, methyl ester $$ Methyl all-cis-9,12,15-octadecatrienoate $$ Methyl lino

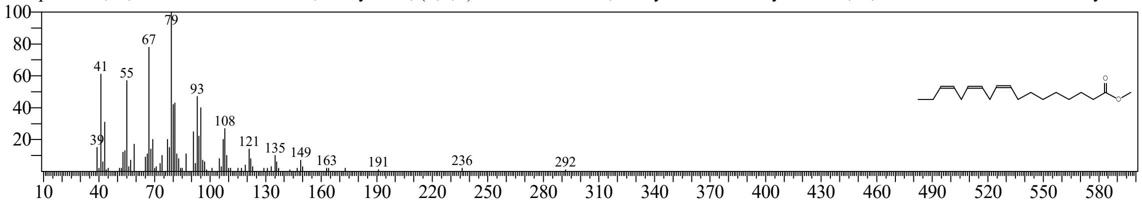

Hit#:5 Entry:135724 Library:NIST17-1.lib
SI:94 Formula:C19H32O2 CAS:301-00-8 MolWeight:292 RetIndex:2101
CompName:9,12,15-Octadecatrienoic acid, methyl ester, (Z,Z,Z)- $$ Linolenic acid, methyl ester $$ Methyl all-cis-9,12,15-octadecatrienoate $$ Methyl lino

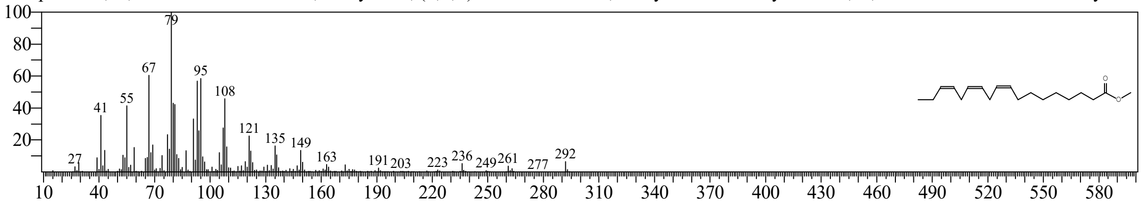

<< Target >>
Line#:15 R.Time:30.292(Scan#:2436) MassPeaks:376
RawMode:Averaged 30.283-30.300(2435-2437) BasePeak:79.05(6235)
BG Mode:Calc. from Peak Group 1 - Event 1

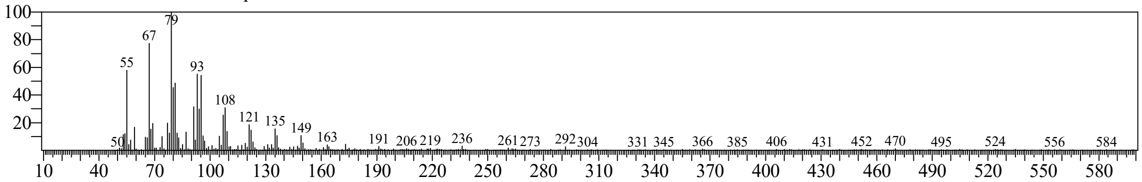

Hit#:1 Entry:135724 Library:NIST17-1.lib
SI:96 Formula:C19H32O2 CAS:301-00-8 MolWeight:292 RetIndex:2101
CompName:9,12,15-Octadecatrienoic acid, methyl ester, (Z,Z,Z)- $$ Linolenic acid, methyl ester $$ Methyl all-cis-9,12,15-octadecatrienoate $$ Methyl lino

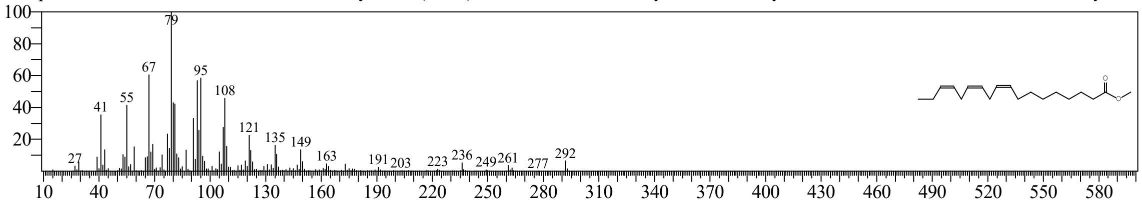

Hit#:2 Entry:124228 Library:NIST14.lib
SI:96 Formula:C19H32O2 CAS:301-00-8 MolWeight:292 RetIndex:2101
CompName:9,12,15-Octadecatrienoic acid, methyl ester, (Z,Z,Z)- $$ Linolenic acid, methyl ester $$ Methyl all-cis-9,12,15-octadecatrienoate $$ Methyl lino

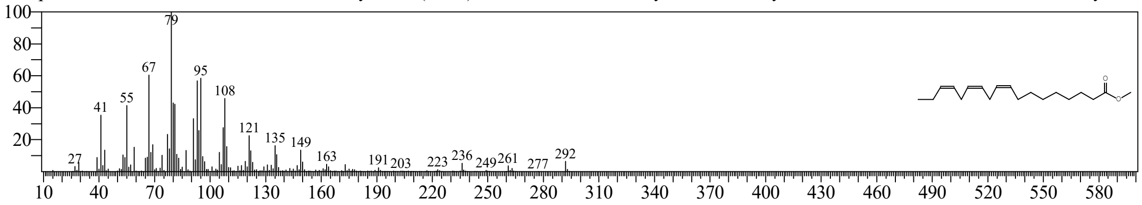

Hit#:3 Entry:27872 Library:NIST14s.lib
SI:95 Formula:C19H32O2 CAS:301-00-8 MolWeight:292 RetIndex:2101
CompName:9,12,15-Octadecatrienoic acid, methyl ester, (Z,Z,Z)- $$ Linolenic acid, methyl ester $$ Methyl all-cis-9,12,15-octadecatrienoate $$ Methyl lino

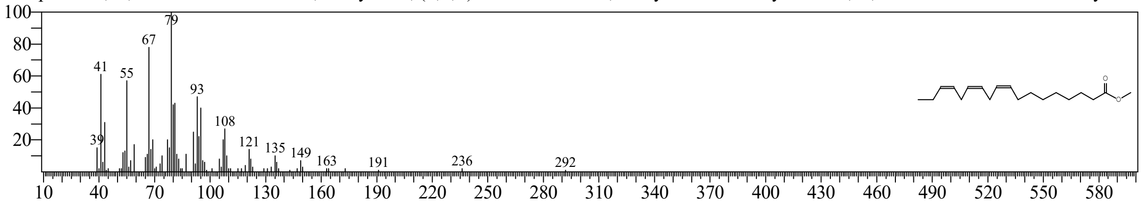

Hit#:4 Entry:32437 Library:NIST17s.lib
SI:95 Formula:C19H32O2 CAS:301-00-8 MolWeight:292 RetIndex:2101
CompName:9,12,15-Octadecatrienoic acid, methyl ester, (Z,Z,Z)- $$ Linolenic acid, methyl ester $$ Methyl all-cis-9,12,15-octadecatrienoate $$ Methyl lino

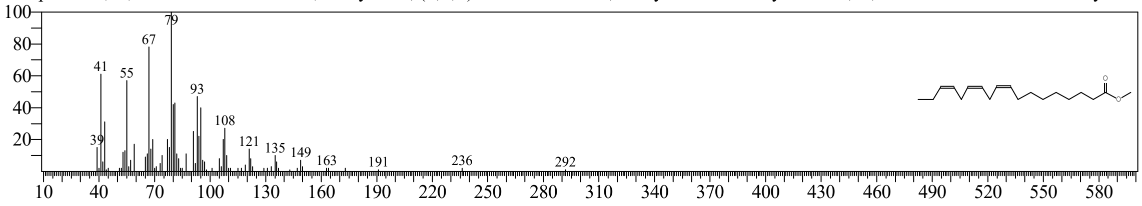

Hit#:5 Entry:121536 Library:NIST17-1.lib
SI:94 Formula:C18H30O2 CAS:0-00-0 MolWeight:278 RetIndex:2002
CompName:Methyl 8,11,14-heptadecatrienoate

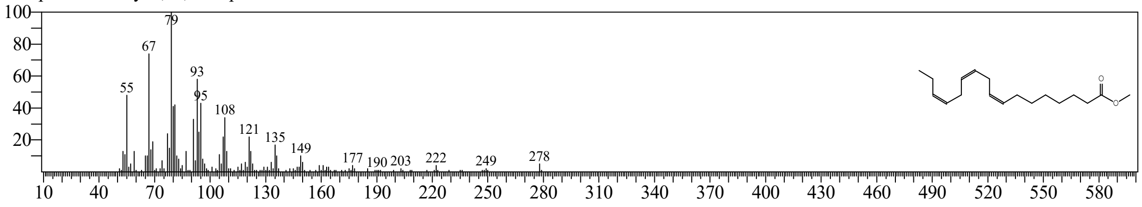

<< Target >>
Line#:16 R.Time:30.600(Scan#:2473) MassPeaks:365
RawMode:Averaged 30.592-30.608(2472-2474) BasePeak:79.05(74920)
BG Mode:Calc. from Peak Group 1 - Event 1

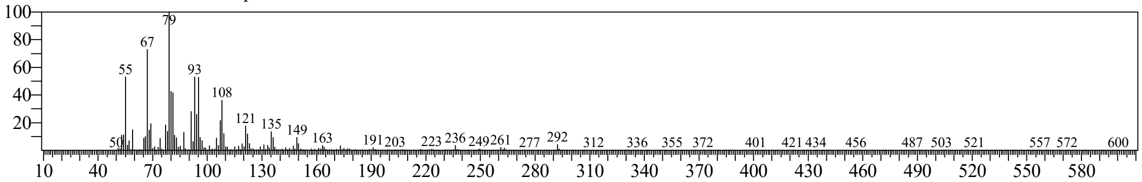

Hit#:1 Entry:135724 Library:NIST17-1.lib
SI:96 Formula:C19H32O2 CAS:301-00-8 MolWeight:292 RetIndex:2101
CompName:9,12,15-Octadecatrienoic acid, methyl ester, (Z,Z,Z)- $$ Linolenic acid, methyl ester $$ Methyl all-cis-9,12,15-octadecatrienoate $$ Methyl lino

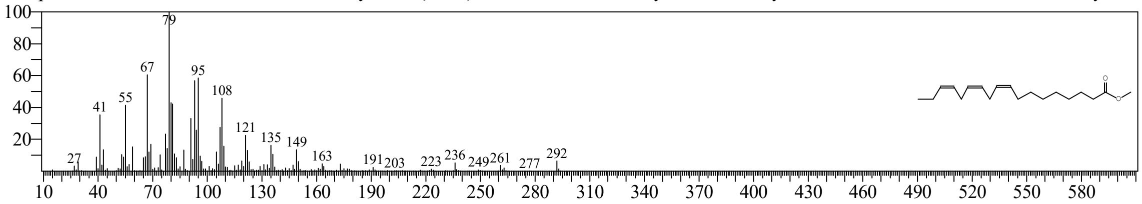

Hit#:2 Entry:124228 Library:NIST14.lib
SI:96 Formula:C19H32O2 CAS:301-00-8 MolWeight:292 RetIndex:2101
CompName:9,12,15-Octadecatrienoic acid, methyl ester, (Z,Z,Z)- $$ Linolenic acid, methyl ester $$ Methyl all-cis-9,12,15-octadecatrienoate $$ Methyl lino

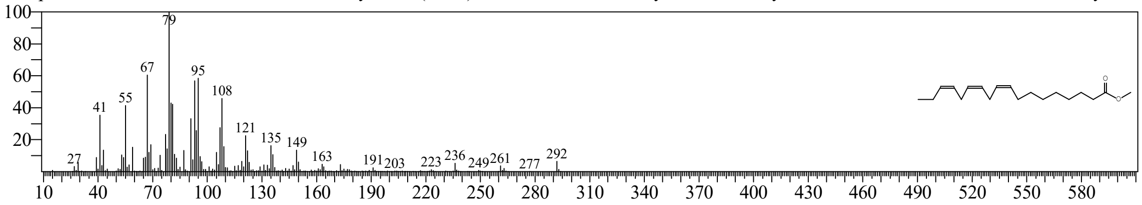

Hit#:3 Entry:32438 Library:NIST17s.lib
SI:95 Formula:C19H32O2 CAS:301-00-8 MolWeight:292 RetIndex:2101
CompName:9,12,15-Octadecatrienoic acid, methyl ester, (Z,Z,Z)- $$ Linolenic acid, methyl ester $$ Methyl all-cis-9,12,15-octadecatrienoate $$ Methyl lino

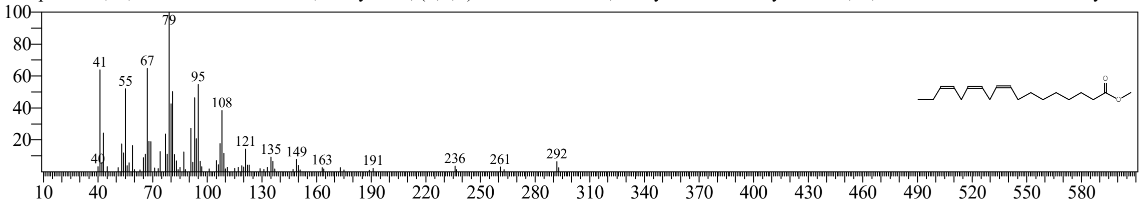

Hit#:4 Entry:27872 Library:NIST14s.lib
SI:95 Formula:C19H32O2 CAS:301-00-8 MolWeight:292 RetIndex:2101
CompName:9,12,15-Octadecatrienoic acid, methyl ester, (Z,Z,Z)- $$ Linolenic acid, methyl ester $$ Methyl all-cis-9,12,15-octadecatrienoate $$ Methyl lino

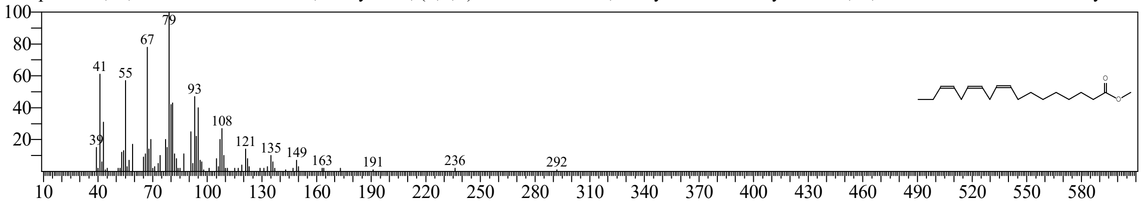

Hit#:5 Entry:32437 Library:NIST17s.lib
SI:95 Formula:C19H32O2 CAS:301-00-8 MolWeight:292 RetIndex:2101
CompName:9,12,15-Octadecatrienoic acid, methyl ester, (Z,Z,Z)- $$ Linolenic acid, methyl ester $$ Methyl all-cis-9,12,15-octadecatrienoate $$ Methyl lino

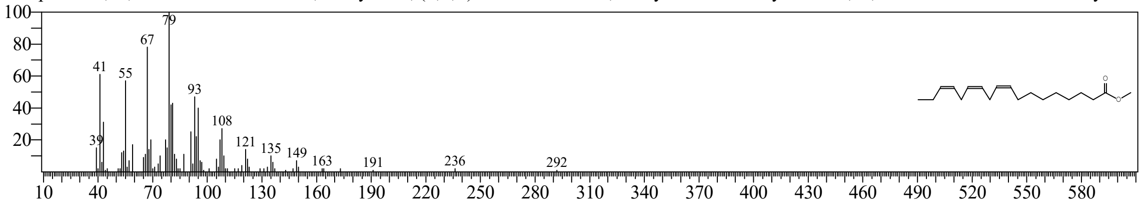

<< Target >>
Line#:17 R.Time:30.925(Scan#:2512) MassPeaks:326
RawMode:Averaged 30.917-30.933(2511-2513) BasePeak:67.00(4330)
BG Mode:Calc. from Peak Group 1 - Event 1

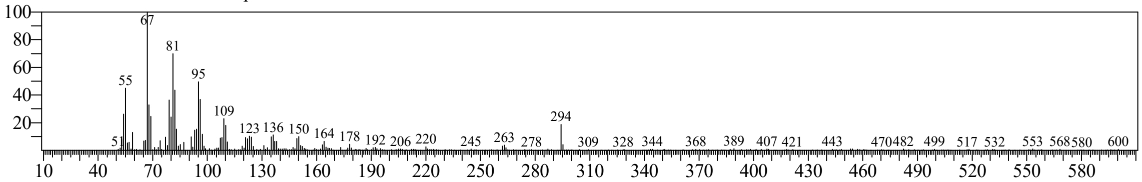

Hit#:1 Entry:137666 Library:NIST17-1.lib
SI:97 Formula:C19H34O2 CAS:13058-52-1 MolWeight:294 RetIndex:2093
CompName:Methyl 9-cis,11-trans-octadecadienoate

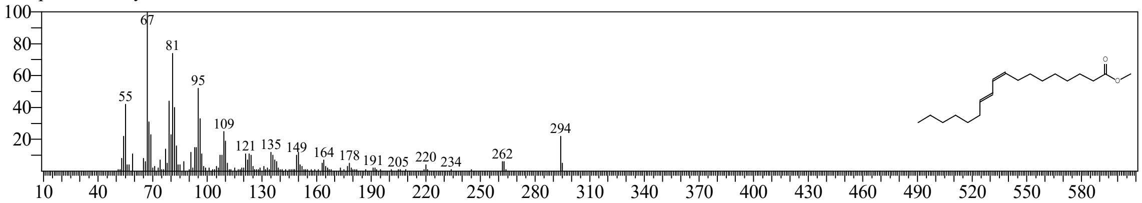

Hit#:2 Entry:137664 Library:NIST17-1.lib
SI:97 Formula:C19H34O2 CAS:0-00-0 MolWeight:294 RetIndex:2093
CompName:Methyl 10-trans,12-cis-octadecadienoate

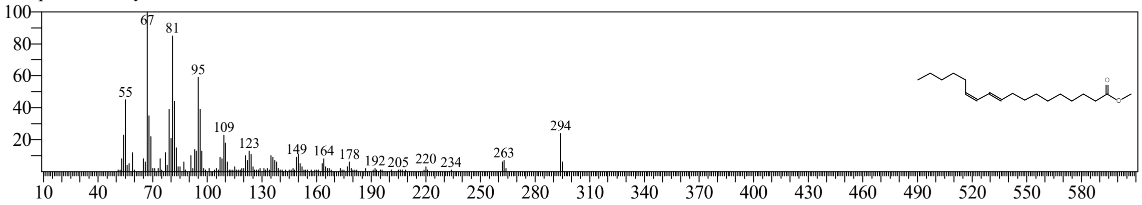

Hit#:3 Entry:125933 Library:NIST14.lib
SI:97 Formula:C19H34O2 CAS:0-00-0 MolWeight:294 RetIndex:2093
CompName:Methyl 10-trans,12-cis-octadecadienoate

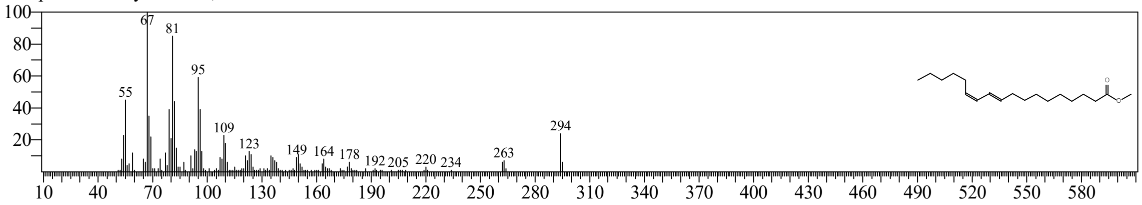

Hit#:4 Entry:125932 Library:NIST14.lib
SI:97 Formula:C19H34O2 CAS:0-00-0 MolWeight:294 RetIndex:2093
CompName:Methyl 9-cis,11-trans-octadecadienoate

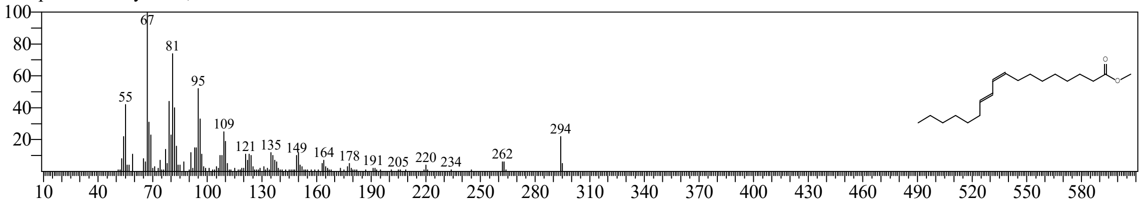

Hit#:5 Entry:32587 Library:NIST17s.lib
SI:96 Formula:C19H34O2 CAS:13038-47-6 MolWeight:294 RetIndex:2093
CompName:9,11-Octadecadienoic acid, methyl ester, (E,E)- $$ Methyl trans-9,trans-11-octadecadienoate $$ Methyl trans,trans-9,11-octadecadienoate $$ M

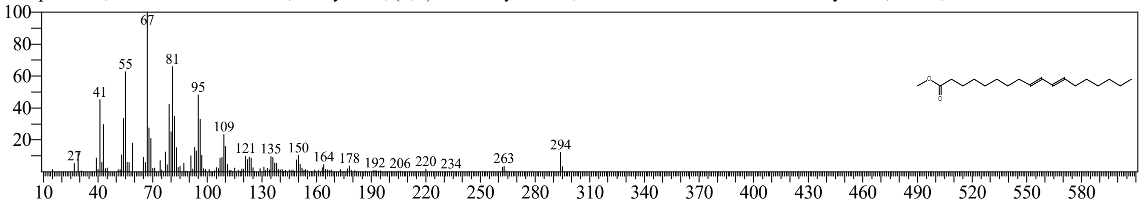

<< Target >>
Line#:18 R.Time:31.258(Scan#:2552) MassPeaks:352
RawMode:Averaged 31.250-31.267(2551-2553) BasePeak:67.05(4805)
BG Mode:Calc. from Peak Group 1 - Event 1

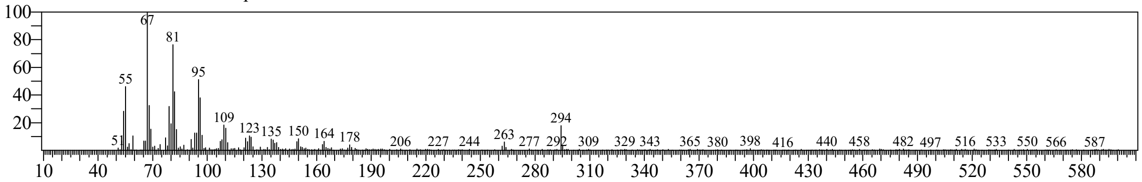

Hit#:1 Entry:137664 Library:NIST17-1.lib
SI:96 Formula:C19H34O2 CAS:0-00-0 MolWeight:294 RetIndex:2093
CompName:Methyl 10-trans,12-cis-octadecadienoate

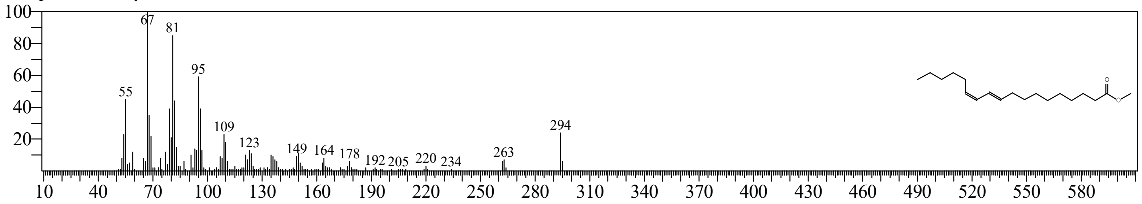

Hit#:2 Entry:125933 Library:NIST14.lib
SI:96 Formula:C19H34O2 CAS:0-00-0 MolWeight:294 RetIndex:2093
CompName:Methyl 10-trans,12-cis-octadecadienoate

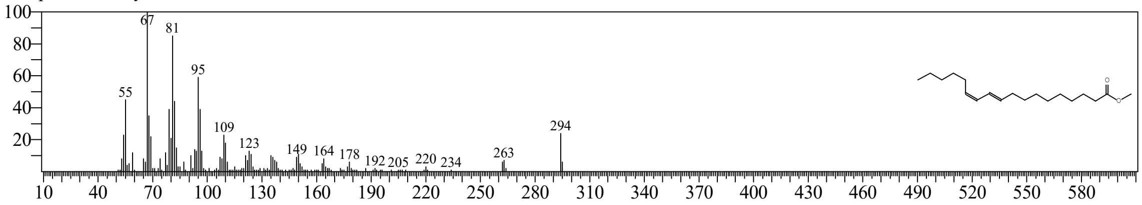

Hit#:3 Entry:137666 Library:NIST17-1.lib
SI:96 Formula:C19H34O2 CAS:13058-52-1 MolWeight:294 RetIndex:2093
CompName:Methyl 9-cis,11-trans-octadecadienoate

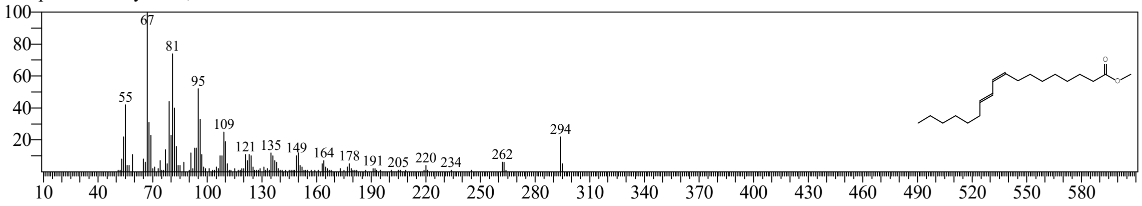

Hit#:4 Entry:125932 Library:NIST14.lib
SI:96 Formula:C19H34O2 CAS:0-00-0 MolWeight:294 RetIndex:2093
CompName:Methyl 9-cis,11-trans-octadecadienoate

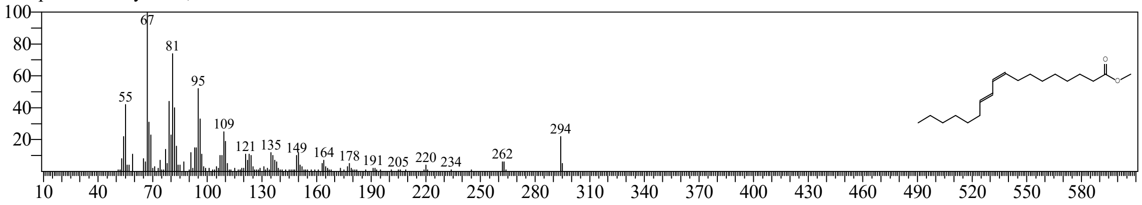

Hit#:5 Entry:32587 Library:NIST17s.lib
SI:94 Formula:C19H34O2 CAS:13038-47-6 MolWeight:294 RetIndex:2093
CompName:9,11-Octadecadienoic acid, methyl ester, (E,E)- $$ Methyl trans-9,trans-11-octadecadienoate $$ Methyl trans,trans-9,11-octadecadienoate $$ M

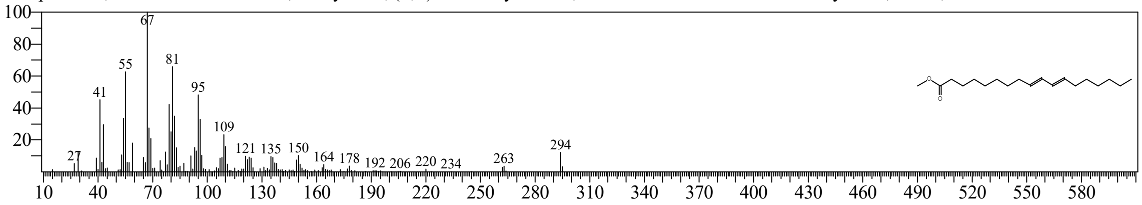

<< Target >>
Line#:19 R.Time:32.950(Scan#:2755) MassPeaks:339
RawMode:Averaged 32.942-32.958(2754-2756) BasePeak:95.10(5455)
BG Mode:Calc. from Peak Group 1 - Event 1

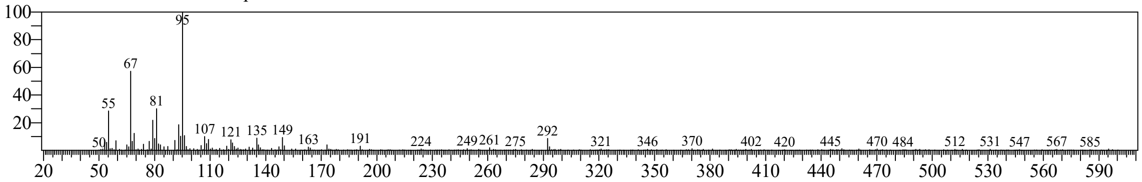

Hit#:1 Entry:23403 Library:NIST14.lib
SI:84 Formula:C12H20 CAS:0-00-0 MolWeight:164 RetIndex:1041
CompName:Cis-3-ethyl-endo-tricyclo[5.2.1.0(2.6)]decane

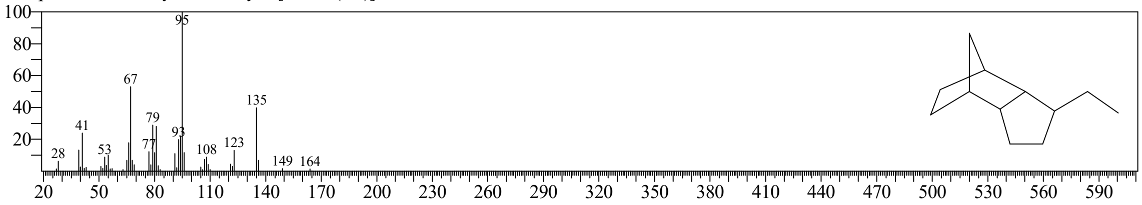

Hit#:2 Entry:24424 Library:NIST17-1.lib
SI:84 Formula:C12H20 CAS:0-00-0 MolWeight:164 RetIndex:1041
CompName:Cis-3-ethyl-endo-tricyclo[5.2.1.0(2.6)]decane

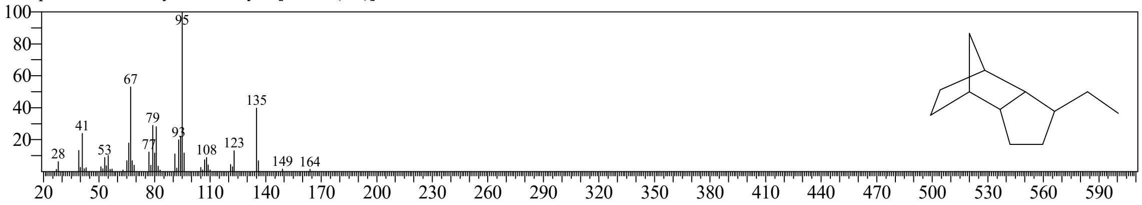

Hit#:3 Entry:16582 Library:NIST17-1.lib
SI:83 Formula:C11H18 CAS:0-00-0 MolWeight:150 RetIndex:1115
CompName:4-Cyclohexenyl-3-methylbutene

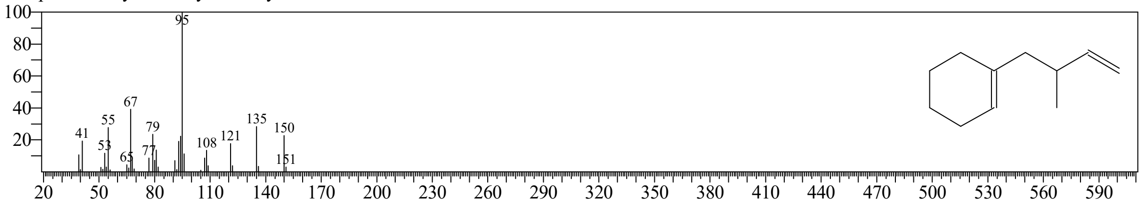

Hit#:4 Entry:16897 Library:NIST14.lib
SI:81 Formula:C10H16O CAS:60485-45-2 MolWeight:152 RetIndex:991
CompName:Santolina epoxide $$ 2-Methyl-2-(3-methyl-1-vinyl-2-butenyl)oxirane # $$

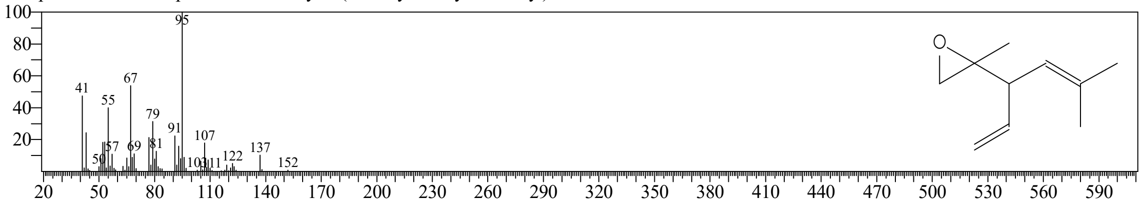

Hit#:5 Entry:17571 Library:NIST17-1.lib
SI:81 Formula:C10H16O CAS:60485-45-2 MolWeight:152 RetIndex:991
CompName:Santolina epoxide $$ 2-Methyl-2-(3-methyl-1-vinyl-2-butenyl)oxirane # $$

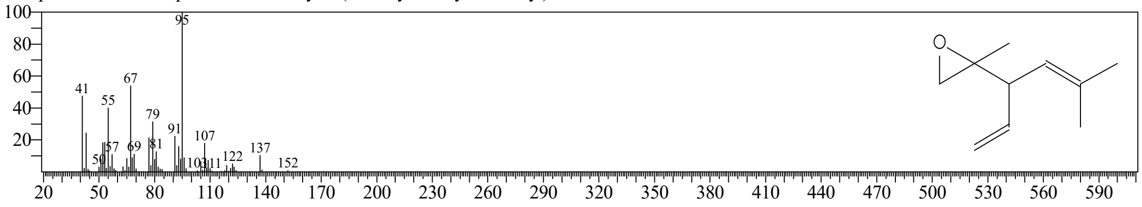

<< Target >>
Line#:20 R.Time:33.492(Scan#:2820) MassPeaks:357
RawMode:Averaged 33.483-33.500(2819-2821) BasePeak:74.05(16057)
BG Mode:Calc. from Peak Group 1 - Event 1

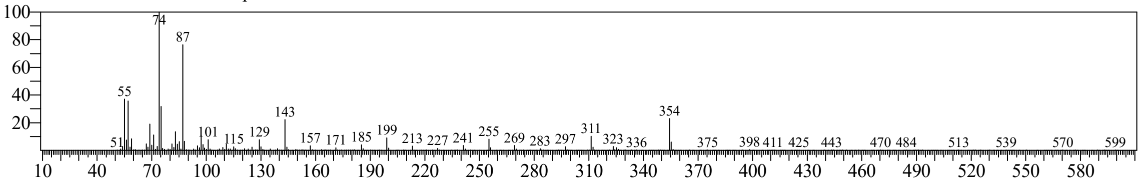

Hit#:1 Entry:196443 Library:NIST17-1.lib
SI:97 Formula:C23H46O2 CAS:929-77-1 MolWeight:354 RetIndex:2475
CompName:Docosanoic acid, methyl ester $$ Behenic acid, methyl ester $$ Methyl behenate $$ Methyl docosanoate $$ n-Docosanoic acid methyl ester $$

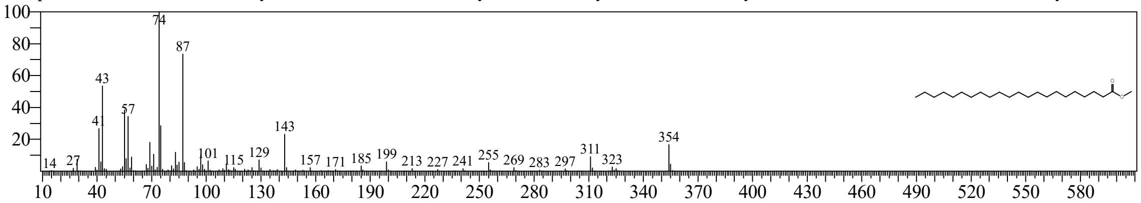

Hit#:2 Entry:178051 Library:NIST14.lib
SI:97 Formula:C23H46O2 CAS:929-77-1 MolWeight:354 RetIndex:2475
CompName:Docosanoic acid, methyl ester $$ Behenic acid, methyl ester $$ Methyl behenate $$ Methyl docosanoate $$ n-Docosanoic acid methyl ester $$

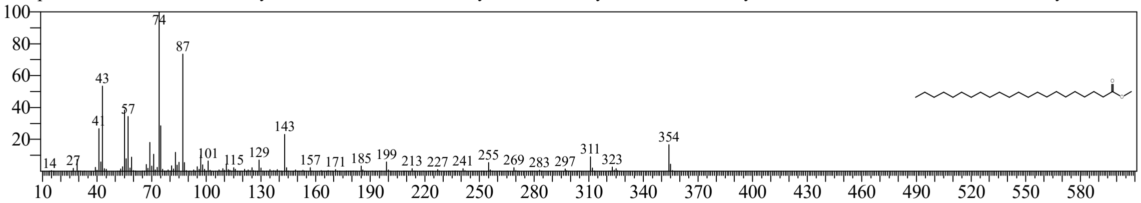

Hit#:3 Entry:196444 Library:NIST17-1.lib
SI:95 Formula:C23H46O2 CAS:0-00-0 MolWeight:354 RetIndex:2411
CompName:Methyl 20-methyl-heneicosanoate

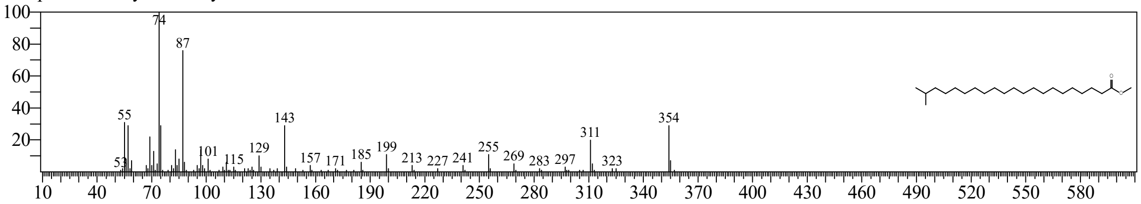

Hit#:4 Entry:178052 Library:NIST14.lib
SI:95 Formula:C23H46O2 CAS:0-00-0 MolWeight:354 RetIndex:2411
CompName:Methyl 20-methyl-heneicosanoate

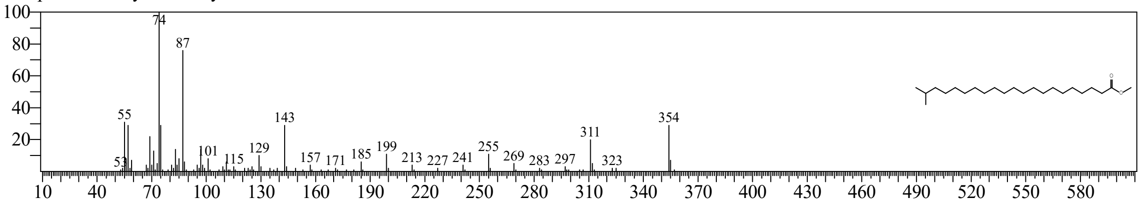

Hit#:5 Entry:31088 Library:NIST14s.lib
SI:94 Formula:C23H46O2 CAS:929-77-1 MolWeight:354 RetIndex:2475
CompName:Docosanoic acid, methyl ester $$ Behenic acid, methyl ester $$ Methyl behenate $$ Methyl docosanoate $$ n-Docosanoic acid methyl ester $$

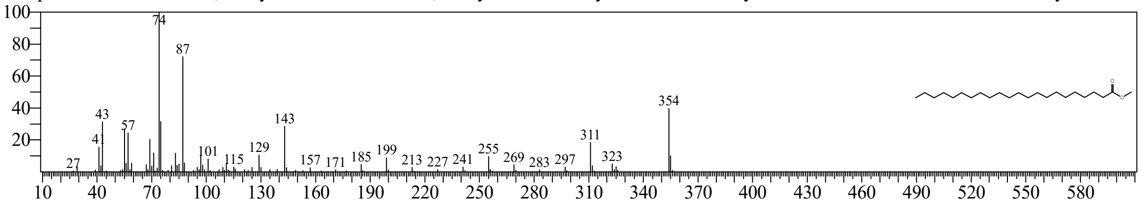

<< Target >>
Line#:21 R.Time:37.783(Scan#:3335) MassPeaks:297
RawMode:Averaged 37.775-37.792(3334-3336) BasePeak:74.05(3802)
BG Mode:Calc. from Peak Group 1 - Event 1

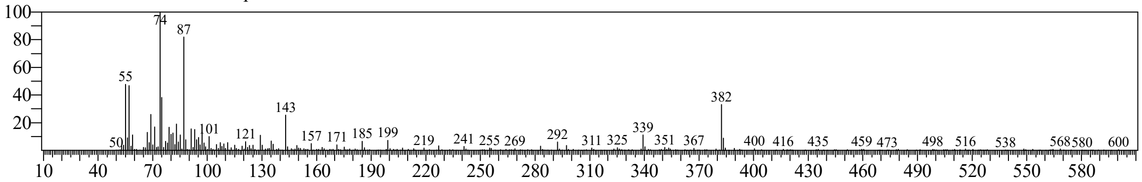

Hit#:1 Entry:31970 Library:NIST14s.lib
SI:85 Formula:C25H50O2 CAS:2442-49-1 MolWeight:382 RetIndex:2674
CompName:Tetracosanoic acid, methyl ester $$ Methyl lignocerate $$ Methyl tetracosanoate $$ Lignoceric acid methyl ester $$

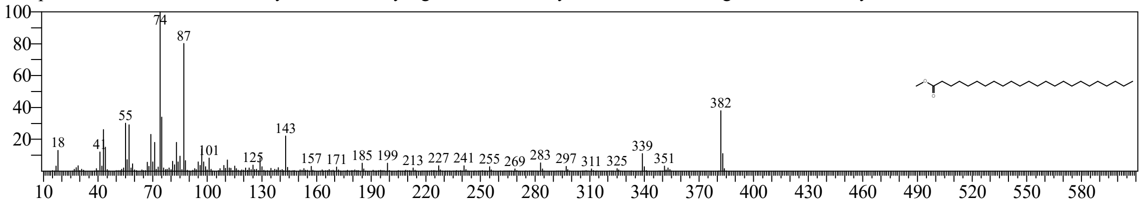

Hit#:2 Entry:37106 Library:NIST17s.lib
SI:85 Formula:C25H50O2 CAS:2442-49-1 MolWeight:382 RetIndex:2674
CompName:Tetracosanoic acid, methyl ester $$ Methyl lignocerate $$ Methyl tetracosanoate $$ Lignoceric acid methyl ester $$

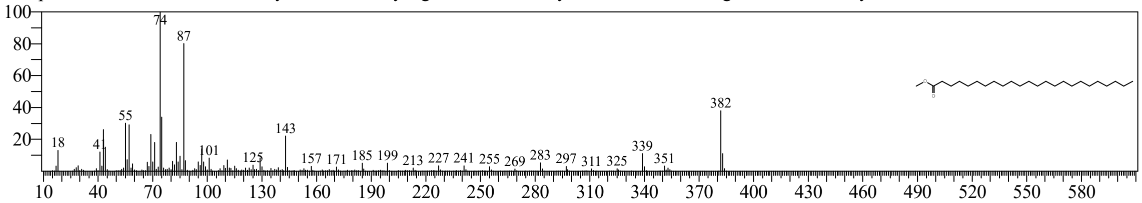

Hit#:3 Entry:31971 Library:NIST14s.lib
SI:82 Formula:C25H50O2 CAS:2442-49-1 MolWeight:382 RetIndex:2674
CompName:Tetracosanoic acid, methyl ester $$ Methyl lignocerate $$ Methyl tetracosanoate $$ Lignoceric acid methyl ester $$

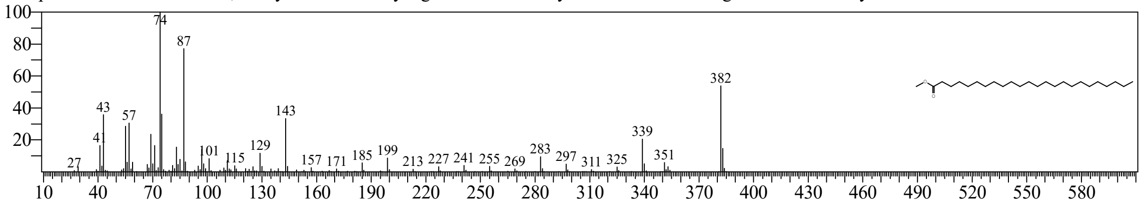

Hit#:4 Entry:37107 Library:NIST17s.lib
SI:82 Formula:C25H50O2 CAS:2442-49-1 MolWeight:382 RetIndex:2674
CompName:Tetracosanoic acid, methyl ester $$ Methyl lignocerate $$ Methyl tetracosanoate $$ Lignoceric acid methyl ester $$

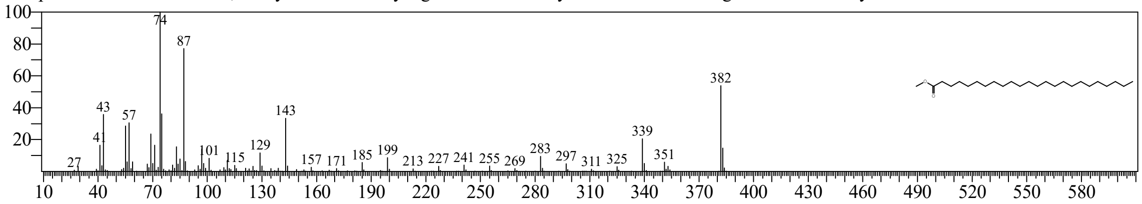

Hit#:5 Entry:33211 Library:NIST14s.lib
SI:81 Formula:C31H62O2 CAS:629-83-4 MolWeight:466 RetIndex:3270
CompName:Triacontanoic acid, methyl ester $$ Methyl melissate $$ Methyl melissicate $$ Methyl triacontanoate $$

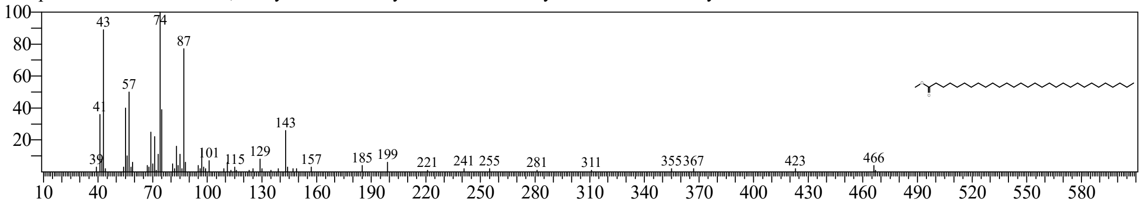

<< Target >>
Line#:22 R.Time:38.300(Scan#:3397) MassPeaks:350
RawMode:Averaged 38.292-38.308(3396-3398) BasePeak:79.05(709)
BG Mode:Calc. from Peak Group 1 - Event 1

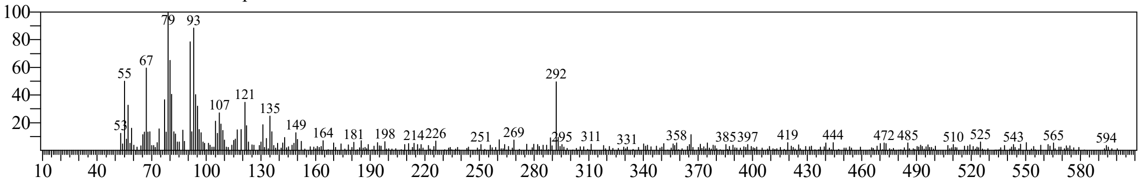

Hit#:1 Entry:124230 Library:NIST14.lib
SI:89 Formula:C19H32O2 CAS:0-00-0 MolWeight:292 RetIndex:2101
CompName:Methyl 9.cis.,11.trans.t,13.trans.-octadecatrienoate

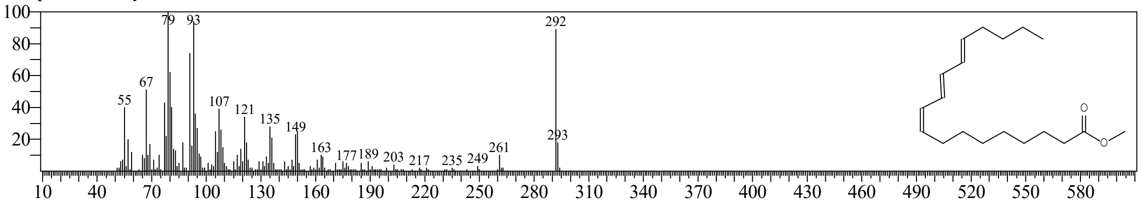

Hit#:2 Entry:135726 Library:NIST17-1.lib
SI:89 Formula:C19H32O2 CAS:0-00-0 MolWeight:292 RetIndex:2101
CompName:Methyl 9.cis.,11.trans.t,13.trans.-octadecatrienoate

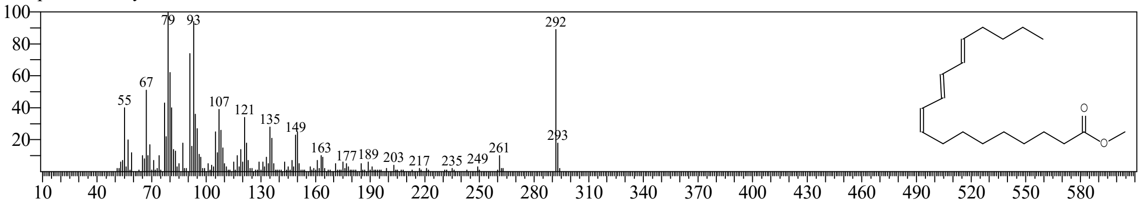

Hit#:3 Entry:135722 Library:NIST17-1.lib
SI:86 Formula:C19H32O2 CAS:0-00-0 MolWeight:292 RetIndex:2101
CompName:Methyl 6-cis,9-cis,11-trans-octadecatrienoate

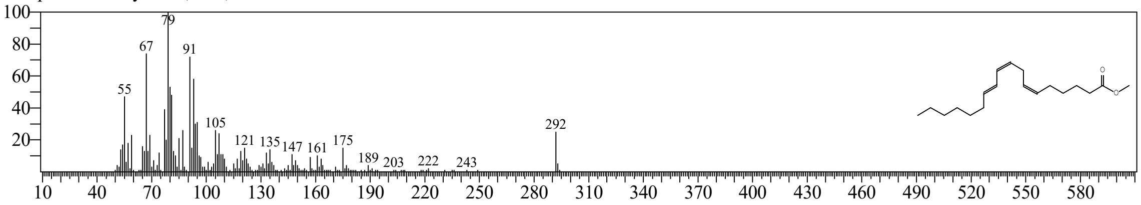

Hit#:4 Entry:124226 Library:NIST14.lib
SI:86 Formula:C19H32O2 CAS:0-00-0 MolWeight:292 RetIndex:2101
CompName:Methyl 6-cis,9-cis,11-trans-octadecatrienoate

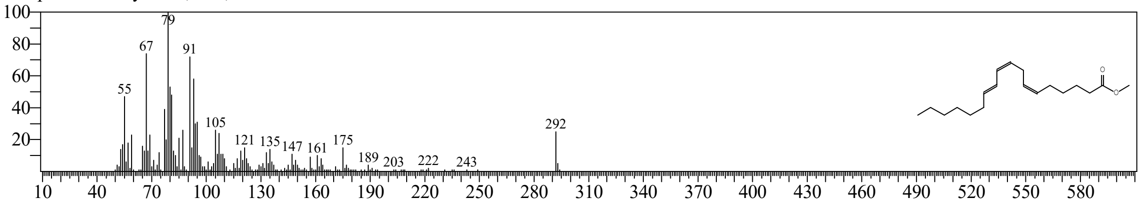

Hit#:5 Entry:135724 Library:NIST17-1.lib
SI:86 Formula:C19H32O2 CAS:301-00-8 MolWeight:292 RetIndex:2101
CompName:9,12,15-Octadecatrienoic acid, methyl ester, (Z,Z,Z)- $$ Linolenic acid, methyl ester $$ Methyl all-cis-9,12,15-octadecatrienoate $$ Methyl lino

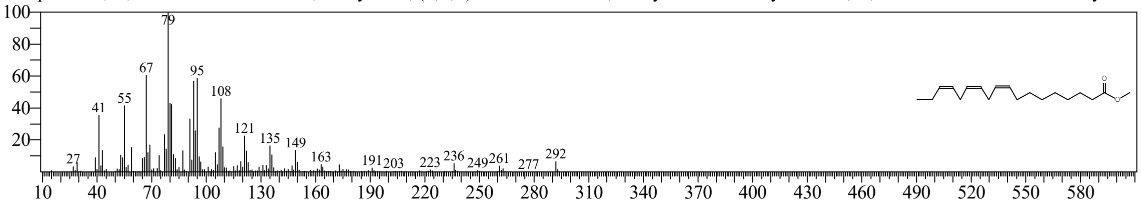

<< Target >>
Line#:23 R.Time:38.742(Scan#:3450) MassPeaks:315
RawMode:Averaged 38.733-38.750(3449-3451) BasePeak:79.05(1050)
BG Mode:Calc. from Peak Group 1 - Event 1

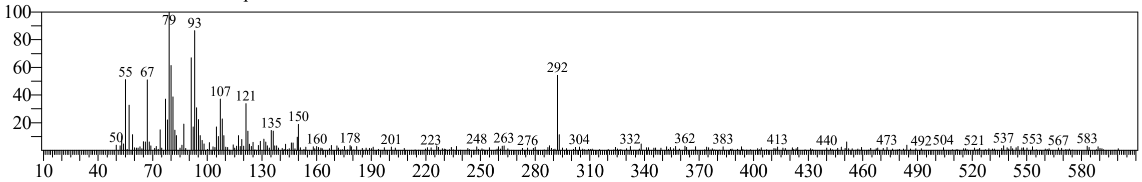

Hit#:1 Entry:135726 Library:NIST17-1.lib
SI:89 Formula:C19H32O2 CAS:0-00-0 MolWeight:292 RetIndex:2101
CompName:Methyl 9.cis.,11.trans.t,13.trans.-octadecatrienoate

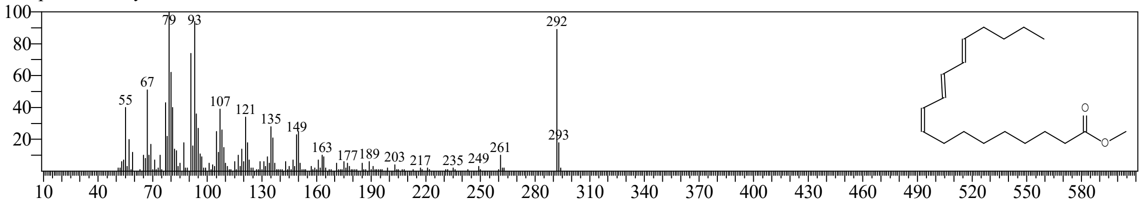

Hit#:2 Entry:124230 Library:NIST14.lib
SI:89 Formula:C19H32O2 CAS:0-00-0 MolWeight:292 RetIndex:2101
CompName:Methyl 9.cis.,11.trans.t,13.trans.-octadecatrienoate

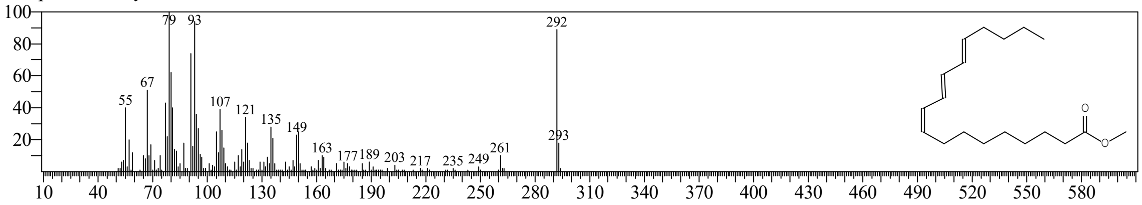

Hit#:3 Entry:135724 Library:NIST17-1.lib
SI:85 Formula:C19H32O2 CAS:301-00-8 MolWeight:292 RetIndex:2101
CompName:9,12,15-Octadecatrienoic acid, methyl ester, (Z,Z,Z)- $$ Linolenic acid, methyl ester $$ Methyl all-cis-9,12,15-octadecatrienoate $$ Methyl lino

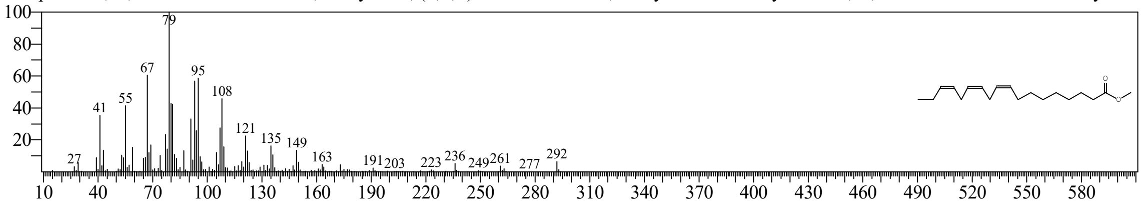

Hit#:4 Entry:124228 Library:NIST14.lib
SI:85 Formula:C19H32O2 CAS:301-00-8 MolWeight:292 RetIndex:2101
CompName:9,12,15-Octadecatrienoic acid, methyl ester, (Z,Z,Z)- $$ Linolenic acid, methyl ester $$ Methyl all-cis-9,12,15-octadecatrienoate $$ Methyl lino

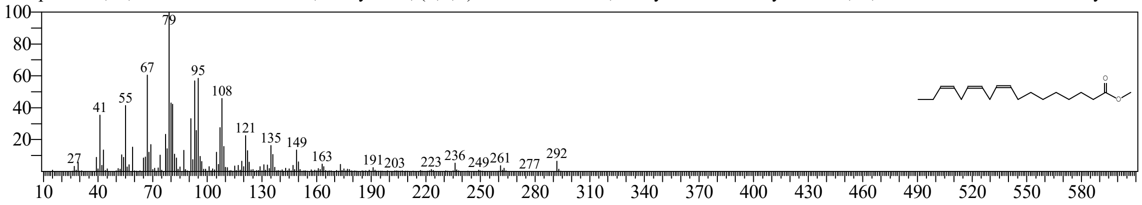

Hit#:5 Entry:124225 Library:NIST14.lib
SI:85 Formula:C19H32O2 CAS:0-00-0 MolWeight:292 RetIndex:2101
CompName:.gamma.-Linolenic acid, methyl ester

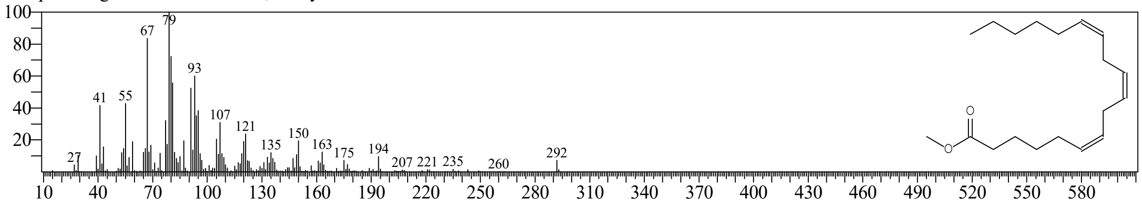

<< Target >>
Line#:24 R.Time:38.875(Scan#:3466) MassPeaks:325
RawMode:Averaged 38.867-38.883(3465-3467) BasePeak:93.10(969)
BG Mode:Calc. from Peak Group 1 - Event 1

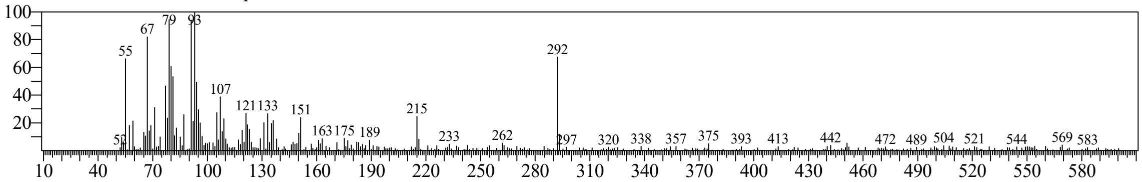

Hit#:1 Entry:135726 Library:NIST17-1.lib
SI:86 Formula:C19H32O2 CAS:0-00-0 MolWeight:292 RetIndex:2101
CompName:Methyl 9.cis.,11.trans.t,13.trans.-octadecatrienoate

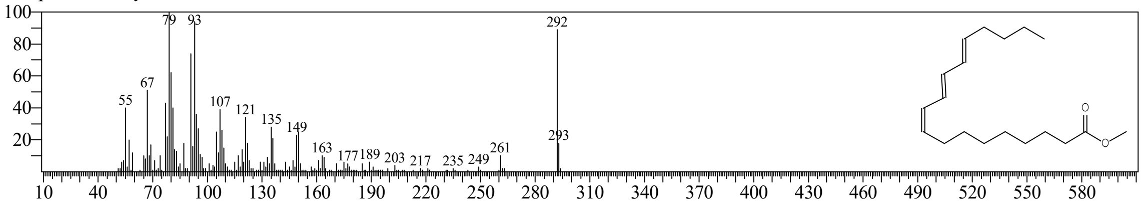

Hit#:2 Entry:124230 Library:NIST14.lib
SI:86 Formula:C19H32O2 CAS:0-00-0 MolWeight:292 RetIndex:2101
CompName:Methyl 9.cis.,11.trans.t,13.trans.-octadecatrienoate

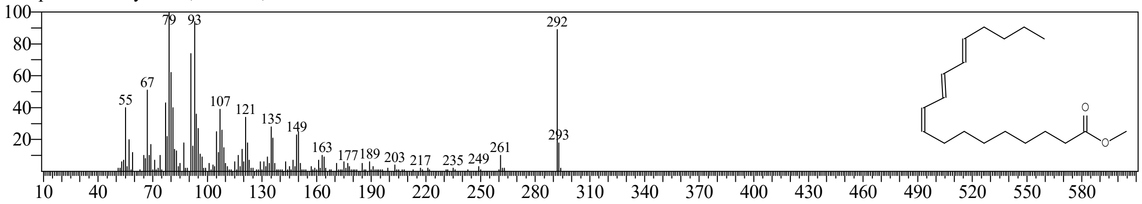

Hit#:3 Entry:124226 Library:NIST14.lib
SI:84 Formula:C19H32O2 CAS:0-00-0 MolWeight:292 RetIndex:2101
CompName:Methyl 6-cis,9-cis,11-trans-octadecatrienoate

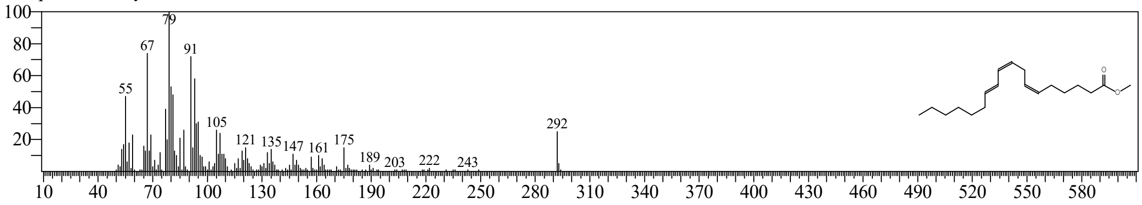

Hit#:4 Entry:135722 Library:NIST17-1.lib
SI:84 Formula:C19H32O2 CAS:0-00-0 MolWeight:292 RetIndex:2101
CompName:Methyl 6-cis,9-cis,11-trans-octadecatrienoate

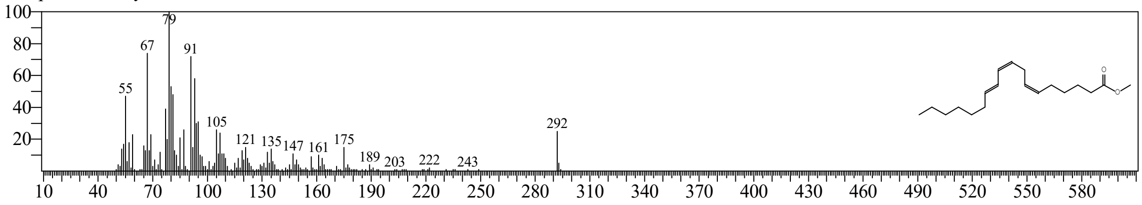

Hit#:5 Entry:124225 Library:NIST14.lib
SI:83 Formula:C19H32O2 CAS:0-00-0 MolWeight:292 RetIndex:2101
CompName:.gamma.-Linolenic acid, methyl ester

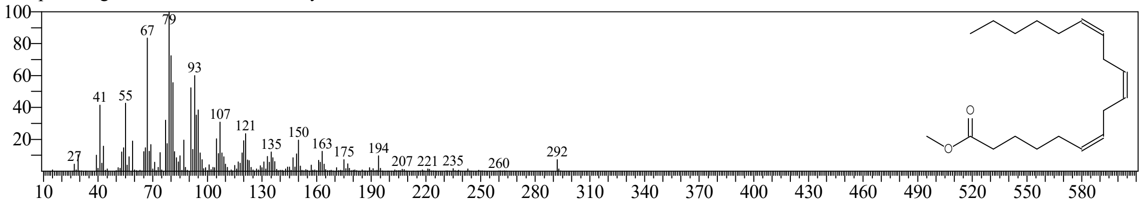

Supplement: Supplementary file 1 [file molecules-31-02891-s001.zip › 28d-QG200-3.pdf]
