# Supplementary material for: Selective Inhibition of Lipid Oxidation by a Quercetagetin-Rich Extract from Tagetes erecta: A Chemometric Approach Using OPLS-DA and Selectivity Index Analysis
Source: Molecules. 2026 Aug 19;31(16):2891. doi: 10.3390/molecules31162891 (PMC13515886; doi:10.3390/molecules31162891)

K-1-56d

<<Column>>
Name : HP-88
Thickness : 0.20um
Length : 100.0m
Inside Diameter : 0.25mm

Method

===== Analytical Line 1 =====

Column Oven Temp.	:120.0 °C	
Injection Temp.	:250.00 °C	
Injection Mode	:Split	
Column Flow	:1.04 mL/min	
Split Ratio	:20.0	
Oven Temp. Program		
Rate	Temperature(°C)	Hold Time(min)
-	120.0	1.00
10.00	175.0	10.00
5.00	210.0	5.00
5.00	230.0	15.00

[GCMS-QP2010]
IonSourceTemp :200.00 °C
Interface Temp. :220.00 °C
m/z scanning range：45～500m/z

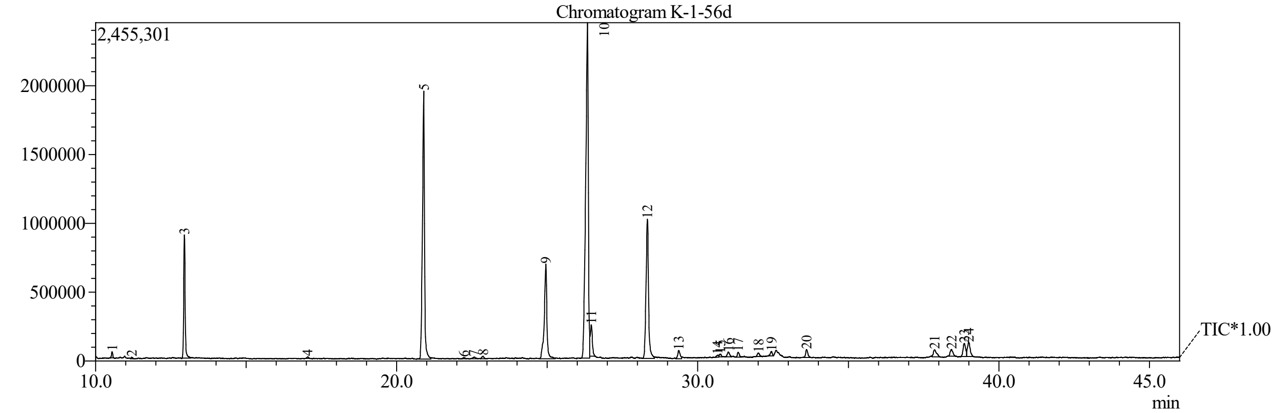

Peak Report TIC

Peak#	R.Time	Area	Area%	Height	Height%	A/H	Name
1	10.554	123402	0.30	43829	0.56	2.82	Octanoic acid, methyl ester
2	11.202	37231	0.09	10853	0.14	3.43	Nonanoic acid, methyl ester
3	12.952	2927047	7.01	894874	11.35	3.27	Undecanoic acid, methyl ester
4	17.049	47146	0.11	13068	0.17	3.61	Methyl tetradecanoate
5	20.899	9302529	22.29	1944584	24.66	4.78	Hexadecanoic acid, methyl ester
6	22.235	46507	0.11	10778	0.14	4.31	7-Hexadecenoic acid, methyl ester, (Z)-
7	22.563	79864	0.19	12528	0.16	6.37	Octanedioic acid, dimethyl ester
8	22.871	84833	0.20	17242	0.22	4.92	Heptadecanoic acid, methyl ester
9	24.955	4146366	9.93	688928	8.74	6.02	Methyl stearate
10	26.338	14837800	35.55	2432755	30.85	6.10	11-Octadecenoic acid, methyl ester
11	26.464	1031704	2.47	229571	2.91	4.49	trans-13-Octadecenoic acid, methyl ester
12	28.326	5761865	13.81	1009042	12.80	5.71	9,12-Octadecadienoic acid, methyl ester
13	29.368	320867	0.77	59026	0.75	5.44	Eicosanoic acid, methyl ester
14	30.658	76591	0.18	15724	0.20	4.87	11,14,17-Eicosatrienoic acid, methyl ester

Peak#	R.Time	Area	Area%	Height	Height%	A/H	Name
15	30.748	79215	0.19	19148	0.24	4.14	cis-Methyl 11-eicosenoate
16	31.013	196083	0.47	34780	0.44	5.64	9,11-Octadecadienoic acid, methyl ester, (E,E)
17	31.336	167184	0.40	35502	0.45	4.71	11,14-Octadecadienoic acid, methyl ester
18	32.013	123706	0.30	25610	0.32	4.83	Methyl 9-cis,11-trans-octadecadienoate
19	32.449	143974	0.34	31627	0.40	4.55	Dodecanoic acid, ethenyl ester
20	33.611	287598	0.69	57891	0.73	4.97	Docosanoic acid, methyl ester
21	37.862	387924	0.93	52444	0.67	7.40	Methyl 9.cis.,11.trans.t,13.trans.-octadecatrien
22	38.422	306459	0.73	47992	0.61	6.39	Methyl 9.cis.,11.trans.t,13.trans.-octadecatrien
23	38.849	572217	1.37	94877	1.20	6.03	Methyl 9.cis.,11.trans.t,13.trans.-octadecatrien
24	38.996	647888	1.55	102107	1.29	6.35	Methyl 6-cis,9-cis,11-trans-octadecatrienoate
		41736000	100.00	7884780	100.00		

Library

<< Target >>
Line#:1 R.Time:10.550(Scan#:67) MassPeaks:291
RawMode:Averaged 10.542-10.558(66-68) BasePeak:74.05(14175)
BG Mode:Calc. from Peak Group 1 - Event 1

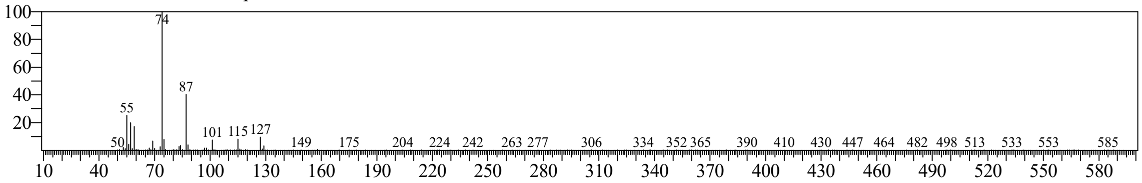

Hit#:1 Entry:11044 Library:NIST14s.lib
SI:97 Formula:C9H18O2 CAS:111-11-5 MolWeight:158 RetIndex:1083
CompName:Octanoic acid, methyl ester $$ Caprylic acid methyl ester $$ Methyl caprylate $$ Methyl n-octanoate $$ Methyl octanoate $$ Uniphat A20 $$ n

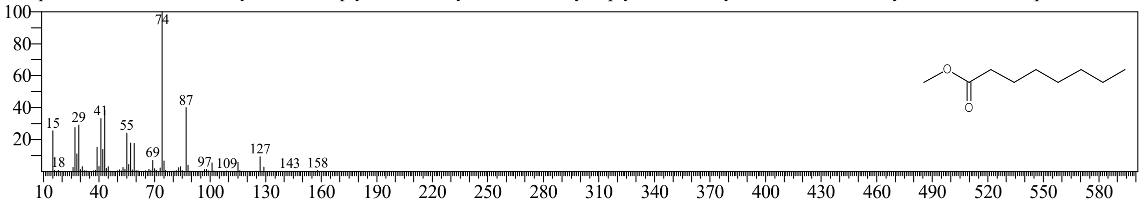

Hit#:2 Entry:12802 Library:NIST17s.lib
SI:97 Formula:C9H18O2 CAS:111-11-5 MolWeight:158 RetIndex:1083
CompName:Octanoic acid, methyl ester $$ Caprylic acid methyl ester $$ Methyl caprylate $$ Methyl n-octanoate $$ Methyl octanoate $$ Uniphat A20 $$ n

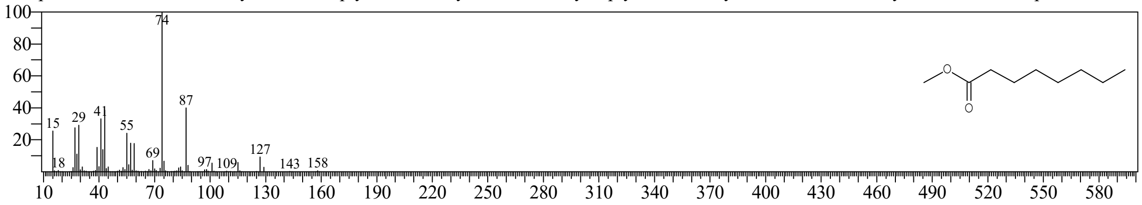

Hit#:3 Entry:12803 Library:NIST17s.lib
SI:96 Formula:C9H18O2 CAS:111-11-5 MolWeight:158 RetIndex:1083
CompName:Octanoic acid, methyl ester $$ Caprylic acid methyl ester $$ Methyl caprylate $$ Methyl n-octanoate $$ Methyl octanoate $$ Uniphat A20 $$ n

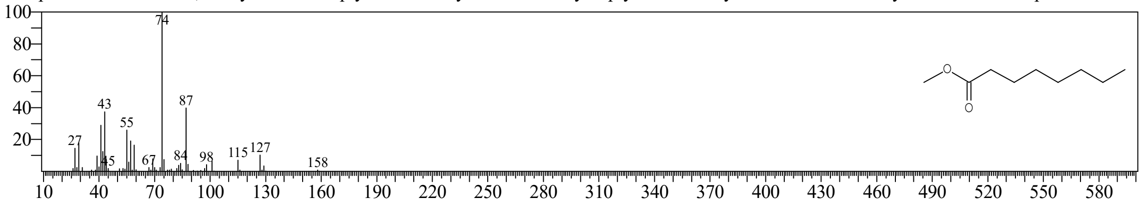

Hit#:4 Entry:11045 Library:NIST14s.lib
SI:96 Formula:C9H18O2 CAS:111-11-5 MolWeight:158 RetIndex:1083
CompName:Octanoic acid, methyl ester $$ Caprylic acid methyl ester $$ Methyl caprylate $$ Methyl n-octanoate $$ Methyl octanoate $$ Uniphat A20 $$ n

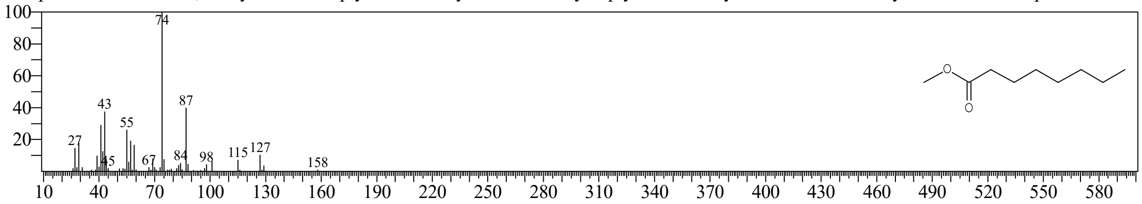

<< Target >>
Line#:1   R.Time:10.550(Scan#:67) MassPeaks:291
RawMode:Averaged 10.542-10.558(66-68) BasePeak:74.05(14175)
BG Mode:Calc. from Peak Group 1 - Event 1

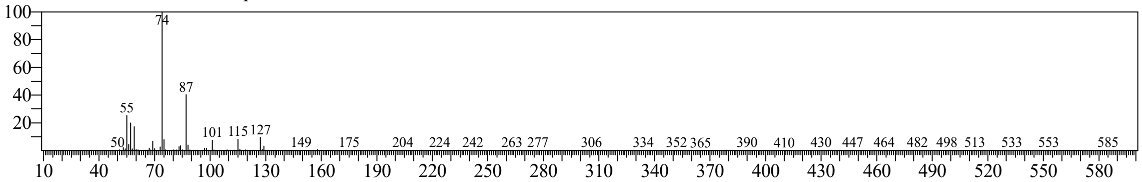

Hit#:5 Entry:12801 Library:NIST17s.lib
SI:95 Formula:C9H18O2 CAS:111-11-5 MolWeight:158 RetIndex:1083
CompName:Octanoic acid, methyl ester $$ Caprylic acid methyl ester $$ Methyl caprylate $$ Methyl n-octanoate $$ Methyl octanoate $$ Uniphat A20 $$ n

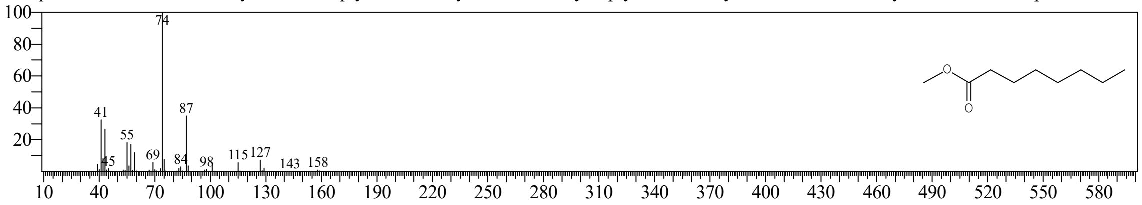

<< Target >>
Line#:2 R.Time:11.200(Scan#:145) MassPeaks:315
RawMode:Averaged 11.192-11.208(144-146) BasePeak:74.05(2127)
BG Mode:Calc. from Peak Group 1 - Event 1

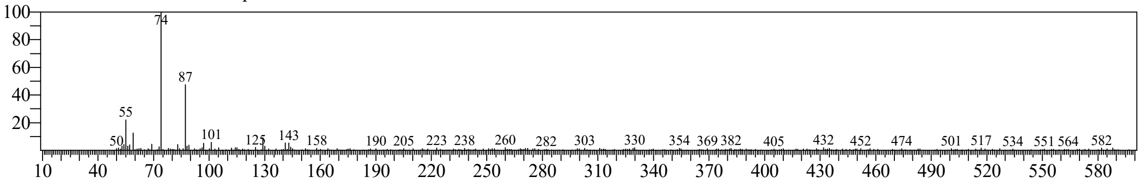

Hit#:1 Entry:13673 Library:NIST14s.lib
SI:83 Formula:C10H20O2 CAS:1731-84-6 MolWeight:172 RetIndex:1183
CompName:Nonanoic acid, methyl ester $$ Methyl n-nonanoate $$ Methyl nonanoate $$ Methyl nonylate $$ Methyl pelargonate $$ Pelargonic acid methyl

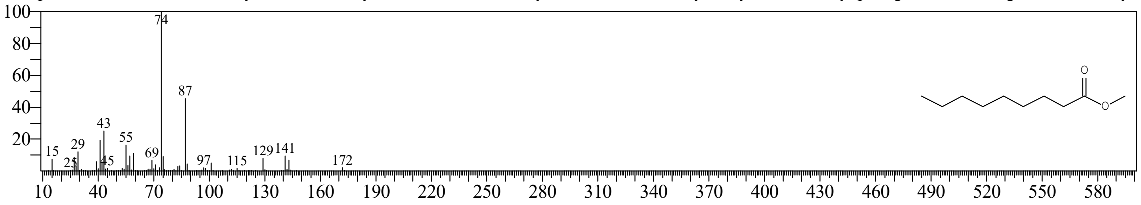

Hit#:2 Entry:15895 Library:NIST17s.lib
SI:83 Formula:C10H20O2 CAS:1731-84-6 MolWeight:172 RetIndex:1183
CompName:Nonanoic acid, methyl ester $$ Methyl n-nonanoate $$ Methyl nonanoate $$ Methyl nonylate $$ Methyl pelargonate $$ Pelargonic acid methyl

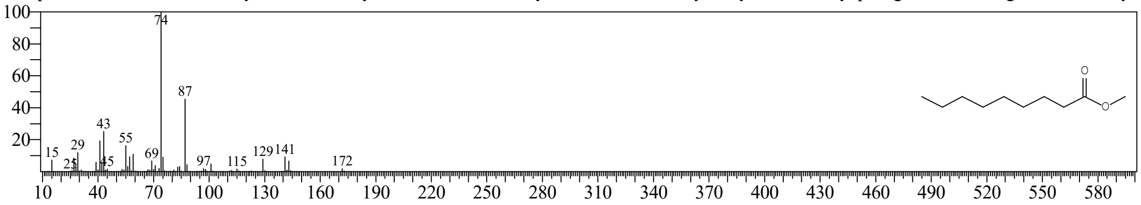

Hit#:3 Entry:15896 Library:NIST17s.lib
SI:83 Formula:C10H20O2 CAS:1731-84-6 MolWeight:172 RetIndex:1183
CompName:Nonanoic acid, methyl ester $$ Methyl n-nonanoate $$ Methyl nonanoate $$ Methyl nonylate $$ Methyl pelargonate $$ Pelargonic acid methyl

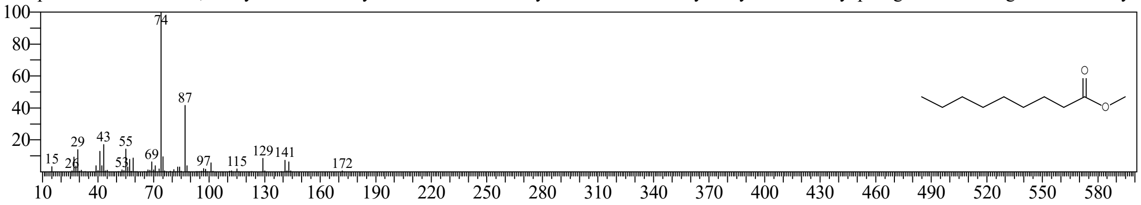

Hit#:4 Entry:13674 Library:NIST14s.lib
SI:83 Formula:C10H20O2 CAS:1731-84-6 MolWeight:172 RetIndex:1183
CompName:Nonanoic acid, methyl ester $$ Methyl n-nonanoate $$ Methyl nonanoate $$ Methyl nonylate $$ Methyl pelargonate $$ Pelargonic acid methyl

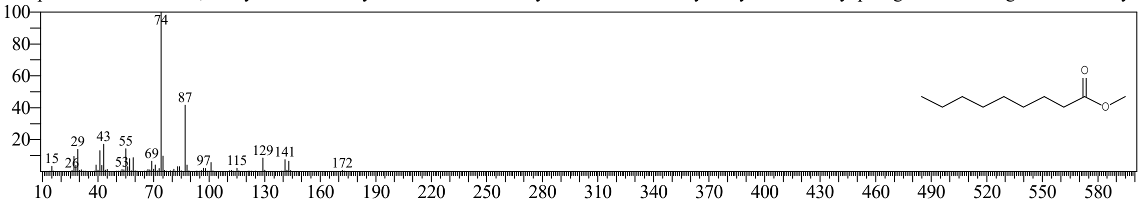

Hit#:5 Entry:15894 Library:NIST17s.lib
SI:82 Formula:C10H20O2 CAS:1731-84-6 MolWeight:172 RetIndex:1183
CompName:Nonanoic acid, methyl ester $$ Methyl n-nonanoate $$ Methyl nonanoate $$ Methyl nonylate $$ Methyl pelargonate $$ Pelargonic acid methyl

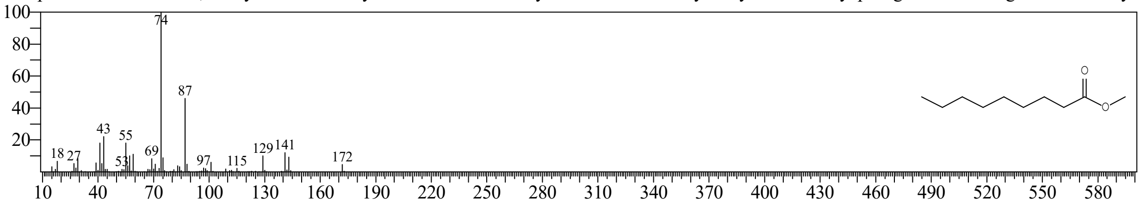

<< Target >>
Line#:3 R.Time:12.950(Scan#:355) MassPeaks:296
RawMode:Averaged 12.942-12.958(354-356) BasePeak:74.05(257501)
BG Mode:Calc. from Peak Group 1 - Event 1

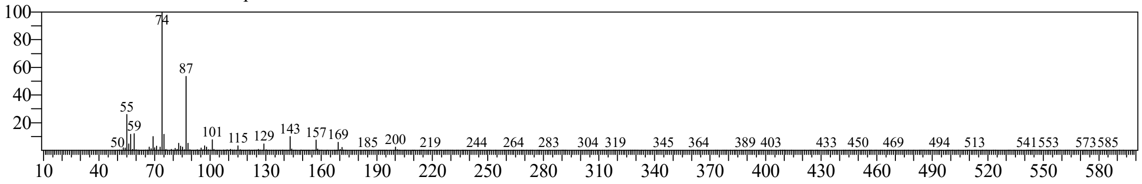

Hit#:1 Entry:49598 Library:NIST17-1.lib
SI:96 Formula:C12H24O2 CAS:1731-86-8 MolWeight:200 RetIndex:1381
CompName:Undecanoic acid, methyl ester $$ Methyl undecanoate $$ Methyl undecylenate $$ n-Undecanoic acid methyl ester $$ Methyl ester of undecanoi

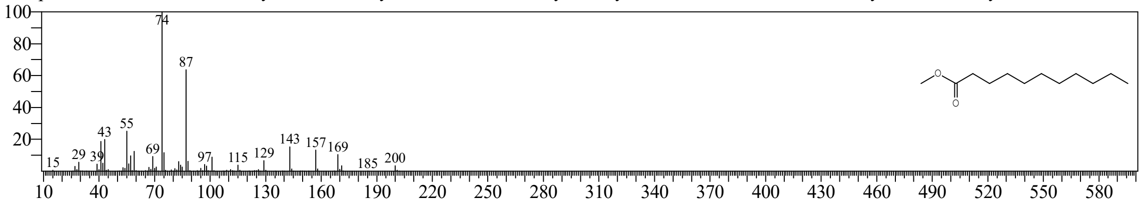

Hit#:2 Entry:46966 Library:NIST14.lib
SI:96 Formula:C12H24O2 CAS:1731-86-8 MolWeight:200 RetIndex:1381
CompName:Undecanoic acid, methyl ester $$ Methyl undecanoate $$ Methyl undecylenate $$ n-Undecanoic acid methyl ester $$ Methyl ester of undecanoi

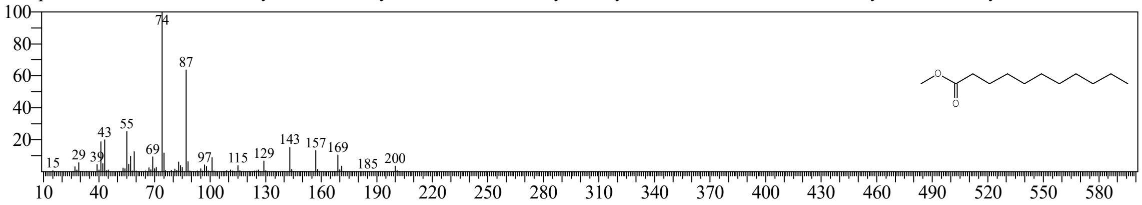

Hit#:3 Entry:21618 Library:NIST17s.lib
SI:95 Formula:C12H24O2 CAS:1731-86-8 MolWeight:200 RetIndex:1381
CompName:Undecanoic acid, methyl ester $$ Methyl undecanoate $$ Methyl undecylenate $$ n-Undecanoic acid methyl ester $$ Methyl ester of undecanoi

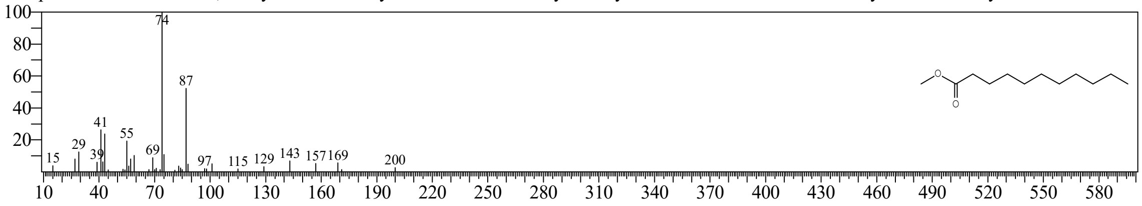

Hit#:4 Entry:21619 Library:NIST17s.lib
SI:94 Formula:C12H24O2 CAS:1731-86-8 MolWeight:200 RetIndex:1381
CompName:Undecanoic acid, methyl ester $$ Methyl undecanoate $$ Methyl undecylenate $$ n-Undecanoic acid methyl ester $$ Methyl ester of undecanoi

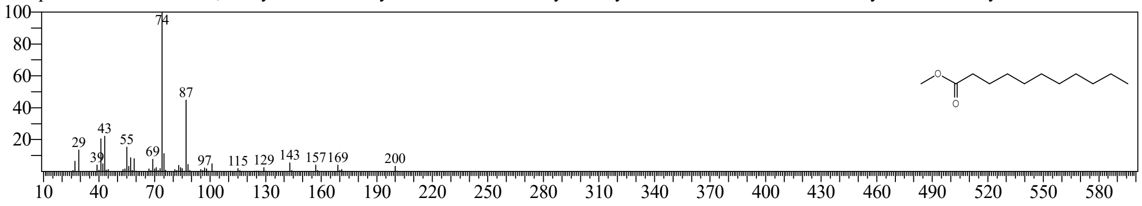

Hit#:5 Entry:18487 Library:NIST14s.lib
SI:94 Formula:C12H24O2 CAS:1731-86-8 MolWeight:200 RetIndex:1381
CompName:Undecanoic acid, methyl ester $$ Methyl undecanoate $$ Methyl undecylenate $$ n-Undecanoic acid methyl ester $$ Methyl ester of undecanoi

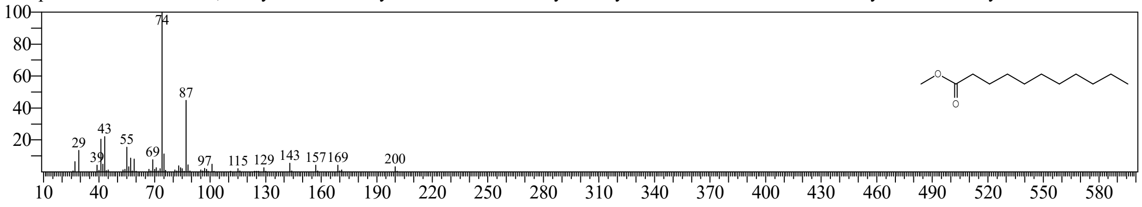

<< Target >>
Line#:4 R.Time:17.050(Scan#:847) MassPeaks:324
RawMode:Averaged 17.042-17.058(846-848) BasePeak:74.05(2825)
BG Mode:Calc. from Peak Group 1 - Event 1

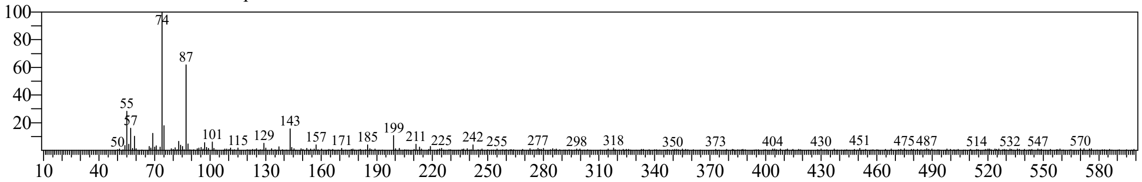

Hit#:1 Entry:86786 Library:NIST17-1.lib
SI:94 Formula:C15H30O2 CAS:124-10-7 MolWeight:242 RetIndex:1680
CompName:Methyl tetradecanoate $$ Tetradecanoic acid, methyl ester $$ Myristic acid, methyl ester $$ Metholeneat 2495 $$ Methyl myristate $$ Methyl r

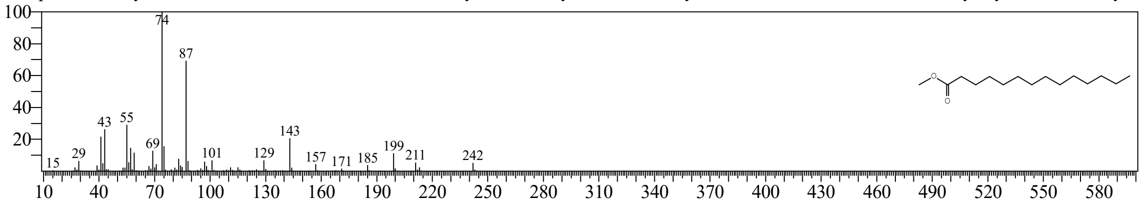

Hit#:2 Entry:80645 Library:NIST14.lib
SI:94 Formula:C15H30O2 CAS:124-10-7 MolWeight:242 RetIndex:1680
CompName:Methyl tetradecanoate $$ Tetradecanoic acid, methyl ester $$ Myristic acid, methyl ester $$ Metholeneat 2495 $$ Methyl myristate $$ Methyl r

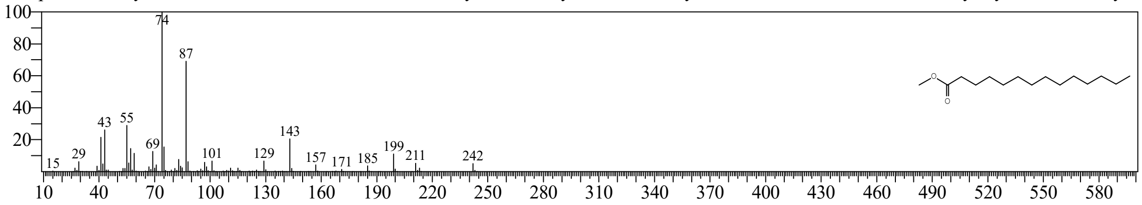

Hit#:3 Entry:23848 Library:NIST14s.lib
SI:93 Formula:C15H30O2 CAS:124-10-7 MolWeight:242 RetIndex:1680
CompName:Methyl tetradecanoate $$ Tetradecanoic acid, methyl ester $$ Myristic acid, methyl ester $$ Metholeneat 2495 $$ Methyl myristate $$ Methyl r

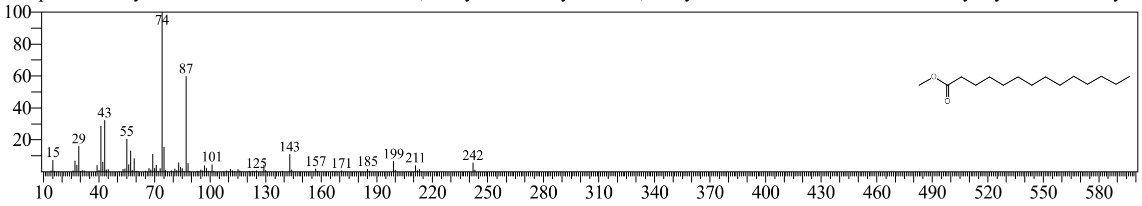

Hit#:4 Entry:27874 Library:NIST17s.lib
SI:93 Formula:C15H30O2 CAS:124-10-7 MolWeight:242 RetIndex:1680
CompName:Methyl tetradecanoate $$ Tetradecanoic acid, methyl ester $$ Myristic acid, methyl ester $$ Metholeneat 2495 $$ Methyl myristate $$ Methyl r

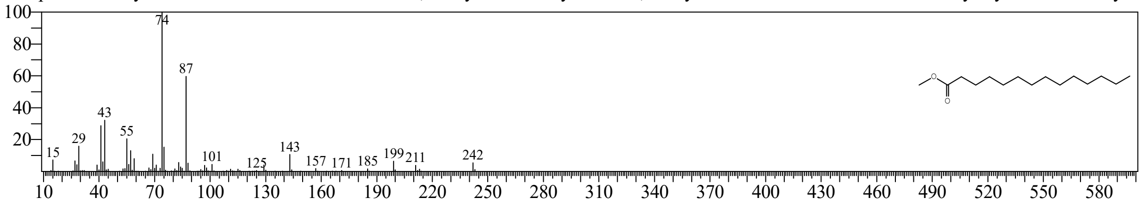

Hit#:5 Entry:23847 Library:NIST14s.lib
SI:93 Formula:C15H30O2 CAS:124-10-7 MolWeight:242 RetIndex:1680
CompName:Methyl tetradecanoate $$ Tetradecanoic acid, methyl ester $$ Myristic acid, methyl ester $$ Metholeneat 2495 $$ Methyl myristate $$ Methyl r

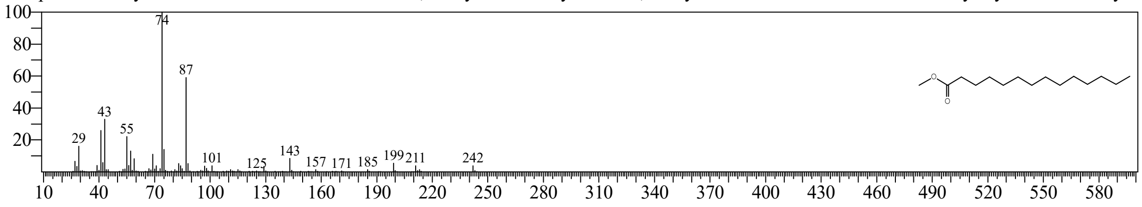

<< Target >>
Line#:5 R.Time:20.900(Scan#:1309) MassPeaks:376
RawMode:Averaged 20.892-20.908(1308-1310) BasePeak:74.05(443803)
BG Mode:Calc. from Peak Group 1 - Event 1

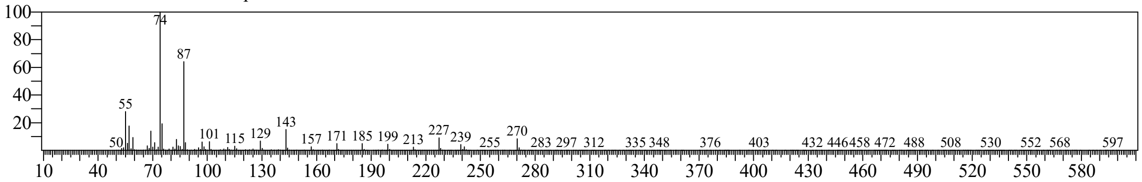

Hit#:1 Entry:104648 Library:NIST14.lib
SI:97 Formula:C17H34O2 CAS:112-39-0 MolWeight:270 RetIndex:1878
CompName:Hexadecanoic acid, methyl ester $$ Palmitic acid, methyl ester $$ n-Hexadecanoic acid methyl ester $$ Metholene 2216 $$ Methyl hexadecano:

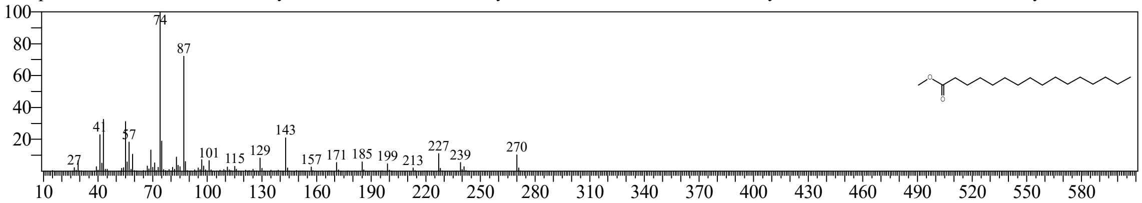

Hit#:2 Entry:113663 Library:NIST17-1.lib
SI:97 Formula:C17H34O2 CAS:112-39-0 MolWeight:270 RetIndex:1878
CompName:Hexadecanoic acid, methyl ester $$ Palmitic acid, methyl ester $$ n-Hexadecanoic acid methyl ester $$ Metholene 2216 $$ Methyl hexadecano:

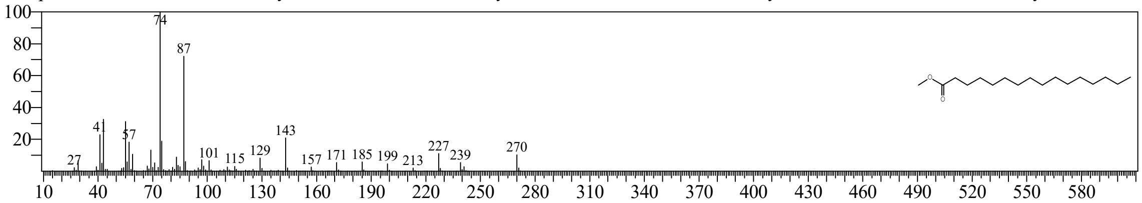

Hit#:3 Entry:26272 Library:NIST14s.lib
SI:97 Formula:C17H34O2 CAS:112-39-0 MolWeight:270 RetIndex:1878
CompName:Hexadecanoic acid, methyl ester $$ Palmitic acid, methyl ester $$ n-Hexadecanoic acid methyl ester $$ Metholene 2216 $$ Methyl hexadecano:

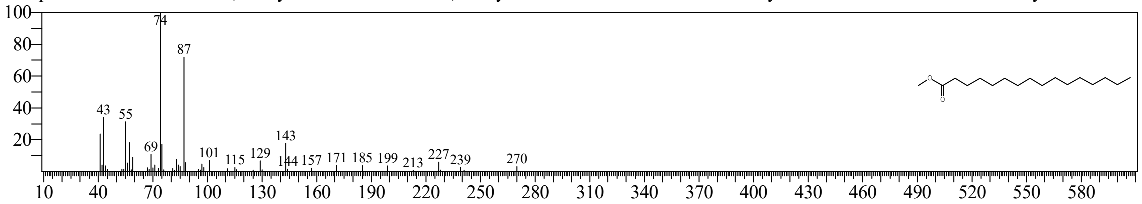

Hit#:4 Entry:30647 Library:NIST17s.lib
SI:97 Formula:C17H34O2 CAS:112-39-0 MolWeight:270 RetIndex:1878
CompName:Hexadecanoic acid, methyl ester $$ Palmitic acid, methyl ester $$ n-Hexadecanoic acid methyl ester $$ Metholene 2216 $$ Methyl hexadecano:

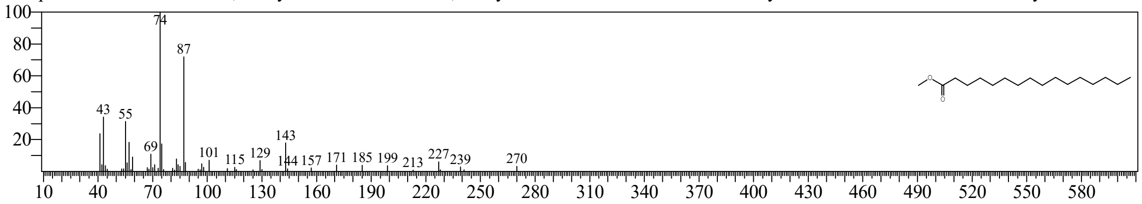

Hit#:5 Entry:26271 Library:NIST14s.lib
SI:96 Formula:C17H34O2 CAS:112-39-0 MolWeight:270 RetIndex:1878
CompName:Hexadecanoic acid, methyl ester $$ Palmitic acid, methyl ester $$ n-Hexadecanoic acid methyl ester $$ Metholene 2216 $$ Methyl hexadecano:

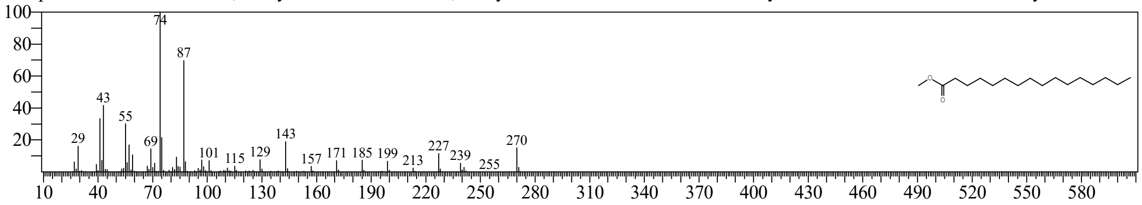

<< Target >>
Line#:6 R.Time:22.233(Scan#:1469) MassPeaks:293
RawMode:Averaged 22.225-22.242(1468-1470) BasePeak:55.10(1041)
BG Mode:Calc. from Peak Group 1 - Event 1

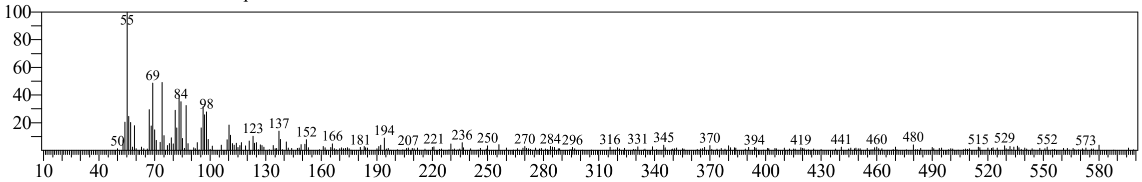

Hit#:1 Entry:111510 Library:NIST17-1.lib
SI:91 Formula:C17H32O2 CAS:56875-67-3 MolWeight:268 RetIndex:1886
CompName:7-Hexadecenoic acid, methyl ester, (Z)- $$ Methyl (7E)-7-hexadecenoate # $$

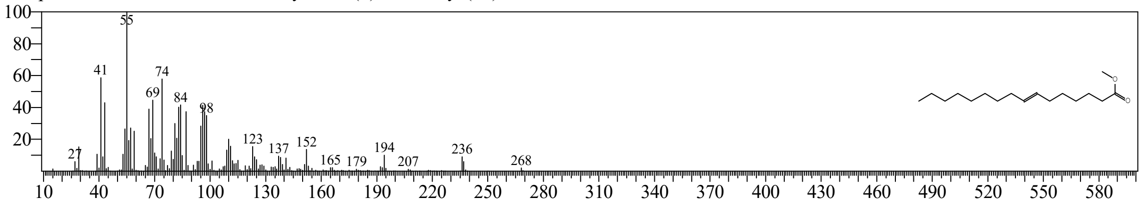

Hit#:2 Entry:26063 Library:NIST14s.lib
SI:90 Formula:C17H32O2 CAS:56875-67-3 MolWeight:268 RetIndex:1886
CompName:7-Hexadecenoic acid, methyl ester, (Z)- $$ Methyl (7E)-7-hexadecenoate # $$

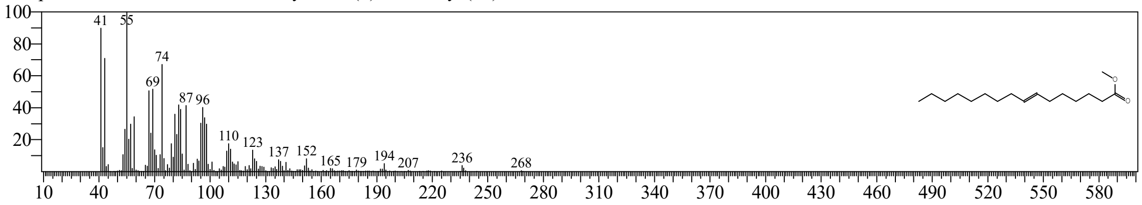

Hit#:3 Entry:30412 Library:NIST17s.lib
SI:90 Formula:C17H32O2 CAS:56875-67-3 MolWeight:268 RetIndex:1886
CompName:7-Hexadecenoic acid, methyl ester, (Z)- $$ Methyl (7E)-7-hexadecenoate # $$

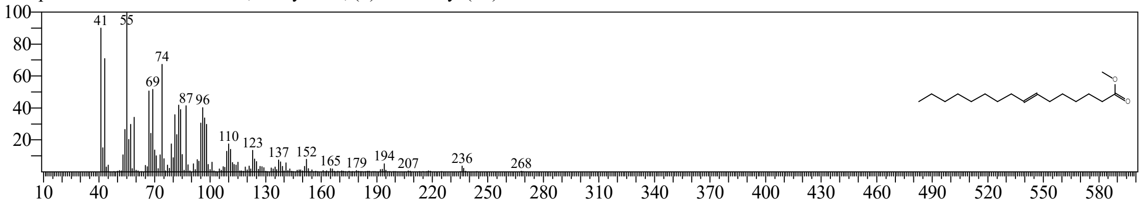

Hit#:4 Entry:30413 Library:NIST17s.lib
SI:90 Formula:C17H32O2 CAS:1120-25-8 MolWeight:268 RetIndex:1886
CompName:9-Hexadecenoic acid, methyl ester, (Z)- $$ Methyl palmitoleate $$ Methyl palmitoleinate $$ Palmitoleic acid, methyl ester $$ Methyl (9Z)-9-he:

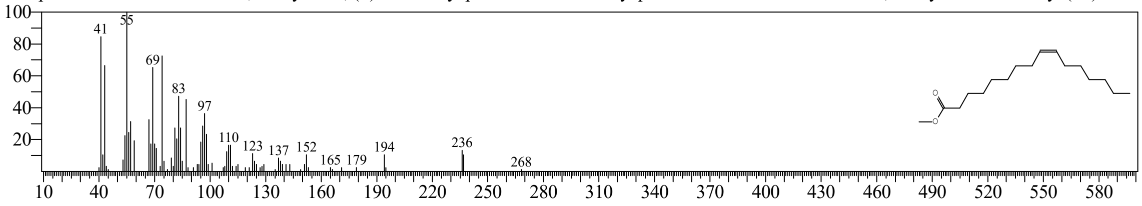

Hit#:5 Entry:26064 Library:NIST14s.lib
SI:90 Formula:C17H32O2 CAS:1120-25-8 MolWeight:268 RetIndex:1886
CompName:9-Hexadecenoic acid, methyl ester, (Z)- $$ Methyl palmitoleate $$ Methyl palmitoleinate $$ Palmitoleic acid, methyl ester $$ Methyl (9Z)-9-he:

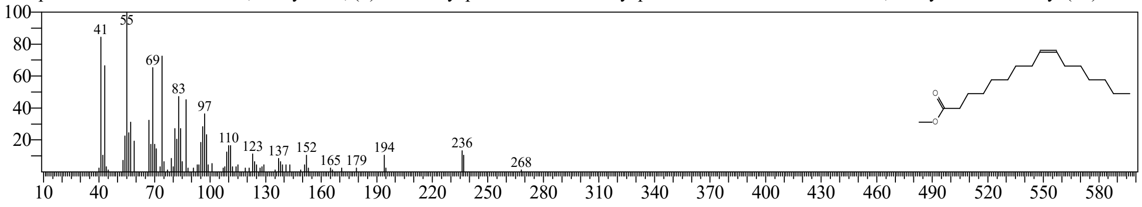

<< Target >>
Line#:7 R.Time:22.567(Scan#:1509) MassPeaks:295
RawMode:Averaged 22.558-22.575(1508-1510) BasePeak:55.05(1276)
BG Mode:Calc. from Peak Group 1 - Event 1

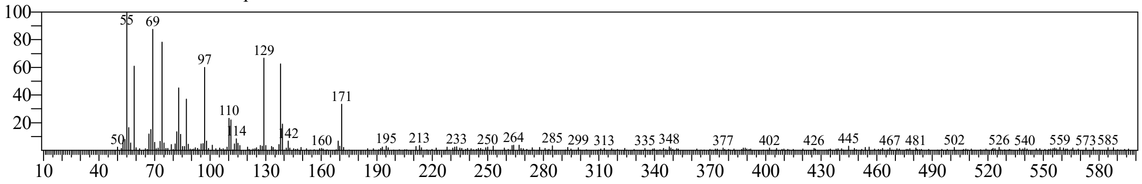

Hit#:1 Entry:50683 Library:NIST17-1.lib
SI:92 Formula:C10H18O4 CAS:1732-09-8 MolWeight:202 RetIndex:1350
CompName:Octanedioic acid, dimethyl ester $$ Suberic acid, dimethyl ester $$ Dimethyl octanedioate $$ Dimethyl suberate $$ Dimethyl ester of octanedio

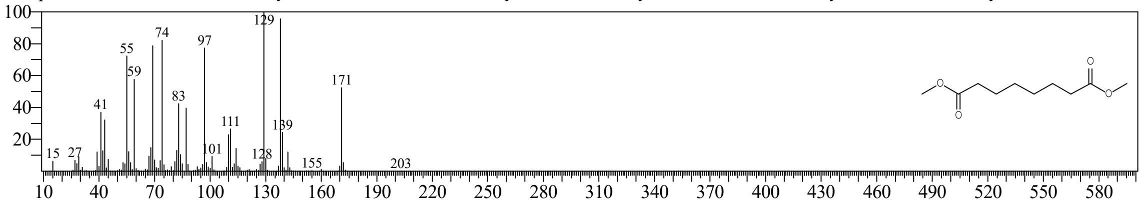

Hit#:2 Entry:47957 Library:NIST14.lib
SI:92 Formula:C10H18O4 CAS:1732-09-8 MolWeight:202 RetIndex:1350
CompName:Octanedioic acid, dimethyl ester $$ Suberic acid, dimethyl ester $$ Dimethyl octanedioate $$ Dimethyl suberate $$ Dimethyl ester of octanedio

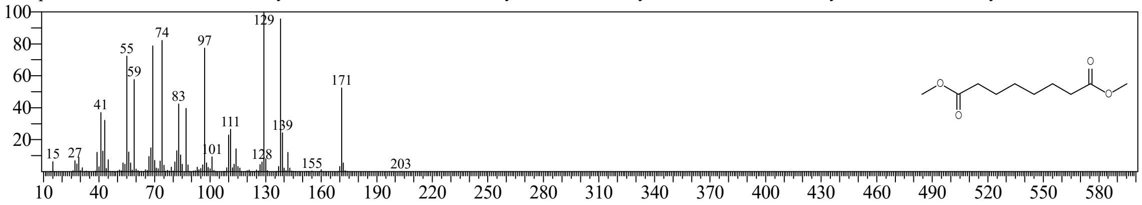

Hit#:3 Entry:18672 Library:NIST14s.lib
SI:91 Formula:C10H18O4 CAS:1732-09-8 MolWeight:202 RetIndex:1350
CompName:Octanedioic acid, dimethyl ester $$ Suberic acid, dimethyl ester $$ Dimethyl octanedioate $$ Dimethyl suberate $$ Dimethyl ester of octanedio

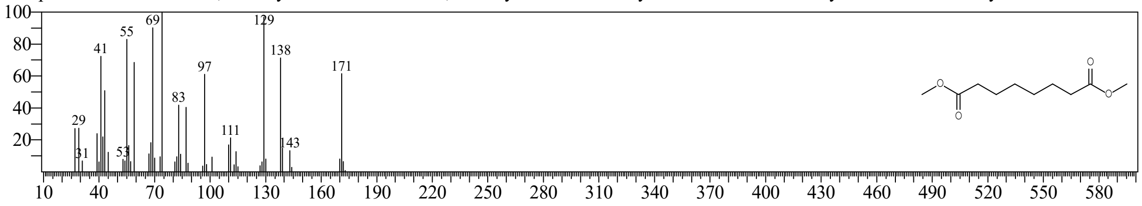

Hit#:4 Entry:18670 Library:NIST14s.lib
SI:90 Formula:C10H18O4 CAS:1732-09-8 MolWeight:202 RetIndex:1350
CompName:Octanedioic acid, dimethyl ester $$ Suberic acid, dimethyl ester $$ Dimethyl octanedioate $$ Dimethyl suberate $$ Dimethyl ester of octanedio

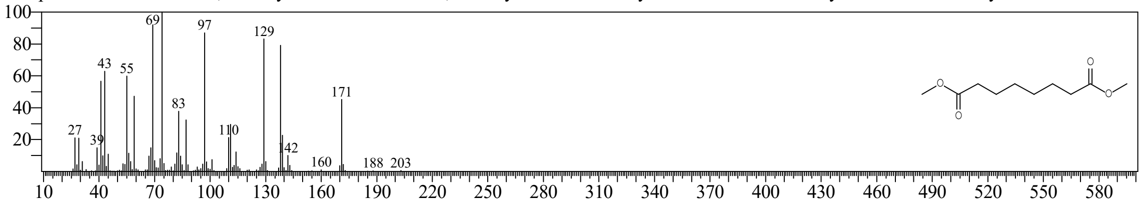

Hit#:5 Entry:21831 Library:NIST17s.lib
SI:90 Formula:C10H18O4 CAS:1732-09-8 MolWeight:202 RetIndex:1350
CompName:Octanedioic acid, dimethyl ester $$ Suberic acid, dimethyl ester $$ Dimethyl octanedioate $$ Dimethyl suberate $$ Dimethyl ester of octanedio

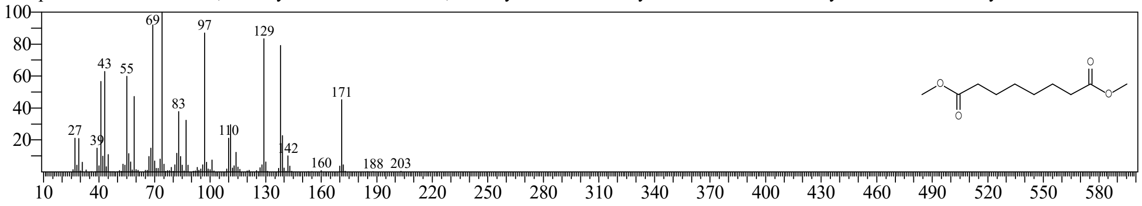

<< Target >>
Line#:8 R.Time:22.867(Scan#:1545) MassPeaks:309
RawMode:Averaged 22.858-22.875(1544-1546) BasePeak:74.05(3742)
BG Mode:Calc. from Peak Group 1 - Event 1

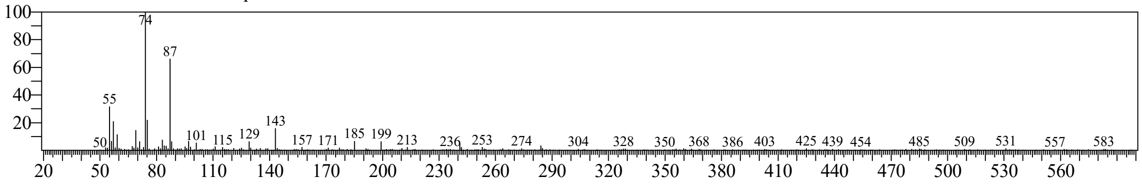

Hit#:1 Entry:31784 Library:NIST17s.lib
SI:94 Formula:C18H36O2 CAS:1731-92-6 MolWeight:284 RetIndex:1978
CompName:Heptadecanoic acid, methyl ester $$ Margaric acid methyl ester $$ Methyl heptadecanoate $$ Methyl margarate $$ n-Heptadecanoic acid methy

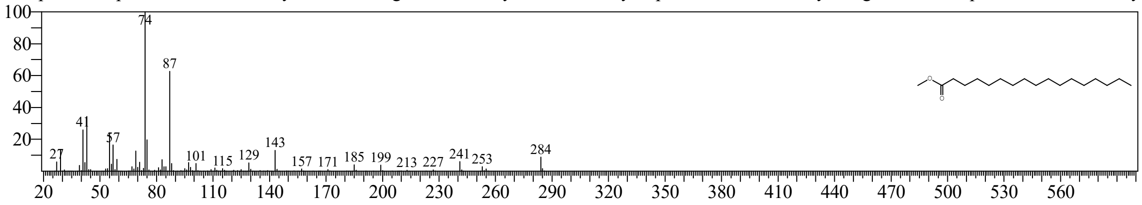

Hit#:2 Entry:27275 Library:NIST14s.lib
SI:94 Formula:C18H36O2 CAS:1731-92-6 MolWeight:284 RetIndex:1978
CompName:Heptadecanoic acid, methyl ester $$ Margaric acid methyl ester $$ Methyl heptadecanoate $$ Methyl margarate $$ n-Heptadecanoic acid methy

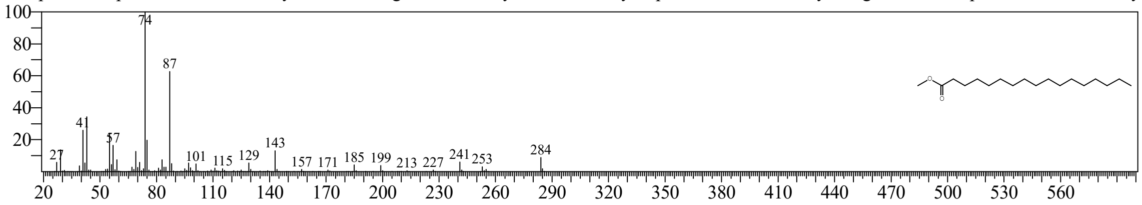

Hit#:3 Entry:117104 Library:NIST14.lib
SI:94 Formula:C18H36O2 CAS:6929-04-0 MolWeight:284 RetIndex:1914
CompName:Hexadecanoic acid, 15-methyl-, methyl ester $$ Methyl isoheptadecanoate $$ Methyl 15-methylhexadecanoate $$

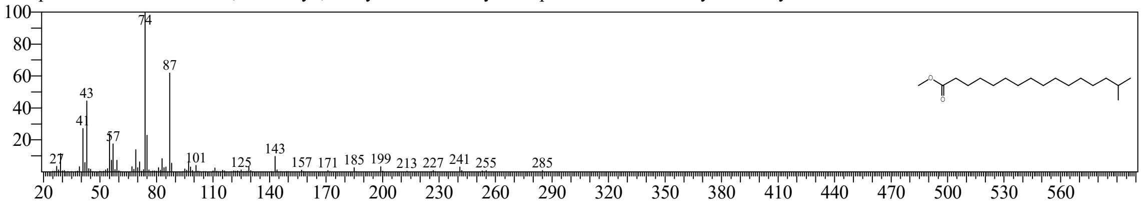

Hit#:4 Entry:31783 Library:NIST17s.lib
SI:93 Formula:C18H36O2 CAS:1731-92-6 MolWeight:284 RetIndex:1978
CompName:Heptadecanoic acid, methyl ester $$ Margaric acid methyl ester $$ Methyl heptadecanoate $$ Methyl margarate $$ n-Heptadecanoic acid methy

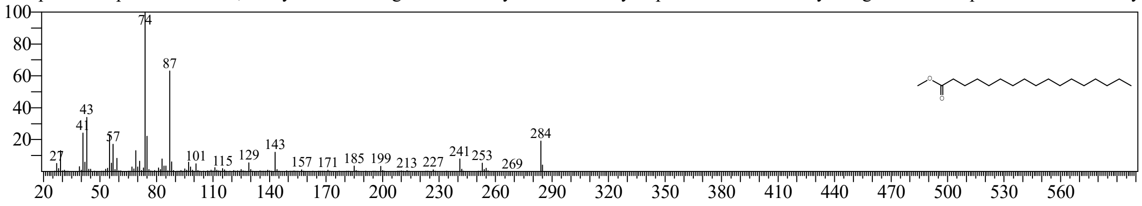

Hit#:5 Entry:27274 Library:NIST14s.lib
SI:93 Formula:C18H36O2 CAS:1731-92-6 MolWeight:284 RetIndex:1978
CompName:Heptadecanoic acid, methyl ester $$ Margaric acid methyl ester $$ Methyl heptadecanoate $$ Methyl margarate $$ n-Heptadecanoic acid methy

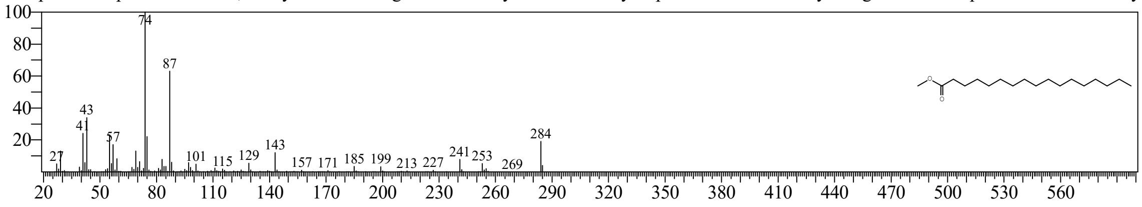

<< Target >>
Line#:9 R.Time:24.958(Scan#:1796) MassPeaks:365
RawMode:Averaged 24.950-24.967(1795-1797) BasePeak:74.05(143726)
BG Mode:Calc. from Peak Group 1 - Event 1

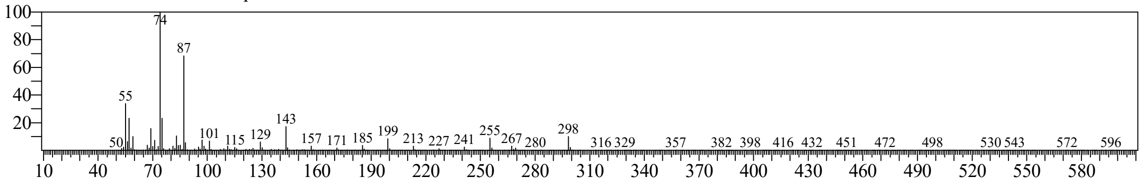

Hit#:1 Entry:32875 Library:NIST17s.lib
SI:97 Formula:C19H38O2 CAS:112-61-8 MolWeight:298 RetIndex:2077
CompName:Methyl stearate $$ Octadecanoic acid, methyl ester $$ Stearic acid, methyl ester $$ n-Octadecanoic acid, methyl ester $$ Kemester 9718 $$ Me

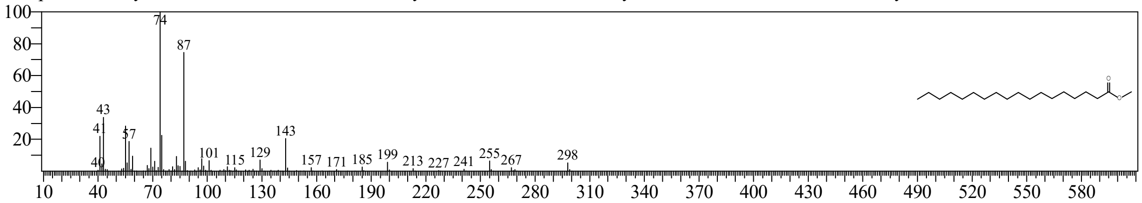

Hit#:2 Entry:28257 Library:NIST14s.lib
SI:97 Formula:C19H38O2 CAS:112-61-8 MolWeight:298 RetIndex:2077
CompName:Methyl stearate $$ Octadecanoic acid, methyl ester $$ Stearic acid, methyl ester $$ n-Octadecanoic acid, methyl ester $$ Kemester 9718 $$ Me

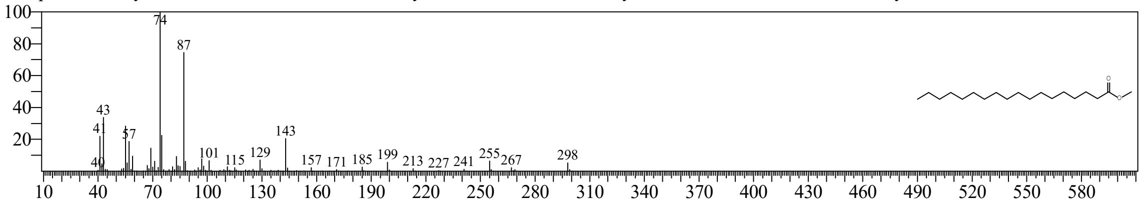

Hit#:3 Entry:129694 Library:NIST14.lib
SI:97 Formula:C19H38O2 CAS:112-61-8 MolWeight:298 RetIndex:2077
CompName:Methyl stearate $$ Octadecanoic acid, methyl ester $$ Stearic acid, methyl ester $$ n-Octadecanoic acid, methyl ester $$ Kemester 9718 $$ Me

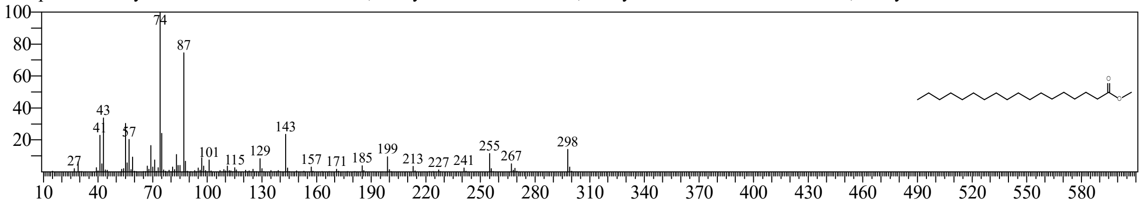

Hit#:4 Entry:141978 Library:NIST17-1.lib
SI:97 Formula:C19H38O2 CAS:112-61-8 MolWeight:298 RetIndex:2077
CompName:Methyl stearate $$ Octadecanoic acid, methyl ester $$ Stearic acid, methyl ester $$ n-Octadecanoic acid, methyl ester $$ Kemester 9718 $$ Me

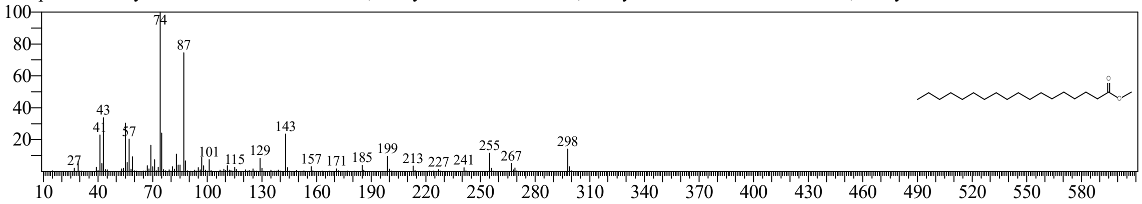

Hit#:5 Entry:28255 Library:NIST14s.lib
SI:97 Formula:C19H38O2 CAS:112-61-8 MolWeight:298 RetIndex:2077
CompName:Methyl stearate $$ Octadecanoic acid, methyl ester $$ Stearic acid, methyl ester $$ n-Octadecanoic acid, methyl ester $$ Kemester 9718 $$ Me

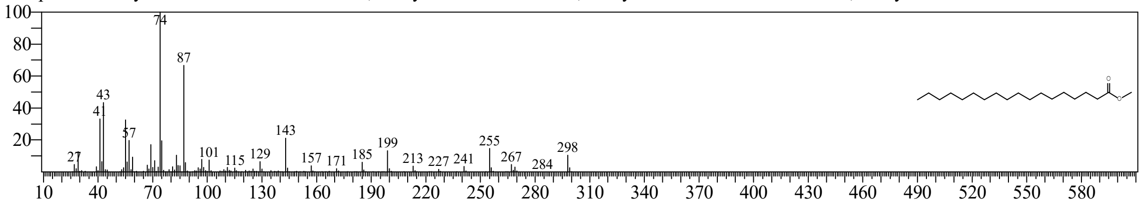

<< Target >>
Line#:10   R.Time:26.342(Scan#:1962) MassPeaks:370
RawMode:Averaged 26.333-26.350(1961-1963) BasePeak:55.05(211988)
BG Mode:Calc. from Peak Group 1 - Event 1

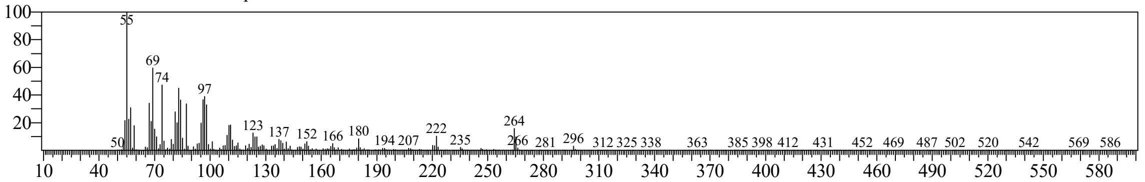

Hit#:1 Entry:28139 Library:NIST14s.lib
SI:97 Formula:C19H36O2 CAS:52380-33-3 MolWeight:296 RetIndex:2085
CompName:11-Octadecenoic acid, methyl ester $$ Methyl 11-octadecenoate $$ Octadec-11-enoic acid, methyl ester $$ Methyl (11E)-11-octadecenoate # $$

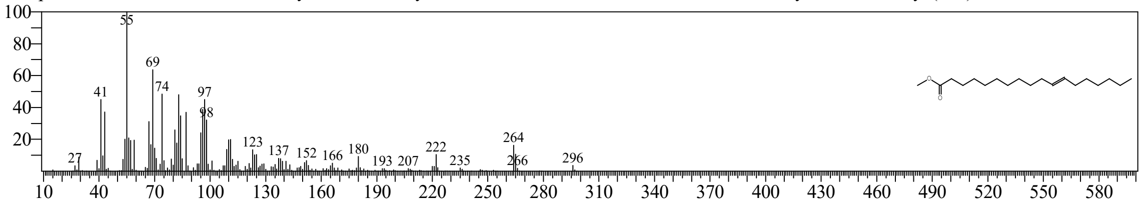

Hit#:2 Entry:32748 Library:NIST17s.lib
SI:97 Formula:C19H36O2 CAS:52380-33-3 MolWeight:296 RetIndex:2085
CompName:11-Octadecenoic acid, methyl ester $$ Methyl 11-octadecenoate $$ Octadec-11-enoic acid, methyl ester $$ Methyl (11E)-11-octadecenoate # $$

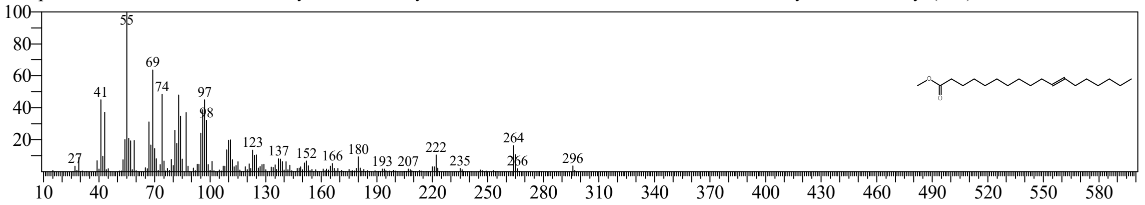

Hit#:3 Entry:139636 Library:NIST17-1.lib
SI:95 Formula:C19H36O2 CAS:13481-95-3 MolWeight:296 RetIndex:2085
CompName:10-Octadecenoic acid, methyl ester $$ Methyl 10-octadecenoate $$

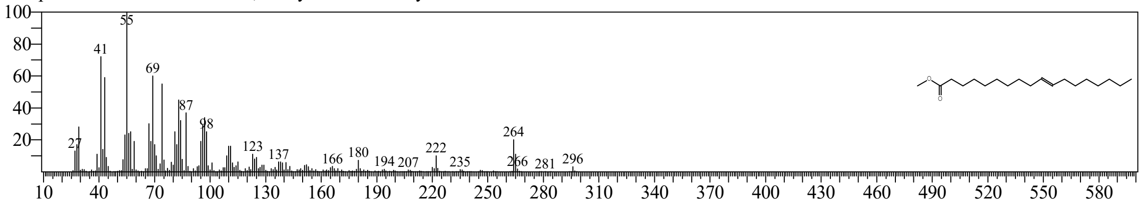

Hit#:4 Entry:127650 Library:NIST14.lib
SI:95 Formula:C19H36O2 CAS:13481-95-3 MolWeight:296 RetIndex:2085
CompName:10-Octadecenoic acid, methyl ester $$ Methyl 10-octadecenoate $$

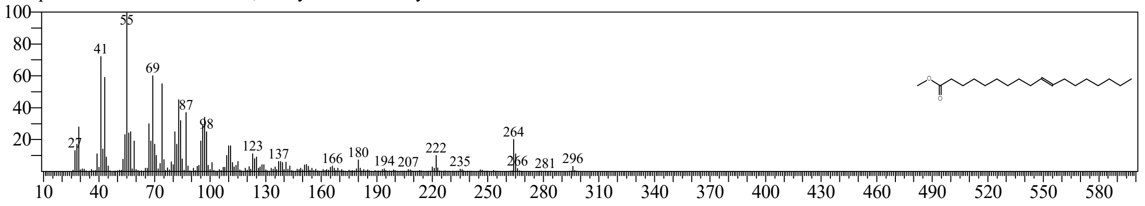

Hit#:5 Entry:127661 Library:NIST14.lib
SI:95 Formula:C19H36O2 CAS:0-00-0 MolWeight:296 RetIndex:2085
CompName:cis-13-Octadecenoic acid, methyl ester

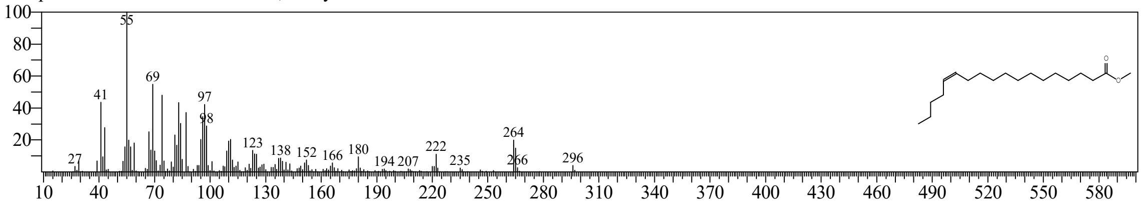

<< Target >>
Line#:11 R.Time:26.467(Scan#:1977) MassPeaks:347
RawMode:Averaged 26.458-26.475(1976-1978) BasePeak:55.10(14224)
BG Mode:Calc. from Peak Group 1 - Event 1

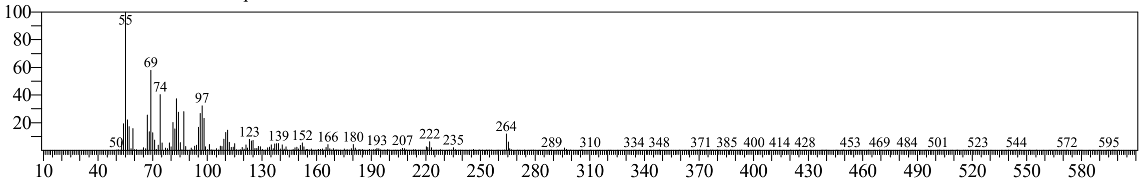

Hit#:1 Entry:127658 Library:NIST14.lib
SI:95 Formula:C19H36O2 CAS:0-00-0 MolWeight:296 RetIndex:2085
CompName:trans-13-Octadecenoic acid, methyl ester

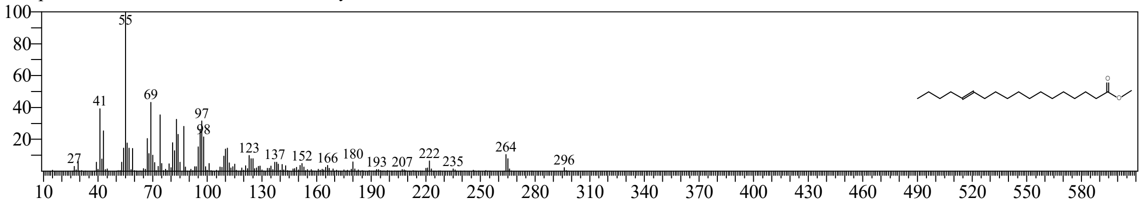

Hit#:2 Entry:139644 Library:NIST17-1.lib
SI:95 Formula:C19H36O2 CAS:0-00-0 MolWeight:296 RetIndex:2085
CompName:trans-13-Octadecenoic acid, methyl ester

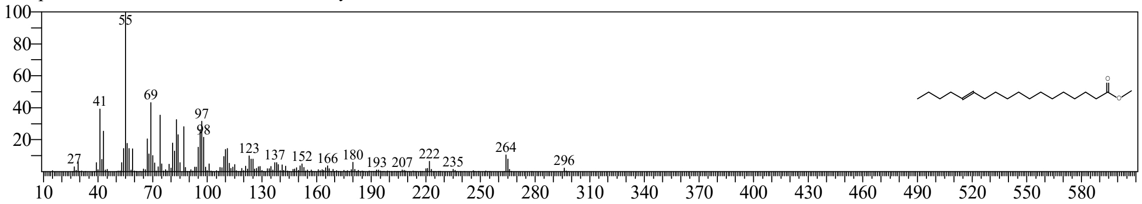

Hit#:3 Entry:139638 Library:NIST17-1.lib
SI:95 Formula:C19H36O2 CAS:56554-47-3 MolWeight:296 RetIndex:2085
CompName:13-Octadecenoic acid, methyl ester $$ Methyl (13E)-13-octadecenoate # $$

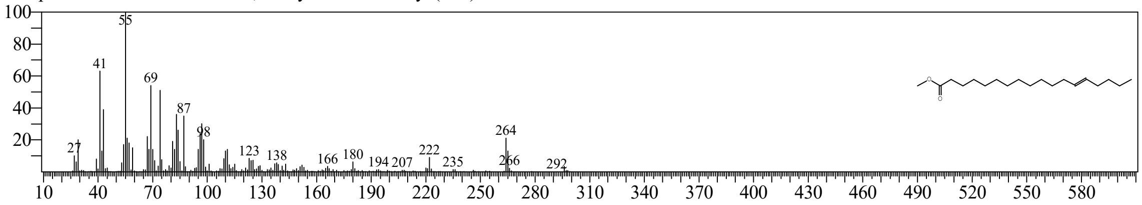

Hit#:4 Entry:127652 Library:NIST14.lib
SI:95 Formula:C19H36O2 CAS:56554-47-3 MolWeight:296 RetIndex:2085
CompName:13-Octadecenoic acid, methyl ester $$ Methyl (13E)-13-octadecenoate # $$

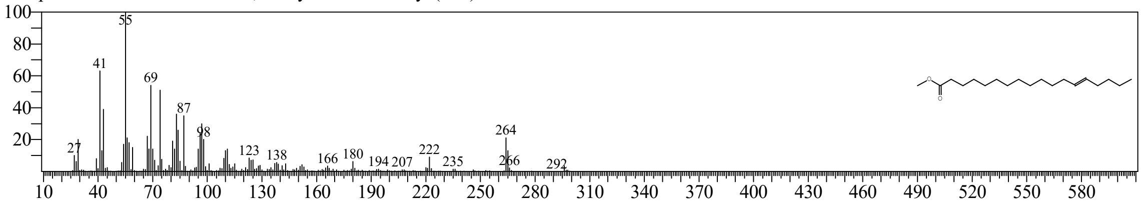

Hit#:5 Entry:28140 Library:NIST14s.lib
SI:94 Formula:C19H36O2 CAS:56554-47-3 MolWeight:296 RetIndex:2085
CompName:13-Octadecenoic acid, methyl ester $$ Methyl (13E)-13-octadecenoate # $$

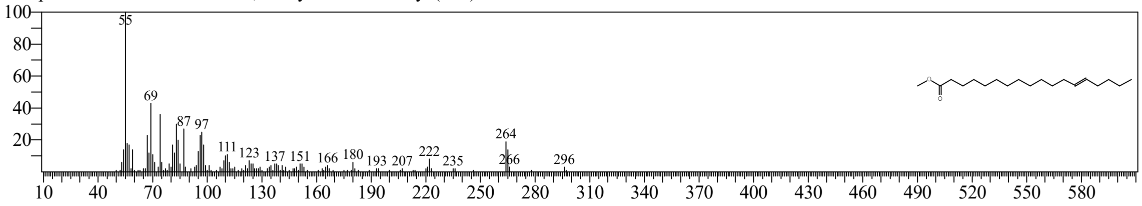

<< Target >>
Line#:12   R.Time:28.325(Scan#:2200) MassPeaks:366
RawMode:Averaged 28.317-28.333(2199-2201) BasePeak:67.05(99128)
BG Mode:Calc. from Peak Group 1 - Event 1

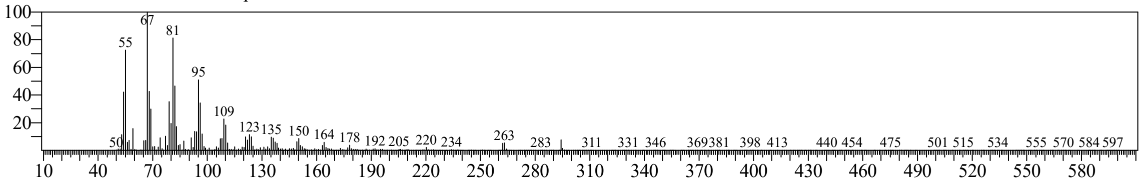

Hit#:1 Entry:137663 Library:NIST17-1.lib
SI:97 Formula:C19H34O2 CAS:2462-85-3 MolWeight:294 RetIndex:2093
CompName:9,12-Octadecadienoic acid, methyl ester $$ 9,12-Octadecenoic acid, methyl ester $$ Methyl octadeca-9,12-dienoate $$ Methyl 9,12-octadecadi

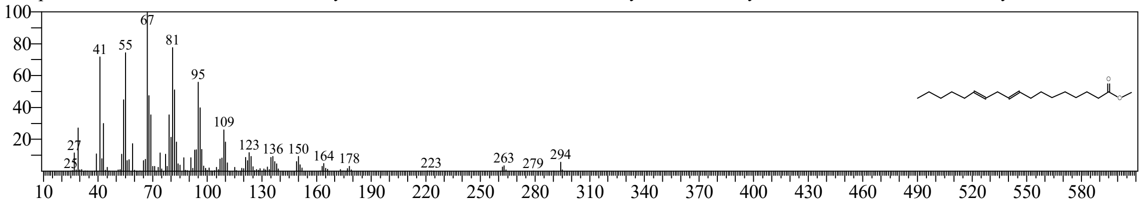

Hit#:2 Entry:125931 Library:NIST14.lib
SI:97 Formula:C19H34O2 CAS:2462-85-3 MolWeight:294 RetIndex:2093
CompName:9,12-Octadecadienoic acid, methyl ester $$ 9,12-Octadecenoic acid, methyl ester $$ Methyl octadeca-9,12-dienoate $$ Methyl 9,12-octadecadi

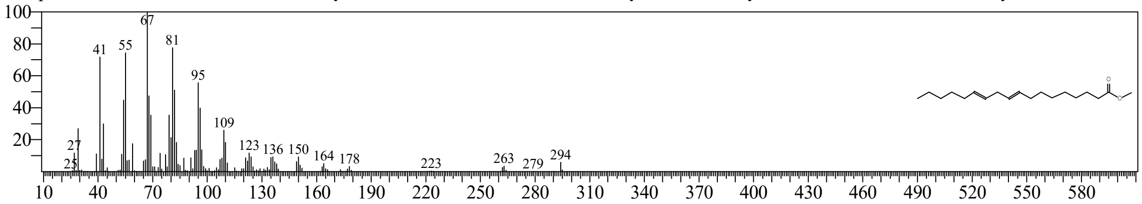

Hit#:3 Entry:32588 Library:NIST17s.lib
SI:96 Formula:C19H34O2 CAS:112-63-0 MolWeight:294 RetIndex:2093
CompName:9,12-Octadecadienoic acid (Z,Z)-, methyl ester $$ Linoleic acid, methyl ester $$ Methyl cis,cis-9,12-octadecadienoate $$ Methyl linoleate $$ M

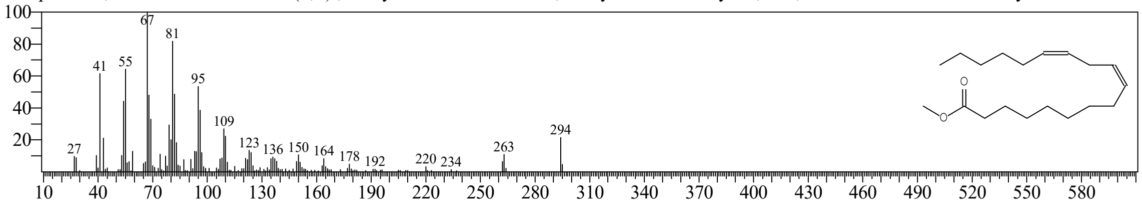

Hit#:4 Entry:27999 Library:NIST14s.lib
SI:96 Formula:C19H34O2 CAS:112-63-0 MolWeight:294 RetIndex:2093
CompName:9,12-Octadecadienoic acid (Z,Z)-, methyl ester $$ Linoleic acid, methyl ester $$ Methyl cis,cis-9,12-octadecadienoate $$ Methyl linoleate $$ M

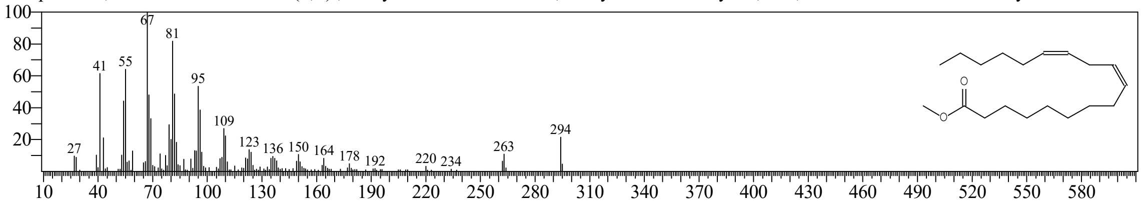

Hit#:5 Entry:32589 Library:NIST17s.lib
SI:96 Formula:C19H34O2 CAS:112-63-0 MolWeight:294 RetIndex:2093
CompName:9,12-Octadecadienoic acid (Z,Z)-, methyl ester $$ Linoleic acid, methyl ester $$ Methyl cis,cis-9,12-octadecadienoate $$ Methyl linoleate $$ M

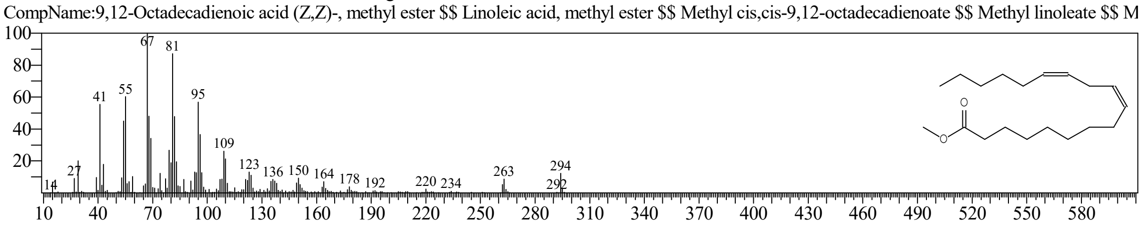

<< Target >>
Line#:13 R.Time:29.367(Scan#:2325) MassPeaks:381
RawMode:Averaged 29.358-29.375(2324-2326) BasePeak:74.05(9825)
BG Mode:Calc. from Peak Group 1 - Event 1

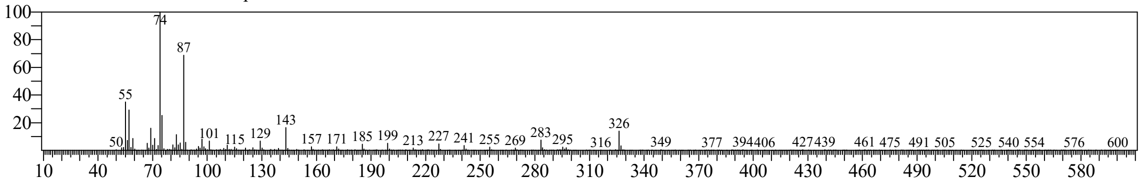

Hit#:1 Entry:34726 Library:NIST17s.lib
SI:96 Formula:C21H42O2 CAS:1120-28-1 MolWeight:326 RetIndex:2276
CompName:Eicosanoic acid, methyl ester $$ Methyl arachisate $$ Methyl eicosanoate $$ Arachidic acid methyl ester $$ Kemester 2050 $$ Methyl aracidat

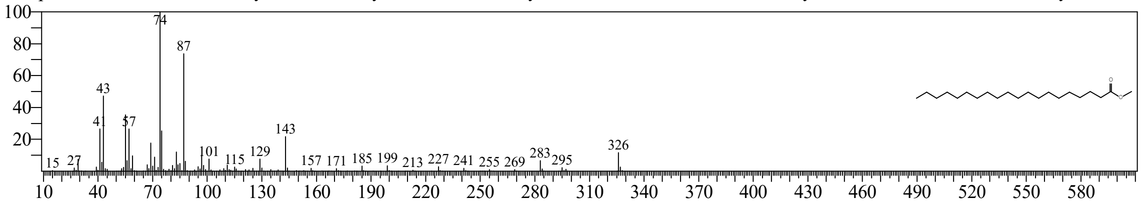

Hit#:2 Entry:154704 Library:NIST14.lib
SI:96 Formula:C21H42O2 CAS:0-00-0 MolWeight:326 RetIndex:2212
CompName:Methyl 18-methylnonadecanoate

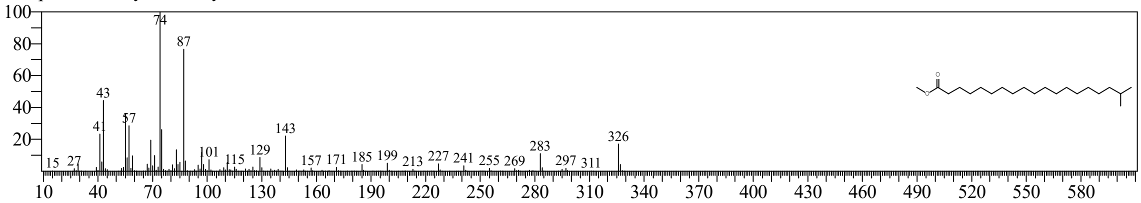

Hit#:3 Entry:170238 Library:NIST17-1.lib
SI:96 Formula:C21H42O2 CAS:0-00-0 MolWeight:326 RetIndex:2212
CompName:Methyl 18-methylnonadecanoate

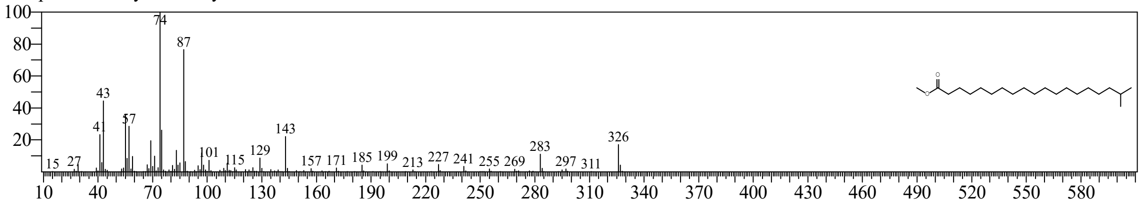

Hit#:4 Entry:29931 Library:NIST14s.lib
SI:94 Formula:C21H42O2 CAS:1120-28-1 MolWeight:326 RetIndex:2276
CompName:Eicosanoic acid, methyl ester $$ Methyl arachisate $$ Methyl eicosanoate $$ Arachidic acid methyl ester $$ Kemester 2050 $$ Methyl aracidat

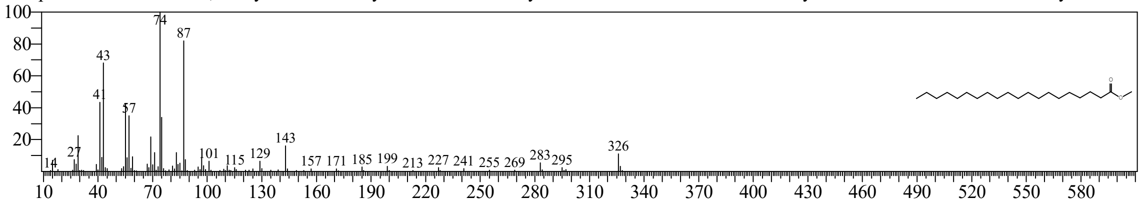

Hit#:5 Entry:34724 Library:NIST17s.lib
SI:94 Formula:C21H42O2 CAS:1120-28-1 MolWeight:326 RetIndex:2276
CompName:Eicosanoic acid, methyl ester $$ Methyl arachisate $$ Methyl eicosanoate $$ Arachidic acid methyl ester $$ Kemester 2050 $$ Methyl aracidat

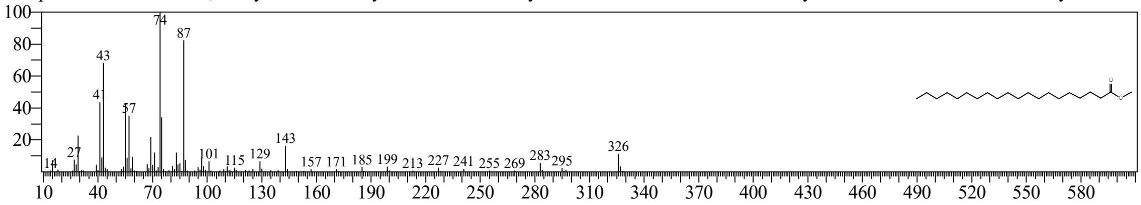

<< Target >>
Line#:14 R.Time:30.658(Scan#:2480) MassPeaks:272
RawMode:Averaged 30.650-30.667(2479-2481) BasePeak:79.10(1057)
BG Mode:Calc. from Peak Group 1 - Event 1

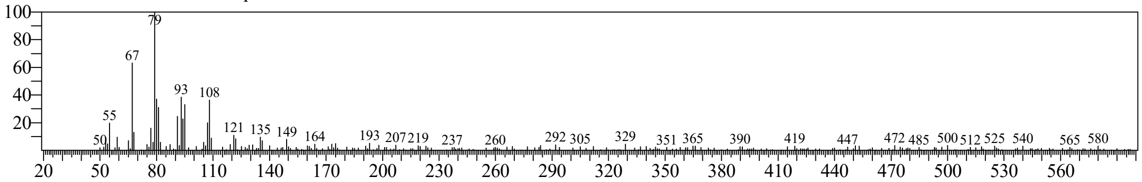

Hit#:1 Entry:29609 Library:NIST14s.lib
SI:84 Formula:C21H36O2 CAS:55682-88-7 MolWeight:320 RetIndex:2300
CompName:11,14,17-Eicosatrienoic acid, methyl ester $$ Methyl 11,14,17-icosatrienoate $$ Methyl 11,14,17-eicosatrienoate $$

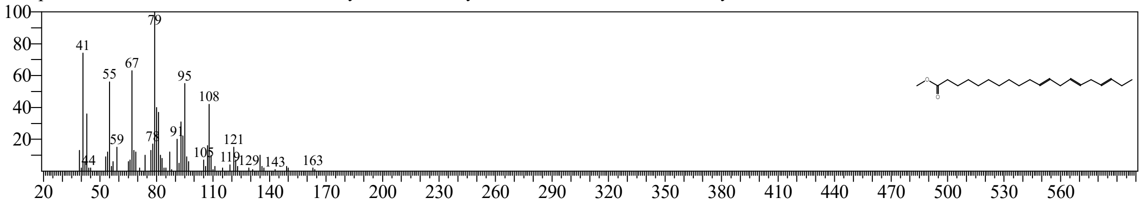

Hit#:2 Entry:34362 Library:NIST17s.lib
SI:84 Formula:C21H36O2 CAS:55682-88-7 MolWeight:320 RetIndex:2300
CompName:11,14,17-Eicosatrienoic acid, methyl ester $$ Methyl 11,14,17-icosatrienoate $$ Methyl 11,14,17-eicosatrienoate $$

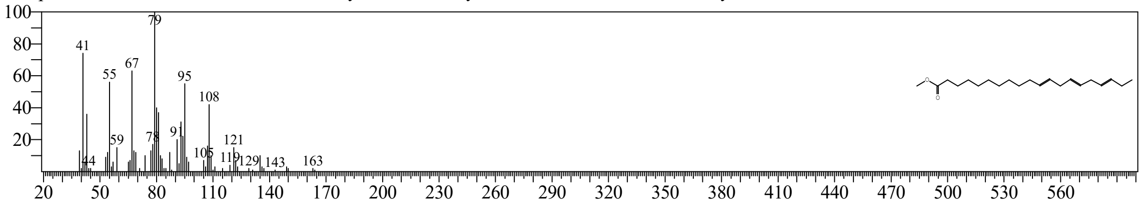

Hit#:3 Entry:94157 Library:NIST17-1.lib
SI:84 Formula:C16H26O2 CAS:7561-64-0 MolWeight:250 RetIndex:1992
CompName:7,10,13-Hexadecatrienoic acid, (Z,Z,Z)- $$ 7,10,13-Hexadecatrienoic acid, (7Z,10Z,13Z)- $$ (7Z,10Z,13Z)-7,10,13-Hexadecatrienoic acid $$ (

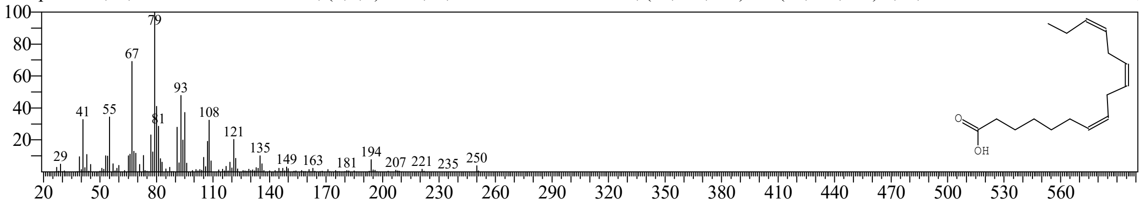

Hit#:4 Entry:72462 Library:NIST14.lib
SI:84 Formula:C17H28 CAS:10482-53-8 MolWeight:232 RetIndex:0
CompName:1,8,11,14-Heptadecatetraene, (Z,Z,Z)-

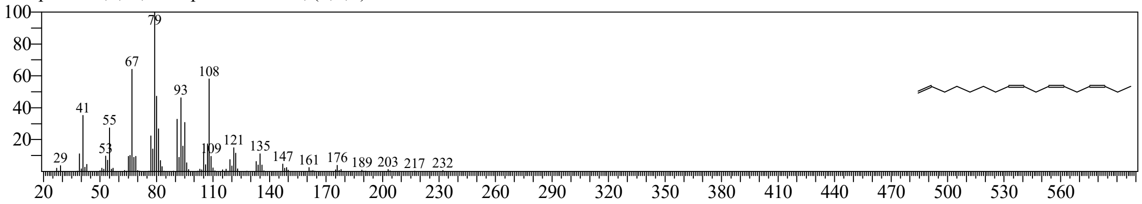

Hit#:5 Entry:77643 Library:NIST17-1.lib
SI:84 Formula:C17H28 CAS:10482-53-8 MolWeight:232 RetIndex:1725
CompName:1,8,11,14-Heptadecatetraene, (Z,Z,Z)-

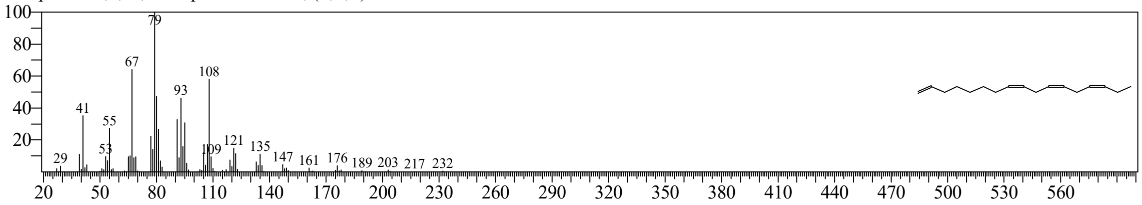

<< Target >>
Line#:15 R.Time:30.750(Scan#:2491) MassPeaks:270
RawMode:Averaged 30.742-30.758(2490-2492) BasePeak:55.05(1364)
BG Mode:Calc. from Peak Group 1 - Event 1

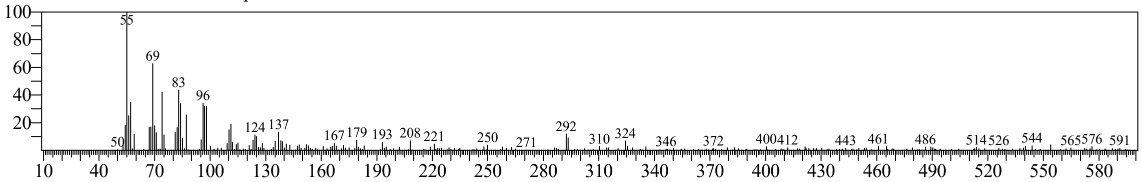

Hit#:1 Entry:152749 Library:NIST14.lib
SI:91 Formula:C21H40O2 CAS:2390-09-2 MolWeight:324 RetIndex:0
CompName:cis-Methyl 11-eicosenoate $$ 11-Eicosenoic acid, methyl ester, (Z)- $$

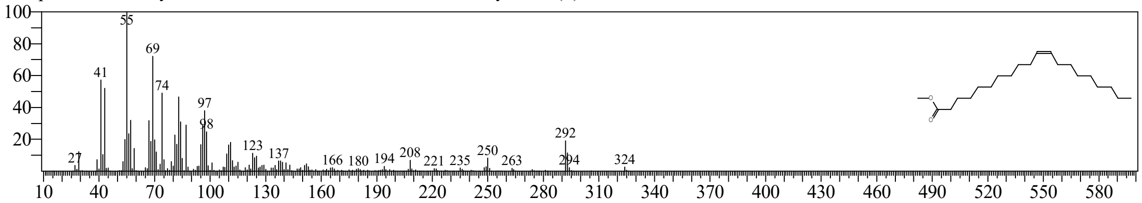

Hit#:2 Entry:152750 Library:NIST14.lib
SI:89 Formula:C21H40O2 CAS:3946-08-5 MolWeight:324 RetIndex:2284
CompName:11-Eicosenoic acid, methyl ester $$ Methyl (11E)-11-icosenoate # $$

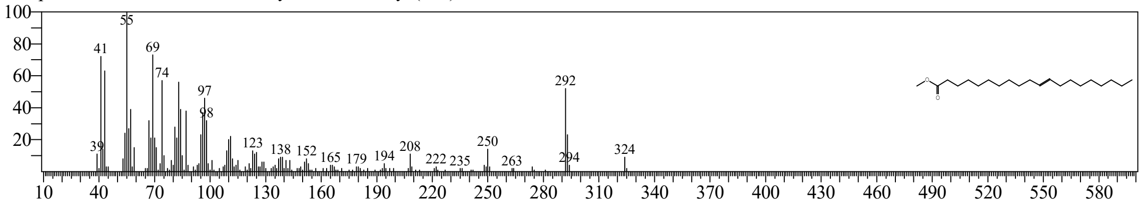

Hit#:3 Entry:168023 Library:NIST17-1.lib
SI:89 Formula:C21H40O2 CAS:3946-08-5 MolWeight:324 RetIndex:2284
CompName:11-Eicosenoic acid, methyl ester $$ Methyl (11E)-11-icosenoate # $$

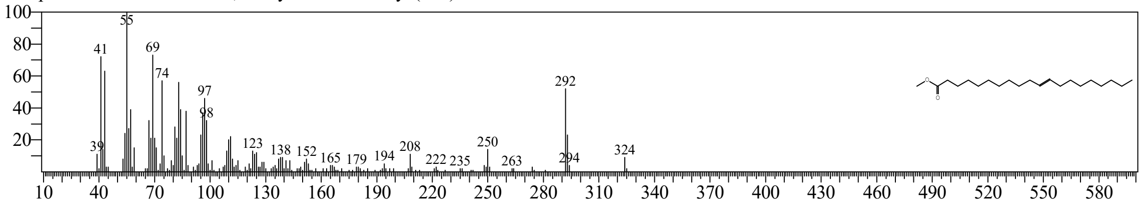

Hit#:4 Entry:152755 Library:NIST14.lib
SI:89 Formula:C21H40O2 CAS:0-00-0 MolWeight:324 RetIndex:2284
CompName:cis-11-Eicosenoic acid, methyl ester

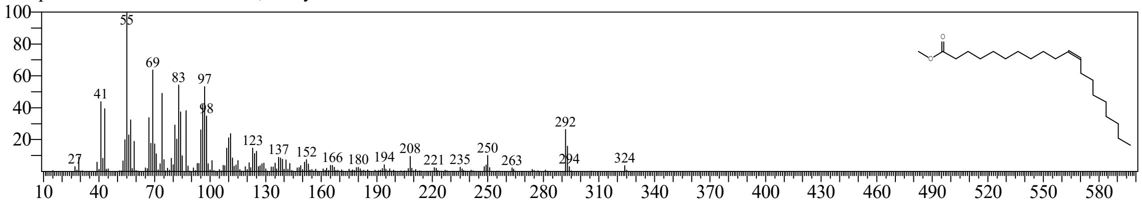

Hit#:5 Entry:168029 Library:NIST17-1.lib
SI:89 Formula:C21H40O2 CAS:2390-09-2 MolWeight:324 RetIndex:2284
CompName:cis-Methyl 11-eicosenoate $$ 11-Eicosenoic acid, methyl ester, (Z)- $$

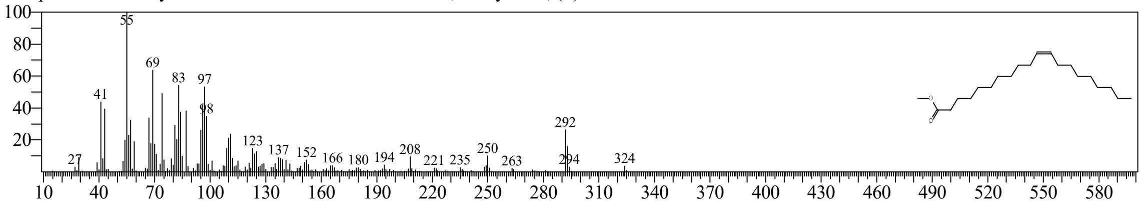

<< Target >>
Line#:16 R.Time:31.017(Scan#:2523) MassPeaks:293
RawMode:Averaged 31.008-31.025(2522-2524) BasePeak:67.05(3823)
BG Mode:Calc. from Peak Group 1 - Event 1

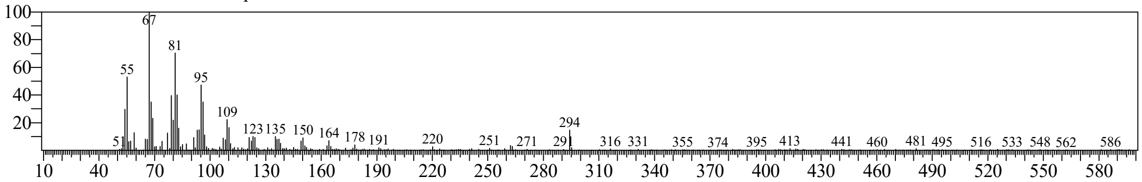

Hit#:1 Entry:32587 Library:NIST17s.lib
SI:96 Formula:C19H34O2 CAS:13038-47-6 MolWeight:294 RetIndex:2093
CompName:9,11-Octadecadienoic acid, methyl ester, (E,E)- $$ Methyl trans-9,trans-11-octadecadienoate $$ Methyl trans,trans-9,11-octadecadienoate $$ M

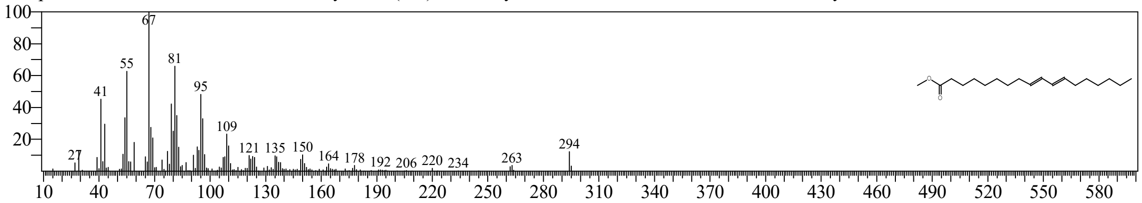

Hit#:2 Entry:137664 Library:NIST17-1.lib
SI:96 Formula:C19H34O2 CAS:0-00-0 MolWeight:294 RetIndex:2093
CompName:Methyl 10-trans,12-cis-octadecadienoate

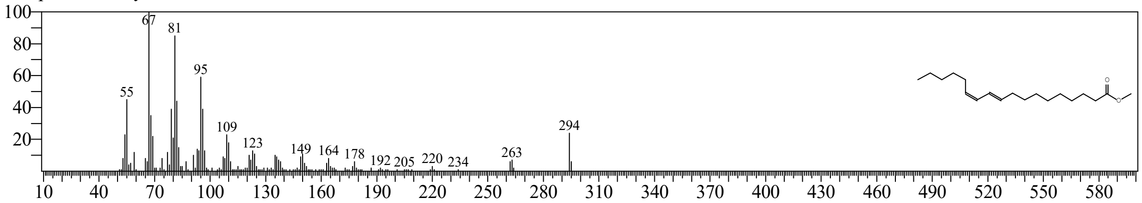

Hit#:3 Entry:125933 Library:NIST14.lib
SI:96 Formula:C19H34O2 CAS:0-00-0 MolWeight:294 RetIndex:2093
CompName:Methyl 10-trans,12-cis-octadecadienoate

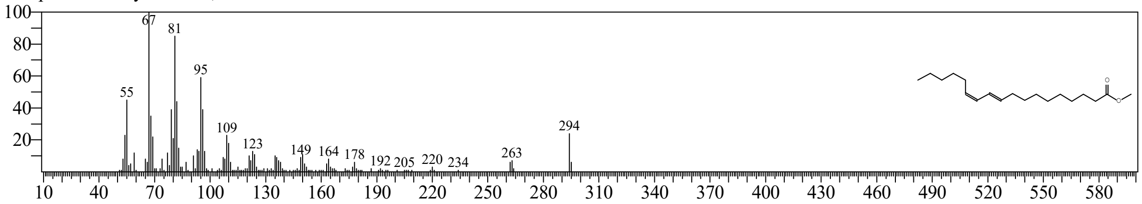

Hit#:4 Entry:137666 Library:NIST17-1.lib
SI:96 Formula:C19H34O2 CAS:13058-52-1 MolWeight:294 RetIndex:2093
CompName:Methyl 9-cis,11-trans-octadecadienoate

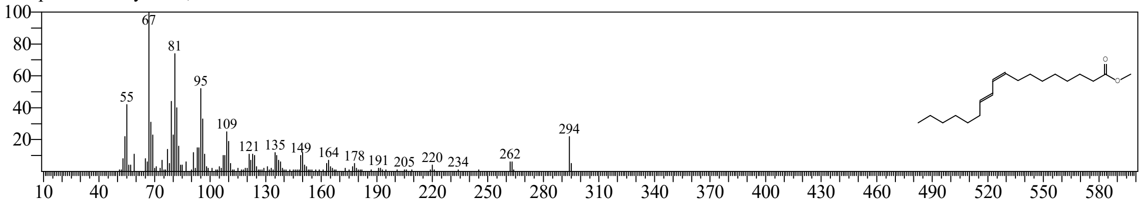

Hit#:5 Entry:125932 Library:NIST14.lib
SI:96 Formula:C19H34O2 CAS:0-00-0 MolWeight:294 RetIndex:2093
CompName:Methyl 9-cis,11-trans-octadecadienoate

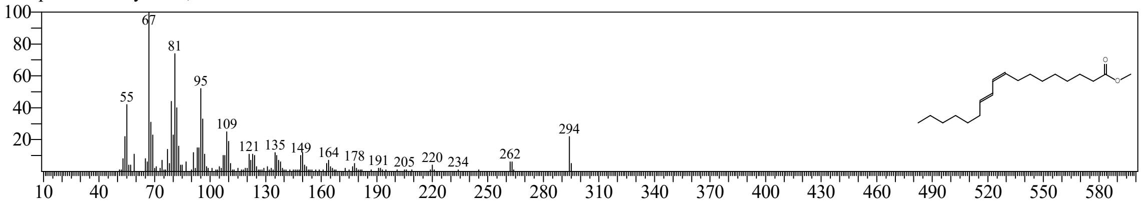

<< Target >>
Line#:17 R.Time:31.333(Scan#:2561) MassPeaks:345
RawMode:Averaged 31.325-31.342(2560-2562) BasePeak:67.10(3909)
BG Mode:Calc. from Peak Group 1 - Event 1

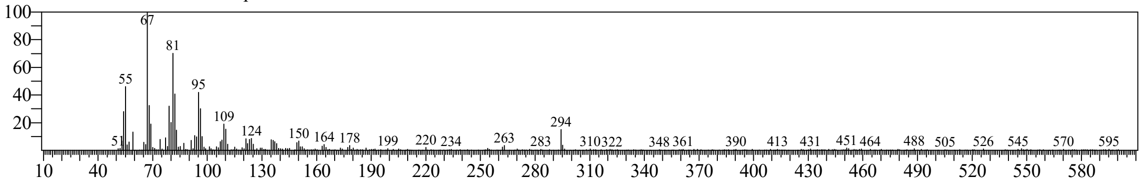

Hit#:1 Entry:32586 Library:NIST17s.lib
SI:94 Formula:C19H34O2 CAS:56554-61-1 MolWeight:294 RetIndex:2093
CompName:11,14-Octadecadienoic acid, methyl ester $$ Methyl (11E,14E)-11,14-octadecadienoate # $$

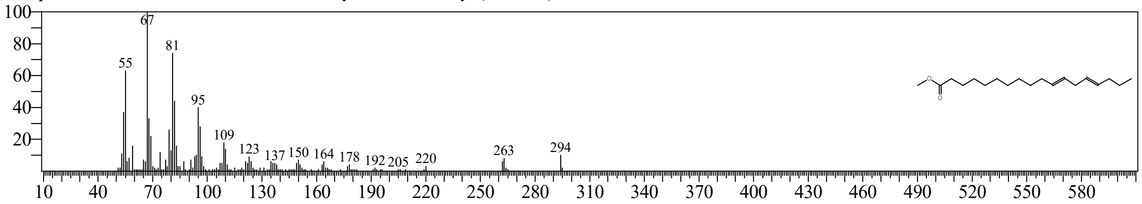

Hit#:2 Entry:27998 Library:NIST14s.lib
SI:94 Formula:C19H34O2 CAS:56554-61-1 MolWeight:294 RetIndex:2093
CompName:11,14-Octadecadienoic acid, methyl ester $$ Methyl (11E,14E)-11,14-octadecadienoate # $$

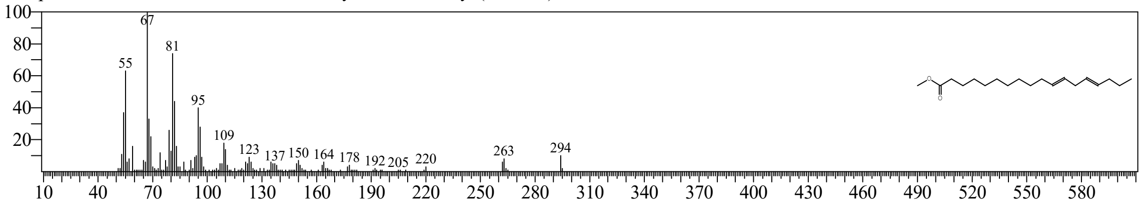

Hit#:3 Entry:32587 Library:NIST17s.lib
SI:94 Formula:C19H34O2 CAS:13038-47-6 MolWeight:294 RetIndex:2093
CompName:9,11-Octadecadienoic acid, methyl ester, (E,E)- $$ Methyl trans-9,trans-11-octadecadienoate $$ Methyl trans,trans-9,11-octadecadienoate $$ M

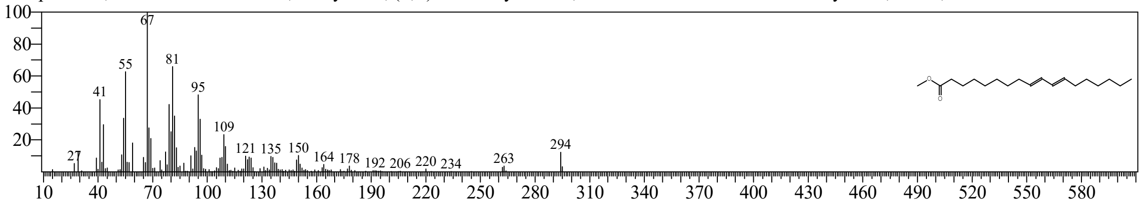

Hit#:4 Entry:125933 Library:NIST14.lib
SI:94 Formula:C19H34O2 CAS:0-00-0 MolWeight:294 RetIndex:2093
CompName:Methyl 10-trans,12-cis-octadecadienoate

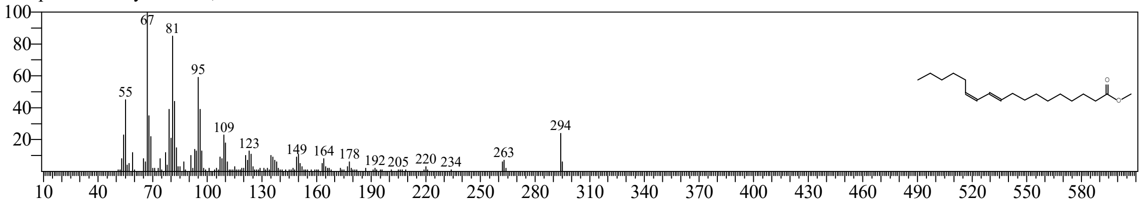

Hit#:5 Entry:125932 Library:NIST14.lib
SI:94 Formula:C19H34O2 CAS:0-00-0 MolWeight:294 RetIndex:2093
CompName:Methyl 9-cis,11-trans-octadecadienoate

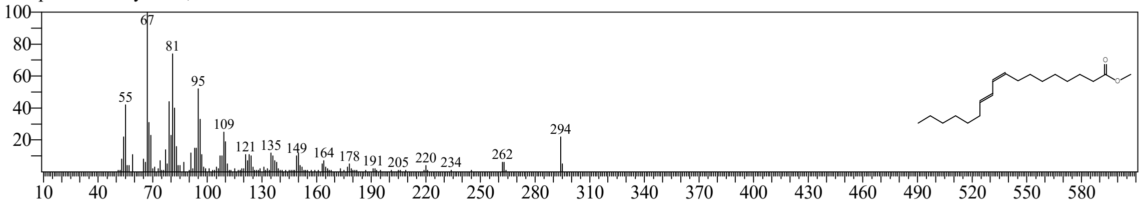

<< Target >>
Line#:18 R.Time:32.017(Scan#:2643) MassPeaks:310
RawMode:Averaged 32.008-32.025(2642-2644) BasePeak:67.05(2626)
BG Mode:Calc. from Peak Group 1 - Event 1

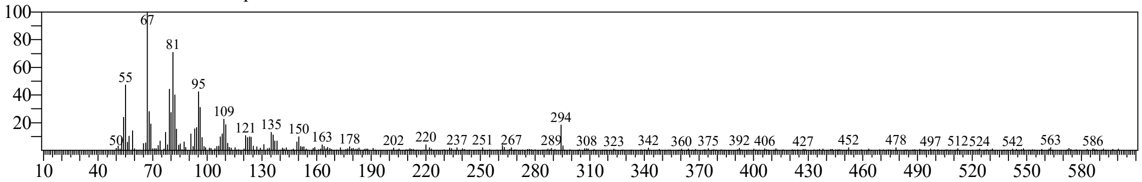

Hit#:1 Entry:137666 Library:NIST17-1.lib
SI:95 Formula:C19H34O2 CAS:13058-52-1 MolWeight:294 RetIndex:2093
CompName:Methyl 9-cis,11-trans-octadecadienoate

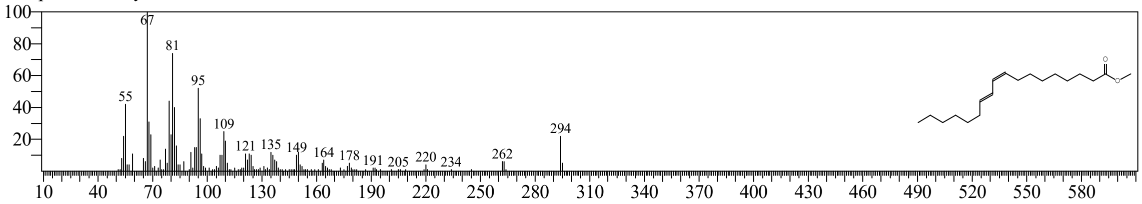

Hit#:2 Entry:125932 Library:NIST14.lib
SI:95 Formula:C19H34O2 CAS:0-00-0 MolWeight:294 RetIndex:2093
CompName:Methyl 9-cis,11-trans-octadecadienoate

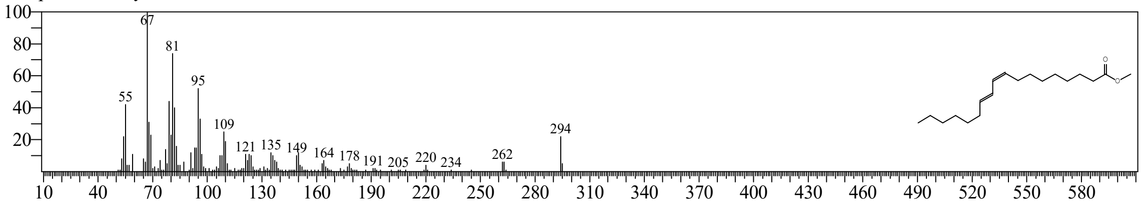

Hit#:3 Entry:32587 Library:NIST17s.lib
SI:95 Formula:C19H34O2 CAS:13038-47-6 MolWeight:294 RetIndex:2093
CompName:9,11-Octadecadienoic acid, methyl ester, (E,E)- $$ Methyl trans-9,trans-11-octadecadienoate $$ Methyl trans,trans-9,11-octadecadienoate $$ M

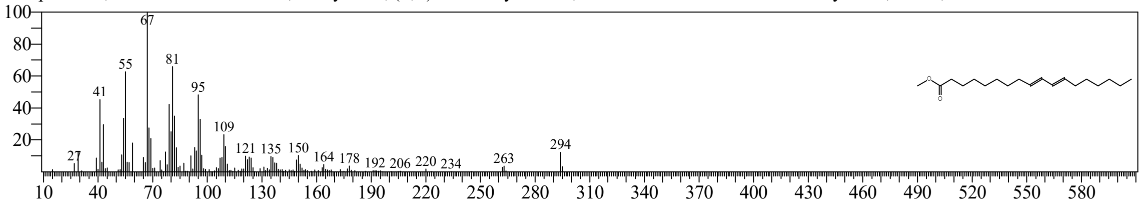

Hit#:4 Entry:125933 Library:NIST14.lib
SI:93 Formula:C19H34O2 CAS:0-00-0 MolWeight:294 RetIndex:2093
CompName:Methyl 10-trans,12-cis-octadecadienoate

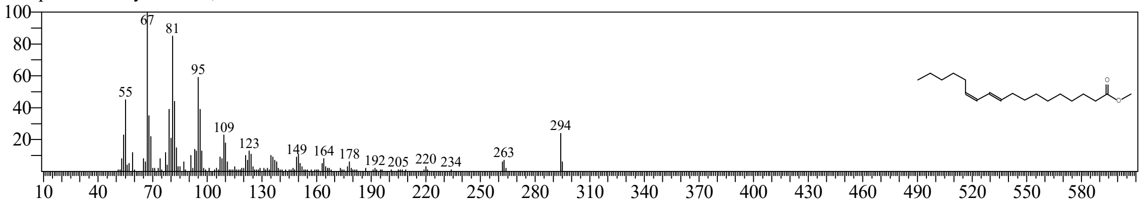

Hit#:5 Entry:137664 Library:NIST17-1.lib
SI:93 Formula:C19H34O2 CAS:0-00-0 MolWeight:294 RetIndex:2093
CompName:Methyl 10-trans,12-cis-octadecadienoate

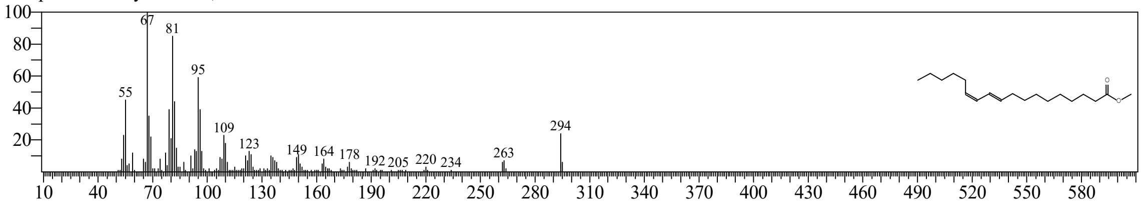

<< Target >>
Line#:19 R.Time:32.450(Scan#:2695) MassPeaks:307
RawMode:Averaged 32.442-32.458(2694-2696) BasePeak:71.05(5176)
BG Mode:Calc. from Peak Group 1 - Event 1

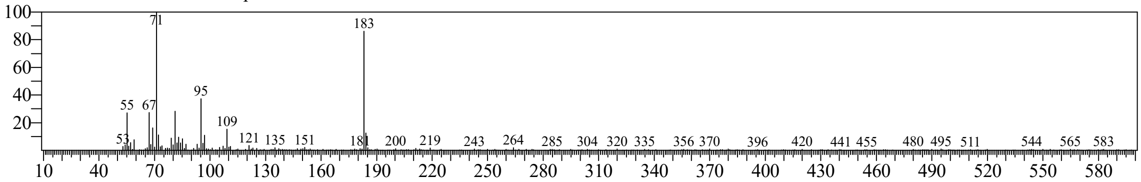

Hit#:1 Entry:72357 Library:NIST17-1.lib
SI:77 Formula:C14H26O2 CAS:2146-71-6 MolWeight:226 RetIndex:1570
CompName:Dodecanoic acid, ethenyl ester $$ Lauric acid, vinyl ester $$ Vinyl laurate $$ Vinyl dodecanoate $$

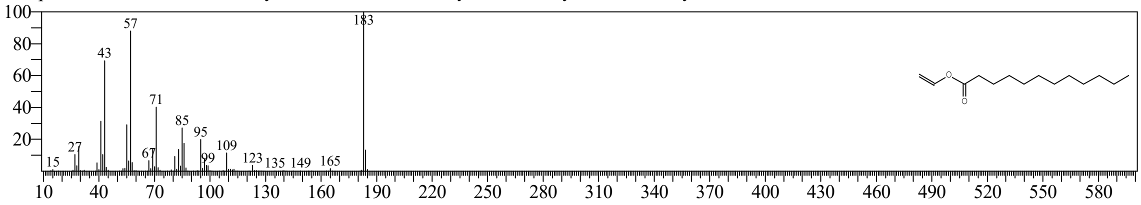

Hit#:2 Entry:67683 Library:NIST14.lib
SI:77 Formula:C14H26O2 CAS:2146-71-6 MolWeight:226 RetIndex:1570
CompName:Dodecanoic acid, ethenyl ester $$ Lauric acid, vinyl ester $$ Vinyl laurate $$ Vinyl dodecanoate $$

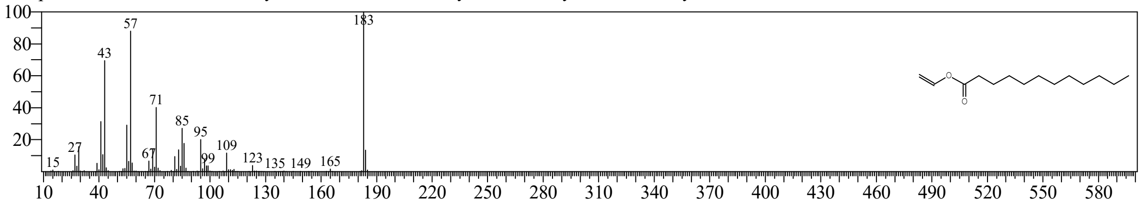

Hit#:3 Entry:196117 Library:NIST17-1.lib
SI:76 Formula:C21H38O4 CAS:0-00-0 MolWeight:354 RetIndex:2387
CompName:Succinic acid, dodec-2-enyl 3-methylbutyl ester

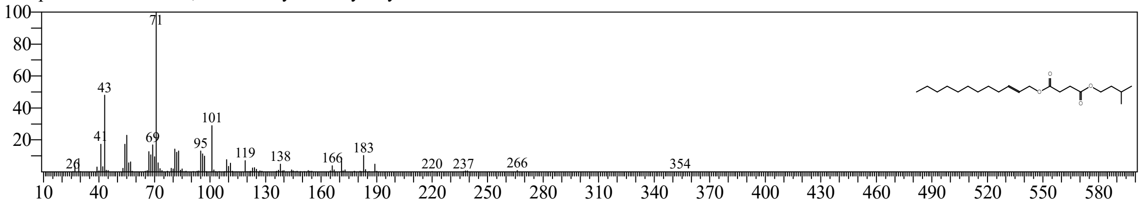

Hit#:4 Entry:177742 Library:NIST14.lib
SI:76 Formula:C21H38O4 CAS:0-00-0 MolWeight:354 RetIndex:2387
CompName:Succinic acid, dodec-2-enyl 3-methylbutyl ester

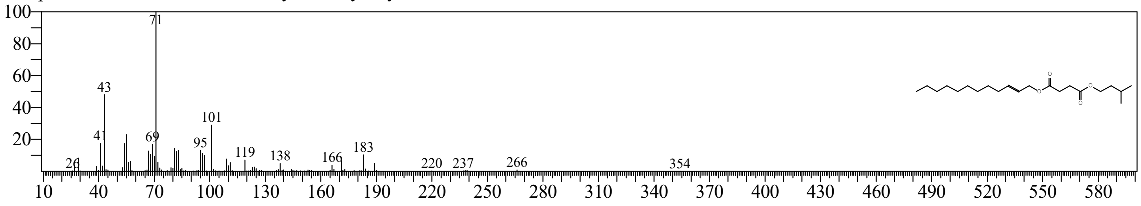

Hit#:5 Entry:56932 Library:NIST14.lib
SI:76 Formula:C13H27NO CAS:832-29-1 MolWeight:213 RetIndex:1893
CompName:Cyclododecanol, 1-aminomethyl- $$ 1-(Aminomethyl)cyclododecanol # $$

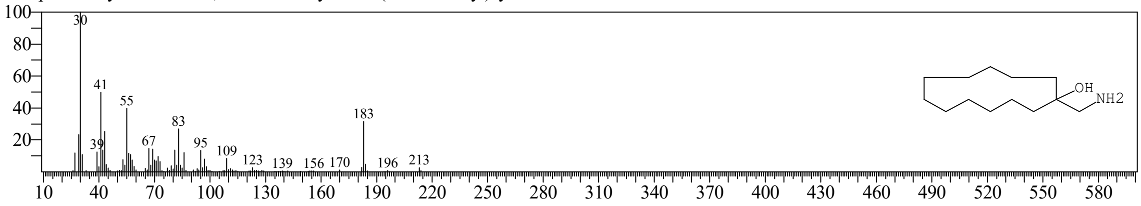

<< Target >>
Line#:20 R.Time:33.608(Scan#:2834) MassPeaks:341
RawMode:Averaged 33.600-33.617(2833-2835) BasePeak:74.05(10077)
BG Mode:Calc. from Peak Group 1 - Event 1

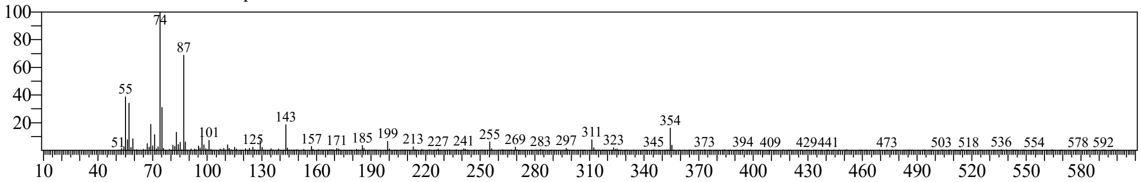

Hit#:1 Entry:178051 Library:NIST14.lib
SI:97 Formula:C23H46O2 CAS:929-77-1 MolWeight:354 RetIndex:2475
CompName:Docosanoic acid, methyl ester $$ Behenic acid, methyl ester $$ Methyl behenate $$ Methyl docosanoate $$ n-Docosanoic acid methyl ester $$

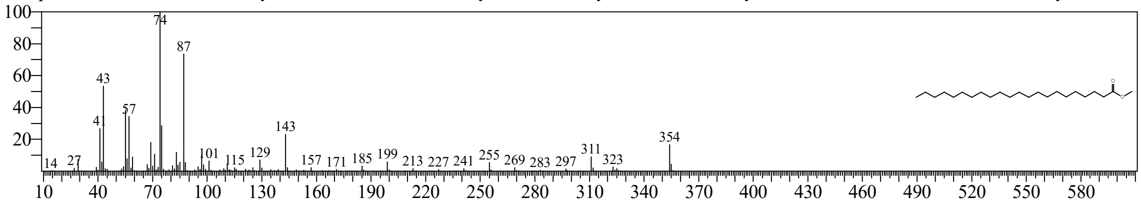

Hit#:2 Entry:196443 Library:NIST17-1.lib
SI:97 Formula:C23H46O2 CAS:929-77-1 MolWeight:354 RetIndex:2475
CompName:Docosanoic acid, methyl ester $$ Behenic acid, methyl ester $$ Methyl behenate $$ Methyl docosanoate $$ n-Docosanoic acid methyl ester $$

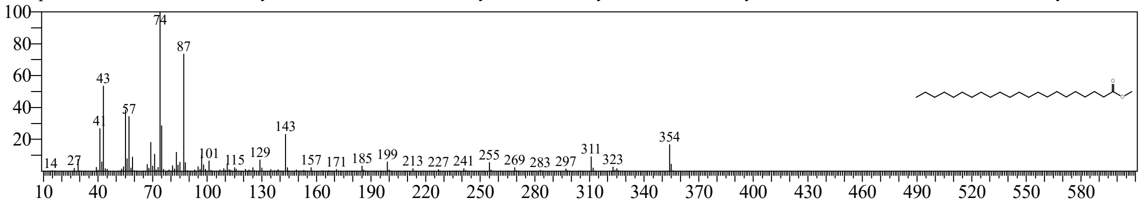

Hit#:3 Entry:31087 Library:NIST14s.lib
SI:95 Formula:C23H46O2 CAS:929-77-1 MolWeight:354 RetIndex:2475
CompName:Docosanoic acid, methyl ester $$ Behenic acid, methyl ester $$ Methyl behenate $$ Methyl docosanoate $$ n-Docosanoic acid methyl ester $$

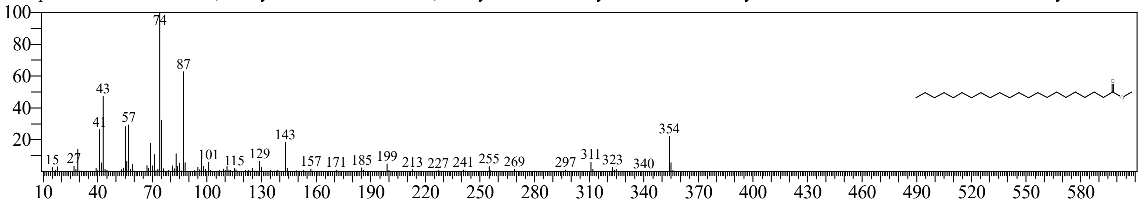

Hit#:4 Entry:36092 Library:NIST17s.lib
SI:95 Formula:C23H46O2 CAS:929-77-1 MolWeight:354 RetIndex:2475
CompName:Docosanoic acid, methyl ester $$ Behenic acid, methyl ester $$ Methyl behenate $$ Methyl docosanoate $$ n-Docosanoic acid methyl ester $$

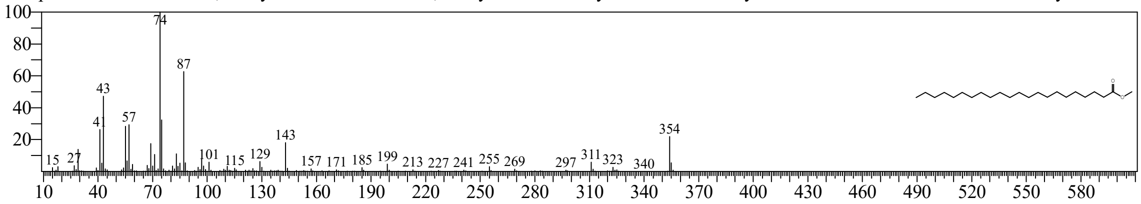

Hit#:5 Entry:36091 Library:NIST17s.lib
SI:95 Formula:C23H46O2 CAS:929-77-1 MolWeight:354 RetIndex:2475
CompName:Docosanoic acid, methyl ester $$ Behenic acid, methyl ester $$ Methyl behenate $$ Methyl docosanoate $$ n-Docosanoic acid methyl ester $$

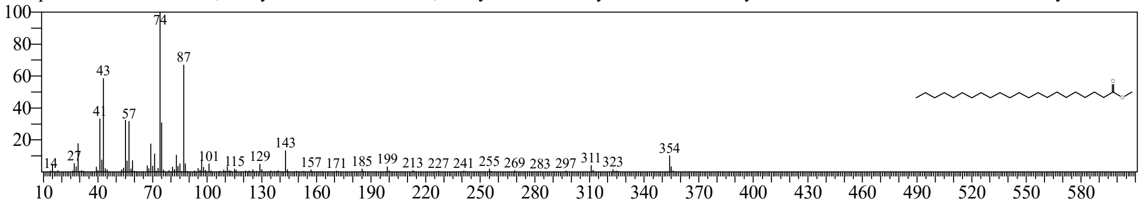

<< Target >>
Line#:21 R.Time:37.858(Scan#:3344) MassPeaks:294
RawMode:Averaged 37.850-37.867(3343-3345) BasePeak:79.05(3758)
BG Mode:Calc. from Peak Group 1 - Event 1

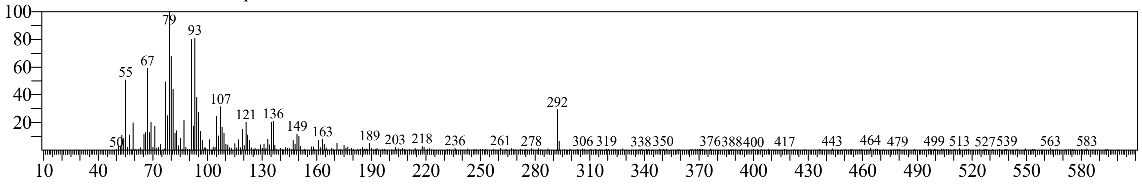

Hit#:1 Entry:124230 Library:NIST14.lib
SI:91 Formula:C19H32O2 CAS:0-00-0 MolWeight:292 RetIndex:2101
CompName:Methyl 9.cis.,11.trans.t,13.trans.-octadecatrienoate

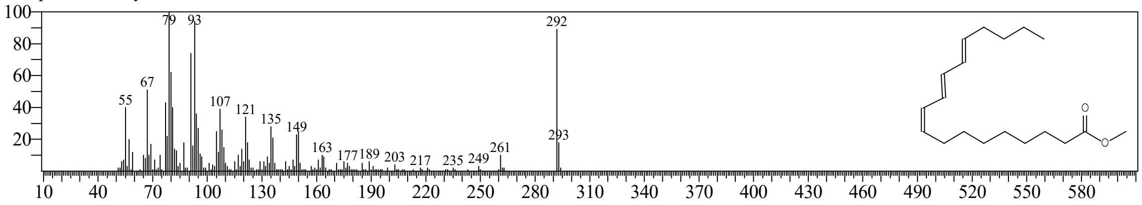

Hit#:2 Entry:135726 Library:NIST17-1.lib
SI:91 Formula:C19H32O2 CAS:0-00-0 MolWeight:292 RetIndex:2101
CompName:Methyl 9.cis.,11.trans.t,13.trans.-octadecatrienoate

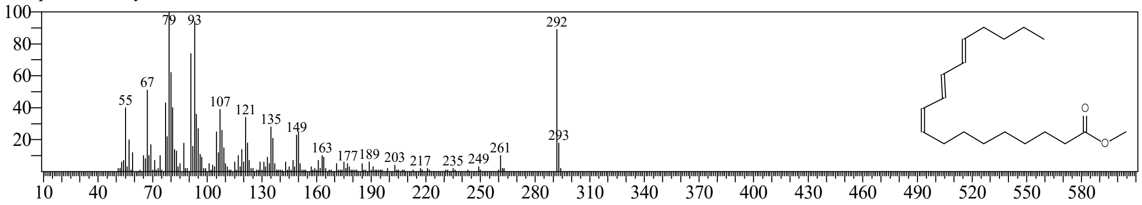

Hit#:3 Entry:135722 Library:NIST17-1.lib
SI:91 Formula:C19H32O2 CAS:0-00-0 MolWeight:292 RetIndex:2101
CompName:Methyl 6-cis,9-cis,11-trans-octadecatrienoate

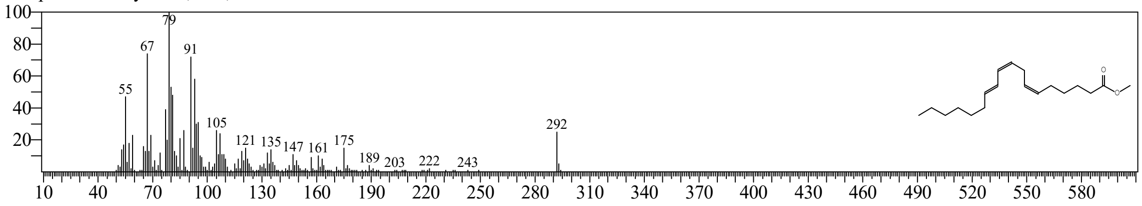

Hit#:4 Entry:124226 Library:NIST14.lib
SI:91 Formula:C19H32O2 CAS:0-00-0 MolWeight:292 RetIndex:2101
CompName:Methyl 6-cis,9-cis,11-trans-octadecatrienoate

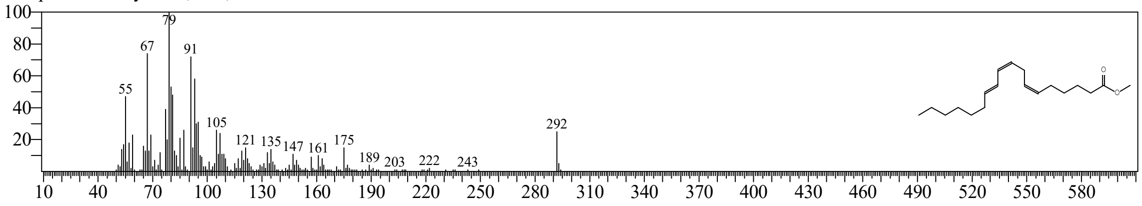

Hit#:5 Entry:124225 Library:NIST14.lib
SI:89 Formula:C19H32O2 CAS:0-00-0 MolWeight:292 RetIndex:2101
CompName:.gamma.-Linolenic acid, methyl ester

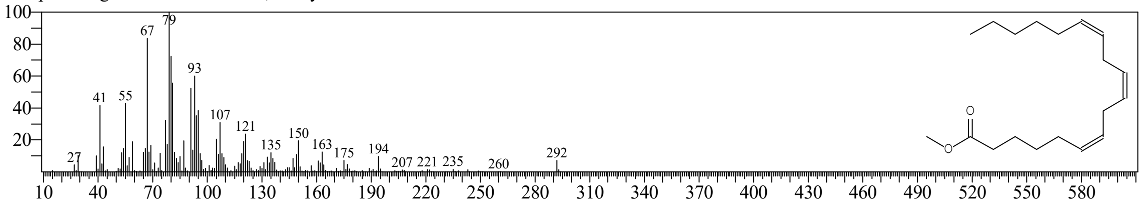

<< Target >>
Line#:22 R.Time:38.425(Scan#:3412) MassPeaks:304
RawMode:Averaged 38.417-38.433(3411-3413) BasePeak:79.10(4091)
BG Mode:Calc. from Peak Group 1 - Event 1

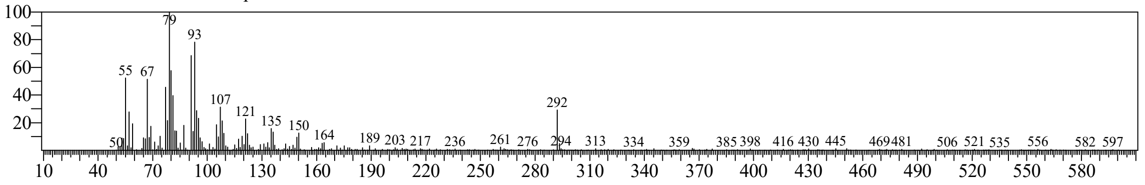

Hit#:1 Entry:135726 Library:NIST17-1.lib
SI:92 Formula:C19H32O2 CAS:0-00-0 MolWeight:292 RetIndex:2101
CompName:Methyl 9.cis.,11.trans.t,13.trans.-octadecatrienoate

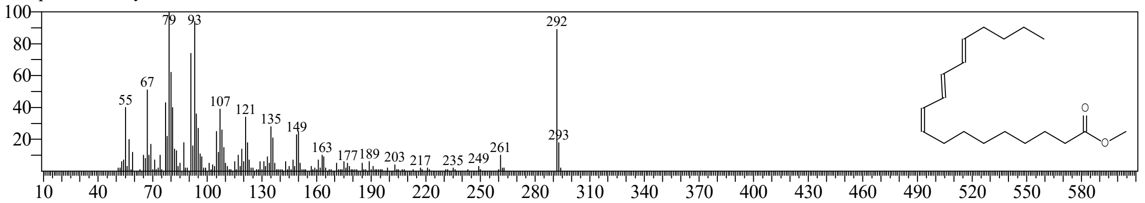

Hit#:2 Entry:124230 Library:NIST14.lib
SI:92 Formula:C19H32O2 CAS:0-00-0 MolWeight:292 RetIndex:2101
CompName:Methyl 9.cis.,11.trans.t,13.trans.-octadecatrienoate

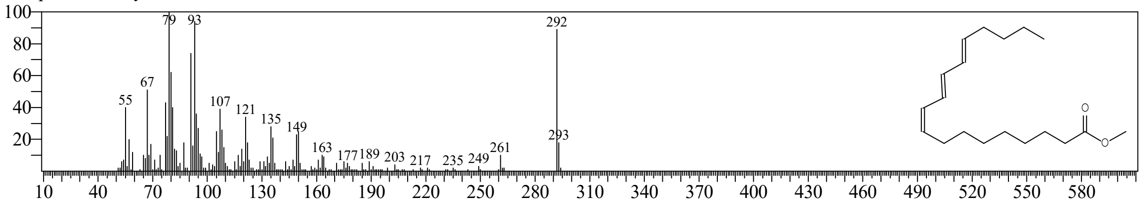

Hit#:3 Entry:124226 Library:NIST14.lib
SI:90 Formula:C19H32O2 CAS:0-00-0 MolWeight:292 RetIndex:2101
CompName:Methyl 6-cis,9-cis,11-trans-octadecatrienoate

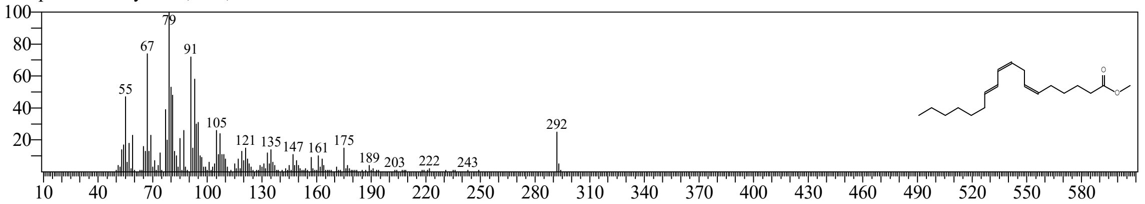

Hit#:4 Entry:135722 Library:NIST17-1.lib
SI:90 Formula:C19H32O2 CAS:0-00-0 MolWeight:292 RetIndex:2101
CompName:Methyl 6-cis,9-cis,11-trans-octadecatrienoate

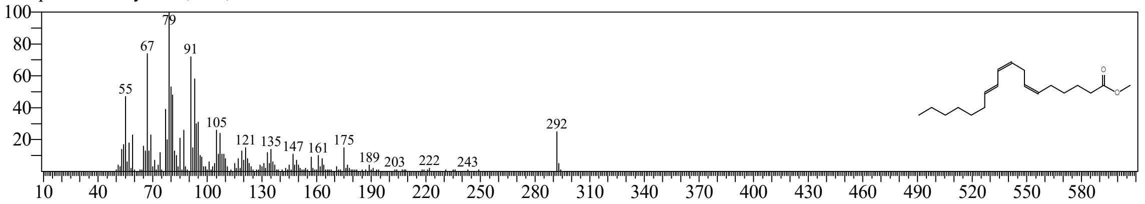

Hit#:5 Entry:135721 Library:NIST17-1.lib
SI:90 Formula:C19H32O2 CAS:16326-32-2 MolWeight:292 RetIndex:2101
CompName:Methyl .gamma.-linolenate

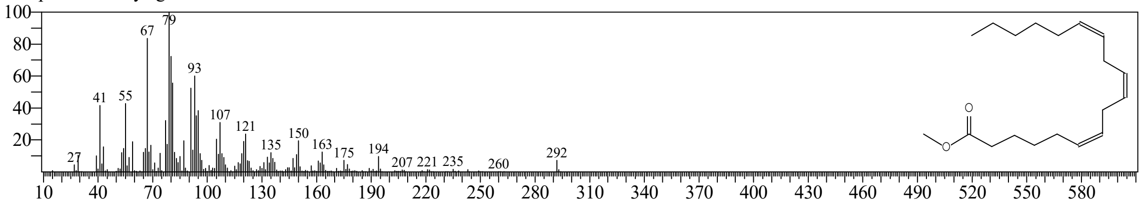

<< Target >>
Line#:23   R.Time:38.850(Scan#:3463) MassPeaks:360
RawMode:Averaged 38.842-38.858(3462-3464) BasePeak:79.10(5893)
BG Mode:Calc. from Peak Group 1 - Event 1

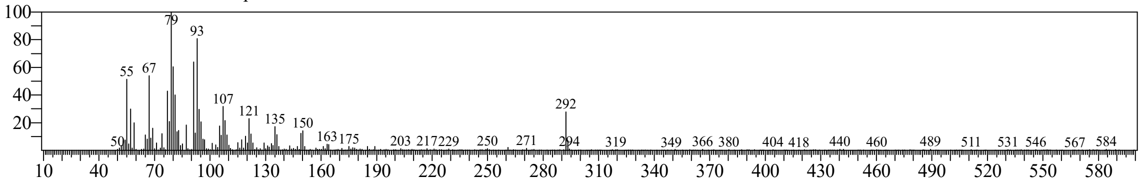

Hit#:1 Entry:135726 Library:NIST17-1.lib
SI:91 Formula:C19H32O2 CAS:0-00-0 MolWeight:292 RetIndex:2101
CompName:Methyl 9.cis.,11.trans.t,13.trans.-octadecatrienoate

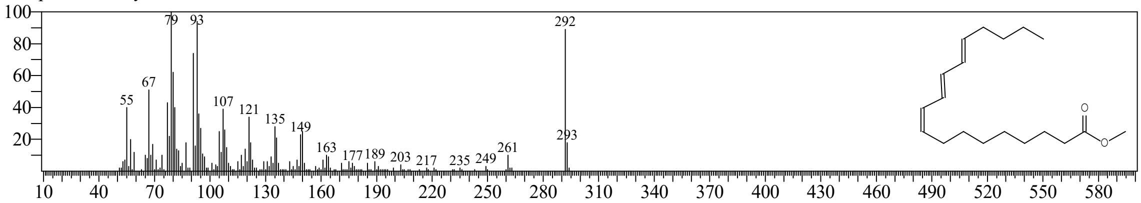

Hit#:2 Entry:124230 Library:NIST14.lib
SI:91 Formula:C19H32O2 CAS:0-00-0 MolWeight:292 RetIndex:2101
CompName:Methyl 9.cis.,11.trans.t,13.trans.-octadecatrienoate

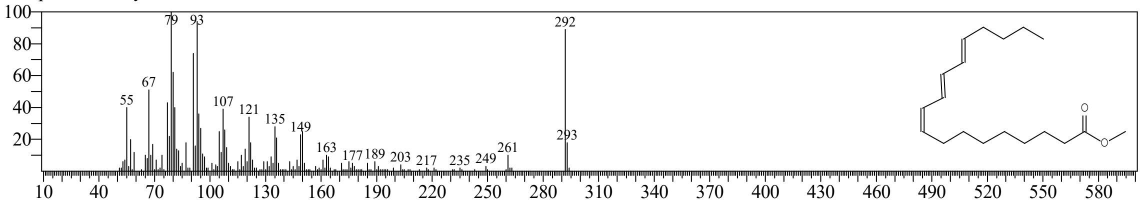

Hit#:3 Entry:124226 Library:NIST14.lib
SI:90 Formula:C19H32O2 CAS:0-00-0 MolWeight:292 RetIndex:2101
CompName:Methyl 6-cis,9-cis,11-trans-octadecatrienoate

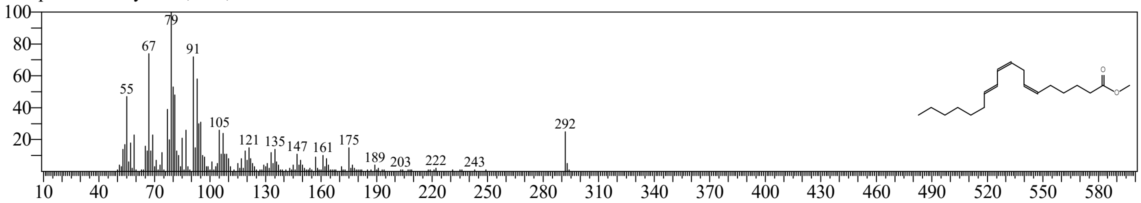

Hit#:4 Entry:135722 Library:NIST17-1.lib
SI:90 Formula:C19H32O2 CAS:0-00-0 MolWeight:292 RetIndex:2101
CompName:Methyl 6-cis,9-cis,11-trans-octadecatrienoate

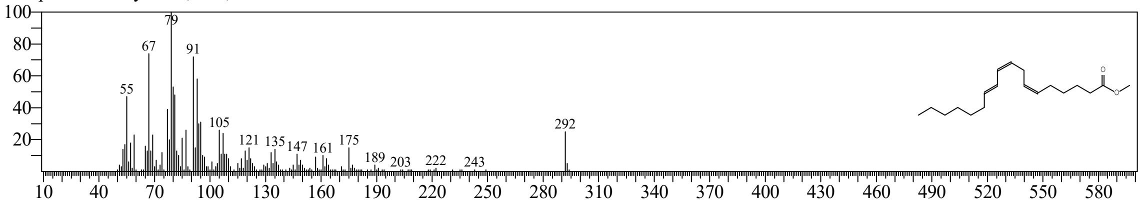

Hit#:5 Entry:135721 Library:NIST17-1.lib
SI:89 Formula:C19H32O2 CAS:16326-32-2 MolWeight:292 RetIndex:2101
CompName:Methyl .gamma.-linolenate

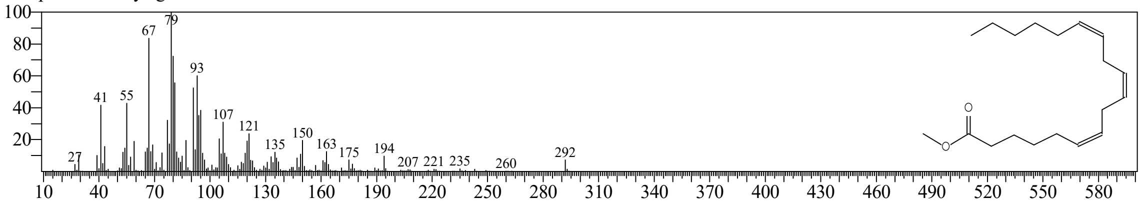

<< Target >>
Line#:24 R.Time:38.992(Scan#:3480) MassPeaks:329
RawMode:Averaged 38.983-39.000(3479-3481) BasePeak:79.10(5676)
BG Mode:Calc. from Peak Group 1 - Event 1

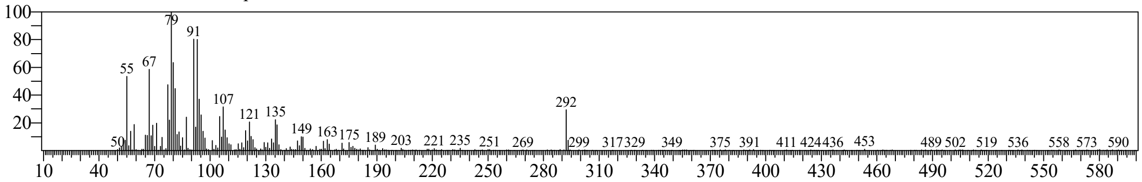

Hit#:1 Entry:135722 Library:NIST17-1.lib
SI:92 Formula:C19H32O2 CAS:0-00-0 MolWeight:292 RetIndex:2101
CompName:Methyl 6-cis,9-cis,11-trans-octadecatrienoate

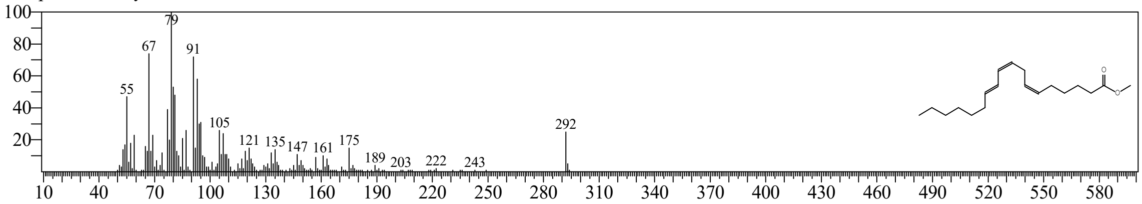

Hit#:2 Entry:124226 Library:NIST14.lib
SI:92 Formula:C19H32O2 CAS:0-00-0 MolWeight:292 RetIndex:2101
CompName:Methyl 6-cis,9-cis,11-trans-octadecatrienoate

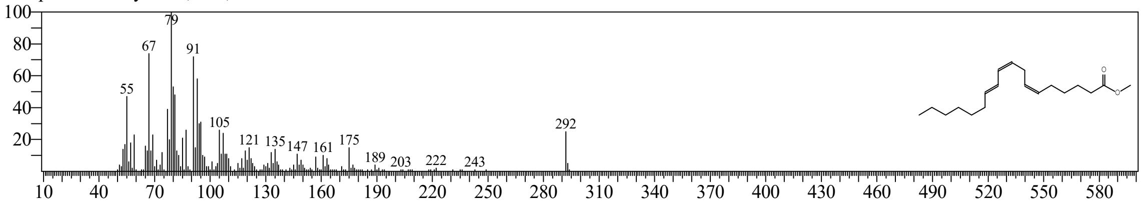

Hit#:3 Entry:124230 Library:NIST14.lib
SI:92 Formula:C19H32O2 CAS:0-00-0 MolWeight:292 RetIndex:2101
CompName:Methyl 9.cis.,11.trans.t,13.trans.-octadecatrienoate

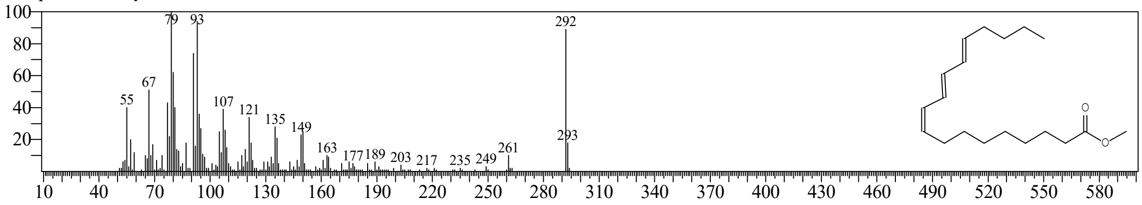

Hit#:4 Entry:135726 Library:NIST17-1.lib
SI:92 Formula:C19H32O2 CAS:0-00-0 MolWeight:292 RetIndex:2101
CompName:Methyl 9.cis.,11.trans.t,13.trans.-octadecatrienoate

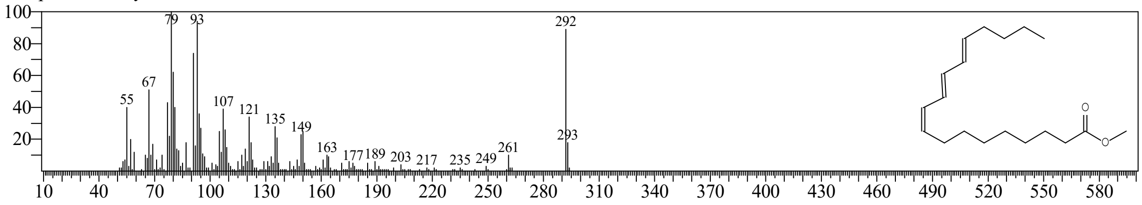

Hit#:5 Entry:124225 Library:NIST14.lib
SI:91 Formula:C19H32O2 CAS:0-00-0 MolWeight:292 RetIndex:2101
CompName:.gamma.-Linolenic acid, methyl ester

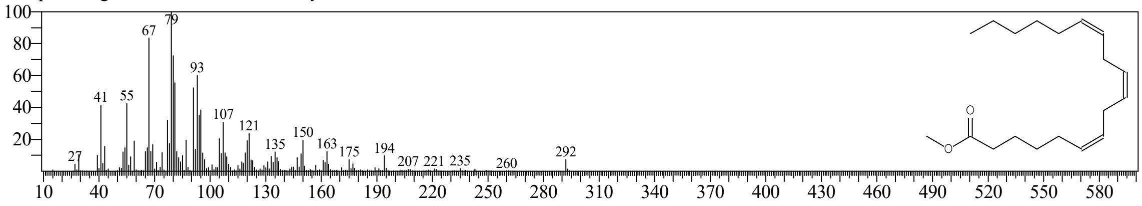

Supplement: Supplementary file 1 [file molecules-31-02891-s001.zip › 56d-CK-1.pdf]
