# Supplementary material for: Selective Inhibition of Lipid Oxidation by a Quercetagetin-Rich Extract from Tagetes erecta: A Chemometric Approach Using OPLS-DA and Selectivity Index Analysis
Source: Molecules. 2026 Aug 19;31(16):2891. doi: 10.3390/molecules31162891 (PMC13515886; doi:10.3390/molecules31162891)

K-2-56d

<<Column>>
Name : HP-88
Thickness : 0.20um
Length : 100.0m
Inside Diameter : 0.25mm

Method

===== Analytical Line 1 =====

Column Oven Temp.	:120.0 °C	
Injection Temp.	:250.00 °C	
Injection Mode	:Split	
Column Flow	:1.04 mL/min	
Split Ratio	:20.0	
Oven Temp. Program		
Rate	Temperature(°C)	Hold Time(min)
-	120.0	1.00
10.00	175.0	10.00
5.00	210.0	5.00
5.00	230.0	15.00

[GCMS-QP2010]
IonSourceTemp :200.00 °C
Interface Temp. :220.00 °C
m/z scanning range：45～500m/z

Chromatogram K-2-56d

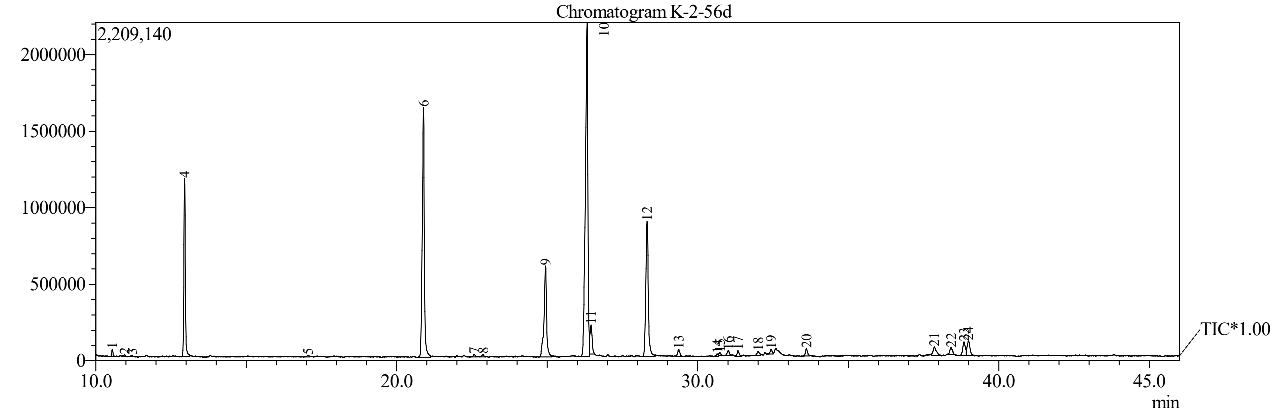

Peak Report TIC

Peak#	R.Time	Area	Area%	Height	Height%	A/H	Name
1	10.551	129161	0.35	46234	0.63	2.79	Octanoic acid, methyl ester
2	10.965	46384	0.12	12232	0.17	3.79	Octadecane, 1,1-dimethoxy-
3	11.209	31390	0.08	9147	0.13	3.43	Nonanoic acid, methyl ester
4	12.952	3790570	10.19	1164540	15.96	3.25	Undecanoic acid, methyl ester
5	17.051	49618	0.13	10944	0.15	4.53	Methyl tetradecanoate
6	20.889	7756615	20.85	1633012	22.39	4.75	Hexadecanoic acid, methyl ester
7	22.571	84524	0.23	16995	0.23	4.97	Octanedioic acid, dimethyl ester
8	22.866	71000	0.19	16663	0.23	4.26	Heptadecanoic acid, methyl ester
9	24.943	3512030	9.44	594290	8.15	5.91	Methyl stearate
10	26.323	12754118	34.29	2180657	29.89	5.85	11-Octadecenoic acid, methyl ester
11	26.455	856197	2.30	190441	2.61	4.50	trans-13-Octadecenoic acid, methyl ester
12	28.317	5048769	13.57	884484	12.13	5.71	9,12-Octadecadienoic acid, methyl ester
13	29.363	221225	0.59	44626	0.61	4.96	Eicosanoic acid, methyl ester
14	30.655	98264	0.26	16391	0.22	5.99	1,8,11,14-Heptadecatetraene, (Z,Z,Z)-

Peak#	R.Time	Area	Area%	Height	Height%	A/H	Name
15	30.739	63308	0.17	17520	0.24	3.61	cis-Methyl 11-eicosenoate
16	31.015	177420	0.48	32530	0.45	5.45	9,11-Octadecadienoic acid, methyl ester, (E,E)
17	31.334	171706	0.46	36781	0.50	4.67	9,11-Octadecadienoic acid, methyl ester, (E,E)
18	32.000	129057	0.35	25038	0.34	5.15	Methyl 9-cis,11-trans-octadecadienoate
19	32.435	132104	0.36	30682	0.42	4.31	Succinic acid, dodec-2-enyl 3-methylbutyl este
20	33.609	230635	0.62	49044	0.67	4.70	Docosanoic acid, methyl ester
21	37.854	458680	1.23	56052	0.77	8.18	Methyl 9.cis.,11.trans.t,13.trans.-octadecatrien
22	38.416	277372	0.75	45517	0.62	6.09	Methyl 9.cis.,11.trans.t,13.trans.-octadecatrien
23	38.844	517575	1.39	86837	1.19	5.96	Methyl 9.cis.,11.trans.t,13.trans.-octadecatrien
24	38.987	590227	1.59	94035	1.29	6.28	Methyl 9.cis.,11.trans.t,13.trans.-octadecatrien
		37197949	100.00	7294692	100.00		

Library

<< Target >>
Line#:1 R.Time:10.550(Scan#:67) MassPeaks:308
RawMode:Averaged 10.542-10.558(66-68) BasePeak:74.00(13532)
BG Mode:Calc. from Peak Group 1 - Event 1

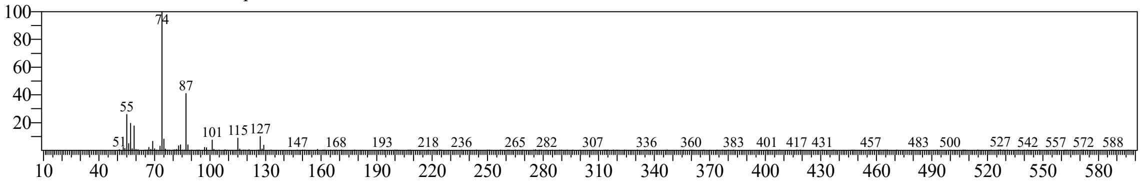

Hit#:1 Entry:11044 Library:NIST14s.lib
SI:97 Formula:C9H18O2 CAS:111-11-5 MolWeight:158 RetIndex:1083
CompName:Octanoic acid, methyl ester $$ Caprylic acid methyl ester $$ Methyl caprylate $$ Methyl n-octanoate $$ Methyl octanoate $$ Uniphat A20 $$ n

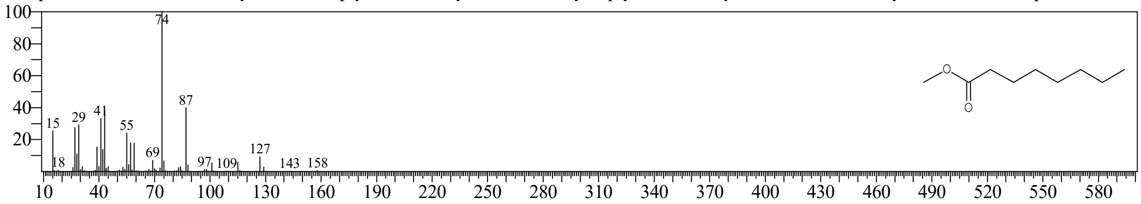

Hit#:2 Entry:12802 Library:NIST17s.lib
SI:97 Formula:C9H18O2 CAS:111-11-5 MolWeight:158 RetIndex:1083
CompName:Octanoic acid, methyl ester $$ Caprylic acid methyl ester $$ Methyl caprylate $$ Methyl n-octanoate $$ Methyl octanoate $$ Uniphat A20 $$ n

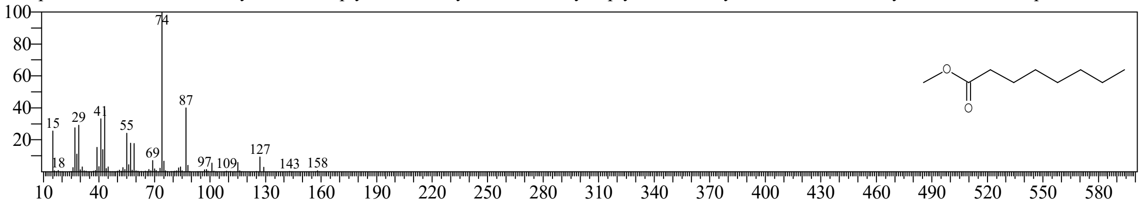

Hit#:3 Entry:12803 Library:NIST17s.lib
SI:96 Formula:C9H18O2 CAS:111-11-5 MolWeight:158 RetIndex:1083
CompName:Octanoic acid, methyl ester $$ Caprylic acid methyl ester $$ Methyl caprylate $$ Methyl n-octanoate $$ Methyl octanoate $$ Uniphat A20 $$ n

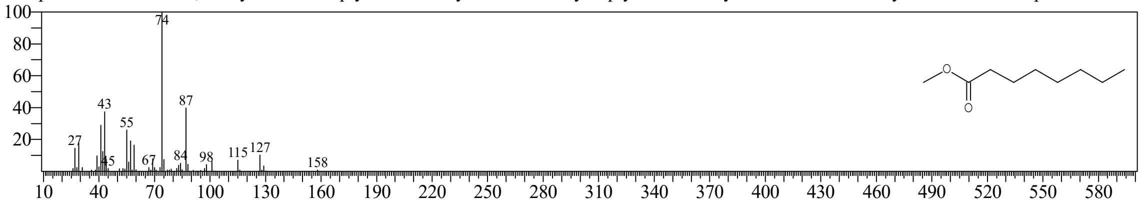

Hit#:4 Entry:11045 Library:NIST14s.lib
SI:96 Formula:C9H18O2 CAS:111-11-5 MolWeight:158 RetIndex:1083
CompName:Octanoic acid, methyl ester $$ Caprylic acid methyl ester $$ Methyl caprylate $$ Methyl n-octanoate $$ Methyl octanoate $$ Uniphat A20 $$ n

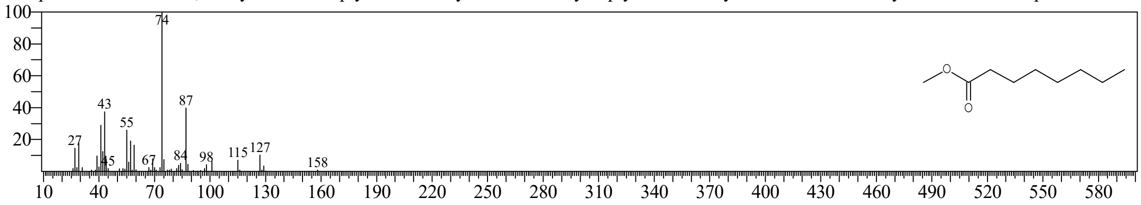

<< Target >>
Line#:1 R.Time:10.550(Scan#:67) MassPeaks:308
RawMode:Averaged 10.542-10.558(66-68) BasePeak:74.00(13532)
BG Mode:Calc. from Peak Group 1 - Event 1

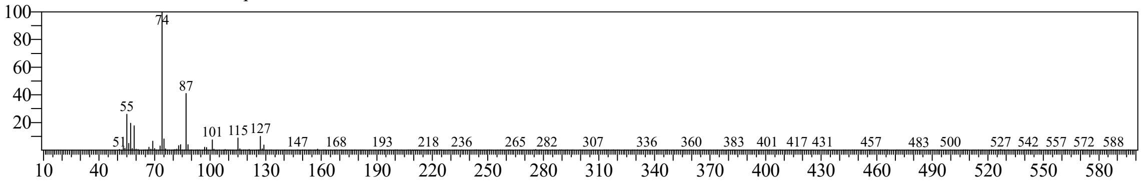

Hit#:5 Entry:12801 Library:NIST17s.lib
SI:95 Formula:C9H18O2 CAS:111-11-5 MolWeight:158 RetIndex:1083
CompName:Octanoic acid, methyl ester $$ Caprylic acid methyl ester $$ Methyl caprylate $$ Methyl n-octanoate $$ Methyl octanoate $$ Uniphat A20 $$ n

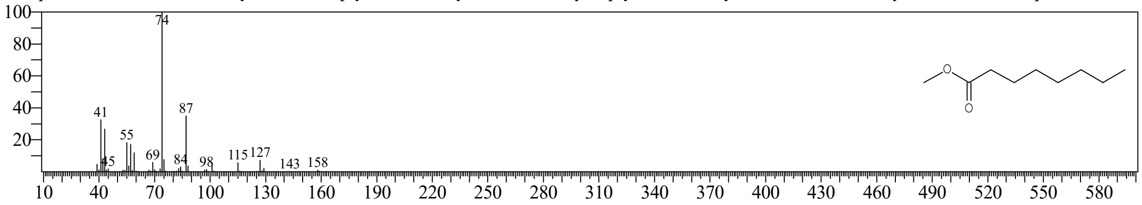

<< Target >>
Line#:2 R.Time:10.967(Scan#:117) MassPeaks:281
RawMode:Averaged 10.958-10.975(116-118) BasePeak:75.05(5187)
BG Mode:Calc. from Peak Group 1 - Event 1

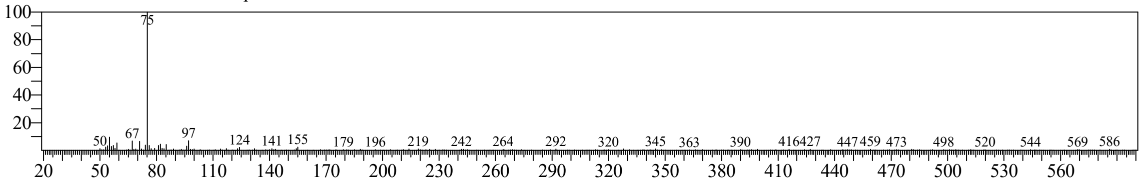

Hit#:1 Entry:158393 Library:NIST17-1.lib
SI:84 Formula:C20H42O2 CAS:14620-55-4 MolWeight:314 RetIndex:2097
CompName:Octadecane, 1,1-dimethoxy- $$ Stearaldehyde, dimethyl acetal $$ 1,1-Dimethoxyoctadecane # $$

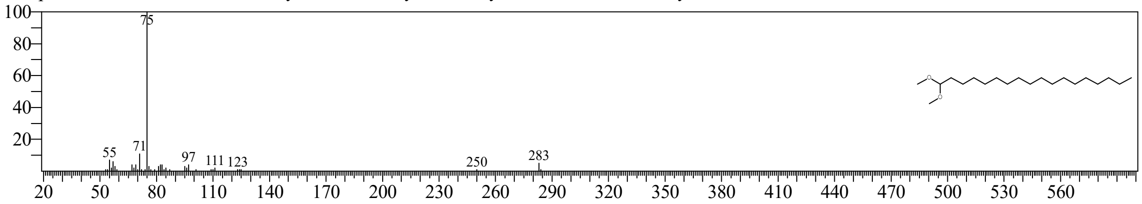

Hit#:2 Entry:144211 Library:NIST14.lib
SI:84 Formula:C20H42O2 CAS:14620-55-4 MolWeight:314 RetIndex:2097
CompName:Octadecane, 1,1-dimethoxy- $$ Stearaldehyde, dimethyl acetal $$ 1,1-Dimethoxyoctadecane # $$

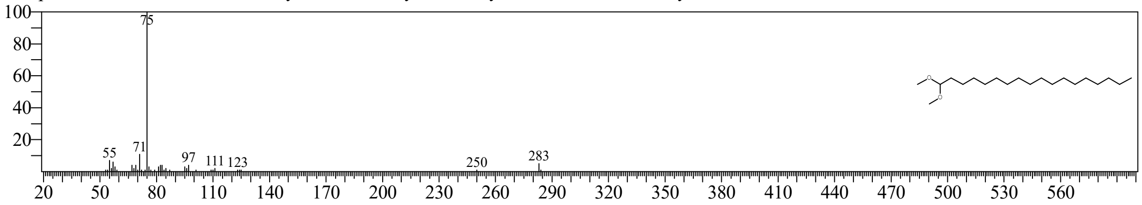

Hit#:3 Entry:70845 Library:NIST14.lib
SI:83 Formula:C14H30O2 CAS:14620-52-1 MolWeight:230 RetIndex:1501
CompName:Dodecane, 1,1-dimethoxy- $$ Lauraldehyde, dimethyl acetal $$ Dodecanal dimethyl acetal $$ Laural dimethyl acetal $$ n-Dodecanal dimethyl a

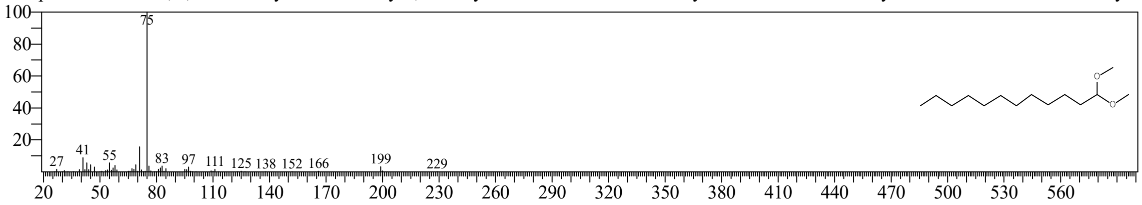

Hit#:4 Entry:75842 Library:NIST17-1.lib
SI:83 Formula:C14H30O2 CAS:14620-52-1 MolWeight:230 RetIndex:1501
CompName:Dodecane, 1,1-dimethoxy- $$ Lauraldehyde, dimethyl acetal $$ Dodecanal dimethyl acetal $$ Laural dimethyl acetal $$ n-Dodecanal dimethyl a

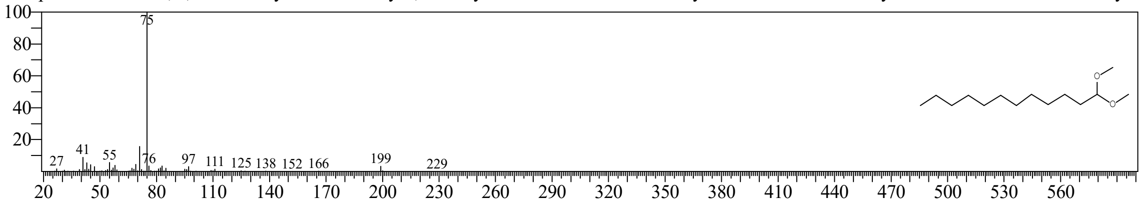

Hit#:5 Entry:30650 Library:NIST17-1.lib
SI:83 Formula:C9H18O3 CAS:60090-77-9 MolWeight:174 RetIndex:1192
CompName:Heptanal, 7,7-dimethoxy- $$ 7,7-Dimethoxyheptanal # $$

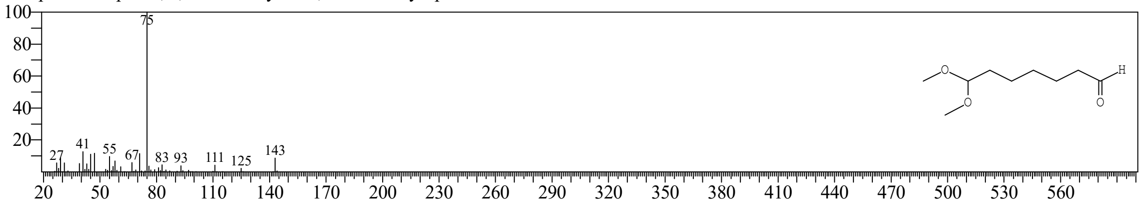

<< Target >>
Line#:3 R.Time:11.208(Scan#:146) MassPeaks:278
RawMode:Averaged 11.200-11.217(145-147) BasePeak:74.05(2309)
BG Mode:Calc. from Peak Group 1 - Event 1

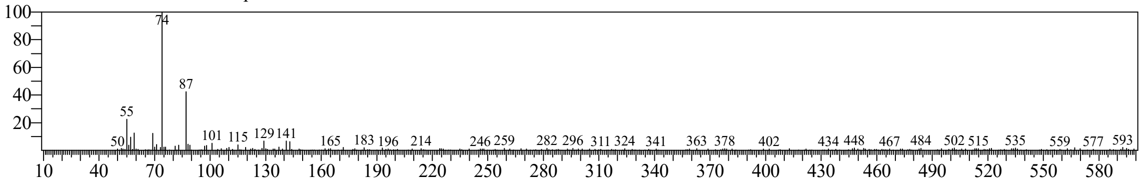

Hit#:1 Entry:13673 Library:NIST14s.lib
SI:87 Formula:C10H20O2 CAS:1731-84-6 MolWeight:172 RetIndex:1183
CompName:Nonanoic acid, methyl ester $$ Methyl n-nonanoate $$ Methyl nonanoate $$ Methyl nonylate $$ Methyl pelargonate $$ Pelargonic acid methyl

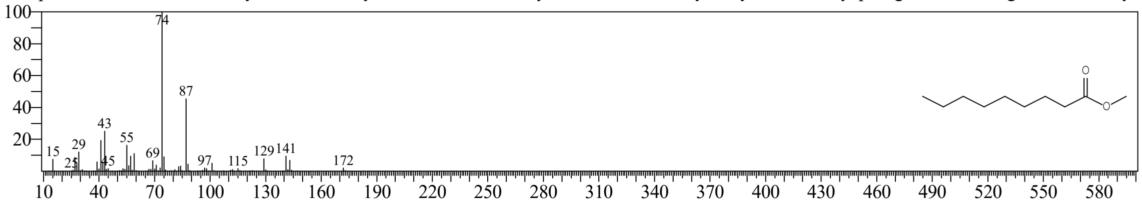

Hit#:2 Entry:13672 Library:NIST14s.lib
SI:87 Formula:C10H20O2 CAS:1731-84-6 MolWeight:172 RetIndex:1183
CompName:Nonanoic acid, methyl ester $$ Methyl n-nonanoate $$ Methyl nonanoate $$ Methyl nonylate $$ Methyl pelargonate $$ Pelargonic acid methyl

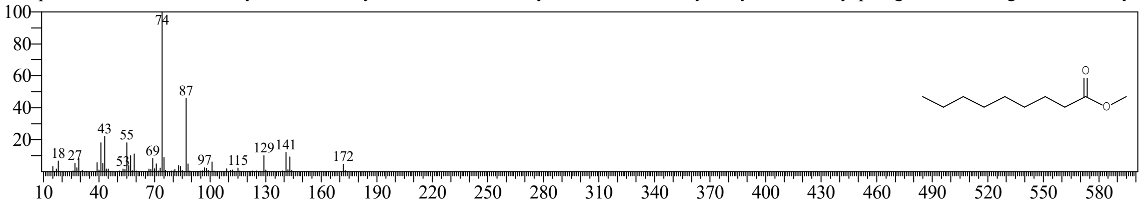

Hit#:3 Entry:15894 Library:NIST17s.lib
SI:87 Formula:C10H20O2 CAS:1731-84-6 MolWeight:172 RetIndex:1183
CompName:Nonanoic acid, methyl ester $$ Methyl n-nonanoate $$ Methyl nonanoate $$ Methyl nonylate $$ Methyl pelargonate $$ Pelargonic acid methyl

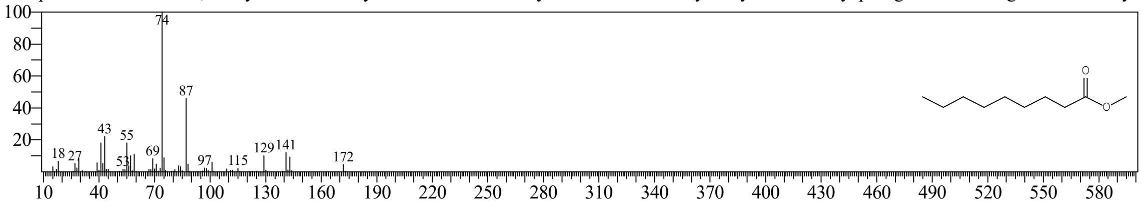

Hit#:4 Entry:15895 Library:NIST17s.lib
SI:87 Formula:C10H20O2 CAS:1731-84-6 MolWeight:172 RetIndex:1183
CompName:Nonanoic acid, methyl ester $$ Methyl n-nonanoate $$ Methyl nonanoate $$ Methyl nonylate $$ Methyl pelargonate $$ Pelargonic acid methyl

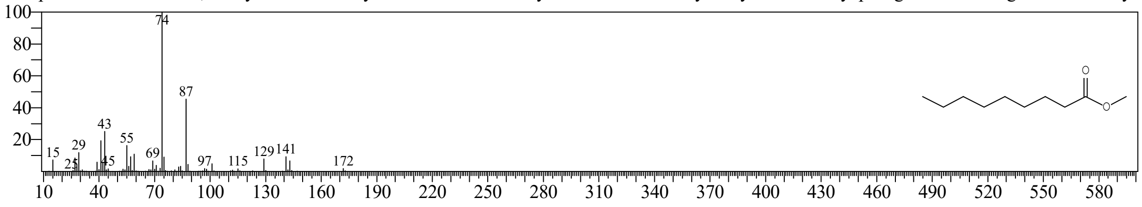

Hit#:5 Entry:15896 Library:NIST17s.lib
SI:86 Formula:C10H20O2 CAS:1731-84-6 MolWeight:172 RetIndex:1183
CompName:Nonanoic acid, methyl ester $$ Methyl n-nonanoate $$ Methyl nonanoate $$ Methyl nonylate $$ Methyl pelargonate $$ Pelargonic acid methyl

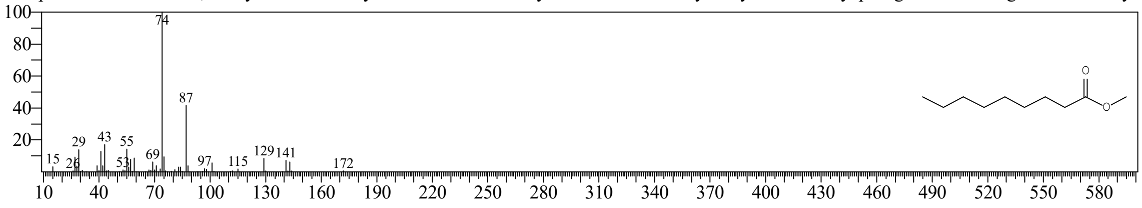

<< Target >>
Line#:4 R.Time:12.950(Scan#:355) MassPeaks:306
RawMode:Averaged 12.942-12.958(354-356) BasePeak:74.05(336535)
BG Mode:Calc. from Peak Group 1 - Event 1

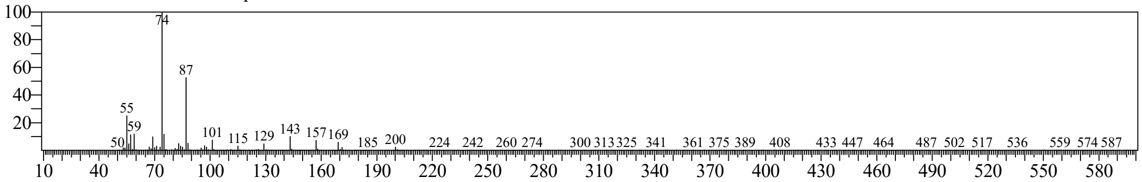

Hit#:1 Entry:49598 Library:NIST17-1.lib
SI:95 Formula:C12H24O2 CAS:1731-86-8 MolWeight:200 RetIndex:1381
CompName:Undecanoic acid, methyl ester $$ Methyl undecanoate $$ Methyl undecylenate $$ n-Undecanoic acid methyl ester $$ Methyl ester of undecanoi

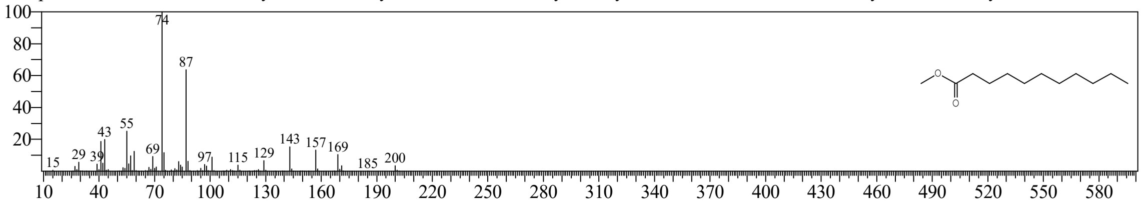

Hit#:2 Entry:46966 Library:NIST14.lib
SI:95 Formula:C12H24O2 CAS:1731-86-8 MolWeight:200 RetIndex:1381
CompName:Undecanoic acid, methyl ester $$ Methyl undecanoate $$ Methyl undecylenate $$ n-Undecanoic acid methyl ester $$ Methyl ester of undecanoi

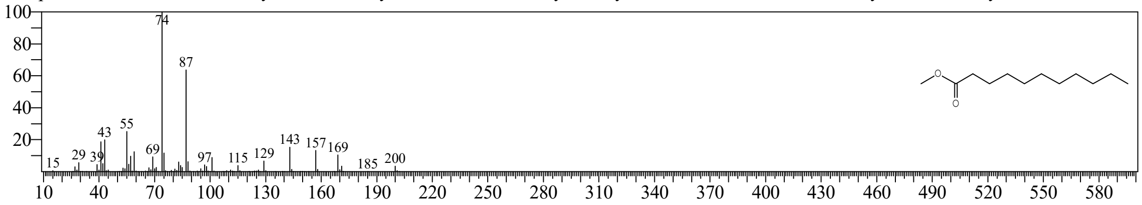

Hit#:3 Entry:21618 Library:NIST17s.lib
SI:95 Formula:C12H24O2 CAS:1731-86-8 MolWeight:200 RetIndex:1381
CompName:Undecanoic acid, methyl ester $$ Methyl undecanoate $$ Methyl undecylenate $$ n-Undecanoic acid methyl ester $$ Methyl ester of undecanoi

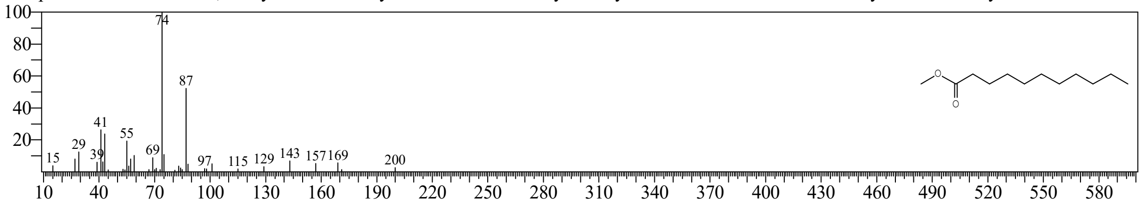

Hit#:4 Entry:21619 Library:NIST17s.lib
SI:95 Formula:C12H24O2 CAS:1731-86-8 MolWeight:200 RetIndex:1381
CompName:Undecanoic acid, methyl ester $$ Methyl undecanoate $$ Methyl undecylenate $$ n-Undecanoic acid methyl ester $$ Methyl ester of undecanoi

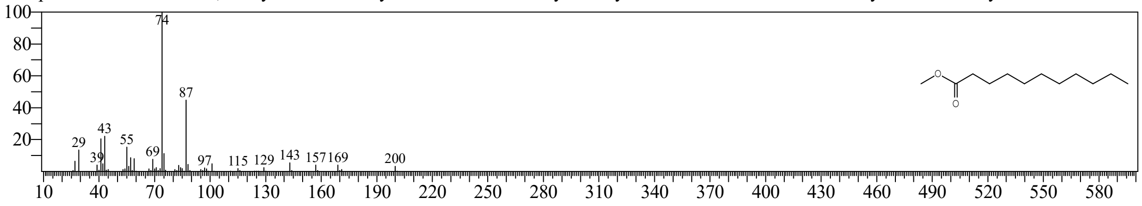

Hit#:5 Entry:18487 Library:NIST14s.lib
SI:95 Formula:C12H24O2 CAS:1731-86-8 MolWeight:200 RetIndex:1381
CompName:Undecanoic acid, methyl ester $$ Methyl undecanoate $$ Methyl undecylenate $$ n-Undecanoic acid methyl ester $$ Methyl ester of undecanoi

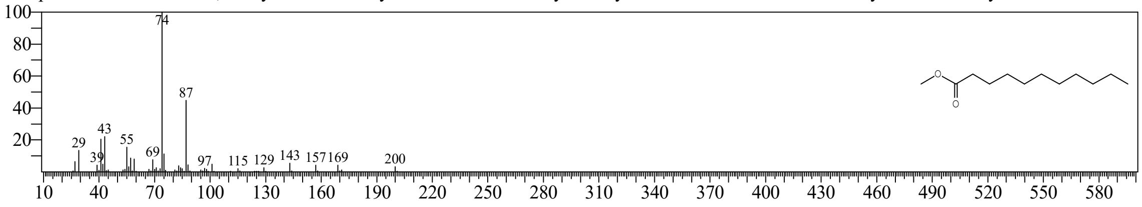

<< Target >>
Line#:5 R.Time:17.050(Scan#:847) MassPeaks:264
RawMode:Averaged 17.042-17.058(846-848) BasePeak:74.00(2624)
BG Mode:Calc. from Peak Group 1 - Event 1

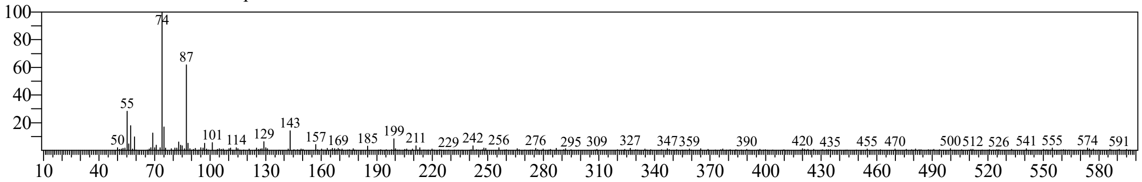

Hit#:1 Entry:86786 Library:NIST17-1.lib
SI:92 Formula:C15H30O2 CAS:124-10-7 MolWeight:242 RetIndex:1680
CompName:Methyl tetradecanoate $$ Tetradecanoic acid, methyl ester $$ Myristic acid, methyl ester $$ Metholeneat 2495 $$ Methyl myristate $$ Methyl r

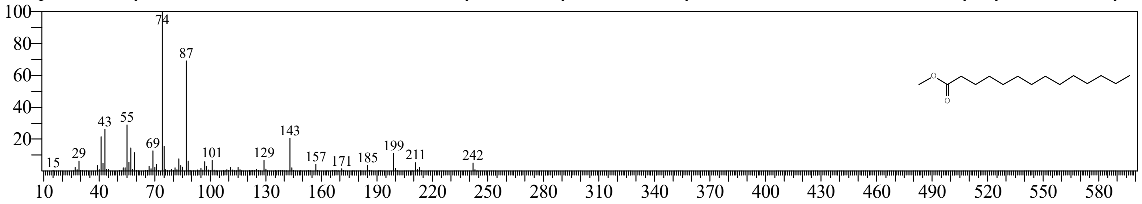

Hit#:2 Entry:80645 Library:NIST14.lib
SI:92 Formula:C15H30O2 CAS:124-10-7 MolWeight:242 RetIndex:1680
CompName:Methyl tetradecanoate $$ Tetradecanoic acid, methyl ester $$ Myristic acid, methyl ester $$ Metholeneat 2495 $$ Methyl myristate $$ Methyl r

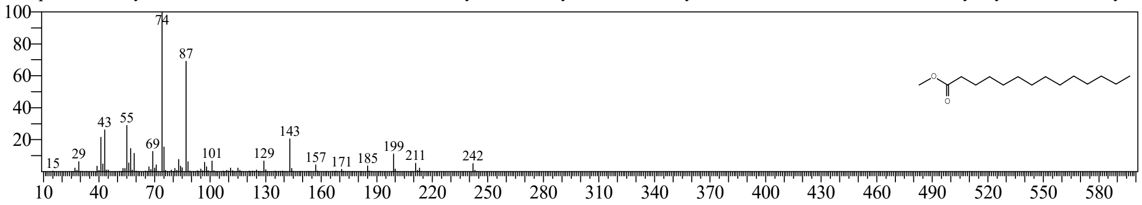

Hit#:3 Entry:23848 Library:NIST14s.lib
SI:91 Formula:C15H30O2 CAS:124-10-7 MolWeight:242 RetIndex:1680
CompName:Methyl tetradecanoate $$ Tetradecanoic acid, methyl ester $$ Myristic acid, methyl ester $$ Metholeneat 2495 $$ Methyl myristate $$ Methyl r

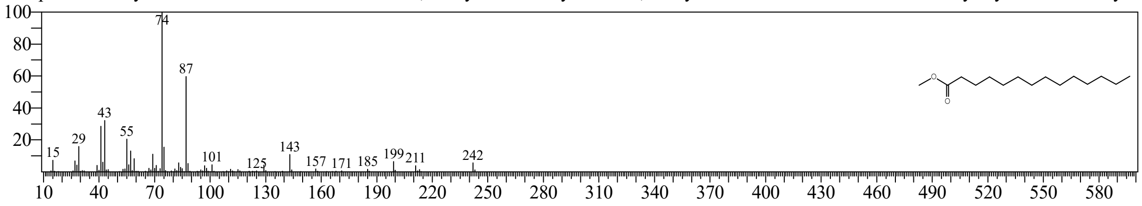

Hit#:4 Entry:27874 Library:NIST17s.lib
SI:91 Formula:C15H30O2 CAS:124-10-7 MolWeight:242 RetIndex:1680
CompName:Methyl tetradecanoate $$ Tetradecanoic acid, methyl ester $$ Myristic acid, methyl ester $$ Metholeneat 2495 $$ Methyl myristate $$ Methyl r

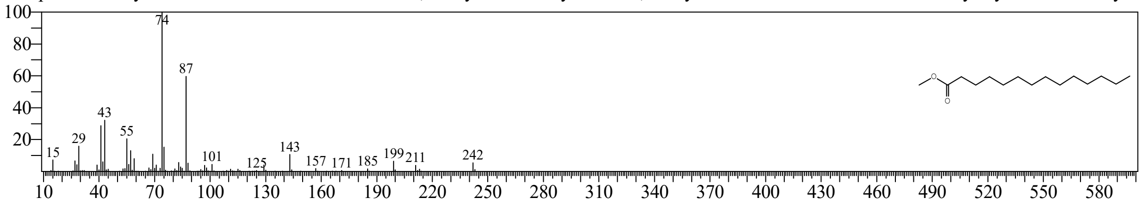

Hit#:5 Entry:23847 Library:NIST14s.lib
SI:91 Formula:C15H30O2 CAS:124-10-7 MolWeight:242 RetIndex:1680
CompName:Methyl tetradecanoate $$ Tetradecanoic acid, methyl ester $$ Myristic acid, methyl ester $$ Metholeneat 2495 $$ Methyl myristate $$ Methyl r

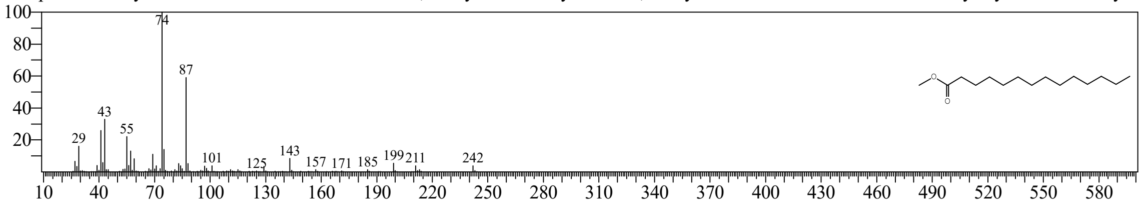

<< Target >>
Line#:6 R.Time:20.892(Scan#:1308) MassPeaks:355
RawMode:Averaged 20.883-20.900(1307-1309) BasePeak:74.05(379100)
BG Mode:Calc. from Peak Group 1 - Event 1

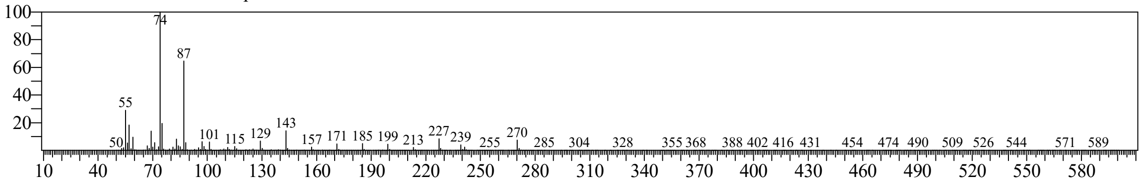

Hit#:1 Entry:104648 Library:NIST14.lib
SI:97 Formula:C17H34O2 CAS:112-39-0 MolWeight:270 RetIndex:1878
CompName:Hexadecanoic acid, methyl ester $$ Palmitic acid, methyl ester $$ n-Hexadecanoic acid methyl ester $$ Metholene 2216 $$ Methyl hexadecano

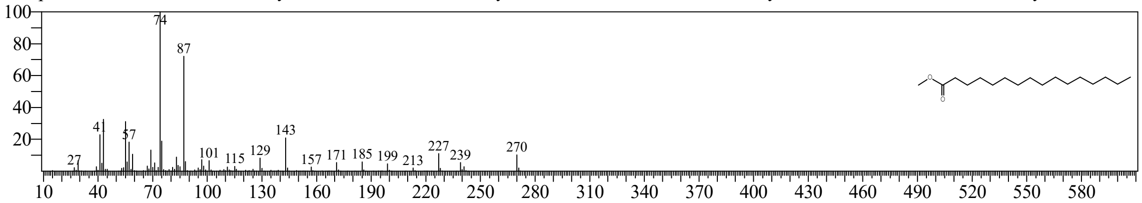

Hit#:2 Entry:113663 Library:NIST17-1.lib
SI:97 Formula:C17H34O2 CAS:112-39-0 MolWeight:270 RetIndex:1878
CompName:Hexadecanoic acid, methyl ester $$ Palmitic acid, methyl ester $$ n-Hexadecanoic acid methyl ester $$ Metholene 2216 $$ Methyl hexadecano

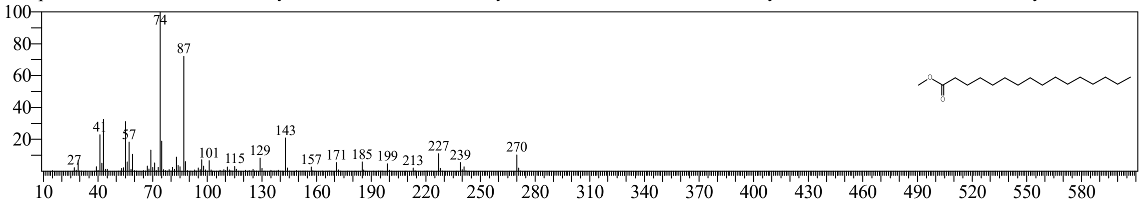

Hit#:3 Entry:26272 Library:NIST14s.lib
SI:97 Formula:C17H34O2 CAS:112-39-0 MolWeight:270 RetIndex:1878
CompName:Hexadecanoic acid, methyl ester $$ Palmitic acid, methyl ester $$ n-Hexadecanoic acid methyl ester $$ Metholene 2216 $$ Methyl hexadecano

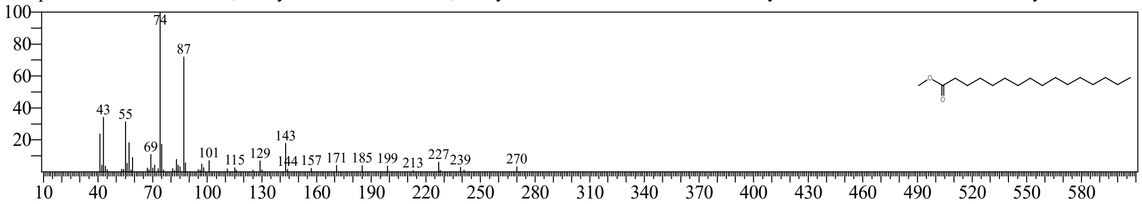

Hit#:4 Entry:30647 Library:NIST17s.lib
SI:97 Formula:C17H34O2 CAS:112-39-0 MolWeight:270 RetIndex:1878
CompName:Hexadecanoic acid, methyl ester $$ Palmitic acid, methyl ester $$ n-Hexadecanoic acid methyl ester $$ Metholene 2216 $$ Methyl hexadecano

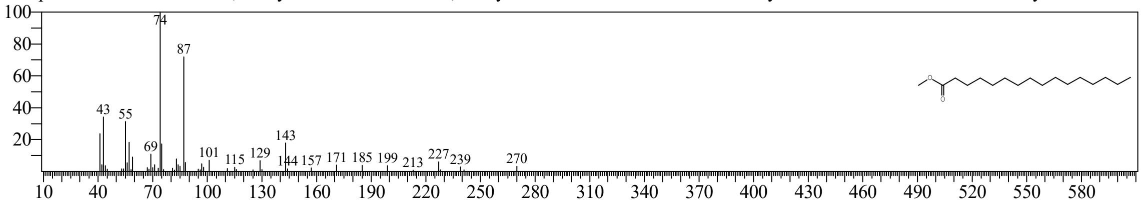

Hit#:5 Entry:26271 Library:NIST14s.lib
SI:96 Formula:C17H34O2 CAS:112-39-0 MolWeight:270 RetIndex:1878
CompName:Hexadecanoic acid, methyl ester $$ Palmitic acid, methyl ester $$ n-Hexadecanoic acid methyl ester $$ Metholene 2216 $$ Methyl hexadecano

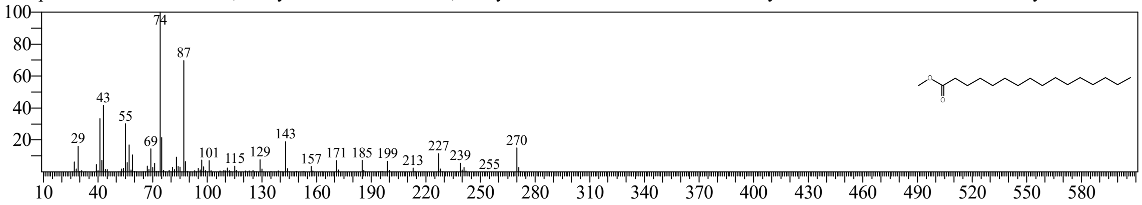

<< Target >>
Line#:7 R.Time:22.567(Scan#:1509) MassPeaks:358
RawMode:Averaged 22.558-22.575(1508-1510) BasePeak:55.10(1350)
BG Mode:Calc. from Peak Group 1 - Event 1

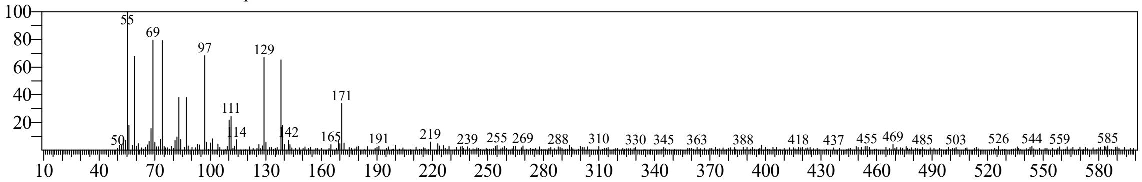

Hit#:1 Entry:18672 Library:NIST14s.lib
SI:89 Formula:C10H18O4 CAS:1732-09-8 MolWeight:202 RetIndex:1350
CompName:Octanedioic acid, dimethyl ester $$ Suberic acid, dimethyl ester $$ Dimethyl octanedioate $$ Dimethyl suberate $$ Dimethyl ester of octanedio

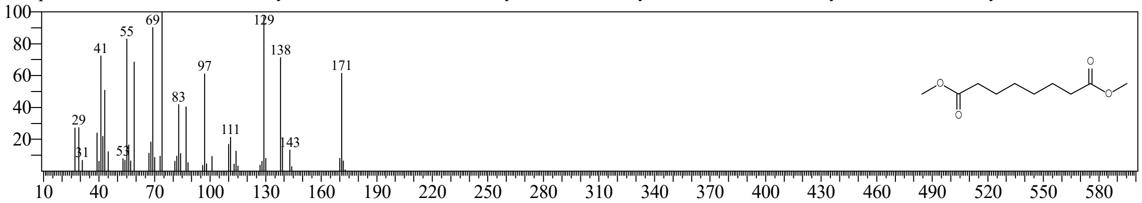

Hit#:2 Entry:50683 Library:NIST17-1.lib
SI:88 Formula:C10H18O4 CAS:1732-09-8 MolWeight:202 RetIndex:1350
CompName:Octanedioic acid, dimethyl ester $$ Suberic acid, dimethyl ester $$ Dimethyl octanedioate $$ Dimethyl suberate $$ Dimethyl ester of octanedio

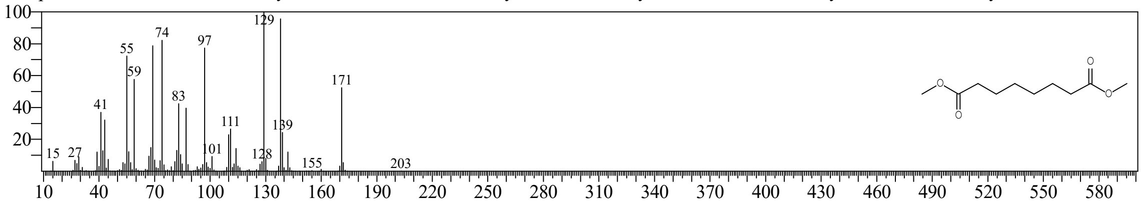

Hit#:3 Entry:47957 Library:NIST14.lib
SI:88 Formula:C10H18O4 CAS:1732-09-8 MolWeight:202 RetIndex:1350
CompName:Octanedioic acid, dimethyl ester $$ Suberic acid, dimethyl ester $$ Dimethyl octanedioate $$ Dimethyl suberate $$ Dimethyl ester of octanedio

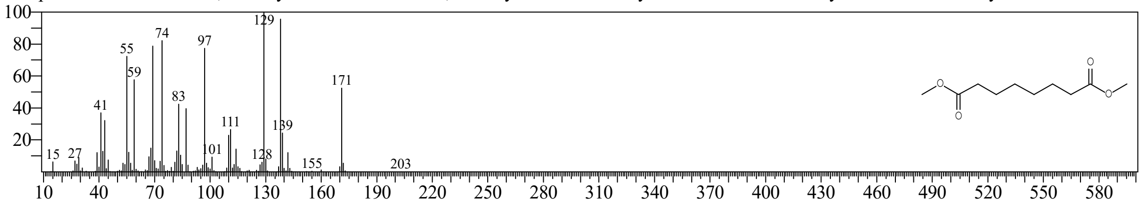

Hit#:4 Entry:18670 Library:NIST14s.lib
SI:88 Formula:C10H18O4 CAS:1732-09-8 MolWeight:202 RetIndex:1350
CompName:Octanedioic acid, dimethyl ester $$ Suberic acid, dimethyl ester $$ Dimethyl octanedioate $$ Dimethyl suberate $$ Dimethyl ester of octanedio

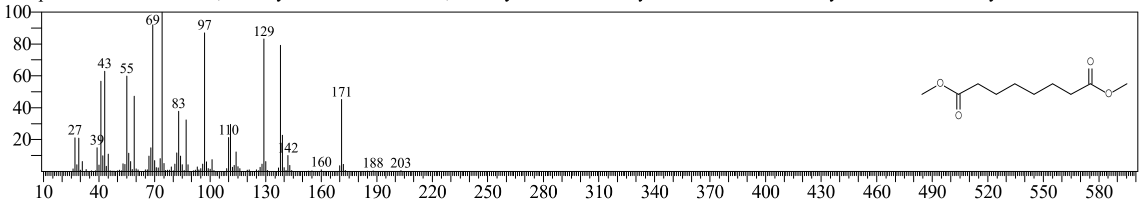

Hit#:5 Entry:21831 Library:NIST17s.lib
SI:88 Formula:C10H18O4 CAS:1732-09-8 MolWeight:202 RetIndex:1350
CompName:Octanedioic acid, dimethyl ester $$ Suberic acid, dimethyl ester $$ Dimethyl octanedioate $$ Dimethyl suberate $$ Dimethyl ester of octanedio

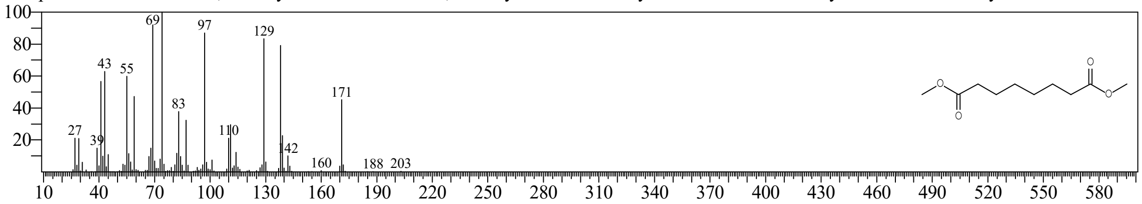

<< Target >>
Line#:8 R.Time:22.867(Scan#:1545) MassPeaks:323
RawMode:Averaged 22.858-22.875(1544-1546) BasePeak:74.05(3083)
BG Mode:Calc. from Peak Group 1 - Event 1

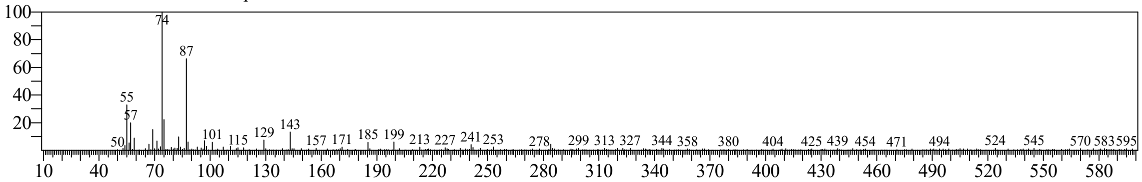

Hit#:1 Entry:31784 Library:NIST17s.lib
SI:91 Formula:C18H36O2 CAS:1731-92-6 MolWeight:284 RetIndex:1978
CompName:Heptadecanoic acid, methyl ester $$ Margaric acid methyl ester $$ Methyl heptadecanoate $$ Methyl margarate $$ n-Heptadecanoic acid methy

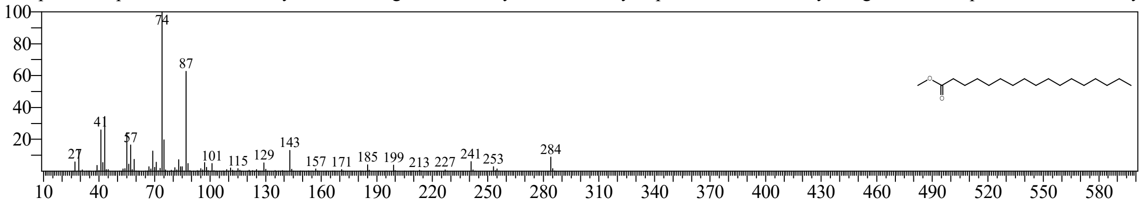

Hit#:2 Entry:27275 Library:NIST14s.lib
SI:91 Formula:C18H36O2 CAS:1731-92-6 MolWeight:284 RetIndex:1978
CompName:Heptadecanoic acid, methyl ester $$ Margaric acid methyl ester $$ Methyl heptadecanoate $$ Methyl margarate $$ n-Heptadecanoic acid methy

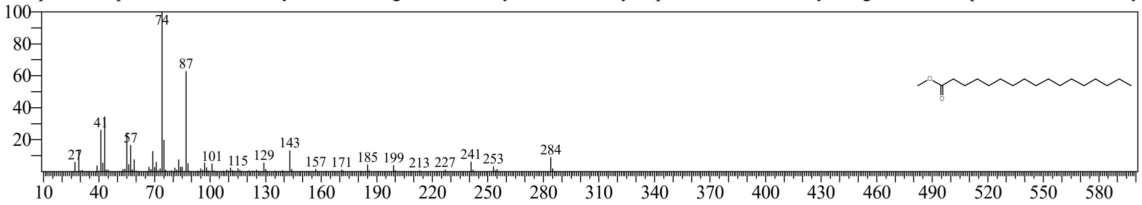

Hit#:3 Entry:27274 Library:NIST14s.lib
SI:91 Formula:C18H36O2 CAS:1731-92-6 MolWeight:284 RetIndex:1978
CompName:Heptadecanoic acid, methyl ester $$ Margaric acid methyl ester $$ Methyl heptadecanoate $$ Methyl margarate $$ n-Heptadecanoic acid methy

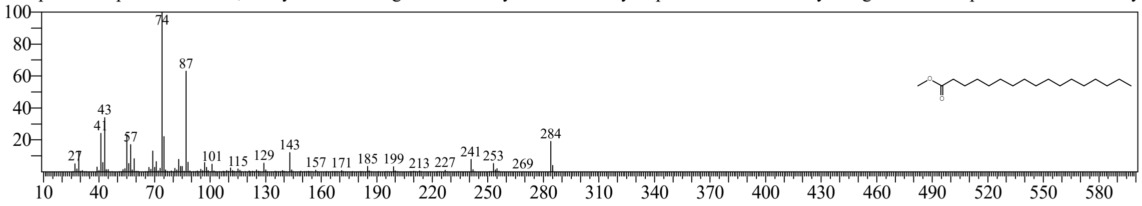

Hit#:4 Entry:31783 Library:NIST17s.lib
SI:91 Formula:C18H36O2 CAS:1731-92-6 MolWeight:284 RetIndex:1978
CompName:Heptadecanoic acid, methyl ester $$ Margaric acid methyl ester $$ Methyl heptadecanoate $$ Methyl margarate $$ n-Heptadecanoic acid methy

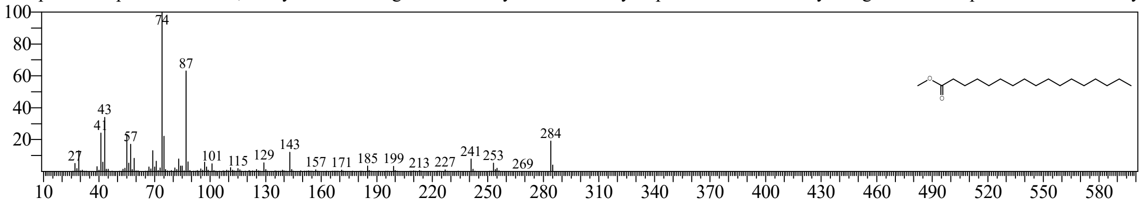

Hit#:5 Entry:117102 Library:NIST14.lib
SI:91 Formula:C18H36O2 CAS:1731-92-6 MolWeight:284 RetIndex:1978
CompName:Heptadecanoic acid, methyl ester $$ Margaric acid methyl ester $$ Methyl heptadecanoate $$ Methyl margarate $$ n-Heptadecanoic acid methy

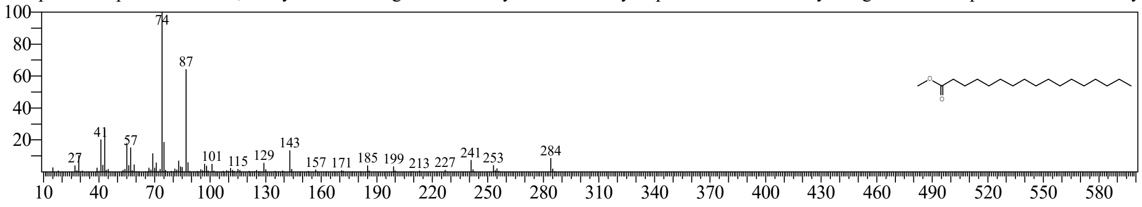

<< Target >>
Line#:9 R.Time:24.942(Scan#:1794) MassPeaks:346
RawMode:Averaged 24.933-24.950(1793-1795) BasePeak:74.05(125710)
BG Mode:Calc. from Peak Group 1 - Event 1

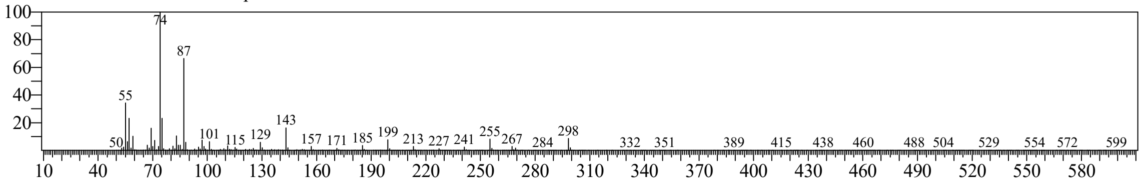

Hit#:1 Entry:28257 Library:NIST14s.lib
SI:97 Formula:C19H38O2 CAS:112-61-8 MolWeight:298 RetIndex:2077
CompName:Methyl stearate $$ Octadecanoic acid, methyl ester $$ Stearic acid, methyl ester $$ n-Octadecanoic acid, methyl ester $$ Kemester 9718 $$ Me

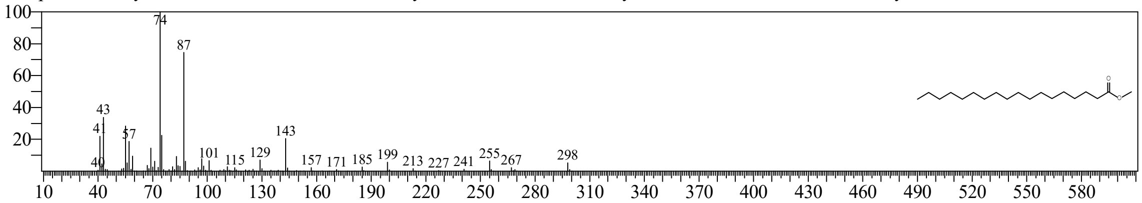

Hit#:2 Entry:32875 Library:NIST17s.lib
SI:97 Formula:C19H38O2 CAS:112-61-8 MolWeight:298 RetIndex:2077
CompName:Methyl stearate $$ Octadecanoic acid, methyl ester $$ Stearic acid, methyl ester $$ n-Octadecanoic acid, methyl ester $$ Kemester 9718 $$ Me

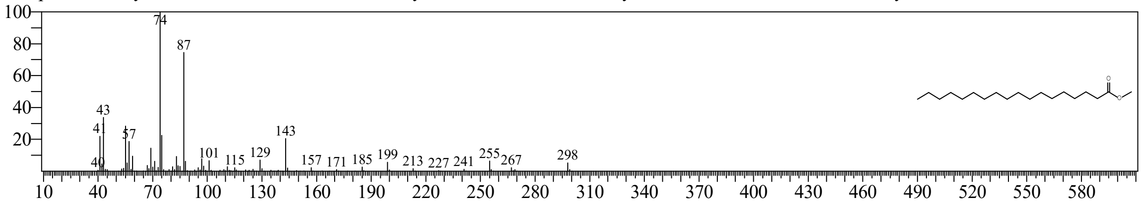

Hit#:3 Entry:28255 Library:NIST14s.lib
SI:96 Formula:C19H38O2 CAS:112-61-8 MolWeight:298 RetIndex:2077
CompName:Methyl stearate $$ Octadecanoic acid, methyl ester $$ Stearic acid, methyl ester $$ n-Octadecanoic acid, methyl ester $$ Kemester 9718 $$ Me

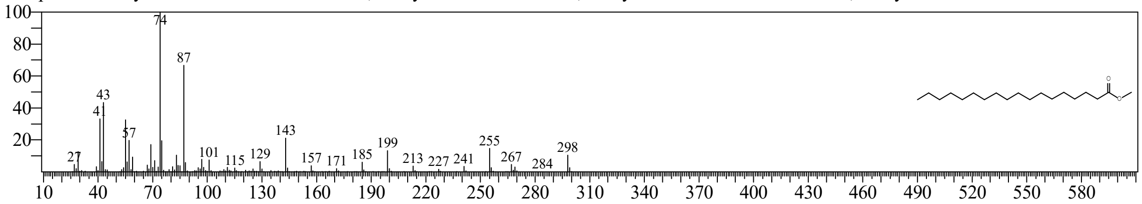

Hit#:4 Entry:129694 Library:NIST14.lib
SI:96 Formula:C19H38O2 CAS:112-61-8 MolWeight:298 RetIndex:2077
CompName:Methyl stearate $$ Octadecanoic acid, methyl ester $$ Stearic acid, methyl ester $$ n-Octadecanoic acid, methyl ester $$ Kemester 9718 $$ Me

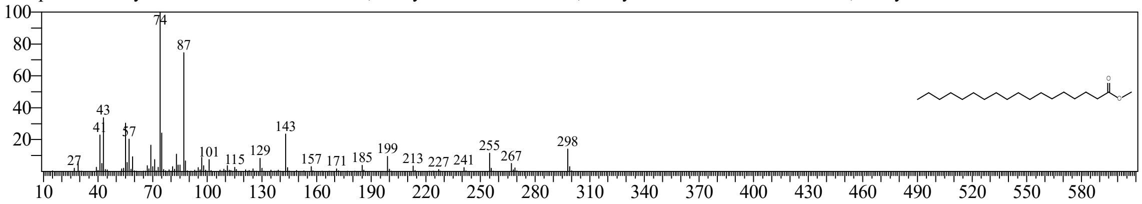

Hit#:5 Entry:141978 Library:NIST17-1.lib
SI:96 Formula:C19H38O2 CAS:112-61-8 MolWeight:298 RetIndex:2077
CompName:Methyl stearate $$ Octadecanoic acid, methyl ester $$ Stearic acid, methyl ester $$ n-Octadecanoic acid, methyl ester $$ Kemester 9718 $$ Me

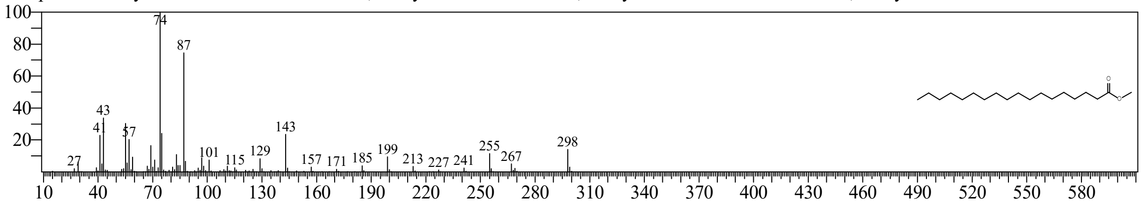

<< Target >>
Line#:10 R.Time:26.325(Scan#:1960) MassPeaks:372
RawMode:Averaged 26.317-26.333(1959-1961) BasePeak:55.05(194942)
BG Mode:Calc. from Peak Group 1 - Event 1

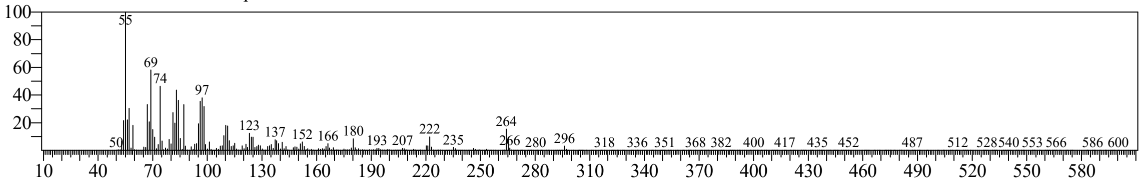

Hit#:1 Entry:28139 Library:NIST14s.lib
SI:97 Formula:C19H36O2 CAS:52380-33-3 MolWeight:296 RetIndex:2085
CompName:11-Octadecenoic acid, methyl ester $$ Methyl 11-octadecenoate $$ Octadec-11-enoic acid, methyl ester $$ Methyl (11E)-11-octadecenoate # $$

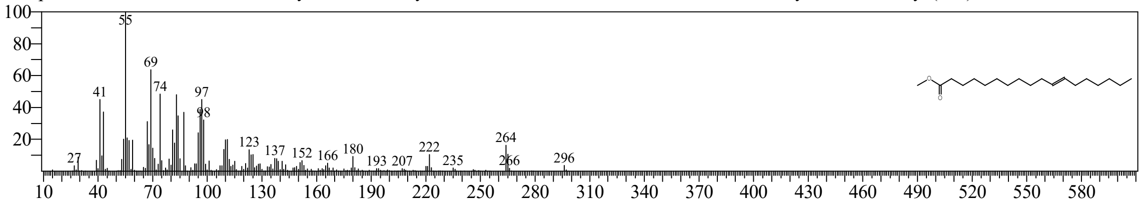

Hit#:2 Entry:32748 Library:NIST17s.lib
SI:97 Formula:C19H36O2 CAS:52380-33-3 MolWeight:296 RetIndex:2085
CompName:11-Octadecenoic acid, methyl ester $$ Methyl 11-octadecenoate $$ Octadec-11-enoic acid, methyl ester $$ Methyl (11E)-11-octadecenoate # $$

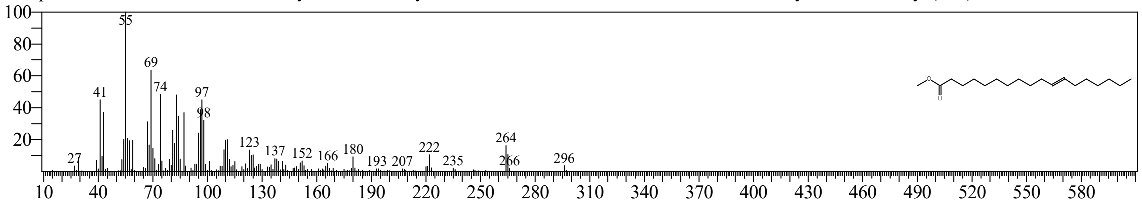

Hit#:3 Entry:139647 Library:NIST17-1.lib
SI:95 Formula:C19H36O2 CAS:0-00-0 MolWeight:296 RetIndex:2085
CompName:cis-13-Octadecenoic acid, methyl ester

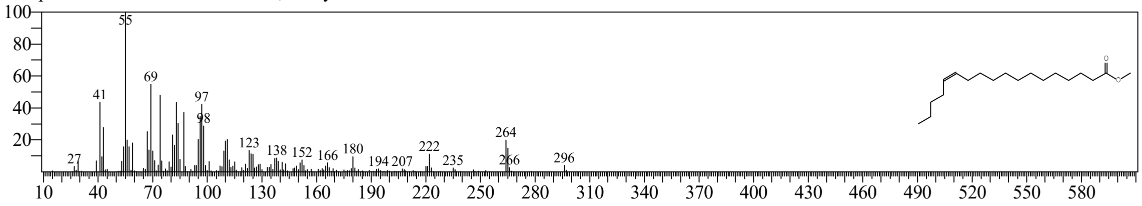

Hit#:4 Entry:127661 Library:NIST14.lib
SI:95 Formula:C19H36O2 CAS:0-00-0 MolWeight:296 RetIndex:2085
CompName:cis-13-Octadecenoic acid, methyl ester

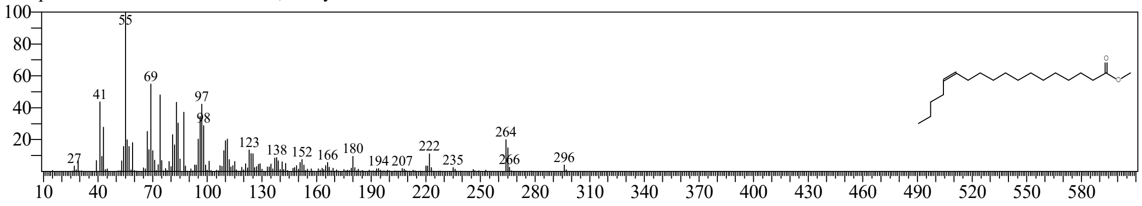

Hit#:5 Entry:127650 Library:NIST14.lib
SI:95 Formula:C19H36O2 CAS:13481-95-3 MolWeight:296 RetIndex:2085
CompName:10-Octadecenoic acid, methyl ester $$ Methyl 10-octadecenoate $$

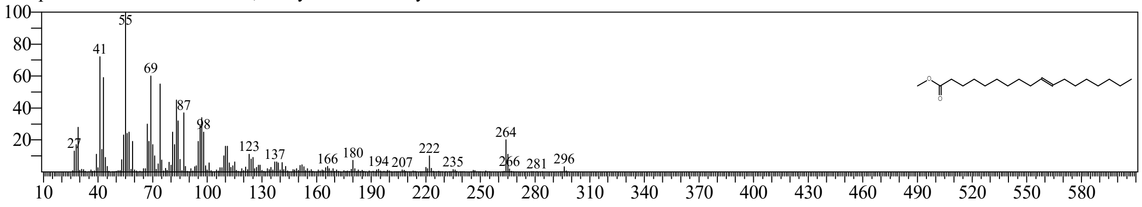

<< Target >>
Line#:11 R.Time:26.458(Scan#:1976) MassPeaks:335
RawMode:Averaged 26.450-26.467(1975-1977) BasePeak:55.05(12608)
BG Mode:Calc. from Peak Group 1 - Event 1

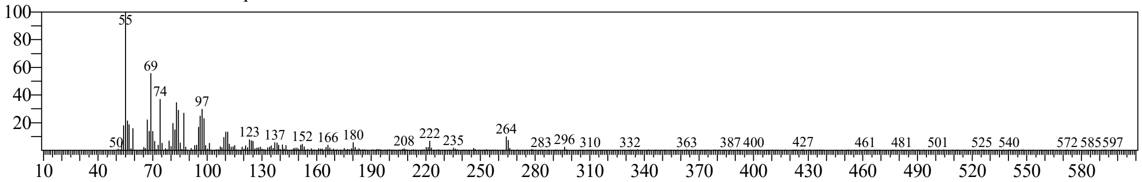

Hit#:1 Entry:127658 Library:NIST14.lib
SI:96 Formula:C19H36O2 CAS:0-00-0 MolWeight:296 RetIndex:2085
CompName:trans-13-Octadecenoic acid, methyl ester

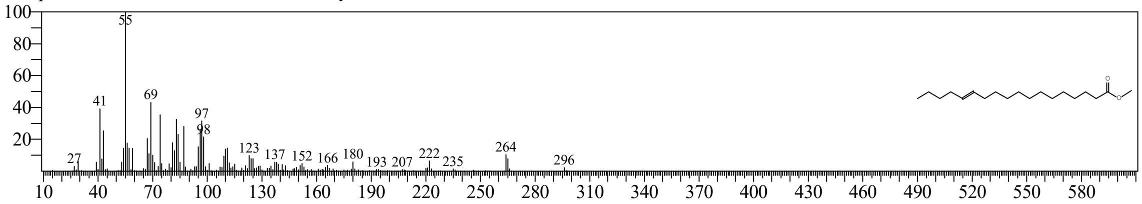

Hit#:2 Entry:139644 Library:NIST17-1.lib
SI:96 Formula:C19H36O2 CAS:0-00-0 MolWeight:296 RetIndex:2085
CompName:trans-13-Octadecenoic acid, methyl ester

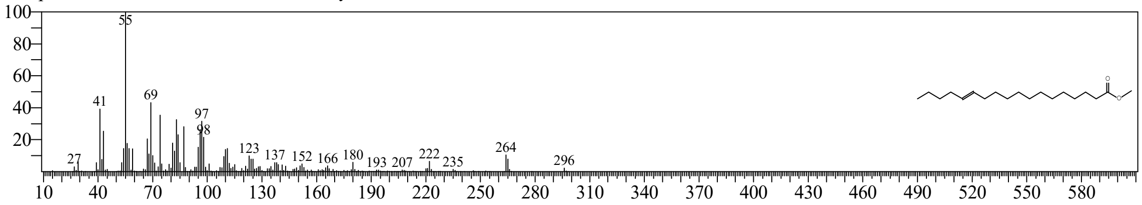

Hit#:3 Entry:28140 Library:NIST14s.lib
SI:95 Formula:C19H36O2 CAS:56554-47-3 MolWeight:296 RetIndex:2085
CompName:13-Octadecenoic acid, methyl ester $$ Methyl (13E)-13-octadecenoate # $$

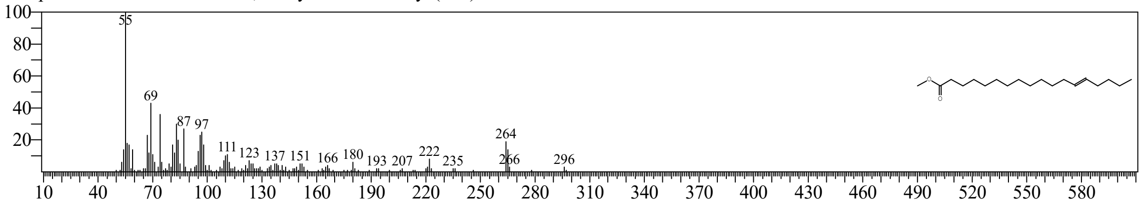

Hit#:4 Entry:32749 Library:NIST17s.lib
SI:95 Formula:C19H36O2 CAS:56554-47-3 MolWeight:296 RetIndex:2085
CompName:13-Octadecenoic acid, methyl ester $$ Methyl (13E)-13-octadecenoate # $$

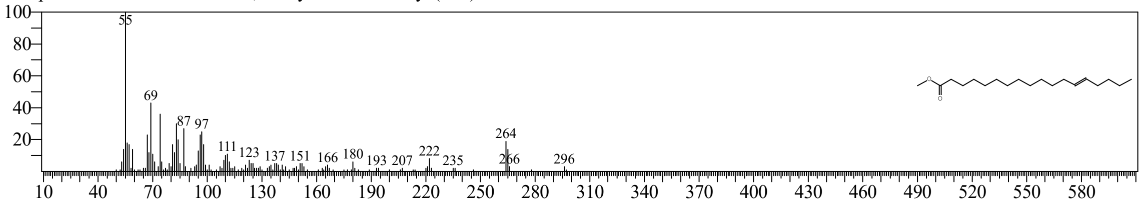

Hit#:5 Entry:127652 Library:NIST14.lib
SI:95 Formula:C19H36O2 CAS:56554-47-3 MolWeight:296 RetIndex:2085
CompName:13-Octadecenoic acid, methyl ester $$ Methyl (13E)-13-octadecenoate # $$

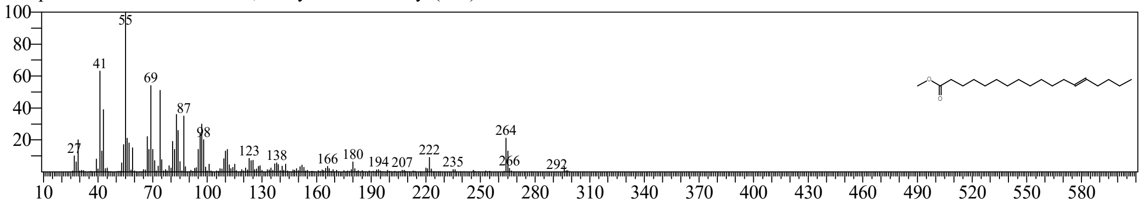

<< Target >>
Line#:12   R.Time:28.317(Scan#:2199) MassPeaks:401
RawMode:Averaged 28.308-28.325(2198-2200) BasePeak:67.05(86833)
BG Mode:Calc. from Peak Group 1 - Event 1

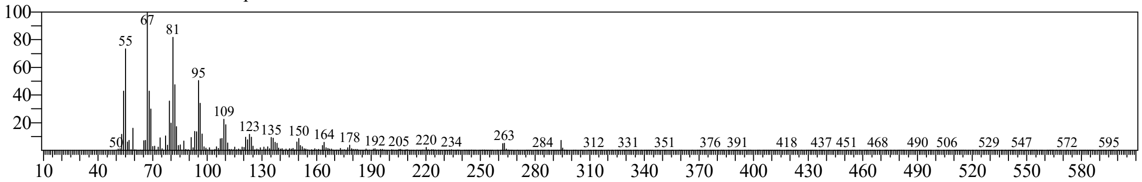

Hit#:1 Entry:125931 Library:NIST14.lib
SI:97 Formula:C19H34O2 CAS:2462-85-3 MolWeight:294 RetIndex:2093
CompName:9,12-Octadecadienoic acid, methyl ester $$ 9,12-Octadecenoic acid, methyl ester $$ Methyl octadeca-9,12-dienoate $$ Methyl 9,12-octadecadi

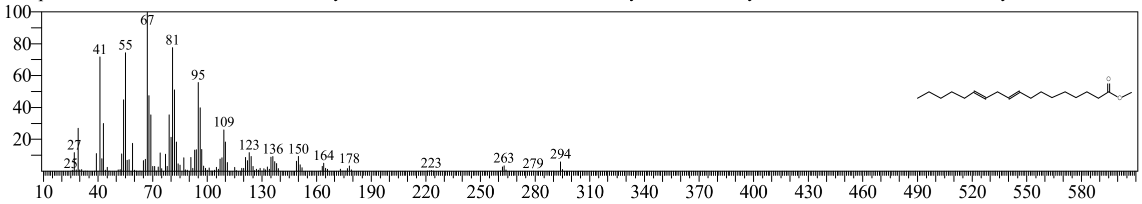

Hit#:2 Entry:137663 Library:NIST17-1.lib
SI:97 Formula:C19H34O2 CAS:2462-85-3 MolWeight:294 RetIndex:2093
CompName:9,12-Octadecadienoic acid, methyl ester $$ 9,12-Octadecenoic acid, methyl ester $$ Methyl octadeca-9,12-dienoate $$ Methyl 9,12-octadecadi

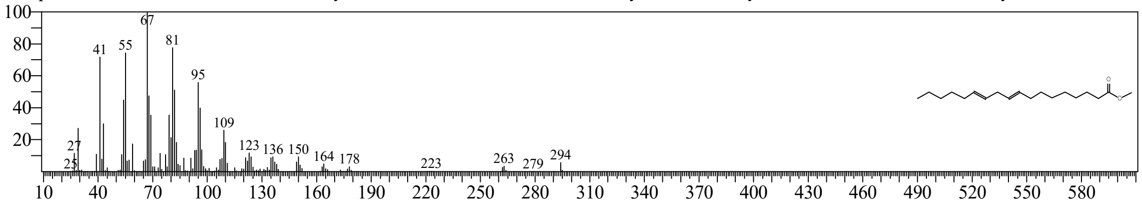

Hit#:3 Entry:32588 Library:NIST17s.lib
SI:96 Formula:C19H34O2 CAS:112-63-0 MolWeight:294 RetIndex:2093
CompName:9,12-Octadecadienoic acid (Z,Z)-, methyl ester $$ Linoleic acid, methyl ester $$ Methyl cis,cis-9,12-octadecadienoate $$ Methyl linoleate $$ M

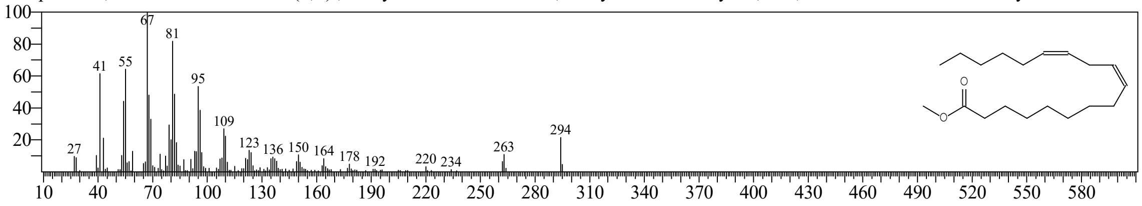

Hit#:4 Entry:27999 Library:NIST14s.lib
SI:96 Formula:C19H34O2 CAS:112-63-0 MolWeight:294 RetIndex:2093
CompName:9,12-Octadecadienoic acid (Z,Z)-, methyl ester $$ Linoleic acid, methyl ester $$ Methyl cis,cis-9,12-octadecadienoate $$ Methyl linoleate $$ M

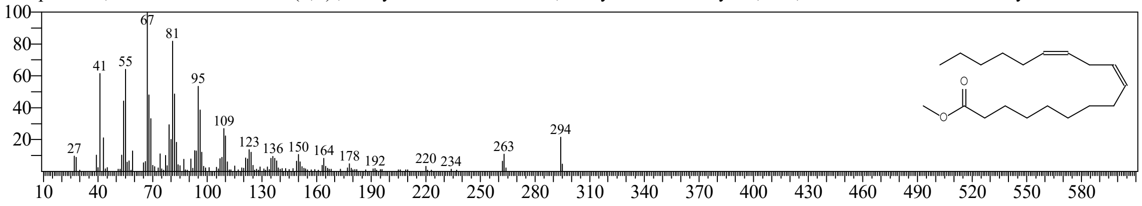

Hit#:5 Entry:32589 Library:NIST17s.lib
SI:96 Formula:C19H34O2 CAS:112-63-0 MolWeight:294 RetIndex:2093
CompName:9,12-Octadecadienoic acid (Z,Z)-, methyl ester $$ Linoleic acid, methyl ester $$ Methyl cis,cis-9,12-octadecadienoate $$ Methyl linoleate $$ M

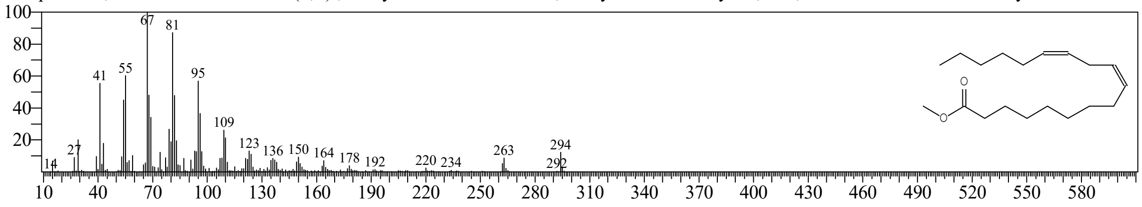

<< Target >>
Line#:13 R.Time:29.358(Scan#:2324) MassPeaks:305
RawMode:Averaged 29.350-29.367(2323-2325) BasePeak:74.05(8126)
BG Mode:Calc. from Peak Group 1 - Event 1

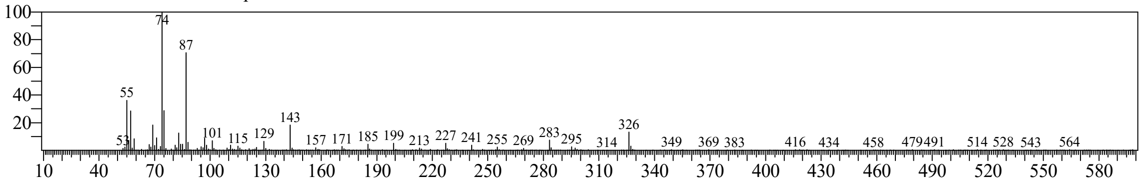

Hit#:1 Entry:34726 Library:NIST17s.lib
SI:97 Formula:C21H42O2 CAS:1120-28-1 MolWeight:326 RetIndex:2276
CompName:Eicosanoic acid, methyl ester $$ Methyl arachisate $$ Methyl eicosanoate $$ Arachidic acid methyl ester $$ Kemester 2050 $$ Methyl aracidat

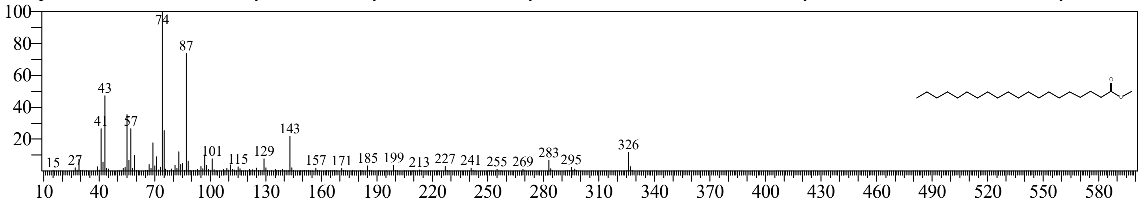

Hit#:2 Entry:154704 Library:NIST14.lib
SI:97 Formula:C21H42O2 CAS:0-00-0 MolWeight:326 RetIndex:2212
CompName:Methyl 18-methylnonadecanoate

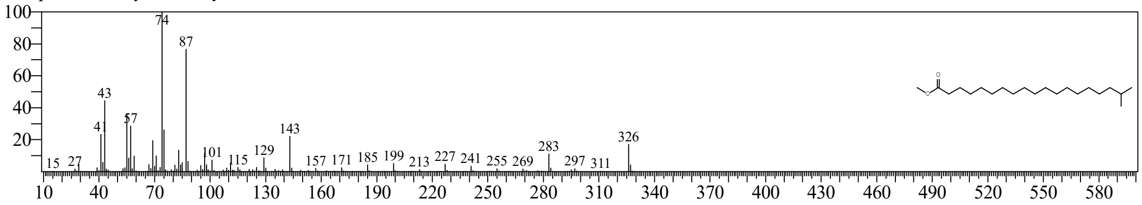

Hit#:3 Entry:170238 Library:NIST17-1.lib
SI:97 Formula:C21H42O2 CAS:0-00-0 MolWeight:326 RetIndex:2212
CompName:Methyl 18-methylnonadecanoate

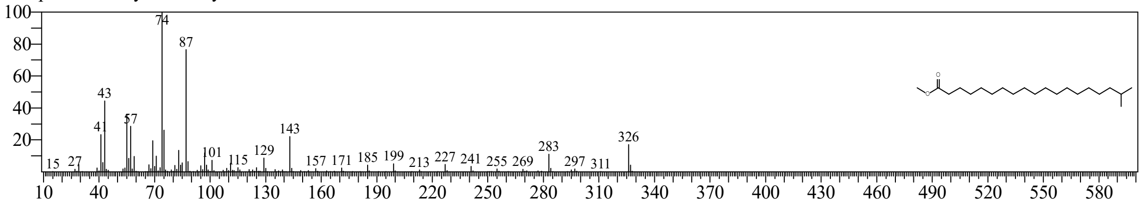

Hit#:4 Entry:34724 Library:NIST17s.lib
SI:95 Formula:C21H42O2 CAS:1120-28-1 MolWeight:326 RetIndex:2276
CompName:Eicosanoic acid, methyl ester $$ Methyl arachisate $$ Methyl eicosanoate $$ Arachidic acid methyl ester $$ Kemester 2050 $$ Methyl aracidat

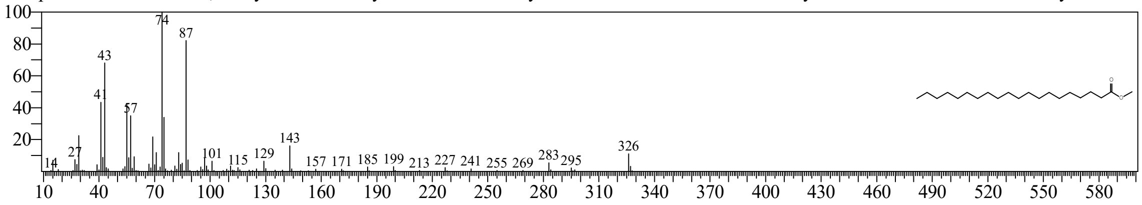

Hit#:5 Entry:29931 Library:NIST14s.lib
SI:95 Formula:C21H42O2 CAS:1120-28-1 MolWeight:326 RetIndex:2276
CompName:Eicosanoic acid, methyl ester $$ Methyl arachisate $$ Methyl eicosanoate $$ Arachidic acid methyl ester $$ Kemester 2050 $$ Methyl aracidat

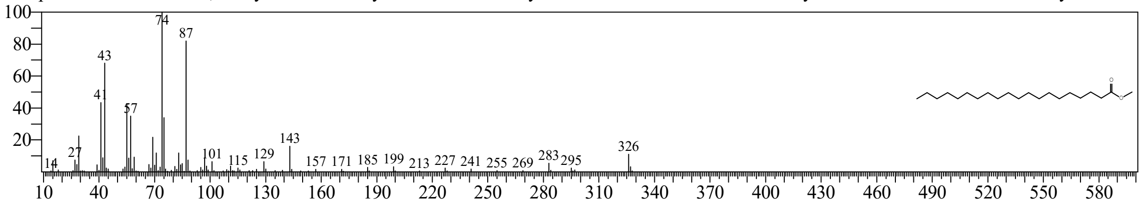

<< Target >>
Line#:14 R.Time:30.658(Scan#:2480) MassPeaks:272
RawMode:Averaged 30.650-30.667(2479-2481) BasePeak:79.10(846)
BG Mode:Calc. from Peak Group 1 - Event 1

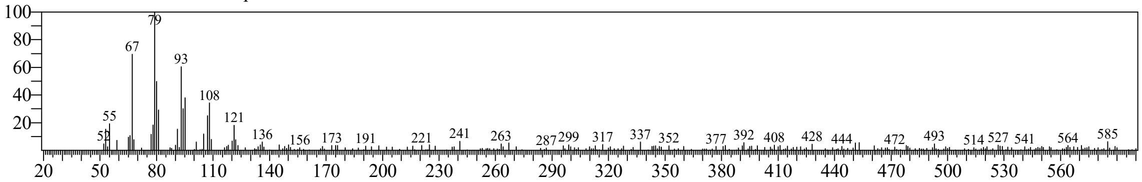

Hit#:1 Entry:72462 Library:NIST14.lib
SI:81 Formula:C17H28 CAS:10482-53-8 MolWeight:232 RetIndex:0
CompName:1,8,11,14-Heptadecatetraene, (Z,Z,Z)-

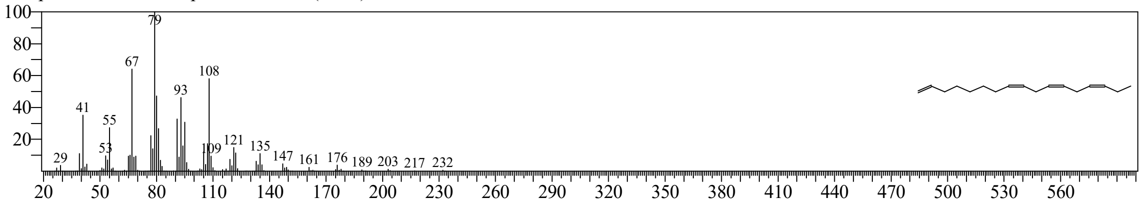

Hit#:2 Entry:77643 Library:NIST17-1.lib
SI:81 Formula:C17H28 CAS:10482-53-8 MolWeight:232 RetIndex:1725
CompName:1,8,11,14-Heptadecatetraene, (Z,Z,Z)-

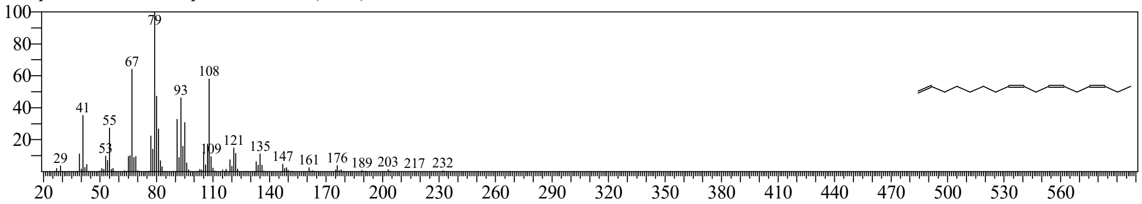

Hit#:3 Entry:10335 Library:NIST17-1.lib
SI:80 Formula:C10H16 CAS:42210-02-6 MolWeight:136 RetIndex:1003
CompName:Tricyclo[4.3.1.0(2,5)]decane

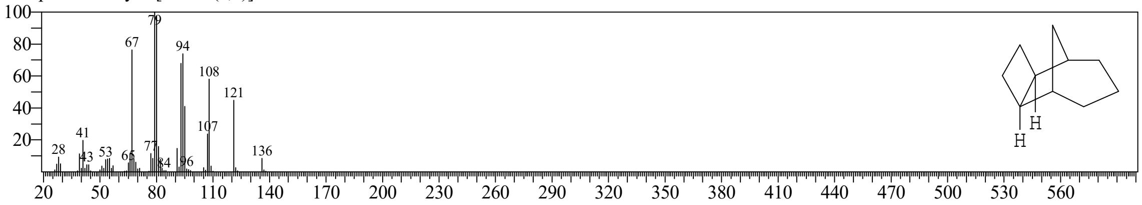

Hit#:4 Entry:9964 Library:NIST14.lib
SI:80 Formula:C10H16 CAS:42210-02-6 MolWeight:136 RetIndex:1003
CompName:Tricyclo[4.3.1.0(2,5)]decane

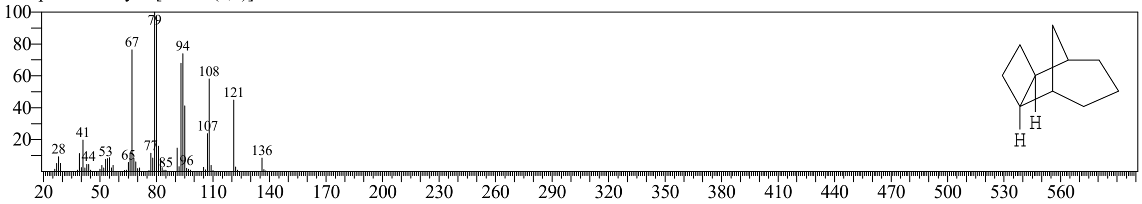

Hit#:5 Entry:94157 Library:NIST17-1.lib
SI:80 Formula:C16H26O2 CAS:7561-64-0 MolWeight:250 RetIndex:1992
CompName:7,10,13-Hexadecatrienoic acid, (Z,Z,Z)- $$ 7,10,13-Hexadecatrienoic acid, (7Z,10Z,13Z)- $$ (7Z,10Z,13Z)-7,10,13-Hexadecatrienoic acid $$ (

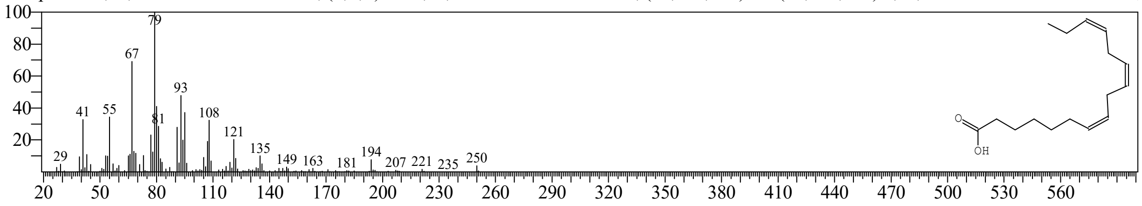

<< Target >>
Line#:15   R.Time:30.742(Scan#:2490) MassPeaks:304
RawMode:Averaged 30.733-30.750(2489-2491) BasePeak:55.10(1215)
BG Mode:Calc. from Peak Group 1 - Event 1

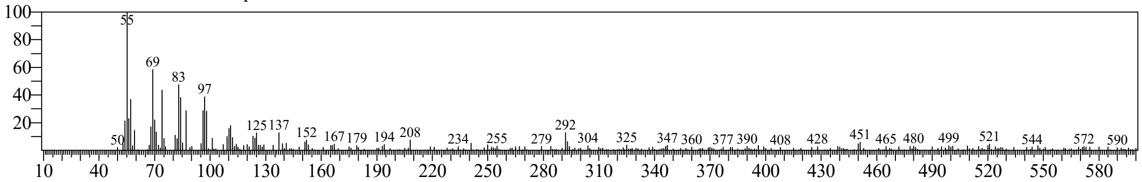

Hit#:1 Entry:152749 Library:NIST14.lib
SI:89 Formula:C21H40O2 CAS:2390-09-2 MolWeight:324 RetIndex:0
CompName:cis-Methyl 11-eicosenoate $$ 11-Eicosenoic acid, methyl ester, (Z)- $$

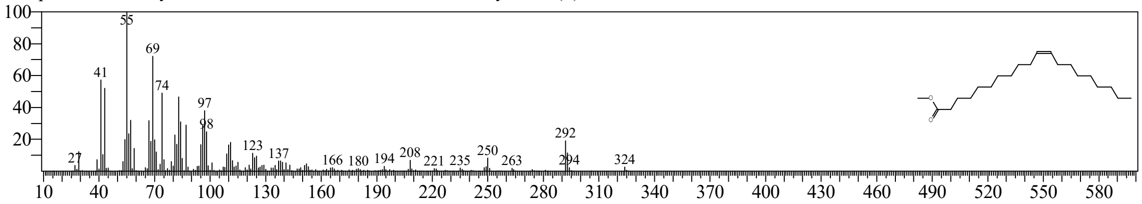

Hit#:2 Entry:152755 Library:NIST14.lib
SI:88 Formula:C21H40O2 CAS:0-00-0 MolWeight:324 RetIndex:2284
CompName:cis-11-Eicosenoic acid, methyl ester

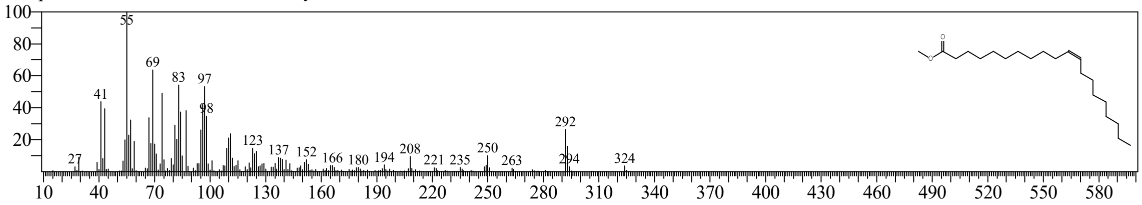

Hit#:3 Entry:168029 Library:NIST17-1.lib
SI:88 Formula:C21H40O2 CAS:2390-09-2 MolWeight:324 RetIndex:2284
CompName:cis-Methyl 11-eicosenoate $$ 11-Eicosenoic acid, methyl ester, (Z)- $$

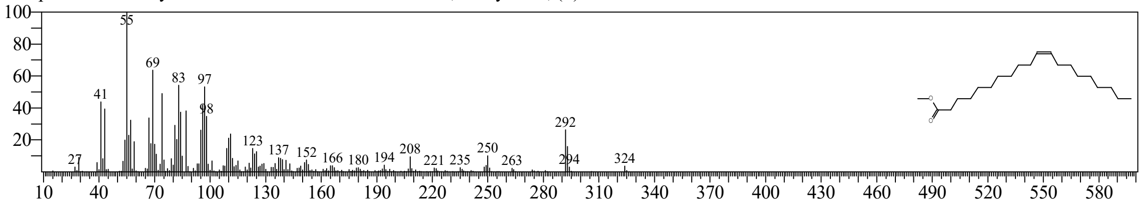

Hit#:4 Entry:31597 Library:NIST17s.lib
SI:87 Formula:C18H34O2 CAS:10152-61-1 MolWeight:282 RetIndex:1941
CompName:Cyclopropaneoctanoic acid, 2-hexyl-, methyl ester $$ Methyl 8-(2-hexylcyclopropyl)octanoate # $$

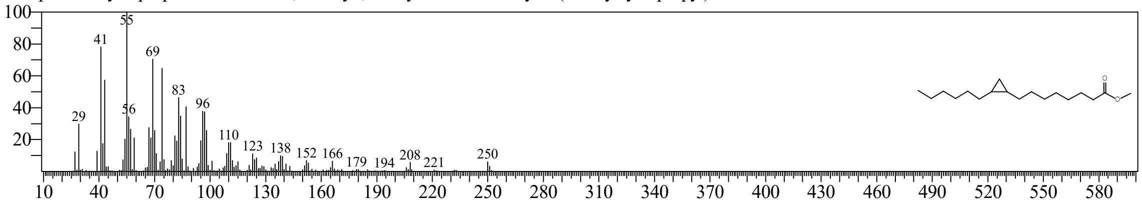

Hit#:5 Entry:27110 Library:NIST14s.lib
SI:87 Formula:C18H34O2 CAS:10152-61-1 MolWeight:282 RetIndex:1941
CompName:Cyclopropaneoctanoic acid, 2-hexyl-, methyl ester $$ Methyl 8-(2-hexylcyclopropyl)octanoate # $$

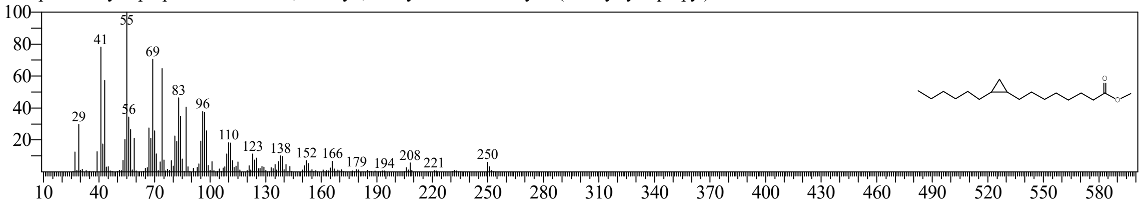

<< Target >>
Line#:16 R.Time:31.017(Scan#:2523) MassPeaks:309
RawMode:Averaged 31.008-31.025(2522-2524) BasePeak:67.05(3393)
BG Mode:Calc. from Peak Group 1 - Event 1

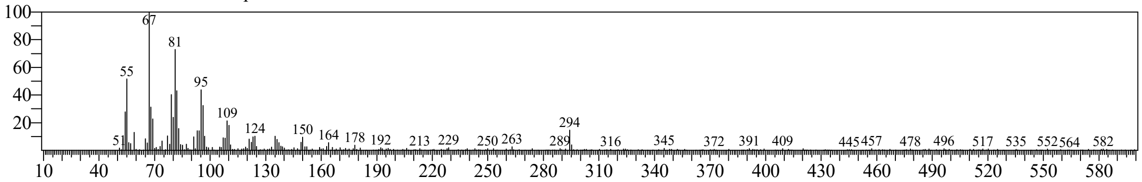

Hit#:1 Entry:32587 Library:NIST17s.lib
SI:96 Formula:C19H34O2 CAS:13038-47-6 MolWeight:294 RetIndex:2093
CompName:9,11-Octadecadienoic acid, methyl ester, (E,E)- $$ Methyl trans-9,trans-11-octadecadienoate $$ Methyl trans,trans-9,11-octadecadienoate $$ M

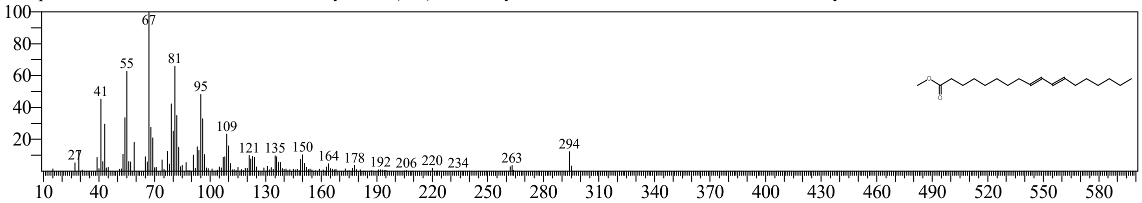

Hit#:2 Entry:125932 Library:NIST14.lib
SI:95 Formula:C19H34O2 CAS:0-00-0 MolWeight:294 RetIndex:2093
CompName:Methyl 9-cis,11-trans-octadecadienoate

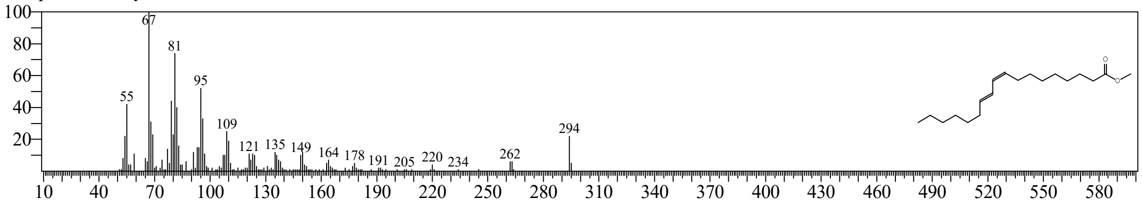

Hit#:3 Entry:137666 Library:NIST17-1.lib
SI:95 Formula:C19H34O2 CAS:13058-52-1 MolWeight:294 RetIndex:2093
CompName:Methyl 9-cis,11-trans-octadecadienoate

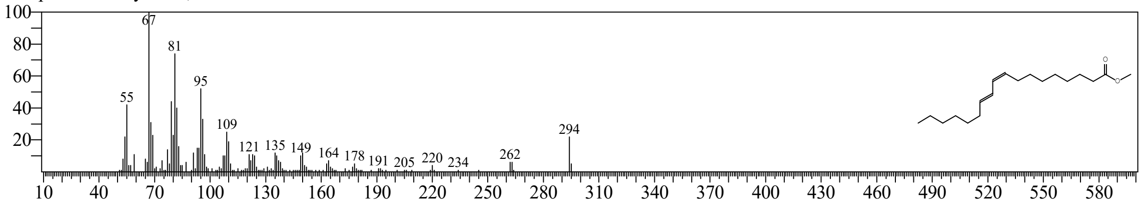

Hit#:4 Entry:137664 Library:NIST17-1.lib
SI:95 Formula:C19H34O2 CAS:0-00-0 MolWeight:294 RetIndex:2093
CompName:Methyl 10-trans,12-cis-octadecadienoate

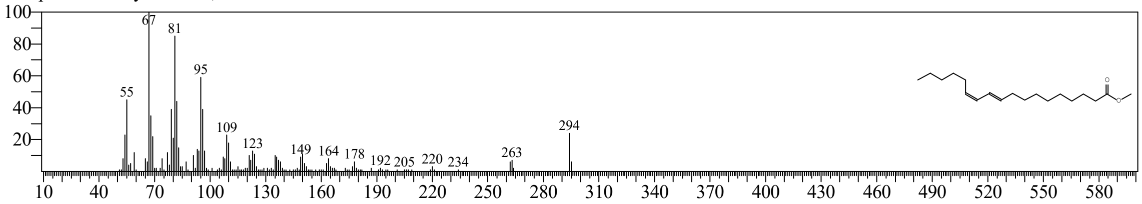

Hit#:5 Entry:125933 Library:NIST14.lib
SI:95 Formula:C19H34O2 CAS:0-00-0 MolWeight:294 RetIndex:2093
CompName:Methyl 10-trans,12-cis-octadecadienoate

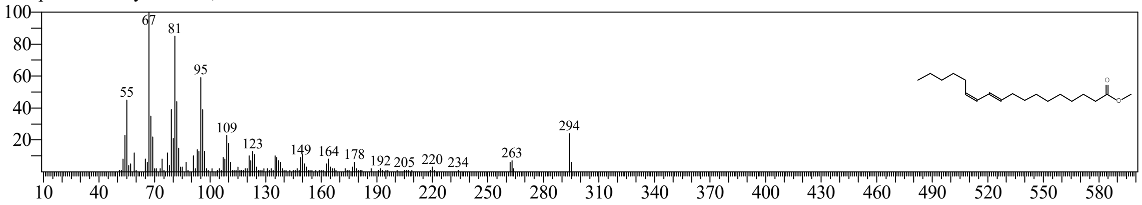

<< Target >>
Line#:17 R.Time:31.333(Scan#:2561) MassPeaks:381
RawMode:Averaged 31.325-31.342(2560-2562) BasePeak:67.05(3564)
BG Mode:Calc. from Peak Group 1 - Event 1

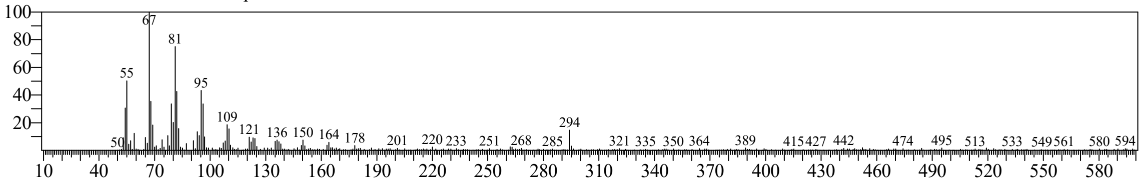

Hit#:1 Entry:32587 Library:NIST17s.lib
SI:95 Formula:C19H34O2 CAS:13038-47-6 MolWeight:294 RetIndex:2093
CompName:9,11-Octadecadienoic acid, methyl ester, (E,E)- $$ Methyl trans-9,trans-11-octadecadienoate $$ Methyl trans,trans-9,11-octadecadienoate $$ M

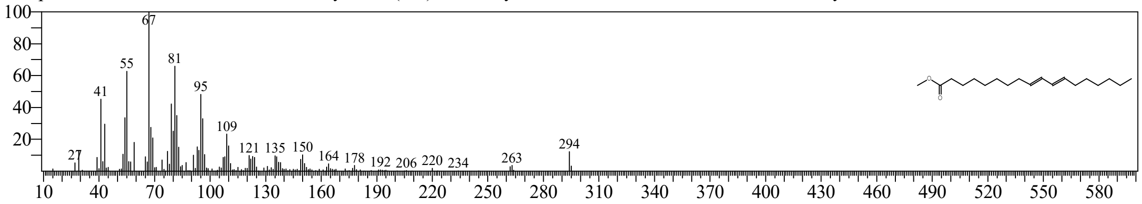

Hit#:2 Entry:137664 Library:NIST17-1.lib
SI:95 Formula:C19H34O2 CAS:0-00-0 MolWeight:294 RetIndex:2093
CompName:Methyl 10-trans,12-cis-octadecadienoate

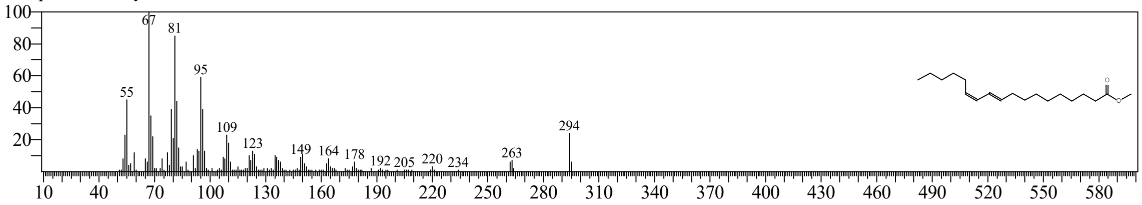

Hit#:3 Entry:125933 Library:NIST14.lib
SI:95 Formula:C19H34O2 CAS:0-00-0 MolWeight:294 RetIndex:2093
CompName:Methyl 10-trans,12-cis-octadecadienoate

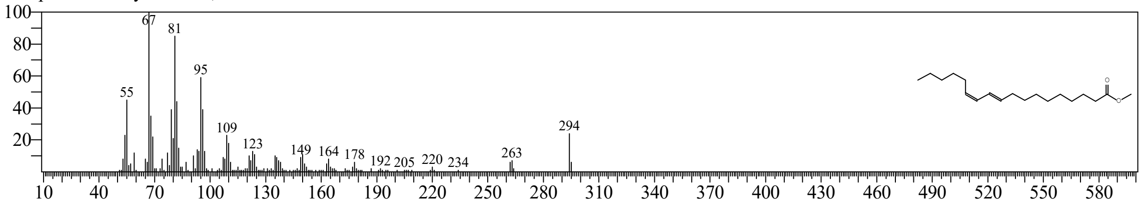

Hit#:4 Entry:32586 Library:NIST17s.lib
SI:95 Formula:C19H34O2 CAS:56554-61-1 MolWeight:294 RetIndex:2093
CompName:11,14-Octadecadienoic acid, methyl ester $$ Methyl (11E,14E)-11,14-octadecadienoate # $$

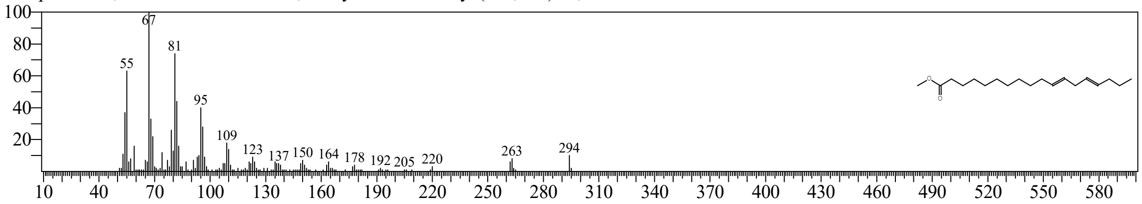

Hit#:5 Entry:27998 Library:NIST14s.lib
SI:95 Formula:C19H34O2 CAS:56554-61-1 MolWeight:294 RetIndex:2093
CompName:11,14-Octadecadienoic acid, methyl ester $$ Methyl (11E,14E)-11,14-octadecadienoate # $$

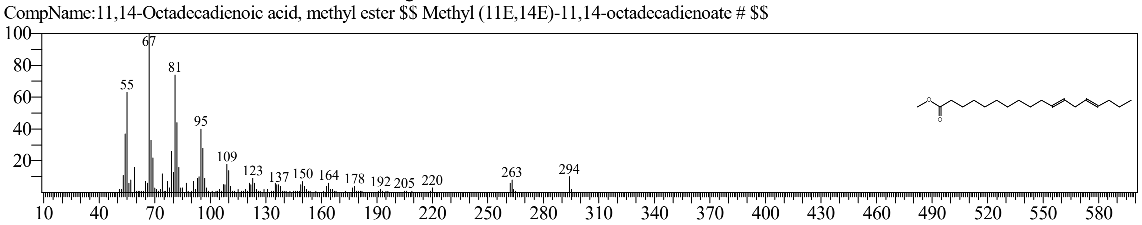

<< Target >>
Line#:18 R.Time:32.000(Scan#:2641) MassPeaks:300
RawMode:Averaged 31.992-32.008(2640-2642) BasePeak:67.05(2008)
BG Mode:Calc. from Peak Group 1 - Event 1

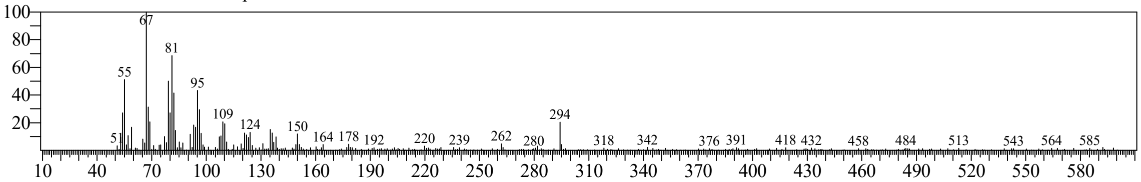

Hit#:1 Entry:137666 Library:NIST17-1.lib
SI:94 Formula:C19H34O2 CAS:13058-52-1 MolWeight:294 RetIndex:2093
CompName:Methyl 9-cis,11-trans-octadecadienoate

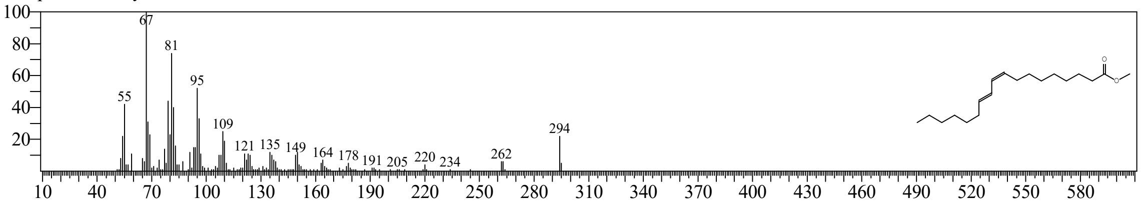

Hit#:2 Entry:125932 Library:NIST14.lib
SI:94 Formula:C19H34O2 CAS:0-00-0 MolWeight:294 RetIndex:2093
CompName:Methyl 9-cis,11-trans-octadecadienoate

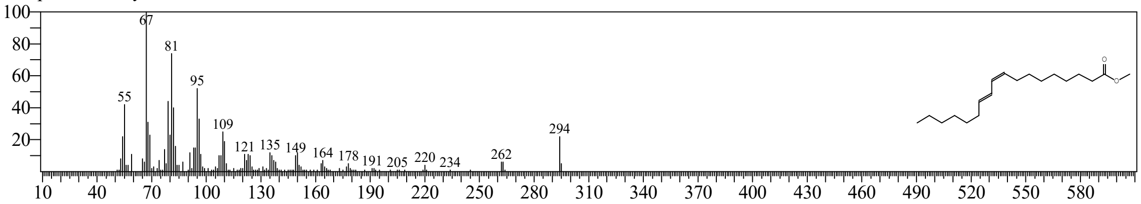

Hit#:3 Entry:32587 Library:NIST17s.lib
SI:94 Formula:C19H34O2 CAS:13038-47-6 MolWeight:294 RetIndex:2093
CompName:9,11-Octadecadienoic acid, methyl ester, (E,E)- $$ Methyl trans-9,trans-11-octadecadienoate $$ Methyl trans,trans-9,11-octadecadienoate $$ M

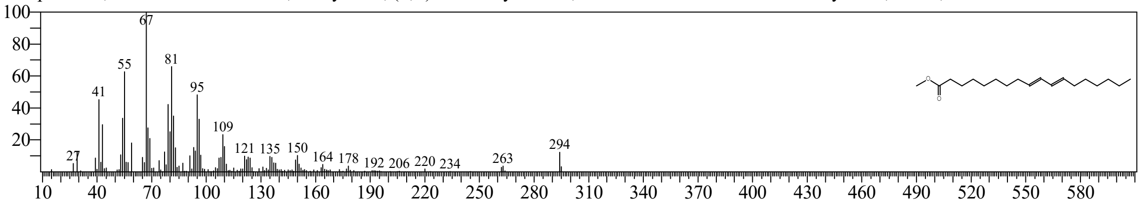

Hit#:4 Entry:125933 Library:NIST14.lib
SI:93 Formula:C19H34O2 CAS:0-00-0 MolWeight:294 RetIndex:2093
CompName:Methyl 10-trans,12-cis-octadecadienoate

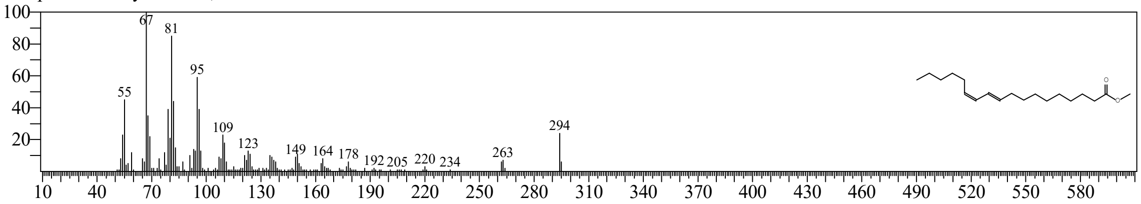

Hit#:5 Entry:137664 Library:NIST17-1.lib
SI:93 Formula:C19H34O2 CAS:0-00-0 MolWeight:294 RetIndex:2093
CompName:Methyl 10-trans,12-cis-octadecadienoate

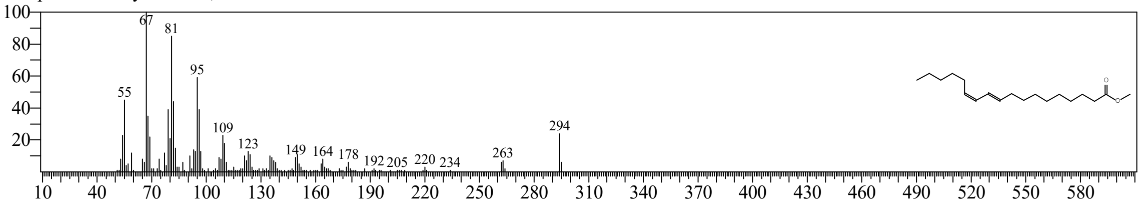

<< Target >>
Line#:19 R.Time:32.433(Scan#:2693) MassPeaks:328
RawMode:Averaged 32.425-32.442(2692-2694) BasePeak:71.05(5075)
BG Mode:Calc. from Peak Group 1 - Event 1

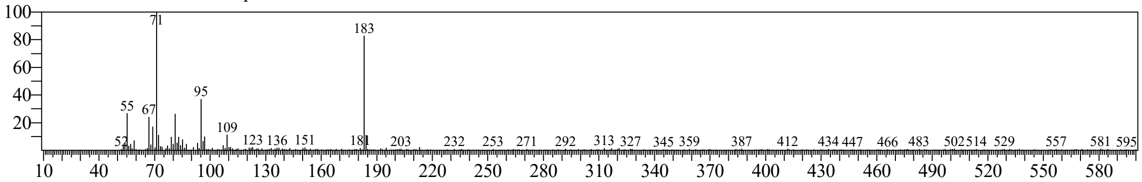

Hit#:1 Entry:196117 Library:NIST17-1.lib
SI:77 Formula:C21H38O4 CAS:0-00-0 MolWeight:354 RetIndex:2387
CompName:Succinic acid, dodec-2-enyl 3-methylbutyl ester

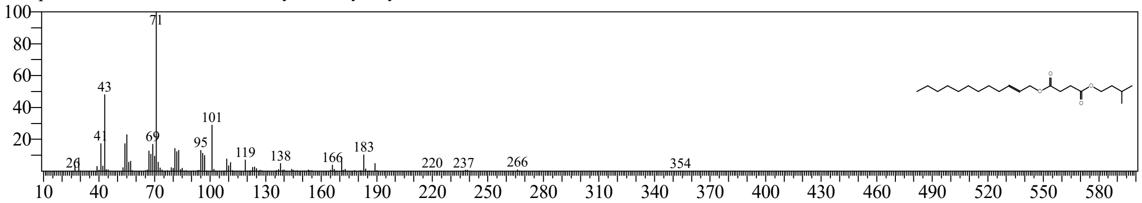

Hit#:2 Entry:177742 Library:NIST14.lib
SI:77 Formula:C21H38O4 CAS:0-00-0 MolWeight:354 RetIndex:2387
CompName:Succinic acid, dodec-2-enyl 3-methylbutyl ester

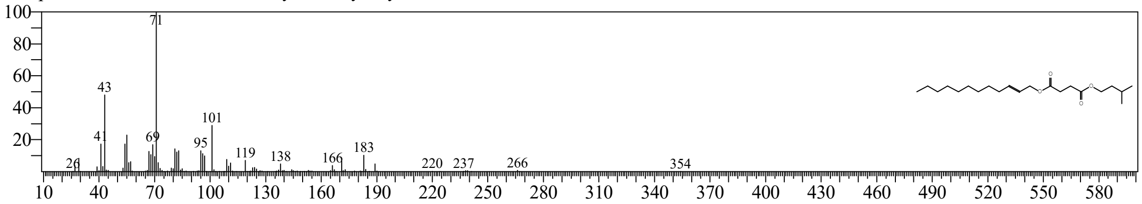

Hit#:3 Entry:67683 Library:NIST14.lib
SI:76 Formula:C14H26O2 CAS:2146-71-6 MolWeight:226 RetIndex:1570
CompName:Dodecanoic acid, ethenyl ester $$ Lauric acid, vinyl ester $$ Vinyl laurate $$ Vinyl dodecanoate $$

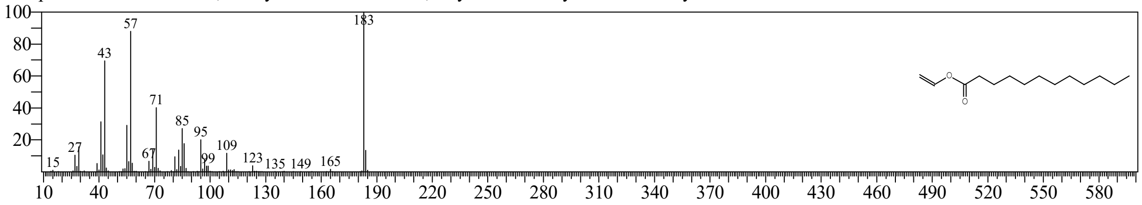

Hit#:4 Entry:72357 Library:NIST17-1.lib
SI:76 Formula:C14H26O2 CAS:2146-71-6 MolWeight:226 RetIndex:1570
CompName:Dodecanoic acid, ethenyl ester $$ Lauric acid, vinyl ester $$ Vinyl laurate $$ Vinyl dodecanoate $$

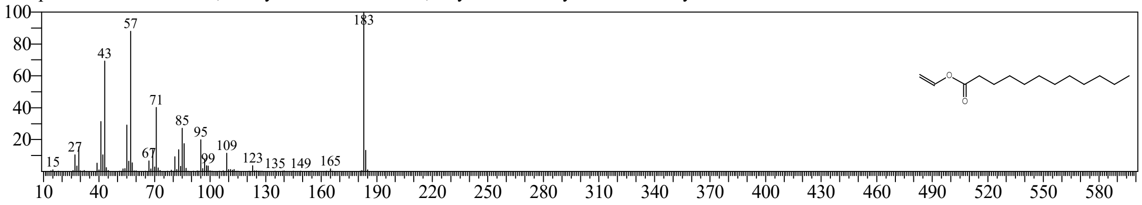

Hit#:5 Entry:60448 Library:NIST17-1.lib
SI:76 Formula:C13H27NO CAS:832-29-1 MolWeight:213 RetIndex:1893
CompName:Cyclododecanol, 1-aminomethyl- $$ 1-(Aminomethyl)cyclododecanol # $$

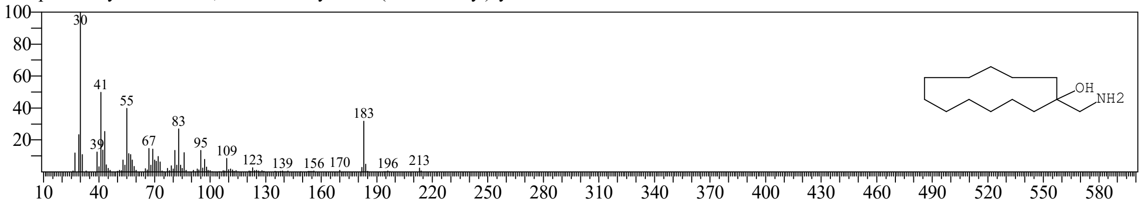

<< Target >>
Line#:20 R.Time:33.608(Scan#:2834) MassPeaks:303
RawMode:Averaged 33.600-33.617(2833-2835) BasePeak:74.05(8446)
BG Mode:Calc. from Peak Group 1 - Event 1

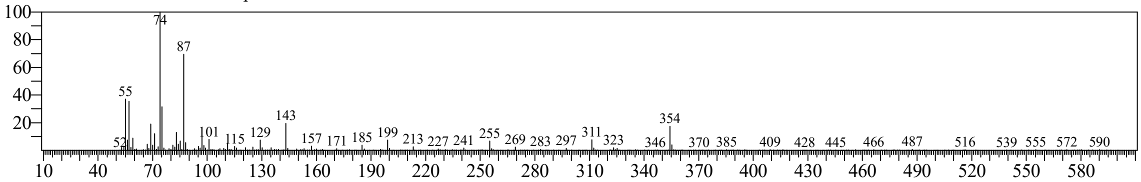

Hit#:1 Entry:178051 Library:NIST14.lib
SI:97 Formula:C23H46O2 CAS:929-77-1 MolWeight:354 RetIndex:2475
CompName:Docosanoic acid, methyl ester $$ Behenic acid, methyl ester $$ Methyl behenate $$ Methyl docosanoate $$ n-Docosanoic acid methyl ester $$

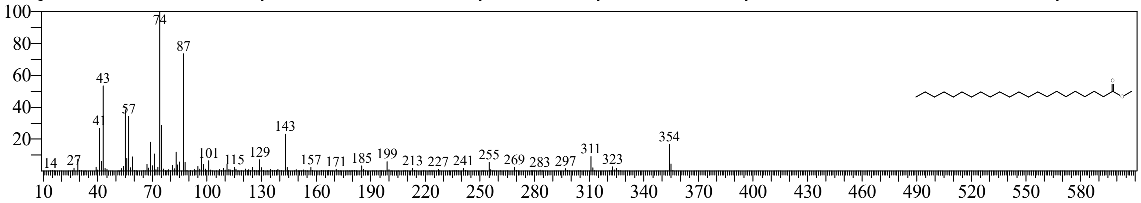

Hit#:2 Entry:196443 Library:NIST17-1.lib
SI:97 Formula:C23H46O2 CAS:929-77-1 MolWeight:354 RetIndex:2475
CompName:Docosanoic acid, methyl ester $$ Behenic acid, methyl ester $$ Methyl behenate $$ Methyl docosanoate $$ n-Docosanoic acid methyl ester $$

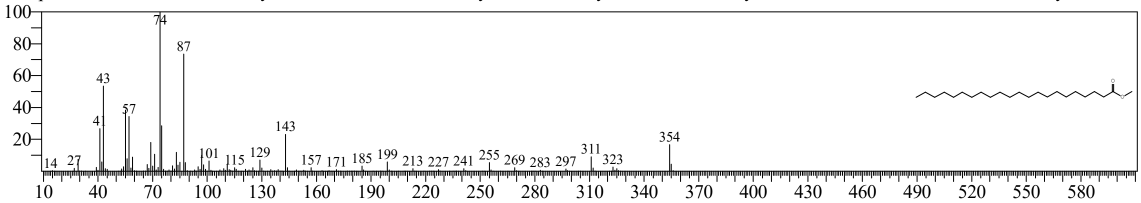

Hit#:3 Entry:36091 Library:NIST17s.lib
SI:94 Formula:C23H46O2 CAS:929-77-1 MolWeight:354 RetIndex:2475
CompName:Docosanoic acid, methyl ester $$ Behenic acid, methyl ester $$ Methyl behenate $$ Methyl docosanoate $$ n-Docosanoic acid methyl ester $$

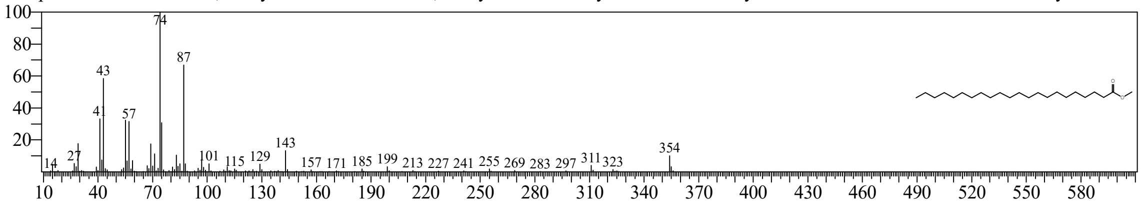

Hit#:4 Entry:31086 Library:NIST14s.lib
SI:94 Formula:C23H46O2 CAS:929-77-1 MolWeight:354 RetIndex:2475
CompName:Docosanoic acid, methyl ester $$ Behenic acid, methyl ester $$ Methyl behenate $$ Methyl docosanoate $$ n-Docosanoic acid methyl ester $$

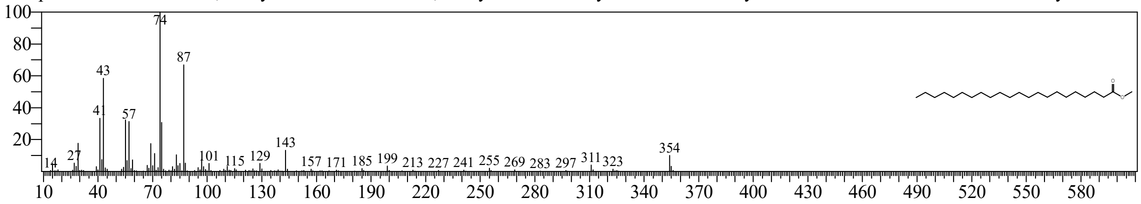

Hit#:5 Entry:36092 Library:NIST17s.lib
SI:94 Formula:C23H46O2 CAS:929-77-1 MolWeight:354 RetIndex:2475
CompName:Docosanoic acid, methyl ester $$ Behenic acid, methyl ester $$ Methyl behenate $$ Methyl docosanoate $$ n-Docosanoic acid methyl ester $$

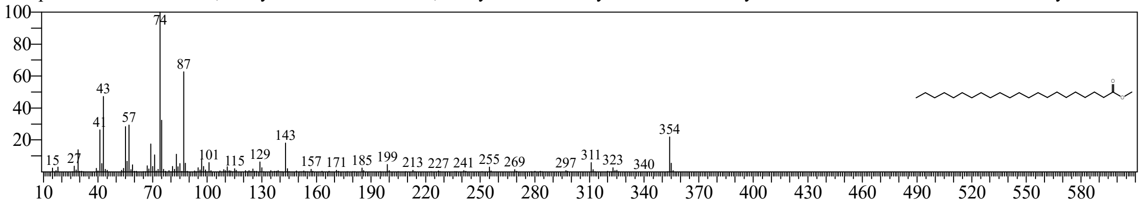

<< Target >>
Line#:21 R.Time:37.850(Scan#:3343) MassPeaks:371
RawMode:Averaged 37.842-37.858(3342-3344) BasePeak:79.10(3404)
BG Mode:Calc. from Peak Group 1 - Event 1

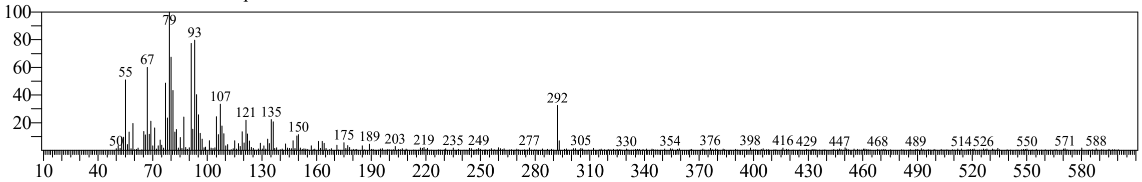

Hit#:1 Entry:135726 Library:NIST17-1.lib
SI:92 Formula:C19H32O2 CAS:0-00-0 MolWeight:292 RetIndex:2101
CompName:Methyl 9.cis.,11.trans.t,13.trans.-octadecatrienoate

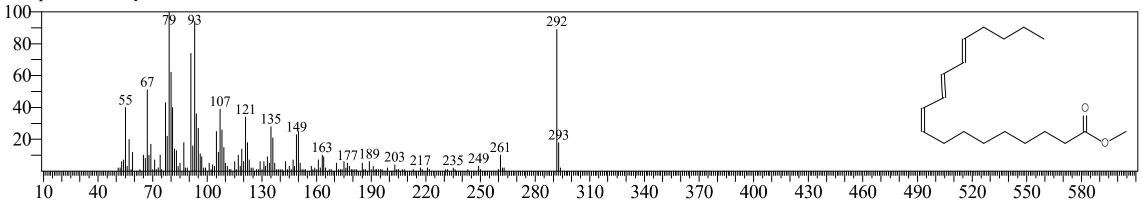

Hit#:2 Entry:124230 Library:NIST14.lib
SI:92 Formula:C19H32O2 CAS:0-00-0 MolWeight:292 RetIndex:2101
CompName:Methyl 9.cis.,11.trans.t,13.trans.-octadecatrienoate

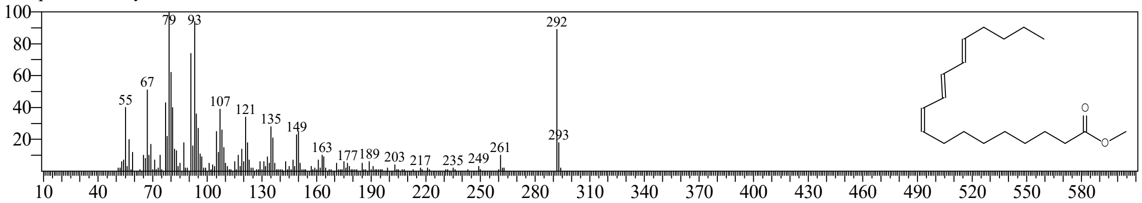

Hit#:3 Entry:124226 Library:NIST14.lib
SI:91 Formula:C19H32O2 CAS:0-00-0 MolWeight:292 RetIndex:2101
CompName:Methyl 6-cis,9-cis,11-trans-octadecatrienoate

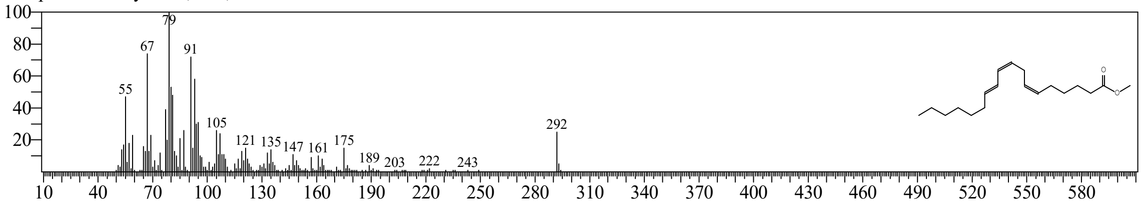

Hit#:4 Entry:135722 Library:NIST17-1.lib
SI:91 Formula:C19H32O2 CAS:0-00-0 MolWeight:292 RetIndex:2101
CompName:Methyl 6-cis,9-cis,11-trans-octadecatrienoate

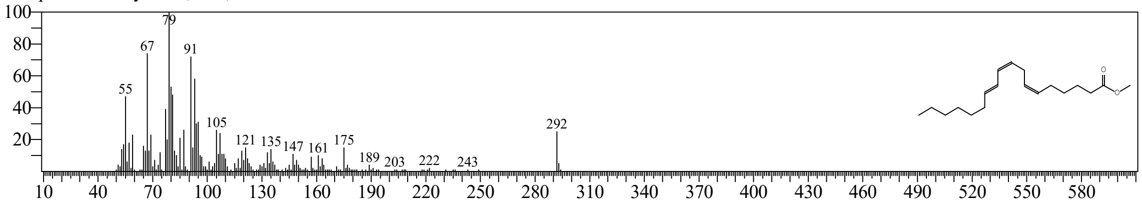

Hit#:5 Entry:124225 Library:NIST14.lib
SI:90 Formula:C19H32O2 CAS:0-00-0 MolWeight:292 RetIndex:2101
CompName:.gamma.-Linolenic acid, methyl ester

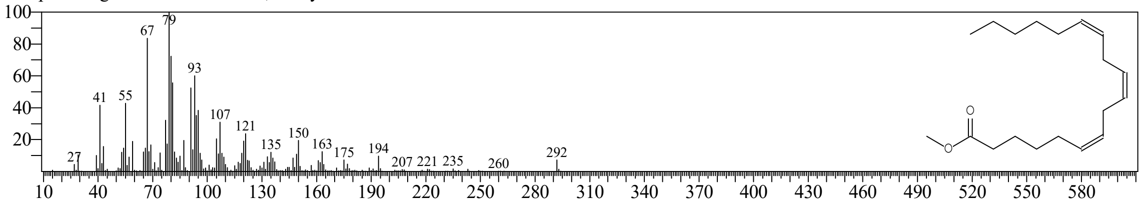

<< Target >>
Line#:22 R.Time:38.417(Scan#:3411) MassPeaks:352
RawMode:Averaged 38.408-38.425(3410-3412) BasePeak:79.10(3695)
BG Mode:Calc. from Peak Group 1 - Event 1

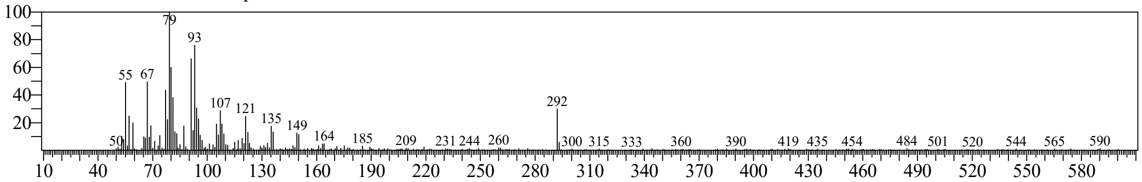

Hit#:1 Entry:135726 Library:NIST17-1.lib
SI:93 Formula:C19H32O2 CAS:0-00-0 MolWeight:292 RetIndex:2101
CompName:Methyl 9.cis.,11.trans.t,13.trans.-octadecatrienoate

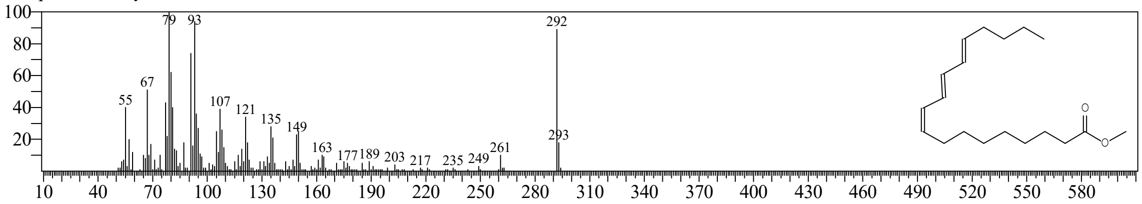

Hit#:2 Entry:124230 Library:NIST14.lib
SI:93 Formula:C19H32O2 CAS:0-00-0 MolWeight:292 RetIndex:2101
CompName:Methyl 9.cis.,11.trans.t,13.trans.-octadecatrienoate

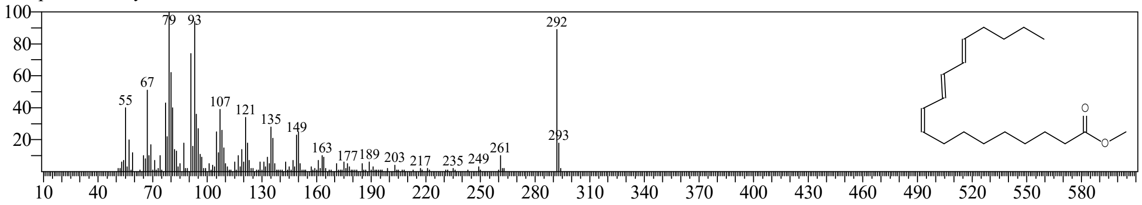

Hit#:3 Entry:124225 Library:NIST14.lib
SI:90 Formula:C19H32O2 CAS:0-00-0 MolWeight:292 RetIndex:2101
CompName:.gamma.-Linolenic acid, methyl ester

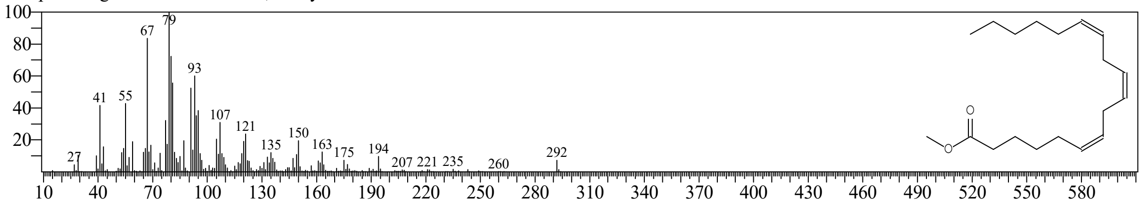

Hit#:4 Entry:135722 Library:NIST17-1.lib
SI:90 Formula:C19H32O2 CAS:0-00-0 MolWeight:292 RetIndex:2101
CompName:Methyl 6-cis,9-cis,11-trans-octadecatrienoate

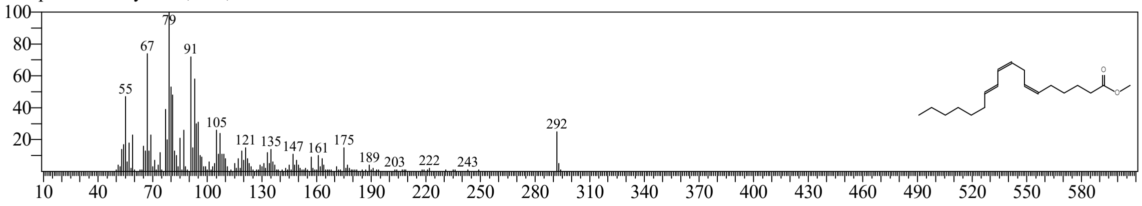

Hit#:5 Entry:124226 Library:NIST14.lib
SI:90 Formula:C19H32O2 CAS:0-00-0 MolWeight:292 RetIndex:2101
CompName:Methyl 6-cis,9-cis,11-trans-octadecatrienoate

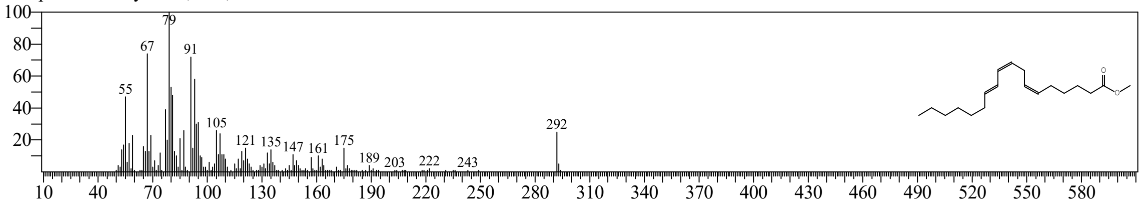

<< Target >>
Line#:23 R.Time:38.842(Scan#:3462) MassPeaks:334
RawMode:Averaged 38.833-38.850(3461-3463) BasePeak:79.10(5273)
BG Mode:Calc. from Peak Group 1 - Event 1

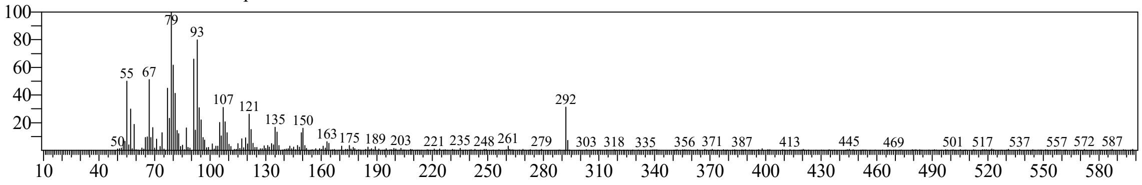

Hit#:1 Entry:124230 Library:NIST14.lib
SI:93 Formula:C19H32O2 CAS:0-00-0 MolWeight:292 RetIndex:2101
CompName:Methyl 9.cis.,11.trans.t,13.trans.-octadecatrienoate

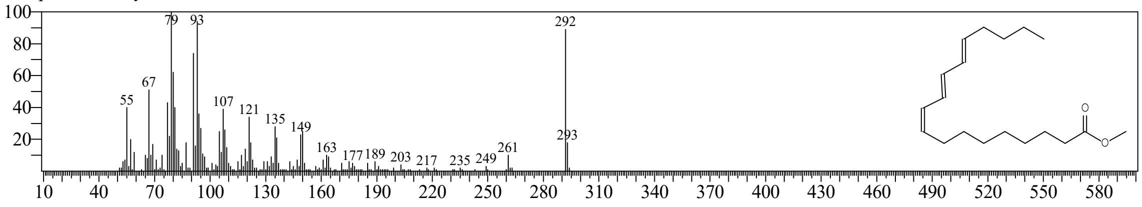

Hit#:2 Entry:135726 Library:NIST17-1.lib
SI:93 Formula:C19H32O2 CAS:0-00-0 MolWeight:292 RetIndex:2101
CompName:Methyl 9.cis.,11.trans.t,13.trans.-octadecatrienoate

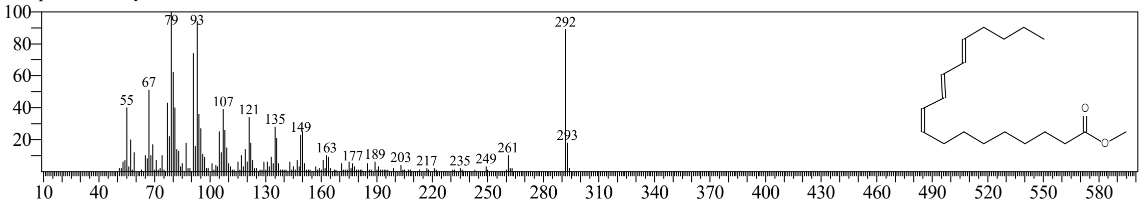

Hit#:3 Entry:124225 Library:NIST14.lib
SI:89 Formula:C19H32O2 CAS:0-00-0 MolWeight:292 RetIndex:2101
CompName:.gamma.-Linolenic acid, methyl ester

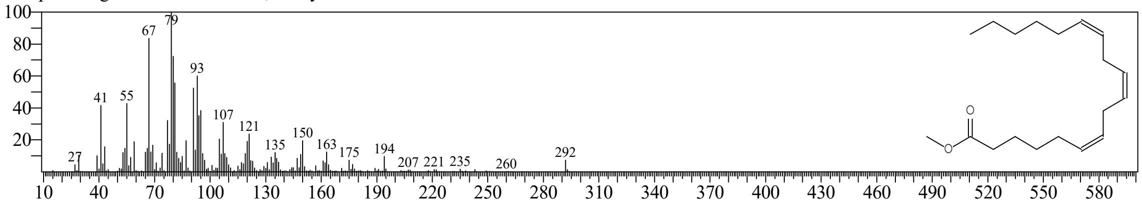

Hit#:4 Entry:135721 Library:NIST17-1.lib
SI:89 Formula:C19H32O2 CAS:16326-32-2 MolWeight:292 RetIndex:2101
CompName:Methyl .gamma.-linolenate

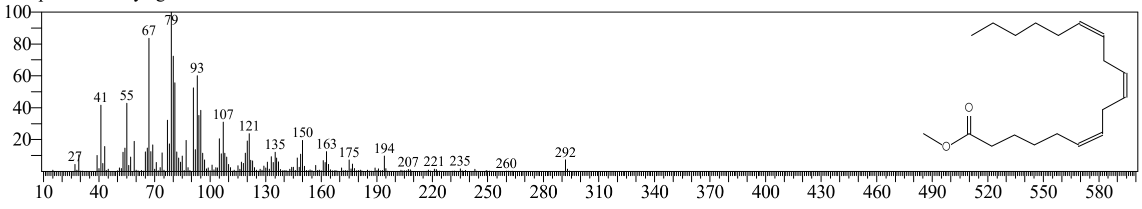

Hit#:5 Entry:135722 Library:NIST17-1.lib
SI:89 Formula:C19H32O2 CAS:0-00-0 MolWeight:292 RetIndex:2101
CompName:Methyl 6-cis,9-cis,11-trans-octadecatrienoate

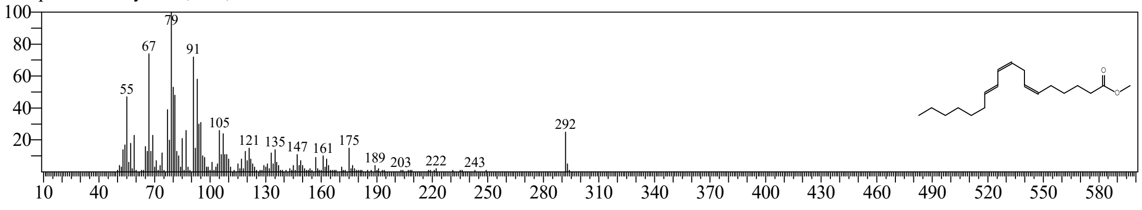

<< Target >>
Line#:24 R.Time:38.983(Scan#:3479) MassPeaks:367
RawMode:Averaged 38.975-38.992(3478-3480) BasePeak:79.10(4581)
BG Mode:Calc. from Peak Group 1 - Event 1

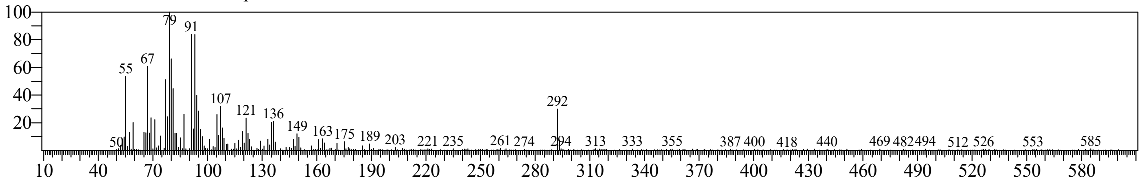

Hit#:1 Entry:135726 Library:NIST17-1.lib
SI:92 Formula:C19H32O2 CAS:0-00-0 MolWeight:292 RetIndex:2101
CompName:Methyl 9.cis.,11.trans.t,13.trans.-octadecatrienoate

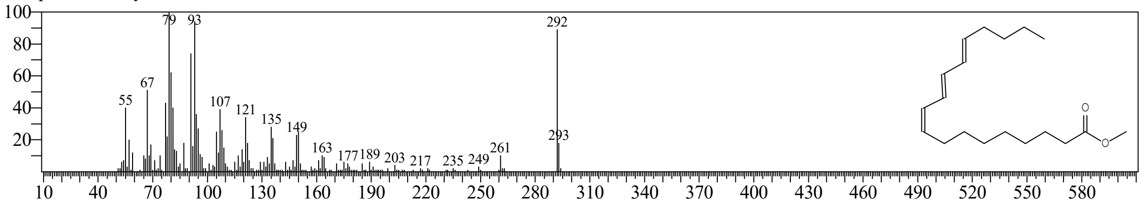

Hit#:2 Entry:124230 Library:NIST14.lib
SI:92 Formula:C19H32O2 CAS:0-00-0 MolWeight:292 RetIndex:2101
CompName:Methyl 9.cis.,11.trans.t,13.trans.-octadecatrienoate

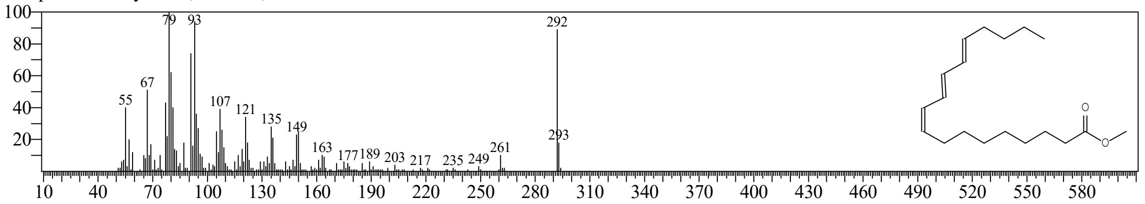

Hit#:3 Entry:124226 Library:NIST14.lib
SI:91 Formula:C19H32O2 CAS:0-00-0 MolWeight:292 RetIndex:2101
CompName:Methyl 6-cis,9-cis,11-trans-octadecatrienoate

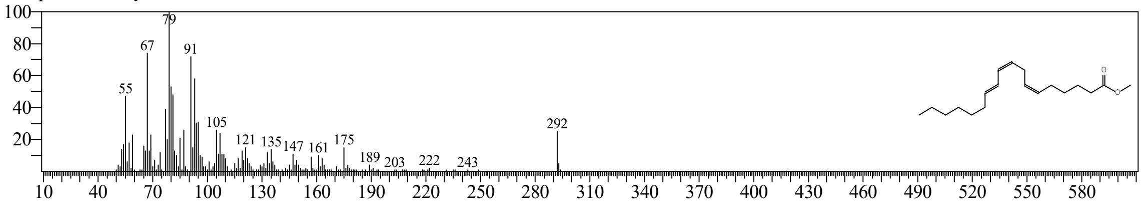

Hit#:4 Entry:135722 Library:NIST17-1.lib
SI:91 Formula:C19H32O2 CAS:0-00-0 MolWeight:292 RetIndex:2101
CompName:Methyl 6-cis,9-cis,11-trans-octadecatrienoate

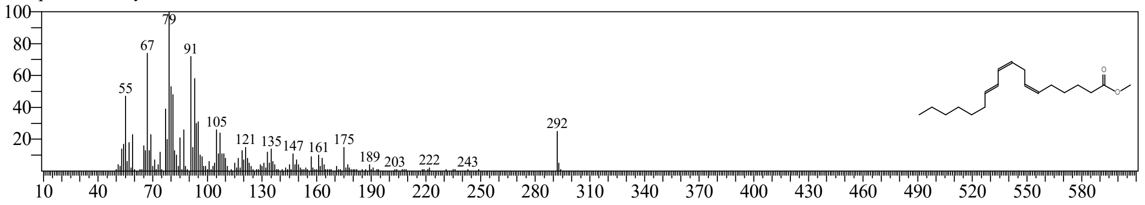

Hit#:5 Entry:135721 Library:NIST17-1.lib
SI:89 Formula:C19H32O2 CAS:16326-32-2 MolWeight:292 RetIndex:2101
CompName:Methyl .gamma.-linolenate

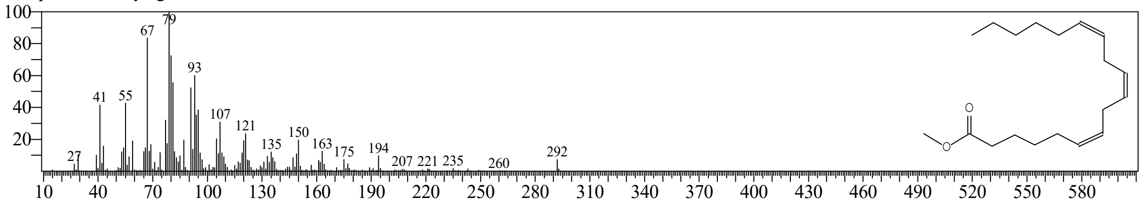

Supplement: Supplementary file 1 [file molecules-31-02891-s001.zip › 56d-CK-2.pdf]
