# Supplementary material for: Selective Inhibition of Lipid Oxidation by a Quercetagetin-Rich Extract from Tagetes erecta: A Chemometric Approach Using OPLS-DA and Selectivity Index Analysis
Source: Molecules. 2026 Aug 19;31(16):2891. doi: 10.3390/molecules31162891 (PMC13515886; doi:10.3390/molecules31162891)

K-3-56d

<<Column>>
Name : HP-88
Thickness : 0.20um
Length : 100.0m
Inside Diameter : 0.25mm

Method

===== Analytical Line 1 =====

Column Oven Temp.	:120.0 °C	
Injection Temp.	:250.00 °C	
Injection Mode	:Split	
Column Flow	:1.04 mL/min	
Split Ratio	:20.0	
Oven Temp. Program		
Rate	Temperature(°C)	Hold Time(min)
-	120.0	1.00
10.00	175.0	10.00
5.00	210.0	5.00
5.00	230.0	15.00

[GCMS-QP2010]
IonSourceTemp :200.00 °C
Interface Temp. :220.00 °C
m/z scanning range：45～500m/z

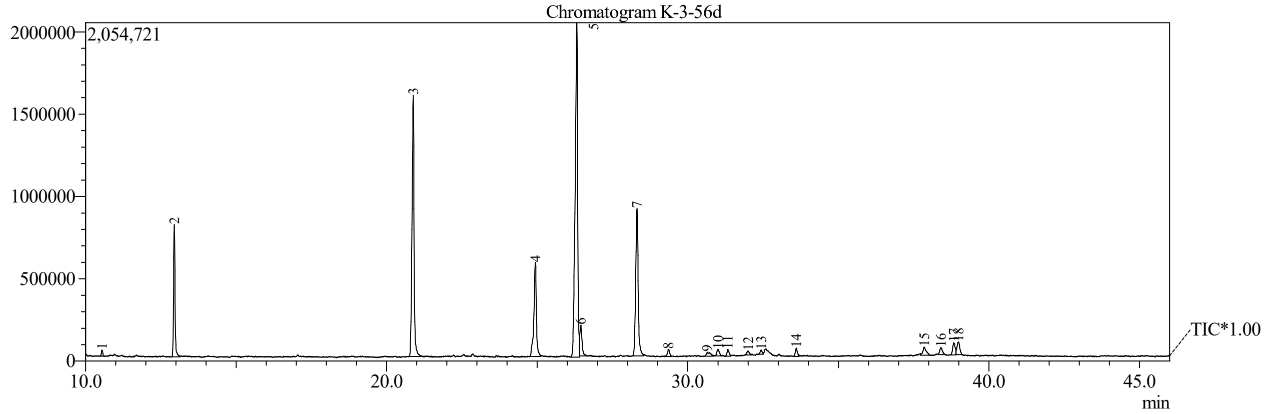

Peak Report TIC

Peak#	R.Time	Area	Area%	Height	Height%	A/H	Name
1	10.549	107684	0.32	36866	0.56	2.92	Octanoic acid, methyl ester
2	12.948	2610721	7.66	802235	12.17	3.25	Undecanoic acid, methyl ester
3	20.884	7441600	21.84	1586274	24.06	4.69	Hexadecanoic acid, methyl ester
4	24.940	3290291	9.66	572725	8.69	5.74	Methyl stearate
5	26.314	11780052	34.57	2029537	30.78	5.80	11-Octadecenoic acid, methyl ester
6	26.449	862642	2.53	188204	2.85	4.58	trans-13-Octadecenoic acid, methyl ester
7	28.314	5058852	14.85	895701	13.58	5.65	9,12-Octadecadienoic acid, methyl ester
8	29.354	220894	0.65	42753	0.65	5.17	Eicosanoic acid, methyl ester
9	30.648	236023	0.69	23283	0.35	10.14	9,12,15-Octadecatrienoic acid, methyl ester, (Z
10	31.005	235296	0.69	40523	0.61	5.81	9,11-Octadecadienoic acid, methyl ester, (E,E)
11	31.327	196323	0.58	39588	0.60	4.96	11,14-Octadecadienoic acid, methyl ester
12	32.001	126240	0.37	22817	0.35	5.53	Methyl 9-cis,11-trans-octadecadienoate
13	32.435	111251	0.33	24772	0.38	4.49	Dodecanoic acid, ethenyl ester
14	33.605	235871	0.69	49042	0.74	4.81	Docosanoic acid, methyl ester

Peak#	R.Time	Area	Area%	Height	Height%	A/H	Name
15	37.848	383203	1.12	47844	0.73	8.01	Methyl 9.cis.,11.trans.t,13.trans.-octadecatrien
16	38.410	261792	0.77	42247	0.64	6.20	Methyl 9.cis.,11.trans.t,13.trans.-octadecatrien
17	38.834	430738	1.26	72394	1.10	5.95	Methyl 9.cis.,11.trans.t,13.trans.-octadecatrien
18	38.988	483913	1.42	77004	1.17	6.28	Methyl 6-cis,9-cis,11-trans-octadecatrienoate
		34073386	100.00	6593809	100.00		

Library

<< Target >>
Line#:1 R.Time:31.325(Scan#:2560) MassPeaks:330
RawMode:Single 31.325(2560) BasePeak:67.05(2706)
BG Mode:Averaged 31.233-31.417(2549-2571) Group 1 - Event 1

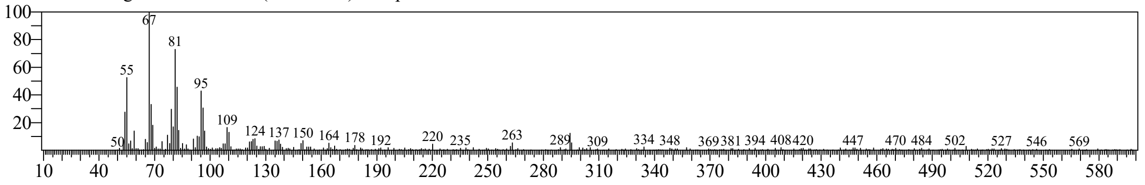

Hit#:1 Entry:32586 Library:NIST17s.lib
SI:93 Formula:C19H34O2 CAS:56554-61-1 MolWeight:294 RetIndex:2093
CompName:11,14-Octadecadienoic acid, methyl ester $$ Methyl (11E,14E)-11,14-octadecadienoate # $$

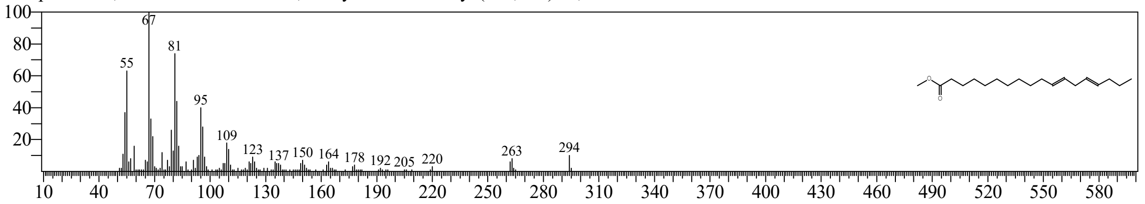

Hit#:2 Entry:27998 Library:NIST14s.lib
SI:93 Formula:C19H34O2 CAS:56554-61-1 MolWeight:294 RetIndex:2093
CompName:11,14-Octadecadienoic acid, methyl ester $$ Methyl (11E,14E)-11,14-octadecadienoate # $$

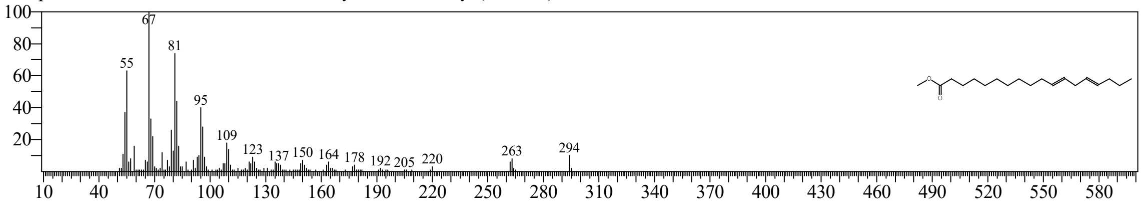

Hit#:3 Entry:32587 Library:NIST17s.lib
SI:93 Formula:C19H34O2 CAS:13038-47-6 MolWeight:294 RetIndex:2093
CompName:9,11-Octadecadienoic acid, methyl ester, (E,E)- $$ Methyl trans-9,trans-11-octadecadienoate $$ Methyl trans,trans-9,11-octadecadienoate $$ M

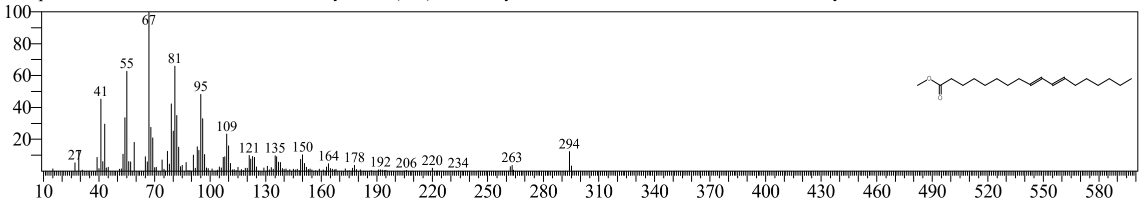

Hit#:4 Entry:137666 Library:NIST17-1.lib
SI:93 Formula:C19H34O2 CAS:13058-52-1 MolWeight:294 RetIndex:2093
CompName:Methyl 9-cis,11-trans-octadecadienoate

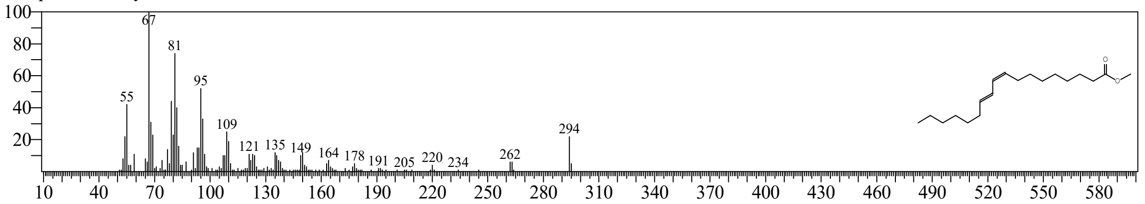

Hit#:5 Entry:125932 Library:NIST14.lib
SI:93 Formula:C19H34O2 CAS:0-00-0 MolWeight:294 RetIndex:2093
CompName:Methyl 9-cis,11-trans-octadecadienoate

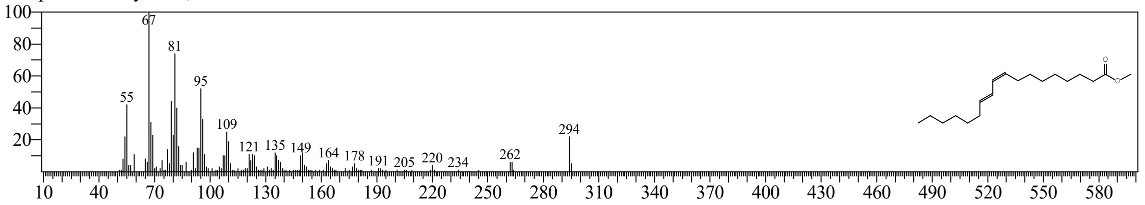

<< Target >>
Line#:2 R.Time:10.550(Scan#:67) MassPeaks:296
RawMode:Averaged 10.542-10.558(66-68) BasePeak:74.05(12156)
BG Mode:Calc. from Peak Group 1 - Event 1

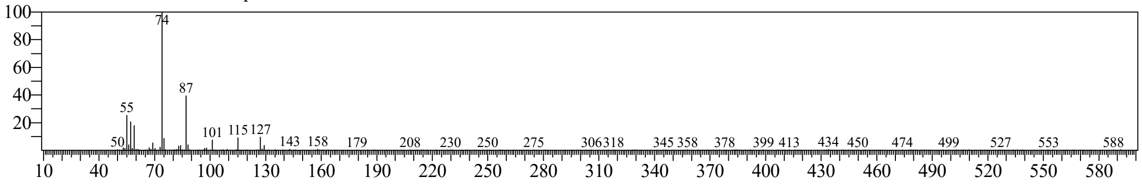

Hit#:1 Entry:12802 Library:NIST17s.lib
SI:96 Formula:C9H18O2 CAS:111-11-5 MolWeight:158 RetIndex:1083
CompName:Octanoic acid, methyl ester $$ Caprylic acid methyl ester $$ Methyl caprylate $$ Methyl n-octanoate $$ Methyl octanoate $$ Uniphat A20 $$ n

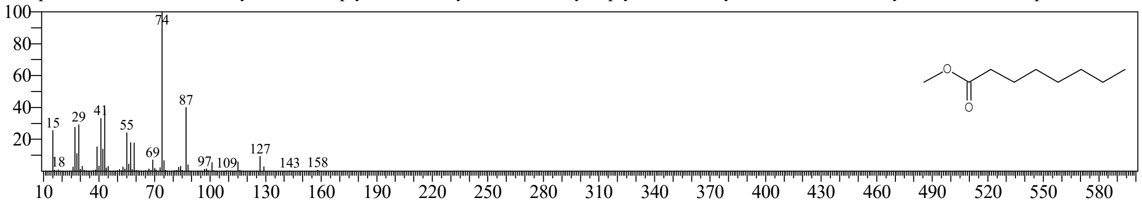

Hit#:2 Entry:11044 Library:NIST14s.lib
SI:96 Formula:C9H18O2 CAS:111-11-5 MolWeight:158 RetIndex:1083
CompName:Octanoic acid, methyl ester $$ Caprylic acid methyl ester $$ Methyl caprylate $$ Methyl n-octanoate $$ Methyl octanoate $$ Uniphat A20 $$ n

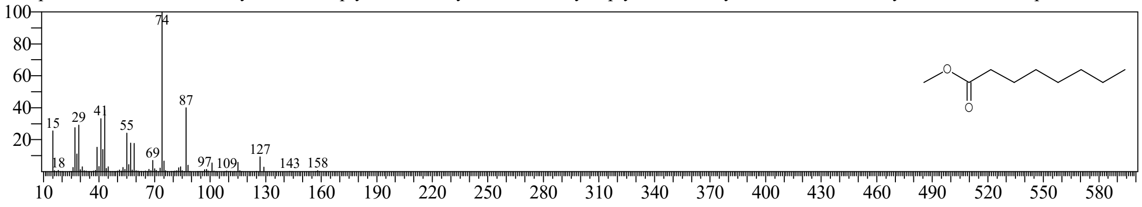

Hit#:3 Entry:11045 Library:NIST14s.lib
SI:95 Formula:C9H18O2 CAS:111-11-5 MolWeight:158 RetIndex:1083
CompName:Octanoic acid, methyl ester $$ Caprylic acid methyl ester $$ Methyl caprylate $$ Methyl n-octanoate $$ Methyl octanoate $$ Uniphat A20 $$ n

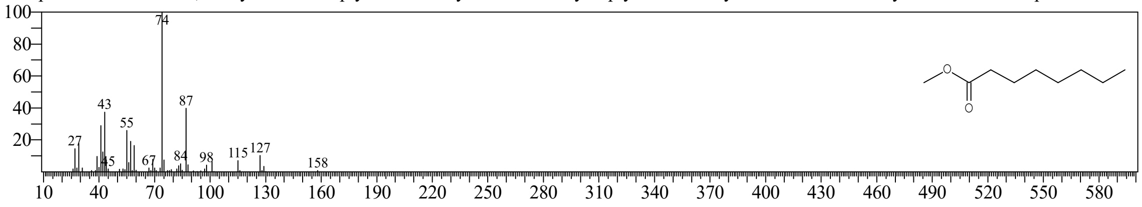

Hit#:4 Entry:12803 Library:NIST17s.lib
SI:95 Formula:C9H18O2 CAS:111-11-5 MolWeight:158 RetIndex:1083
CompName:Octanoic acid, methyl ester $$ Caprylic acid methyl ester $$ Methyl caprylate $$ Methyl n-octanoate $$ Methyl octanoate $$ Uniphat A20 $$ n

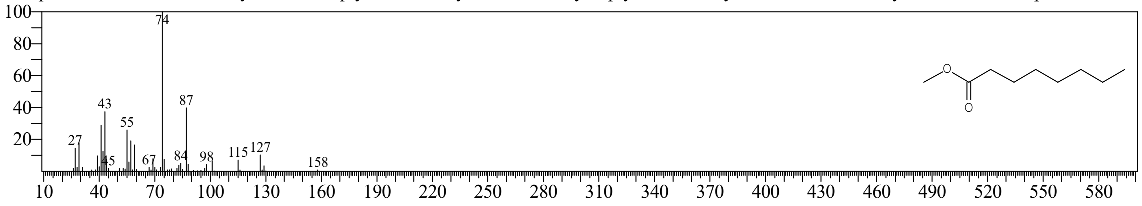

Hit#:5 Entry:12801 Library:NIST17s.lib
SI:95 Formula:C9H18O2 CAS:111-11-5 MolWeight:158 RetIndex:1083
CompName:Octanoic acid, methyl ester $$ Caprylic acid methyl ester $$ Methyl caprylate $$ Methyl n-octanoate $$ Methyl octanoate $$ Uniphat A20 $$ n

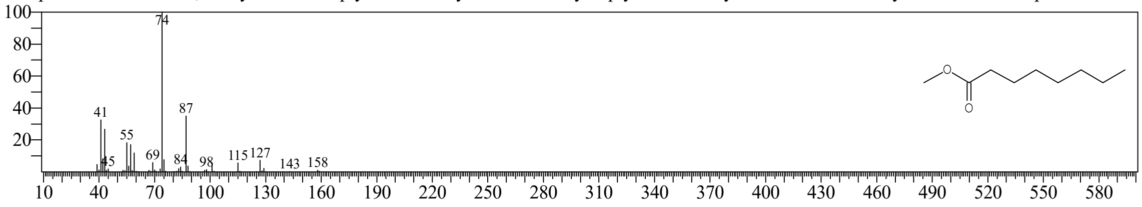

<< Target >>
Line#:3   R.Time:12.950(Scan#:355) MassPeaks:347
RawMode:Averaged 12.942-12.958(354-356) BasePeak:74.05(228328)
BG Mode:Calc. from Peak Group 1 - Event 1

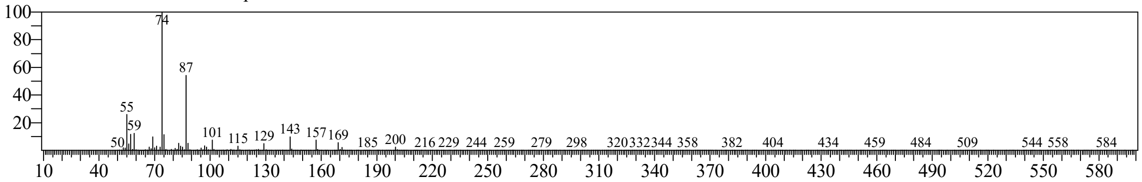

Hit#:1 Entry:49598 Library:NIST17-1.lib
SI:96 Formula:C12H24O2 CAS:1731-86-8 MolWeight:200 RetIndex:1381
CompName:Undecanoic acid, methyl ester $$ Methyl undecanoate $$ Methyl undecylenate $$ n-Undecanoic acid methyl ester $$ Methyl ester of undecanoi

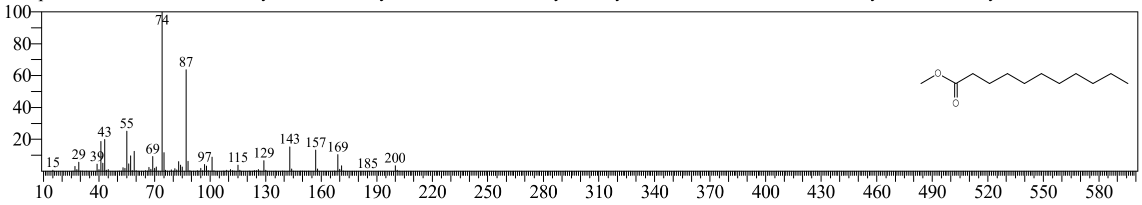

Hit#:2 Entry:46966 Library:NIST14.lib
SI:96 Formula:C12H24O2 CAS:1731-86-8 MolWeight:200 RetIndex:1381
CompName:Undecanoic acid, methyl ester $$ Methyl undecanoate $$ Methyl undecylenate $$ n-Undecanoic acid methyl ester $$ Methyl ester of undecanoi

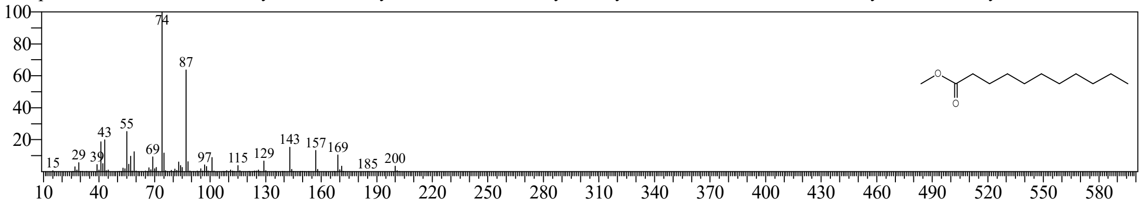

Hit#:3 Entry:21618 Library:NIST17s.lib
SI:95 Formula:C12H24O2 CAS:1731-86-8 MolWeight:200 RetIndex:1381
CompName:Undecanoic acid, methyl ester $$ Methyl undecanoate $$ Methyl undecylenate $$ n-Undecanoic acid methyl ester $$ Methyl ester of undecanoi

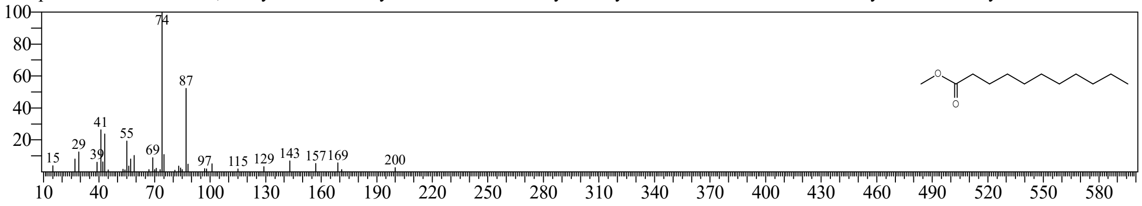

Hit#:4 Entry:21619 Library:NIST17s.lib
SI:94 Formula:C12H24O2 CAS:1731-86-8 MolWeight:200 RetIndex:1381
CompName:Undecanoic acid, methyl ester $$ Methyl undecanoate $$ Methyl undecylenate $$ n-Undecanoic acid methyl ester $$ Methyl ester of undecanoi

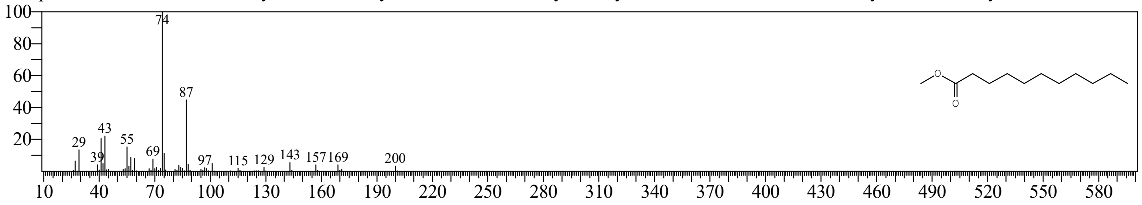

Hit#:5 Entry:18487 Library:NIST14s.lib
SI:94 Formula:C12H24O2 CAS:1731-86-8 MolWeight:200 RetIndex:1381
CompName:Undecanoic acid, methyl ester $$ Methyl undecanoate $$ Methyl undecylenate $$ n-Undecanoic acid methyl ester $$ Methyl ester of undecanoi

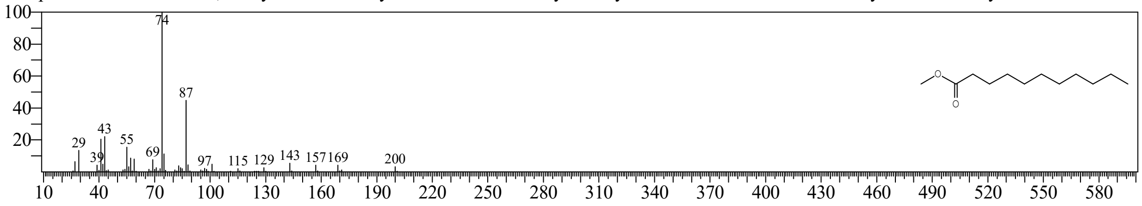

<< Target >>
Line#:4 R.Time:20.883(Scan#:1307) MassPeaks:379
RawMode:Averaged 20.875-20.892(1306-1308) BasePeak:74.05(369984)
BG Mode:Calc. from Peak Group 1 - Event 1

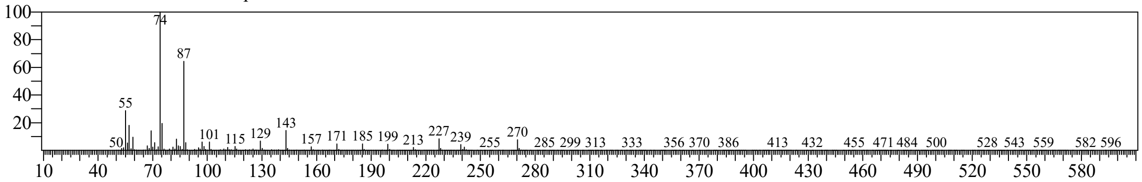

Hit#:1 Entry:113663 Library:NIST17-1.lib
SI:97 Formula:C17H34O2 CAS:112-39-0 MolWeight:270 RetIndex:1878
CompName:Hexadecanoic acid, methyl ester $$ Palmitic acid, methyl ester $$ n-Hexadecanoic acid methyl ester $$ Metholene 2216 $$ Methyl hexadecano

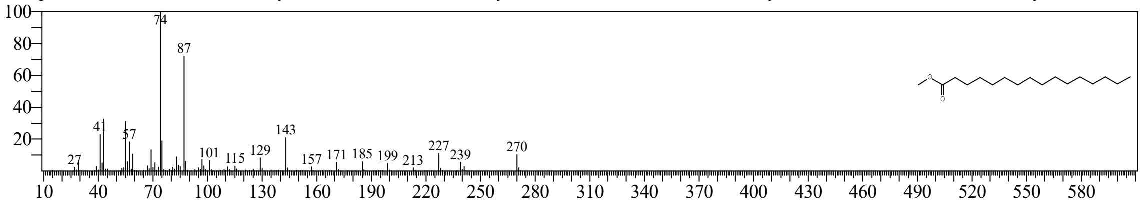

Hit#:2 Entry:104648 Library:NIST14.lib
SI:97 Formula:C17H34O2 CAS:112-39-0 MolWeight:270 RetIndex:1878
CompName:Hexadecanoic acid, methyl ester $$ Palmitic acid, methyl ester $$ n-Hexadecanoic acid methyl ester $$ Metholene 2216 $$ Methyl hexadecano

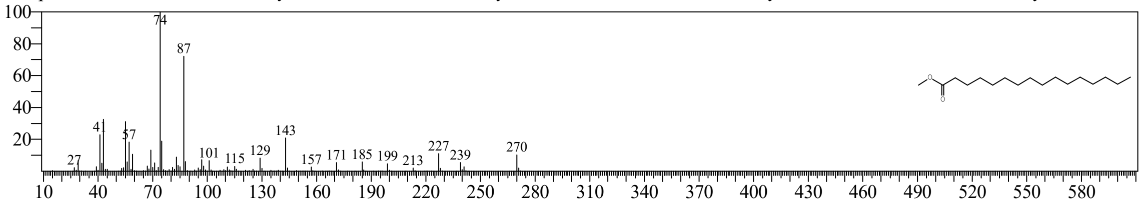

Hit#:3 Entry:26272 Library:NIST14s.lib
SI:97 Formula:C17H34O2 CAS:112-39-0 MolWeight:270 RetIndex:1878
CompName:Hexadecanoic acid, methyl ester $$ Palmitic acid, methyl ester $$ n-Hexadecanoic acid methyl ester $$ Metholene 2216 $$ Methyl hexadecano

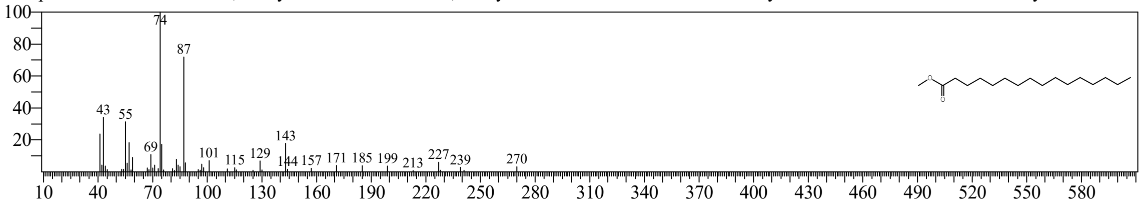

Hit#:4 Entry:30647 Library:NIST17s.lib
SI:97 Formula:C17H34O2 CAS:112-39-0 MolWeight:270 RetIndex:1878
CompName:Hexadecanoic acid, methyl ester $$ Palmitic acid, methyl ester $$ n-Hexadecanoic acid methyl ester $$ Metholene 2216 $$ Methyl hexadecano

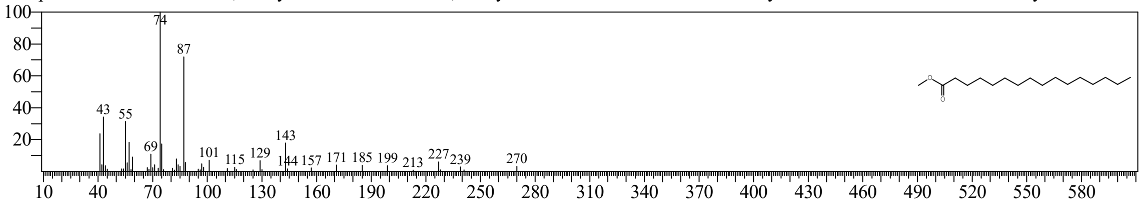

Hit#:5 Entry:26271 Library:NIST14s.lib
SI:96 Formula:C17H34O2 CAS:112-39-0 MolWeight:270 RetIndex:1878
CompName:Hexadecanoic acid, methyl ester $$ Palmitic acid, methyl ester $$ n-Hexadecanoic acid methyl ester $$ Metholene 2216 $$ Methyl hexadecano

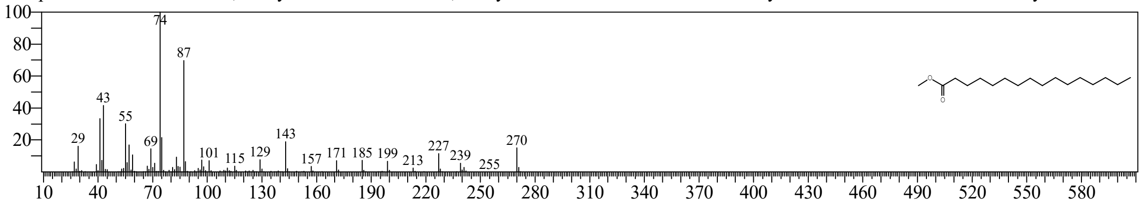

<< Target >>
Line#:5 R.Time:24.942(Scan#:1794) MassPeaks:302
RawMode:Averaged 24.933-24.950(1793-1795) BasePeak:74.05(121683)
BG Mode:Calc. from Peak Group 1 - Event 1

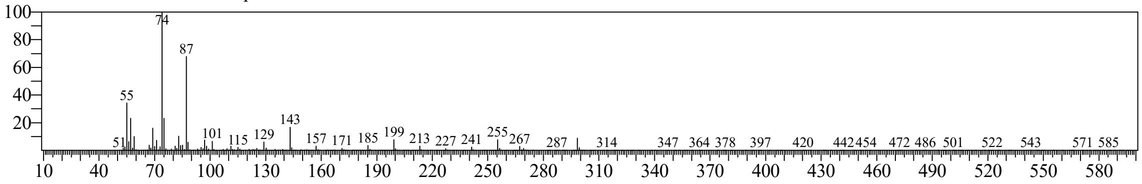

Hit#:1 Entry:32875 Library:NIST17s.lib
SI:97 Formula:C19H38O2 CAS:112-61-8 MolWeight:298 RetIndex:2077
CompName:Methyl stearate $$ Octadecanoic acid, methyl ester $$ Stearic acid, methyl ester $$ n-Octadecanoic acid, methyl ester $$ Kemester 9718 $$ Me

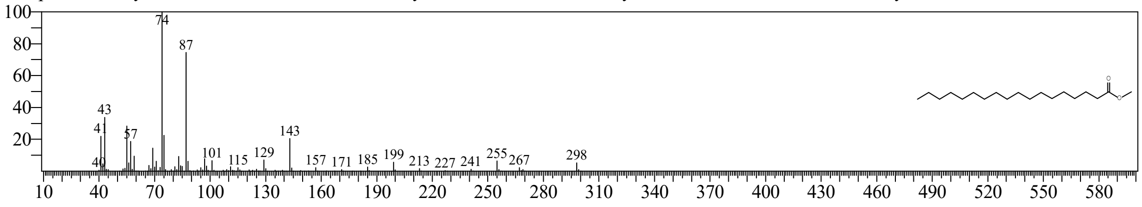

Hit#:2 Entry:28257 Library:NIST14s.lib
SI:97 Formula:C19H38O2 CAS:112-61-8 MolWeight:298 RetIndex:2077
CompName:Methyl stearate $$ Octadecanoic acid, methyl ester $$ Stearic acid, methyl ester $$ n-Octadecanoic acid, methyl ester $$ Kemester 9718 $$ Me

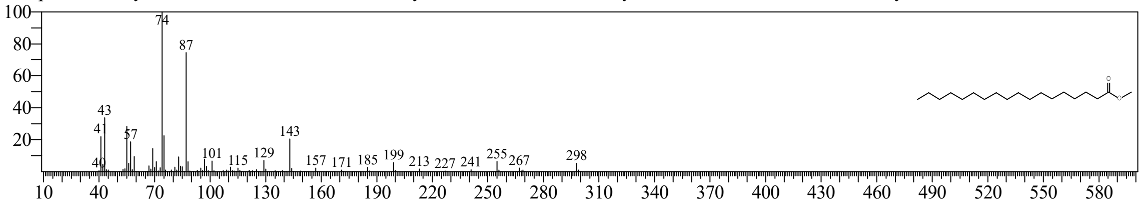

Hit#:3 Entry:129694 Library:NIST14.lib
SI:96 Formula:C19H38O2 CAS:112-61-8 MolWeight:298 RetIndex:2077
CompName:Methyl stearate $$ Octadecanoic acid, methyl ester $$ Stearic acid, methyl ester $$ n-Octadecanoic acid, methyl ester $$ Kemester 9718 $$ Me

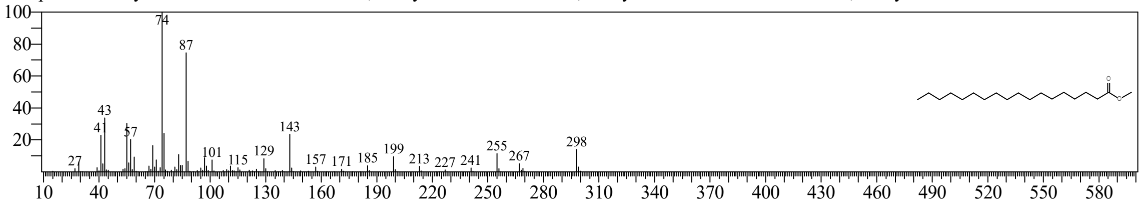

Hit#:4 Entry:141978 Library:NIST17-1.lib
SI:96 Formula:C19H38O2 CAS:112-61-8 MolWeight:298 RetIndex:2077
CompName:Methyl stearate $$ Octadecanoic acid, methyl ester $$ Stearic acid, methyl ester $$ n-Octadecanoic acid, methyl ester $$ Kemester 9718 $$ Me

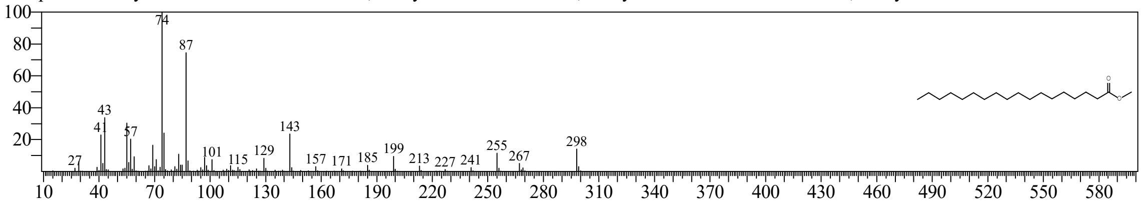

Hit#:5 Entry:28255 Library:NIST14s.lib
SI:96 Formula:C19H38O2 CAS:112-61-8 MolWeight:298 RetIndex:2077
CompName:Methyl stearate $$ Octadecanoic acid, methyl ester $$ Stearic acid, methyl ester $$ n-Octadecanoic acid, methyl ester $$ Kemester 9718 $$ Me

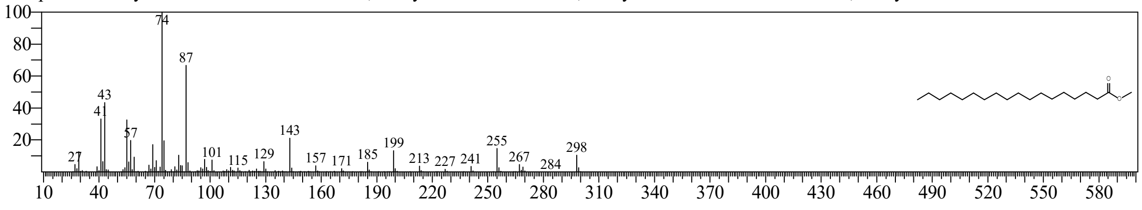

<< Target >>
Line#:6 R.Time:26.317(Scan#:1959) MassPeaks:383
RawMode:Averaged 26.308-26.325(1958-1960) BasePeak:55.05(184582)
BG Mode:Calc. from Peak Group 1 - Event 1

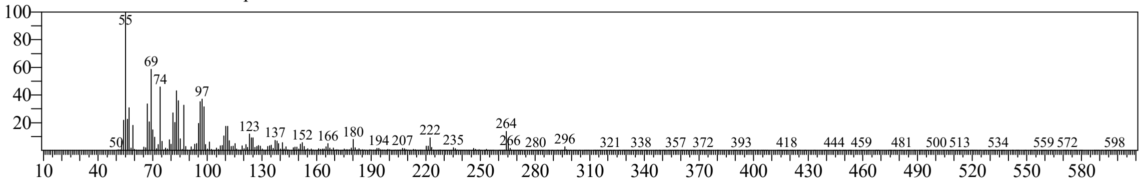

Hit#:1 Entry:28139 Library:NIST14s.lib
SI:96 Formula:C19H36O2 CAS:52380-33-3 MolWeight:296 RetIndex:2085
CompName:11-Octadecenoic acid, methyl ester $$ Methyl 11-octadecenoate $$ Octadec-11-enoic acid, methyl ester $$ Methyl (11E)-11-octadecenoate # $$

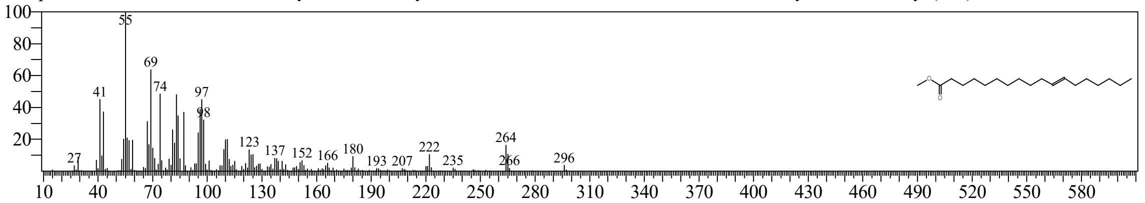

Hit#:2 Entry:32748 Library:NIST17s.lib
SI:96 Formula:C19H36O2 CAS:52380-33-3 MolWeight:296 RetIndex:2085
CompName:11-Octadecenoic acid, methyl ester $$ Methyl 11-octadecenoate $$ Octadec-11-enoic acid, methyl ester $$ Methyl (11E)-11-octadecenoate # $$

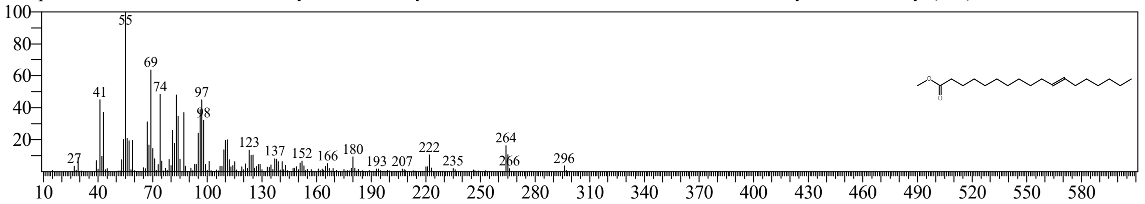

Hit#:3 Entry:139636 Library:NIST17-1.lib
SI:95 Formula:C19H36O2 CAS:13481-95-3 MolWeight:296 RetIndex:2085
CompName:10-Octadecenoic acid, methyl ester $$ Methyl 10-octadecenoate $$

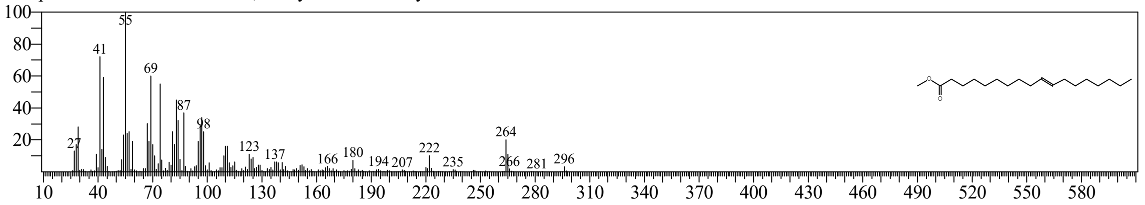

Hit#:4 Entry:127650 Library:NIST14.lib
SI:95 Formula:C19H36O2 CAS:13481-95-3 MolWeight:296 RetIndex:2085
CompName:10-Octadecenoic acid, methyl ester $$ Methyl 10-octadecenoate $$

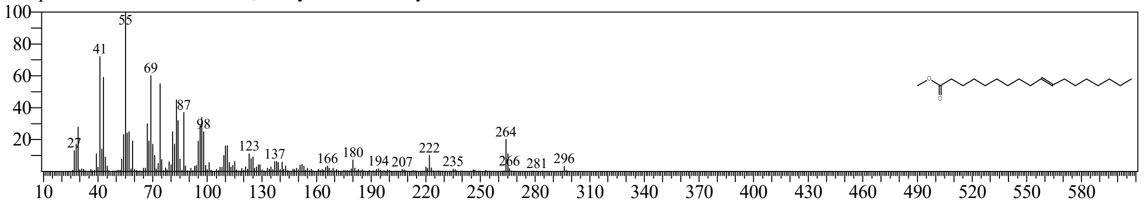

Hit#:5 Entry:28134 Library:NIST14s.lib
SI:95 Formula:C19H36O2 CAS:1937-62-8 MolWeight:296 RetIndex:2085
CompName:9-Octadecenoic acid, methyl ester, (E)- $$ Elaidic acid, methyl ester $$ Methyl elaidate $$ Methyl trans-9-octadecenoate $$ (E)-9-Octadecenoic

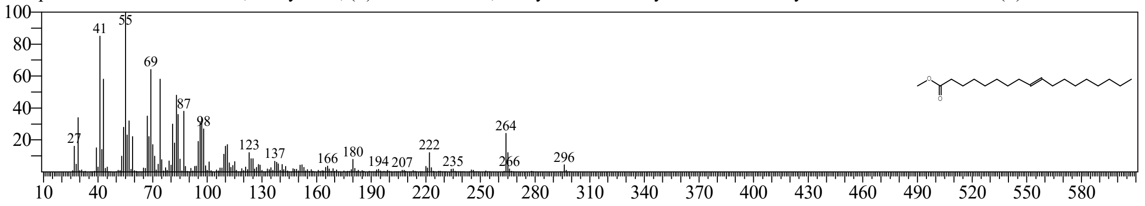

<< Target >>
Line#:7 R.Time:26.450(Scan#:1975) MassPeaks:364
RawMode:Averaged 26.442-26.458(1974-1976) BasePeak:55.10(10469)
BG Mode:Calc. from Peak Group 1 - Event 1

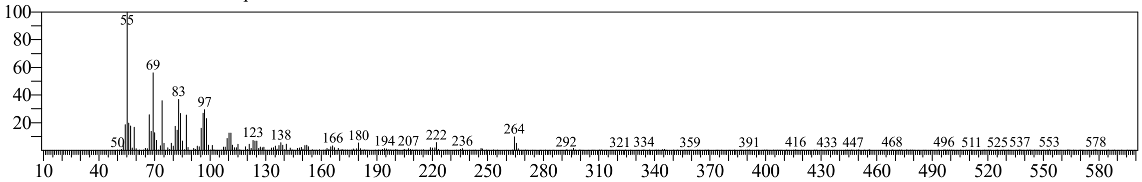

Hit#:1 Entry:127658 Library:NIST14.lib
SI:96 Formula:C19H36O2 CAS:0-00-0 MolWeight:296 RetIndex:2085
CompName:trans-13-Octadecenoic acid, methyl ester

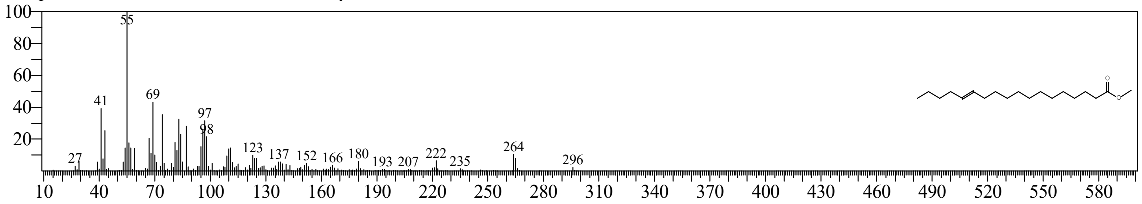

Hit#:2 Entry:139644 Library:NIST17-1.lib
SI:96 Formula:C19H36O2 CAS:0-00-0 MolWeight:296 RetIndex:2085
CompName:trans-13-Octadecenoic acid, methyl ester

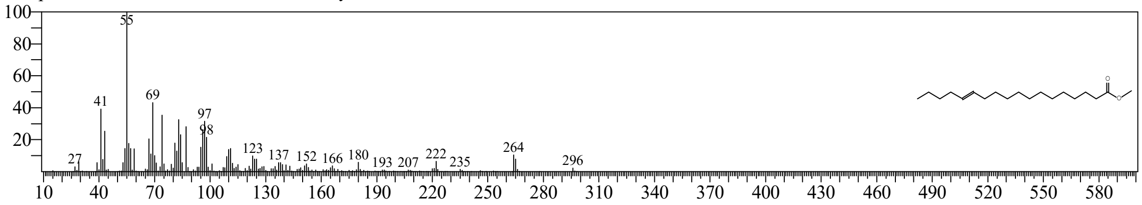

Hit#:3 Entry:28140 Library:NIST14s.lib
SI:95 Formula:C19H36O2 CAS:56554-47-3 MolWeight:296 RetIndex:2085
CompName:13-Octadecenoic acid, methyl ester $$ Methyl (13E)-13-octadecenoate # $$

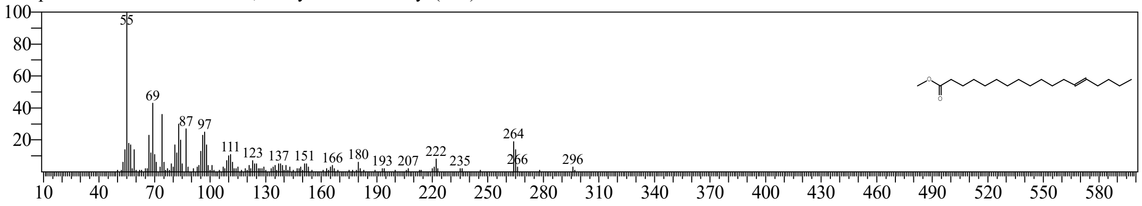

Hit#:4 Entry:32749 Library:NIST17s.lib
SI:95 Formula:C19H36O2 CAS:56554-47-3 MolWeight:296 RetIndex:2085
CompName:13-Octadecenoic acid, methyl ester $$ Methyl (13E)-13-octadecenoate # $$

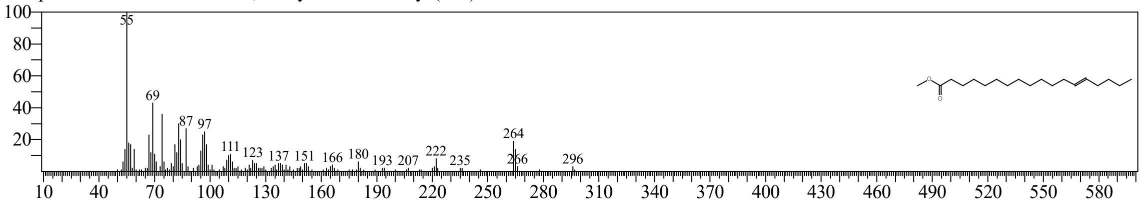

Hit#:5 Entry:127652 Library:NIST14.lib
SI:94 Formula:C19H36O2 CAS:56554-47-3 MolWeight:296 RetIndex:2085
CompName:13-Octadecenoic acid, methyl ester $$ Methyl (13E)-13-octadecenoate # $$

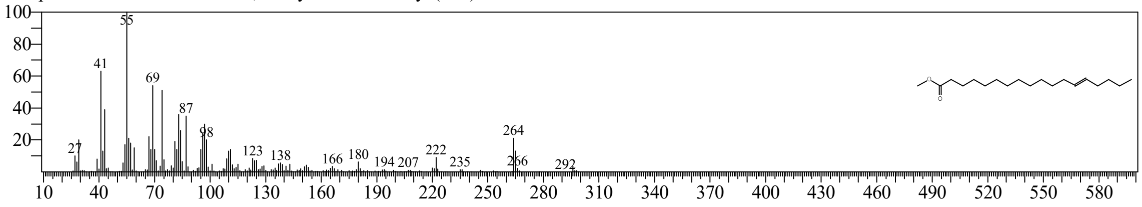

<< Target >>
Line#:8 R.Time:28.317(Scan#:2199) MassPeaks:371
RawMode:Averaged 28.308-28.325(2198-2200) BasePeak:67.10(89520)
BG Mode:Calc. from Peak Group 1 - Event 1

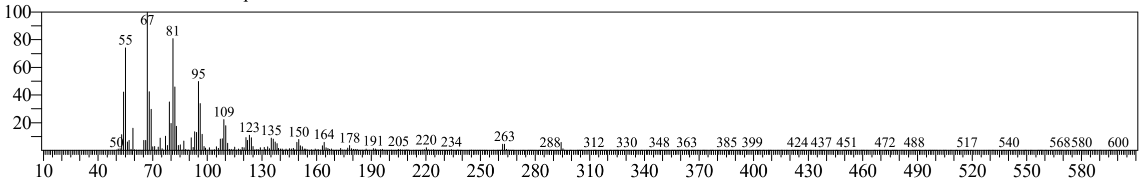

Hit#:1 Entry:137663 Library:NIST17-1.lib
SI:97 Formula:C19H34O2 CAS:2462-85-3 MolWeight:294 RetIndex:2093
CompName:9,12-Octadecadienoic acid, methyl ester $$ 9,12-Octadecenoic acid, methyl ester $$ Methyl octadeca-9,12-dienoate $$ Methyl 9,12-octadecadi

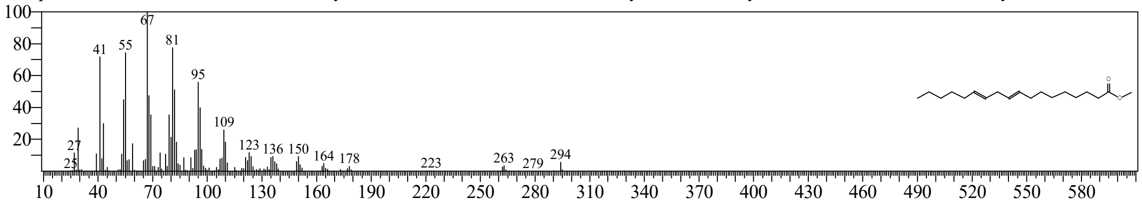

Hit#:2 Entry:125931 Library:NIST14.lib
SI:97 Formula:C19H34O2 CAS:2462-85-3 MolWeight:294 RetIndex:2093
CompName:9,12-Octadecadienoic acid, methyl ester $$ 9,12-Octadecenoic acid, methyl ester $$ Methyl octadeca-9,12-dienoate $$ Methyl 9,12-octadecadi

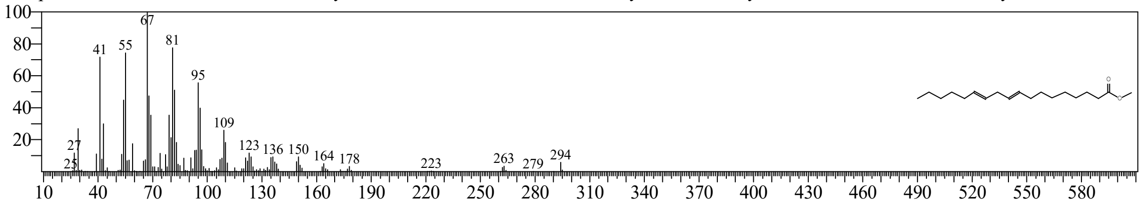

Hit#:3 Entry:32588 Library:NIST17s.lib
SI:96 Formula:C19H34O2 CAS:112-63-0 MolWeight:294 RetIndex:2093
CompName:9,12-Octadecadienoic acid (Z,Z)-, methyl ester $$ Linoleic acid, methyl ester $$ Methyl cis,cis-9,12-octadecadienoate $$ Methyl linoleate $$ M

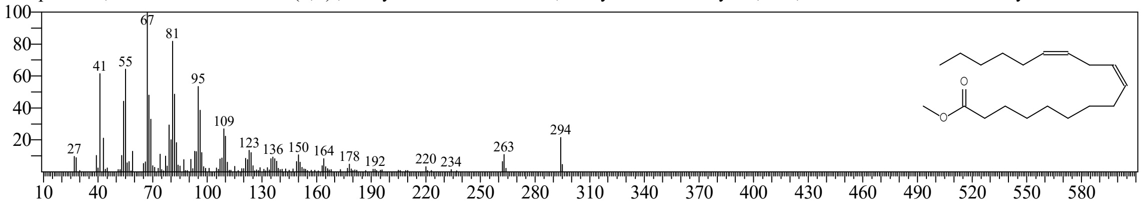

Hit#:4 Entry:27999 Library:NIST14s.lib
SI:96 Formula:C19H34O2 CAS:112-63-0 MolWeight:294 RetIndex:2093
CompName:9,12-Octadecadienoic acid (Z,Z)-, methyl ester $$ Linoleic acid, methyl ester $$ Methyl cis,cis-9,12-octadecadienoate $$ Methyl linoleate $$ M

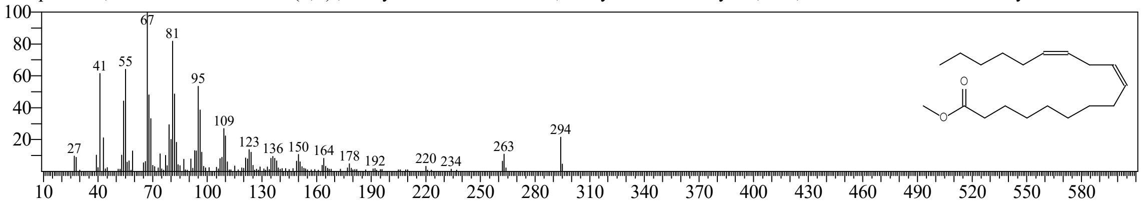

Hit#:5 Entry:32589 Library:NIST17s.lib
SI:96 Formula:C19H34O2 CAS:112-63-0 MolWeight:294 RetIndex:2093
CompName:9,12-Octadecadienoic acid (Z,Z)-, methyl ester $$ Linoleic acid, methyl ester $$ Methyl cis,cis-9,12-octadecadienoate $$ Methyl linoleate $$ M

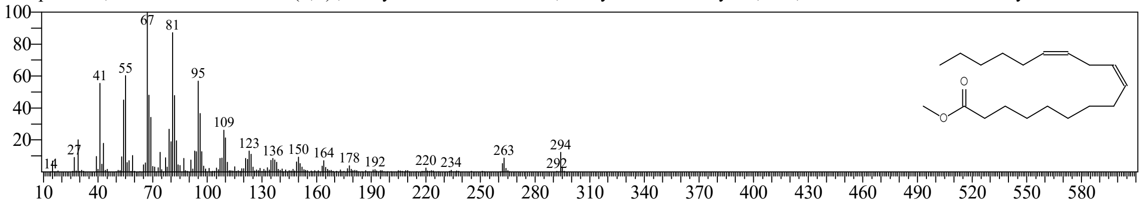

<< Target >>
Line#:9 R.Time:29.350(Scan#:2323) MassPeaks:332
RawMode:Averaged 29.342-29.358(2322-2324) BasePeak:74.05(7567)
BG Mode:Calc. from Peak Group 1 - Event 1

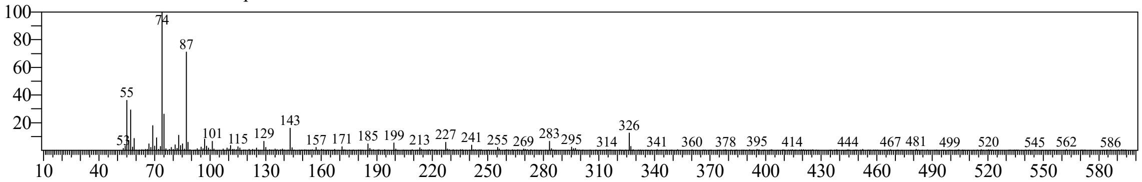

Hit#:1 Entry:34726 Library:NIST17s.lib
SI:97 Formula:C21H42O2 CAS:1120-28-1 MolWeight:326 RetIndex:2276
CompName:Eicosanoic acid, methyl ester $$ Methyl arachisate $$ Methyl eicosanoate $$ Arachidic acid methyl ester $$ Kemester 2050 $$ Methyl aracidat

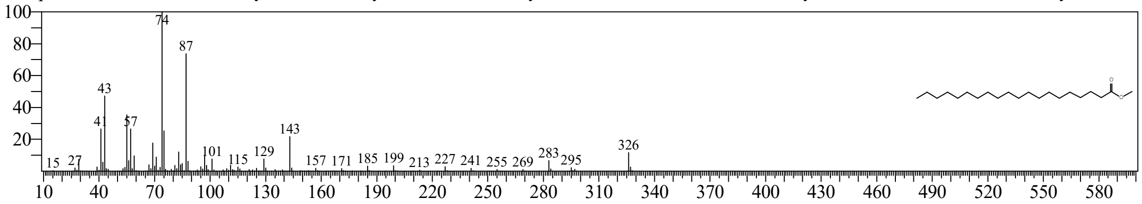

Hit#:2 Entry:170238 Library:NIST17-1.lib
SI:96 Formula:C21H42O2 CAS:0-00-0 MolWeight:326 RetIndex:2212
CompName:Methyl 18-methylnonadecanoate

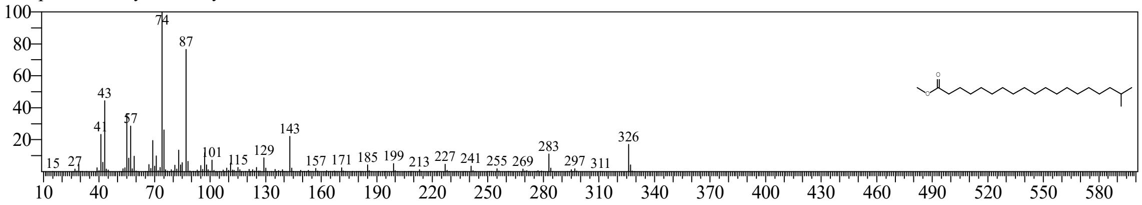

Hit#:3 Entry:154704 Library:NIST14.lib
SI:96 Formula:C21H42O2 CAS:0-00-0 MolWeight:326 RetIndex:2212
CompName:Methyl 18-methylnonadecanoate

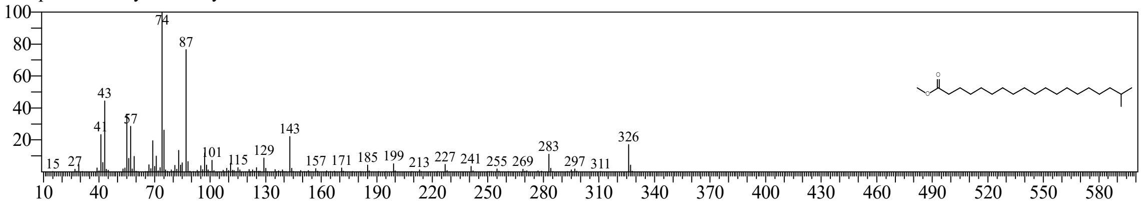

Hit#:4 Entry:34724 Library:NIST17s.lib
SI:95 Formula:C21H42O2 CAS:1120-28-1 MolWeight:326 RetIndex:2276
CompName:Eicosanoic acid, methyl ester $$ Methyl arachisate $$ Methyl eicosanoate $$ Arachidic acid methyl ester $$ Kemester 2050 $$ Methyl aracidat

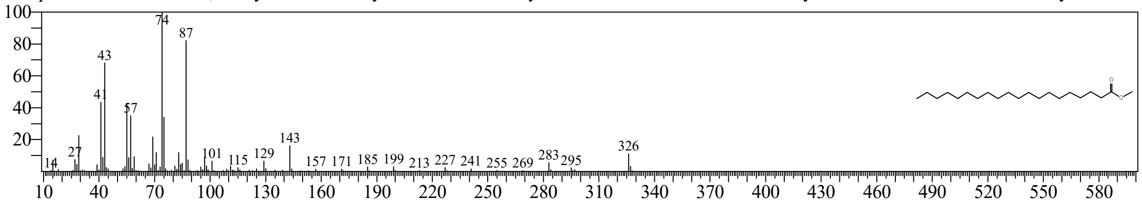

Hit#:5 Entry:29931 Library:NIST14s.lib
SI:95 Formula:C21H42O2 CAS:1120-28-1 MolWeight:326 RetIndex:2276
CompName:Eicosanoic acid, methyl ester $$ Methyl arachisate $$ Methyl eicosanoate $$ Arachidic acid methyl ester $$ Kemester 2050 $$ Methyl aracidat

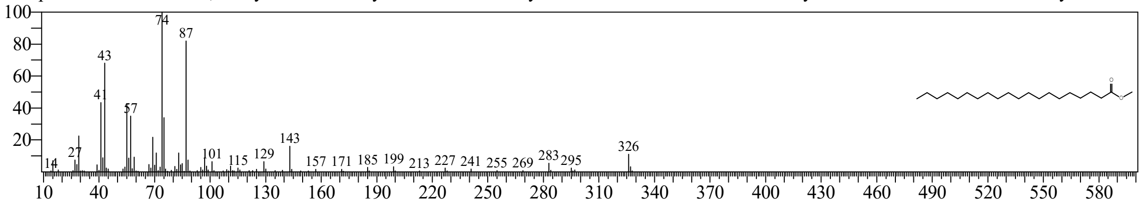

<< Target >>
Line#:10 R.Time:30.650(Scan#:2479) MassPeaks:357
RawMode:Averaged 30.642-30.658(2478-2480) BasePeak:79.10(2050)
BG Mode:Calc. from Peak Group 1 - Event 1

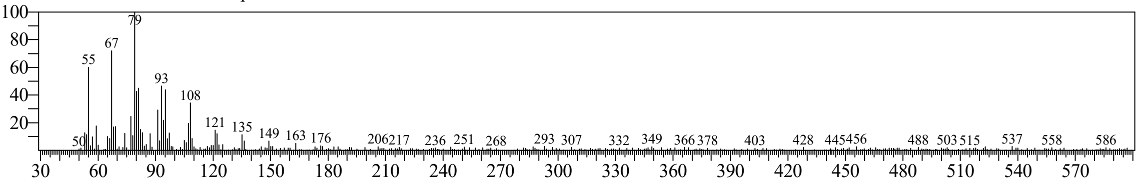

Hit#:1 Entry:32438 Library:NIST17s.lib
SI:95 Formula:C19H32O2 CAS:301-00-8 MolWeight:292 RetIndex:2101
CompName:9,12,15-Octadecatrienoic acid, methyl ester, (Z,Z,Z)- $$ Linolenic acid, methyl ester $$ Methyl all-cis-9,12,15-octadecatrienoate $$ Methyl lino

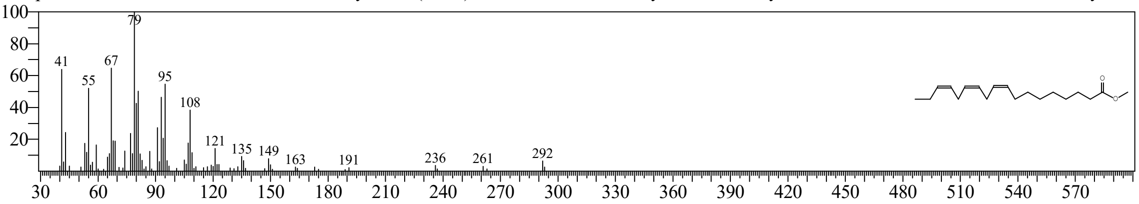

Hit#:2 Entry:32437 Library:NIST17s.lib
SI:94 Formula:C19H32O2 CAS:301-00-8 MolWeight:292 RetIndex:2101
CompName:9,12,15-Octadecatrienoic acid, methyl ester, (Z,Z,Z)- $$ Linolenic acid, methyl ester $$ Methyl all-cis-9,12,15-octadecatrienoate $$ Methyl lino

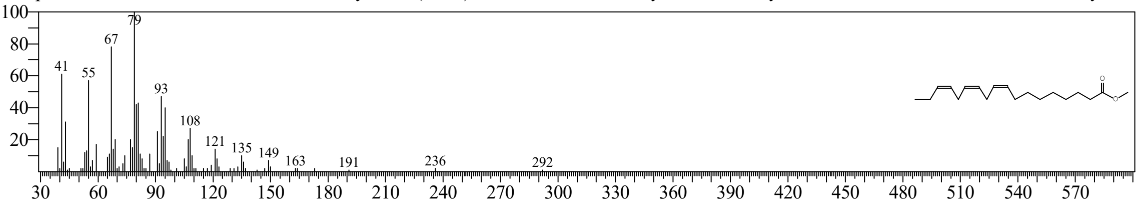

Hit#:3 Entry:27872 Library:NIST14s.lib
SI:94 Formula:C19H32O2 CAS:301-00-8 MolWeight:292 RetIndex:2101
CompName:9,12,15-Octadecatrienoic acid, methyl ester, (Z,Z,Z)- $$ Linolenic acid, methyl ester $$ Methyl all-cis-9,12,15-octadecatrienoate $$ Methyl lino

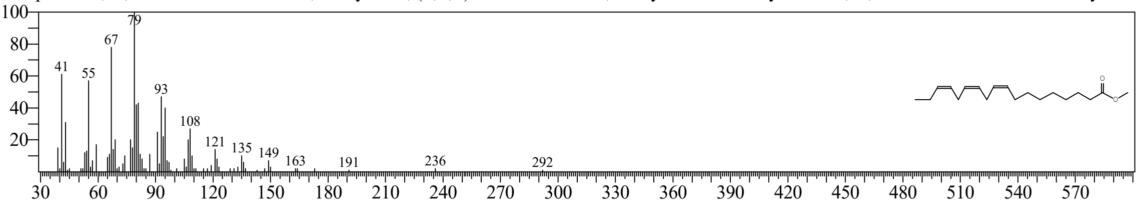

Hit#:4 Entry:111654 Library:NIST14.lib
SI:93 Formula:C18H30O2 CAS:0-00-0 MolWeight:278 RetIndex:2002
CompName:Methyl 8,11,14-heptadecatrienoate

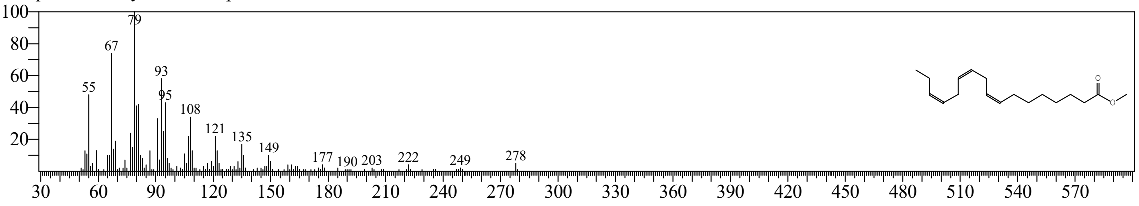

Hit#:5 Entry:121536 Library:NIST17-1.lib
SI:93 Formula:C18H30O2 CAS:0-00-0 MolWeight:278 RetIndex:2002
CompName:Methyl 8,11,14-heptadecatrienoate

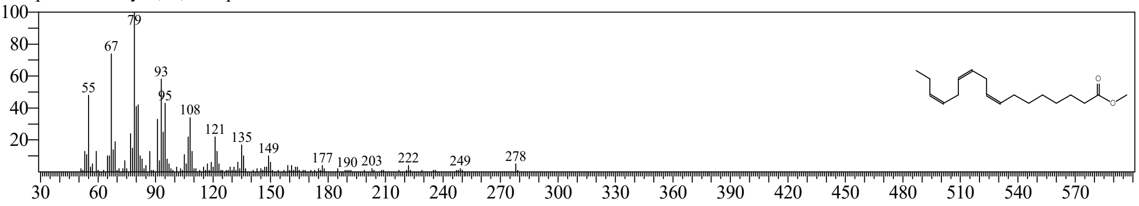

<< Target >>
Line#:11   R.Time:31.008(Scan#:2522) MassPeaks:304
RawMode:Averaged 31.000-31.017(2521-2523) BasePeak:67.05(4376)
BG Mode:Calc. from Peak Group 1 - Event 1

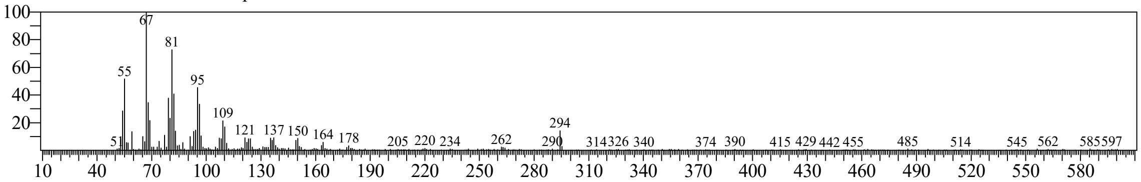

Hit#:1 Entry:32587 Library:NIST17s.lib
SI:96 Formula:C19H34O2 CAS:13038-47-6 MolWeight:294 RetIndex:2093
CompName:9,11-Octadecadienoic acid, methyl ester, (E,E)- $$ Methyl trans-9,trans-11-octadecadienoate $$ Methyl trans,trans-9,11-octadecadienoate $$ M

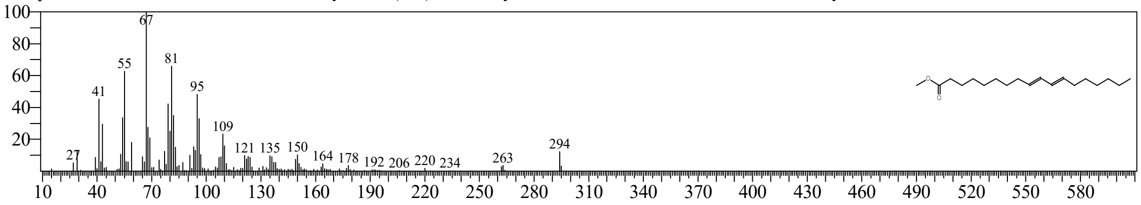

Hit#:2 Entry:125932 Library:NIST14.lib
SI:95 Formula:C19H34O2 CAS:0-00-0 MolWeight:294 RetIndex:2093
CompName:Methyl 9-cis,11-trans-octadecadienoate

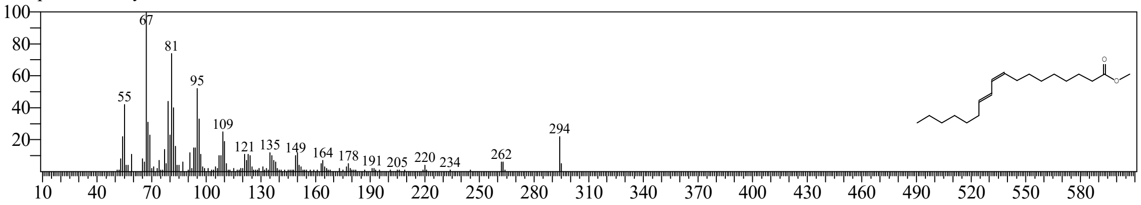

Hit#:3 Entry:137666 Library:NIST17-1.lib
SI:95 Formula:C19H34O2 CAS:13058-52-1 MolWeight:294 RetIndex:2093
CompName:Methyl 9-cis,11-trans-octadecadienoate

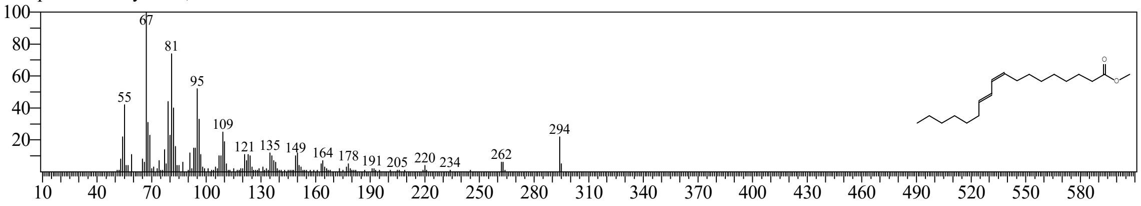

Hit#:4 Entry:137664 Library:NIST17-1.lib
SI:95 Formula:C19H34O2 CAS:0-00-0 MolWeight:294 RetIndex:2093
CompName:Methyl 10-trans,12-cis-octadecadienoate

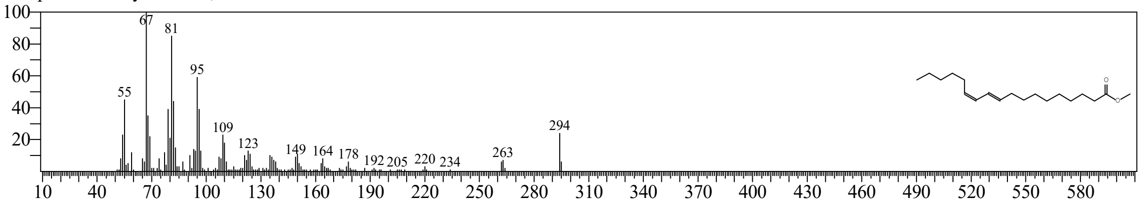

Hit#:5 Entry:125933 Library:NIST14.lib
SI:95 Formula:C19H34O2 CAS:0-00-0 MolWeight:294 RetIndex:2093
CompName:Methyl 10-trans,12-cis-octadecadienoate

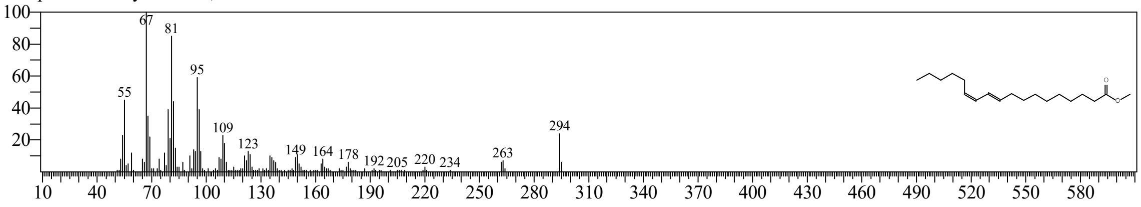

<< Target >>
Line#:12   R.Time:31.325(Scan#:2560) MassPeaks:336
RawMode:Averaged 31.317-31.333(2559-2561) BasePeak:67.05(4400)
BG Mode:Calc. from Peak Group 1 - Event 1

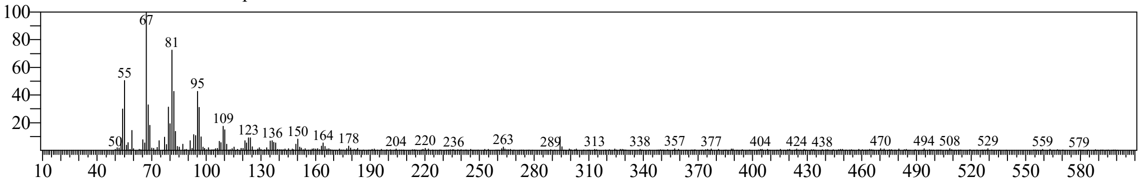

Hit#:1 Entry:27998 Library:NIST14s.lib
SI:95 Formula:C19H34O2 CAS:56554-61-1 MolWeight:294 RetIndex:2093
CompName:11,14-Octadecadienoic acid, methyl ester $$ Methyl (11E,14E)-11,14-octadecadienoate # $$

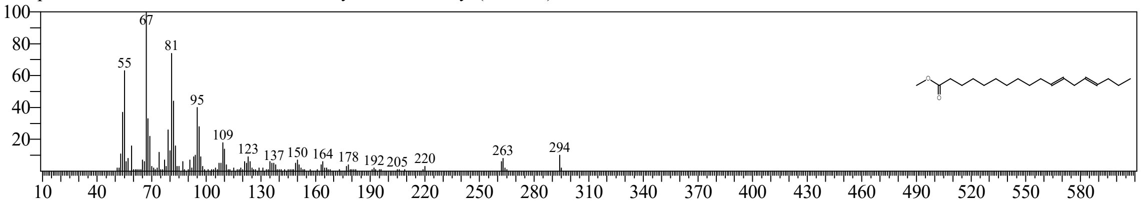

Hit#:2 Entry:32586 Library:NIST17s.lib
SI:95 Formula:C19H34O2 CAS:56554-61-1 MolWeight:294 RetIndex:2093
CompName:11,14-Octadecadienoic acid, methyl ester $$ Methyl (11E,14E)-11,14-octadecadienoate # $$

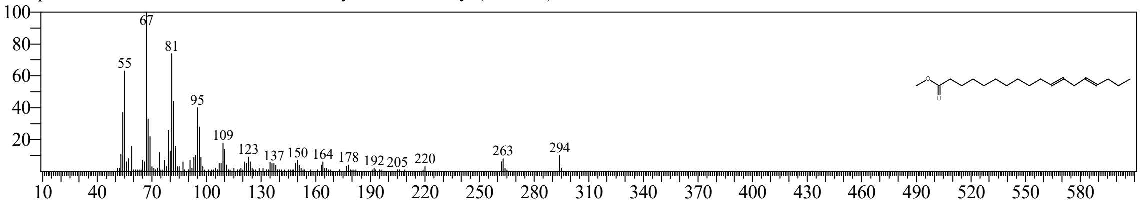

Hit#:3 Entry:32587 Library:NIST17s.lib
SI:95 Formula:C19H34O2 CAS:13038-47-6 MolWeight:294 RetIndex:2093
CompName:9,11-Octadecadienoic acid, methyl ester, (E,E)- $$ Methyl trans-9,trans-11-octadecadienoate $$ Methyl trans,trans-9,11-octadecadienoate $$ M

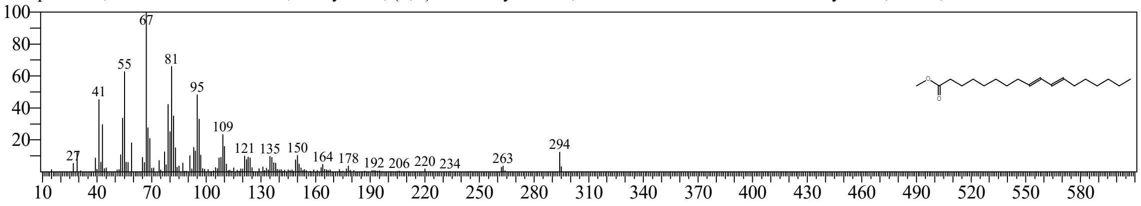

Hit#:4 Entry:27995 Library:NIST14s.lib
SI:94 Formula:C19H34O2 CAS:2566-97-4 MolWeight:294 RetIndex:2093
CompName:9,12-Octadecadienoic acid, methyl ester, (E,E)- $$ Linolelaidic acid, methyl ester $$ Methyl linolelaidate $$ Methyl trans,trans-9,12-octadecadi

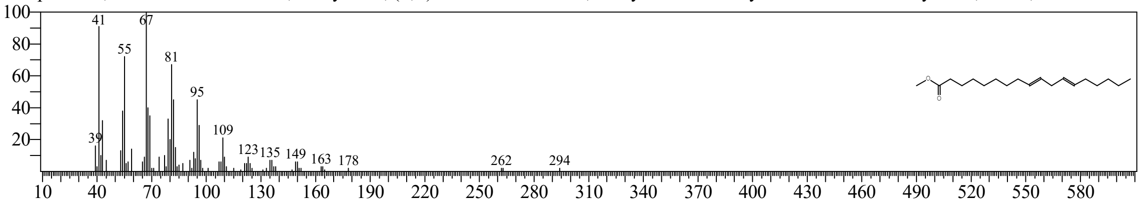

Hit#:5 Entry:32583 Library:NIST17s.lib
SI:94 Formula:C19H34O2 CAS:2566-97-4 MolWeight:294 RetIndex:2093
CompName:9,12-Octadecadienoic acid, methyl ester, (E,E)- $$ Linolelaidic acid, methyl ester $$ Methyl linolelaidate $$ Methyl trans,trans-9,12-octadecadi

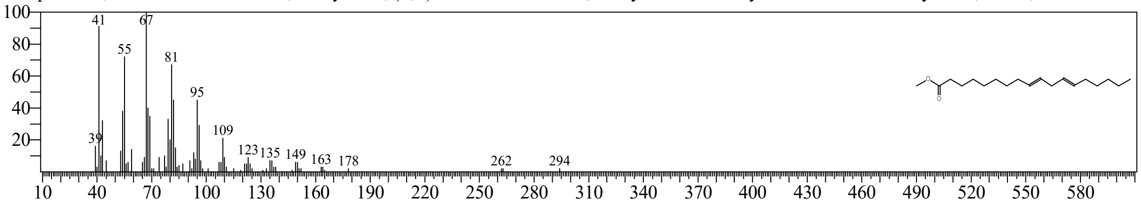

<< Target >>
Line#:13 R.Time:32.000(Scan#:2641) MassPeaks:305
RawMode:Averaged 31.992-32.008(2640-2642) BasePeak:67.05(2223)
BG Mode:Calc. from Peak Group 1 - Event 1

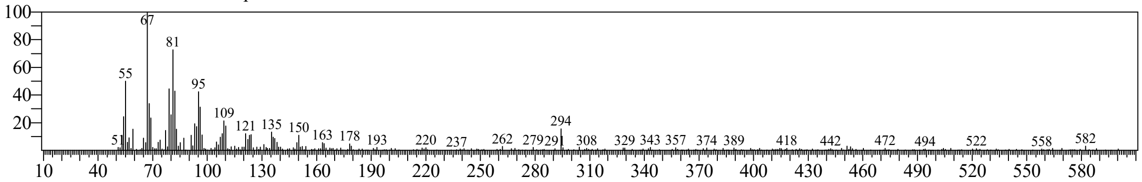

Hit#:1 Entry:137666 Library:NIST17-1.lib
SI:94 Formula:C19H34O2 CAS:13058-52-1 MolWeight:294 RetIndex:2093
CompName:Methyl 9-cis,11-trans-octadecadienoate

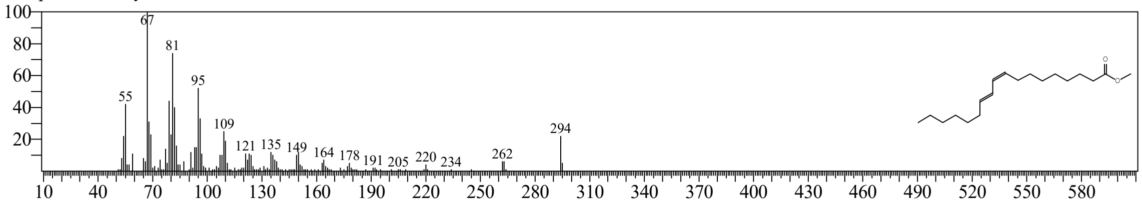

Hit#:2 Entry:32587 Library:NIST17s.lib
SI:94 Formula:C19H34O2 CAS:13038-47-6 MolWeight:294 RetIndex:2093
CompName:9,11-Octadecadienoic acid, methyl ester, (E,E)- $$ Methyl trans-9,trans-11-octadecadienoate $$ Methyl trans,trans-9,11-octadecadienoate $$ M

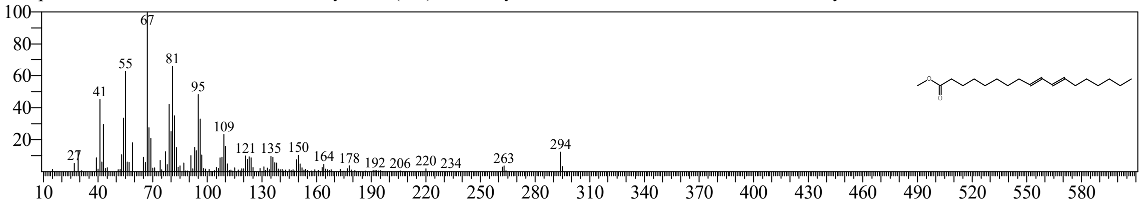

Hit#:3 Entry:125932 Library:NIST14.lib
SI:94 Formula:C19H34O2 CAS:0-00-0 MolWeight:294 RetIndex:2093
CompName:Methyl 9-cis,11-trans-octadecadienoate

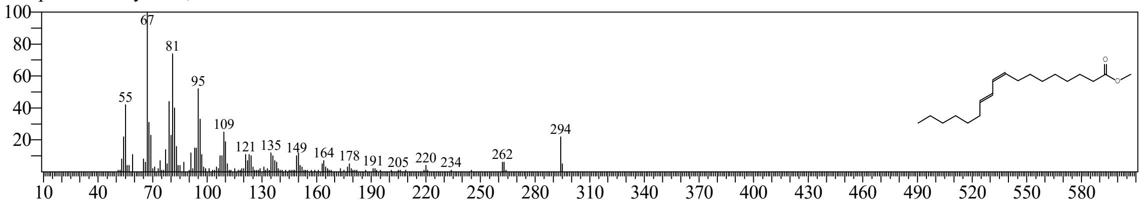

Hit#:4 Entry:125933 Library:NIST14.lib
SI:93 Formula:C19H34O2 CAS:0-00-0 MolWeight:294 RetIndex:2093
CompName:Methyl 10-trans,12-cis-octadecadienoate

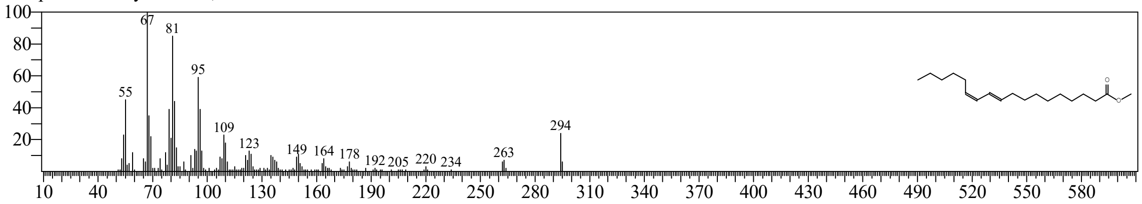

Hit#:5 Entry:137664 Library:NIST17-1.lib
SI:93 Formula:C19H34O2 CAS:0-00-0 MolWeight:294 RetIndex:2093
CompName:Methyl 10-trans,12-cis-octadecadienoate

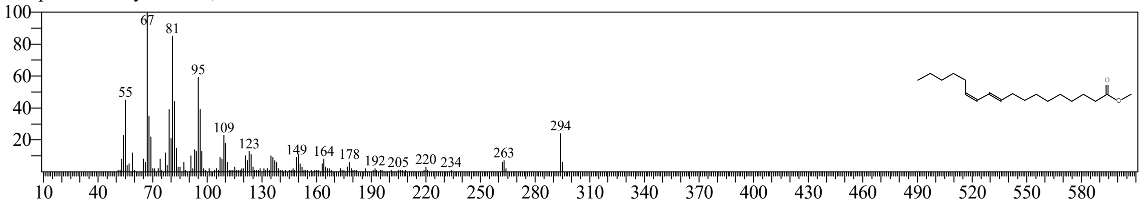

<< Target >>
Line#:14 R.Time:32.433(Scan#:2693) MassPeaks:290
RawMode:Averaged 32.425-32.442(2692-2694) BasePeak:71.05(4256)
BG Mode:Calc. from Peak Group 1 - Event 1

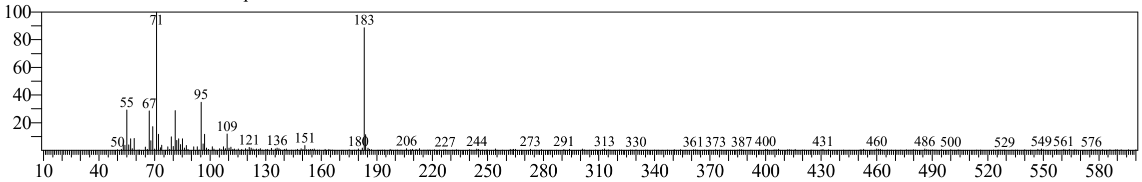

Hit#:1 Entry:72357 Library:NIST17-1.lib
SI:78 Formula:C14H26O2 CAS:2146-71-6 MolWeight:226 RetIndex:1570
CompName:Dodecanoic acid, ethenyl ester $$ Lauric acid, vinyl ester $$ Vinyl laurate $$ Vinyl dodecanoate $$

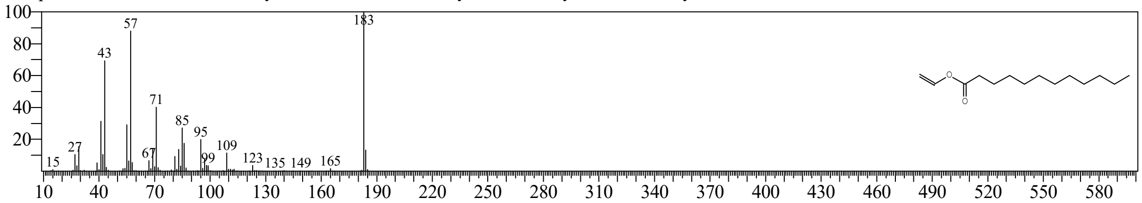

Hit#:2 Entry:67683 Library:NIST14.lib
SI:78 Formula:C14H26O2 CAS:2146-71-6 MolWeight:226 RetIndex:1570
CompName:Dodecanoic acid, ethenyl ester $$ Lauric acid, vinyl ester $$ Vinyl laurate $$ Vinyl dodecanoate $$

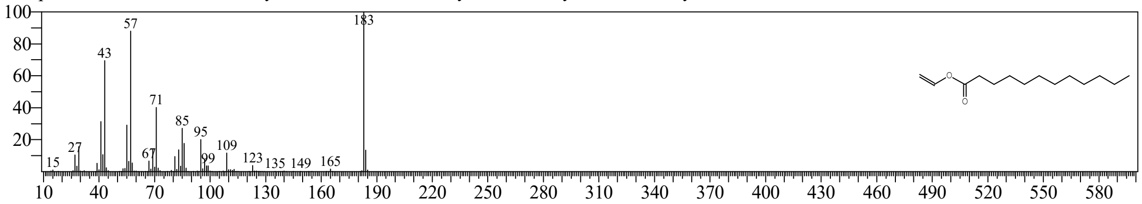

Hit#:3 Entry:60448 Library:NIST17-1.lib
SI:77 Formula:C13H27NO CAS:832-29-1 MolWeight:213 RetIndex:1893
CompName:Cyclododecanol, 1-aminomethyl- $$ 1-(Aminomethyl)cyclododecanol # $$

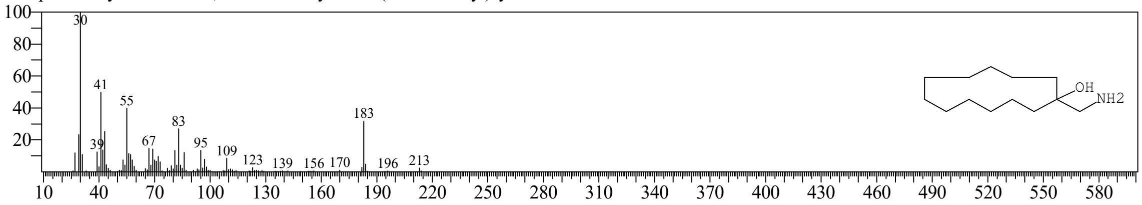

Hit#:4 Entry:56932 Library:NIST14.lib
SI:77 Formula:C13H27NO CAS:832-29-1 MolWeight:213 RetIndex:1893
CompName:Cyclododecanol, 1-aminomethyl- $$ 1-(Aminomethyl)cyclododecanol # $$

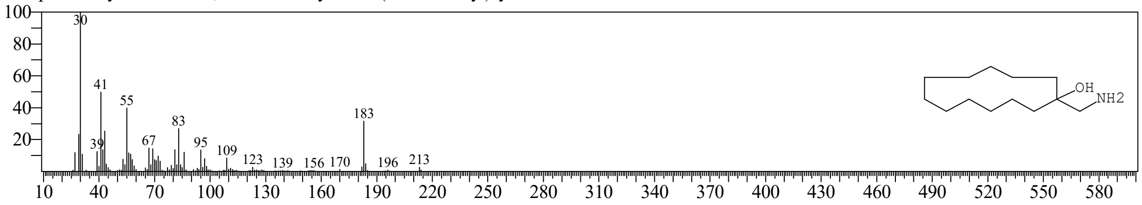

Hit#:5 Entry:25920 Library:NIST17s.lib
SI:77 Formula:C14H26O2 CAS:2146-71-6 MolWeight:226 RetIndex:1570
CompName:Dodecanoic acid, ethenyl ester $$ Lauric acid, vinyl ester $$ Vinyl laurate $$ Vinyl dodecanoate $$

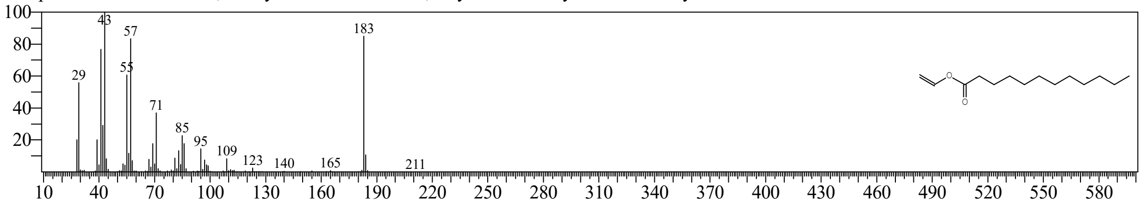

<< Target >>
Line#:15 R.Time:33.608(Scan#:2834) MassPeaks:348
RawMode:Averaged 33.600-33.617(2833-2835) BasePeak:74.05(8184)
BG Mode:Calc. from Peak Group 1 - Event 1

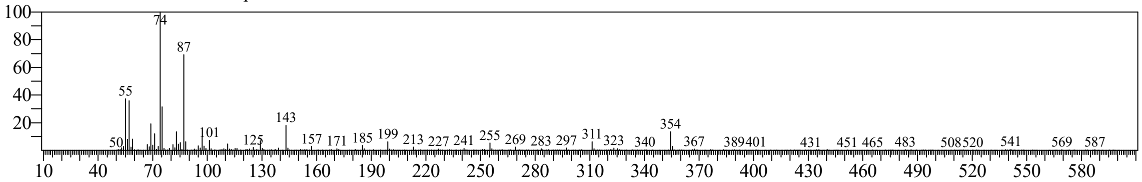

Hit#:1 Entry:196443 Library:NIST17-1.lib
SI:96 Formula:C23H46O2 CAS:929-77-1 MolWeight:354 RetIndex:2475
CompName:Docosanoic acid, methyl ester $$ Behenic acid, methyl ester $$ Methyl behenate $$ Methyl docosanoate $$ n-Docosanoic acid methyl ester $$

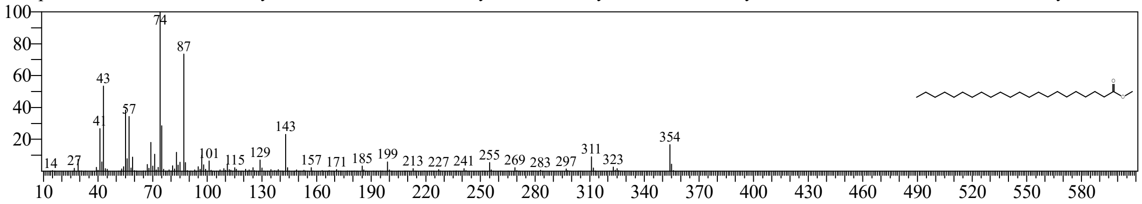

Hit#:2 Entry:178051 Library:NIST14.lib
SI:96 Formula:C23H46O2 CAS:929-77-1 MolWeight:354 RetIndex:2475
CompName:Docosanoic acid, methyl ester $$ Behenic acid, methyl ester $$ Methyl behenate $$ Methyl docosanoate $$ n-Docosanoic acid methyl ester $$

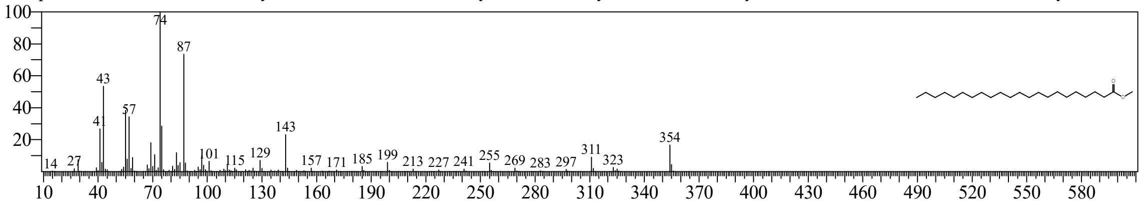

Hit#:3 Entry:36091 Library:NIST17s.lib
SI:96 Formula:C23H46O2 CAS:929-77-1 MolWeight:354 RetIndex:2475
CompName:Docosanoic acid, methyl ester $$ Behenic acid, methyl ester $$ Methyl behenate $$ Methyl docosanoate $$ n-Docosanoic acid methyl ester $$

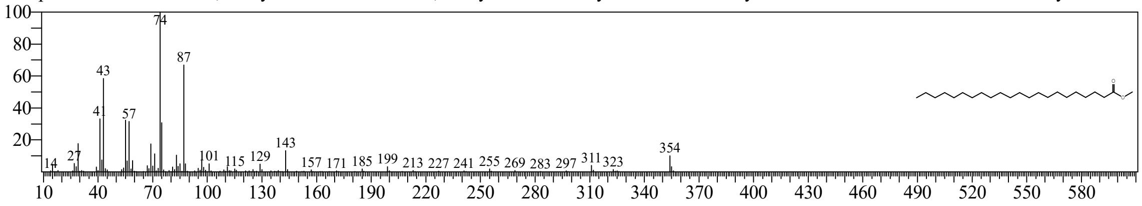

Hit#:4 Entry:31086 Library:NIST14s.lib
SI:96 Formula:C23H46O2 CAS:929-77-1 MolWeight:354 RetIndex:2475
CompName:Docosanoic acid, methyl ester $$ Behenic acid, methyl ester $$ Methyl behenate $$ Methyl docosanoate $$ n-Docosanoic acid methyl ester $$

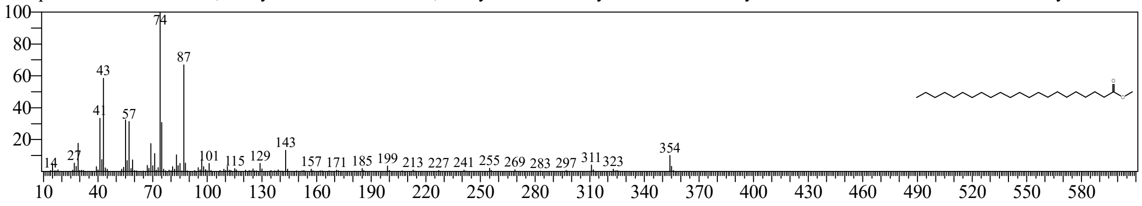

Hit#:5 Entry:36092 Library:NIST17s.lib
SI:95 Formula:C23H46O2 CAS:929-77-1 MolWeight:354 RetIndex:2475
CompName:Docosanoic acid, methyl ester $$ Behenic acid, methyl ester $$ Methyl behenate $$ Methyl docosanoate $$ n-Docosanoic acid methyl ester $$

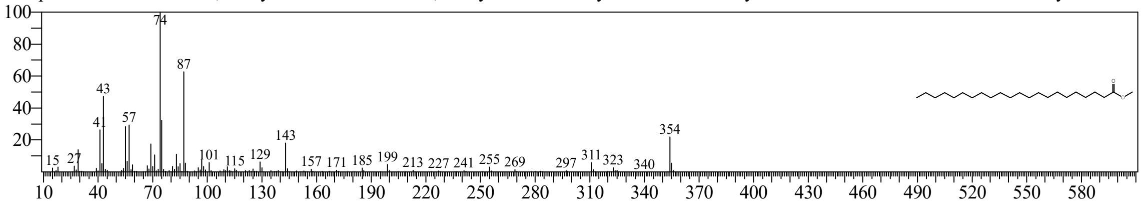

<< Target >>
Line#:16 R.Time:37.850(Scan#:3343) MassPeaks:381
RawMode:Averaged 37.842-37.858(3342-3344) BasePeak:79.10(3101)
BG Mode:Calc. from Peak Group 1 - Event 1

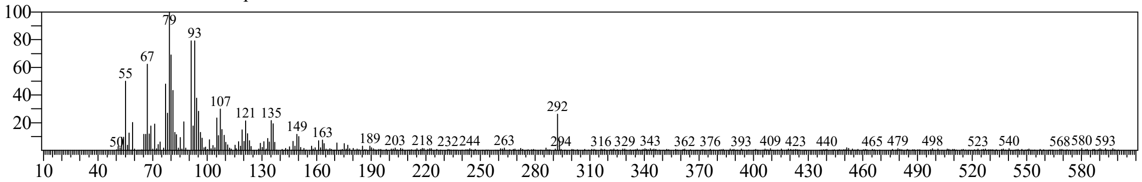

Hit#:1 Entry:135726 Library:NIST17-1.lib
SI:91 Formula:C19H32O2 CAS:0-00-0 MolWeight:292 RetIndex:2101
CompName:Methyl 9.cis.,11.trans.t,13.trans.-octadecatrienoate

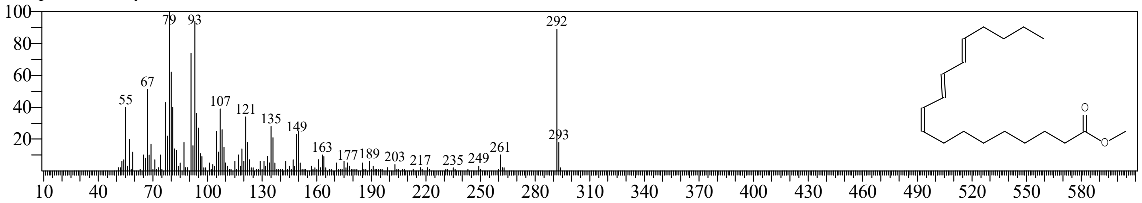

Hit#:2 Entry:124230 Library:NIST14.lib
SI:91 Formula:C19H32O2 CAS:0-00-0 MolWeight:292 RetIndex:2101
CompName:Methyl 9.cis.,11.trans.t,13.trans.-octadecatrienoate

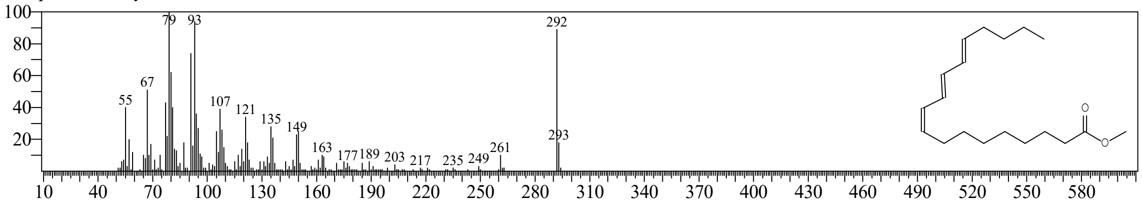

Hit#:3 Entry:124226 Library:NIST14.lib
SI:91 Formula:C19H32O2 CAS:0-00-0 MolWeight:292 RetIndex:2101
CompName:Methyl 6-cis,9-cis,11-trans-octadecatrienoate

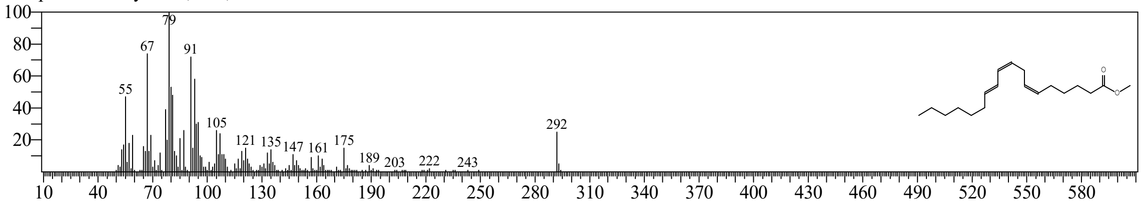

Hit#:4 Entry:135722 Library:NIST17-1.lib
SI:91 Formula:C19H32O2 CAS:0-00-0 MolWeight:292 RetIndex:2101
CompName:Methyl 6-cis,9-cis,11-trans-octadecatrienoate

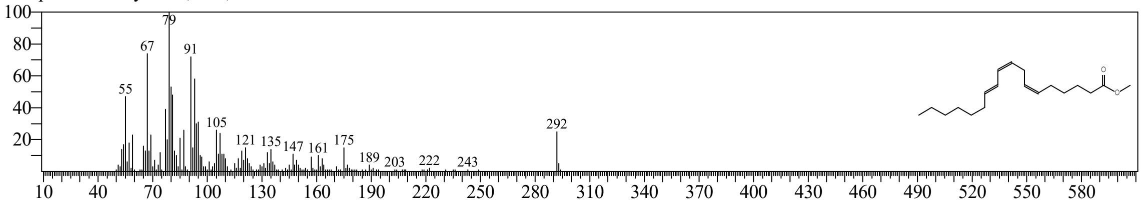

Hit#:5 Entry:124225 Library:NIST14.lib
SI:90 Formula:C19H32O2 CAS:0-00-0 MolWeight:292 RetIndex:2101
CompName:.gamma.-Linolenic acid, methyl ester

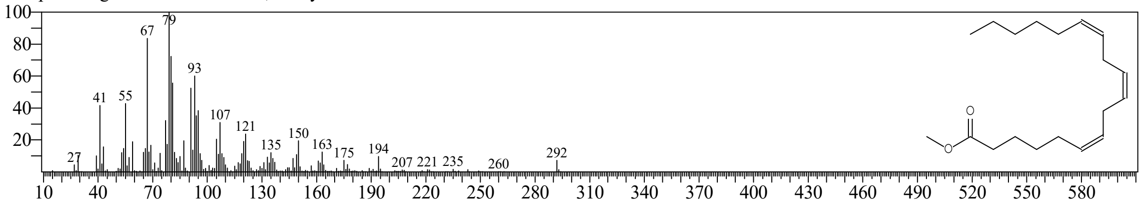

<< Target >>
Line#:17 R.Time:38.408(Scan#:3410) MassPeaks:330
RawMode:Averaged 38.400-38.417(3409-3411) BasePeak:79.10(3282)
BG Mode:Calc. from Peak Group 1 - Event 1

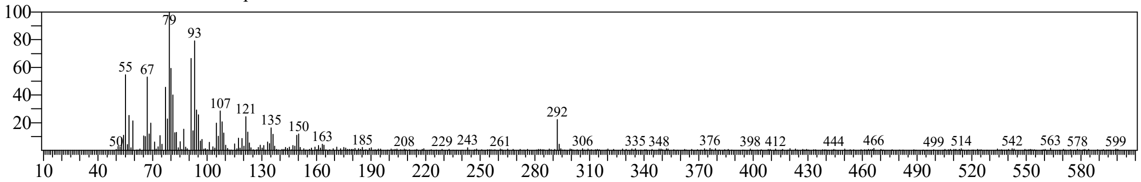

Hit#:1 Entry:135726 Library:NIST17-1.lib
SI:91 Formula:C19H32O2 CAS:0-00-0 MolWeight:292 RetIndex:2101
CompName:Methyl 9.cis.,11.trans.t,13.trans.-octadecatrienoate

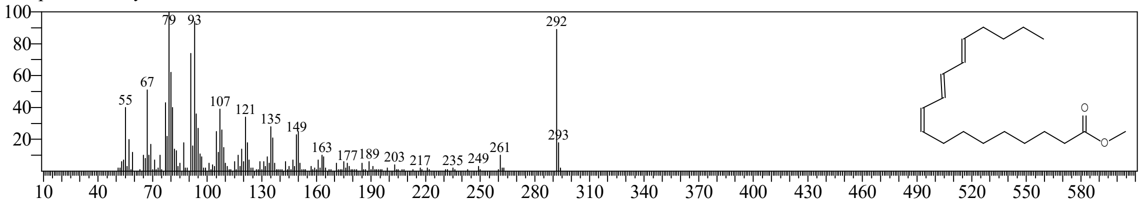

Hit#:2 Entry:124230 Library:NIST14.lib
SI:91 Formula:C19H32O2 CAS:0-00-0 MolWeight:292 RetIndex:2101
CompName:Methyl 9.cis.,11.trans.t,13.trans.-octadecatrienoate

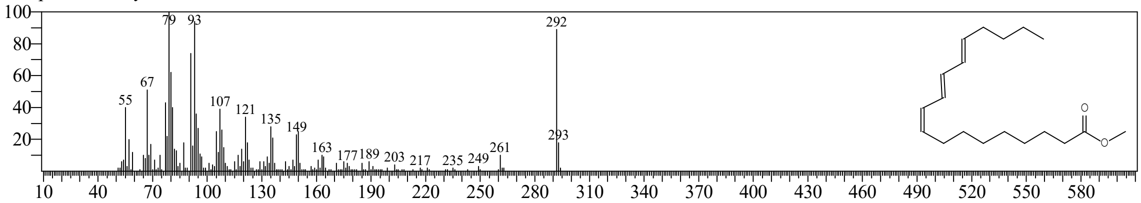

Hit#:3 Entry:124226 Library:NIST14.lib
SI:90 Formula:C19H32O2 CAS:0-00-0 MolWeight:292 RetIndex:2101
CompName:Methyl 6-cis,9-cis,11-trans-octadecatrienoate

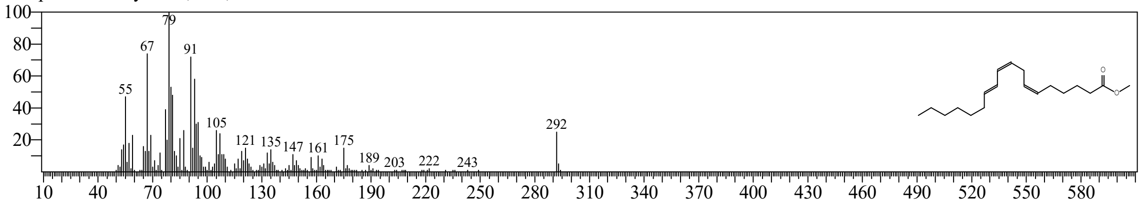

Hit#:4 Entry:135722 Library:NIST17-1.lib
SI:90 Formula:C19H32O2 CAS:0-00-0 MolWeight:292 RetIndex:2101
CompName:Methyl 6-cis,9-cis,11-trans-octadecatrienoate

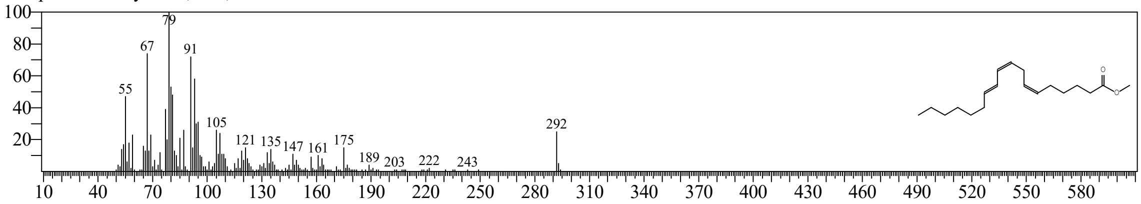

Hit#:5 Entry:124228 Library:NIST14.lib
SI:90 Formula:C19H32O2 CAS:301-00-8 MolWeight:292 RetIndex:2101
CompName:9,12,15-Octadecatrienoic acid, methyl ester, (Z,Z,Z)- $$ Linolenic acid, methyl ester $$ Methyl all-cis-9,12,15-octadecatrienoate $$ Methyl lino

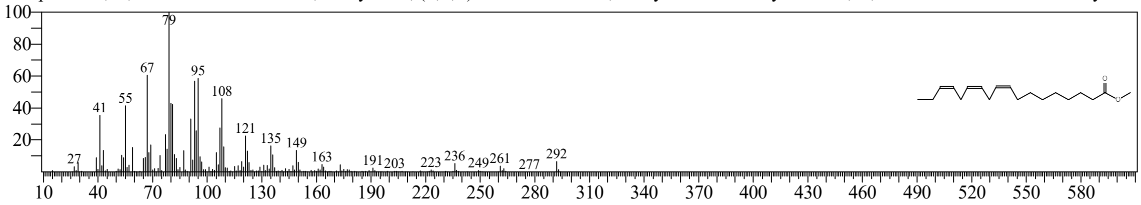

<< Target >>
Line#:18 R.Time:38.833(Scan#:3461) MassPeaks:339
RawMode:Averaged 38.825-38.842(3460-3462) BasePeak:79.10(4585)
BG Mode:Calc. from Peak Group 1 - Event 1

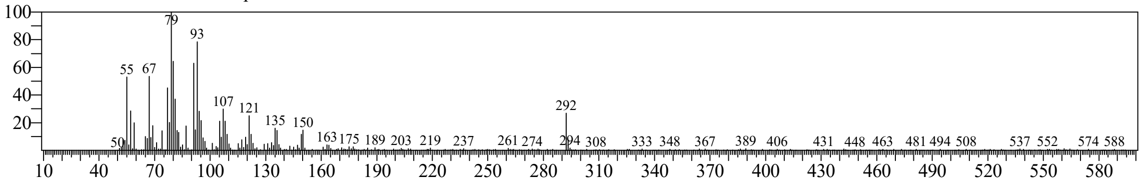

Hit#:1 Entry:135726 Library:NIST17-1.lib
SI:91 Formula:C19H32O2 CAS:0-00-0 MolWeight:292 RetIndex:2101
CompName:Methyl 9.cis.,11.trans.t,13.trans.-octadecatrienoate

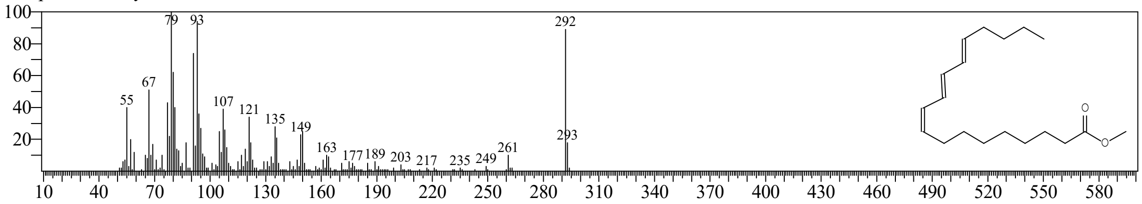

Hit#:2 Entry:124230 Library:NIST14.lib
SI:91 Formula:C19H32O2 CAS:0-00-0 MolWeight:292 RetIndex:2101
CompName:Methyl 9.cis.,11.trans.t,13.trans.-octadecatrienoate

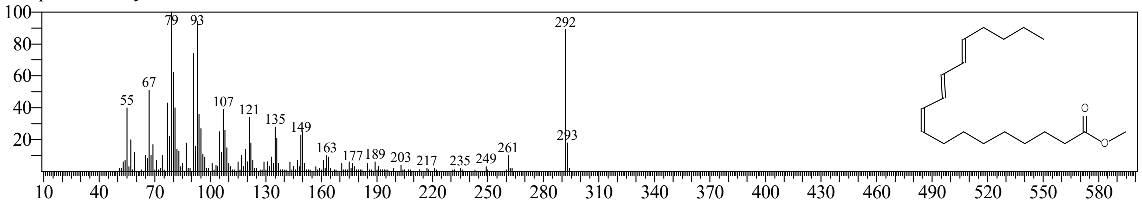

Hit#:3 Entry:135724 Library:NIST17-1.lib
SI:89 Formula:C19H32O2 CAS:301-00-8 MolWeight:292 RetIndex:2101
CompName:9,12,15-Octadecatrienoic acid, methyl ester, (Z,Z,Z)- $$ Linolenic acid, methyl ester $$ Methyl all-cis-9,12,15-octadecatrienoate $$ Methyl lino

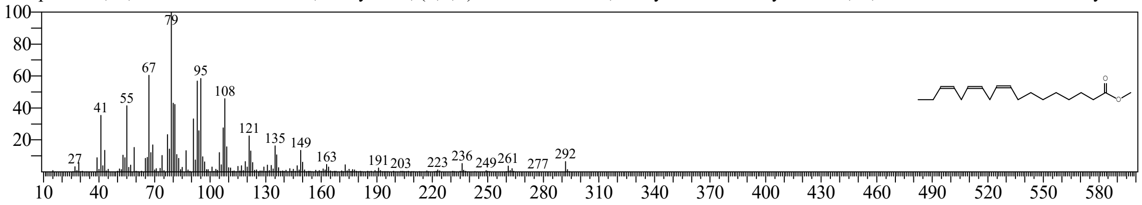

Hit#:4 Entry:124228 Library:NIST14.lib
SI:89 Formula:C19H32O2 CAS:301-00-8 MolWeight:292 RetIndex:2101
CompName:9,12,15-Octadecatrienoic acid, methyl ester, (Z,Z,Z)- $$ Linolenic acid, methyl ester $$ Methyl all-cis-9,12,15-octadecatrienoate $$ Methyl lino

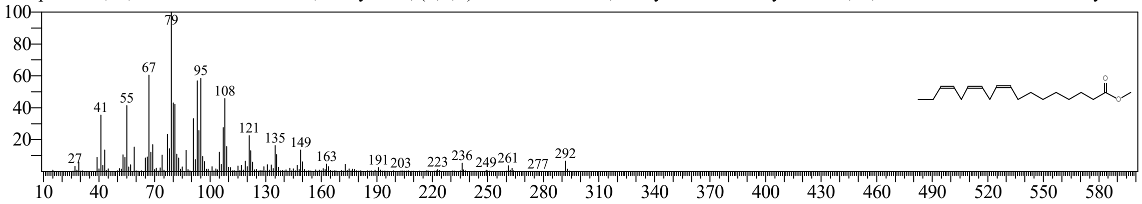

Hit#:5 Entry:135722 Library:NIST17-1.lib
SI:88 Formula:C19H32O2 CAS:0-00-0 MolWeight:292 RetIndex:2101
CompName:Methyl 6-cis,9-cis,11-trans-octadecatrienoate

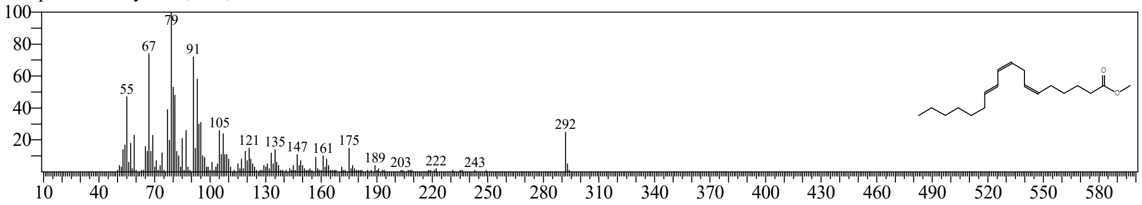

<< Target >>
Line#:19 R.Time:38.992(Scan#:3480) MassPeaks:351
RawMode:Averaged 38.983-39.000(3479-3481) BasePeak:79.10(4519)
BG Mode:Calc. from Peak Group 1 - Event 1

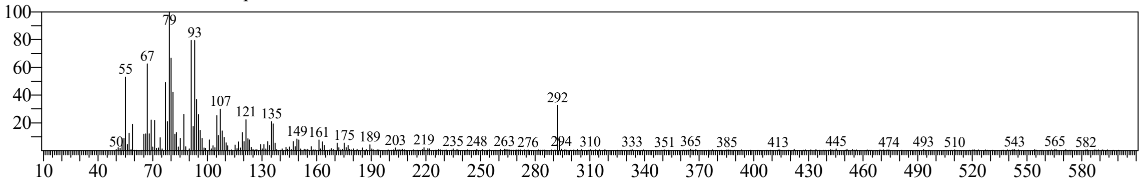

Hit#:1 Entry:124226 Library:NIST14.lib
SI:92 Formula:C19H32O2 CAS:0-00-0 MolWeight:292 RetIndex:2101
CompName:Methyl 6-cis,9-cis,11-trans-octadecatrienoate

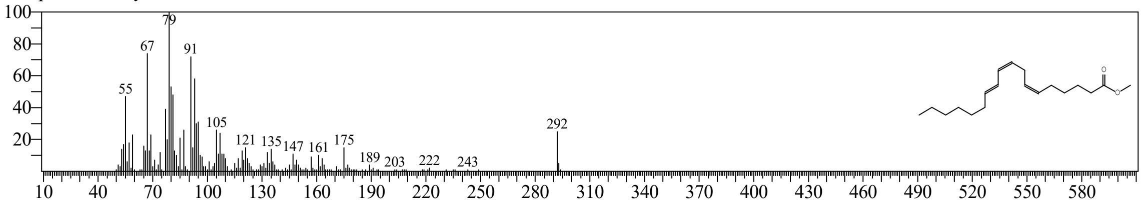

Hit#:2 Entry:135722 Library:NIST17-1.lib
SI:92 Formula:C19H32O2 CAS:0-00-0 MolWeight:292 RetIndex:2101
CompName:Methyl 6-cis,9-cis,11-trans-octadecatrienoate

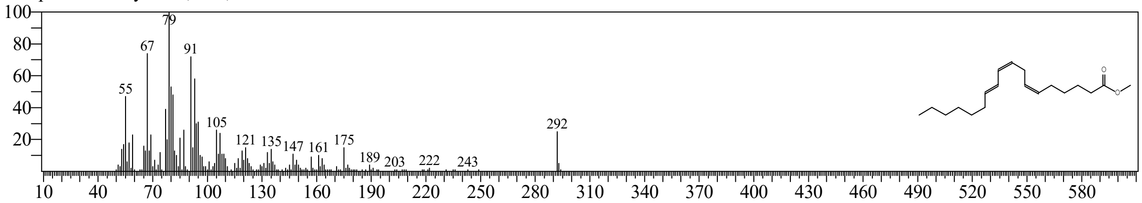

Hit#:3 Entry:124230 Library:NIST14.lib
SI:92 Formula:C19H32O2 CAS:0-00-0 MolWeight:292 RetIndex:2101
CompName:Methyl 9.cis.,11.trans.t,13.trans.-octadecatrienoate

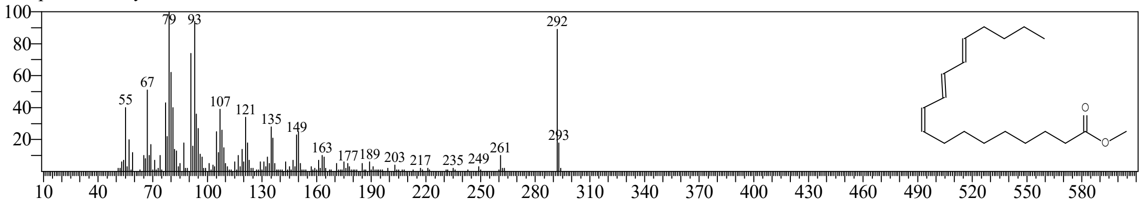

Hit#:4 Entry:135726 Library:NIST17-1.lib
SI:92 Formula:C19H32O2 CAS:0-00-0 MolWeight:292 RetIndex:2101
CompName:Methyl 9.cis.,11.trans.t,13.trans.-octadecatrienoate

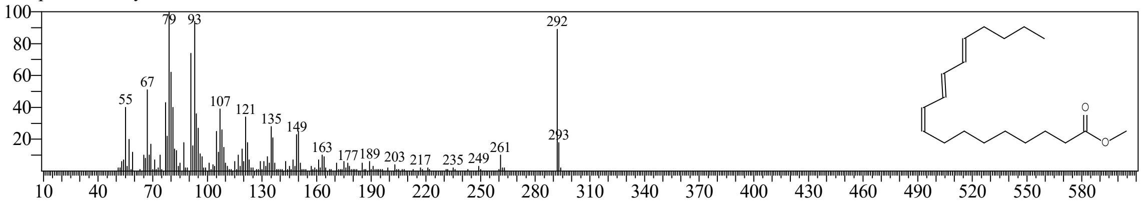

Hit#:5 Entry:135721 Library:NIST17-1.lib
SI:90 Formula:C19H32O2 CAS:16326-32-2 MolWeight:292 RetIndex:2101
CompName:Methyl .gamma.-linolenate

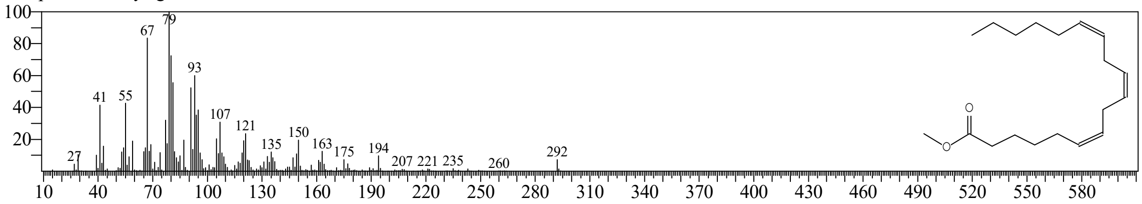

Supplement: Supplementary file 1 [file molecules-31-02891-s001.zip › 56d-CK-3.pdf]
