# Supplementary material for: Selective Inhibition of Lipid Oxidation by a Quercetagetin-Rich Extract from Tagetes erecta: A Chemometric Approach Using OPLS-DA and Selectivity Index Analysis
Source: Molecules. 2026 Aug 19;31(16):2891. doi: 10.3390/molecules31162891 (PMC13515886; doi:10.3390/molecules31162891)

QG200-3-56d

<<Column>>
Name : HP-88
Thickness : 0.20um
Length : 100.0m
Inside Diameter : 0.25mm

Method

===== Analytical Line 1 =====

Column Oven Temp.	:120.0 °C	
Injection Temp.	:250.00 °C	
Injection Mode	:Split	
Column Flow	:1.04 mL/min	
Split Ratio	:20.0	
Oven Temp. Program		
Rate	Temperature(°C)	Hold Time(min)
-	120.0	1.00
10.00	175.0	10.00
5.00	210.0	5.00
5.00	230.0	15.00

[GCMS-QP2010]
IonSourceTemp :200.00 °C
Interface Temp. :220.00 °C
m/z scanning range：45～500m/z

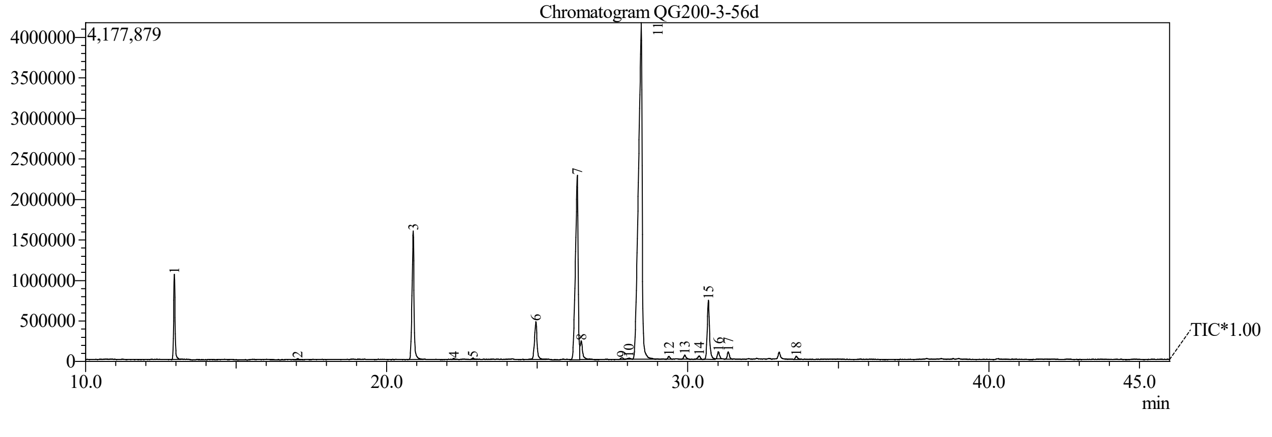

Peak Report TIC

Peak#	R.Time	Area	Area%	Height	Height%	A/H	Name
1	12.950	3442512	4.83	1051426	9.63	3.27	Undecanoic acid, methyl ester
2	17.048	58885	0.08	13398	0.12	4.40	Methyl tetradecanoate
3	20.883	7462332	10.46	1587250	14.54	4.70	Hexadecanoic acid, methyl ester
4	22.235	52821	0.07	12723	0.12	4.15	7-Hexadecenoic acid, methyl ester, (Z)-
5	22.865	99761	0.14	18956	0.17	5.26	Heptadecanoic acid, methyl ester
6	24.957	2835245	3.98	468346	4.29	6.05	Methyl stearate
7	26.330	14376259	20.16	2273218	20.83	6.32	11-Octadecenoic acid, methyl ester
8	26.463	1300998	1.82	230524	2.11	5.64	trans-13-Octadecenoic acid, methyl ester
9	27.816	135004	0.19	20859	0.19	6.47	9,12-Octadecadienoic acid, methyl ester
10	28.042	82051	0.12	11850	0.11	6.92	9,12-Octadecadienoic acid, methyl ester
11	28.455	35811904	50.22	4148650	38.01	8.63	9,12-Octadecadienoic acid (Z,Z)-, methyl ester
12	29.375	170244	0.24	36241	0.33	4.70	Eicosanoic acid, methyl ester
13	29.898	264883	0.37	50349	0.46	5.26	11,14,17-Eicosatrienoic acid, methyl ester
14	30.381	243774	0.34	43315	0.40	5.63	9,12,15-Octadecatrienoic acid, methyl ester, (Z

Peak#	R.Time	Area	Area%	Height	Height%	A/H	Name
15	30.684	3876152	5.44	729527	6.68	5.31	9,12,15-Octadecatrienoic acid, methyl ester, (Z
16	31.012	452803	0.63	88512	0.81	5.12	9,11-Octadecadienoic acid, methyl ester, (E,E)
17	31.346	455521	0.64	91151	0.84	5.00	Methyl 10-trans,12-cis-octadecadienoate
18	33.605	193204	0.27	37316	0.34	5.18	Docosanoic acid, methyl ester
		71314353	100.00	10913611	100.00		

Library

<< Target >>
Line#:1 R.Time:12.950(Scan#:355) MassPeaks:332
RawMode:Averaged 12.942-12.958(354-356) BasePeak:74.05(302385)
BG Mode:Calc. from Peak Group 1 - Event 1

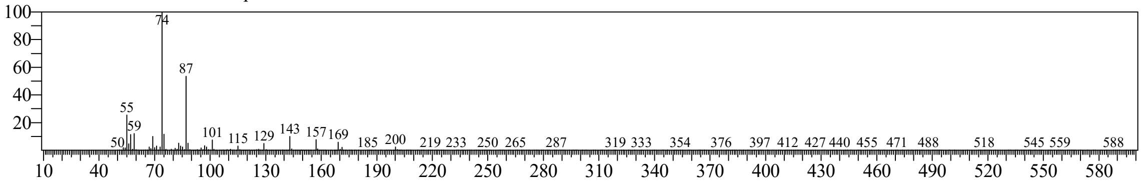

Hit#:1 Entry:46966 Library:NIST14.lib
SI:96 Formula:C12H24O2 CAS:1731-86-8 MolWeight:200 RetIndex:1381
CompName:Undecanoic acid, methyl ester $$ Methyl undecanoate $$ Methyl undecylenate $$ n-Undecanoic acid methyl ester $$ Methyl ester of undecanoi

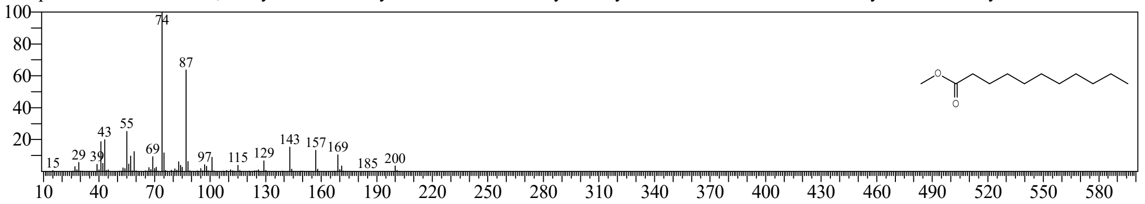

Hit#:2 Entry:49598 Library:NIST17-1.lib
SI:96 Formula:C12H24O2 CAS:1731-86-8 MolWeight:200 RetIndex:1381
CompName:Undecanoic acid, methyl ester $$ Methyl undecanoate $$ Methyl undecylenate $$ n-Undecanoic acid methyl ester $$ Methyl ester of undecanoi

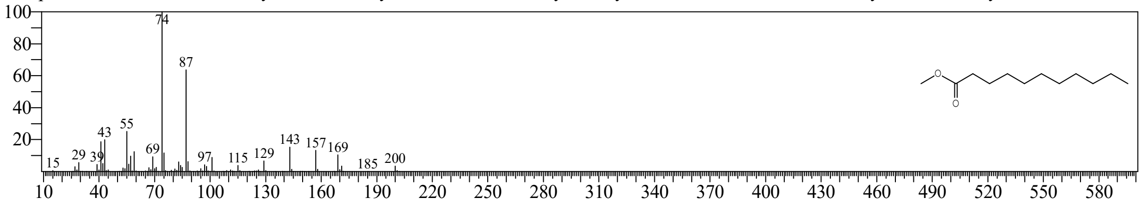

Hit#:3 Entry:21618 Library:NIST17s.lib
SI:95 Formula:C12H24O2 CAS:1731-86-8 MolWeight:200 RetIndex:1381
CompName:Undecanoic acid, methyl ester $$ Methyl undecanoate $$ Methyl undecylenate $$ n-Undecanoic acid methyl ester $$ Methyl ester of undecanoi

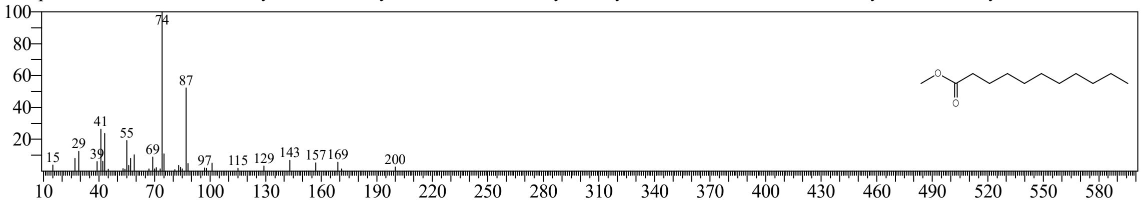

Hit#:4 Entry:18487 Library:NIST14s.lib
SI:94 Formula:C12H24O2 CAS:1731-86-8 MolWeight:200 RetIndex:1381
CompName:Undecanoic acid, methyl ester $$ Methyl undecanoate $$ Methyl undecylenate $$ n-Undecanoic acid methyl ester $$ Methyl ester of undecanoi

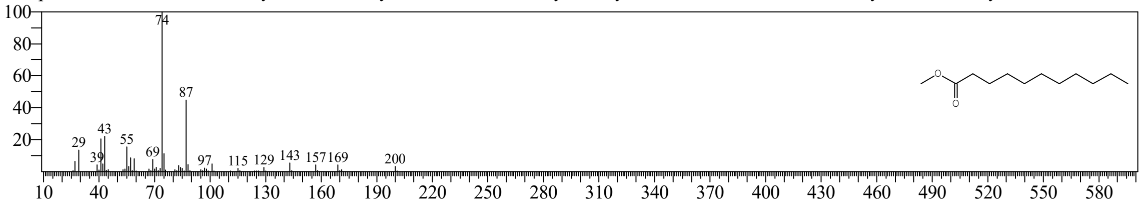

Hit#:5 Entry:21619 Library:NIST17s.lib
SI:94 Formula:C12H24O2 CAS:1731-86-8 MolWeight:200 RetIndex:1381
CompName:Undecanoic acid, methyl ester $$ Methyl undecanoate $$ Methyl undecylenate $$ n-Undecanoic acid methyl ester $$ Methyl ester of undecanoi

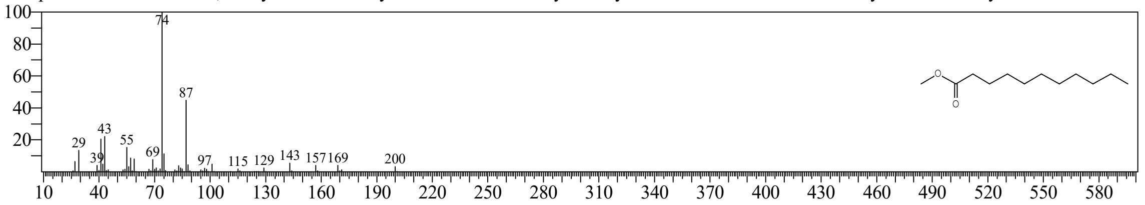

<< Target >>
Line#:2 R.Time:17.050(Scan#:847) MassPeaks:301
RawMode:Averaged 17.042-17.058(846-848) BasePeak:74.00(2894)
BG Mode:Calc. from Peak Group 1 - Event 1

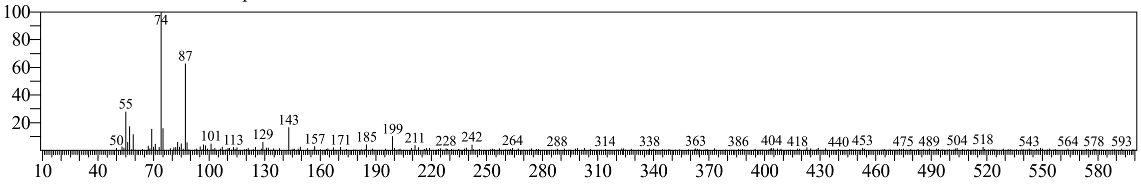

Hit#:1 Entry:86786 Library:NIST17-1.lib
SI:93 Formula:C15H30O2 CAS:124-10-7 MolWeight:242 RetIndex:1680
CompName:Methyl tetradecanoate $$ Tetradecanoic acid, methyl ester $$ Myristic acid, methyl ester $$ Metholeneat 2495 $$ Methyl myristate $$ Methyl r

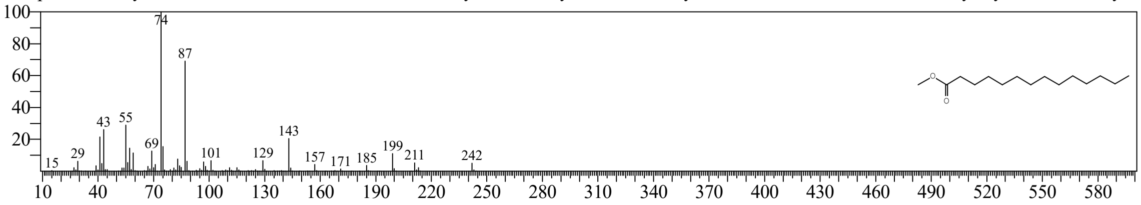

Hit#:2 Entry:80645 Library:NIST14.lib
SI:93 Formula:C15H30O2 CAS:124-10-7 MolWeight:242 RetIndex:1680
CompName:Methyl tetradecanoate $$ Tetradecanoic acid, methyl ester $$ Myristic acid, methyl ester $$ Metholeneat 2495 $$ Methyl myristate $$ Methyl r

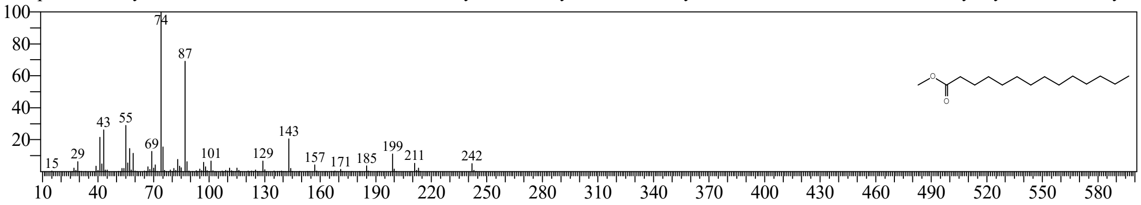

Hit#:3 Entry:86785 Library:NIST17-1.lib
SI:92 Formula:C15H30O2 CAS:5129-58-8 MolWeight:242 RetIndex:1615
CompName:Tridecanoic acid, 12-methyl-, methyl ester $$ Methyl isomyristate $$ Methyl 12-methyltridecanoate $$

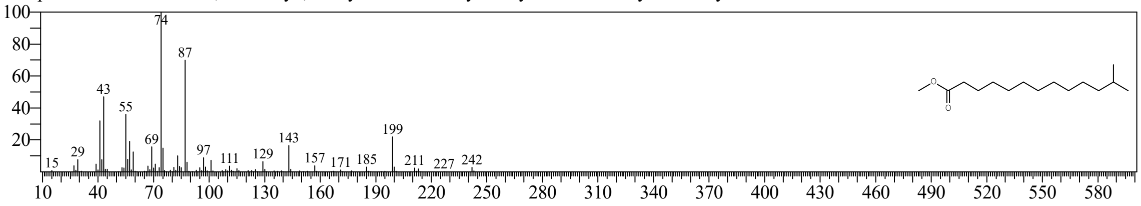

Hit#:4 Entry:80644 Library:NIST14.lib
SI:92 Formula:C15H30O2 CAS:5129-58-8 MolWeight:242 RetIndex:1615
CompName:Tridecanoic acid, 12-methyl-, methyl ester $$ Methyl isomyristate $$ Methyl 12-methyltridecanoate $$

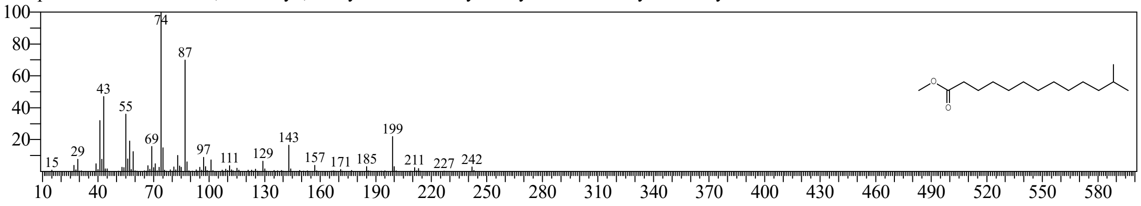

Hit#:5 Entry:23848 Library:NIST14s.lib
SI:91 Formula:C15H30O2 CAS:124-10-7 MolWeight:242 RetIndex:1680
CompName:Methyl tetradecanoate $$ Tetradecanoic acid, methyl ester $$ Myristic acid, methyl ester $$ Metholeneat 2495 $$ Methyl myristate $$ Methyl r

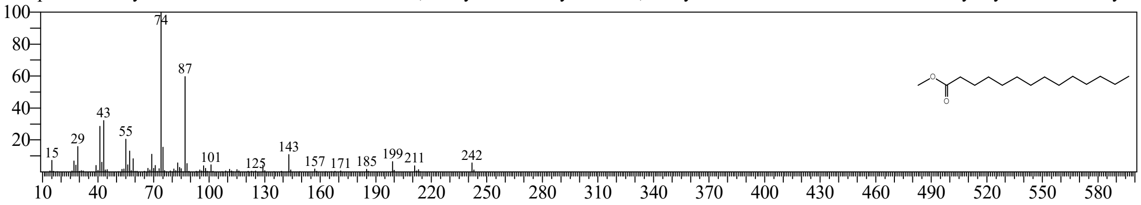

<< Target >>
Line#:3 R.Time:20.883(Scan#:1307) MassPeaks:376
RawMode:Averaged 20.875-20.892(1306-1308) BasePeak:74.05(366638)
BG Mode:Calc. from Peak Group 1 - Event 1

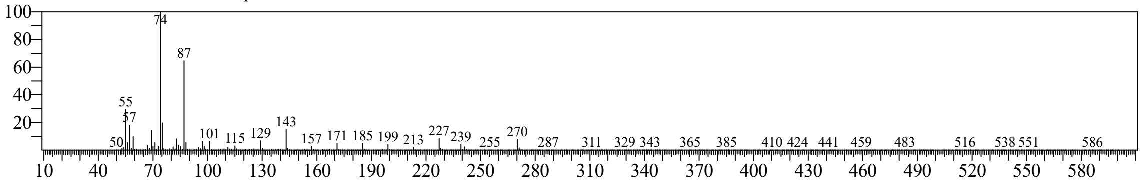

Hit#:1 Entry:104648 Library:NIST14.lib
SI:97 Formula:C17H34O2 CAS:112-39-0 MolWeight:270 RetIndex:1878
CompName:Hexadecanoic acid, methyl ester $$ Palmitic acid, methyl ester $$ n-Hexadecanoic acid methyl ester $$ Metholene 2216 $$ Methyl hexadecano:

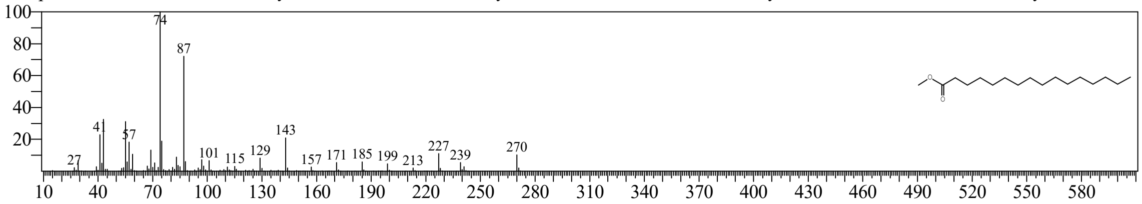

Hit#:2 Entry:113663 Library:NIST17-1.lib
SI:97 Formula:C17H34O2 CAS:112-39-0 MolWeight:270 RetIndex:1878
CompName:Hexadecanoic acid, methyl ester $$ Palmitic acid, methyl ester $$ n-Hexadecanoic acid methyl ester $$ Metholene 2216 $$ Methyl hexadecano:

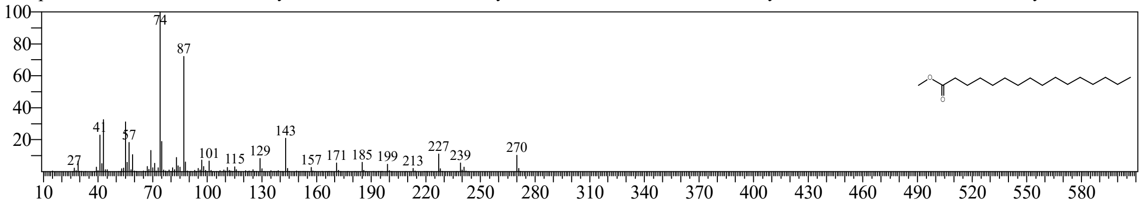

Hit#:3 Entry:26272 Library:NIST14s.lib
SI:97 Formula:C17H34O2 CAS:112-39-0 MolWeight:270 RetIndex:1878
CompName:Hexadecanoic acid, methyl ester $$ Palmitic acid, methyl ester $$ n-Hexadecanoic acid methyl ester $$ Metholene 2216 $$ Methyl hexadecano:

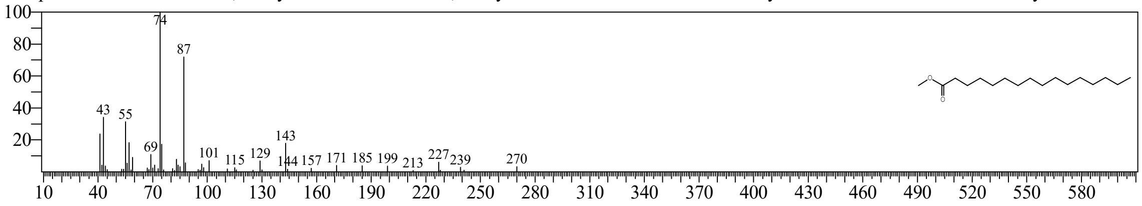

Hit#:4 Entry:30647 Library:NIST17s.lib
SI:97 Formula:C17H34O2 CAS:112-39-0 MolWeight:270 RetIndex:1878
CompName:Hexadecanoic acid, methyl ester $$ Palmitic acid, methyl ester $$ n-Hexadecanoic acid methyl ester $$ Metholene 2216 $$ Methyl hexadecano:

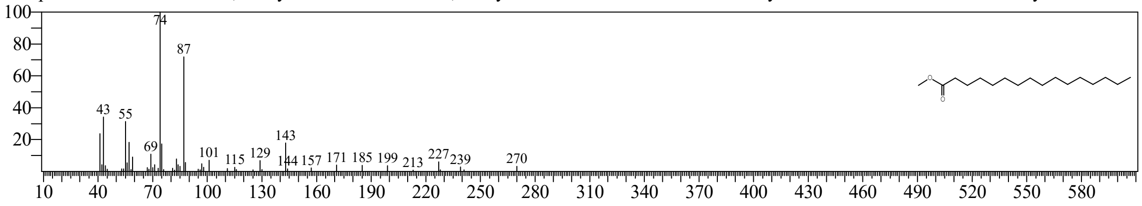

Hit#:5 Entry:26271 Library:NIST14s.lib
SI:96 Formula:C17H34O2 CAS:112-39-0 MolWeight:270 RetIndex:1878
CompName:Hexadecanoic acid, methyl ester $$ Palmitic acid, methyl ester $$ n-Hexadecanoic acid methyl ester $$ Metholene 2216 $$ Methyl hexadecano:

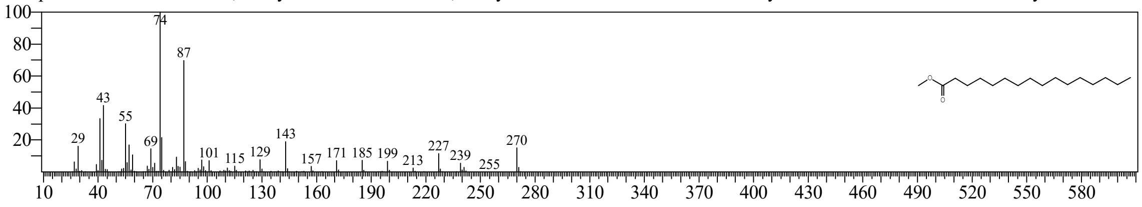

<< Target >>
Line#:4 R.Time:22.233(Scan#:1469) MassPeaks:315
RawMode:Averaged 22.225-22.242(1468-1470) BasePeak:55.05(1112)
BG Mode:Calc. from Peak Group 1 - Event 1

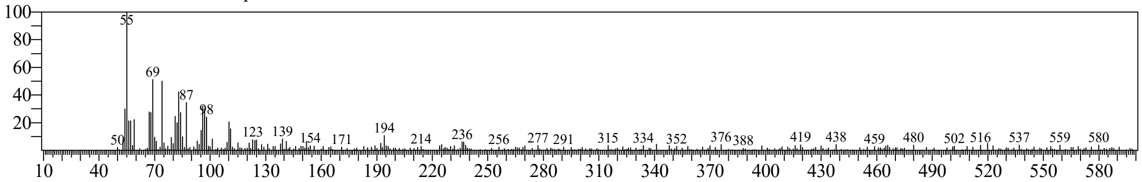

Hit#:1 Entry:111510 Library:NIST17-1.lib
SI:91 Formula:C17H32O2 CAS:56875-67-3 MolWeight:268 RetIndex:1886
CompName:7-Hexadecenoic acid, methyl ester, (Z)- $$ Methyl (7E)-7-hexadecenoate # $$

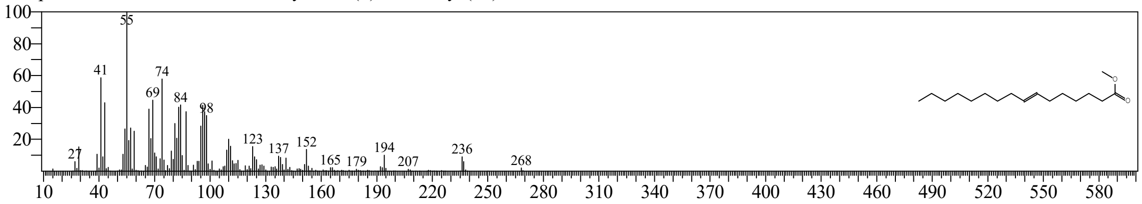

Hit#:2 Entry:26064 Library:NIST14s.lib
SI:91 Formula:C17H32O2 CAS:1120-25-8 MolWeight:268 RetIndex:1886
CompName:9-Hexadecenoic acid, methyl ester, (Z)- $$ Methyl palmitoleate $$ Methyl palmitoleinate $$ Palmitoleic acid, methyl ester $$ Methyl (9Z)-9-he:

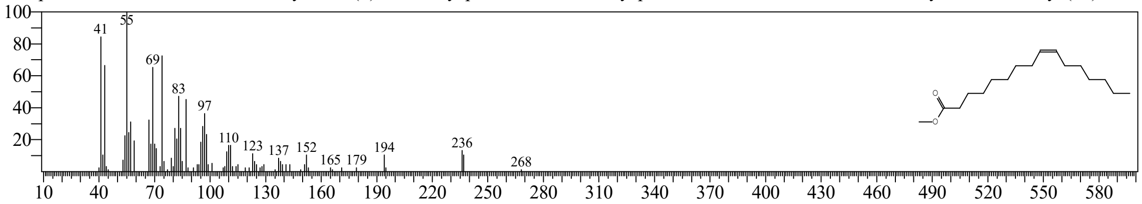

Hit#:3 Entry:30413 Library:NIST17s.lib
SI:91 Formula:C17H32O2 CAS:1120-25-8 MolWeight:268 RetIndex:1886
CompName:9-Hexadecenoic acid, methyl ester, (Z)- $$ Methyl palmitoleate $$ Methyl palmitoleinate $$ Palmitoleic acid, methyl ester $$ Methyl (9Z)-9-he:

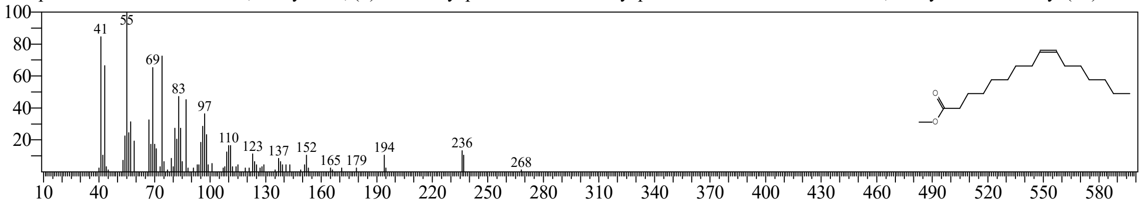

Hit#:4 Entry:28136 Library:NIST14s.lib
SI:91 Formula:C19H36O2 CAS:112-62-9 MolWeight:296 RetIndex:2085
CompName:9-Octadecenoic acid (Z)-, methyl ester $$ Oleic acid, methyl ester $$ Emery oleic acid ester 2301 $$ Methyl cis-9-octadecenoate $$ Methyl ole:

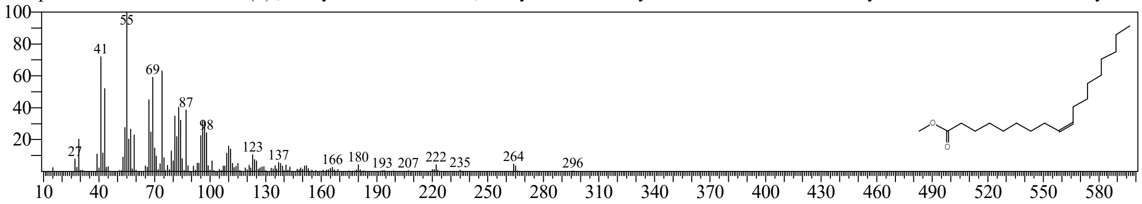

Hit#:5 Entry:32745 Library:NIST17s.lib
SI:91 Formula:C19H36O2 CAS:112-62-9 MolWeight:296 RetIndex:2085
CompName:9-Octadecenoic acid (Z)-, methyl ester $$ Oleic acid, methyl ester $$ Emery oleic acid ester 2301 $$ Methyl cis-9-octadecenoate $$ Methyl ole:

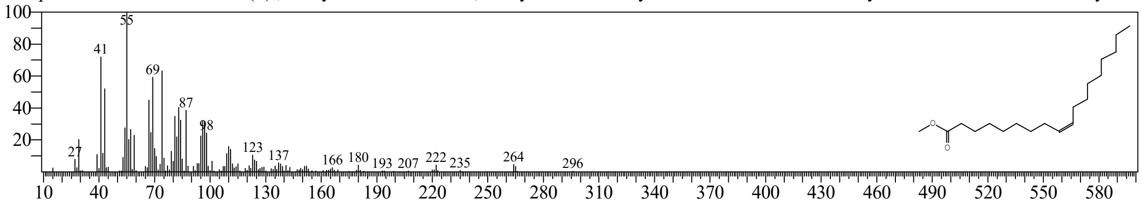

<< Target >>
Line#:5 R.Time:22.867(Scan#:1545) MassPeaks:354
RawMode:Averaged 22.858-22.875(1544-1546) BasePeak:74.05(2978)
BG Mode:Calc. from Peak Group 1 - Event 1

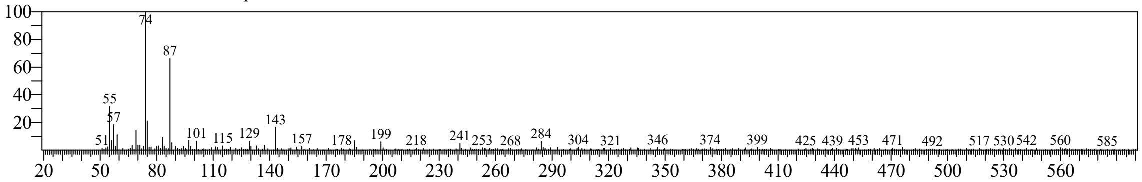

Hit#:1 Entry:31784 Library:NIST17s.lib
SI:90 Formula:C18H36O2 CAS:1731-92-6 MolWeight:284 RetIndex:1978
CompName:Heptadecanoic acid, methyl ester $$ Margaric acid methyl ester $$ Methyl heptadecanoate $$ Methyl margarate $$ n-Heptadecanoic acid methy

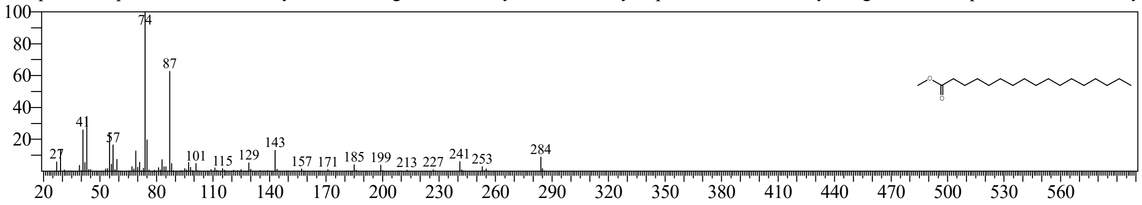

Hit#:2 Entry:27275 Library:NIST14s.lib
SI:90 Formula:C18H36O2 CAS:1731-92-6 MolWeight:284 RetIndex:1978
CompName:Heptadecanoic acid, methyl ester $$ Margaric acid methyl ester $$ Methyl heptadecanoate $$ Methyl margarate $$ n-Heptadecanoic acid methy

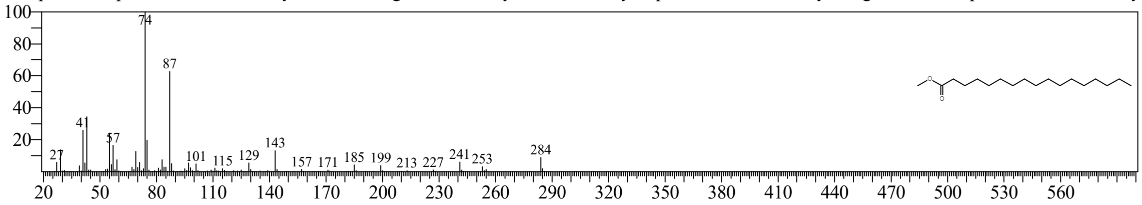

Hit#:3 Entry:31783 Library:NIST17s.lib
SI:89 Formula:C18H36O2 CAS:1731-92-6 MolWeight:284 RetIndex:1978
CompName:Heptadecanoic acid, methyl ester $$ Margaric acid methyl ester $$ Methyl heptadecanoate $$ Methyl margarate $$ n-Heptadecanoic acid methy

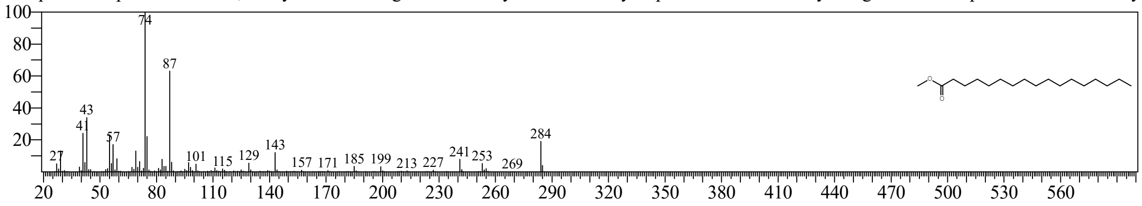

Hit#:4 Entry:27274 Library:NIST14s.lib
SI:89 Formula:C18H36O2 CAS:1731-92-6 MolWeight:284 RetIndex:1978
CompName:Heptadecanoic acid, methyl ester $$ Margaric acid methyl ester $$ Methyl heptadecanoate $$ Methyl margarate $$ n-Heptadecanoic acid methy

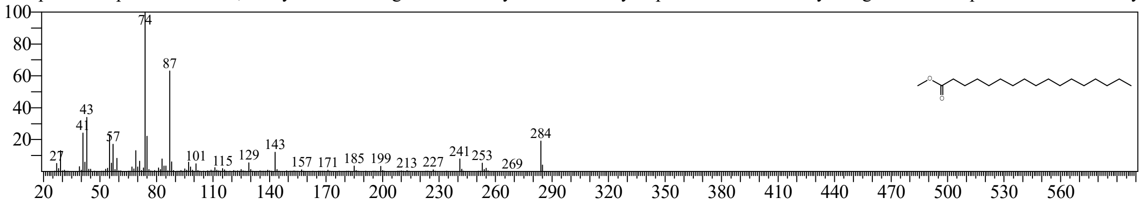

Hit#:5 Entry:117104 Library:NIST14.lib
SI:89 Formula:C18H36O2 CAS:6929-04-0 MolWeight:284 RetIndex:1914
CompName:Hexadecanoic acid, 15-methyl-, methyl ester $$ Methyl isoheptadecanoate $$ Methyl 15-methylhexadecanoate $$

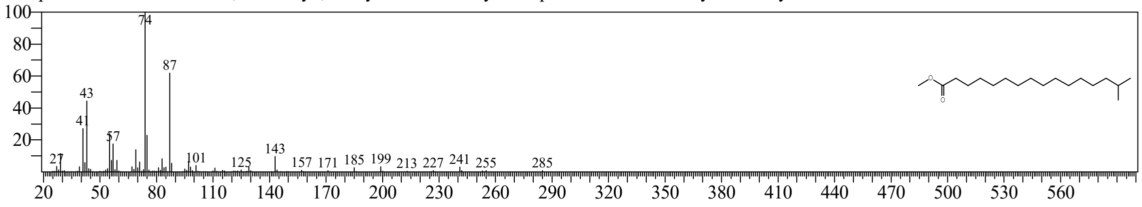

<< Target >>
Line#:6 R.Time:24.958(Scan#:1796) MassPeaks:353
RawMode:Averaged 24.950-24.967(1795-1797) BasePeak:74.05(100354)
BG Mode:Calc. from Peak Group 1 - Event 1

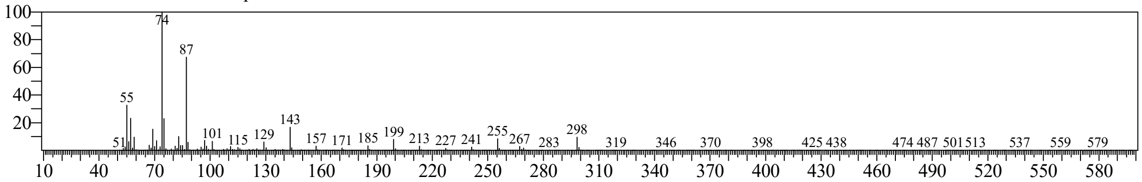

Hit#:1 Entry:32875 Library:NIST17s.lib
SI:97 Formula:C19H38O2 CAS:112-61-8 MolWeight:298 RetIndex:2077
CompName:Methyl stearate $$ Octadecanoic acid, methyl ester $$ Stearic acid, methyl ester $$ n-Octadecanoic acid, methyl ester $$ Kemester 9718 $$ Me

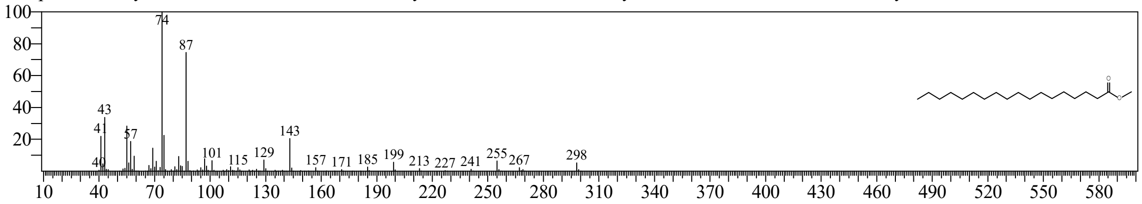

Hit#:2 Entry:28257 Library:NIST14s.lib
SI:97 Formula:C19H38O2 CAS:112-61-8 MolWeight:298 RetIndex:2077
CompName:Methyl stearate $$ Octadecanoic acid, methyl ester $$ Stearic acid, methyl ester $$ n-Octadecanoic acid, methyl ester $$ Kemester 9718 $$ Me

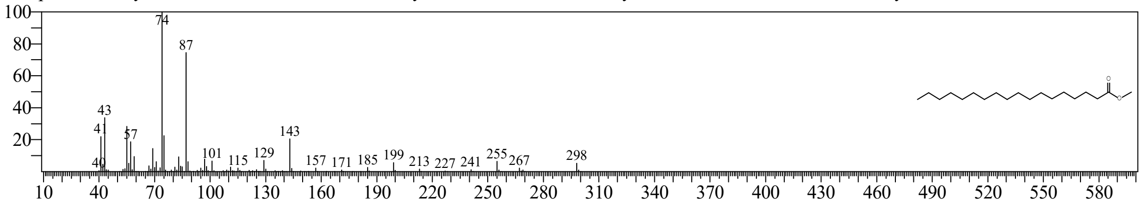

Hit#:3 Entry:28255 Library:NIST14s.lib
SI:97 Formula:C19H38O2 CAS:112-61-8 MolWeight:298 RetIndex:2077
CompName:Methyl stearate $$ Octadecanoic acid, methyl ester $$ Stearic acid, methyl ester $$ n-Octadecanoic acid, methyl ester $$ Kemester 9718 $$ Me

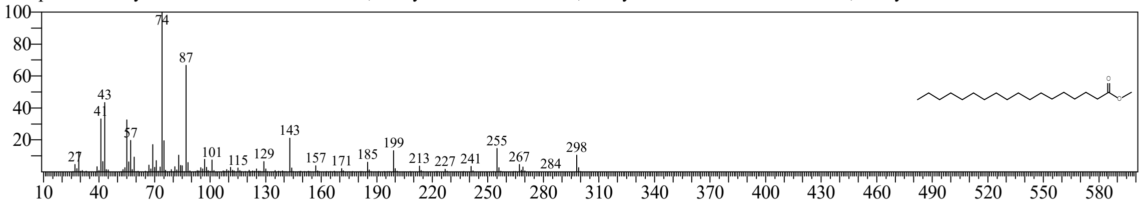

Hit#:4 Entry:141978 Library:NIST17-1.lib
SI:96 Formula:C19H38O2 CAS:112-61-8 MolWeight:298 RetIndex:2077
CompName:Methyl stearate $$ Octadecanoic acid, methyl ester $$ Stearic acid, methyl ester $$ n-Octadecanoic acid, methyl ester $$ Kemester 9718 $$ Me

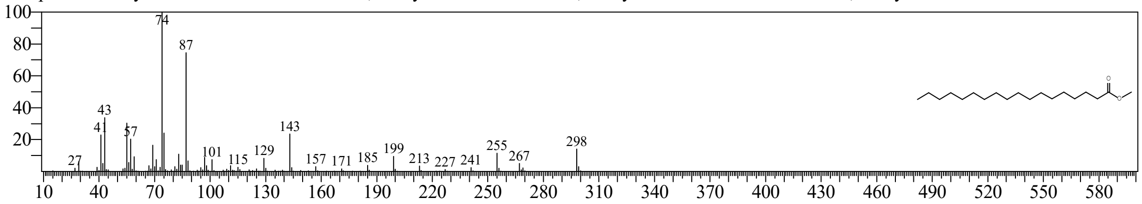

Hit#:5 Entry:129694 Library:NIST14.lib
SI:96 Formula:C19H38O2 CAS:112-61-8 MolWeight:298 RetIndex:2077
CompName:Methyl stearate $$ Octadecanoic acid, methyl ester $$ Stearic acid, methyl ester $$ n-Octadecanoic acid, methyl ester $$ Kemester 9718 $$ Me

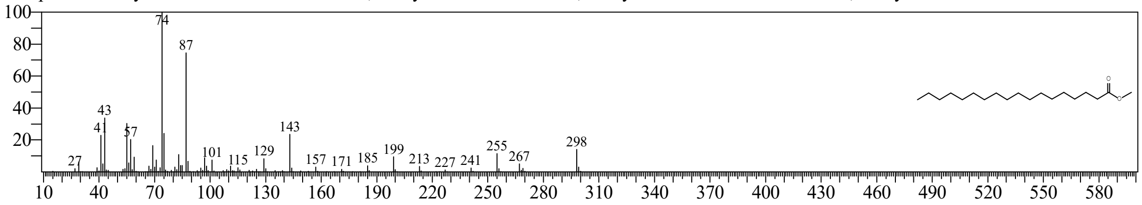

<< Target >>
Line#:7 R.Time:26.333(Scan#:1961) MassPeaks:372
RawMode:Averaged 26.325-26.342(1960-1962) BasePeak:55.05(202632)
BG Mode:Calc. from Peak Group 1 - Event 1

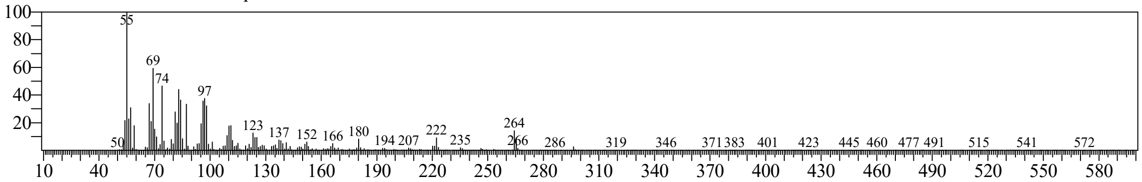

Hit#:1 Entry:28139 Library:NIST14s.lib
SI:96 Formula:C19H36O2 CAS:52380-33-3 MolWeight:296 RetIndex:2085
CompName:11-Octadecenoic acid, methyl ester $$ Methyl 11-octadecenoate $$ Octadec-11-enoic acid, methyl ester $$ Methyl (11E)-11-octadecenoate # $$

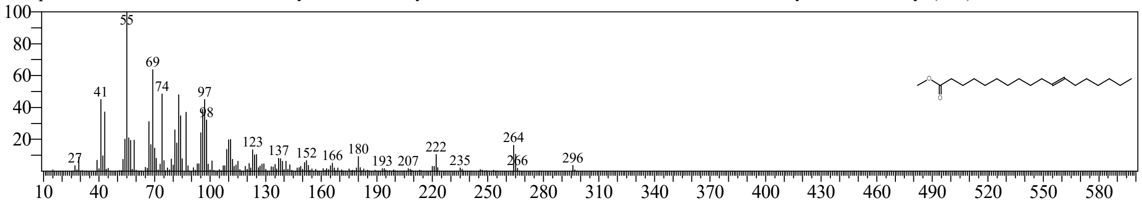

Hit#:2 Entry:32748 Library:NIST17s.lib
SI:96 Formula:C19H36O2 CAS:52380-33-3 MolWeight:296 RetIndex:2085
CompName:11-Octadecenoic acid, methyl ester $$ Methyl 11-octadecenoate $$ Octadec-11-enoic acid, methyl ester $$ Methyl (11E)-11-octadecenoate # $$

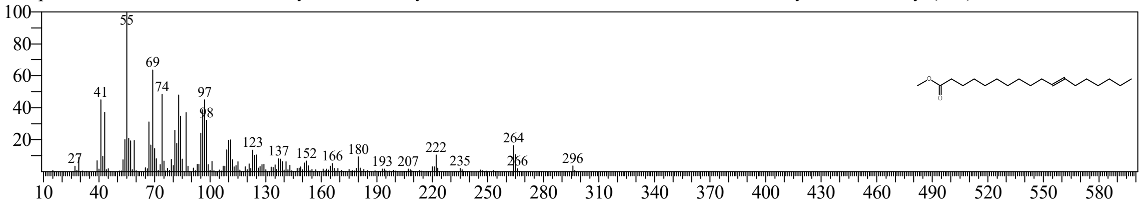

Hit#:3 Entry:139636 Library:NIST17-1.lib
SI:95 Formula:C19H36O2 CAS:13481-95-3 MolWeight:296 RetIndex:2085
CompName:10-Octadecenoic acid, methyl ester $$ Methyl 10-octadecenoate $$

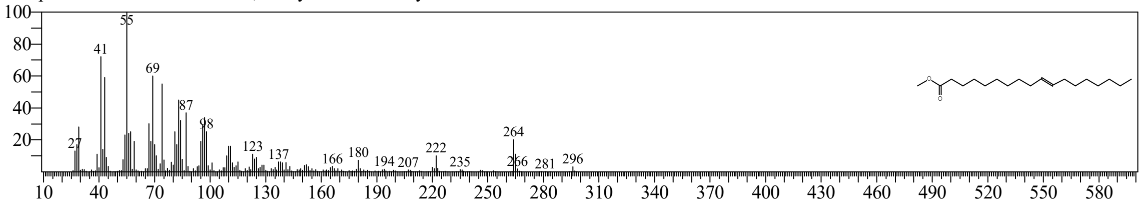

Hit#:4 Entry:127650 Library:NIST14.lib
SI:95 Formula:C19H36O2 CAS:13481-95-3 MolWeight:296 RetIndex:2085
CompName:10-Octadecenoic acid, methyl ester $$ Methyl 10-octadecenoate $$

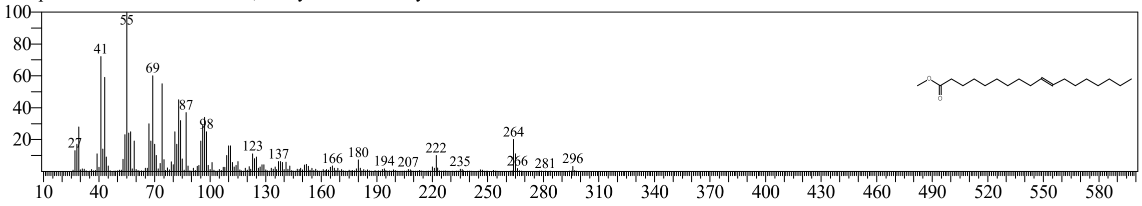

Hit#:5 Entry:28134 Library:NIST14s.lib
SI:95 Formula:C19H36O2 CAS:1937-62-8 MolWeight:296 RetIndex:2085
CompName:9-Octadecenoic acid, methyl ester, (E)- $$ Elaidic acid, methyl ester $$ Methyl elaidate $$ Methyl trans-9-octadecenoate $$ (E)-9-Octadecenoic

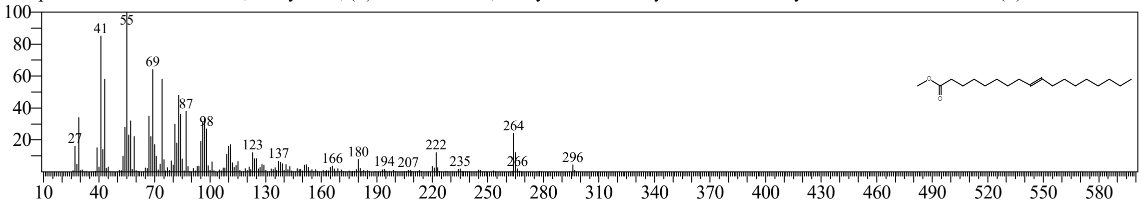

<< Target >>
Line#:8 R.Time:26.467(Scan#:1977) MassPeaks:347
RawMode:Averaged 26.458-26.475(1976-1978) BasePeak:55.10(10922)
BG Mode:Calc. from Peak Group 1 - Event 1

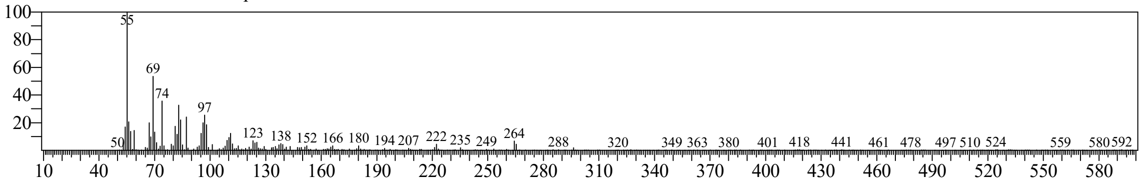

Hit#:1 Entry:139644 Library:NIST17-1.lib
SI:95 Formula:C19H36O2 CAS:0-00-0 MolWeight:296 RetIndex:2085
CompName:trans-13-Octadecenoic acid, methyl ester

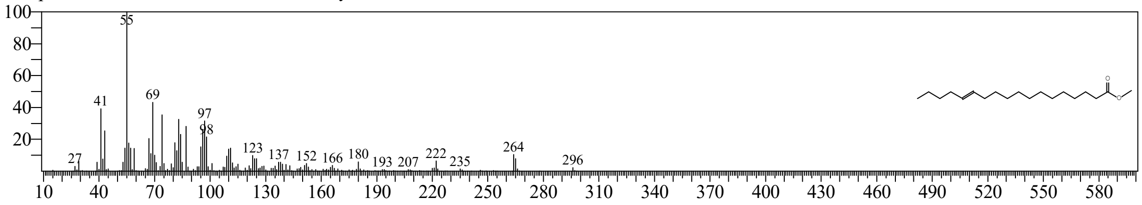

Hit#:2 Entry:127658 Library:NIST14.lib
SI:95 Formula:C19H36O2 CAS:0-00-0 MolWeight:296 RetIndex:2085
CompName:trans-13-Octadecenoic acid, methyl ester

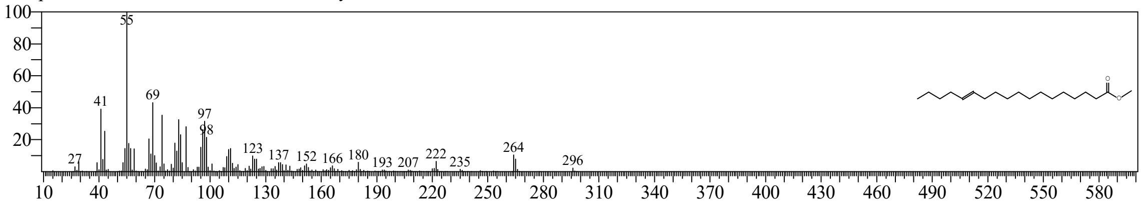

Hit#:3 Entry:28140 Library:NIST14s.lib
SI:94 Formula:C19H36O2 CAS:56554-47-3 MolWeight:296 RetIndex:2085
CompName:13-Octadecenoic acid, methyl ester $$ Methyl (13E)-13-octadecenoate # $$

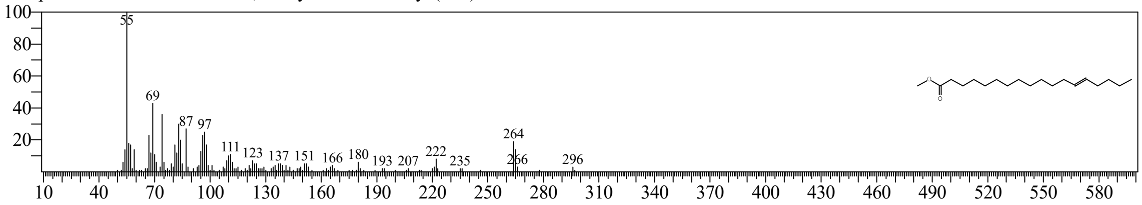

Hit#:4 Entry:32749 Library:NIST17s.lib
SI:94 Formula:C19H36O2 CAS:56554-47-3 MolWeight:296 RetIndex:2085
CompName:13-Octadecenoic acid, methyl ester $$ Methyl (13E)-13-octadecenoate # $$

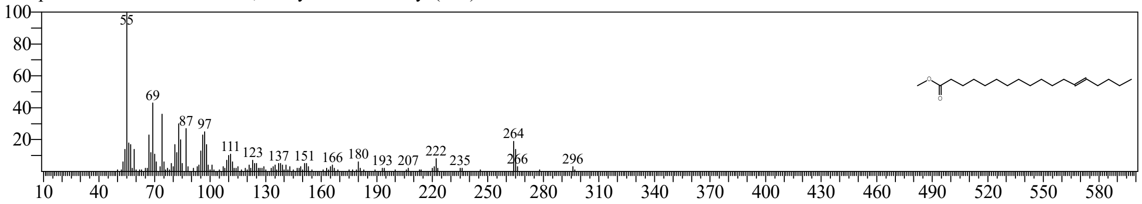

Hit#:5 Entry:139638 Library:NIST17-1.lib
SI:94 Formula:C19H36O2 CAS:56554-47-3 MolWeight:296 RetIndex:2085
CompName:13-Octadecenoic acid, methyl ester $$ Methyl (13E)-13-octadecenoate # $$

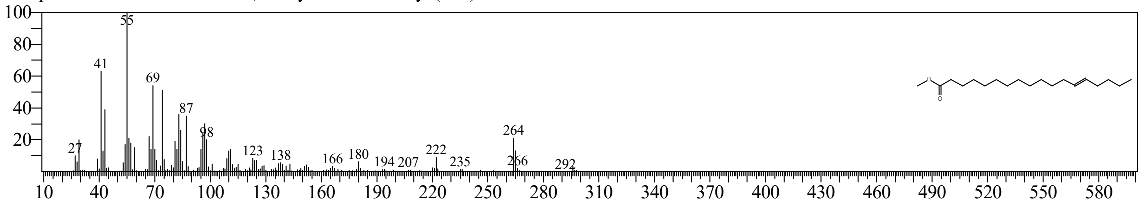

<< Target >>
Line#:9   R.Time:27.817(Scan#:2139) MassPeaks:319
RawMode:Averaged 27.808-27.825(2138-2140) BasePeak:67.10(1811)
BG Mode:Calc. from Peak Group 1 - Event 1

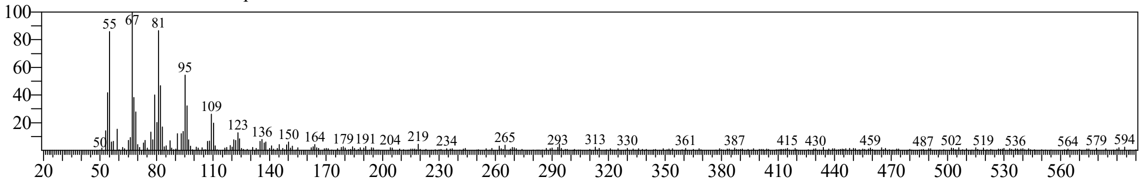

Hit#:1 Entry:125931 Library:NIST14.lib
SI:94 Formula:C19H34O2 CAS:2462-85-3 MolWeight:294 RetIndex:2093
CompName:9,12-Octadecadienoic acid, methyl ester $$ 9,12-Octadecenoic acid, methyl ester $$ Methyl octadeca-9,12-dienoate $$ Methyl 9,12-octadecadi

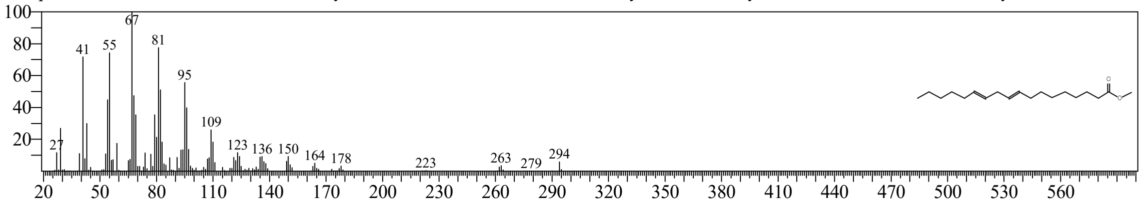

Hit#:2 Entry:137663 Library:NIST17-1.lib
SI:94 Formula:C19H34O2 CAS:2462-85-3 MolWeight:294 RetIndex:2093
CompName:9,12-Octadecadienoic acid, methyl ester $$ 9,12-Octadecenoic acid, methyl ester $$ Methyl octadeca-9,12-dienoate $$ Methyl 9,12-octadecadi

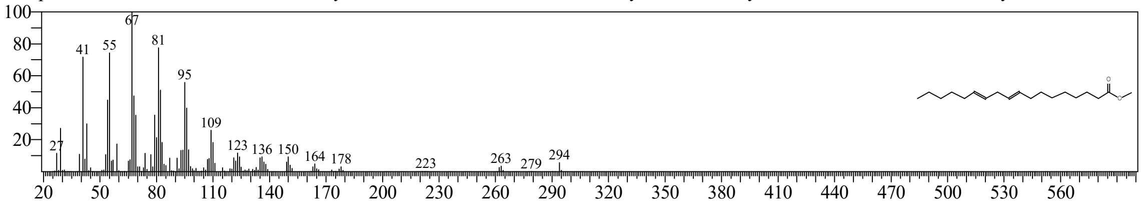

Hit#:3 Entry:27995 Library:NIST14s.lib
SI:93 Formula:C19H34O2 CAS:2566-97-4 MolWeight:294 RetIndex:2093
CompName:9,12-Octadecadienoic acid, methyl ester, (E,E)- $$ Linolelaidic acid, methyl ester $$ Methyl linolelaidate $$ Methyl trans,trans-9,12-octadecadi

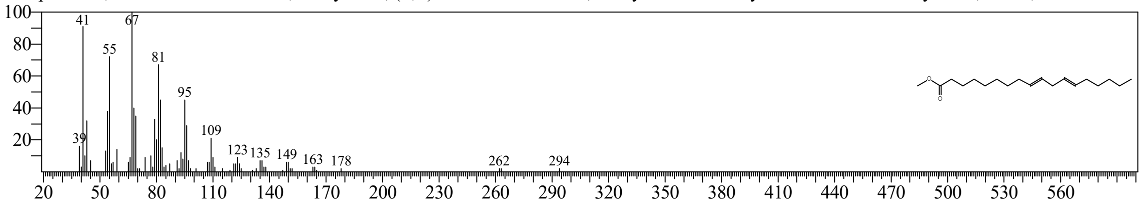

Hit#:4 Entry:32583 Library:NIST17s.lib
SI:93 Formula:C19H34O2 CAS:2566-97-4 MolWeight:294 RetIndex:2093
CompName:9,12-Octadecadienoic acid, methyl ester, (E,E)- $$ Linolelaidic acid, methyl ester $$ Methyl linolelaidate $$ Methyl trans,trans-9,12-octadecadi

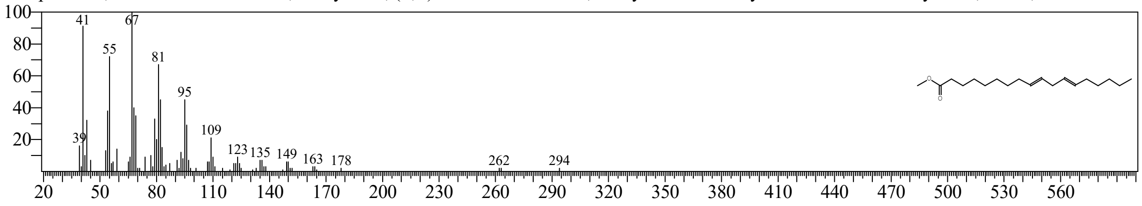

Hit#:5 Entry:151102 Library:NIST14.lib
SI:93 Formula:C21H38O2 CAS:2463-02-7 MolWeight:322 RetIndex:2292
CompName:11,14-Eicosadienoic acid, methyl ester $$ Methyl (11E,14E)-11,14-icosadienoate # $$

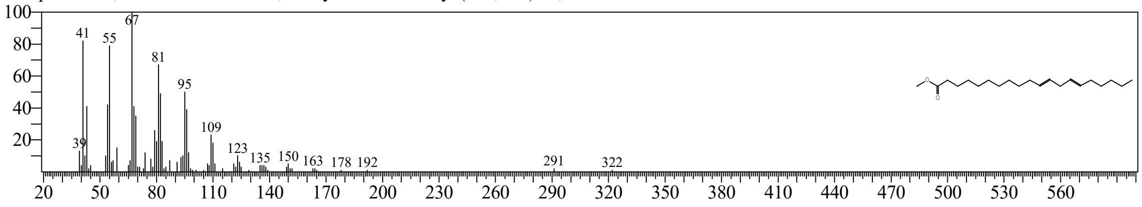

<< Target >>
Line#:10 R.Time:28.042(Scan#:2166) MassPeaks:298
RawMode:Averaged 28.033-28.050(2165-2167) BasePeak:67.05(1100)
BG Mode:Calc. from Peak Group 1 - Event 1

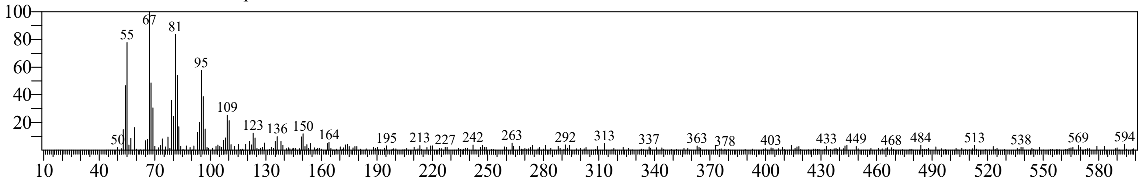

Hit#:1 Entry:137663 Library:NIST17-1.lib
SI:93 Formula:C19H34O2 CAS:2462-85-3 MolWeight:294 RetIndex:2093
CompName:9,12-Octadecadienoic acid, methyl ester $$ 9,12-Octadecenoic acid, methyl ester $$ Methyl octadeca-9,12-dienoate $$ Methyl 9,12-octadecadi

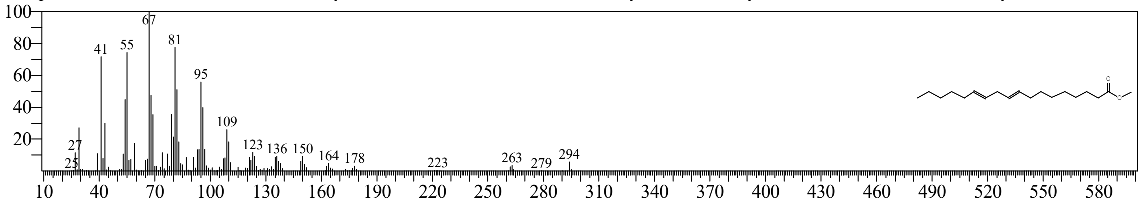

Hit#:2 Entry:125931 Library:NIST14.lib
SI:93 Formula:C19H34O2 CAS:2462-85-3 MolWeight:294 RetIndex:2093
CompName:9,12-Octadecadienoic acid, methyl ester $$ 9,12-Octadecenoic acid, methyl ester $$ Methyl octadeca-9,12-dienoate $$ Methyl 9,12-octadecadi

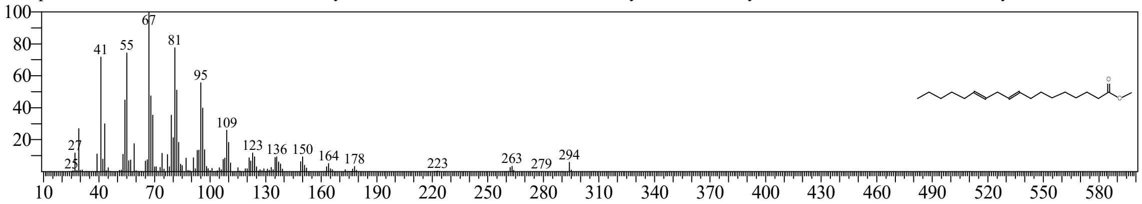

Hit#:3 Entry:32588 Library:NIST17s.lib
SI:92 Formula:C19H34O2 CAS:112-63-0 MolWeight:294 RetIndex:2093
CompName:9,12-Octadecadienoic acid (Z,Z)-, methyl ester $$ Linoleic acid, methyl ester $$ Methyl cis,cis-9,12-octadecadienoate $$ Methyl linoleate $$ M

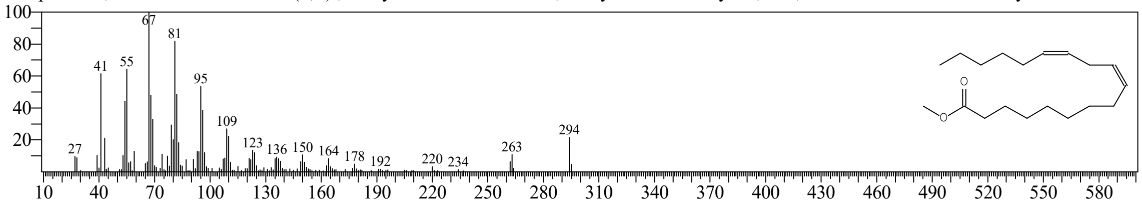

Hit#:4 Entry:27999 Library:NIST14s.lib
SI:92 Formula:C19H34O2 CAS:112-63-0 MolWeight:294 RetIndex:2093
CompName:9,12-Octadecadienoic acid (Z,Z)-, methyl ester $$ Linoleic acid, methyl ester $$ Methyl cis,cis-9,12-octadecadienoate $$ Methyl linoleate $$ M

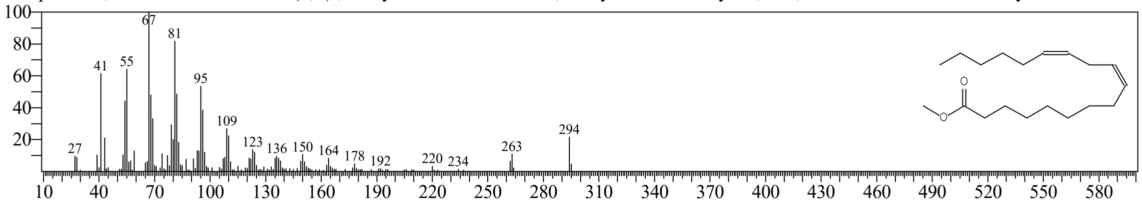

Hit#:5 Entry:28000 Library:NIST14s.lib
SI:92 Formula:C19H34O2 CAS:112-63-0 MolWeight:294 RetIndex:2093
CompName:9,12-Octadecadienoic acid (Z,Z)-, methyl ester $$ Linoleic acid, methyl ester $$ Methyl cis,cis-9,12-octadecadienoate $$ Methyl linoleate $$ M

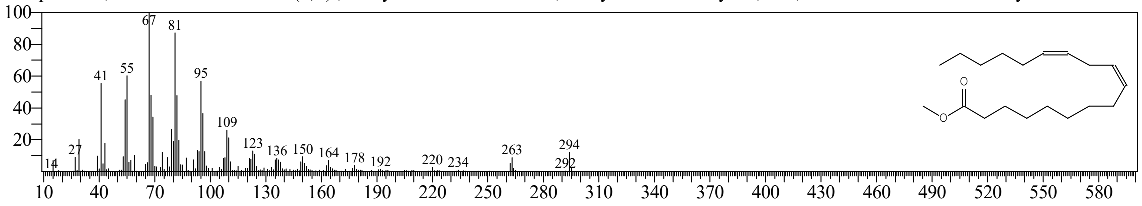

<< Target >>
Line#:11   R.Time:28.458(Scan#:2216) MassPeaks:398
RawMode:Averaged 28.450-28.467(2215-2217) BasePeak:67.10(405512)
BG Mode:Calc. from Peak Group 1 - Event 1

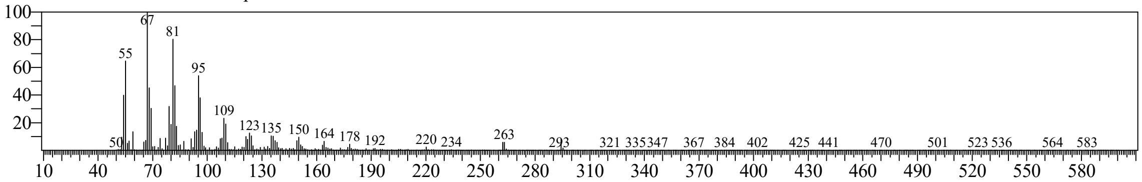

Hit#:1 Entry:32588 Library:NIST17s.lib
SI:97 Formula:C19H34O2 CAS:112-63-0 MolWeight:294 RetIndex:2093
CompName:9,12-Octadecadienoic acid (Z,Z)-, methyl ester $$ Linoleic acid, methyl ester $$ Methyl cis,cis-9,12-octadecadienoate $$ Methyl linoleate $$ M

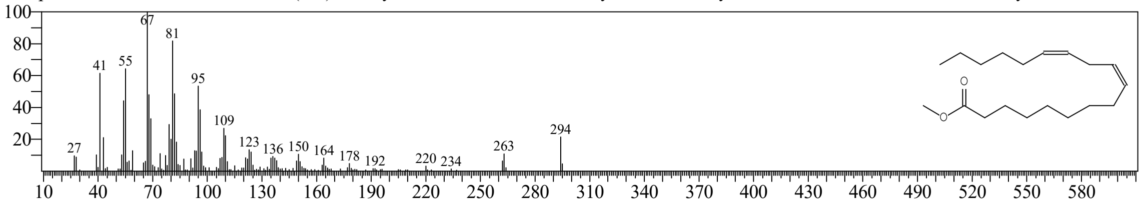

Hit#:2 Entry:27999 Library:NIST14s.lib
SI:97 Formula:C19H34O2 CAS:112-63-0 MolWeight:294 RetIndex:2093
CompName:9,12-Octadecadienoic acid (Z,Z)-, methyl ester $$ Linoleic acid, methyl ester $$ Methyl cis,cis-9,12-octadecadienoate $$ Methyl linoleate $$ M

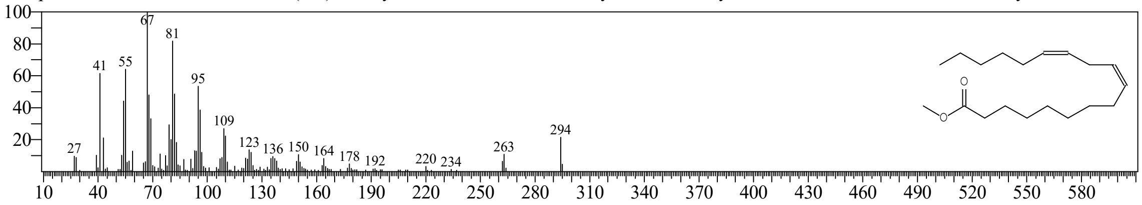

Hit#:3 Entry:32589 Library:NIST17s.lib
SI:97 Formula:C19H34O2 CAS:112-63-0 MolWeight:294 RetIndex:2093
CompName:9,12-Octadecadienoic acid (Z,Z)-, methyl ester $$ Linoleic acid, methyl ester $$ Methyl cis,cis-9,12-octadecadienoate $$ Methyl linoleate $$ M

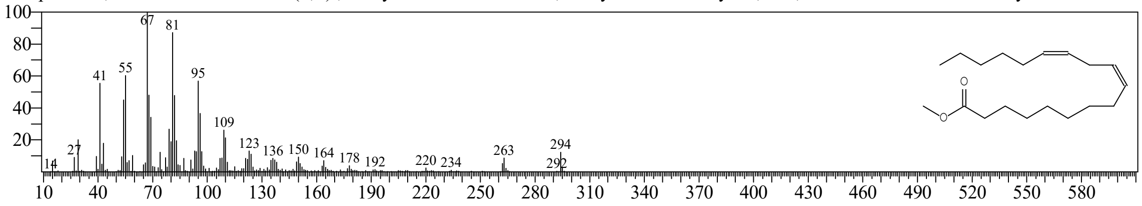

Hit#:4 Entry:28000 Library:NIST14s.lib
SI:97 Formula:C19H34O2 CAS:112-63-0 MolWeight:294 RetIndex:2093
CompName:9,12-Octadecadienoic acid (Z,Z)-, methyl ester $$ Linoleic acid, methyl ester $$ Methyl cis,cis-9,12-octadecadienoate $$ Methyl linoleate $$ M

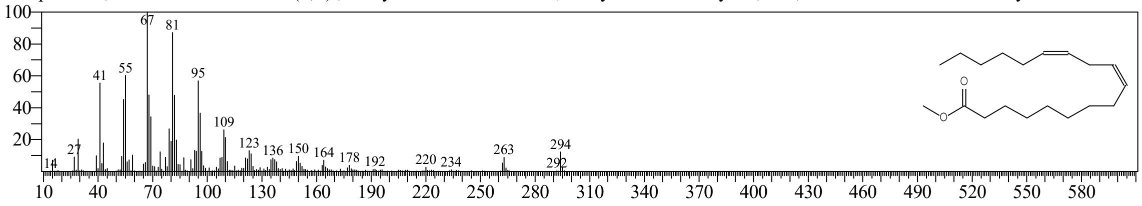

Hit#:5 Entry:125931 Library:NIST14.lib
SI:96 Formula:C19H34O2 CAS:2462-85-3 MolWeight:294 RetIndex:2093
CompName:9,12-Octadecadienoic acid, methyl ester $$ 9,12-Octadecenoic acid, methyl ester $$ Methyl octadeca-9,12-dienoate $$ Methyl 9,12-octadecadi

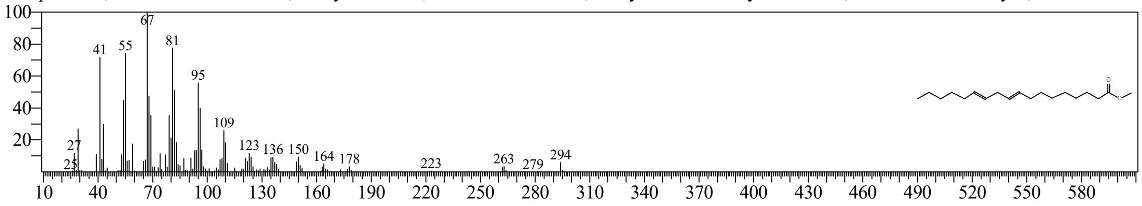

<< Target >>
Line#:12 R.Time:29.375(Scan#:2326) MassPeaks:285
RawMode:Averaged 29.367-29.383(2325-2327) BasePeak:74.05(6942)
BG Mode:Calc. from Peak Group 1 - Event 1

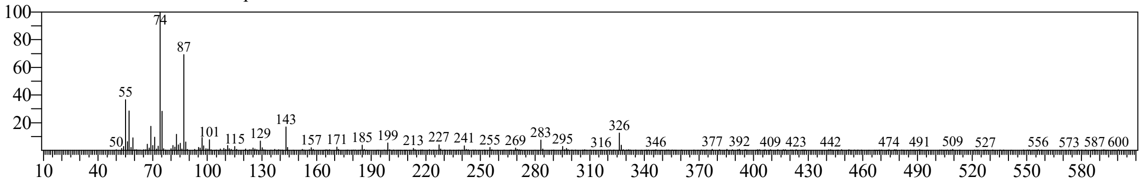

Hit#:1 Entry:34726 Library:NIST17s.lib
SI:97 Formula:C21H42O2 CAS:1120-28-1 MolWeight:326 RetIndex:2276
CompName:Eicosanoic acid, methyl ester $$ Methyl arachisate $$ Methyl eicosanoate $$ Arachidic acid methyl ester $$ Kemester 2050 $$ Methyl [illegible]

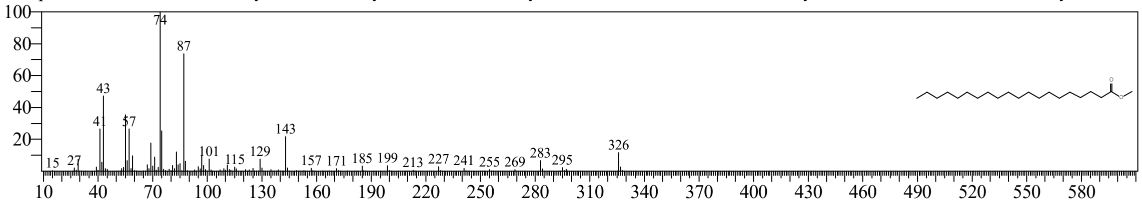

Hit#:2 Entry:170238 Library:NIST17-1.lib
SI:96 Formula:C21H42O2 CAS:0-00-0 MolWeight:326 RetIndex:2212
CompName:Methyl 18-methylnonadecanoate

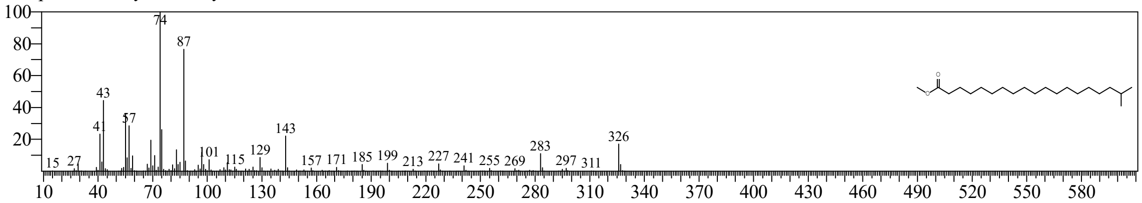

Hit#:3 Entry:154704 Library:NIST14.lib
SI:96 Formula:C21H42O2 CAS:0-00-0 MolWeight:326 RetIndex:2212
CompName:Methyl 18-methylnonadecanoate

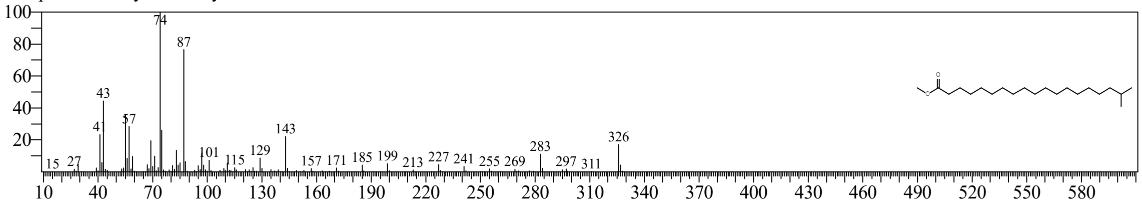

Hit#:4 Entry:29931 Library:NIST14s.lib
SI:95 Formula:C21H42O2 CAS:1120-28-1 MolWeight:326 RetIndex:2276
CompName:Eicosanoic acid, methyl ester $$ Methyl arachisate $$ Methyl eicosanoate $$ Arachidic acid methyl ester $$ Kemester 2050 $$ Methyl [illegible]

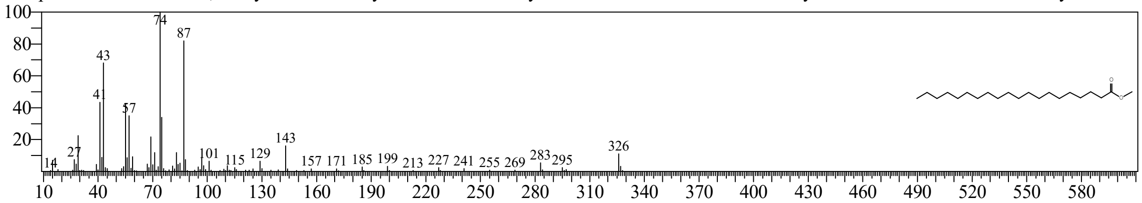

Hit#:5 Entry:34724 Library:NIST17s.lib
SI:95 Formula:C21H42O2 CAS:1120-28-1 MolWeight:326 RetIndex:2276
CompName:Eicosanoic acid, methyl ester $$ Methyl arachisate $$ Methyl eicosanoate $$ Arachidic acid methyl ester $$ Kemester 2050 $$ Methyl [illegible]

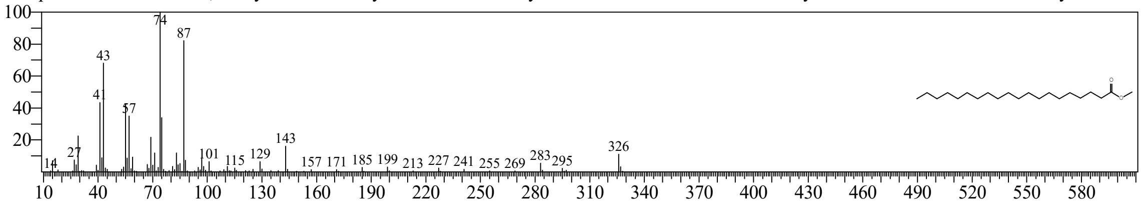

<< Target >>
Line#:13 R.Time:29.900(Scan#:2389) MassPeaks:347
RawMode:Averaged 29.892-29.908(2388-2390) BasePeak:79.10(5766)
BG Mode:Calc. from Peak Group 1 - Event 1

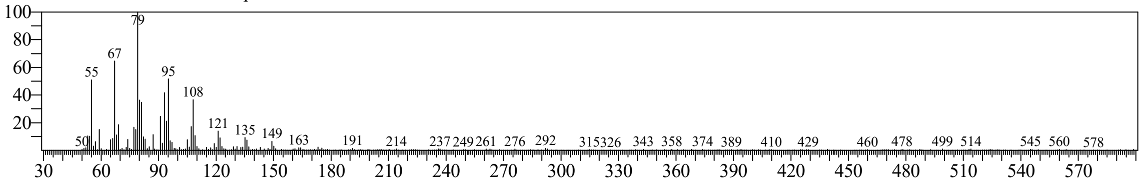

Hit#:1 Entry:29609 Library:NIST14s.lib
SI:95 Formula:C21H36O2 CAS:55682-88-7 MolWeight:320 RetIndex:2300
CompName:11,14,17-Eicosatrienoic acid, methyl ester $$ Methyl 11,14,17-icosatrienoate $$ Methyl 11,14,17-eicosatrienoate $$

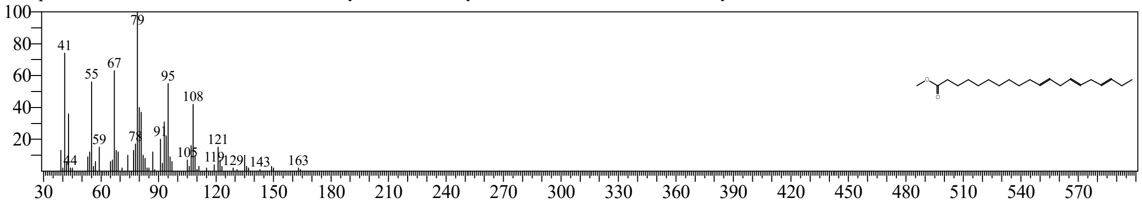

Hit#:2 Entry:34362 Library:NIST17s.lib
SI:95 Formula:C21H36O2 CAS:55682-88-7 MolWeight:320 RetIndex:2300
CompName:11,14,17-Eicosatrienoic acid, methyl ester $$ Methyl 11,14,17-icosatrienoate $$ Methyl 11,14,17-eicosatrienoate $$

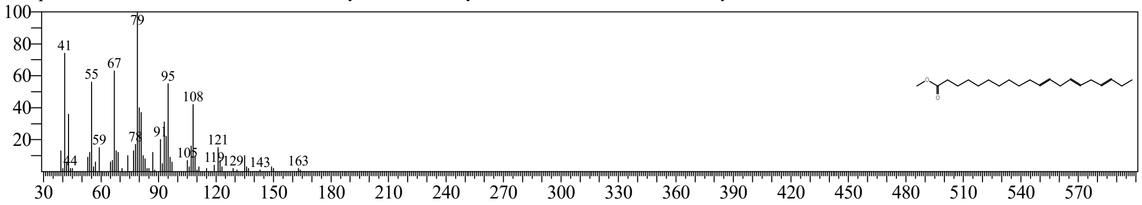

Hit#:3 Entry:32438 Library:NIST17s.lib
SI:95 Formula:C19H32O2 CAS:301-00-8 MolWeight:292 RetIndex:2101
CompName:9,12,15-Octadecatrienoic acid, methyl ester, (Z,Z,Z)- $$ Linolenic acid, methyl ester $$ Methyl all-cis-9,12,15-octadecatrienoate $$ Methyl lino

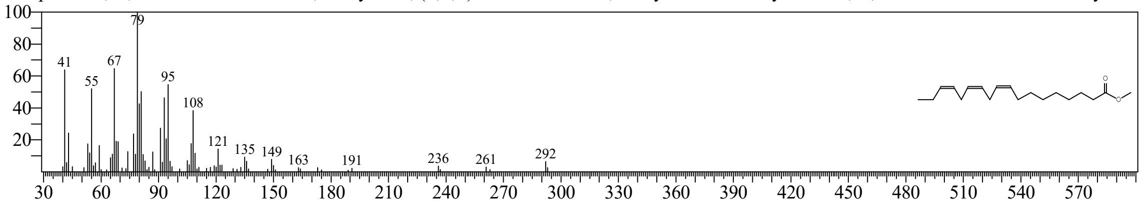

Hit#:4 Entry:32437 Library:NIST17s.lib
SI:95 Formula:C19H32O2 CAS:301-00-8 MolWeight:292 RetIndex:2101
CompName:9,12,15-Octadecatrienoic acid, methyl ester, (Z,Z,Z)- $$ Linolenic acid, methyl ester $$ Methyl all-cis-9,12,15-octadecatrienoate $$ Methyl lino

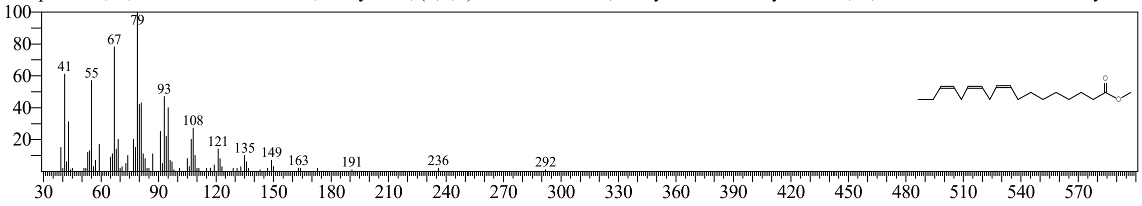

Hit#:5 Entry:27872 Library:NIST14s.lib
SI:95 Formula:C19H32O2 CAS:301-00-8 MolWeight:292 RetIndex:2101
CompName:9,12,15-Octadecatrienoic acid, methyl ester, (Z,Z,Z)- $$ Linolenic acid, methyl ester $$ Methyl all-cis-9,12,15-octadecatrienoate $$ Methyl lino

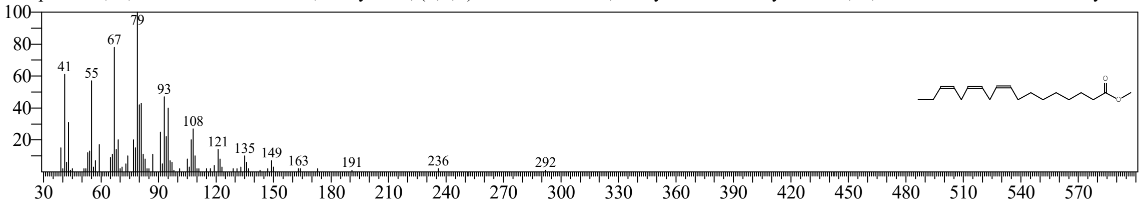

<< Target >>
Line#:14 R.Time:30.383(Scan#:2447) MassPeaks:353
RawMode:Averaged 30.375-30.392(2446-2448) BasePeak:79.10(4288)
BG Mode:Calc. from Peak Group 1 - Event 1

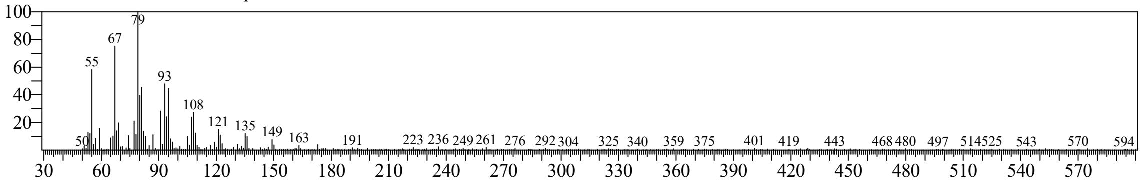

Hit#:1 Entry:27872 Library:NIST14s.lib
SI:96 Formula:C19H32O2 CAS:301-00-8 MolWeight:292 RetIndex:2101
CompName:9,12,15-Octadecatrienoic acid, methyl ester, (Z,Z,Z)- $$ Linolenic acid, methyl ester $$ Methyl all-cis-9,12,15-octadecatrienoate $$ Methyl lino

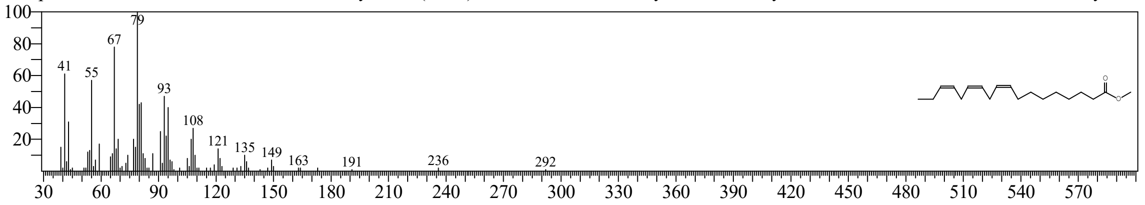

Hit#:2 Entry:32437 Library:NIST17s.lib
SI:96 Formula:C19H32O2 CAS:301-00-8 MolWeight:292 RetIndex:2101
CompName:9,12,15-Octadecatrienoic acid, methyl ester, (Z,Z,Z)- $$ Linolenic acid, methyl ester $$ Methyl all-cis-9,12,15-octadecatrienoate $$ Methyl lino

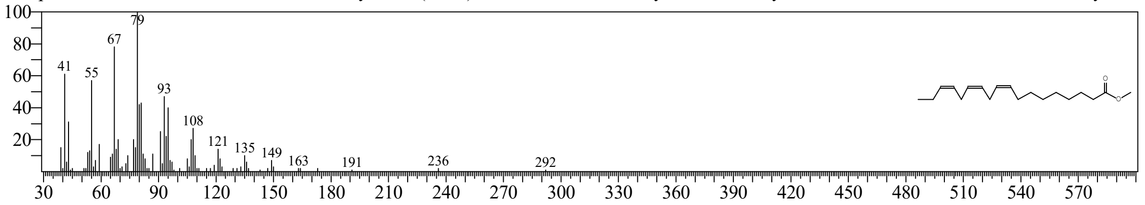

Hit#:3 Entry:121536 Library:NIST17-1.lib
SI:94 Formula:C18H30O2 CAS:0-00-0 MolWeight:278 RetIndex:2002
CompName:Methyl 8,11,14-heptadecatrienoate

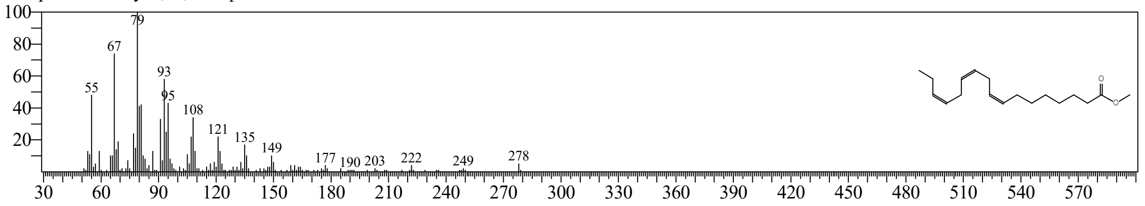

Hit#:4 Entry:111654 Library:NIST14.lib
SI:94 Formula:C18H30O2 CAS:0-00-0 MolWeight:278 RetIndex:2002
CompName:Methyl 8,11,14-heptadecatrienoate

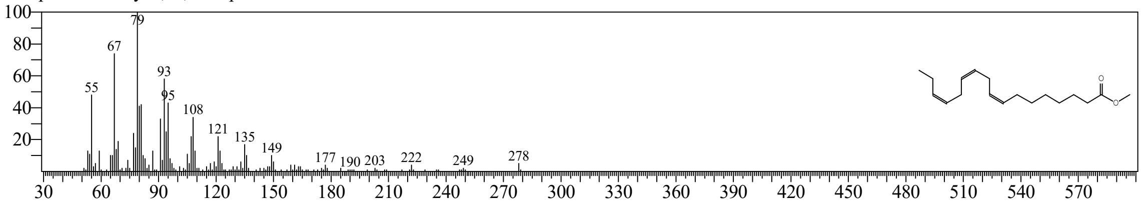

Hit#:5 Entry:32438 Library:NIST17s.lib
SI:94 Formula:C19H32O2 CAS:301-00-8 MolWeight:292 RetIndex:2101
CompName:9,12,15-Octadecatrienoic acid, methyl ester, (Z,Z,Z)- $$ Linolenic acid, methyl ester $$ Methyl all-cis-9,12,15-octadecatrienoate $$ Methyl lino

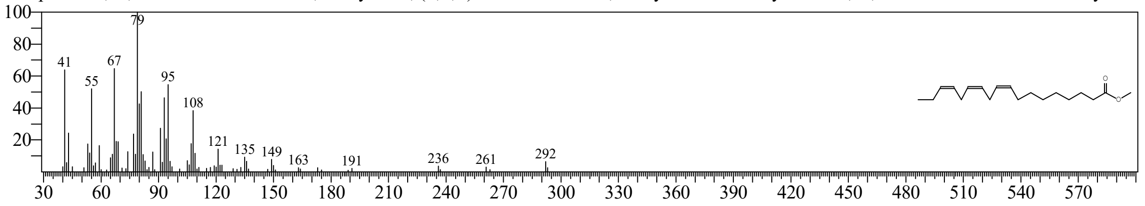

<< Target >>
Line#:15 R.Time:30.683(Scan#:2483) MassPeaks:384
RawMode:Averaged 30.675-30.692(2482-2484) BasePeak:79.05(78813)
BG Mode:Calc. from Peak Group 1 - Event 1

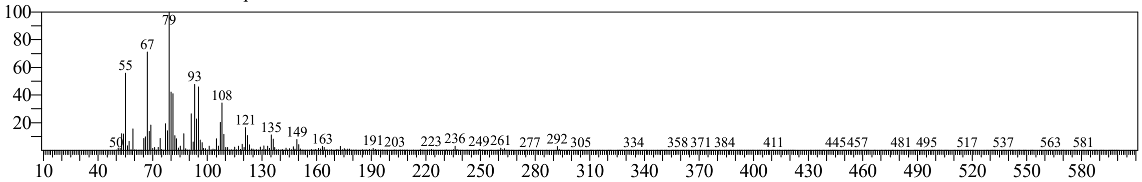

Hit#:1 Entry:27872 Library:NIST14s.lib
SI:96 Formula:C19H32O2 CAS:301-00-8 MolWeight:292 RetIndex:2101
CompName:9,12,15-Octadecatrienoic acid, methyl ester, (Z,Z,Z)- $$ Linolenic acid, methyl ester $$ Methyl all-cis-9,12,15-octadecatrienoate $$ Methyl lino

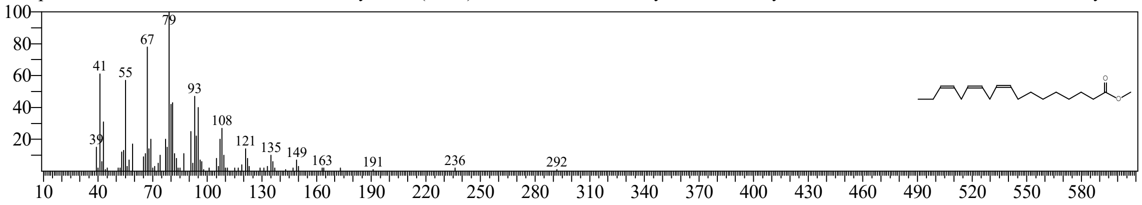

Hit#:2 Entry:32437 Library:NIST17s.lib
SI:96 Formula:C19H32O2 CAS:301-00-8 MolWeight:292 RetIndex:2101
CompName:9,12,15-Octadecatrienoic acid, methyl ester, (Z,Z,Z)- $$ Linolenic acid, methyl ester $$ Methyl all-cis-9,12,15-octadecatrienoate $$ Methyl lino

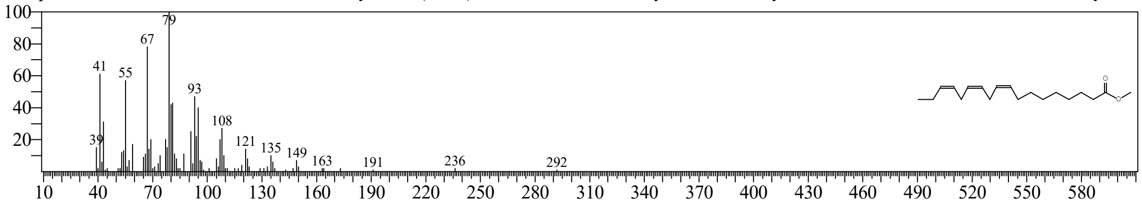

Hit#:3 Entry:32438 Library:NIST17s.lib
SI:96 Formula:C19H32O2 CAS:301-00-8 MolWeight:292 RetIndex:2101
CompName:9,12,15-Octadecatrienoic acid, methyl ester, (Z,Z,Z)- $$ Linolenic acid, methyl ester $$ Methyl all-cis-9,12,15-octadecatrienoate $$ Methyl lino

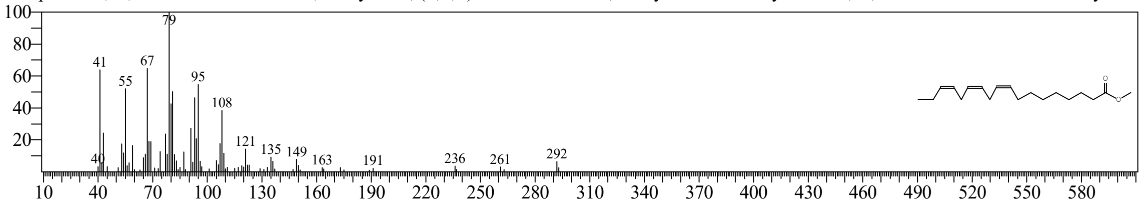

Hit#:4 Entry:124228 Library:NIST14.lib
SI:95 Formula:C19H32O2 CAS:301-00-8 MolWeight:292 RetIndex:2101
CompName:9,12,15-Octadecatrienoic acid, methyl ester, (Z,Z,Z)- $$ Linolenic acid, methyl ester $$ Methyl all-cis-9,12,15-octadecatrienoate $$ Methyl lino

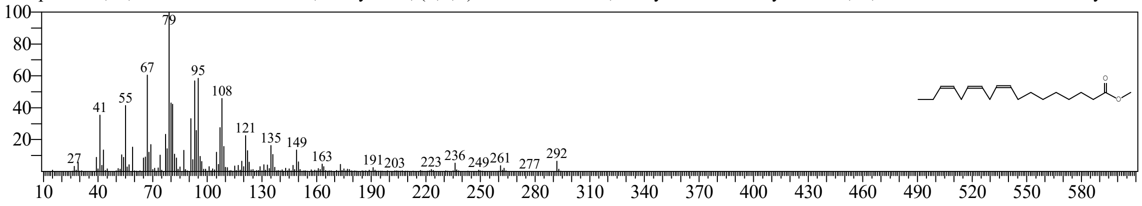

Hit#:5 Entry:135724 Library:NIST17-1.lib
SI:95 Formula:C19H32O2 CAS:301-00-8 MolWeight:292 RetIndex:2101
CompName:9,12,15-Octadecatrienoic acid, methyl ester, (Z,Z,Z)- $$ Linolenic acid, methyl ester $$ Methyl all-cis-9,12,15-octadecatrienoate $$ Methyl lino

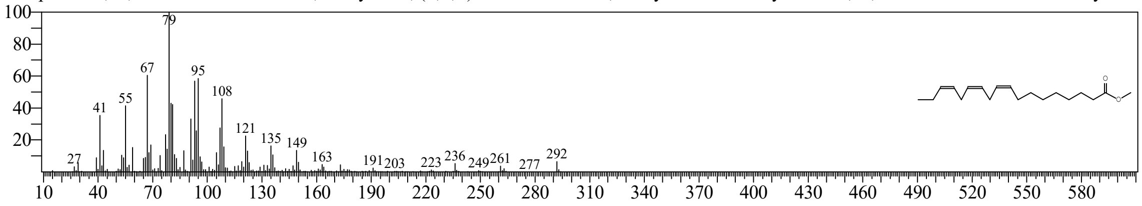

<< Target >>
Line#:16   R.Time:31.008(Scan#:2522)   MassPeaks:359
RawMode:Averaged 31.000-31.017(2521-2523) BasePeak:67.05(9440)
BG Mode:Calc. from Peak   Group 1 - Event 1

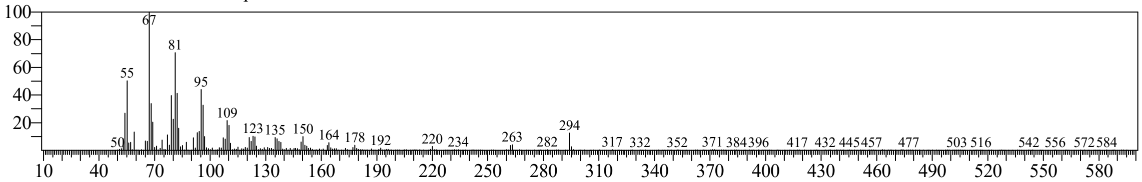

Hit#:1   Entry:32587   Library:NIST17s.lib
SI:96   Formula:C19H34O2   CAS:13038-47-6   MolWeight:294   RetIndex:2093
CompName:9,11-Octadecadienoic acid, methyl ester, (E,E)- $$ Methyl trans-9,trans-11-octadecadienoate $$ Methyl trans,trans-9,11-octadecadienoate $$ M

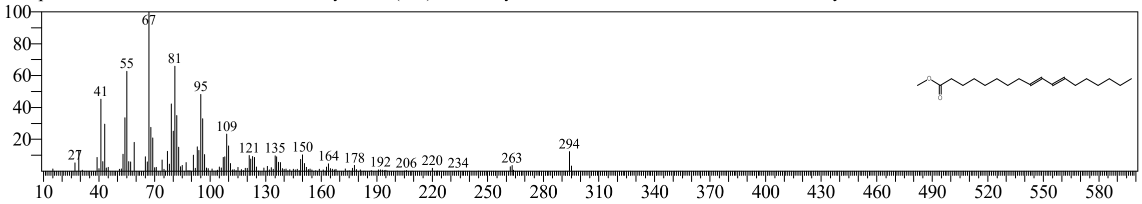

Hit#:2   Entry:125932   Library:NIST14.lib
SI:96   Formula:C19H34O2   CAS:0-00-0   MolWeight:294   RetIndex:2093
CompName:Methyl 9-cis,11-trans-octadecadienoate

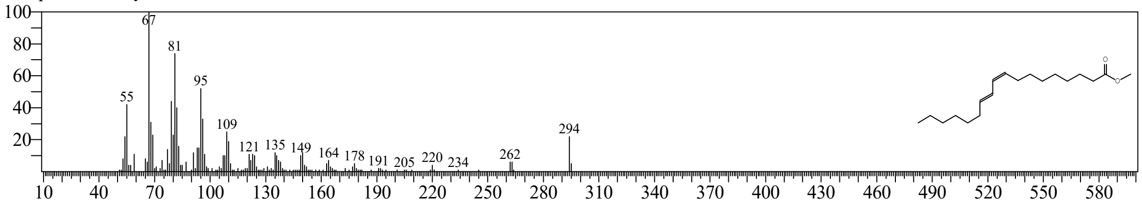

Hit#:3   Entry:137666   Library:NIST17-1.lib
SI:96   Formula:C19H34O2   CAS:13058-52-1   MolWeight:294   RetIndex:2093
CompName:Methyl 9-cis,11-trans-octadecadienoate

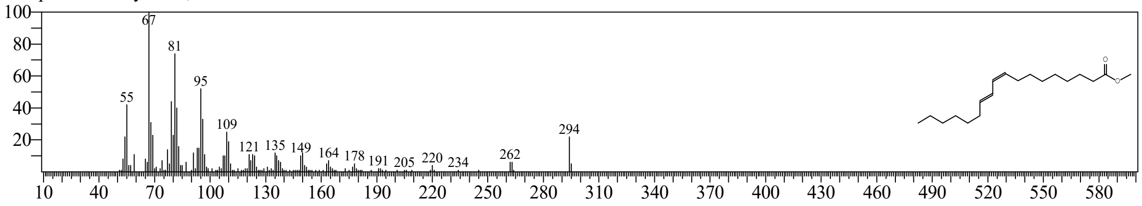

Hit#:4   Entry:137664   Library:NIST17-1.lib
SI:96   Formula:C19H34O2   CAS:0-00-0   MolWeight:294   RetIndex:2093
CompName:Methyl 10-trans,12-cis-octadecadienoate

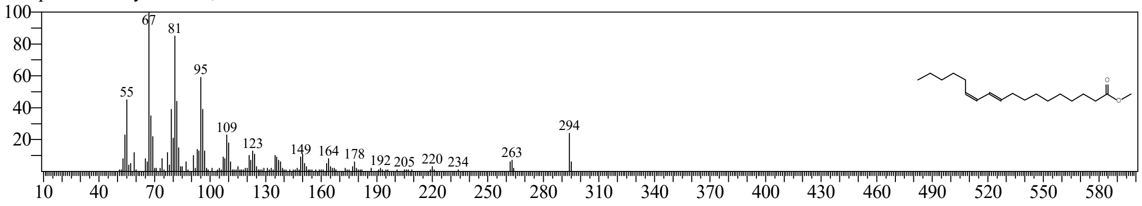

Hit#:5   Entry:125933   Library:NIST14.lib
SI:96   Formula:C19H34O2   CAS:0-00-0   MolWeight:294   RetIndex:2093
CompName:Methyl 10-trans,12-cis-octadecadienoate

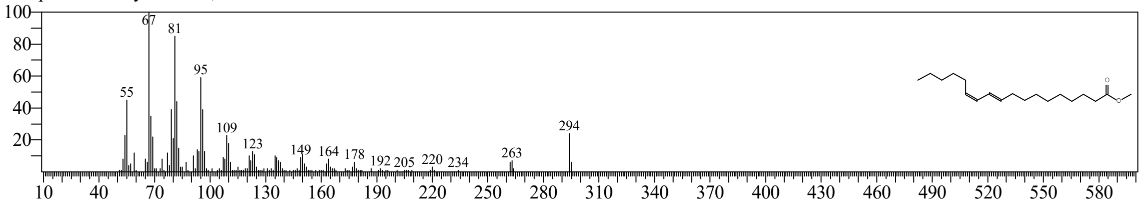

<< Target >>
Line#:17 R.Time:31.342(Scan#:2562) MassPeaks:340
RawMode:Averaged 31.333-31.350(2561-2563) BasePeak:67.05(10466)
BG Mode:Calc. from Peak Group 1 - Event 1

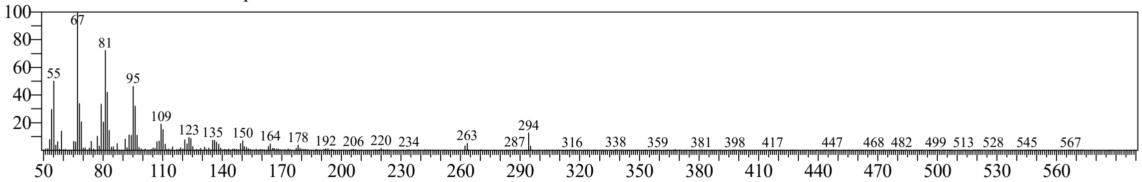

Hit#:1 Entry:137664 Library:NIST17-1.lib
SI:95 Formula:C19H34O2 CAS:0-00-0 MolWeight:294 RetIndex:2093
CompName:Methyl 10-trans,12-cis-octadecadienoate

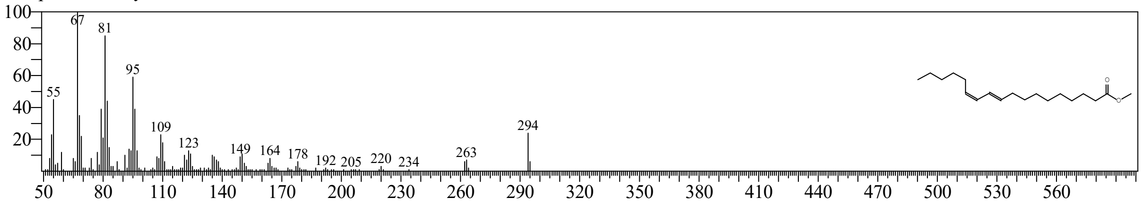

Hit#:2 Entry:27998 Library:NIST14s.lib
SI:95 Formula:C19H34O2 CAS:56554-61-1 MolWeight:294 RetIndex:2093
CompName:11,14-Octadecadienoic acid, methyl ester $$ Methyl (11E,14E)-11,14-octadecadienoate # $$

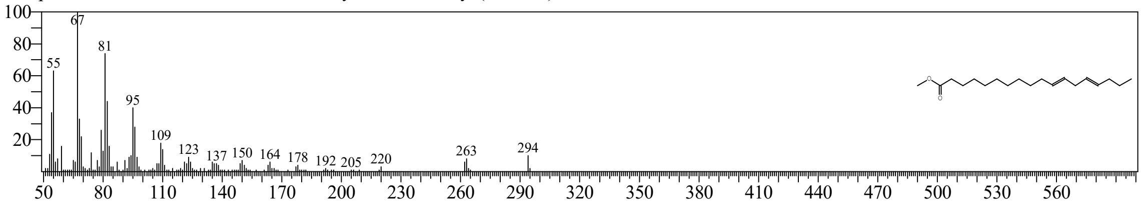

Hit#:3 Entry:32586 Library:NIST17s.lib
SI:95 Formula:C19H34O2 CAS:56554-61-1 MolWeight:294 RetIndex:2093
CompName:11,14-Octadecadienoic acid, methyl ester $$ Methyl (11E,14E)-11,14-octadecadienoate # $$

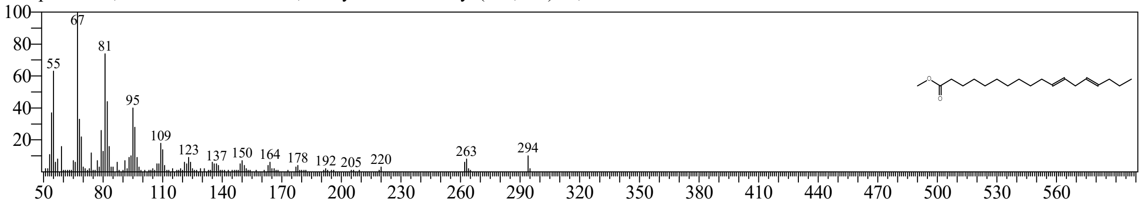

Hit#:4 Entry:125933 Library:NIST14.lib
SI:95 Formula:C19H34O2 CAS:0-00-0 MolWeight:294 RetIndex:2093
CompName:Methyl 10-trans,12-cis-octadecadienoate

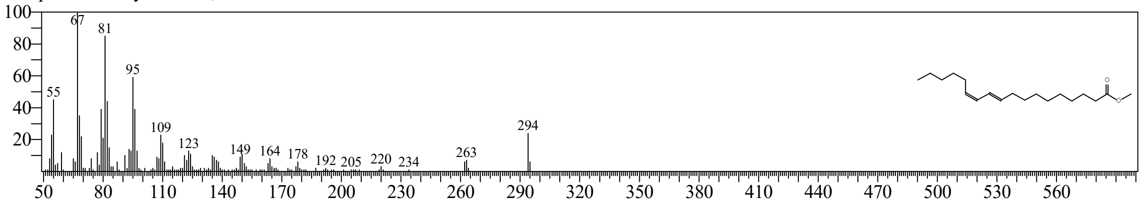

Hit#:5 Entry:125932 Library:NIST14.lib
SI:95 Formula:C19H34O2 CAS:0-00-0 MolWeight:294 RetIndex:2093
CompName:Methyl 9-cis,11-trans-octadecadienoate

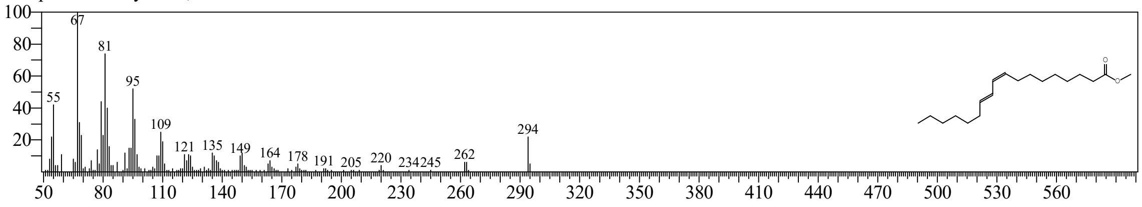

<< Target >>
Line#:18 R.Time:33.608(Scan#:2834) MassPeaks:290
RawMode:Averaged 33.600-33.617(2833-2835) BasePeak:74.05(6575)
BG Mode:Calc. from Peak Group 1 - Event 1

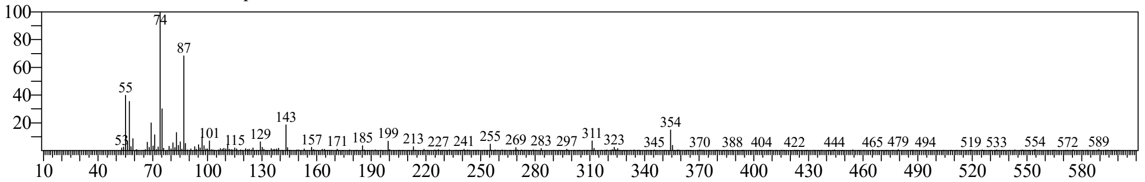

Hit#:1 Entry:196443 Library:NIST17-1.lib
SI:96 Formula:C23H46O2 CAS:929-77-1 MolWeight:354 RetIndex:2475
CompName:Docosanoic acid, methyl ester $$ Behenic acid, methyl ester $$ Methyl behenate $$ Methyl docosanoate $$ n-Docosanoic acid methyl ester $$

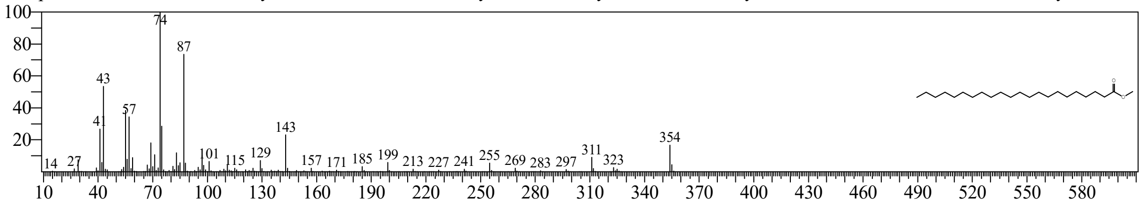

Hit#:2 Entry:178051 Library:NIST14.lib
SI:96 Formula:C23H46O2 CAS:929-77-1 MolWeight:354 RetIndex:2475
CompName:Docosanoic acid, methyl ester $$ Behenic acid, methyl ester $$ Methyl behenate $$ Methyl docosanoate $$ n-Docosanoic acid methyl ester $$

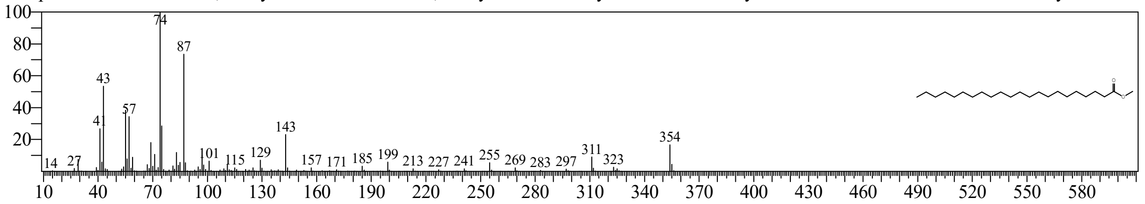

Hit#:3 Entry:31086 Library:NIST14s.lib
SI:95 Formula:C23H46O2 CAS:929-77-1 MolWeight:354 RetIndex:2475
CompName:Docosanoic acid, methyl ester $$ Behenic acid, methyl ester $$ Methyl behenate $$ Methyl docosanoate $$ n-Docosanoic acid methyl ester $$

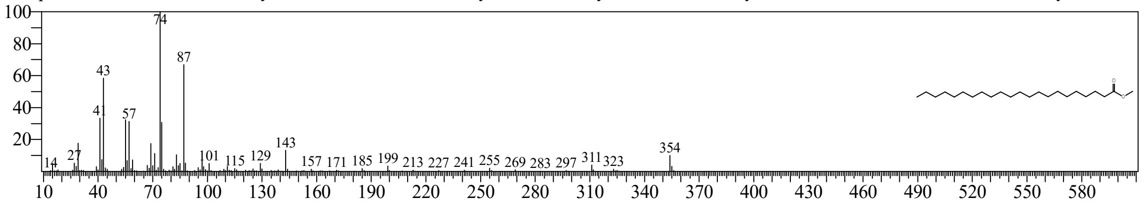

Hit#:4 Entry:36091 Library:NIST17s.lib
SI:95 Formula:C23H46O2 CAS:929-77-1 MolWeight:354 RetIndex:2475
CompName:Docosanoic acid, methyl ester $$ Behenic acid, methyl ester $$ Methyl behenate $$ Methyl docosanoate $$ n-Docosanoic acid methyl ester $$

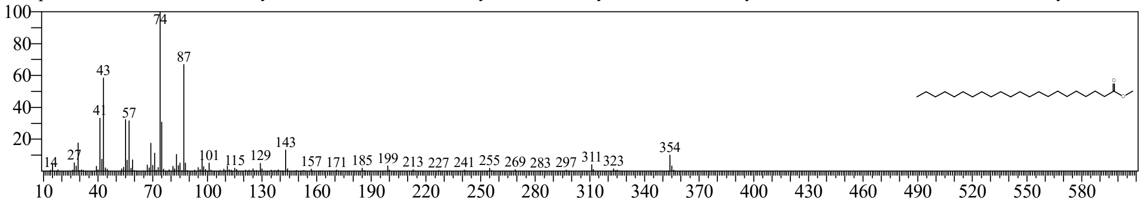

Hit#:5 Entry:31087 Library:NIST14s.lib
SI:95 Formula:C23H46O2 CAS:929-77-1 MolWeight:354 RetIndex:2475
CompName:Docosanoic acid, methyl ester $$ Behenic acid, methyl ester $$ Methyl behenate $$ Methyl docosanoate $$ n-Docosanoic acid methyl ester $$

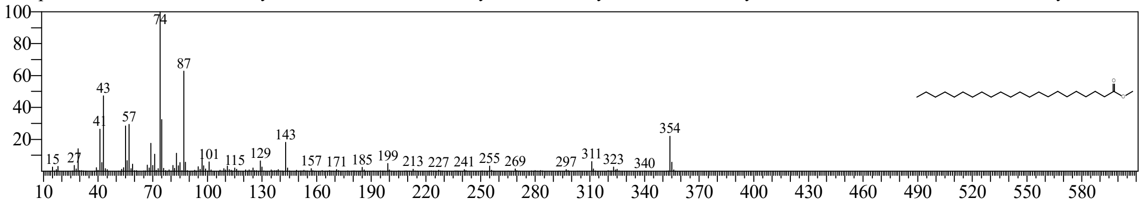

Supplement: Supplementary file 1 [file molecules-31-02891-s001.zip › 56d-QG200-3.pdf]
