# Supplementary material for: Selective Inhibition of Lipid Oxidation by a Quercetagetin-Rich Extract from Tagetes erecta: A Chemometric Approach Using OPLS-DA and Selectivity Index Analysis
Source: Molecules. 2026 Aug 19;31(16):2891. doi: 10.3390/molecules31162891 (PMC13515886; doi:10.3390/molecules31162891)

QG200-3-7d

<<Column>>
Name : HP-88
Thickness : 0.20um
Length : 100.0m
Inside Diameter : 0.25mm

Method

===== Analytical Line 1 =====

Column Oven Temp.	:120.0 °C	
Injection Temp.	:250.00 °C	
Injection Mode	:Split	
Column Flow	:1.04 mL/min	
Split Ratio	:20.0	
Oven Temp. Program		
Rate	Temperature(°C)	Hold Time(min)
-	120.0	1.00
10.00	175.0	10.00
5.00	210.0	5.00
5.00	230.0	15.00

[GCMS-QP2010]
IonSourceTemp :200.00 °C
Interface Temp. :220.00 °C
m/z scanning range：45～500m/z

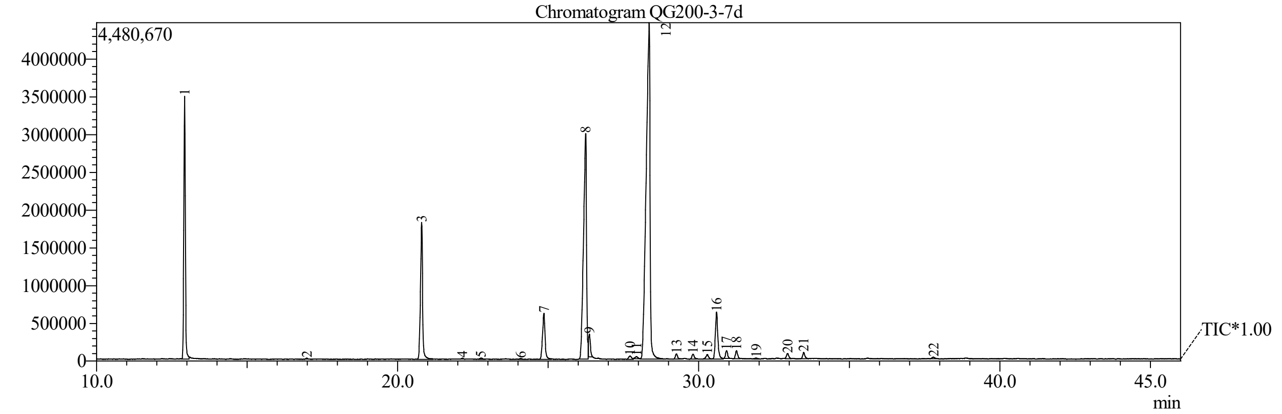

Peak Report TIC

Peak#	R.Time	Area	Area%	Height	Height%	A/H	Name
1	12.929	12351133	13.16	3479094	23.23	3.55	Undecanoic acid, methyl ester
2	16.989	55839	0.06	13137	0.09	4.25	Methyl tetradecanoate
3	20.801	8880013	9.46	1813863	12.11	4.90	Hexadecanoic acid, methyl ester
4	22.152	59727	0.06	13453	0.09	4.44	9-Hexadecenoic acid, methyl ester, (Z)-
5	22.767	90588	0.10	16984	0.11	5.33	Heptadecanoic acid, methyl ester
6	24.091	55881	0.06	12175	0.08	4.59	cis-10-Heptadecenoic acid, methyl ester
7	24.860	3649279	3.89	611985	4.09	5.96	Methyl stearate
8	26.246	20504113	21.85	2982033	19.91	6.88	9-Octadecenoic acid, methyl ester, (E)-
9	26.368	1203970	1.28	299863	2.00	4.02	11-Octadecenoic acid, methyl ester
10	27.714	292106	0.31	44017	0.29	6.64	9,12-Octadecadienoic acid, methyl ester
11	27.934	190963	0.20	28733	0.19	6.65	9,12-Octadecadienoic acid, methyl ester
12	28.353	39835862	42.44	4451009	29.71	8.95	9,12-Octadecadienoic acid (Z,Z)-, methyl estei
13	29.260	356631	0.38	67329	0.45	5.30	Methyl 18-methylnonadecanoate
14	29.807	368422	0.39	67029	0.45	5.50	9,12,15-Octadecatrienoic acid, methyl ester, (Z

Peak#	R.Time	Area	Area%	Height	Height%	A/H	Name
15	30.284	373893	0.40	60053	0.40	6.23	9,12,15-Octadecatrienoic acid, methyl ester, (Z
16	30.591	3550257	3.78	622203	4.15	5.71	9,12,15-Octadecatrienoic acid, methyl ester, (Z
17	30.924	515594	0.55	104153	0.70	4.95	Methyl 9-cis,11-trans-octadecadienoate
18	31.253	540956	0.58	105135	0.70	5.15	Methyl 10-trans,12-cis-octadecadienoate
19	31.907	59549	0.06	12221	0.08	4.87	9,11-Octadecadienoic acid, methyl ester, (E,E)
20	32.952	361597	0.39	69142	0.46	5.23	Cis-3-ethyl-endo-tricyclo[5.2.1.0(2.6)]decane
21	33.483	439320	0.47	84146	0.56	5.22	Docosanoic acid, methyl ester
22	37.795	121992	0.13	21426	0.14	5.69	Tetracosanoic acid, methyl ester
		93857685	100.00	14979183	100.00		

Library

<< Target >>
Line#:1 R.Time:12.925(Scan#:352) MassPeaks:350
RawMode:Averaged 12.917-12.933(351-353) BasePeak:74.00(1087285)
BG Mode:Calc. from Peak Group 1 - Event 1

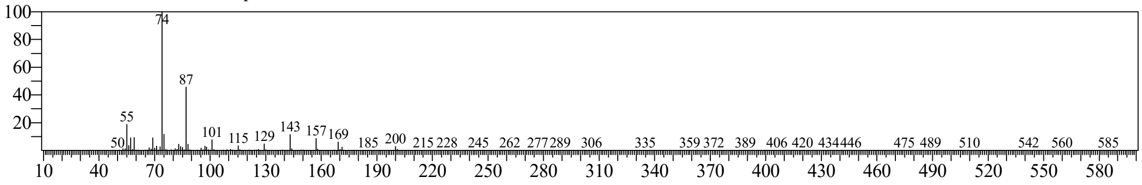

Hit#:1 Entry:21619 Library:NIST17s.lib
SI:96 Formula:C12H24O2 CAS:1731-86-8 MolWeight:200 RetIndex:1381
CompName:Undecanoic acid, methyl ester $$ Methyl undecanoate $$ Methyl undecylenate $$ n-Undecanoic acid methyl ester $$ Methyl ester of undecanoi

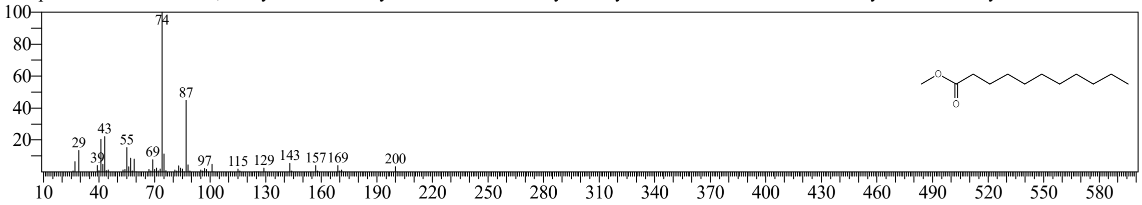

Hit#:2 Entry:18487 Library:NIST14s.lib
SI:96 Formula:C12H24O2 CAS:1731-86-8 MolWeight:200 RetIndex:1381
CompName:Undecanoic acid, methyl ester $$ Methyl undecanoate $$ Methyl undecylenate $$ n-Undecanoic acid methyl ester $$ Methyl ester of undecanoi

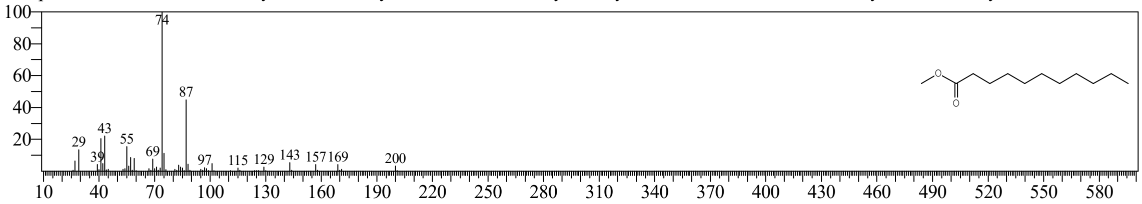

Hit#:3 Entry:21618 Library:NIST17s.lib
SI:95 Formula:C12H24O2 CAS:1731-86-8 MolWeight:200 RetIndex:1381
CompName:Undecanoic acid, methyl ester $$ Methyl undecanoate $$ Methyl undecylenate $$ n-Undecanoic acid methyl ester $$ Methyl ester of undecanoi

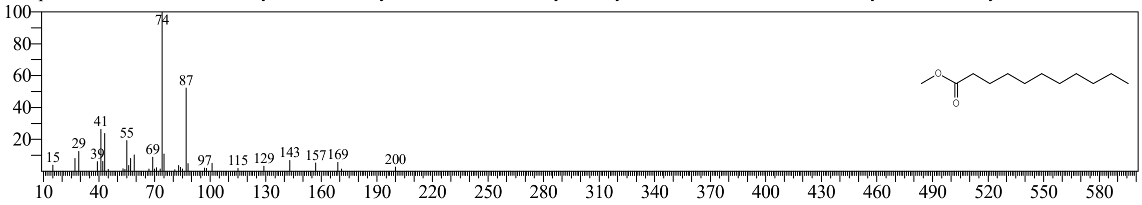

Hit#:4 Entry:18488 Library:NIST14s.lib
SI:94 Formula:C12H24O2 CAS:1731-86-8 MolWeight:200 RetIndex:1381
CompName:Undecanoic acid, methyl ester $$ Methyl undecanoate $$ Methyl undecylenate $$ n-Undecanoic acid methyl ester $$ Methyl ester of undecanoi

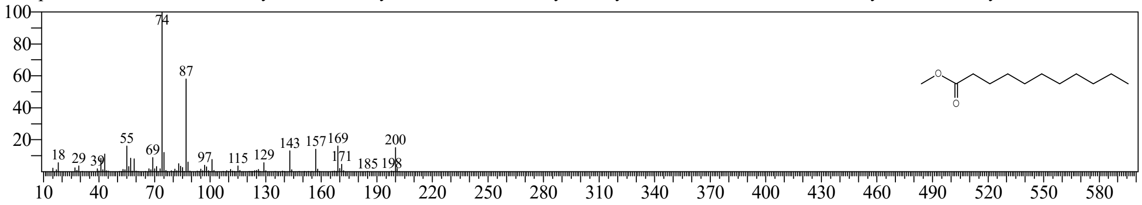

<< Target >>
Line#:1 R.Time:12.925(Scan#:352) MassPeaks:350
RawMode:Averaged 12.917-12.933(351-353) BasePeak:74.00(1087285)
BG Mode:Calc. from Peak Group 1 - Event 1

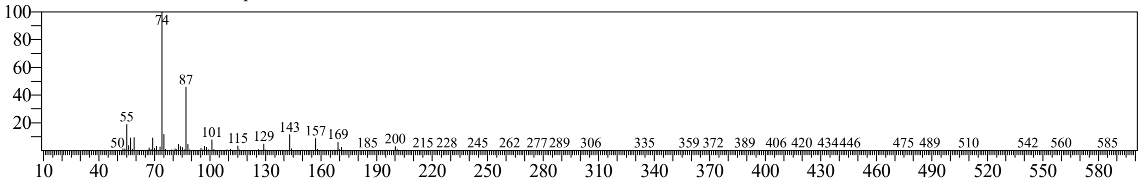

Hit#:5 Entry:21620 Library:NIST17s.lib
SI:94 Formula:C12H24O2 CAS:1731-86-8 MolWeight:200 RetIndex:1381
CompName:Undecanoic acid, methyl ester $$ Methyl undecanoate $$ Methyl undecylenate $$ n-Undecanoic acid methyl ester $$ Methyl ester of undecanoi

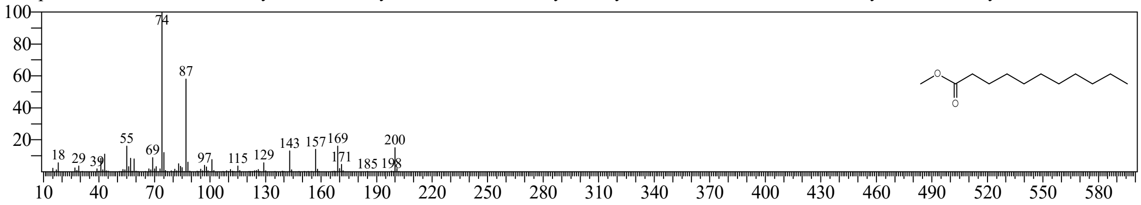

<< Target >>
Line#:2 R.Time:16.992(Scan#:840) MassPeaks:298
RawMode:Averaged 16.983-17.000(839-841) BasePeak:74.00(3292)
BG Mode:Calc. from Peak Group 1 - Event 1

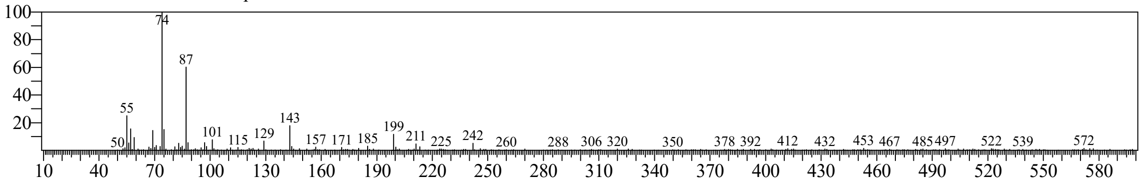

Hit#:1 Entry:86786 Library:NIST17-1.lib
SI:94 Formula:C15H30O2 CAS:124-10-7 MolWeight:242 RetIndex:1680
CompName:Methyl tetradecanoate $$ Tetradecanoic acid, methyl ester $$ Myristic acid, methyl ester $$ Metholeneat 2495 $$ Methyl myristate $$ Methyl r

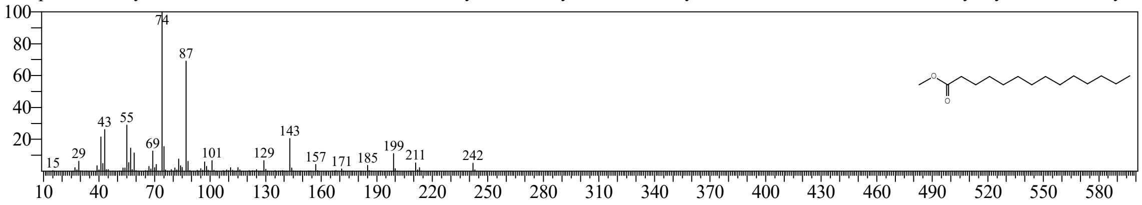

Hit#:2 Entry:80645 Library:NIST14.lib
SI:94 Formula:C15H30O2 CAS:124-10-7 MolWeight:242 RetIndex:1680
CompName:Methyl tetradecanoate $$ Tetradecanoic acid, methyl ester $$ Myristic acid, methyl ester $$ Metholeneat 2495 $$ Methyl myristate $$ Methyl r

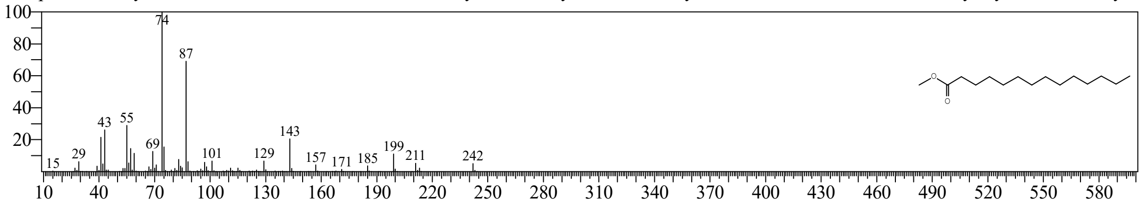

Hit#:3 Entry:27874 Library:NIST17s.lib
SI:94 Formula:C15H30O2 CAS:124-10-7 MolWeight:242 RetIndex:1680
CompName:Methyl tetradecanoate $$ Tetradecanoic acid, methyl ester $$ Myristic acid, methyl ester $$ Metholeneat 2495 $$ Methyl myristate $$ Methyl r

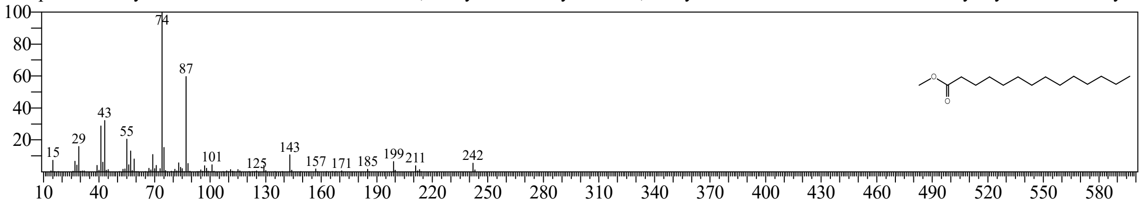

Hit#:4 Entry:23848 Library:NIST14s.lib
SI:94 Formula:C15H30O2 CAS:124-10-7 MolWeight:242 RetIndex:1680
CompName:Methyl tetradecanoate $$ Tetradecanoic acid, methyl ester $$ Myristic acid, methyl ester $$ Metholeneat 2495 $$ Methyl myristate $$ Methyl r

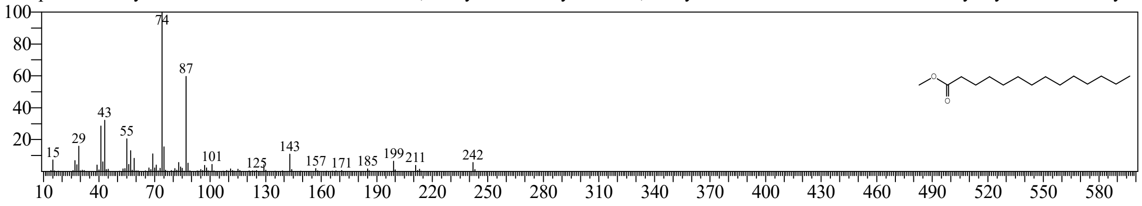

Hit#:5 Entry:27873 Library:NIST17s.lib
SI:93 Formula:C15H30O2 CAS:124-10-7 MolWeight:242 RetIndex:1680
CompName:Methyl tetradecanoate $$ Tetradecanoic acid, methyl ester $$ Myristic acid, methyl ester $$ Metholeneat 2495 $$ Methyl myristate $$ Methyl r

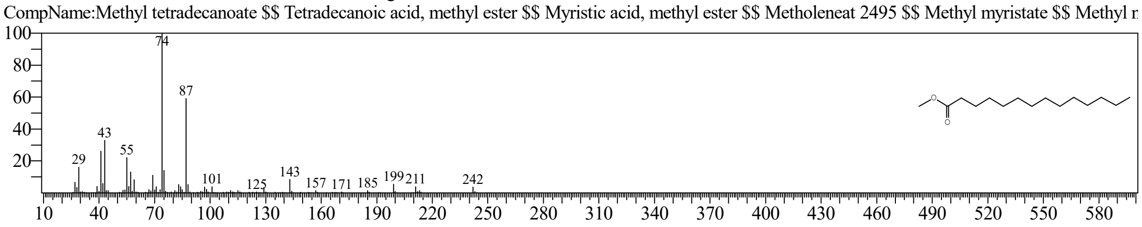

<< Target >>
Line#:3 R.Time:20.800(Scan#:1297) MassPeaks:349
RawMode:Averaged 20.792-20.808(1296-1298) BasePeak:74.00(428928)
BG Mode:Calc. from Peak Group 1 - Event 1

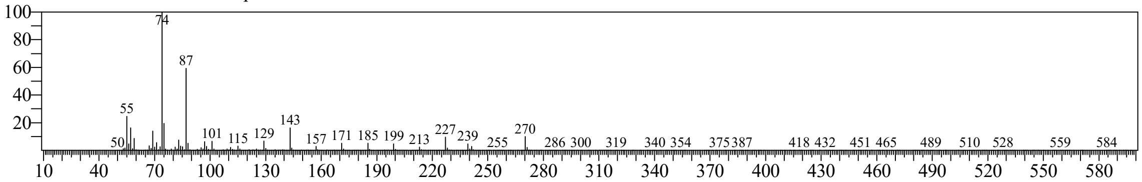

Hit#:1 Entry:104648 Library:NIST14.lib
SI:97 Formula:C17H34O2 CAS:112-39-0 MolWeight:270 RetIndex:1878
CompName:Hexadecanoic acid, methyl ester $$ Palmitic acid, methyl ester $$ n-Hexadecanoic acid methyl ester $$ Metholene 2216 $$ Methyl hexadecano

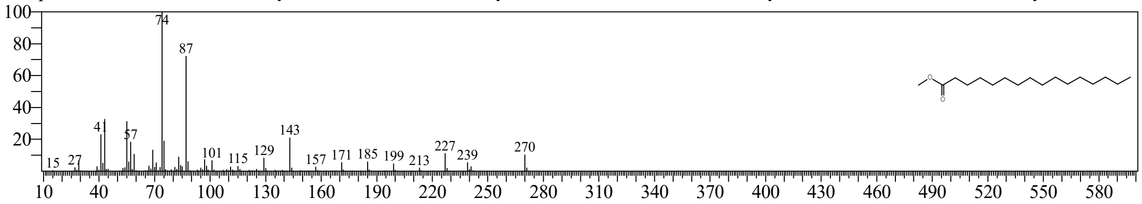

Hit#:2 Entry:113663 Library:NIST17-1.lib
SI:97 Formula:C17H34O2 CAS:112-39-0 MolWeight:270 RetIndex:1878
CompName:Hexadecanoic acid, methyl ester $$ Palmitic acid, methyl ester $$ n-Hexadecanoic acid methyl ester $$ Metholene 2216 $$ Methyl hexadecano

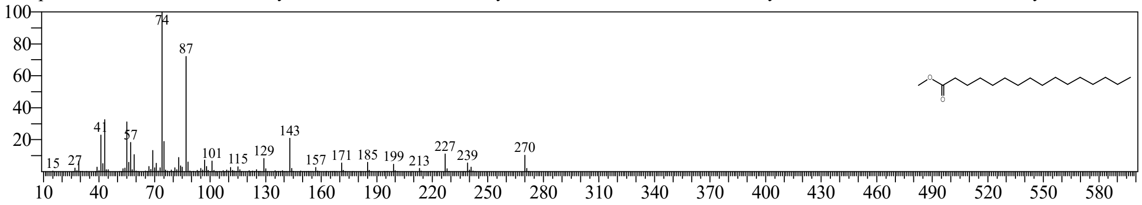

Hit#:3 Entry:26273 Library:NIST14s.lib
SI:96 Formula:C17H34O2 CAS:112-39-0 MolWeight:270 RetIndex:1878
CompName:Hexadecanoic acid, methyl ester $$ Palmitic acid, methyl ester $$ n-Hexadecanoic acid methyl ester $$ Metholene 2216 $$ Methyl hexadecano

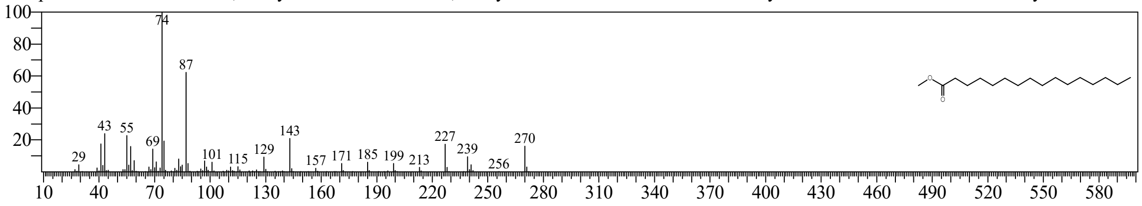

Hit#:4 Entry:30644 Library:NIST17s.lib
SI:96 Formula:C17H34O2 CAS:112-39-0 MolWeight:270 RetIndex:1878
CompName:Hexadecanoic acid, methyl ester $$ Palmitic acid, methyl ester $$ n-Hexadecanoic acid methyl ester $$ Metholene 2216 $$ Methyl hexadecano

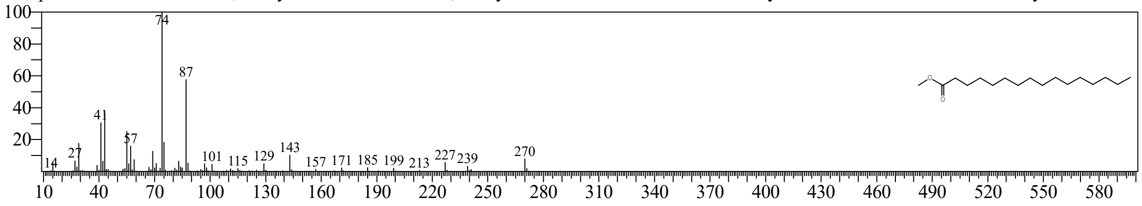

Hit#:5 Entry:26269 Library:NIST14s.lib
SI:96 Formula:C17H34O2 CAS:112-39-0 MolWeight:270 RetIndex:1878
CompName:Hexadecanoic acid, methyl ester $$ Palmitic acid, methyl ester $$ n-Hexadecanoic acid methyl ester $$ Metholene 2216 $$ Methyl hexadecano

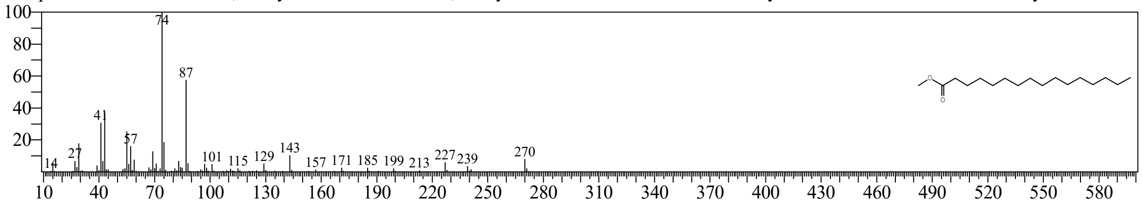

<< Target >>
Line#:4 R.Time:22.150(Scan#:1459) MassPeaks:324
RawMode:Averaged 22.142-22.158(1458-1460) BasePeak:55.05(1183)
BG Mode:Calc. from Peak Group 1 - Event 1

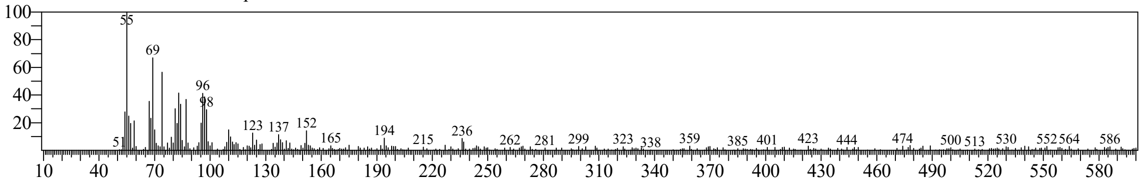

Hit#:1 Entry:30415 Library:NIST17s.lib
SI:93 Formula:C17H32O2 CAS:1120-25-8 MolWeight:268 RetIndex:1886
CompName:9-Hexadecenoic acid, methyl ester, (Z)- $$ Methyl palmitoleate $$ Methyl palmitoleinate $$ Palmitoleic acid, methyl ester $$ Methyl (9Z)-9-he:

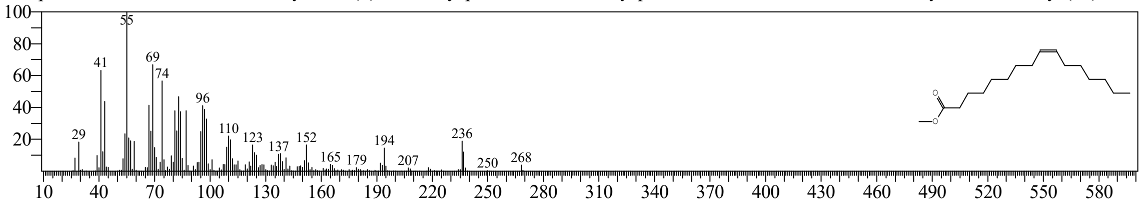

Hit#:2 Entry:26066 Library:NIST14s.lib
SI:93 Formula:C17H32O2 CAS:1120-25-8 MolWeight:268 RetIndex:1886
CompName:9-Hexadecenoic acid, methyl ester, (Z)- $$ Methyl palmitoleate $$ Methyl palmitoleinate $$ Palmitoleic acid, methyl ester $$ Methyl (9Z)-9-he:

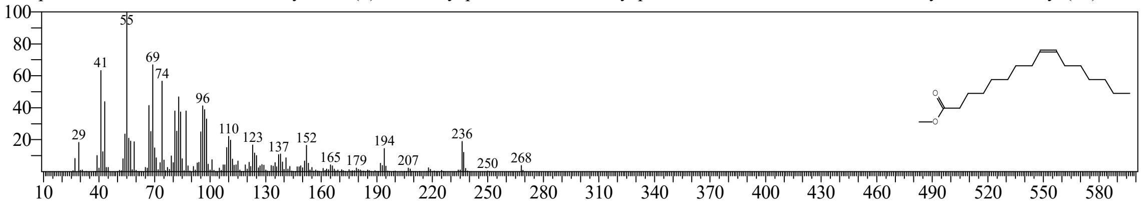

Hit#:3 Entry:26064 Library:NIST14s.lib
SI:92 Formula:C17H32O2 CAS:1120-25-8 MolWeight:268 RetIndex:1886
CompName:9-Hexadecenoic acid, methyl ester, (Z)- $$ Methyl palmitoleate $$ Methyl palmitoleinate $$ Palmitoleic acid, methyl ester $$ Methyl (9Z)-9-he:

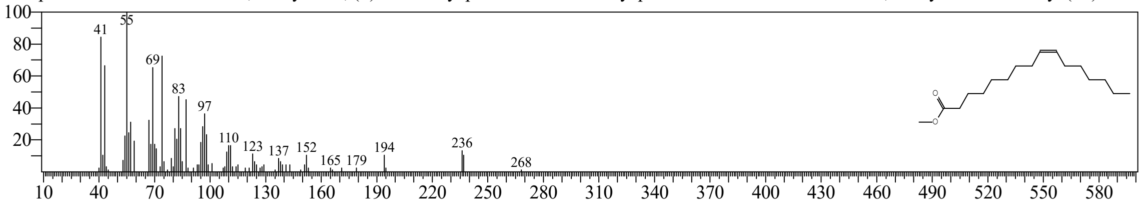

Hit#:4 Entry:30413 Library:NIST17s.lib
SI:92 Formula:C17H32O2 CAS:1120-25-8 MolWeight:268 RetIndex:1886
CompName:9-Hexadecenoic acid, methyl ester, (Z)- $$ Methyl palmitoleate $$ Methyl palmitoleinate $$ Palmitoleic acid, methyl ester $$ Methyl (9Z)-9-he:

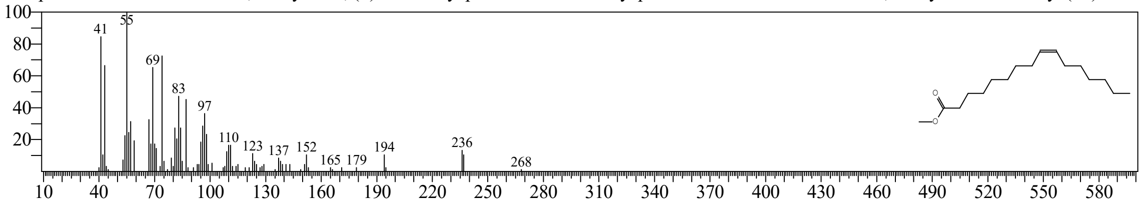

Hit#:5 Entry:111510 Library:NIST17-1.lib
SI:92 Formula:C17H32O2 CAS:56875-67-3 MolWeight:268 RetIndex:1886
CompName:7-Hexadecenoic acid, methyl ester, (Z)- $$ Methyl (7E)-7-hexadecenoate # $$

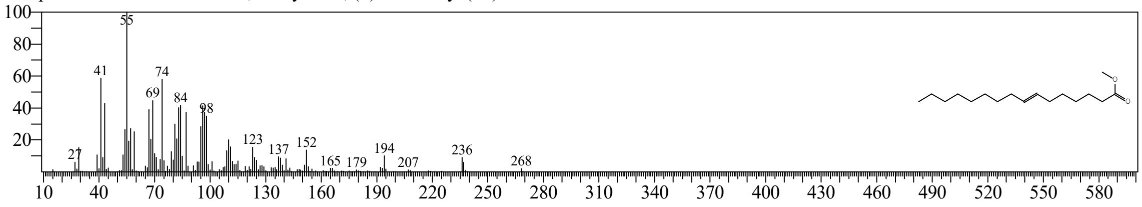

<< Target >>
Line#:5 R.Time:22.767(Scan#:1533) MassPeaks:305
RawMode:Averaged 22.758-22.775(1532-1534) BasePeak:74.00(3057)
BG Mode:Calc. from Peak Group 1 - Event 1

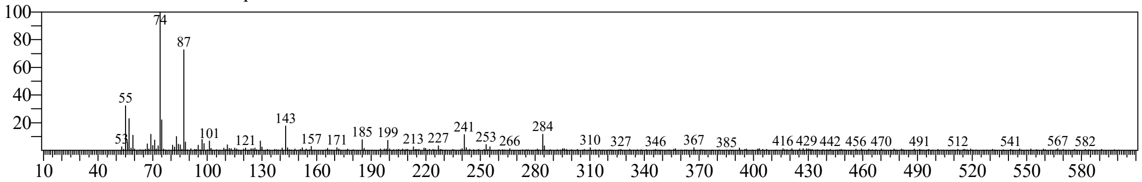

Hit#:1 Entry:27279 Library:NIST14s.lib
SI:94 Formula:C18H36O2 CAS:1731-92-6 MolWeight:284 RetIndex:1978
CompName:Heptadecanoic acid, methyl ester $$ Margaric acid methyl ester $$ Methyl heptadecanoate $$ Methyl margarate $$ n-Heptadecanoic acid methy

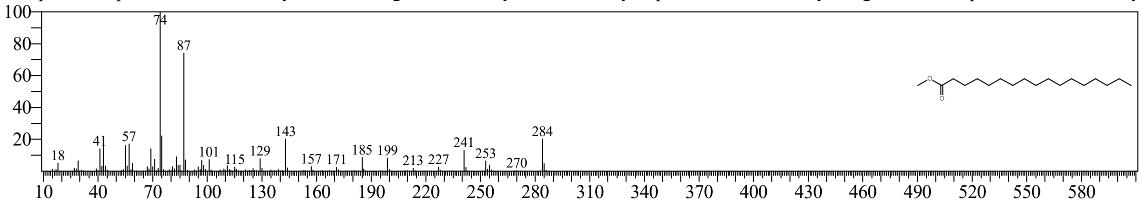

Hit#:2 Entry:31787 Library:NIST17s.lib
SI:94 Formula:C18H36O2 CAS:1731-92-6 MolWeight:284 RetIndex:1978
CompName:Heptadecanoic acid, methyl ester $$ Margaric acid methyl ester $$ Methyl heptadecanoate $$ Methyl margarate $$ n-Heptadecanoic acid methy

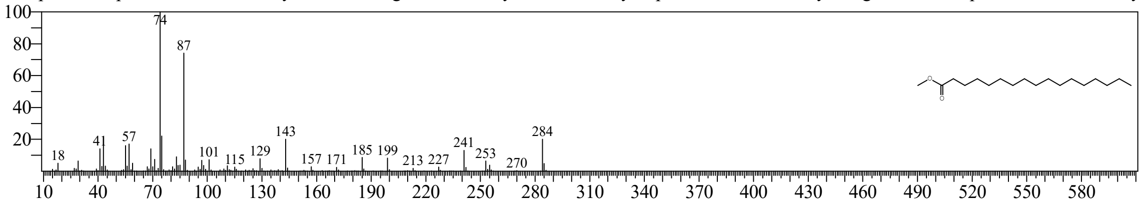

Hit#:3 Entry:127714 Library:NIST17-1.lib
SI:93 Formula:C18H36O2 CAS:6929-04-0 MolWeight:284 RetIndex:1914
CompName:Hexadecanoic acid, 15-methyl-, methyl ester $$ Methyl isoheptadecanoate $$ Methyl 15-methylhexadecanoate $$

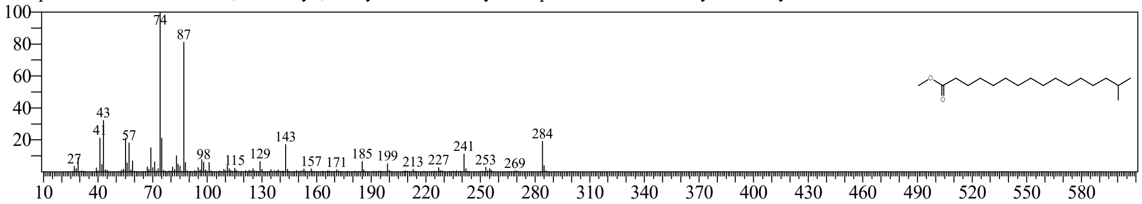

Hit#:4 Entry:27277 Library:NIST14s.lib
SI:93 Formula:C18H36O2 CAS:6929-04-0 MolWeight:284 RetIndex:1914
CompName:Hexadecanoic acid, 15-methyl-, methyl ester $$ Methyl isoheptadecanoate $$ Methyl 15-methylhexadecanoate $$

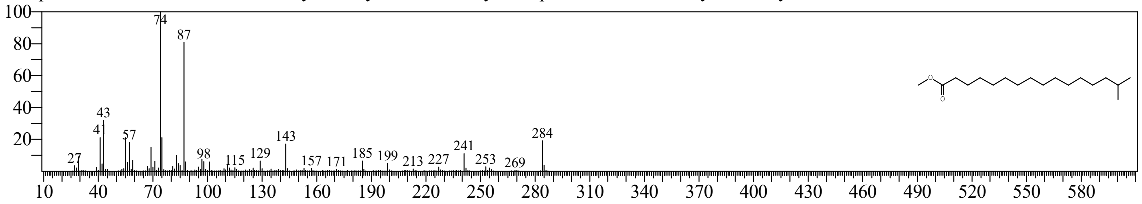

Hit#:5 Entry:27274 Library:NIST14s.lib
SI:92 Formula:C18H36O2 CAS:1731-92-6 MolWeight:284 RetIndex:1978
CompName:Heptadecanoic acid, methyl ester $$ Margaric acid methyl ester $$ Methyl heptadecanoate $$ Methyl margarate $$ n-Heptadecanoic acid methy

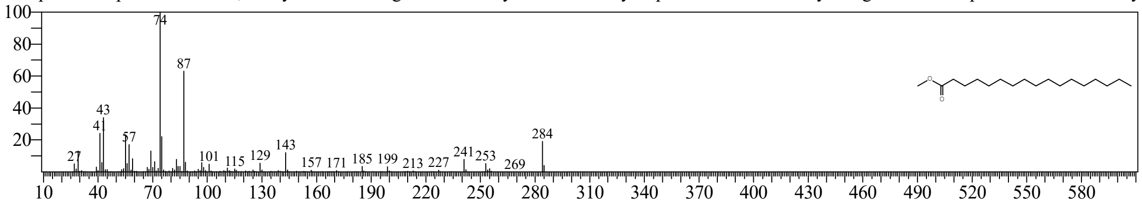

<< Target >>
Line#:6 R.Time:24.092(Scan#:1692) MassPeaks:365
RawMode:Averaged 24.083-24.100(1691-1693) BasePeak:55.00(601)
BG Mode:Calc. from Peak Group 1 - Event 1

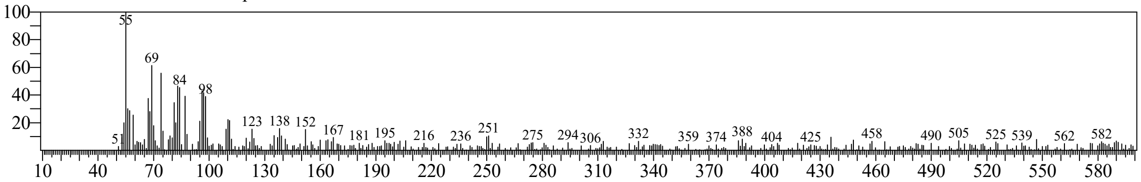

Hit#:1 Entry:125438 Library:NIST17-1.lib
SI:87 Formula:C18H34O2 CAS:0-00-0 MolWeight:282 RetIndex:1986
CompName:cis-10-Heptadecenoic acid, methyl ester

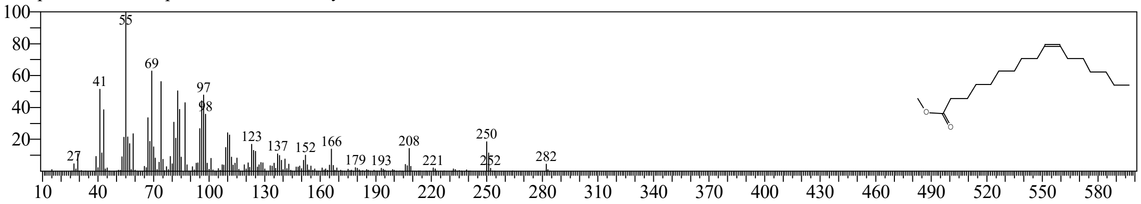

Hit#:2 Entry:115094 Library:NIST14.lib
SI:87 Formula:C18H34O2 CAS:0-00-0 MolWeight:282 RetIndex:1986
CompName:cis-10-Heptadecenoic acid, methyl ester

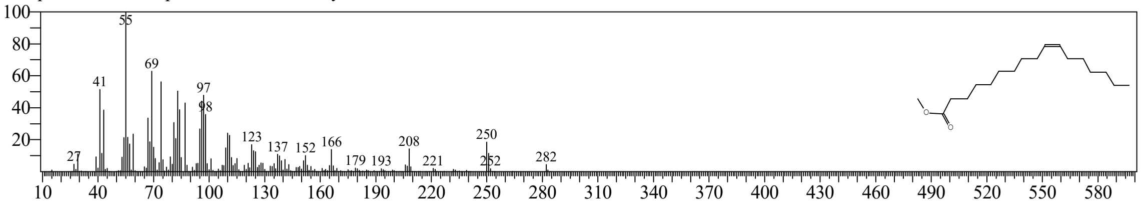

Hit#:3 Entry:28135 Library:NIST14s.lib
SI:86 Formula:C19H36O2 CAS:112-62-9 MolWeight:296 RetIndex:2085
CompName:9-Octadecenoic acid (Z)-, methyl ester $$ Oleic acid, methyl ester $$ Emery oleic acid ester 2301 $$ Methyl cis-9-octadecenoate $$ Methyl ole

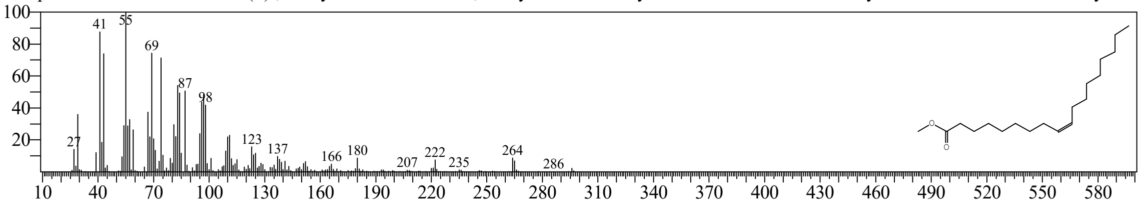

Hit#:4 Entry:32744 Library:NIST17s.lib
SI:86 Formula:C19H36O2 CAS:112-62-9 MolWeight:296 RetIndex:2085
CompName:9-Octadecenoic acid (Z)-, methyl ester $$ Oleic acid, methyl ester $$ Emery oleic acid ester 2301 $$ Methyl cis-9-octadecenoate $$ Methyl ole

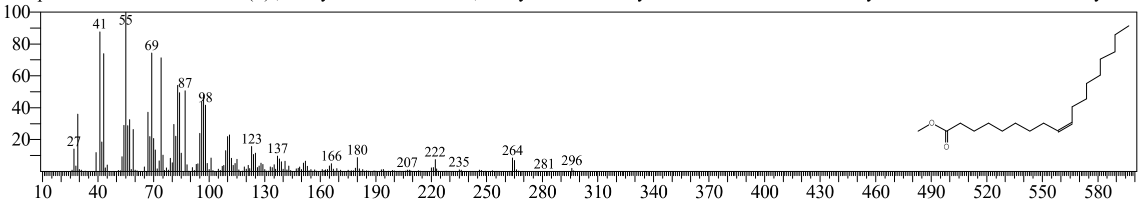

Hit#:5 Entry:27110 Library:NIST14s.lib
SI:86 Formula:C18H34O2 CAS:10152-61-1 MolWeight:282 RetIndex:1941
CompName:Cyclopropaneoctanoic acid, 2-hexyl-, methyl ester $$ Methyl 8-(2-hexylcyclopropyl)octanoate # $$

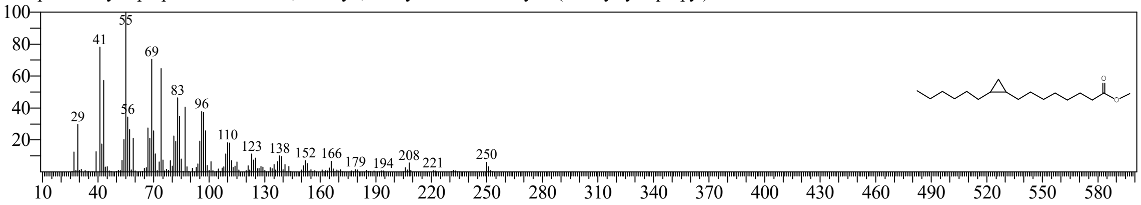

<< Target >>
Line#:7 R.Time:24.858(Scan#:1784) MassPeaks:385
RawMode:Averaged 24.850-24.867(1783-1785) BasePeak:74.00(123900)
BG Mode:Calc. from Peak Group 1 - Event 1

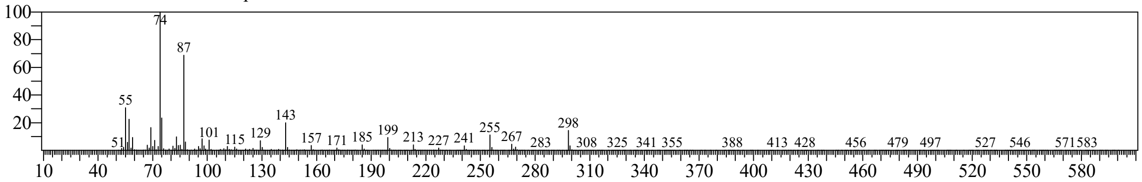

Hit#:1 Entry:129694 Library:NIST14.lib
SI:98 Formula:C19H38O2 CAS:112-61-8 MolWeight:298 RetIndex:2077
CompName:Methyl stearate $$ Octadecanoic acid, methyl ester $$ Stearic acid, methyl ester $$ n-Octadecanoic acid, methyl ester $$ Kemester 9718 $$ Me

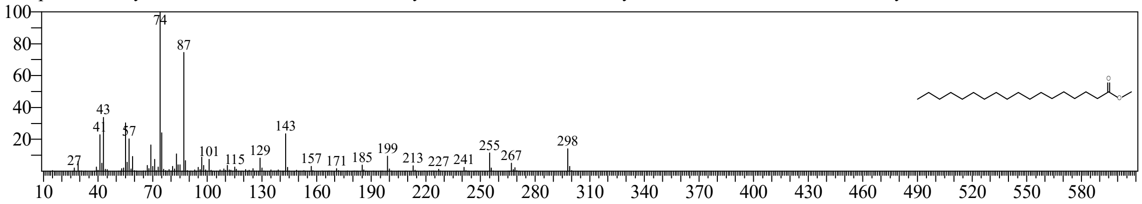

Hit#:2 Entry:141978 Library:NIST17-1.lib
SI:98 Formula:C19H38O2 CAS:112-61-8 MolWeight:298 RetIndex:2077
CompName:Methyl stearate $$ Octadecanoic acid, methyl ester $$ Stearic acid, methyl ester $$ n-Octadecanoic acid, methyl ester $$ Kemester 9718 $$ Me

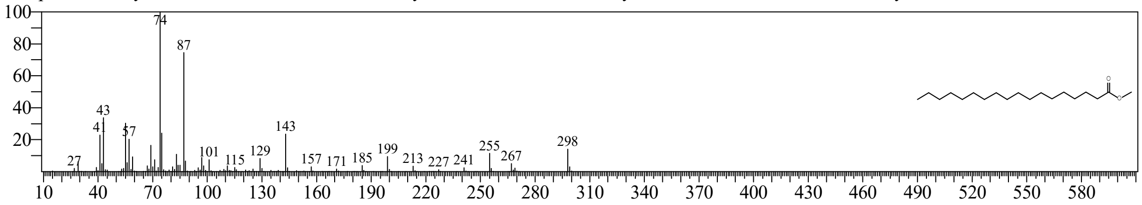

Hit#:3 Entry:28255 Library:NIST14s.lib
SI:97 Formula:C19H38O2 CAS:112-61-8 MolWeight:298 RetIndex:2077
CompName:Methyl stearate $$ Octadecanoic acid, methyl ester $$ Stearic acid, methyl ester $$ n-Octadecanoic acid, methyl ester $$ Kemester 9718 $$ Me

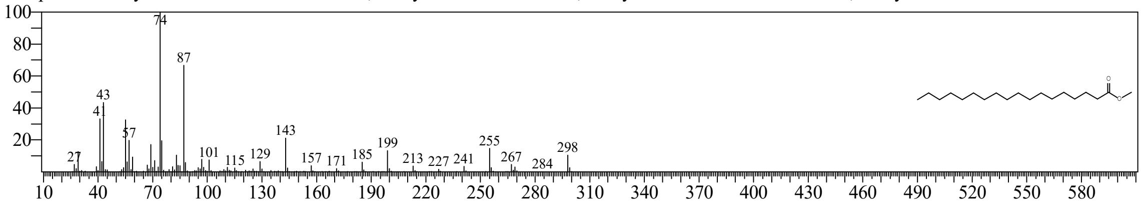

Hit#:4 Entry:32873 Library:NIST17s.lib
SI:97 Formula:C19H38O2 CAS:112-61-8 MolWeight:298 RetIndex:2077
CompName:Methyl stearate $$ Octadecanoic acid, methyl ester $$ Stearic acid, methyl ester $$ n-Octadecanoic acid, methyl ester $$ Kemester 9718 $$ Me

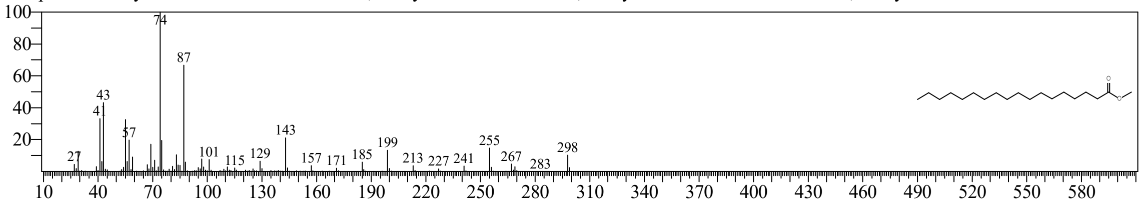

Hit#:5 Entry:28257 Library:NIST14s.lib
SI:96 Formula:C19H38O2 CAS:112-61-8 MolWeight:298 RetIndex:2077
CompName:Methyl stearate $$ Octadecanoic acid, methyl ester $$ Stearic acid, methyl ester $$ n-Octadecanoic acid, methyl ester $$ Kemester 9718 $$ Me

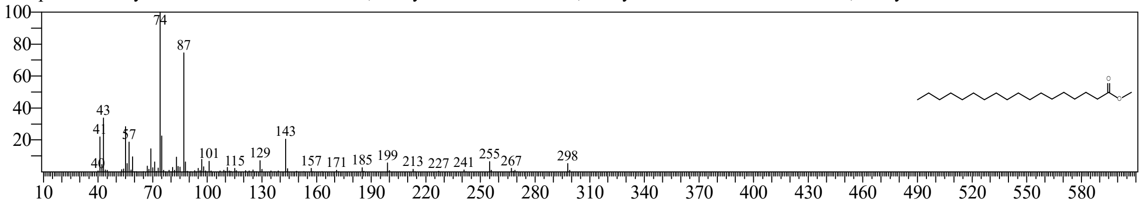

<< Target >>
Line#:8 R.Time:26.242(Scan#:1950) MassPeaks:381
RawMode:Averaged 26.233-26.250(1949-1951) BasePeak:55.05(226720)
BG Mode:Calc. from Peak Group 1 - Event 1

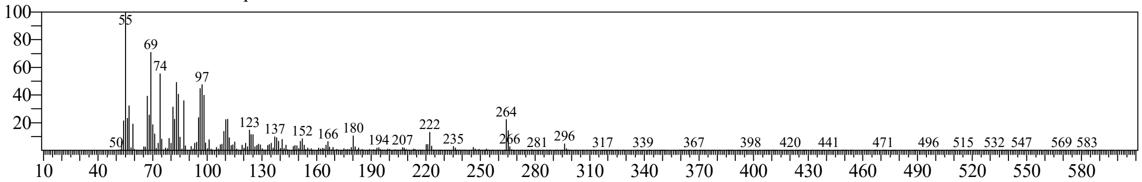

Hit#:1 Entry:32747 Library:NIST17s.lib
SI:97 Formula:C19H36O2 CAS:1937-62-8 MolWeight:296 RetIndex:2085
CompName:9-Octadecenoic acid, methyl ester, (E)- $$ Elaidic acid, methyl ester $$ Methyl elaidate $$ Methyl trans-9-octadecenoate $$ (E)-9-Octadecenoic

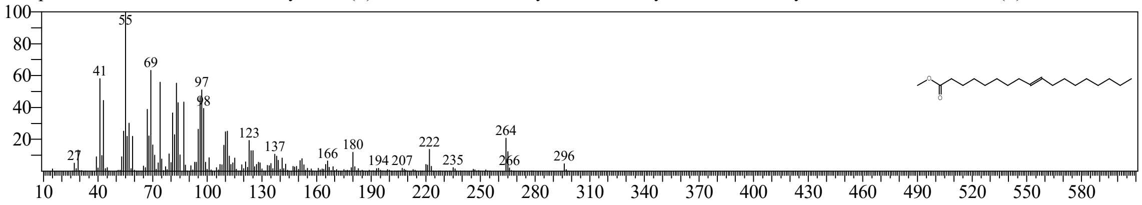

Hit#:2 Entry:28138 Library:NIST14s.lib
SI:97 Formula:C19H36O2 CAS:1937-62-8 MolWeight:296 RetIndex:2085
CompName:9-Octadecenoic acid, methyl ester, (E)- $$ Elaidic acid, methyl ester $$ Methyl elaidate $$ Methyl trans-9-octadecenoate $$ (E)-9-Octadecenoic

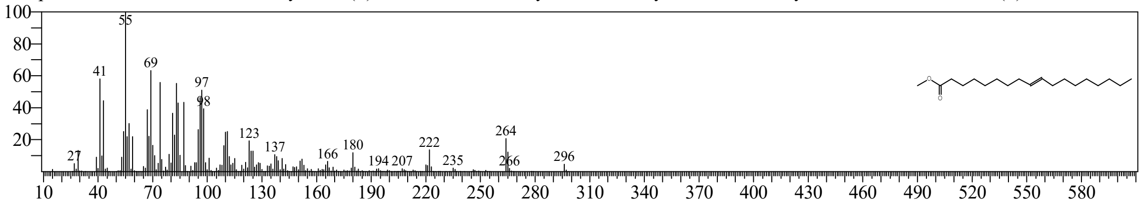

Hit#:3 Entry:127664 Library:NIST14.lib
SI:96 Formula:C19H36O2 CAS:112-62-9 MolWeight:296 RetIndex:2085
CompName:9-Octadecenoic acid (Z)-, methyl ester $$ Oleic acid, methyl ester $$ Emery oleic acid ester 2301 $$ Methyl cis-9-octadecenoate $$ Methyl ole

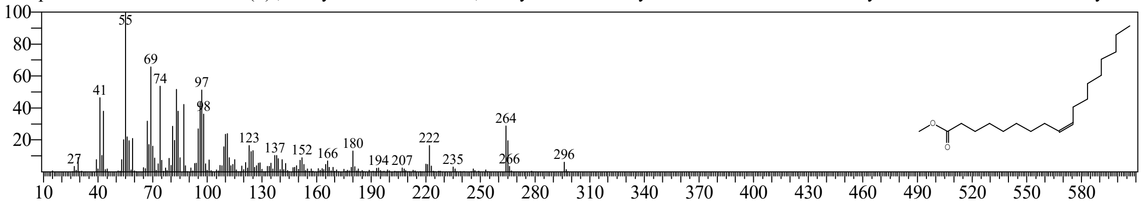

Hit#:4 Entry:139650 Library:NIST17-1.lib
SI:96 Formula:C19H36O2 CAS:112-62-9 MolWeight:296 RetIndex:2085
CompName:9-Octadecenoic acid (Z)-, methyl ester $$ Oleic acid, methyl ester $$ Emery oleic acid ester 2301 $$ Methyl cis-9-octadecenoate $$ Methyl ole

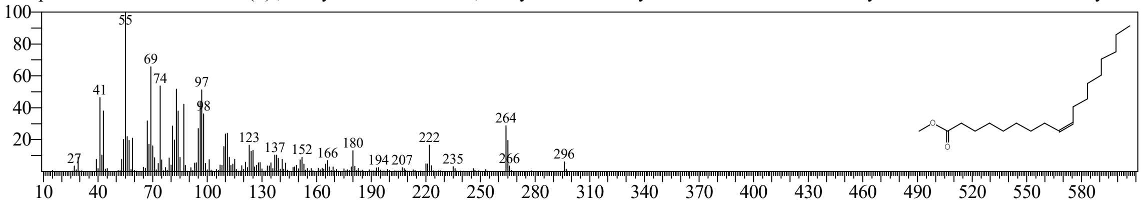

Hit#:5 Entry:28139 Library:NIST14s.lib
SI:95 Formula:C19H36O2 CAS:52380-33-3 MolWeight:296 RetIndex:2085
CompName:11-Octadecenoic acid, methyl ester $$ Methyl 11-octadecenoate $$ Octadec-11-enoic acid, methyl ester $$ Methyl (11E)-11-octadecenoate # $$

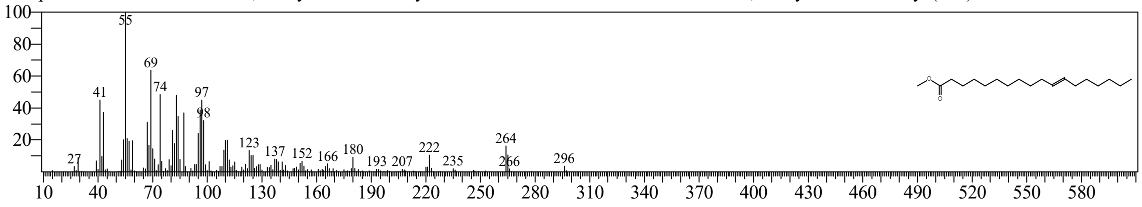

<< Target >>
Line#:9 R.Time:26.367(Scan#:1965) MassPeaks:327
RawMode:Averaged 26.358-26.375(1964-1966) BasePeak:55.05(15319)
BG Mode:Calc. from Peak Group 1 - Event 1

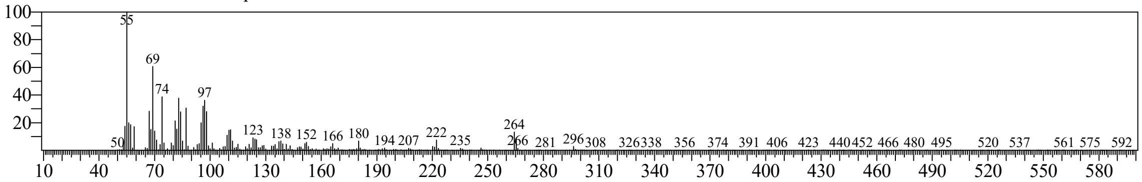

Hit#:1 Entry:32748 Library:NIST17s.lib
SI:95 Formula:C19H36O2 CAS:52380-33-3 MolWeight:296 RetIndex:2085
CompName:11-Octadecenoic acid, methyl ester $$ Methyl 11-octadecenoate $$ Octadec-11-enoic acid, methyl ester $$ Methyl (11E)-11-octadecenoate # $$

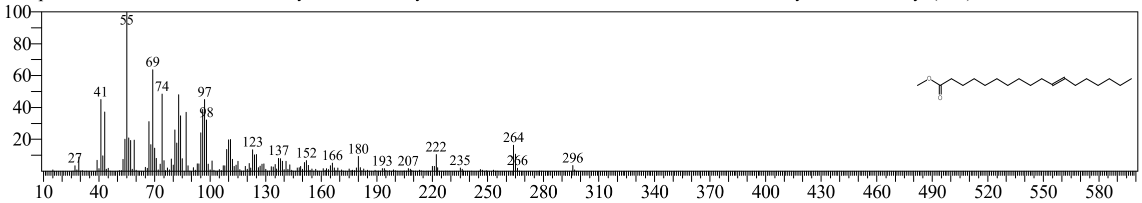

Hit#:2 Entry:28139 Library:NIST14s.lib
SI:95 Formula:C19H36O2 CAS:52380-33-3 MolWeight:296 RetIndex:2085
CompName:11-Octadecenoic acid, methyl ester $$ Methyl 11-octadecenoate $$ Octadec-11-enoic acid, methyl ester $$ Methyl (11E)-11-octadecenoate # $$

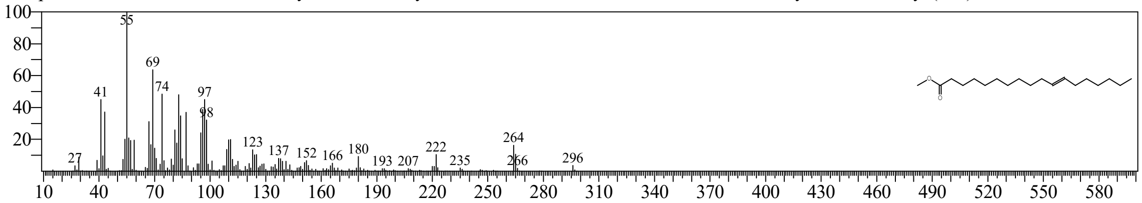

Hit#:3 Entry:127661 Library:NIST14.lib
SI:95 Formula:C19H36O2 CAS:0-00-0 MolWeight:296 RetIndex:2085
CompName:cis-13-Octadecenoic acid, methyl ester

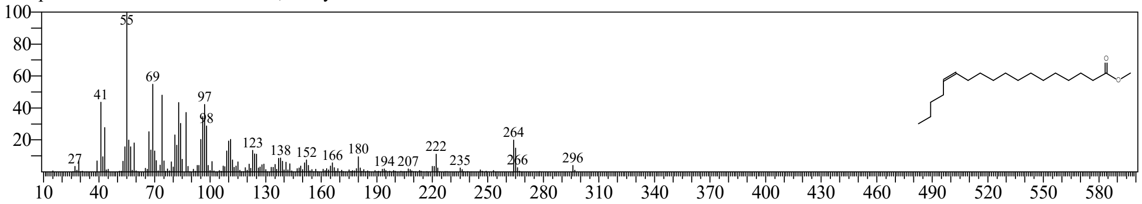

Hit#:4 Entry:139647 Library:NIST17-1.lib
SI:95 Formula:C19H36O2 CAS:0-00-0 MolWeight:296 RetIndex:2085
CompName:cis-13-Octadecenoic acid, methyl ester

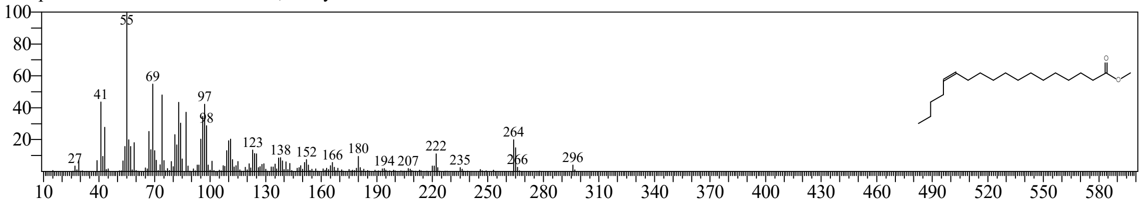

Hit#:5 Entry:127658 Library:NIST14.lib
SI:94 Formula:C19H36O2 CAS:0-00-0 MolWeight:296 RetIndex:2085
CompName:trans-13-Octadecenoic acid, methyl ester

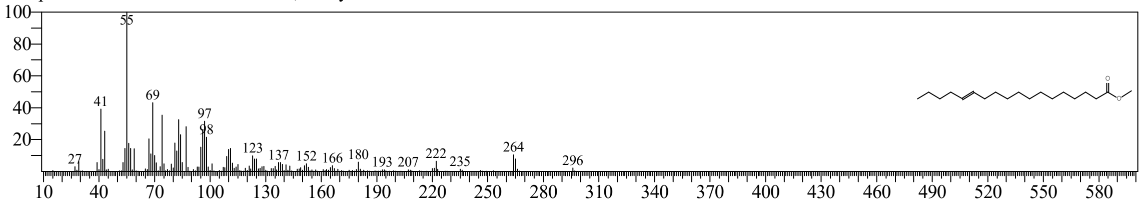

<< Target >>
Line#:10 R.Time:27.717(Scan#:2127) MassPeaks:367
RawMode:Averaged 27.708-27.725(2126-2128) BasePeak:67.00(3584)
BG Mode:Calc. from Peak Group 1 - Event 1

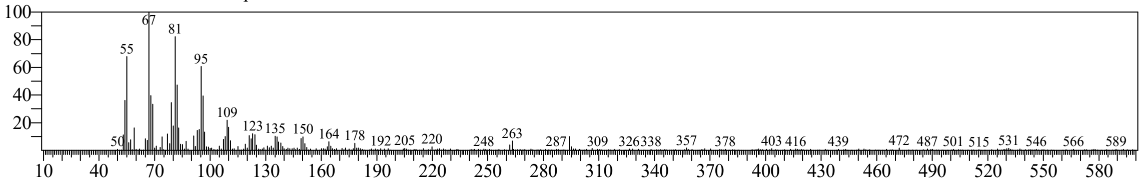

Hit#:1 Entry:125931 Library:NIST14.lib
SI:96 Formula:C19H34O2 CAS:2462-85-3 MolWeight:294 RetIndex:2093
CompName:9,12-Octadecadienoic acid, methyl ester $$ 9,12-Octadecenoic acid, methyl ester $$ Methyl octadeca-9,12-dienoate $$ Methyl 9,12-octadecadi

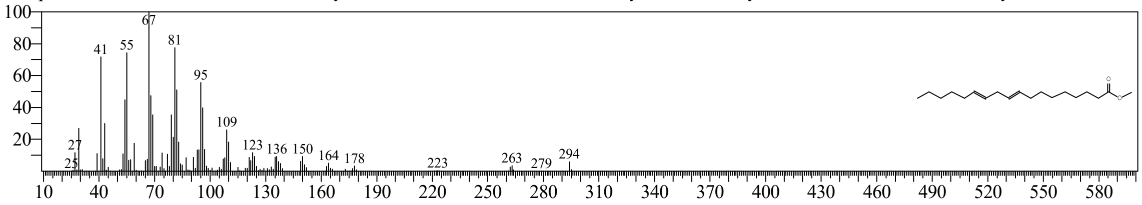

Hit#:2 Entry:137663 Library:NIST17-1.lib
SI:96 Formula:C19H34O2 CAS:2462-85-3 MolWeight:294 RetIndex:2093
CompName:9,12-Octadecadienoic acid, methyl ester $$ 9,12-Octadecenoic acid, methyl ester $$ Methyl octadeca-9,12-dienoate $$ Methyl 9,12-octadecadi

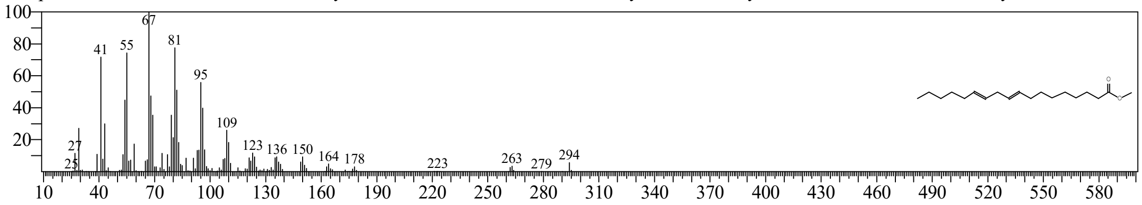

Hit#:3 Entry:32588 Library:NIST17s.lib
SI:96 Formula:C19H34O2 CAS:112-63-0 MolWeight:294 RetIndex:2093
CompName:9,12-Octadecadienoic acid (Z,Z)-, methyl ester $$ Linoleic acid, methyl ester $$ Methyl cis,cis-9,12-octadecadienoate $$ Methyl linoleate $$ M

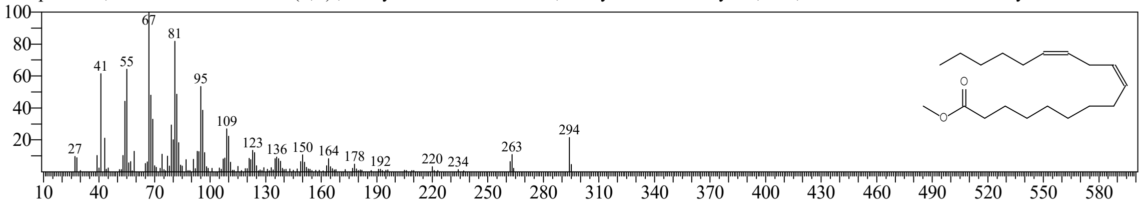

Hit#:4 Entry:27999 Library:NIST14s.lib
SI:96 Formula:C19H34O2 CAS:112-63-0 MolWeight:294 RetIndex:2093
CompName:9,12-Octadecadienoic acid (Z,Z)-, methyl ester $$ Linoleic acid, methyl ester $$ Methyl cis,cis-9,12-octadecadienoate $$ Methyl linoleate $$ M

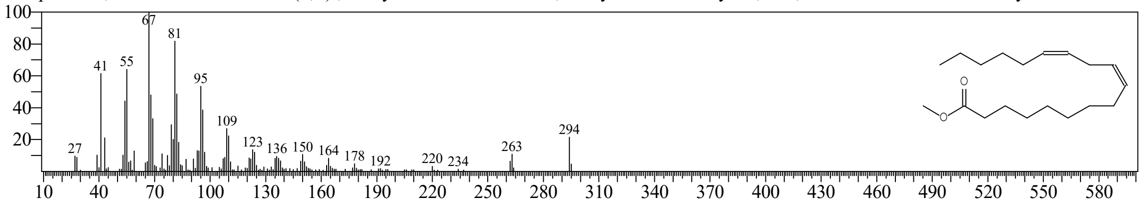

Hit#:5 Entry:32589 Library:NIST17s.lib
SI:95 Formula:C19H34O2 CAS:112-63-0 MolWeight:294 RetIndex:2093
CompName:9,12-Octadecadienoic acid (Z,Z)-, methyl ester $$ Linoleic acid, methyl ester $$ Methyl cis,cis-9,12-octadecadienoate $$ Methyl linoleate $$ M

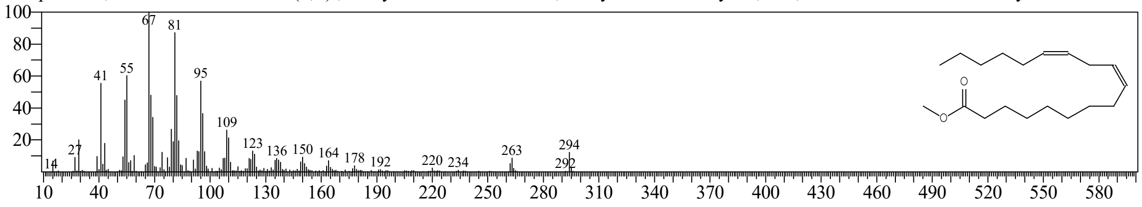

<< Target >>
Line#:11   R.Time:27.933(Scan#:2153) MassPeaks:329
RawMode:Averaged 27.925-27.942(2152-2154) BasePeak:67.00(2519)
BG Mode:Calc. from Peak Group 1 - Event 1

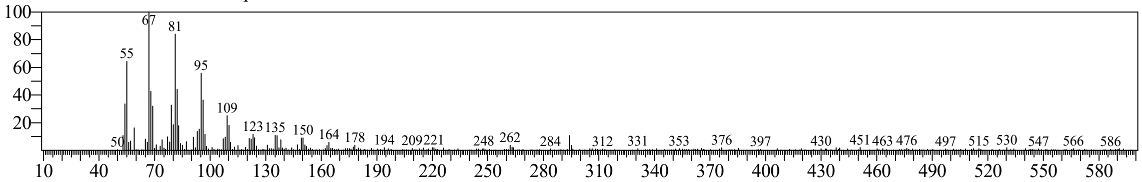

Hit#:1 Entry:125931 Library:NIST14.lib
SI:96 Formula:C19H34O2 CAS:2462-85-3 MolWeight:294 RetIndex:2093
CompName:9,12-Octadecadienoic acid, methyl ester $$ 9,12-Octadecenoic acid, methyl ester $$ Methyl octadeca-9,12-dienoate $$ Methyl 9,12-octadecadi

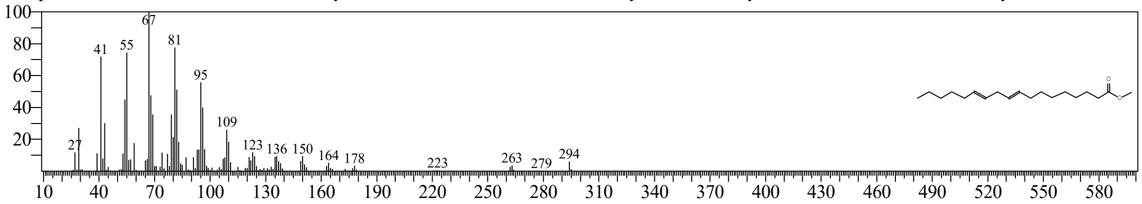

Hit#:2 Entry:137663 Library:NIST17-1.lib
SI:96 Formula:C19H34O2 CAS:2462-85-3 MolWeight:294 RetIndex:2093
CompName:9,12-Octadecadienoic acid, methyl ester $$ 9,12-Octadecenoic acid, methyl ester $$ Methyl octadeca-9,12-dienoate $$ Methyl 9,12-octadecadi

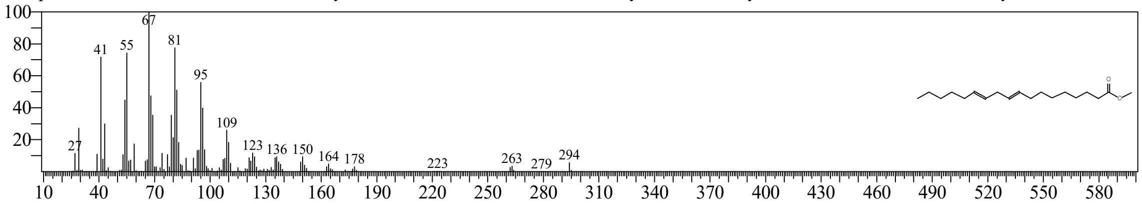

Hit#:3 Entry:32588 Library:NIST17s.lib
SI:95 Formula:C19H34O2 CAS:112-63-0 MolWeight:294 RetIndex:2093
CompName:9,12-Octadecadienoic acid (Z,Z)-, methyl ester $$ Linoleic acid, methyl ester $$ Methyl cis,cis-9,12-octadecadienoate $$ Methyl linoleate $$ M

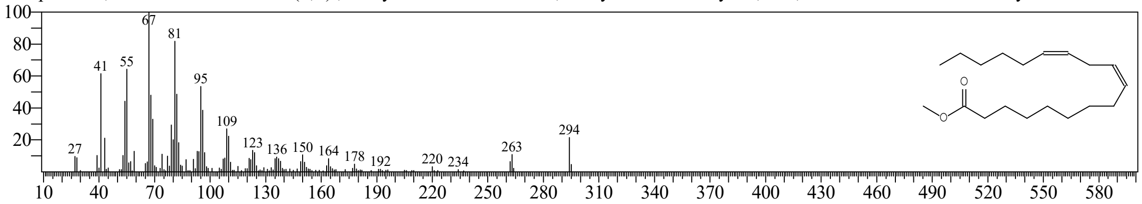

Hit#:4 Entry:27999 Library:NIST14s.lib
SI:95 Formula:C19H34O2 CAS:112-63-0 MolWeight:294 RetIndex:2093
CompName:9,12-Octadecadienoic acid (Z,Z)-, methyl ester $$ Linoleic acid, methyl ester $$ Methyl cis,cis-9,12-octadecadienoate $$ Methyl linoleate $$ M

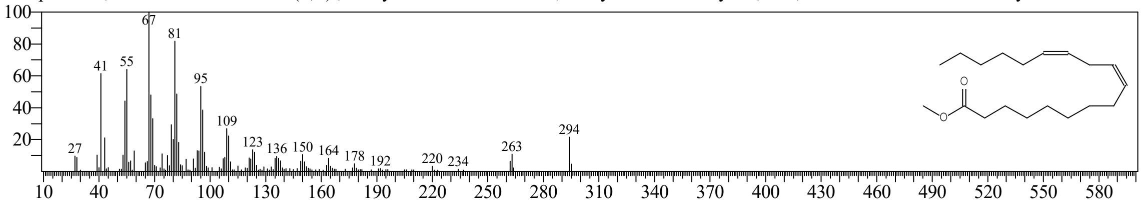

Hit#:5 Entry:32587 Library:NIST17s.lib
SI:95 Formula:C19H34O2 CAS:13038-47-6 MolWeight:294 RetIndex:2093
CompName:9,11-Octadecadienoic acid, methyl ester, (E,E)- $$ Methyl trans-9,trans-11-octadecadienoate $$ Methyl trans,trans-9,11-octadecadienoate $$ M

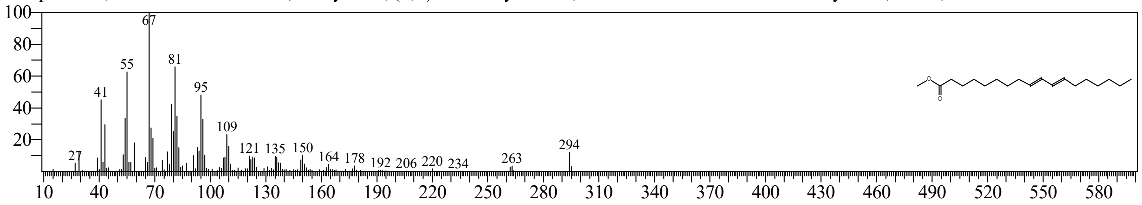

<< Target >>
Line#:12 R.Time:28.350(Scan#:2203) MassPeaks:375
RawMode:Averaged 28.342-28.358(2202-2204) BasePeak:67.05(437809)
BG Mode:Calc. from Peak Group 1 - Event 1

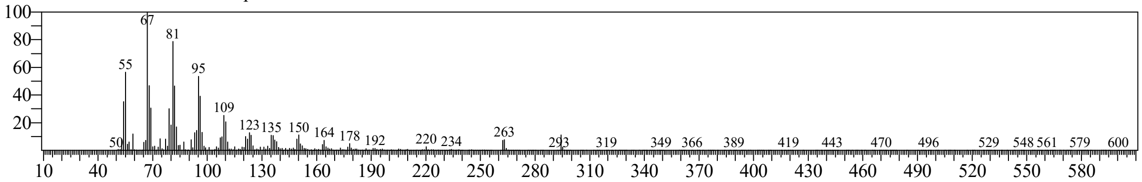

Hit#:1 Entry:32588 Library:NIST17s.lib
SI:97 Formula:C19H34O2 CAS:112-63-0 MolWeight:294 RetIndex:2093
CompName:9,12-Octadecadienoic acid (Z,Z)-, methyl ester $$ Linoleic acid, methyl ester $$ Methyl cis,cis-9,12-octadecadienoate $$ Methyl linoleate $$ M

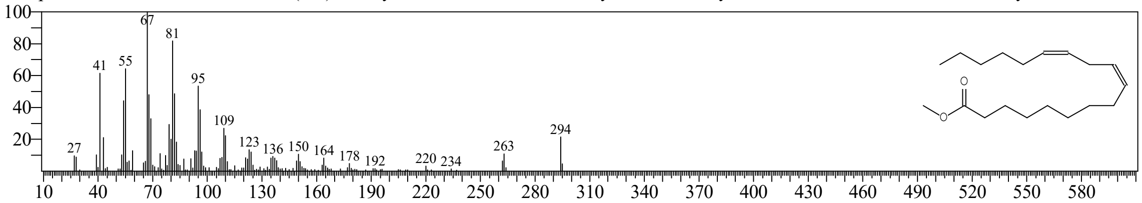

Hit#:2 Entry:27999 Library:NIST14s.lib
SI:97 Formula:C19H34O2 CAS:112-63-0 MolWeight:294 RetIndex:2093
CompName:9,12-Octadecadienoic acid (Z,Z)-, methyl ester $$ Linoleic acid, methyl ester $$ Methyl cis,cis-9,12-octadecadienoate $$ Methyl linoleate $$ M

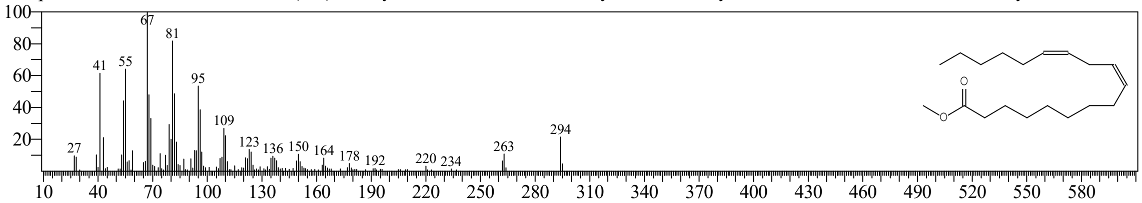

Hit#:3 Entry:32589 Library:NIST17s.lib
SI:96 Formula:C19H34O2 CAS:112-63-0 MolWeight:294 RetIndex:2093
CompName:9,12-Octadecadienoic acid (Z,Z)-, methyl ester $$ Linoleic acid, methyl ester $$ Methyl cis,cis-9,12-octadecadienoate $$ Methyl linoleate $$ M

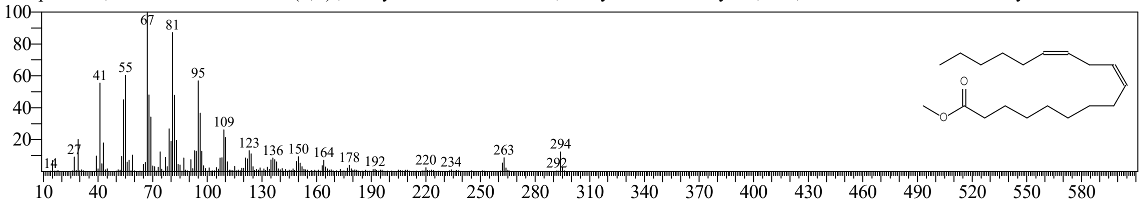

Hit#:4 Entry:28000 Library:NIST14s.lib
SI:96 Formula:C19H34O2 CAS:112-63-0 MolWeight:294 RetIndex:2093
CompName:9,12-Octadecadienoic acid (Z,Z)-, methyl ester $$ Linoleic acid, methyl ester $$ Methyl cis,cis-9,12-octadecadienoate $$ Methyl linoleate $$ M

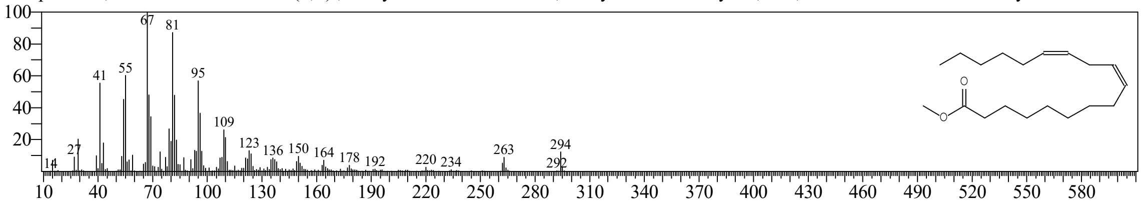

Hit#:5 Entry:125933 Library:NIST14.lib
SI:95 Formula:C19H34O2 CAS:0-00-0 MolWeight:294 RetIndex:2093
CompName:Methyl 10-trans,12-cis-octadecadienoate

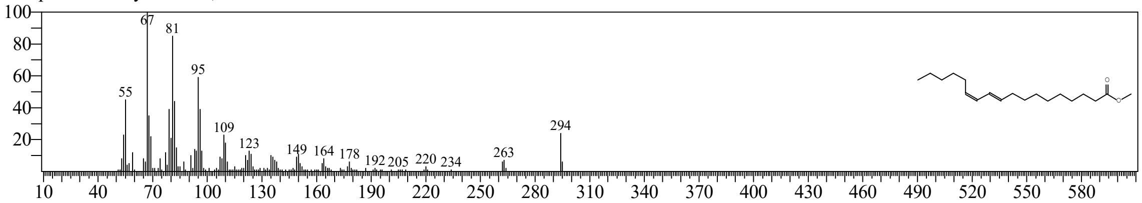

<< Target >>
Line#:13   R.Time:29.258(Scan#:2312) MassPeaks:353
RawMode:Averaged 29.250-29.267(2311-2313) BasePeak:74.00(11751)
BG Mode:Calc. from Peak Group 1 - Event 1

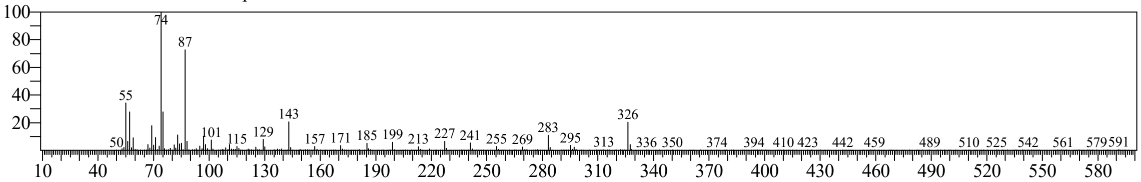

Hit#:1 Entry:154704 Library:NIST14.lib
SI:97 Formula:C21H42O2 CAS:0-00-0 MolWeight:326 RetIndex:2212
CompName:Methyl 18-methylnonadecanoate

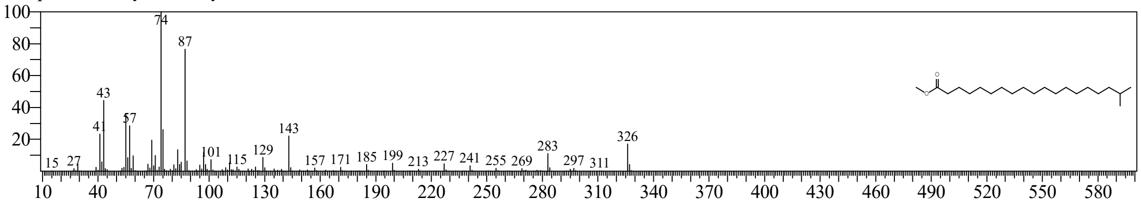

Hit#:2 Entry:170238 Library:NIST17-1.lib
SI:97 Formula:C21H42O2 CAS:0-00-0 MolWeight:326 RetIndex:2212
CompName:Methyl 18-methylnonadecanoate

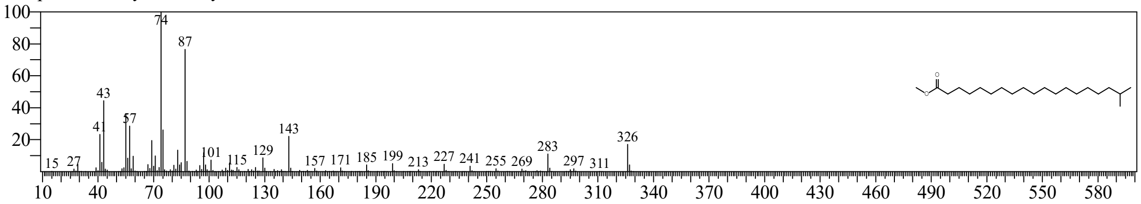

Hit#:3 Entry:34726 Library:NIST17s.lib
SI:96 Formula:C21H42O2 CAS:1120-28-1 MolWeight:326 RetIndex:2276
CompName:Eicosanoic acid, methyl ester $$ Methyl arachisate $$ Methyl eicosanoate $$ Arachidic acid methyl ester $$ Kemester 2050 $$ Methyl aracidat

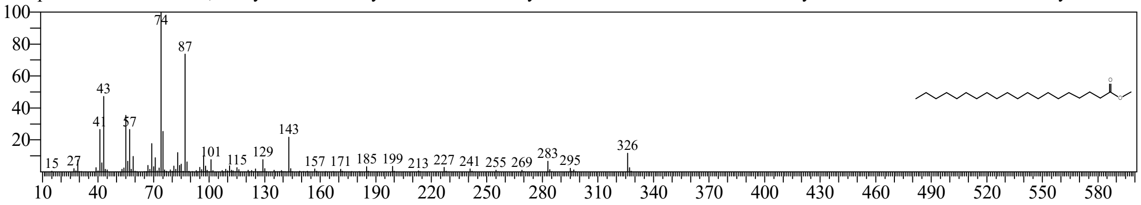

Hit#:4 Entry:34724 Library:NIST17s.lib
SI:93 Formula:C21H42O2 CAS:1120-28-1 MolWeight:326 RetIndex:2276
CompName:Eicosanoic acid, methyl ester $$ Methyl arachisate $$ Methyl eicosanoate $$ Arachidic acid methyl ester $$ Kemester 2050 $$ Methyl aracidat

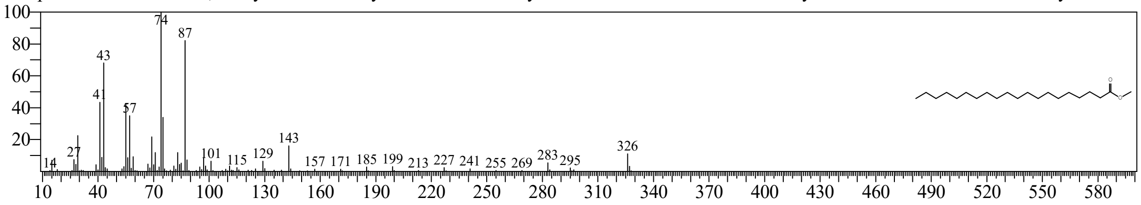

Hit#:5 Entry:29931 Library:NIST14s.lib
SI:93 Formula:C21H42O2 CAS:1120-28-1 MolWeight:326 RetIndex:2276
CompName:Eicosanoic acid, methyl ester $$ Methyl arachisate $$ Methyl eicosanoate $$ Arachidic acid methyl ester $$ Kemester 2050 $$ Methyl aracidat

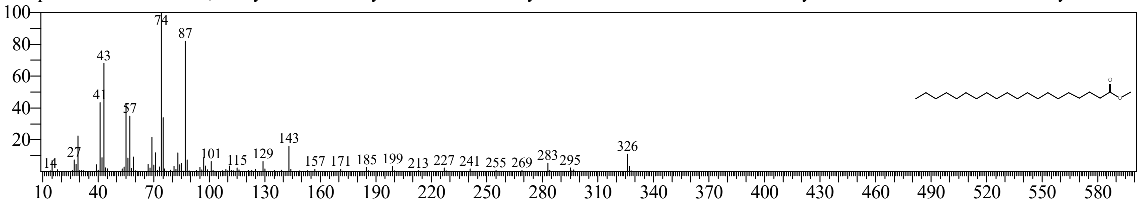

<< Target >>
Line#:14 R.Time:29.808(Scan#:2378) MassPeaks:335
RawMode:Averaged 29.800-29.817(2377-2379) BasePeak:79.05(7282)
BG Mode:Calc. from Peak Group 1 - Event 1

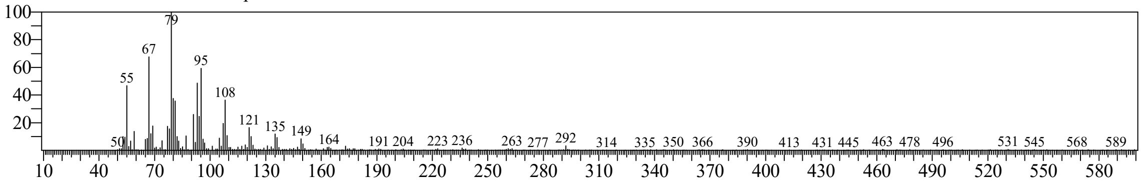

Hit#:1 Entry:27872 Library:NIST14s.lib
SI:95 Formula:C19H32O2 CAS:301-00-8 MolWeight:292 RetIndex:2101
CompName:9,12,15-Octadecatrienoic acid, methyl ester, (Z,Z,Z)- $$ Linolenic acid, methyl ester $$ Methyl all-cis-9,12,15-octadecatrienoate $$ Methyl lino

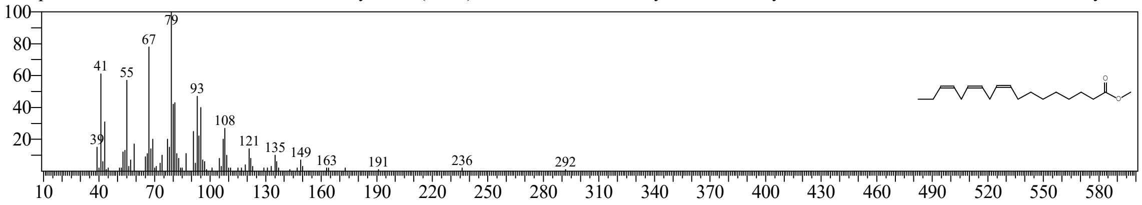

Hit#:2 Entry:135724 Library:NIST17-1.lib
SI:95 Formula:C19H32O2 CAS:301-00-8 MolWeight:292 RetIndex:2101
CompName:9,12,15-Octadecatrienoic acid, methyl ester, (Z,Z,Z)- $$ Linolenic acid, methyl ester $$ Methyl all-cis-9,12,15-octadecatrienoate $$ Methyl lino

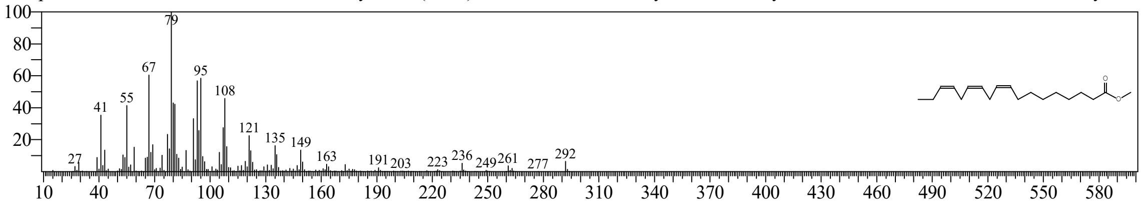

Hit#:3 Entry:124228 Library:NIST14.lib
SI:95 Formula:C19H32O2 CAS:301-00-8 MolWeight:292 RetIndex:2101
CompName:9,12,15-Octadecatrienoic acid, methyl ester, (Z,Z,Z)- $$ Linolenic acid, methyl ester $$ Methyl all-cis-9,12,15-octadecatrienoate $$ Methyl lino

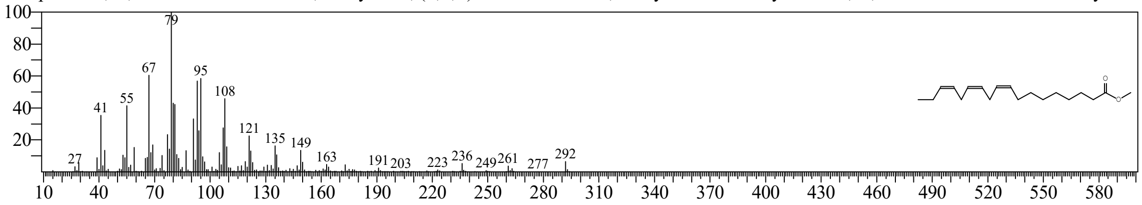

Hit#:4 Entry:32437 Library:NIST17s.lib
SI:95 Formula:C19H32O2 CAS:301-00-8 MolWeight:292 RetIndex:2101
CompName:9,12,15-Octadecatrienoic acid, methyl ester, (Z,Z,Z)- $$ Linolenic acid, methyl ester $$ Methyl all-cis-9,12,15-octadecatrienoate $$ Methyl lino

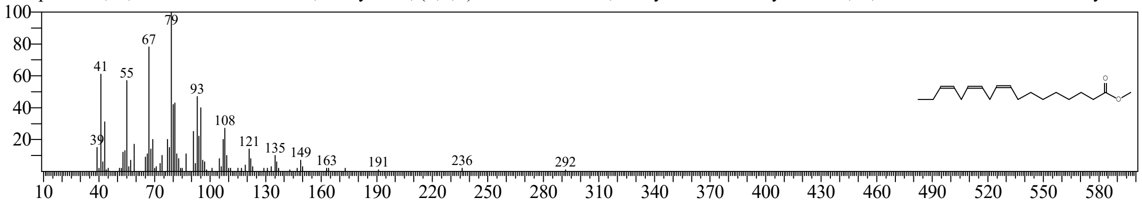

Hit#:5 Entry:32438 Library:NIST17s.lib
SI:95 Formula:C19H32O2 CAS:301-00-8 MolWeight:292 RetIndex:2101
CompName:9,12,15-Octadecatrienoic acid, methyl ester, (Z,Z,Z)- $$ Linolenic acid, methyl ester $$ Methyl all-cis-9,12,15-octadecatrienoate $$ Methyl lino

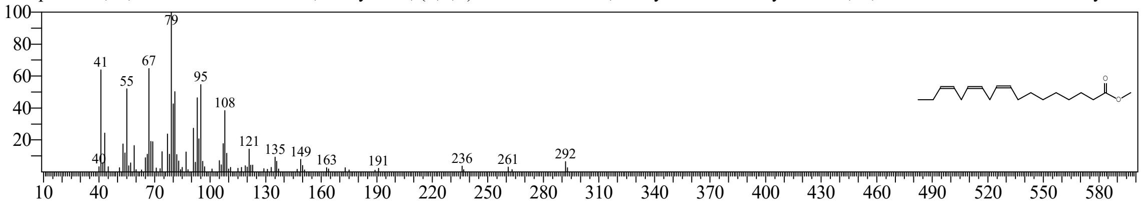

<< Target >>
Line#:15   R.Time:30.283(Scan#:2435) MassPeaks:331
RawMode:Averaged 30.275-30.292(2434-2436) BasePeak:79.05(5644)
BG Mode:Calc. from Peak Group 1 - Event 1

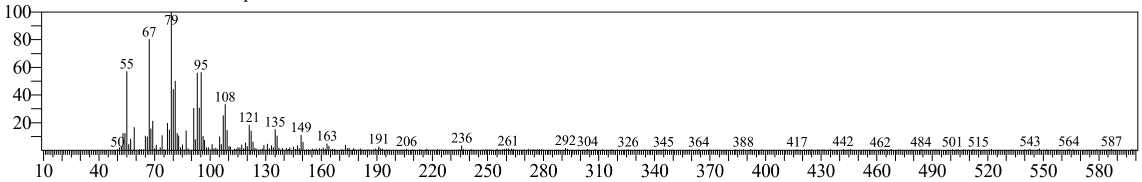

Hit#:1 Entry:124228 Library:NIST14.lib
SI:95 Formula:C19H32O2 CAS:301-00-8 MolWeight:292 RetIndex:2101
CompName:9,12,15-Octadecatrienoic acid, methyl ester, (Z,Z,Z)- $$ Linolenic acid, methyl ester $$ Methyl all-cis-9,12,15-octadecatrienoate $$ Methyl lino

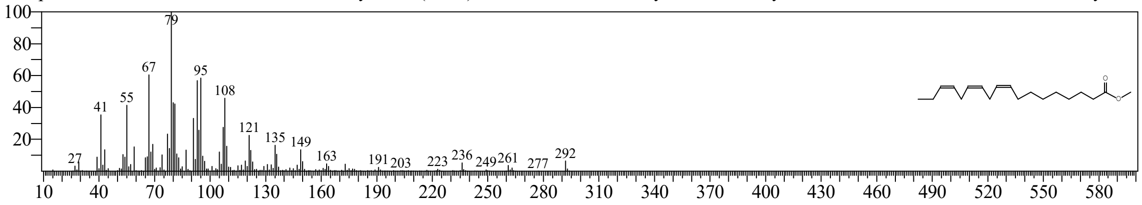

Hit#:2 Entry:135724 Library:NIST17-1.lib
SI:95 Formula:C19H32O2 CAS:301-00-8 MolWeight:292 RetIndex:2101
CompName:9,12,15-Octadecatrienoic acid, methyl ester, (Z,Z,Z)- $$ Linolenic acid, methyl ester $$ Methyl all-cis-9,12,15-octadecatrienoate $$ Methyl lino

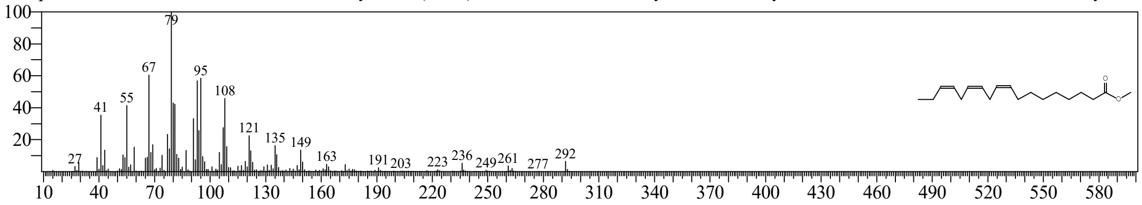

Hit#:3 Entry:121536 Library:NIST17-1.lib
SI:95 Formula:C18H30O2 CAS:0-00-0 MolWeight:278 RetIndex:2002
CompName:Methyl 8,11,14-heptadecatrienoate

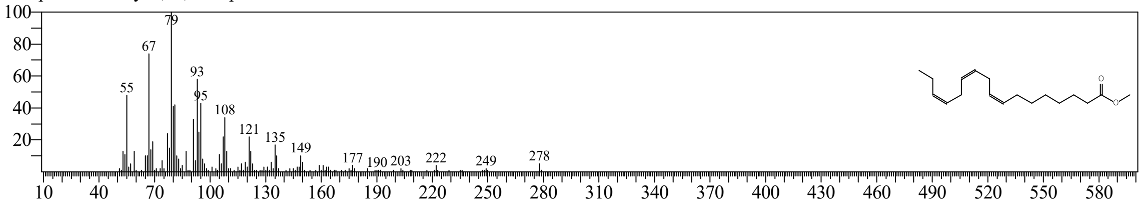

Hit#:4 Entry:111654 Library:NIST14.lib
SI:95 Formula:C18H30O2 CAS:0-00-0 MolWeight:278 RetIndex:2002
CompName:Methyl 8,11,14-heptadecatrienoate

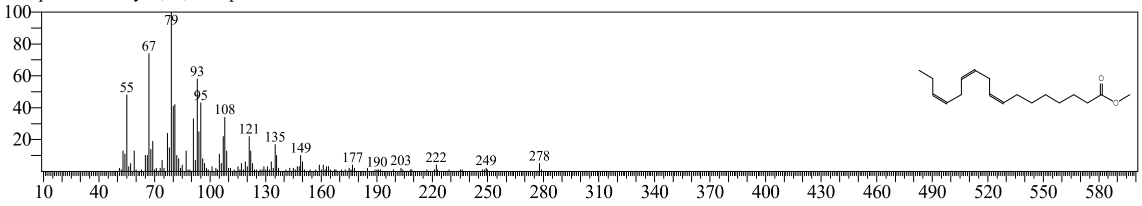

Hit#:5 Entry:27872 Library:NIST14s.lib
SI:94 Formula:C19H32O2 CAS:301-00-8 MolWeight:292 RetIndex:2101
CompName:9,12,15-Octadecatrienoic acid, methyl ester, (Z,Z,Z)- $$ Linolenic acid, methyl ester $$ Methyl all-cis-9,12,15-octadecatrienoate $$ Methyl lino

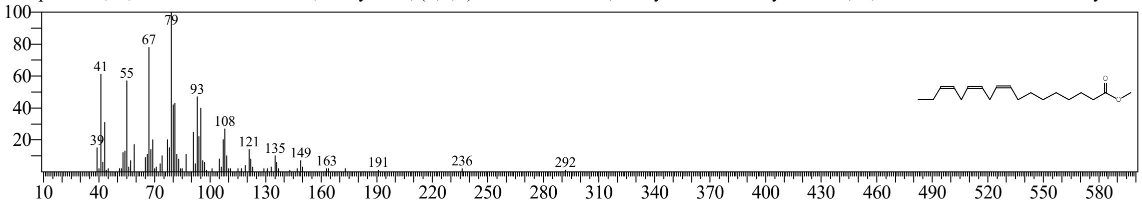

<< Target >>
Line#:16 R.Time:30.592(Scan#:2472) MassPeaks:391
RawMode:Averaged 30.583-30.600(2471-2473) BasePeak:79.05(64219)
BG Mode:Calc. from Peak Group 1 - Event 1

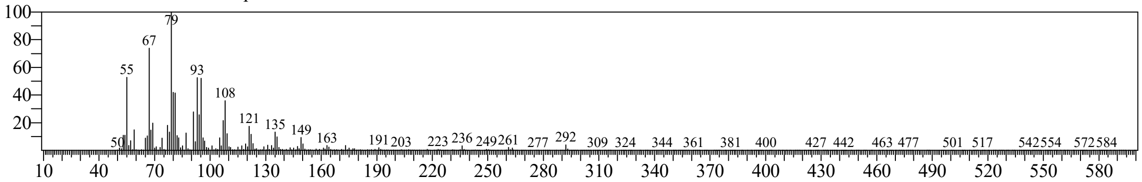

Hit#:1 Entry:135724 Library:NIST17-1.lib
SI:96 Formula:C19H32O2 CAS:301-00-8 MolWeight:292 RetIndex:2101
CompName:9,12,15-Octadecatrienoic acid, methyl ester, (Z,Z,Z)- $$ Linolenic acid, methyl ester $$ Methyl all-cis-9,12,15-octadecatrienoate $$ Methyl lino

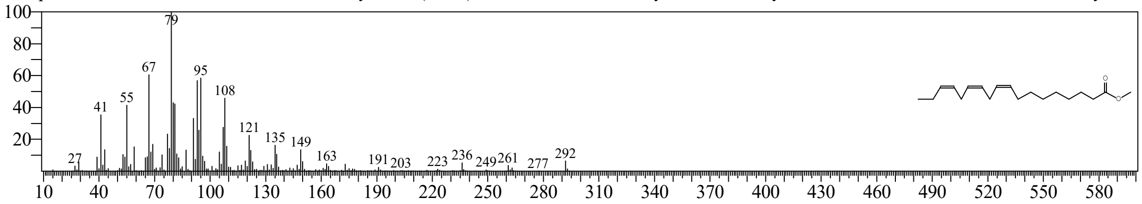

Hit#:2 Entry:124228 Library:NIST14.lib
SI:96 Formula:C19H32O2 CAS:301-00-8 MolWeight:292 RetIndex:2101
CompName:9,12,15-Octadecatrienoic acid, methyl ester, (Z,Z,Z)- $$ Linolenic acid, methyl ester $$ Methyl all-cis-9,12,15-octadecatrienoate $$ Methyl lino

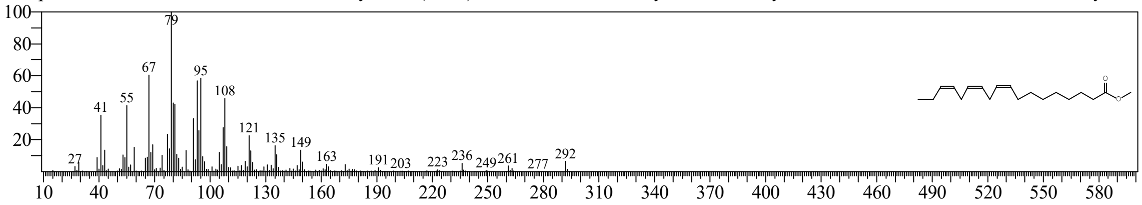

Hit#:3 Entry:32438 Library:NIST17s.lib
SI:95 Formula:C19H32O2 CAS:301-00-8 MolWeight:292 RetIndex:2101
CompName:9,12,15-Octadecatrienoic acid, methyl ester, (Z,Z,Z)- $$ Linolenic acid, methyl ester $$ Methyl all-cis-9,12,15-octadecatrienoate $$ Methyl lino

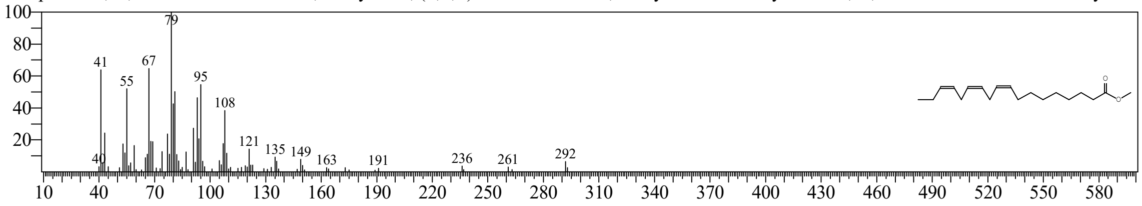

Hit#:4 Entry:27872 Library:NIST14s.lib
SI:95 Formula:C19H32O2 CAS:301-00-8 MolWeight:292 RetIndex:2101
CompName:9,12,15-Octadecatrienoic acid, methyl ester, (Z,Z,Z)- $$ Linolenic acid, methyl ester $$ Methyl all-cis-9,12,15-octadecatrienoate $$ Methyl lino

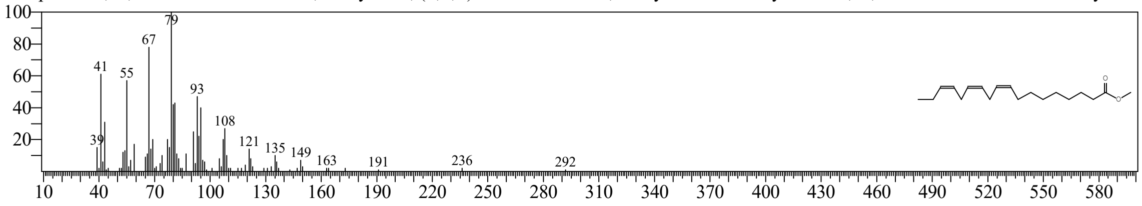

Hit#:5 Entry:32437 Library:NIST17s.lib
SI:95 Formula:C19H32O2 CAS:301-00-8 MolWeight:292 RetIndex:2101
CompName:9,12,15-Octadecatrienoic acid, methyl ester, (Z,Z,Z)- $$ Linolenic acid, methyl ester $$ Methyl all-cis-9,12,15-octadecatrienoate $$ Methyl lino

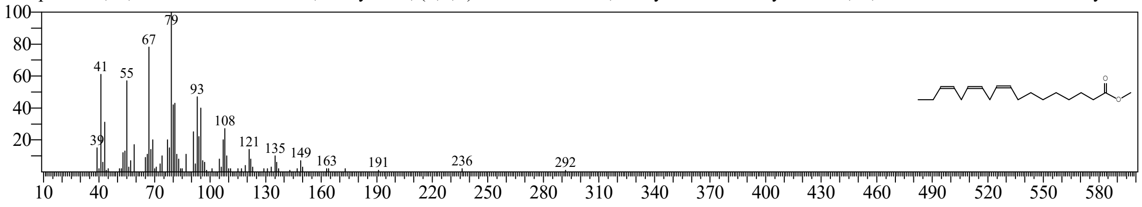

<< Target >>
Line#:17 R.Time:30.925(Scan#:2512) MassPeaks:368
RawMode:Averaged 30.917-30.933(2511-2513) BasePeak:67.00(10855)
BG Mode:Calc. from Peak Group 1 - Event 1

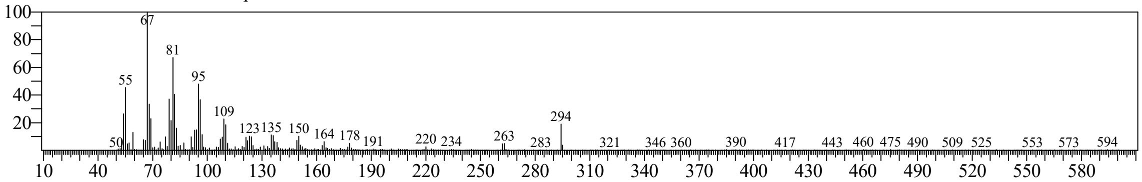

Hit#:1 Entry:137666 Library:NIST17-1.lib
SI:97 Formula:C19H34O2 CAS:13058-52-1 MolWeight:294 RetIndex:2093
CompName:Methyl 9-cis,11-trans-octadecadienoate

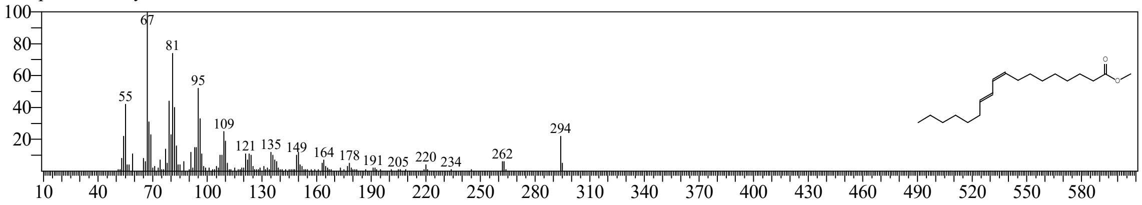

Hit#:2 Entry:125932 Library:NIST14.lib
SI:97 Formula:C19H34O2 CAS:0-00-0 MolWeight:294 RetIndex:2093
CompName:Methyl 9-cis,11-trans-octadecadienoate

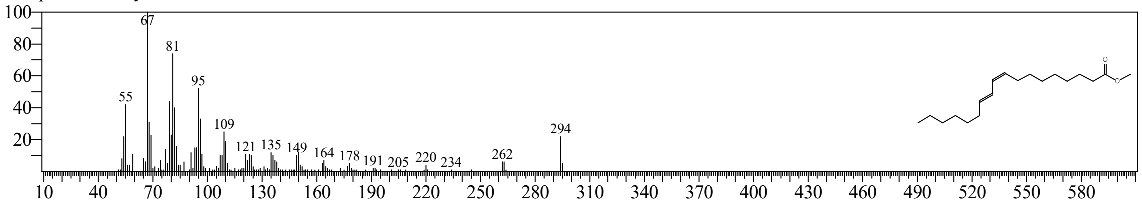

Hit#:3 Entry:125933 Library:NIST14.lib
SI:97 Formula:C19H34O2 CAS:0-00-0 MolWeight:294 RetIndex:2093
CompName:Methyl 10-trans,12-cis-octadecadienoate

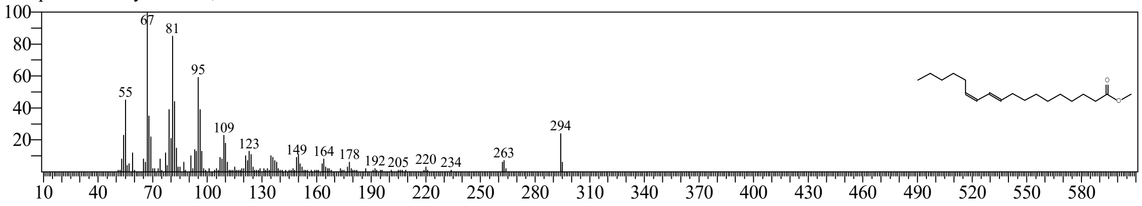

Hit#:4 Entry:137664 Library:NIST17-1.lib
SI:97 Formula:C19H34O2 CAS:0-00-0 MolWeight:294 RetIndex:2093
CompName:Methyl 10-trans,12-cis-octadecadienoate

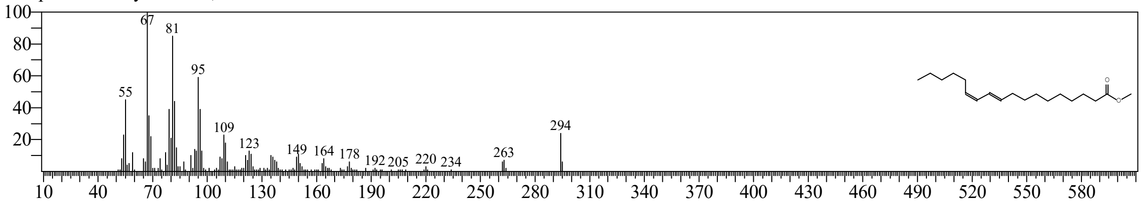

Hit#:5 Entry:32587 Library:NIST17s.lib
SI:95 Formula:C19H34O2 CAS:13038-47-6 MolWeight:294 RetIndex:2093
CompName:9,11-Octadecadienoic acid, methyl ester, (E,E)- $$ Methyl trans-9,trans-11-octadecadienoate $$ Methyl trans,trans-9,11-octadecadienoate $$ M

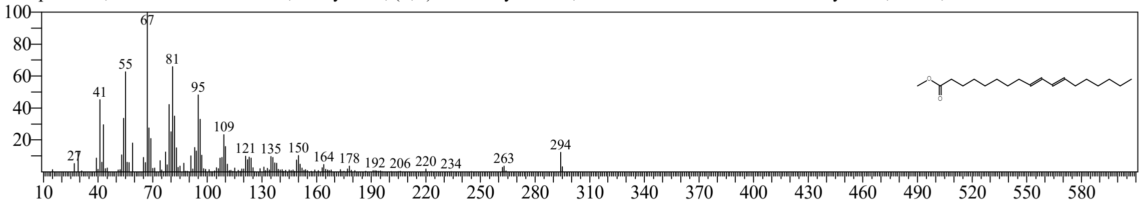

<< Target >>
Line#:18 R.Time:31.250(Scan#:2551) MassPeaks:381
RawMode:Averaged 31.242-31.258(2550-2552) BasePeak:67.05(11549)
BG Mode:Calc. from Peak Group 1 - Event 1

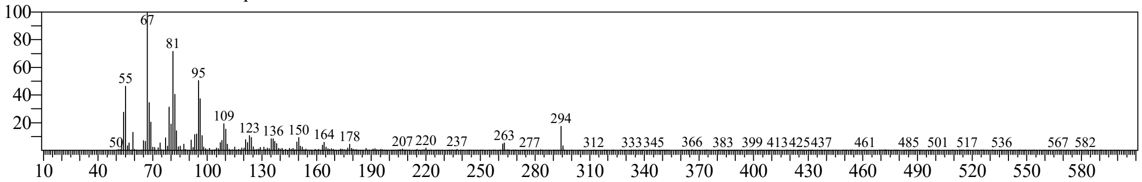

Hit#:1 Entry:125933 Library:NIST14.lib
SI:96 Formula:C19H34O2 CAS:0-00-0 MolWeight:294 RetIndex:2093
CompName:Methyl 10-trans,12-cis-octadecadienoate

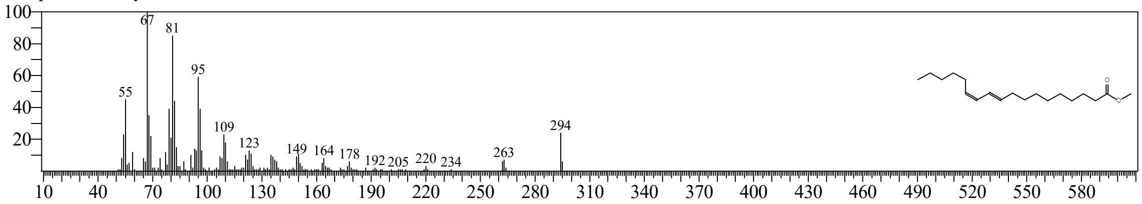

Hit#:2 Entry:137664 Library:NIST17-1.lib
SI:96 Formula:C19H34O2 CAS:0-00-0 MolWeight:294 RetIndex:2093
CompName:Methyl 10-trans,12-cis-octadecadienoate

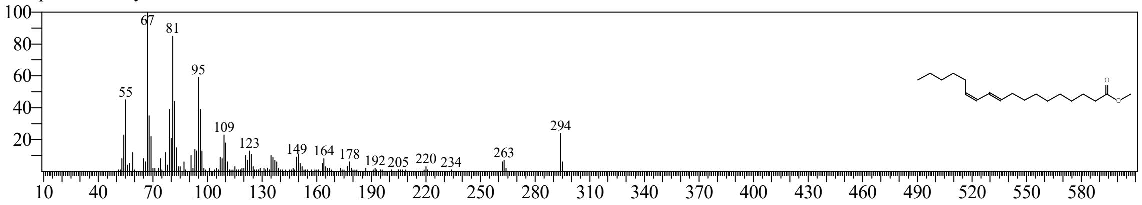

Hit#:3 Entry:125932 Library:NIST14.lib
SI:96 Formula:C19H34O2 CAS:0-00-0 MolWeight:294 RetIndex:2093
CompName:Methyl 9-cis,11-trans-octadecadienoate

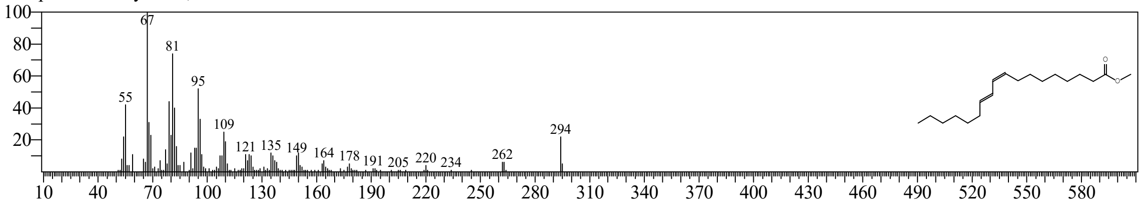

Hit#:4 Entry:137666 Library:NIST17-1.lib
SI:96 Formula:C19H34O2 CAS:13058-52-1 MolWeight:294 RetIndex:2093
CompName:Methyl 9-cis,11-trans-octadecadienoate

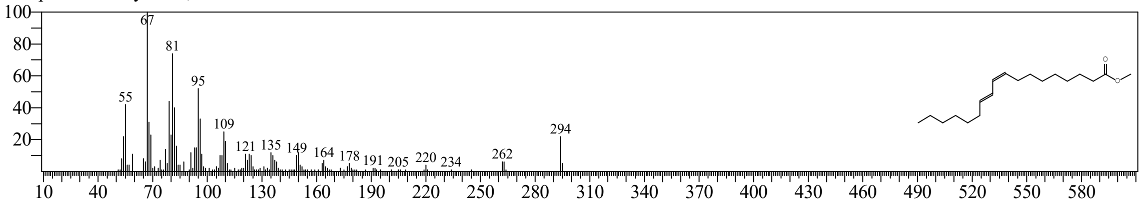

Hit#:5 Entry:32587 Library:NIST17s.lib
SI:95 Formula:C19H34O2 CAS:13038-47-6 MolWeight:294 RetIndex:2093
CompName:9,11-Octadecadienoic acid, methyl ester, (E,E)- $$ Methyl trans-9,trans-11-octadecadienoate $$ Methyl trans,trans-9,11-octadecadienoate $$ M

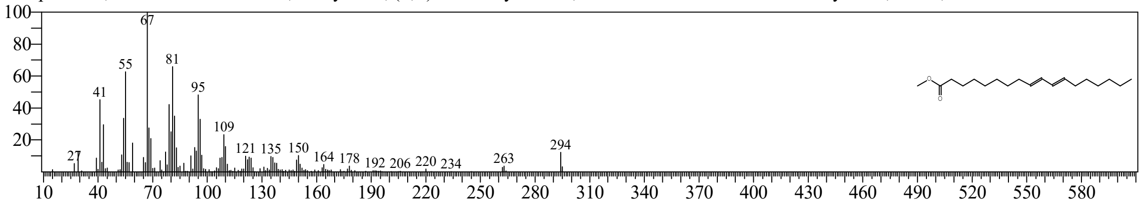

<< Target >>
Line#:19 R.Time:31.908(Scan#:2630) MassPeaks:329
RawMode:Averaged 31.900-31.917(2629-2631) BasePeak:67.00(1117)
BG Mode:Calc. from Peak Group 1 - Event 1

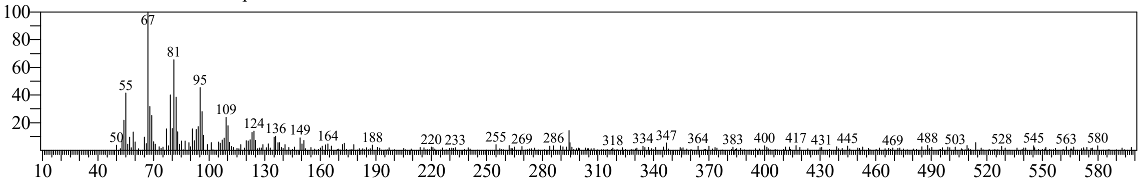

Hit#:1 Entry:32587 Library:NIST17s.lib
SI:90 Formula:C19H34O2 CAS:13038-47-6 MolWeight:294 RetIndex:2093
CompName:9,11-Octadecadienoic acid, methyl ester, (E,E)- $$ Methyl trans-9,trans-11-octadecadienoate $$ Methyl trans,trans-9,11-octadecadienoate $$ M

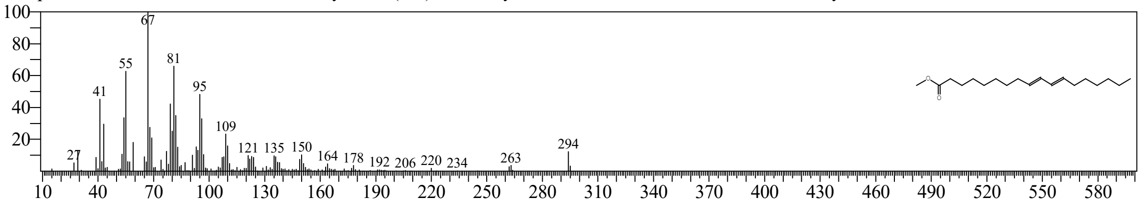

Hit#:2 Entry:137666 Library:NIST17-1.lib
SI:89 Formula:C19H34O2 CAS:13058-52-1 MolWeight:294 RetIndex:2093
CompName:Methyl 9-cis,11-trans-octadecadienoate

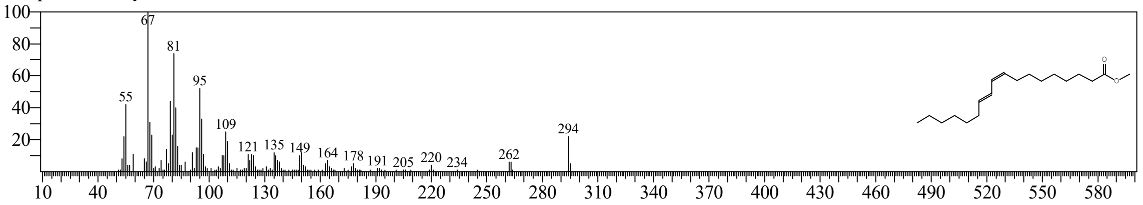

Hit#:3 Entry:125932 Library:NIST14.lib
SI:89 Formula:C19H34O2 CAS:0-00-0 MolWeight:294 RetIndex:2093
CompName:Methyl 9-cis,11-trans-octadecadienoate

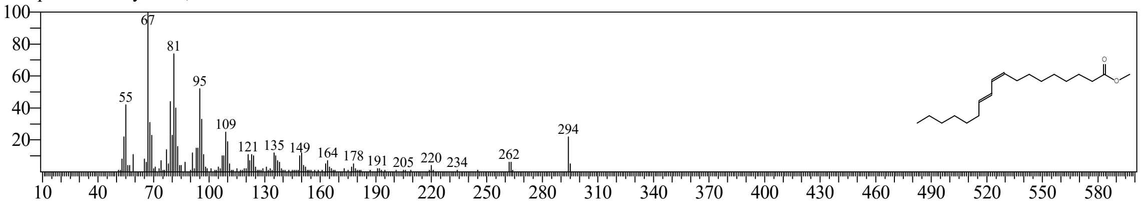

Hit#:4 Entry:125933 Library:NIST14.lib
SI:89 Formula:C19H34O2 CAS:0-00-0 MolWeight:294 RetIndex:2093
CompName:Methyl 10-trans,12-cis-octadecadienoate

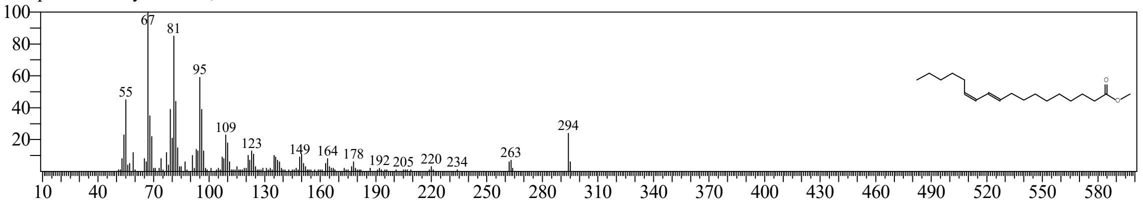

Hit#:5 Entry:137664 Library:NIST17-1.lib
SI:89 Formula:C19H34O2 CAS:0-00-0 MolWeight:294 RetIndex:2093
CompName:Methyl 10-trans,12-cis-octadecadienoate

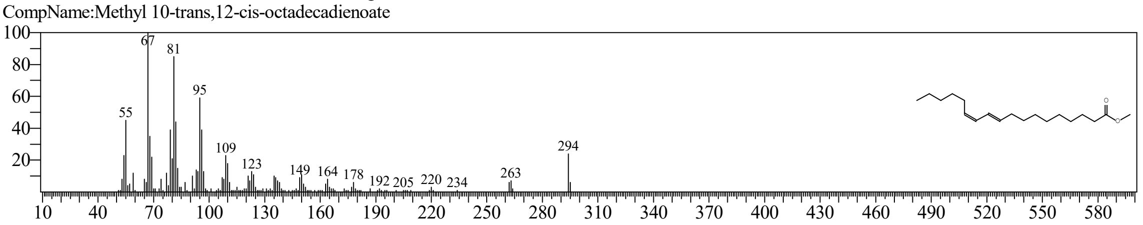

<< Target >>
Line#:20 R.Time:32.950(Scan#:2755) MassPeaks:360
RawMode:Averaged 32.942-32.958(2754-2756) BasePeak:95.10(12260)
BG Mode:Calc. from Peak Group 1 - Event 1

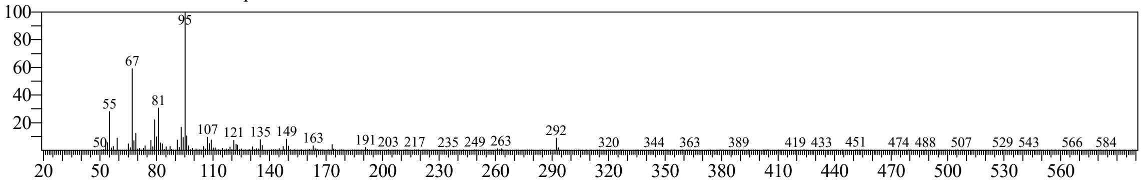

Hit#:1 Entry:24424 Library:NIST17-1.lib
SI:85 Formula:C12H20 CAS:0-00-0 MolWeight:164 RetIndex:1041
CompName:Cis-3-ethyl-endo-tricyclo[5.2.1.0(2.6)]decane

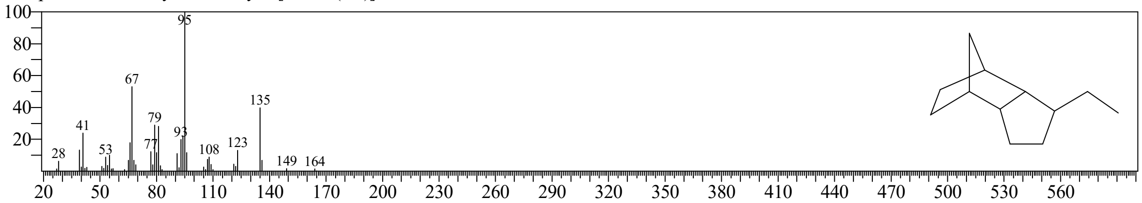

Hit#:2 Entry:23403 Library:NIST14.lib
SI:85 Formula:C12H20 CAS:0-00-0 MolWeight:164 RetIndex:1041
CompName:Cis-3-ethyl-endo-tricyclo[5.2.1.0(2.6)]decane

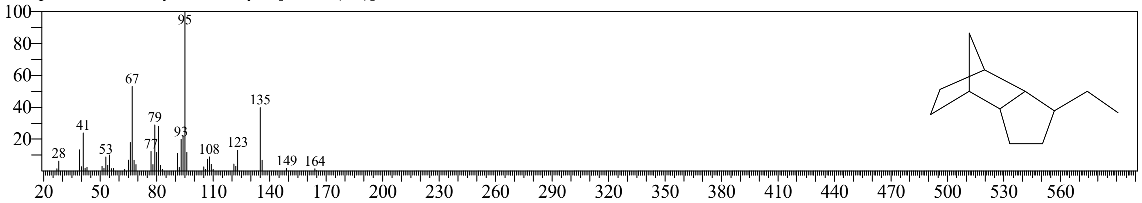

Hit#:3 Entry:16897 Library:NIST14.lib
SI:83 Formula:C10H16O CAS:60485-45-2 MolWeight:152 RetIndex:991
CompName:Santolina epoxide $$ 2-Methyl-2-(3-methyl-1-vinyl-2-butenyl)oxirane # $$

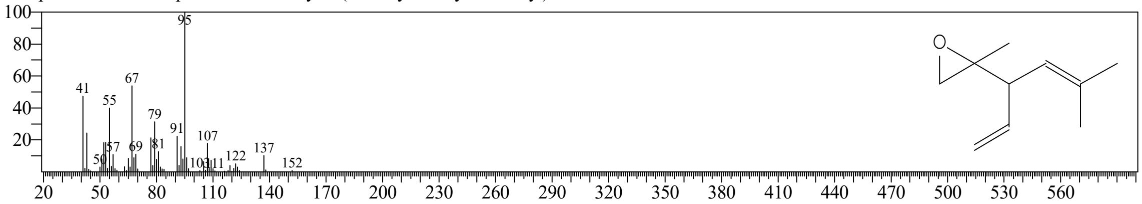

Hit#:4 Entry:17571 Library:NIST17-1.lib
SI:83 Formula:C10H16O CAS:60485-45-2 MolWeight:152 RetIndex:991
CompName:Santolina epoxide $$ 2-Methyl-2-(3-methyl-1-vinyl-2-butenyl)oxirane # $$

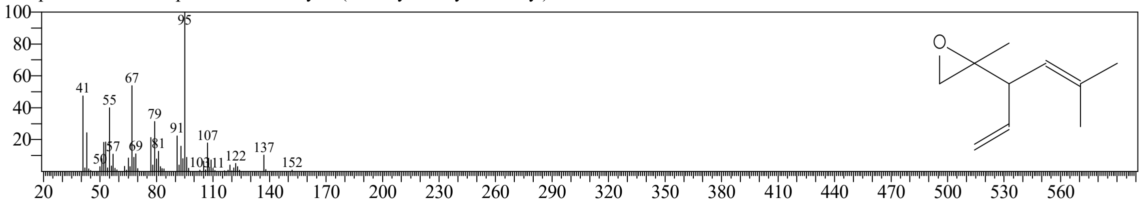

Hit#:5 Entry:16582 Library:NIST17-1.lib
SI:83 Formula:C11H18 CAS:0-00-0 MolWeight:150 RetIndex:1115
CompName:4-Cyclohexenyl-3-methylbutene

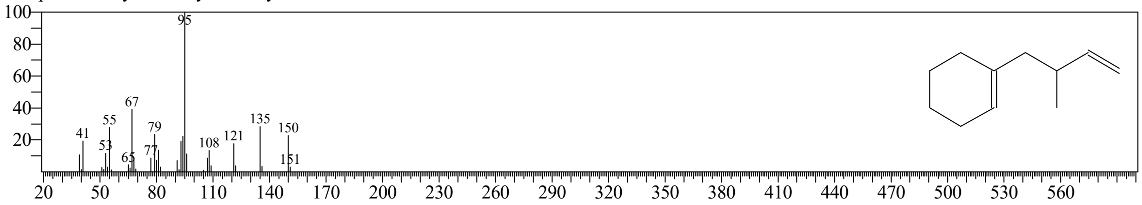

<< Target >>
Line#:21 R.Time:33.483(Scan#:2819) MassPeaks:331
RawMode:Averaged 33.475-33.492(2818-2820) BasePeak:74.00(13710)
BG Mode:Calc. from Peak Group 1 - Event 1

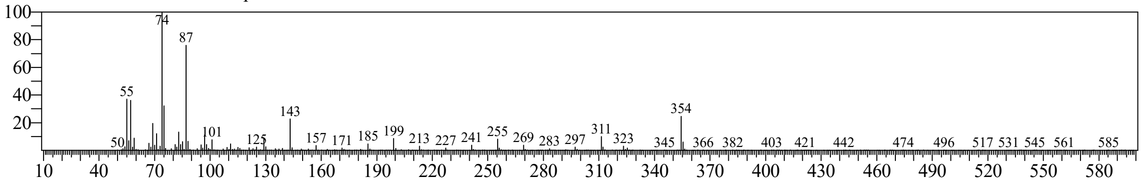

Hit#:1 Entry:196443 Library:NIST17-1.lib
SI:96 Formula:C23H46O2 CAS:929-77-1 MolWeight:354 RetIndex:2475
CompName:Docosanoic acid, methyl ester $$ Behenic acid, methyl ester $$ Methyl behenate $$ Methyl docosanoate $$ n-Docosanoic acid methyl ester $$

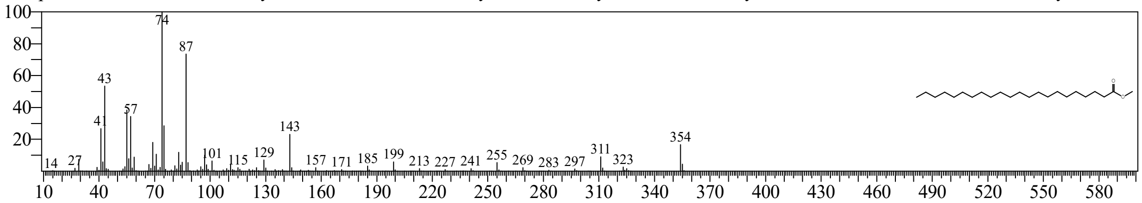

Hit#:2 Entry:178051 Library:NIST14.lib
SI:96 Formula:C23H46O2 CAS:929-77-1 MolWeight:354 RetIndex:2475
CompName:Docosanoic acid, methyl ester $$ Behenic acid, methyl ester $$ Methyl behenate $$ Methyl docosanoate $$ n-Docosanoic acid methyl ester $$

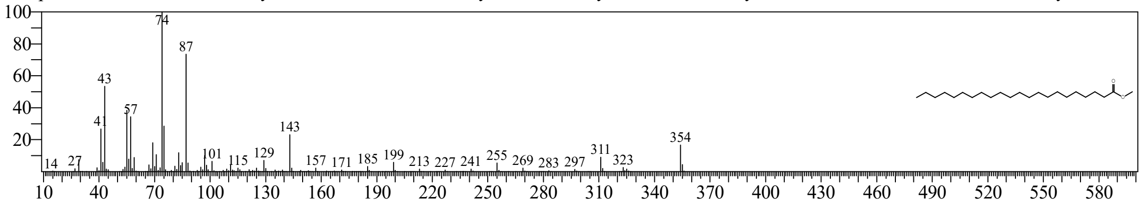

Hit#:3 Entry:196444 Library:NIST17-1.lib
SI:95 Formula:C23H46O2 CAS:0-00-0 MolWeight:354 RetIndex:2411
CompName:Methyl 20-methyl-heneicosanoate

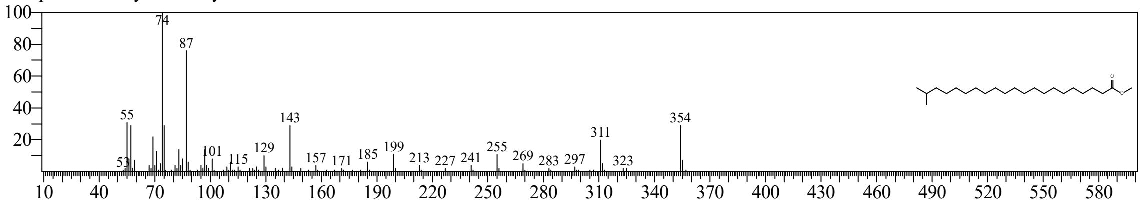

Hit#:4 Entry:178052 Library:NIST14.lib
SI:95 Formula:C23H46O2 CAS:0-00-0 MolWeight:354 RetIndex:2411
CompName:Methyl 20-methyl-heneicosanoate

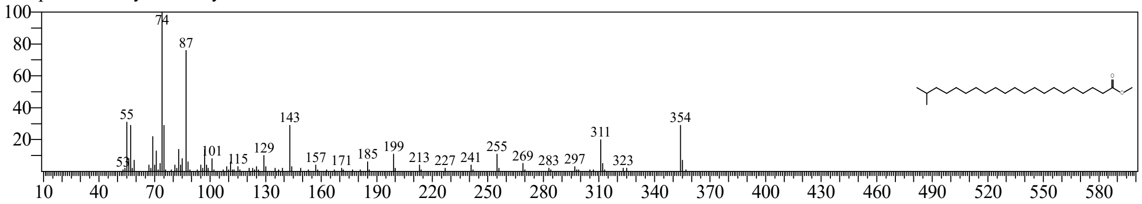

Hit#:5 Entry:31088 Library:NIST14s.lib
SI:95 Formula:C23H46O2 CAS:929-77-1 MolWeight:354 RetIndex:2475
CompName:Docosanoic acid, methyl ester $$ Behenic acid, methyl ester $$ Methyl behenate $$ Methyl docosanoate $$ n-Docosanoic acid methyl ester $$

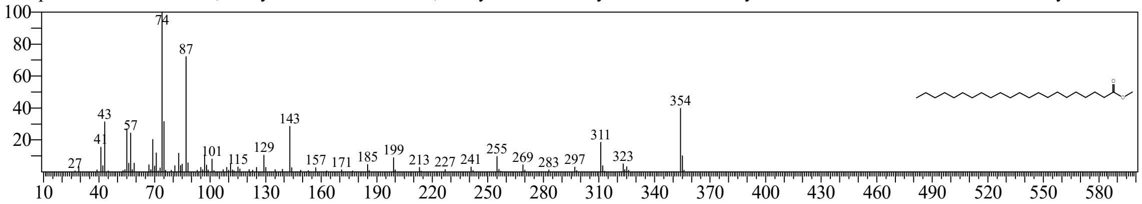

<< Target >>
Line#:22 R.Time:37.792(Scan#:3336) MassPeaks:300
RawMode:Averaged 37.783-37.800(3335-3337) BasePeak:74.05(3028)
BG Mode:Calc. from Peak Group 1 - Event 1

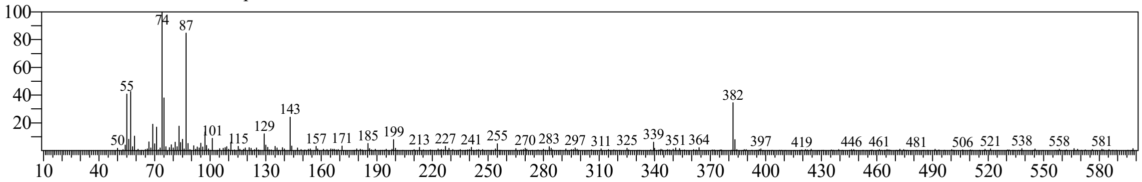

Hit#:1 Entry:31970 Library:NIST14s.lib
SI:92 Formula:C25H50O2 CAS:2442-49-1 MolWeight:382 RetIndex:2674
CompName:Tetracosanoic acid, methyl ester $$ Methyl lignocerate $$ Methyl tetracosanoate $$ Lignoceric acid methyl ester $$

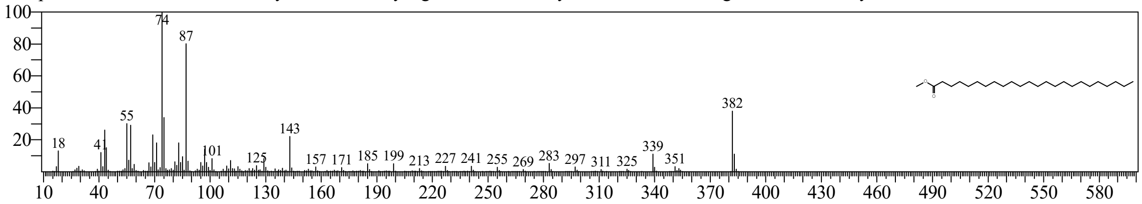

Hit#:2 Entry:37106 Library:NIST17s.lib
SI:92 Formula:C25H50O2 CAS:2442-49-1 MolWeight:382 RetIndex:2674
CompName:Tetracosanoic acid, methyl ester $$ Methyl lignocerate $$ Methyl tetracosanoate $$ Lignoceric acid methyl ester $$

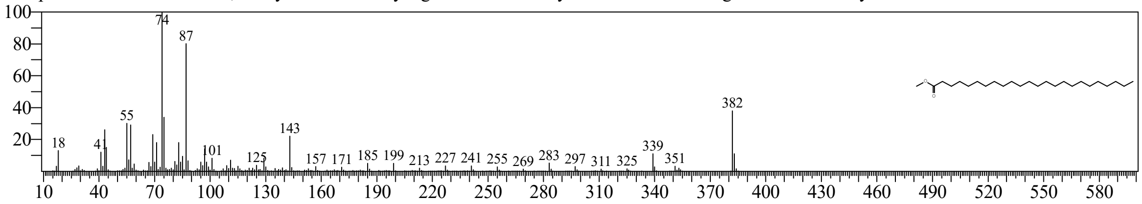

Hit#:3 Entry:31971 Library:NIST14s.lib
SI:89 Formula:C25H50O2 CAS:2442-49-1 MolWeight:382 RetIndex:2674
CompName:Tetracosanoic acid, methyl ester $$ Methyl lignocerate $$ Methyl tetracosanoate $$ Lignoceric acid methyl ester $$

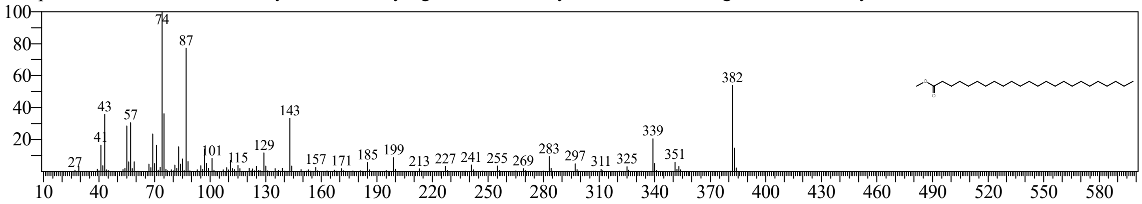

Hit#:4 Entry:37107 Library:NIST17s.lib
SI:89 Formula:C25H50O2 CAS:2442-49-1 MolWeight:382 RetIndex:2674
CompName:Tetracosanoic acid, methyl ester $$ Methyl lignocerate $$ Methyl tetracosanoate $$ Lignoceric acid methyl ester $$

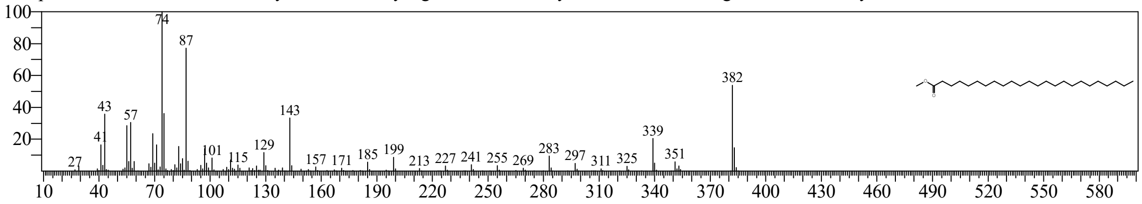

Hit#:5 Entry:37105 Library:NIST17s.lib
SI:89 Formula:C25H50O2 CAS:2442-49-1 MolWeight:382 RetIndex:2674
CompName:Tetracosanoic acid, methyl ester $$ Methyl lignocerate $$ Methyl tetracosanoate $$ Lignoceric acid methyl ester $$

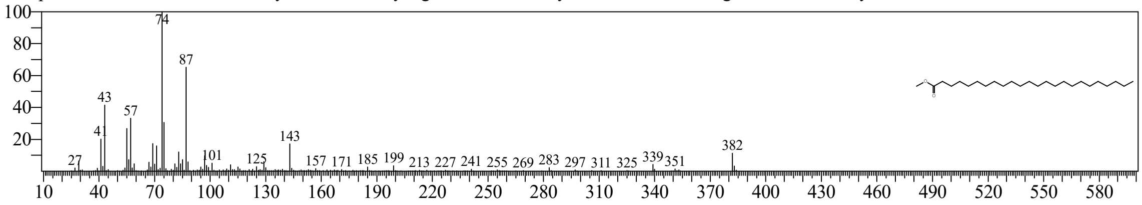

Supplement: Supplementary file 1 [file molecules-31-02891-s001.zip › 7d-QG200-3.pdf]
